# Imidazo[1,5‐*a*]pyridines – A Versatile Platform for Structurally Distinct *N*‐Heterocyclic Olefins and π‐Extended Heterocycles

**DOI:** 10.1002/anie.202506305

**Published:** 2025-05-19

**Authors:** Robin Esken, Patrick W. Antoni, Yannick Lorenz, Chris Burdenski, Jan‐Lukas Kirchhoff, Carsten Strohmann, Max M. Hansmann

**Affiliations:** ^1^ Fakultät für Chemie und Chemische Biologie Technische Universität Dortmund Otto‐Hahn‐Str. 6 44227 Dortmund Germany

**Keywords:** Imidazo[2,1,5‐*de*]quinolizines, Donor ligands, Fluorescent organic radicals, *N*‐heterocyclic olefins, Paratropic ring current

## Abstract

The synthesis of polarized *N*‐heterocyclic olefins (NHOs) based on an imidazo[1,5‐*a*]pyridine scaffold is presented. In contrast to regular NHOs the unique bicyclic heterocyclic core allows the incorporation of various substituents in close proximity to the highly polarized exocyclic C─C bond. The donor properties of the new carbon‐based ligand class were quantified and the coordination chemistry explored. Alkenyl or alkynyl groups at the C5‐position lead to spontaneous cyclization to yield imidazo[2,1,5‐*de*]quinolizines. The unique cyclization strategy was compatible with a wide range of substitution patterns and yields highly electron‐rich π‐delocalized heterocycles. The electronic structure of the novel partially antiaromatic heterocycle was thoroughly investigated. One‐electron oxidation occurs at low potentials and lead to a stable monomeric radical‐cation confirmed by XRD, which showed solution phase fluorescence expanding into the field of open‐shell organic materials.

## Graphical abstract

The synthesis of *N*‐heterocyclic olefins (NHOs) based on an imidazo[1,5‐*a*]pyridine scaffold is described. While C5‐alkyl or aryl substituents yield stable NHOs, alkenyl or alkynyl groups at C5 induce 6‐*endo* cyclization to form (dihydro)imidazo[2,1,5‐*de*]quinolizines with strong paratropic ring currents. These heterocycles undergo low‐potential oxidation to produce fluorescent radical cations, expanding the scope of fluorescent organic radicals.

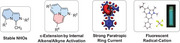

Fused polycyclic heteroarenes play a crucial role in diverse fields, with applications ranging from bioimaging and pharmaceuticals to organic materials.^[^ ^1^ ^]^ Among them, π‐extended imidazoles have emerged as a valuable subclass due to their structural versatility and tunable electronic properties.^[^ ^2^, ^3^, ^4^, ^5^, ^6^, ^7^ ^]^ To date, three main synthetic strategies have been developed to access π‐extended imidazoles with multiple annulation points (**III**, **V**, **VII**; Scheme 1a),^[^ ^8^ ^]^ all of which use transition metal‐catalyzed C─H activation as a central step to extend the conjugated π‐system.^[^ ^9^, ^10^, ^11^, ^12^, ^13^, ^14^, ^15^, ^16^ ^]^ However, there are no examples of neutral π‐extended imidazo[1,5‐*a*]pyridines in which both nitrogen atoms are substituted.^[^ ^17^ ^]^ This particular structural motif is reminiscent of the *N*‐heterocyclic carbenes (NHCs) derived from imidazo[1,5‐*a*]pyridines (Scheme 1b).^[^ ^18^, ^19^, ^20^, ^21^ ^]^ This class of NHCs is defined by the donor properties typical of imidazole‐based NHCs, with enhanced π‐acceptor capabilities, and is further distinguished by the ability to incorporate aromatic substituents specifically at the C5 position.^[^ ^22^, ^23^, ^24^, ^25^, ^26^ ^]^ Substituents at C5 are almost collinearly oriented with a coordinated metal atom and can thus greatly enhance the stability of key intermediates in catalysis, similar to Buchwald type ligands.^[^ ^27^, ^28^, ^29^ ^]^ *N*‐Heterocyclic olefins (NHOs) in which formally a CH₂ fragment is attached to a carbene,^[^ ^30^, ^31^ ^]^ contain a strongly polarized exocyclic C─C bond and are strong carbon σ‐donors (Scheme 1c). NHOs have become widely used as ligands in transition metal and main group chemistry, as well as potent nucleophiles in organic synthesis.^[^ ^32^, ^33^, ^34^, ^35^, ^36^, ^37^ ^]^

**Scheme 1:**
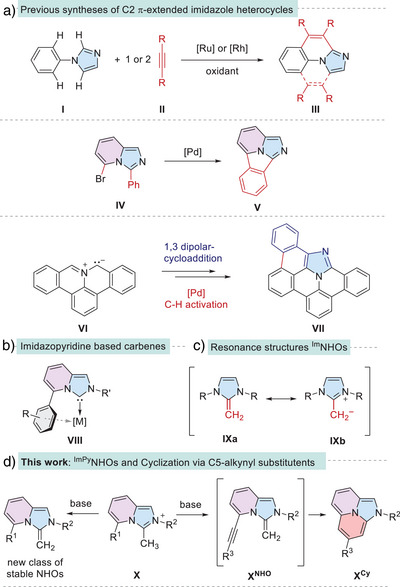
a) Synthetic strategies toward neutral, π‐extended imidazole‐based heterocycles; b) Imidazopyridine‐based NHCs, highlighting the C5‐aryl‐metal interaction; c) Resonance structures of imidazole based NHOs; d) Deprotonation of imidazo[1,5‐*a*]pyridinium salt **X** to form stable NHOs and π‐extended heterocycles **X^Cy^**.

Interestingly, while imidazo[1,5‐*a*]pyridine based carbenes have been well investigated and the structural diversity of NHOs has expanded significantly in recent years,^[^ ^38^, ^39^ ^]^ the corresponding imidazo[1,5‐*a*]pyridine‐based NHOs are unknown. Here we describe both structurally simple imidazo[1,5‐*a*]pyridine NHOs, as well as the possibility of the intramolecular cyclization by the introduction of an alkyne or alkene moiety on the C5 position (Scheme 1d).

To establish the synthetic accessibility and stability of imidazo[1,5‐*a*]pyridine (ImPy) derived NHOs we first aimed to synthesize the structurally most simple derivatives. As such, deprotonation of salt **1a**, accessible over three steps (see Supporting Information), with KH or KHMDS gave NHO **2a** (Scheme 2).

**Scheme 2:**
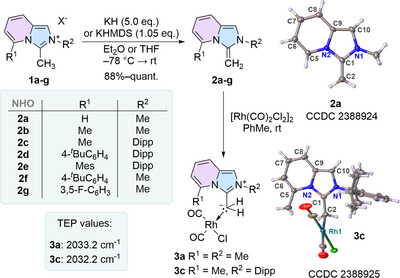
Synthesis of a series of structurally diverse C5‐substituted imidazopyridine NHOs **2a–g** and their reaction to form two representative Rh(I) complexes for TEP determination. X‐ray solid‐state structures of **2a** and **3c** shown without disorders. Thermal ellipsoids are shown with 50% probability. *X* = I^−^ or OTf^−^.

We noticed a different stability behavior dependent on the base used. Deprotonation with KH afforded an intense dark red solution that showed no signs of decomposition at room temperature, whereas KHMDS led to a slow decomposition of the NHO (Figure S386). This process is likely catalyzed by a reversible reaction of the formed protonated HMDS, followed by a dimerization process. Inspired by the work of Lassaletta et al. who described an increase in the stability of the ImPy carbene by a methyl group at C5 position,^[^ ^18^ ^]^ NHO **2b** was synthesized. Indeed, **2b** showed increased stability even upon deprotonation with KHMDS. We next increased the steric bulk by installing a 2,6‐diisopropylphenyl (Dipp; **2c**) moiety on the N2 atom as well as substitution on the C5 position (Scheme 2). All NHOs (**2a‐2g**), showed a significant high field shift of both the ^1^H‐ and ^13^C‐NMR signals of the core as well as of the exocyclic CH_2_‐fragment [*δ*(^1^H) = 1.92–3.57 ppm), *δ*(^13^C) = 40.3–53.3 ppm], similar to imidazole based NHOs, supporting the electron rich nature of this novel class of ^Impy^NHOs. Furthermore, the solid‐state structure of **2a** (Scheme 2)^[^ ^40^ ^]^ as well as **2c** (Figure S447) showed significant bond length alternations in the heterocycle [**2a**: C5─C6 1.343(3) Å; C6─C7 1.452(3) Å; C7─C8 1.359(3) Å; C8─C9: 1.421(3) Å] as well as a C1─C2 bond length [**2a**: 1.354(3) Å; **2c**: 1.305(10) Å/1.291(10) Å] in the range of a classical C─C double bond.

To get first insights into the coordination chemistry and to determine the overall donor properties of ^ImPy^NHOs, **2a** and **2c** were reacted with [RhCl(CO)_2_]_2_. The end‐on binding mode by the exocyclic methylene carbon to rhodium in **3a** (yield: 90%) could be verified by X‐ray diffraction (Scheme 2). IR stretching frequencies of the Rh‐complex **3a** and **3c** were evaluated in solution (CH_2_Cl_2_) to determine the overall donor properties of the corresponding olefins **2a** and **2c** by the Tolman electronic parameter (TEP). According to TEP = 0.8001 *v*_av _+ 420 cm^−1^,^[^ ^41^ ^]^ **3a** features a TEP of 2033.2 cm^−1^, with only a negligible effect of the *N*‐Dipp substitution (**3c**; TEP = 2032.2 cm^−1^). This positions **2a** and **2c** as stronger donors than NHC carbenes (IPr: TEP = 2051.1 cm^−1^, ^ImPy^NHC: TEP = 2051.8 cm^−1^),^[^ ^18^, ^38^ ^]^ while being slightly weaker than imidazole derived NHOs (IPr = CH_2_: TEP = 2031.4 cm^−1^).^[^ ^42^, ^43^, ^44^ ^]^

Upon studying the impact of an alkynyl substituent at the C5‐position on the stability of the corresponding ^ImPy^NHOs, we observed a rapid color change from dark blue to intense yellow‐green upon warming up **4a** in the presence of 1.05 eq. KHMDS. Variable temperature NMR experiments confirmed the initial formation of NHO **5a** (Figure S387), which underwent 6‐*endo* cyclization at −30 °C to yield the unknown imidazo[2,1,5‐*de*]quinolizine **6a** (Scheme 3).

As far as we know this is the first intramolecular cyclization of any NHO and represents a unique reactivity for a strong carbon nucleophile. Interestingly, there is a coupled flow of electron density in which the addition of the strongly negatively polarized C2 atom (Mulliken charge: −0.77e) leads to the concurrent formation of an electron deficient heterocycle, hence activating the alkyne substituent. Typically, the nucleophilic attack on an unactivated alkyne moiety requires activation by a carbophilic Lewis acid, as seen in frustrated Lewis pair (FLP) chemistry or Au‐catalysis.^[^ ^45^, ^46^, ^47^, ^48^ ^]^ In stark contrast to the very high reactivity of 5‐alkyne substituted NHOs, the TIPS‐substituted derivative (**4b**) displayed very slow cyclization tendency, as the NHO (**5b**) was detected for hours at room temperature by NMR spectroscopy. Prolonged reaction times (5 days at rt) or heating to 80 °C for 30 min, furnished compound **6b** in good yield (88%). This observation aligns with increased charge density at the alkyne moiety as well as sterical shielding. Treatment of **5b** with (dms)AuCl furnishes the end‐on complex **5b‐Au**, showing no signs of cyclization (Scheme 3). Such an approach should be interesting for ligand design to access metal NHO complexes which could interact intramolecularly with the alkyne π‐system.

**Scheme 3:**
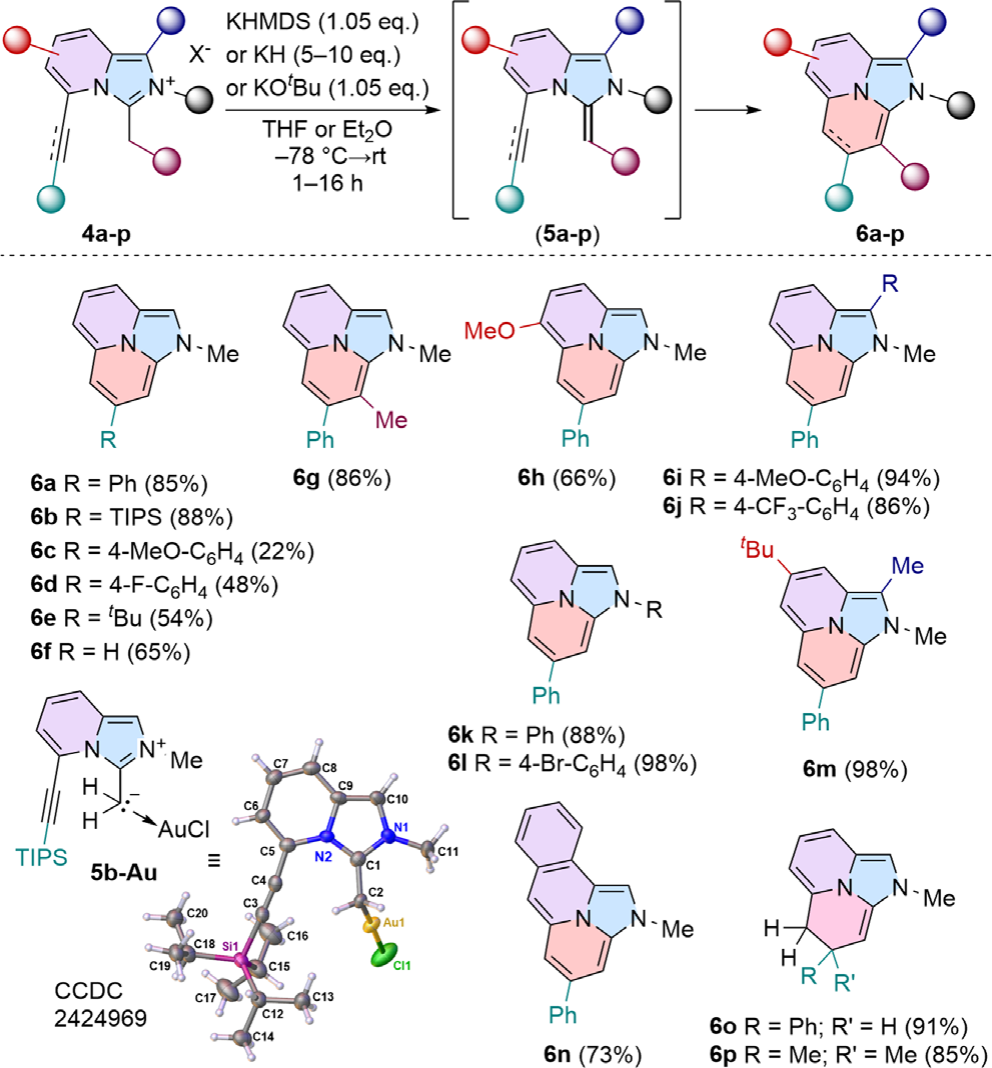
Reaction scope of imidazo[2,1,5‐*de*]quinolizines **6a**–**p**. X‐ray solid‐state structure of **5b‐Au**. Thermal ellipsoids are shown with 50% probability.

Next the scope of the cyclization was investigated (Scheme 3). Donor‐substituted (**4c**), electron‐deficient aryl (**4d**) or alkyl‐substituted alkynes (**4e**) were tolerated and yielded the new heterocycles (**6c**–**6e**) in good yields. Even the terminal alkyne (**4f**) furnished the unsubstituted imidazo[2,1,5‐*de*]quinolizine **6f** (65% yield). The cyclization strategy was not restricted to a NHO with an exocyclic CH_2_ fragment but also an internal olefin (CHMe) afforded the cyclization product (**6g**). Additionally, substitution at the pyridine core (**6h**) as well as imidazole core (**6i**–**6j**) gave the desired cyclization products in high yield (86%–94%). Substitution of the *N*‐aryl substituents on the imidazole core (**6k**–**6l**), substitution on the pyridine and imidazole part (**6m**) and π‐extension (**6n**) were all compatible with the cyclization strategy. Additionally, the cyclization not only proceeded with alkynes but also alkenes, such as **4o** and **4p** to form the dihydroimidazo[2,1,5‐*de*]quinolizines **6o** and **6p** in excellent yield (91% and 85% respectively).

A computational evaluation of the reaction mechanism using a combination of DFT and DLPNO approximated coupled cluster theory [r^2^SCAN‐3c(SMD:THF)//DLPNO‐CCSD(T)/def2‐TZVPP] revealed that the transition state for a potential 5‐*exo*‐*dig* cyclization (**TS^5^1**) is disfavored by nearly 9 kcal mol^−1^ compared to the corresponding 6‐*endo*‐*dig* transition state **TS^6^1** (Figure 1). **TS^6^1** is low in energy (+18.9 kcal mol^−1^) in good agreement with a spontaneous cyclization at room temperature and leads to the formation of an isoenergetic zwitterionic intermediate/remote carbene (**Int^6^ 1**), which subsequently undergoes intermolecular proton transfer to yield product **6a**. In contrast, for alkene **4o** the rate‐determining transition state of the cyclization occurs at a slightly higher energy (19.1 kcal mol^−1^), while the overall process is significantly less exergonic (−18.1 kcal mol^−1^; see Supporting Information).

**Figure 1:**
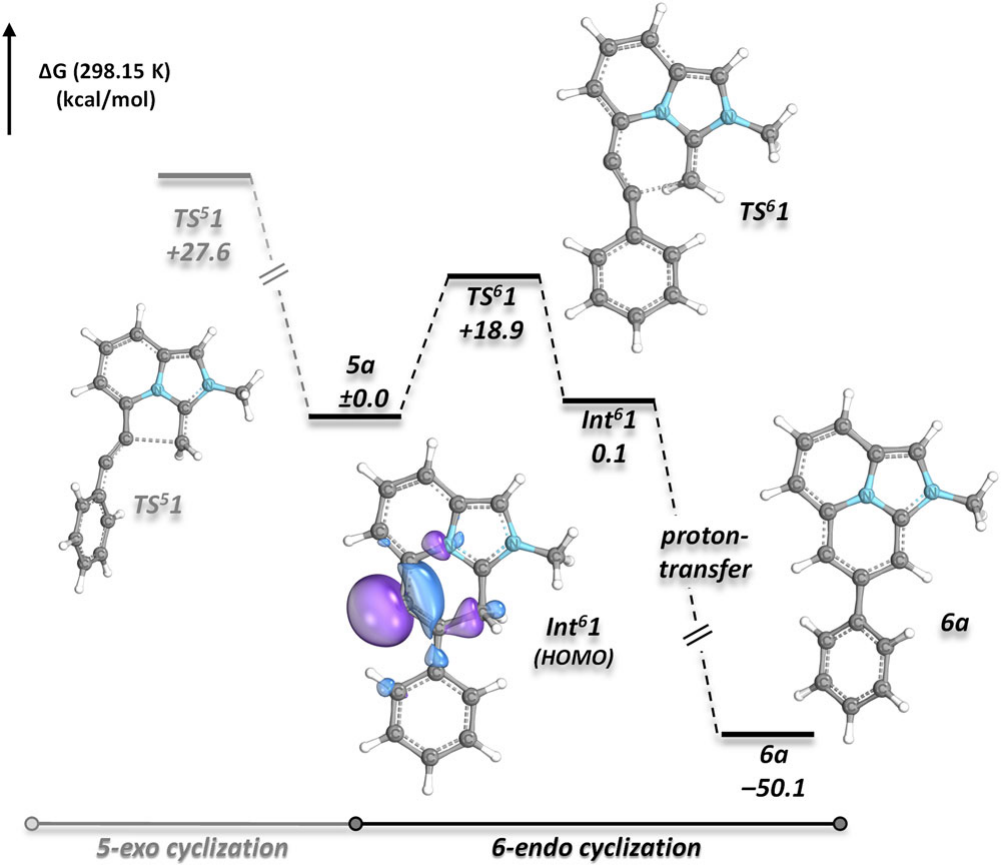
Cyclization of C5‐alkynyl‐substituted imidazopyridine NHO **5a** to (dihydro)imidazo[2,1,5‐*de*]quinolizine **6a**: Computational comparison of 5‐*exo* versus 6‐*endo* cyclization pathways [r2SCAN‐3c(SMD:THF)//DLPNO‐CCSD(T)/def2‐TZVPP].

Interestingly, all unsaturated derivatives **6a**–**6n** exhibited significantly high‐field shifted ^13^C NMR signals at positions C2, C4, and C6─C8 (ranging from 75 to 100 ppm, see Table S9), as well as unusually high‐field shifted ^1^H NMR signals [δ(^1^H): 3–5 ppm]. These observations prompted us to investigate the electronic structure and magnetic properties of the newly synthesized imidazo[2,1,5‐*de*]quinolizine framework in more detail. Single crystals of **6b** suitable for X‐ray diffraction analysis were obtained (Figure 2a). Structural analysis revealed a slight bond length alternation within the core fragment, which is in excellent agreement with the DFT‐optimized structure.

**Figure 2:**
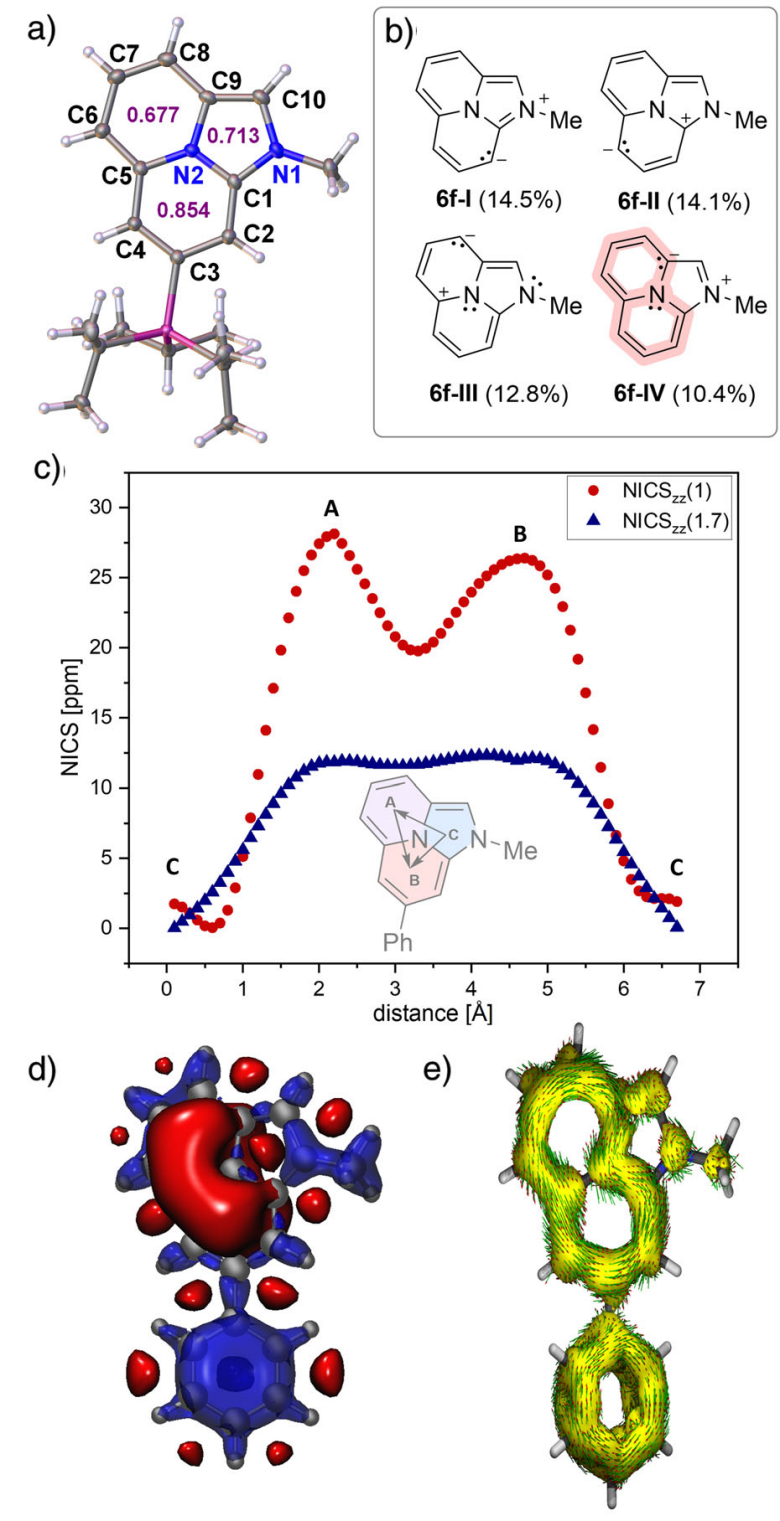
a) Solid state structure of **6b**, thermal ellipsoids are shown at 50% probability level, with respective HOMA score (purple); b) NRT calculation [r^2^SCAN‐3c(SMD:THF)//b3lyp/def2‐tzvp]; c) NICS scan at identical level of theory; d) 3D‐NICS [r^2^SCAN‐3c(SMD:THF)//CAM‐b3lyp/6–311 + g(2d,p)] iso‐surface values (visualized with VMD): blue: −20; red: +7; e) ACID plot of the π system of **6a** [r^2^SCAN‐3c(SMD:THF)// CAM‐b3lyp/6–311 + g(2d,p)].

While all C─C bonds in rings B and C are slightly longer than those typically observed in ideal aromatic systems, ring B exhibits only minor bond alternations [C1─C2 1.3965(16) Å; C2─C3 1.4123(16) Å; C3─C4 1.3965(16) Å; C4─C5 1.4209(16) Å]. In contrast, ring A shows more pronounced bond length variations [C5─C6 1.3903(16) Å; C6─C7 1.4195(17) Å; C7─C8 1.3671(18) Å], while ring C includes a notably short bond between C9 and C10 [1.3597(18) Å]. Analysis of the geometrical parameters using the Harmonic Oscillator Model of Aromaticity (HOMA, Figure 2a violet) yields positive values for all three rings (A: 0.677, B: 0.854, and C: 0.713).^[^ ^49^, ^50^, ^51^ ^]^ However, these values are significantly smaller than 1.0,^[^ ^52^, ^53^, ^54^, ^55^ ^]^ which instead reflect partial conjugation within specific regions of the imidazo[2,1,5‐*de*]quinolizine framework. Next, we performed Natural Resonance Theory (NRT) calculations on a DFT‐optimized structure of **6f**.^[^ ^56^, ^57^, ^58^, ^59^ ^]^ These calculations revealed three dominant resonance structures, all featuring a localized C9─C10 double bond—a result that aligns well with the experimentally observed bond lengths in the solid‐state structure. Additionally, NRT analysis assigns negative partial charges to positions C2,^[^ ^60^ ^]^ C4, and C8 within the heterocyclic core, in line with Mulliken and natural population analyses (Mulliken charge/natural charge [e]: C2 −0.33/−0.41, C4 −0.28/−0.33, C8 −0.29/−0.32, C1 0.26/0.39, and C5 0.19/0.21).

To assess aromaticity or antiaromaticity within the system, we performed NICSzz(1) and NICSzz(1.7) calculations on the r2SCAN‐3c(SMD:THF)‐optimized structure of **6a** (Figure 2c).^[^ ^61^, ^62^ ^]^ Rings A [NICSzz(1): +29.3 ppm] and B [NICSzz(1): +28.2 ppm] show strongly positive values — typically indicative of antiaromatic character — while ring C displays only slightly positive values [NICSzz(1): +0.9 ppm], suggesting non‐aromatic behavior in this region (Figure 2c). The shallow difference between both maxima in the NICSzz(1.7) scan further supports strong conjugation between rings A and B but limited interaction with ring C. The formation of a paratropic ring current is further corroborated by three‐dimensional NICS iso‐surface maps (Figure 2d), which reveal positive shielding contributions concentrated within the quinazoline core while showing negligible participation from the imidazole heterocycle. Similarly, ACID (anisotropy of induced current density) plots based on the same structures depict a connected paratropic ring current spanning rings A and B via the central nitrogen atom N2 (Figure 2e;^[^ ^63^, ^64^ ^]^ for ACID analysis based on the solid‐state structure of **6b** see Figure S454). This behavior aligns with a 12π‐electron/10‐atom antiaromatic system, as highlighted by the fourth leading resonance structure (**6f–IV**, Figure 2b).^[^ ^65^, ^66^, ^67^, ^68^, ^69^ ^]^

Prompted by the high charge accumulation combined with partial antiaromatic character, we investigated the electrochemical behavior of the imidazo[2,1,5‐*de*]quinolizine framework (Figure 3a).

**Figure 3:**
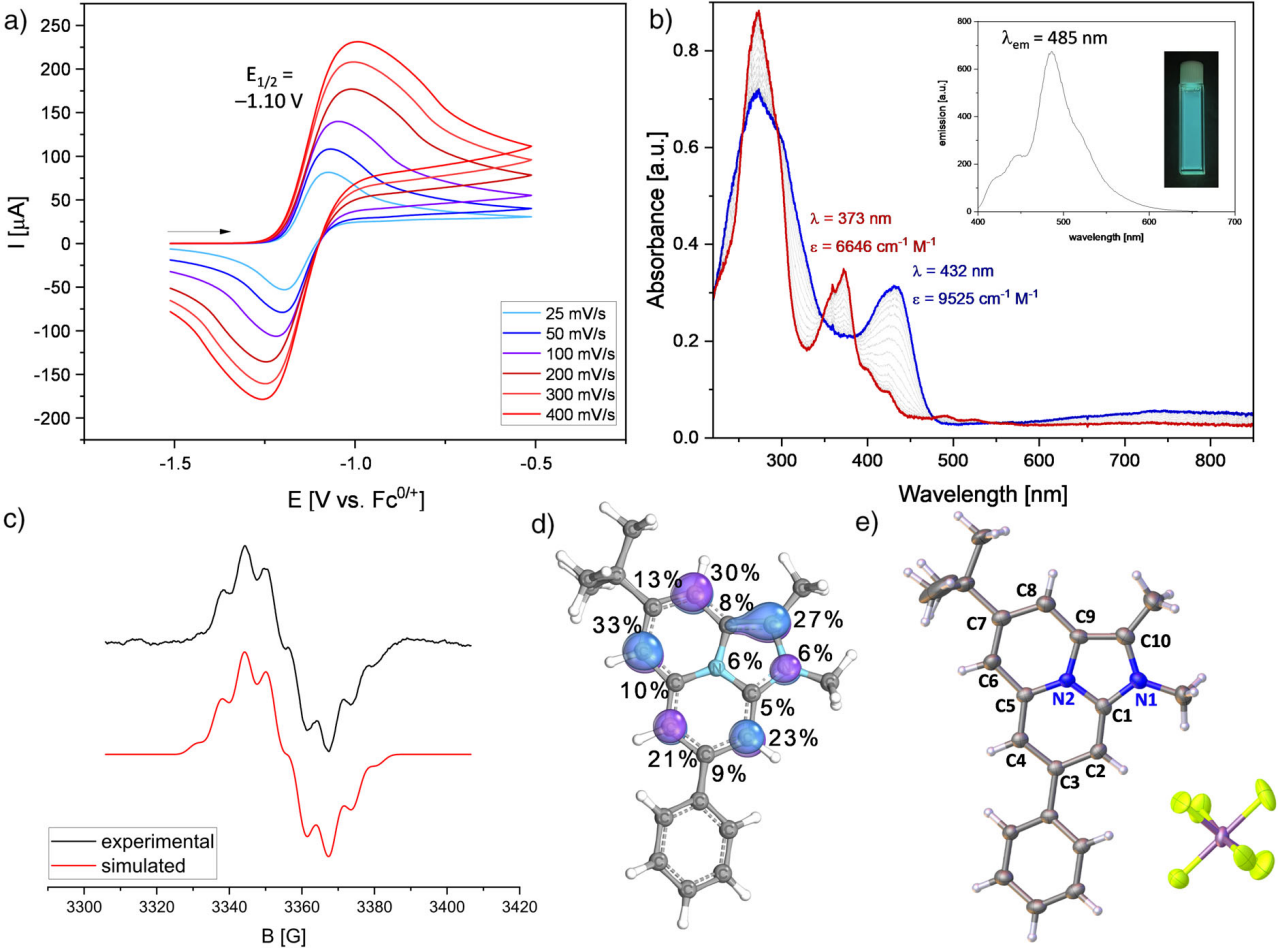
a) Scan rate dependent CV measurement of **6m** in THF (3 mg mL^−1^); b) Spectroelectrochemical measurement of **6m** (blue) to form **6m^+•^** (red) in THF, from −1.3 V (versus Ag^0/+^) with 2.0 mV s^−1^, with insert: Fluorescence spectrum of **6m^+•^** in THF, *λ*_ex _= 387 nm and picture of the cuvette under UV‐irradiation; c) X‐Band EPR spectrum of **6m^+•^** in THF (1 mM). Fitting parameter: *g* = 2.0024; LW 0.284; Hyperfine coupling: 1xN: 2.392 MHz; 1xN: 4.238 MHz; 1xH: 16.718 MHz; 1xH: 11.043 MHz, 1xH: 18.633 MHz, 1xH: 23.932 MHz, 3xH: 17.954 MHz, 3xH: 4.785 MHz; d) SOMO of **6m^+•^** with the significant Mulliken spin‐densities. Isovalue: 0.5; e) Solid state structure of **6m^+•^**, thermal ellipsoids shown at 50% probability level, disordered moieties were omitted for clarity.

All derivatives exhibited an oxidation event at low redox potentials (Figures S394–S411). This oxidation was typically irreversible except for **6m**, which showed a chemical reversible redox event at *E* ^1^_1/2_ = −1.10 V (Figure 3a), along with a second irreversible oxidation at *E* ^2^_1/2_ = −0.14 V. Spectroelectrochemical oxidation revealed a decrease in the distinct absorption of **6m** at 432 nm and the broad HOMO‐LUMO transition around 800 nm, accompanied by the emergence of a new band at 373 nm (Figure 3b). Chemical oxidation of **6m** with one equivalent of AgSbF_6_ in CH_2_Cl_2_ afforded the radical cation **6m^+˙^** in good yield (62%). EPR spectroscopy confirmed the paramagnetic nature of **6m^+•^**, revealing an intense signal with two small nitrogen hyperfine splitting constants [A(^14^N) = 2.39 and 4.24 MHz] and distinct proton hyperfine coupling constants (Figure 3c). These experimental results are in good agreement with DFT calculations, which predict that the SOMO is primarily localized on carbon atoms C2, C4, C6, C8, and C10 (Figure 3d). Notably, these positions are protected by substituents in **6m**, which grants the radical cation high robustness against intermolecular dimerization or decomposition. Single‐crystal X‐ray diffraction analysis further confirmed the monomeric nature of **6m^+•^**, even in the solid‐state (Figure 3e). Importantly, **6m^+•^** exhibited an intense blue fluorescence (*λ*_em_ = 485 nm) in solution upon exposure to UV light. This observation highlights **6m^+•^** as a promising candidate for further studies toward organic materials since the structural space of luminescent organic radicals is severely limited.^[^ ^70^, ^71^, ^72^, ^73^, ^74^ ^]^

In conclusion, we successfully developed the first synthetic approach to imidazo[1,5‐*a*]pyridine‐based NHOs. In contrast to previously reported NHOs, the C5‐position of the heterocyclic core allows to install a substituent in close proximity to the strongly polarized double bond. Investigations into C5‐alkynyl‐ and alkenyl‐substituted derivatives gave access to an unprecedented cyclization pathway, resulting in a library of novel redox‐active imidazo[2,1,5‐*de*]quinolizines that exhibit partial antiaromatic character within a 12π‐electron/10‐atom conjugated ring system. Notably, **6m** yielded a stable monomeric radical cation **6m^+•^** upon oxidation, which exhibits intense blue fluorescence—a rare property among organic radicals. The ^ImPy^NHOs ligand design should open up plenty of possibilities in coordination chemistry including main‐group and transition metals. Additionally, the work paves the way for other NHO cyclization reactions as well to explore the chemistry and applications of neutral and open‐shell imidazo[2,1,5‐*de*]quinolizines in material science.

## Supporting Information

The authors have cited additional references within the Supporting Information.

## Conflict of Interests

The authors declare no conflict of interest.

## Supporting information



Supporting Information

compound_8bCy

## Data Availability

The data that support the findings of this study are openly available in [TUDOdata] at [https://doi.org/10.17877/RESOLV‐2025‐M9I8RY5L].
