# Supplementary material for: Imidazo[1,5‐a]pyridines – A Versatile Platform for Structurally Distinct N‐Heterocyclic Olefins and π‐Extended Heterocycles
Source: Angew Chem Int Ed Engl. 2025 May 19;64(29):e202506305. doi: 10.1002/anie.202506305 (PMC12258668; doi:10.1002/anie.202506305)
Supplement: Supplementary file 1 — Supporting Information [file ANIE-64-e202506305-s002.pdf]

# Supplementary Materials for

**Imidazo[1,5-*a*]pyridines – A Versatile Platform for Structurally Distinct *N*-Heterocyclic Olefins and π-Extended Heterocycles**

Robin Esken, Patrick W. Antoni, Yannick Lorenz, Chris Burdenski, Jan-Lukas Kirchhoff, Carsten Strohmann and Max M. Hansmann*

Fakultät für Chemie und Chemische Biologie, Technische Universität Dortmund, Otto-Hahn-Str. 6, 44227 Dortmund, Germany.

## Table of Contents


1. Materials and Methods .......... 3
2. Synthetic Procedures and Characterization Data .......... 4
3. NMR Spectra .......... 74
   3.1 NMR Spectra of Isolated Compounds .......... 74
   3.2 Kinetic Experiments .......... 267
4. UV-Vis Spectroscopy .......... 268
5. EPR Spectroscopy .......... 279
6. Cyclic Voltammetry .......... 281
   6.1 Reversibility Investigations of the First Redox Event of **6m** .......... 291
   6.2 Solvent-Influence on **6m** .......... 296
7. Spectroelectrochemistry .......... 300
8. X-Ray Characterization Data .......... 301
9. Computational Section .......... 310
   9.1 Mechanistic Investigation .......... 311
   9.2 Determination of NICS Values and GIAO Calculations .......... 332
   9.3 ACID plots .......... 334
   9.4 NBO / NRT calculations .......... 337
   9.5 TD-DFT calculation .......... 368
10. Literature .......... 377

## 1. Materials and Methods

All solvents were purified by distillation over the drying agents indicated, or stored over molecular sieves and degassed with argon. Reactions were carried out either under $N_2$ or Ar atmosphere. Solids were handled and NMR samples were prepared in a nitrogen filled glovebox. High resolution MS (ESI): Finnigan MAT 95, accurate mass determinations: Bruker APEX III FT-MS (7 T magnet) and LTQ-Orbitrap-XL (Thermo Scientific) equipped with a heated electrospray ionization source (HESI). NMR: NMR spectra were measured on the spectrometers Bruker AV 500 Avance NEO and AV 600 Avance III HD and chemical shifts (δ) are referenced to their solvent signals [$C_6D_6$, 7.16 ($^1$H NMR) 128.06 ($^{13}$C NMR); $CD_3CN$, 1.94 ($^1$H NMR) 118.26 ($^{13}$C NMR); $d_6$-DMSO, 2.50 ($^1$H NMR), 39.52 ($^{13}$C NMR); $CDCl_3$, 7.26 ($^1$H NMR) 77.16 ($^{13}$C NMR); $d_8$-THF, 3.58, 1.73 ($^1$H NMR) and 67.57 ($^{13}$C NMR); $CD_2Cl_2$, 5.32 ($^1$H NMR) 53.84 ($^{13}$C NMR)], coupling constants ($J$) in Hz. $^{15}$N chemical shifts are referenced against liquid $NH_3$. All spectra were recorded in 5 mm NMR tubes at the temperatures indicated. The solvent signals were used as references and the chemical shifts converted to the TMS scale. UV-Vis spectra were recorded on an Agilent Cary60. Fluorescence spectra were recorded on an Agilent Cary Eclipse Fluorescence Spectrometer. Flash chromatography was performed with Merck 60 silica gel (40-63 µm). Thin-layer chromatography (TLC) analysis was performed using Merck silica gel 60 F254 TLC plates and visualized by UV irradiation and/or ceric ammonium molybdate, $KMnO_4$ or *p*-anisaldehyde. All commercially available compounds (Acros, ABCR, Alfa Aesar, Sigma Aldrich, Fluorochem, BLDpharm) were used as received. IR-ATR measurements (diamond) were performed in reflection mode on a Bruker Alpha II inside a glovebox, wavenumbers in $cm^{-1}$. Solution IR spectra were recorded with 1 mg $mL^{-1}$ solution in $CH_2Cl_2$ using a transmission cell with KBr windows. Melting points were measured with a Büchi M-560 apparatus.

## 2. Synthetic Procedures and Characterization Data

### 2.1 Overview of synthetic routes of NHO precursors

**Route 1**:

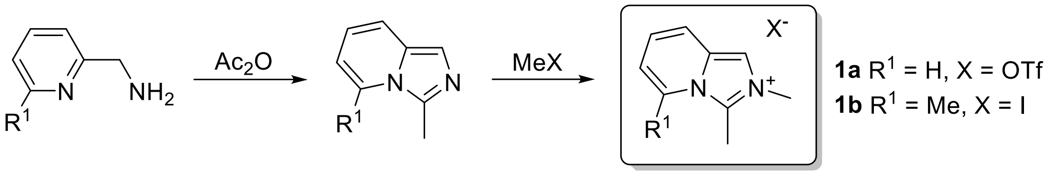


**Route 2**:

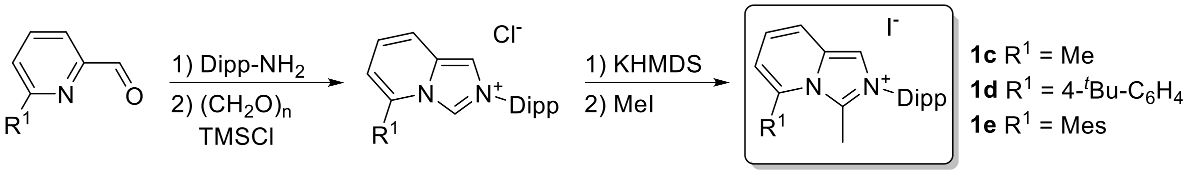


**Route 3**:

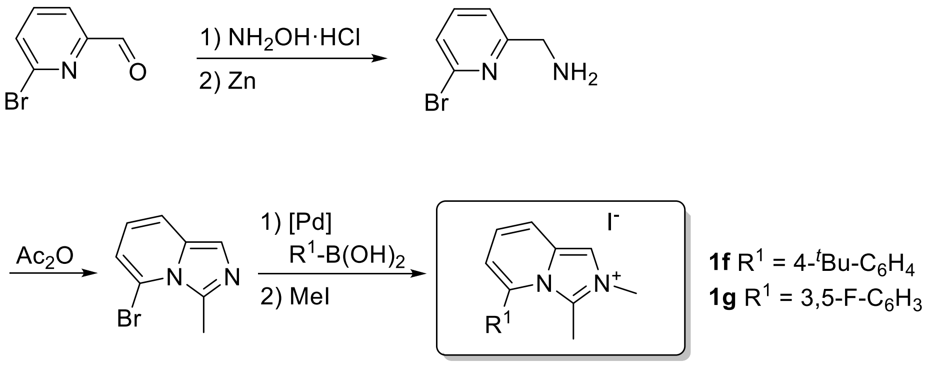


**Scheme S1**: Synthesis of ImPyNHO precursors.

### 2.2 Overview of synthetic routes of imidazo[2,1,5-*de*]quinolizine precursors

**Route 1**:

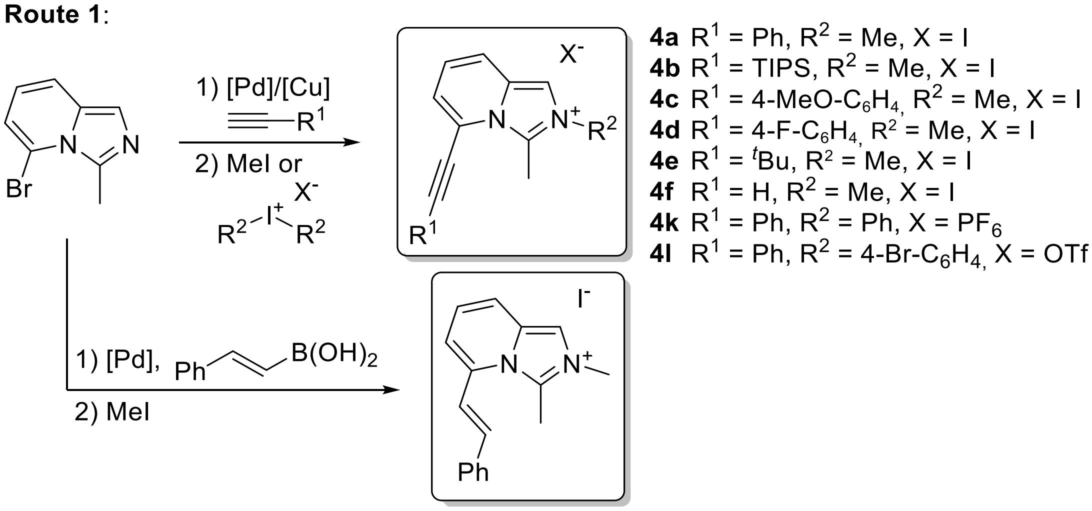


**4o**

**Route 2**:

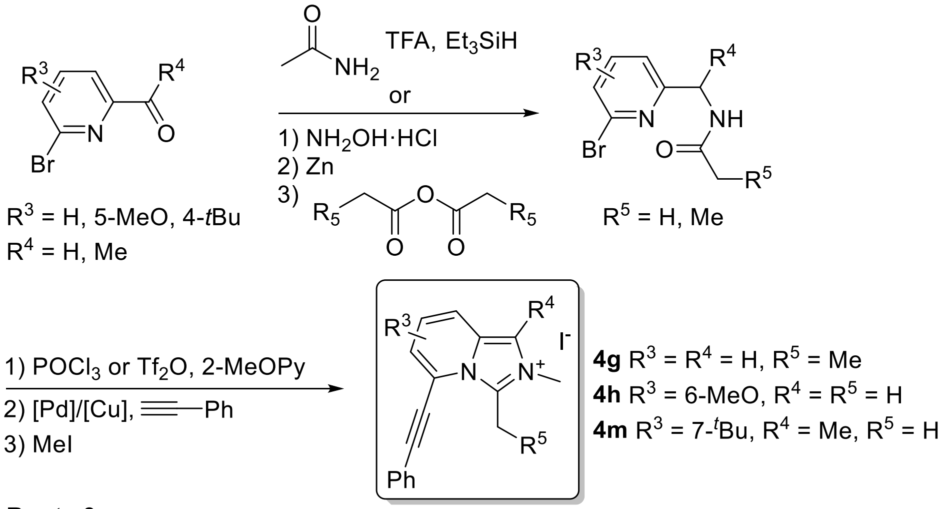


**Route 3**:

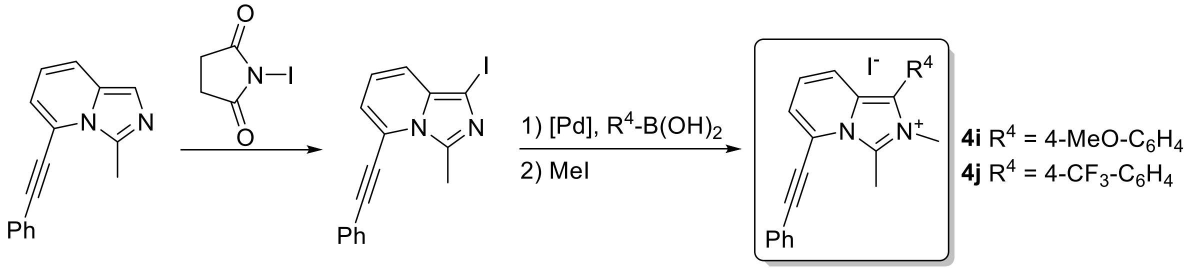


**Route 4**:

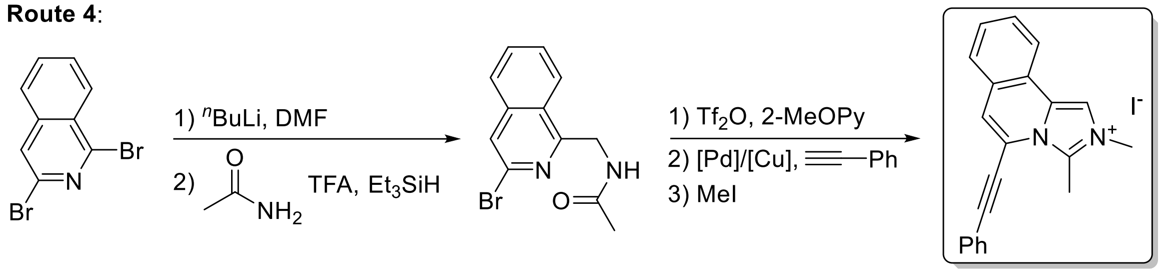


**4n**

**Route 5**:

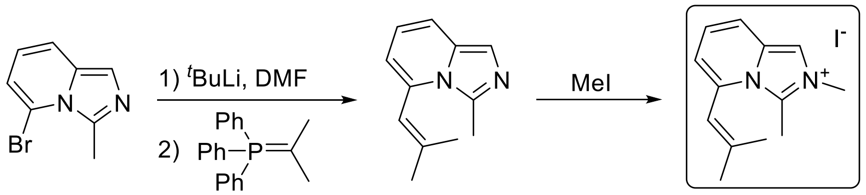


**4p**

**Scheme S2**: Synthesis of imidazo[2,1,5-*de*]quinolizine precursors.

## 2.3 Synthesis of Imidazo[1,5-*a*]pyridinium/-isoquinolinium Salts

Synthesis of **S2**

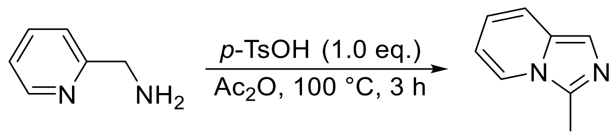


2-Picolylamine (**S1**, 5.00 g, 46.2 mmol, 1.00 eq.) and *p*-TsOH monohydrate (8.79 g, 46.2 mmol, 1.0 eq.) were dissolved in $Ac_2O$ (25 mL) and stirred at 100 °C for 3 h. The dark blue reaction mixture was poured into water (100 mL) and made alkaline using 20w% NaOH. The dark yellow solution was extracted with $CH_2Cl_2$ (3x50 mL), the combined organic phases were washed with water (50 mL) and dried over $Na_2SO_4$. Removal of the solvent under reduced pressure furnished **S2** as a dark yellow sticky liquid that solidified after cooling to –25 °C for 5 minutes. The crude product was further purified by recrystallisation from $Et_2O$ at –25 °C, furnishing **S2** as a bright yellow crystalline solid (4.10 g, 31.0 mmol, 67%). The spectroscopic data agrees with literature data.[1]

**$^1$H NMR** (500 MHz, $CDCl_3$): δ [ppm] = 7.40 – 7.33 (m, 1H, ImPy-H), 7.18 (dt, *J* = 9.2, 1.3 Hz, 1H, ImPy-H), 7.16 (s, 1H, ImPy-H), 6.39 (ddd, *J* = 9.2, 6.3, 1.0 Hz, 1H, ImPy-H), 6.20 (ddd, *J* = 7.4, 6.3, 1.2 Hz, 1H, ImPy-H), 2.41 (s, 3H, ImPy-$CH_3$). **$^{13}$C NMR** (126 MHz, $CDCl_3$): δ [ppm] = 132.7 (ImPy-$C_q$), 128.2 (ImPy-$C_q$), 118.7 (ImPy-*C*H), 116.2 (ImPy-*C*H), 116.0 (ImPy-*C*H), 115.4 (ImPy-*C*H), 109.7 (ImPy-*C*H), 10.3 (ImPy-$CH_3$).

Synthesis of **1a**

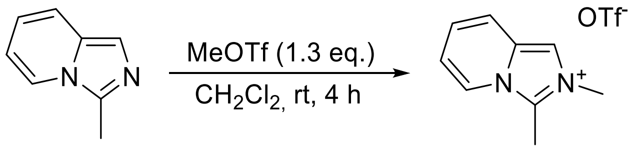


To a solution of **S2** (500 mg, 3.78 mmol, 1.00 eq.) in $CH_2Cl_2$ (5 mL) was added MeOTf (807 mg, 538 µL, 3.56 mmol, 1.30 eq.) at 0 °C. The reaction mixture was warmed to rt and stirred for 4 h. The solvent was removed under reduced pressure and the remaining off-white solid was washed twice with $Et_2O$ (2x20 mL). Drying under reduced pressure furnished **1a** as a colorless solid (847 mg, 2.86 mmol, 75%).

**m.p.** 143 °C (decomp.); **$^1$H NMR** (500 MHz, $CD_3CN$): δ [ppm] = 8.08 (dq, *J* = 7.3, 1.1 Hz, 1H, ImPy-H), 7.73 (s, 1H, ImPy-H), 7.67 (dt, *J* = 9.4, 1.2 Hz, 1H, ImPy-H), 7.17 (ddd, *J* = 9.4, 6.7, 0.9 Hz, 1H, ImPy-H), 7.09 – 7.06 (m, 1H, ImPy-H), 4.00 (s, 3H, N-$CH_3$), 2.79 (s, 3H, ImPy-$CH_3$); **$^{13}$C NMR** (126 MHz, $CD_3CN$): δ [ppm] = 129.8 (ImPy-$C_q$), 124.9 (ImPy-*C*H), 123.1 (ImPy-*C*H), 120.9 (ImPy-$C_q$), 119.0 (ImPy-*C*H), 118.0 (ImPy-*C*H), 114.3 (ImPy-*C*H), 36.7 (N-$CH_3$),

9.8 (ImPy-$CH_3$); **IR** [$cm^{-1}$]: ṽ = 1656.4, 1268.9, 1222.91171.3, 1137.5, 1032.2, 841.4, 767.3, 747.9, 633.9, 571.4, 515.9, 432.8; **HR-MS-ESI(+)** calc. $C_9H_{11}N_2^+$ $[M]^+$ 147.0917; found 147.0915.

Synthesis of **S4**

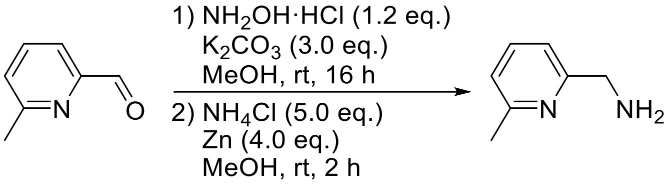


To a suspension of 6-methylpicolinaldehyde (**S3**, 5.00 g, 41.2 mmol, 1.0 eq.) and potassium carbonate (17.1 g, 124 mmol, 3.0 eq.) in methanol (100 mL) was added hydroxylammonium hydrochloride (3.44 g, 49.5 mmol, 1.2 eq.) and the reaction mixture was stirred overnight. After ammonium chloride (11.0 g, 206 mmol, 5.0 eq.) was added, zinc (10.8 g, 165 mmol, 4.0 eq.) was added all at once, leading to an exothermic reaction. After 2 hours the mixture was filtrated and the solvent of the filtrate was removed under reduced pressure. The crude product was then dissolved in $CH_2Cl_2$ (25 mL) and afterwards hydrochloric acid (15 mL, 37%) was added and the solution was stirred for 15 minutes. Aqueous sodium hydroxide (80 mL, 20w%) was then added, the solution was subsequently extracted with $CH_2Cl_2$ thrice (3x100 mL), dried with $Na_2SO_4$ and the solvent was removed under reduced pressure to furnish **S4** as pale-yellow oil (4.70 g, 41.3 mmol, 93%). The spectroscopic data agrees with literature data.[2]

**$^1$H NMR** (500 MHz, $CDCl_3$): δ [ppm] = 7.49 (t, $J$ = 7.6 Hz, 1H, Py-H), 7.04 (d, $J$ = 7.6 Hz, 1H, Py-H), 6.97 (d, $J$ = 7.6 Hz, 1H, Py-H), 3.89 (s, 2H, Py-$CH_2$-), 2.50 (s, 3H, Py-$CH_3$), 1.78 (s, 2H, -$NH_2$).; **$^{13}$C NMR** (126 MHz, $CDCl_3$): δ [ppm] = 161.4 (Py-$C_q$), 158.0 (Py-$C_q$), 136.9 (Py-*C*H), 121.4 (Py-*C*H), 118.1 (Py-*C*H), 48.0 (Py-$CH_2$-), 24.5 (Py-$CH_3$).

Synthesis of **S5**

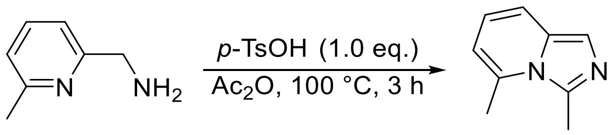


A mixture of **S4** (4.00 g, 32.7 mmol, 1.0 eq.) and *p*-TsOH monohydrate (6.54 g, 32.7 mmol, 1.0 eq.) were dissolved in $Ac_2O$ (20 mL) and stirred at 100 °C for 3 h. The dark blue reaction mixture was poured into water (100 mL) and made alkaline using 20w% NaOH. The now dark yellow solution was extracted with $CH_2Cl_2$ (3 x 50 mL), the combined organic phases were washed with water (50 mL) and dried over $Na_2SO_4$. Removal of the solvent under reduced pressure furnished **S5** as a dark yellow sticky liquid that solidified after cooling to –25 °C for 5

minutes. The crude product can be further purified by recrystallisation form $Et_2O$, furnishing **S5** as a bright yellow crystalline solid (3.70 g, 25.3 mmol, 77%).

**m.p.** 67 °C; **$^1$H NMR** (500 MHz, $CDCl_3$): δ [ppm] = 7.23 (s, 1H, ImPy-H), 7.19 (d, *J* = 9.1 Hz, 1H, ImPy-H), 6.45 (dd, *J* = 9.1, 6.4 Hz, 1H, ImPy-H), 6.17 – 6.11 (m, 1H, ImPy-H), 2.97 (s, 3H, ImPy-$CH_3$), 2.76 (s, 3H, ImPy-$CH_3$); **$^{13}$C NMR** (126 MHz, $CDCl_3$): δ [ppm] = 136.5 (ImPy-$C_q$), 132.9 (ImPy-$C_q$), 132.6 (ImPy-$C_q$), 118.4 (ImPy-*C*H, 2C), 116.9 (ImPy-*C*H), 113.0 (ImPy-*C*H), 20.9 (ImPy-$CH_3$), 18.1 (ImPy-$CH_3$); **IR** [cm$^{-1}$]: ṽ = 2978.6, 1640.5, 1535.9, 1450.1, 1409.2, 1389.9, 1368.9, 1337.0, 1303.7, 1270.4, 1243.6, 1169.1, 1148.2, 1077.5, 1039.4, 981.6, 966.1, 836.7, 780.1, 757.9, 711.1, 661.7, 565.9; **HR-MS-ESI(+)** calc. $C_9H_{11}N_2^+$ $[M+H]^+$ 147.0917; found 147.0915.

Synthesis of **1b**

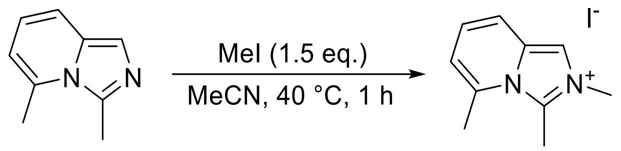


To a solution of **S5** (1.50 g, 10.3 mmol, 1.00 eq.) in MeCN (10 mL) was added MeI (2.18 g, 960 µL, 15.5 mmol, 1.50 eq.). The mixture was stirred for 1 h at 40 °C. The initially clear solution became turbid after 15 min and solidified completely after 45 min. $Et_2O$ (40 mL) was added and the solid block was crushed to small pieces. The solution was filtered off and the remaining off-white solid was washed thrice with $Et_2O$ (3x30 mL). Drying under reduced pressure furnished **1b** as a slightly green solid (2.60 g, 9.02 mmol, 88%).

**m.p.** 218 °C (decomp.); **$^1$H NMR** (500 MHz, $CD_3CN$): δ [ppm] = 7.78 (s, 1H, ImPy-H), 7.52 (d, *J* = 9.3 Hz, 1H, ImPy-H), 7.01 (dd, *J* = 9.3, 6.8 Hz, 1H, ImPy-H), 6.76 (dt, *J* = 6.8, 1.2 Hz, 1H, ImPy-H), 4.00 (s, 3H, N-$CH_3$), 3.08 (s, 3H, ImPy-$CH_3$), 2.86 (s, 3H, ImPy-$CH_3$); **$^{13}$C NMR** (126 MHz, $CD_3CN$): δ [ppm] = 136.6 (ImPy-$C_q$), 135.5 (ImPy-$C_q$), 131.6 (ImPy-$C_q$), 125.1 (ImPy-*C*H), 118.8 (ImPy-*C*H), 117.1 (ImPy-*C*H), 114.6 (ImPy-*C*H), 37.4 (N-$CH_3$), 21.0 (ImPy-$CH_3$), 14.0 (ImPy-$CH_3$); **IR** [cm$^{-1}$]: ṽ = 3049.2, 1656.8, 1551.0, 1444.9, 1418.8, 1398.3, 1171.0, 1131.3, 1088.0, 813.8, 721.6, 639.6; **HR-MS-ESI(+)** calc. $C_{10}H_{13}N_2^+$ $[M]^+$ 161.1073; found 161.1072.

Synthesis of **S6**

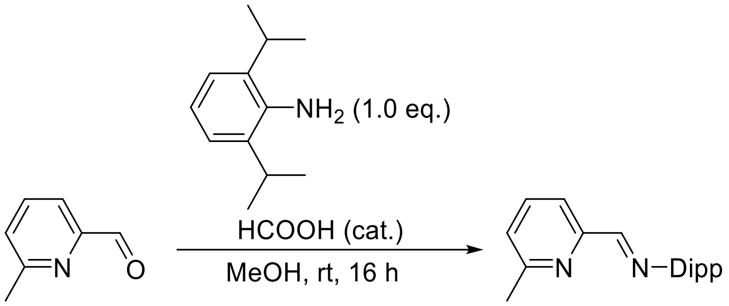


A mixture of 2,6-diisopropylaniline (7.76 g, 8.25 mL, 43.8 mmol, 1.0 eq.), 6-methyl-2-formylpyridine (**S3**, 5.3 g, 43.8 mmol, 1.0 eq.) and 5 drops of formic acid in MeOH (150 mL) was stirred 16 h at rt. The solvent was removed under reduced pressure and the resulting, highly viscous yellow paste was subjected to high vacuum at 40 °C for 30 min. The crude product was then recrystallized from MeOH (50 mL) at –25 °C, furnishing **S6** as crystalline yellow solid (5.5 g, 19.6 mmol, 45%).

**m.p.** 82 °C; **$^{1}$H NMR** (600 MHz, $CDCl_3$): δ [ppm] = 8.30 (s, 1H, C(*H*)=N), 8.10 (d, *J* = 7.7 Hz, 1H, Py-*H*), 7.73 (t, *J* = 7.7 Hz, 1H, Py-*H*), 7.27 (d, *J* = 7.7 Hz, 1H, Py-*H*), 7.17 (dd, *J* = 8.1, 1.3 Hz, 2H, Ar-*H*), 7.12 (ddd, *J* = 8.2, 6.6, 1.2 Hz, 1H, Ar-*H*), 2.98 (hept, *J* = 6.9 Hz, 2H, C*H*($CH_3$)$_2$), 2.64 (s, 3H, Py-C$H_3$), 1.18 (d, *J* = 6.9 Hz, 12H, CH(C$H_3$)$_2$); **$^{13}$C NMR** (151 MHz, $CDCl_3$): δ [ppm] = 163.3 (*C*(H)=N), 158.6 (Py-$C_q$), 154.0 (Py-$C_q$), 148.6 (Dipp-$C_q$), 137.3 (Py-*C*H), 137.0 (Dipp-$C_q$), 125.1 (Py-*C*H), 124.5 (Dipp-*C*H), 123.1 (Dipp-*C*H), 118.5 (Py-*C*H), 28.0 (*C*H($CH_3$)$_2$), 24.5 (Py-*C*$H_3$), 23.5 (CH(*C*$H_3$)$_2$); **IR** [$cm^{-1}$]: ṽ = 2958.8, 1639.9, 1587.0, 1572.5, 1453.9, 1362.3, 1319.6, 1187.1, 1148.1, 1088.1, 986.8, 855.1, 795.0, 774.0, 746.1; **HR-MS-ESI(+)** calc. $C_{19}H_{25}N_2^+$ $[M+H]^+$ 281.2012; found 281.2014.

Synthesis of **S7**

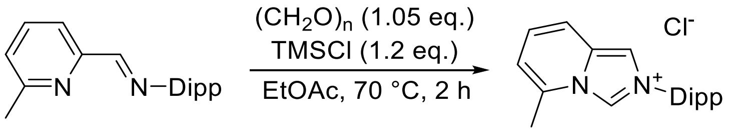


**S6** (4.00 g, 14.3 mmol, 1.0 eq.) and paraformaldehyde (471 mg; 15.7 mmol; 1.05 eq.) were dissolved in EtOAc (100 mL) and heated to 70 °C under argon atmosphere. TMSCl (2.01 g, 18.5 mmol, 1.30 eq.) was added dropwise, leading to instantaneous formation of a colorless precipitate. The reaction mixture was stirred for 2 h, cooled to rt and filtered over a glass frit. The colorless fine powdered crude product was washed twice with $Et_2O$ (2x20 mL) and dried under reduced pressure, furnishing **S7** as a colorless solid (3.80 g, 11.6 mmol, 81%).

**Note**: If the compound is yellow after the reaction, dissolve it in the minimum amount $CH_2Cl_2$, add $Et_2O$ and filtrate it (repeat it thrice), causing the compound to become colorless.

**m.p.** 294 °C (decomp.)**;** **$^1$H NMR** (700 MHz, $CDCl_3$): δ [ppm] = 10.89 (s, 1H, ImPy-NC(H)N), 7.91 (d, J = 1.7 Hz, 1H, ImPy-CH), 7.87 (d, J = 9.3 Hz, 1H, ImPy-CH), 7.47 (t, J = 7.9 Hz, 1H, $Ar_{Dipp}$-CH), 7.24 (d, J = 7.9 Hz, 2H, $Ar_{Dipp}$-CH), 7.22 (dd, J = 9.4, 6.9 Hz, 1H, ImPy-CH), 6.91 (d, J = 6.9 Hz, 1H, ImPy-CH), 2.92 (s, 3H, ImPy-$CH_3$), 2.07 (hept, *J* = 6.8 Hz, 2H, C*H*($CH_3$)$_2$), 1.15 (d, *J* = 6.9 Hz, 6H, CH(C$H_3$)$_2$), 1.04 (d, *J* = 6.9 Hz, 6H, CH(C$H_3$)$_2$); **$^{13}$C NMR** (176 MHz, $CDCl_3$): δ [ppm] = 145.0 (Dipp-$C_q$), 134.3 (ImPy-$C_q$), 131.8 (Dipp-*C*H), 130.9 (ImPy-$C_q$), 127.3 (Dipp-$C_q$), 127.2 (ImPy-*C*H), 126.2 (ImPy-*C*H), 124.4 (Dipp-*C*H), 117.4 (ImPy-*C*H), 116.3 (ImPy-*C*H), 115.8 (ImPy-*C*H), 28.6 (*C*H($CH_3$)$_2$), 24.5 (CH(*C*$H_3$)$_2$), 24.3 (CH(*C*$H_3$)$_2$), 19.6 (ImPy-*C*$H_3$); **IR** [$cm^{-1}$]: ṽ = 3141.7, 2967.1, 1662.9, 1561.6, 1459.6, 1393.0, 1357.2, 1326.9, 1222.8, 1185.5, 1165.8, 1050.8, 1026.6, 939.5, 809.9, 762.9, 672.3, 570.9, 521.2, 488.5; **HR-MS-ESI(+)** calc. $C_{20}H_{25}N_2$ $[M]^+$ 293.2012; found 293.2013.

Synthesis of **1c**

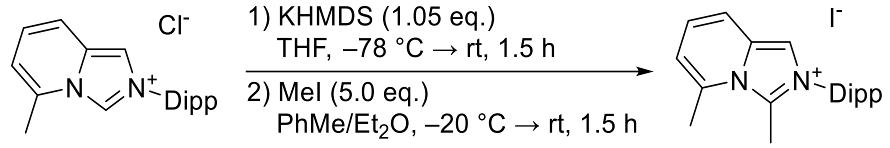


To a mixture of **S7** (1.50 g, 3.94 mmol, 1.0 eq.) and KHMDS (826 mg, 4.14 mmol; 1.05 eq.) was added THF (40 mL) at –78 °C. The mixture was stirred for 5 min, before it was warmed to rt and stirred for additional 1.5 h, resulting in a slightly yellow suspension. The solvent was removed under reduced pressure. The resulting sticky off-white solid was redissolved in toluene (20 mL + 10 mL) and transferred to a second Schlenk flask containing a cooled (–20 °C) solution of $CH_3I$ (1.3 mL, 19.7 mmol, 5.0 eq.) in $Et_2O$ (20 mL) by filter cannula. After complete addition the mixture was warmed to rt and stirred for 1 h. The solvent was filtered off and the remaining colorless solid was washed twice with $Et_2O$ (2x20 mL). Drying the crude product under reduced pressure gave **1c** (1.35 g, 3.11 mmol, 78%) as colorless solid.

**m.p.** 293 °C; **$^1$H NMR** (600 MHz, $CD_3CN$): δ [ppm] = 7.93 (s, 1H, ImPy-C*H*), 7.67 (t, *J* = 7.8 Hz, 1H, Dipp-C*H*), 7.63 (d, *J* = 9.3 Hz, 1H, ImPy-*CH*), 7.49 (d, *J* = 7.9 Hz, 2H, Dipp-C*H*), 7.15 (dd, *J* = 9.3, 6.8 Hz, 1H, ImPy-C*H*), 6.91 (d, *J* = 6.8 Hz, 1H, ImPy-C*H*), 2.96 (s, 3H, ImPy-C$H_3$), 2.87 (s, 3H, ImPy-C$H_3$), 2.17 (hept, *J* = 6.8 Hz, 2H,C*H*($CH_3$)$_2$), 1.15 (d, *J* = 6.8 Hz, 6H, CH(C$H_3$)$_2$), 1.11 (d, *J* = 6.8 Hz, 6H, CH(C$H_3$)$_2$); **$^{13}$C NMR** (151 MHz, $CD_3CN$): δ [ppm] = 146.4 (Dipp-$C_q$), 137.0 (ImPy-$C_q$), 136.6 (ImPy-$C_q$), 133.0 (Dipp-*C*H), 132.6 (ImPy-$C_q$), 131.0 (Dipp-$C_q$), 126.2 (ImPy-*C*H), 125.9 (Dipp-*C*H), 119.2 (ImPy-*C*H), 117.4 (ImPy-*C*H), 115.0 (ImPy-*C*H), 29.0 (*C*H($CH_3$)$_2$), 24.9 (CH(*C*$H_3$)$_2$), 23.4 (CH(*C*$H_3$)$_2$), 20.9 (ImPy-$CH_3$), 14.3 (ImPy-

$CH_3$); **IR** [$cm^{-1}$]: ṽ = 3064.1, 2960.3, 2869.4, 1659.6, 1554.7, 1500.7, 1458.9, 1425.6, 1395.9, 1367.4, 1309.3, 1242.5, 1178.7, 1145.9, 1111.0, 1089.0, 1077.2, 1058.5, 1048.4, 999.7, 936.4, 867.9, 814.5, 796.0, 776.8, 758.9, 719.5, 682.0, 611.3, 591.8, 499.1, 439.4; **HR-MS-ESI(+)** calc. $C_{21}H_{27}N_2^+$ $[M]^+$ 307.2169; found 307.2169.

Synthesis of **S9**

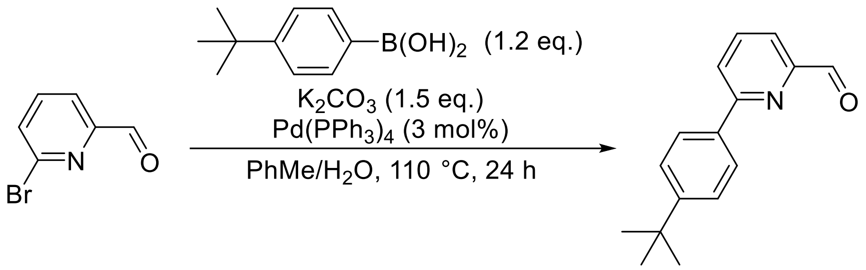


**S8** (2.00 g, 10.8 mmol, 1.00 eq.), (4-(*tert*-butyl)phenyl)boronic acid (2.30 g, 12.9 mmol, 1.20 eq.) and $K_2CO_3$ (2.23 g, 16.1 mmol, 1.50 eq.) were dissolved in a mixture of toluene and water (4:1, 100 mL). After degassing the mixture, $Pd(PPh_3)_4$ (373 mg, 322 µmol, 3 mol%) was added at once. The reaction mixture was heated to 110 °C for 24 h. After separation of the phases the aqueous phase was extracted twice with $CH_2Cl_2$ (50 mL). The combined organic phases were dried over $Na_2SO_4$, the solvent was removed under reduced pressure and the resulting yellow solid purified by column chromatography (silica, 1% EtOAc in cyclohexane to 5%). **S9** (2.30 g, 8.36 mmol, 78%) was obtained as colorless solid. The spectroscopic data agrees with literature data.[3]

**$^1$H NMR** (500 MHz, $CDCl_3$): δ [ppm] = 10.17 (d, *J* = 0.7 Hz, 1H, C*H*O), 8.02 (d, *J* = 8.5 Hz, 2H, Ar-*H*), 7.95 – 7.86 (m, 3H, Py-*H*), 7.55 (d, *J* = 8.5 Hz, 2H, Ar-*H*), 1.38 (s, 9H, C(C*H*$_3$)$_3$); **$^{13}$C NMR** (126 MHz, $CDCl_3$): δ [ppm] = 194.2 (Py-*C*HO), 158.1 (Py-$C_q$), 153.0 (Ar-$C_q$), 152.8 (Py-$C_q$), 137.8 (Py-*C*H), 135.5 (Ar-$C_q$), 126.8 (Ar-*C*H), 126.0 (Ar-*C*H), 124.4 (Py-*C*H), 119.6 (Py-*C*H), 34.9 (*C*(CH$_3$)$_3$), 31.4 (C(*C*H$_3$)$_3$).

Synthesis of **S10**

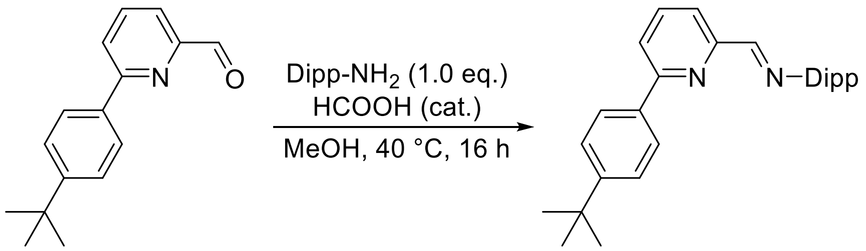


A mixture of 2,6-diisopropylaniline (1.48 g, 1.58 mL, 8.36 mmol, 1.0 eq.), **S9** (2.00 g, 8.36 mmol, 1.0 eq.) and 2 drops of formic acid in MeOH (50 mL) was stirred for 16 h at 40 °C. The solvent was removed under reduced pressure yielding a pale-yellow solid. The crude

product was then recrystallized from MeOH (50 mL) at –25 °C, furnishing **S10** as crystalline yellow solid (2.90 g, 7.28 mmol, 87%).

**m.p.** 93 °C; **$^{1}$H NMR** (500 MHz, $CDCl_3$): δ [ppm] = 8.41 (s, 1H, *C*(H)=N-Dipp), 8.23 (dd, *J* = 7.3, 0.8 Hz, 1H, Py-H), 8.04 – 7.98 (m, 2H, Ar-H), 7.93 – 7.86 (m, 1H, Ar-H), 7.83 (dd, *J* = 7.9, 1.1 Hz, 1H, Py-H), 7.57 – 7.51 (m, 2H, Ar-H), 7.21 – 7.18 (m, 2H, Ar-H), 7.17 – 7.11 (m, 1H, Py-H), 3.02 (hept, *J* = 6.9 Hz, 2H, $CH(CH_3)_2$), 1.38 (s, 9H, $C(CH_3)_3$), 1.20 (d, *J* = 6.9 Hz, 12H, $CH(CH_3)_2$).; **$^{13}$C NMR** (126 MHz, $CDCl_3$): δ [ppm] = 163.9 (*C*(H)=N-Dipp), 157.6 (Ar-$C_q$), 154.5 (Py-$C_q$), 152.6 (Ar-$C_q$), 148.7 (Ar-$C_q$), 137.5 (Py-$C_q$), 137.4 (Ar-*C*H), 136.3 (Ar-$C_q$), 126.9 (Ar-*C*H), 126.0 (Ar-*C*H), 124.4 (Py-*C*H), 123.2 (Ar-*C*H), 121.9 (Py-*C*H), 119.2 (Py-*C*H), 34.9 ($C(CH_3)_3$), 31.4 ($C(CH_3)_3$), 28.1 ($CH(CH_3)_2$), 23.6 ($CH(CH_3)_2$). **IR** [$cm^{-1}$]: ṽ = 2960.6, 1636.0, 1587.0, 1559.4, 1455.6, 1361.3, 1308.2, 1267.9, 1180.6, 1161.3, 1120.9, 1011.1, 990.2, 932.9, 893.4, 850.4, 798.7, 768.8, 750.8, 704.7, 641.9, 565.8, 535.3; **HR-MS-ESI(+)** calc. $C_{28}H_{35}N_2$ $[M+H]^+$ 399.2795 found 399.2791.

Synthesis of **S11**

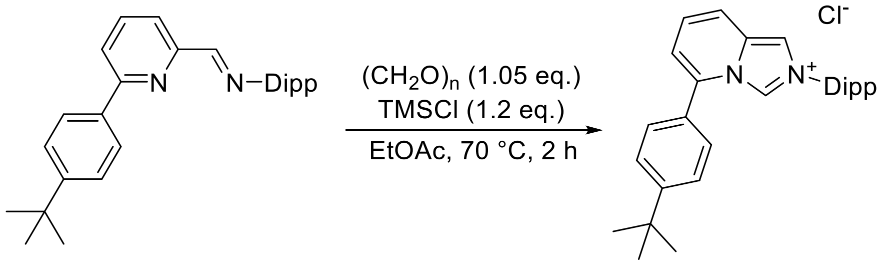


**S10** (2.50 g, 6.27 mmol, 1.0 eq.) and paraformaldehyde (207 mg; 6.90 mmol; 1.05 eq.) were dissolved in EtOAc (50 mL) and heated to 70 °C under argon atmosphere. TMSCl (885 mg, 1.03 mL, 8.15 mmol, 1.30 eq.) was added dropwise, leading to instantaneous formation of a colorless precipitate. The reaction mixture was stirred for 2 h, cooled to rt and filtered over a glass frit. The colorless residue was washed thrice with $Et_2O$ (3 x 25 mL) and dried under reduced pressure, furnishing **S11** as a colorless solid (2.10 g, 4.70 mmol, 75%).

**m.p.** 326 °C**;** **$^{1}$H NMR** (500 MHz, $CD_3CN$): δ [ppm] = 9.20 (d, *J* = 1.0 Hz, 1H, ImPy-H), 8.14 (d, *J* = 1.8 Hz, 1H, ImPy-H), 7.89 (d, *J* = 9.4 Hz, 1H, ImPy-H), 7.67 – 7.64 (m, 4H, Ar-H), 7.62 (t, *J* = 7.9 Hz, 1H, Ar-H), 7.48 (dd, *J* = 9.4, 7.0 Hz, 1H, ImPy-H), 7.43 (d, *J* = 7.8 Hz, 2H, Ar-H), 7.21 (dd, *J* = 6.9, 1.0 Hz, 1H, ImPy-H), 2.26 (hept, *J* = 6.8 Hz, 2H, $CH(CH_3)_2$), 1.35 (s, 9H, $C(CH_3)_3$), 1.16 (d, *J* = 6.8 Hz, 6H, $CH(CH_3)_2$), 1.12 (d, *J* = 6.8 Hz, 6H, $CH(CH_3)_2$); **$^{13}$C NMR** (126 MHz, $CD_3CN$): δ [ppm] = 155.3 (Ar-$C_q$), 146.4 (Ar-$C_q$), 137.2 (ImPy-$C_q$), 132.9 (ImPy-$C_q$), 132.9 (Ar-*C*H), 131.9 (Ar-$C_q$), 129.5 (Ar-$C_q$), 129.2 (Ar-*C*H), 127.7 (Ar-*C*H), 127.5 (ImPy-*C*H), 126.2 (Ar-$C_q$), 125.4 (Ar-*C*H and ImPy-*C*H (2 carbons)), 119.5 (ImPy-*C*H), 118.2 (ImPy-*C*H), 117.3 (ImPy-*C*H), 35.6 ($C(CH_3)_3$), 31.4 ($C(CH_3)_3$), 29.1 ($CH(CH_3)_2$), 24.4 ($CH(CH_3)_2$), 24.2

$(CH(CH_3)_2)$.; **IR** [$cm^{-1}$]: ṽ = 2961.0, 2867.9, 1651.9, 1463.1, 1363.8, 1298.1, 1163.4, 1123.6, 1104.6, 827.3, 808.6, 762.9, 750.4, 676.7, 616.4, 569.0, 557.8, 492.2; **HR-MS-ESI(+)** calc. $C_{29}H_{35}N_2^+$ $[M]^+$ 411.2795; found 411.2788.

Synthesis of **1d**

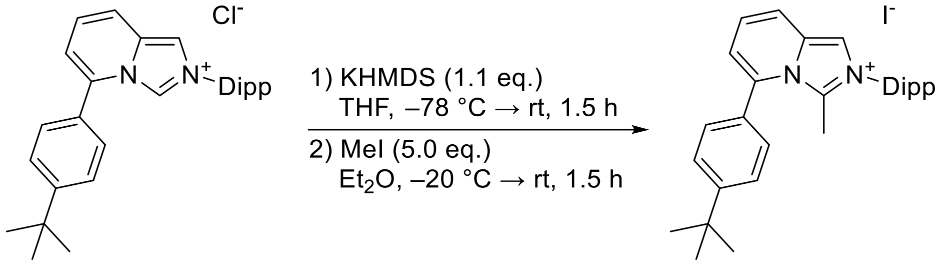


To a mixture of **S11** (1.00 g, 2.24 mmol, 1.0 eq.) and KHMDS (491 mg, 2.46 mmol, 1.1 eq.) was added THF (50 mL) at –78 °C. The reaction mixture was stirred for 15 minutes and then thawed to rt for 1 hour. The solvent was removed under reduced pressure. Then, the remaining solid was dissolved in toluene (2x20 mL) and added dropwise to a methyl iodide solution (418 µL, 6.71 mmol, 3.0 eq.) in $Et_2O$ (30 mL) at –20 °C. After full addition, the reaction mixture was thawed to rt and was stirred for 1 hour, yielding a white precipitate. The solution was then filtered, and the colorless residue was washed thrice with $Et_2O$ (3x25 mL) and dried under reduced pressure, furnishing **1d** as colorless solid (1.03 g, 1.86 mmol, 83%).

**m.p.** 232 °C; **$^1$H NMR** (500 MHz, $CD_3CN$): δ [ppm] = 8.03 (s, 1H, ImPy-H), 7.83 (dd, *J* = 9.3, 1.2 Hz, 1H, ImPy-H), 7.64 (t, *J* = 7.9 Hz, 1H, Dipp-H), 7.64 – 7.58 (m, 2H, Ar-H), 7.58 – 7.52 (m, 2H, Ar-H), 7.45 (d, *J* = 7.9 Hz, 2H, Dipp-H), 7.34 (dd, *J* = 9.3, 6.8 Hz, 1H, ImPy-H), 7.02 (dd, *J* = 6.8, 1.2 Hz, 1H, ImPy-H), 2.15 (hept, *J* = 6.9 Hz, 2H), 1.81 (s, 3H, ImPy-$CH_3$), 1.36 (s, 9H, $C(CH_3)_3$), 1.12 (d, *J* = 6.8 Hz, 6H, $CH(CH_3)_2$), 1.09 (d, *J* = 6.8 Hz, 6H, $CH(CH_3)_2$); **$^{13}$C NMR** (126 MHz, $CD_3CN$): δ [ppm] = 155.0 (Ar-$C_q$), 146.4 (Dipp-$C_q$), 137.9 (ImPy-$C_q$), 136.6 (ImPy-$C_q$), 133.0 (Dipp-*C*H), 132.5 (ImPy-$C_q$), 131.0 (Ar-*C*H), 130.9 (Dipp-$C_q$), 130.5 (Ar-$C_q$), 126.3 (Ar-*C*H), 125.9 (Dipp-*C*H), 125.8 (ImPy-*C*H), 121.1 (ImPy-*C*H), 118.7 (ImPy-*C*H), 115.4 (ImPy-*C*H), 35.5 ($C(CH_3)_3$), 31.4 ($C(CH_3)_3$), 29.0 ($CH(CH_3)_2$), 24.9 ($CH(CH_3)_2$), 23.3 ($CH(CH_3)_2$), 14.0 (ImPy-$CH_3$); **IR** [$cm^{-1}$]: ṽ = 2960.0, 2866.8, 1654.7, 1551.3, 1505.5, 1461.4, 1401.6, 1381.7, 1361.0, 1301.3, 1269.7, 1176.7, 1112.4, 1058.2, 837.4, 799.1, 775.9, 736.7, 696.7, 678.8, 648.9, 604.7, 585.6, 564.3, 466.1; **HR-MS-ESI(+)** calc. $C_{30}H_{37}N_2^+$ $[M]^+$ 425.2951; found 425.2945.

Synthesis of **S12**

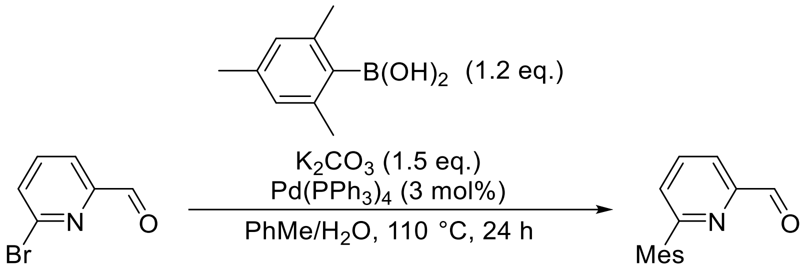


6-bromopicolinaldehyde (**S8**, 4.00 g, 21.5 mmol, 1.00 eq.), mesitylboronic acid (4.23 g, 25.8 mmol, 1.20 eq.) and $Na_2CO_3$ (5.01 g, 47.31 mmol, 2.20 eq.) were dissolved in a degassed mixture of toluene and water (4:1, 100 mL). The reaction mixture was heated to 110 °C for 24 h. After separation of the phases the aqueous phase was extracted twice with $CH_2Cl_2$ (50 mL). The combined organic phases were dried over $Na_2SO_4$, the solvent was removed under reduced pressure and the resulting yellow solid purified by column chromatography (silica, silica, 1% EtOAc in cyclohexane to 5%). **S12** (4.80 g, 19.9 mmol, 93%) was obtained as a colorless, highly viscous oil. The spectroscopic data agrees with literature data.[4]

**$^1$H NMR** (500 MHz, $CDCl_3$): δ [ppm] = 10.12 (s, 1H, C(*H*)=O), 7.96 – 7.93 (m, 2H, Py-H), 7.47 (dd, J = 5.5, 3.4 Hz, 1H, Py-H), 6.98 (s, 2H, Mes-H), 2.35 (s, 3H, Mes-$CH_3$), 2.04 (s, 6H, Mes-$CH_3$).; **$^{13}$C NMR** (126 MHz, $CDCl_3$): δ [ppm] = 194.0, 160.9, 152.9, 138.2, 137.4, 136.6, 135.7, 129.2, 128.6, 119.5, 21.2, 20.2.

Synthesis of **S13**

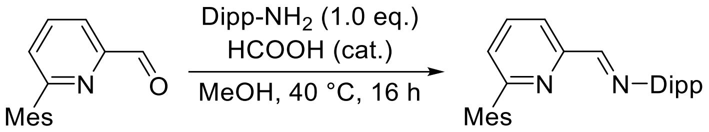


A mixture of 2,6-diisopropylaniline (3.54 g, 3.77 mL, 19.9 mmol, 1.0 eq.), **S12** (4.50 g, 19.9 mmol, 1.0 eq.) and 2 drops of formic acid in MeOH (50 mL) was stirred for 16 h at 40 °C. The solvent was removed under reduced pressure giving a pale-yellow solid. The crude product was then recrystallized from MeOH (50 mL) at -25 °C, furnishing **S13** as crystalline yellow solid (5.60 g, 14.6 mmol, 73%).

**m.p.** 131 °C**;** **$^1$H NMR** (500 MHz, $CDCl_3$): δ [ppm] = 8.35 (d, *J* = 0.7 Hz, 1H, C(*H*)=N), 8.26 (dd, *J* = 7.9, 1.1 Hz, 1H, Py-H), 7.91 (td, *J* = 7.7, 0.7 Hz, 1H, Py-H), 7.36 (dd, *J* = 7.6, 1.1 Hz, 1H, Py-H), 7.21 – 7.09 (m, 3H, Dipp-H), 6.98 (s, 2H, Mes-H), 3.01 (hept, *J* = 6.9 Hz, 2H, Dipp-$CH_{Alkyl}$), 2.34 (s, 3H, Mes-$CH_3$), 2.10 (s, 6H, Mes-$(CH_3)_2$), 1.19 (d, *J* = 6.9 Hz, 12H, Dipp-$CH_3$); **$^{13}$C NMR** (126 MHz, $CDCl_3$): δ [ppm] = 163.6 (*C*(H)=N), 160.1 (Py-C), 154.5 (Py-C), 148.6 (Ar-C), 138.0 (Ar-C), 137.4 (Ar-C), 137.2 (Ar-C), 137.0 (Py-CH), 135.9 (Ar-C), 128.7 (Ar-C), 126.6

(Py-CH), 124.5 (Dipp-CH), 123.1 (Dipp-CH), 119.1 (Py-CH), 28.1 (Dipp-$CH_{Alkyl}$), 23.6 (Dipp-$CH_3$), 21.2 (Mes-$CH_3$), 20.4 (Mes-$(CH_3)_2$); **IR** [$cm^{-1}$]: ṽ = 2955.1, 2921.2, 1636.9, 1612.2, 1584.9, 1562.2, 1451.9, 1381.5, 1362.3, 1315.7, 1182.9, 1056.3, 988.4, 932.6, 884.6, 859.5, 843.8, 818.7, 796.3, 760.2, 752.7, 726.3, 629.8, 531.7; **HR-MS-ESI(+)** calc. $C_{27}H_{33}N_2$ $[M+H]^+$ 385.2638; found 385.2635.

Synthesis of **S14**

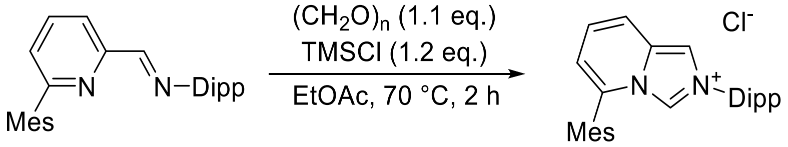


**S13** (2.00 g, 5.20 mmol, 1.0 eq.) and paraformaldehyde (172 mg; 5.72 mmol; 1.1 eq.) were dissolved in EtOAc (50 mL) and heated to 70 °C under argon atmosphere. TMSCl (734 mg, 864 mL, 6.76 mmol, 1.3 eq.) was added dropwise, leading to the instantaneous formation of colorless precipitate. The reaction mixture was stirred for 2 h, cooled to rt and filtered over a glass frit. The colorless residue was washed thrice with $Et_2O$ (3x25 mL) and dried under reduced pressure, furnishing **S14** as colorless solid (2.00 g, 4.62 mmol, 89%).

**m.p.** 312 °C**;** $^1$**H NMR** (501 MHz, $CD_3CN$): δ [ppm] = 8.69 (d, *J* = 0.9 Hz, 1H, ImPy-H), 8.16 (d, *J* = 1.8 Hz, 1H, ImPy-H), 7.97 (d, *J* = 9.5 Hz, 1H, ImPy-H), 7.60 (t, *J* = 7.8 Hz, 1H, Dipp-H), 7.53 (dd, *J* = 9.4, 6.9 Hz, 1H, ImPy-H), 7.40 (d, *J* = 7.8 Hz, 2H, Dipp-H), 7.17 (dd, *J* = 7.0, 1.0 Hz, 1H, ImPy-H), 7.11 (s, 2H, Mes-H), 2.33 (s, 3H, Mes-$CH_3$), 2.13 (hept, *J* = 7.0 Hz, 2H, $C\mathit{H}(CH_3)_2$), 2.05 (s, 6H, Mes-$(CH_3)_2$), 1.16 (d, *J* = 6.8 Hz, 6H, $CH(C\mathit{H}_3)_2$), 1.04 (d, *J* = 6.9 Hz, 6H, $CH(C\mathit{H}_3)_2$).; $^{13}$**C NMR** (126 MHz, $CD_3CN$): δ [ppm] = 146.2 (Ar-$C_q$), 142.0 (Ar-$C_q$), 138.5 (ImPy-$C_q$), 135.1 (Ar-$C_q$), 132.9 (Ar-*C*H), 132.7 (ImPy-$C_q$), 131.7 (Ar-$C_q$), 130.2 (Ar-*C*H), 127.8 (Ar-$C_q$), 127.4 (ImPy-*C*H), 125.8 (Ar-$C_q$), 125.4 (ImPy-*C*H and Ar-*C*H (2 carbons)), 121.4 (ImPy-*C*H), 118.7 (ImPy-*C*H), 118.0 (ImPy-*C*H), 29.3 ($\mathit{C}H(CH_3)_2$), 24.3 ($CH(\mathit{C}H_3)_2$), 24.1 ($CH(\mathit{C}H_3)_2$), 21.3 (Mes-$\mathit{C}H_3$), 19.4 (Mes-$(\mathit{C}H_3)_2$); **IR** [$cm^{-1}$]: ṽ = 2962.7, 1722.6, 1651.9, 1553.1, 1449.2, 1367.4, 1321.5, 1213.2, 1173.7, 1155.4, 1040.8, 879.6, 803.6, 757.3, 677.5, 618.6, 602.5, 570.4, 563.8, 477.9; **HR-MS-ESI(+)** calc. $C_{28}H_{33}N_2$ $[M]^+$ 397.2638; found 397.2635.

Synthesis of **1e**

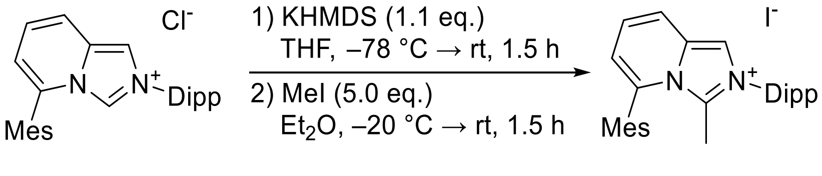


To a mixture of **S14** (800 mg, 1.65 mmol, 1.0 eq.) and KHMDS (362 mg, 1.82 mmol, 1.1 eq.) was added THF (50 mL) at –78 °C. The reaction mixture was stirred for 15 minutes and then

thawed to rt for 1 hour. The solvent was removed under reduced pressure. Then, the remaining solid was dissolved in toluene (2x20 mL) and added dropwise to a methyl iodide solution (308 µL, 4.95 mmol, 3.00 eq.) in $Et_2O$ (30 mL) at –20 °C. After full addition, the reaction mixture was thawed to rt and was stirred for 1 hour, yielding a white precipitate. The solution was then filtered, and the colorless residue was washed thrice with $Et_2O$ (3x25 mL) and dried under reduced pressure, furnishing **1e** as colorless solid (889 mg, 1.65 mmol, quant.).

**m.p.** 302 °C; **$^1$H NMR** (500 MHz, $CD_3CN$): δ [ppm] = 8.11 (s, 1H, ImPy-H), 7.89 (dd, *J* = 9.4, 1.2 Hz, 1H, ImPy-H), 7.65 (t, *J* = 7.9 Hz, 1H, Dipp-H), 7.46 (d, *J* = 7.9 Hz, 2H, Dipp-H), 7.40 (dd, *J* = 9.3, 6.8 Hz, 1H, ImPy-H), 7.08 (s, 2H, Mes-H), 7.06 (dd, *J* = 6.8, 1.2 Hz, 1H, ImPy-H), 2.34 (s, 3H, Mes-$CH_3$), 2.07 (s, 6H, Mes-$(CH_3)_2$), 2.04 (d, *J* = 6.6 Hz, 2H, Dipp-CH), 1.74 (s, 3H, ImPy-$CH_3$), 1.13 (d, *J* = 6.7 Hz, 6H, Dipp-$CH_3$), 1.08 (d, *J* = 6.8 Hz, 6H, Dipp-$CH_3$); **$^{13}$C NMR** (126 MHz, $CD_3CN$): δ [ppm] = 146.3 (Ar-$C_q$), 142.1 (Ar-$C_q$), 139.2 (Ar-$C_q$), 135.8 (ImPy-$C_q$), 135.5 (ImPy-$C_q$), 133.1 (Ar-*C*H), 132.7 (ImPy-$C_q$), 130.7 (Ar-$C_q$), 129.4 (Ar-*C*H), 129.4 (Ar-$C_q$), 126.1 (ImPy-*C*H), 126.0 (Ar-*CH*), 122.0 (ImPy-*C*H), 119.1 (ImPy-*C*H), 116.1 (ImPy-*C*H), 29.3 (Dipp-*C*H), 24.8 (Dipp-$CH_3$), 23.2 (Dipp-$CH_3$), 21.3 (Mes-$CH_3$), 20.0 (Mes-$(CH_3)_2$), 12.1 (ImPy-$CH_3$); **IR** [$cm^{-1}$]: ṽ = 3000.1, 2961.9, 1654.5, 1547.8, 1454.5, 1420.9, 1391.0, 1201.5, 1060.2, 853.5, 843.2, 828.1, 811.5, 774.9; **HR-MS-ESI(+)** calc. $C_{27}H_{35}N_2^+$ $[M]^+$ 411.2795; found 411.2790.

Synthesis of **S15**

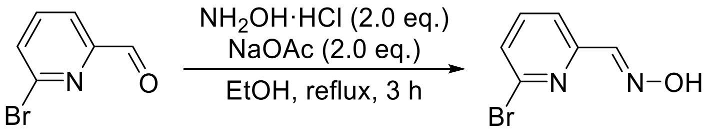


To a solution of 6-bromopicolinaldehyde (**S8**, 3.00 g, 16.1 mmol, 1.0 eq.) in EtOH (50 mL) at rt was added hydroxylamine hydrochloride (2.24 g, 32.3 mmol, 2.0 eq.) and sodium acetate (2.65 g, 32.3 mmol, 2.0 eq.). Upon addition the colorless solution was stirred at 90 °C for 3 hours. The solution was then cooled to rt and the solvent was removed under reduced pressure. The resulting colorless solid was dissolved in EtOAc (150 mL) and washed with water (100 mL). The aqueous phase was extracted four times with EtOAc (4x150 mL). Afterwards the organic phase was dried with $Na_2SO_4$, filtrated and the solvent was removed under reduced pressure to yield **S15** (3.10 g, 15.4 mmol, 96%) as a colorless solid. The spectroscopic data agrees with literature data.[5]

**$^1$H NMR** (500 MHz, DMSO-$d_6$): δ [ppm] = 11.90 (s, 1H, C(H)=N-O*H*), 8.04 (s, 1H, C(*H*)=N-OH), 7.82 – 7.74 (m, 2H, Py-H), 7.63 (dd, *J* = 7.1, 1.7 Hz, 1H, Py-H); **$^{13}$C NMR** (126 MHz, DMSO-$d_6$): δ [ppm] = 153.3 (Py-$C_q$), 147.5 (*C*(H)=N-OH), 141.0 (Py-$C_q$), 140.1 (Py-*C*H), 128.1 (Py-*C*H), 119.3 (Py-*C*H).

Synthesis of **S16**

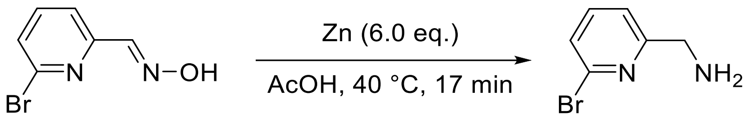

To a solution of **S15** (3.00 g, 14.9 mmol, 1.0 eq.) in AcOH (100 mL, 0.15 M) in a 500 mL round-bottom flask, activated zinc dust (5.85 g, 89.5 mmol, 6.0 eq.) was poured in the flask in one portion. The mixture was first placed in an ultrasonic bath at rt for 5 min and then it was stirred for 12 min at 40 °C and 1000 rpm. Afterwards the mixture was filtered over Celite and the filtrate was concentrated under reduced pressure. The orange oil was then dissolved in $CH_2Cl_2$ (50 mL) and mixed with concentrated hydrochloric acid (20 mL) and then stirred for 15 minutes at 0 °C. Afterwards, water (80 mL) and then aqueous NaOH (80 mL, 20w%) were added and the mixture was extracted four times with $CH_2Cl_2$ (4x100 mL). Then, the organic phase was dried with $Na_2SO_4$, filtrated and the solvent was removed under reduced pressure to yield **S16** (2.08 g, 11.1 mmol, 75%) as a pale-yellow oil. The spectroscopic data agrees with literature data.[6]

**$^1$H NMR** (500 MHz, $CDCl_3$): δ [ppm] = 7.50 (t, *J* = 7.7 Hz, 1H, Py-H), 7.35 (d, *J* = 8.0 Hz, 1H, Py-H), 7.25 (d, *J* = 7.4 Hz, 1H, Py-H), 3.94 (s, 2H, Py-$CH_2$-), 1.76 (s, 2H, -$NH_2$); **$^{13}$C NMR** (126 MHz, $CDCl_3$): δ [ppm] = 163.9 (Py-$C_q$), 142.0 (Py-$C_q$), 139.1 (Py-*C*H), 126.3 (Py-*C*H), 120.1 (Py-*C*H), 47.5 (Py-*C*$H_2$-).

Synthesis of **S17**

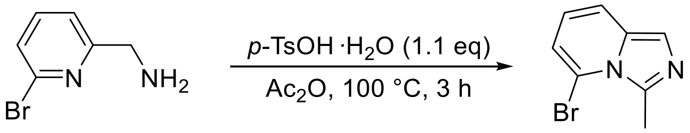

A mixture of **S16** (4.90 g, 26.2 mmol, 1.0 eq.) and *p*-TsOH monohydrate (5.48 g, 28.8 mmol, 1.1 eq.) was dissolved in $Ac_2O$ (40 mL) at 0 °C, placed in a preheated 100 °C oil bath and was stirred for 3 hours. The reaction mixture was poured into water (100 mL) and made alkaline using 20w% NaOH at 0 °C. The solution was extracted with $CH_2Cl_2$ (4 x 200 mL), the combined organic phases were dried over $Na_2SO_4$, and the solvent was removed under reduced pressure. The crude product was purified by flash column chromatography (silica, 20% EtOAc in cyclohexane) and subsequent recrystallization from hot pentane (~200 mL) at –25 °C furnished **S17** (2.28 g, 10.8 mmol, 41%) as a pale-yellow solid.

**m.p.** 80 °C**;** **$^1$H NMR** (500 MHz, $CDCl_3$): δ [ppm] = 7.33 (s, 1H, ImPy-H), 7.31 (dd, *J* = 9.0, 1.1 Hz, 1H, ImPy-H), 6.70 (dd, *J* = 6.7, 1.1 Hz, 1H, ImPy-H), 6.39 (dd, *J* = 9.0, 6.8 Hz, 1H, ImPy-H), 3.07 (s, 3H, ImPy-$CH_3$); **$^{13}$C NMR** (126 MHz, $CDCl_3$): δ [ppm] = 138.1 (ImPy-$C_q$), 133.7

(ImPy-*C*$_q$), 119.6 (ImPy-*C*H), 118.4 (ImPy-*C*H), 118.3 (ImPy-*C*H), 118.2 (ImPy-*C*H), 111.5 (ImPy-*C*H), 18.9 (ImPy-*C*H$_3$); **IR** [cm$^{-1}$]: ṽ = 3073, 2972, 2930, 1627, 1542, 1499, 1484, 1434, 1373, 1325, 1278, 1264, 1207, 1155, 1065, 1040, 1030, 975, 940, 898, 787, 747, 709, 696, 656, 587, 557; **HR-MS-ESI(+)** calc. $C_8H_8BrN_2^+$ $[M+H]^+$ 210.9865; found 210.9863.

Synthesis of **S18**

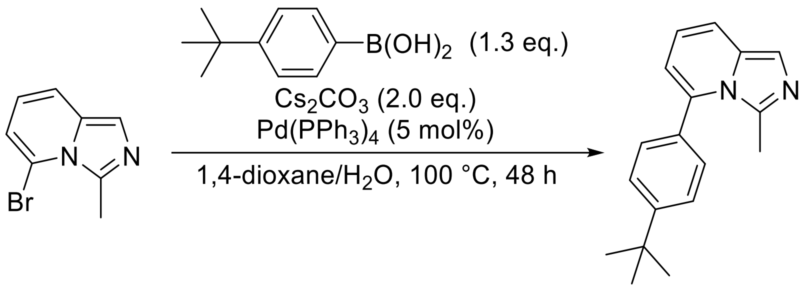


**S17** (500 mg, 2.37 mmol, 1.0 eq., 0.3M in 1,4-dioxane), (4-(tert-butyl)phenyl)boronic acid (549 mg, 3.08 mmol, 1.3 eq.) and $Cs_2CO_3$ (1.54 g, 4.74 mmol, 2.0 eq., 2M in $H_2O$) were dissolved in a mixture of 1,4-dioxane and water (7:2, 18 mL). After degassing the mixture, $Pd(PPh_3)_4$ (137 mg, 119 µmol, 5 mol%) was added at once. The reaction mixture was heated to 100 °C for 48 h. The mixture was cooled to rt and then 60 mL of EtOAc was added. After separation of the phases the organic phase was washed twice with brine (2x25 mL). The organic phase was dried over $Na_2SO_4$, the solvent was removed under reduced pressure and the resulting yellow solid purified by flash column chromatography (silica, 15% EtOAc in cyclohexane). **S18** (570 mg, 2.16 mmol, 91%) was obtained as pale-yellow oil.

**m.p.** <25 °C**;** **$^1$H NMR** (500 MHz, $CDCl_3$): δ [ppm] = 7.50 – 7.44 (m, 2H, Ar-H), 7.40 (s, 1H, ImPy-H), 7.38 (dd, *J* = 9.0, 1.2 Hz, 1H, ImPy-H), 7.36 – 7.30 (m, 2H, Ar-H), 6.65 (dd, *J* = 9.1, 6.4 Hz, 1H, ImPy-H), 6.34 (dd, *J* = 6.4, 1.2 Hz, 1H, ImPy-H), 2.02 (s, 3H, ImPy-CH$_3$), 1.38 (s, 9H, C(CH$_3$)$_3$); **$^{13}$C NMR** (126 MHz, $CDCl_3$): δ [ppm] = 152.6 (Ar-*C*$_q$), 137.1 (ImPy-*C*$_q$), 135.9 (Ar-*C*$_q$), 132.5 (ImPy-*C*$_q$), 132.5 (ImPy-*C*$_q$), 129.7 (Ar-*C*H), 124.9 (Ar-*C*H), 118.8 (ImPy-*C*H), 117.9 (ImPy-*C*H), 117.7 (ImPy-*C*H), 114.7 (ImPy-*C*H), 35.0 (*C*(CH$_3$)$_3$), 31.5 (C(*C*H$_3$)$_3$), 17.7 (ImPy-*C*H$_3$); **IR** [cm$^{-1}$]: ṽ = 2961, 2866, 1678, 1634, 1501, 1439, 1394, 1364, 1339, 1300, 1267, 1185, 1154, 1105, 1068, 1019, 978, 945, 839, 794, 758, 719, 661, 612, 563, 542; **HR-MS-ESI(+)** calc. $C_{18}H_{21}N_2^+$ $[M+H]^+$ 265.1699; found 265.1703.

Synthesis of **1f**

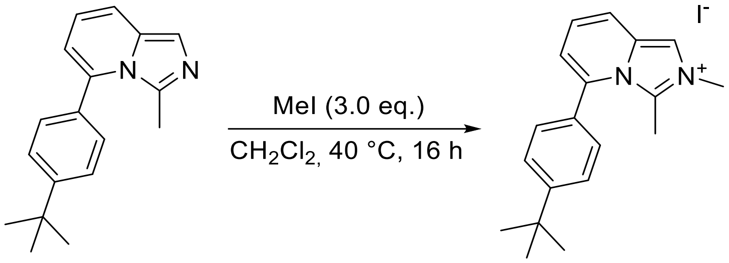


To a solution of **S18** (370 mg, 1.40 mmol, 1.0 eq.) in $CH_2Cl_2$ (10 mL) was added MeI (596 mg, 260 µL, 4.20 mmol, 3.0 eq.). The mixture was stirred for 16 h at 40 °C. Afterwards, the solution was reduced to a small volume of $CH_2Cl_2$ (~3 mL) and then $Et_2O$ (50 mL) was added while stirring vigorously. The solution was filtered off and the remaining off-white solid was washed thrice with $Et_2O$ (3x20 mL). Drying under reduced pressure furnished **1f** as an off-white solid (460 mg, 1.13 mmol, 81%).

**m.p.** 264 °C**;** $^{1}$**H NMR** (500 MHz, $CD_3CN$): δ [ppm] = 7.93 (s, 1H, ImPy-H), 7.72 (dd, *J* = 9.3, 1.2 Hz, 1H, ImPy-H), 7.64 – 7.59 (m, 2H, Ar-H), 7.48 – 7.44 (m, 2H, Ar-H), 7.19 (dd, *J* = 9.3, 6.7 Hz, 1H, ImPy-H), 6.86 (dd, *J* = 6.8, 1.2 Hz, 1H, ImPy-H), 3.96 (s, 3H, N-$CH_3$), 2.07 (s, 3H, ImPy-$CH_3$), 1.38 (s, 9H, $C(CH_3)_3$); $^{13}$**C NMR** (126 MHz, $CD_3CN$): δ [ppm] = 154.9 (Ar-$C_q$), 137.2 (Ar-$C_q$), 136.6 (ImPy-$C_q$), 131.3 (ImPy-$C_q$), 130.9 (ImPy-$C_q$), 130.6 (Ar-*C*H), 126.5 (Ar-*C*H), 124.7 (ImPy-*C*H), 120.5 (ImPy-*C*H), 118.4 (ImPy-*C*H), 114.9 (ImPy-*C*H), 37.2 (N-$CH_3$), 35.6 (*C*($CH_3$)$_3$), 31.4 (C($CH_3$)$_3$), 13.9 (ImPy-$CH_3$); **IR** [$cm^{-1}$]: ṽ = 3032, 2954, 1651, 1507, 1477, 1403, 1380, 1363, 1324, 1269, 1142, 1125, 1105, 1030, 1014, 851, 808, 771, 748, 727, 648, 632, 613, 595, 548; **HR-MS-ESI(+)** calc. $C_{19}H_{23}N_2^+$ $[M]^+$ 279.1856; found 279.1857.

Synthesis of **S19**

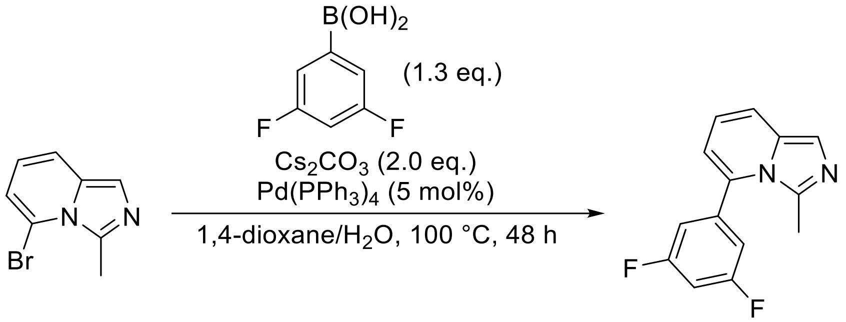


**S17** (950 mg, 4.50 mmol, 1.0 eq., 0.3M in 1,4-dioxane), (3,5-difluorophenyl)boronic acid (924 mg, 5.85 mmol, 1.3 eq.) and $Cs_2CO_3$ (2.93 g, 9.00 mmol, 2.0 eq., 2M in $H_2O$) were dissolved in a mixture of 1,4-dioxane and water (7:2, 20 mL). After degassing the mixture, $Pd(PPh_3)_4$ (260 mg, 225 µmol, 5 mol%) was added at once. The reaction mixture was heated to 100 °C for 48 h. The mixture was cooled to rt, then 100 mL of EtOAc was added and it was

filtrated over a Celite frit. After separation of the phases the organic phase was washed twice with brine (2x25 mL). The organic phase was dried over $Na_2SO_4$, the solvent was removed under reduced pressure and the resulting yellow solid purified by column chromatography (silica, 30% EtOAc in cyclohexane). **S19** (835 mg, 3.42 mmol, 76%) was obtained as light-green solid.

**m.p.** 146 °C**;** $^{1}$**H NMR** (400 MHz, $CDCl_3$): δ [ppm] = 7.50 – 7.41 (m, 2H, ImPy-H), 7.03 – 6.91 (m, 3H, Ar-H), 6.68 (dd, *J* = 9.1, 6.5 Hz, 1H, ImPy-H), 6.39 (dd, *J* = 6.5, 1.2 Hz, 1H, ImPy-H), 2.13 (s, 3H, ImPy-$CH_3$); $^{13}$**C NMR** (101 MHz, $CDCl_3$) : δ [ppm] = 162.6 (dd, *J* = 251.2, 12.9 Hz, 2 Ar-*C*-F), 138.2 (t, *J* = 10.0 Hz, Ar-*C*-F), 136.6 (ImPy-$C_q$), 133.0 (ImPy-$C_q$), 132.3 (ImPy-$C_q$), 119.4 (ImPy-*C*H), 119.0 (ImPy-*C*H), 117.7 (ImPy-*C*H), 115.5 (ImPy-*C*H), 113.5 – 112.9 (m, 2 Ar-*C*H), 105.0 (t, *J* = 25.0 Hz, Ar-*C*H), 17.7 (ImPy-$CH_3$); $^{19}$**F NMR** (377 MHz, $CDCl_3$): δ [ppm] = -108.50 (t, *J* = 7.7 Hz, 2F, Ar-F); **IR** [$cm^{-1}$]: ṽ = 3089, 3042, 2924, 1619, 1589, 1490, 1454, 1442, 1429, 1411, 1377, 1356, 1321, 1294, 1263, 1234, 1184, 1156, 1117, 1075, 1040, 1013, 987, 904, 877, 859, 836, 808, 761, 722, 711, 692, 663, 639, 624, 560, 529, 508; **HR-MS-ESI(+)** calc. $C_{14}H_{11}F_2N_2^+$ $[M+H]^+$ 245.0885; found 245.0883.

Synthesis of **1g**

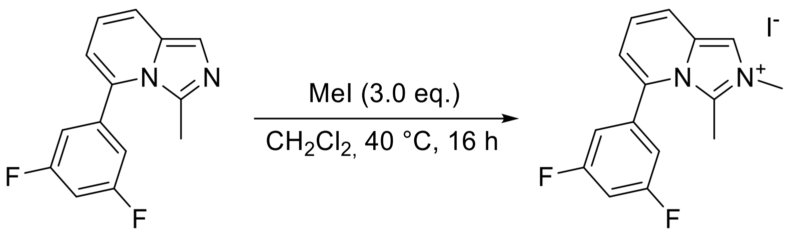


To a solution of **S19** (735 mg, 3.01 mmol, 1.0 eq.) in $CH_2Cl_2$ (10 mL) was added MeI (1.28 g, 562 µL, 9.03 mmol, 3.0 eq.). The mixture was stirred for 16 h at 40 °C. Afterwards, $Et_2O$ (40 mL) was added while stirring vigorously. The solution was filtered off and the remaining off-white solid was washed thrice with $Et_2O$ (3x20 mL). Drying under reduced pressure furnished **1g** as an off-white solid (1.04 g, 2.69 mmol, 90%).

**m.p.** 262 °C**;** $^{1}$**H NMR** (400 MHz, $CD_3CN$): δ [ppm] = 8.00 (s, 1H, ImPy-H), 7.79 (dd, *J* = 9.3, 1.2 Hz, 1H, ImPy-H), 7.30 – 7.17 (m, 4H, ImPy- and Ar-H), 6.95 (dd, *J* = 6.8, 1.2 Hz, 1H, ImPy-H), 4.00 (s, 3H, N-$CH_3$), 2.21 (s, 3H, ImPy-$CH_3$); $^{13}$**C NMR** (101 MHz, $CD_3CN$): δ [ppm] = 163.6 (dd, *J* = 249.4, 13.3 Hz, Ar-$C_q$), 136.7 (t, *J* = 10.6 Hz, ImPy- and Ar-$C_q$), 134.3 (ImPy-$C_q$), 131.2 (ImPy-$C_q$), 124.3 (ImPy-*C*H), 121.3 (ImPy-*C*H), 119.5 (ImPy-*C*H), 115.4 (ImPy-*C*H), 114.7 (m, 2 Ar-*C*H), 106.8 (t, *J* = 10.6 Hz, Ar-*C*H), 37.4 (N-$CH_3$), 14.4 (ImPy-$CH_3$); $^{19}$**F NMR** (377 MHz, $CD_3CN$): δ [ppm] = -109.61 – -109.71 (m, 2F, Ar-F); **IR** [$cm^{-1}$]: ṽ = 3083, 3023, 1652, 1621, 1594, 1525, 1443, 1427, 1416, 1369, 1335, 1321, 1240, 1178, 1137, 1121, 1009, 986, 911,

884, 852, 837, 790, 728, 689, 630, 504; **HR-MS-ESI(+)** calc. $C_{15}H_{13}F_2N_2^+$ $[M]^+$ 259.1041; found 259.1049.

Synthesis of **S20**

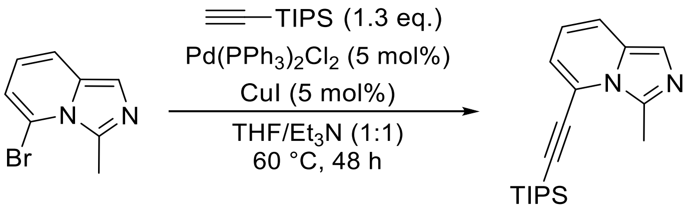


To a degassed solution of dry THF and $NEt_3$ (20 mL, 1:1) under argon was added $Pd(PPh_3)_2Cl_2$ (83 mg, 118 µmol, 5 mol%) and the resulting suspension was stirred for 15 min. Afterwards, **S17** (500 mg, 2.37 mmol, 1.0 eq.), TIPS-acetylene (691 µL, 3.08 mmol, 1.3 eq.) and CuI (23 mg, 118 µmol, 5 mol%) were added, the reaction mixture was heated to 60 °C and was stirred for 48 h. The mixture was then cooled to rt, the solvent was removed under reduced pressure and the crude product was purified by flash column chromatography (silica, 10% to 15% EtOAc in cyclohexane) to yield **S20** (666 mg, 2.13 mmol, 90%) as a yellow solid.

**m.p.** 70 °C**;** $^1$**H NMR** (500 MHz, $CDCl_3$): δ [ppm] = 7.40 (s, 1H, ImPy-H), 7.38 (dd, *J* = 9.1, 1.2 Hz, 1H, ImPy-H), 6.84 (dd, *J* = 6.7, 1.2 Hz, 1H, ImPy-H), 6.58 (dd, *J* = 9.1, 6.7 Hz, 1H, ImPy-H), 3.13 (s, 3H, ImPy-$CH_3$), 1.20 – 1.12 (m, 21H, -Si-($CH(CH_3)_2)_3$); $^{13}$**C NMR** (126 MHz, $CDCl_3$): δ [ppm] = 138.0 (ImPy-$C_q$), 131.6 (ImPy-$C_q$), 122.6 (ImPy-*C*H), 119.8 (ImPy-*C*H), 118.6 (ImPy-*C*H), 118.1 (ImPy-*C*H), 117.8 (ImPy-$C_q$), 102.0 (-C≡C-), 99.9 (-C≡C-), 18.7 (-Si-(CH($C$H$_3$)$_2$)$_3$), 16.5 (ImPy-$C$H$_3$), 11.5 (-Si-($C$H(CH$_3$)$_2$)$_3$); **IR** [cm$^{-1}$]: ṽ = 3072, 2944, 2891, 2864, 2146, 1621, 1550, 1502, 1460, 1408, 1381, 1335, 1297, 1240, 1170, 1093, 1074, 1060, 1047, 1035, 1017, 995, 974, 919, 881, 850, 794, 754, 720, 706, 678, 653, 614, 588, 577, 542, 526, 494, 462, 414; **HR-MS-ESI(+)** calc. $C_{19}H_{29}N_2Si^+$ $[M+H]^+$ 313.2095; found 313.2087.

Synthesis of **4b**

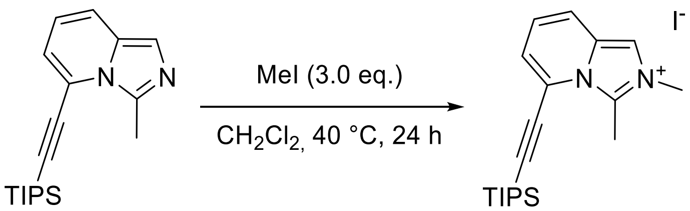


To a solution of **S20** (577 mg, 1.85 mmol, 1.0 eq.) in $CH_2Cl_2$ (10 mL) was added MeI (786 mg, 345 µL, 5.54 mmol, 3.0 eq.). The mixture was stirred for 24 h at 40 °C. Afterwards, $Et_2O$ (40 mL) was added while stirring vigorously. The solution was filtered off and the remaining solid was washed thrice with $Et_2O$ (3x20 mL). Drying under reduced pressure furnished **4b** as an off-white solid (676 mg, 1.49 mmol, 80%).

**m.p.** 208 °C (decomp.)**;** $^{1}$**H NMR** (500 MHz, DMSO-$d_6$): δ [ppm] = 8.27 (s, 1H, ImPy-H), 7.90 (dd, *J* = 9.3, 1.1 Hz, 1H, ImPy-H), 7.44 (dd, *J* = 7.0, 1.1 Hz, 1H, ImPy-H), 7.13 (dd, *J* = 9.2, 7.0 Hz, 1H, ImPy-H), 4.04 (s, 3H, ImPy-N-$CH_3$), 3.23 (s, 3H, ImPy-$CH_3$), 1.27 – 1.17 (m, 3H, -Si-(C*H*$(CH_3)_2)_3$), 1.13 (d, *J* = 6.8 Hz, 18H, -Si-(CH(C$H_3)_2)_3$); $^{13}$**C NMR** (126 MHz, DMSO-$d_6$): δ [ppm] = 137.0 (ImPy-$C_q$), 128.9 (ImPy-$C_q$), 126.4 (ImPy-*C*H), 123.1 (ImPy-*C*H), 120.1 (ImPy-*C*H), 117.0 (ImPy-$C_q$), 115.2 (ImPy-*C*H), 104.4 (-C≡C-), 98.0 (-C≡C-), 36.0 (ImPy-N-$CH_3$), 18.4 (Si-(CH($CH_3)_2)_3$), 12.3 (ImPy-$CH_3$), 10.7 (Si-(*C*H$(CH_3)_2)_3$); **IR** [$cm^{-1}$]: ṽ = 3058, 2943, 2864, 2144, 1643, 1527, 1463, 1366, 1320, 1255, 1181, 1141, 1073, 1019, 990, 919, 883, 811, 793, 767, 712, 666, 643, 628, 575, 552, 525, 492, 460, 420; **HR-MS-ESI(+)** calc. $C_{20}H_{31}N_2Si^+$ $[M]^+$ 327.2251; found 327.2253.

Synthesis of **S21**

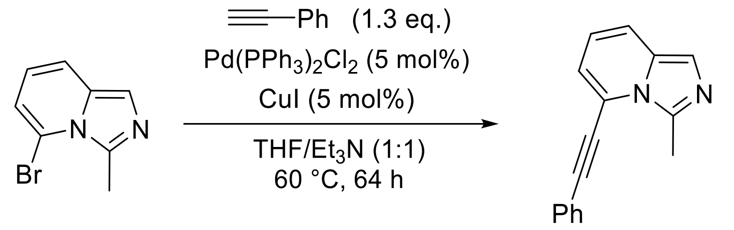


To a degassed solution of dry THF and $NEt_3$ (25 mL, 1:1) under argon was added $Pd(PPh_3)_2Cl_2$ (80 mg, 120 µmol, 5 mol%) and the resulting suspension was stirred for 15 min. Afterwards, **S17** (500 mg, 2.37 mmol, 1.0 eq.), phenylacetylene (340 µL, 3.08 mmol, 1.3 eq.) and CuI (20 mg, 120 µmol, 5 mol%) were added, the reaction mixture was heated to 60 °C and was stirred for 64 h. The mixture was then cooled to rt, the solvent was removed under reduced pressure and the crude product was purified by flash column chromatography (silica, 15% EtOAc in cyclohexane) to yield **S21** (380 mg, 1.64 mmol, 69%) as a black solid.

**m.p.** 103 °C**;** $^{1}$**H NMR** (500 MHz, $CDCl_3$): δ [ppm] =7.56 – 7.53 (m, 2H, Ar-H), 7.43 – 7.39 (m, 4H, ImPy-H*,* Ar-H), 7.38 (dd, *J* = 9.1, 1.1 Hz, 1H, ImPy-H), 6.83 (dd, *J* = 6.7, 1.1 Hz, 1H, ImPy-H), 6.58 (dd, *J* = 9.1, 6.7 Hz, 1H, ImPy-H), 3.15 (s, 3 H, ImPy-$CH_3$); $^{13}$**C NMR** (126 MHz, $CDCl_3$): δ [ppm] =138.1 (ImPy-$C_q$), 131.8 (ImPy-$C_q$), 131.2 (Ar-*C*H), 129.5 (Ar-*C*H), 128.8 (Ar-*C*H), 122.0 (Ar-$C_q$), 120.8 (ImPy-*C*H), 119.8 (ImPy-*C*H), 119.7 (ImPy-*C*H), 117.8 (ImPy-$C_q$), 117.7 (ImPy-*C*H), 96.7 (-C≡C-), 83.8 (-C≡C-), 16.8 (ImPy-$CH_3$); **IR** [$cm^{-1}$]: ṽ =3072, 2926, 2213, 1707, 1619, 1548, 1567, 1484, 1441, 1407, 1380, 1349, 1309, 1287, 1257, 1200, 1156, 1140, 1088, 1068, 1041, 1022, 994, 974, 911, 879, 853, 792, 753, 705, 686, 661, 630, 597, 542, 529, 515, 428; **HR-MS-ESI(+)** calc. $[M+H]^+$ $C_{16}H_{13}N_2^+$ 233.1100; found 233.1075.

Synthesis of **4a**

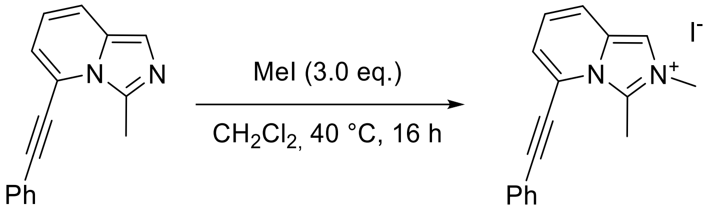


To a solution of **S21** (330 mg, 1.42 mmol, 1.0 eq.) in $CH_2Cl_2$ (10 mL) was added MeI (605 mg, 265 µL, 4.26 mmol, 3.0 eq.). The mixture was stirred for 16 h at 40 °C. Afterwards, $Et_2O$ (40 mL) was added while stirring vigorously. The solution was filtered off and the remaining solid was washed thrice with $Et_2O$ (3x20 mL). Drying under reduced pressure furnished **4a** as a yellow solid (270 mg, 721 µmol, 51%).

**m.p.** 265 °C (decomp.)**;** $^{1}$**H NMR** (500 MHz, DMSO-$d_6$): δ [ppm] = 8.29 (s, 1H, ImPy-H), 7.91 (dd, J = 9.3, 1.1 Hz, 1H, ImPy-H), 7.75 – 7.66 (m, 2H, Ar-H), 7.59 – 7.45 (m, 4H, 3x Ar-H, 1x ImPy-H), 7.20 (dd, J = 9.3, 7.0 Hz, 1H, ImPy-H), 4.09 (s, 3H, ImPy-N-$CH_3$), 3.28 (s, 3H, ImPy-$CH_3$); $^{13}$**C NMR** (126 MHz, DMSO-$d_6$): δ [ppm] = 137.0 (ImPy-$C_q$), 131.2 (Ar-*C*H), 130.5 (Ar-*C*H), 129.1 (Ar-*C*H), 129.0 (ImPy-$C_q$), 124.9 (ImPy-*C*H), 123.2 (ImPy-*C*H), 120.1 (Ar-$C_q$), 119.6 (ImPy-*C*H), 117.5 (ImPy-$C_q$), 115.2 (ImPy-*C*H), 99.2 (-C≡C-), 81.6 (-C≡C-), 36.1 (ImPy-N-$CH_3$), 12.3 (ImPy-$CH_3$); **IR** [cm$^{-1}$]: ṽ = 3092, 2996, 2204, 1643, 1601, 1524, 1443, 1328, 1260, 1197, 1139, 1019, 894, 806, 748, 713, 687, 637, 593, 527, 511, 495, 434; **HR-MS-ESI(+)** calc. $[M]^+$ $C_{17}H_{15}N_2^+$ 247.1200; found 247.1232.

Synthesis of **S22**

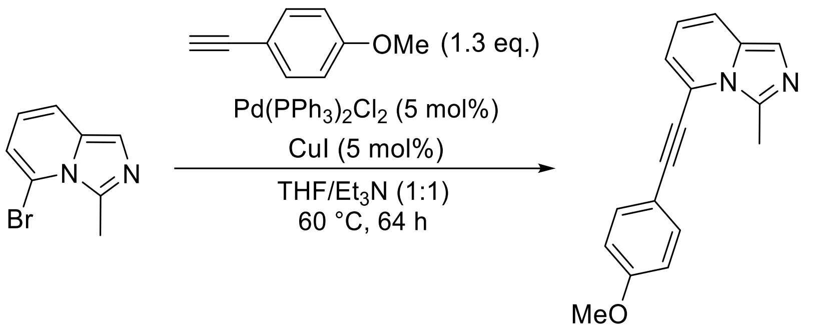


To a degassed solution of dry THF and $NEt_3$ (25 mL, 1:1) under argon was added $Pd(PPh_3)_2Cl_2$ (80 mg, 120 µmol, 5 mol%) and the resulting suspension was stirred for 15 min. Afterwards, **S17** (500 mg, 2.37 mmol, 1.0 eq.), 4-methoxyphenylacetylene (1.05 mL, 3.08 mmol, 1.3 eq.) and CuI (20 mg, 120 µmol, 5 mol%) were added, the reaction mixture was heated to 60 °C and was stirred for 64 h. The mixture was then cooled to rt, the solvent was removed under reduced pressure and the crude product was purified by flash column chromatography (silica, 15% EtOAc in cyclohexane) to yield **S22** (470 mg, 1.80 mmol, 76%) as a yellow solid.

**m.p.** 115 °C**;** **$^1$H NMR** (500 MHz, $CDCl_3$): δ [ppm] = 7.47 (d, *J* = 8.8 Hz, 2H, Ar-H), 7.36 (s, 1H, ImPy-H), 7.34 (dd, *J* = 9.0, 1.1 Hz, 1H, ImPy-H), 6.91 (d, *J* = 8.8 Hz, 2H, Ar-H), 6.77 (dd, *J* = 6.7, 1.2 Hz, 1H, ImPy-H), 6.55 (dd, *J* = 9.1, 6.6 Hz, 1H, ImPy-H), 3.84 (s, 3H, Ar-O-$CH_3$), 3.13 (s, 3H, ImPy-$CH_3$); **$^{13}$C NMR** (126 MHz, $CDCl_3$): δ [ppm] = 160.4 (Ar-$C_q$), 138.0 (ImPy-$C_q$), 132.7 (Ar-*C*H), 131.7 (ImPy-$C_q$), 120.0 (ImPy-*C*H), 119.6 (ImPy-*C*H), 119.1 (ImPy-*C*H), 118.1 (ImPy-$C_q$), 117.5 (ImPy-*C*H), 114.4 (Ar-*C*H), 113.9 (Ar-$C_q$), 96.8 (-C≡C-), 82.60 (-C≡C-), 55.4 (Ar-O-$CH_3$), 16.7 (ImPy-$CH_3$); **IR** [$cm^{-1}$]: ṽ = 2193, 1623, 1601, 1517, 1501, 1443, 1381, 1309, 1290, 1253, 1208, 1193, 1180, 1158, 1108, 1086, 1015, 974, 884, 855, 828, 806, 785, 743, 726, 703, 659, 613, 588, 525, 497, 451; **HR-MS-ESI(+)** calc. $[M+H]^+$ $C_{17}H_{15}N_2O^+$ 263.1200, found 263.1179.

Synthesis of **4c**

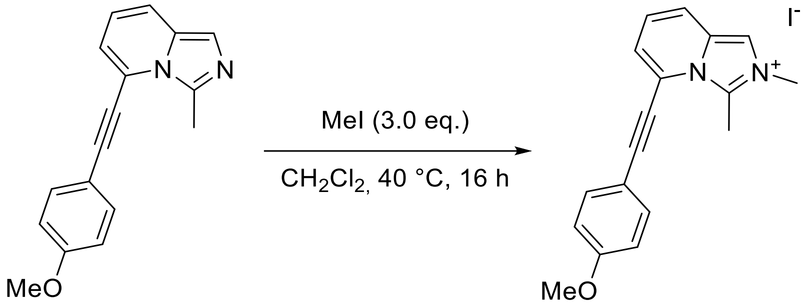


To a solution of **S22** (440 mg, 1.68 mmol, 1.0 eq.) in $CH_2Cl_2$ (10 mL) was added MeI (714 mg, 313 µL, 5.03 mmol, 3.0 eq.). The mixture was stirred for 16 h at 40 °C. Afterwards, $Et_2O$ (40 mL) was added while stirring vigorously. The solution was filtered off and the remaining solid was washed thrice with $Et_2O$ (3x20 mL). Drying under reduced pressure furnished **4c** as an off-white solid (380 mg, 940 µmol, 56%).

**m.p.** 251 °C**;** **$^1$H NMR** (500 MHz, DMSO-$d_6$): δ [ppm] = 8.27 (s, 1H, ImPy-H), 7.86 (dd, J = 9.3, 1.1 Hz, 1H, ImPy-H), 7.64 (m, 2H, Ar-H), 7.45 (dd, J = 7.0, 1.2 Hz, 1H, ImPy-H), 7.18 (dd, J = 9.2, 7.0 Hz, 1H, ImPy-H), 7.11 – 7.06 (m, 2H, Ar-H), 4.08 (s, 3H, ImPy-N-$CH_3$), 3.84 (s, 3H, Ar-O-$CH_3$), 3.27 (s, 3H, ImPy-$CH_3$); **$^{13}$C NMR** (126 MHz, DMSO-$d_6$): δ [ppm] = 160.8 (Ar-$C_q$), 137.0 (ImPy-$C_q$), 133.1 (Ar-*C*H), 129.1 (ImPy-$C_q$), 124.2 (ImPy-*C*H), 123.3 (ImPy-*C*H), 119.1 (ImPy-*C*H), 118.0 (ImPy-$C_q$), 115.1 (ImPy-*C*H), 114.8 (Ar-*C*H), 111.9 (Ar-$C_q$), 99.8 (-C≡C-), 80.5 (-C≡C-), 55.5 (Ar-O-$CH_3$), 36.1 (ImPy-N-$CH_3$), 12.2 (ImPy-$CH_3$); **IR** [$cm^{-1}$]: ṽ = 3080, 2202, 1645, 1603, 1564, 1527, 1506, 1452, 1308, 1293, 1258, 1200, 1178, 1157, 1141, 1106, 1006, 895, 831, 806, 776, 761, 711, 670, 639, 594, 580, 531, 495, 433; **HR-MS-ESI(+)** calc. $[M]^+$ $C_{18}H_{17}N_2O^+$ 277.1300; found 277.1335.

Synthesis of **S23**

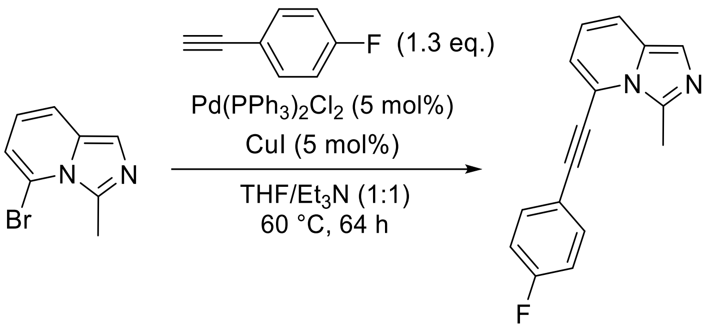

To a degassed solution of dry THF and $NEt_3$ (25 mL, 1:1) under argon was added $Pd(PPh_3)_2Cl_2$ (80 mg, 120 µmol, 5 mol%) and the resulting suspension was stirred for 15 min. Afterwards, **S17** (500 mg, 2.37 mmol, 1.0 eq.), 4-fluorophenylacetylene (1.05 mL, 3.08 mmol, 1.3 eq.) and CuI (20 mg, 120 µmol, 5 mol%) were added, the reaction mixture was heated to 60 °C and was stirred for 64 h. The mixture was then cooled to rt, the solvent was removed under reduced pressure and the crude product was purified by flash column chromatography (silica, 15% EtOAc in cyclohexane) to yield **S23** (540 mg, 2.15 mmol, 91%) as a yellow solid.

**m.p.** 123 °C**;** **$^{1}$H NMR** (500 MHz, $CDCl_3$): δ [ppm] = 7.55 – 7.49 (m, 2H, Ar-H), 7.38 (s, 1H, ImPy-H), 7.38 (d, *J* = 1.2 Hz, 1H, ImPy-H), 7.12 – 7.06 (m, 2H, Ar-H), 6.80 (dd, *J* = 6.7, 1.2 Hz, 1H, ImPy-H), 6.56 (dd, *J* = 9.0, 6.7 Hz, 1H, ImPy-H), 3.12 (s, 3H, ImPy-$CH_3$); **$^{13}$C NMR** (126 MHz, $CDCl_3$): δ [ppm] = 163.2 (d, $^{1}J_{C,F}$ = 251.7 Hz, Ar-$C_q$), 138.0 (ImPy-$C_q$), 133.2 (d, $^{3}J_{C,F}$ = 8.6 Hz, Ar-*C*H), 131.8 (ImPy-$C_q$), 120.8 (ImPy-$C_q$), 119.9 (ImPy-*C*H), 119.7 (ImPy-*C*H), 118.1 (d, $^{4}J_{C,F}$ = 3.5 Hz, Ar-$C_q$), 117.6 (ImPy-$C_q$), 117.5 (ImPy-*C*H), 116.2 (d, $^{2}J_{C,F}$ = 22.4 Hz, Ar-*C*H), 95.6 (-C≡C-), 83.6 (-C≡C-), 16.8 (ImPy-$CH_3$); **$^{19}$F NMR** (565 MHz, $CDCl_3$): δ [ppm] = -113.0 (Ar-*F*); **IR** [$cm^{-1}$]: ṽ = 2211, 1707, 1620, 1597, 1512, 1494, 1434, 1405, 1379, 1347, 1312, 1287, 1224, 1153, 1092, 1039, 1008, 973, 856, 833, 813, 796, 756, 723, 701, 660, 613, 582, 531, 479, 426; **HR-MS-ESI(+)** calc. $[M+H]^+$ $C_{16}H_{12}N_2F^+$ 251.1000; found 251.0980.

Synthesis of **4d**

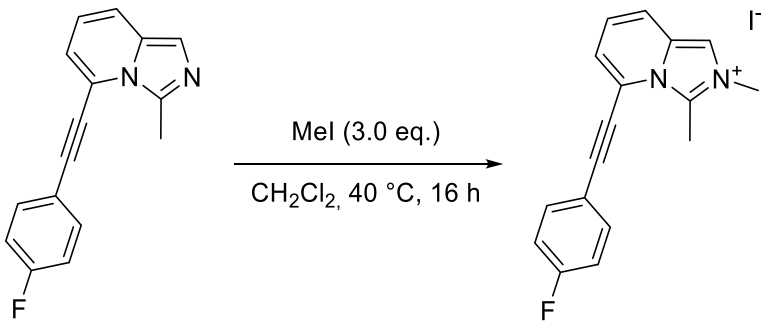

To a solution of **S23** (490 mg, 1.95 mmol, 1.0 eq.) in $CH_2Cl_2$ (10 mL) was added MeI (834 mg, 366 µL, 5.87 mmol, 3.0 eq.). The mixture was stirred for 16 h at 40 °C. Afterwards, $Et_2O$

(40 mL) was added while stirring vigorously. The solution was filtered off and the remaining solid was washed thrice with $Et_2O$ (3x20 mL). Drying under reduced pressure furnished **4d** as an off-white solid (410 mg, 1.05 mmol, 53%).

**m.p.** 307 °C**;** **$^1$H NMR** (500 MHz, DMSO-d$_6$): δ [ppm] = 8.29 (s, 1H, ImPy-H), 7.90 (dd, J = 9.2, 1.2 Hz, 1H, ImPy-H), 7.81 – 7.75 (m, 2H, Ar-H), 7.51 (dd, J = 6.9, 1.1 Hz, 1H, ImPy-H), 7.40 (m, 2H, Ar-H), 7.20 (dd, J = 9.3, 7.0 Hz, 1H, ImPy-H), 4.08 (s, 3H, ImPy-N-$CH_3$), 3.26 (s, 3H, ImPy-$CH_3$); **$^{13}$C NMR** (126 MHz, DMSO-d$_6$): δ [ppm] = 163.0 (d, $^1J_{C,F}$ = 250.3 Hz, Ar-$C_q$), 137.1 (ImPy-$C_q$), 134.0 (d, $^3J_{C,F}$ = 9.0 Hz, Ar-*C*H), 129.0 (ImPy-$C_q$), 125.0 (ImPy-*C*H), 123.2 (ImPy-*C*H), 119.7 (ImPy-*C*H), 117.5 (ImPy-$C_q$), 116.7 (d, $^4J_{C,F}$ = 3.5 Hz, Ar-$C_q$), 116.6 (d, $^2J_{C,F}$ = 22.4 Hz, Ar-*C*H) 115.3 (ImPy-*C*H), 98.3 (-C≡C-), 81.3 (-C≡C-), 36.1 (ImPy-N-*C*$H_3$), 12.2 (ImPy-*C*$H_3$); **$^{19}$F NMR** (565 MHz, DMSO-d$_6$): δ [ppm] = -107.6 (Ar-*F*); **IR** [cm$^{-1}$]: ṽ = 3091, 2207, 1644, 1598, 1525, 1501, 1404, 1328, 1227, 1196, 1160, 1137, 1098, 1015, 893, 837, 803, 783, 713, 670, 638, 593, 578, 541, 528, 514, 472; **HR-MS-ESI(+)** calc. $[M]^+$ $C_{17}H_{14}FN_2^+$ 265.1100; found 265.1134.

Synthesis of **S24**

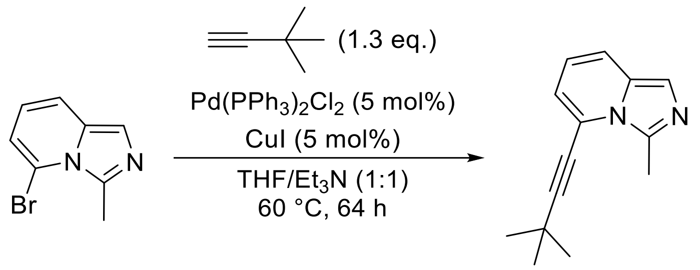


To a degassed solution of dry THF and $NEt_3$ (20 mL, 1:1) under argon was added $Pd(PPh_3)_2Cl_2$ (60 mg, 90.0 µmol, 5 mol%) and the resulting suspension was stirred for 15 min. Afterwards, **S17** (380 mg, 1.80 mmol, 1.0 eq.), 3,3-dimethylbut-1-yne (290 µL, 2.34 mmol, 1.3 eq.) and CuI (20 mg, 120 µmol, 5 mol%) were added, the reaction mixture was heated to 60 °C and was stirred for 64 h. The mixture was then cooled to rt, the solvent was removed under reduced pressure and the crude product was purified by flash column chromatography (silica, 15% EtOAc in cyclohexane) to yield **S24** (350 mg, 1.66 mmol, 92%) as a dark brown-orange oil.

**m.p.** <25 °C**;** **$^1$H NMR** (500 MHz, $CDCl_3$): δ [ppm] = 7.34 (s, 1H, ImPy-H), 7.30 (dd, *J* = 9.1, 1.2 Hz, 1H, ImPy-H), 6.65 (dd, *J* = 6.7, 1.2 Hz, 1H, ImPy-H), 6.53 (dd, *J* = 9.1, 6.6 Hz, 1H, ImPy-H), 3.07 (s, 3H, ImPy-$CH_3$), 1.35 (s, 9H, $C(CH_3)_3$); **$^{13}$C NMR** (126 MHz, $CDCl_3$): δ [ppm] = 138.0 (ImPy-$C_q$), 131.8 (ImPy-$C_q$), 119.8 (ImPy-*C*H) ,119.4 (ImPy-*C*H), 118.8 (ImPy-*C*H), 118.4 (ImPy-$C_q$), 117.6 (ImPy-*C*H), 105.7 (-C≡C-), 74.2 (-C≡C-), 30.3 (C(*C*$H_3$)$_3$), 28.5 (*C*($CH_3$)$_3$), 17.0 (ImPy-$CH_3$); **IR** [cm$^{-1}$]: ṽ = 2967, 2928, 2867, 2221, 1710, 1620, 1475, 1457, 1407, 1378, 1341, 1302, 1259, 1202, 1174, 1148, 1054, 1031, 974, 895, 822, 794, 755, 727,

705, 661, 613, 555, 542, 515; **HR-MS-ESI(+)** calc. $[M+H]^+$ $C_{14}H_{17}N_2^+$ 213.1400; found 213.1387.

Synthesis of **4e**

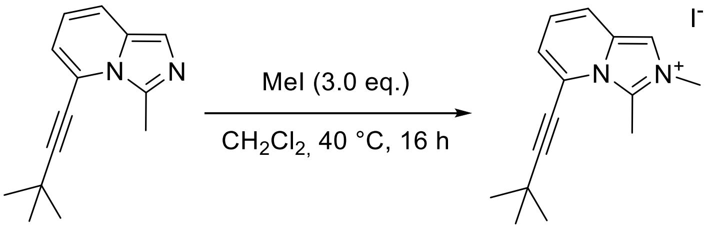


To a solution of **S24** (300 mg, 1.41 mmol, 1.0 eq.) in $CH_2Cl_2$ (10 mL) was added MeI (602 mg, 264 µL, 4.24 mmol, 3.0 eq.). The mixture was stirred for 16 h at 40 °C. Afterwards, $Et_2O$ (40 mL) was added while stirring vigorously. The solution was filtered off and the remaining solid was washed thrice with $Et_2O$ (3x20 mL). Drying under reduced pressure furnished **4e** as an off-white solid (320 mg, 903 µmol, 64%).

**m.p.** 260 °C**;** $^{1}$**H NMR** (500 MHz, DMSO-d$_6$): δ [ppm] = 8.2 (s, 1H, ImPy-H), 7.8 (dd, J = 9.3, 1.1 Hz, 1H, ImPy-H), 7.3 (dd, J = 6.9, 1.1 Hz, 1H, ImPy-H), 7.1 (dd, J = 9.2, 6.9 Hz, 1H, ImPy-H), 4.0 (s, 3H, ImPy-N-$CH_3$), 3.2 (s, 3H, ImPy-$CH_3$), 1.4 (s, 9H, $C(CH_3)_3$); $^{13}$**C NMR** (126 MHz, DMSO-d$_6$): δ [ppm] = 136.8 (ImPy-$C_q$), 129.0 (ImPy-$C_q$), 124.0 (ImPy-*C*H), 123.1 (ImPy-*C*H), 118.9 (ImPy-*C*H), 117.9 (ImPy-$C_q$), 114.9 (ImPy-*C*H), 109.1 (-C≡C-), 72.0 (-C≡C-), 36.0 (ImPy-N-*C*$H_3$), 29.4 (C(*C*$H_3$)$_3$), 28.2 (*C*($CH_3$)$_3$), 12.3 (ImPy-*C*$H_3$); **IR** [cm$^{-1}$]: ṽ = 3026, 2966, 2214, 1649, 1592, 1525, 1458, 1393, 1361, 1316, 1282, 1237, 1213, 1137, 1070, 862, 819, 773, 713, 639, 546, 515, 454, 429; **HR-MS-ESI(+)** calc. $[M]^+$ $C_{15}H_{19}N_2^+$ 227.1500; found 227.1552.

Synthesis of **S25**

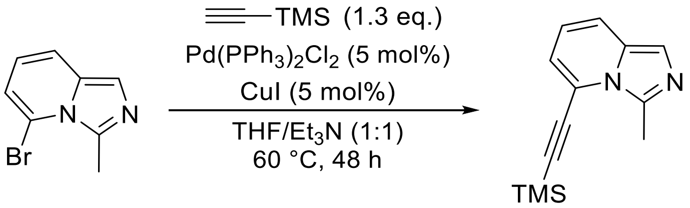


To a degassed solution of dry THF and $NEt_3$ (76 mL, 1:1) under argon was added $Pd(PPh_3)_2Cl_2$ (250 mg, 360 µmol, 5 mol%) and the resulting suspension was stirred for 15 min. Afterwards, **S17** (1.50 g, 7.11 mmol, 1.0 eq.), trimethylsilylacetylene (1.29 mL, 9.24 mmol, 1.3 eq.) and CuI (70 mg, 360 µmol, 5 mol%) were added, the reaction mixture was heated to 60 °C and was stirred for 48 h. The mixture was then cooled to rt, the solvent was removed under reduced pressure and the crude product was purified by flash column chromatography (silica, 10% to

25% EtOAc in cyclohexane) to yield **S25** (1.14 g, 4.99 mmol, 70%) as a dark brown-orange oil.

**m.p.** <25 °C**;** $^{1}$**H NMR** (500 MHz, $CDCl_3$): δ [ppm] = 7.35 (s, 1H, ImPy-H), 7.36 – 7.32 (dd, 1H, ImPy-H), 6.75 (dd, J = 6.7, 1.1 Hz, 1H, ImPy-H), 6.50 (dd, J = 9.1, 6.7 Hz, 1H, ImPy-H), 3.04 (s, 3H, ImPy-$CH_3$), 0.28 (s, 9H, -Si-$(CH_3)_3$); $^{13}$**C NMR** (126 MHz, $CDCl_3$): δ [ppm] = 138.3 (ImPy-$C_q$), 131.7 (ImPy-$C_q$), 121.4 (ImPy-*C*H), 120.0 (ImPy-*C*H), 119.9 (ImPy-*C*H), 117.6 (ImPy-$C_q$), 117.3 (ImPy-*C*H), 103.7 (-C≡C-), 98.7 (-C≡C-), 16.7 (ImPy-$CH_3$), -0.55 (-Si-$(CH_3)_3$; **IR** [$cm^{-1}$]: ṽ = 2960, 2153, 1620, 1488, 1438, 1408, 1381, 1336, 1298, 1249, 1175, 1096, 1033, 975, 838, 796, 757, 702, 661, 628, 548, 453; **HR-MS-ESI(+)** calc. $[M+H]^+$ $C_{13}H_{17}N_2Si^+$ 228.1100; found 229.1158.

Synthesis of **S26**

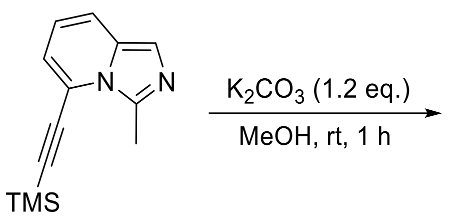


In a round-bottom flask **S25** (1.05 g, 4.58 mmol, 1.0 eq.) was dissolved in MeOH (10 mL) and $K_2CO_3$ (760 mg, 5.49 mmol, 1.2 eq.) was added. The orange suspension was stirred for 1 h at rt. MeOH was removed under reduced pressure and the residue was dissolved as a two-phase-mixture in $CH_2Cl_2$ and $H_2O$. The aqueous phase was extracted with $CH_2Cl_2$ (4x30 mL) and dried over $Na_2SO_4$ to give **S26** (707 mg, 4.53 mmol, 99%) as an orange-brown solid.

**m.p.** 103 °C**;** $^{1}$**H NMR** (500 MHz, $CDCl_3$): δ [ppm] = 7.39 – 7.36 (m, 2H, ImPy-H), 6.80 (dd, *J* = 6.7, 1.2 Hz, 1H, ImPy-H), 6.52 (dd, *J* = 9.1, 6.6 Hz, 1H, ImPy-H), 3.59 (s, 1H, ImPy-C≡C-H), 3.04 (s, 3H, ImPy-$CH_3$); $^{13}$**C NMR** (126 MHz, $CDCl_3$): δ [ppm] = 138.3 (ImPy-$C_q$), 131.6 (ImPy-$C_q$), 122.2 (ImPy-*C*H), 120.4 (ImPy-*C*H), 120.0 (ImPy-*C*H), 117.2 (ImPy-*C*H), 116.6 (ImPy-$C_q$), 85.6 (ImPy-C≡*C*-H), 77.9 (ImPy-*C*≡C-H), 16.6 (ImPy-$CH_3$); **IR** [$cm^{-1}$]: ṽ = 3147, 3105, 2091, 1620, 1552, 1506, 1485, 1433, 1411, 1380, 1336, 1299, 1263, 1232, 1174, 1148, 1094, 1054, 1039, 1025, 975, 877, 837, 803, 786, 755, 714, 699, 659, 639, 602, 541, 524, 489, 468, 442, 408; **HR-MS-ESI(+)** calc. $C_{10}H_9N_2^+$ $[M+H]^+$ 157.0760; found 157.0761.

Synthesis of **4f**

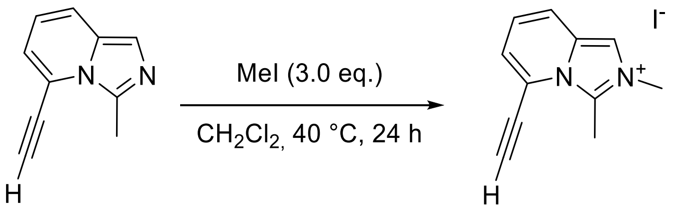

To a solution of **S26** (200 mg, 1.28 mmol, 1.0 eq.) in $CH_2Cl_2$ (10 mL) was added MeI (545 mg, 239 µL, 3.84 mmol, 3.0 eq.). The mixture was stirred for 24 h at 40 °C. Afterwards, $Et_2O$ (40 mL) was added while stirring vigorously. The solution was filtered off and the remaining solid was washed thrice with $Et_2O$ (3x20 mL). Drying under reduced pressure furnished **4f** as an off-white solid (261 mg, 875 µmol, 68%).

**m.p.** 205 (decomp.) °C**;** $^{1}$**H NMR** (500 MHz, DMSO-$d_6$): δ [ppm] = 8.28 (s, 1H, ImPy-H), 7.90 (dd, *J* = 9.2, 1.1 Hz, 1H, ImPy-H), 7.44 (dd, *J* = 7.0, 1.1 Hz, 1H, ImPy-H), 7.14 (dd, *J* = 9.3, 6.9 Hz, 1H, ImPy-H), 5.34 (s, 1H, ImPy-C≡C-H), 4.06 (s, 3H, ImPy-N-$CH_3$), 3.19 (s, 3H, ImPy-$CH_3$); $^{13}$**C NMR** (126 MHz, DMSO-$d_6$): δ [ppm] = 137.1 (ImPy-$C_q$), 128.9 (ImPy-$C_q$), 125.9 (ImPy-*C*H), 122.9 (ImPy-*C*H), 120.1 (ImPy-*C*H), 116.8 (ImPy-$C_q$), 115.2 (ImPy-*C*H), 92.6 (ImPy-C≡*C*-H), 75.5 (ImPy-*C*≡C-H), 36.1 (ImPy-N-*C*$H_3$), 12.2 (ImPy-*C*$H_3$); **IR** [$cm^{-1}$]: ṽ = 3150, 3067, 2157, 1648, 1609, 1569, 1527, 1480, 1438, 1399, 1379, 1320, 1249, 1210, 1175, 1136, 1086, 1064, 1028, 886, 800, 748, 686, 621, 592, 543, 458, 419; **HR-MS-ESI(+)** calc. $C_{17}H_{17}N_2^+$ $[M]^+$ 171.0917; found 171.0921.

Synthesis of **S27**

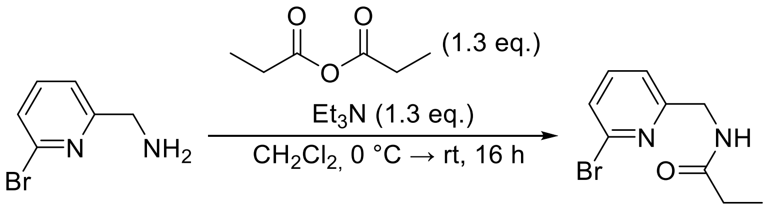


To a solution of **S16** (2.48 g, 13.3 mmol, 1.0 eq.) in $CH_2Cl_2$ (40 mL) under argon atmosphere, triethylamine (2.40 mL, 17.2 mmol, 1.3 eq.) was added at once. The solution was cooled to 0 °C and propionic anhydride (2.20 mL, 17.2 mmol, 1.3 eq.) was added dropwise. After full addition, the reaction mixture was stirred at rt for 16 h. Afterwards, the solution was quenched by addition of saturated aqueous sodium bicarbonate solution (50 mL) and extracted four times with $CH_2Cl_2$ (4x80 mL). The combined organic extracts were dried over $Na_2SO_4$ and the solvent was removed under reduced pressure. Purification by flash column chromatography (silica, 80% EtOAc in cyclohexane) furnished **S27** (2.83 g,11.6 mmol, 88%) as colorless solid.

**m.p.** 41 °C**;** $^{1}$**H NMR** (500 MHz, $CDCl_3$): δ [ppm] = 7.43 (t, *J* = 7.7 Hz, 1H, Py-H), 7.29 (d, *J* = 7.8 Hz, 1H, Py-H), 7.16 (d, *J* = 7.6 Hz, 1H, Py-H), 6.88 (s, 1H, Py-$CH_2$-N*H*), 4.41 (d, *J* = 5.5 Hz, 2H, Py-C*H*$_2$-NH), 2.21 (q, *J* = 7.6 Hz, 2H, (C=O)C*H*$_2$$CH_3$), 1.09 (t, *J* = 7.6 Hz, 3H, (C=O)$CH_2$C*H*$_3$); $^{13}$**C NMR** (126 MHz, $CDCl_3$): δ [ppm] = 174.2 ((*C*=O)$CH_2CH_3$), 158.8 (Py-$C_q$), 141.4 (Py-$C_q$), 139.1 (Py-*C*H), 126.6 (Py-*C*H), 120.8 (Py-*C*H), 44.1 (Py-*C*$H_2$-NH), 29.4 ((C=O)*C*$H_2CH_3$), 9.7 ((C=O)$CH_2$*C*$H_3$); **IR** [$cm^{-1}$]: ṽ = 3309, 3042, 2977, 2939, 1649, 1584, 1550, 1443, 1430, 1410, 1371, 1233, 1132, 1107, 1032, 986, 835, 785, 721, 673, 514; **HR-MS-ESI(+)** calc. $C_9H_{12}BrN_2O^+$ $[M+H]^+$ 243.0128; found 243.0130.

Synthesis of **S28**

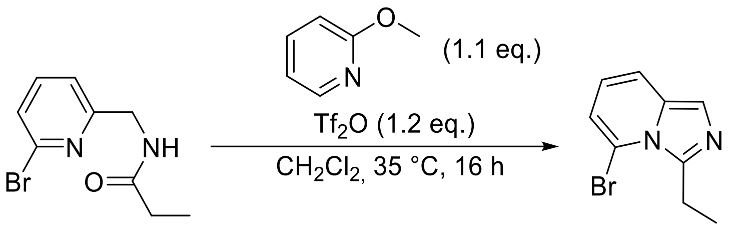

To a solution of **S27** (500 mg, 2.06 mmol, 1.0 eq.) and 2-methoxypyridine (238 µL, 247 mg, 2.26 mmol, 1.1 eq.) in dry $CH_2Cl_2$ (0.5 M, 4 mL) was added $Tf_2O$ (415 µL, 696 mg, 2.47 mmol, 1.2 eq.) at rt. Afterwards, the solution was heated to 35 °C and stirred for 16 h. The mixture was allowed to cool to rt and saturated aqueous $NaHCO_3$ solution (50 mL) was added, which was then extracted thrice ($CH_2Cl_2$, 3x50 mL), dried over $Na_2SO_4$ and filtered off. The solvent was removed under reduced pressure and purification by flash column chromatography (silica, 15% EtOAc to 50% EtOAc in cyclohexane) furnished **S28** (392 mg, 1.74 mmol, 85%) as light-yellow oil.

**m.p.** <25 °C**;** $^1$**H NMR** (500 MHz, $CDCl_3$): δ [ppm] = 7.39 (s, 1H, ImPy-H), 7.35 (dd, *J* = 8.9, 1.1 Hz, 1H, ImPy-H), 6.73 (dd, *J* = 6.7, 1.1 Hz, 1H, ImPy-H), 6.42 (dd, *J* = 9.0, 6.7 Hz, 1H, ImPy-H), 3.52 (q, *J* = 7.4 Hz, 2H, ImPy-C$H_2$CH$_3$), 1.45 (t, *J* = 7.4 Hz, 3H, ImPy-CH$_2$C$H_3$); $^{13}$**C NMR** (126 MHz, $CDCl_3$): δ [ppm] = 143.1 (ImPy-$C_q$), 133.8 (ImPy-$C_q$), 119.9 (ImPy-*C*H), 118.7 (ImPy-*C*H), 118.2 (ImPy-*C*H), 118.2 (ImPy-*C*H), 111.2 (ImPy-$C_q$-Br), 24.7 (ImPy-*C*$H_2$CH$_3$), 14.0 (ImPy-CH$_2$C$H_3$); **IR** [cm$^{-1}$]: ṽ = 2975, 2932, 2872, 1624, 1496, 1480, 1451, 1411, 1371, 1336, 1298, 1279, 1241, 1201, 1160, 1084, 1021, 953, 939, 797, 752, 693, 655; **HR-MS-ESI(+)** calc. $C_9H_{10}BrN_2^+$ $[M+H]^+$ 225.0022; found 225.0021.

Synthesis of **S29**

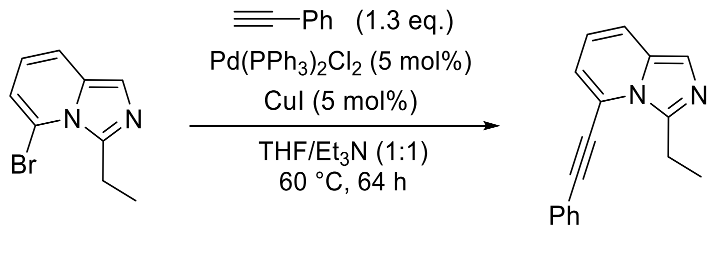

To a degassed solution of dry THF and $NEt_3$ (20 mL, 1:1) under argon was added $Pd(PPh_3)_2Cl_2$ (57.7 mg, 82.2 µmol, 5 mol%) and the resulting suspension was stirred for 15 min. Afterwards, **S28** (370 mg, 1.64 mmol, 1.0 eq.), phenylacetylene (218 mg, 235 µL, 2.14 mmol, 1.3 eq.) and CuI (15.7 mg, 82.2 µmol, 5 mol%) were added, the reaction mixture was heated to 60 °C and was stirred for 64 h. The mixture was then cooled to rt, the solvent was removed under reduced pressure and the crude product was purified by flash column chromatography (silica, 25% to 50% EtOAc in cyclohexane) to yield **S29** (340 mg, 1.38 mmol, 84%) as a brown oil.

**m.p.** <25 °C**;** **$^1$H NMR** (600 MHz, $CDCl_3$): δ [ppm] = 7.57 – 7.54 (m, 2H, Ar-H), 7.49 (s, 1H, ImPy-H), 7.43 – 7.38 (m, 4H, 1 ImPy-H and 3 Ar-H), 6.86 (d, *J* = 6.6 Hz, 1H, ImPy-H), 6.59 (dd, *J* = 9.0, 6.6 Hz, 1H, ImPy-H), 3.61 (q, *J* = 7.4 Hz, 2H, ImPy-C$H_2$CH$_3$), 1.50 (d, *J* = 6.5 Hz, 3H, ImPy-CH$_2$C$H_3$); **$^{13}$C NMR** (151 MHz, $CDCl_3$): δ [ppm] = 132.3 (ImPy-$C_q$), 132.3 (ImPy-$C_q$), 131.3 (Ar-*C*H), 129.4 (Ar-*C*H), 128.8 (Ar-*C*H), 122.1 (Ar-$C_q$), 121.1 (ImPy-*C*H), 120.2 (ImPy-*C*H), 119.9 (ImPy-*C*H), 117.6 (ImPy-$C_q$), 117.5 (ImPy-*C*H), 96.2 (-C≡C-), 83.9 (-C≡C-), 23.2 (ImPy-*C*H$_2$CH$_3$), 13.7 (ImPy-CH$_2$*C*H$_3$); **IR** [cm$^{-1}$]: ṽ = 2937, 2853, 2795, 1707, 1620, 1483, 1442, 1367, 1307, 1199, 1105, 1032, 1011, 858, 802, 755, 689; **HR-MS-ESI(+)** calc. $C_{17}H_{15}N_2^+$ $[M+H]^+$ 247.1230; found 247.1233.

Synthesis of **4g**

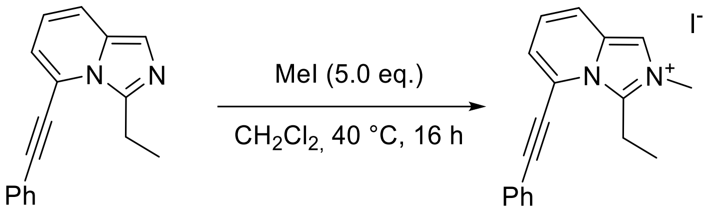


To a solution of **S29** (340 mg, 1.38 mmol, 1.0 eq.) in $CH_2Cl_2$ (10 mL) was added MeI (980 mg, 430 µL, 6.90 mmol, 5.0 eq.). The mixture was stirred for 16 h at 40 °C. Afterwards, $Et_2O$ (40 mL) was added while stirring vigorously. The solution was filtered off and the remaining solid was washed thrice with $Et_2O$ (3x20 mL). Drying under reduced pressure furnished **4g** as a yellow solid (450 mg, 1.16 mmol, 84%).

**m.p.** 245 °C**;** **$^1$H NMR** (600 MHz, DMSO-d$_6$): δ [ppm] = 8.34 (s, 1H, ImPy-H), 7.94 (dd, *J* = 9.3, 1.1 Hz, 1H, ImPy-H), 7.72 – 7.67 (m, 2H, Ar-H), 7.60 – 7.51 (m, 4H, 1 ImPy-H and 3 Ar-H), 7.24 (dd, *J* = 9.2, 7.0 Hz, 1H, ImPy-H), 4.15 (s, 3H, ImPy-N-CH$_3$), 3.76 (q, *J* = 7.6 Hz, 2H, ImPy-C$H_2$CH$_3$), 1.44 (t, *J* = 7.6 Hz, 3H, ImPy-CH$_2$C$H_3$); **$^{13}$C NMR** (151 MHz, DMSO-d$_6$): δ [ppm] = 140.0 (ImPy-$C_q$), 131.4 (Ar-*C*H), 130.5 (Ar-*C*H), 129.2 (ImPy-$C_q$), 129.2 (Ar-*C*H), 125.5 (ImPy-*C*H), 123.3 (ImPy-*C*H), 120.1 (Ar-$C_q$), 119.8 (ImPy-*C*H), 117.1 (ImPy-$C_q$), 115.7 (ImPy-*C*H), 98.3 (-C≡C-), 81.1 (-C≡C-), 35.8 (ImPy-N-CH$_3$), 18.2 (ImPy-*C*H$_2$CH$_3$), 12.5 (ImPy-CH$_2$*C*H$_3$); **IR** [cm$^{-1}$]: ṽ = 3062, 2205, 1645, 1568, 1520, 1481, 1437, 1332, 1307, 1141, 1099, 1072, 1057, 806, 753, 734, 718, 688, 653, 593, 549, 532, 438; **HR-MS-ESI(+)** calc. $C_{18}H_{17}N_2^+$ $[M]^+$ 261.1386; found 261.1389.

Synthesis of **S31**

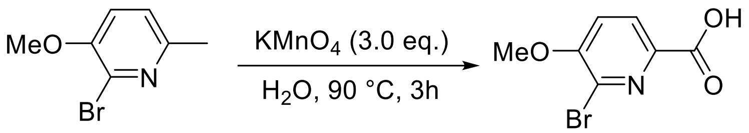

A suspension of 2-bromo-3-methoxy-6-methylpyridine (**S30**, 3.00 g, 15.3 mmol, 1.0 eq.) in water (60 mL) was heated to 90 °C. $KMnO_4$ (7.04 g, 44.5 mmol, 3.0 eq.) was added in portions over 30 min. Afterwards, the deep purple solution was filtered over Celite while still hot and was subsequently cooled down and acidified to pH 2 with concentrated hydrochloric acid. The resulting precipitate was collected by filtration and washed with water. Then, the solvent was removed under reduced pressure to yield **S31** (1.66 g, 7.15 mmol, 48%) as colorless solid. The spectroscopic data agrees with literature data.[7]

**$^1$H NMR** (500 MHz, DMSO-$d_6$): δ [ppm] = 13.18 (s, 1H, Py-$CO_2$H), 8.05 (d, *J* = 8.4 Hz, 1H, Py-H), 7.60 (d, *J* = 8.5 Hz, 1H, Py-H), 3.97 (s, 3H, Py-O-$CH_3$), **$^{13}$C NMR** (126 MHz, DMSO-$d_6$): δ [ppm] = 164.6 (Py-*C*$O_2$H), 155.0 (Py-$C_q$), 140.0 (Py-$C_q$), 131.1 (Py-$C_q$), 126.4 (Py-*C*H), 119.8 (Py-*C*H), 57.0 (Py-O-*C*$H_3$).

Synthesis of **S32**

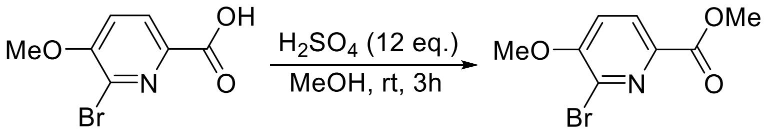

To a suspension of **S31** (1.60 g, 6.90 mmol, 1.0 eq.) in MeOH (35 mL) was added $H_2SO_4$ (4.43 mL, 82.6 mmol, 12 eq.) dropwise and the suspension was stirred over 3 h at rt. Afterwards, the suspension was poured into 200 mL saturated aqueous $NaHCO_3$ solution, which resulted in strong gas evolution. Then, the reaction mixture was extracted twice with $CH_2Cl_2$ (2x100 mL) and the combined organic extracts were dried over $Na_2SO_4$. After filtration and removal of the solvent under reduced pressure, **S32** (1.48 g, 6.01 mmol, 87%) was obtained as a colorless solid. The spectroscopic data agrees with literature data.[7]

**$^1$H NMR** (500 MHz, $CDCl_3$): δ [ppm] = 8.07 (d, *J* = 8.0 Hz, 1H, Py-H), 7.19 (d, *J* = 8.2 Hz, 1H, Py-H), 3.97 (s, 3H, Py-O-$CH_3$), 3.95 (s, 3H, Py-$CO_2CH_3$), **$^{13}$C NMR** (126 MHz, $CDCl_3$): δ [ppm] = 164.5 (Py-*C*$O_2CH_3$), 155.9 (Py-$C_q$), 139.9 (Py-$C_q$), 132.7 (Py-$C_q$), 126.2 (Py-*C*H), 117.9 (Py-*C*H), 56.7 (Py-O-*C*$H_3$), 53.0 (Py-*C*$H_3$).

Synthesis of **S33**

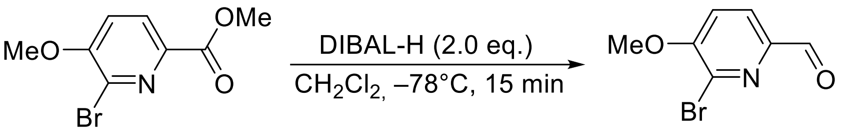

To a solution of **S32** (1.35 g, 5.49 mmol, 1.0 eq.) in dry $CH_2Cl_2$ (30 mL) under an argon atmosphere was added DIBAL-H (9.14 mL, 11.0 mmol, 2.0 eq., 1.2 M in toluene) at –78 °C dropwise over 2 min. The solution was stirred for 15 min and then MeOH (9 mL) was added and the solution was allowed to thaw up to rt over 1 h. Then, the reaction mixture was diluted with 120 mL of $CH_2Cl_2$ and an aqueous solution of Rochelle salt (100 mL) was added. The organic phase was then washed with water (100 mL) and brine (100 mL), subsequently dried over $Na_2SO_4$ and after filtration the solvent was removed under reduced pressure to yield **S33** (1.13 g, 5.23 mmol, 95%) as a colorless solid.

**m.p.** 148 °C**;** $^{1}$**H NMR** (500 MHz, $CDCl_3$): δ [ppm] = 9.93 (d, *J* = 0.8 Hz, 1H, Py-CHO), 7.95 (d, *J* = 8.3 Hz, 1H, Py-H), 7.25 (dd, *J* = 8.1, 0.9 Hz, 1H, Py-H), 4.02 (s, 3H, Py-O-$CH_3$); $^{13}$**C NMR** (126 MHz, $CDCl_3$): δ [ppm] = 190.8 (Py-*C*HO), 156.7 (Py-$C_q$), 145.9 (Py-$C_q$), 133.1 (Py-$C_q$), 122.7 (Py-*C*H), 118.2 (Py-*C*H), 56.9 (Py-O-$CH_3$); **IR** [$cm^{-1}$]: ṽ = 3075, 2982, 2948, 1729, 1574, 1560, 1431, 1420, 1382, 1312, 1279, 1255, 1196, 1154, 1129, 1072, 998, 978, 851, 779, 722, 681, 626; **HR-MS-ESI(+)** calc. $C_7H_7BrNO_2^+$ $[M+H]^+$ 215.9655; found 215.9660.

Synthesis of **S34**

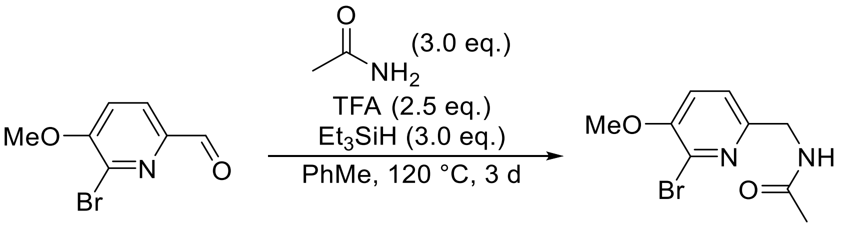

To a solution of **S33** (500 mg, 2.31 mmol, 1.0 eq.) in dry toluene (10 mL) in a sealed vessel was added acetamide (410 mg, 6.94 mmol, 3.0 eq.), triethylsilane (1.11 mL, 807 mg, 6.94 mmol, 3.0 eq.) and finally trifluoroacetic acid (446 µL, 660 mg, 5.79 mmol, 2.5 eq.). The reaction mixture was heated to 120 °C and was stirred over 3 days. Afterwards, the reaction mixture was allowed to cool to rt and saturated aqueous $NaHCO_3$ solution (150 mL) was added. The mixture was extracted with $CH_2Cl_2$ thrice (3x100 mL), the organic extracts were dried over $Na_2SO_4$ and filtered off. After removal of the solvent under reduced pressure, the crude product was purified by flash column chromatography (silica, 80% EtOAc in cyclohexane to 100% EtOAc) to obtain **S34** (320 mg, 1.24 mmol, 53%) as a pale-yellow solid.

**m.p.** 115 °C**;** $^{1}$**H NMR** (500 MHz, $CDCl_3$): δ [ppm] = 7.23 (d, *J* = 8.2 Hz, 1H, Py-H), 7.11 (d, *J* = 8.2 Hz, 1H, Py-H), 6.43 (s, 1H, Py-$CH_2$-N*H*), 4.44 (d, *J* = 5.5 Hz, 2H, Py-C*H*$_2$-NH), 3.90 (s, 3H, Py-O-$CH_3$), 2.03 (s, 3H, C(=O)$CH_3$); $^{13}$**C NMR** (126 MHz, $CDCl_3$): δ [ppm] = 170.2 (*C*(=O)NH), 152.2 (Py-$C_q$), 149.1 (Py-$C_q$), 131.9 (Py-$C_q$), 122.4 (Py-*C*H), 119.5 (Py-*C*H), 56.5 (Py-O-*C*H$_3$), 43.7 (Py-*C*H$_2$NH), 23.4 ($CH_3$*C*(=O)NH); **IR** [cm$^{-1}$]: ṽ = 3296, 3065, 3018, 2980, 2923, 2852, 1628, 1559, 1533, 1461, 1432, 1378, 1357, 1293, 1264, 1205, 1081, 1011, 845, 671, 632, 590, 564, 484, 465; **HR-MS-ESI(+)** calc. $C_9H_{12}BrN_2O_2^+$ $[M+H]^+$ 259.0077; found 259.0077.

Synthesis of **S35**

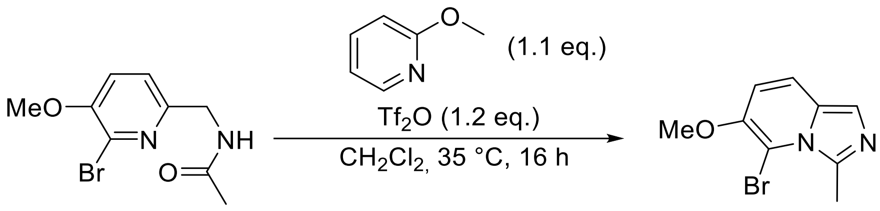


To a solution of **S34** (500 mg, 1.93 mmol, 1.0 eq.) and 2-methoxypyridine (222 µL, 232 mg, 2.12 mmol, 1.1 eq.) in dry $CH_2Cl_2$ (0.5M, 4 mL) was added $Tf_2O$ (389 µL, 653 mg, 2.32 mmol, 1.2 eq.) at rt. Afterwards, the solution was heated to 35 °C and stirred for 16 h. The mixture was allowed to cool to rt and saturated aqueous $NaHCO_3$ solution (50 mL) was added, which was then extracted thrice ($CH_2Cl_2$, 3x50 mL), dried over $Na_2SO_4$ and filtered off. The solvent was removed under reduced pressure and purification by flash column chromatography (silica, 15% EtOAc in cyclohexane to 100% EtOAc) yielded **S35** (420 mg, 1.74 mmol, 90%) as a light-yellow solid.

**m.p.** 96 °C**;** $^{1}$**H NMR** (700 MHz, $CDCl_3$): δ [ppm] = 7.32 (s, 1H, ImPy-H), 7.30 (d, *J* = 9.5 Hz, 1H, ImPy-H), 6.57 (d, *J* = 9.6 Hz, 1H, ImPy-H), 3.86 (s, 3H, ImPy-O-$CH_3$), 3.07 (s, 3H, ImPy-$CH_3$); $^{13}$**C NMR** (176 MHz, $CDCl_3$): δ [ppm] = 146.3 (ImPy-$C_q$), 138.7 (ImPy-$C_q$), 131.5 (ImPy-$C_q$), 120.0 (ImPy-*C*H), 118.3 (ImPy-*C*H), 111.6 (ImPy-*C*H), 102.0 (ImPy-$C_q$-Br), 58.9 (ImPy-O-*C*H$_3$), 19.0 (ImPy-*C*H$_3$); **IR** [cm$^{-1}$]: ṽ = 3097, 3064, 2925, 2848, 1632, 1544, 1487, 1458, 1443, 1421, 1367, 1305, 1275, 1238, 1179, 1082, 1045, 977, 958, 869, 813; **HR-MS-ESI(+)** calc. $C_9H_{10}BrN_2O^+$ $[M+H]^+$ 240.9971; found 242.9971.

Synthesis of **S36**

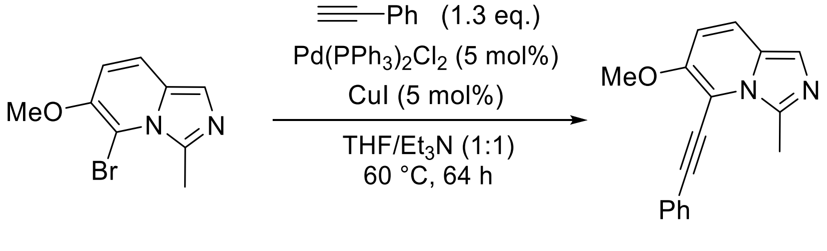

To a degassed solution of dry THF and $NEt_3$ (16 mL, 1:1) under argon was added $Pd(PPh_3)_2Cl_2$ (46.0 mg, 65.9 µmol, 5 mol%) and the resulting suspension was stirred for 15 min. Afterwards, **S35** (318 mg, 1.32 mmol, 1.0 eq.), phenylacetylene (175 mg, 188 µL, 1.71 mmol, 1.3 eq.) and CuI (12.5 mg, 65.9 µmol, 5 mol%) were added, the reaction mixture was heated to 60 °C and was stirred for 64 h. The mixture was then cooled to rt, the solvent was removed under reduced pressure and the crude product was purified by flash column chromatography (silica, 25% to 50% EtOAc in cyclohexane) to yield **S36** (210 mg, 800 µmol, 61%) as a brown oil.

**m.p.** <25 °C**;** $^{1}$**H NMR** (500 MHz, $CDCl_3$): δ [ppm] = 7.60 – 7.51 (m, 2H, Ar-H), 7.42 – 7.33 (m, 5H, 2 ImPy-H and 3 Ar-H), 6.65 (d, *J* = 9.7 Hz, 1H, ImPy-H), 3.97 (s, 3H, ImPy-O-$CH_3$), 3.13 (s, 3H, ImPy-$CH_3$); $^{13}$**C NMR** (126 MHz, $CDCl_3$): δ [ppm] = 153.6 (ImPy-$C_q$), 132.3 (ImPy-$C_q$), 131.0 (Ar-*C*H), 129.1 (Ar-*C*H), 128.7 (Ar-*C*H), 128.6 (ImPy-$C_q$), 122.8 (Ar-$C_q$), 120.8 (ImPy-*C*H), 119.9 (ImPy-*C*H), 111.7 (ImPy-$C_q$), 110.3 (ImPy-*C*H), 102.7 (-C≡C-), 80.6 (-C≡C-), 58.5 (ImPy-O-$CH_3$), 16.9 (ImPy-$CH_3$); **IR** [$cm^{-1}$]: ṽ = 3056, 2931, 2842, 1703, 1624, 1540, 1474, 1385, 1350, 1304, 1278, 1241, 1178, 1111, 1056, 1024, 975, 905, 801, 756, 691, 661, 540; **HR-MS-ESI(+)** calc. $C_{17}H_{15}N_2O^+$ $[M+H]^+$ 263.1179; found 263.1177.

Synthesis of **4h**

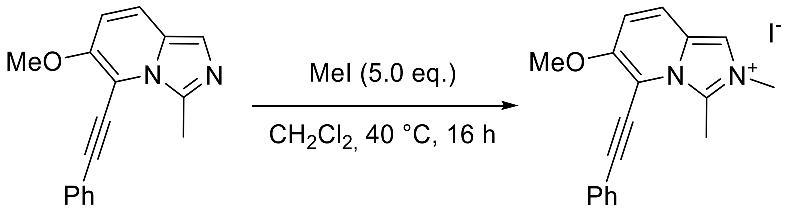


To a solution of **S36** (210 mg, 801 µmol, 1.0 eq.) in $CH_2Cl_2$ (10 mL) was added MeI (568 mg, 249 µL, 4.00 mmol, 5.0 eq.). The mixture was stirred for 16 h at 40 °C. Afterwards, $Et_2O$ (40 mL) was added while stirring vigorously. The solution was filtered off and the remaining solid was washed thrice with $Et_2O$ (3x20 mL). Drying under reduced pressure furnished **4h** (250 mg, 618 µmol, 77%) as a light-green solid.

**m.p.** 216 °C**;** $^{1}$**H NMR** (600 MHz, DMSO-$d_6$): δ [ppm] = 8.28 (s, 1H, ImPy-H), 7.99 (d, *J* = 10.1 Hz, 1H, ImPy-H), 7.64 – 7.61 (m, 2H, Ar-H), 7.54 – 7.50 (m, 3H, Ar-H), 7.50 (d, *J* = 10.1 Hz, 1H, ImPy-H), 4.05 (s, 3H, , ImPy-O-$CH_3$), 4.04 (s, 3H, ImPy-N-$CH_3$), 3.23 (s, 3H, ImPy-$CH_3$); $^{13}$**C NMR** (151 MHz, DMSO-$d_6$): δ [ppm] = 155.6 (ImPy-$C_q$), 136.0 (ImPy-$C_q$), 130.8 (Ar-*C*H), 130.0 (Ar-*C*H), 129.1 (Ar-*C*H), 126.4 (ImPy-$C_q$), 121.0 (Ar-$C_q$), 120.5 (ImPy-*C*H), 115.9 (ImPy-*C*H), 115.5 (ImPy-*C*H), 104.8 (ImPy-$C_q$), 104.6 (-C≡C-), 78.6 (-C≡C-), 58.0 (ImPy-O-$CH_3$), 36.1 (ImPy-N-$CH_3$), 12.1 (ImPy-$CH_3$); **IR** [$cm^{-1}$]: ṽ = 3079, 1568, 1465, 1438, 1343, 1275, 1147, 1115, 1073, 1039, 928, 797, 762, 696, 622, 525, 493, 435; **HR-MS-ESI(+)** calc. $C_{18}H_{17}N_2O^+$ $[M]^+$ 277.1335; found 277.1333.

Synthesis of **S37**

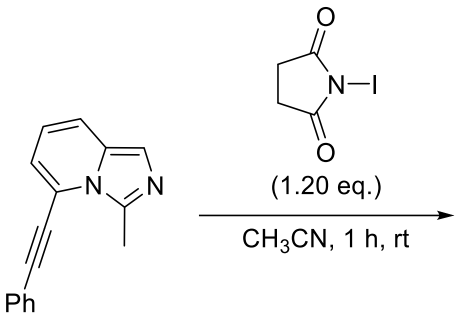


To a solution of **S21** (600 mg, 2.58 mmol, 1.0 eq.) in $CH_3CN$ (20 mL) under argon was added *N*-iodosuccinimide (697 mg, 3.10 mmol, 1.2 eq.), resulting in a solid crashing out of the solution. After stirring the suspension for 1 h, TLC analysis indicated complete conversion and the solvent was removed under reduced pressure. The resulting solid was dissolved in $CH_2Cl_2$ (80 mL) and saturated aqueous $Na_2S_2O_3$ solution was added. The aqueous phase was extracted thrice (3x100 mL) and the combined organic extracts were washed with aqueous NaOH solution (1M, 100 mL) to remove residual succinimide. After drying with $Na_2SO_4$, the solvent was removed under reduced pressure and the residual solid was purified by flash column chromatography (silica, 15% EtOAc in cyclohexane) to yield **S37** (885 mg, 2.47 mmol, 96%) as an orange solid.

**m.p.** 158 °C (decomp.) **;** $^{1}$**H NMR** (700 MHz, $CDCl_3$): δ [ppm] = 7.55 – 7.50 (m, 2H, Ar-H), 7.44 – 7.37 (m, 3H, Ar-H), 7.24 (dd, *J* = 9.1, 1.2 Hz, 1H, ImPy-H), 6.85 (dd, *J* = 6.7, 1.2 Hz, 1H, ImPy-H), 6.64 (dd, *J* = 9.1, 6.7 Hz, 1H, ImPy-H), 3.14 (s, 3H, ImPy-$CH_3$); $^{13}$**C NMR** (176 MHz, $CD_3CN$) : δ [ppm] = 140.3 (ImPy-$C_q$), 133.4 (ImPy-$C_q$), 131.2 (Ar-*C*H), 129.7 (Ar-*C*H), 128.8 (Ar-*C*H), 121.7 (Ar-$C_q$), 121.3 (ImPy-*C*H), 119.7 (ImPy-*C*H), 119.0 (ImPy-*C*H), 118.4 (ImPy-$C_q$), 97.5 (-C≡C-), 83.1 (-C≡C-), 73.3 (ImPy-*C*I), 16.9 (ImPy-$CH_3$); **IR** [$cm^{-1}$]: ṽ = 2922, 1615, 1568, 1506, 1479, 1439, 1400, 1367, 1338, 1314, 1291, 1220, 1089, 1062, 1042, 1024, 997, 918, 877, 776, 758, 706, 689, 652, 635, 565, 531, 513, 438; **HR-MS-ESI(+)** calc. $C_{16}H_{12}IN_2^+$ $[M+H]^+$ 359.0040; found 359.0038.

Synthesis of **S38**

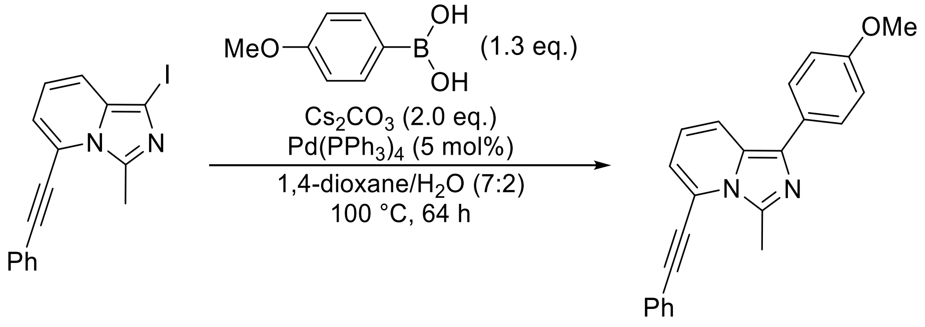

**S37** (420 mg, 1.17 mmol, 1.0 eq., 0.3M in 1,4-dioxane), 4-methoxyphenylboronic acid (232 mg, 1.52 mmol, 1.3 eq.) and $Cs_2CO_3$ (764 mg, 2.35 mmol, 2.0 eq., 2M in $H_2O$) were dissolved in a mixture of 1,4-dioxane and water (7:2, 5 mL). After degassing the mixture, $Pd(PPh_3)_4$ (68 mg, 58.6 µmol, 5 mol%) was added at once. The reaction mixture was heated to 100 °C for 64 h. The mixture was cooled to rt, then a small amount of $Na_2SO_4$ was added and the suspension was filtered over a frit loaded with $Na_2SO_4$. The frit was washed several times with EtOAc, the solvent was removed under reduced pressure and the resulting red solid was purified by flash column chromatography (silica, 20% EtOAc in cyclohexane). **S38** (221 mg, 653 µmol, 56%) was obtained as a red solid.

**m.p.** 175 °C**;** $^{1}$**H NMR** (500 MHz, $CDCl_3$): δ [ppm] = 7.79 – 7.71 (m, 2H, Ar-H), 7.67 (dd, *J* = 9.2, 1.1 Hz, 1H, ImPy-H), 7.59 – 7.51 (m, 2H, Ar-H), 7.45 – 7.36 (m, 3H, Ar-H), 7.03 – 6.96 (m, 2H, Ar-H), 6.83 (dd, *J* = 6.6, 1.1 Hz, 1H, ImPy-H), 6.58 (dd, *J* = 9.2, 6.6 Hz, 1H, ImPy-H), 3.85 (s, 3H, Ar-O-$CH_3$), 3.20 (s, 3H, ImPy-$CH_3$); $^{13}$**C NMR** (126 MHz, $CDCl_3$): 158.6 (Ar-$C_q$), 137.7 (ImPy-$C_q$), 131.5 (ImPy-$C_q$), 131.2 (Ar-*C*H), 129.4 (Ar-*C*H), 128.8 (Ar-*C*H), 128.2 (Ar-*C*H), 127.7 (Ar-$C_q$), 127.3 (ImPy-$C_q$), 122.1 (Ar-$C_q$), 121.1 (ImPy-*C*H), 120.1 (ImPy-*C*H), 117.9 (ImPy-$C_q$), 117.8 (ImPy-*C*H), 114.3 (Ar-*C*H), 96.8 (-C≡C-), 84.0 (-C≡C-), 55.5 (Ar-O-$CH_3$), 16.9 (ImPy-$CH_3$); **IR** [$cm^{-1}$]: ṽ = 3048, 2982, 2971, 2923, 2832, 1610, 1537, 1512, 1500, 1483, 1440, 1391, 1352, 1292, 1275, 1241, 1174, 1158, 1107, 1034, 1020, 1003, 886, 832, 814, 771, 755, 741, 730, 712, 687, 657, 576, 533, 521, 510; **HR-MS-ESI(+)** calc. $C_{23}H_{18}N_2O^+$ $[M+H]^+$ 339.1492; found 339.1481.

Synthesis of **4i**

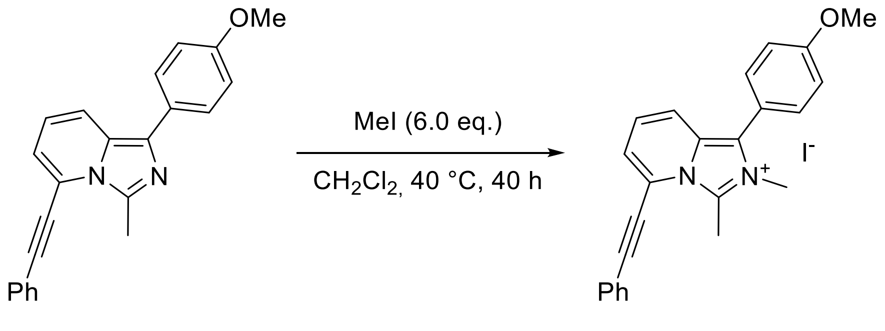


To a solution of **S38** (200 mg, 591 µmol, 1.0 eq.) in $CH_2Cl_2$ (15 mL) was added MeI (503 mg, 221 µL, 3.55 mmol, 6.0 eq.). The mixture was stirred for 40 h at 40 °C. Afterwards, $Et_2O$ (40 mL) was added while stirring vigorously. The solution was filtered off and the remaining solid was washed thrice with $Et_2O$ (3x20 mL). Drying under reduced pressure furnished **4i** as a brown-yellow solid (170 mg, 591 µmol, 60%).

**m.p.** 255 °C**;** $^{1}$**H NMR** (600 MHz, $CD_3CN$): δ [ppm] = 7.71 – 7.68 (m, 2H, Ar-H), 7.58 – 7.45 (m, 6H, 5 Ar-H and 1 ImPy-H), 7.39 (dd, *J* = 7.0, 1.1 Hz, 1H, ImPy-H), 7.21 – 7.16 (m, 2H, Ar-H), 7.09 (dd, *J* = 9.3, 7.0 Hz, 1H, ImPy-H), 3.90 (s, 3H, N-$CH_3$), 3.85 (s, 3H, Ar-O-$CH_3$), 3.39 (s,

3H, ImPy-$CH_3$); **$^{13}$C NMR** (151 MHz, $CD_3CN$): δ [ppm] = 162.0 (Ar-$C_q$), 136.8 (ImPy-$C_q$), 132.9 (Ar-*C*H), 132.0 (Ar-*C*H), 131.0 (Ar-*C*H), 129.6 (Ar-*C*H), 128.2 (ImPy-$C_q$), 126.7 (ImPy-$C_q$), 126.2 (ImPy-*C*H), 123.9 (ImPy-*C*H), 121.2 (Ar-$C_q$), 119.5 (ImPy-*C*H), 118.6 (ImPy-$C_q$), 117.2 (Ar-$C_q$), 115.5 (Ar-*C*H), 100.5 (-C≡C-), 81.7 (-C≡C-), 55.9 (N-$CH_3$), 34.3 (Ar-O-$CH_3$), 13.1 (ImPy-$CH_3$); **IR** [$cm^{-1}$]: ṽ = 3012, 1610, 1575, 1511, 1484, 1438, 1342, 1292, 1245, 1178, 1143, 1114, 1017, 908, 854, 832, 789, 765, 725, 692, 665, 637, 577, 529, 445; **HR-MS-ESI(+)** calc. $C_{23}H_{18}N_2O^+$ $[M]^+$ 353.1648; found 353.1650.

Synthesis of **S39**

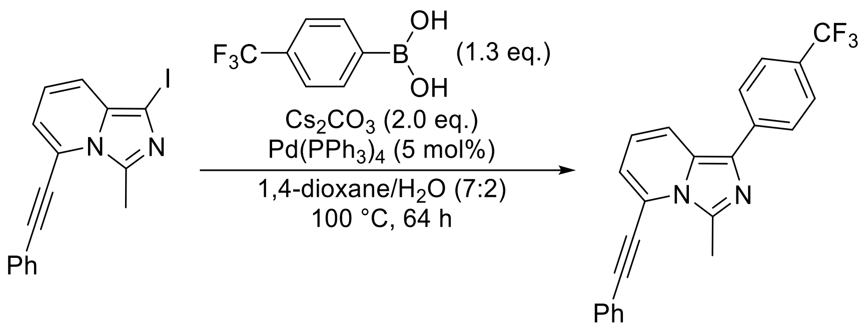


**S37** (800 mg, 2.23 mmol, 1.0 eq., 0.3M in 1,4-dioxane), 4-trifluoromethylphenylboronic acid (551 mg, 2.90 mmol, 1.3 eq.) and $Cs_2CO_3$ (1.46 g, 4.47 mmol, 2.0 eq., 2M in $H_2O$) were dissolved in a mixture of 1,4-dioxane and water (7:2, 10 mL). After degassing the mixture, $Pd(PPh_3)_4$ (129 mg, 112 µmol, 5 mol%) was added at once. The reaction mixture was heated to 100 °C for 64 h. The mixture was cooled to rt, then a small amount of $Na_2SO_4$ was added and the suspension was filtered over a frit loaded with $Na_2SO_4$. The frit was washed several times with EtOAc, the solvent was removed under reduced pressure and the resulting red solid was purified by flash column chromatography (silica, 20% EtOAc in cyclohexane). **S39** (450 mg, 1.20 mmol, 54%) was obtained as a red solid.

**m.p.** 191 °C**; $^1$H NMR** (600 MHz, $CDCl_3$): δ [ppm]: 7.80 – 7.75 (m, 2H, Ar-H), 7.57 (dd, *J* = 9.2, 1.1 Hz, 1H, ImPy-H), 7.51 (d, *J* = 8.0 Hz, 2H, Ar-H), 7.43 – 7.36 (m, 2H, Ar-H), 7.29 – 7.21 (m, 3H, Ar-H), 6.73 (dd, *J* = 6.7, 1.1 Hz, 1H, ImPy-H), 6.56 (dd, *J* = 9.2, 6.7 Hz, 1H, ImPy-H), 3.04 (s, 3H, ImPy-$CH_3$); **$^{13}$C NMR** (151 MHz, $CDCl_3$): δ [ppm] = 138.7 (Ar-$C_q$), 138.5 (ImPy-$C_q$), 131.3 (Ar-*C*H), 129.7 (ImPy-$C_q$), 129.7 (Ar-*C*H), 128.9 (Ar-*C*H), 128.8 (ImPy-$C_q$), 128.2 (q, *J* = 32.4 Hz, Ar-$C_q$), 126.7 (Ar-*C*H), 125.8 (q, *J* = 3.8 Hz, Ar-*C*H), 124.6 (q, *J* = 271.9 Hz, Ar-$CF_3$), 121.8 (Ar-$C_q$), 121.2 (ImPy-*C*H), 119.6 (ImPy-*C*H), 119.4 (ImPy-*C*H), 118.5 (ImPy-$C_q$), 97.5 (-C≡C-), 83.6 (-C≡C-), 17.0 (ImPy-$CH_3$); **$^{19}$F NMR** (565 MHz, $CDCl_3$): δ [ppm] = -62.2 (-C$F_3$); **IR** [$cm^{-1}$]: ṽ = 2923, 1611, 1412, 1320, 1289, 1162, 1108, 1063, 1013, 1003, 885, 864, 844, 776, 754, 717, 687, 646, 617, 532, 517, 412; **HR-MS-ESI(+)** calc. $C_{23}H_{16}F_3N_2^+$ $[M+H]^+$ 377.1260; found 377.1267.

Synthesis of **4j**

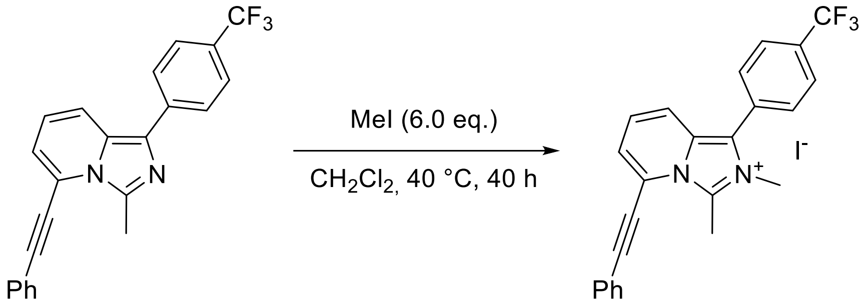


To a solution of **S39** (300 mg, 797 µmol, 1.0 eq.) in $CH_2Cl_2$ (10 mL) was added MeI (679 mg, 298 µL, 4.78 mmol, 6.0 eq.). The mixture was stirred for 40 h at 40 °C. Afterwards, $Et_2O$ (40 mL) was added while stirring vigorously. The solution was filtered off and the remaining solid was washed thrice with $Et_2O$ (3x20 mL). Drying under reduced pressure furnished **4j** as a yellow solid (230 mg, 444 µmol, 56%).

**m.p.** 241 °C**;** $^{1}$**H NMR** (500 MHz, DMSO-$d_6$): δ [ppm] = 8.06 (d, *J* = 7.9 Hz, 2H, Ar-H), 7.90 (d, *J* = 8.0 Hz, 2H, Ar-H), 7.77 – 7.70 (m, 3H, ImPy-H and Ar-H), 7.61 (d, *J* = 7.0 Hz, 1H, ImPy-H), 7.59 – 7.52 (m, 3H, Ar-H), 7.26 (dd, *J* = 9.3, 7.0 Hz, 1H, ImPy-H), 3.98 (s, 3H, ImPy-N-$CH_3$), 3.43 (s, 3H, ImPy-$CH_3$); $^{13}$**C NMR** (126 MHz, DMSO-$d_6$): δ [ppm] = 137.8 (ImPy-$C_q$), 131.6 (Ar-*C*H), 131.3 (Ar-*C*H), 130.6 (Ar-*C*H), 130.3 (q, *J* = 32.1 Hz, Ar-$C_q$), 129.2 (Ar-*C*H), 129.0 (Ar-$C_q$), 127.5 (ImPy-$C_q$), 126.4 (q, *J* = 3.8 Hz, Ar-*C*H), 125.6 (ImPy-*C*H), 124.5 (ImPy-*C*H), 123.9 (q, *J* = 272.6 Hz, Ar-$CF_3$), 123.6 (ImPy-$C_q$), 120.1 (Ar-$C_q$), 118.7 (ImPy-*C*H), 117.7 (ImPy-$C_q$), 99.8 (-C≡C-), 81.6 (-C≡C-), 34.3 (ImPy-N-$CH_3$), 12.9 (ImPy-$CH_3$); $^{19}$**F NMR** (470 MHz, DMSO-$d_6$): δ [ppm] = -61.4 (-$CF_3$);**IR** [$cm^{-1}$]: ṽ = 3027, 2982, 1618, 1534, 1487, 1439, 1330, 1167, 1118, 1073, 1061, 1016, 909, 863, 847, 836, 772, 758, 732, 717, 695, 669, 638, 596, 575, 535, 523, 463, 416; **HR-MS-ESI(+)** calc. $C_{24}H_{18}F_3N_2^+$ $[M]^+$ 391.1417; found 391.1422.

Synthesis of **4k**

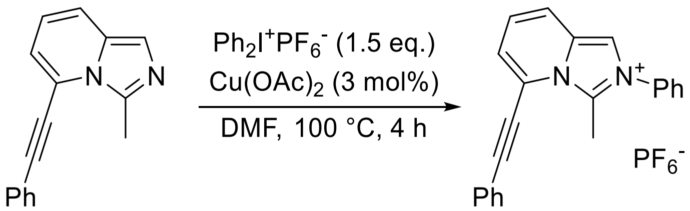


To a solution of **S21** (300 mg, 1.29 mmol, 1.0 eq.) and $Cu(OAc)_2$ (7 mg, 38.7 µmol, 3 mol%) in dry DMF (6 mL) was added $Ph_2I^+PF_6^-$ (825 mg, 1.94 mmol, 1.5 eq.) at once. The reaction mixture was heated to 100 °C and stirred for 4 h. After cooling the mixture to rt, the solvent was removed under reduced pressure. The remaining brown oil was suspended in aqueous ammonia (10 mL) to remove remaining copper salts and was subsequently extracted with $CH_2Cl_2$ (3x50 mL). The combined organic extracts were dried over $Na_2SO_4$ and the solvent

was reduced to a small volume (~5 mL) under reduced pressure. It was then added dropwise to a vigorously stirring $Et_2O$ (50 mL) containing flask, yielding a grey precipitate. After repeating the precipitation thrice, the precipitate was dried under reduced pressure to yield **4k** (500 mg, 1.10 mmol, 85%) as a light-grey solid.

**m.p.** 195 °C**;** $^{1}$**H NMR** (600 MHz, DMSO-$d_6$): δ [ppm] = 8.56 (s, 1H, ImPy-H), 7.94 (d, *J* = 9.7 Hz, 1H, ImPy-H), 7.77 – 7.73 (m, 3H, Ar-H), 7.71 – 7.67 (m, 4H, Ar-H), 7.63 (d, *J* = 7.0 Hz, 1H, ImPy-H), 7.59 – 7.50 (m, 3H, Ar-H), 7.31 (dd, *J* = 9.3, 7.0 Hz, 1H, ImPy-H), 3.15 (s, 3H, ImPy-$CH_3$); $^{13}$**C NMR** (151 MHz, DMSO-$d_6$): δ [ppm] = 137.5 (ImPy-$C_q$), 134.7 (Ar-$C_q$), 131.3 (Ar-*C*H), 131.1 (Ar-*C*H), 130.6 (Ar-*C*H), 130.1 (Ar-*C*H), 129.5 (ImPy-$C_q$), 129.2 (Ar-*C*H), 126.5 (Ar-*C*H), 125.3 (ImPy-*C*H), 124.1 (ImPy-*C*H), 120.1 (Ar-$C_q$), 119.7 (ImPy-*C*H), 117.9 (ImPy-$C_q$), 115.4 (ImPy-*C*H), 99.4 (-C≡C-), 81.5 (-C≡C-), 13.6 (ImPy-$CH_3$); **IR** [$cm^{-1}$]: ṽ = 3157, 1649, 1596, 1528, 1513, 1493, 1442, 1409, 1302, 1194, 1033, 926, 879, 831, 789, 774, 759, 733, 710, 691, 664, 611, 590, 578, 557, 529, 498, 446; **HR-MS-ESI(+)** calc. $C_{22}H_{17}N_2^+$ $[M]^+$ 309.1386; found 309.1386.

Synthesis of **4l**

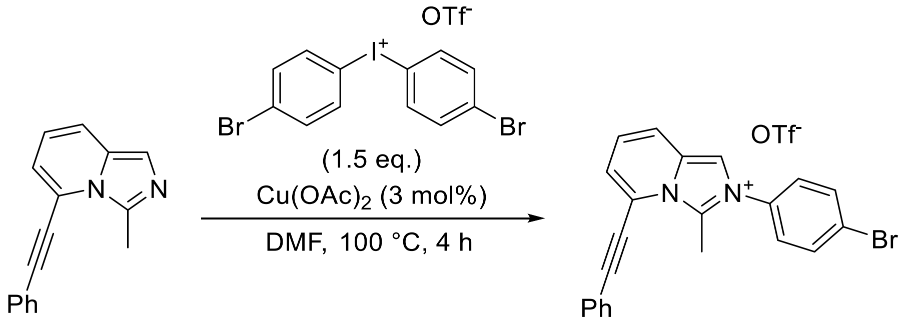


To a solution of **S21** (400 mg, 1.72 mmol, 1.0 eq.) and $Cu(OAc)_2$ (10 mg, 51.7 µmol, 3 mol%) in dry DMF (8 mL) was added bis(4-bromophenyl)iodonium triflate (1.52 g, 2.58 mmol, 1.5 eq.) at once. The reaction mixture was heated to 100 °C and stirred for 4 h. After cooling the mixture to rt, the solvent was removed under reduced pressure. The remaining brown oil was suspended in aqueous ammonia (30 mL) to remove remaining copper salts and was subsequently extracted with $CH_2Cl_2$ (3x50 mL). The combined organic extracts were dried over $Na_2SO_4$ and the solvent was reduced to minimum volume (~5 mL) under reduced pressure. It was then added dropwise to a vigorously stirring $Et_2O$ (50 mL) containing flask, yielding a brown precipitate. After repeating the precipitation twice, the precipitate was dried under reduced pressure to yield **4l** (555 mg, 1.03 mmol, 60%) as a light-brown solid.

**m.p.** 232 °C**;** $^{1}$**H NMR** (600 MHz, DMSO-$d_6$): δ [ppm] = 8.56 (s, 1H, ImPy-H), 7.98 (m, 2H, Ar-H), 7.95 (d, *J* = 9.3 Hz, 1H, ImPy-H), 7.72 – 7.67 (m, 2H, Ar-H), 7.69 – 7.63 (m, 2H, Ar-H), 7.63 (d, *J* = 7.0 Hz, 1H, ImPy-H), 7.59 – 7.50 (m, 3H, Ar-H), 7.31 (dd, *J* = 9.3, 7.0 Hz, 1H, ImPy-H),

3.15 (s, 3H, ImPy-$CH_3$); **$^{13}$C NMR** (151 MHz, DMSO-$d_6$): δ [ppm] = 137.6 (ImPy-$C_q$), 133.9 (Ar-$C_q$), 133.1 (Ar-*C*H), 131.3 (Ar-*C*H), 130.6 (Ar-*C*H), 129.5 (ImPy-$C_q$), 129.1 (Ar-*C*H), 128.7 (Ar-*C*H), 125.4 (ImPy-*C*H), 124.6 (Ar-$C_q$), 124.1 (ImPy-*C*H), 120.7 (q, *J* = 322.3 Hz, $CF_3SO_3^-$), 120.1 (Ar-$C_q$), 119.7 (ImPy-*C*H), 117.8 (ImPy-$C_q$), 115.4 (ImPy-*C*H), 99.5 (-C≡C-), 81.4 (-C≡C-), 13.6 (ImPy-$CH_3$); **IR** [$cm^{-1}$]: ṽ = 3092, 1502, 1488, 1404, 1276, 1258, 1223, 1204, 1147, 1069, 1030, 1015, 843, 806, 764, 741, 715, 689, 665, 636, 620, 573, 548, 530, 516, 502; **HR-MS-ESI(+)** calc. $C_{22}H_{16}BrN_2^+$ $[M]^+$ 387.0491; found 387.0491.

Synthesis of **S41**

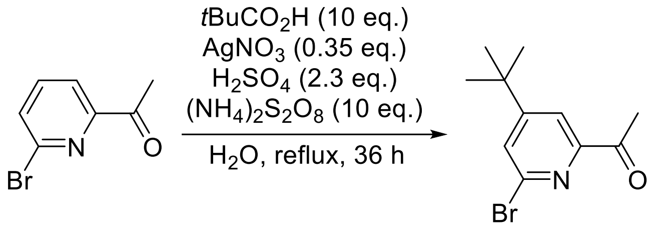


To a suspension of 2-acetyl-6-bromopyridine (**S40**, 5.00 g, 25.0 mmol, 1.0 eq.) in $H_2O$ (250 mL), pivalic acid (25.5 g, 250 mmol, 10 eq.), silver(I)nitrate (1.49 g, 8.75 mmol, 0.35 eq.) and sulfuric acid (3.08 mL, 57.5 mmol, 2.3 eq.) was added $(NH_4)_2S_2O_8$ (57.0 g, 250 mmol, 10 eq.) to give a grey suspension which was heated to reflux for 36 h. The suspension was then allowed to cool down to rt and was then neutralized with saturated aqueous sodium bicarbonate solution. Afterwards the aqueous phase was extracted four times with EtOAc (4x150 mL), the combined organic extracts were washed with deionized water (100 mL) and dried over $Na_2SO_4$. Silica gel (ca. 20 mL) was added and the solvent was removed under reduced pressure. The residue was purified by flash column chromatography (silica, cyclohexane) to furnish **S41** (3.35 g, 13.1 mmol, 52%) as a pale-yellow oil. The spectroscopic data agrees with the literature.[8]

**$^1$H NMR** (500 MHz, $CDCl_3$): δ [ppm] = 7.99 (d, *J* = 1.6 Hz, 1H, Py-H), 7.61 (d, *J* = 1.7 Hz, 1H, Py-H), 2.69 (s, 3H, Py-CO-$CH_3$), 1.33 (s, 9H, $C(CH_3)_3$); **$^{13}$C NMR** (126 MHz, $CDCl_3$): δ [ppm] = 199.2, 164.5, 154.3, 141.9, 128.8, 118.2, 35.5, 30.5, 26.1.

Synthesis of **S42**

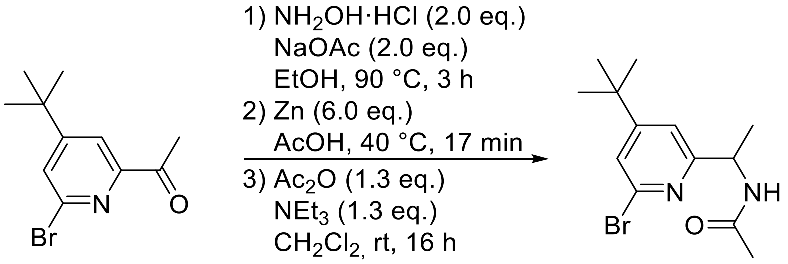

To a solution of **S41** (3.00 g, 11.7 mmol, 1.0 eq.) in EtOH (50 mL) at rt was added hydroxylamine hydrochloride (1.92 g, 23.4 mmol, 2.0 eq.) and sodium acetate (1.63 g, 23.4 mmol, 2.0 eq.). Upon addition the colorless solution was stirred at 90 °C for 3 hours. The solution was then cooled to rt and the solvent was removed under reduced pressure. The resulting colorless solid was dissolved in EtOAc (150 mL) and washed with water (100 mL). The aqueous phase was washed four times with EtOAc (4x150 mL). Afterwards the organic phase was dried with $Na_2SO_4$, filtrated and the solvent was removed under reduced pressure to yield the ketoxime, which was then dissolved in acetic acid (100 mL) in a 500 mL round-bottom flask. Then, activated zinc dust (4.59 g, 70.3 mmol, 6.0 eq.) was poured in the flask in one portion. The mixture was first placed in an ultrasonic bath at rt for 5 min and then it was stirred for 12 min at 40 °C and 1000 rpm. Afterwards the mixture was filtered over Celite and the filtrate was concentrated under reduced pressure. The orange oil was then dissolved in $CH_2Cl_2$ (50 mL) and mixed with hydrochloric acid (20 mL) and then stirred for 15 minutes at 0 °C. Afterwards, water (80 mL) and then aqueous NaOH (80 mL, 20w%) were added and the mixture was extracted four times with $CH_2Cl_2$ (4x100 mL). Then, the organic phase was dried with $Na_2SO_4$, filtrated and the solvent was removed under reduced pressure to yield the primary amine, which was then dissolved in dry $CH_2Cl_2$ (40 mL). At 0 °C, $NEt_3$ (2.12 mL, 15.2 mmol, 1.3 eq.) and $Ac_2O$ (1.44 mL, 15.2 mmol, 1.3 eq.) were added and the solution was stirred for 16 h. To this solution saturated $NaHCO_3$ solution (100 mL) was added and the mixture was extracted thrice with $CH_2Cl_2$ (3x100 mL). The combined organic phases were dried over $Na_2SO_4$ and the solvent was removed under reduced pressure. Purification by flash column chromatography (silica, 10% EtOAc in cyclohexane to 100% EtOAc) furnished **S42** (1.20 g, 4.01 mmol, 34% over 3 steps) as a light-yellow oil.

**m.p.** >25 °C**;** $^{1}$**H NMR** (500 MHz, $CDCl_3$): δ [ppm] = 7.34 (d, *J* = 1.5 Hz, 1H, Py-H), 7.14 (d, *J* = 1.5 Hz, 1H, Py-H), 6.60 (br. d, *J* = 7.8 Hz, 1H, C(=O)NH), 5.10 (dq, *J* = 8.1, 6.8 Hz, 1H, Py-C*H*$CH_3$), 2.01 (s, 3H, C(=O)$CH_3$), 1.43 (d, *J* = 6.8 Hz, 3H, Py-CHC$H_3$), 1.28 (s, 9H, Py-C($CH_3$)$_3$); $^{13}$**C NMR** (126 MHz, $CDCl_3$): δ [ppm] = 169.4 (*C*(=O)NH), 164.5 (Py-$C_q$), 162.4 (Py-$C_q$), 142.3 (Py-$C_q$), 124.1 (Py-*C*H), 118.0 (Py-*C*H), 49.8 (Py-*C*H$CH_3$), 35.3 (-*C*($CH_3$)$_3$), 30.6 (-C(*C*$H_3$)$_3$), 23.6 (C(=O)*C*$H_3$), 22.9 (Py-CH*C*$H_3$); **IR** [$cm^{-1}$]: ṽ = 3276, 2967, 2870, 1650, 1592, 1532, 1478, 1447, 1394, 1370, 1304, 1279, 1165, 1041, 989, 965, 868, 828, 763, 598, 521; **HR-MS-ESI(+)** calc. $C_{13}H_{20}BrN_2O^+$ $[M+H]^+$ 299.0754; found 299.0757.

Synthesis of **S43**

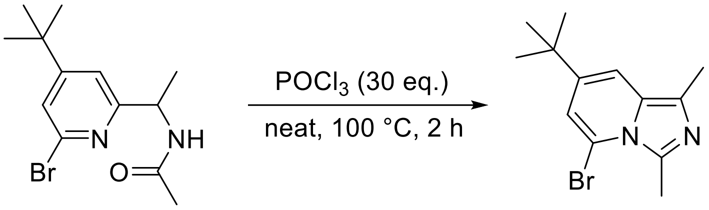

Under an atmosphere of argon, **S42** (1.06 g, 3.54 mmol, 1.0 eq.) was dissolved in $POCl_3$ (9.94 mL, 106 mmol, 30.0 eq.) and heated to 100 °C. After stirring the suspension for 2 h, the reaction was allowed to cool down to rt and was poured onto ice. The pH was adjusted to 14 with aqueous NaOH solution (20 w%), causing a solid to crash out. The suspension was filtrated and washed thoroughly with deionized water and $CH_2Cl_2$ until decolorization of the precipitate and the resulting mixture was extracted four times (4x100 mL) with $CH_2Cl_2$. The combined organic extracts were dried over $Na_2SO_4$ and the solvent was removed under reduced pressure to yield **S43** (880 mg, 3.13 mmol, 88%) as a light-brown solid.

**m.p.** 98 °C**;** $^{1}$**H NMR** (600 MHz, $CDCl_3$): δ [ppm] = 7.02 (d, *J* = 1.7 Hz, 1H, ImPy-H), 6.68 (d, *J* = 1.7 Hz, 1H, ImPy-H), 3.00 (s, 3H, ImPy-$CH_3$), 2.41 (s, 3H, ImPy-$CH_3$), 1.26 (s, 9H, ImPy-$C(CH_3)_3$); $^{13}$**C NMR** (151 MHz, $CDCl_3$): δ [ppm] = 140.0 (ImPy-$C_q$), 135.4 (ImPy-$C_q$), 129.6 (ImPy-$C_q$), 126.4 (ImPy-$C_q$), 117.9 (ImPy-*C*H), 111.2 (ImPy-*C*H), 111.0 (ImPy-$C_q$), 34.2 (ImPy-$C(CH_3)_3$), 30.2 (ImPy-$C(CH_3)_3$), 18.4 (ImPy-$CH_3$), 12.6 (ImPy-$CH_3$); **IR** [$cm^{-1}$]: ṽ = 2960, 2869, 1630, 1513, 1440, 1409, 1394, 1364, 1333, 1287, 1255, 1212, 1202, 1095, 1044, 1029, 998, 893, 866, 844, 836, 789, 706, 637, 626, 535; **HR-MS-ESI(+)** calc. $C_{13}H_{18}BrN_2^+$ $[M+H]^+$ 281.0648; found 281.0648.

Synthesis of **S44**

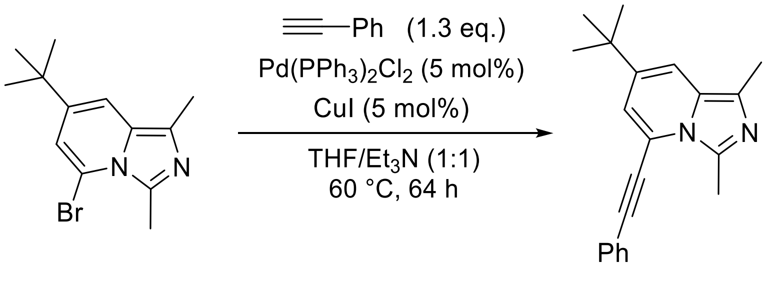

To a degassed solution of dry THF and $NEt_3$ (26 mL, 1:1) under argon was added $Pd(PPh_3)_2Cl_2$ (79 mg, 113 µmol, 5 mol%) and the resulting suspension was stirred for 15 min. Afterwards, **S43** (631 mg, 2.24 mmol, 1.0 eq.), phenylacetylene (298 mg, 320 µL, 2.92 mmol, 1.3 eq.) and CuI (21.4 mg, 113 µmol, 5 mol%) were added, the reaction mixture was heated to 60 °C and was stirred for 64 h. The mixture was then cooled to rt, the solvent was removed under reduced

pressure and the crude product was purified by flash column chromatography (silica, 25% to 50% EtOAc in cyclohexane) to yield **S44** (650 mg, 2.15 mmol, 96%) as a red-orange solid.

**m.p.** 124 °C**;** **$^{1}$H NMR** (600 MHz, $CDCl_3$): δ [ppm] = 7.57 – 7.53 (m, 2H, Ar-H), 7.41 – 7.37 (m, 3H, Ar-H), 7.12 (d, *J* = 1.9 Hz, 1H, ImPy-H), 6.85 (d, *J* = 1.9 Hz, 1H, ImPy-H), 3.07 (s, 3H, ImPy-$CH_3$), 2.45 (s, 3H, ImPy-$CH_3$), 1.30 (s, 9H, ImPy-$C(CH_3)_3$); **$^{13}$C NMR** (151 MHz, $CDCl_3$): δ [ppm] = 138.8 (ImPy-$C_q$), 135.5 (ImPy-$C_q$), 131.1 (Ar-*C*H), 129.3 (Ar-*C*H), 128.8 (Ar-*C*H), 127.9 (ImPy-$C_q$), 126.9 (ImPy-$C_q$), 122.2 (Ar-$C_q$), 120.4 (ImPy-$C_q$), 117.1 (ImPy-*C*H), 113.1 (ImPy-*C*H), 95.7 (-C≡C-), 84.4 (-C≡C-), 34.2 (ImPy-C($C$H$_3$)$_3$), 30.4 (ImPy-*C*($CH_3$)$_3$), 16.5 (ImPy-*C*$H_3$), 12.6 (ImPy-*C*$H_3$); **IR** [cm$^{-1}$]: ṽ = 2962, 2909, 2865, 1698, 1628, 1591, 1477, 1440, 1412, 1391, 1361, 1342, 1329, 1251, 1204, 1103, 1069, 964, 896, 866, 849, 748, 706, 685, 636, 521, 422; **HR-MS-ESI(+)** calc. $C_{21}H_{23}N_2^+$ $[M+H]^+$ 303.1856; found 303.1856.

Synthesis of **4m**

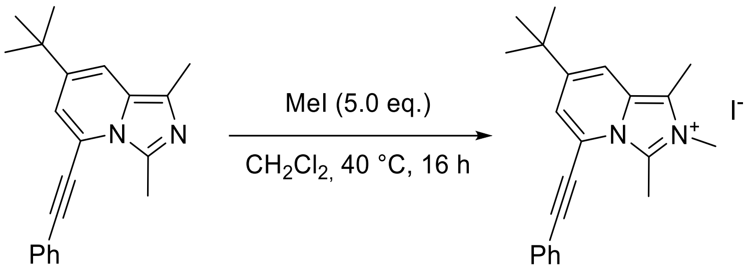


To a solution of **S44** (300 mg, 991 µmol, 1.0 eq.) in $CH_2Cl_2$ (10 mL) was added MeI (704 mg, 308 µL, 4.96 mmol, 5.0 eq.). The mixture was stirred for 16 h at 40 °C. Afterwards, $Et_2O$ (40 mL) was added while stirring vigorously. The solution was filtered off and the remaining solid was washed thrice with $Et_2O$ (3x20 mL). Drying under reduced pressure furnished **4m** (354 mg, 797 µmol, 80%) as a light-yellow solid.

**m.p.** 308 °C (decomp.)**;** **$^{1}$H NMR** (600 MHz, DMSO-$d_6$): δ [ppm] = 7.73 – 7.68 (m, 2H, Ar-H), 7.67 (d, *J* = 1.8 Hz, 1H, ImPy-H), 7.64 (d, *J* = 1.8 Hz, 1H, ImPy-H), 7.59 – 7.50 (m, 3H, Ar-H), 3.94 (s, 3H, ImPy-N-$CH_3$), 3.28 (s, 3H, ImPy-$CH_3$), 2.64 (s, 3H, ImPy-$CH_3$), 1.32 (s, 9H, ImPy-$C(CH_3)_3$); **$^{13}$C NMR** (151 MHz, DMSO-$d_6$): δ [ppm] = 144.2 (ImPy-$C_q$), 135.1 (ImPy-$C_q$), 131.2 (Ar-*C*H), 130.3 (Ar-*C*H), 129.1 (Ar-*C*H), 126.2 (ImPy-$C_q$), 124.8 (ImPy-*C*H), 121.8 (ImPy-$C_q$), 120.3 (Ar-$C_q$), 117.0 (ImPy-$C_q$), 112.9 (ImPy-*C*H), 98.6 (-C≡C-), 82.0 (-C≡C-), 34.6 (ImPy-*C*($CH_3$)$_3$), 32.7 (ImPy-N-*C*$H_3$), 29.5 (ImPy-C($C$H$_3$)$_3$), 12.2 (ImPy-*C*$H_3$), 8.3 (ImPy-*C*$H_3$); **IR** [cm$^{-1}$]: ṽ = 2960, 2867, 1651, 1488, 1441, 1395, 1365, 1328, 1260, 1183, 1154, 905, 826, 765, 691, 637, 571, 525, 420; **HR-MS-ESI(+)** calc. $C_{22}H_{25}N_2^+$ $[M]^+$ 317.2012; found 317.2016.

Synthesis of **S46**

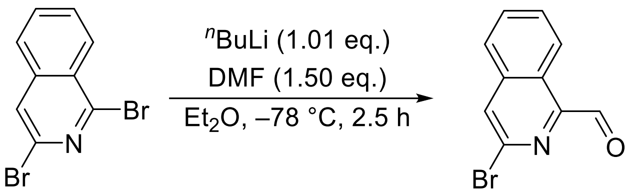


A solution of **S45** (2.00 g, 6.97 mmol, 1.0 eq.) in dry $Et_2O$ (60 mL) was cooled to –78 °C, to which was then added $^n$BuLi (4.40 mL, 7.04 mmol, 1.6 M in hexanes, 1.01 eq.) dropwise over 5 min. After full addition, the mixture was stirred for 30 min, then DMF (809 µL, 764 mg, 10.5 mmol, 1.5 eq.) was added dropwise and the mixture was stirred for another 2 hours. Aqueous HCl solution (1 M, 40 mL) was added to quench the reaction, which was then allowed to warm to rt. The biphasic mixture was then neutralized with saturated aqueous $NaHCO_3$ solution and extracted thrice with EtOAc (3x100 mL). The combined organic phases were then dried over $Na_2SO_4$, filtered off and the solvent was reduced under removed pressure. Purification by flash column chromatography ($SiO_2$, 10% EtOAc in cyclohexane) furnished **S46** (1.30 g, 5.51 mmol, 79%) as an off-white powder.

**m.p.** 132 °C**;** $^{1}$**H NMR** (500 MHz, $CDCl_3$): δ [ppm] = 10.30 (s, 1H, Qu-CHO), 9.29 – 9.24 (m, 1H, Qu-H), 8.12 (s, 1H, Qu-H), 7.83 (dd, *J* = 6.9, 3.0 Hz, 1H, Qu-H), 7.80 – 7.73 (m, 2H, Qu-H); $^{13}$**C NMR** (126 MHz, $CDCl_3$): δ [ppm] = 194.2 (Qu-*C*HO), 150.1 (Qu-$C_q$), 139.4 (Qu-$C_q$), 134.8 (Qu-$C_q$), 131.9 (Qu-*C*H), 130.7 (Qu-*C*H), 129.3 (Qu-*C*H), 126.2 (Qu-*C*H), 126.2 (Qu-*C*H), 125.3 (Qu-$C_q$); **IR** [cm$^{-1}$]: ṽ = 2862, 1707, 1544, 1484, 1263, 1217, 1175, 1151, 1095, 1049, 894, 883, 857, 840, 778, 751, 719, 692, 653, 585, 542, 494, 472; **HR-MS-ESI(+)** calc. $C_{10}H_7BrNO^+$ $[M+H]^+$ 235.9706; found 235.9706.

Synthesis of **S47**

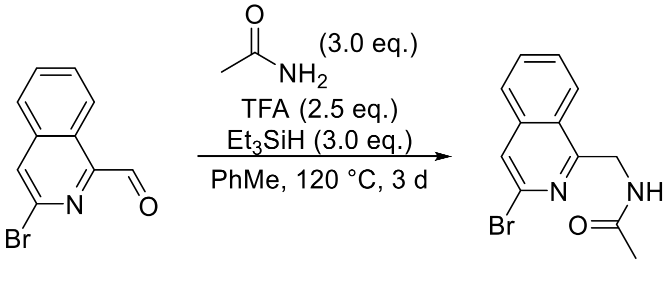


To a solution of **S46** (1.10 g, 4.66 mmol, 1.0 eq.) in dry toluene (30 mL) in a sealed vessel was added acetamide (826 mg, 14.0 mmol, 3.0 eq.), triethylsilane (2.23 mL, 1.63 g, 14.0 mmol, 3.0 eq.) and finally trifluoroacetic acid (891 µL, 1.33 g, 11.7 mmol, 2.5 eq.). The reaction mixture was heated to 120 °C and was stirred over 3 days. Afterwards, the reaction mixture was allowed to cool to rt and saturated aqueous $NaHCO_3$ solution (150 mL) was added. The mixture was extracted with $CH_2Cl_2$ thrice (3x100 mL), the organic extracts were dried over

$Na_2SO_4$ and filtered off. After removal of the solvent under reduced pressure, the crude product was purified by flash column chromatography (silica, 25% EtOAc in cyclohexane to 100% EtOAc) to obtain **S47** (144 mg, 1.24 mmol, 11%) as a brown solid.

**m.p.** 167 °C**;** $^{1}$**H NMR** (600 MHz, $CDCl_3$): δ [ppm] = 8.10 (dd, *J* = 8.5, 1.0 Hz, 1H, Qu-H), 7.82 (d, *J* = 1.0 Hz, 1H, Qu-H), 7.75 (dt, *J* = 8.3, 1.0 Hz, 1H, Qu-H), 7.72 (ddd, *J* = 8.2, 6.7, 1.1 Hz, 1H, Qu-H), 7.63 (ddd, *J* = 8.3, 6.7, 1.4 Hz, 1H, Qu-H), 7.18 (s, 1H, C(=O)NH), 5.04 (d, *J* = 4.4 Hz, 2H, Qu-$CH_2$-C(=O)), 2.15 (s, 3H, C(=O)$CH_3$); $^{13}$**C NMR** (151 MHz, $CDCl_3$): δ [ppm] = 170.4 (*C*(=O)NH), 156.3 (Qu-$C_q$), 138.2 (Qu-$C_q$), 134.0 (Qu-$C_q$), 131.6 (Qu-*C*H), 128.3 (Qu-*C*H), 126.6 (Qu-*C*H), 124.9 (Qu-$C_q$), 124.4 (Qu-*C*H), 123.7 (Qu-*C*H), 41.9 (Qu-$CH_2$-C(=O)), 23.5 (C(=O)$CH_3$); **IR** [$cm^{-1}$]: ṽ = 3231, 3063, 1627, 1554, 1500, 1400, 1370, 1292, 1198, 1143, 1092, 1024, 1006, 897, 886, 858, 839, 794, 779, 747, 728, 684, 630, 602, 538, 513, 501, 475, 451, 436, 424; **HR-MS-ESI(+)** calc. $C_{12}H_{12}BrN_2O^+$ $[M+H]^+$ 279.0128; found 279.0120.

Synthesis of **S48**

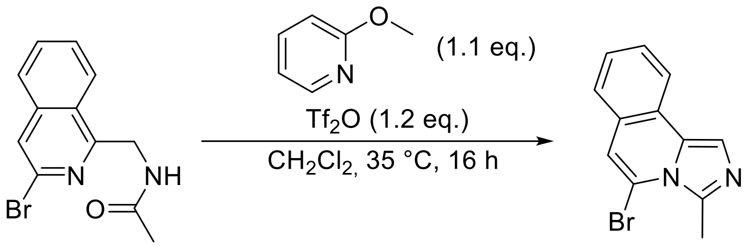


To a solution of **S47** (140 mg, 516 µmol, 1.0 eq.) and 2-methoxypyridine (60.0 µL, 62.0 mg, 567 µmol, 1.1 eq.) in dry $CH_2Cl_2$ (0.25 M, 2 mL) was added $Tf_2O$ (104 µL, 175 mg, 619 µmol, 1.2 eq.) at rt. Afterwards, the solution was heated to 35 °C and stirred for 16 h. The mixture was allowed to cool to rt and saturated aqueous $NaHCO_3$ solution (50 mL) was added, which was then extracted thrice ($CH_2Cl_2$, 3x50 mL), dried over $Na_2SO_4$ and filtered off. The solvent was removed under reduced pressure and purification by flash column chromatography (silica, 15% EtOAc in cyclohexane to 25% EtOAc) yielded **S48** (100 mg, 383 µmol, 74%) as a light-yellow oil.

**m.p.** >25 °C**;** $^{1}$**H NMR** (700 MHz, $CDCl_3$): δ [ppm] = 7.9 (ddd, *J* = 8.0, 1.3, 0.7 Hz, 1H. ImQu-H), 7.7 (s, 1H, ImQu-H), 7.5 (ddd, *J* = 8.1, 7.1, 1.3 Hz, 1H, ImQu-H), 7.4 (dd, *J* = 8.0, 1.4 Hz, 1H, ImQu-H), 7.4 (ddd, *J* = 8.1, 7.1, 1.2 Hz, 1H, ImQu-H), 7.0 (s, 1H, ImQu-H), 3.1 (s, 3H, ImQu-$CH_3$); $^{13}$**C NMR** (176 MHz, $CDCl_3$): δ [ppm] = 140.7 (ImQu-$C_q$), 131.5 (ImQu-$C_q$), 128.6 (ImQu-*C*H), 127.5 (ImQu-$C_q$), 127.1 (ImQu-*C*H), 125.9 (ImQu-*C*H), 124.5 (ImQu-$C_q$), 122.0 (ImQu-*C*H), 119.5 (ImQu-*C*H), 118.9 (ImQu-*C*H), 110.7 (ImQu-$C_q$-Br), 19.3 (ImQu-$CH_3$); **IR** [$cm^{-1}$]: ṽ = 3058, 2930, 2160, 1622, 1542, 1495, 1475, 1458, 1443, 1376, 1326, 1262, 1225, 1191, 1162, 1129, 988, 966, 834, 798, 752, 656, 453; **HR-MS-ESI(+)** calc. $C_{12}H_{10}BrN_2^+$ $[M+H]^+$ 261.0022; found 261.0018.

Synthesis of **S49**

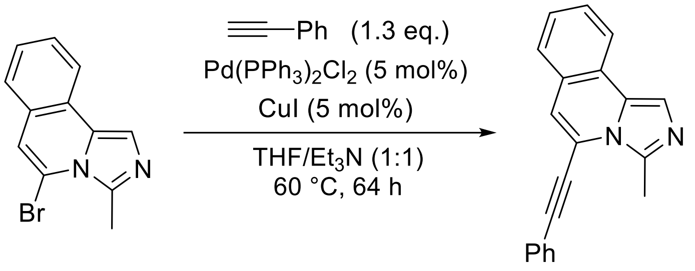


To a degassed solution of dry THF and $NEt_3$ (6 mL, 1:1) under argon was added $Pd(PPh_3)_2Cl_2$ (14 mg, 19.2 µmol, 5 mol%) and the resulting suspension was stirred for 15 min. Afterwards, **S48** (100 mg, 383 µmol, 1.0 eq.), phenylacetylene (55 µL, 498 µmol, 1.3 eq.) and CuI (4 mg, 19.2 µmol, 5 mol%) were added, the reaction mixture was heated to 60 °C and was stirred for 64 h. The mixture was then cooled to rt, the solvent was removed under reduced pressure and the crude product was purified by flash column chromatography (silica, 15% EtOAc in cyclohexane) to yield **S49** (102 mg, 361 µmol, 94%) as a brown oil.

**m.p.** <25 °C**;** $^{1}$**H NMR** (500 MHz, $CDCl_3$): 7.94 (dd, *J* = 7.9, 1.0 Hz, 1H, ImQu-H), 7.76 (br.s, 1H, ImQu-H), 7.61 – 7.52 (m, 2H, Ar-H), 7.52 – 7.44 (m, 2H, ImQu-H), 7.44 – 7.39 (m, 3H, Ar-H), 7.36 (td, *J* = 7.5, 1.2 Hz, 1H, ImQu-H), 7.09 (s, 1H, ImQu-H), 3.13 (s, 3H, ImQu-$CH_3$); $^{13}$**C NMR** (126 MHz, $CDCl_3$): δ [ppm] = 140.4 (ImQu-$C_q$), 132.2 (ImQu-$C_q$), 131.3 (Ar-*C*H), 129.6 (Ar-*C*H), 129.1 (ImQu-*C*H), 128.8 (Ar-*C*H), 126.9 (ImQu-*C*H), 126.8 (ImQu-*C*H), 126.7 (ImQu-$C_q$), 125.4 (ImQu-$C_q$), 122.1 (ImQu-*C*H), 122.0 (Ar-$C_q$), 121.4 (ImQu-*C*H), 119.4 (ImQu-*C*H), 117.2 (ImQu-$C_q$), 95.9 (-C≡C-), 83.7 (-C≡C-), 33.2 (ImQu-$CH_3$); **IR** [$cm^{-1}$]: ṽ = 3054, 2928, 2853, 2212, 1735, 1705, 1603, 1490, 1473, 1460, 1441, 1393, 1266, 1192, 1161, 1129, 1069, 1029, 985, 949, 876, 846, 806, 752, 719, 689, 660, 529, 484, 473; **HR-MS-ESI(+)** calc. $C_{20}H_{15}N_2^+$ $[M+H]^+$ 283.1230; found 283.1236.

Synthesis of **4n**

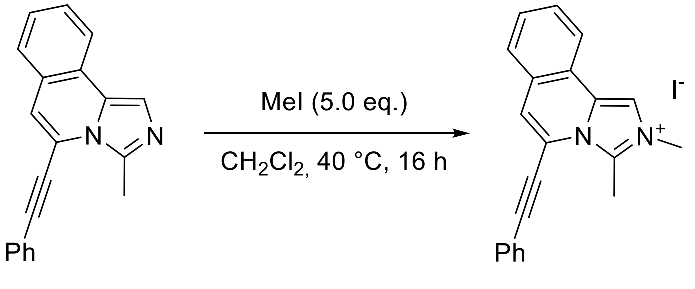


To a solution of **S49** (95 mg, 336 µmol, 1.0 eq.) in $CH_2Cl_2$ (5 mL) was added MeI (239 mg, 105 µL, 1.68 mmol, 5.0 eq.). The mixture was stirred for 16 h at 40 °C. Afterwards, $Et_2O$ (40 mL) was added while stirring vigorously. The solution was filtered off and the remaining

solid was washed thrice with $Et_2O$ (3x20 mL). Drying under reduced pressure furnished **4n** (105 mg, 247 µmol, 74%) as a light-yellow solid.

**m.p.** 212 °C**;** $^{1}$**H NMR** (700 MHz, DMSO-d$_6$): δ [ppm] = 8.83 (s, 1H, ImQu-H), 8.24 (dd, *J* = 7.9, 0.6 Hz, 1H, ImQu-H), 7.95 (s, 1H, ImQu-H), 7.90 (dd, *J* = 7.9, 1.1 Hz, 1H, ImQu-H), 7.79 – 7.76 (m, 1H, ImQu-H), 7.73 – 7.68 (m, 3H, ImQu-H and Ar-H), 7.60 – 7.52 (m, 3H, Ar-H), 4.08 (s, 3H, ImQu-N-$CH_3$), 3.28 (s, 3H, ImQu-$CH_3$); $^{13}$**C NMR** (176 MHz, DMSO-d$_6$): δ [ppm] = 139.7 (ImQu-$C_q$), 131.3 (ImQu-*C*H), 130.9 (Ar-*C*H), 130.5 (ImQu-*C*H), 130.0 (Ar-*C*H), 129.2 (Ar-*C*H), 127.9 (ImQu-*C*H), 127.6 (ImQu-$C_q$), 126.5 (ImQu-$C_q$), 125.6 (ImQu-*C*H), 122.9 (ImQu-*C*H), 121.8 (ImQu-$C_q$), 120.2 (Ar-$C_q$), 115.4 (ImQu-$C_q$), 115.4 (ImQu-*C*H), 98.0 (-C≡C-), 81.6 (-C≡C-), 36.2 (ImQu-$CH_3$), 12.5 (ImQu-$CH_3$); **IR** [cm$^{-1}$]: ṽ = 3059, 3005, 2206, 1554, 1531, 1489, 1467, 1443, 1390, 1324, 1241, 1148, 1115, 1029, 917, 815, 769, 747, 684, 636, 617, 597, 530, 519, 499, 473, 416; **HR-MS-ESI(+)** calc. $C_{21}H_{17}N_2^+$ [M]$^+$ 297.1386; found 297.1390.

Synthesis of **S50**

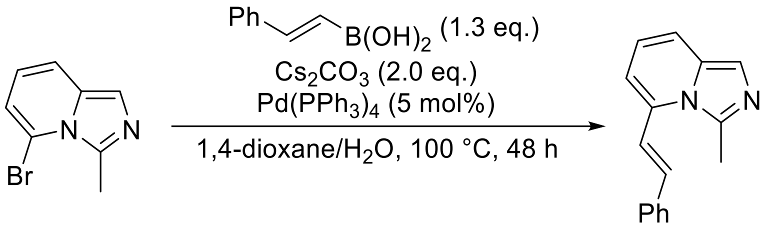


**S17** (550 mg, 2.61 mmol, 1.0 eq., 0.3M in 1,4-dioxane), *(E)*-styrylboronic acid (501 mg, 3.39 mmol, 1.3 eq.) and $Cs_2CO_3$ (1.70 g, 5.21 mmol, 2.0 eq., 2M in $H_2O$) were dissolved in a mixture of 1,4-dioxane and water (7:2, 11 mL). After degassing the mixture, $Pd(PPh_3)_4$ (151 mg, 130 µmol, 5 mol%) was added at once. The reaction mixture was heated to 100 °C for 48 h. The mixture was cooled to rt, then 100 mL of EtOAc was added and it was filtrated over a Celite frit. After separation of the phases the organic phase was washed twice with brine (2x25 mL). The organic phase was dried over $Na_2SO_4$, the solvent was removed under reduced pressure and the resulting yellow solid purified by flash column chromatography (silica, 20% EtOAc in cyclohexane). **S50** (375 mg, 1.60 mmol, 62%) was obtained as orange oil.

**m.p.** <25 °C**;** $^{1}$**H NMR** (500 MHz, $CDCl_3$): δ [ppm] = 7.58 (d, *J* = 15.8 Hz, 1H, CH=CH), 7.54 – 7.48 (m, 2H, Ar-H), 7.41 (t, *J* = 7.5 Hz, 2H, Ar-H), 7.37 – 7.29 (m, 3H, Ar-H and 2 ImPy-H), 7.02 (d, *J* = 15.8 Hz, 1H, CH=CH), 6.61 (d, *J* = 8.0 Hz, 2H, 2 ImPy-H), 2.96 (s, 3H, ImPy-$CH_3$); $^{13}$**C NMR** (126 MHz, $CDCl_3$) : δ [ppm] = 136.7 (ImPy-$C_q$), 136.3 (Ar-$C_q$), 135.0 (ImPy-$C_q$), 133.4 (CH=CH), 132.7 (ImPy-$C_q$), 129.1 (Ar-CH), 128.8 (Ar-CH), 126.9 (Ar-CH), 122.2 (CH=CH), 119.2 (ImPy-CH), 118.3 (ImPy-CH), 118.2 (ImPy-CH), 112.3 (ImPy-CH), 18.3 (ImPy-$CH_3$); **IR** [cm$^{-1}$]: ṽ = 3025, 2925, 1705, 1675, 1624, 1493, 1410, 1375, 1346, 1304, 1266, 1173, 1055,

975, 960, 841, 794, 752, 706, 691, 662, 581, 524, 484; **HR-MS-ESI(+)** calc. $C_{16}H_{15}N_2^+$ $[M+H]^+$ 235.1230; found 235.1237.

Synthesis of **4o**

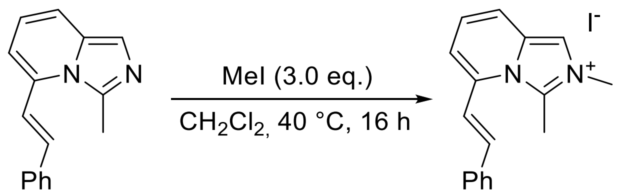

To a solution of **S50** (370 mg, 1.58 mmol, 1.0 eq.) in $CH_2Cl_2$ (5 mL) was added MeI (672 mg, 295 µL, 4.74 mmol, 3.0 eq.). The mixture was stirred for 16 h at 40 °C. Afterwards, $Et_2O$ (40 mL) was added while stirring vigorously. The solution was filtered off and the remaining brown solid was washed thrice with $Et_2O$ (3x20 mL). Drying under reduced pressure furnished **4o** as a brown-yellow solid (500 mg, 1.33 mmol, 84%).

**m.p.** 223 °C**;** $^{1}$**H NMR** (500 MHz, $CD_3CN$): δ [ppm] = 7.83 (s, 1H, ImPy-H), 7.69 – 7.65 (m, 2H, Ar-H), 7.62 (dd, *J* = 8.6, 1.8 Hz, 1H, ImPy-H), 7.54 (d, *J* = 15.9 Hz, 1H, CH=CH), 7.49 – 7.44 (m, 2H, Ar-H), 7.43 – 7.38 (m, 1H, Ar-H), 7.24 (d, *J* = 15.9 Hz, 1H, CH=CH), 7.18 – 7.11 (m, 2H, 2 ImPy-H), 4.01 (s, 3H, N-$CH_3$), 3.05 (s, 3H, ImPy-$CH_3$); $^{13}$**C NMR** (126 MHz, $CD_3CN$) : δ [ppm] = 137.3 (CH=CH), 136.9 (ImPy-$C_q$), 136.8 (ImPy-$C_q$) 136.6 (Ar-$C_q$), 131.4 (ImPy-$C_q$), 130.3 (Ar-CH), 130.0 (Ar-CH), 128.3 (Ar-CH), 125.2 (ImPy-CH), 120.7 (CH=CH), 118.3 (overlapping with solvent signal, ImPy-CH), 118.0 (ImPy-CH), 115.0 (ImPy-CH), 37.3 (ImPy-N-$CH_3$), 14.3 (ImPy-$CH_3$); **IR** [$cm^{-1}$]: ṽ = 3080, 2996, 1651, 1651, 1617, 1533, 1494, 1448, 1402, 1324, 1253, 1206, 1176, 1137, 1094, 1073, 1030, 975, 892, 795, 747, 711, 692, 636, 546, 516, 480; **HR-MS-ESI(+)** calc. $C_{17}H_{17}N_2^+$ $[M]^+$ 249.1386; found 249.1390.

Synthesis of **S51**

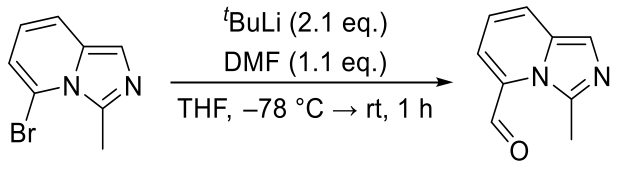

**S17** (400 mg, 1.90 mmol, 1.0 eq.) was dissolved in THF (20 mL) and cooled to –78 °C. $^t$BuLi (1.7 M in pentane, 2.34 mL, 255 mg, 3.98 mmol, 2.1 eq.) was added dropwise to the solution and it was stirred for 10 min. Afterwards, DMF (161 µL, 152 mg, 2.08 mmol, 1.1 eq.) was added dropwise to the reaction mixture and it was thawed up to rt after 10 min. Then, saturated $NH_4Cl$ solution (40 mL) was added and the mixture was extracted thrice with $CH_2Cl_2$ (3x80 mL), the combined organic extracts were washed with brine (2x50 mL) and subsequently dried over

$Na_2SO_4$. After filtration, the solvent was removed under reduced pressure to furnish **S51** (296 mg, 1.85 mmol, 98%) as a red solid.

**m.p.** 125 °C**;** **$^1$H NMR** (600 MHz, $CDCl_3$): δ [ppm] = 9.92 (s, 1H, ImPy-CHO), 7.71 (dd, *J* = 8.8, 1.3 Hz, 1H, ImPy-H), 7.64 (s, 1H, ImPy-H), 7.33 (dd, *J* = 6.7, 1.3 Hz, 1H, ImPy-H), 6.77 (dd, *J* = 8.8, 6.7 Hz, 1H, ImPy-H), 2.81 (s, 3H, ImPy-$CH_3$); **$^{13}$C NMR** (151 MHz, $CDCl_3$): δ [ppm] = 182.7 (ImPy-*C*HO), 140.4 (ImPy-$C_q$), 132.9 (ImPy-$C_q$), 132.2 (ImPy-$C_q$), 127.5 (ImPy-*C*H), 125.9 (ImPy-*C*H), 123.8 (ImPy-*C*H), 115.8 (ImPy-*C*H), 18.7 (ImPy-*C*$H_3$); **IR** [cm$^{-1}$]: ṽ = 1669, 1618, 1479, 1428, 1372, 1328, 1288, 1253, 1224, 1164, 1141, 1090, 1049, 1033, 1003, 969, 842, 807, 780, 757, 702, 659, 635, 591, 572, 541, 517,494; **HR-MS-ESI(+)** calc. $C_9H_9N_2O^+$ $[M+H]^+$ 161.0709; found 161.0711.

Synthesis of **S52**

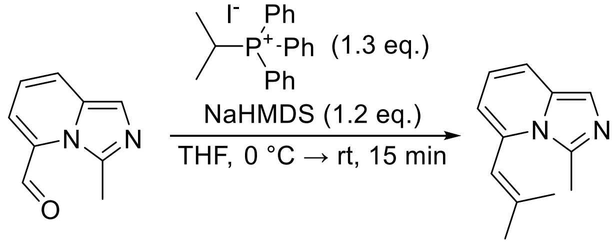


To a solid mixture of isopropyl phosphonium salt (807 mg, 1.87 mmol, 1.3 eq.) and NaHMDS (316 mg, 1.72 mmol, 1.2 eq.) was added THF (20 mL) at 0 °C. The mixture was thawed to rt over 30 min. In a second flask, **S51** (230 mg, 1.44 mmol, 1.0 eq.) was dissolved in THF (10+5 mL) and added dropwise to the Wittig ylide at 0 °C, leading to a color change from red-orange to brown. After full addition, the mixture was thawed to rt and stirred for 15 min. Afterwards, saturated $NaHCO_3$ solution (50 mL) was added and the mixture was extracted with $Et_2O$ (3x100 mL). The combined organic extracts were washed with brine (2x100 mL), dried over $Na_2SO_4$ and the solvent was removed under reduced pressure. The crude mixture was purified by column chromatography (silica, 20% EtOAc to 30% in cyclohexane) to furnish **S52** (180 mg, 966 µmol, 67%) as light-yellow oil.

**m.p.** 237 °C**;** **$^1$H NMR** (600 MHz, $CD_3CN$): δ [ppm] = 7.28 (dt, *J* = 9.2, 1.0 Hz, 1H, ImPy-H), 7.18 (s, 1H, ImPy-H), 6.59 (dd, *J* = 9.1, 6.5 Hz, 1H, ImPy-H), 6.55 (m, 1H, ImPy-C*H*=C($CH_3$)$_2$), 6.21 (dt, *J* = 6.5, 1.3 Hz, 1H, ImPy-$CH_3$), 2.75 (s, 3H, ImPy-$CH_3$), 1.95 (d, *J* = 1.6 Hz, 3H, ImPy-CH=C(C*H*$_3$)$_2$), 1.79 (d, *J* = 1.4 Hz, 3H, ImPy-CH=C(C*H*$_3$)$_2$); **$^{13}$C NMR** (151 MHz, $CD_3CN$): δ [ppm] = 141.3 (ImPy-CH=*C*($CH_3$)$_2$), 137.7 (ImPy-$C_q$), 134.2 (ImPy-$C_q$), 133.4 (ImPy-$C_q$), 120.4 (ImPy-*C*H=C($CH_3$)$_2$), 119.0 (ImPy-*C*H), 118.9 (ImPy-*C*H), 117.6 (ImPy-*C*H), 114.6 (ImPy-*C*H), 25.7 (ImPy-CH=C(*C*$H_3$)$_2$), 19.8 (ImPy-CH=C(*C*$H_3$)$_2$), 17.7 (ImPy-*C*$H_3$); **IR** [cm$^{-1}$]: ṽ =2970, 2929, 2856, 1657, 1632, 1525, 1487, 1440, 1372, 1321, 1294, 1193, 1067, 1041, 1016, 976,

859, 792, 749, 708, 664, 609, 459; **HR-MS-ESI(+)** calc. $C_{12}H_{15}N_2^+$ $[M+H]^+$ 187.1230; found 187.1231.

Synthesis of **4p**

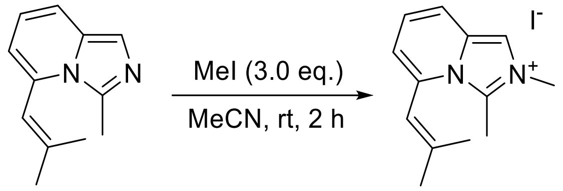


To a solution of **S52** (175 mg, 940 µmol, 1.0 eq.) in MeCN (5 mL) was added MeI (400 mg, 175 µL, 2.82 mmol, 3.0 eq.). The mixture was stirred for 2 h at rt. Afterwards, $Et_2O$ (40 mL) was added while stirring vigorously. The solution was filtered off and the remaining colorless solid was washed thrice with $Et_2O$ (3x20 mL). Drying under reduced pressure furnished **4o** as a colorless solid (150 mg, 457 µmol, 49%).

**m.p.** <25 °C**;** $^{1}$**H NMR** (600 MHz, DMSO-$d_6$): δ [ppm] = 8.15 (s, 1H, ImPy-H), 7.70 (dt, *J* = 9.2, 1.0 Hz, 1H, ImPy-H), 7.15 (dd, *J* = 9.3, 6.8 Hz, 1H, ImPy-H), 6.76 (dt, *J* = 6.8, 1.3 Hz, 1H, ImPy-H), 6.61 – 6.57 (m, 1H, ImPy-C*H*=C($CH_3$)$_2$), 4.03 (s, 3H, ImPy-N-$CH_3$), 2.97 (s, 3H, ImPy-$CH_3$), 2.00 (d, *J* = 1.5 Hz, 3H, ImPy-CH=C(C$H_3$)$_2$), 1.81 (d, *J* = 1.4 Hz, 3H, ImPy-CH=C(C$H_3$)$_2$); $^{13}$**C NMR** (151 MHz, DMSO-$d_6$): δ [ppm] = 143.1 (ImPy-CH=*C*($CH_3$)$_2$), 135.9 (ImPy-$C_q$), 133.4 (ImPy-$C_q$), 129.5 (ImPy-$C_q$), 123.6 (ImPy-*C*H), 118.5 (ImPy-*C*H), 117.5 (ImPy-*C*H=C($CH_3$)$_2$), 116.8 (ImPy-*C*H), 113.9 (ImPy-*C*H), 36.0 (ImPy-N-*C*$H_3$), 25.4 (ImPy-CH=C(*C*$H_3$)$_2$), 19.7 (ImPy-CH=C(*C*$H_3$)$_2$), 13.0 (ImPy-*C*$H_3$); **IR** [$cm^{-1}$]: ṽ = 3107, 3036, 2977, 2938, 1854, 1652, 1542, 1474, 1433, 1386, 1327, 1258, 1198, 1175, 1129, 1095, 1061, 1015, 980, 959, 930, 892, 851, 799, 750, 730, 711, 665, 626, 528, 499, 471; **HR-MS-ESI(+)** calc. $C_{13}H_{17}N_2^+$ $[M]^+$ 201.1386; found 201.1381.

## 2.4 Synthesis of Imidazo[1,5-*a*]pyridine NHOs

Synthesis of **2a**

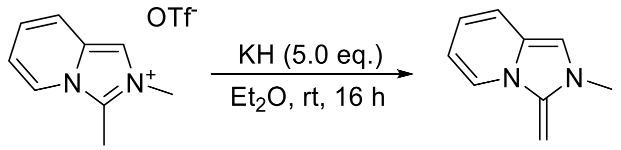

To a mixture of solid **1a** (400 mg, 1.35 mmol, 1.0 eq.) and KH (271 mg, 6.75 mmol, 5.0 eq.) was added $Et_2O$ (40 mL) at rt. The mixture turns intensely purple over the course of 1 h. After stirring for 16 h in total, excess KH and KOTf were filtered off and the solvent was removed under reduced pressure, furnishing **2a** (180 mg, 1.23 mmol, 99%) as a purple solid.

**NOTE: 2a** decomposes over multiple hours at rt, storage at -40 °C is advised.

**m.p.** 35 °C (decomp); **$^1$H NMR** (500 MHz, $C_6D_6$): δ [ppm] = 6.52 (dd, *J* = 7.4, 1.1 Hz, 1H, ImPy-H), 6.16 (d, *J* = 9.5 Hz, 1H, ImPy-H), 5.71 (dd, *J* = 9.5, 6.0 Hz, 1H, ImPy-H), 5.35 (s, 1H, ImPy-H), 5.25 (ddd, *J* = 7.3, 6.0, 1.1 Hz, 1H, ImPy-H), 3.02 (s, 1H, ImPy=$CH_2$), 2.85 (s, 1H, ImPy=$CH_2$), 2.48 (s, 3H, ImPy-N-$CH_3$); **$^{13}$C NMR** (126 MHz, $C_6D_6$): δ [ppm] = 146.9 (ImPy-$C_q$), 124.6 (ImPy-*C*H), 124.1 (ImPy-$C_q$), 120.0 (ImPy-*C*H), 117.8 (ImPy-*C*H), 105.5 (ImPy-*C*H), 102.4 (ImPy-*C*H), 40.3 (ImPy=$CH_2$), 32.4 (ImPy-$CH_3$); **IR** [$cm^{-1}$]: ṽ = 3124, 2924, 2854, 1617, 1574, 1445, 1422, 1401, 1365, 1347, 1295, 1260, 1168, 1145, 1035, 896, 802, 725, 634, 561, 529, 497; **HR-MS-ESI(+)** calc. $C_9H_{11}N_2^+$ $[M+H]^+$ 147.0917; found 147.0916.

Synthesis of **2b**

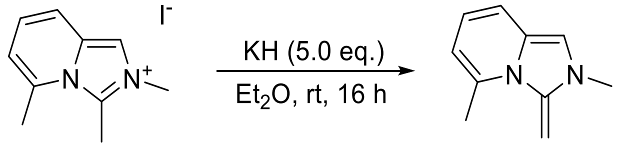

To a mixture of solid **1b** (800 mg, 2.78 mmol, 1.0 eq.) and KH (557 mg, 13.9 mmol, 5.0 eq.) was added $Et_2O$ (80 mL) at rt. The mixture turns intensely red over the course of 1 h. After stirring for 16 h in total, excess KH and KI were filtered off and the solvent was removed under reduced pressure, furnishing **2b** (440 mg, 2.78 mmol, 99%) as an intense red liquid at rt.

**m.p.** <25 °C; **$^1$H NMR** (500 MHz, $C_6D_6$): δ [ppm] = 5.94 (d, *J* = 9.5 Hz, 1H, ImPy-H), 5.55 – 5.51 (m, 1H, ImPy-H), 5.23 (s, 1H, ImPy-H), 4.76 (dp, *J* = 6.1, 1.3 Hz, 1H, ImPy-H), 3.57 (s, 1H, ImPy=$CH_2$), 2.64 (s, 1H, ImPy=$CH_2$), 2.36 (s, 3H, ImPy-N-$CH_3$), 2.25 (s, 3H, ImPy-$CH_3$); **$^{13}$C NMR** (126 MHz, $C_6D_6$): δ [ppm] = 149.0 (ImPy-$C_q$), 138.7 (ImPy-$C_q$), 126.3 (ImPy-$C_q$), 120.4 (ImPy-*C*H), 116.2 (ImPy-*C*H), 105.9 (ImPy-*C*H), 103.4 (ImPy-*C*H), 47.4 (ImPy=$CH_2$), 32.9 (ImPy-N-$CH_3$), 20.8 (ImPy-$CH_3$); **IR** [$cm^{-1}$]: ṽ = 1615.8, 1571.9, 1545.8, 1407.2, 1383.7,

1354.6, 1312.5, 1296.9, 1133.4, 745.4, 626.1, 540.4; **HR-MS-ESI(+)** calc. $C_{10}H_{13}N_2^+$ $[M+H]^+$ 161.1074; found 161.1071.

Synthesis of **2c**

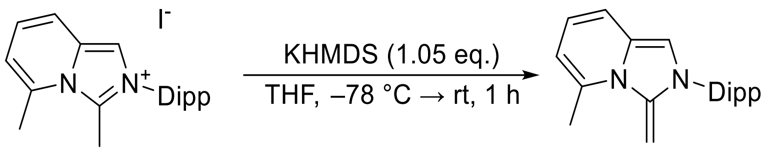


To a mixture of solid **1c** (800 mg, 1.84 mmol, 1.05 eq.) and KHMDS (385 mg, 1.93 mmol, 1.05 eq.) was added THF (50 mL) at 78 °C. The mixture instantaneously turns red violet. After stirring for 5 minutes at –78 °C the solution was warmed to rt and stirred for 1 h. The solvent was removed under reduced pressure and the remaining solid extracted with $Et_2O$ (20 mL+2x10 mL). The solvent was removed under reduced pressure and the crude product recrystallized from a saturated $Et_2O$:pentane (10:1) mixture at -40 °C, furnishing **2c** (520 mg, 1.70 mmol, 92%) as intense red crystalline blocks.

**m.p.** 170 °C**;** $^{1}$**H NMR** (500 MHz, $C_6D_6$): δ [ppm] = 7.23 (dd, *J* = 8.5, 6.9 Hz, 1H, Dipp-H), 7.19 – 7.13 (m, 2H, Dipp-H), 5.94 (d, *J* = 9.5 Hz, 1H, ImPy-H), 5.64 (s, 1H, ImPy-H), 5.52 (dd, *J* = 9.6, 6.1 Hz, 1H, ImPy-H), 4.73 (dt, *J* = 6.1, 1.2 Hz, 1H, ImPy-H), 3.50 (d, *J* = 3.1 Hz, 1H, ImPy=$CH_2$), 3.17 (hept, *J* = 6.9 Hz, 2H, Dipp-C*H*($CH_3$)$_2$), 2.53 (d, *J* = 3.1 Hz, 1H, ImPy=$CH_2$), 2.23 (s, 3H, ImPy-$CH_3$), 1.31 (d, *J* = 6.9 Hz, 6H, Dipp-CH(C$H_3$)$_2$), 1.12 (d, *J* = 6.9 Hz, 6H, Dipp-CH(C$H_3$)$_2$); $^{13}$**C NMR** (126 MHz, $C_6D_6$): δ [ppm] = 149.3 (ImPy-$C_q$), 149.0 (Dipp-$C_q$), 139.3 (ImPy-$C_q$), 134.5 (Dipp-$C_q$), 129.5 (Dipp-*C*H), 128.4 (Dipp-*C*H), 127.7 (ImPy-$C_q$), 124.9 (Dipp-$C_q$), 121.3 (ImPy-*C*H), 116.5 (ImPy-*C*H), 106.1 (ImPy-*C*H), 103.5 (ImPy-*C*H), 49.7 (ImPy=*C*$H_2$), 28.6 (Dipp-*C*H($CH_3$)$_2$), 24.6 (Dipp-CH(*C*$H_3$)$_2$), 23.8 (Dipp-(*C*$H_3$)$_2$), 20.8 (ImPy-*C*$H_3$); **IR** [cm$^{-1}$]: ṽ = 2967, 2867, 1621, 1571, 1548, 1472, 1416, 1383, 1358, 1310, 1232, 1173, 1057, 841, 808, 785, 752, 631, 603; **HR-MS-ESI(+)** calc. $C_{21}H_{27}N_2^+$ $[M+H]^+$ 307.2169; found 307.2169.

Synthesis of **2d**

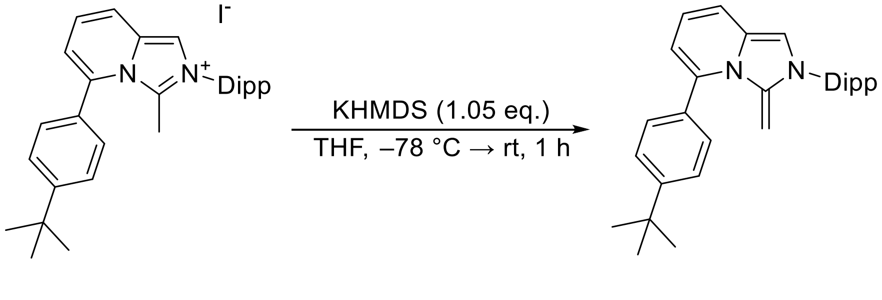


To a mixture of solid **1d** (1.00 g, 1.81 mmol, 1.0 eq.) and KHMDS (379 mg, 1.90 mmol, 1.05 eq.) was added THF (25 mL) at –78 °C. The mixture instantaneously turns dark purple.

After stirring for 5 minutes at –78 °C the solution was warmed to rt and stirred for 1 h. The solvent was removed under reduced pressure and the remaining solid extracted with $Et_2O$ (20 mL+2x10 mL). Drying in vacuo furnished **2d** (690 mg, 1.62 mmol, 89%) as dark violet solid.

**m.p. ;** 148 °C; **$^1$H NMR** (500 MHz, $C_6D_6$): δ [ppm] = 7.32 – 7.29 (m, 2H, Ar-H), 7.17 – 7.13 (m, 3H, Ar-H and Dipp-H), 7.08 (d, *J* = 8.2 Hz, 2H, Dipp-H), 6.18 (dd, *J* = 9.5, 1.2 Hz, 1H, ImPy-H), 5.88 (s, 1H, ImPy-H), 5.73 (dd, *J* = 9.5, 6.2 Hz, 1H, ImPy-H), 5.33 (dd, *J* = 6.2, 1.2 Hz, 1H, ImPy-H), 3.10 (hept, *J* = 6.9 Hz, 2H, Dipp-C*H*$(CH_3)_2$), 2.61 (d, *J* = 2.9 Hz, 1H, ImPy=$CH_2$), 2.09 (d, *J* = 2.8 Hz, 1H, ImPy=$CH_2$), 1.22 (d, *J* = 6.9 Hz, 6H, Dipp-CH$(CH_3)_2$), 1.12 (d, *J* = 6.9 Hz, 6H, Dipp-CH$(CH_3)_2$), 1.10 (s, 9H, $C(CH_3)_3$); **$^{13}$C NMR** (126 MHz, $C_6D_6$): δ [ppm] = 151.1 (Ar-$C_q$), 148.9 (Ar-$C_q$), 147.8 (ImPy-$C_q$), 141.4 (ImPy-$C_q$), 134.6 (Ar-$C_q$), 132.2 (Ar-$C_q$), 129.5 (Ar-*C*H), 129.0 (Ar-*C*H), 128.4 (ImPy-$C_q$), 127.2 (ImPy-$C_q$), 124.8 (Ar-*C*H), 124.1 (Ar-*C*H), 120.4 (ImPy-*C*H), 117.8 (ImPy-*C*H), 110.2 (ImPy-*C*H), 104.4 (ImPy-*C*H), 53.3 (ImPy=$CH_2$), 34.6 ($C(CH_3)_3$), 31.3 ($C(CH_3)_3$), 28.7 (Dipp-*C*H$(CH_3)_2$), 24.6 (Dipp-CH$(CH_3)_2$), 23.7 (Dipp-CH$(CH_3)_2$); **IR** [$cm^{-1}$]: ṽ = 2961.4, 1622.3, 1458.2, 1380.2, 1244.0, 1175.7, 1018.8, 830.4, 807.2, 778.9, 752.5, 744.4, 625.3, 605.8, 559.5; **HR-MS-ESI(+)** calc. $C_{30}H_{37}N_2^+$ $[M+H]^+$ 425.2951; found 425.2949.

Synthesis of **2e**

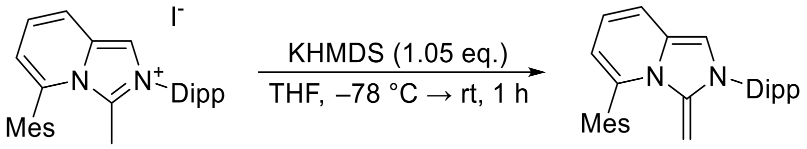


To a mixture of solid **1e** (300 mg, 0.56 mmol, 1.0 eq.) and KHMDS (122 mg, 0.61 mmol, 1.10 eq.) was added THF (25 mL) at 78 °C. The mixture instantaneously turns dark violet. After stirring for 5 minutes at –78 °C the solution was warmed to rt and stirred for 1 h. The solvent was removed under reduced pressure and the remaining solid extracted with $Et_2O$ (20 mL+2x10 mL). After removal of the solvent under reduced pressure, **2e** (207 mg, 0.50 mmol, 91%) was furnished as dark violet solid.

**m.p.** 122 °C**;** **$^1$H NMR** (500 MHz, $C_6D_6$): δ [ppm] = 7.15 (t, *J* = 8.5 Hz, 1H, Ar-H), 7.10 – 7.07 (m, 2H, Ar-H), 6.68 (s, 2H, Ar-H), 6.12 (dd, *J* = 9.4, 1.2 Hz, 1H, ImPy-H), 5.76 – 5.69 (m, 2H, ImPy-H), 5.08 (dd, *J* = 6.1, 1.2 Hz, 1H, ImPy-H), 3.12 (hept, *J* = 6.9 Hz, 2H, C*H*$(CH_3)_2$), 2.39 (s, 6H, Mes-$(CH_3)_2$), 2.29 (d, *J* = 2.8 Hz, 1H, ImPy=$CH_2$), 2.13 (d, *J* = 2.8 Hz, 1H, ImPy=$CH_2$), 2.03 (s, 3H, Mes-$CH_3$), 1.27 (d, *J* = 6.9 Hz, 6H, CH$(CH_3)_2$), 1.13 (d, *J* = 6.9 Hz, 6H, CH$(CH_3)_2$); **$^{13}$C NMR** (126 MHz, $C_6D_6$): δ [ppm] = 148.9 (Ar-$C_q$), 147.8 (ImPy-$C_q$), 140.0 (ImPy-$C_q$), 138.2 (Ar-$C_q$), 137.3 (Ar-$C_q$), 134.6 (Ar-$C_q$), 132.7 (Ar-$C_q$), 129.4 (Ar-$C_q$), 128.4 (Ar-*C*H), 127.9 (Ar-*C*H), 127.0 (ImPy-$C_q$), 124.8 (Ar-*C*H), 120.8 (ImPy-*C*H), 117.3 (ImPy-*C*H), 106.8 (ImPy-*C*H),

103.5 (ImPy-*C*H), 48.2 (ImPy=*C*$H_2$), 28.6 (*C*H($CH_3$)$_2$), 24.5 (*C*H($CH_3$)$_2$), 23.7 (CH(*C*$H_3$)$_2$), 21.1 (Mes-*C*$H_3$), 20.6 (Mes-(*C*$H_3$)$_2$); **IR** [$cm^{-1}$]: ṽ = 2959.4, 2864.7, 1620.5, 1569.6, 1541.3, 1470.2, 1411.5, 1385.8, 1353.5, 1309.5, 1255.3, 1178.2, 1101.7, 1015.0, 935.7, 845.9, 807.7, 779.1, 757.9, 729.2, 654.3, 616.0, 580.2, 438.7; **HR-MS-ESI(+)** calc. $C_{29}H_{35}N_2^+$ $[M+H]^+$ 411.2795; found 411.2794.

Synthesis of **2f**

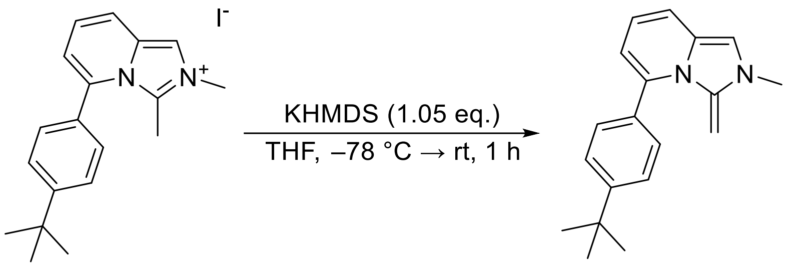


To a mixture of solid **1f** (500 mg, 1.23 mmol, 1.00 eq.) and KHMDS (270 mg, 1.35 mmol, 1.10 eq.) was added THF (30 mL) at –78 °C. The mixture instantaneously turns dark violet. After stirring for 5 minutes at –78 °C the solution was warmed to rt and stirred for 1 h. The solvent was removed under reduced pressure and the remaining solid extracted with $Et_2O$ (20 mL+10 mL). The solvent was removed under reduced pressure, furnishing **2f** (330 mg, 1.19 mmol, 96%) as dark violet solid.

**m.p.** 94 °C; $^1$**H NMR** (500 MHz, $C_6D_6$): δ [ppm] = 7.35 – 7.29 (m, 2H, Ar-H), 7.25 – 7.21 (m, 2H, Ar-H), 6.21 (dd, *J* = 9.4, 1.2 Hz, 1H, ImPy-H), 5.76 (dd, *J* = 9.4, 6.2 Hz, 1H, ImPy-H), 5.51 (s, 1H, ImPy-H), 5.38 (dd, *J* = 6.2, 1.2 Hz, 1H, ImPy-H), 2.81 (s, 1H, ImPy=$CH_2$), 2.42 (s, 3H, ImPy-N-$CH_3$), 2.21 (s, 1H, ImPy=$CH_2$), 1.18 (s, 9H, C($CH_3$)$_3$); $^{13}$**C NMR** (126 MHz, $C_6D_6$): δ [ppm] = 151.1 (Ar-$C_q$), 148.0 (ImPy-$C_q$), 140.9 (ImPy-$C_q$), 132.5 (Ar-$C_q$), 129.0 (Ar-*C*H), 126.0 (ImPy-$C_q$), 124.1 (Ar-*C*H), 119.5 (ImPy-*C*H), 117.5 (ImPy-*C*H), 110.1 (ImPy-*C*H), 104.4 (ImPy-*C*H), 51.1 (ImPy=*C*$H_2$), 34.6 (*C*($CH_3$)$_3$), 32.9 (ImPy-N-*C*$H_3$), 31.4 (C(*C*$H_3$)$_3$); **IR** [$cm^{-1}$]: ṽ = 2956, 1623, 1571, 1506, 1403, 1381, 1306, 1268, 1162, 1135, 1118, 1099, 1071, 1016, 845, 827, 754, 742, 731, 650, 626, 602, 565, 531, 510, 493; **HR-MS-ESI(+)** calc $C_{19}H_{23}N_2^+$ $[M+H]^+$ 279.1856; found 279.1853.

Synthesis of **2g**

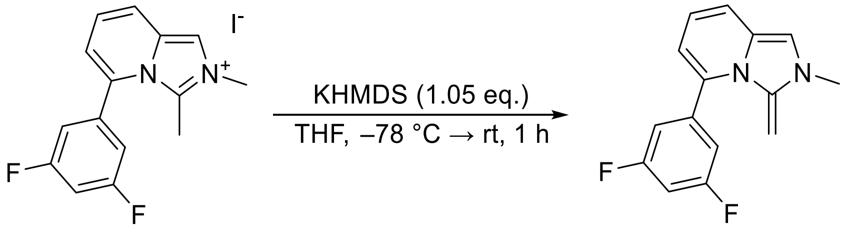

To a mixture of solid **1g** (200 mg, 518 µmol, 1.00 eq.) and KHMDS (108 mg, 544 µmol, 1.05 eq.) was added THF (20 mL) at –78 °C. The mixture instantaneously turns dark blue. After stirring for 5 minutes at –78 °C the solution was warmed to rt and stirred for 1 h. The solvent was removed under reduced pressure and the remaining oil extracted with pentane (20 mL + 10 mL). After removal of the solvent under reduced pressure, **2g** (133 mg, 515 mmol, quant.) was obtained as dark blue oil.

**m.p.** >25 °C; **$^1$H NMR** (400 MHz, $C_6D_6$): δ [ppm] = 6.59 (dddd, *J* = 6.6, 5.5, 4.4, 2.2 Hz, 2H, Ar-H), 6.44 (tt, *J* = 8.9, 2.4 Hz, 1H, Ar-H), 6.16 (dd, *J* = 9.4, 1.1 Hz, 1H, ImPy-H), 5.62 (dd, *J* = 9.4, 6.2 Hz, 1H, ImPy-H), 5.48 (s, 1H, ImPy-H), 5.10 (dd, *J* = 6.2, 1.1 Hz, 1H, ImPy-H), 2.81 (d, *J* = 3.5 Hz, 1H, ImPy=$CH_2$), 2.40 (s, 3H, ImPy-N-$CH_3$), 1.92 (d, *J* = 3.4 Hz, 1H, ImPy=$CH_2$); **$^{13}$C NMR** (101 MHz, $C_6D_6$): δ [ppm] = 162.0 (dd, *J* = 248.2, 12.9 Hz, Ar-$C_q$), 147.3 (ImPy-$C_q$), 137.7 (t, *J* = 2.7 Hz, ImPy-$C_q$), 137.4 (t, *J* = 10.1 Hz, Ar-$C_q$), 125.5 (ImPy-$C_q$), 118.7 (ImPy-*C*H), 118.7 (ImPy-*C*H), 112.1 (m, Ar-*C*H), 111.6 (ImPy-*C*H), 105.3 (ImPy-*C*H), 103.2 (d, *J* = 25.3 Hz, Ar-*C*H), 51.2 (ImPy=*C*$H_2$), 32.9 (N-*C*$H_3$); **$^{19}$F NMR** (377 MHz, $C_6D_6$): δ [ppm] = -110.66 – -110.76 (m, 2F, Ar-F); **IR** [$cm^{-1}$]: ṽ = 1617, 1593, 1573, 1436, 1405, 1391, 1325, 1238, 1119, 1072, 1003, 986, 859, 756, 688, 606; **HR-MS-ESI(+)** calc. $C_9H_{11}N_2^+$ $[M+H]^+$ 259.1041; found 259.1047.

Synthesis of **5b**

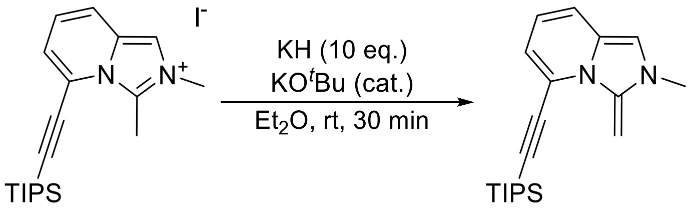


To a mixture of solid **4b** (100 mg, 0.22 mmol, 1.00 eq.), KH (88 mg, 2.20 mmol, 10 eq.) and catalytic amounts of KO*t*Bu was added $Et_2O$ (10 mL) at rt. The suspension was stirred for 30 min, filtered, the residue was washed with pentane (3x2 mL) and the solvent was removed under reduced pressure, furnishing NHO **5b** (64.0 mg, 0.20 mmol, 88%) as dark-blue solid.

**m.p.** 75 °C**;** **$^1$H NMR** (400 MHz, $C_6D_6$): δ [ppm] = 5.90 (dt, *J* = 9.4, 0.8 Hz, 1H, ImPy-H), 5.72 (dd, *J* = 6.4, 1.1 Hz, 1H, ImPy-H), 5.32 (dd, *J* = 9.4, 6.4 Hz, 1H, ImPy-H), 5.23 (s, 1H, ImPy-H), 4.76 (d, *J* = 3.1 Hz, 1H, ImPy=$CH_2$), 2.92 (d, *J* = 3.1 Hz, 1H, ImPy=$CH_2$), 2.34 (s, 3H, ImPy-N-$CH_3$), 1.21 – 1.11 (m, 21H, -Si(CH($CH_3$)$_2$)$_3$); **$^{13}$C NMR** (101 MHz, $C_6D_6$): δ [ppm] = 147.8 (ImPy-$C_q$), 125.4 (ImPy-$C_q$), 122.2 (ImPy-$C_q$), 120.0 (ImPy-*C*H), 118.8 (ImPy-*C*H), 118.1 (ImPy-*C*H), 104.8 (ImPy-*C*H), 102.0 (-C≡C-), 100.0 (-C≡C-), 47.6 (ImPy=*C*$H_2$), 32.6 (ImPy-N-*C*$H_3$), 18.9 (-Si(CH(*C*$H_3$)$_2$)$_3$), 11.8 (-Si(*C*H($CH_3$)$_2$)$_3$); **IR** [$cm^{-1}$]: ṽ = 2941, 2863, 2137, 1622, 1462, 1406, 1395, 1361, 1300, 1212, 1163, 1134, 1076, 1032, 1012, 998, 920, 883, 803, 758, 735,

669, 639, 582, 513, 458, 412; **HR-MS-ESI(+)** calc. $C_{20}H_{31}N_2Si^+$ $[M+H]^+$ 327.2251; found 327.2264; **UV-Vis**: $\boldsymbol{\lambda}_{\mathbf{max}}$: 331 nm (ε = 8443 $cm^{-1}$ $M^{-1}$), 787 nm (ε = 2150 $cm^{-1}$ $M^{-1}$).

## 2.5 Synthesis of Imidazo[2,1,5-*de*]quinolizine Cyclization Products

Synthesis of **6a**

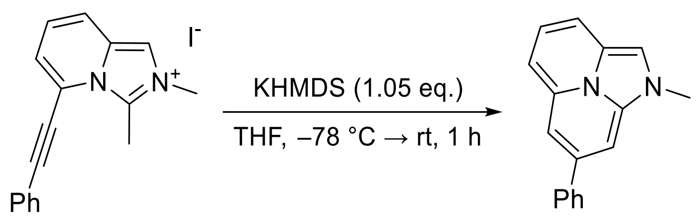


To a mixture of solid **4a** (150 mg, 0.40 mmol, 1.0 eq.) and KHMDS (83.0 mg, 0.42 mmol, 1.05 eq.) was added THF (10 mL) at –78 °C. The mixture instantaneously turned dark blue. After stirring for 5 minutes at –78 °C the solution was warmed to rt and stirred for 1 h, in which time the solution turned dark yellow-green. The solvent was removed under reduced pressure and the remaining solid extracted with $Et_2O$ (2x10 mL). The solvent was removed under reduced pressure, furnishing **6a** (84.0 mg, 0.34 mmol, 85%) as greenish solid.

**m.p.** 121 °C**;** $^{1}$**H NMR** (500 MHz, $C_6D_6$): δ [ppm] = 7.18 – 7.11 (m, 2H, Ar-H), 7.08 – 7.03 (m, 3H, Ar-H), 4.98 (dd, *J* = 9.3, 6.9 Hz, 1H, ImQu-H), 3.97 (dt, *J* = 9.4, 0.7 Hz, 1H, ImQu-H), 3.93 (d, *J* = 1.5 Hz, 1H, ImQu-H), 3.84 (s, 1H, ImQu-H), 3.30 – 3.24 (m, 2H, ImQu-H), 1.43 (s, 3H, ImQu-N-$CH_3$); $^{13}$**C NMR** (126 MHz, $C_6D_6$): δ [ppm] = 153.3 (ImQu-$C_q$), 152.9 (ImQu-$C_q$), 150.3 (ImQu-$C_q$), 140.4 (Ar-$C_q$), 136.6 (ImQu-$C_q$), 135.2 (ImQu-*C*H), 128.6 (Ar-*C*H), 128.5 (Ar-*C*H), 124.7 (Ar-*C*H), 99.7 (ImQu-*C*H), 99.7 (ImQu-*C*H) 98.5 (ImQu-*C*H), 87.7 (ImQu-*C*H), 78.7 (ImQu-*C*H), 29.8 (ImQu-N-$CH_3$); **IR** [$cm^{-1}$]: ṽ = 1617, 1548, 1507, 1463, 1397, 1375, 1311, 1276, 1253, 1210, 1190, 1157, 1131, 1094, 1073, 1034, 972, 821, 792, 762, 746, 690, 626, 584, 544, 500, 437; **HR-MS-ESI(+)** calc. $[M+H]^+$ 247.1200; found 247.1226; **UV-Vis**: **$\lambda_{max}$**: 430 nm (ε = 6205 $cm^{-1}$ $M^{-1}$), 512 nm (ε = 687 $cm^{-1}$ $M^{-1}$), 742 nm (ε = 829 $cm^{-1}$ $M^{-1}$), 825 nm (ε = 777 $cm^{-1}$ $M^{-1}$).

Synthesis of **6b**

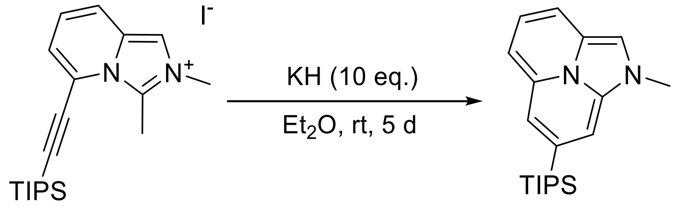


To a mixture of solid **4b** (50 mg, 0.11 mmol, 1.0 eq.) and KH (44 mg, 1.10 mmol, 10 eq.) was added $Et_2O$ (10 mL) at rt for 5 d. The suspension was filtered, the residue was washed with cyclohexane (2x4 mL) and the solvent was removed under reduced pressure, furnishing **6b** (31.0 mg, 0.10 mmol, 88%) as dark-green solid. Alternatively, **5b** can be heated to 80 °C for 30 min in $C_6D_6$ for full conversion.

**m.p.** 132 °C**;** $^{1}$**H NMR** (600 MHz, $C_6D_6$): δ [ppm] = 4.91 (dd, *J* = 9.4, 6.9 Hz, 1H, ImQu-H), 3.90 – 3.84 (m, 2H, ImQu-H), 3.65 (s, 1H, ImQu-H), 3.19 – 3.14 (m, 2H, ImQu-H), 1.27 (s, 3H, ImQu-$CH_3$), 1.12 (d, *J* = 7.5 Hz, 18H, -Si(CH(C$H_3$)$_2$)$_3$), 0.89 (hept, *J* = 7.4 Hz, 3H, -Si(C*H*($CH_3$)$_2$)$_3$); $^{13}$**C NMR** (151 MHz, $C_6D_6$): δ [ppm] = 152.4 (ImQu-$C_q$), 149.3 (ImQu-$C_q$), 148.2 (ImQu-$C_q$), 137.3 (ImQu-$C_q$), 135.7 (ImQu-*C*H), 106.9 (ImQu-*C*H), 99.9 (ImQu-*C*H), 99.3 (ImQu-*C*H), 87.1 (ImQu-*C*H), 84.5 (ImQu-*C*H), 29.5 (ImQu-N-*C*$H_3$), 18.7 (-Si(CH(*C*$H_3$)$_2$)$_3$), 10.8 (-Si(*C*H-($CH_3$)$_2$)$_3$); **IR** [cm$^{-1}$]: ṽ = 2940, 2862, 1637, 1611, 1545, 1490, 1464, 1400, 1317, 1298, 1266, 1232, 1200, 1165, 1135, 1076, 1054, 1037, 1013, 993, 919, 882, 852, 816, 784, 740, 691, 678, 653, 608, 577, 547, 524, 493, 432; **HR-MS-ESI(+)** calc. $C_{20}H_{31}N_2Si^+$ $[M+H]^+$ 327.2251; found 327.2261; **UV-Vis**: $\lambda_{max}$: 358 nm (ε = 6576 cm$^{-1}$ M$^{-1}$), 405 nm (ε = 7342 cm$^{-1}$ M$^{-1}$), 422 nm (ε = 6980 cm$^{-1}$ M$^{-1}$), 654 nm (ε = 479 cm$^{-1}$ M$^{-1}$), 730 nm (ε = 850 cm$^{-1}$ M$^{-1}$), 827 nm (ε = 979 cm$^{-1}$ M$^{-1}$), 954 nm (ε = 523 cm$^{-1}$ M$^{-1}$).

Synthesis of **6c**

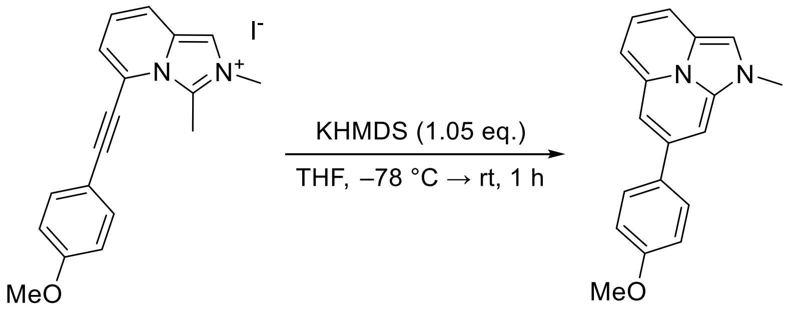


To a mixture of solid **4c** (150 mg, 0.37 mmol, 1.00 eq.) and KHMDS (77.0 mg, 0.39 mmol, 1.05 eq.) was added THF (10 mL) at –78 °C. The mixture instantaneously turned dark blue. After stirring for 5 minutes at –78 °C the solution was warmed to rt and stirred for 1 h, in which time the solution turned dark violet. The solvent was removed under reduced pressure and the remaining solid extracted with $Et_2O$ (2x10 mL). The solvent was removed under reduced pressure, furnishing **6c** (22.0 mg, 0.08 mmol, 22%) as greenish solid.

**m.p.** 130 °C**;** $^{1}$**H NMR** (500 MHz, $C_6D_6$): δ [ppm] = 7.19 – 7.13 (m, 2H, Ar-H), 6.75 – 6.66 (m, 2H, Ar-H), 5.07 (dd, *J* = 9.3, 6.9 Hz, 1H, ImQu-H), 4.06 (d, *J* = 1.5 Hz, 1H, ImQu-H), 4.04 (d, *J* = 9.3 Hz, 1H, ImQu-H), 3.88 (s, 1H, ImQu-H), 3.42 – 3.36 (m, 2H, ImQu-H), 3.29 (s, 3H, Ar-O-$CH_3$), 1.47 (s, 3H, ImQu-N-$CH_3$); $^{13}$**C NMR** (126 MHz, $C_6D_6$): δ [ppm] = 160.4 (Ar-$C_q$), 152.8 (ImQu-$C_q$), 152.7 (ImQu-$C_q$), 150.2 (ImQu-$C_q$), 136.5 (ImQu-$C_q$), 135.2 (ImQu-*C*H), 132.6 (Ar-$C_q$), 126.0 (Ar-*C*H), 113.9 (Ar-*C*H), 99.5 (ImQu-*C*H), 99.3 (ImQu-*C*H), 97.8 (ImQu-*C*H), 87.5 (ImQu-*C*H), 78.4 (ImQu-*C*H), 54.8 (Ar-O-*C*$H_3$), 29.8 (ImQu-N-*C*$H_3$); **IR** [cm$^{-1}$]: ṽ = 1623, 1603, 1577, 1546, 1518, 1468, 1398, 1311, 1287, 1248, 1213, 1179, 1162, 1138, 1096, 1065, 1027, 972, 861, 821, 778, 745, 723, 747, 723, 696, 672, 639, 594, 565, 544, 514, 469; **HRMS-ESI(+)**

calc. [M+H]$^+$ $C_{18}H_{17}N_2O^+$ 277.1300; found 277.1334; **UV-Vis:** $\lambda_{max}$: 428 nm (ε = 4772 $cm^{-1}$ $M^{-1}$), 734 nm (ε = 654 $cm^{-1}$ $M^{-1}$), 818 nm (ε = 626 $cm^{-1}$ $M^{-1}$).

Synthesis of **6d**

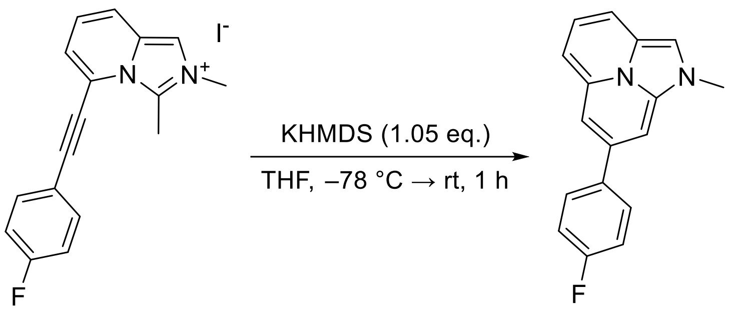


To a mixture of solid **4d** (150 mg, 0.38 mmol, 1.0 eq.) and KHMDS (80.0 mg, 0.40 mmol, 1.05 eq.) was added THF (10 mL) at –78 °C. The mixture instantaneously turned dark blue. After stirring for 5 minutes at –78 °C the solution was warmed to rt and stirred for 1 h, in which time the solution turned dark violet. The solvent was removed under reduced pressure and the remaining solid extracted with $Et_2O$ (2x10 mL). The solvent was removed under reduced pressure, furnishing **6d** (48.0 mg, 0.18 mmol, 48%) as greenish solid.

**m.p.** 117 °C**;** **$^1$H NMR** (500 MHz, $C_6D_6$): δ [ppm] = 6.99 – 6.90 (m, 2H, Ar-H), 6.75 – 6.66 (m, 2H, Ar-H), 5.02 (dd, *J* = 9.3, 6.9 Hz, 1H, ImQu-H), 4.00 (dd, *J* = 9.3, 0.8 Hz, 1H, ImQu-H), 3.82 (m, 2H, ImQu-H), 3.32 (dd, *J* = 6.9, 0.8 Hz, 1H, ImQu-H), 3.15 (d, *J* = 1.5 Hz, 1H, ImQu-H), 1.41 (s, 3H, ImQu-N-$CH_3$); **$^{13}$C NMR** (126 MHz, $C_6D_6$): δ [ppm] = 163.3 (d, $^1J_{C,F}$ = 246.6 Hz, Ar-$C_q$), 152.8 (ImQu-$C_q$), 152.2 (ImQu-$C_q$), 150.3 (ImQu-$C_q$), 136.7 (ImQu-$C_q$), 136.5 (d, $^4J_{C,F}$ = 3.0 Hz, Ar-$C_q$), 135.3 (ImQu-*C*H), 126.6 (d, $^3J_{C,F}$ = 7.8 Hz, Ar-*C*H), 115.4 (d, $^2J_{C,F}$ = 21.3 Hz, Ar-*C*H), 100.1 (ImQu-*C*H), 99.9 (ImQu-*C*H), 98.6 (ImQu-*C*H), 88.0 (ImQu-*C*H), 78.5 (ImQu-*C*H), 30.0 (ImQu-N-$CH_3$); **$^{19}$F NMR** (565 MHz, $C_6D_6$): δ [ppm] = -108.7 (Ar-*F*); **IR** [$cm^{-1}$]: ṽ = 2961, 1625, 1547, 1512, 1470, 1397, 1378, 1302, 1286, 1256, 1220, 1205, 1194, 1156, 1135, 1092, 1066, 1031, 974, 934, 864, 840, 799, 768, 747, 719, 674, 639, 629, 859, 558, 541, 501, 461, 414; **HRMS-ESI(+)** calc. [M+H]$^+$ 265.1100; found 265.1132; **UV-Vis:** $\lambda_{max}$: 429 nm (ε = 7988 $cm^{-1}$ $M^{-1}$), 744 nm (ε =1056 $cm^{-1}$ $M^{-1}$), 833 nm (1054 $cm^{-1}$ $M^{-1}$).

Synthesis of **6e**

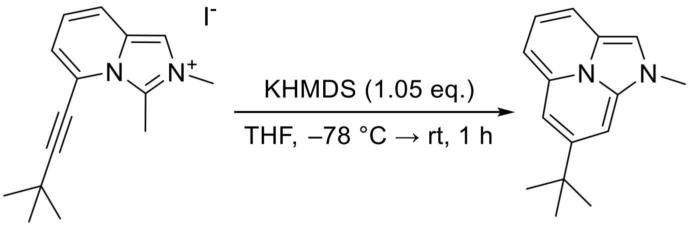

To a mixture of solid **4e** (150 mg, 0.42 mmol, 1.0 eq.) and KHMDS (89.0 mg, 0.45 mmol, 1.05 eq.) was added THF (10 mL) at –78 °C. The mixture instantaneously turned dark blue. After stirring for 5 minutes at –78 °C the solution was warmed to rt and stirred for 1 h, in which time the solution turned dark violet. The solvent was removed under reduced pressure and the remaining solid extracted with $Et_2O$ (2x10 mL). The solvent was removed under reduced pressure, furnishing **6e** (51.0 mg, 0.23 mmol, 54%) as greenish solid.

**m.p.** 90 °C**;** $^{1}$**H NMR** (500 MHz, $C_6D_6$): δ [ppm] = 5.11 (dd, *J* = 9.3, 7.0 Hz, 1H, ImQu-H), 4.05 (dt, *J* = 9.3, 0.6 Hz, 1H, ImQu-H), 3.92 (d, *J* = 1.6 Hz, 1H, ImQu-H), 3.87 (s, *J* = 1.0 Hz, 1H, ImQu-H), 3.38 (dd, *J* = 6.9, 0.9 Hz, 1H, ImQu-H), 3.21 (d, *J* = 1.6 Hz, 1H, ImQu-H), 1.41 (s, *J* = 1.1 Hz, 3H, ImQu-N-$CH_3$), 0.85 (s, 9H, $C(CH_3)_3$); $^{13}$**C NMR** (126 MHz, $C_6D_6$): δ [ppm] = 163.7 (ImQu-$C_q$), 152.7 (ImQu-$C_q$), 150.1 (ImQu-$C_q$), 136.5 (ImQu-$C_q$), 135.5 (ImQu-*C*H), 99.2 (ImQu-*C*H), 98.7 (ImQu-*C*H), 96.4 (ImQu-*C*H), 87.0 (ImQu-*C*H), 77.6 (ImQu-*C*H), 34.1 (ImQu-N-$CH_3$), 34.1 ($C(CH_3)_3$), 29.6 ($C(CH_3)_3$); **IR** [$cm^{-1}$]: ṽ = 2959, 1621, 1546, 1507, 1470, 1403, 1378, 1361, 1280, 1259, 1225, 1200, 1162, 1136, 1117, 1081, 1038, 1025, 974, 906, 822, 778, 748, 724, 711, 679, 640, 594, 539, 497, 476; **HRMS-ESI(+)** calc. $[M+H]^+$ 227.1500; found 227.1547; **UV-Vis:** $λ_{max}$: 396 nm (ε = 3647 $cm^{-1}$ $M^{-1}$), 612 nm (ε = 250 $cm^{-1}$ $M^{-1}$), 678 nm (ε = 533 $cm^{-1}$ $M^{-1}$), 757 nm (ε = 666 $cm^{-1}$ $M^{-1}$), 860 nm (ε = 365 $cm^{-1}$ $M^{-1}$).

Synthesis of **6f**

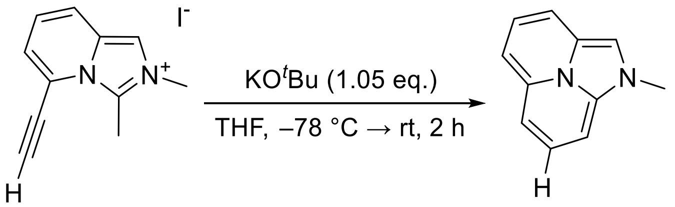


To a precooled (–78 °C) solution of KO*t*Bu (39.5 mg, 352 µmol, 1.05 eq.) in dry THF (30 mL) was added **4f** (100 mg, 335 µmol, 1.0 eq.) at once. The suspension turned purple, then blue and finally completely intransparent while slowly warming up to rt in a cooling bath. The solvent was removed under reduced pressure and the black-purple residue was suspended in toluene (20+10 mL) and filtrated. The solvent was removed under reduced pressure, furnishing **6f** (37.0 mg, 217 µmol, 65%) as sticky black oil.

**m.p.** <25 °C**;** $^{1}$**H NMR** (500 MHz, $C_6D_6$): δ [ppm] = 5.20 (dd, *J* = 8.6, 7.7 Hz, 1H, ImQu-H), 4.93 (dd, *J* = 9.4, 6.9 Hz, 1H, ImQu-H), 3.90 (d, *J* = 9.4 Hz, 1H, ImQu-H), 3.71 – 3.65 (m, 2H, ImQu-H), 3.18 (d, *J* = 6.9 Hz, 1H, ImQu-H), 2.94 (d, *J* = 7.7 Hz, 1H, ImQu-H), 1.25 (s, 3H, ImQu-N-$CH_3$); $^{13}$**C NMR** (126 MHz, $C_6D_6$): δ [ppm] = 153.3 (ImQu-$C_q$), 150.3 (ImQu-$C_q$), 140.3 (ImQu-*C*H), 137.2 (ImQu-$C_q$), 135.4 (ImQu-*C*H), 99.7 (ImQu-*C*H), 99.3 (ImQu-*C*H), 99.3 (ImQu-*C*H), 87.1 (ImQu-*C*H), 79.1 (ImQu-*C*H), 29.5 (ImQu-$CH_3$); **IR** [$cm^{-1}$]: ṽ = 3360, 2921, 2851, 1620,

1551, 1467, 1401, 1375, 1284, 1261, 1194, 1153, 1119, 1035, 798, 691, 542; **HR-MS-ESI(+)** calc. $C_{11}H_{11}N_2^+$ $[M+H]^+$ 171.0917; found 171.0917; **UV-Vis**: $\lambda_{max}$: 353 nm (ε = 4213 $cm^{-1}$ $M^{-1}$), 397 nm (ε = 4228 $cm^{-1}$ $M^{-1}$), 414 nm (ε = 3937 $cm^{-1}$ $M^{-1}$), 489 nm (ε = 860 $cm^{-1}$ $M^{-1}$), 693 nm (ε = 686 $cm^{-1}$ $M^{-1}$), 779 nm (ε = 742 $cm^{-1}$ $M^{-1}$), 892 nm (ε = 379 $cm^{-1}$ $M^{-1}$).

Synthesis of **6g**

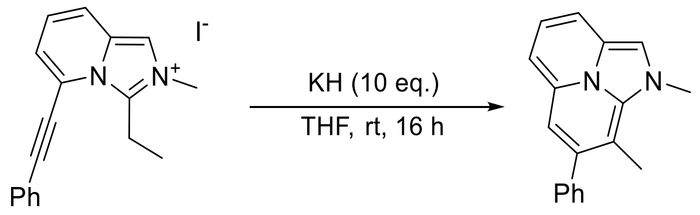


To a mixture of solid **4g** (100 mg, 258 µmol, 1.0 eq.) and KH (103 mg, 2.58 mmol, 10 eq.) was added THF (20 mL) at rt in a nitrogen filled glove box. The mixture turned green-yellow over the course of 1 h. The suspension was stirred overnight. Afterwards, the suspension was filtered over a glass frit with medium porosity, washed twice with THF (2x5 mL) and the solvent of the filtrate was removed under reduced pressure. **6g** (58.0 mg, 223 µmol, 86%) was obtained as a dark-green solid.

**m.p.** 156 °C (decomp.)**;** $^{1}$**H NMR** (600 MHz, $C_6D_6$): δ [ppm] = 7.12 – 7.09 (m, 2H, Ar-H), 7.05 – 7.01 (m, 3H, Ar-H), 5.04 (dd, *J* = 9.2, 7.0 Hz, 1H, ImQu-H), 3.88 (dd, *J* = 9.2, 0.9 Hz, 1H, ImQu-H), 3.70 (s, 1H, ImQu-H), 3.67 (s, 1H, ImQu-H), 3.18 (dd, *J* = 7.1, 0.9 Hz, 1H, ImQu-H), 1.76 (s, 3H, ImQu-$CH_3$), 0.81 (s, 3H, ImQu-N-$CH_3$); $^{13}$**C NMR** (151 MHz, $C_6D_6$): δ [ppm] = 155.7 (ImQu-$C_q$), 152.4 (ImQu-$C_q$), 149.1 (ImQu-$C_q$), 141.2 (Ar-$C_q$), 137.8 (ImQu-$C_q$), 136.1 (ImQu-*C*H), 128.7 (Ar-*C*H), 127.7 (Ar-*C*H), 127.6 (Ar-*C*H), 101.6 (ImQu-*C*H), 101.2 (ImQu-*C*H), 96.8 (ImQu-*C*H), 89.0 (ImQu-$C_q$), 86.0 (ImQu-*C*H), 33.6 (ImQu-$CH_3$), 12.9 (ImQu-N-$CH_3$); **IR** [$cm^{-1}$]: ṽ = 2982, 2857, 1634, 1596, 1544, 1455, 1412, 1374, 1306, 1260, 1207, 1162, 1141, 1098, 1074, 1030, 981, 911, 823, 779, 758, 714, 693, 671, 657, 627, 614, 585, 499, 479; **HR-MS-ESI(+)** calc. $C_{18}H_{17}N_2^+$ $[M+H]^+$ 261.1386; found 261.1381; **UV-Vis**: $\lambda_{max}$: 444 nm (ε = 9205 $cm^{-1}$ $M^{-1}$), 719 nm (ε = 1058 $cm^{-1}$ $M^{-1}$), 802 nm (ε = 1042 $cm^{-1}$ $M^{-1}$).

Synthesis of **6h**

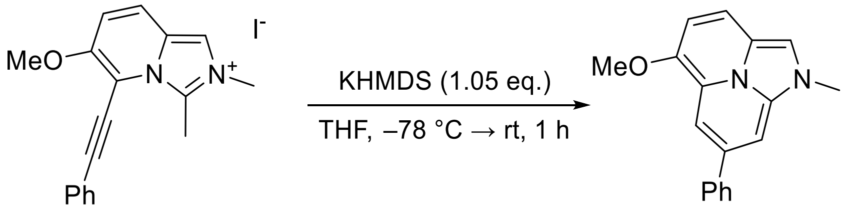


To a mixture of solid **4h** (100 mg, 167 µmol, 1.00 eq.) and KHMDS (56.0 mg, 175 µmol, 1.05 eq.) was added THF (10 mL) at –78 °C. The mixture instantaneously turned green-yellow.

After stirring for 5 minutes at –78 °C the solution was warmed to rt and stirred for 1 h. The solvent was removed under reduced pressure and the remaining solid extracted with toluene (20 + 5 mL) and washed with pentane (10 mL). The solvent was removed under reduced pressure, furnishing **6h** (45.0 mg, 163 µmol, 66%) as green-yellow solid.

**m.p.** 138 °C**;** **$^{1}$H NMR** (600 MHz, $C_6D_6$): δ [ppm] = 7.27 – 7.23 (m, 2H, Ar-H), 7.10 – 7.04 (m, 3H, Ar-H), 4.99 (d, *J* = 9.6 Hz, 1H, ImQu-H), 4.14 (d, *J* = 1.6 Hz, 1H, ImQu-H), 3.76 (d, *J* = 9.8 Hz, 1H, ImQu-H), 3.71 (s, 1H, ImQu-H), 3.07 (d, *J* = 1.6 Hz, 1H, ImQu-H), 3.02 (d, *J* = 0.7 Hz, 3H, ImQu-O-$CH_3$), 1.32 (s, 3H, ImQu-N-$CH_3$); **$^{13}$C NMR** (151 MHz, $C_6D_6$): δ [ppm] = 154.0 (Ar-$C_q$), 151.2 (ImQu-$C_q$), 145.7 (ImQu-$C_q$), 140.5 (ImQu-$C_q$), 136.3 (ImQu-$C_q$), 131.3 (ImQu-*C*H), 128.7 (Ar-*C*H), 128.5 (Ar-*C*H), 128.3 (ImQu-$C_q$), 124.4 (Ar-*C*H), 99.0 (ImQu-*C*H), 98.5 (ImQu-*C*H), 93.6 (ImQu-*C*H), 77.9 (ImQu-*C*H), 57.3 (ImQu-O-$CH_3$), 29.7 (ImQu-N-$CH_3$); **IR** [$cm^{-1}$]: ṽ = 2925, 1634, 1614, 1496, 1466, 1436, 1403, 1370, 1312, 1285, 1240, 1206, 1157, 1085, 1073, 1044, 1022, 991, 778, 752, 722, 698, 646, 622, 599, 543, 506, 490, 463; **HR-MS-ESI(+)** calc. $C_{18}H_{17}N_2O^+$ $[M+H]^+$ 277.1335; found 277.1334; **UV-Vis**: **$\lambda_{max}$**: 445 nm (ε = 5294 $cm^{-1}$ $M^{-1}$), 787 nm (ε = 633 $cm^{-1}$ $M^{-1}$), 888 nm (ε = 676 $cm^{-1}$ $M^{-1}$).

Synthesis of **6i**

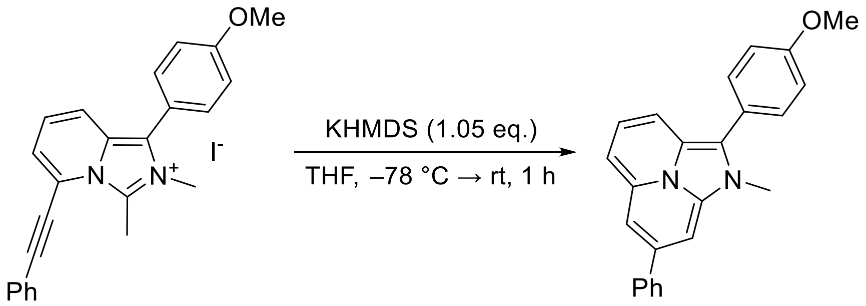


To a mixture of solid **4i** (80.0 mg, 167 µmol, 1.0 eq.) and KHMDS (56.0 mg, 175 µmol, 1.05 eq.) was added THF (10 mL) at –78 °C. The mixture instantaneously turned blue-green. After stirring for 5 minutes at –78 °C the solution was warmed to rt and stirred for 1 h, in which time the solution turned green-yellow. The solvent was removed under reduced pressure and the remaining solid extracted with toluene (2x10 mL) and washed with pentane (10 mL). The solvent was removed under reduced pressure, furnishing **6i** (55.0 mg, 156 µmol, 94%) as light-brown solid, which displays green-yellow color in solution.

**m.p.** 189 °C**;** **$^{1}$H NMR** (500 MHz, $C_6D_6$): δ [ppm] = 7.27 – 7.19 (m, 2H, Ar-H), 7.15 – 7.07 (m, 3H, Ar-H), 6.71 – 6.63 (m, 4H, Ar-H), 5.10 (dd, *J* = 9.3, 6.9 Hz, 1H, ImQu-H), 4.42 (d, *J* = 9.3 Hz, 1H, ImQu-H), 4.14 (d, *J* = 1.5 Hz, 1H, ImQu-H), 3.47 (dd, *J* = 7.0, 0.8 Hz, 1H, ImQu-H), 3.42 (d, *J* = 1.5 Hz, 1H, ImQu-H), 3.30 (s, 3H, Ar-O-$CH_3$), 1.63 (s, 3H, ImQu-N-$CH_3$); **$^{13}$C NMR** (126 MHz, $C_6D_6$): δ [ppm] = 158.0 (Ar-$C_q$), 152.7 (Ar-$C_q$), 152.1 (ImQu-$C_q$), 150.2 (ImQu-$C_q$), 140.0 (ImQu-$C_q$), 135.3 (ImQu-*C*H), 133.2 (ImQu-$C_q$), 128.3 (Ar-*C*H), 128.2 (Ar-*C*H), 126.9

(Ar-*C*H), 124.4 (Ar-*C*H), 121.4 (Ar-*C*$_q$), 114.3 (Ar-*C*H), 111.0 (ImQu-*C*$_q$), 99.2 (ImQu-*C*H), 98.9 (ImQu-*C*H), 89.0 (ImQu-*C*H), 79.4 (ImQu-*C*H), 54.5 (Ar-O-*C*H$_3$), 28.9 (ImQu-N-*C*H$_3$); **IR** [cm$^{-1}$]: ṽ = 2931, 2833, 1621, 1517, 1508, 1457, 1399, 1345, 1299, 1286, 1246, 1206, 1177, 1137, 1043, 763, 688; **HR-MS-ESI(+)** calc. $C_{24}H_{21}N_2O^+$ [M+H]$^+$ 353.1648; found 353.1651; **UV-Vis**: **$\lambda_{max}$**: 474 nm (ε = 23438 cm$^{-1}$ M$^{-1}$), 764 nm (ε = 847 cm$^{-1}$ M$^{-1}$), 850 nm (ε = 840 cm$^{-1}$ M$^{-1}$).

Synthesis of **6j**

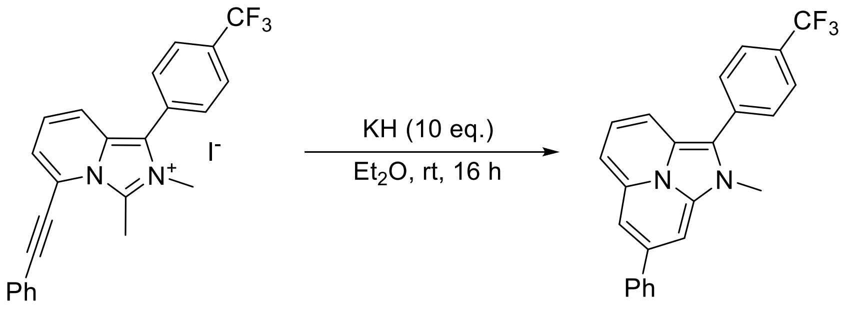


To a mixture of solid **4j** (100 mg, 192 µmol, 1.0 eq.) and KH (77.0 mg, 1.93 mmol, 10 eq.) was added Et$_2$O (20 mL) at rt in a nitrogen filled glove box. The mixture turned purple over the course of 1 h. The suspension was stirred overnight. Afterwards, the suspension was filtered over a glass frit with medium porosity, washed twice with Et$_2$O (2x5 mL) and the solvent of the filtrate was removed under reduced pressure. **6j** (65.0 mg, 166 µmol, 86%) was obtained as a purple solid.

**m.p.** 169 °C**; $^1$H NMR** (600 MHz, $C_6D_6$): δ [ppm] = 7.27 – 7.24 (m, 2H, Ar-H), 7.23 – 7.17 (m, 2H, Ar-H), 7.15 – 7.10 (m, 3H, Ar-H), 6.55 – 6.50 (m, 2H, Ar-H), 5.28 (dd, *J* = 9.2, 7.1 Hz, 1H, ImQu-H), 4.57 (d, *J* = 9.2 Hz, 1H, ImQu-H), 4.33 (d, *J* = 1.5 Hz, 1H, ImQu-H), 3.76 (dd, *J* = 7.1, 0.8 Hz, 1H, ImQu-H), 3.60 (d, *J* = 1.5 Hz, 1H, ImQu-H), 1.60 (s, 3H, ImQu-N-CH$_3$); **$^{13}$C NMR** (151 MHz, $C_6D_6$): δ [ppm] = 152.9 (Ar-*C*$_q$), 151.4 (ImQu-*C*$_q$), 151.1 (ImQu-*C*$_q$), 139.9 (ImQu-*C*$_q$), 137.0 (ImQu-*C*H), 136.1 (ImQu-*C*$_q$), 133.2 (Ar-*C*$_q$), 129.0 (Ar-*C*H), 128.8 (Ar-*C*H), 126.0 (q, *J* = 3.8 Hz, Ar-*C*H), 125.4 (q, *J* = 32.1 Hz, Ar-*C*$_q$), 125.1 (q, *J* = 270.6 Hz, Ar-*C*F$_3$), 125.0 (Ar-*C*H), 123.5 (Ar-*C*H), 110.7 (ImQu-*C*$_q$), 100.6 (ImQu-*C*H), 98.2 (ImQu-*C*H), 92.3 (ImQu-*C*H), 81.4 (ImQu-*C*H), 30.7 (ImQu-N-*C*H$_3$); **$^{19}$F NMR** (565 MHz, $C_6D_6$): δ [ppm] = -61.8 (-C*F*$_3$);**IR** [cm$^{-1}$]: ṽ = 2924, 2853, 1628, 1596, 1523, 1456, 1392, 1320, 1191, 1162, 1135, 1112, 1087, 1064, 879, 834, 751, 697, 524; **HR-MS-ESI(+)** calc. $C_{24}H_{18}F_3N_2^+$ [M+H]$^+$ 391.1417; found 391.1412; **UV-Vis**: **$\lambda_{max:}$** 396 nm (ε = 4264 cm$^{-1}$ M$^{-1}$), 529 nm (ε = 45608 cm$^{-1}$ M$^{-1}$), 740 nm (ε = 1996 cm$^{-1}$ M$^{-1}$), 820 nm (ε = 1934 cm$^{-1}$ M$^{-1}$).

Synthesis of **6k**

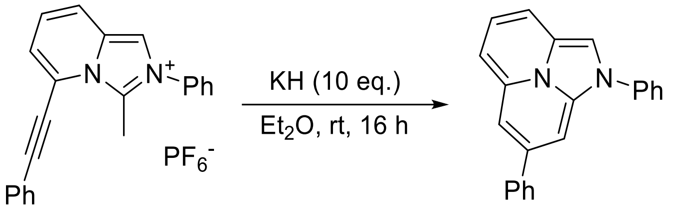

To a mixture of solid **4k** (100 mg, 220 µmol, 1.0 eq.) and KH (88.0 mg, 2.20 mmol, 10 eq.) was added $Et_2O$ (20 mL) at rt in a nitrogen filled glove box. The mixture turned green-yellow over the course of 1 h. The suspension was stirred overnight. Afterwards, the suspension was filtered over a glass frit with medium porosity, washed twice with $Et_2O$ (2x5 mL) and the solvent of the filtrate was removed under reduced pressure. **6k** (60.0 mg, 195 µmol, 88%) was obtained as a light-brown solid, which displays green-yellow color in solution.

**m.p.** 96 °C**;** $^{1}$**H NMR** (600 MHz, $C_6D_6$): δ [ppm] = 7.06 – 7.01 (m, 3H, Ar-H), 7.02 – 6.96 (m, 2H, Ar-H), 6.88 – 6.82 (m, 2H, Ar-H), 6.77 – 6.71 (m, 1H, Ar-H), 6.68 – 6.63 (m, 2H, Ar-H), 5.13 (dd, *J* = 9.3, 6.9 Hz, 1H, ImQu-H), 4.33 (d, *J* = 2.1 Hz, 1H, ImQu-H), 4.19 (d, *J* = 1.5 Hz, 1H, ImQu-H), 4.12 (s, 1H, ImQu-H), 4.11 (d, *J* = 10.2 Hz, 1H, ImQu-H), 3.47 (d, *J* = 6.9 Hz, 1H, ImQu-H); $^{13}$**C NMR** (151 MHz, $C_6D_6$): δ [ppm] = 152.7 (Ar-$C_q$), 152.1 (ImQu-$C_q$), 148.8 (ImQu-$C_q$), 140.0 (ImQu-$C_q$), 137.7 (ImQu-$C_q$), 137.5 (Ar-$C_q$), 135.2 (ImQu-*C*H), 129.6 (Ar-*C*H), 128.6 (Ar-*C*H), 128.6 (Ar-*C*H), 126.0 (Ar-*C*H), 124.9 (Ar-*C*H), 121.4 (Ar-*C*H), 101.4 (ImQu-*C*H), 100.1 (ImQu-*C*H), 98.9 (ImQu-*C*H), 88.6 (ImQu-*C*H), 80.4 (ImQu-*C*H); **IR** [$cm^{-1}$]: ṽ = 2961, 1640, 1618, 1590, 1498, 1464, 1441, 1424, 1404, 1377, 1325, 1313, 1259, 1211, 1191, 1157, 1138, 1068, 1017, 924, 798, 755, 744, 731, 682, 613, 588, 498, 484, 453; **HR-MS-ESI(+)** calc. $C_{22}H_{17}N_2^+$ $[M+H]^+$ 309.1386; found 309.1393; **UV-Vis**: $\boldsymbol{\lambda}_{\mathbf{max}}$: 465 nm (ε = 6816 $cm^{-1}$ $M^{-1}$) 737 nm (ε = 996 $cm^{-1}$ $M^{-1}$) $\lambda_3$ = 819 nm (ε = 1002 $cm^{-1}$ $M^{-1}$).

Synthesis of **6l**

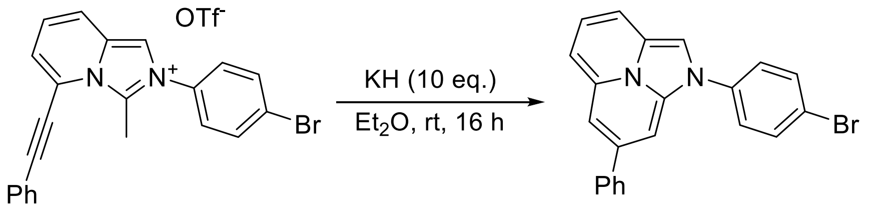

To a mixture of solid **4l** (200 mg, 372 µmol, 1.0 eq.) and KH (149 mg, 3.72 mmol, 10 eq.) was added $Et_2O$ (40 mL) at rt in a nitrogen filled glove box. The mixture turned brown-reddish over the course of 1 h. The suspension was stirred overnight. Afterwards, the suspension was filtered over a glass frit with medium porosity, washed twice with $Et_2O$ (2x5 mL) and the solvent

of the filtrate was removed under reduced pressure. **6l** (141 mg, 364 µmol, 98%) was obtained as a rusty solid, which displays brown-reddish color in solution.

**m.p.** 152 °C (decomp.)**;** **$^1$H NMR** (600 MHz, $C_6D_6$): δ [ppm] = 7.07 (m, 2H, Ar-H), 7.07 – 7.00 (m, 3H, Ar-H), 6.94 – 6.89 (m, 2H, Ar-H), 6.30 – 6.25 (m, 2H, Ar-H), 5.16 (dd, *J* = 9.4, 6.9 Hz, 1H, ImQu-H), 4.24 (d, *J* = 1.5 Hz, 1H, ImQu-H), 4.19 (s, 1H, ImQu-H), 4.14 (d, *J* = 9.3 Hz, 1H, ImQu-H), 4.03 (d, *J* = 1.5 Hz, 1H, ImQu-H), 3.50 (dd, *J* = 7.0, 0.8 Hz, 1H, ImQu-H); **$^{13}$C NMR** (151 MHz, $C_6D_6$): δ [ppm] = 152.0 (Ar-$C_q$), 151.3 (ImQu-$C_q$), 148.0 (ImQu-$C_q$), 139.5 (ImQu-$C_q$), 137.3 (ImQu-$C_q$), 135.9 (Ar-$C_q$), 134.8 (ImQu-*C*H), 132.3 (Ar-*C*H), 128.4 (Ar-*C*H), 128.3 (Ar-*C*H), 124.5 (Ar-*C*H), 122.4 (Ar-*C*H), 118.2 (Ar-$C_q$), 101.7 (ImQu-*C*H), 99.9 (ImQu-*C*H), 98.0 (ImQu-*C*H), 88.5 (ImQu-*C*H), 80.0 (ImQu-*C*H); **IR** [$cm^{-1}$]: ṽ = 3134, 1648, 1621, 1584, 1546, 1493, 1428, 1412, 1329, 1313, 1259, 1190, 1142, 1069, 1030, 818, 801, 765, 740, 705, 687, 649, 636, 574, 517, 503, 481, 454; **HR-MS-ESI(+)** calc. $C_{22}H_{16}BrN_2^+$ $[M+H]^+$ 387.0491; found 387.0488; **UV-Vis**: **$\lambda_{max}$**: 484 nm (ε = 5975 $cm^{-1}$ $M^{-1}$), 730 nm (ε = 1058 $cm^{-1}$ $M^{-1}$), 819 nm (ε = 991 $cm^{-1}$ $M^{-1}$).

Synthesis of **6m**

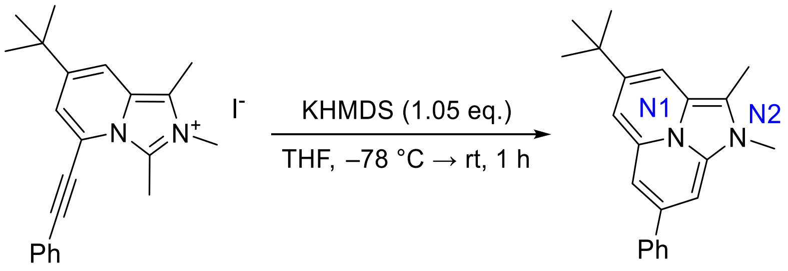


To a mixture of solid **4m** (150 mg, 338 µmol, 1.0 eq.) and KHMDS (71.0 mg, 354 µmol, 1.05 eq.) was added THF (15 mL) at –78 °C. The mixture instantaneously turned turquoise. After stirring for 5 minutes at –78 °C the solution was warmed to rt and stirred for 1 h, in which time the solution turned green-yellow. The solvent was removed under reduced pressure and the remaining solid extracted with $Et_2O$ (3x15 mL). The solvent was removed under reduced pressure, furnishing **6m** (105 mg, 332 µmol, 98%) as dark-green solid, which displays green-yellow color in solution.

**m.p.** 199 °C**;** **$^1$H NMR** (500 MHz, $C_6D_6$): δ [ppm] = 7.35 – 7.28 (m, 2H, Ar-H), 7.12 (m, 3H, Ar-H), 4.17 (d, *J* = 1.6 Hz, 1H, ImQu-H), 4.10 (d, *J* = 1.5 Hz, 1H, ImQu-H), 3.45 (d, *J* = 1.5 Hz, 1H, ImQu-H), 3.43 (d, *J* = 1.5 Hz, 1H, ImQu-H), 1.60 (s, 3H, ImQu-N-$CH_3$), 0.94 (s, 9H, ImQu-$C(CH_3)_3$), 0.91 (s, 3H, ImQu-$CH_3$); **$^1$H NMR** (600 MHz, THF-$d_8$): δ [ppm] = 7.21 – 7.16 (m, 5H, Ar-H), 3.97 (d, *J* = 1.5 Hz, 1H, ImQu-H), 3.91 (d, *J* = 1.6 Hz, 1H, ImQu-H), 3.62 (d, *J* = 1.5 Hz, 1H, ImQu-H), 3.12 (d, *J* = 1.5 Hz, 1H, ImQu-H), 2.55 (s, 3H, ImQu-$CH_3$), 1.44 (s, 3H, ImQu-N-$CH_3$), 0.79 (s, 9H, ImQu-$C(CH_3)_3$); **$^{13}$C NMR** (126 MHz, $C_6D_6$): δ [ppm] = 155.5 (ImQu-$C_q$),

152.4 (Ar-$C_q$), 151.3 (ImQu-$C_q$), 148.8 (ImQu-$C_q$), 141.0 (ImQu-$C_q$), 131.4 (ImQu-$C_q$), 128.6 (Ar-*C*H), 128.4 (Ar-*C*H), 124.8 (Ar-*C*H), 104.7 (ImQu-$C_q$), 98.6 (ImQu-*C*H), 95.0 (ImQu-*C*H), 86.2 (ImQu-*C*H), 77.2 (ImQu-*C*H), 33.7 (ImQu-$C(CH_3)_3$), 29.0 (ImQu-$C(CH_3)_3$), 26.9 (ImQu-N-$CH_3$), 6.6 (ImQu-$CH_3$); **$^{13}$C NMR** (151 MHz, THF-$d_8$): δ [ppm] = 155.8 (ImQu-$C_q$), 152.8 (Ar-$C_q$), 151.7 (ImQu-$C_q$), 149.8 (ImQu-$C_q$), 141.2 (ImQu-$C_q$), 131.9 (ImQu-$C_q$), 128.9 (Ar-*C*H), 128.9 (Ar-*C*H), 125.1 (Ar-*C*H), 106.0 (ImQu-$C_q$), 98.3 (ImQu-*C*H), 95.5 (ImQu-*C*H), 86.2 (ImQu-*C*H), 77.3 (ImQu-*C*H), 34.2 (ImQu-$C(CH_3)_3$), 29.2 (ImQu-$C(CH_3)_3$), 28.0 (ImQu-$CH_3$), 7.0 (ImQu-N-$CH_3$); **$^{15}$N NMR** (61 MHz, THF-$d_8$): δ [ppm] = 184.5 (N1), 116.7 (N2); **IR** [$cm^{-1}$]: ṽ = 2979, 2948, 2898, 2862, 1655, 1620, 1565, 1519, 1466, 1438, 1337, 1313, 1289, 1260, 1207, 1193, 1087, 1072, 1030, 1014, 993, 775, 751, 743, 702, 688, 673, 618, 600; **HR-MS-ESI(+)** calc. $C_{22}H_{25}N_2^+$ [M+H]$^+$ 317.2012; found 317.2017; **UV-Vis**: **$\lambda_{max}$**: 432 nm (ε = 9525 $cm^{-1}$ $M^{-1}$), 744 nm (ε = 983 $cm^{-1}$ $M^{-1}$), 814 nm (ε = 934 $cm^{-1}$ $M^{-1}$).

Synthesis of **6n**

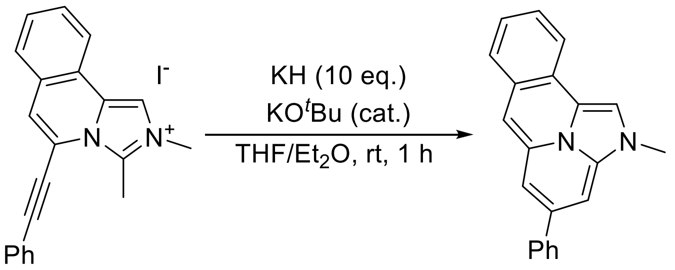


To a suspension of **4n** (82.0 mg, 193 µmol, 1.0 eq.) and KH (78.0 mg, 1.93 mmol, 10 eq.) in a THF/$Et_2O$ mixture (1:1, 40 mL), catalytic amounts of KO*t*Bu were added. The reaction mixture slowly turned dark-green over the course of 1 hour. Excess KH and KI were filtered off and the solvent was removed under reduced pressure to yield **6n** (42.0 mg, 142 µmol, 73%) as a dark-green solid.

**m.p.** 166 °C (decomp.)**;** **$^1$H NMR** (700 MHz, $C_6D_6$): δ [ppm] = 7.39 – 7.34 (m, 2H, Ar-H), 7.18 – 7.10 (m, 3H, Ar-H), 6.61 (td, *J* = 7.5, 1.3 Hz, 1H, ImQu-H), 6.33 (td, *J* = 7.5, 1.1 Hz, 1H, ImQu-H), 6.24 – 6.20 (m, 2H, ImQu-H), 4.81 (d, *J* = 1.5 Hz, 1H, ImQu-H), 4.68 (s, 1H, ImQu-H), 4.18 (s, 1H, ImQu-H), 3.88 (d, *J* = 1.5 Hz, 1H, ImQu-H), 1.74 (s, 3H, ImQu-N-$CH_3$); **$^{13}$C NMR** (176 MHz, $C_6D_6$): δ [ppm] = 149.3 (Ar-$C_q$), 147.8 (ImQu-$C_q$), 145.6 (ImQu-$C_q$), 141.1 (ImQu-$C_q$), 140.9 (ImQu-$C_q$), 130.3 (ImQu-*C*H), 129.5 (ImQu-$C_q$), 128.7 (Ar-*C*H), 128.4 (Ar-*C*H), 125.6 (Ar-*C*H), 123.4 (ImQu-*C*H), 122.0 (ImQu-*C*H), 121.4 (ImQu-*C*H), 121.2 (ImQu-$C_q$), 105.6 (ImQu-*C*H), 99.5 (ImQu-*C*H), 86.2 (ImQu-*C*H), 75.5 (ImQu-*C*H), 30.4 (ImQu-N-$CH_3$); **IR** [$cm^{-1}$]: ṽ = 2926, 2852, 1636, 1618, 1598, 1506, 1458, 1443, 1430, 1340, 1286, 1141, 1065, 1040, 945, 799, 766, 746, 696; **HR-MS-ESI(+)** calc. $C_{21}H_{17}N_2^+$ [M+H]$^+$ 297.1386; found 297.1379; **UV-Vis**: **$\lambda_{max}$**: 254 nm (ε = 7647 $cm^{-1}$ $M^{-1}$), 300 nm (ε = 9942 $cm^{-1}$ $M^{-1}$), 403 nm

(ε = 4353 cm$^{-1}$ M$^{-1}$), 494 nm (ε = 2521 cm$^{-1}$ M$^{-1}$), 620 nm (ε = 707 cm$^{-1}$ M$^{-1}$), 669 nm (ε = 737 cm$^{-1}$ M$^{-1}$).

Synthesis of **6o**

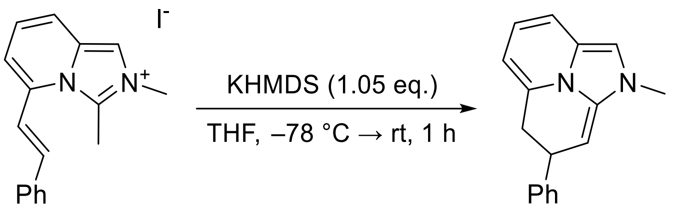


To a mixture of solid **4o** (100 mg, 0.27 mmol, 1.0 eq.) and KHMDS (56.0 mg, 0.28 mmol, 1.05 eq.) was added THF (25 mL) at –78 °C. The mixture instantaneously turned dark blue. After stirring for 5 minutes at –78 °C the solution was warmed to rt and stirred for 1 h, in which time the solution turned dark violet. The solvent was removed under reduced pressure and the remaining solid extracted with $Et_2O$ (2x10 mL). The solvent was removed under reduced pressure, furnishing **6o** (58.0 mg, 0.50 mmol, 91%) as dark violet solid.

**m.p.** 105 °C**;** $^{1}$**H NMR** (600 MHz, $C_6D_6$): δ [ppm] = 7.34 – 7.28 (m, 2H, Ar-H), 7.23 – 7.17 (m, 2H, Ar-H), 7.12 – 7.06 (m, 1H, Ar-H), 6.10 (dd, *J* = 9.6, 1.0 Hz, 1H, ImQu-H), 5.82 (dd, *J* = 9.6, 6.1 Hz, 1H, ImQu-H), 5.28 (s, 1H, ImQu-H), 4.95 (dd, *J* = 6.1, 1.2 Hz, 1H, ImQu-H), 3.79 (dd, *J* = 8.3, 5.5 Hz, 1H, Ph-C*H*-), 3.44 (s, 1H, ImQu=C*H*-), 2.69 – 2.39 (m, 5H, ImPy-N-C*H*$_3$ and -C*H*$_2$-).; $^{13}$**C NMR** (151 MHz, $C_6D_6$): δ [ppm] = 148.2 (Ar-*C*$_q$), 141.6 (ImQu-*C*$_q$), 136.8 (ImQu-*C*$_q$), 127.9 (Ar-*C*H), 127.0 (Ar-*C*H), 125.5 (Ar-*C*H), 124.3 (ImQu-*C*$_q$), 121.8 (ImQu-*C*H), 113.6 (ImQu-*C*H), 100.5 (ImQu-*C*H), 100.3 (ImQu-*C*H), 58.7 (ImQu=*C*H-), 37.8 (-*C*H$_2$-), 35.3 (Ph-*C*H-), 31.1 (ImQu-N-*C*H$_3$); **IR** [cm$^{-1}$]: ṽ = 3060, 3022, 2931, 2889, 2801, 1650, 1600, 1527, 1490, 1433, 1411, 1366, 1325, 1293, 1259, 1201, 1158, 1067, 1023, 952, 808, 745, 698, 662, 624, 587, 556, 498, 463; **HR-MS-ESI(+)** calc. $C_{17}H_{17}N_2^+$ [M+H]$^+$ 249.1386; found 249.1398; **UV-Vis**: **λ$_{max}$**: 341 nm (ε = 23117 cm$^{-1}$ M$^{-1}$), 523 nm (ε = 4285 cm$^{-1}$ M$^{-1}$).

Synthesis of **6p**

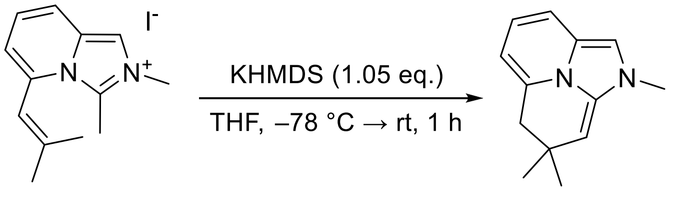


To a mixture of solid **4p** (50 mg, 152 µmol, 1.0 eq.) and KHMDS (32.0 mg, 160 µmol, 1.05 eq.) was added THF (10 mL) at –78 °C. The mixture instantaneously turned dark violet. After stirring for 5 minutes at –78 °C the solution was warmed to rt and stirred for 1 h. The solvent was removed under reduced pressure and the remaining solid extracted with $Et_2O$ (2x5 mL).

The solvent was removed under reduced pressure, furnishing **6p** (26.0 mg, 130 µmol, 85%) as dark violet solid.

**m.p.** 95 °C (decomp.)**;** **$^{1}$H NMR** (500 MHz, $C_6D_6$): δ [ppm] = 6.08 (dd, *J* = 9.6, 1.0 Hz, 1H, ImQu-H), 5.85 (dd, *J* = 9.6, 6.0 Hz, 1H, ImQu-H), 5.25 (s, 1H, ImQu-H), 5.05 (dd, *J* = 6.0, 1.2 Hz, 1H, ImQu-H), 3.30 (s, 1H, ImQu=CH), 2.43 (s, 3H, ImQu-N-$CH_3$), 2.14 (s, 2H, ImQu-C$H_2$-C($CH_3$)$_2$-), 1.17 (s, 6H, ImQu-$CH_2$-C(C$H_3$)$_2$-); **$^{13}$C NMR** (126 MHz, $C_6D_6$): δ [ppm] = 141.4 (ImQu-$C_q$), 138.2 (ImQu-$C_q$), 125.2 (ImQu-$C_q$), 122.8 (ImQu-*C*H), 114.3 (ImQu-*C*H), 101.2 (ImQu-*C*H), 101.0 (ImQu-*C*H), 66.6 (ImQu=*C*H), 42.0 (ImQu-*C*$H_2$-C($CH_3$)$_2$-), 32.1 (ImQu-$CH_2$-C(*C*$H_3$)$_2$-), 31.9 (ImQu-N-*C*$H_3$), 31.0 (ImQu-$CH_2$-*C*($CH_3$)$_2$-); **IR** [cm$^{-1}$]: ṽ = 2952, 2928, 2854, 1655, 1529, 1434, 1413, 1324, 1292, 1254, 1235, 1153, 1068, 1052, 747, 745; **HR-MS-ESI(+)** calc. $C_{13}H_{17}N_2^+$ [M+H]$^+$ 201.1386; found 201.1383; **UV-Vis**: **$\lambda_{max}$**: 256 nm (ε = 10587 cm$^{-1}$ M$^{-1}$), 345 nm (ε = 6975 cm$^{-1}$ M$^{-1}$), 525 nm (ε = 1236 cm$^{-1}$ M$^{-1}$), 554 nm (ε = 1197 cm$^{-1}$ M$^{-1}$).

## 2.6 Miscellaneous Synthesis

Synthesis of **3a**

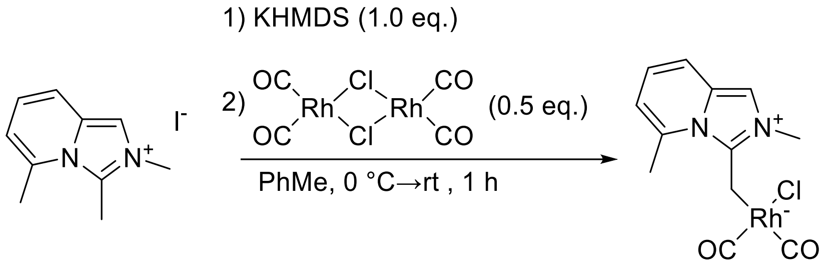


To a mixture of **1a** (75.0 mg, 260 µmol, 1.0 eq.) and KHMDS (51.9 mg, 260 µmol, 1.0 eq.) was added THF (10 ml) at –78 °C. The mixture was stirred for 10 min at this temperature until it was allowed to warm up to 0 °C and stirred for an additional hour at this temperature. The solvent was evaporated and the red residue extracted twice with pentane (2x10 mL), utilizing a filter cannula and maintaining a temperature of 0 °C. The solution was evaporated at 0 °C and the remaining red solid redissolved in toluene (5 mL). This solution was added dropwise to a solution of $[Rh(CO)_2Cl]_2$ in toluene (2 mL) at 0 °C. After complete addition the mixture was stirred for 15 min 0 °C and one additional hour at room temperature. A off white to light beige precipitate formed after complete addition and became more voluminous over the rection time. To this suspension pentane (10 mL) was added, leading to the formation of more, slightly yellow precipitate. The formed solid was filtered off over a short celite pad, eluted with $CH_2Cl_2$ (5 mL), the solvent was evaporated and the filtration step repeated to ensure a nanoparticle-free product is obtained. After the second filtration step 90% of the solvent was removed in vacuo and the product precipitated by the addition of pentane (15 mL). The product was collected by filtration and dried under reduced pressure to furnish **3a** as a light-yellow solid (51 mg, 144 µmol, 55%).

**m.p.** 139.9 °C (decomp.); **$^1$H NMR** (500 MHz, $CD_2Cl_2$): δ [ppm] = 7.09 (d, $J$ = 9.2 Hz, 1H, ImPy-H), 7.07 (s, 1H, ImPy-H), 6.67 (dd, $J$ = 9.3, 6.6 Hz, 1H, ImPy-H), 6.29 (dt, $J$ = 6.6, 1.2 Hz, 1H), 3.77 (s, 3H, ImPy-N-$CH_3$), 3.10 (d, $J$ = 1.0 Hz, 3H, ImPy-$CH_3$), 2.71 (d, $J$ = 2.7 Hz, 2H, ImPy-$CH_2$-[Rh]); **$^{13}$C NMR** (126 MHz, $CD_2Cl_2$): δ [ppm] = 186.2 (d, $J$ = 78.3 Hz, Rh-*C*O), 184.9 (d, $J$ = 57.2 Hz, Rh-CO), 154.8 (ImPy-$C_q$), 136.3 (ImPy-$C_q$), 129.3 (ImPy-$C_q$), 123.0 (ImPy-*C*H), 116.1 (ImPy-*C*H), 115.5 (ImPy-*C*H), 109.7 (ImPy-*C*H), 36.5 (ImPy-N-$CH_3$), 22.6 (ImPy-$CH_3$), 13.3 (d, $J$ = 17.4 Hz, ImPy-$CH_2$-Rh); **IR** (ATR)[$cm^{-1}$]: ṽ = 2976, 2039, 1956, 1655, 1575, 1554, 1506, 1473, 1445, 1414, 1398, 1381, 1336, 1318, 1264, 1202, 1175, 1144, 1077, 1043, 1019, 971, 910, 788, 731, 708, 666, 609, 587, 566, 519, 477, 455, 425; **IR** (1 mg/mL in $CH_2Cl_2$)[$cm^{-1}$]: ṽ = 2057.38, 1975.20; **HR-MS-ESI(+)** calc. $C_{12}H_{12}N_2O_2Rh^+$ $[M-Cl]^+$ 318.9954; found 318.9952.

Synthesis of **3c**

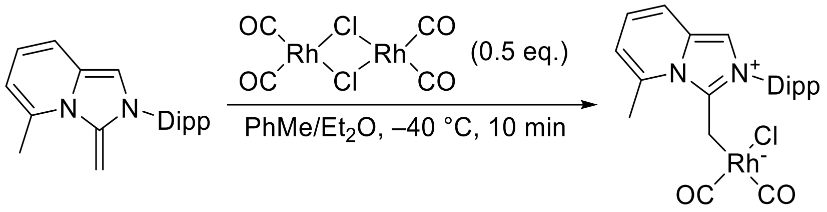


**2c** (50 mg, 163 µmol, 1.0 eq) was dissolved in toluene (5 mL) and added dropwise to a solution of $[Rh(CO)_2Cl]_2$ (24.3 mg, 81.6 µmol, 1.0 eq.) in toluene (2 mL) at –40 °C. The solution was warmed up to room temperature and stirred for 10 minutes. $Et_2O$ (10 mL) was added, the mixture cooled to –40 °C and filtered off cold. The crude product was eluted with $CH_2Cl_2$ (5 mL) and crystallized by vapor diffusion with pentane at –40 °C. The product **3c** was obtained as a light yellow microcrystalline solid (46 mg, 92 µmol, 56 %).

**Note**: CH signals of both isopropyl groups and the coordinating $CH_2$ group are not visible at 25 °C in the $^1$H spectrum (and $^{13}$C spectrum for isopropyl), but they are observable at –20 °C as broad signals.

**m.p.** 177 °C**;** $^1$**H NMR** (500 MHz, $CD_2Cl_2$): δ [ppm] = 7.56 (t, *J* = 7.8 Hz, 1H, Ar-H), 7.37 (d, *J* = 7.8 Hz, 2H, Ar-H), 7.17 (d, *J* = 9.1 Hz, 1H, ImPy-H), 7.01 (s, 1H, ImPy-H), 6.77 (dd, *J* = 9.3, 6.6 Hz, 1H, ImPy-H), 6.37 (dt, *J* = 6.7, 1.2 Hz, 1H, ImPy-H), 3.25 (s, 3H, ImPy-$CH_3$), 2.87 (m, 2H, ImPy-$CH_2$-Rh), 2.07 (m, 1H, C*H*($CH_3$)$_2$), 1.96 (m, 1H, C*H*($CH_3$)$_2$), 1.36 (s, 6H, CH(C$H_3$)$_2$), 1.09 (d, *J* = 6.8 Hz, 6H, CH(C$H_3$)$_2$); $^{13}$**C NMR** (126 MHz, $CD_2Cl_2$): δ [ppm] = 186.0 (d, *J* = 78.3 Hz, Rh-*C*O), 185.1 (d, *J* = 57.7 Hz, Rh-*C*O), 156.7 (ImPy-$C_q$), 146.4 (Dipp-$C_q$), 137.8 (ImPy-$C_q$), 131.7 (Dipp-$C_q$), 131.5 (ImPy-$C_q$), 130.1 (Dipp-*C*H), 125.3 (Dipp-*C*H), 124.0 (ImPy-*C*H), 116.3 (ImPy-*C*H), 115.6 (ImPy-*C*H), 110.2 (ImPy-*C*H), 28.8 (ImPy-*C*$H_2$-Rh), 25.5 (CH(*C*$H_3$)$_2$), 23.8 (CH(*C*$H_3$)$_2$), 23.4 (ImPy-*C*$H_3$); **IR** (ATR)[cm$^{-1}$]: ṽ = 2961.2, 2039.8 (C=O), 1953.3 (C=O), 1657.9, 1554.6, 1468.4, 1408.9, 1299.7, 1178.5, 991.2, 809.4, 780.0, 760.5, 730.6, 688.9, 600.1, 585.8, 542.8, 532.1, 491.8, 452.07; **IR** (1 mg/mL in $CH_2Cl_2$)[cm$^{-1}$]: ṽ = 2056.21, 1973.76; **HR-MS-ESI(+)** calc. $[M-Cl]^+$ 465.1044; found 465.1051.

Synthesis of **5b-Au**

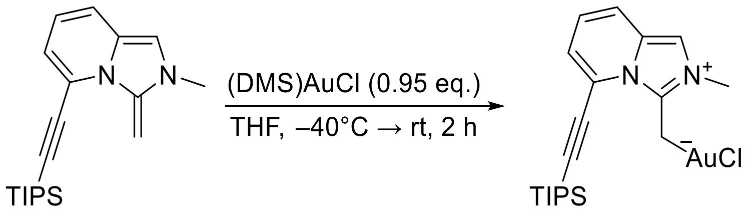


In a nitrogen filled glovebox, (DMS)AuCl (65.1 mg, 221 µmol, 0.95 eq., in 3 mL THF) was added dropwise to a solution of **5b** (76 mg, 233 µmol, 1.0 eq.) in THF (1 mL) at –40 °C, leading

to a slow color change from dark-blue to yellow and a precipitate to crash out. After thawing the solution up over 2 h, pentane (6 mL) was added to fully complete the precipitation and the suspension was then filtered over a Celite loaded pipette. The solid was then washed with $Et_2O$ (2x2 mL), pentane (2x2 mL) and dissolved again in $CH_2Cl_2$ (2x2 mL). After removal of the solvent under reduced pressure, **5b-Au** (80.0 mg, 143 µmol, 65% relative to Au precursor) was obtained as a yellow solid. Crystals suitable for X-ray diffraction could be obtained by vapor diffusion of pentane into a saturated solution of **5b-Au** in 1,2-dichloroethane.

**m.p.** 178 °C (decomp.)**;** $^{1}$**H NMR** (500 MHz, $CD_2Cl_2$): δ [ppm] = 7.69 (s, 1H, ImPy-H), 7.36 (dd, *J* = 9.4, 1.2 Hz, 1H, ImPy-H), 6.84 (dd, *J* = 6.8, 1.2 Hz, 1H, ImPy-H), 6.67 (dd, *J* = 9.3, 6.8 Hz, 1H, ImPy-H), 3.83 (s, 3H, ImPy-N-$CH_3$), 2.95 (s, 2H, ImPy-$CH_2$-AuCl), 1.18 – 1.06 (m, 21H, Si[(CH($CH_3$)$_2$)$_3$]; $^{13}$**C NMR** (126 MHz, $CD_2Cl_2$): δ [ppm] = 153.0 (ImPy-$C_q$), 127.8 (ImPy-$C_q$), 124.7 (ImPy-*C*H), 121.5 (ImPy-*C*H), 119.6 (ImPy-*C*H), 119.1 (ImPy-$C_q$), 111.6 (ImPy-*C*H), 105.6 (-C≡C-), 99.2 (-C≡C-), 35.4 (ImPy-N-*C*$H_3$), 20.1 (ImPy-*C*$H_2$-AuCl), 18.9 (Si[(CH(*C*$H_3$)$_2$)$_3$]), 11.7 (Si[(*C*H($CH_3$)$_2$)$_3$]); **IR** [$cm^{-1}$]: ṽ = 2941, 2890, 2864, 2140, 1640, 1521, 1504, 1463, 1417, 1383, 1364, 1312, 1257, 1181, 1141, 1072, 1018, 996, 914, 882, 796, 669, 584, 524, 491, 460, 443, 413; **HR-MS-ESI(+)** calc. $C_{20}H_{30}AuN_2Si^+$ $[M-Cl]^+$ 523.1838; found 523.1837.

Synthesis of **6m**$^{+\cdot}$

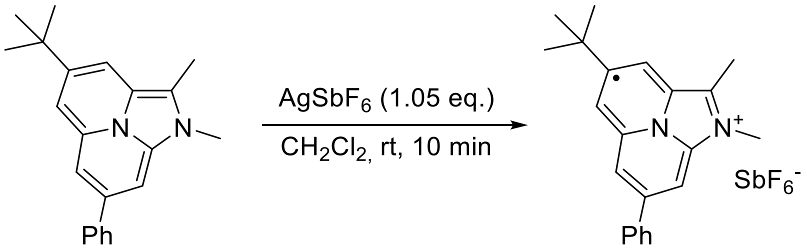


To a solution of **6m** (50.0 mg, 158 µmol, 1.0 eq., 3 mL of $CH_2Cl_2$) was added a solution of $AgSbF_6$ (57.0 mg, 166 µmol, 1.05 eq., 3 mL of $CH_2Cl_2$) dropwise. The solution immediately discolored from green-yellow to green-brown. After stirring the suspension for 10 min, the silver suspension was filtered over a Celite loaded frit and the solvent was reduced to a minimum volume (~2 mL) under reduced pressure. The solution was then triturated by addition of $Et_2O$ (10 mL), causing the product to crash out. After filtration, the residue was washed with pentane (4x2 mL) and dried under reduced pressure to yield **6m**$^{+\cdot}$(54.0 mg, 97.8 µmol, 62%) as green solid.

**m.p.** 287 °C**; EPR**: g = 2.0024 (1xN: 2.3921 MHz; 1xN: 4.2379 MHz; 1xH: 16.7178 MHz; 1xH: 11.0426 MHz, 1xH: 18.6327 MHz, 1xH: 23.9323 MHz, 3xH: 17.9542 MHz, 3xH: 4.7849 MHz); **IR** [$cm^{-1}$]: ṽ = 2965, 1651, 1558, 1504, 1465, 1436, 1370, 1254, 1178, 1030, 925, 879, 835,

768, 694, 654, 574; **HR-MS-ESI(+)** calc. $C_{22}H_{23}N_2^+$ [M-H]$^+$ 315.1856; found 315.1853; **UV-Vis**: **$\lambda_{max}$**: 387 nm ($\varepsilon$ = 6646 cm$^{-1}$ M$^{-1}$), 584 nm ($\varepsilon$ = 368 cm$^{-1}$ M$^{-1}$), 711 nm ($\varepsilon$ = 286 cm$^{-1}$ M$^{-1}$).

## 3. NMR Spectra

### 3.1 NMR Spectra of Isolated Compounds

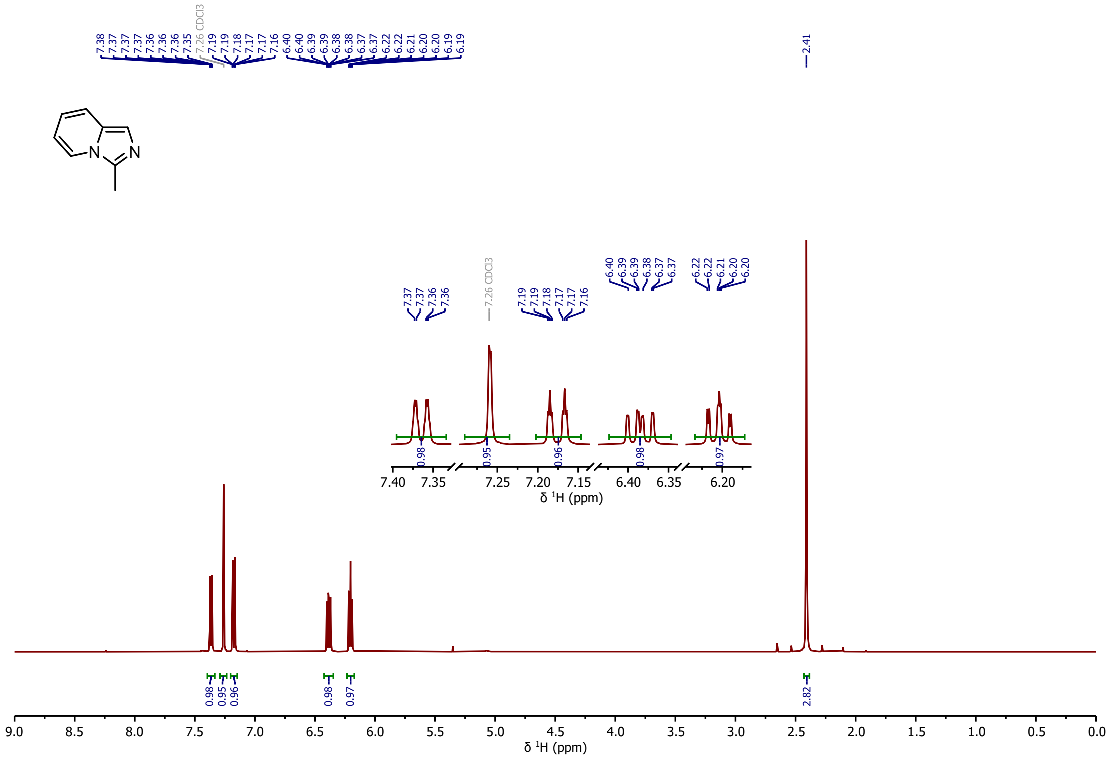


**Figure S1**: **¹H NMR** (500 MHz, $CDCl_3$, 298 K) of **S2.**

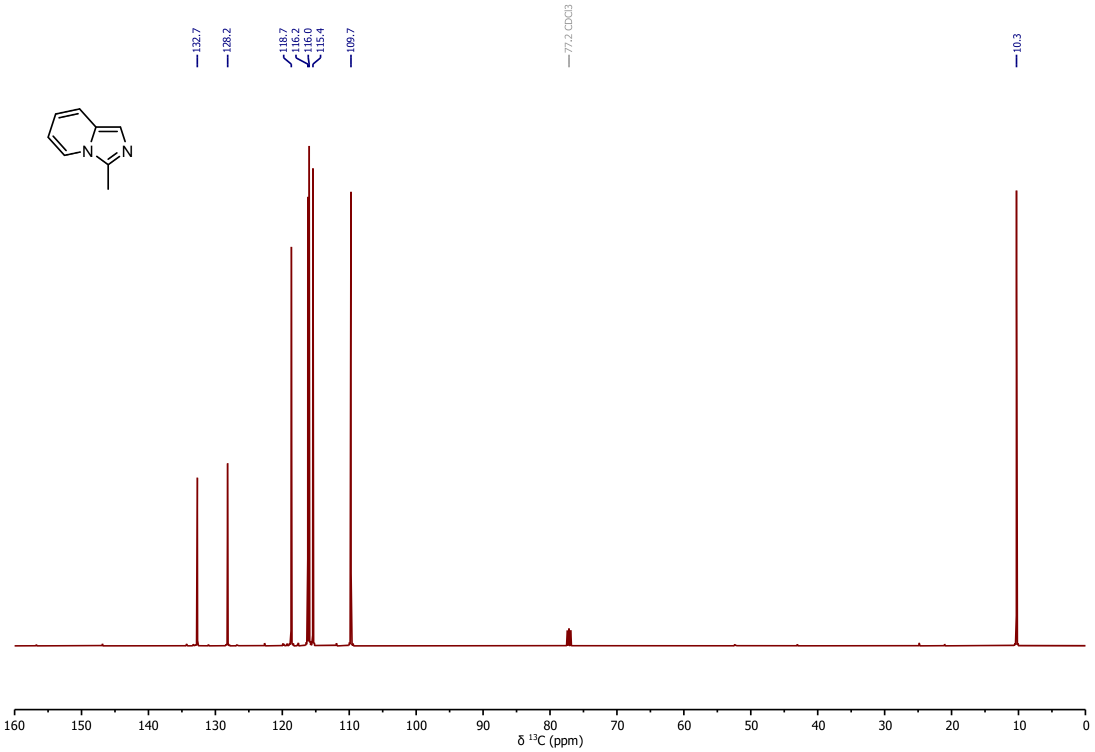


**Figure S2**: **¹³C NMR** (126 MHz, $CDCl_3$, 298 K) of **S2**.

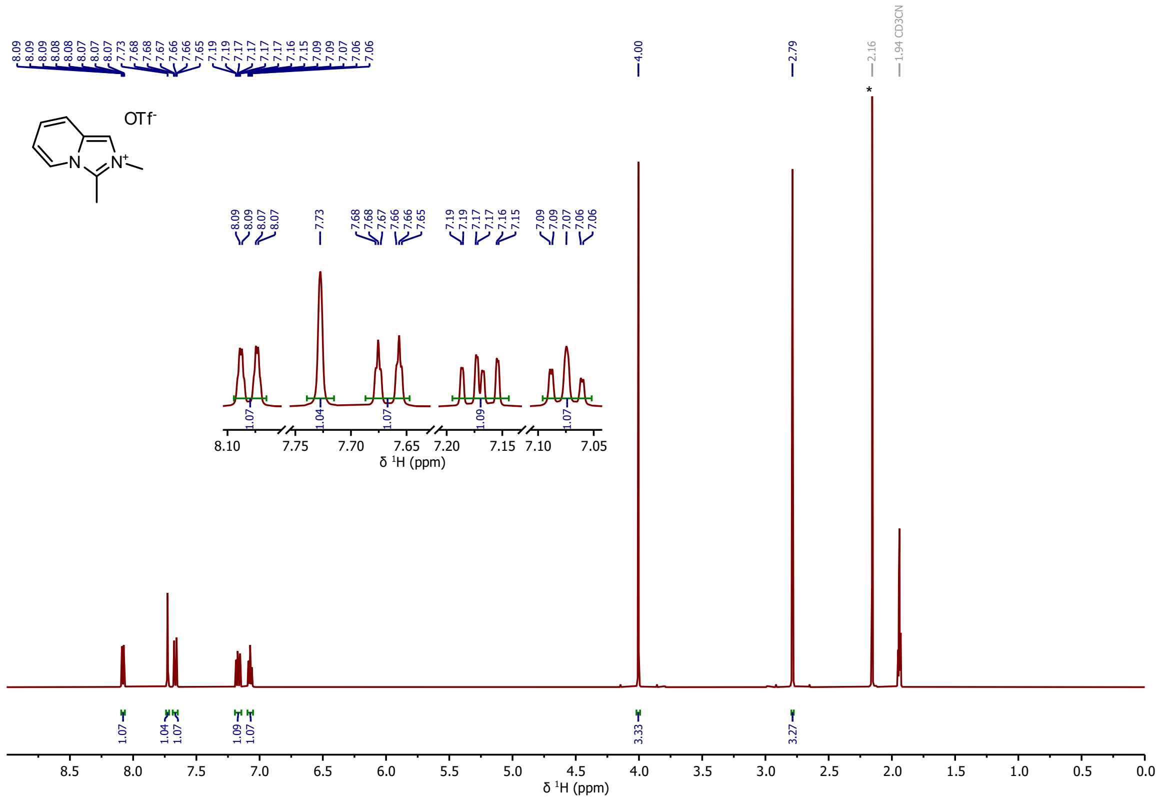

**Figure S3**: **$^1$H NMR** (500 MHz, $CD_3CN$, 298 K) of **1a**. * = $H_2O$.

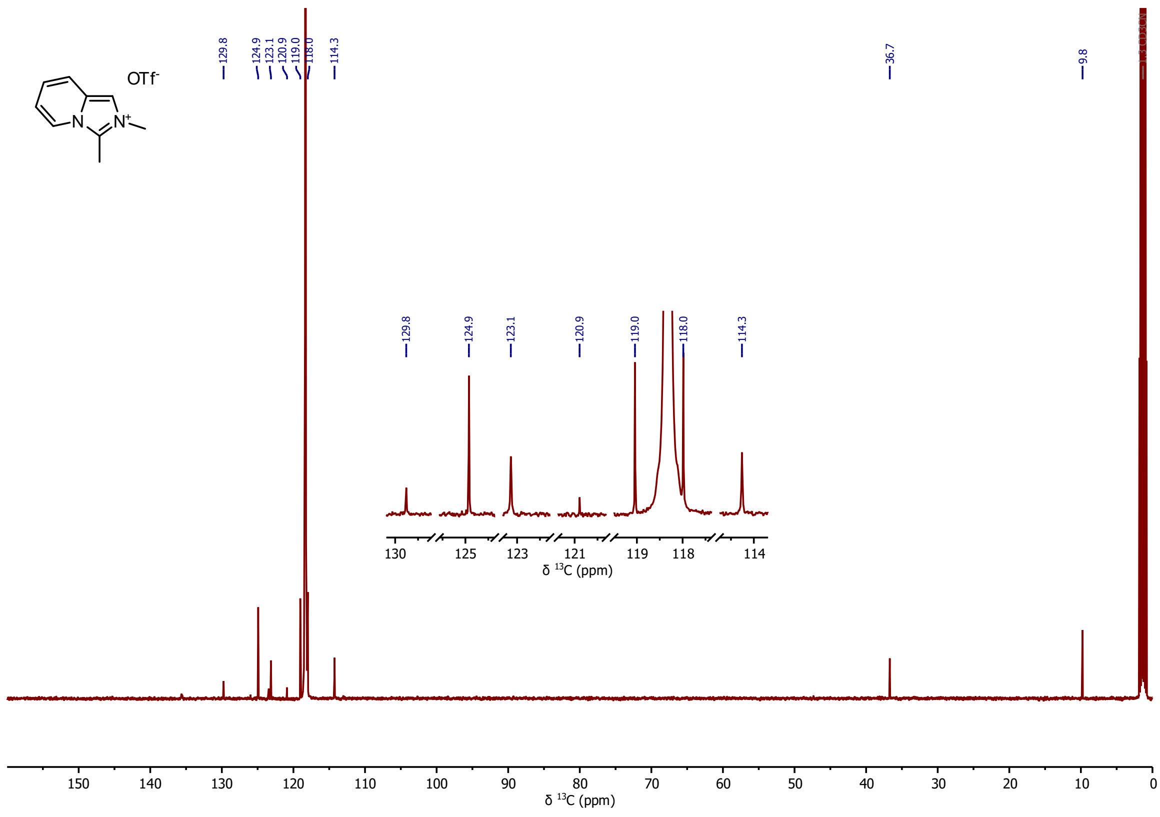

**Figure S4**: **$^{13}$C NMR** (126 MHz, $CD_3CN$, 298 K) of **1a**.

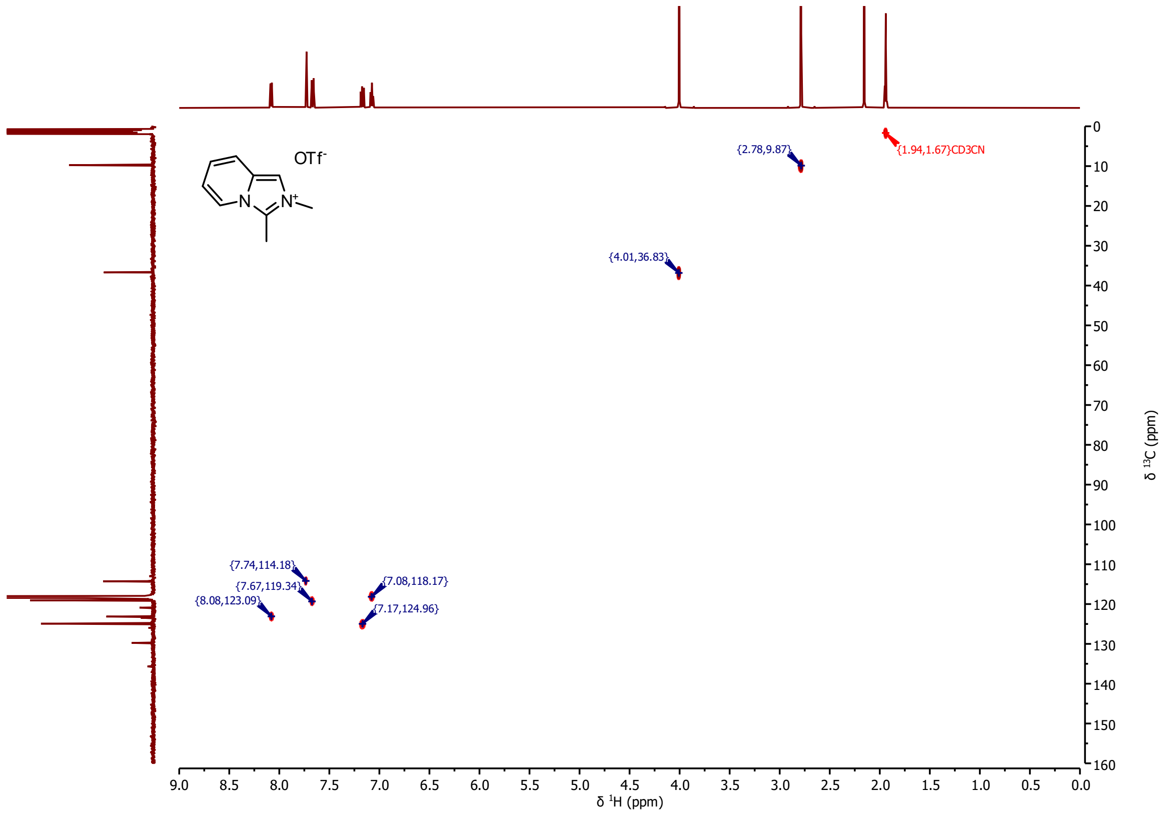


**Figure S5**: **$^{1}$H/$^{13}$C HSQC** (500/126 MHz, $CD_3CN$, 298 K) of **1a**.

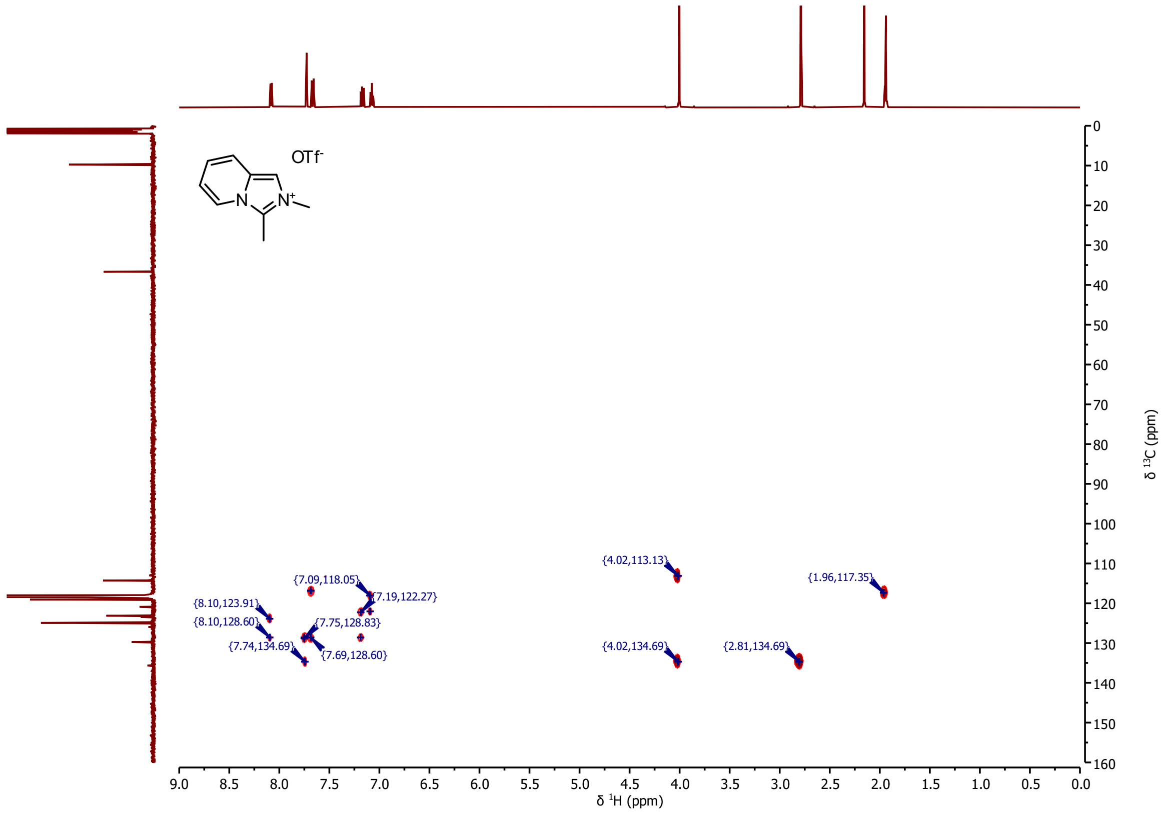


**Figure S6**: **$^{1}$H/$^{13}$C HMBC** (500/126 MHz, $CD_3CN$, 298 K) of **1a**.

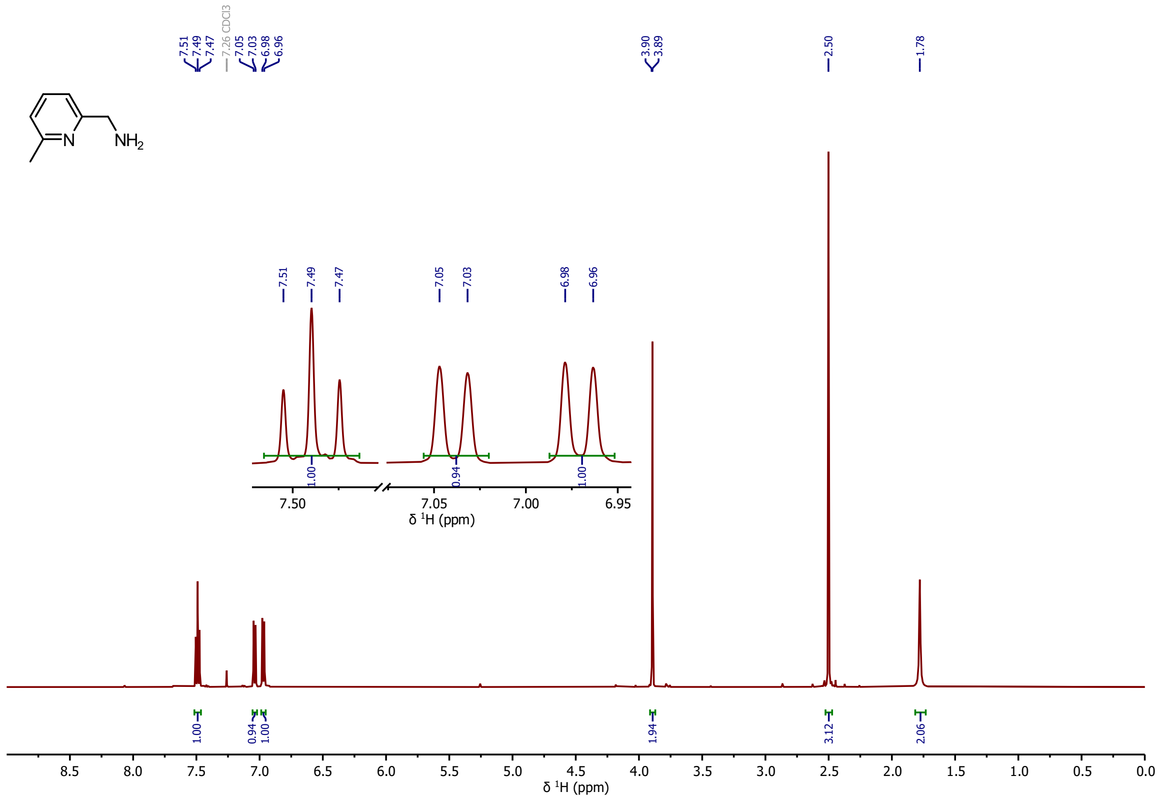


**Figure S7**: **$^{1}$H NMR** (500 MHz, $CDCl_3$, 298 K) of **S4**.

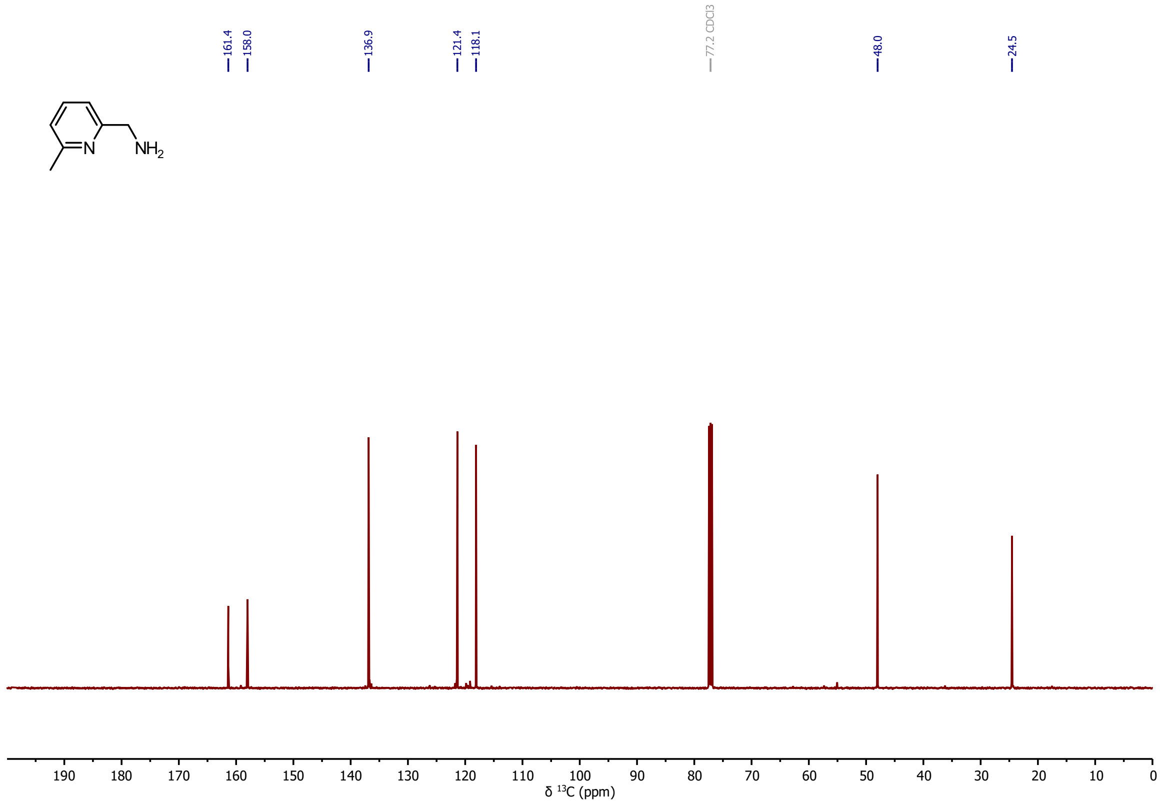


**Figure S8**: **$^{13}$C NMR** (126 MHz, $CDCl_3$, 298 K) of **S4**.

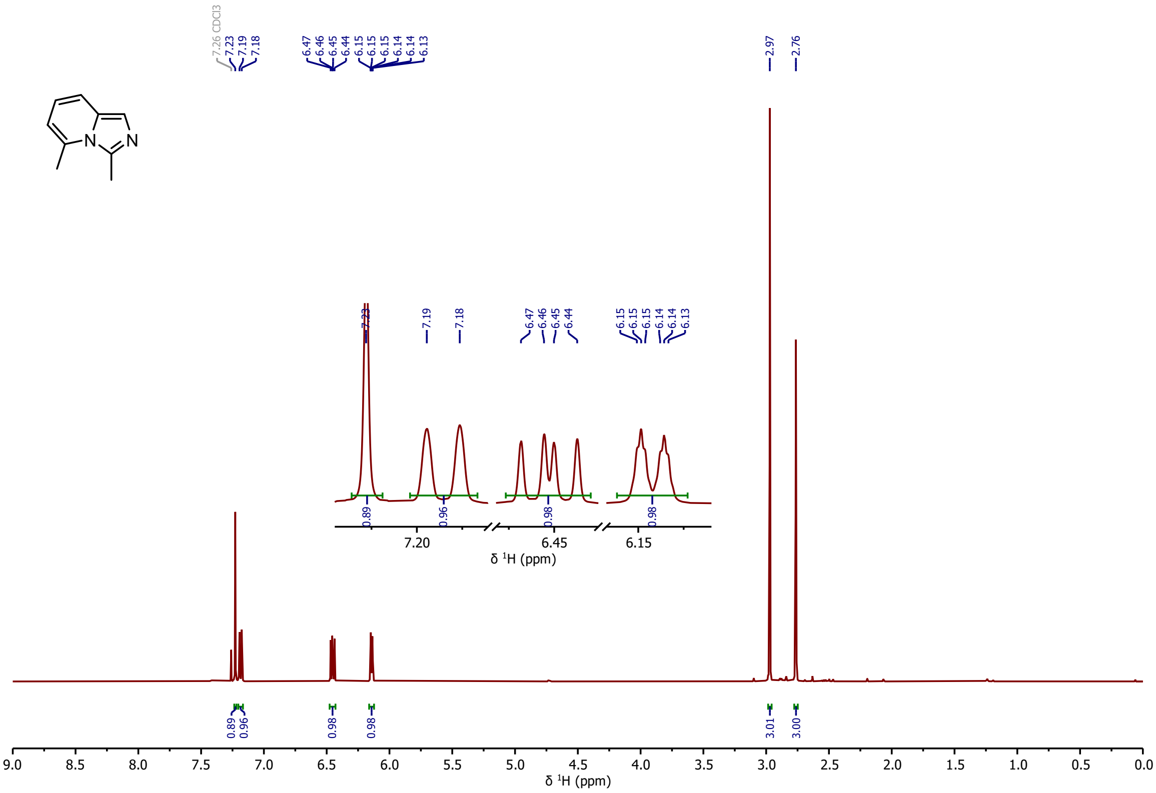


**Figure S9**: **$^{1}$H NMR** (500 MHz, $CDCl_3$, 298 K) of **S5**.

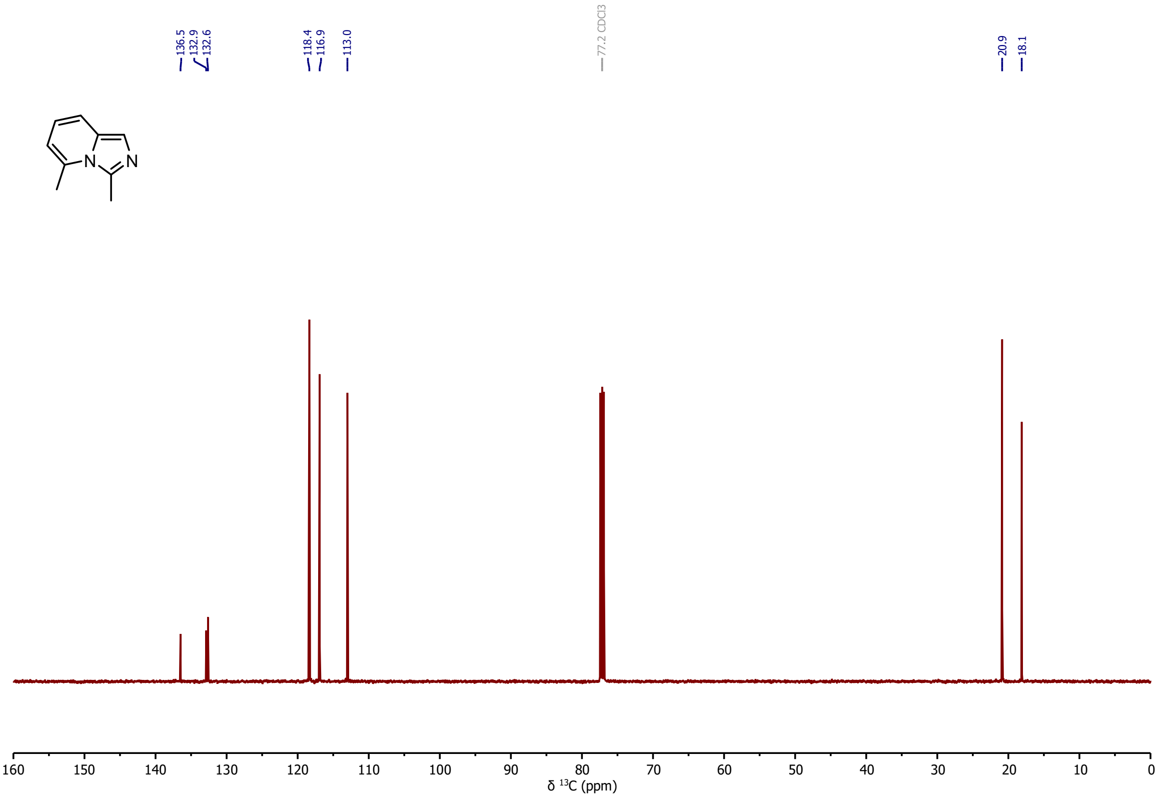


**Figure S10**: **$^{13}$C NMR** (126 MHz, $CDCl_3$, 298 K) of **S5**.

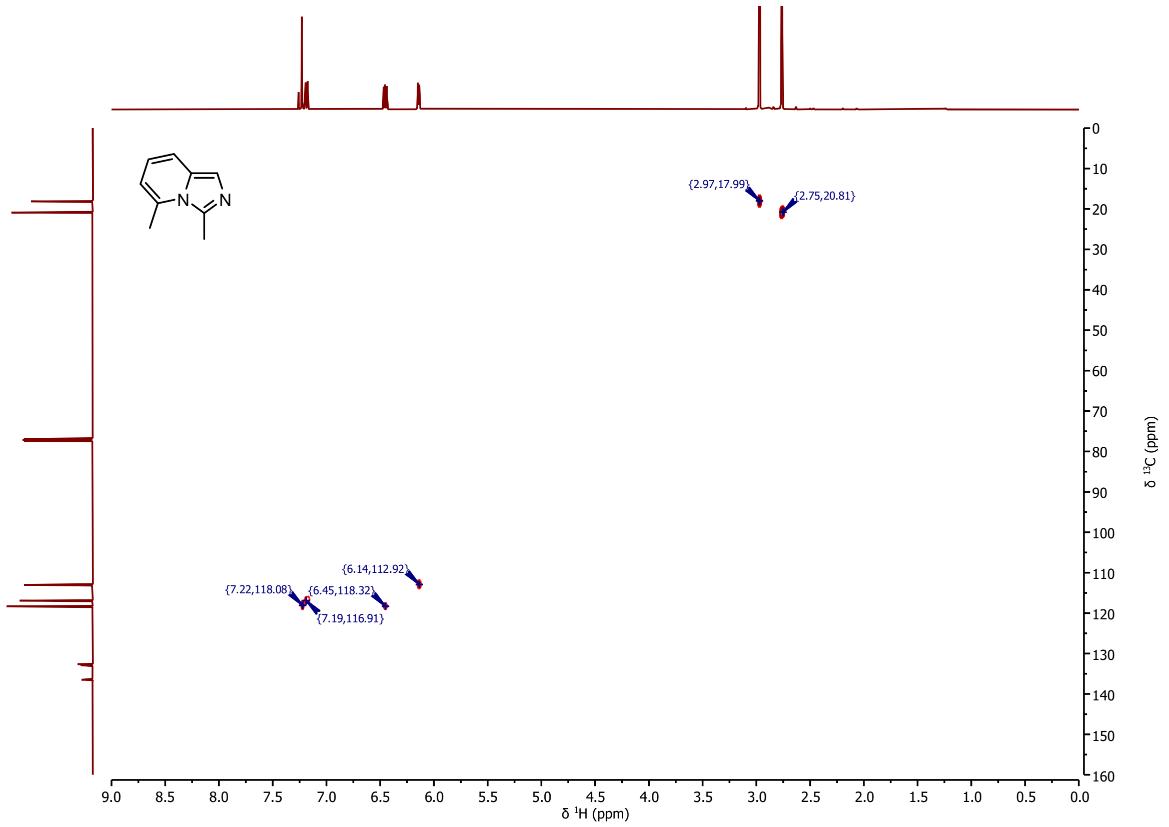


**Figure S11**: **$^{1}$H/$^{13}$C HSQC** (500/126 MHz, $CDCl_3$, 298 K) of **S5**.

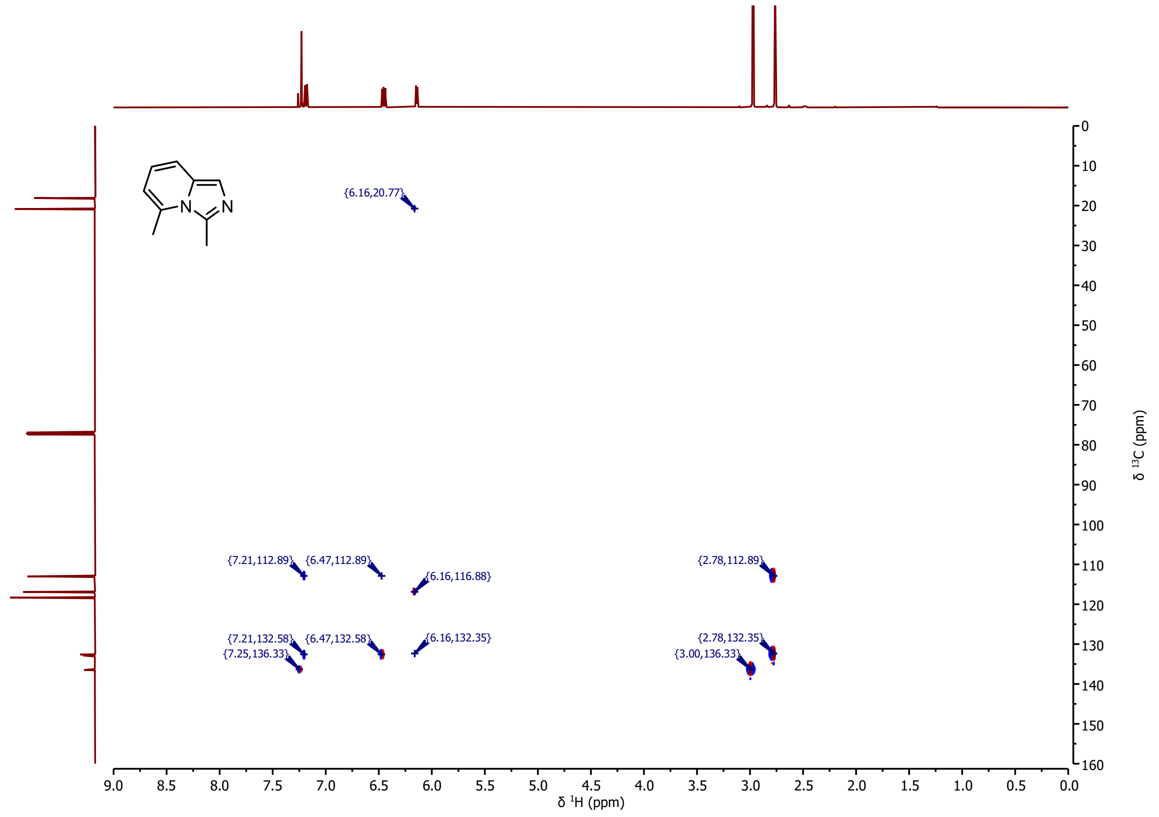


**Figure S12**: **$^{1}$H/$^{13}$C HMBC** (500/126 MHz, $CDCl_3$, 298 K) of **S5**.

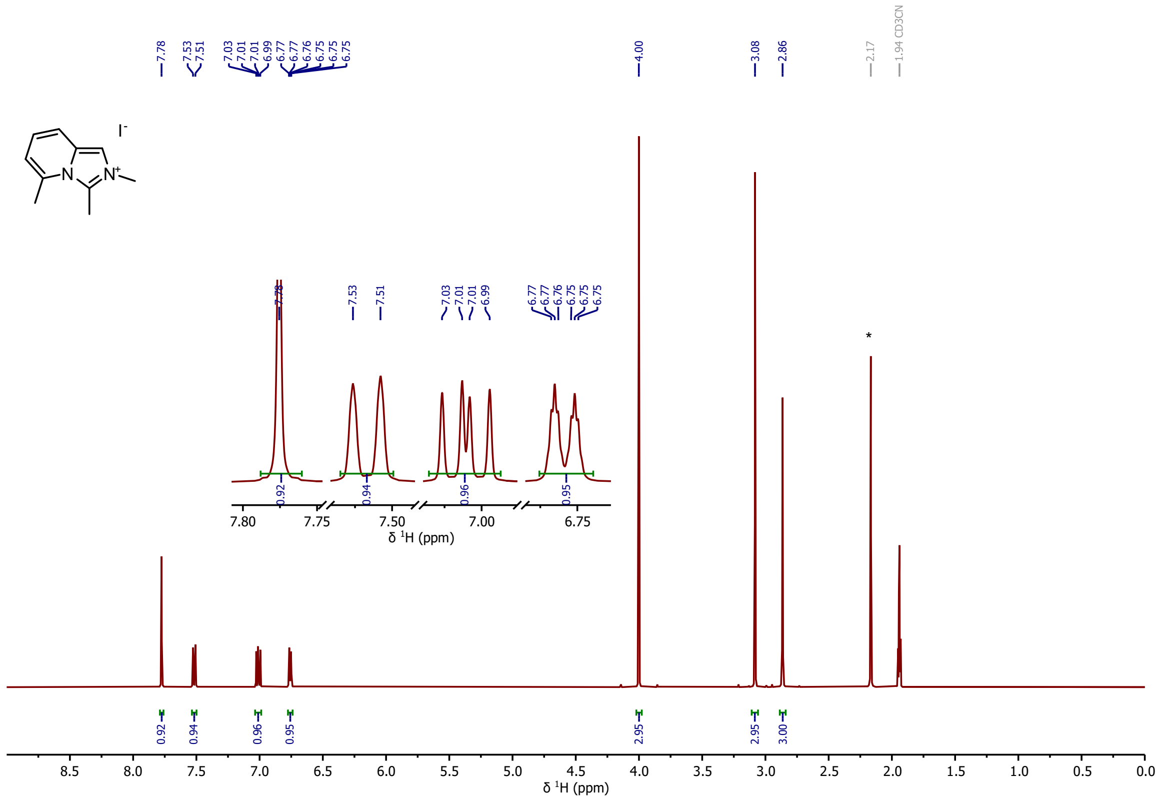


**Figure S13**: **$^{1}$H NMR** (500 MHz, $CD_3CN$, 298 K) of **1b**. * = $H_2O$.

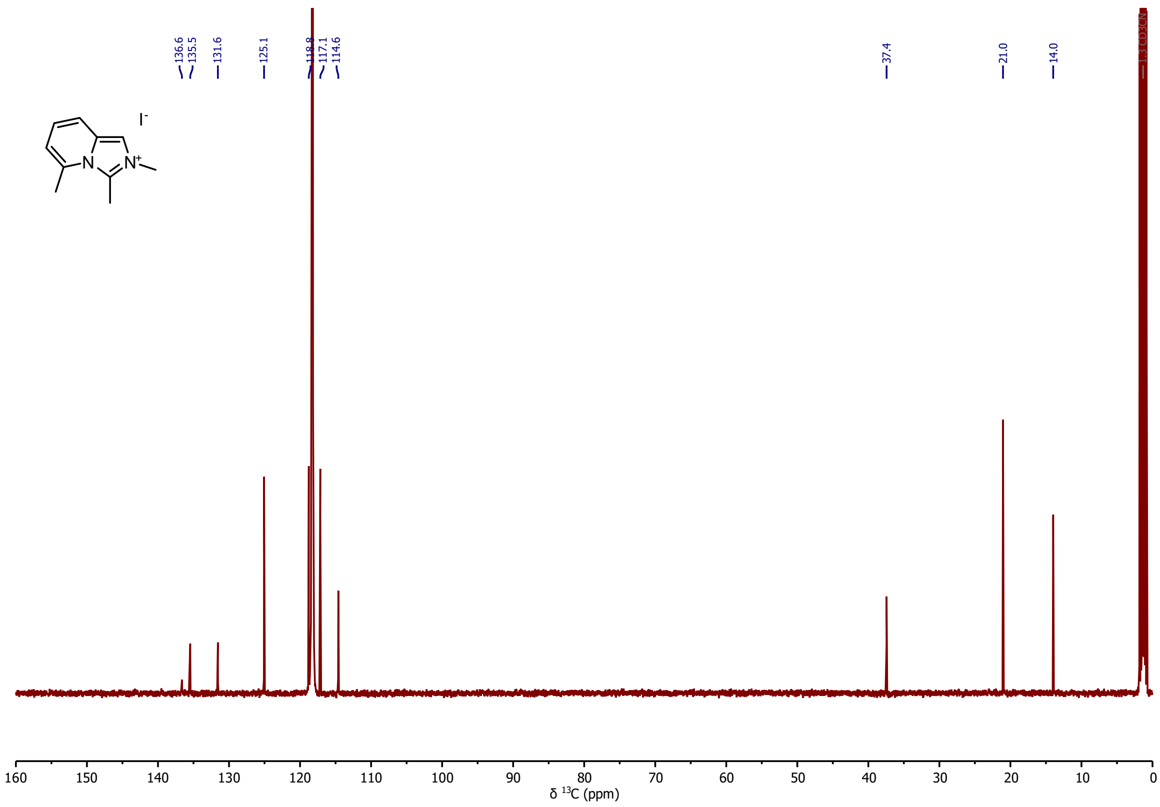


**Figure S14**: **$^{13}$C NMR** (126 MHz, $CD_3CN$, 298 K) of **1b**.

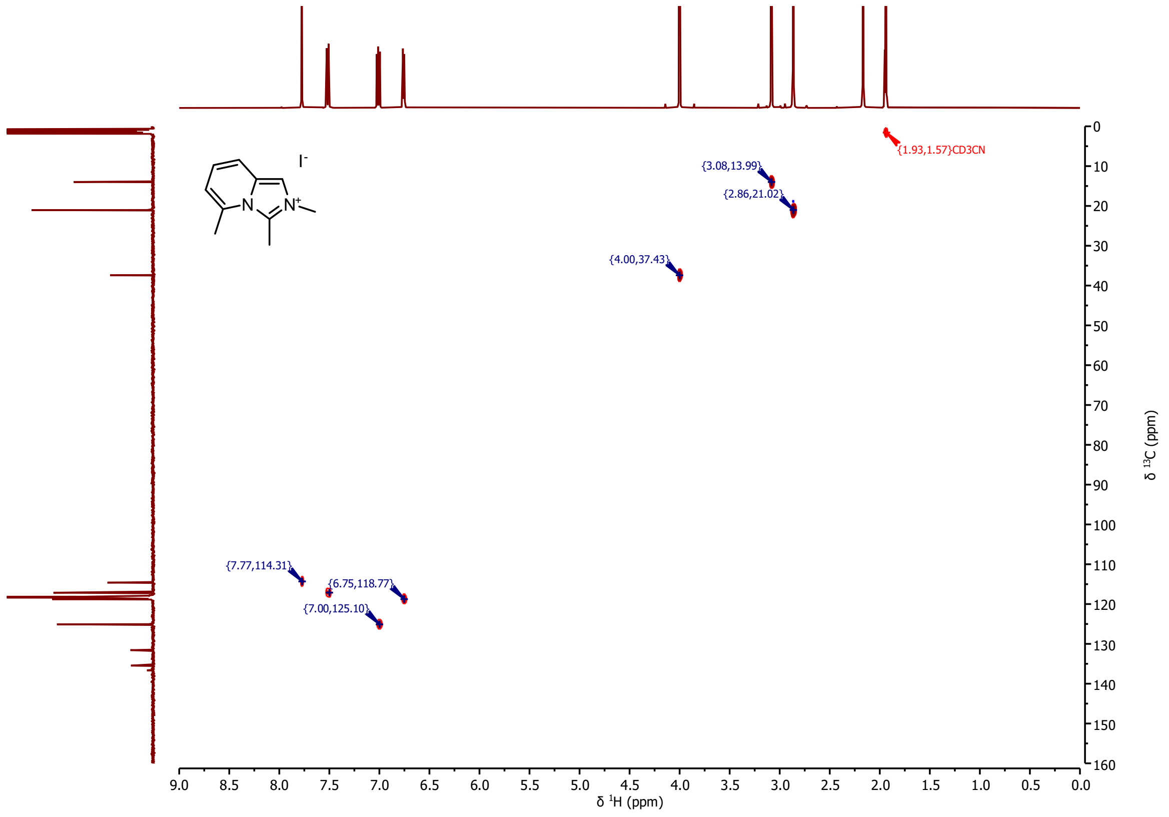


**Figure S15**: **$^{1}$H/$^{13}$C HSQC** (500/126 MHz, $CD_3CN$, 298 K) of **1b**.

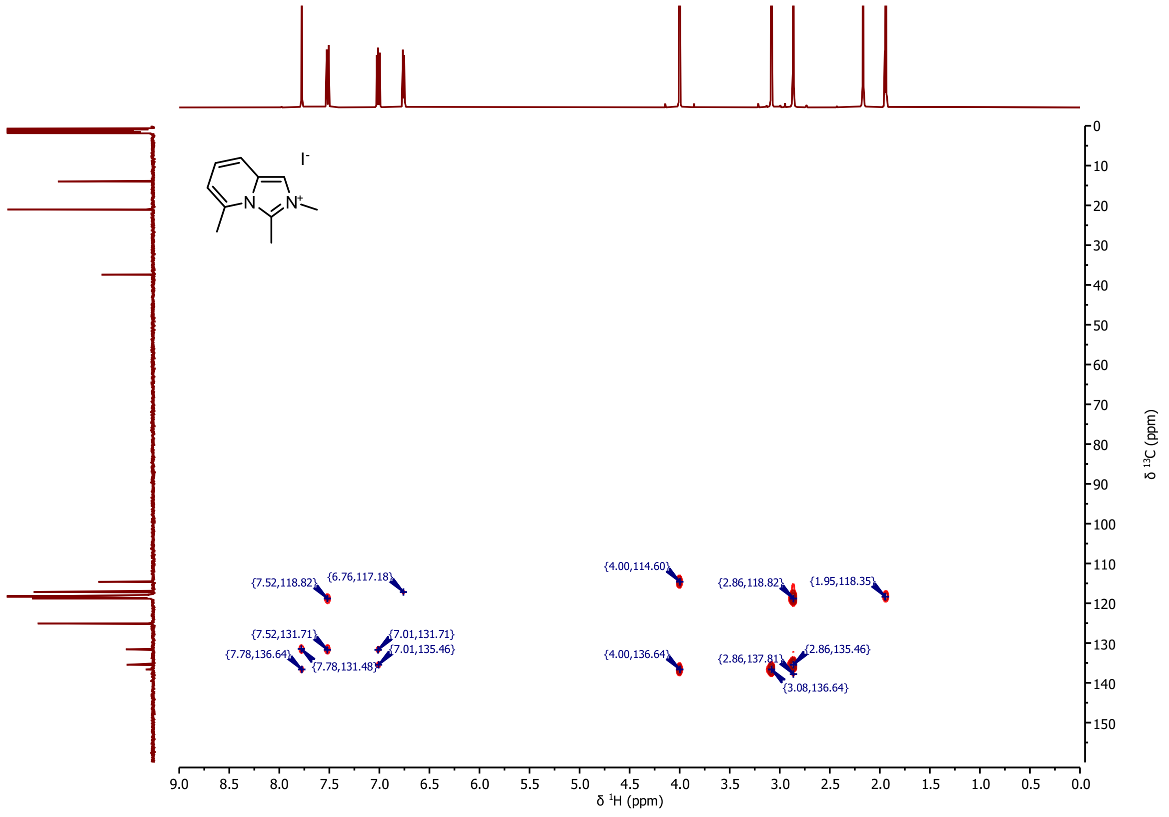


**Figure S16**: **$^{1}$H/$^{13}$C HMBC** (500/126 MHz, $CD_3CN$, 298 K) of **1b**.

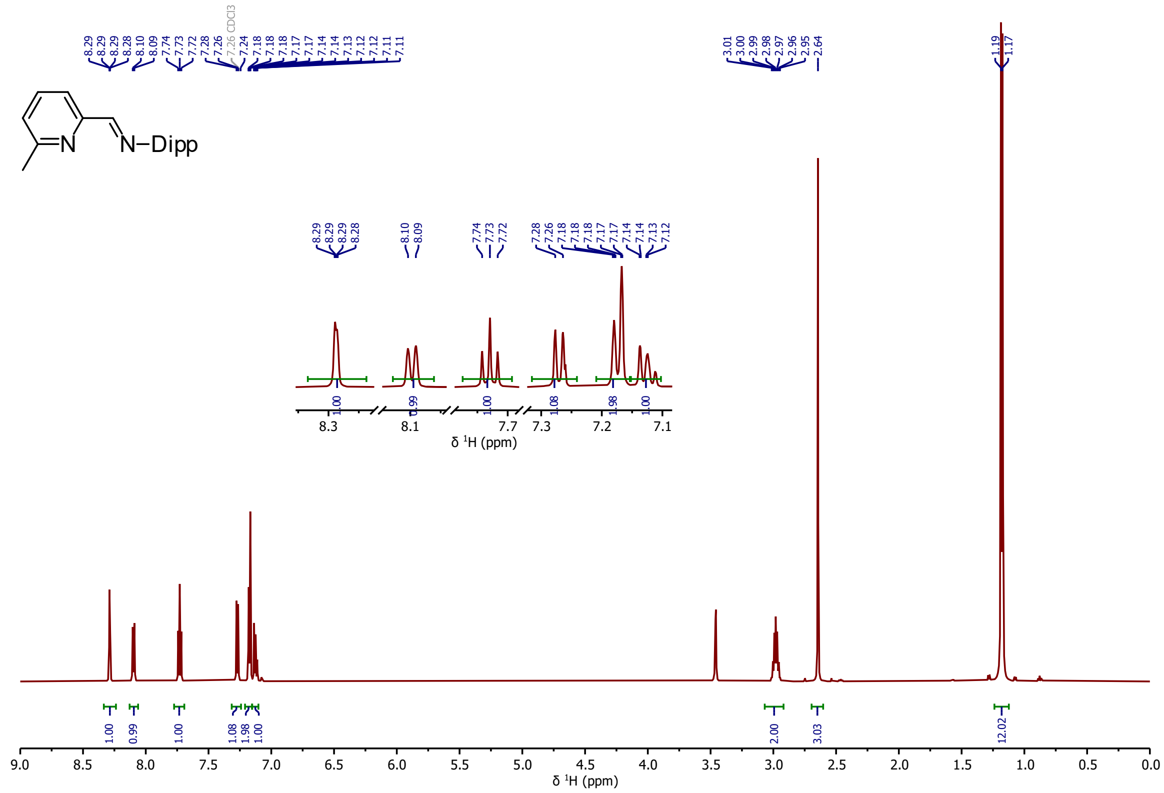


**Figure S17**: **$^1$H NMR** (600 MHz, $CDCl_3$, 298 K) of **S6**.

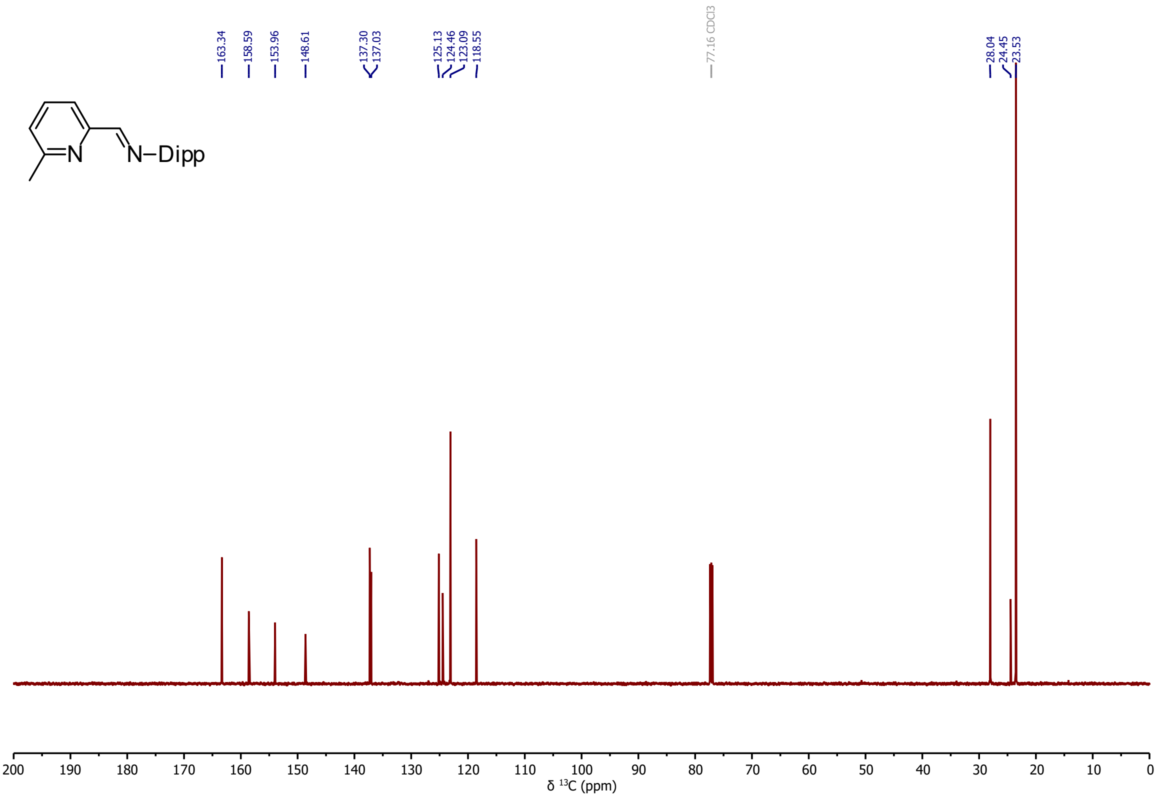


**Figure S18**: **$^{13}$C NMR** (151 MHz, $CDCl_3$, 298 K) of **S6**.

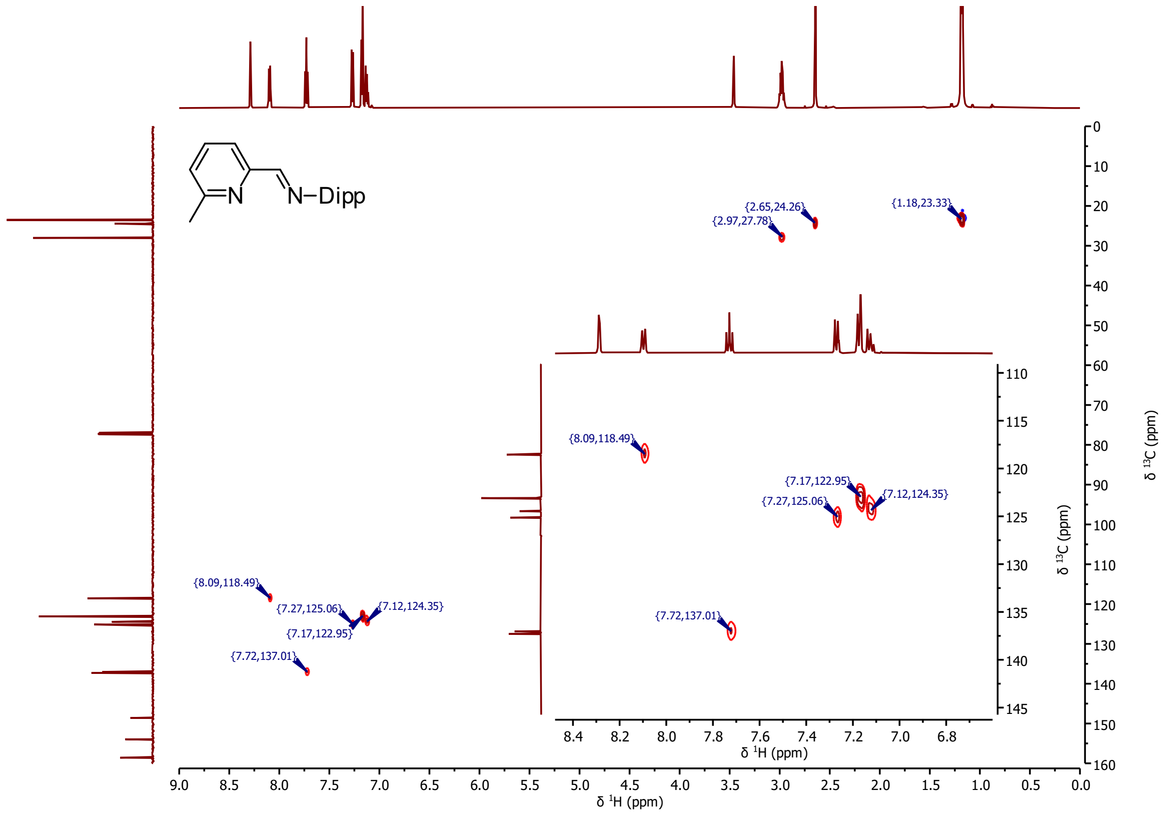


**Figure S19**: **¹H/¹³C HSQC** (600/151 MHz, $CDCl_3$, 298 K) of **S6**.

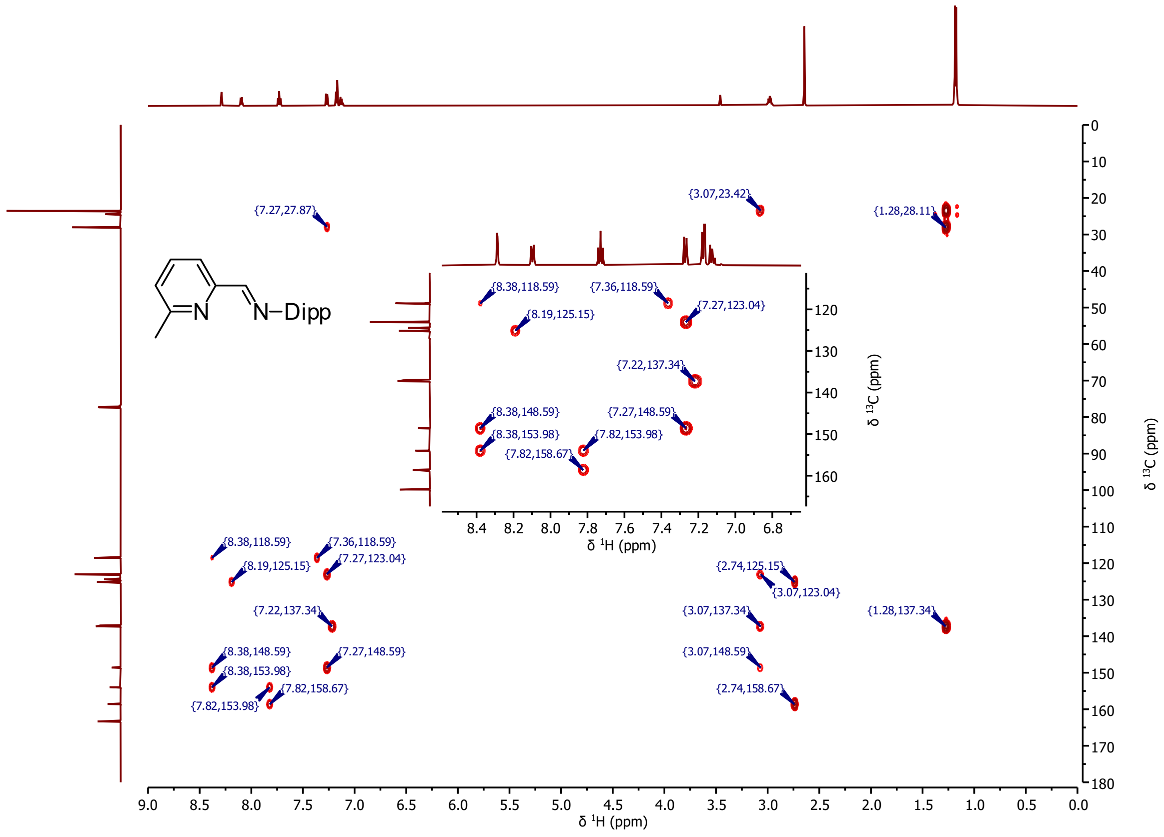


**Figure S20**: **¹H/¹³C HMBC** (600/151 MHz, $CDCl_3$, 298 K) of **S6**.

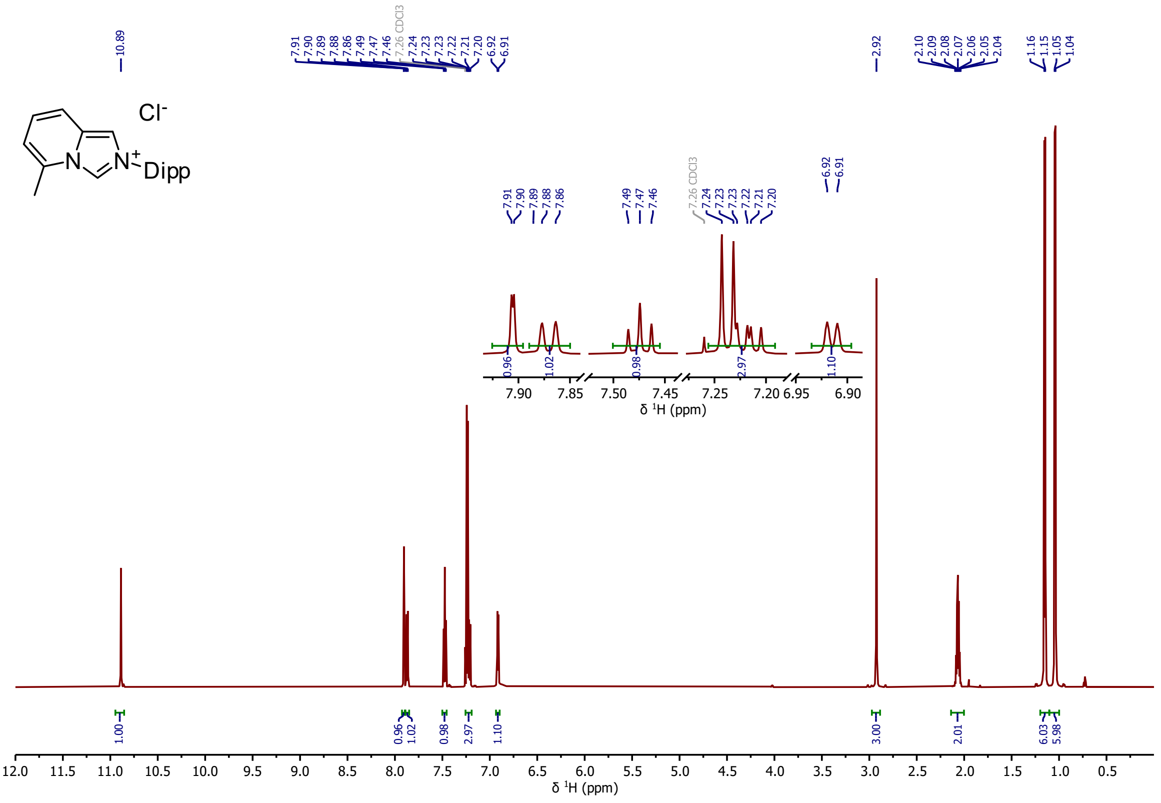


**Figure S21**: $^{1}$**H NMR** (700 MHz, $CDCl_3$, 298 K) of **S7**.

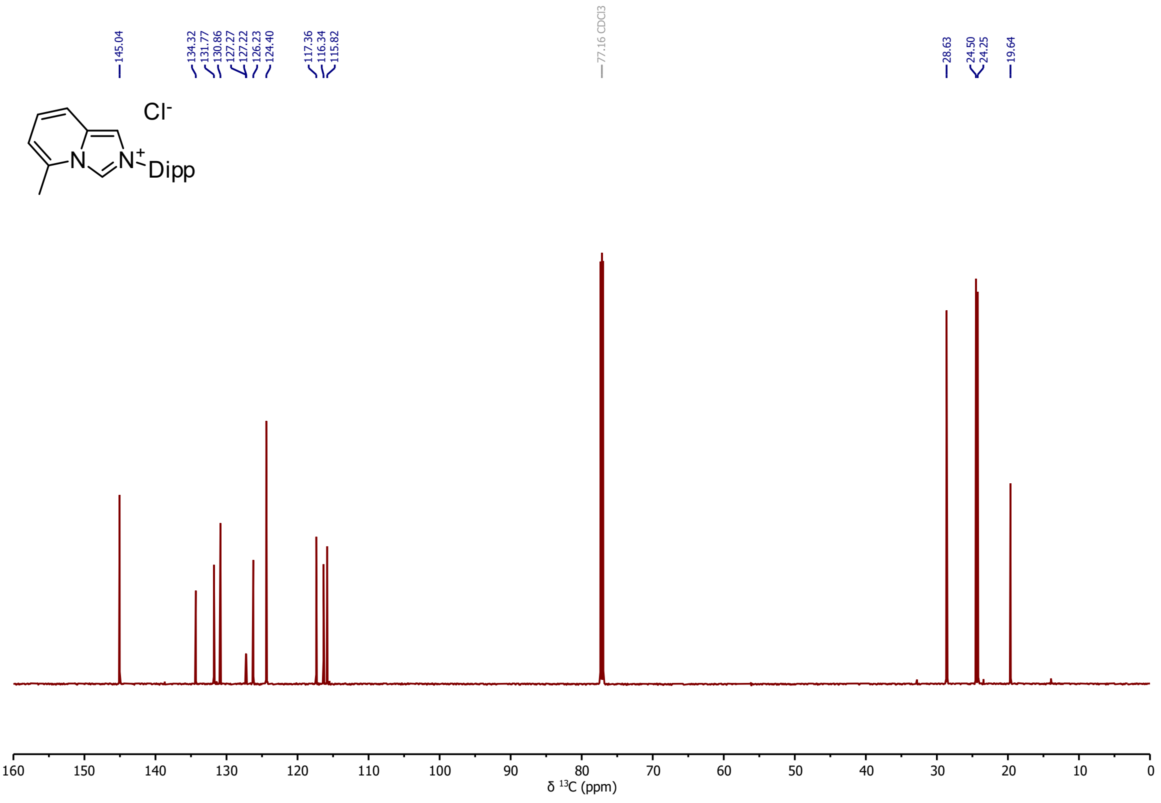


**Figure S22**: $^{13}$**C NMR** (176 MHz, $CDCl_3$, 298 K) of **S7**.

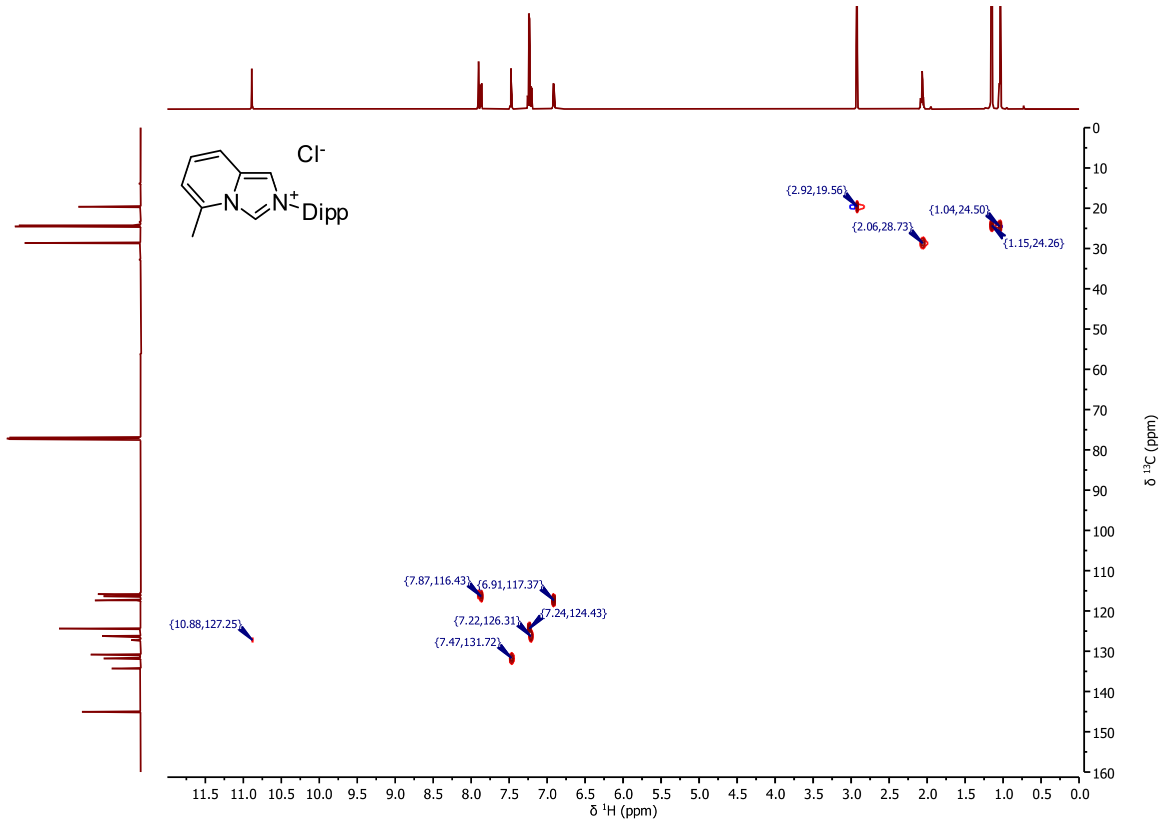

**Figure S23**: **$^{1}$H/$^{13}$C HSQC** (700/176 MHz, $CDCl_3$, 298 K) of **S7**.

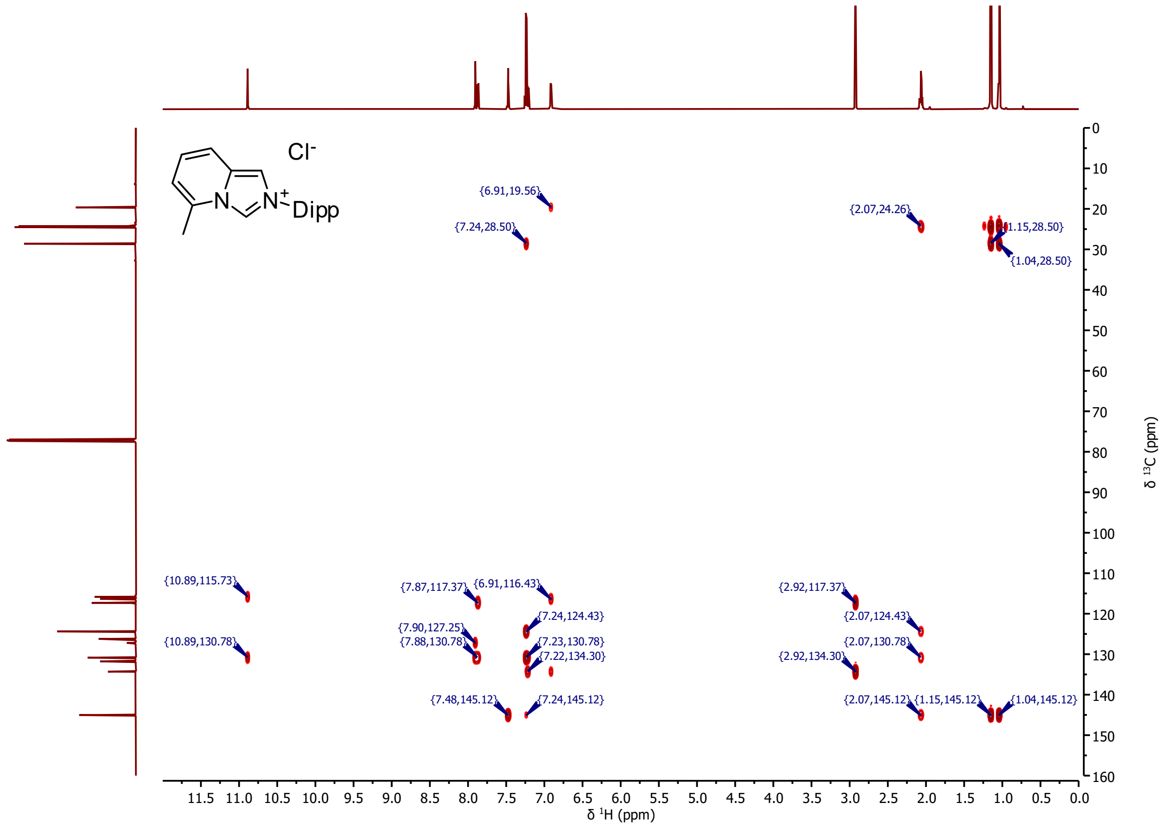

**Figure S24**: **$^{1}$H/$^{13}$C HMBC** (700/176 MHz, $CDCl_3$, 298 K) of **S7**.

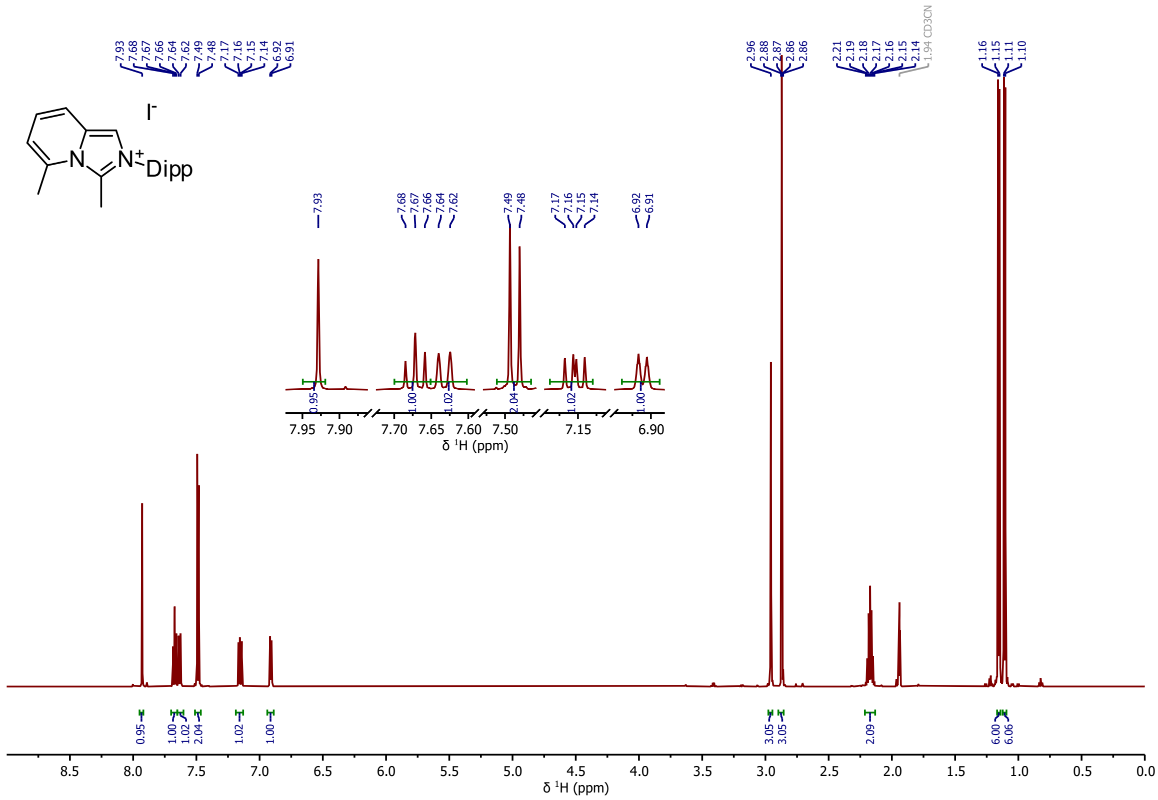


**Figure S25**: **$^1$H NMR** (600 MHz, $CD_3CN$, 298 K) of **1c**.

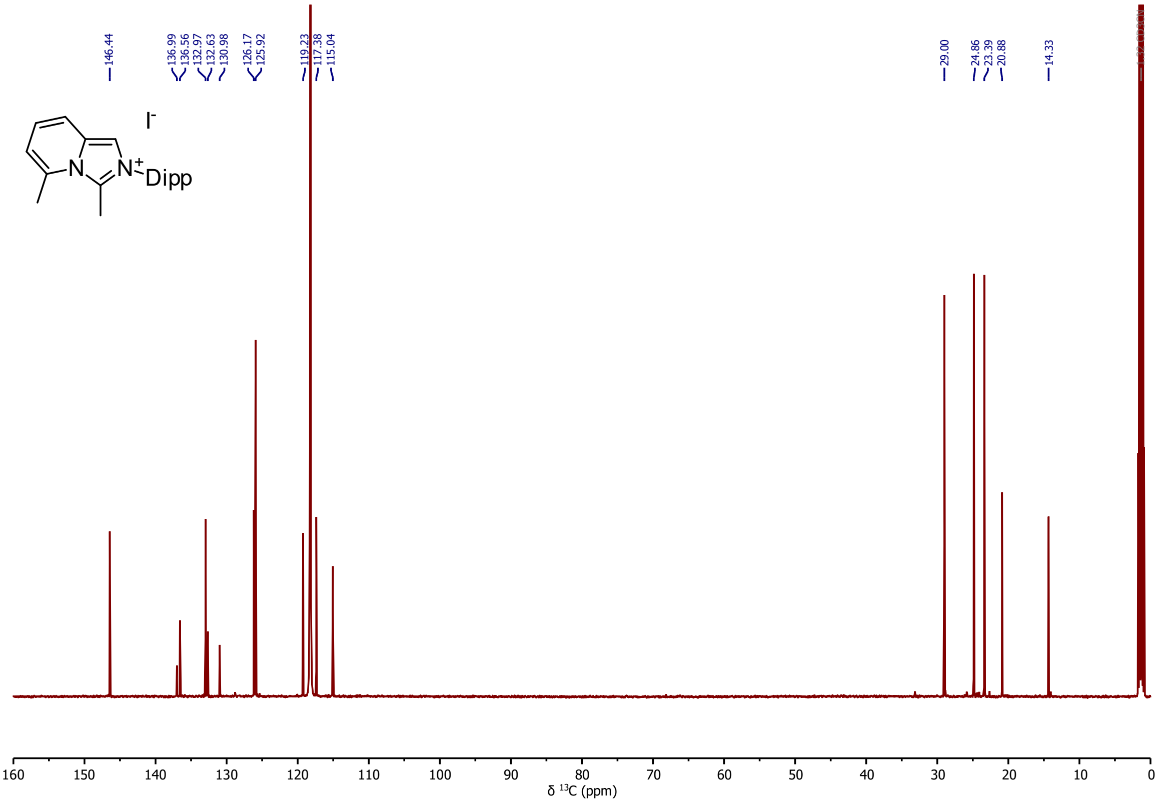


**Figure S26**: **$^{13}$C NMR** (151 MHz, $CD_3CN$, 298 K) of **1c**.

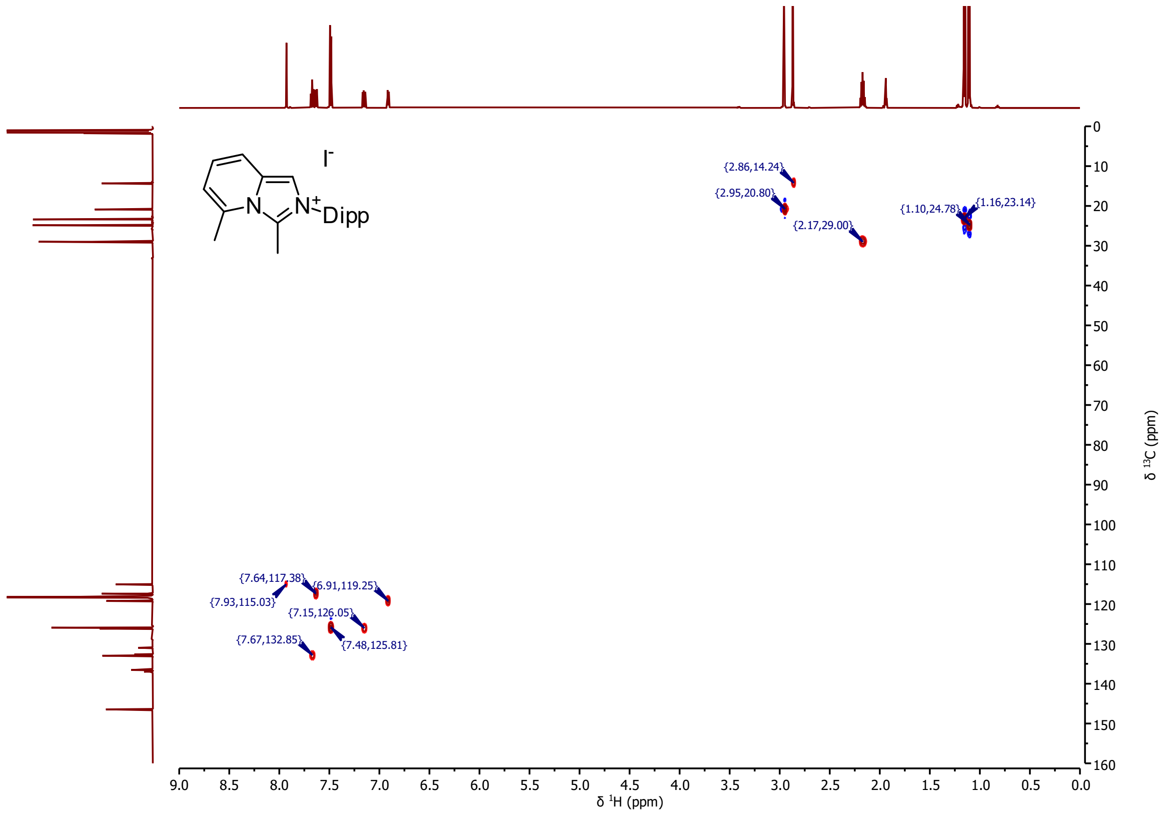


**Figure S27**: **$^{1}H/^{13}C$ HSQC** (600/151 MHz, $CD_3CN$, 298 K) of **1c**.

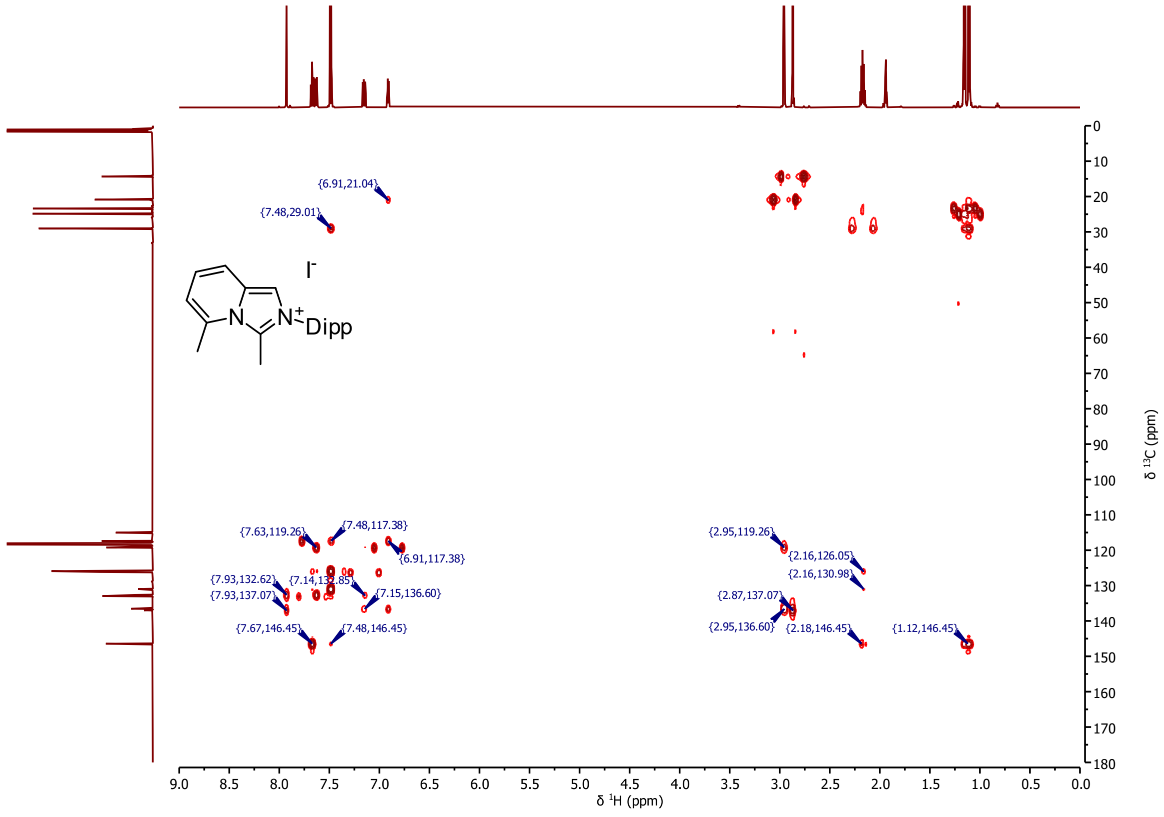


**Figure S28**: **$^{1}H/^{13}C$ HMBC** (600/151 MHz, $CD_3CN$, 298 K) of **1c**.

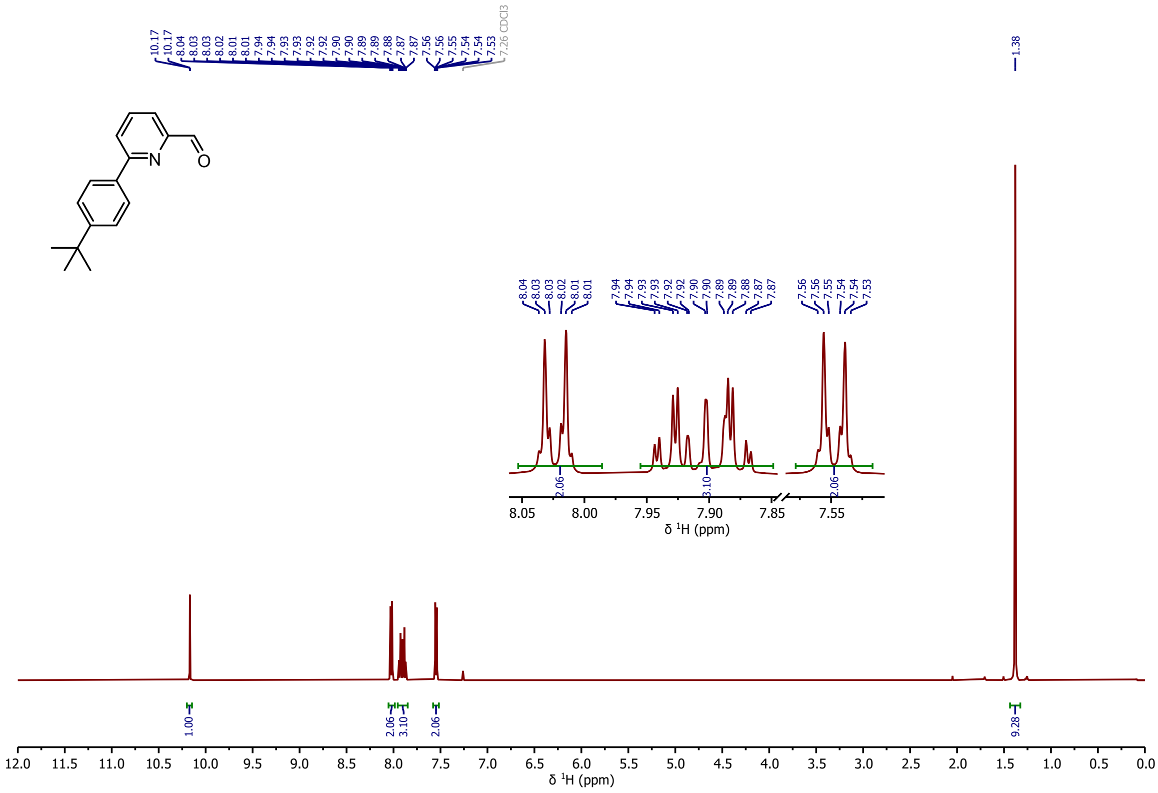

**Figure S29**: **$^1$H NMR** (500 MHz, $CDCl_3$, 298 K) of **S9**.

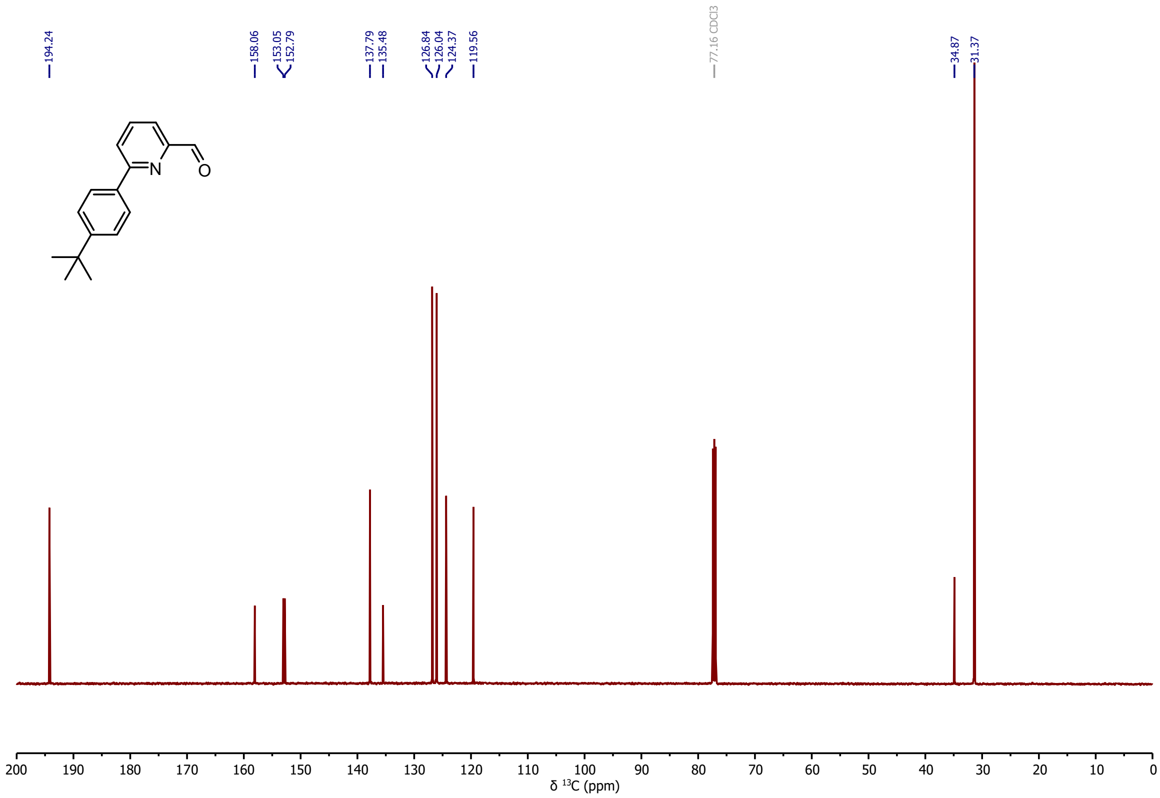

**Figure S30**: **$^{13}$C NMR** (126 MHz, $CDCl_3$, 298 K) of **S9**.

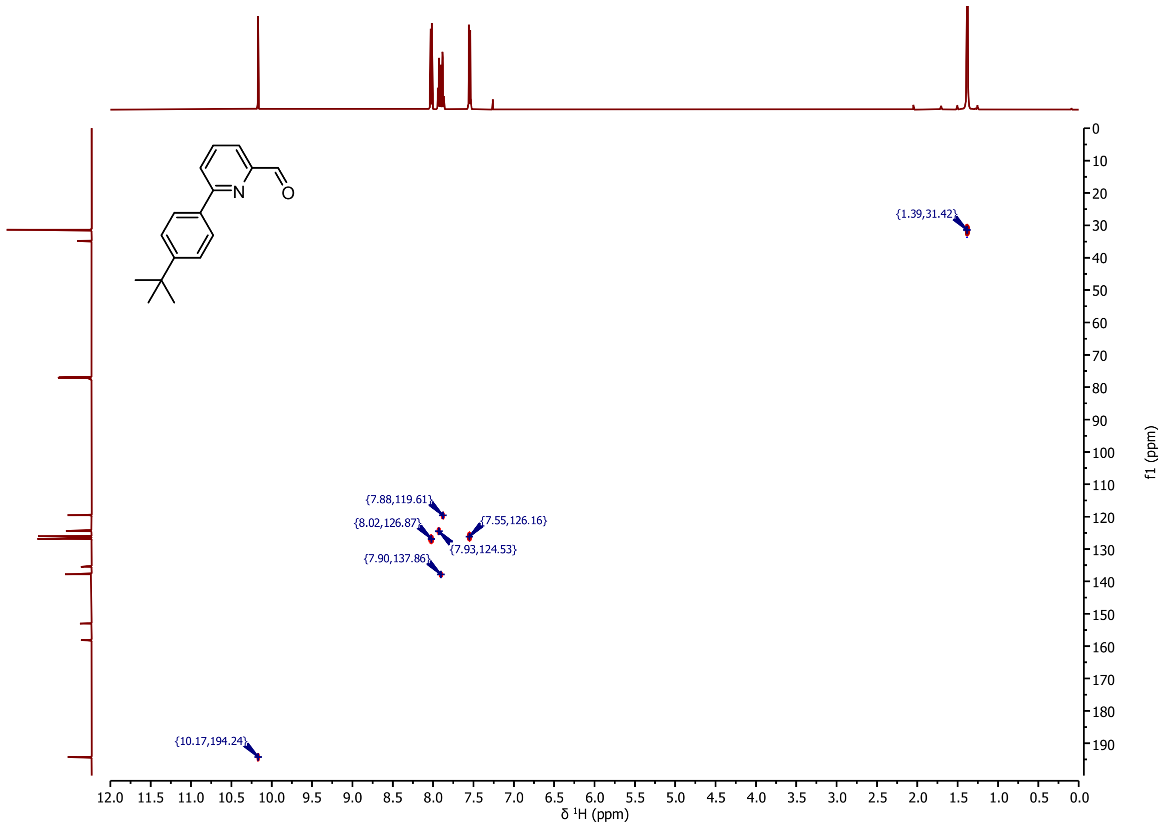

**Figure S31**: **$^{1}$H/$^{13}$C HSQC** (500/126 MHz, $CDCl_3$, 298 K) of **S9**.

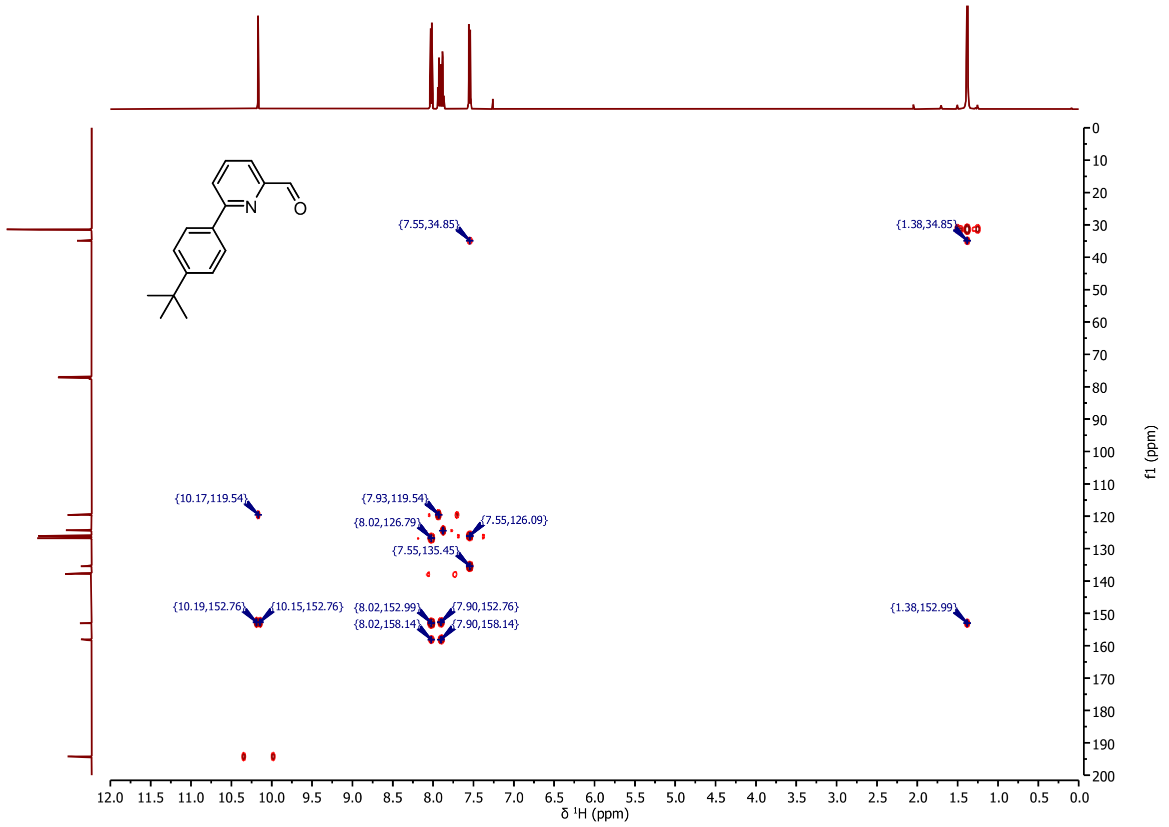

**Figure S32**: **$^{1}$H/$^{13}$C HMBC** (500/126 MHz, $CDCl_3$, 298 K) of **S9**.

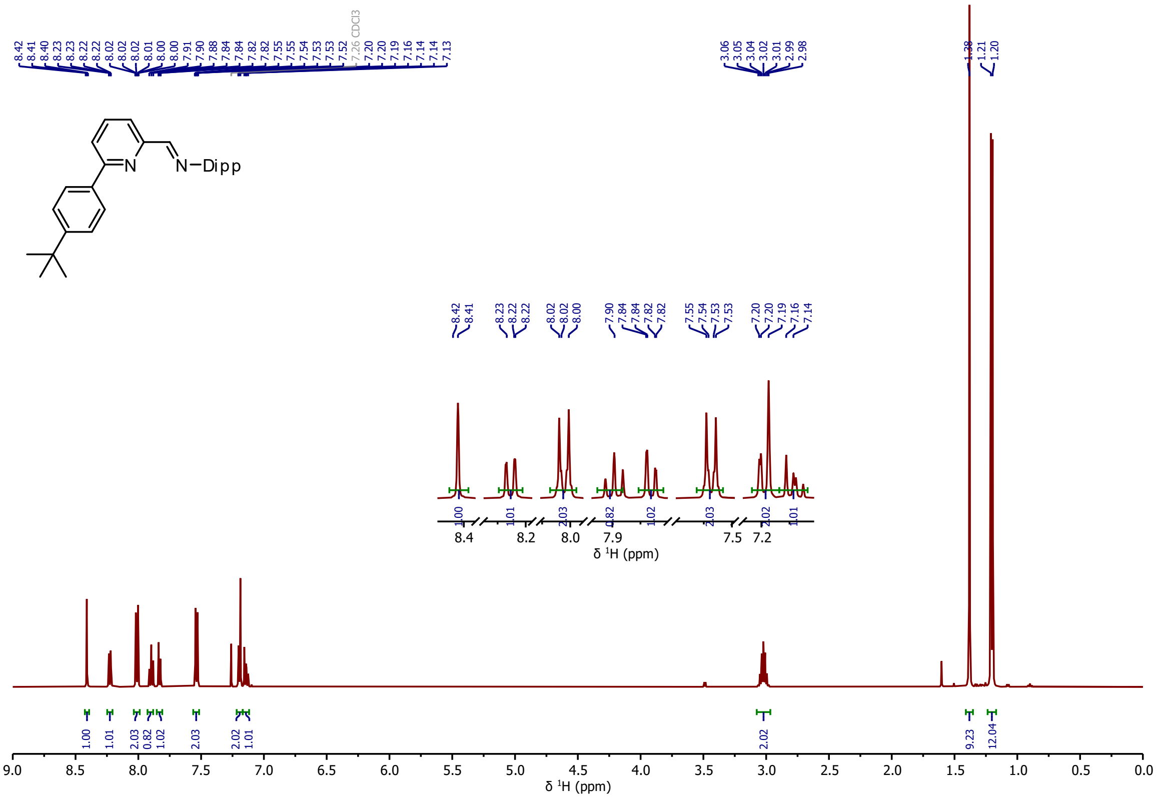

**Figure S33**: **$^1$H NMR** (500 MHz, $CDCl_3$, 298 K) of **S10**.

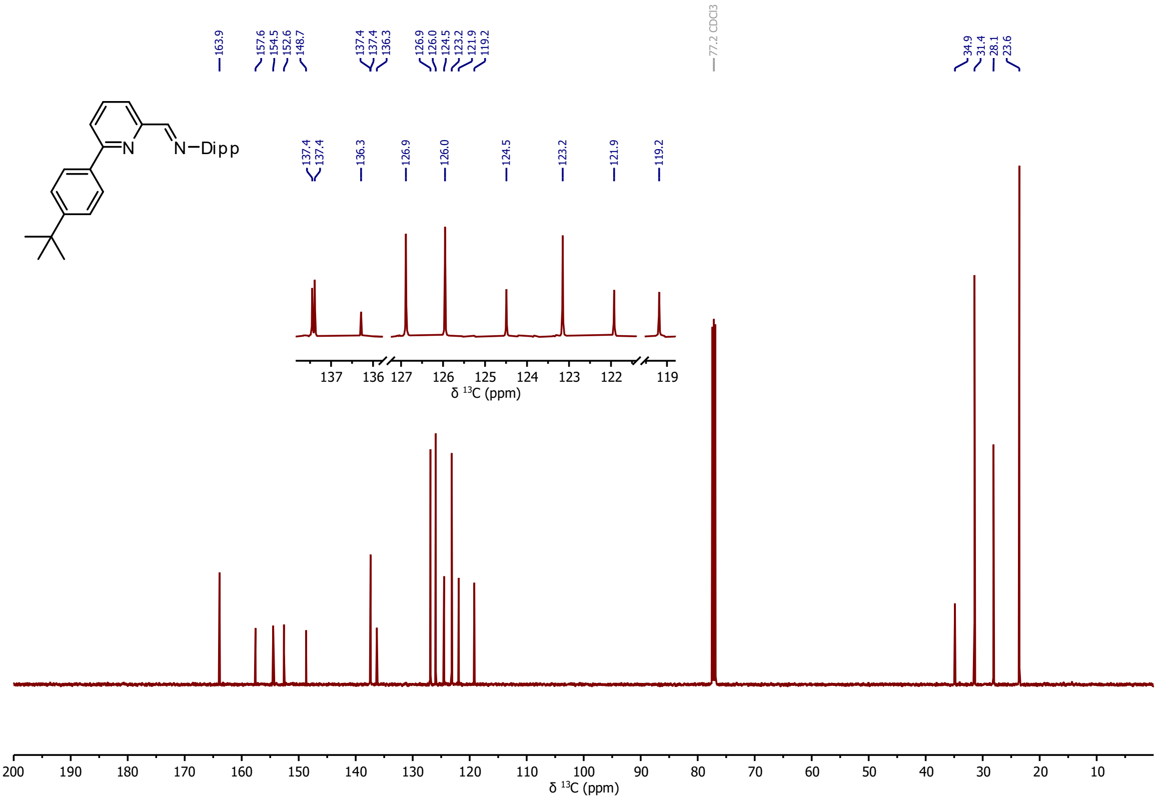

**Figure S34**: **$^{13}$C NMR** (126 MHz, $CDCl_3$, 298 K) of **S10**.

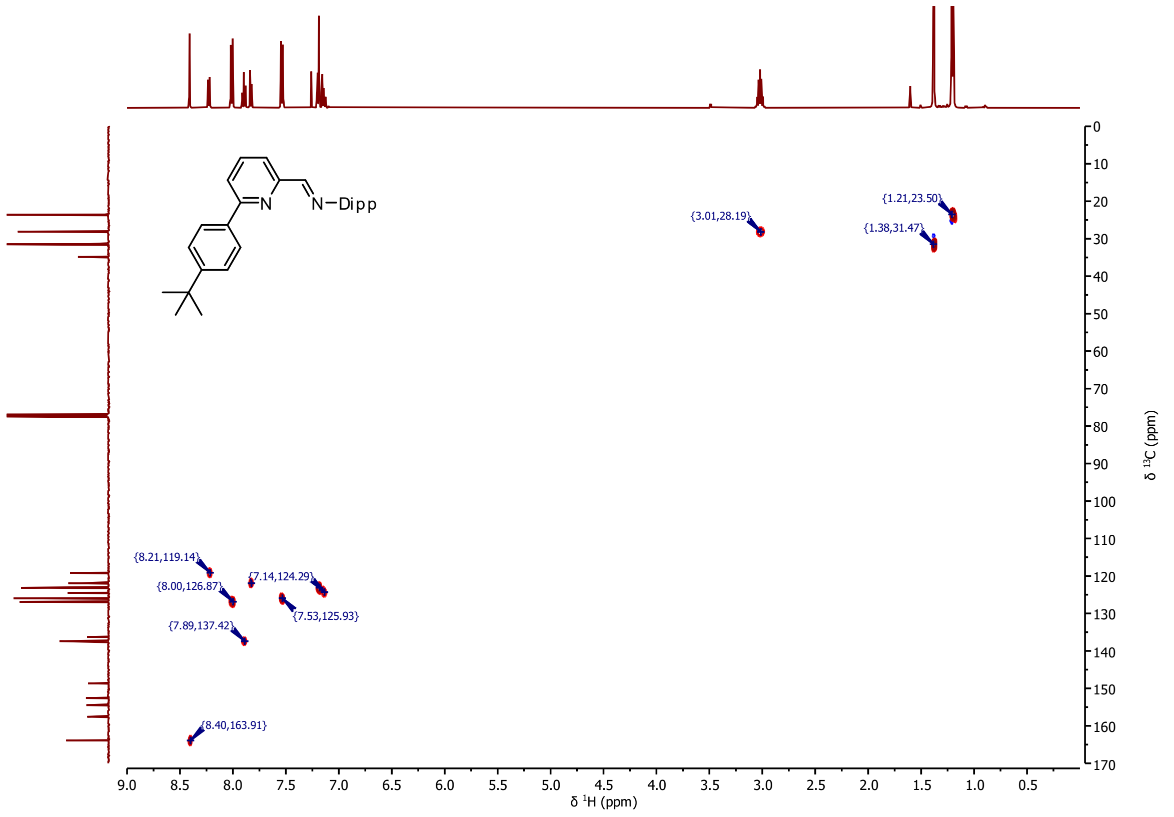


**Figure S35**: **$^{1}$H/$^{13}$C HSQC** (500/126 MHz, $CDCl_3$, 298 K) of **S10**.

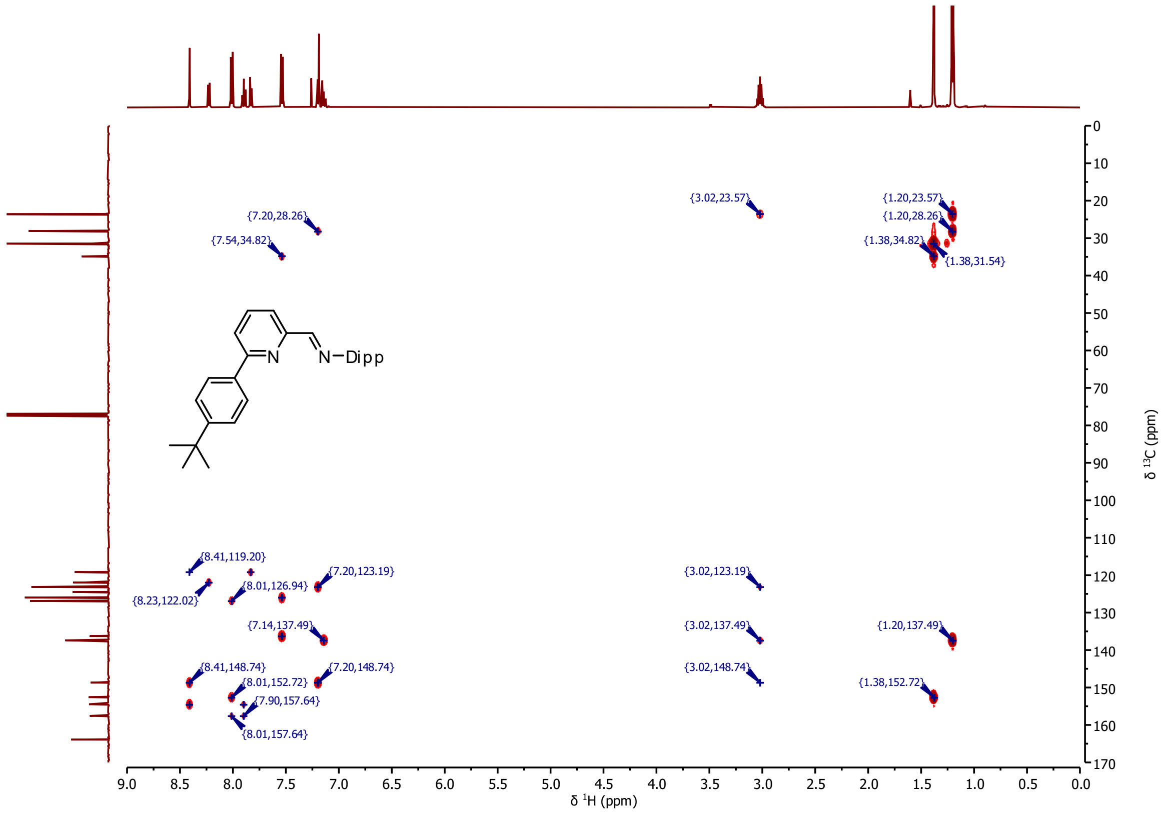


**Figure S36**: **$^{1}$H/$^{13}$C HMBC** (500/126 MHz, $CDCl_3$, 298 K) of **S10**.

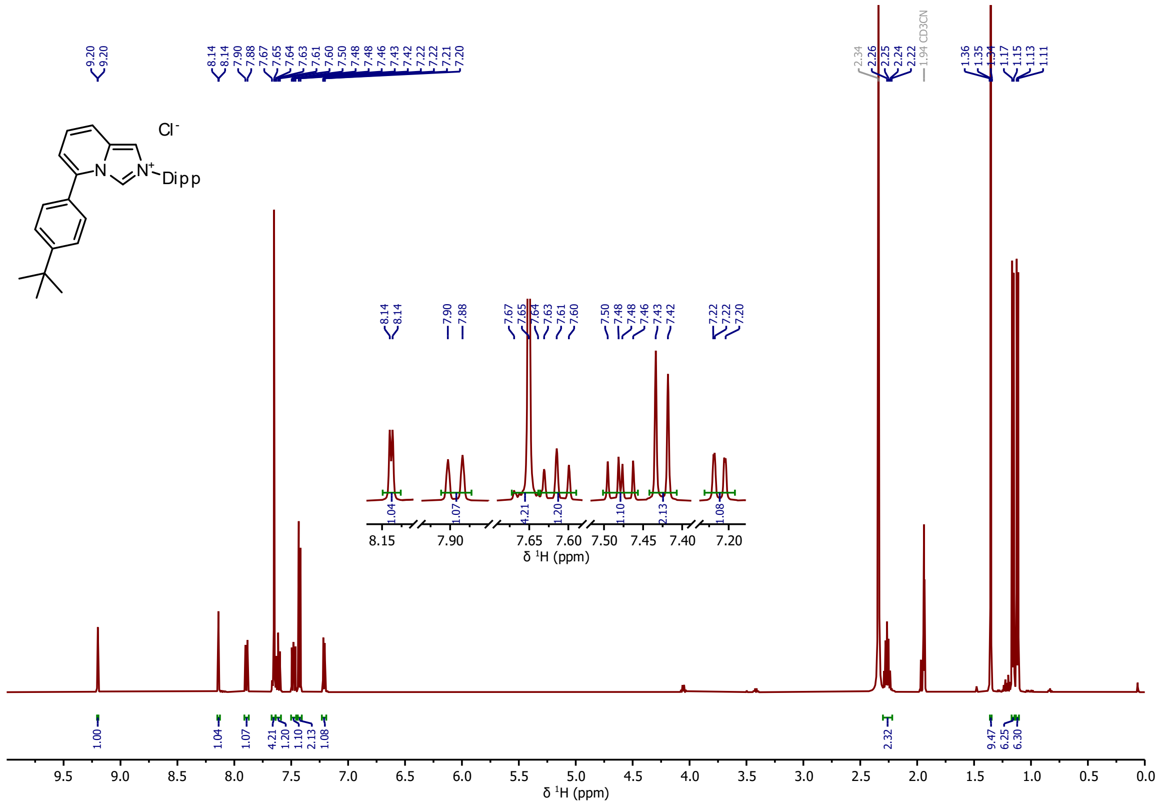


**Figure S37**: **$^1$H NMR** (500 MHz, $CD_3CN$, 298 K) of **S11**.

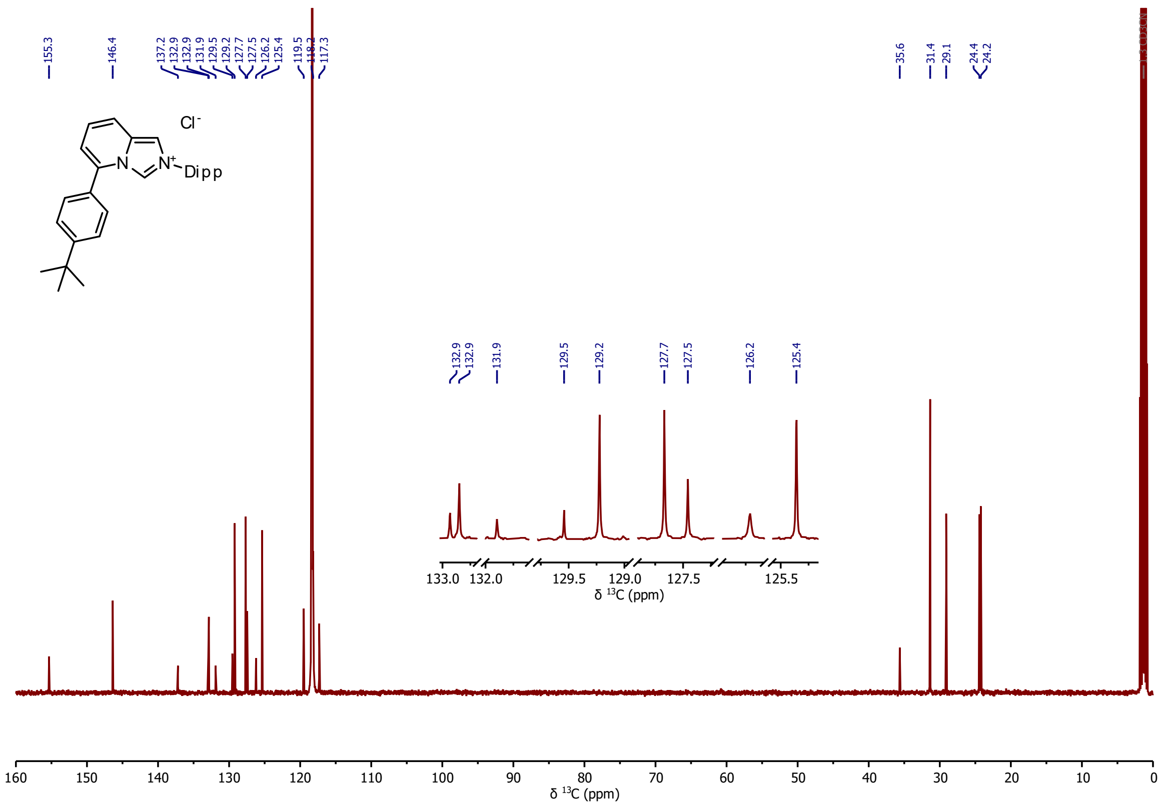


**Figure S38**: **$^{13}$C NMR** (126 MHz, $CD_3CN$, 298 K) of **S11**.

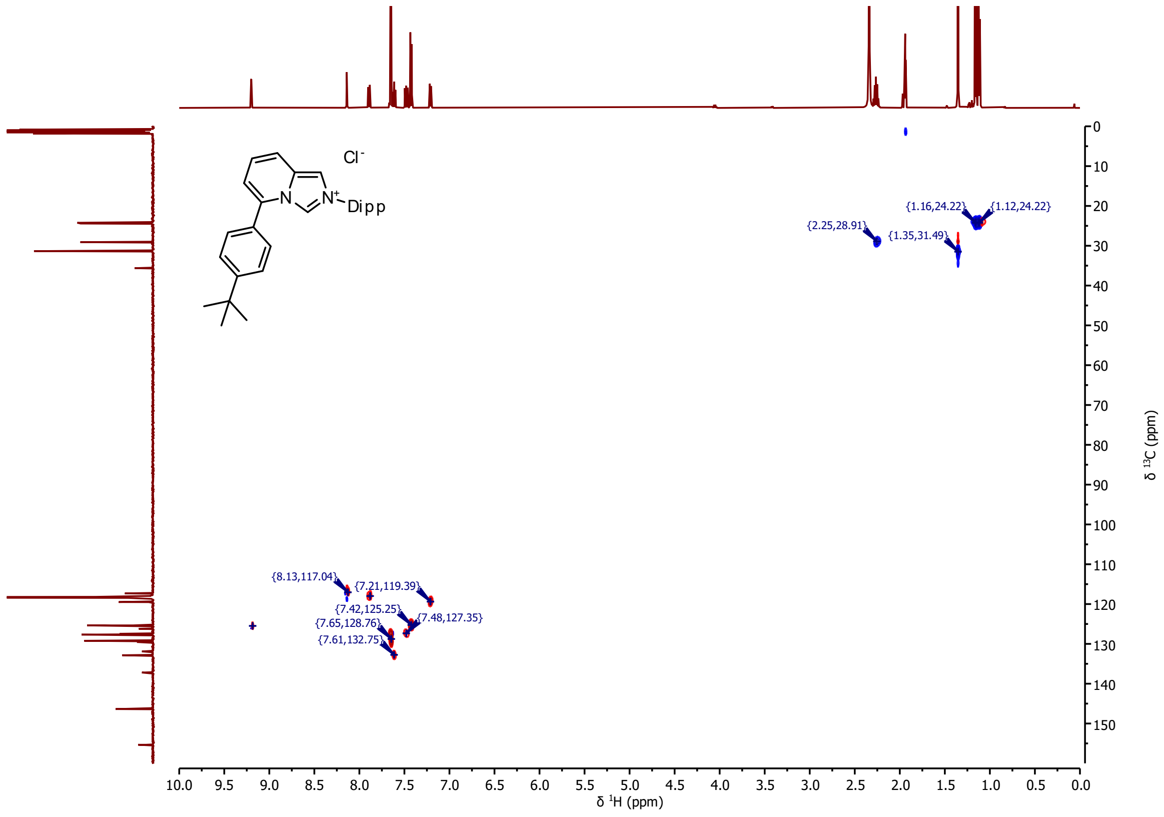

**Figure S39**: **$^{1}$H/$^{13}$C HSQC** (500/126 MHz, $CD_3CN$, 298 K) of **S11**.

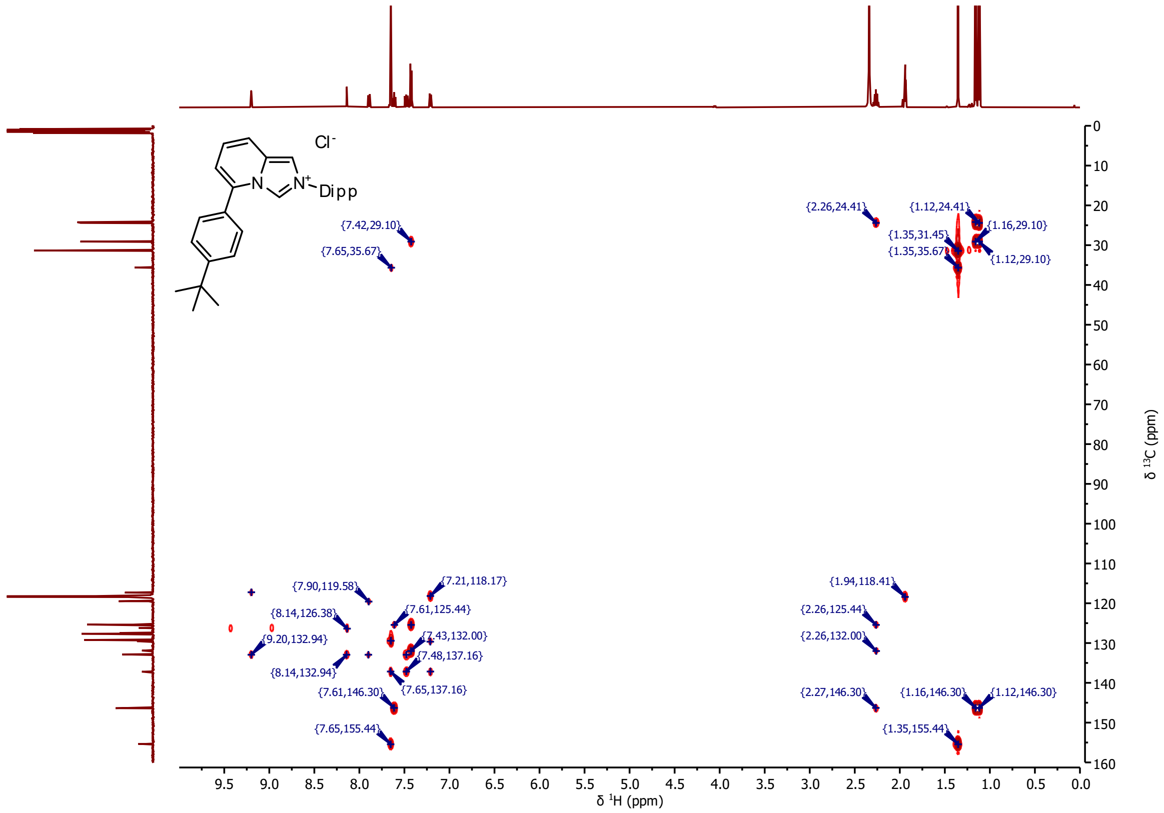

**Figure S40**: **$^{1}$H/$^{13}$C HMBC** (500/126 MHz, $CD_3CN$, 298 K) of **S11**.

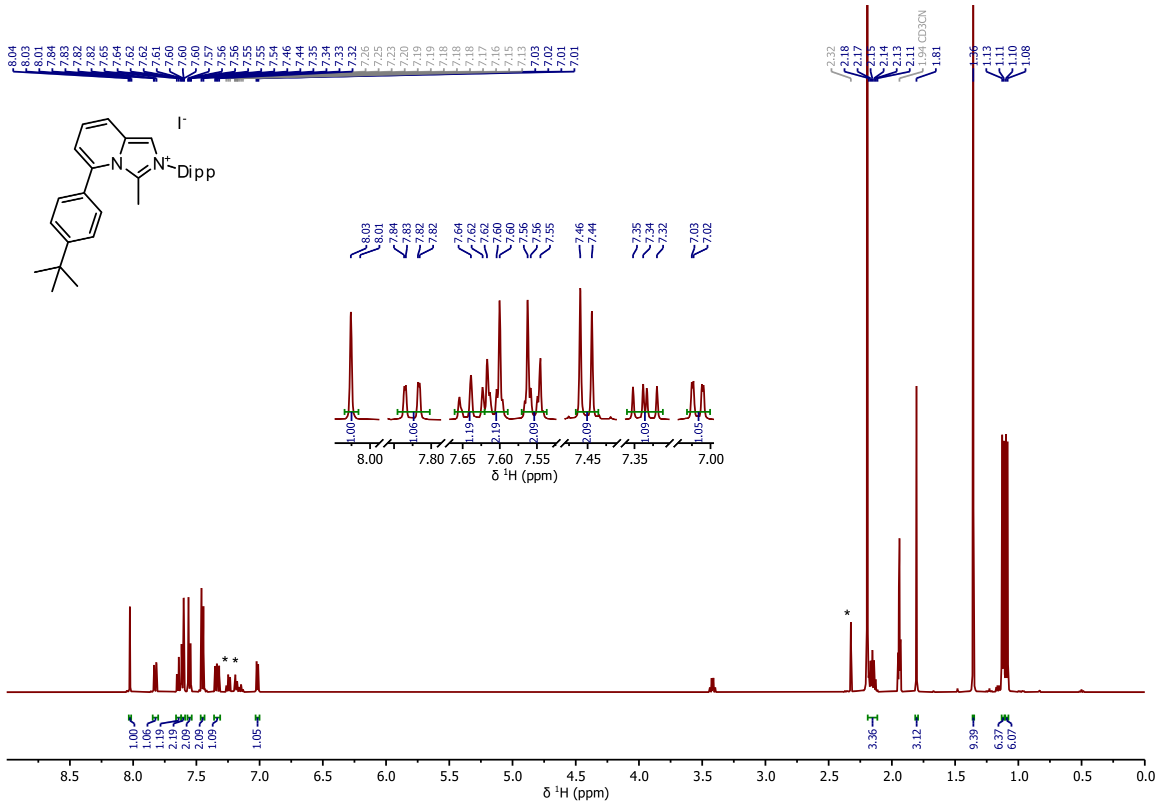


**Figure S41**: **$^{1}$H NMR** (500 MHz, $CD_3CN$, 298 K) of **1d**. * = PhMe.

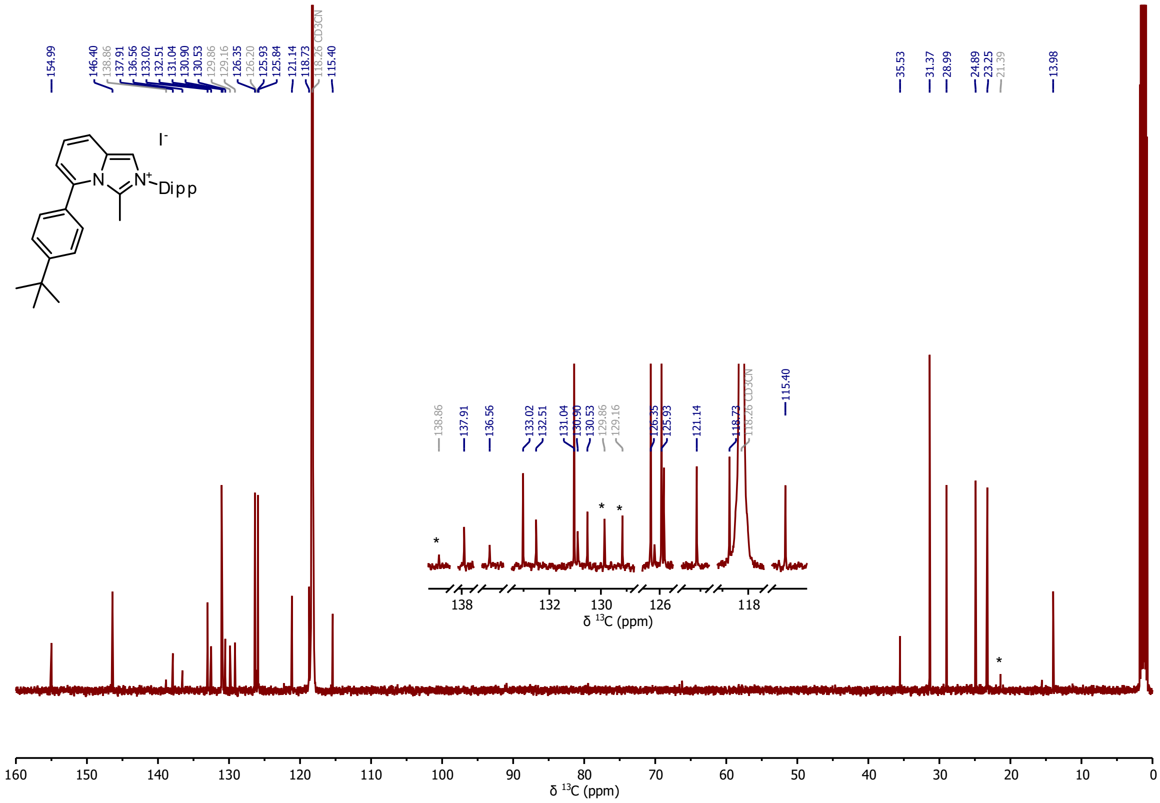


**Figure S42**: **$^{13}$C NMR** (126 MHz, $CD_3CN$, 298 K) of **1d**. * = PhMe.

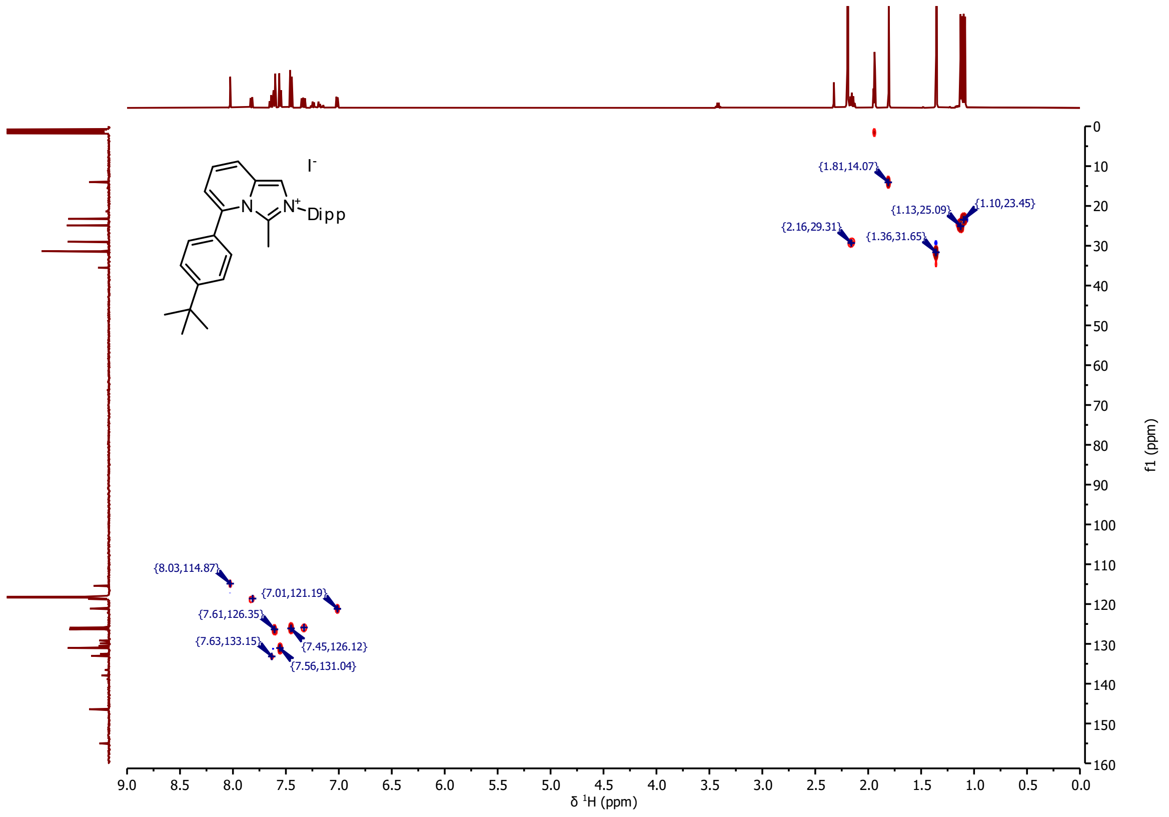

**Figure S43**: **$^{1}$H/$^{13}$C HSQC** (500/126 MHz, $CD_3CN$, 298 K) of **1d**.

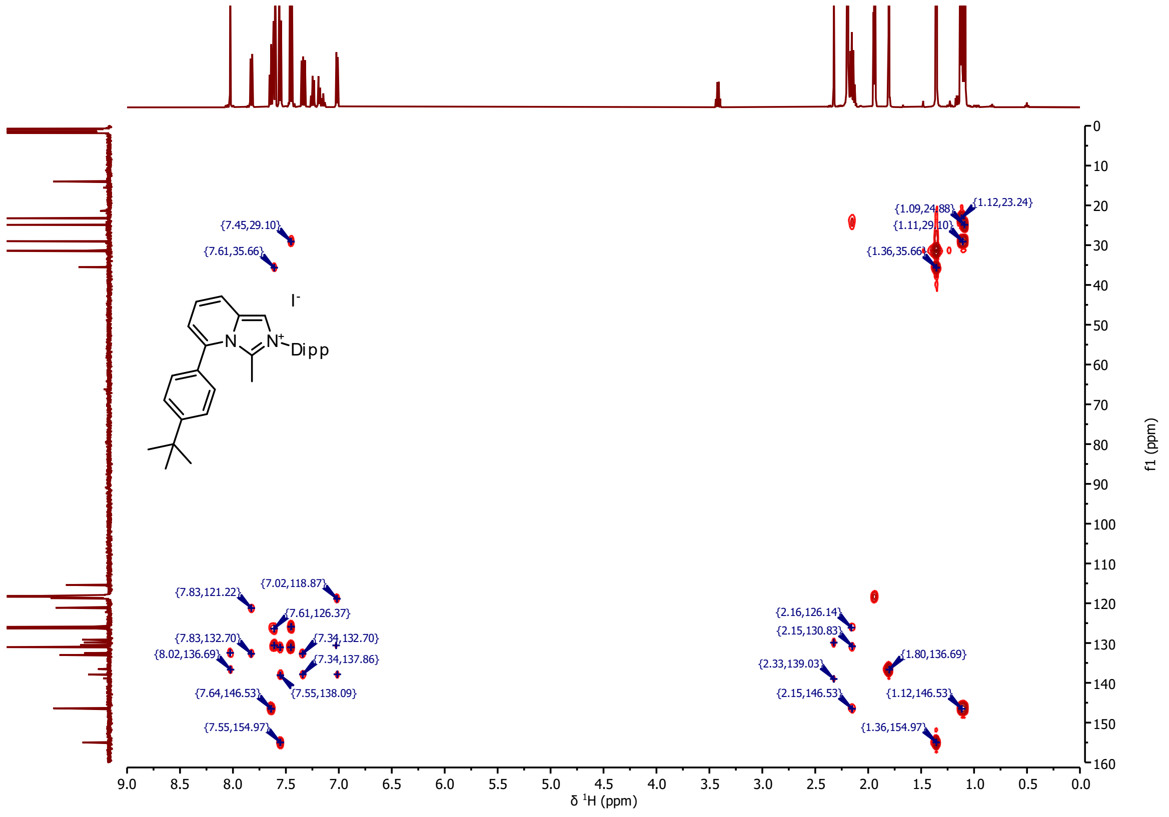

**Figure S44**: **$^{1}$H/$^{13}$C HMBC** (500/126 MHz, $CD_3CN$, 298 K) of **1d**.

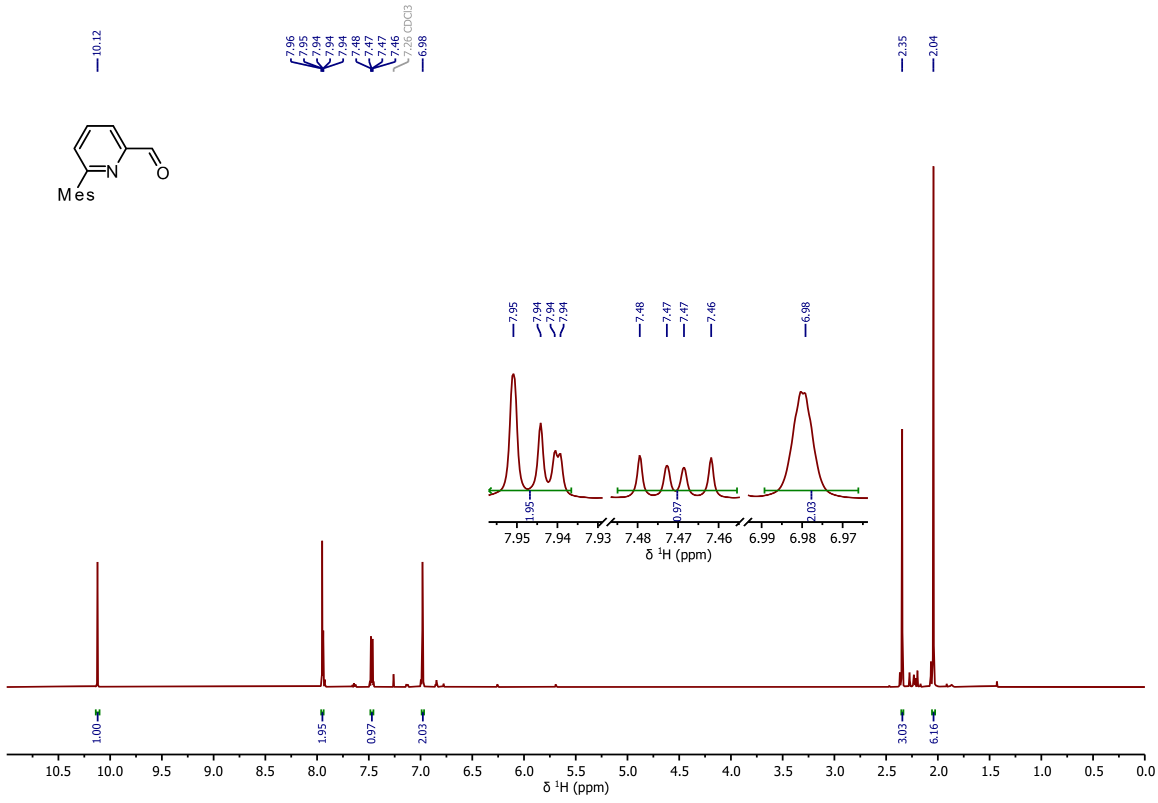


**Figure S45**: **$^{1}$H NMR** (500 MHz, $CDCl_3$, 298 K) of **S12**.

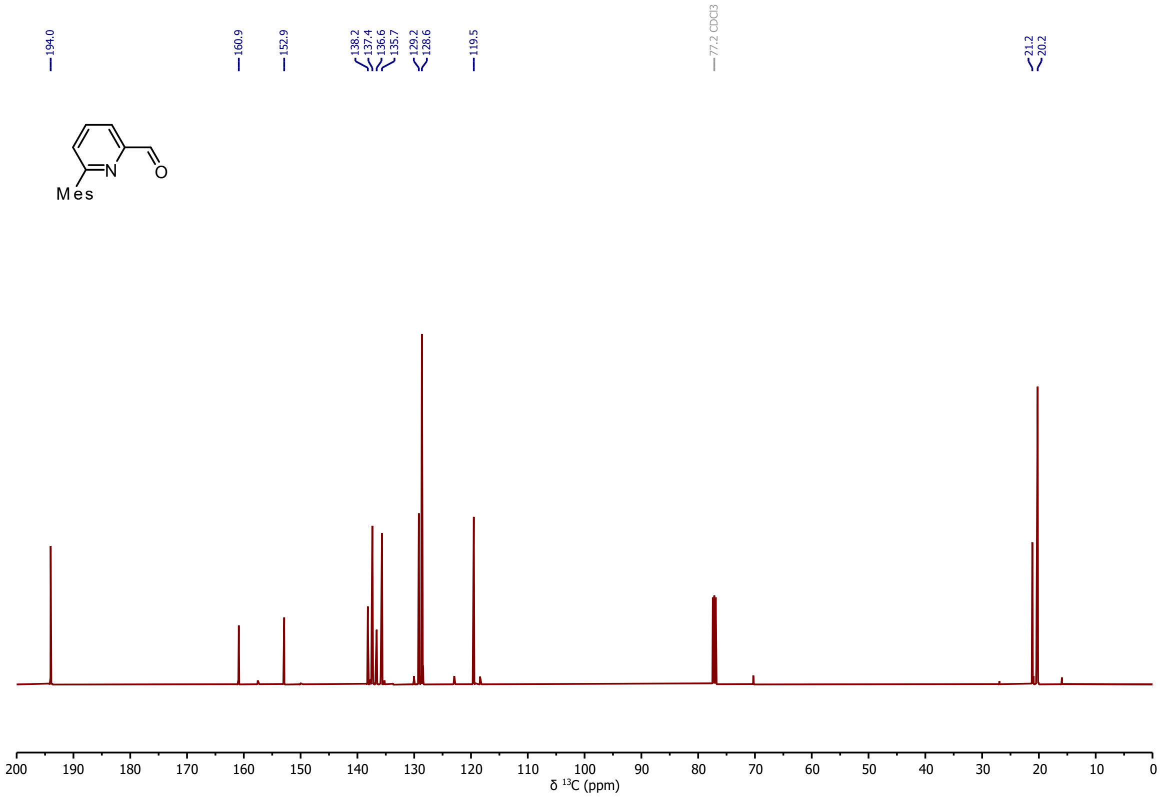


**Figure S46**: **$^{13}$C NMR** (126 MHz, $CDCl_3$, 298 K) of **S12**.

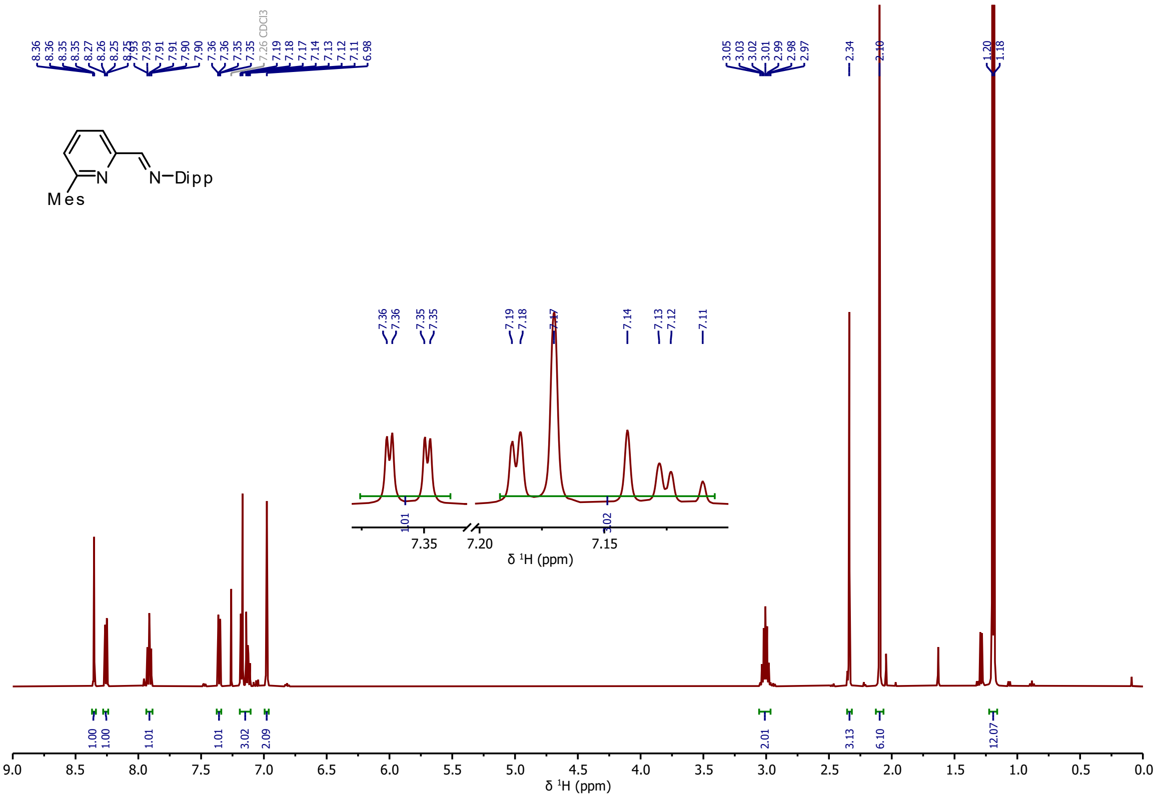

**Figure S47**: $^{1}$**H NMR** (500 MHz, $CDCl_3$, 298 K) of **S13**.

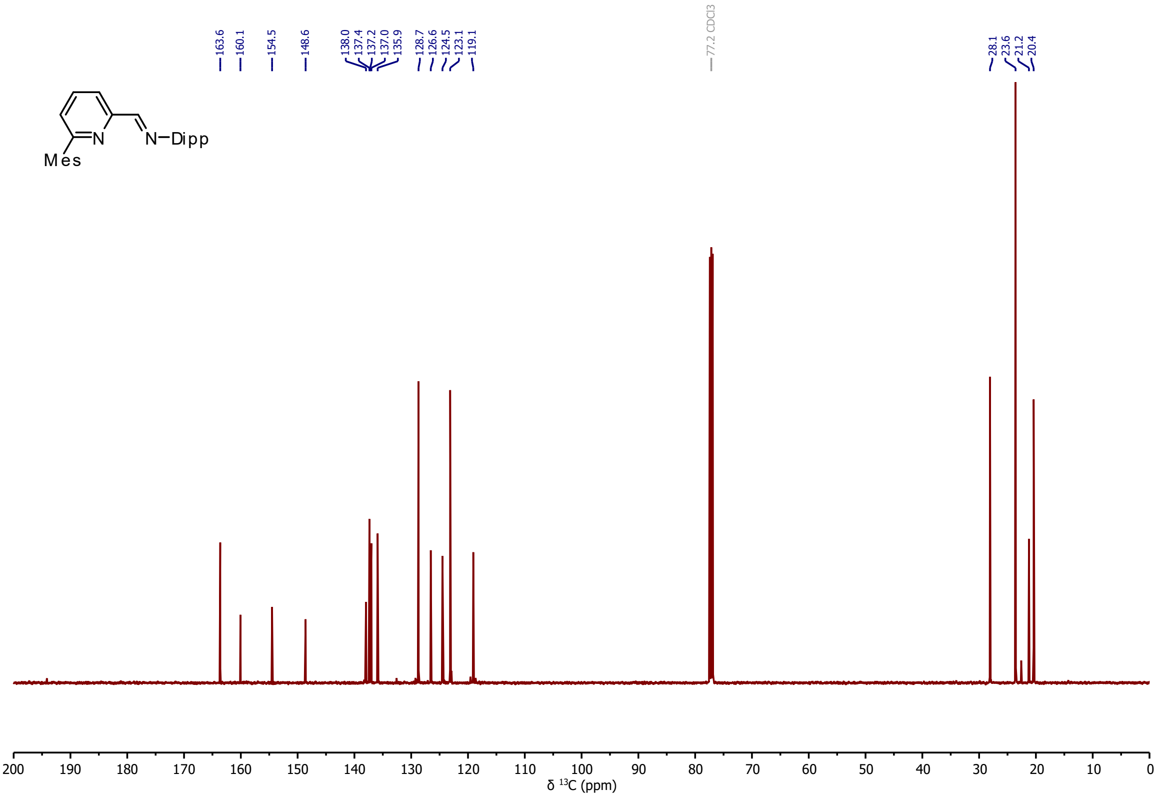

**Figure S48**: $^{13}$**C NMR** (126 MHz, $CDCl_3$, 298 K) of **S13**.

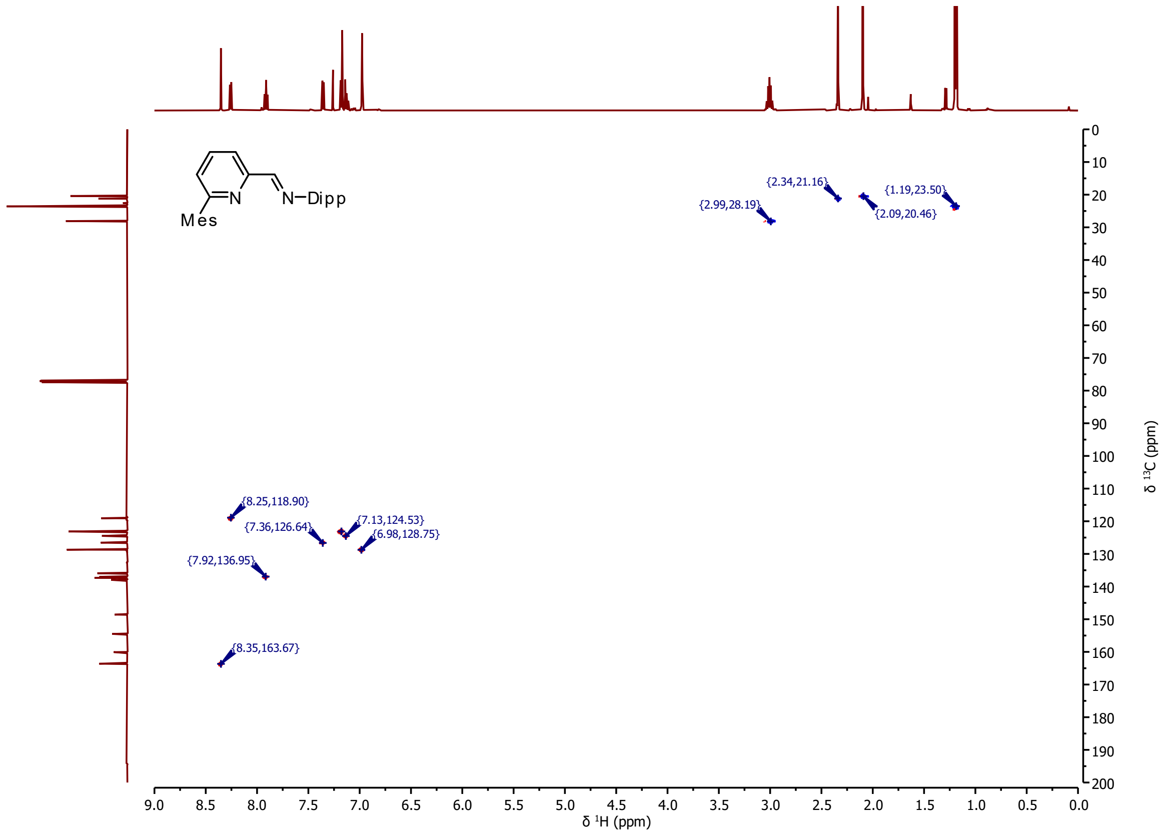


**Figure S49**: **$^{1}$H/$^{13}$C HSQC** (500/126 MHz, $CDCl_3$, 298 K) of **S13**.

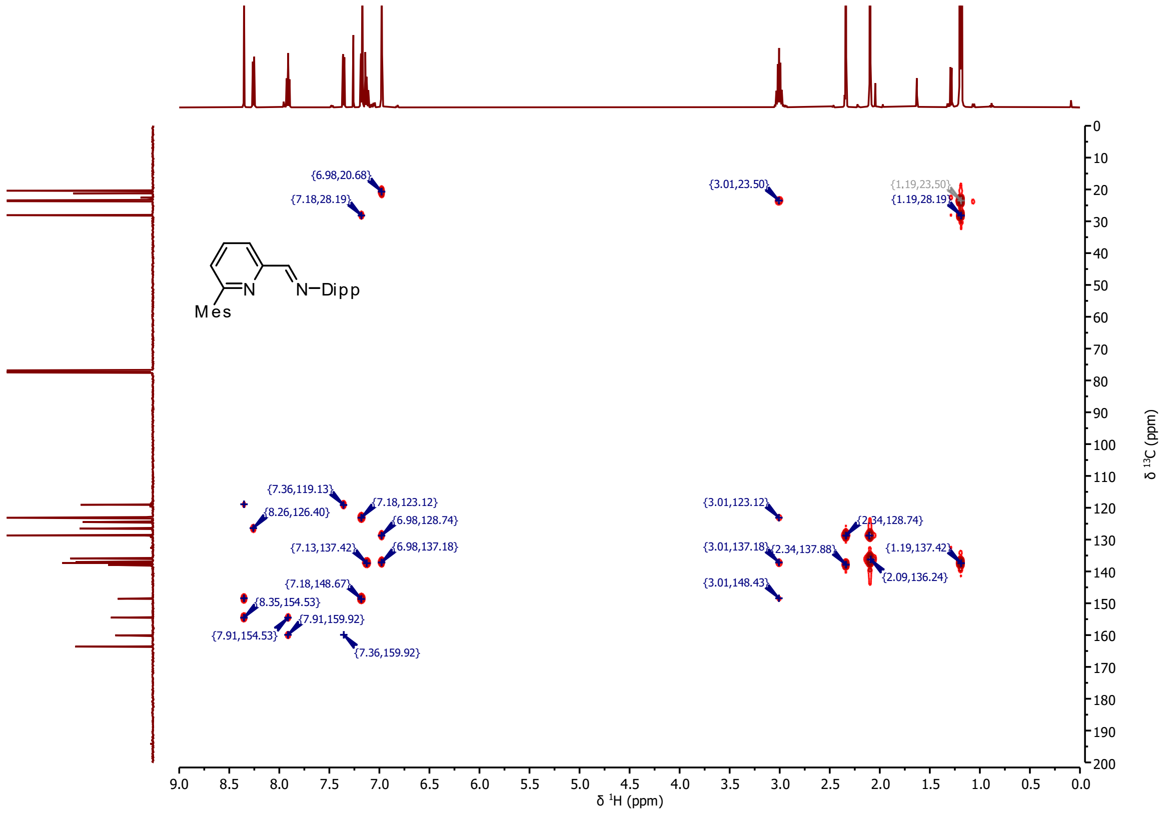


**Figure S50**: **$^{1}$H/$^{13}$C HMBC** (500/126 MHz, $CDCl_3$, 298 K) of **S13**.

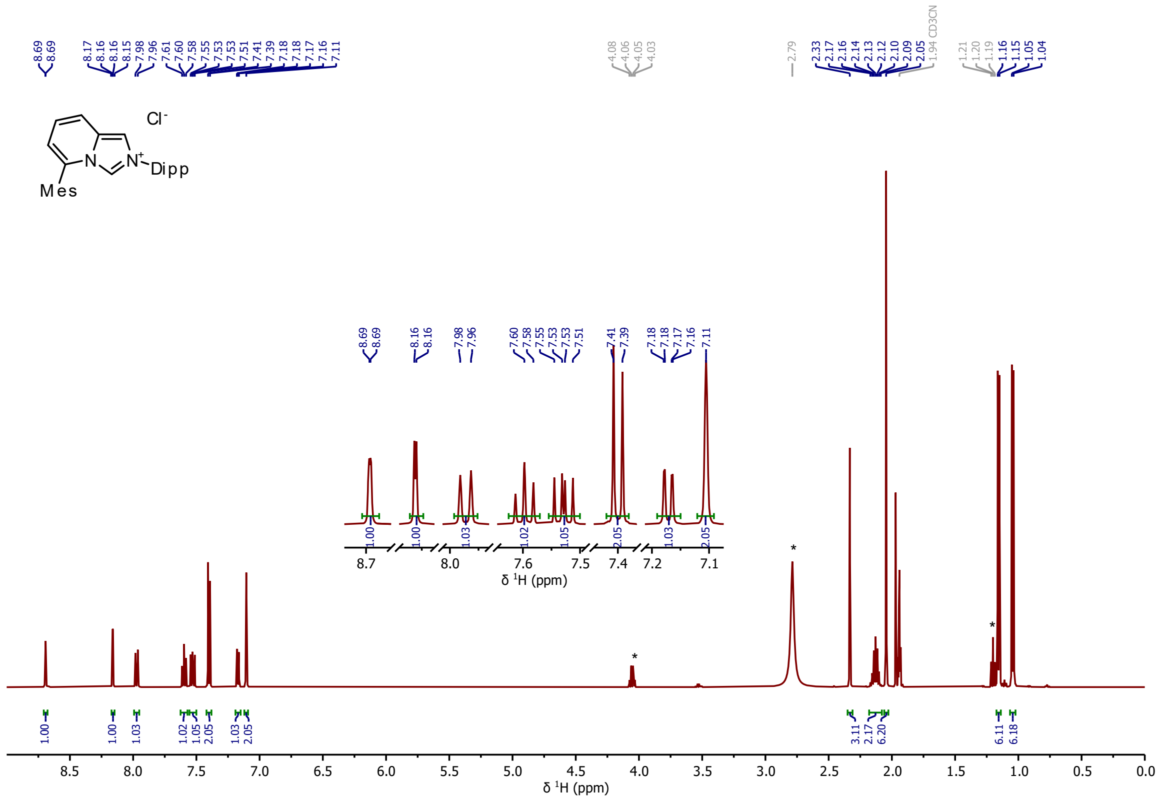

**Figure S51**: **$^1$H NMR** (500 MHz, $CD_3CN$, 298 K) of **S14**. * = $Et_2O$ and $H_2O$.

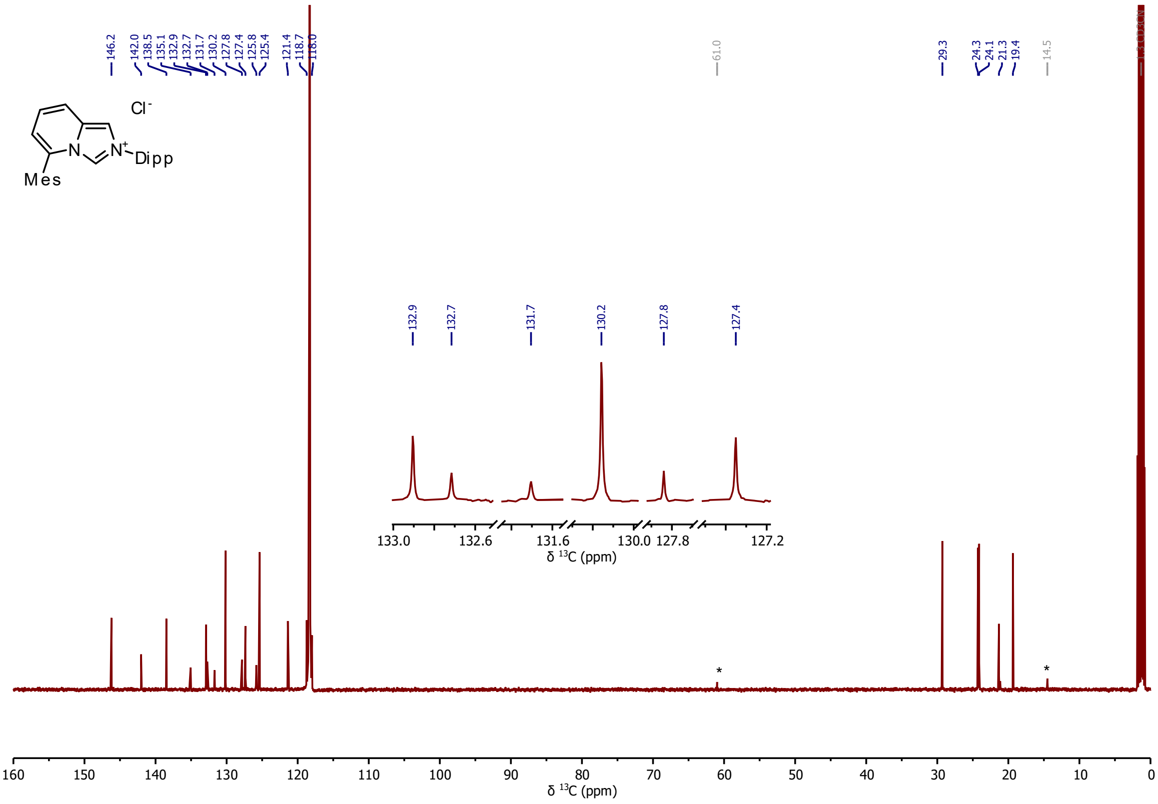

**Figure S52**: **$^{13}$C NMR** (126 MHz, $CD_3CN$, 298 K) of **S14**. * = $Et_2O$.

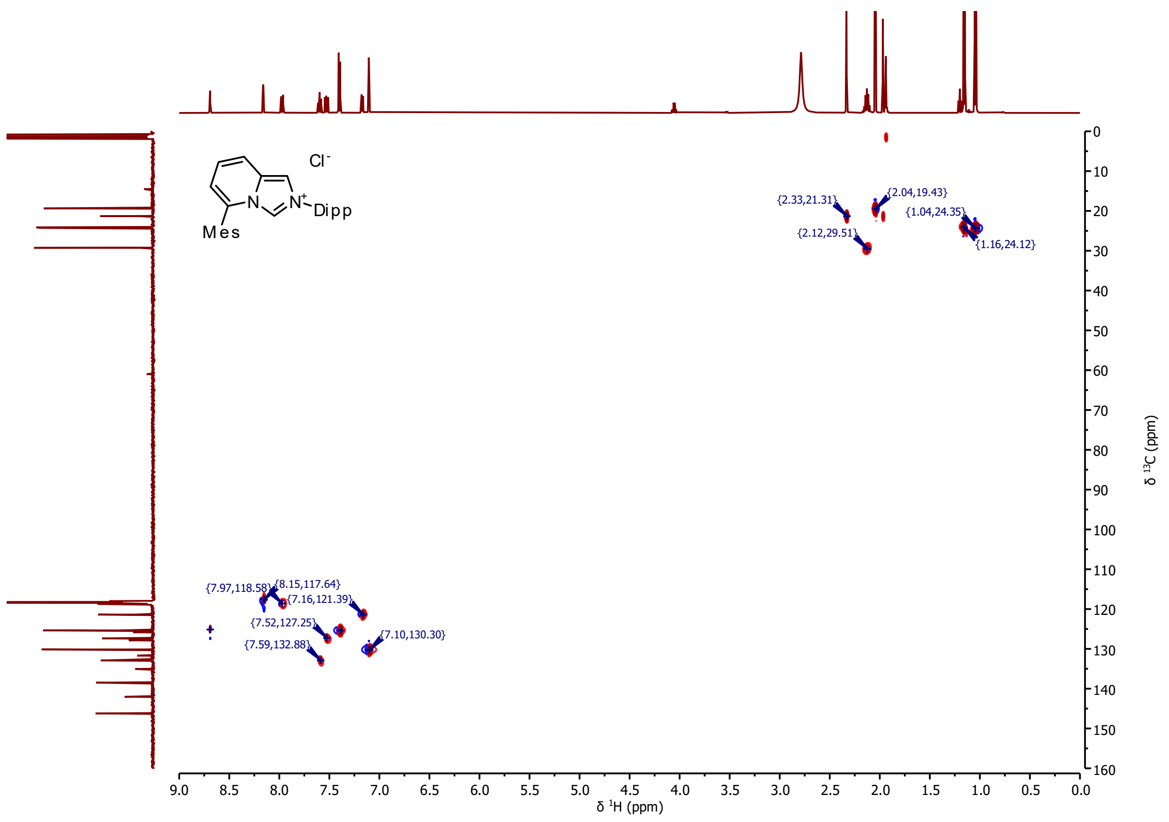

**Figure S53**: **$^{1}$H/$^{13}$C HSQC** (500/126 MHz, $CD_3CN$, 298 K) of **S14**.

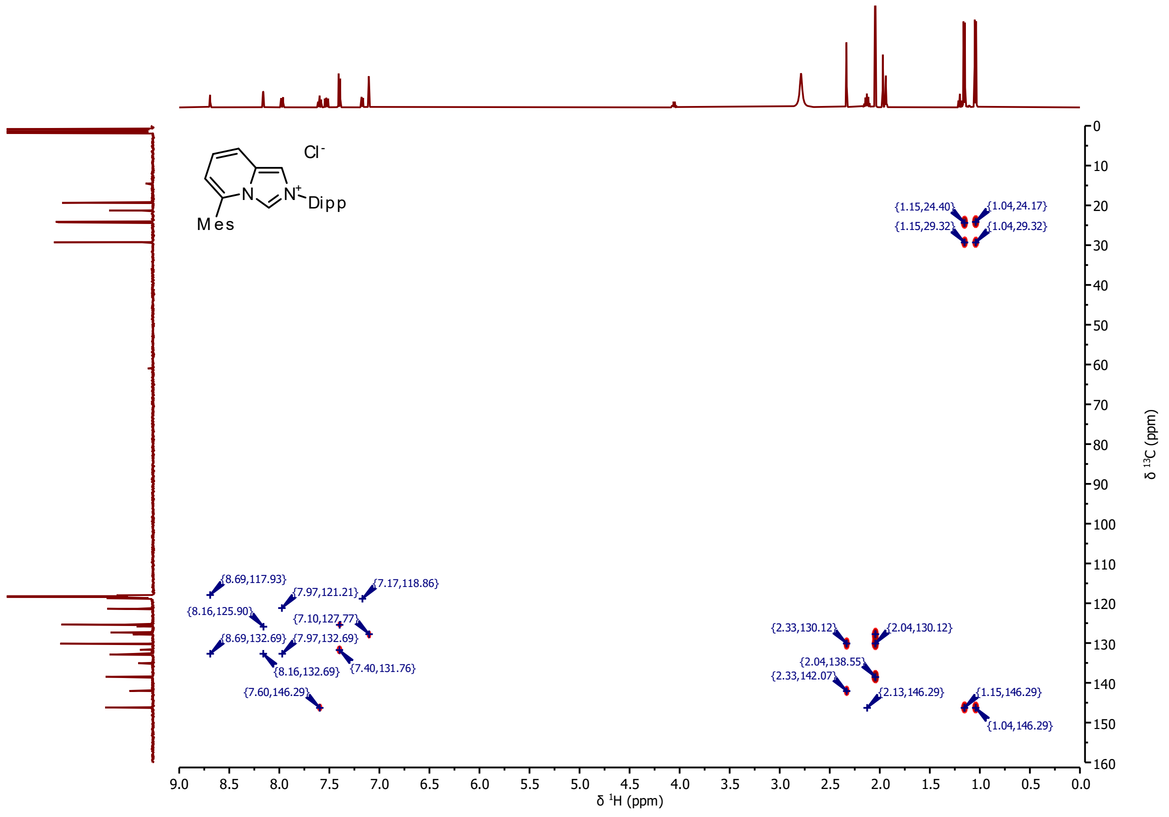

**Figure S54**: **$^{1}$H/$^{13}$C HMBC** (500/126 MHz, $CD_3CN$, 298 K) of **S14**.

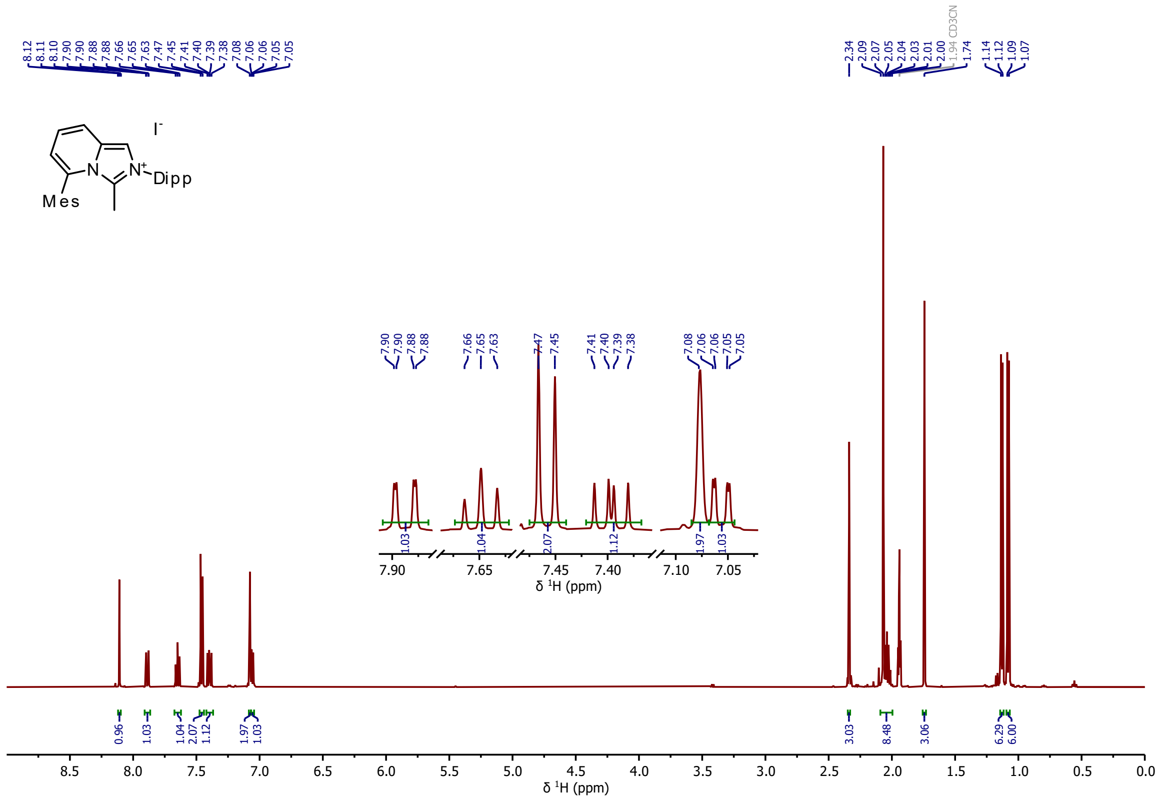

**Figure S55**: **$^{1}$H NMR** (500 MHz, $CD_3CN$, 298 K) of **1e**.

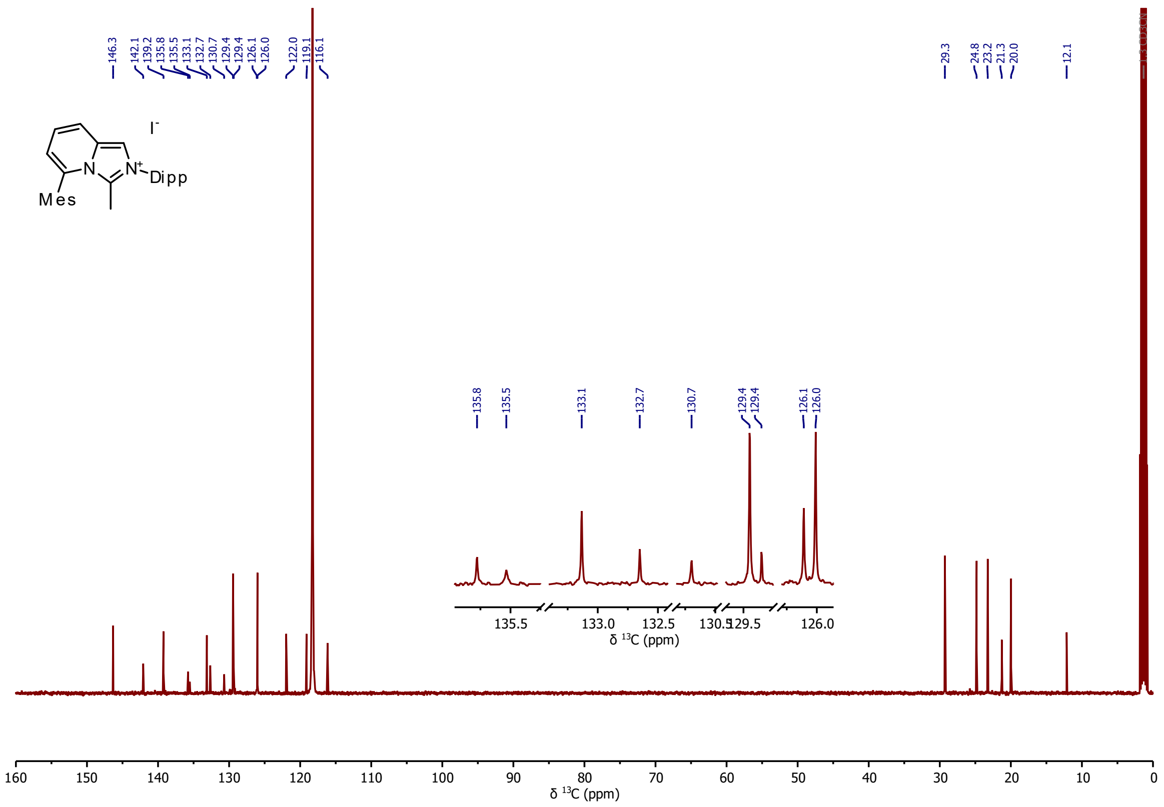

**Figure S56**: **$^{13}$C NMR** (126 MHz, $CD_3CN$, 298 K) of **1e**.

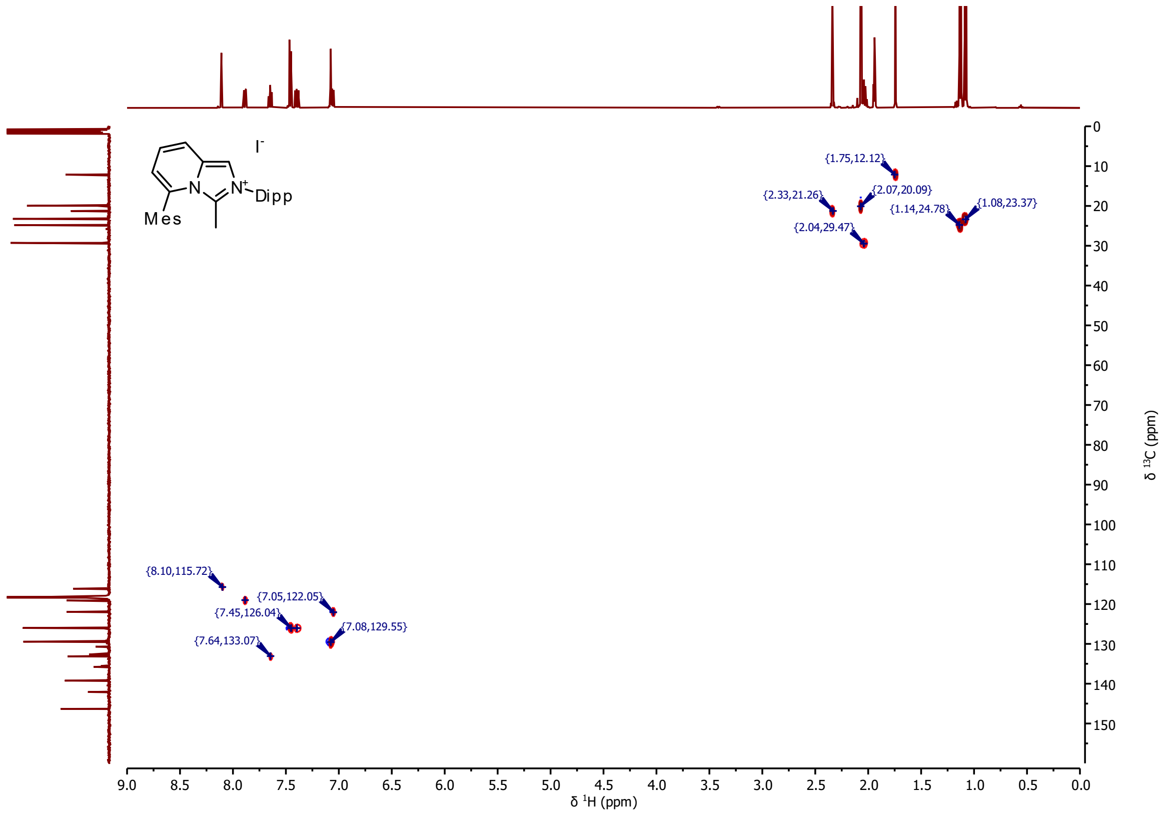


**Figure S57**: **$^{1}$H/$^{13}$C HSQC** (500/126 MHz, $CD_3CN$, 298 K) of **1e**.

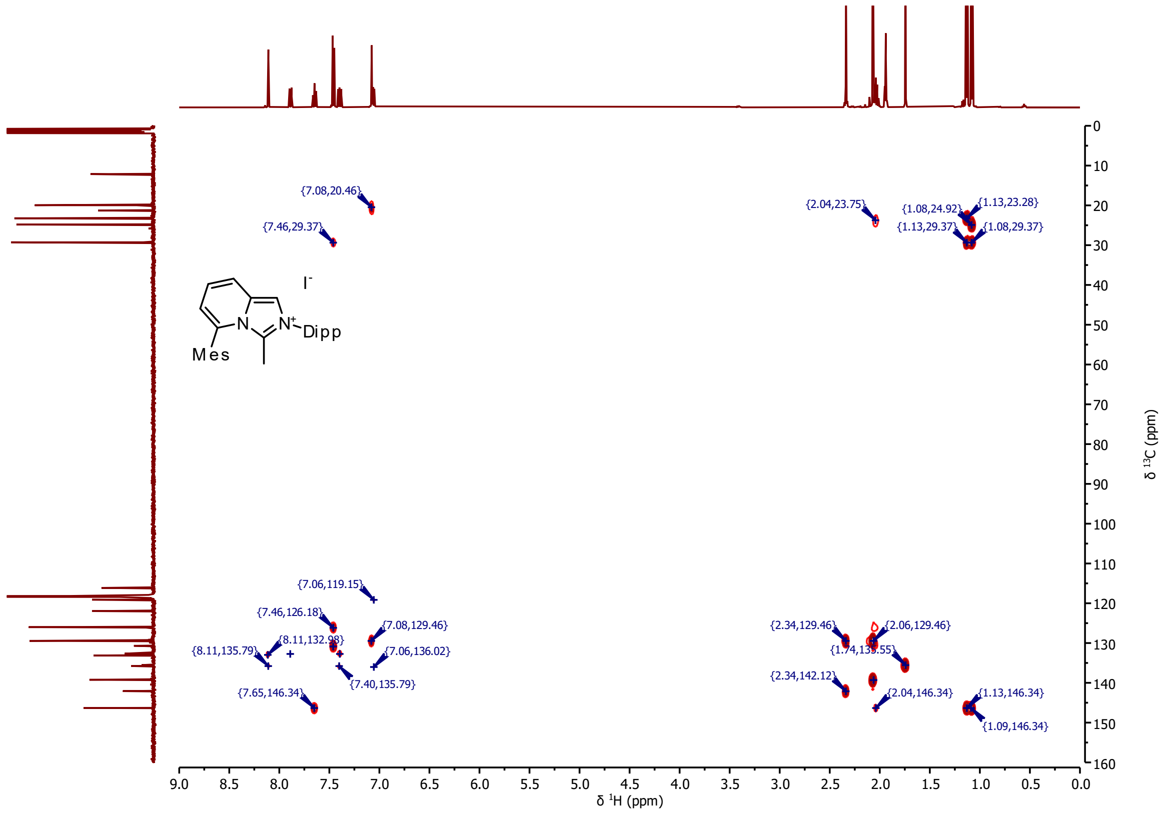


**Figure S58**: **$^{1}$H/$^{13}$C HMBC** (500/126 MHz, $CD_3CN$, 298 K) of **1e**.

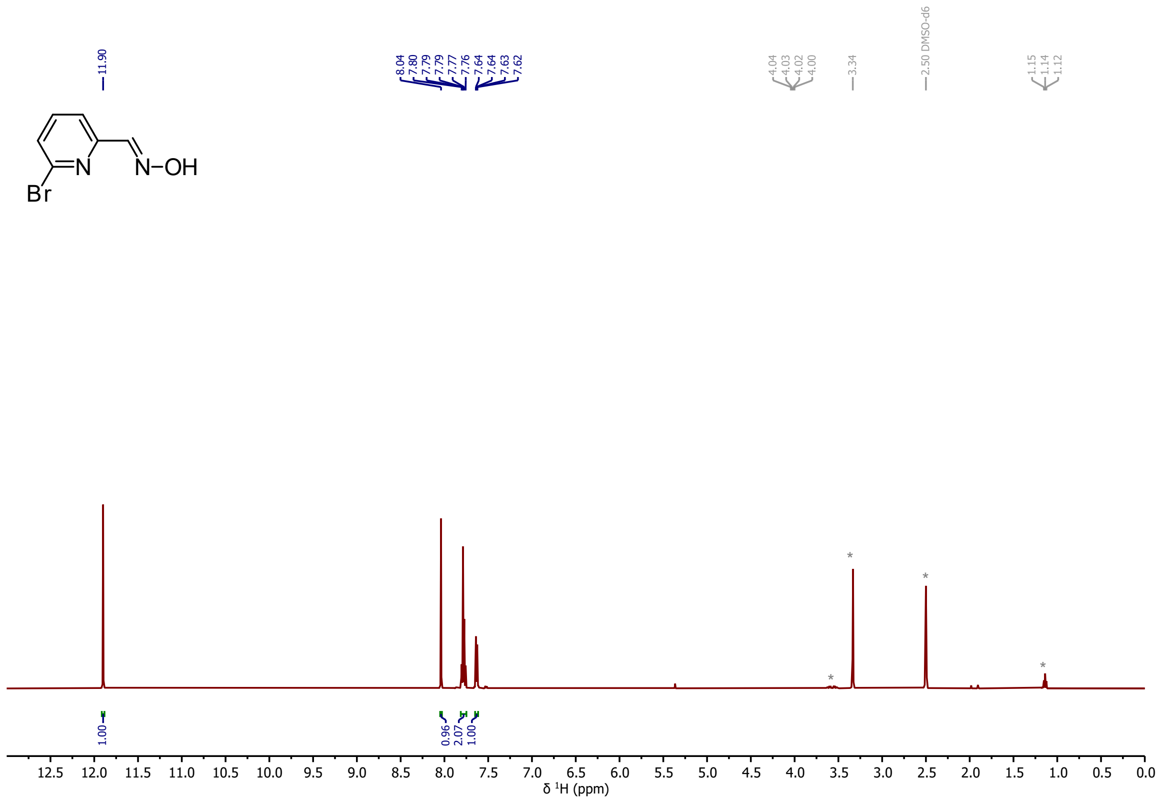

**Figure S59**: **$^{1}$H NMR** (500 MHz, DMSO-d$_6$, 298 K) of **S15**. * = $H_2O$ and EtOAc.

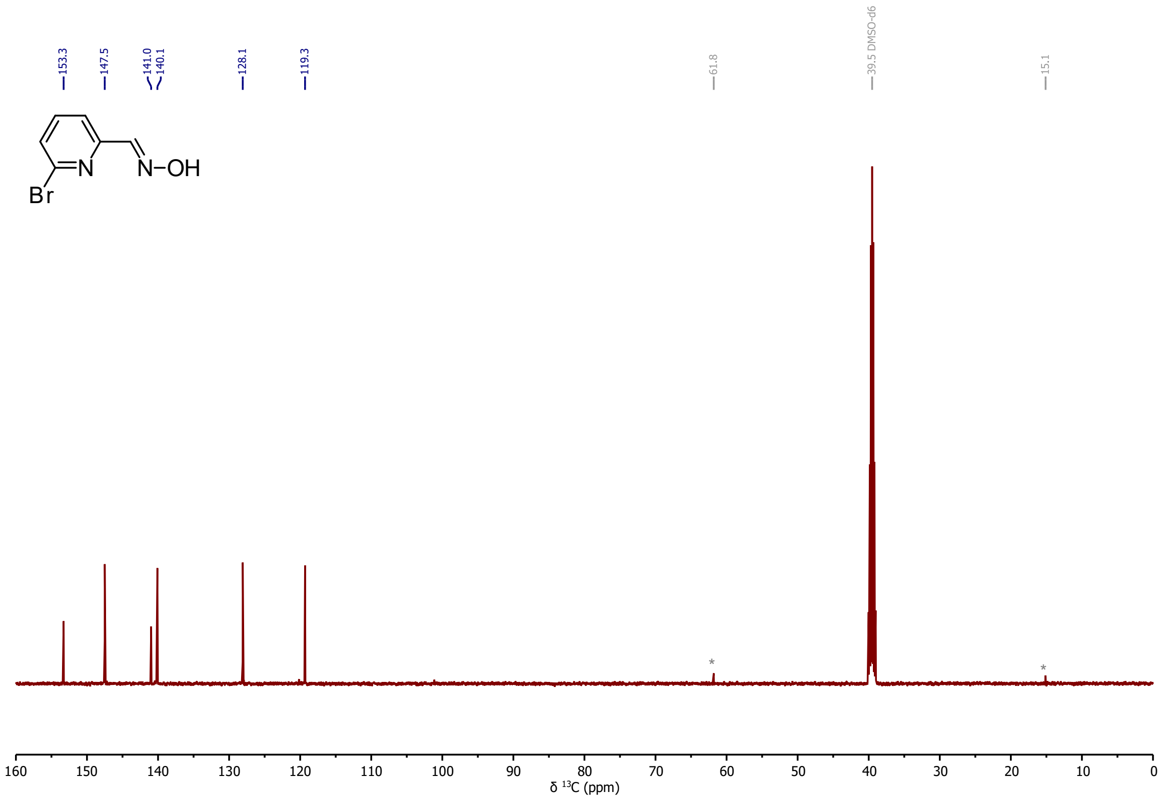

**Figure S60**: **$^{13}$C NMR** (126 MHz, DMSO, 298 K) of **S15**. * = EtOAc.

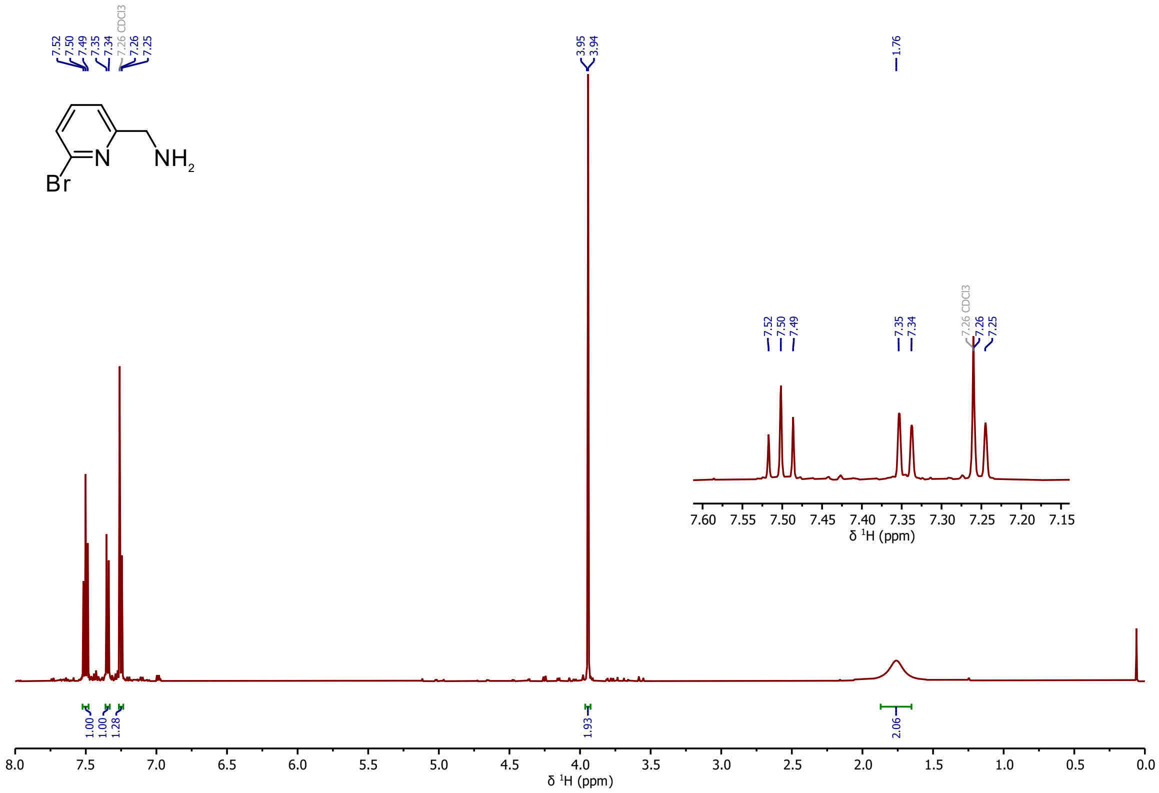


**Figure S61**: **$^{1}$H NMR** (500 MHz, $CDCl_3$, 298 K) of **S16**.

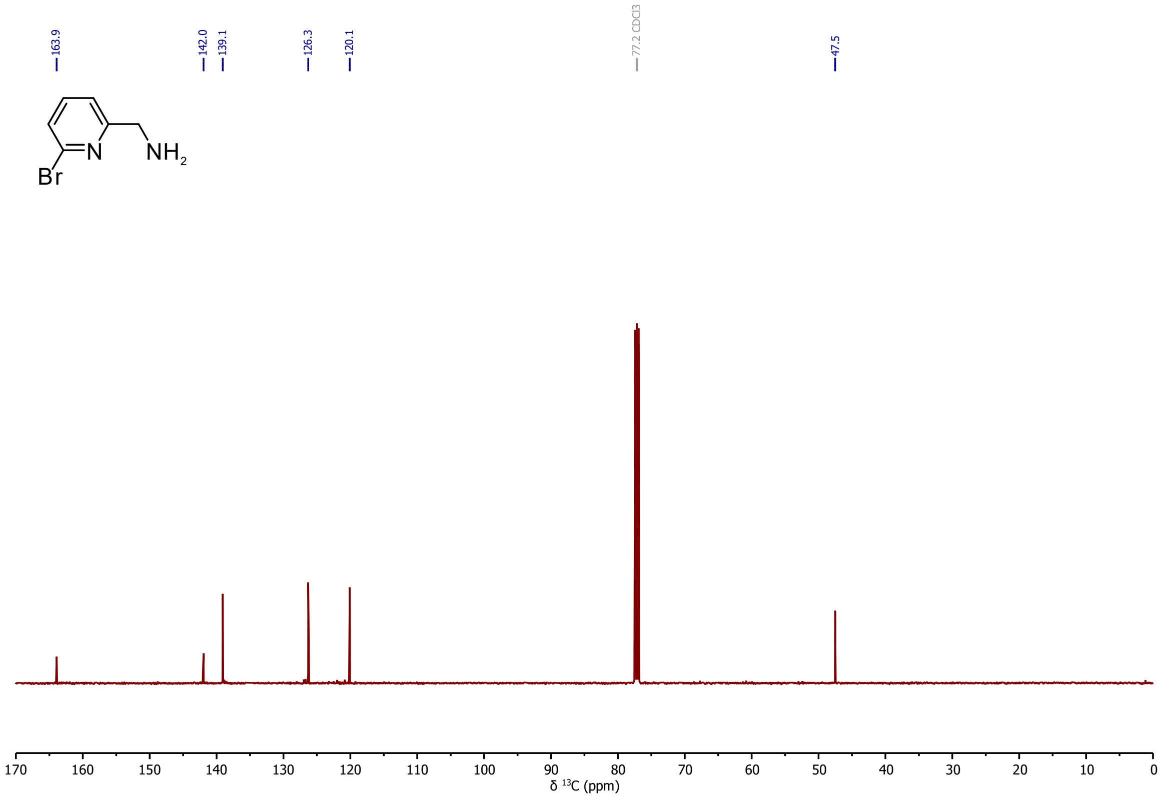


**Figure S62**: **$^{13}$C NMR** (126 MHz, $CDCl_3$, 298 K) of **S16**.

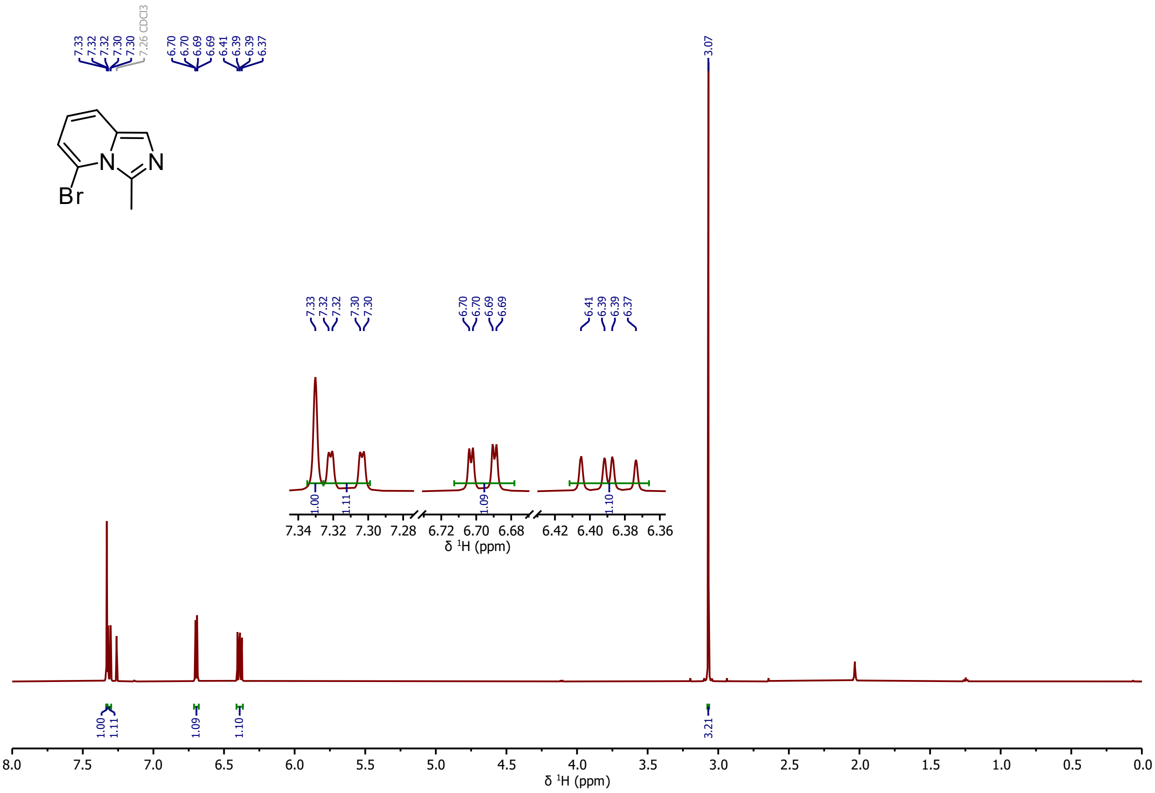


**Figure S63**: **$^{1}$H NMR** (500 MHz, $CDCl_3$, 298 K) of **S17**.

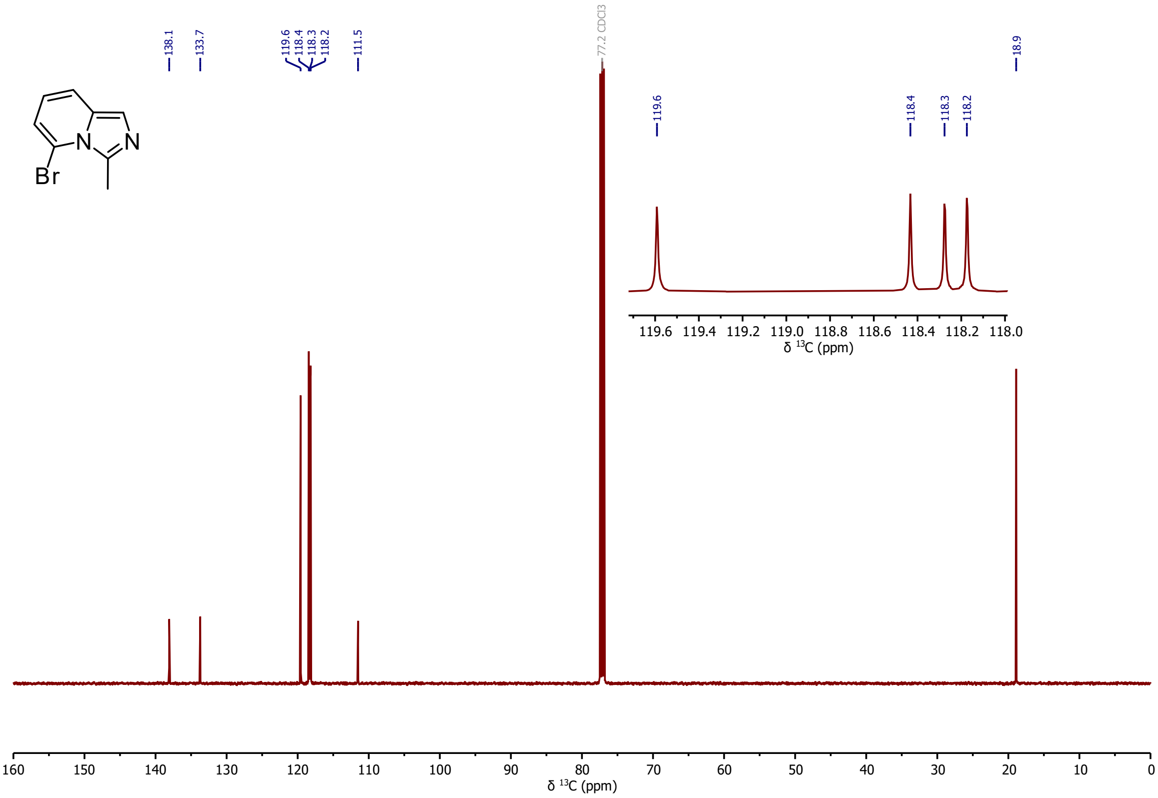


**Figure S64**: **$^{13}$C NMR** (126 MHz, $CDCl_3$, 298 K) of **S17**.

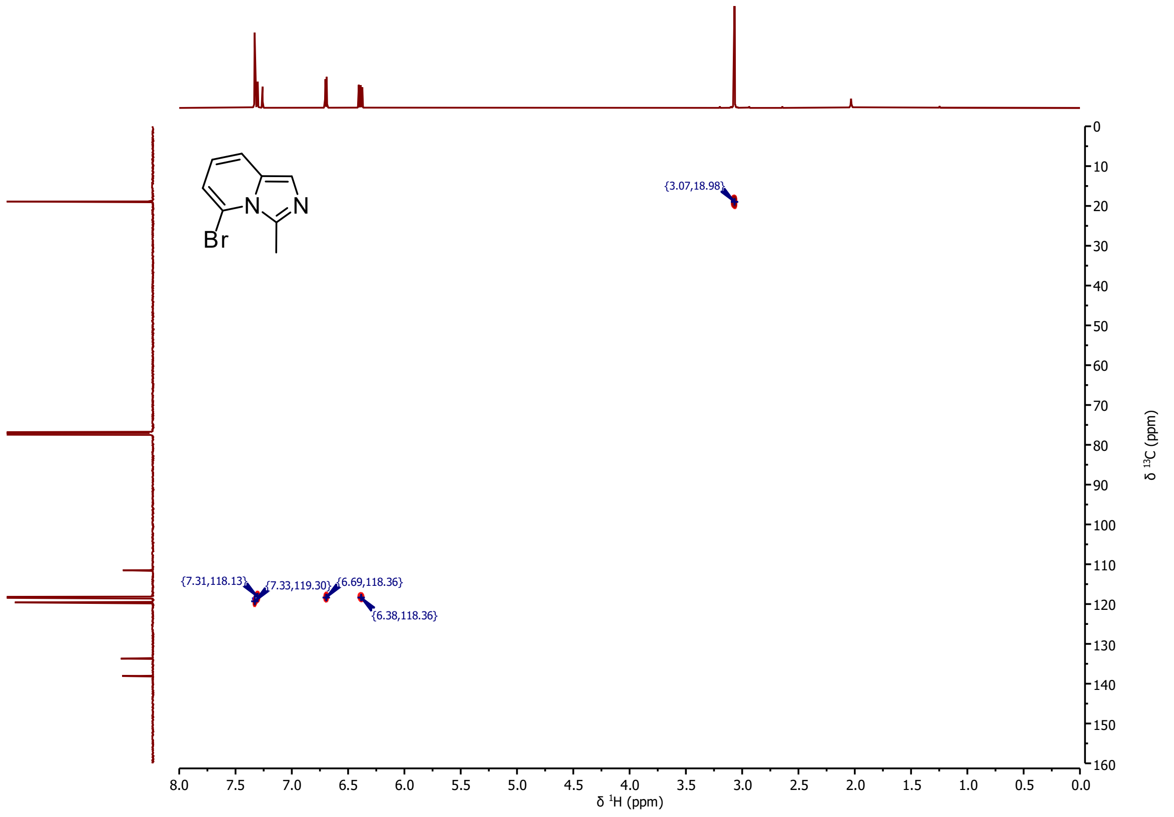


**Figure S65**: **$^1$H/$^{13}$C HSQC** (500/126 MHz, $CDCl_3$, 298 K) of **S17**.

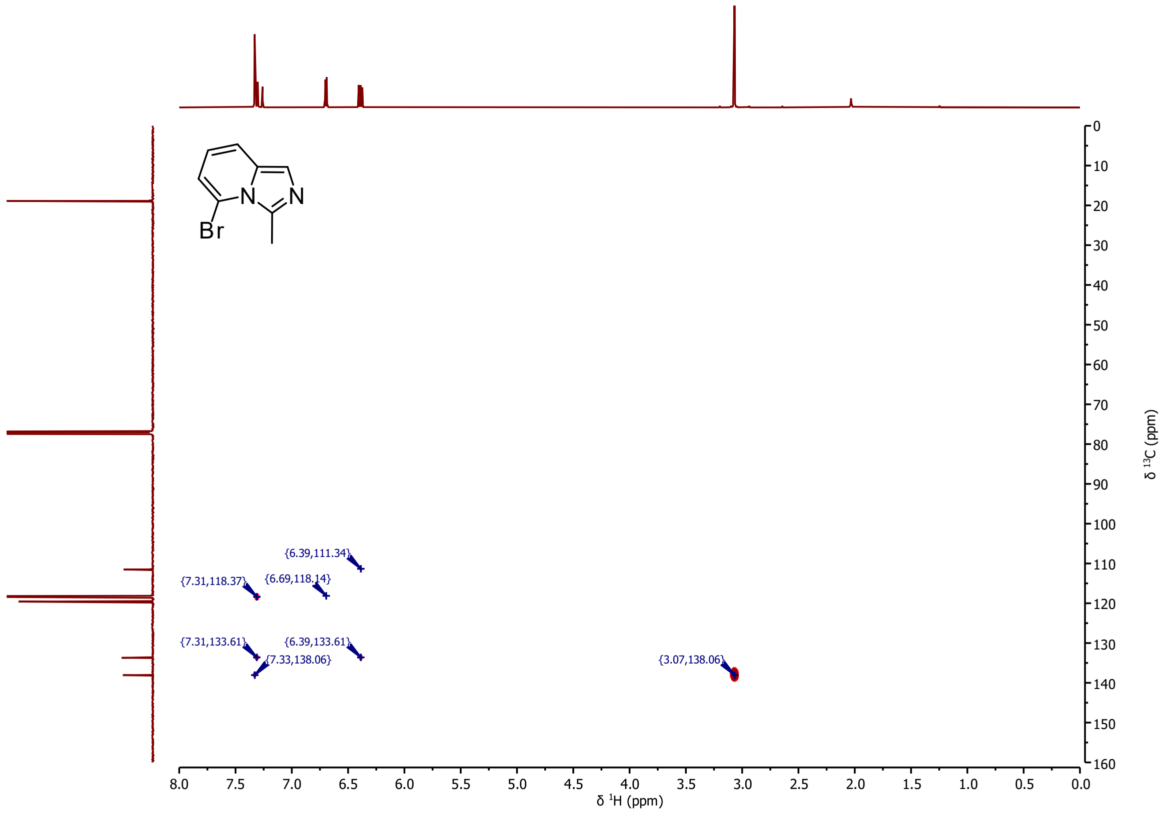


**Figure S66**: **$^1$H/$^{13}$C HMBC** (500/126 MHz, $CDCl_3$, 298 K) of **S17**.

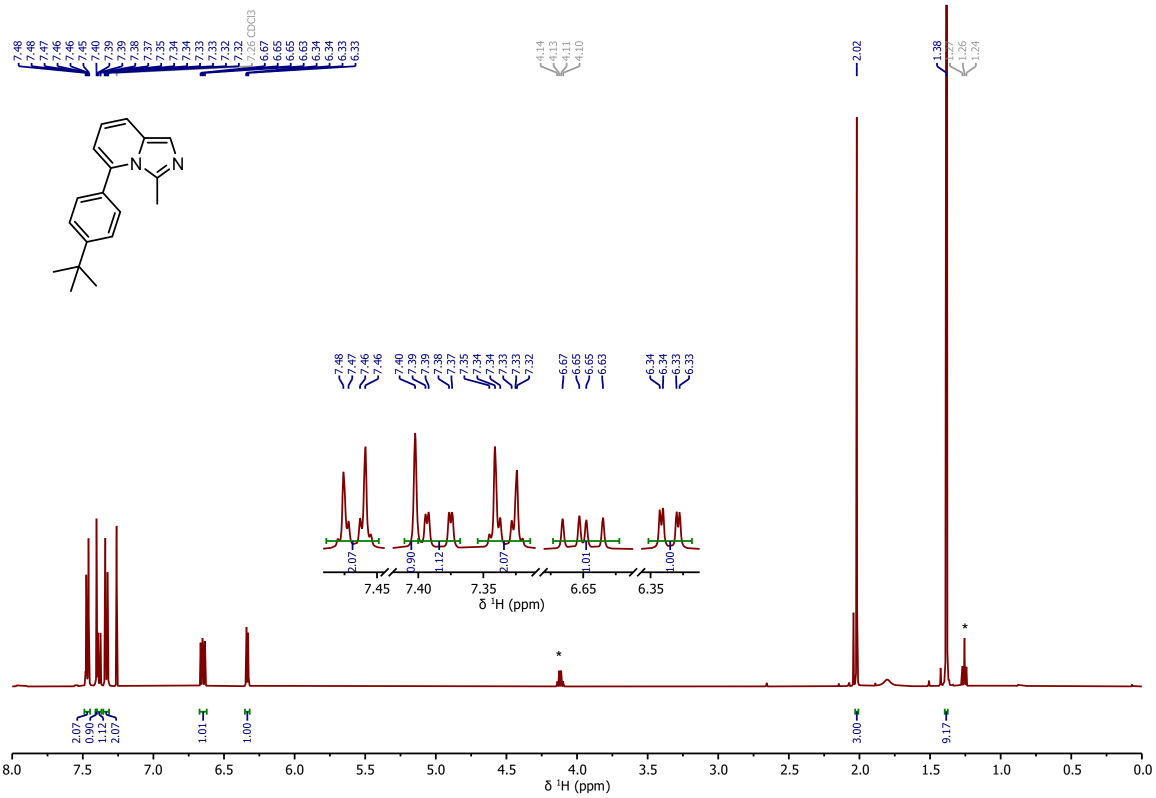


**Figure S67**: **$^{1}$H NMR** (500 MHz, $CDCl_3$, 298 K) of **S18**. * = EtOAc.

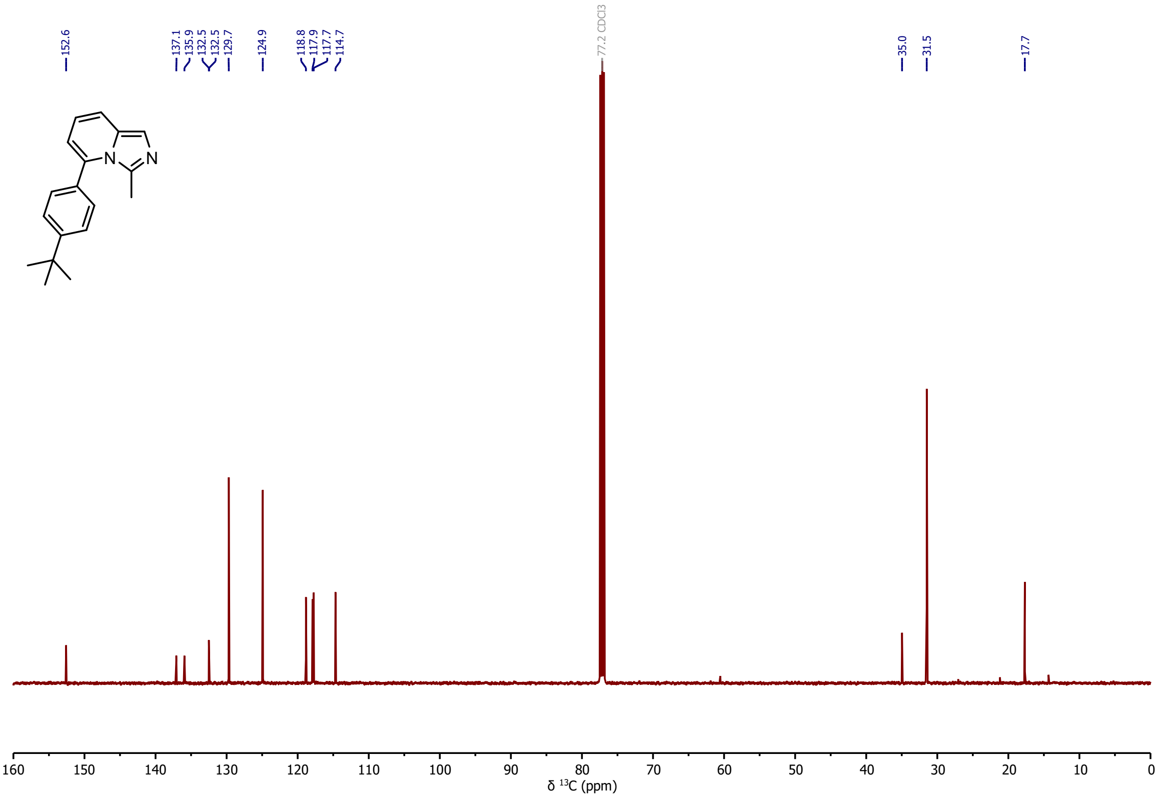


**Figure S68**: **$^{13}$C NMR** (126 MHz, $CDCl_3$, 298 K) of **S18**.

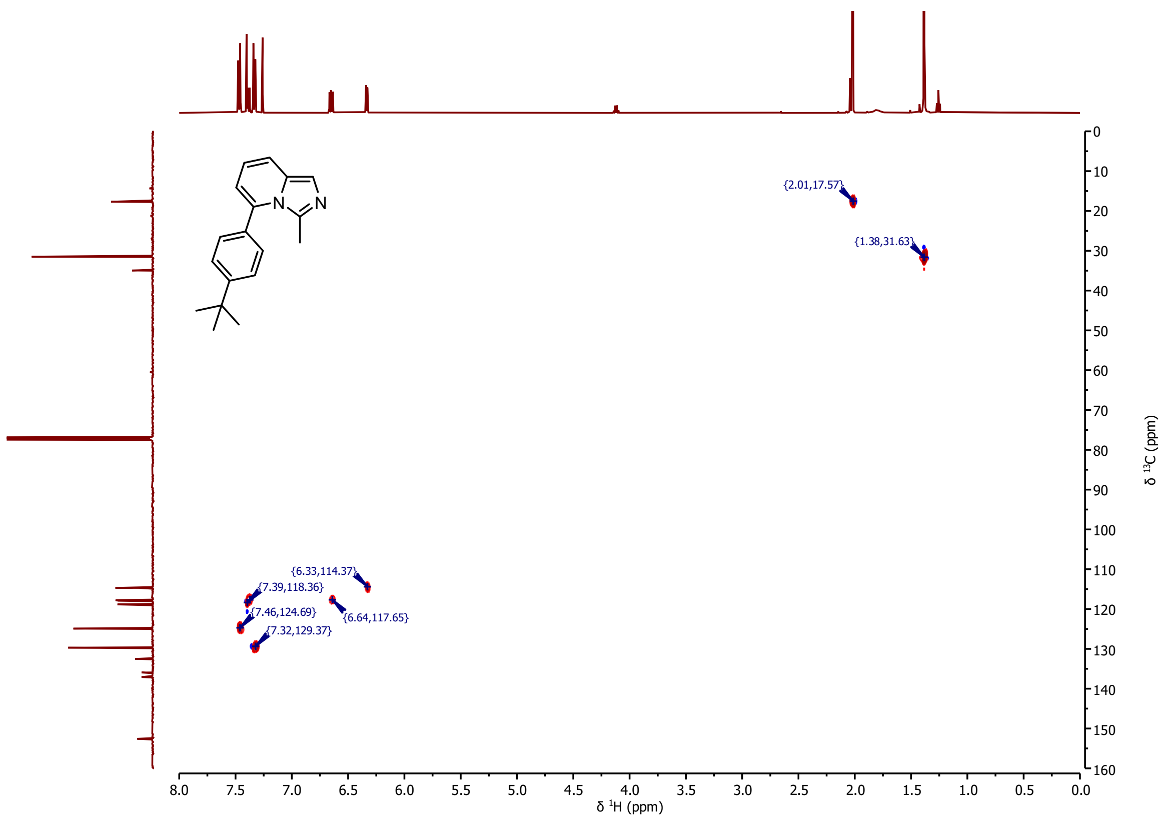


**Figure S69**: **$^{1}H/^{13}C$ HSQC** (500/126 MHz, $CDCl_3$, 298 K) of **S18**.

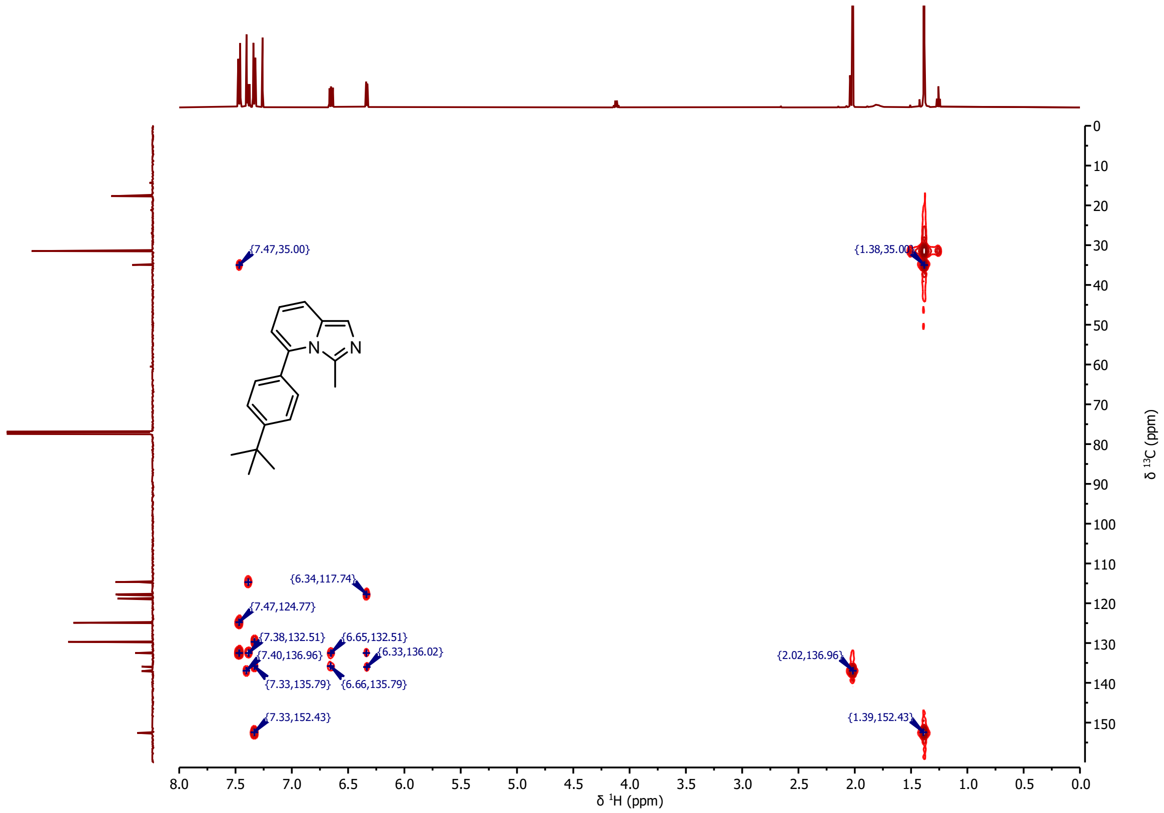


**Figure S70**: **$^{1}H/^{13}C$ HMBC** (500/126 MHz, $CDCl_3$, 298 K) of **S18**.

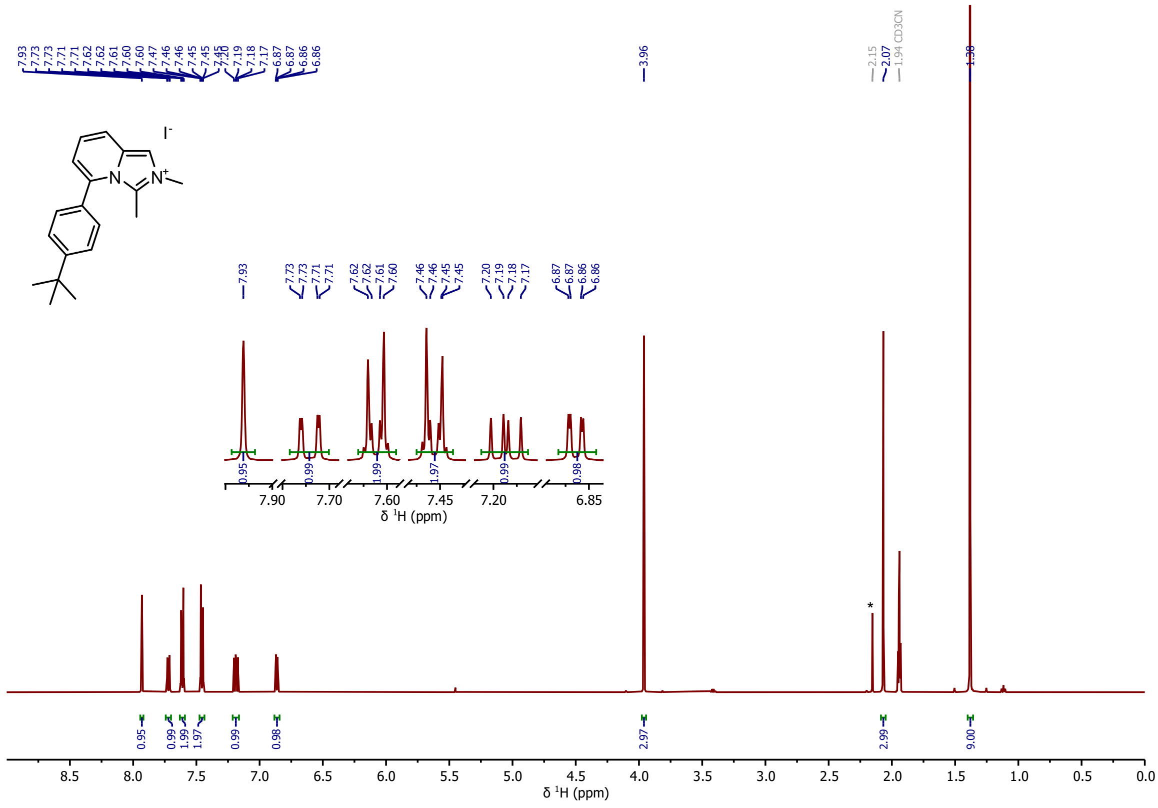

**Figure S71**: **$^1$H NMR** (500 MHz, $CD_3CN$, 298 K) of **1f**. * = $H_2O$.

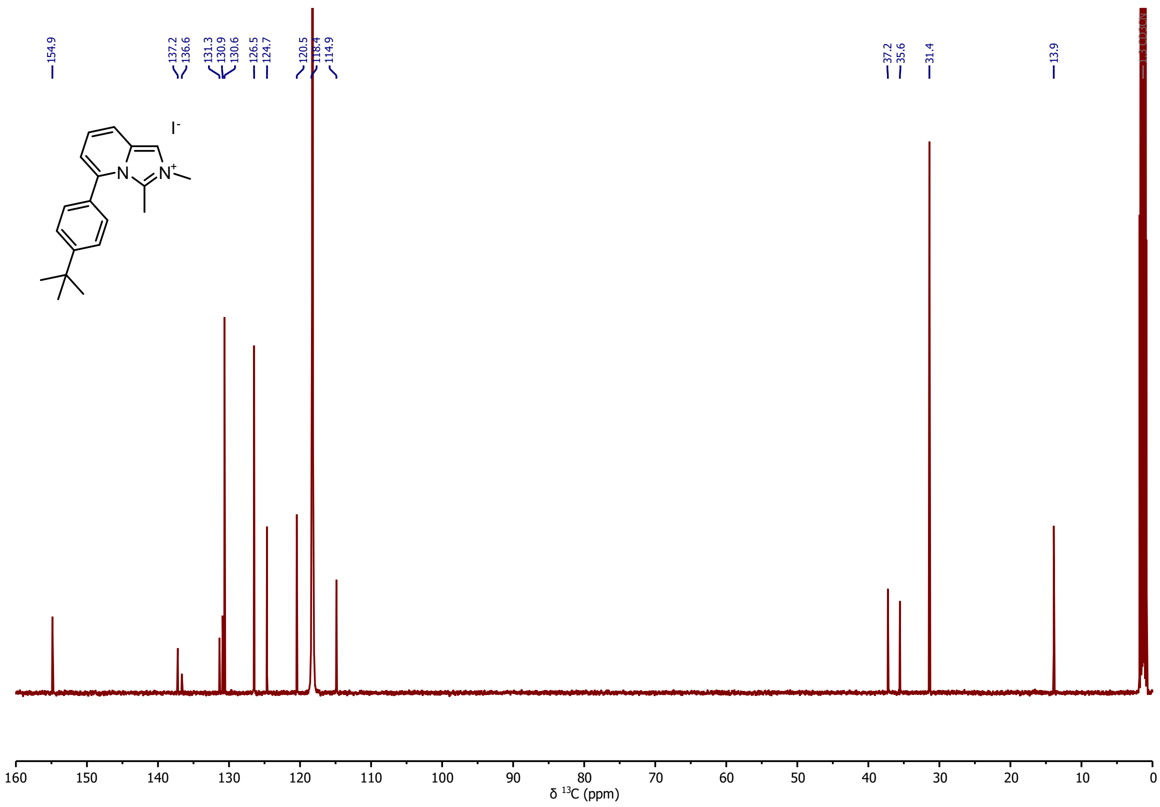

**Figure S72**: **$^{13}$C NMR** (126 MHz, $CD_3CN$, 298 K) of **1f**.

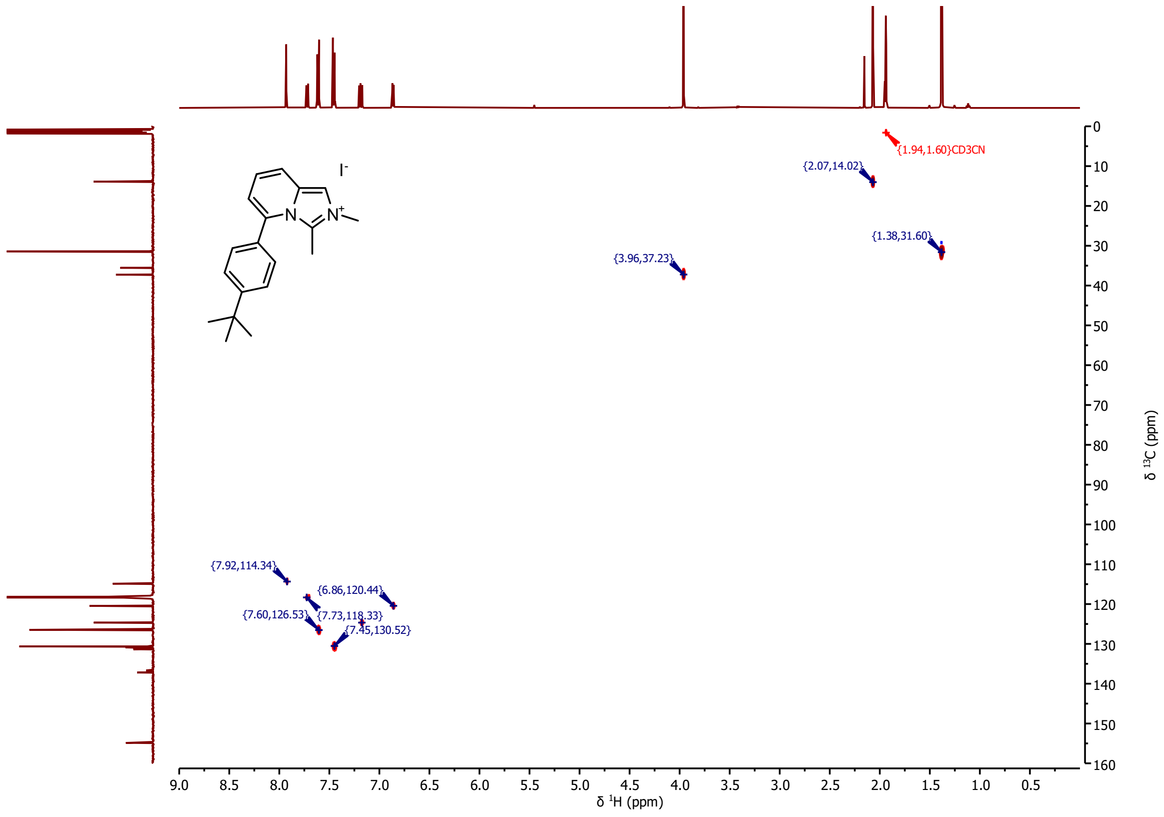


**Figure S73**: **$^{1}H/^{13}C$ HSQC** (500/126 MHz, $CD_3CN$, 298 K) of **1f**.

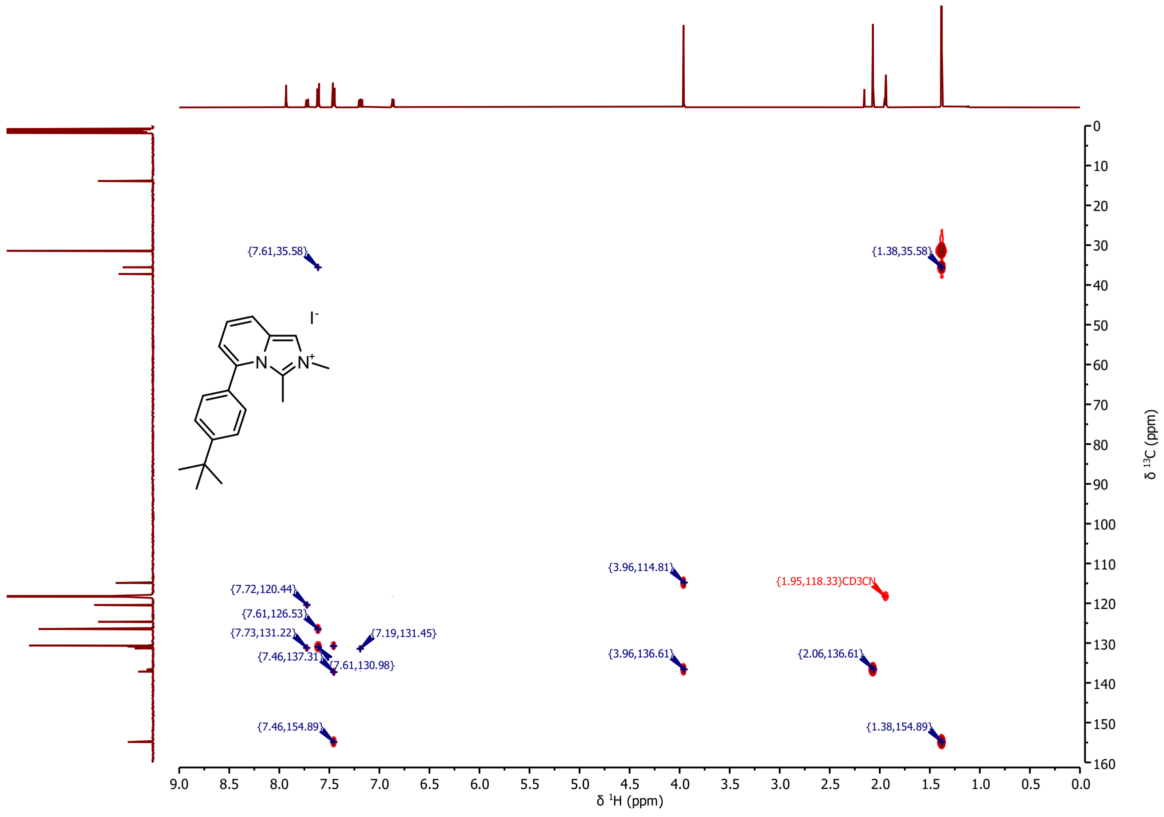


**Figure S74**: **$^{1}H/^{13}C$ HMBC** (500/126 MHz, $CD_3CN$, 298 K) of **1f**.

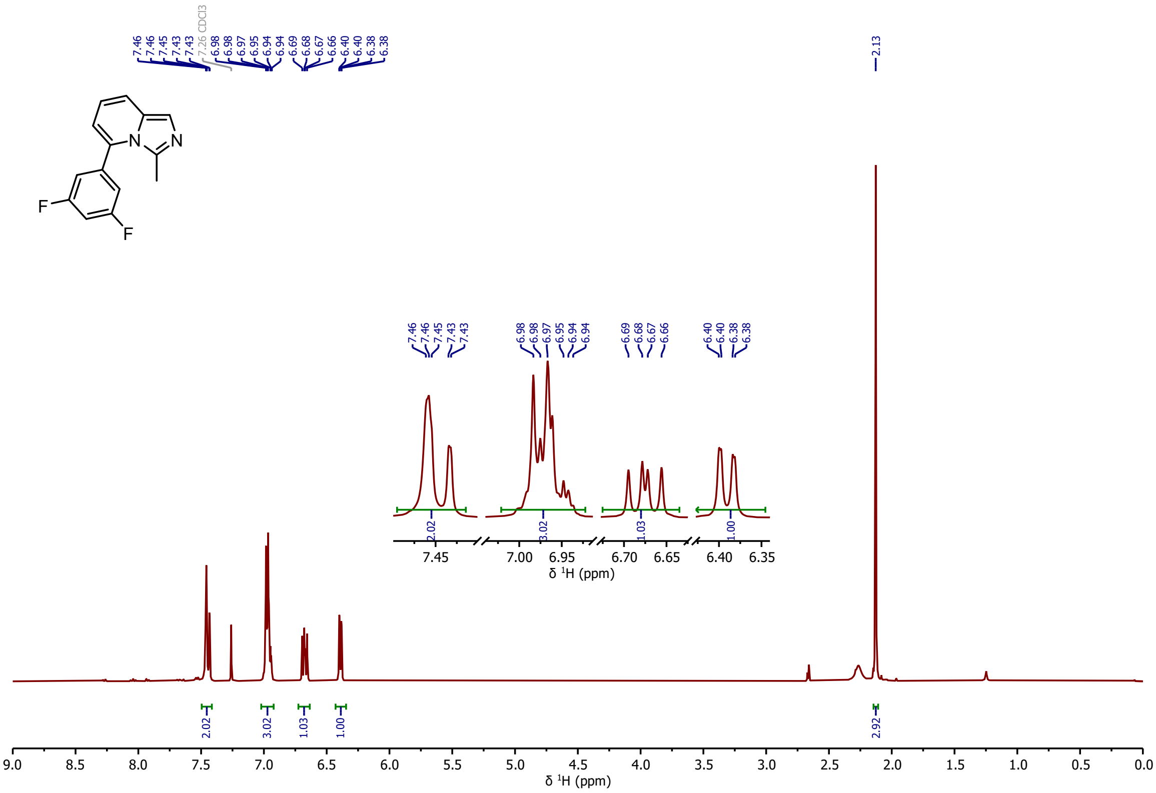

**Figure S75**: **$^{1}$H NMR** (400 MHz, $CDCl_3$, 298 K) of **S19**.

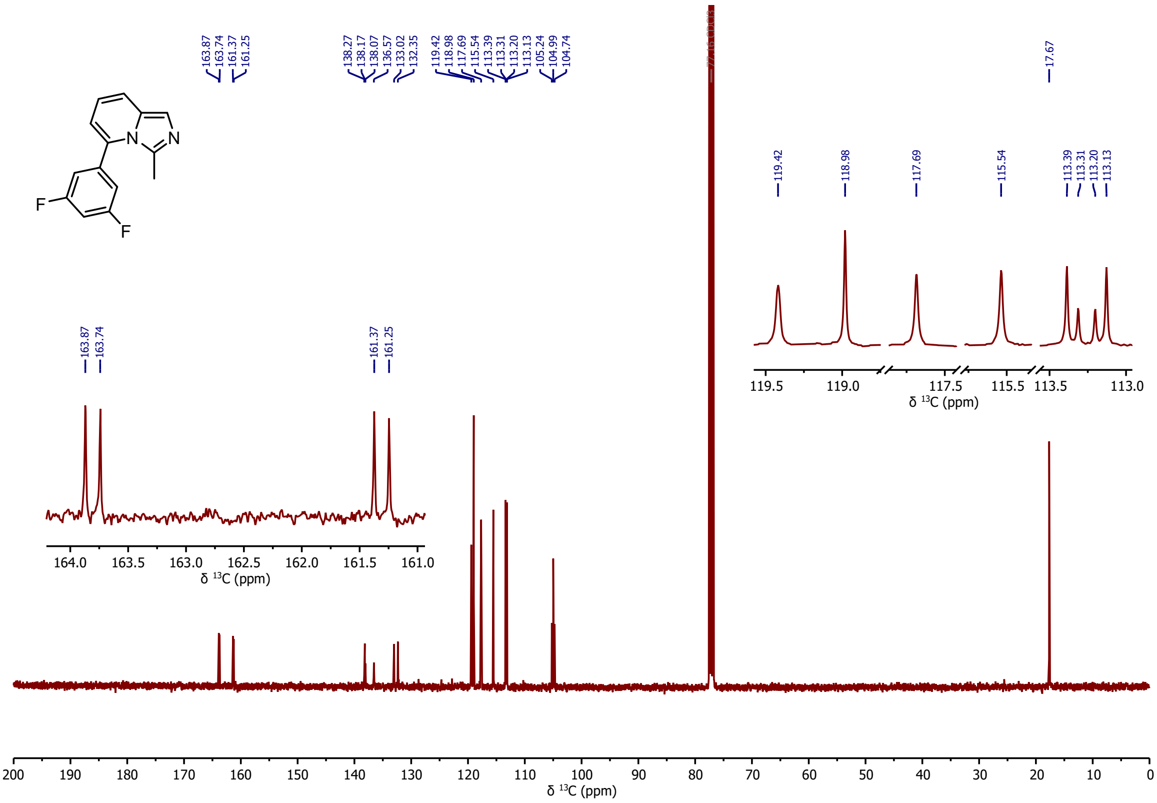

**Figure S76**: **$^{13}$C NMR** (101 MHz, $CDCl_3$, 298 K) of **S19**.

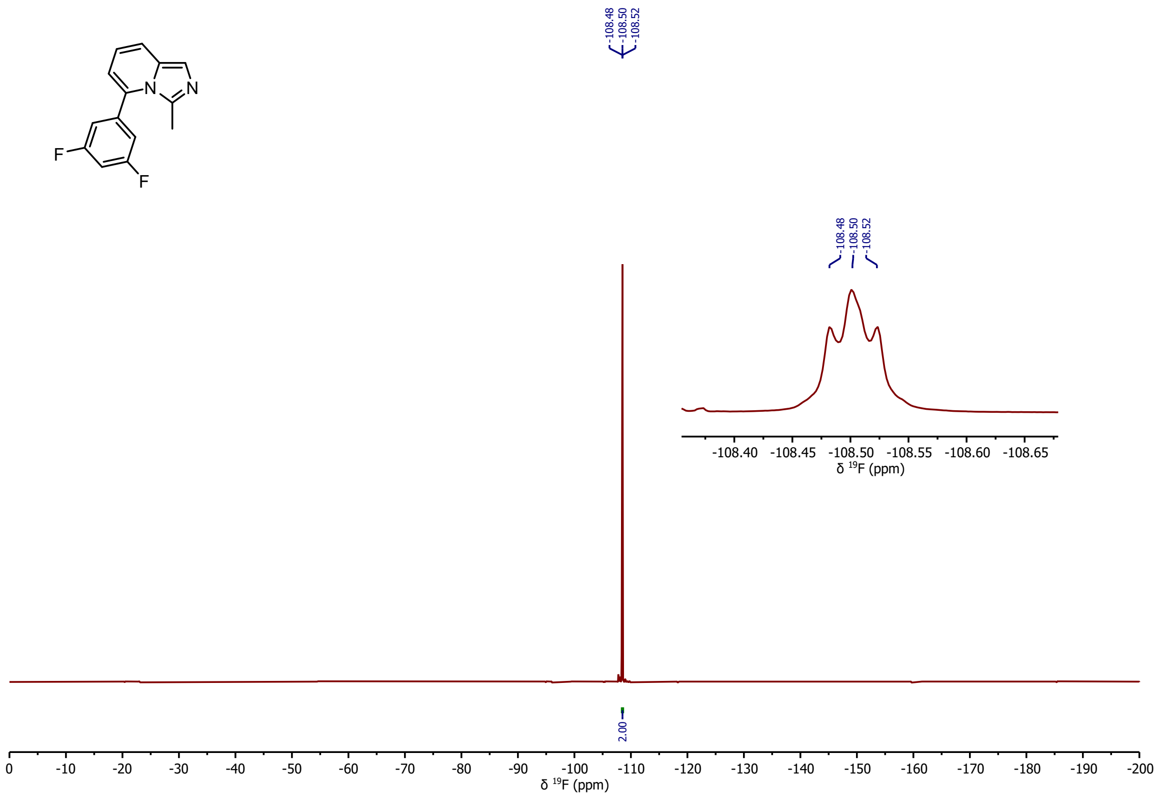


**Figure S77**: **$^{19}$F NMR** (377 MHz, $CDCl_3$, 298K) of **S19**.

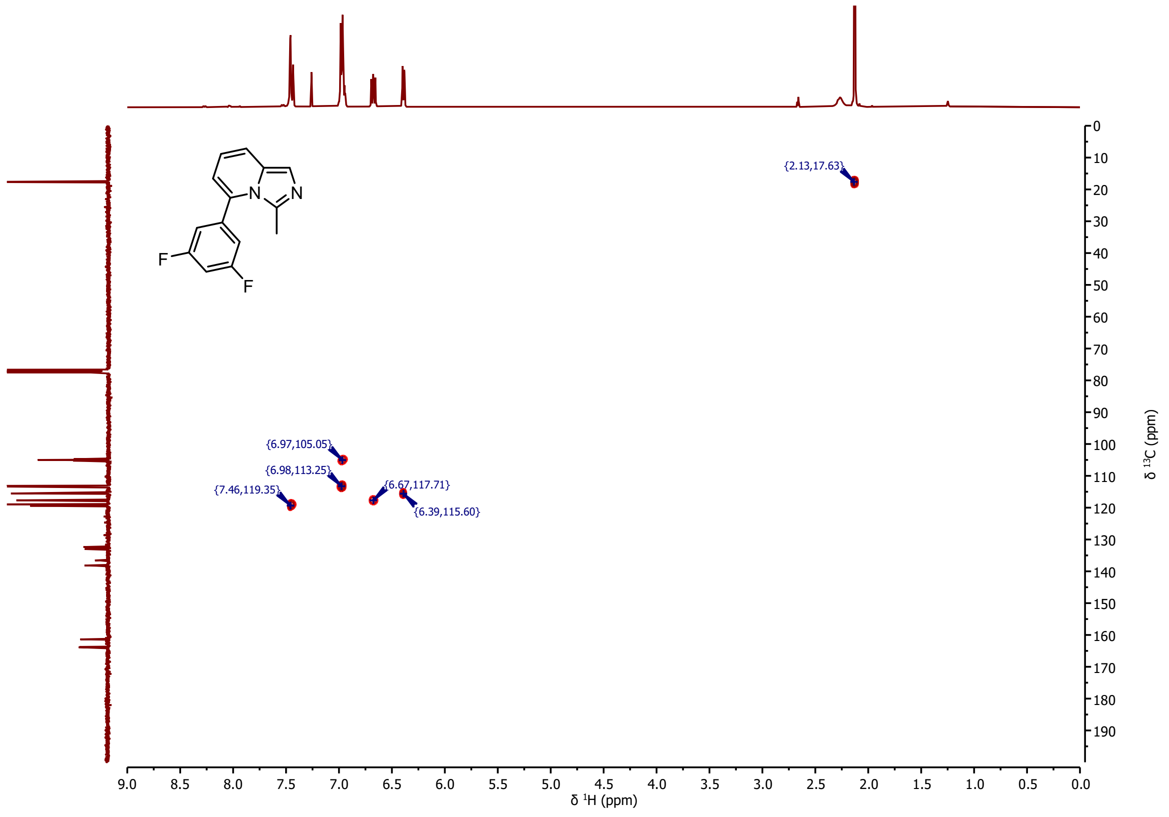


**Figure S78**: **$^{1}$H/$^{13}$C HSQC** (400/101 MHz, $CDCl_3$, 298 K) of **S19**.

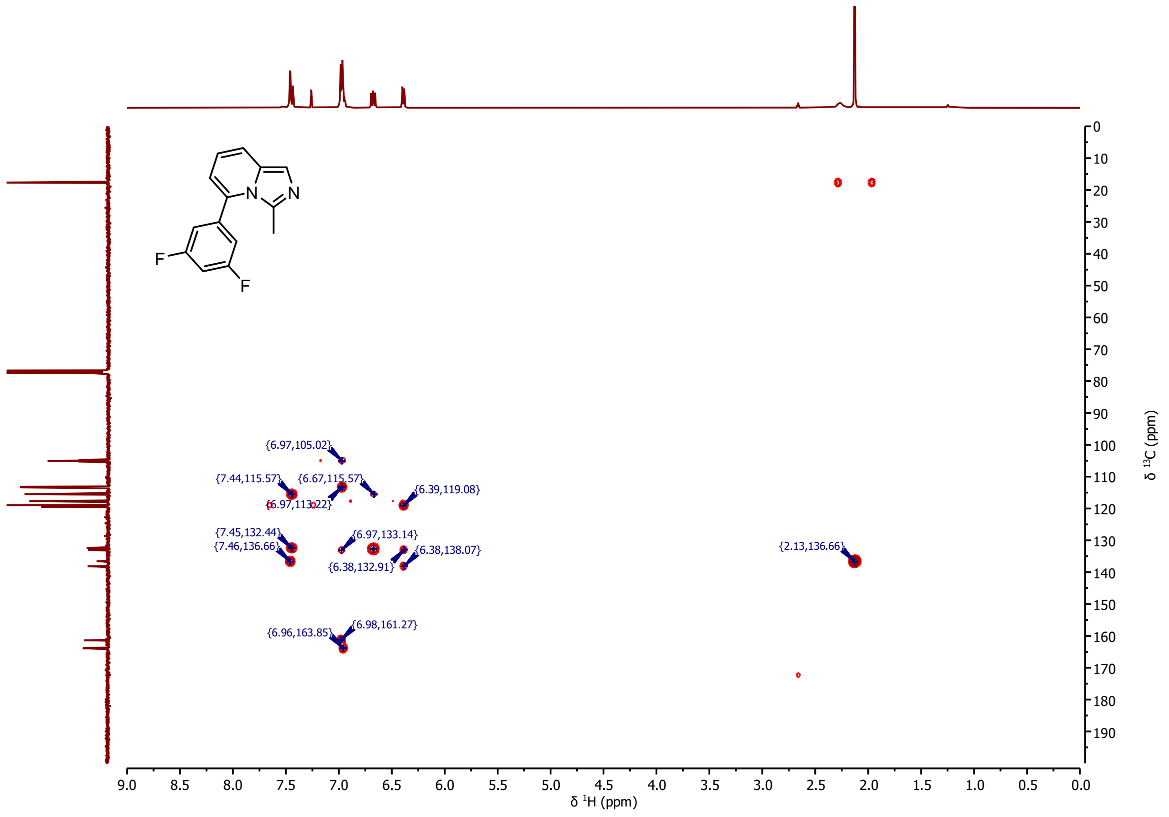

**Figure S79**: **$^1$H/$^{13}$C HMBC** (400/101 MHz, $CDCl_3$, 298 K) of **S19**.

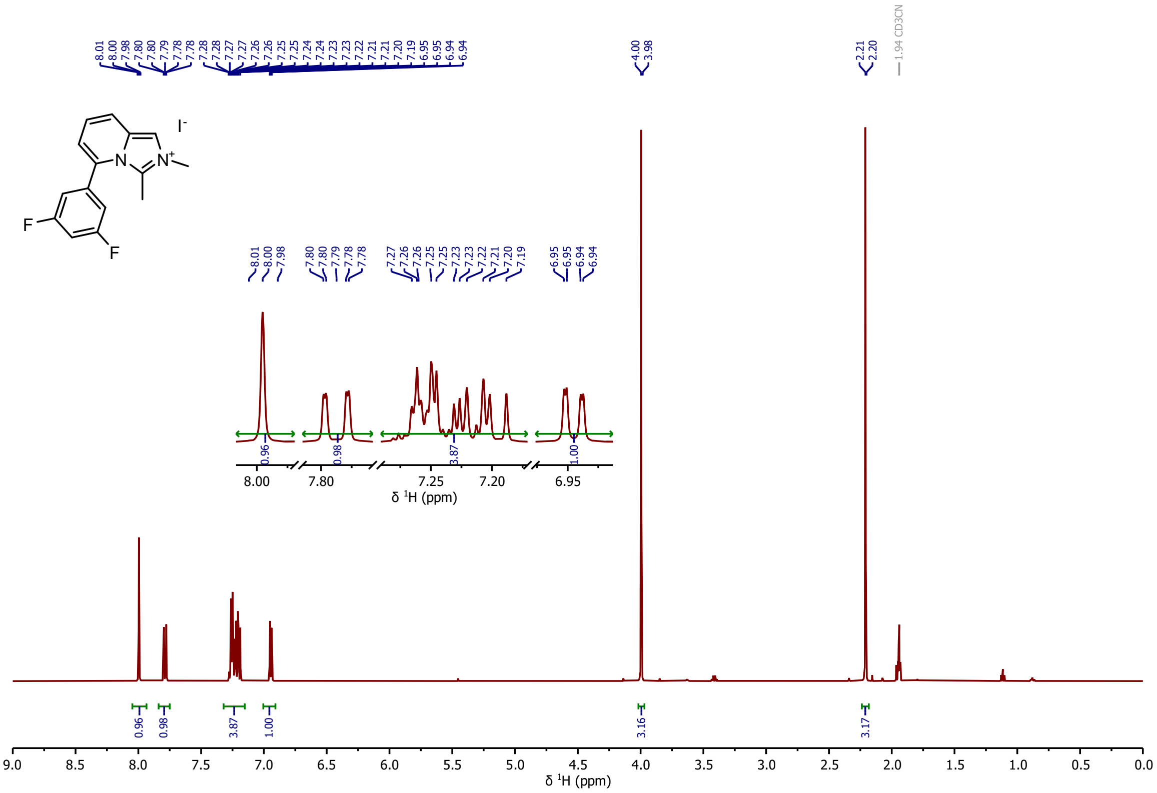

**Figure S80**: **$^1$H NMR** (400 MHz, $CD_3CN$, 298 K) of **1g**.

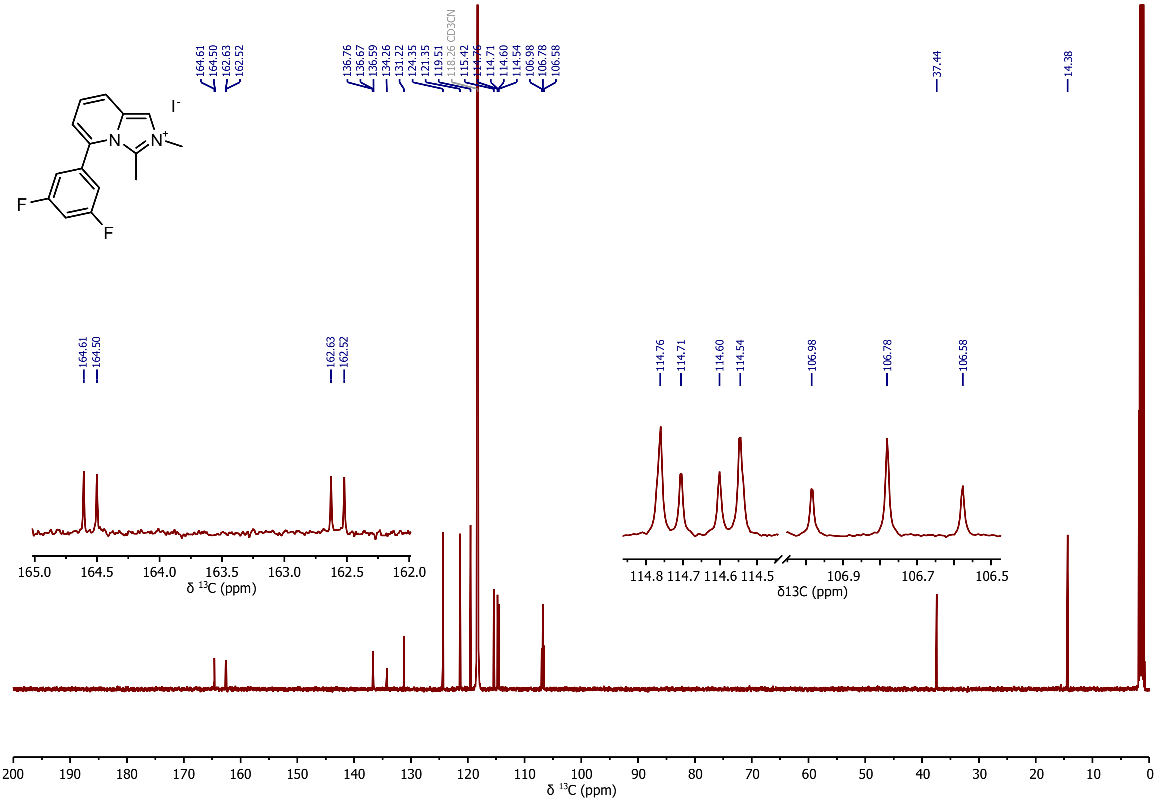

**Figure S81**: $^{13}$**C NMR** (101 MHz, $CD_3CN$, 298 K) of **1g**.

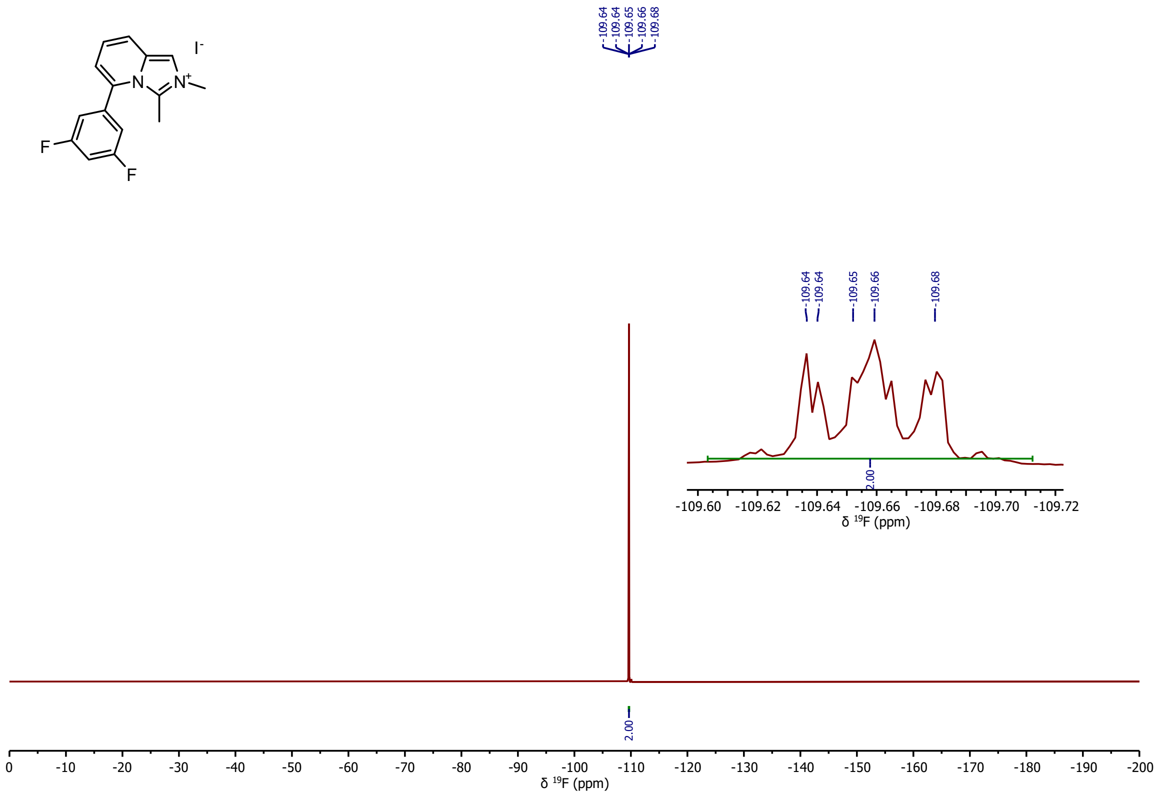

**Figure S82**: $^{19}$**F NMR** (377 MHz, $CD_3CN$, 298K) of **1g**.

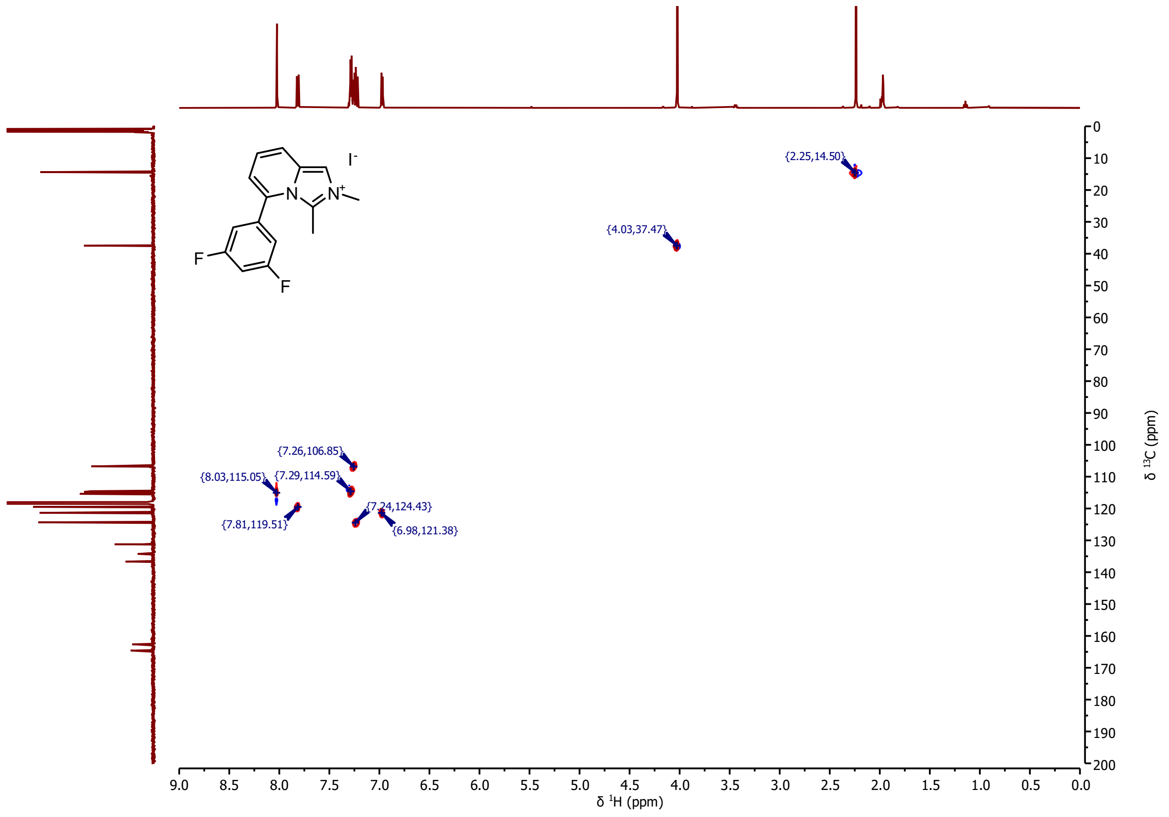

**Figure S83**: **$^{1}$H/$^{13}$C HSQC** (400/101 MHz, $CD_3CN$, 298 K) of **1g**.

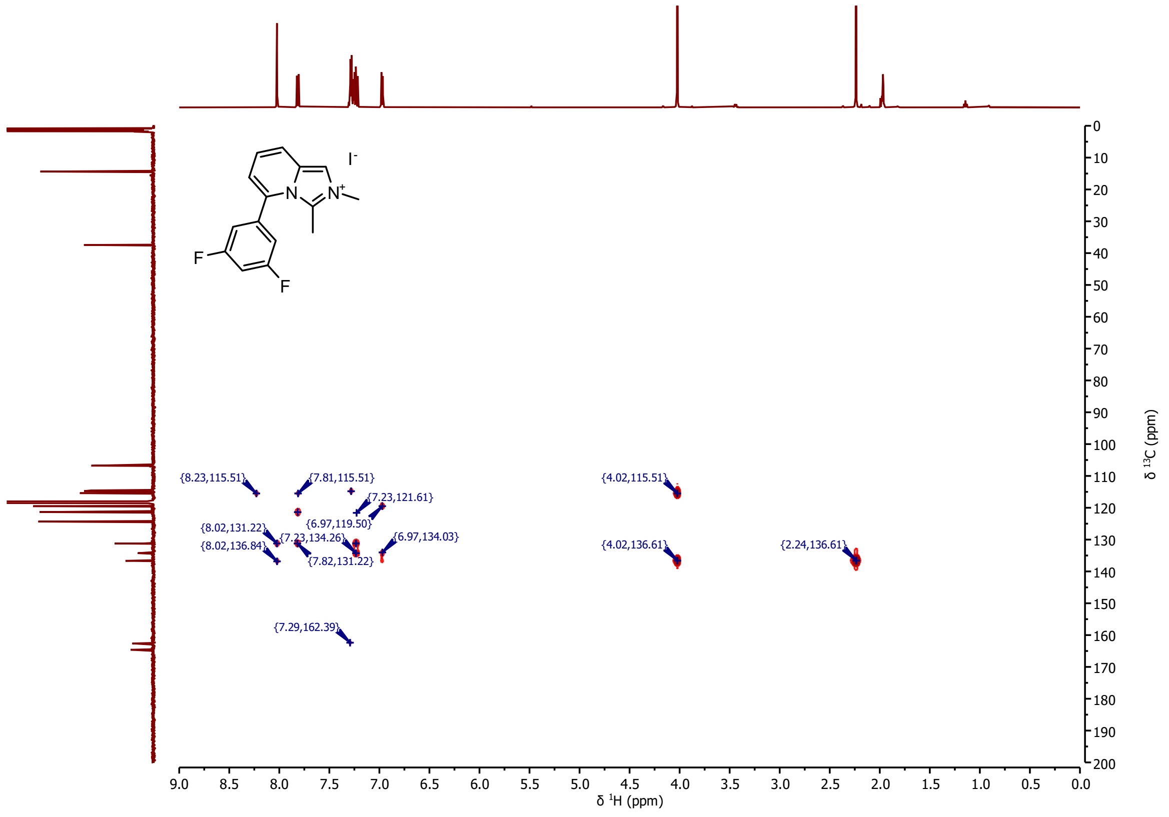

**Figure S84**: **$^{1}$H/$^{13}$C HMBC** (400/101 MHz, $CD_3CN$, 298 K) of **1g**.

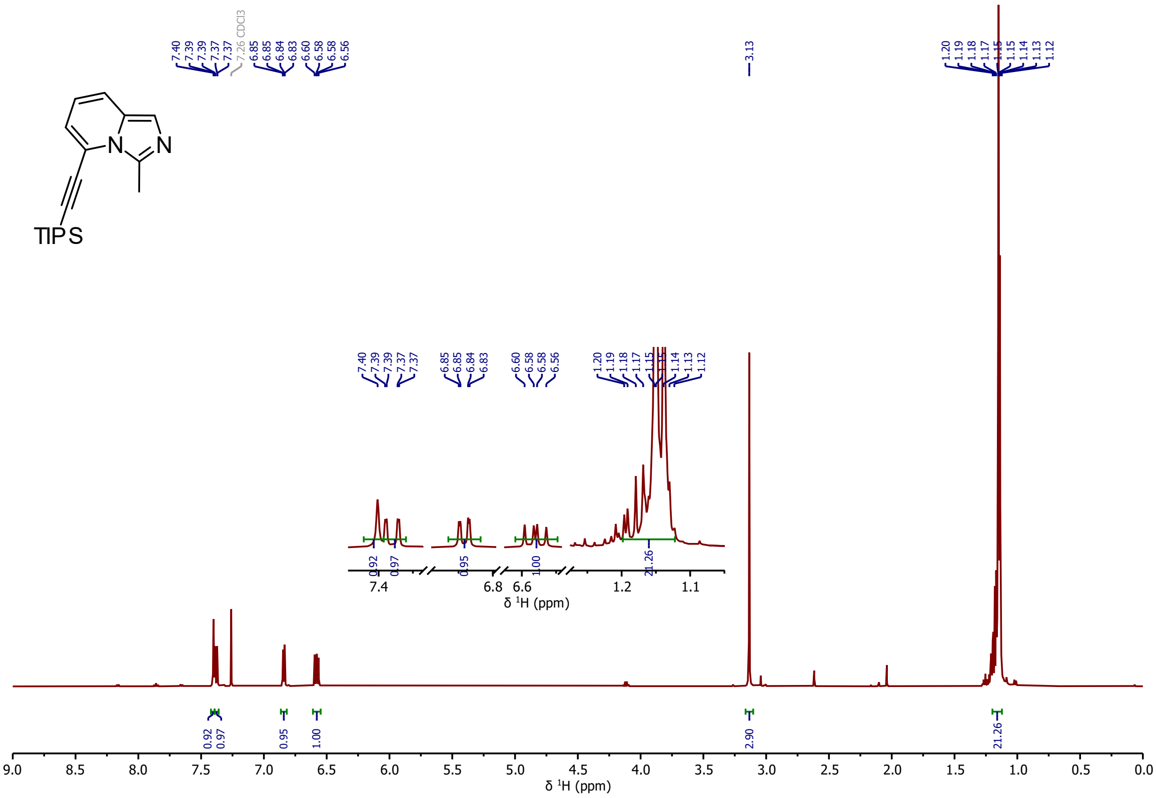


**Figure S85**: **$^{1}$H NMR** (500 MHz, $CDCl_3$, 298 K) of **S20**.

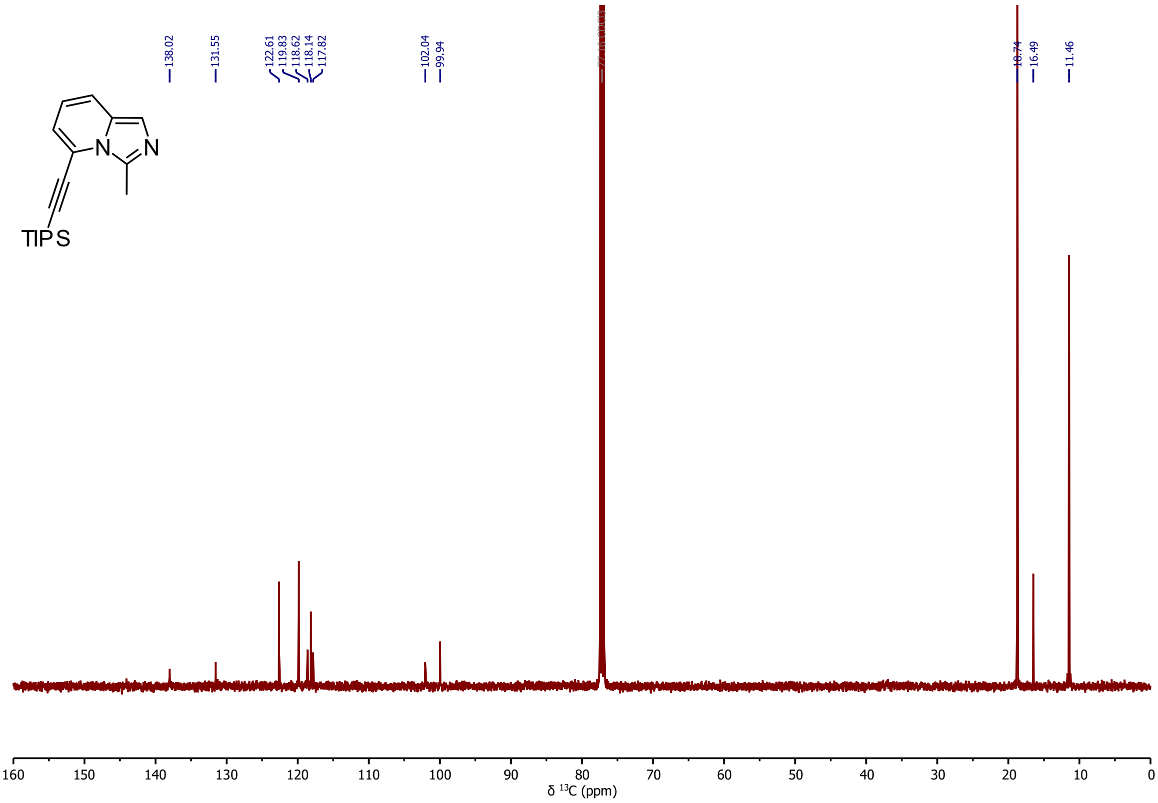


**Figure S86**: **$^{13}$C NMR** (126 MHz, $CDCl_3$, 298 K) of **S20**.

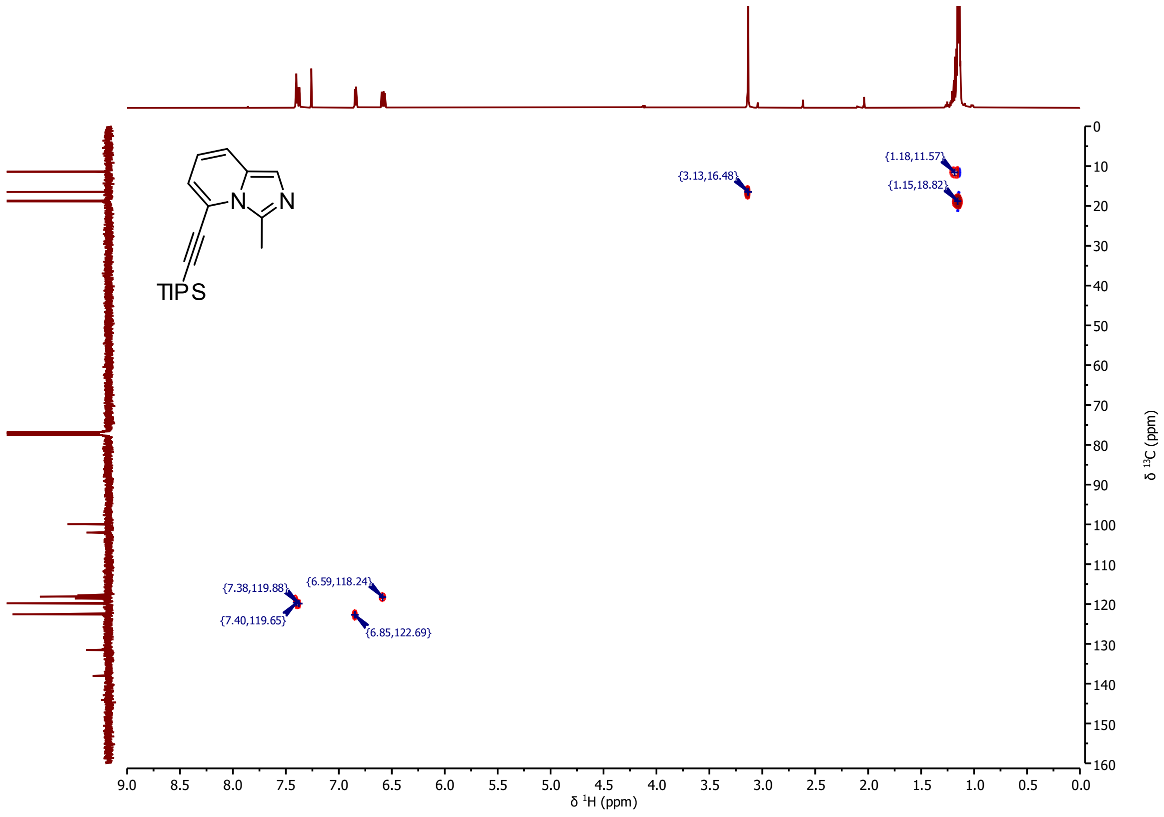


**Figure S87**: $^1$H/$^{13}$C HSQC (500/126 MHz, $CDCl_3$, 298 K) of **S20**.

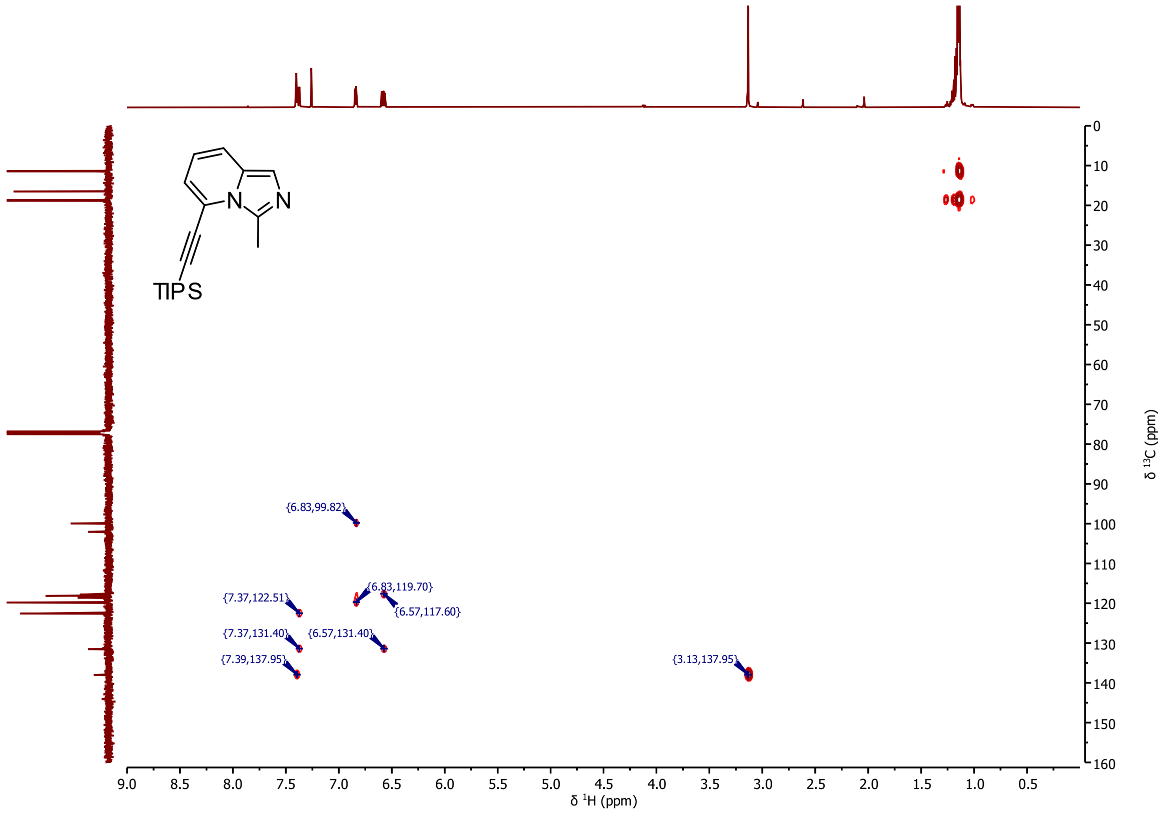


**Figure S88**: $^1$H/$^{13}$C HMBC (500/126 MHz, $CDCl_3$, 298 K) of **S20**.

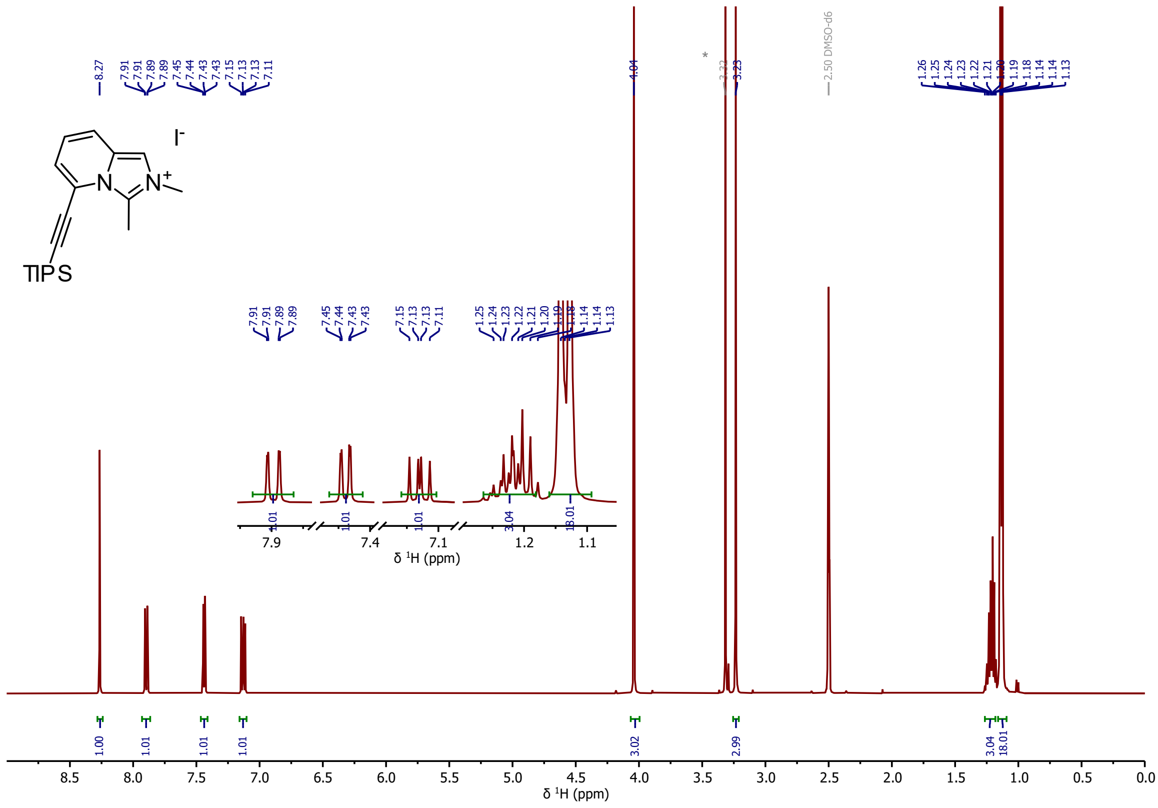


**Figure S89**: **$^1$H NMR** (500 MHz, DMSO-$d_6$, 298 K) of **4b**. * = $H_2O$.

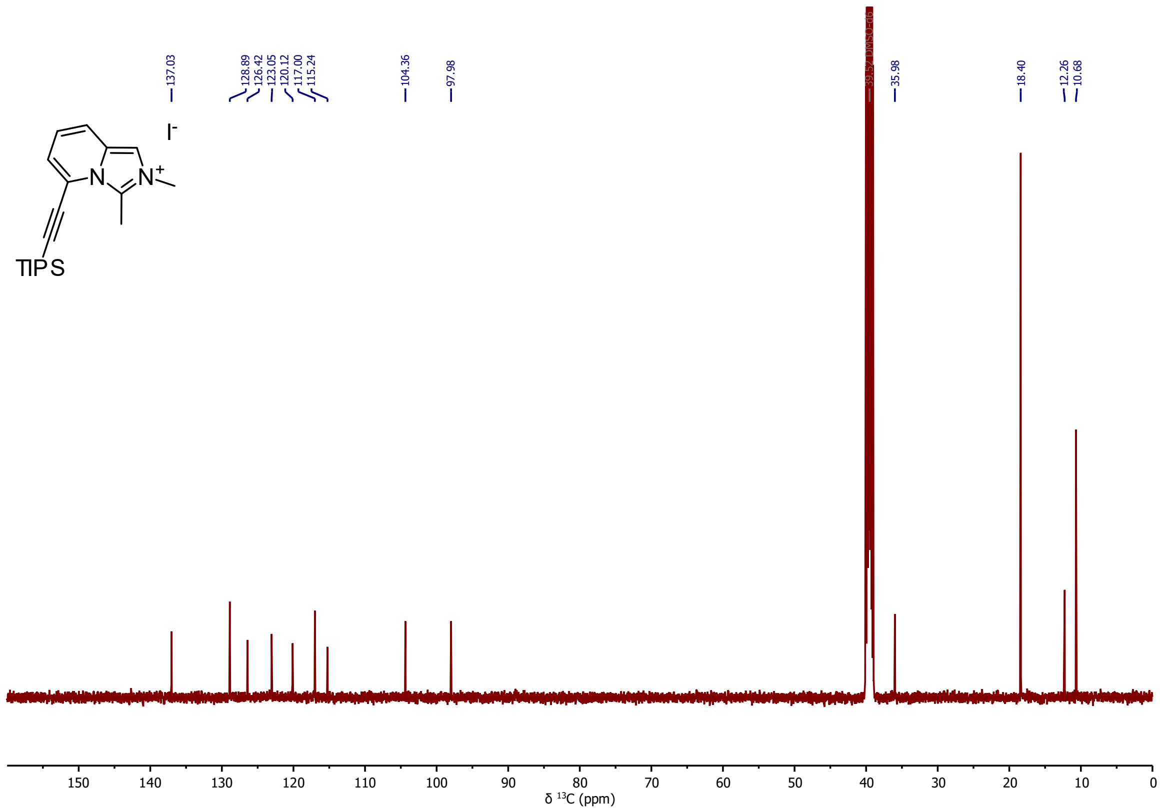


**Figure S90**: **$^{13}$C NMR** (126 MHz, DMSO-$d_6$, 298 K) of **4b**.

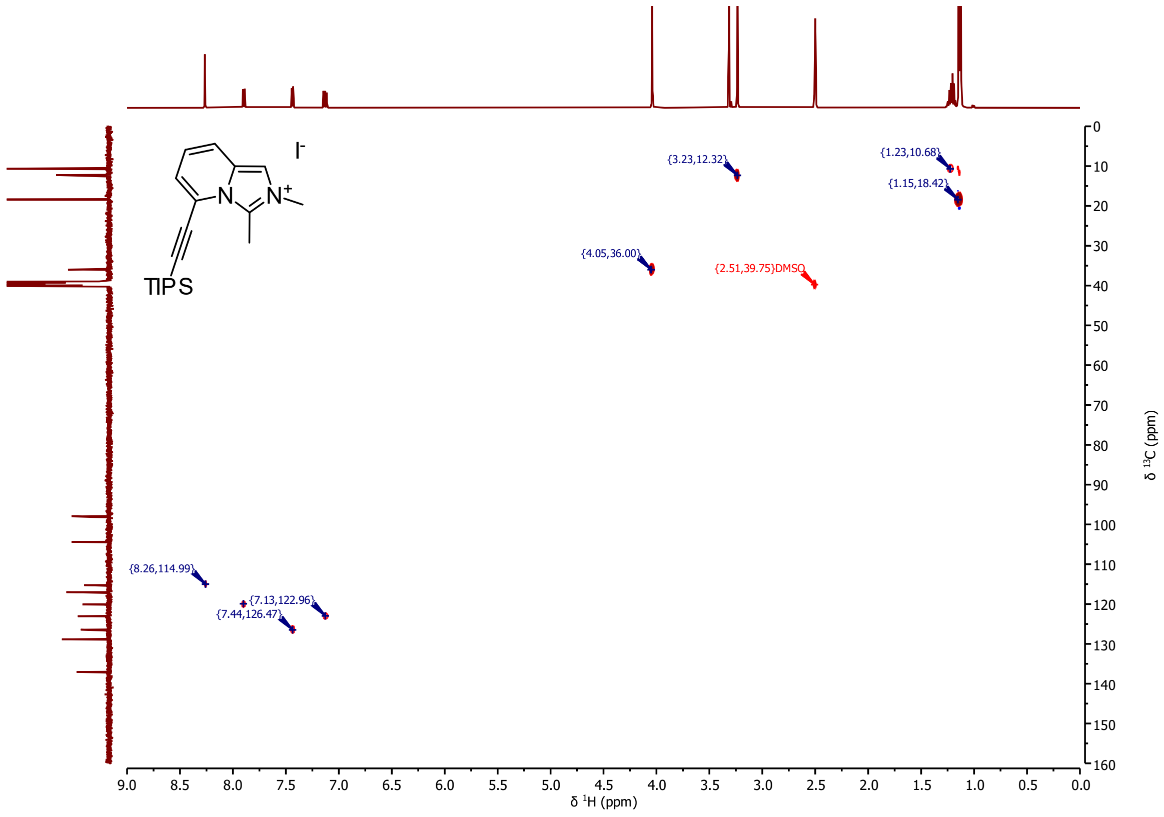


**Figure S91**: **$^1$H/$^{13}$C HSQC** (500/126 MHz, DMSO-d$_6$, 298 K) of **4b**.

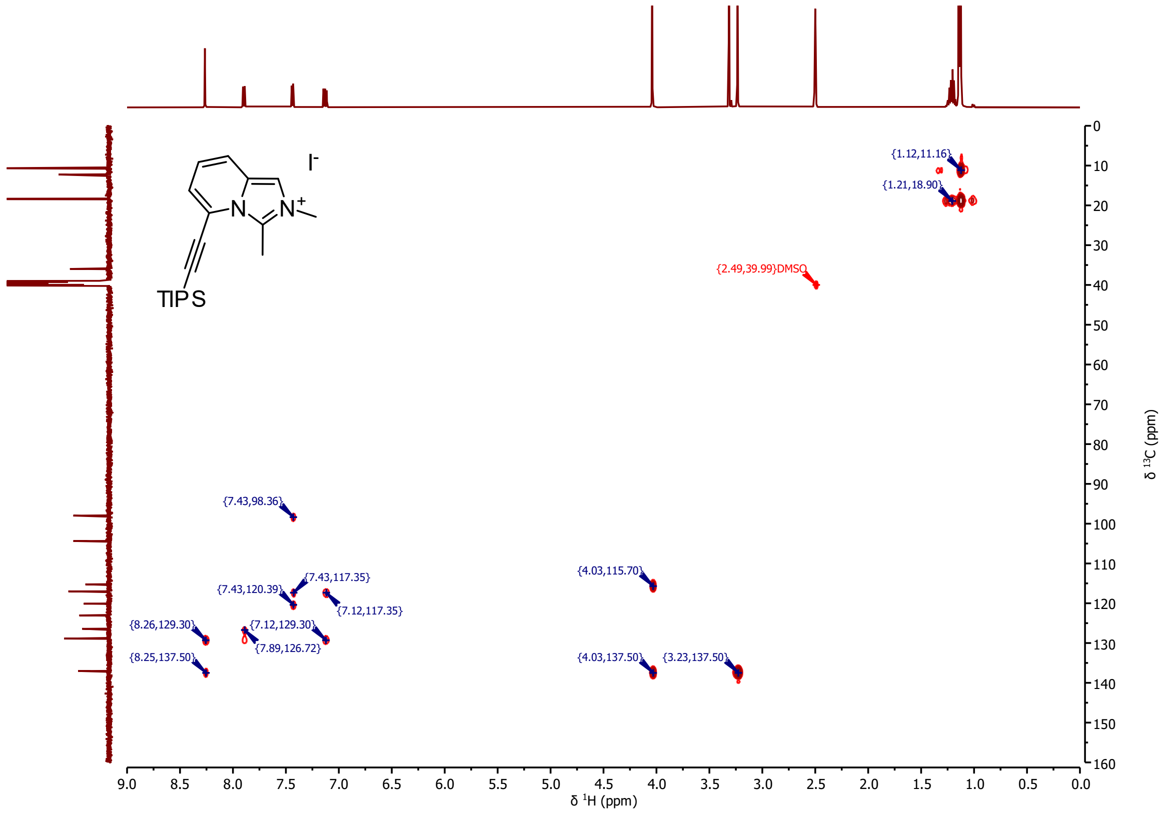


**Figure S92**: **$^1$H/$^{13}$C HMBC** (500/126 MHz, DMSO-d$_6$, 298 K) of **4b**.

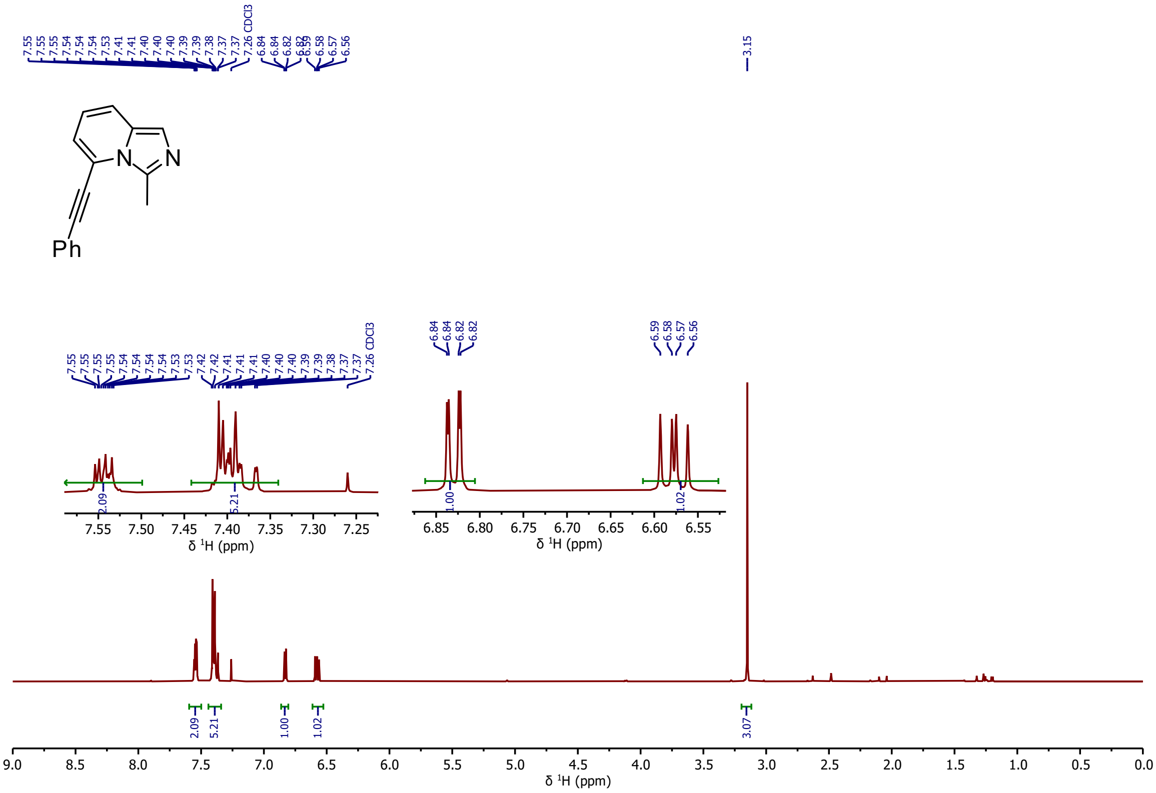

**Figure S93**: **$^1$H NMR** (500 MHz, $CDCl_3$, 298 K) of **S21**.

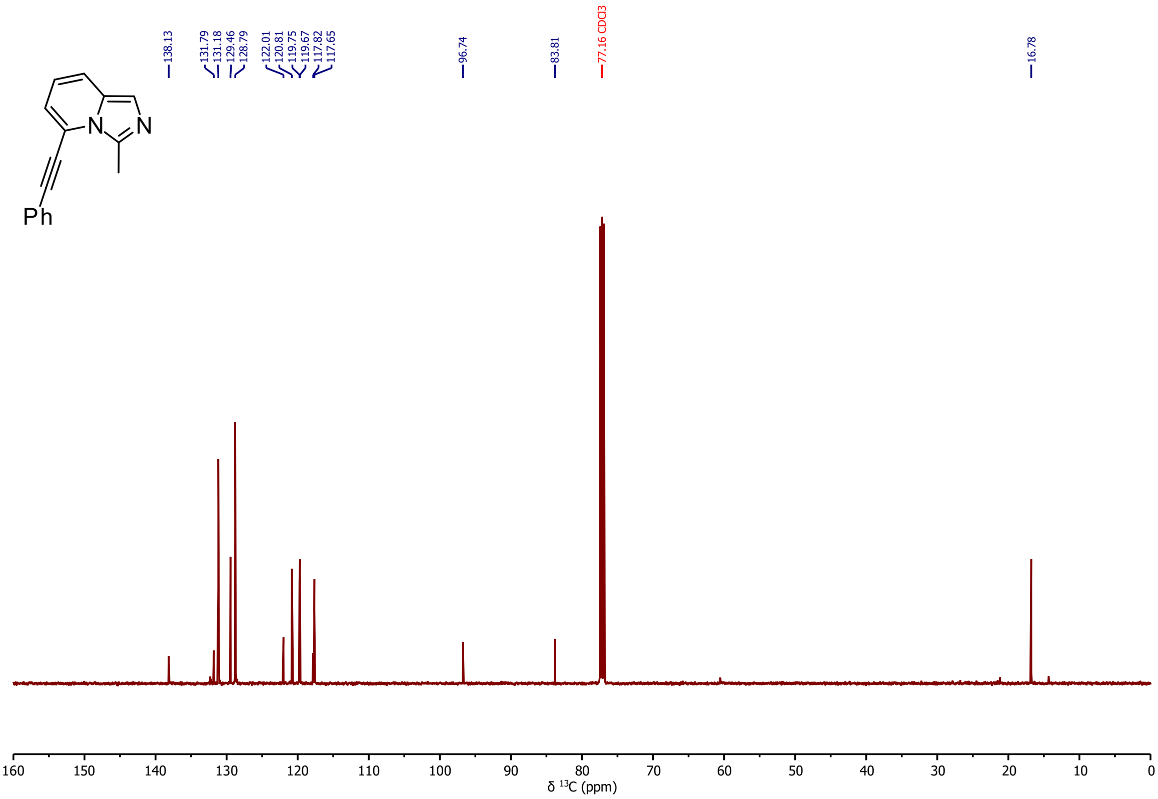

**Figure S94**: **$^{13}$C NMR** (126 MHz, $CDCl_3$, 298 K) of **S21**.

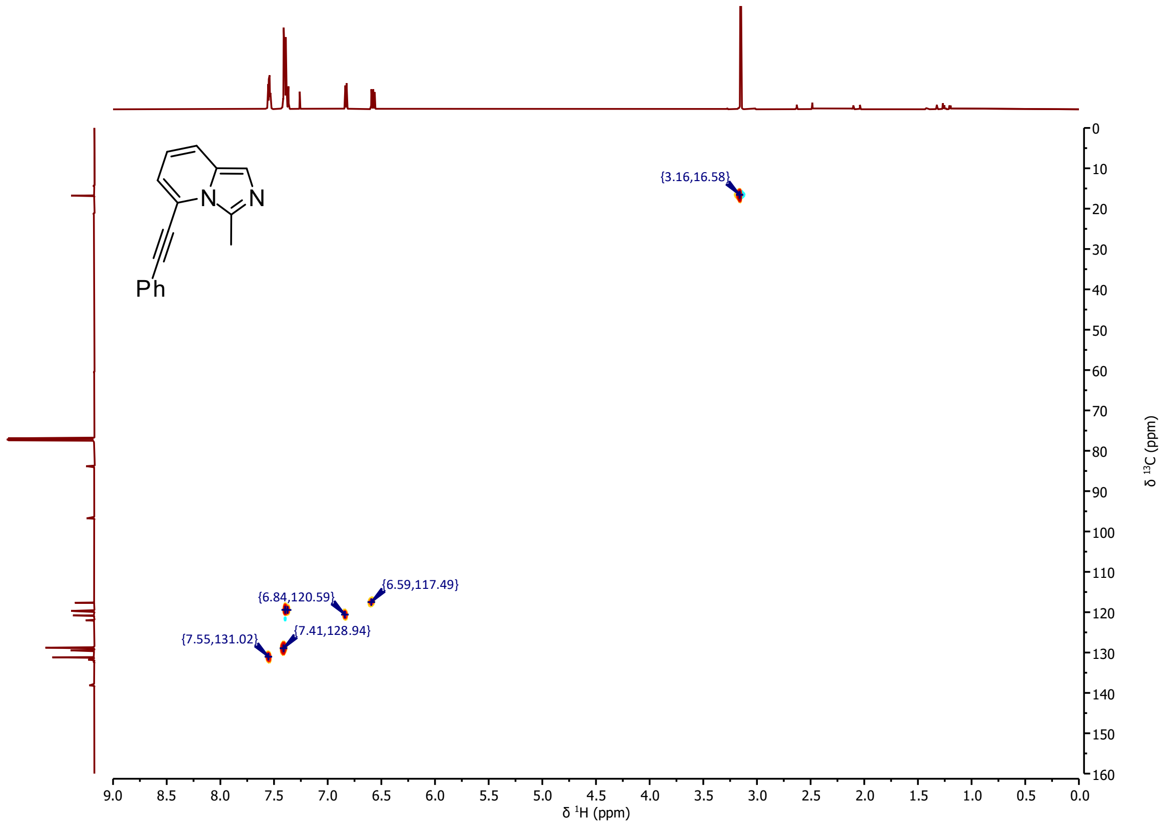


**Figure S95**: **$^{1}$H/$^{13}$C HSQC** (500/126 MHz, $CDCl_3$, 298 K) of **S21**.

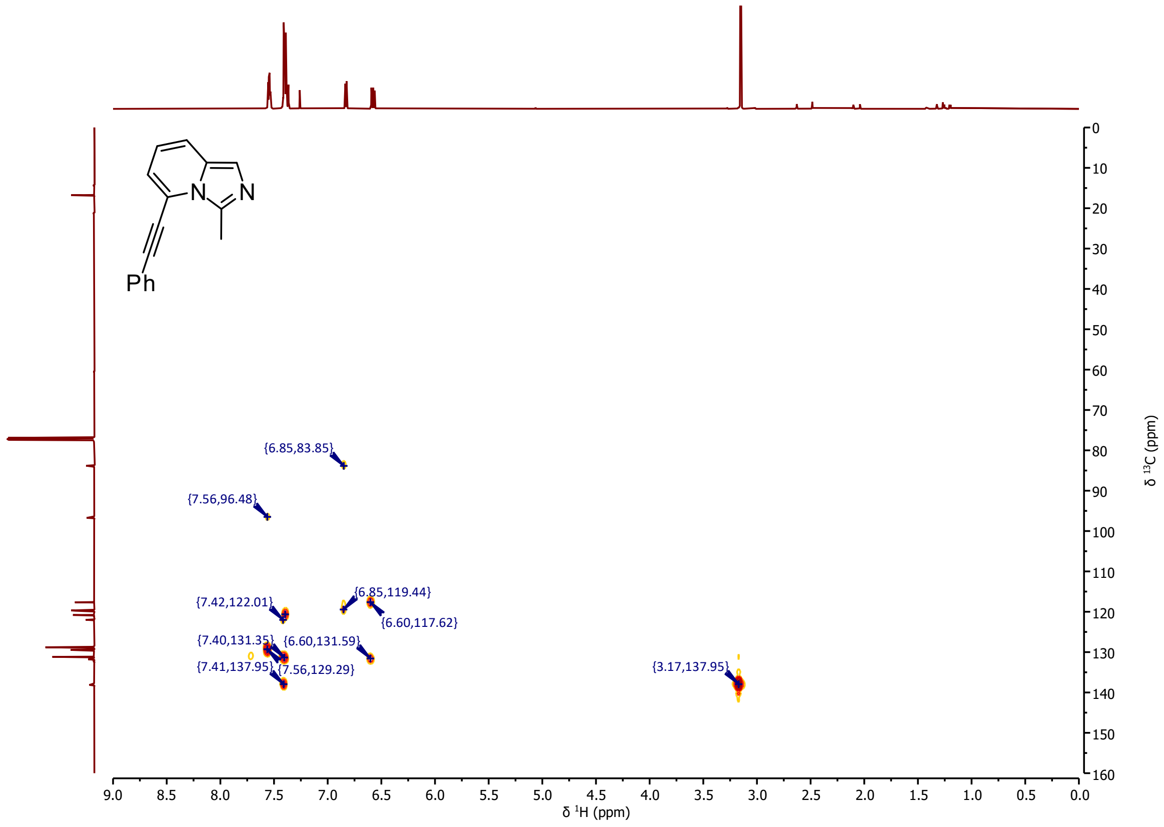


**Figure S96**: **$^{1}$H/$^{13}$C HMBC** (500/126 MHz, $CDCl_3$, 298 K) of **S21**.

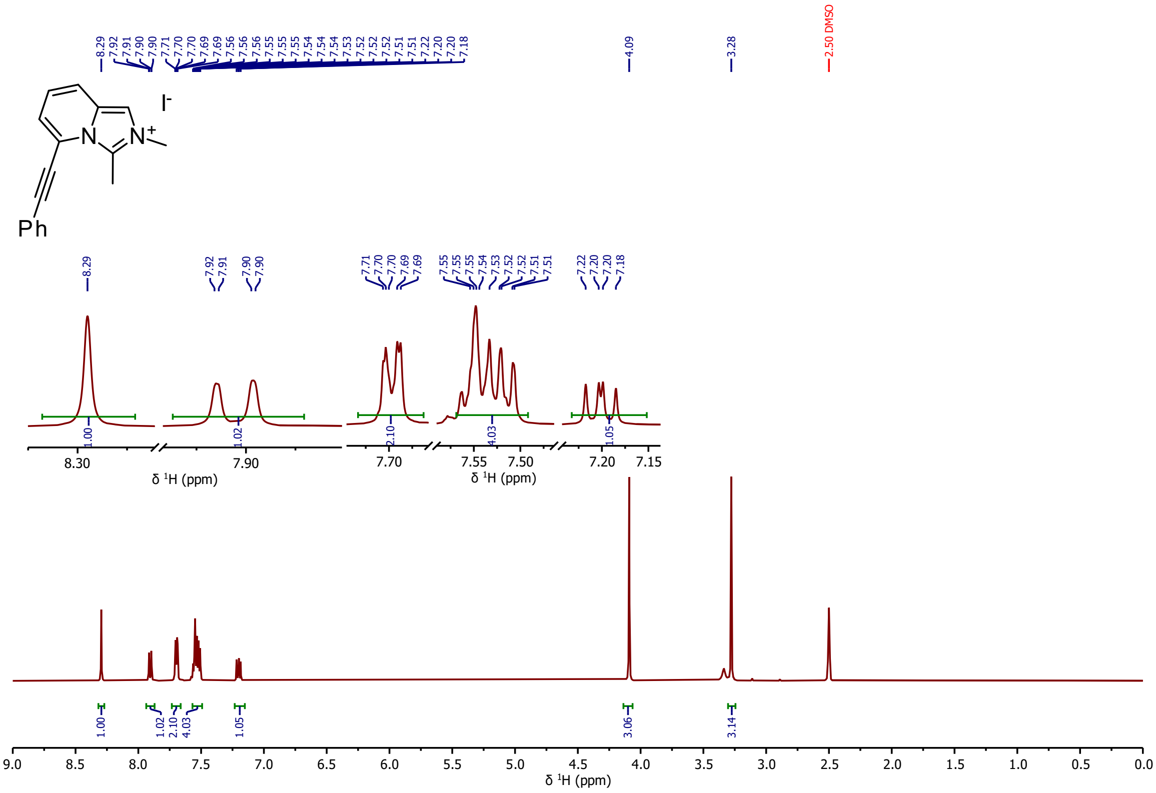

**Figure S97**: **$^{1}$H NMR** (500 MHz, DMSO-$d_6$, 298 K) of **4a**.

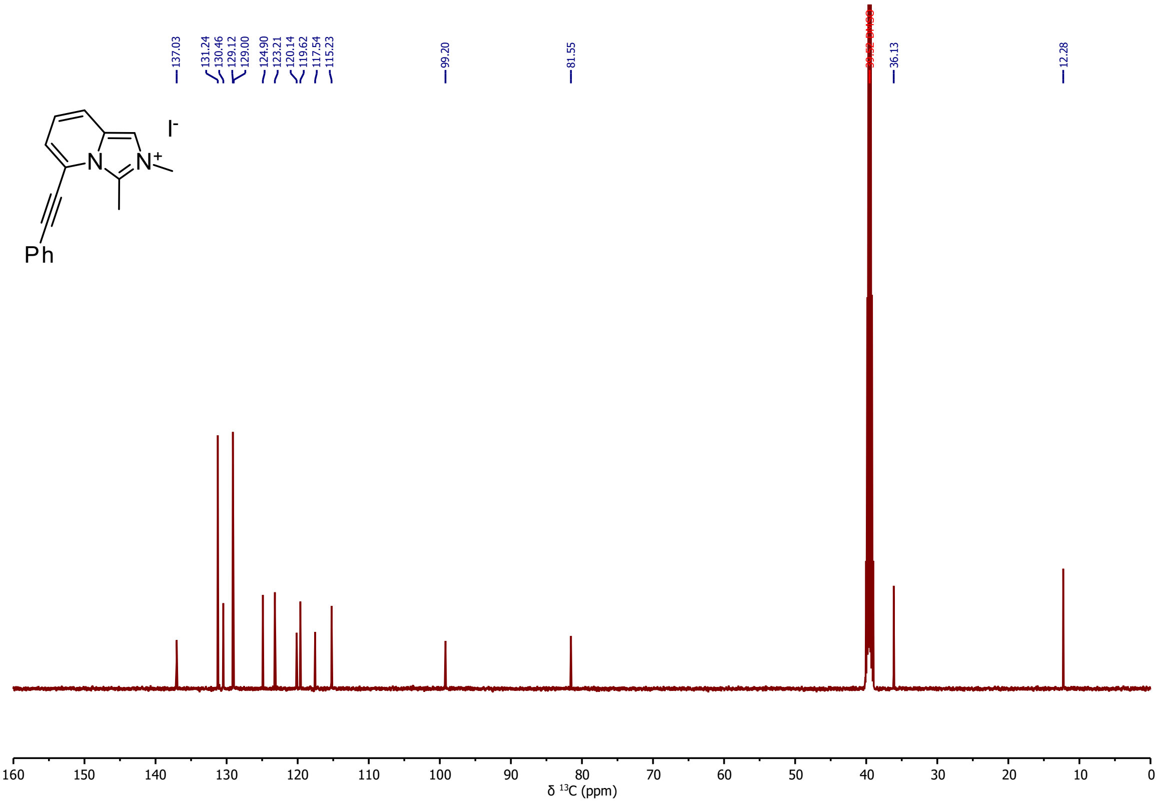

**Figure S98**: **$^{13}$C NMR** (126 MHz, DMSO-$d_6$, 298 K) of **4a**.

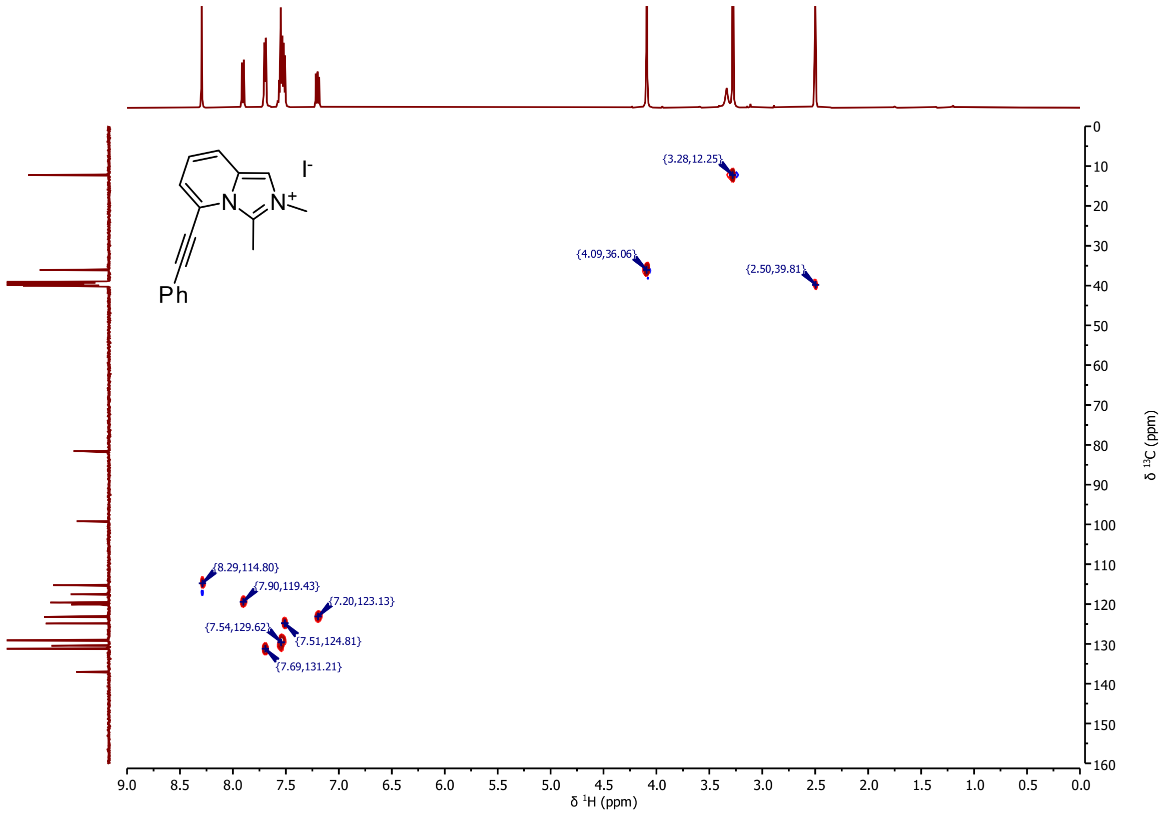

**Figure S99**: $^{1}$**H/**$^{13}$**C HSQC** (500/126 MHz, DMSO-$d_6$, 298 K) of **4a**.

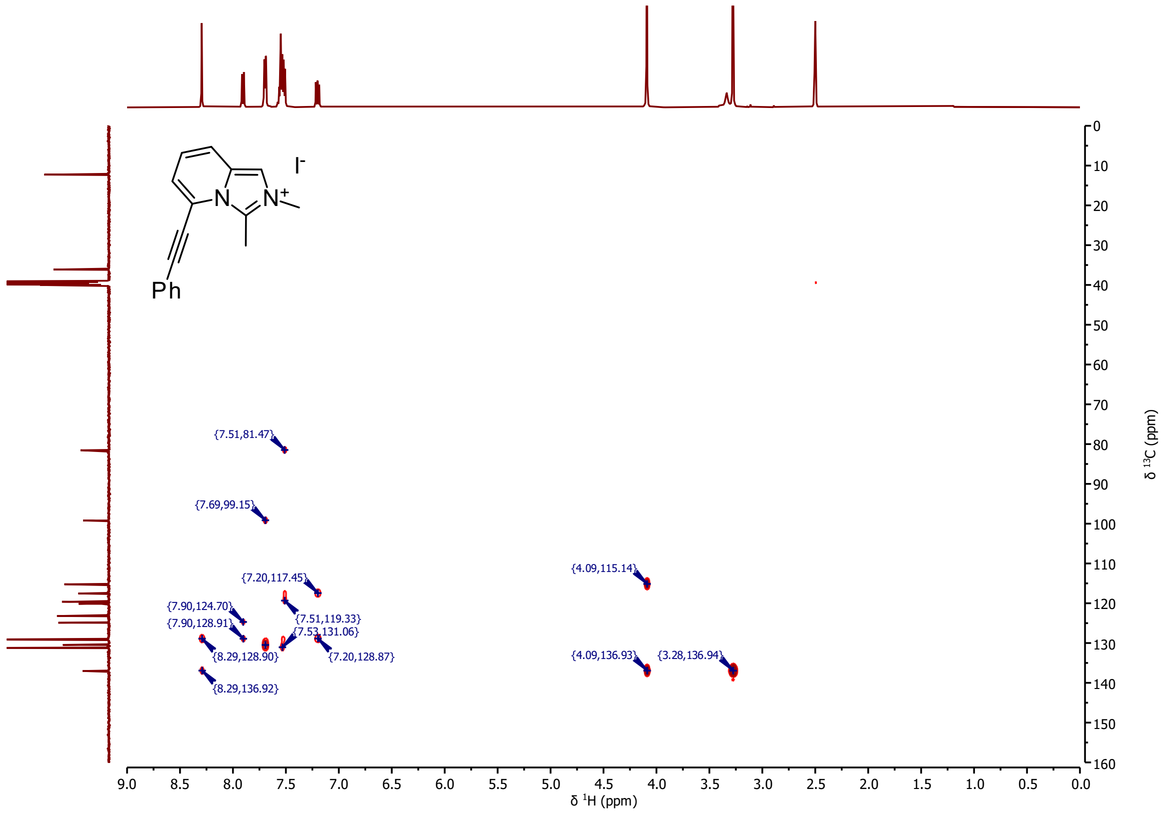

**Figure S100**: $^{1}$**H/**$^{13}$**C HMBC** (500/126 MHz, DMSO-$d_6$, 298 K) of **4a.**

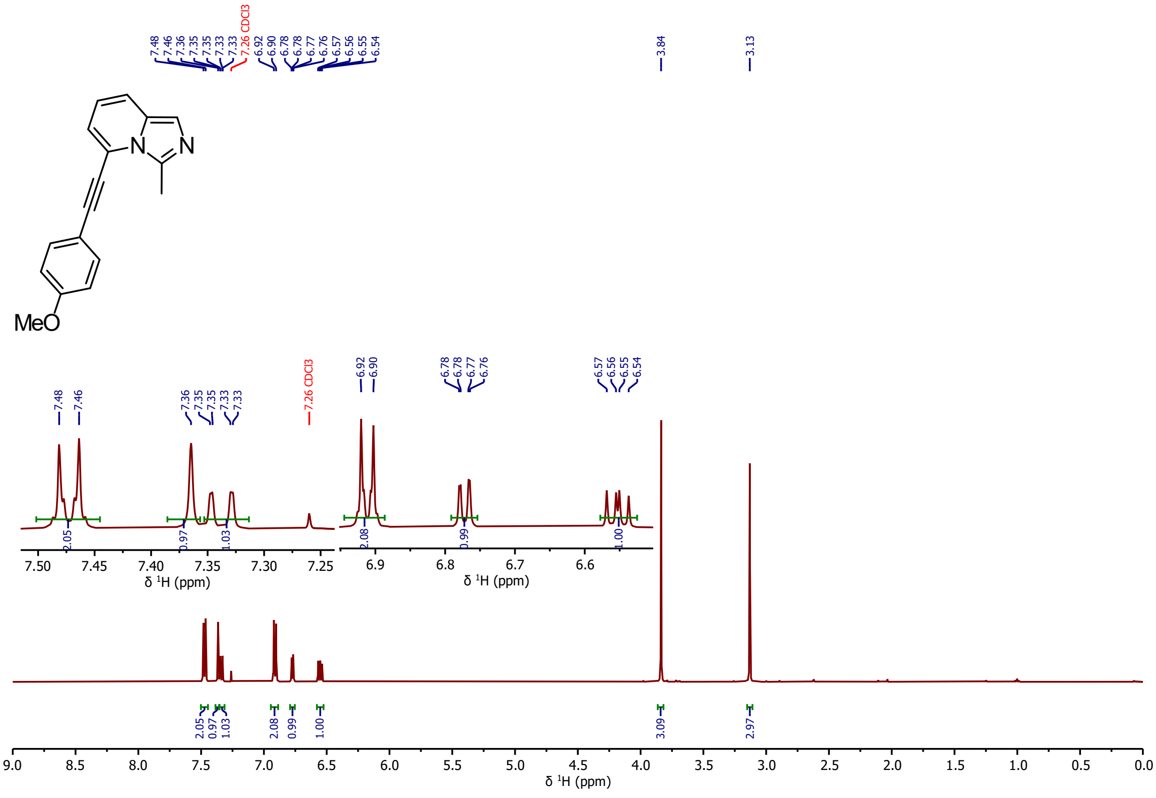

**Figure S101**: **$^1$H NMR** (500 MHz, $CDCl_3$, 298 K) of **S22**.

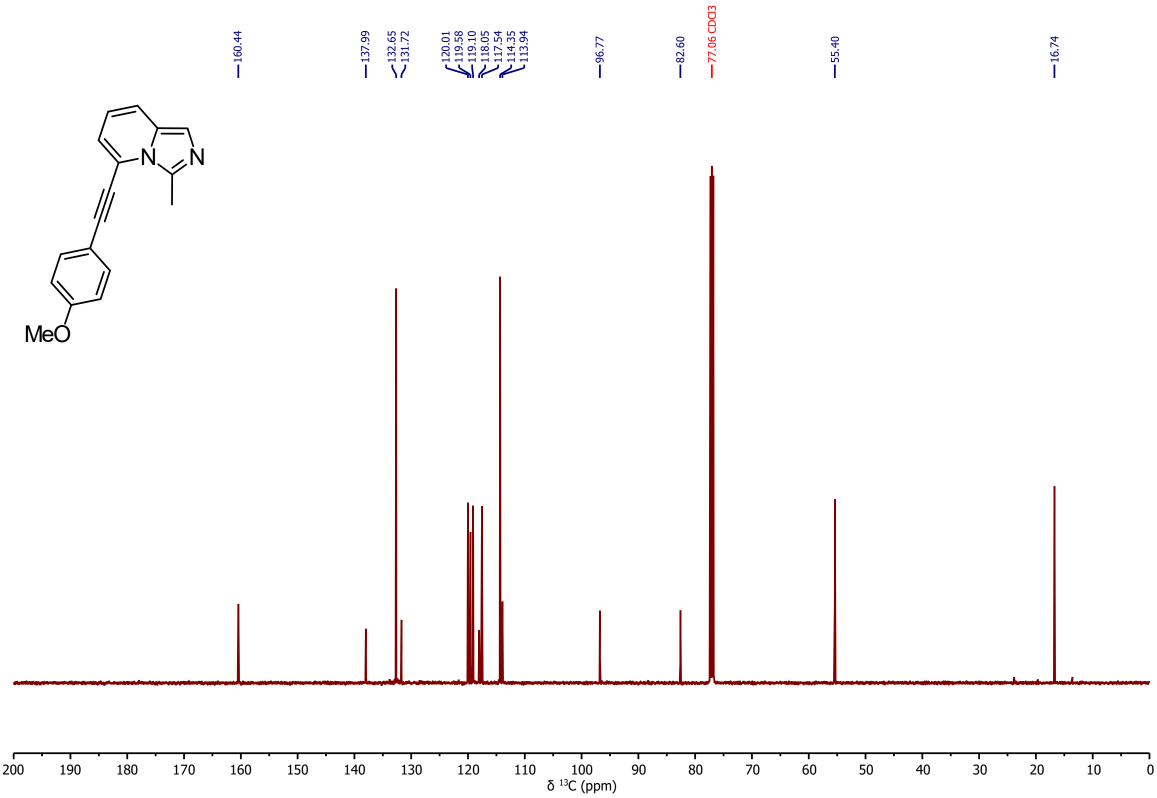

**Figure S102**: **$^{13}$C NMR** (126 MHz, $CDCl_3$, 298 K) of **S22**.

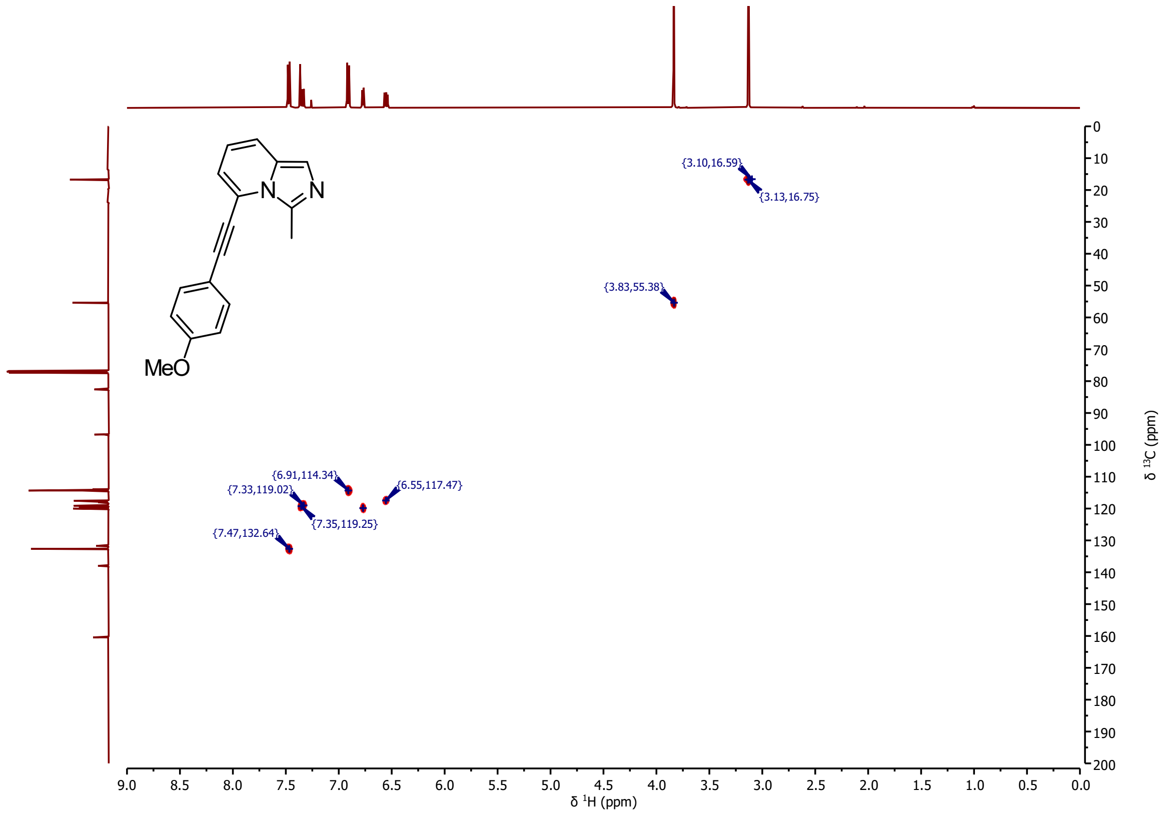

**Figure S103**: **$^{1}$H/$^{13}$C HSQC** (500/126 MHz, $CDCl_3$, 298 K) of **S22**.

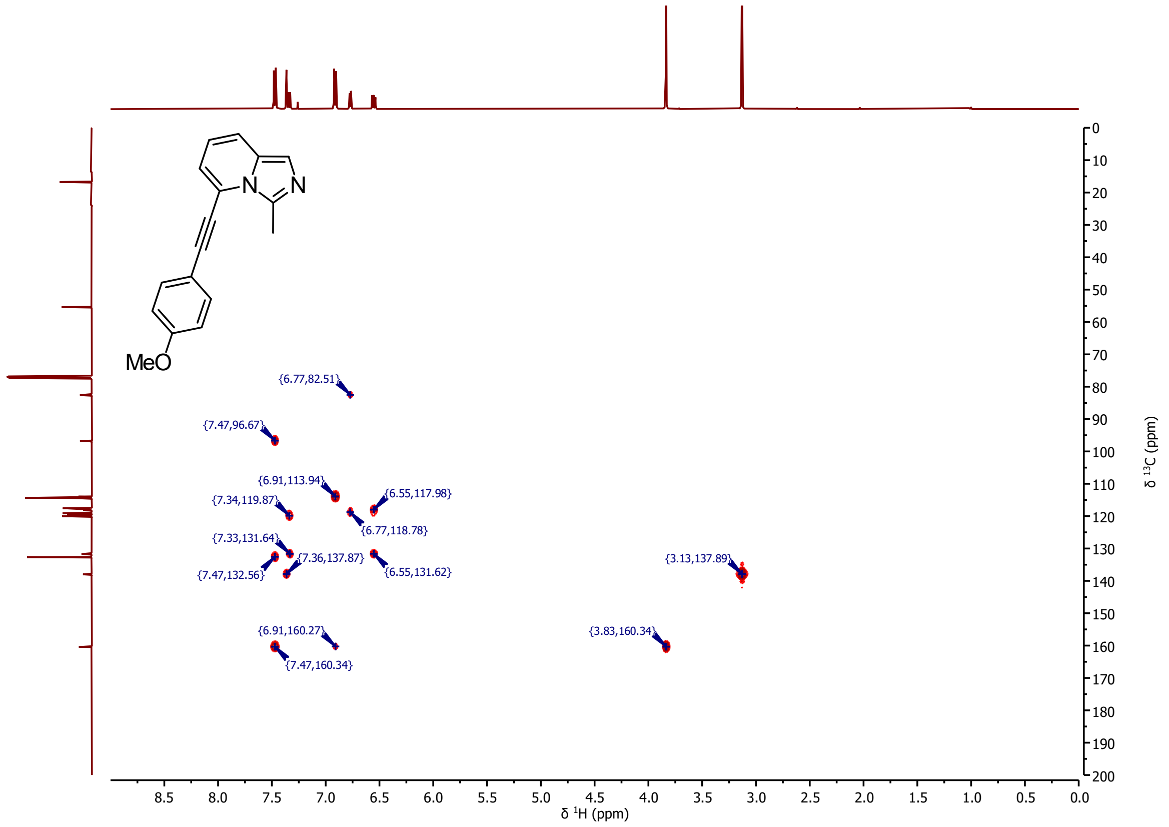

**Figure S104**: **$^{1}$H/$^{13}$C HMBC** (500/126 MHz, $CDCl_3$, 298 K) of **S22**.

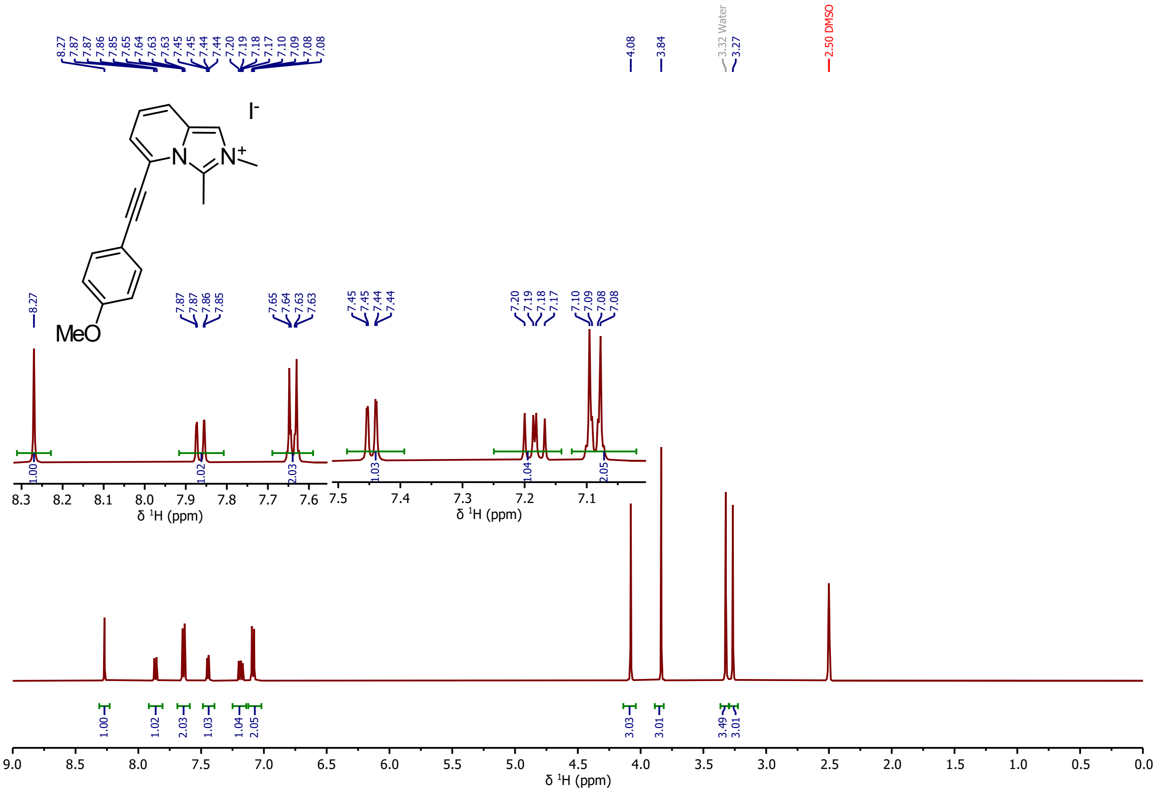


**Figure S105**: **$^1$H NMR** (500 MHz, DMSO-d$_6$, 298 K) of **4c**.

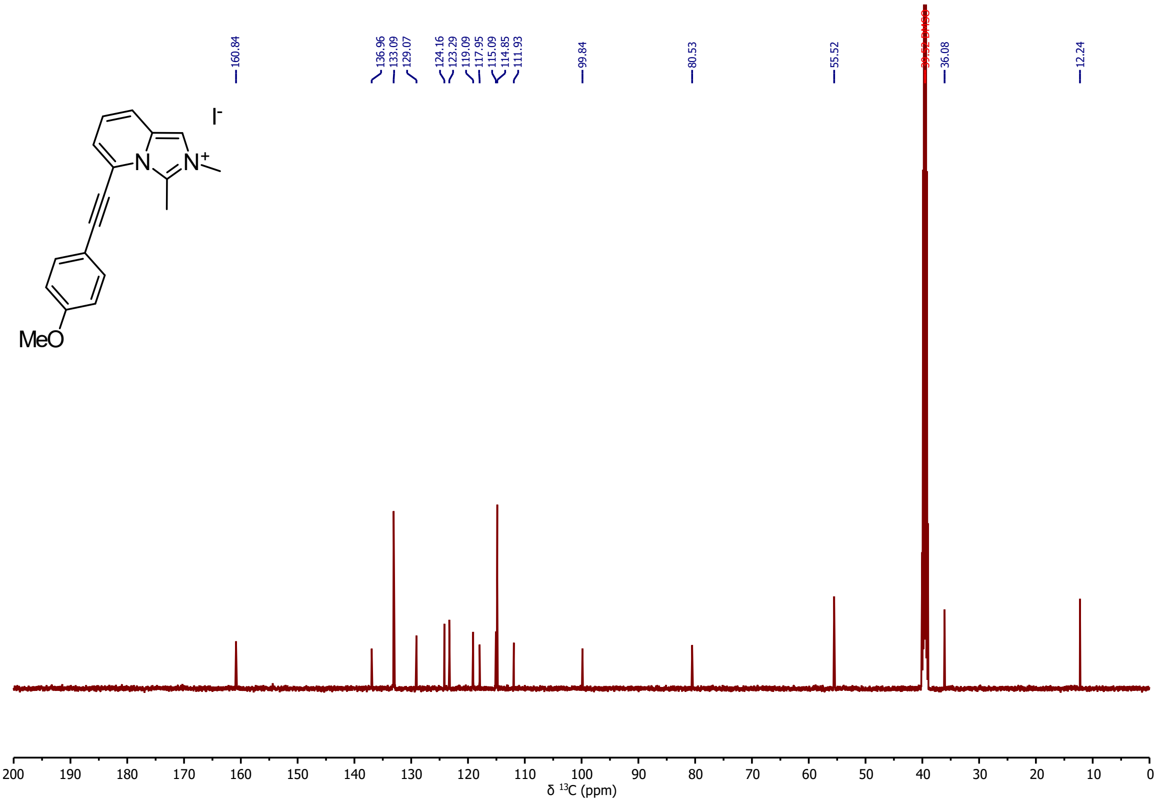


**Figure S106**: **$^{13}$C NMR** (126 MHz, DMSO-d$_6$, 298 K) of **4c**.

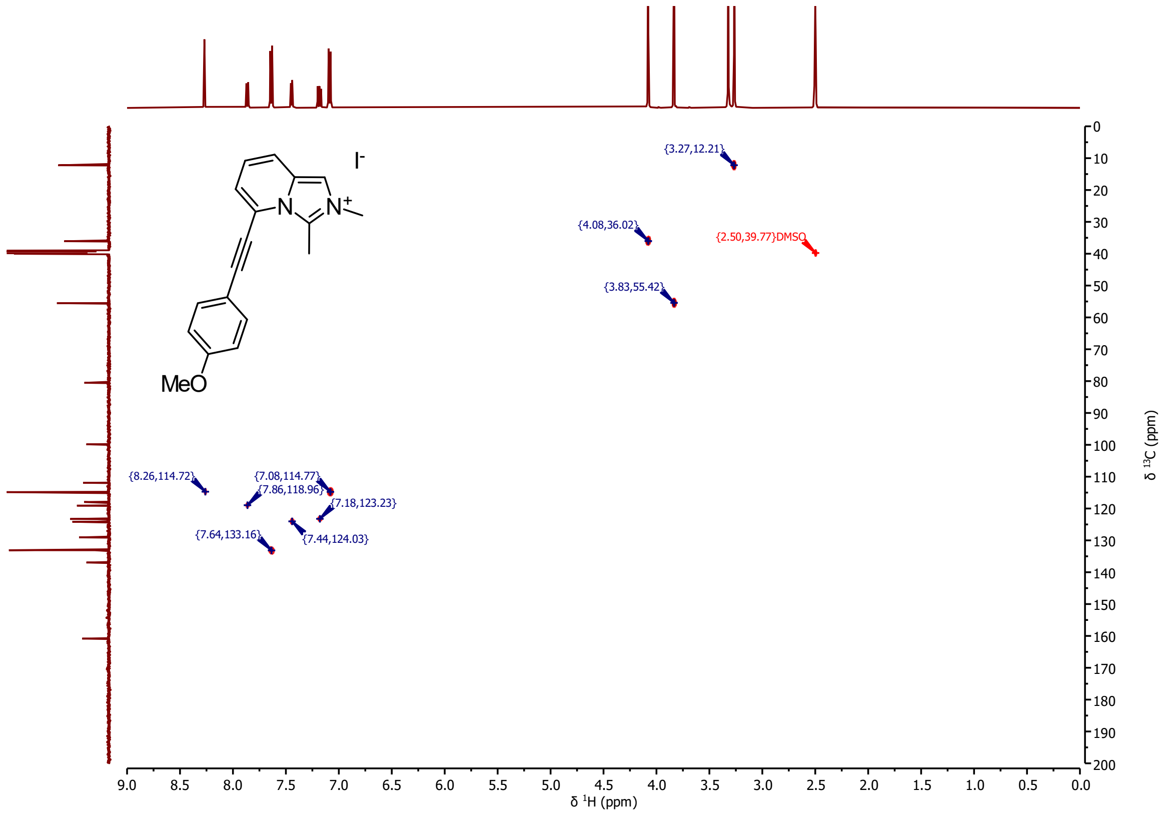


**Figure S107**: **$^1$H/$^{13}$C HSQC** (500/126 MHz, DMSO-$d_6$, 298 K) of **4c**.

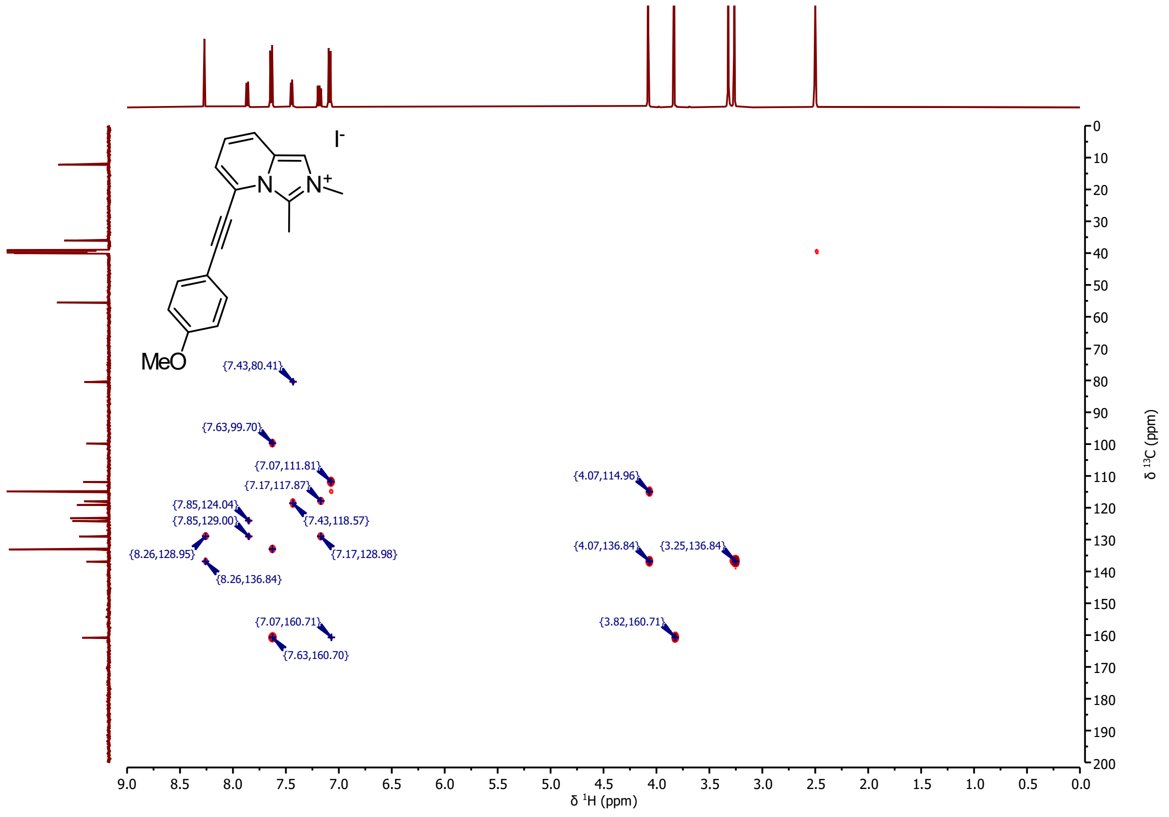


**Figure S108**: **$^1$H/$^{13}$C HMBC** (500/126 MHz, DMSO-$d_6$, 298 K) of **4c**.

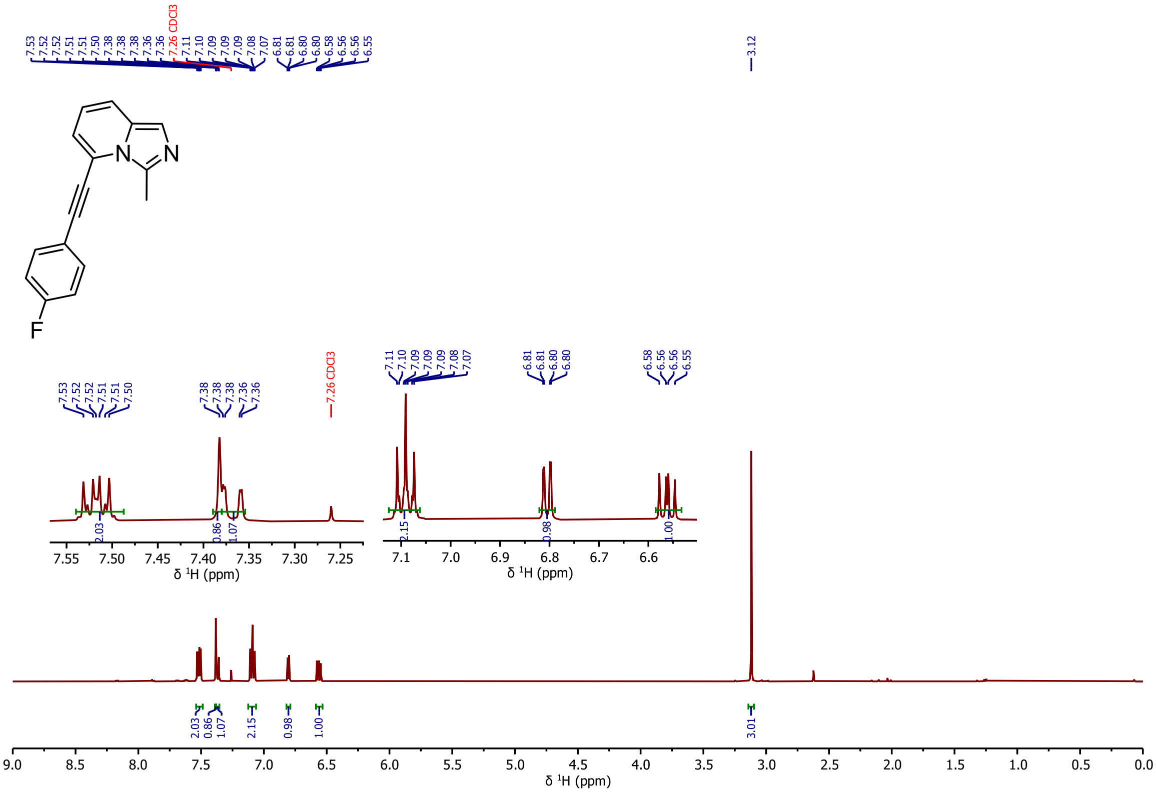


**Figure S109**: **$^{1}$H NMR** (500 MHz, $CDCl_3$, 298 K) of **S23**.

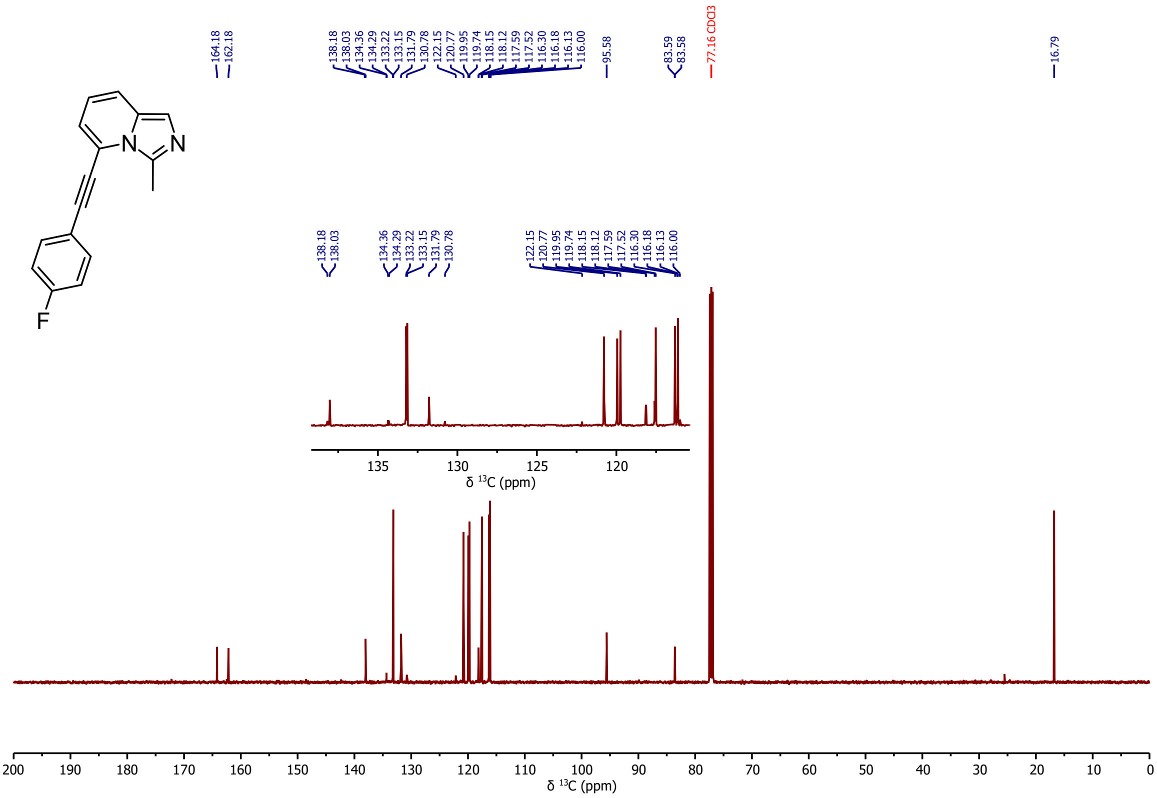


**Figure S110**: **$^{13}$C NMR** (126 MHz, $CDCl_3$, 298 K) of **S23**.

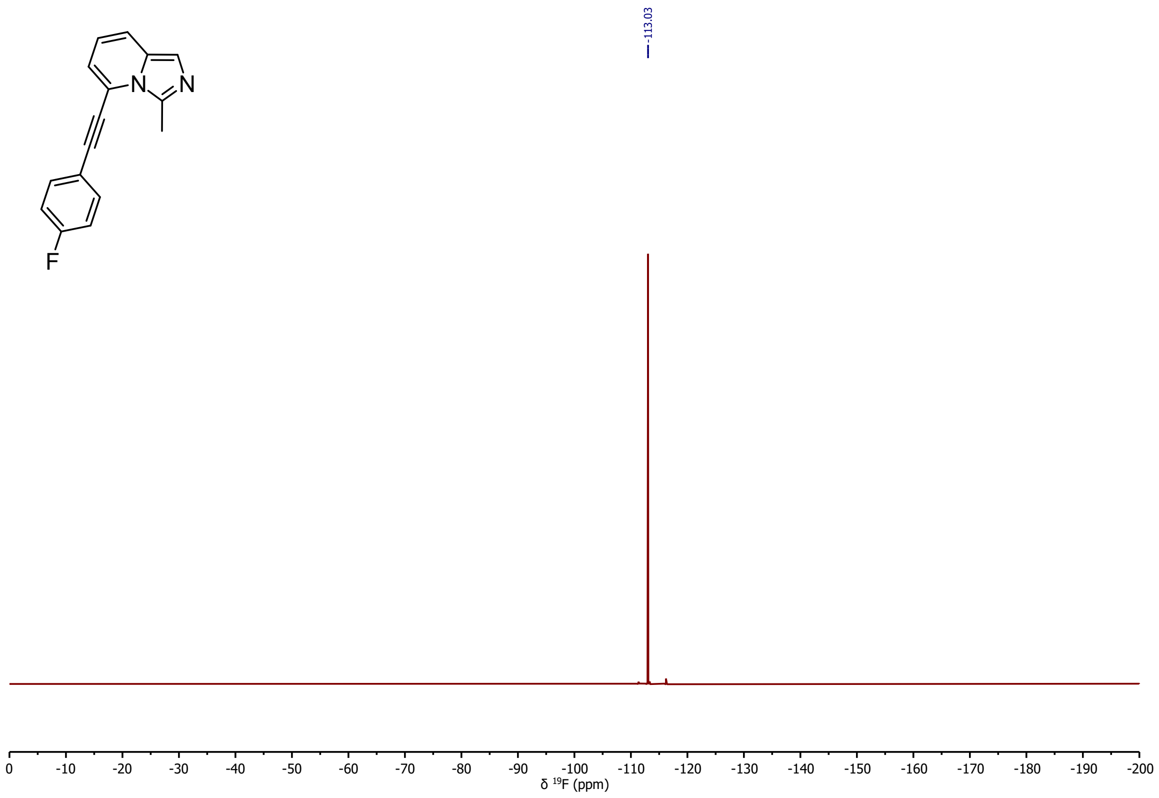


**Figure S111**: **$^{19}$F NMR** (565 MHz, $CDCl_3$, 298 K) of **S23**.

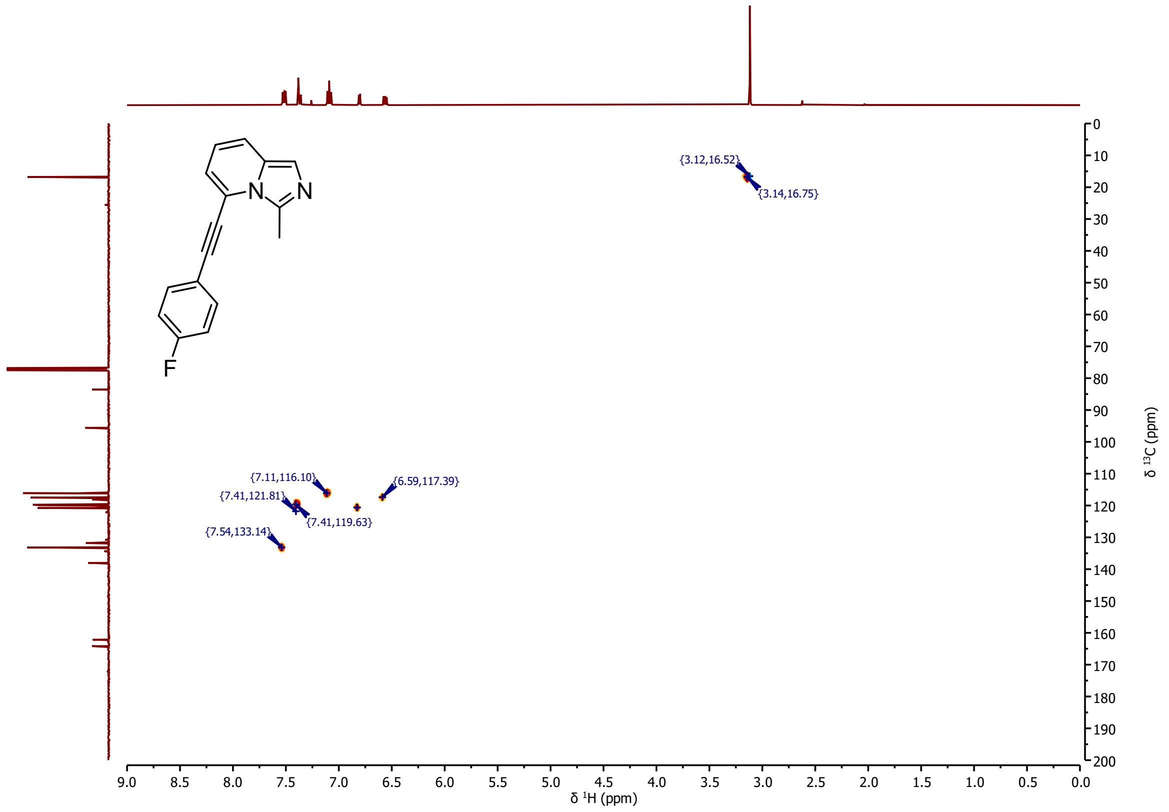


**Figure S112**: **$^{1}$H/$^{13}$C HSQC** (500/126 MHz, $CDCl_3$, 298 K) of **S23**.

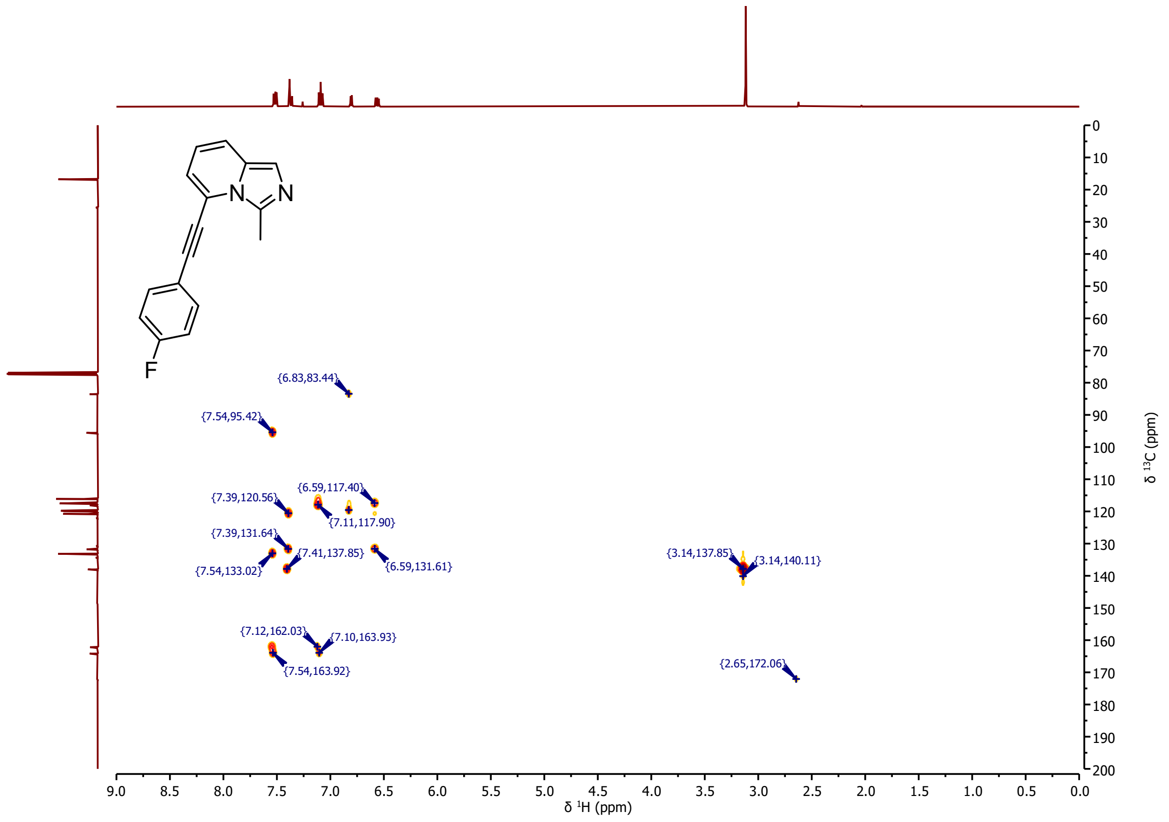

**Figure S113**: **$^1$H/$^{13}$C HMBC** (500/126 MHz, $CDCl_3$, 298 K) of **S23**.

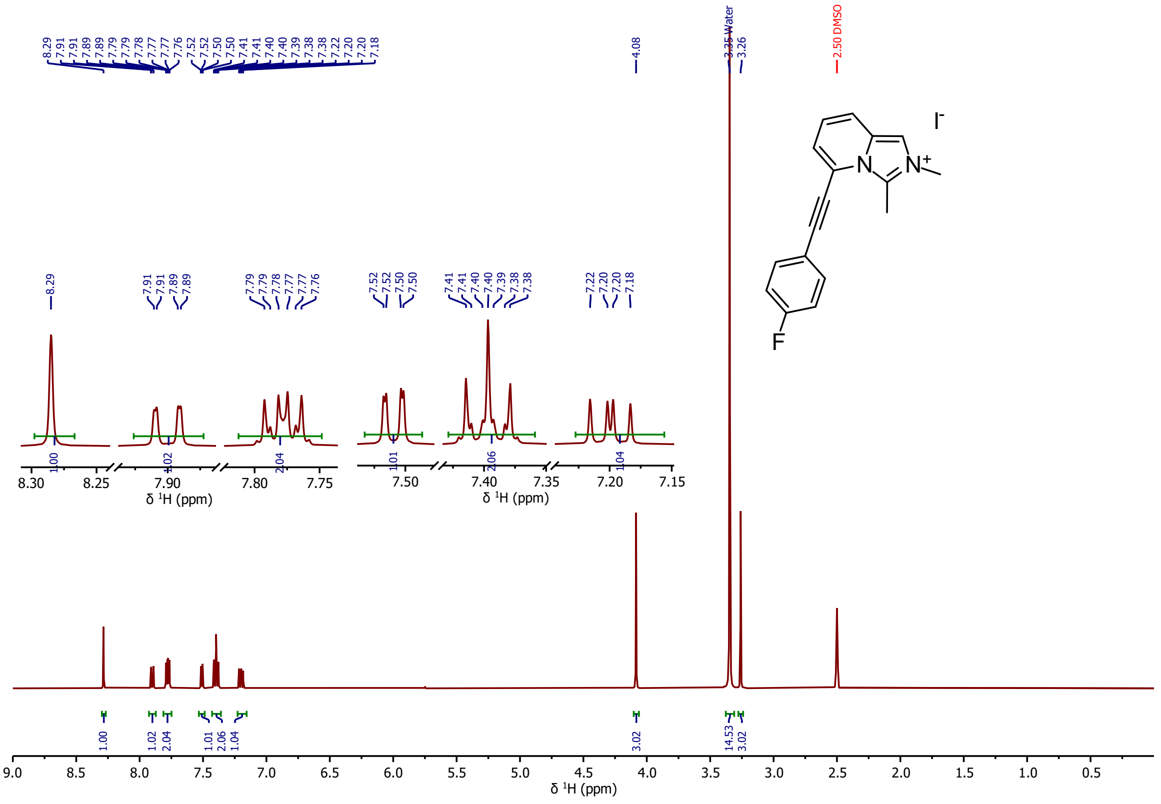

**Figure S114**: **$^1$H NMR** (500 MHz, DMSO-$d_6$, 298 K) of **4d**.

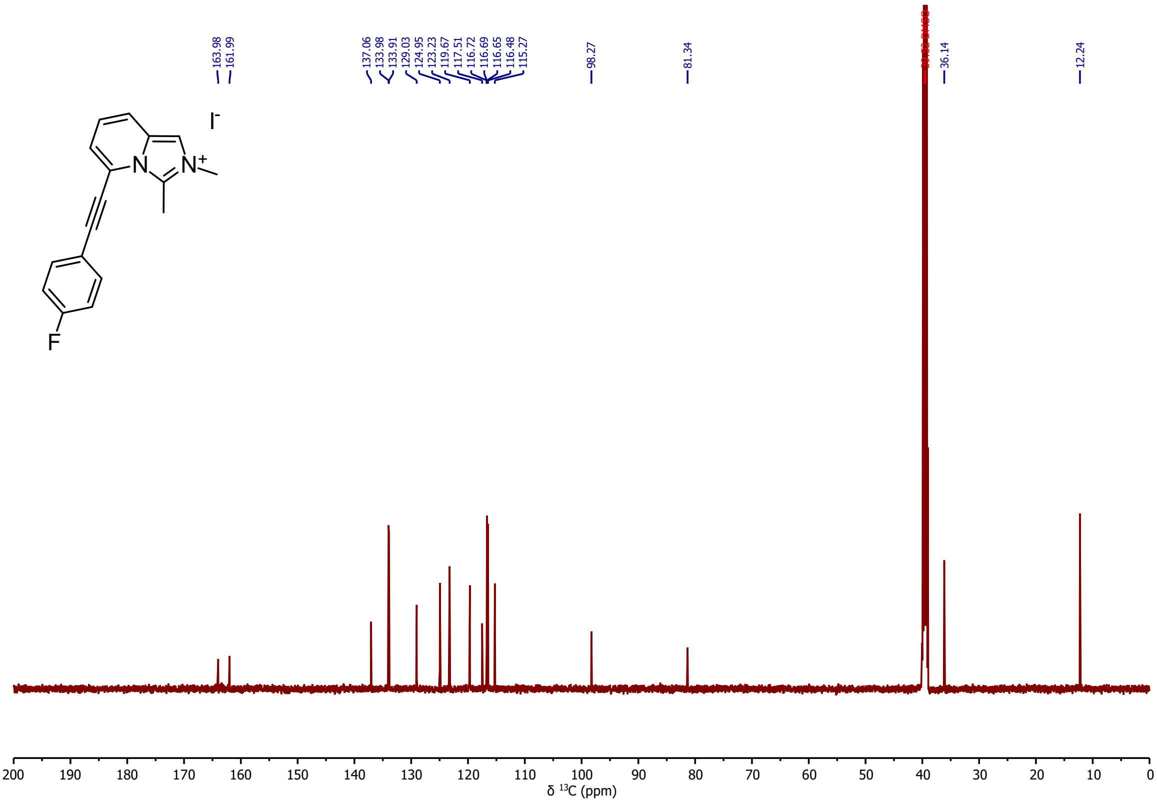


**Figure S115**: **$^{13}$C NMR** (126 MHz, DMSO-d$_6$, 298 K) of **4d**.

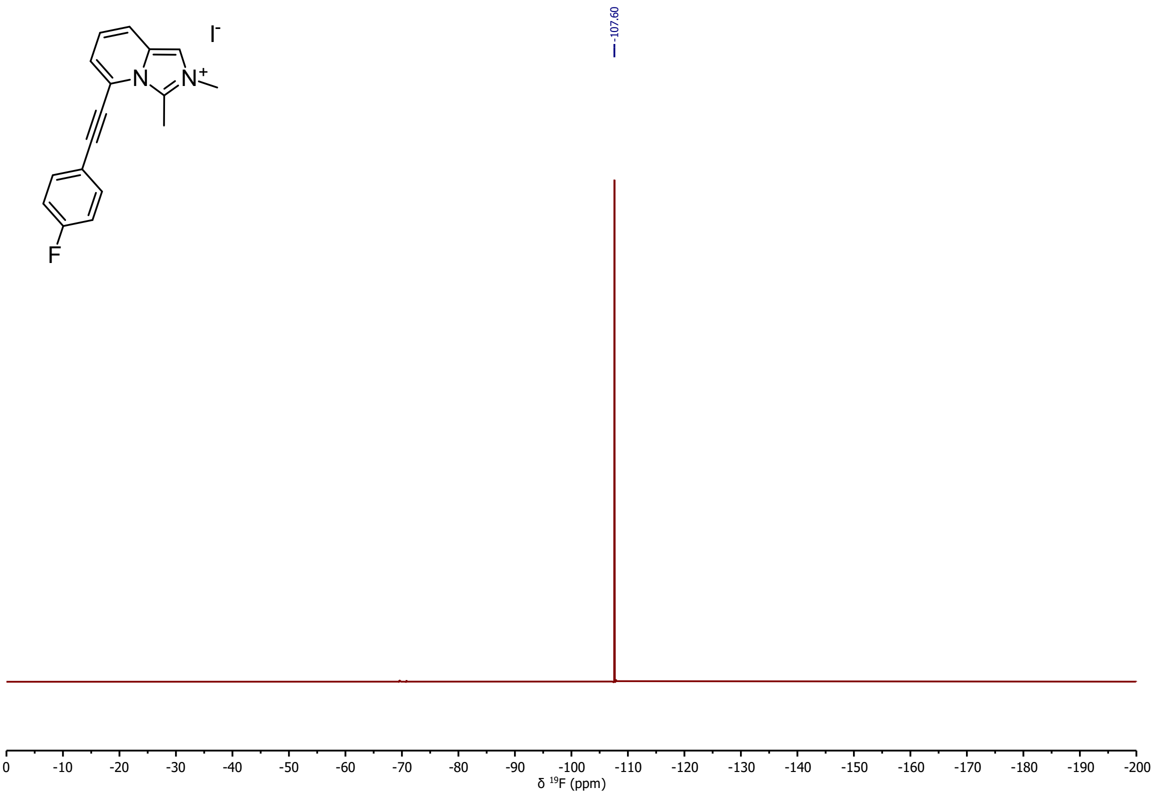


**Figure S116**: **$^{19}$F NMR** (565 MHz, DMSO-d$_6$, 298 K) of **4d**.

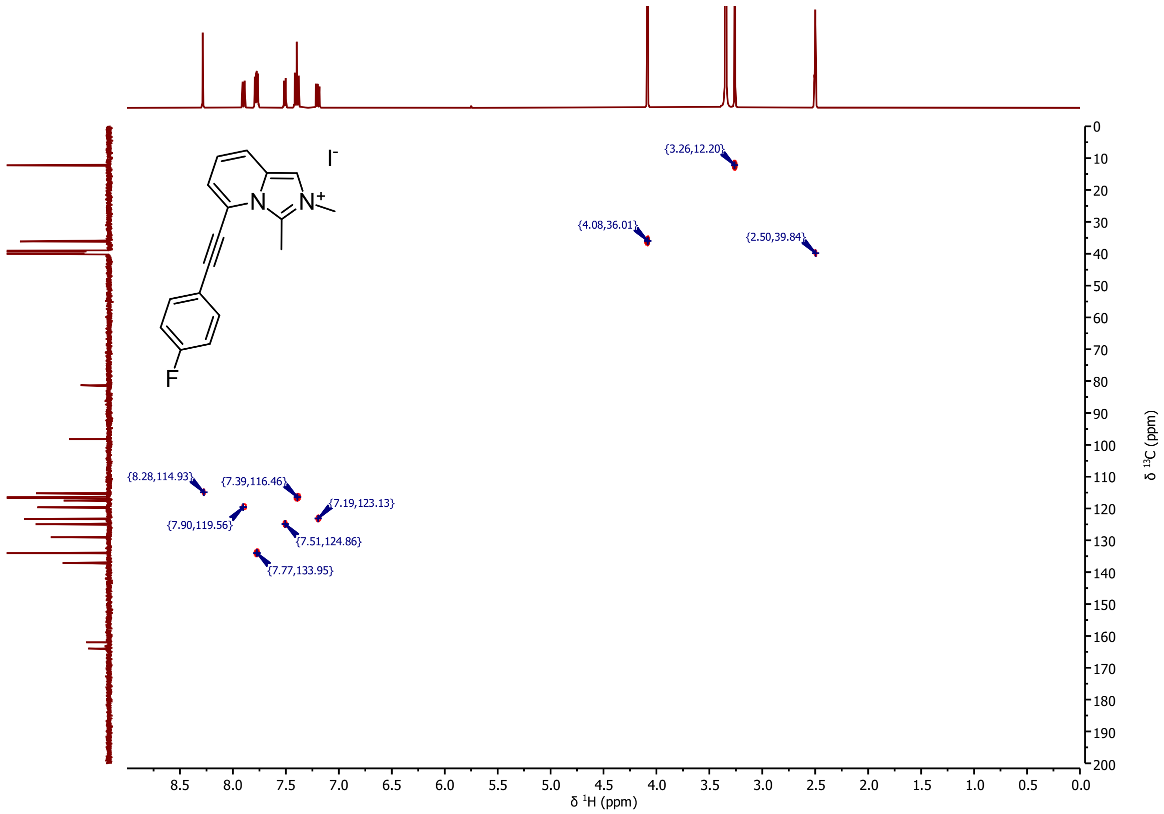


**Figure S117**: **$^1$H/$^{13}$C HSQC** (500/126 MHz, DMSO-d$_6$, 298 K) of **4d**.

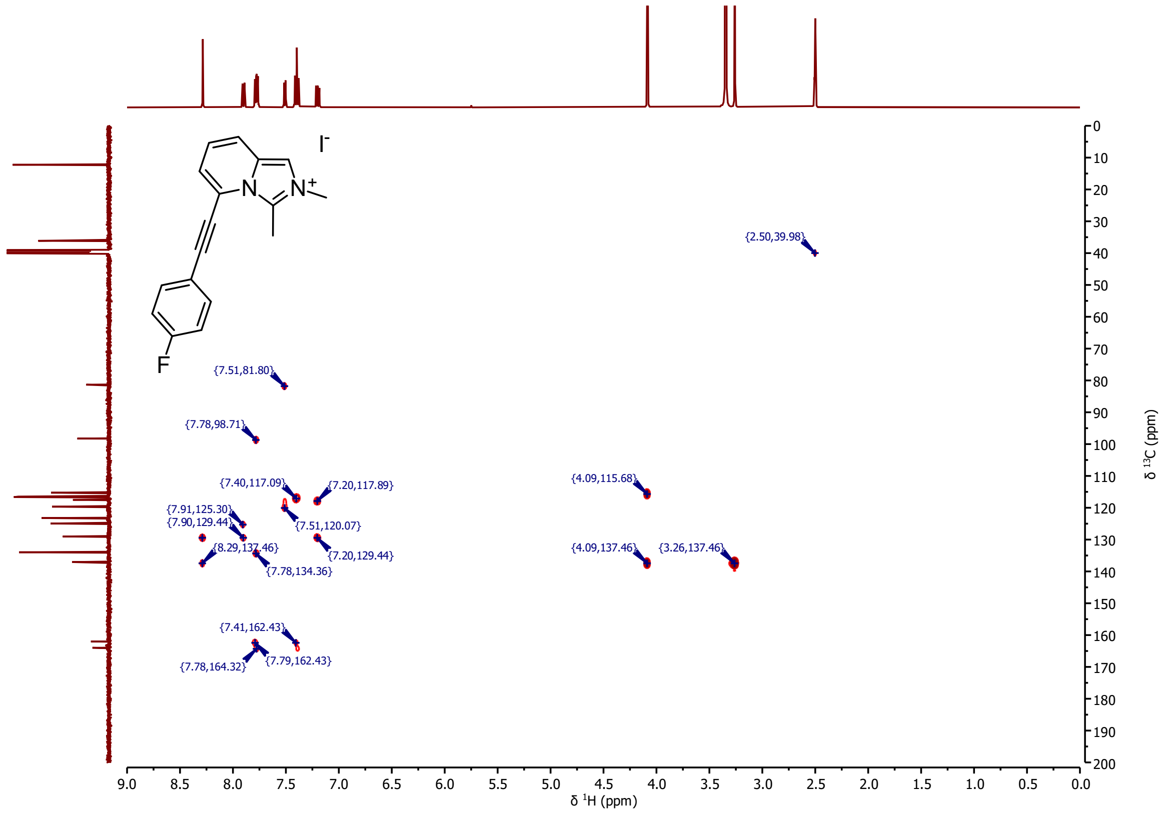


**Figure S118**: **$^1$H/$^{13}$C HMBC** (500/126 MHz, DMSO-d$_6$, 298 K) of **4d**.

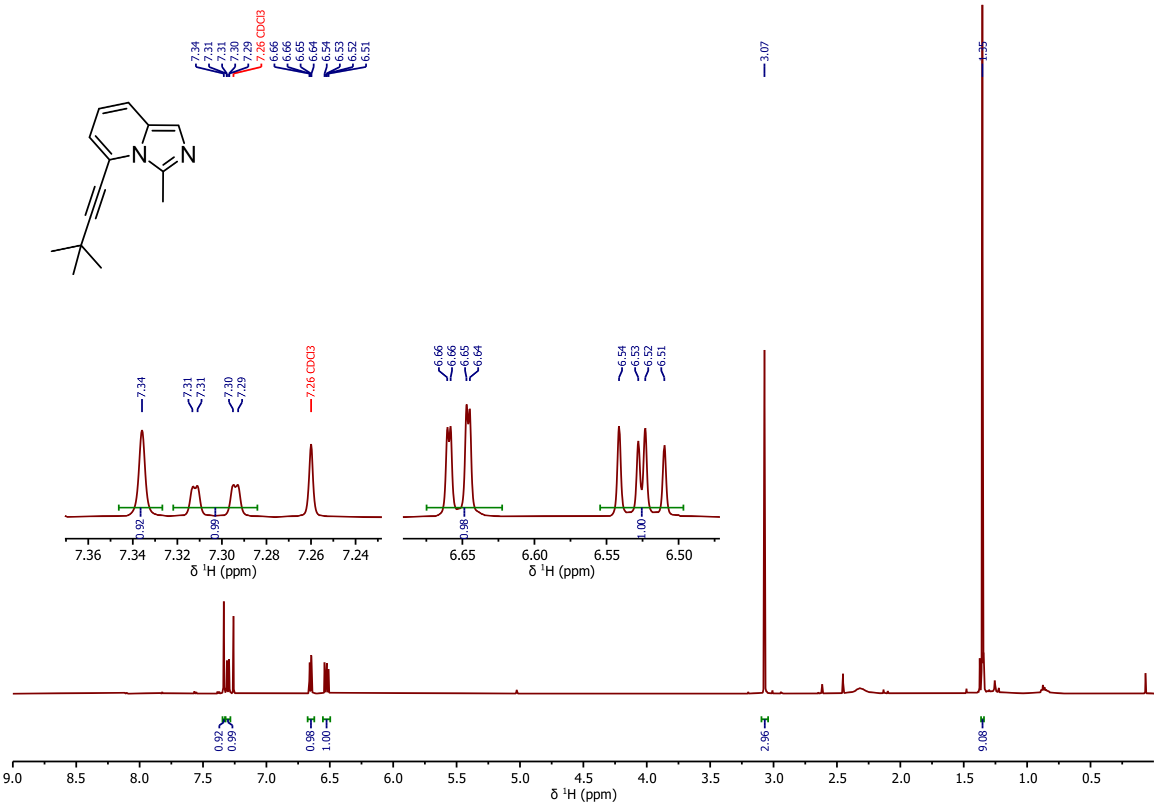

**Figure S119**: **$^1$H NMR** (500 MHz, $CDCl_3$, 298 K) of **S24**.

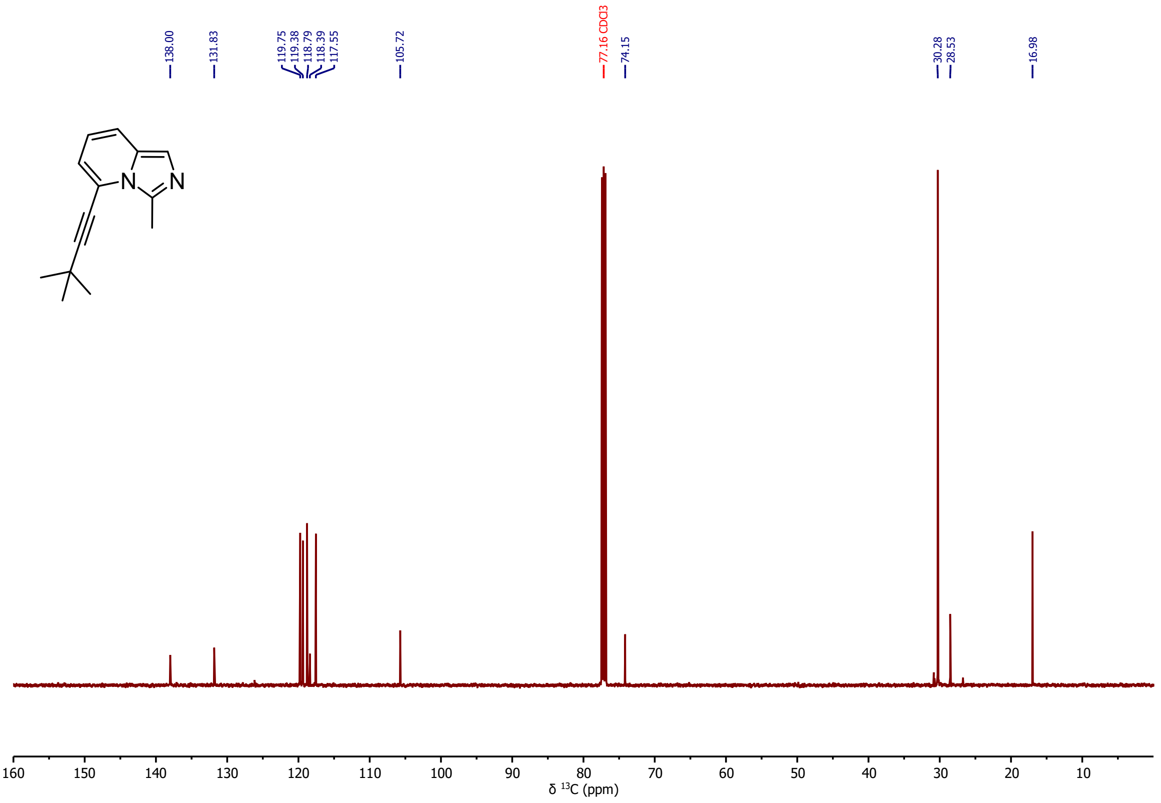

**Figure S120**: **$^{13}$C NMR** (126 MHz, $CDCl_3$, 298 K) of **S24**.

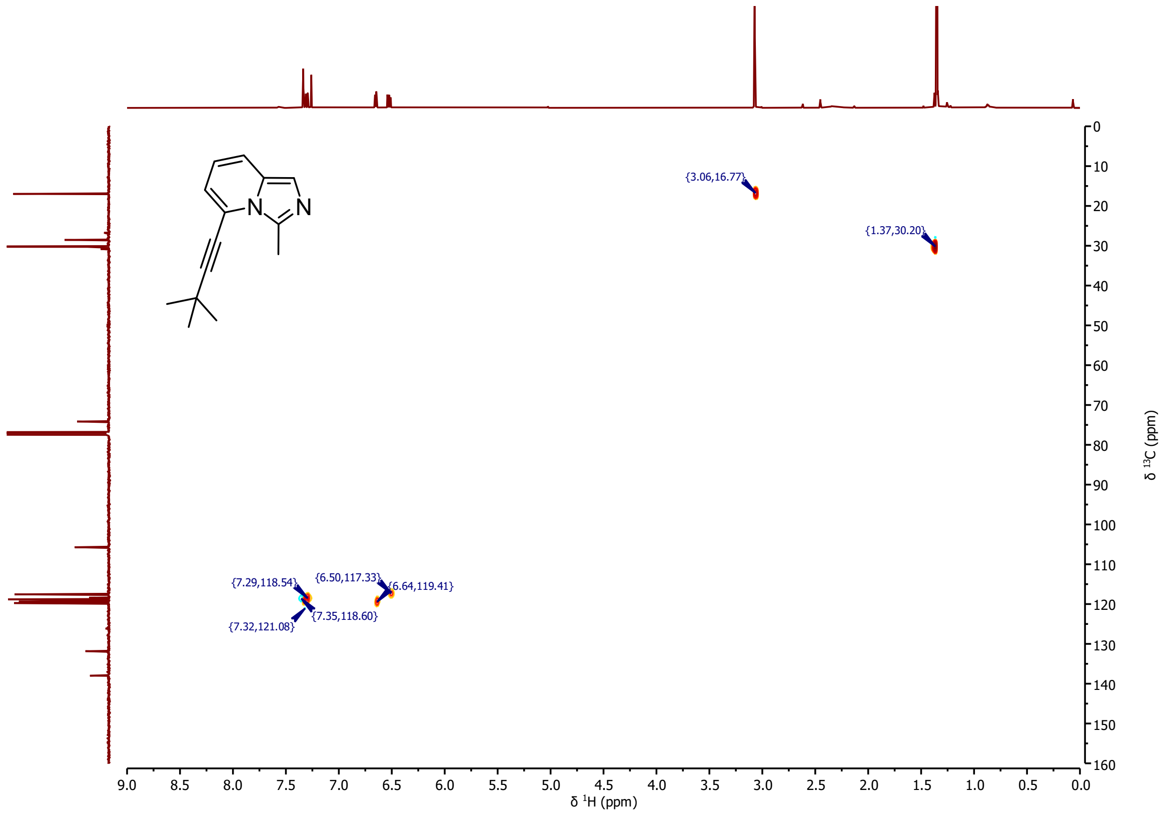


**Figure S121**: **$^{1}$H/$^{13}$C HSQC** (500/126 MHz, $CDCl_3$, 298 K) of **S24**.

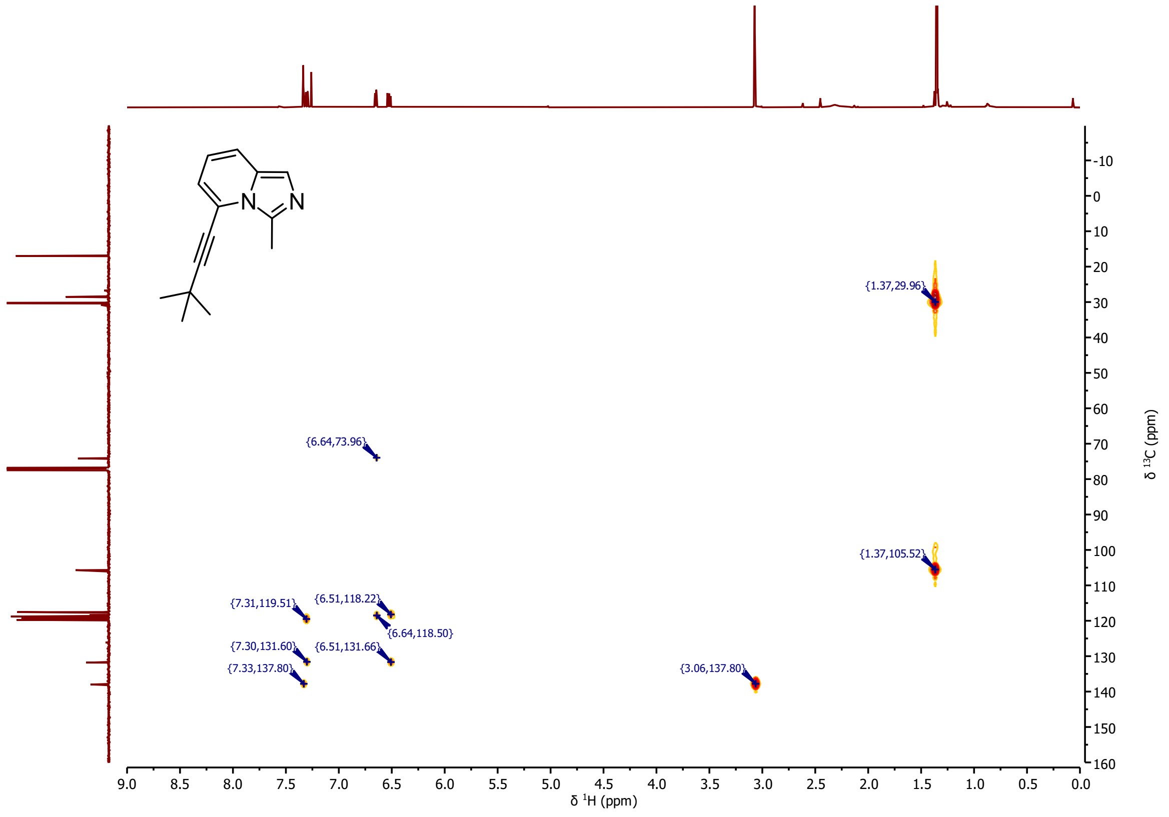


**Figure S122**: **$^{1}$H/$^{13}$C HMBC** (500/126 MHz, $CDCl_3$, 298 K) of **S24**.

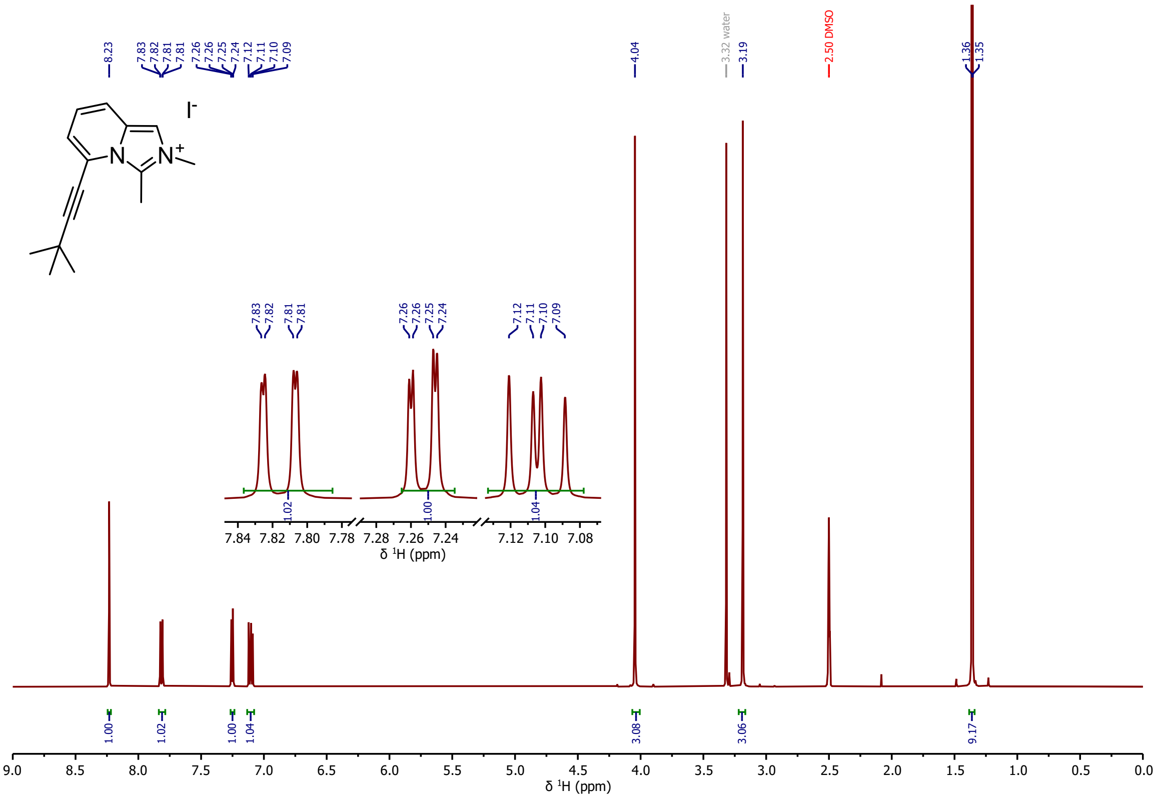

**Figure S123**: **$^{1}$H NMR** (500 MHz, DMSO-$d_6$, 298 K) of **4e**.

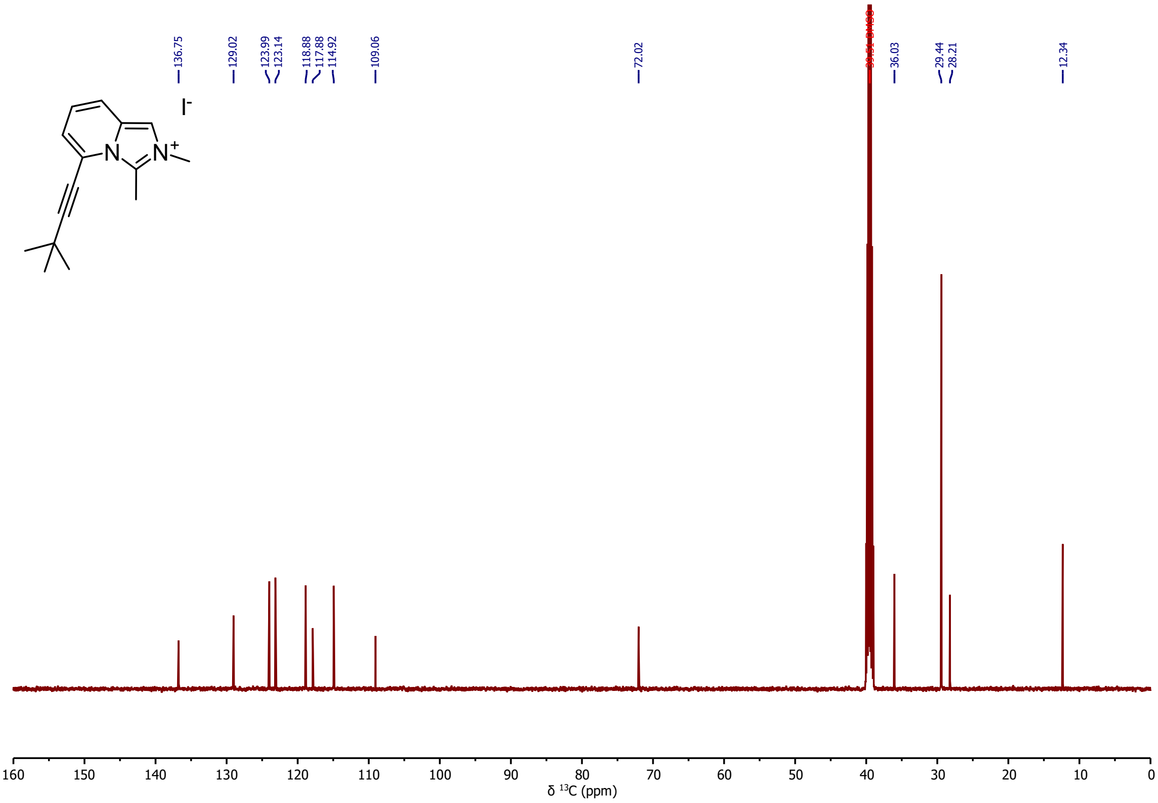

**Figure S124**: **$^{13}$C NMR** (126 MHz, DMSO-$d_6$, 298 K) of **4e**.

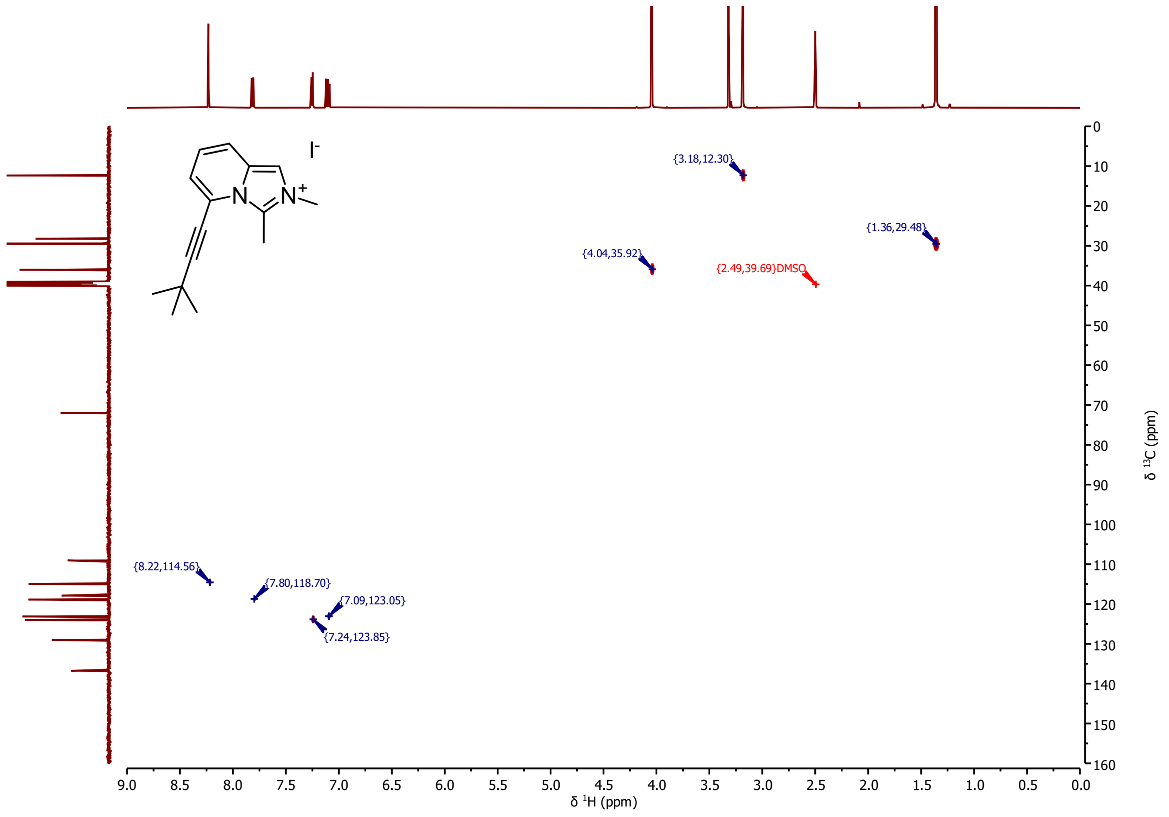


**Figure S125**: **$^{1}$H/$^{13}$C HSQC** (500/126 MHz, DMSO-d$_6$, 298 K) of **4e**.

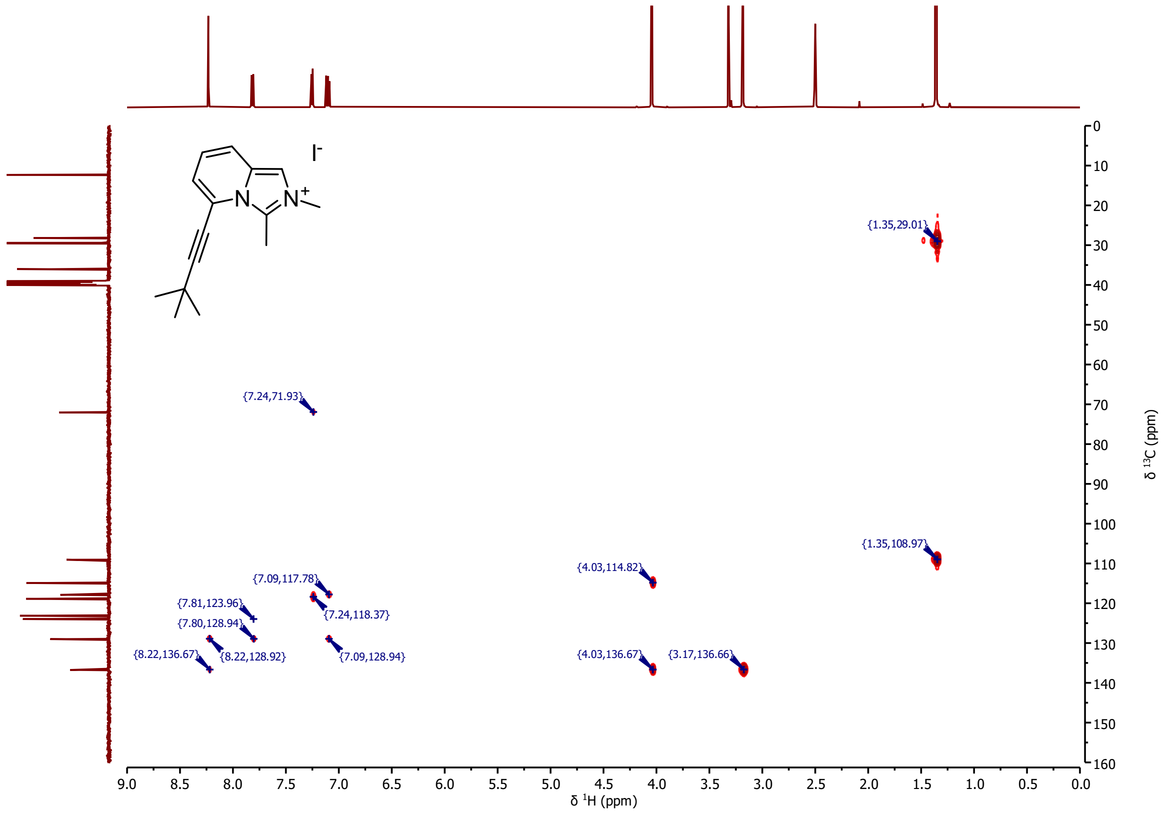


**Figure S126**: **$^{1}$H/$^{13}$C HMBC** (500/126 MHz, DMSO-d$_6$, 298 K) of **4e**.

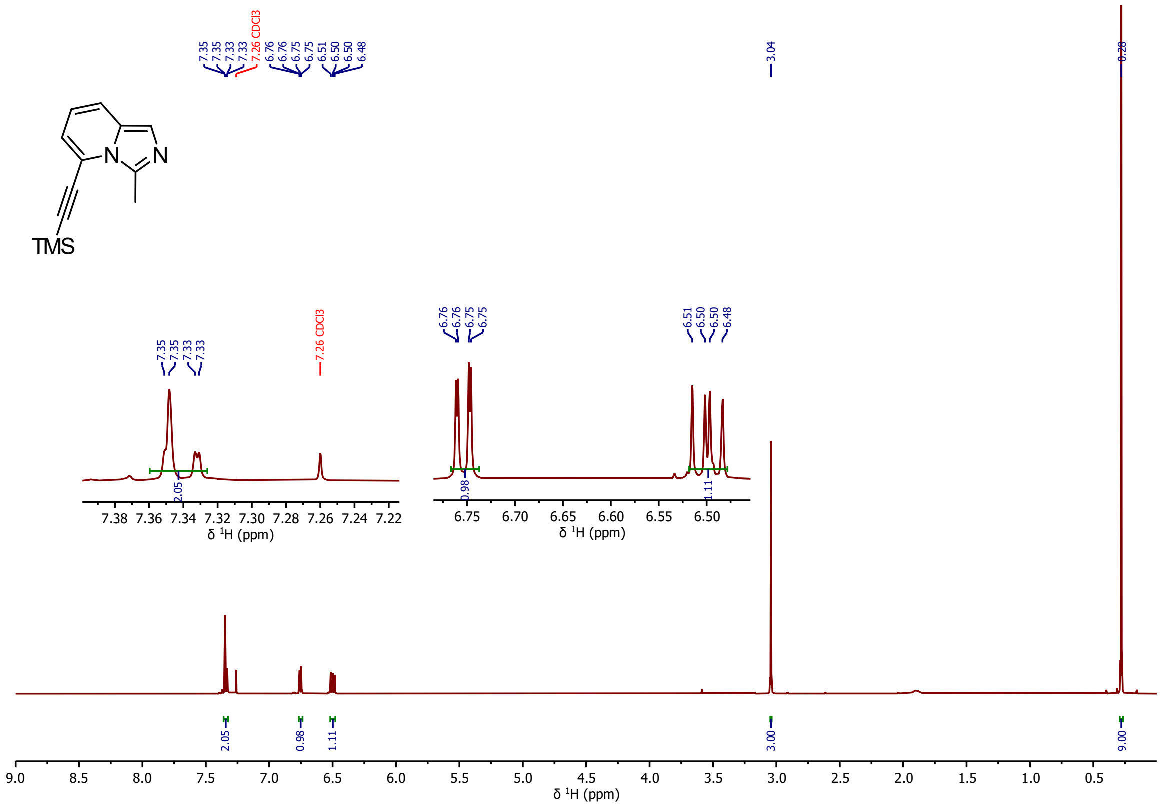

**Figure S127**: **$^1$H NMR** (500 MHz, $CDCl_3$, 298 K) of **S25**.

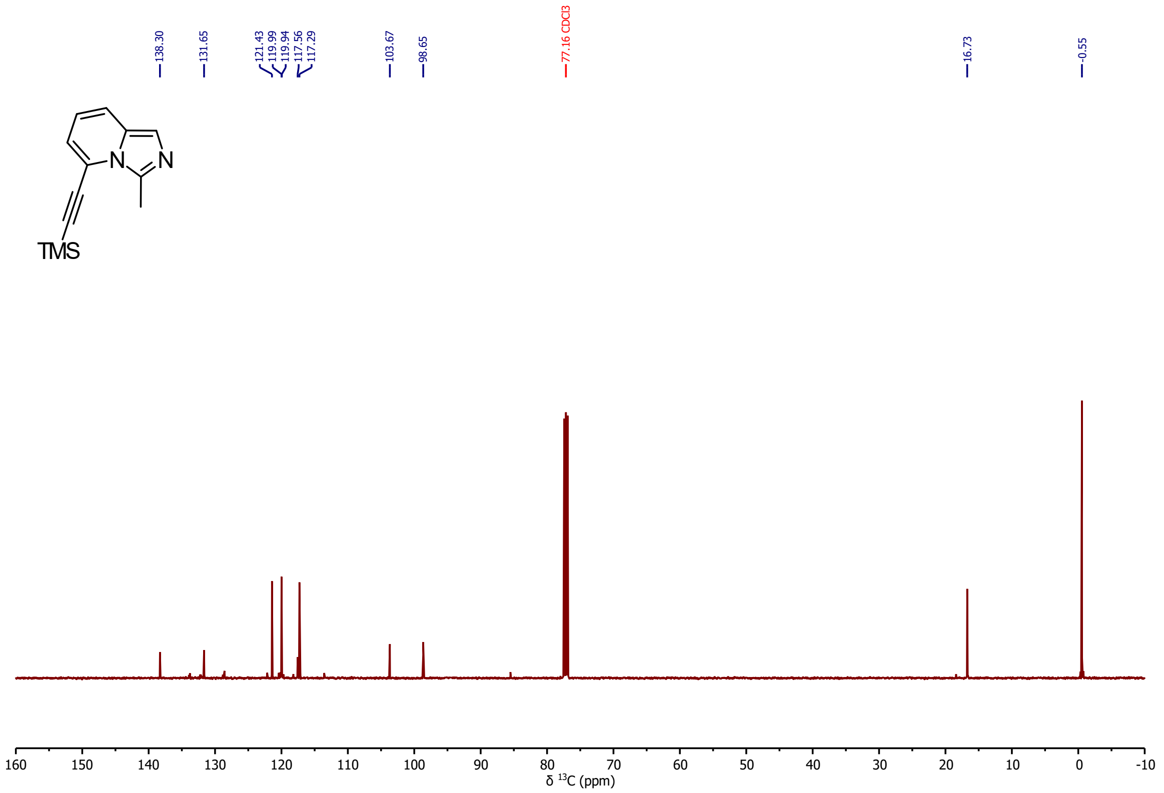

**Figure S128**: **$^{13}$C NMR** (126 MHz, $CDCl_3$, 298 K) of **S25**.

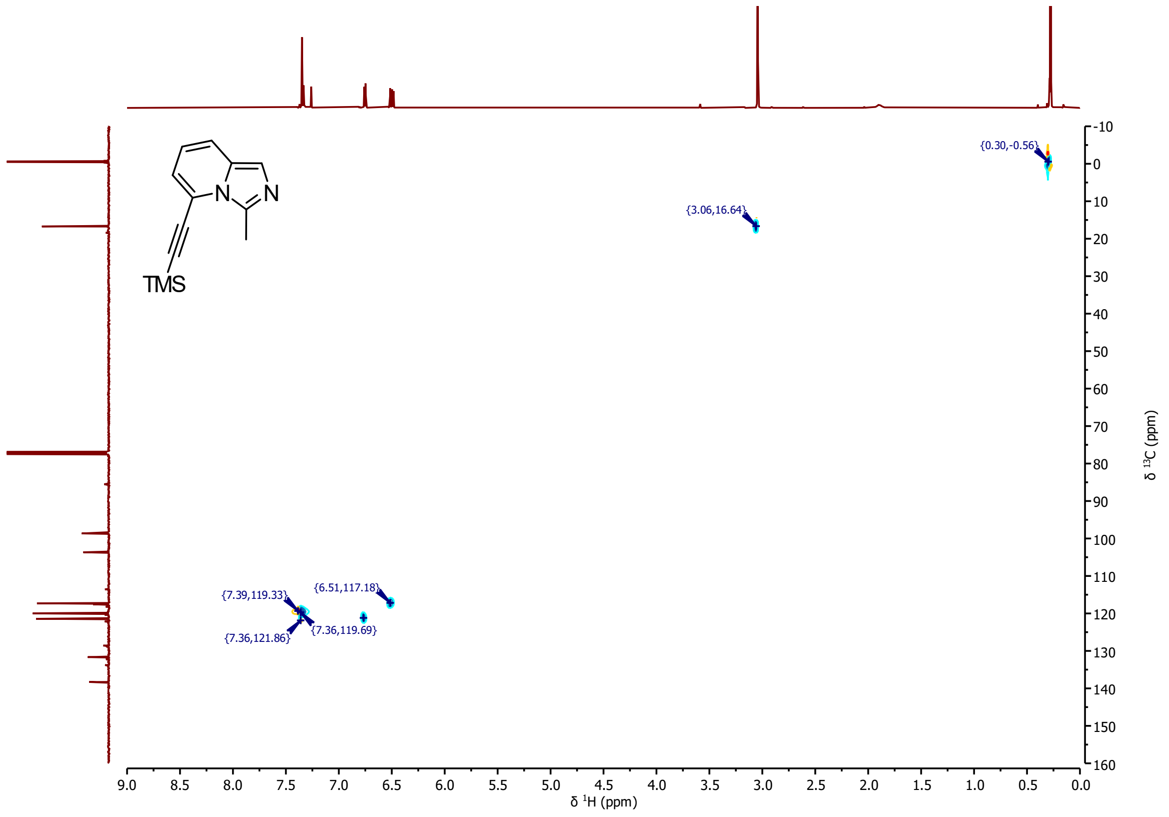


**Figure S129**: **$^1$H/$^{13}$C HSQC** (500/126 MHz, $CDCl_3$, 298 K) of **S25**.

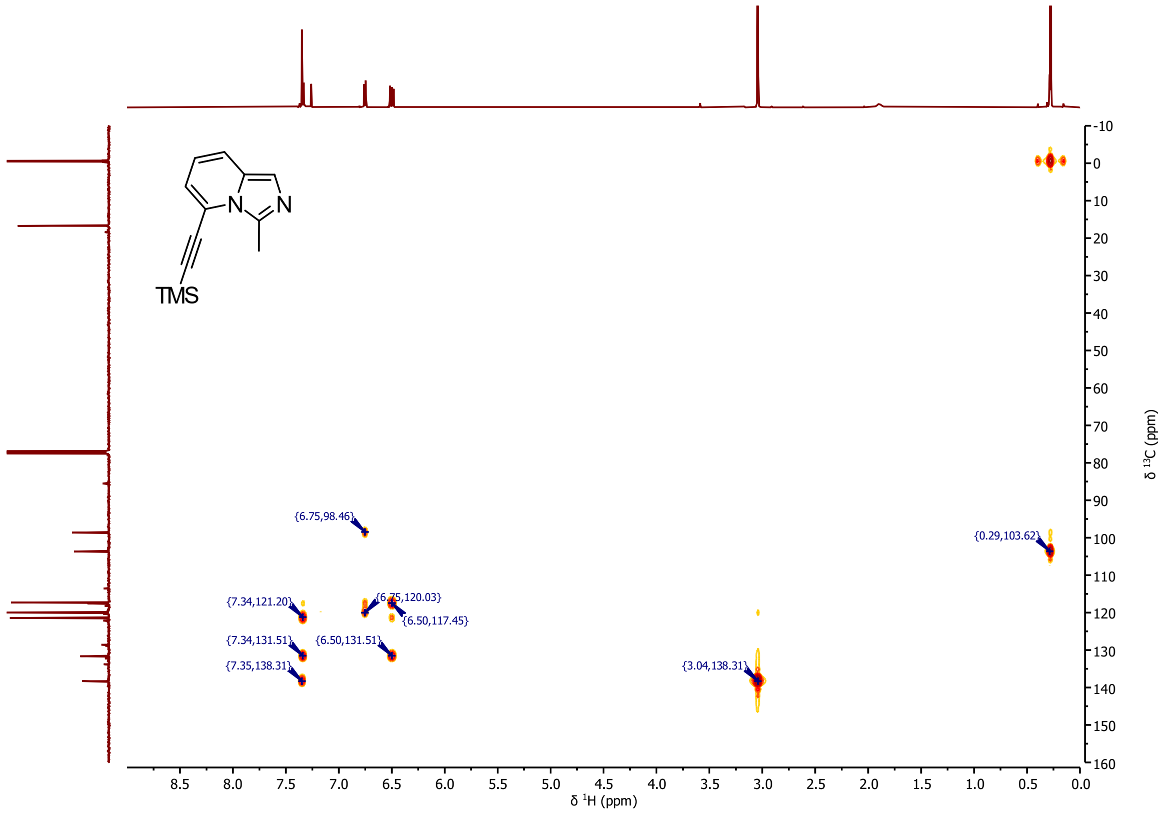


**Figure S130**: **$^1$H/$^{13}$C HMBC** (500/126 MHz, $CDCl_3$, 298 K) of **S25**.

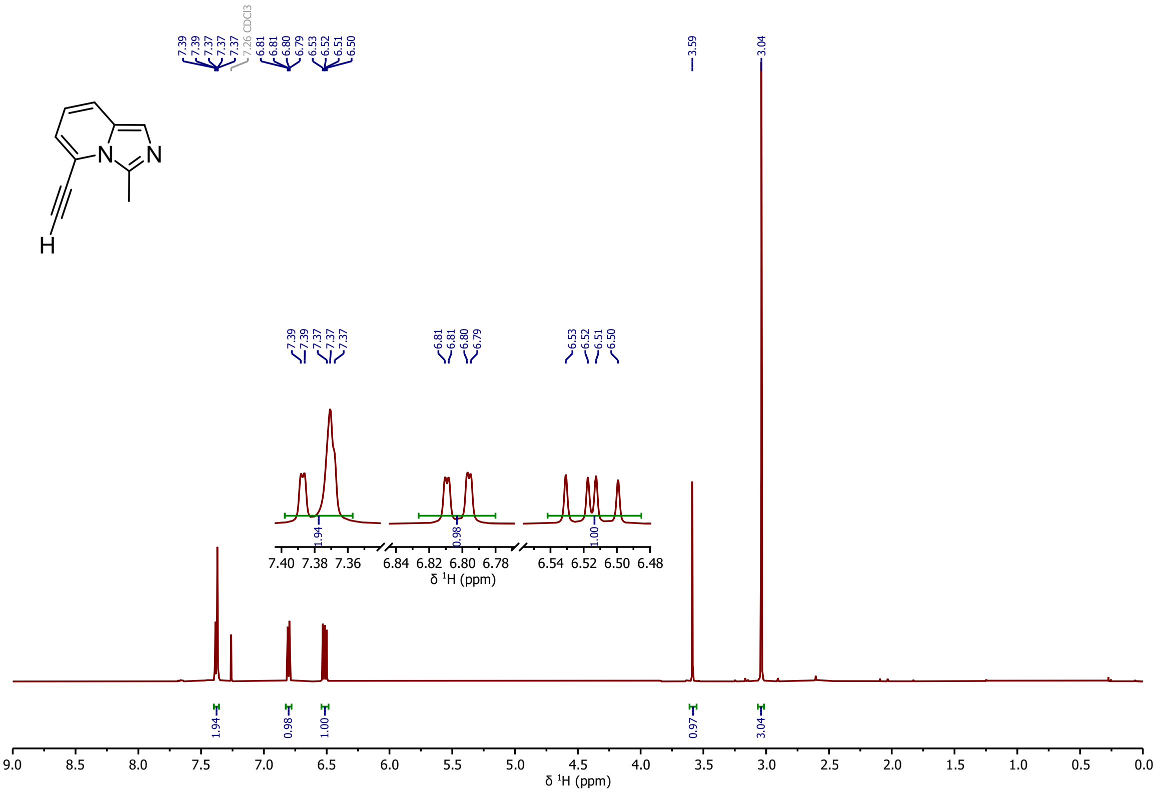

**Figure S131**: **$^{1}$H NMR** (500 MHz, $CDCl_3$, 298 K) of **S26**.

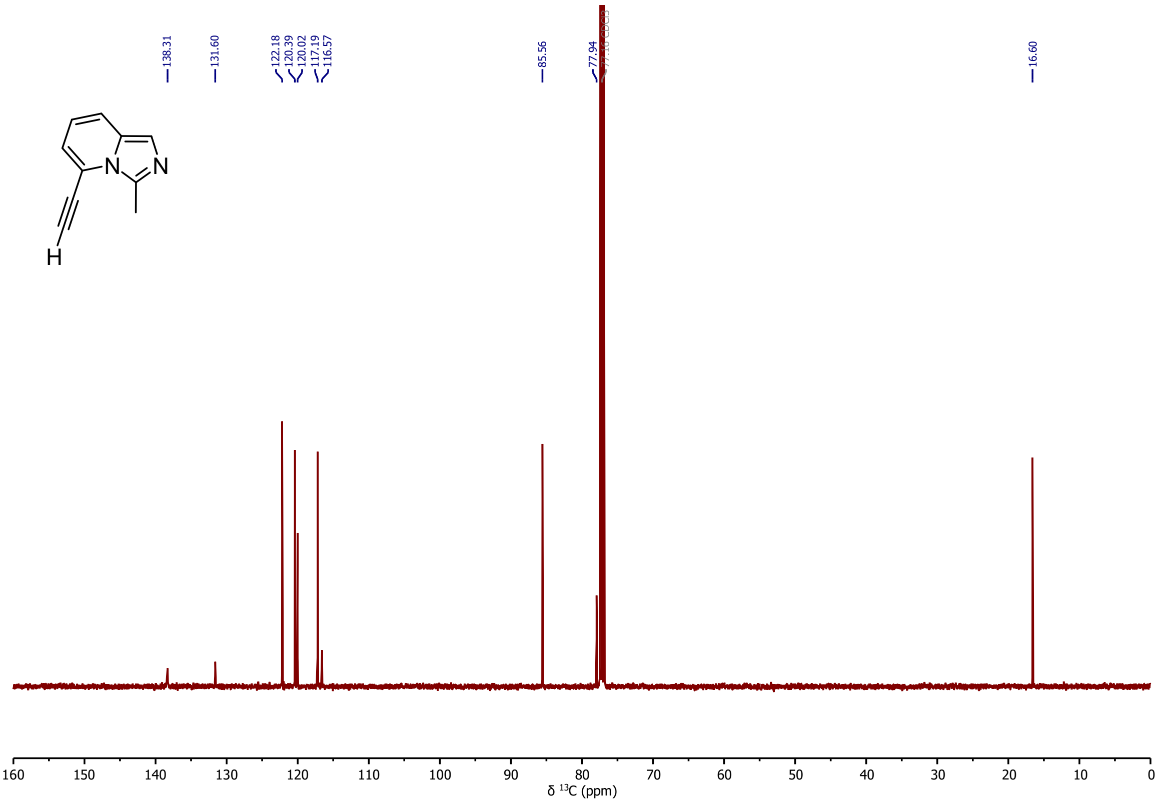

**Figure S132**: **$^{13}$C NMR** (126 MHz, $CDCl_3$, 298 K) of **S26**.

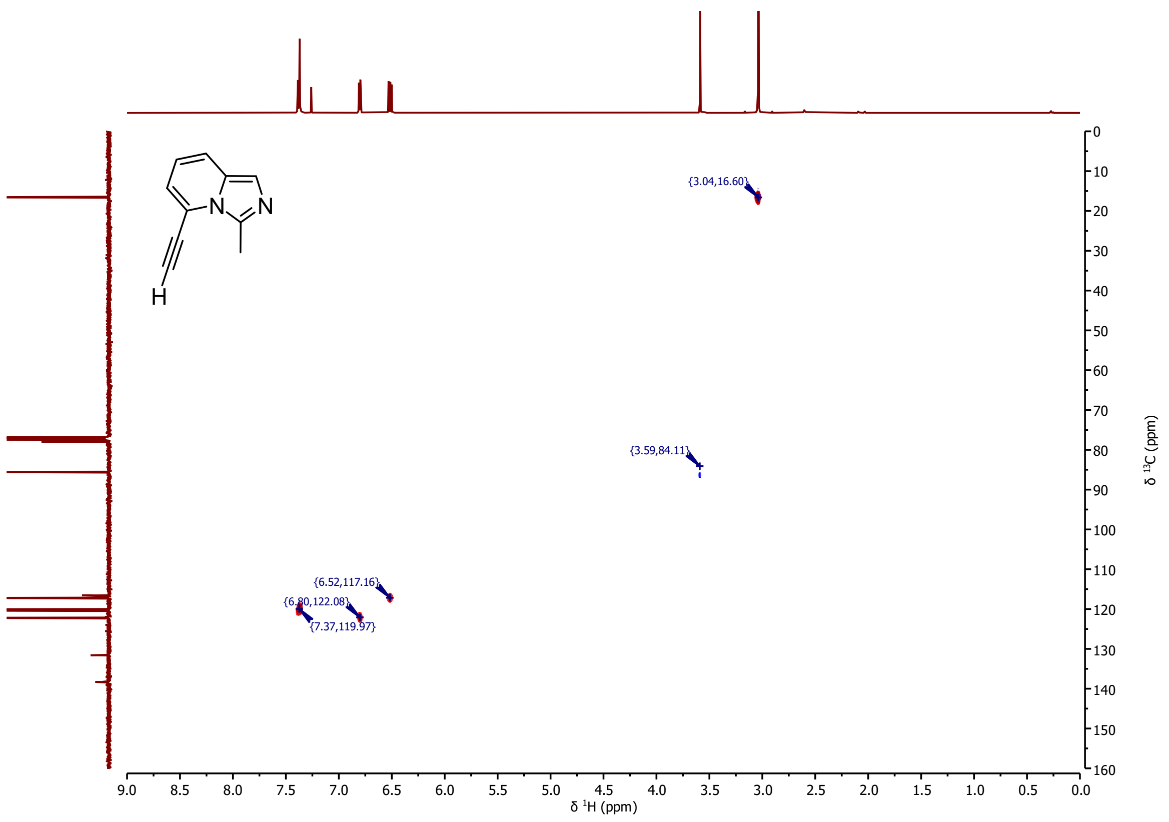


**Figure S133**: **$^{1}$H/$^{13}$C HSQC** (500/126 MHz, $CDCl_3$, 298 K) of **S26**.

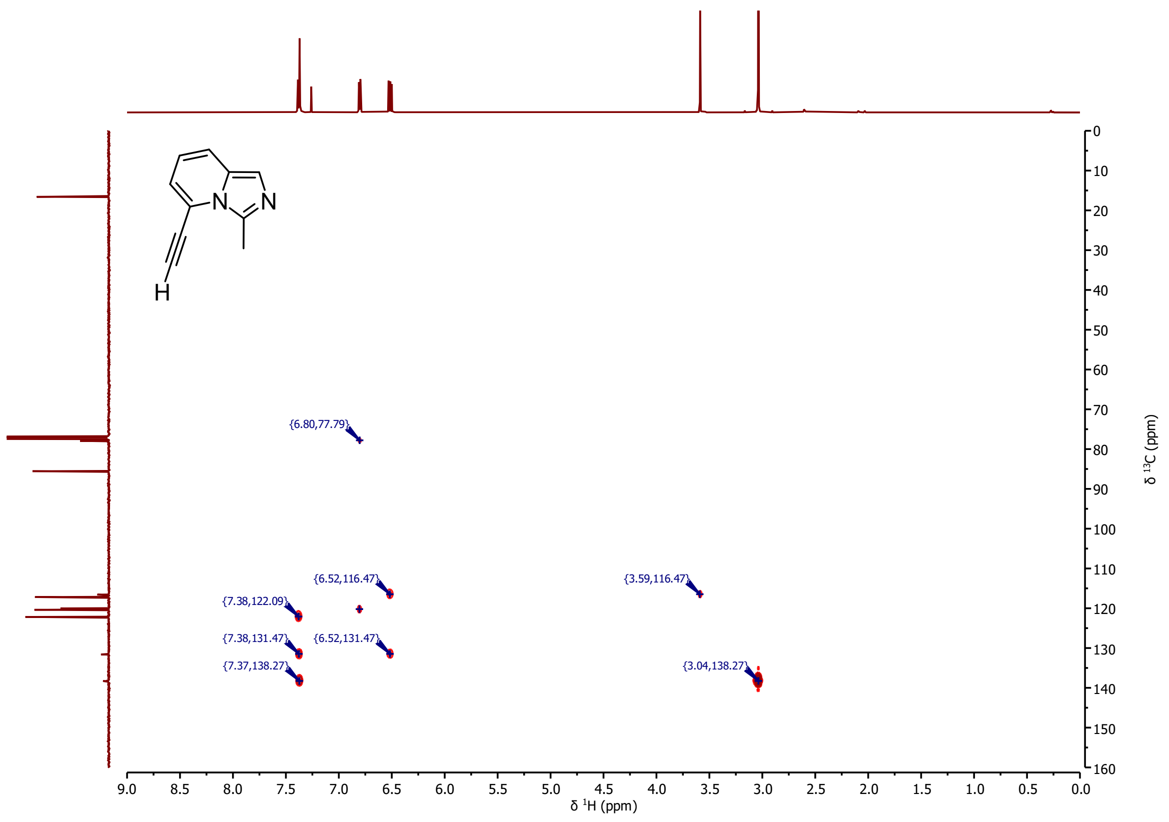


**Figure S134**: **$^{1}$H/$^{13}$C HMBC** (500/126 MHz, $CDCl_3$, 298 K) of **S26**.

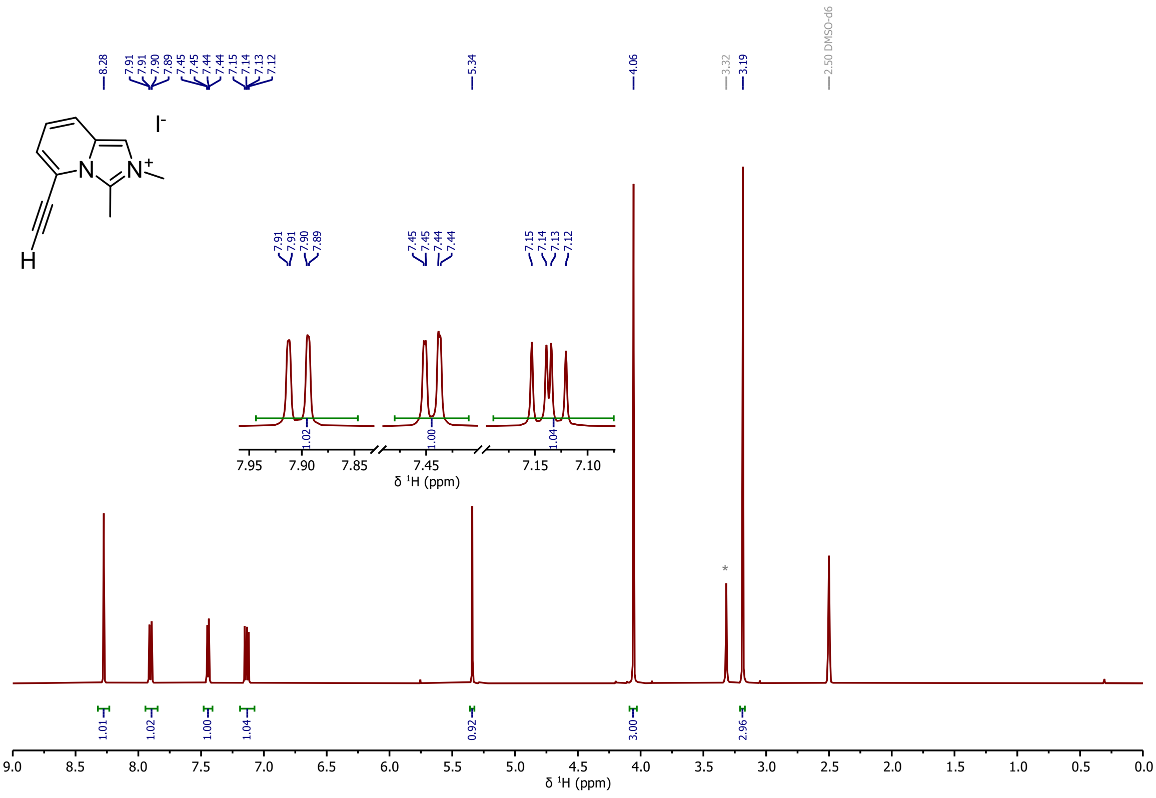

**Figure S135**: **$^{1}$H NMR** (500 MHz, DMSO-d$_6$, 298 K) of **4f**. * = $H_2O$.

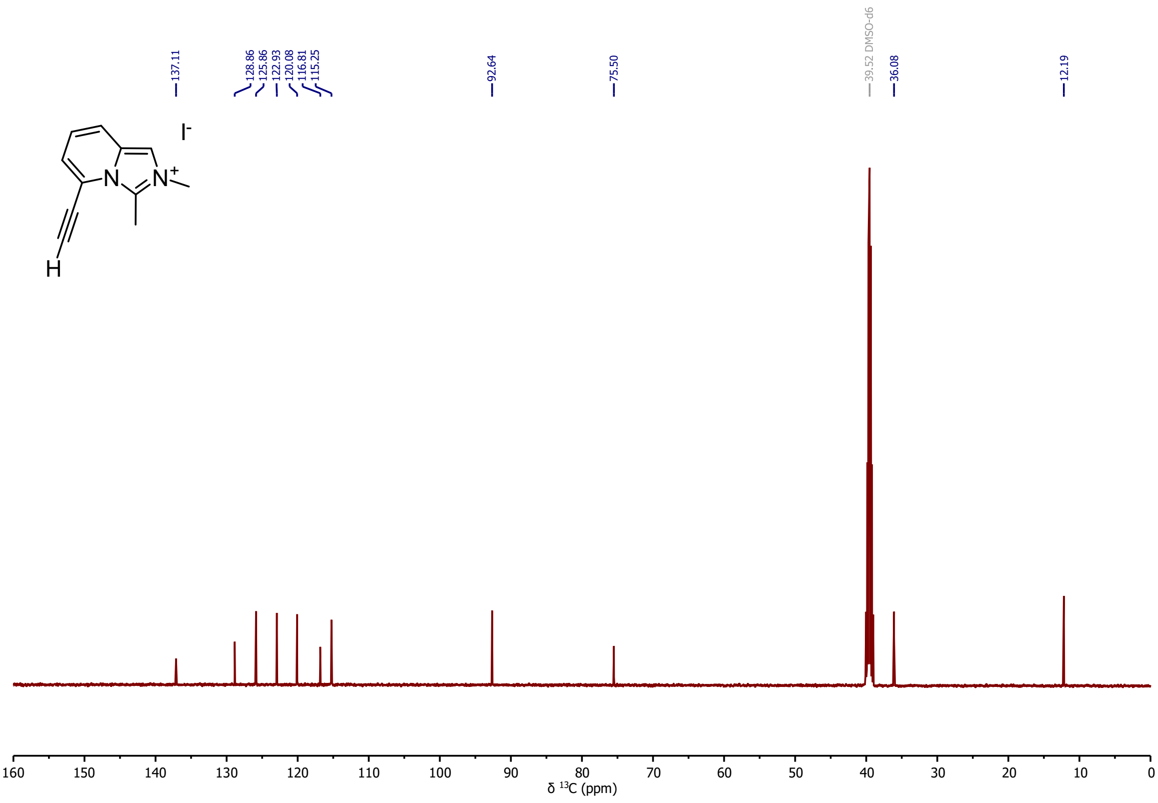

**Figure S136**: **$^{13}$C NMR** (126 MHz, DMSO-d$_6$, 298 K) of **4f**.

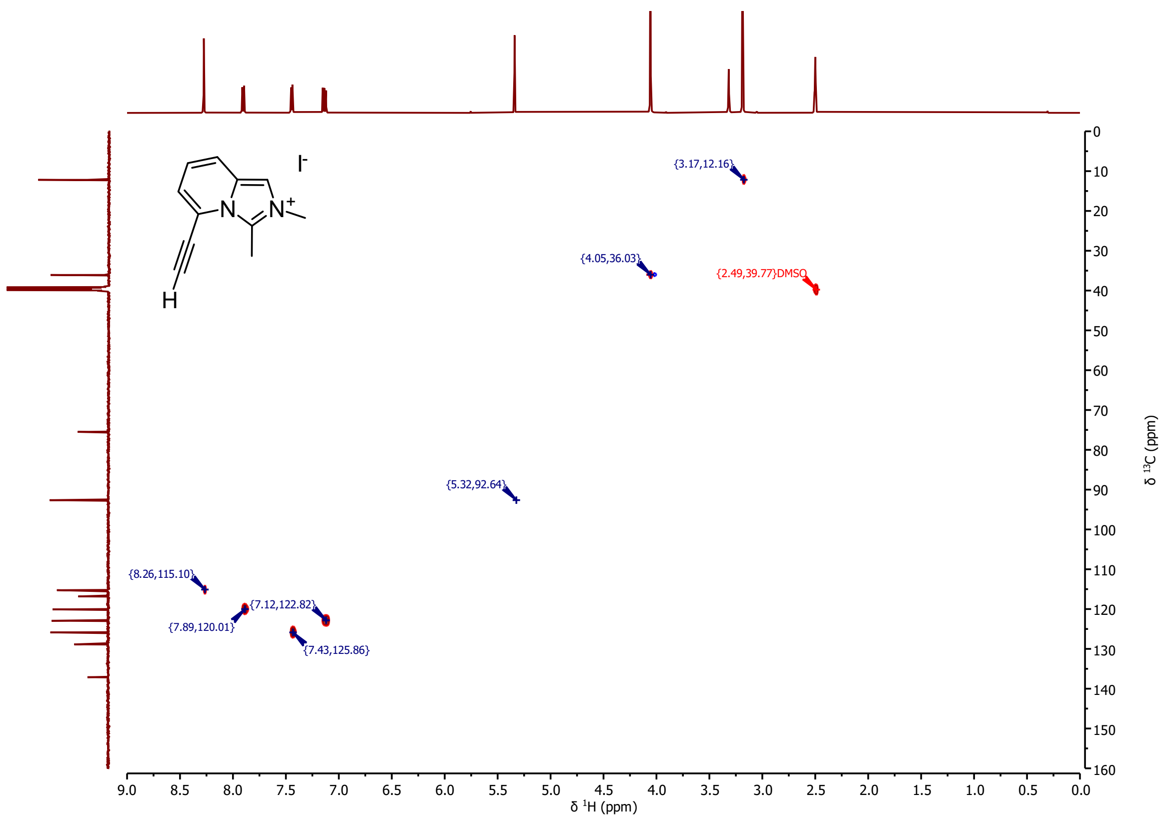


**Figure S137**: **$^1$H/$^{13}$C HSQC** (500/126 MHz, DMSO-d$_6$, 298 K) of **4f**.

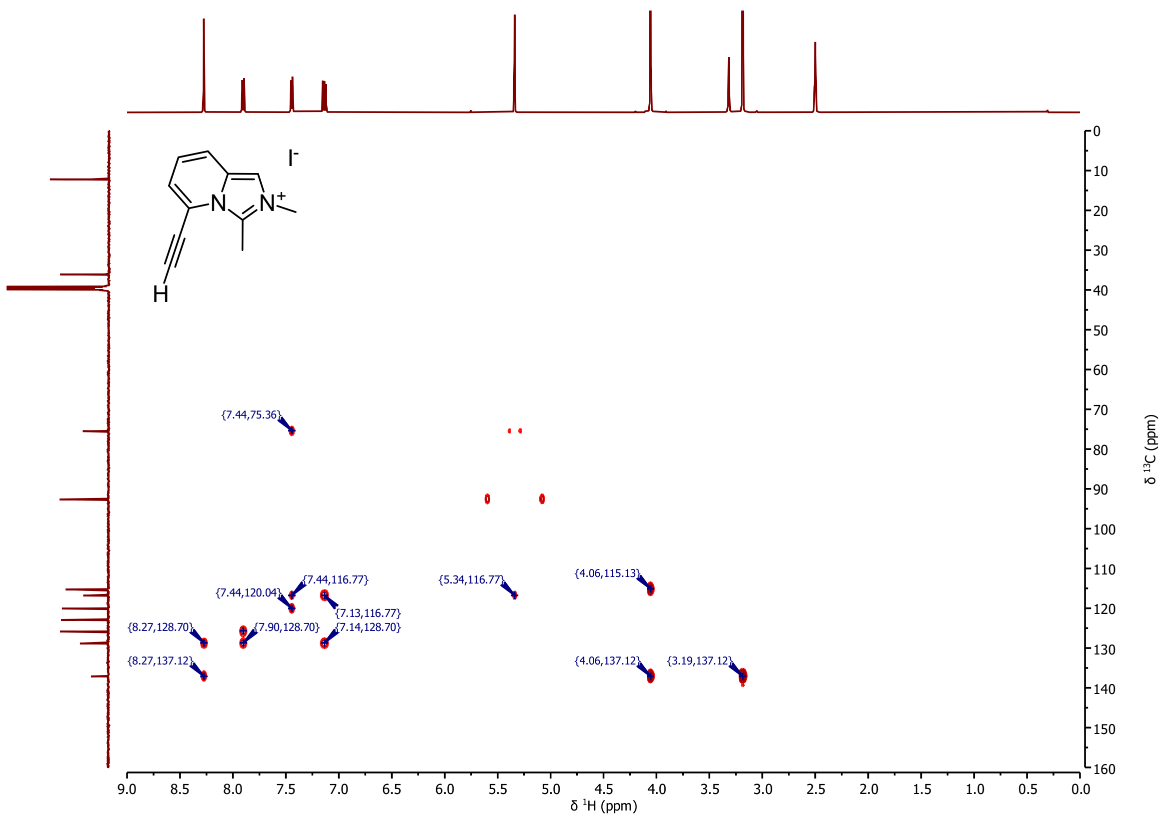


**Figure S138**: **$^1$H/$^{13}$C HMBC** (500/126 MHz, DMSO-d$_6$, 298 K) of **4f**.

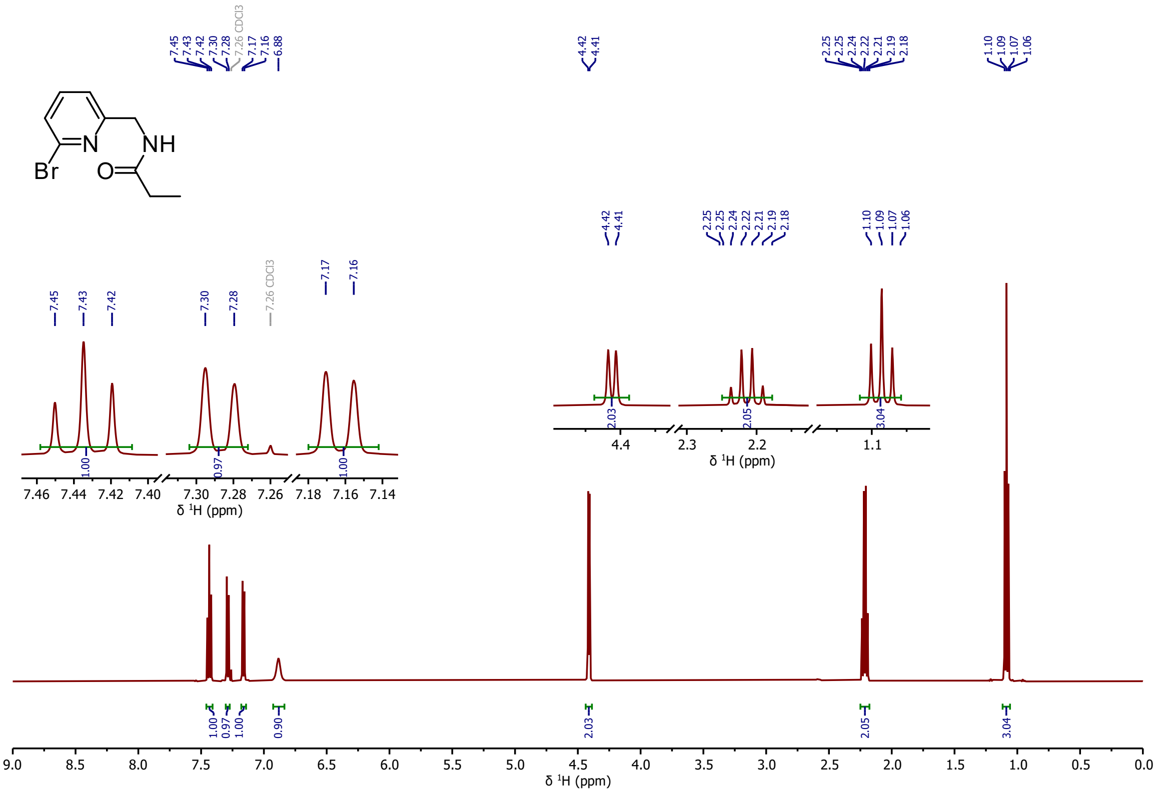

**Figure S139**: **$^{1}$H NMR** (500 MHz, $CDCl_3$, 298 K) of **S27**.

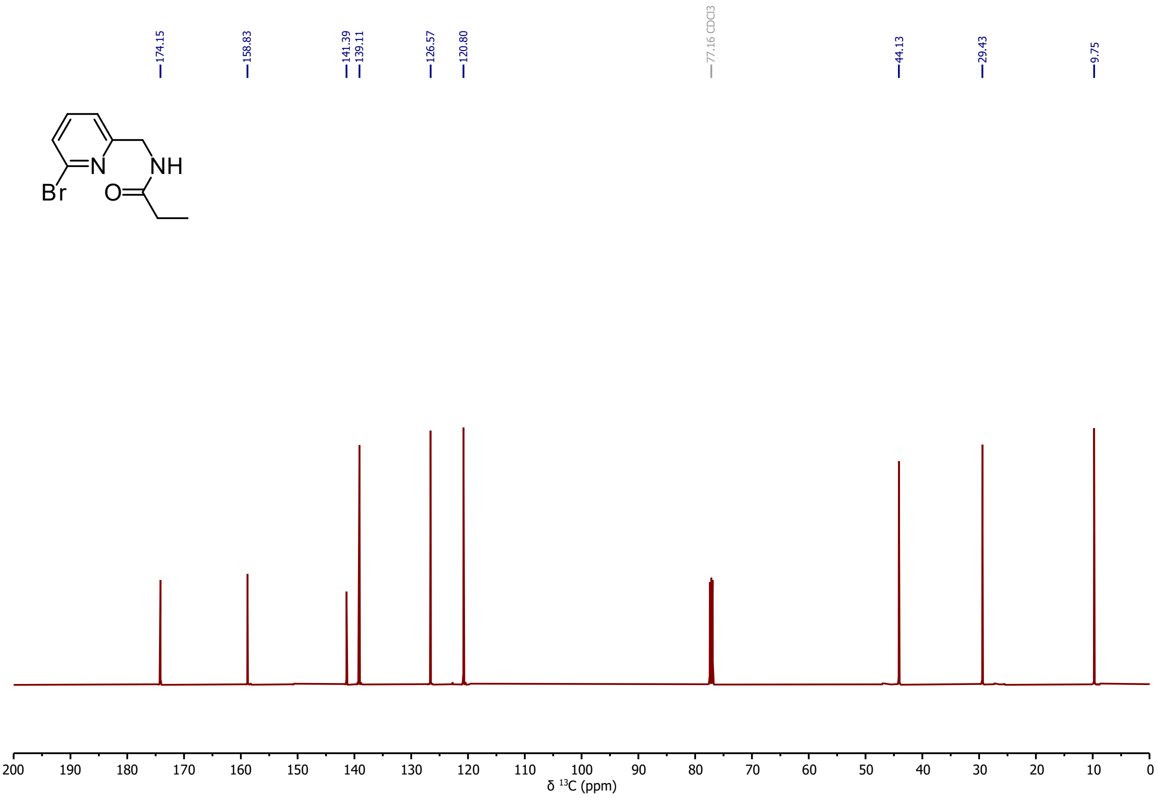

**Figure S140**: **$^{13}$C NMR** (126 MHz, $CDCl_3$, 298 K) of **S27**.

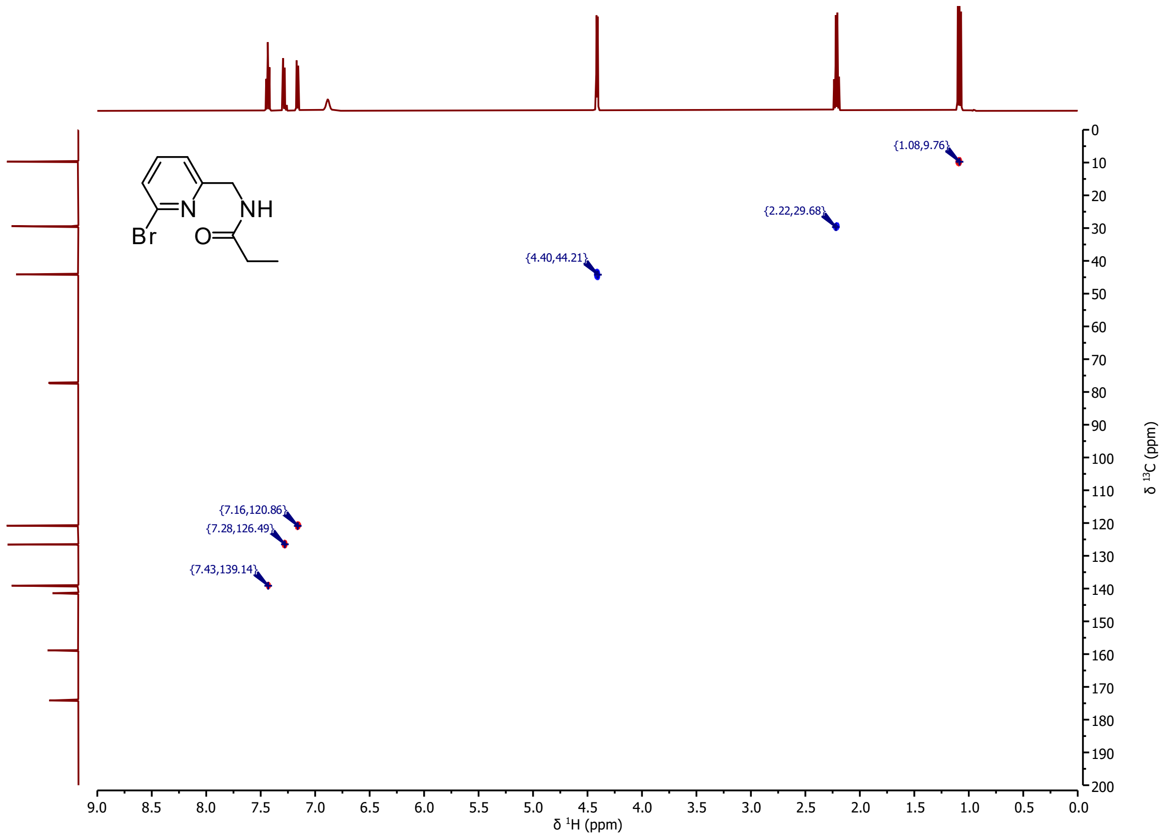


**Figure S141**: $^{1}$**H/**$^{13}$**C HSQC** (500/126 MHz, $CDCl_3$, 298 K) of **S27**.

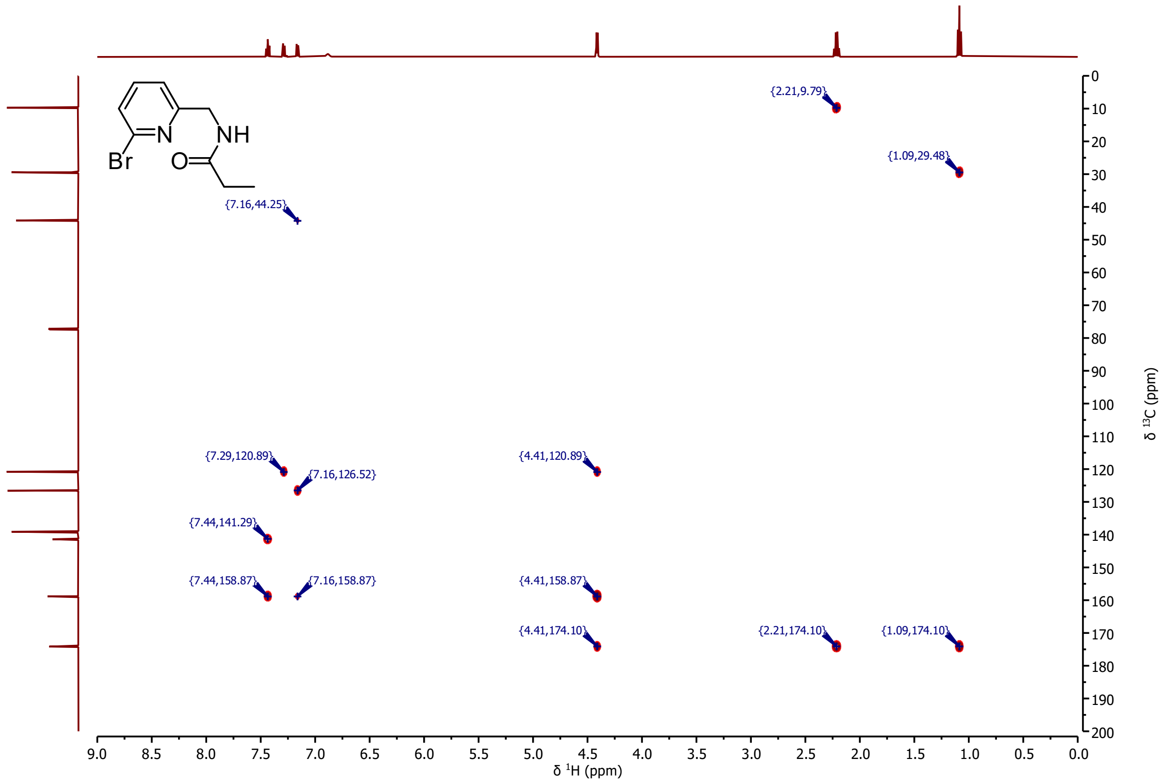


**Figure S142**: $^{1}$**H/**$^{13}$**C HMBC** (500/126 MHz, $CDCl_3$, 298 K) of **S27**.

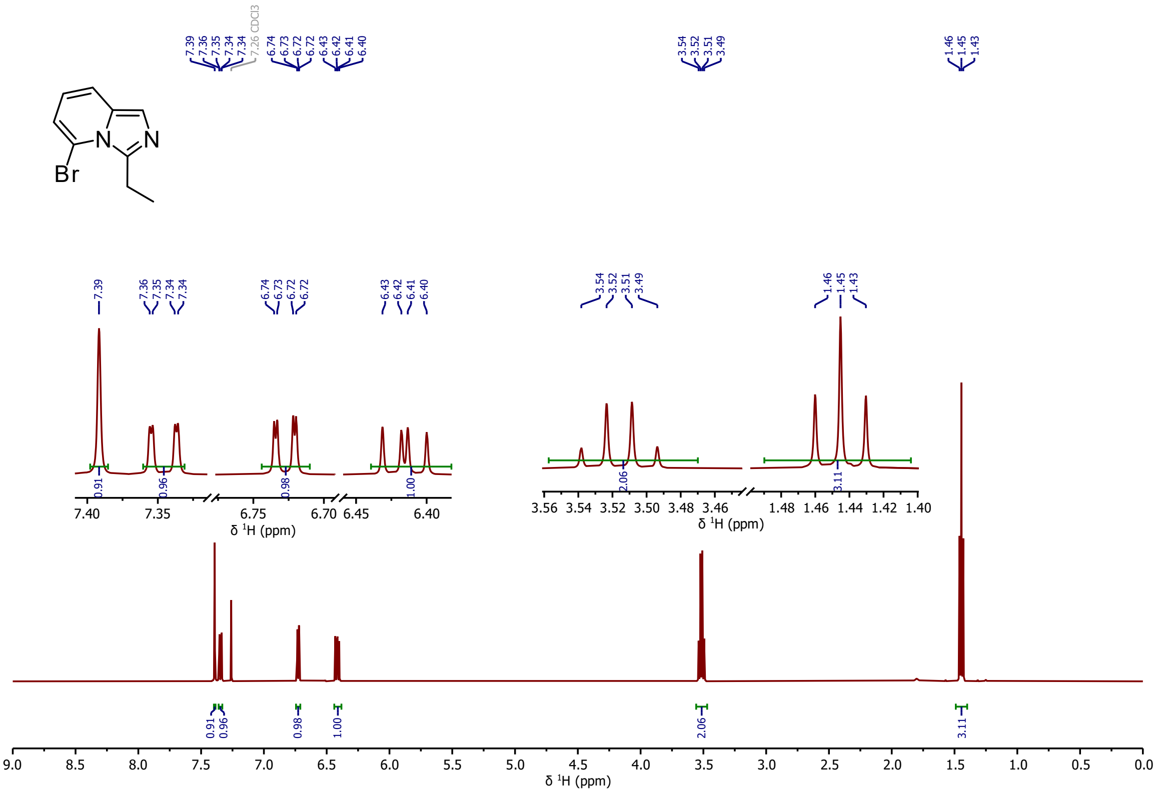

**Figure S143**: **$^{1}$H NMR** (500 MHz, $CDCl_3$, 298 K) of **S28**.

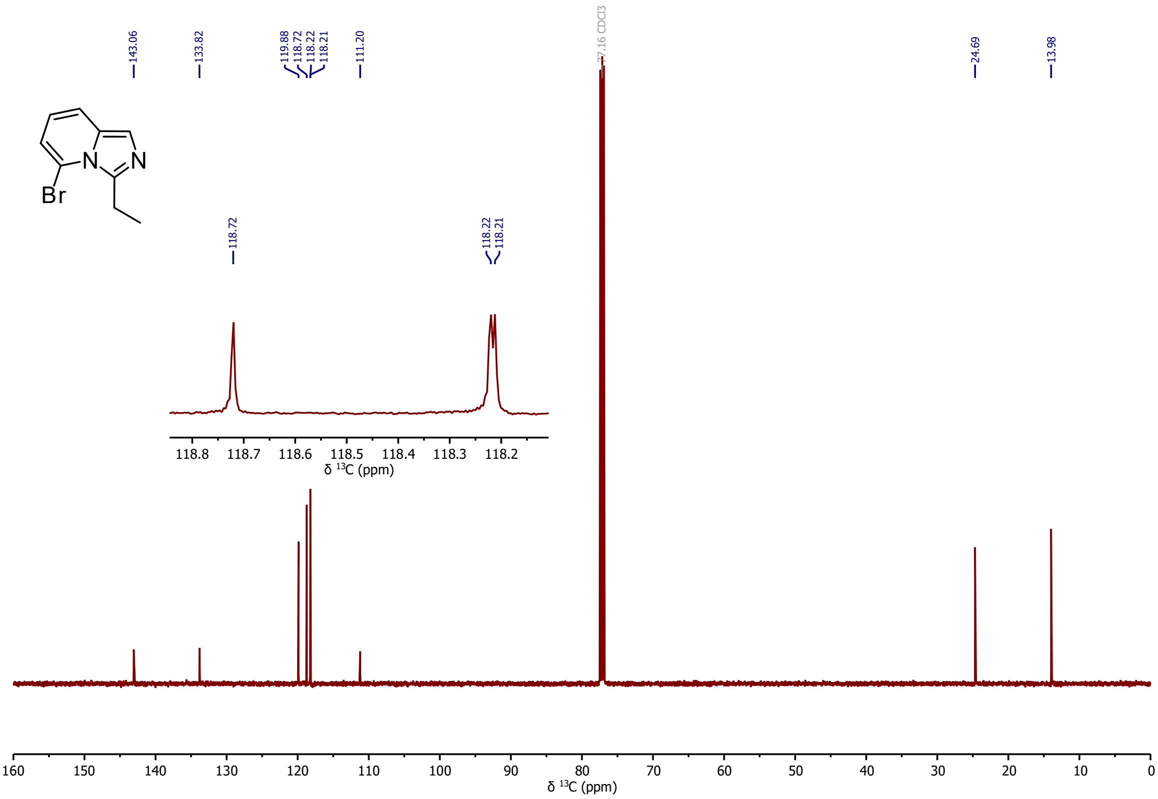

**Figure S144**: **$^{13}$C NMR** (126 MHz, $CDCl_3$, 298 K) of **S28**.

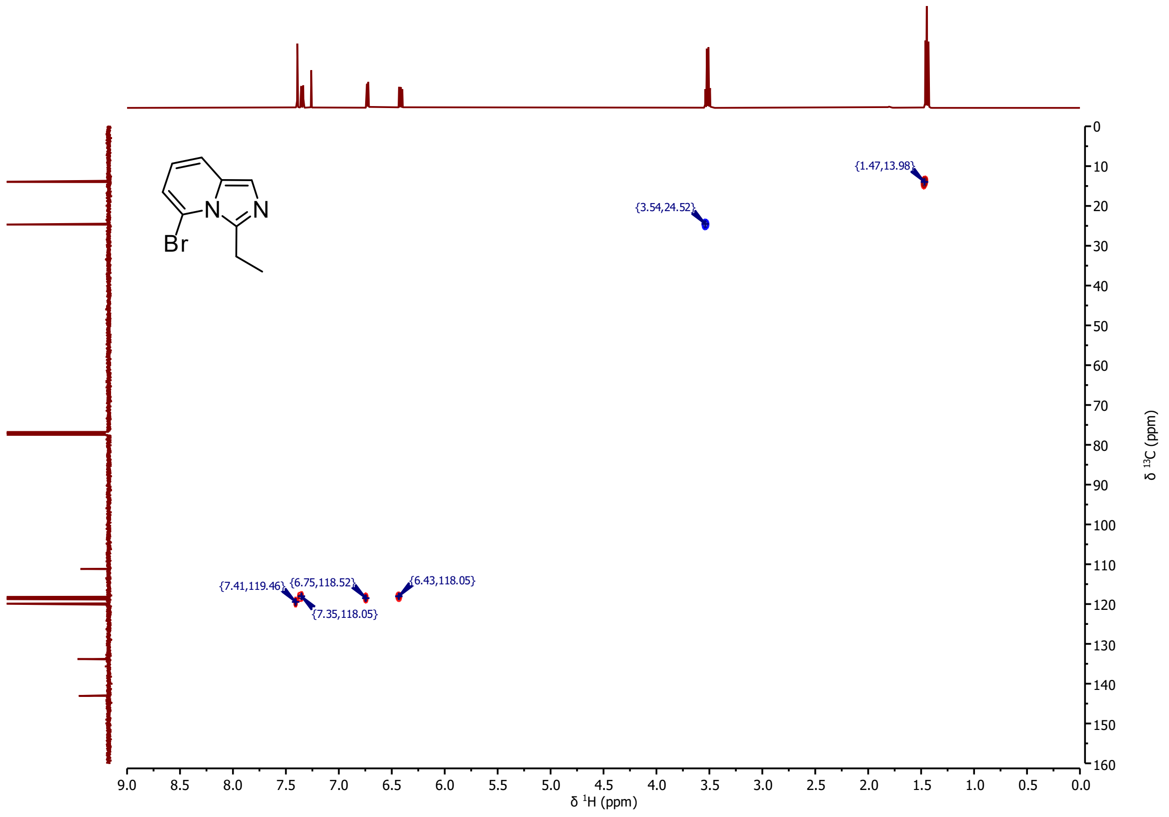


**Figure S145**: **$^1$H/$^{13}$C HSQC** (500/126 MHz, $CDCl_3$, 298 K) of **S28**.

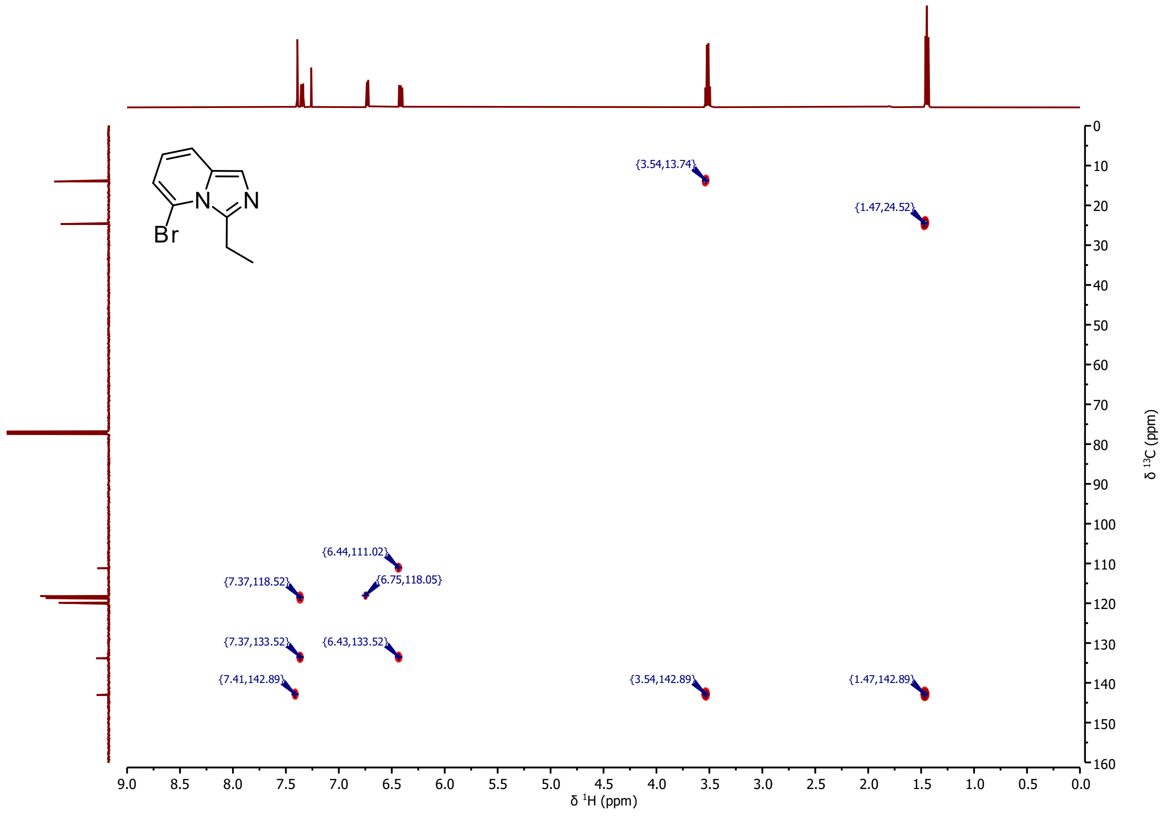


**Figure S146**: **$^1$H/$^{13}$C HMBC** (500/126 MHz, $CDCl_3$, 298 K) of **S28**.

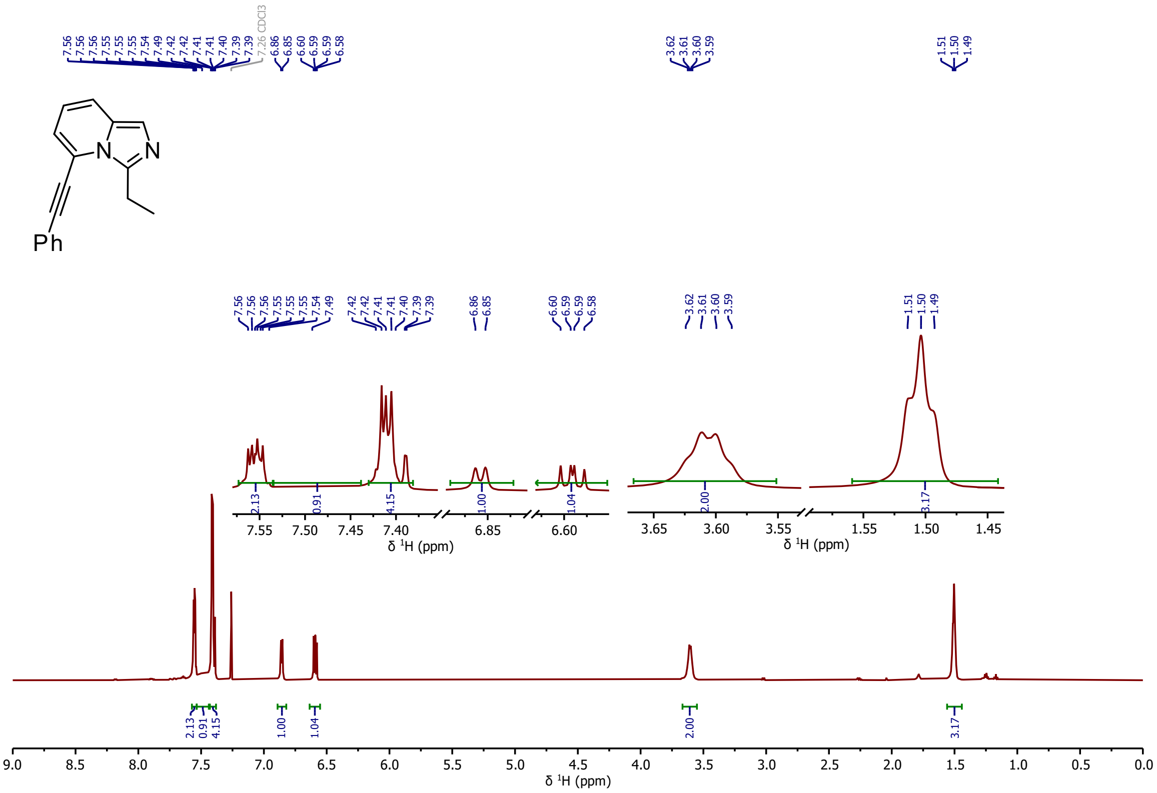

**Figure S147**: **$^1$H NMR** (600 MHz, $CDCl_3$, 298 K) of **S29**.

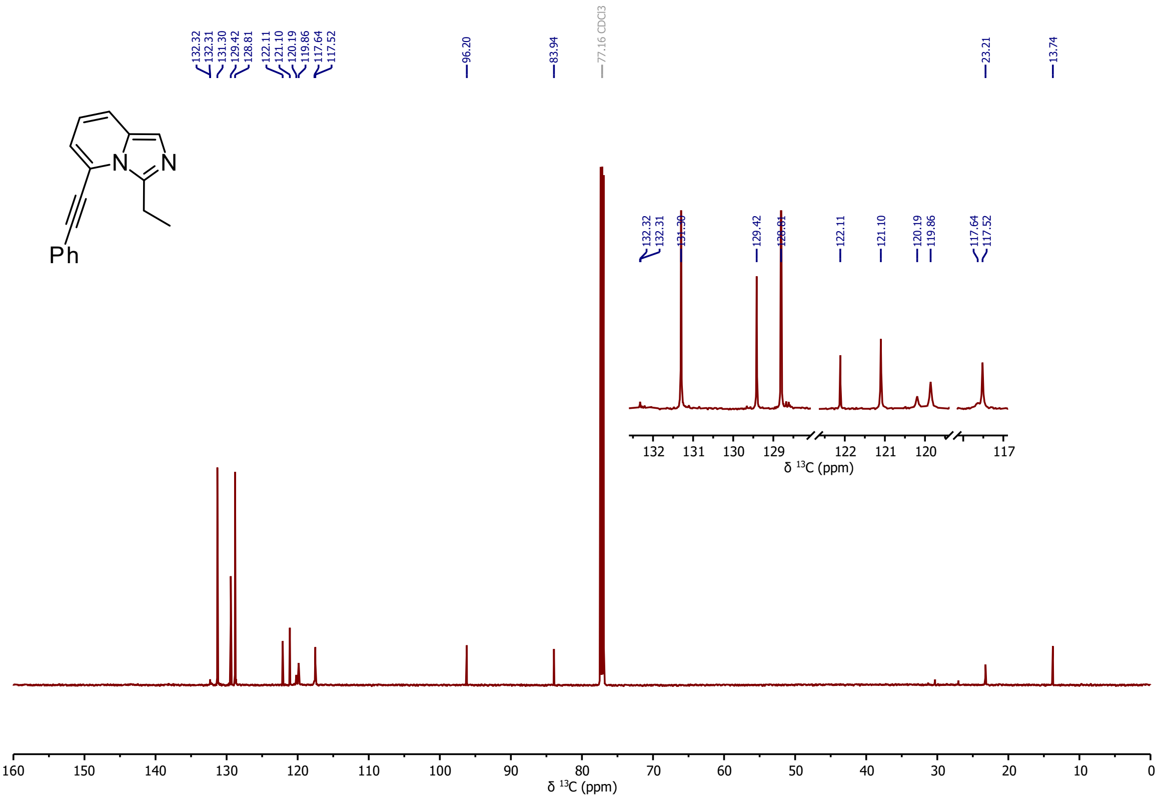

**Figure S148**: **$^{13}$C NMR** (151 MHz, $CDCl_3$, 298 K) of **S29**.

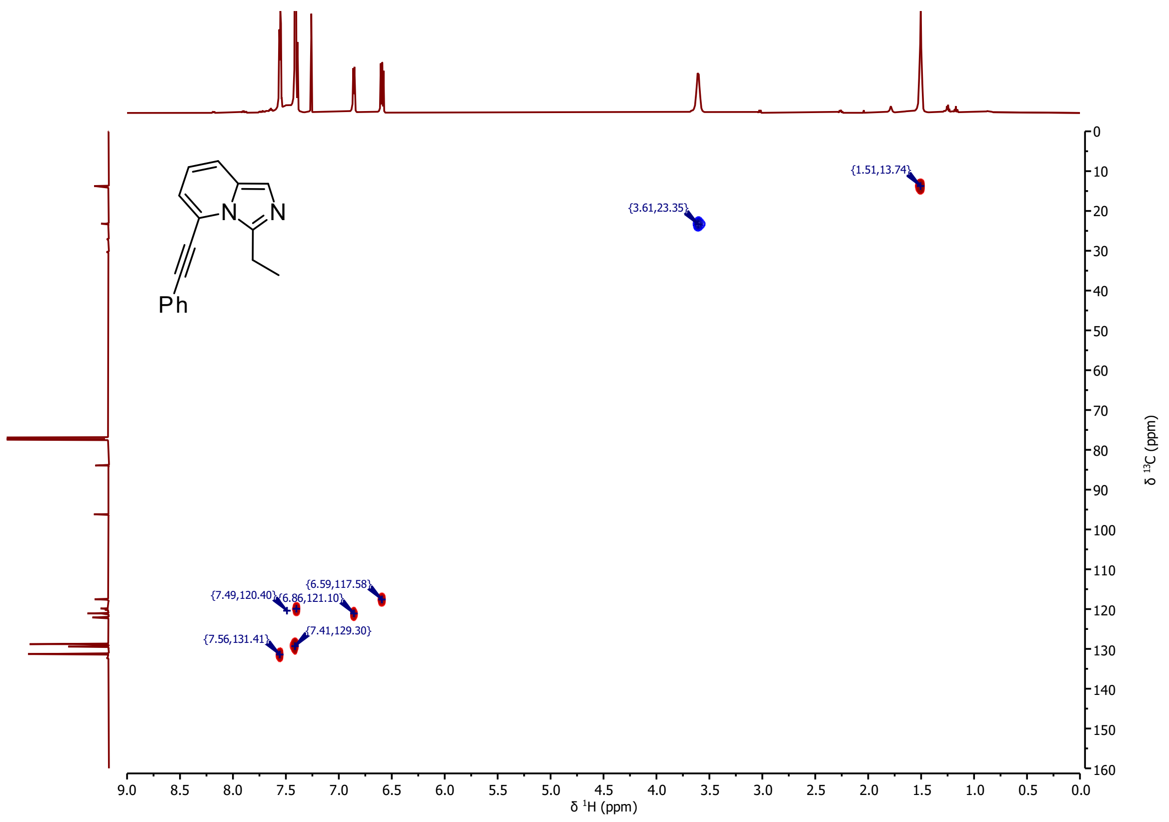


**Figure S149**: **$^1$H/$^{13}$C HSQC** (600/151 MHz, $CDCl_3$, 298 K) of **S29**.

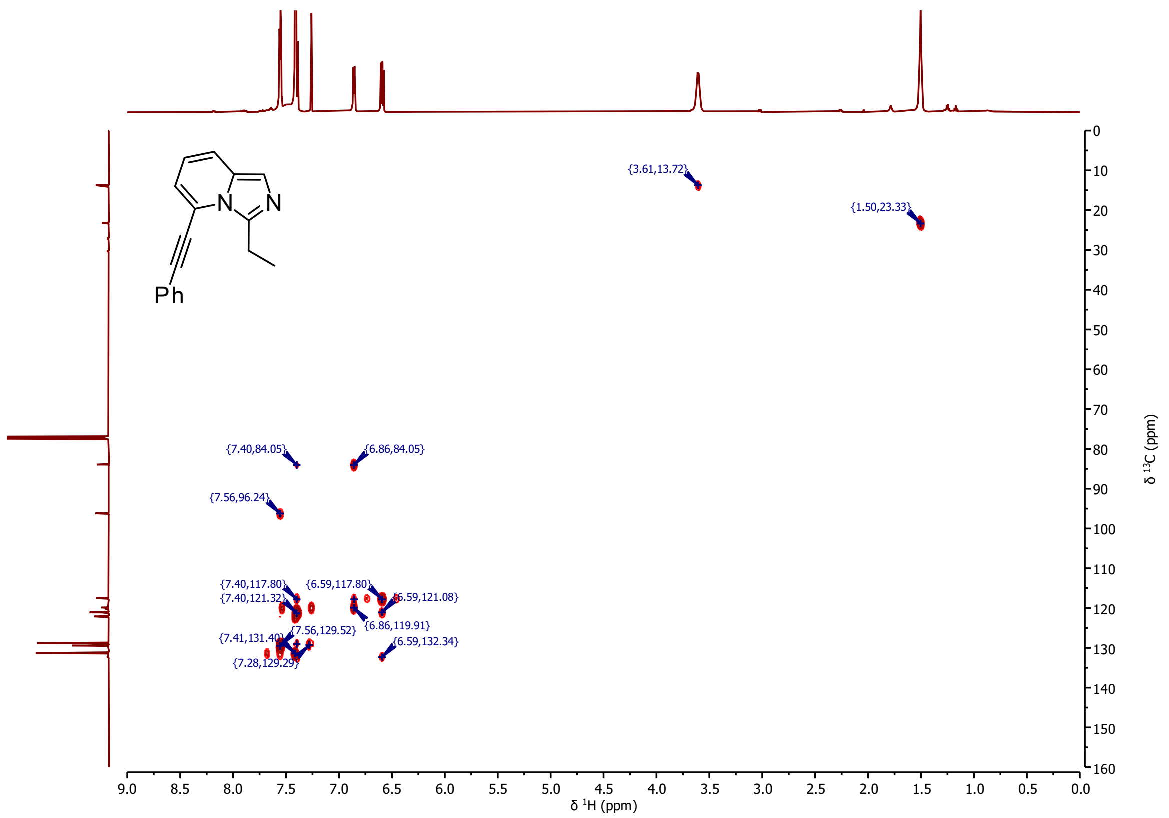


**Figure S150**: **$^1$H/$^{13}$C HMBC** (600/151 MHz, $CDCl_3$, 298 K) of **S29**.

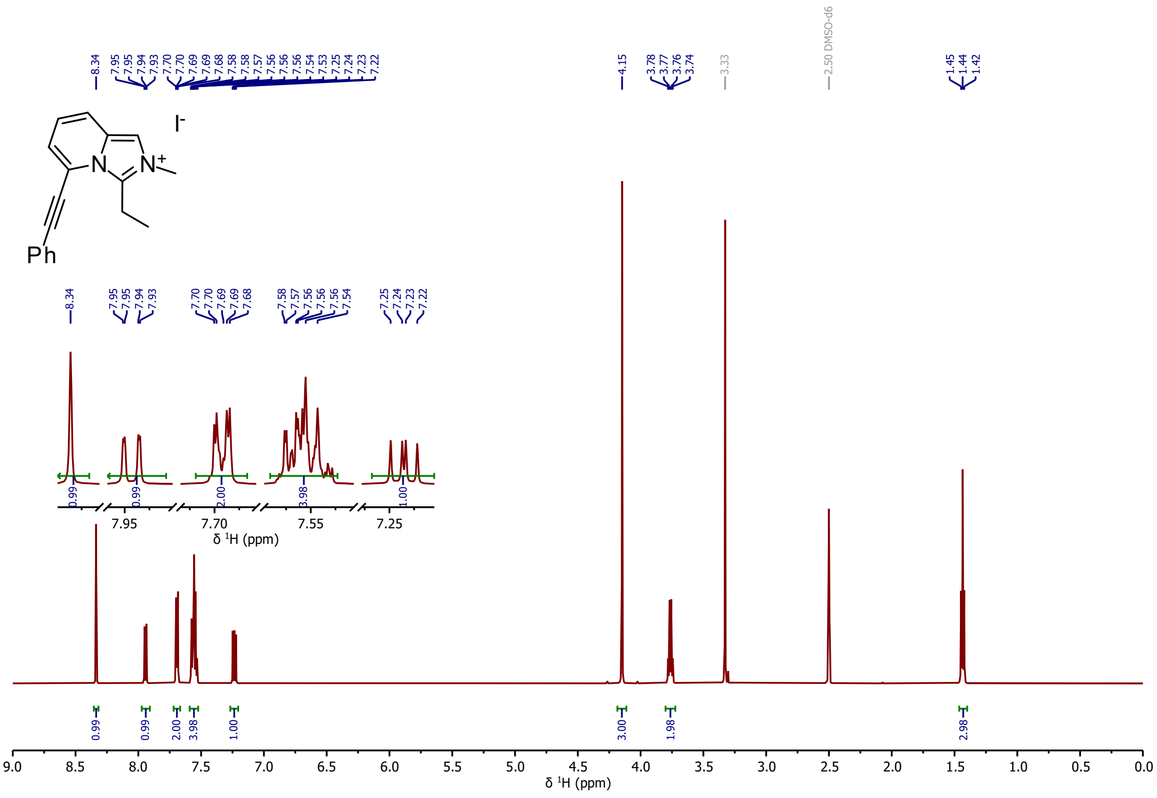

**Figure S151**: **$^1$H NMR** (600 MHz, $C_6D_6$, 298 K) of **4g**.

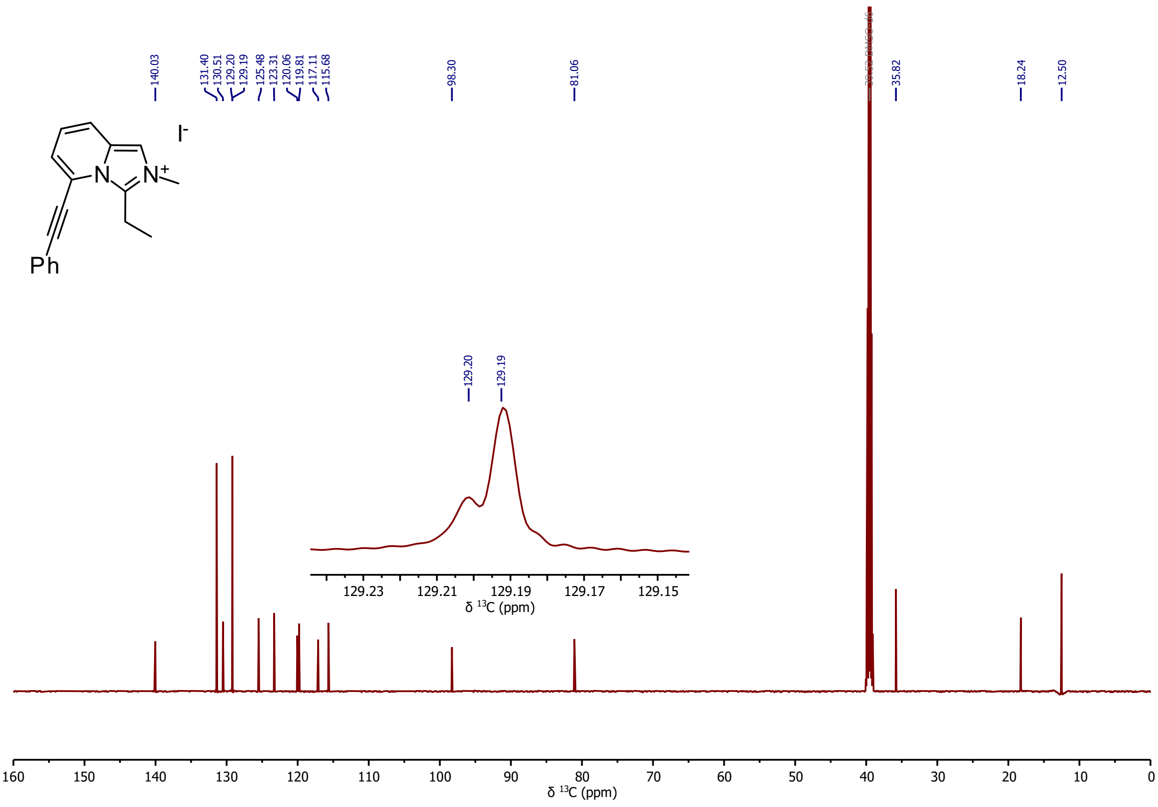

**Figure S152**: **$^{13}$C NMR** (151 MHz, $C_6D_6$, 298 K) of **4g**.

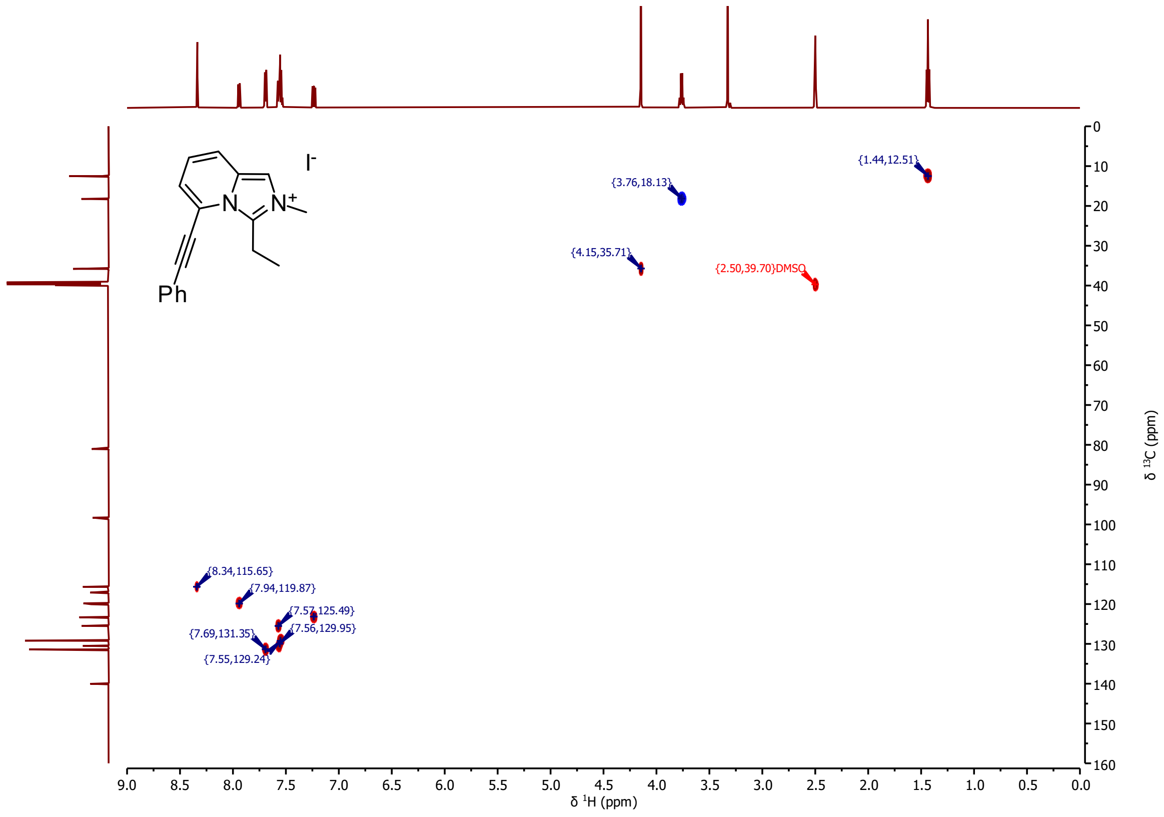


**Figure S153**: **$^1$H/$^{13}$C HSQC** (600/151 MHz, $C_6D_6$, 298 K) of **4g**.

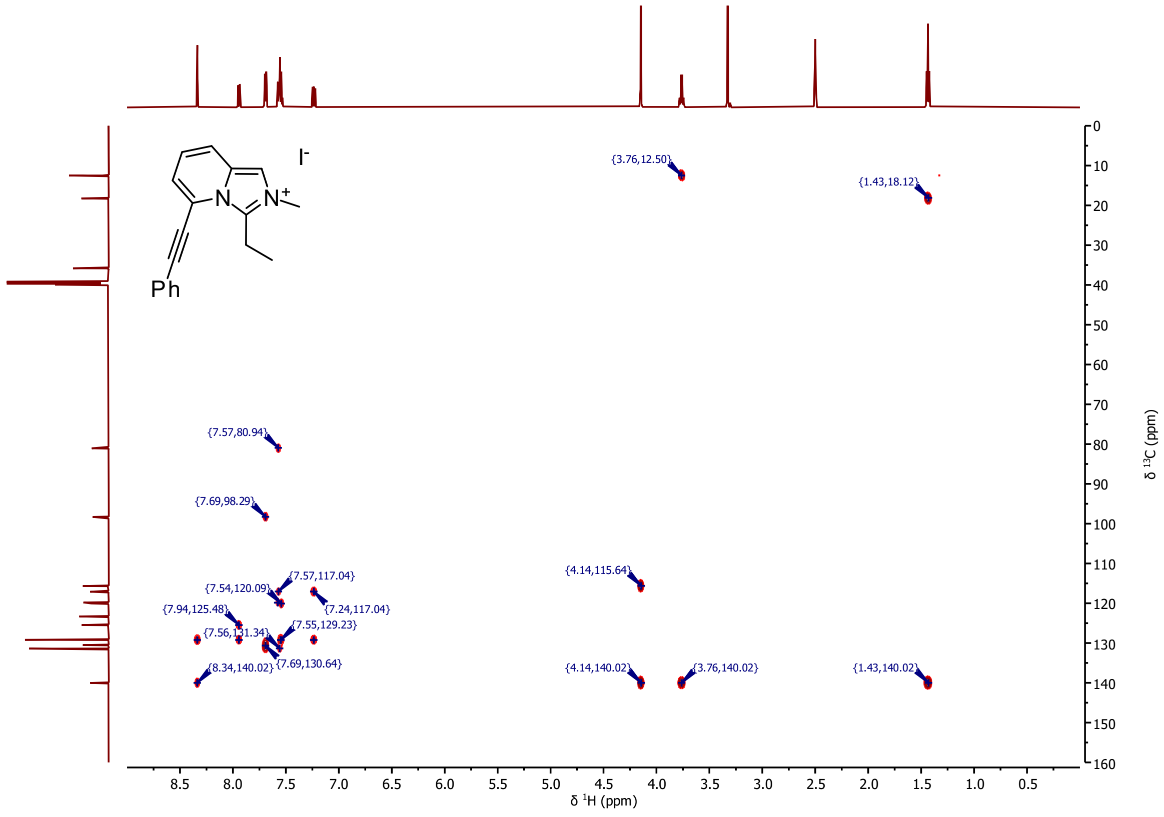


**Figure S154**: **$^1$H/$^{13}$C HMBC** (600/151 MHz, $C_6D_6$, 298 K) of **4g**.

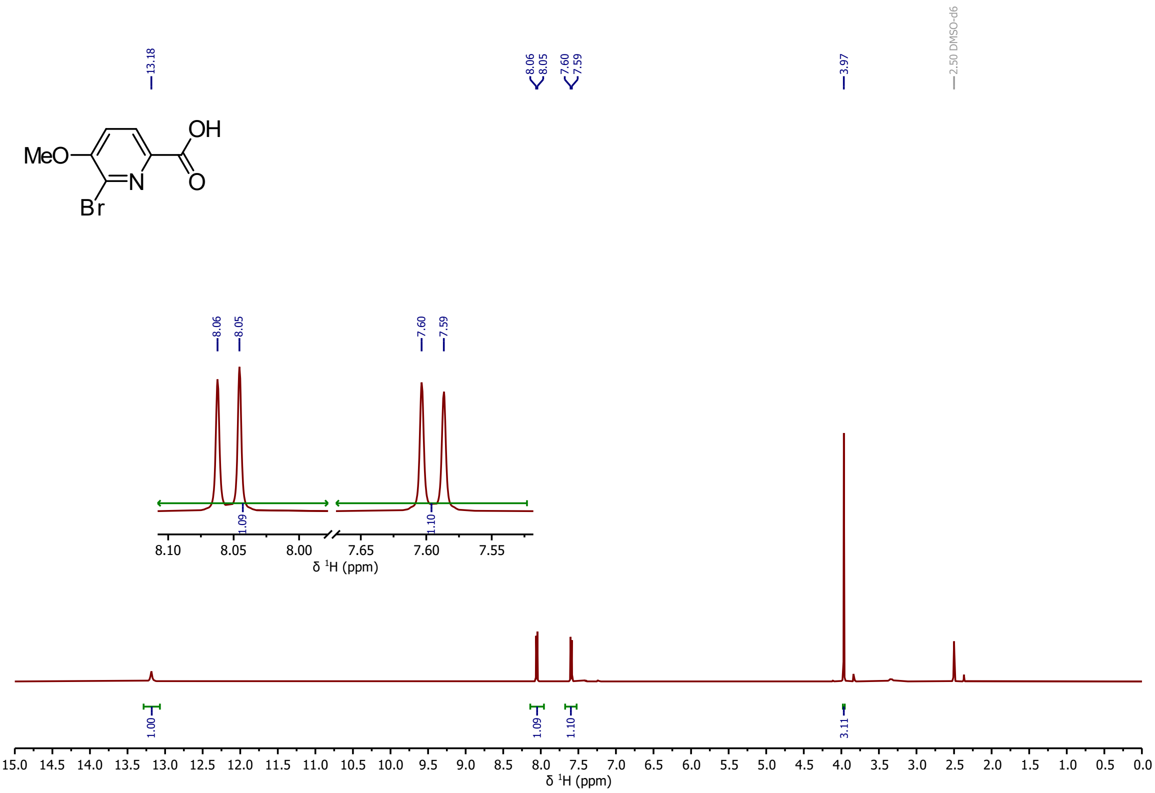


**Figure S155**: **$^1$H NMR** (500 MHz, DMSO-$d_6$, 298 K) of **S31**.

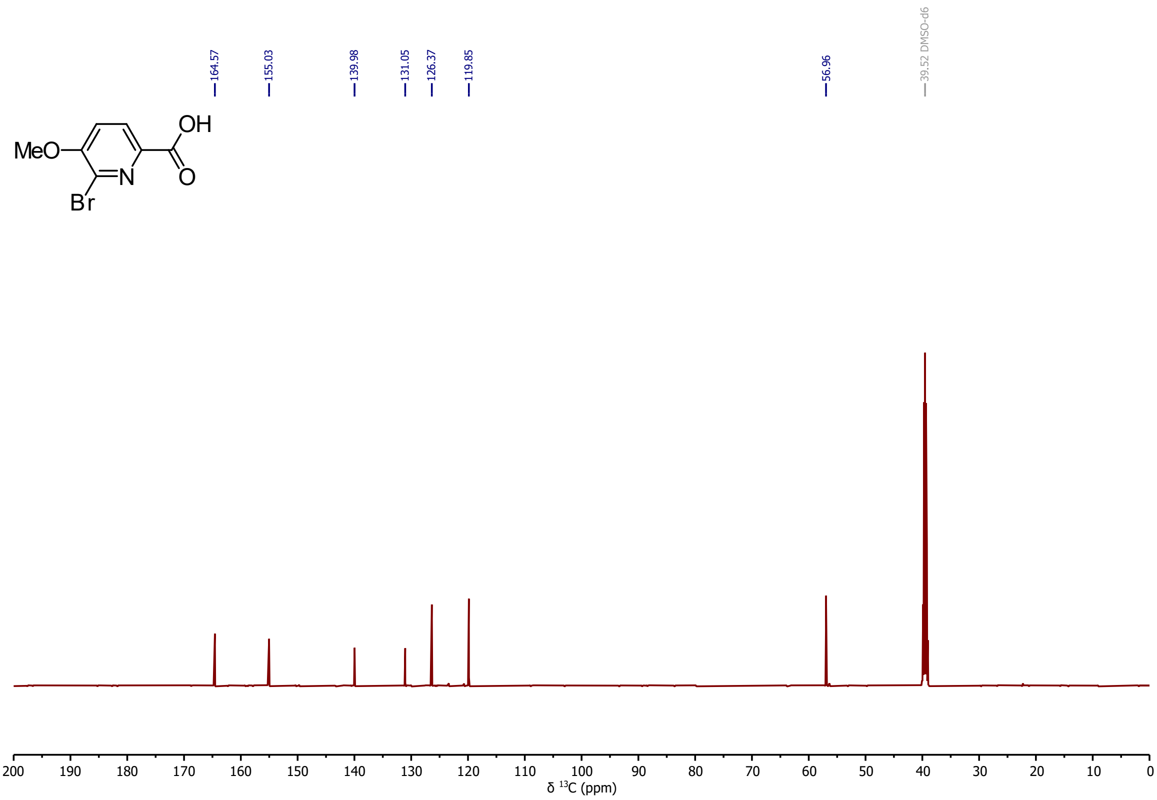


**Figure S156**: **$^{13}$C NMR** (126 MHz, DMSO-$d_6$, 298 K) of **S31**.

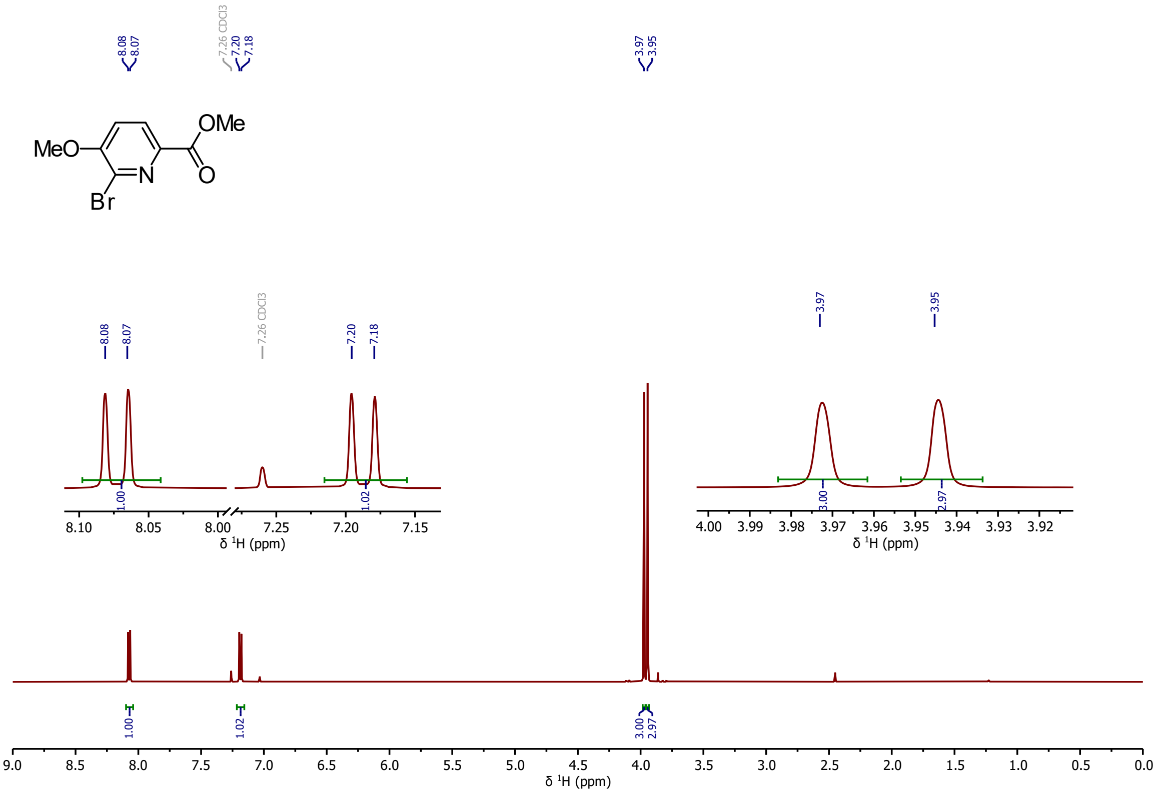


**Figure S157**: **¹H NMR** (500 MHz, $CDCl_3$, 298 K) of **S32**.

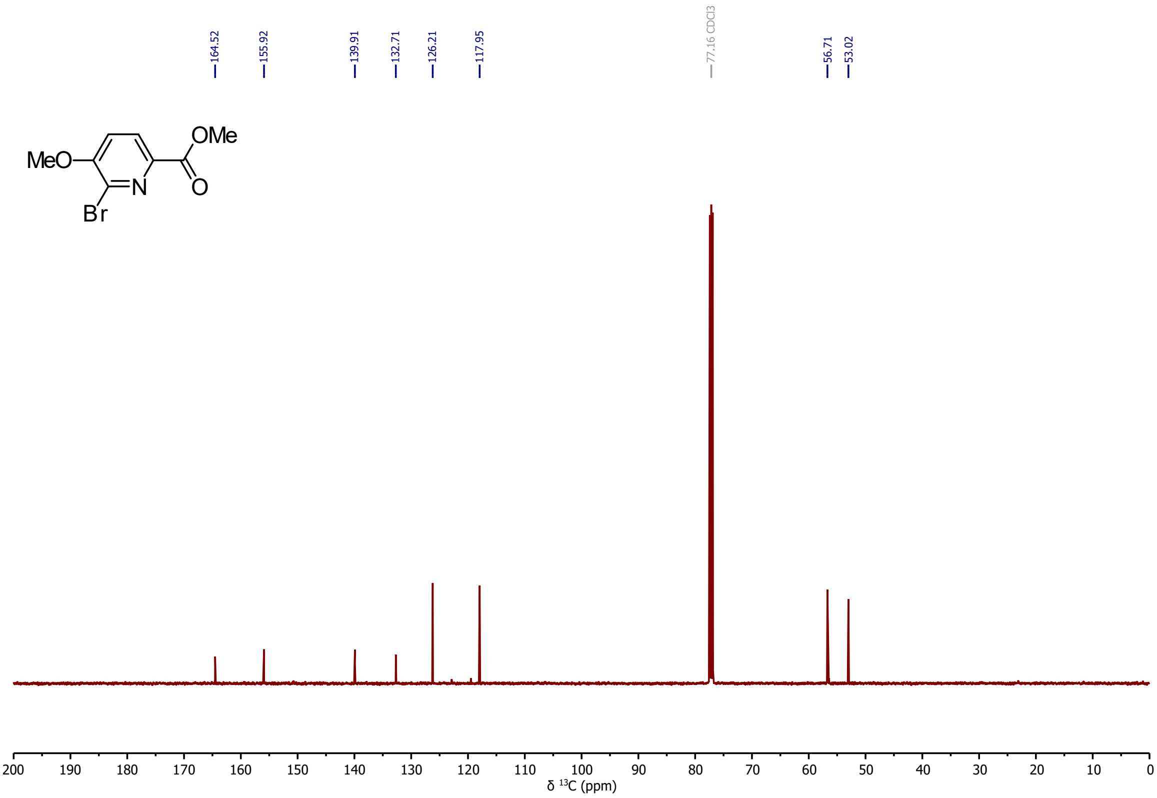


**Figure S158**: **¹³C NMR** (126 MHz, $CDCl_3$, 298 K) of **S32**.

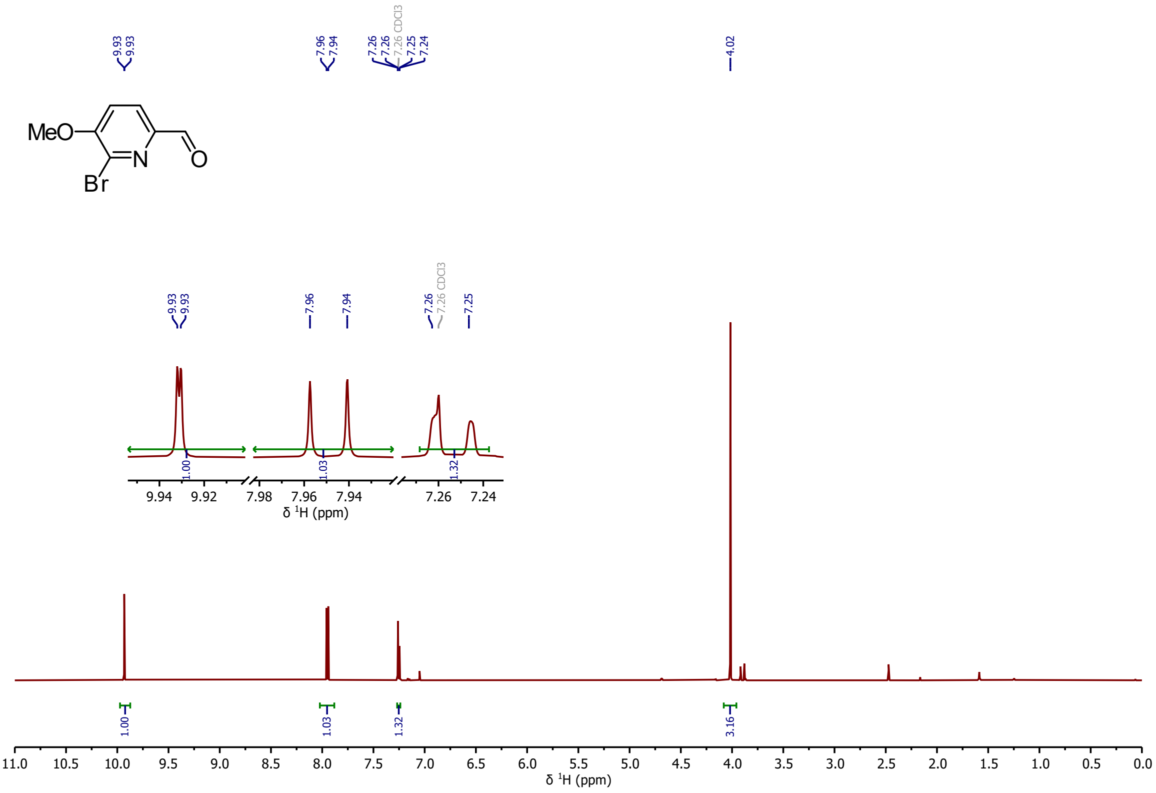


**Figure S159**: **$^{1}$H NMR** (500 MHz, $CDCl_3$, 298 K) of **S33**.

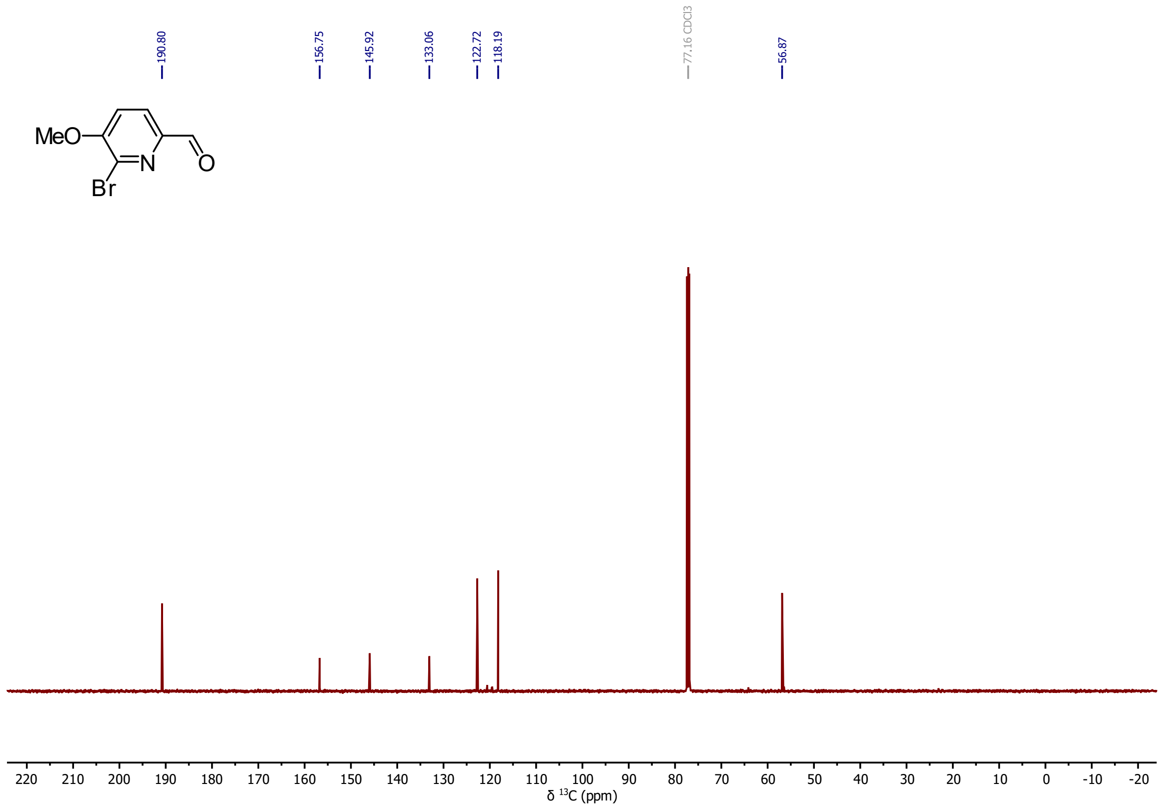


**Figure S160**: **$^{13}$C NMR** (126 MHz, $CDCl_3$, 298 K) of **S33**.

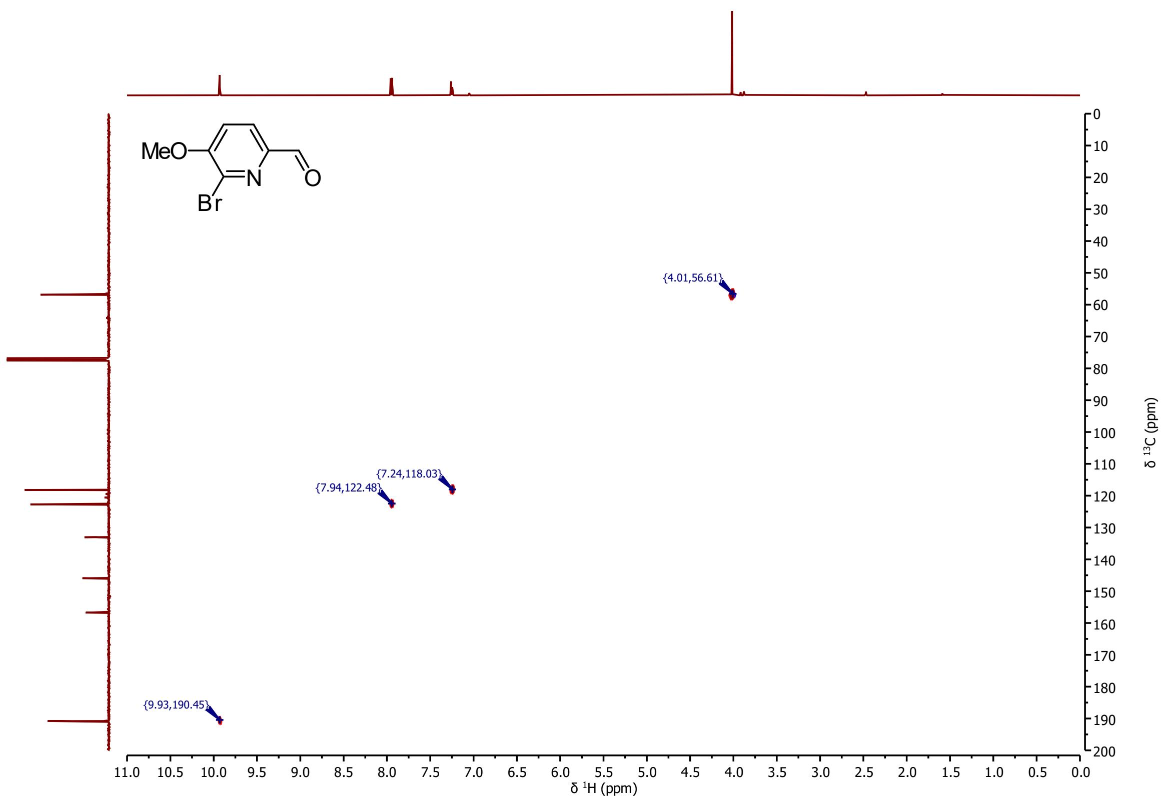


**Figure S161**: **$^{1}$H/$^{13}$C HSQC** (500/126 MHz, $CDCl_3$, 298 K) of **S33**.

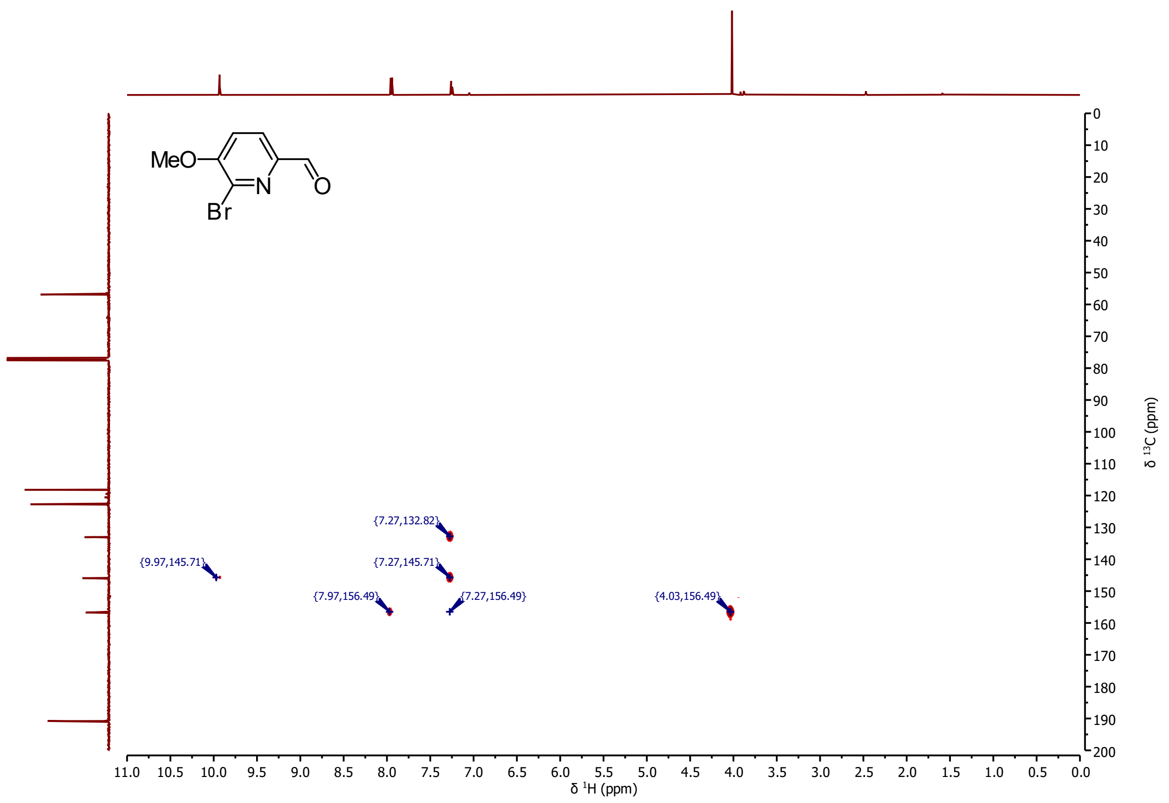


**Figure S162**: **$^{1}$H/$^{13}$C HMBC** (500/126 MHz, $CDCl_3$, 298 K) of **S33**.

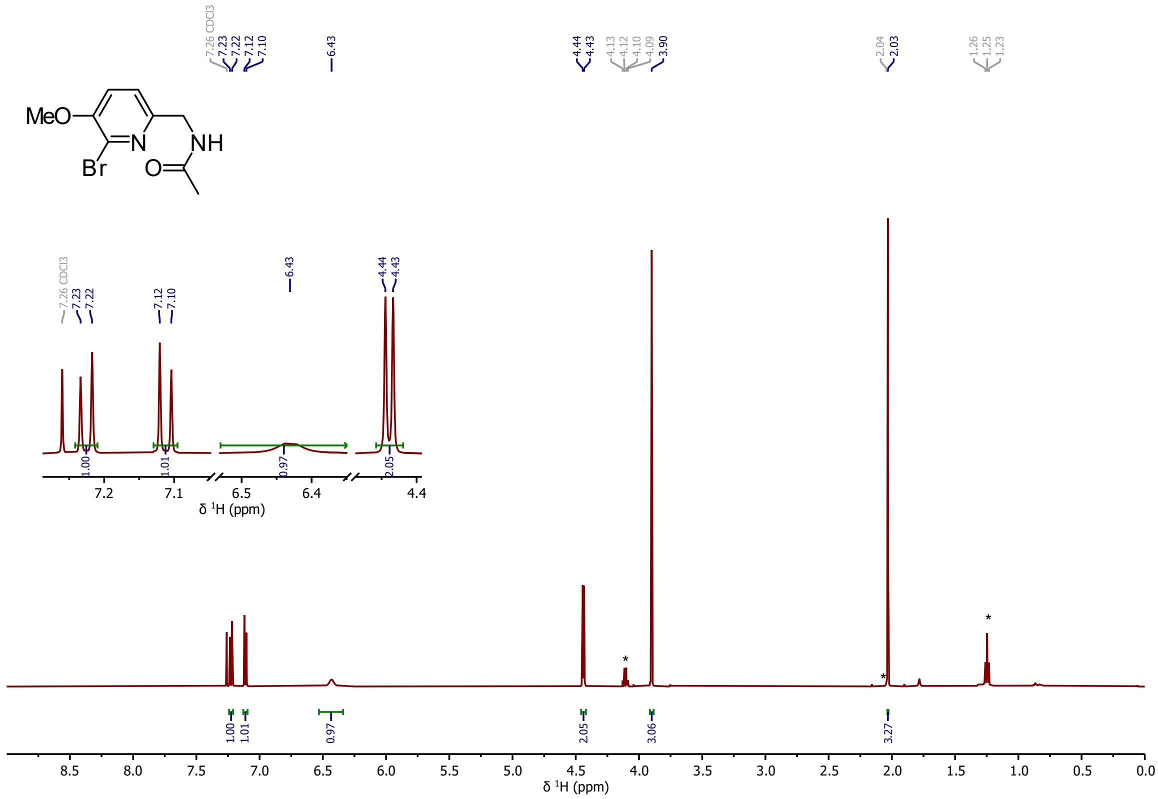

**Figure S163**: **$^1$H NMR** (500 MHz, $CDCl_3$, 298 K) of **S34**. * = EtOAc.

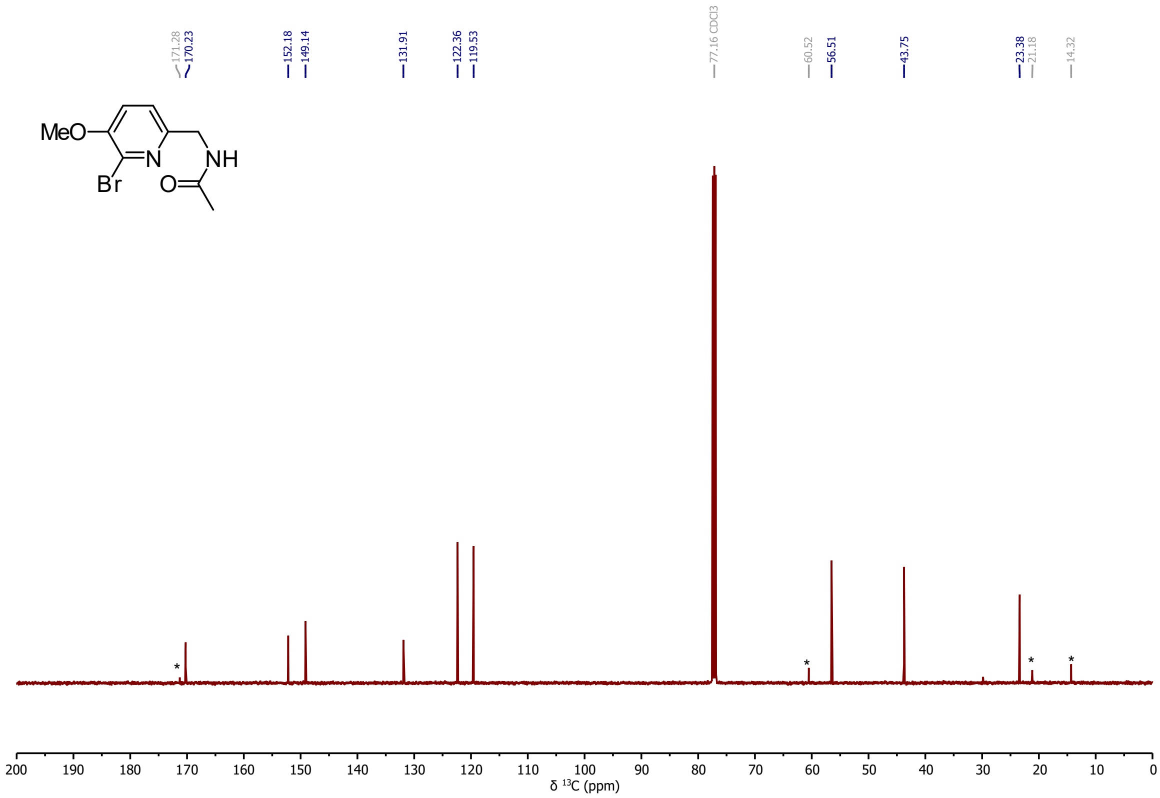

**Figure S164**: **$^{13}$C NMR** (126 MHz, $CDCl_3$, 298 K) of **S34**. * = EtOAc.

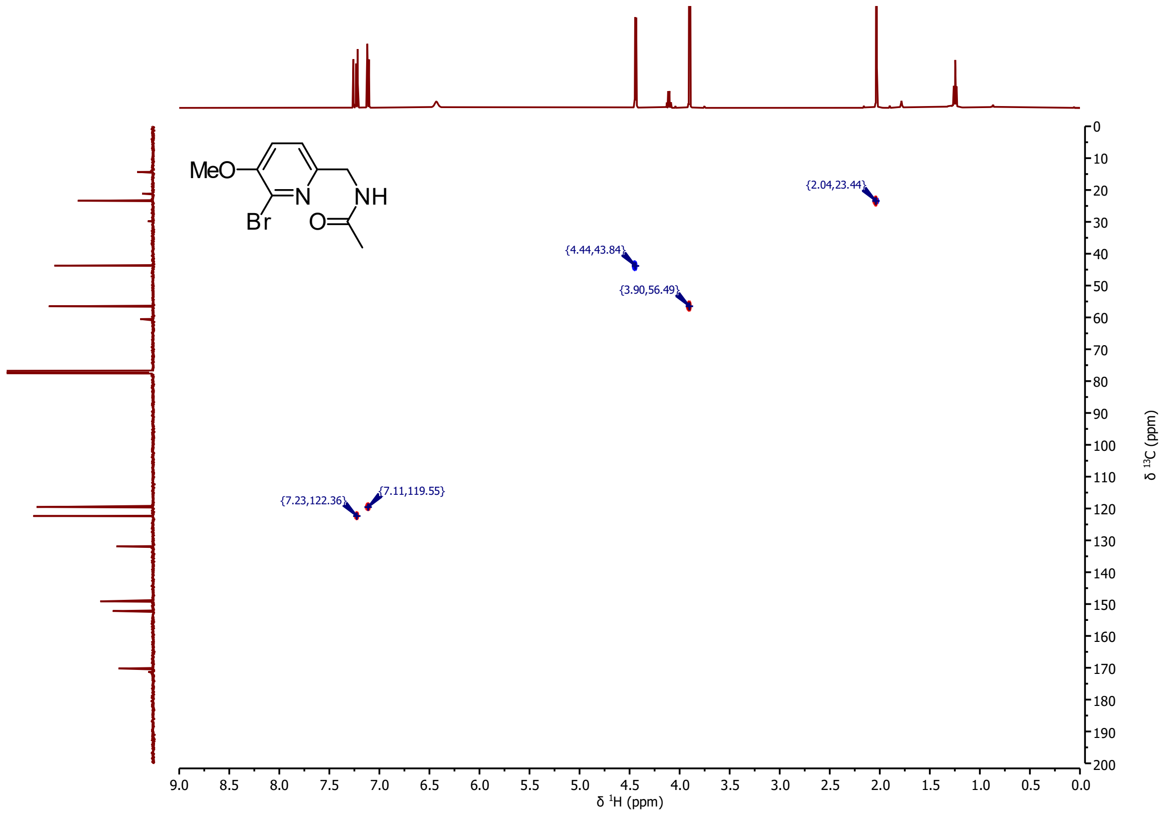


**Figure S165**: **$^{1}$H/$^{13}$C HSQC** (500/126 MHz, $CDCl_3$, 298 K) of **S34**.

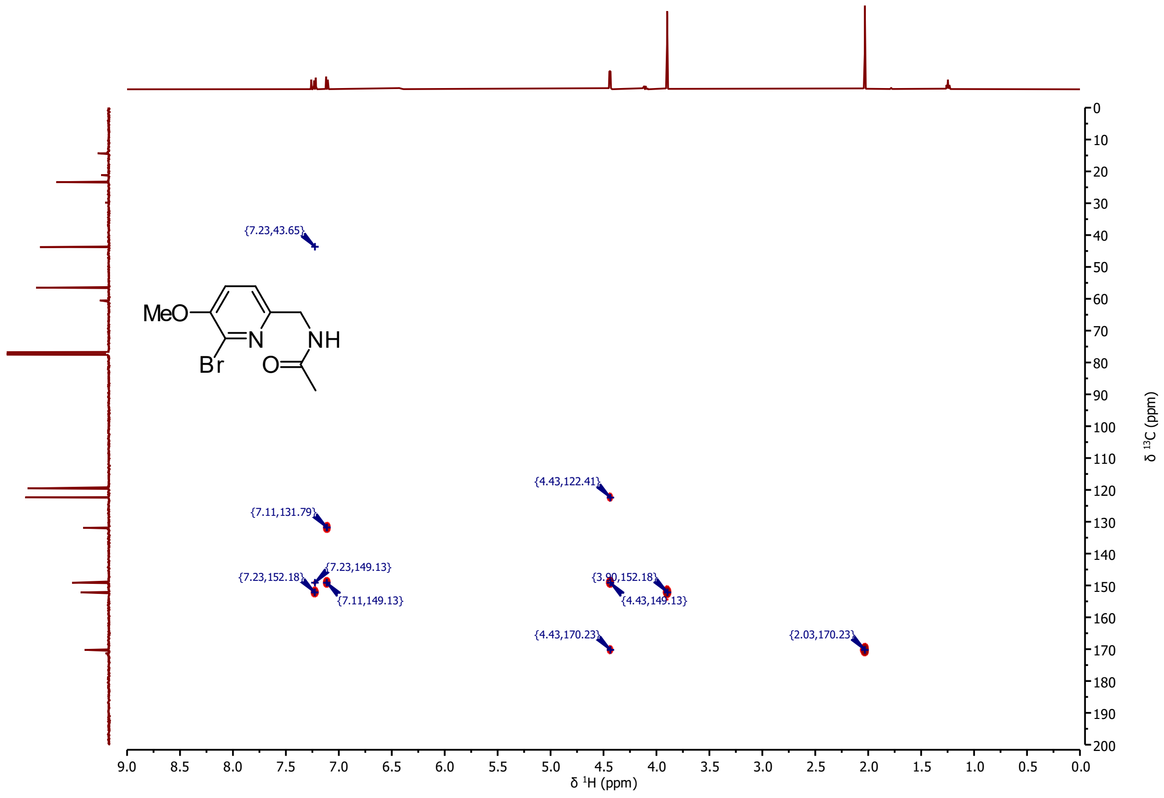


**Figure S166**: **$^{1}$H/$^{13}$C HMBC** (500/126 MHz, $CDCl_3$, 298 K) of **S34**.

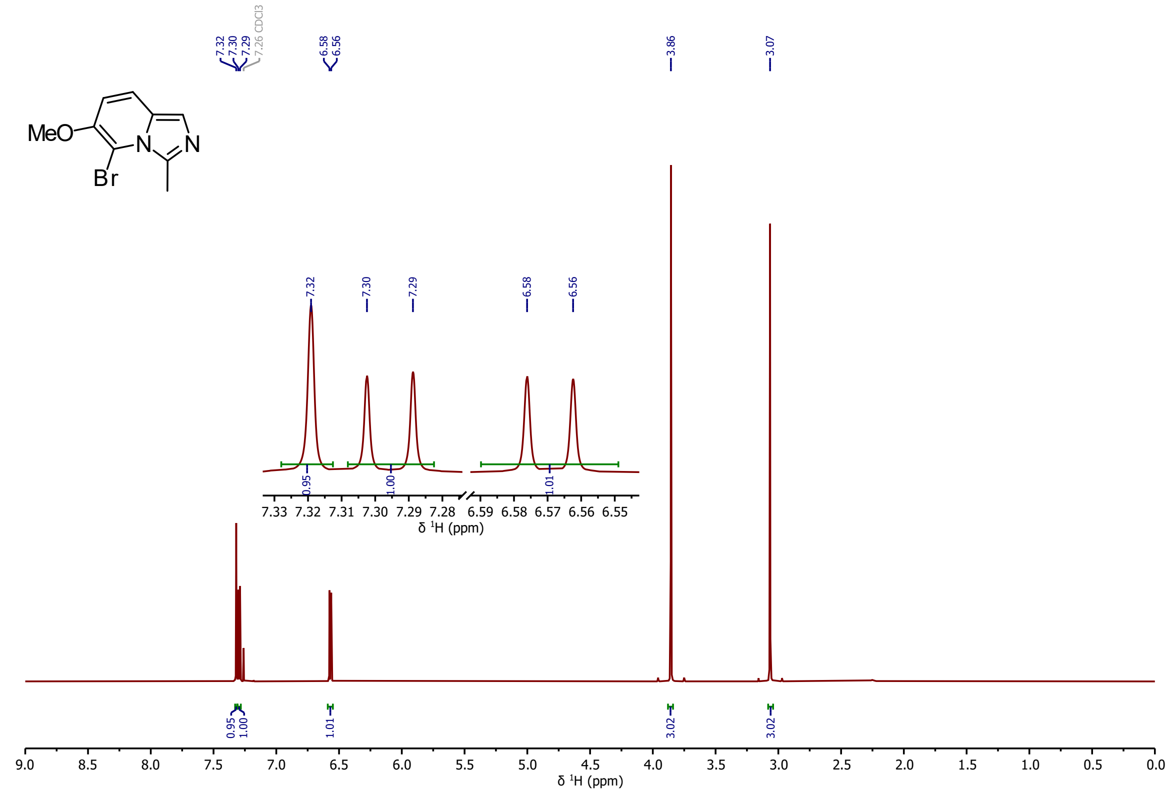


**Figure S167**: **$^{1}$H NMR** (700 MHz, $CDCl_3$, 298 K) of **S35**.

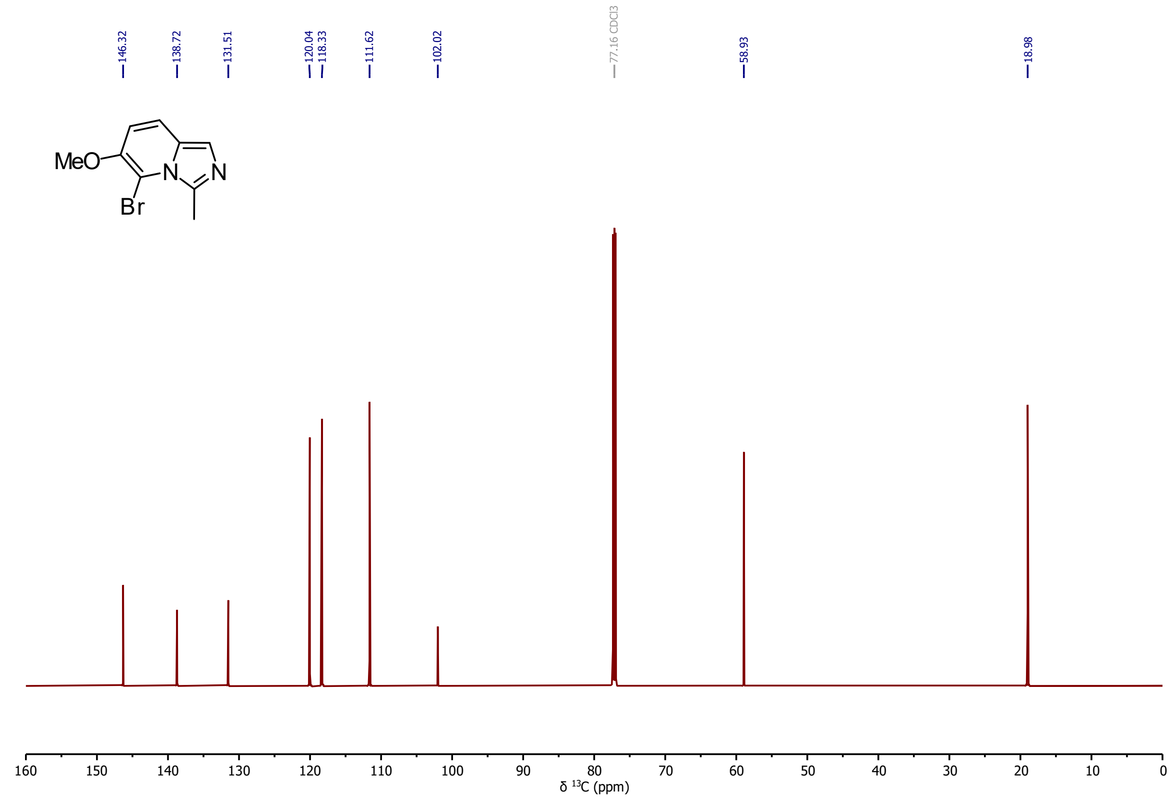


**Figure S168**: **$^{13}$C NMR** (176 MHz, $CDCl_3$, 298 K) of **S35**.

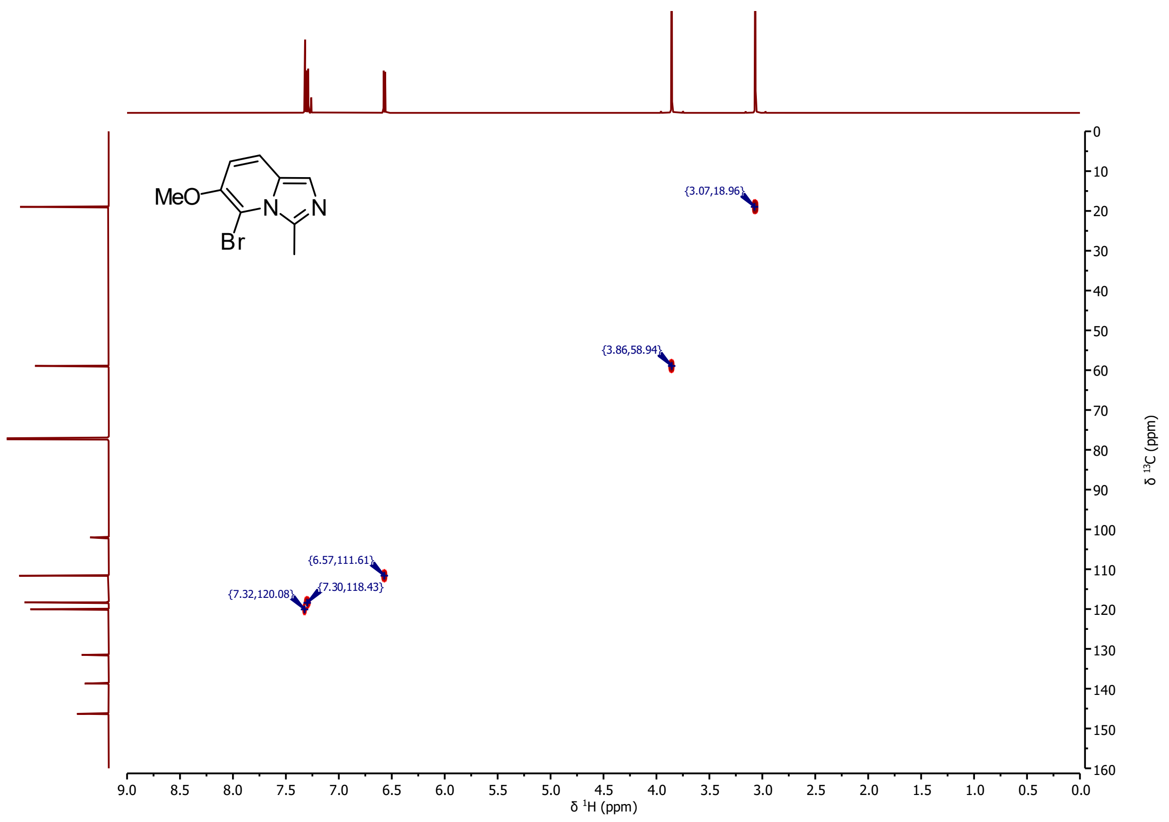


**Figure S169**: **$^{1}$H/$^{13}$C HSQC** (700/176 MHz, $CDCl_3$, 298 K) of **S35**.

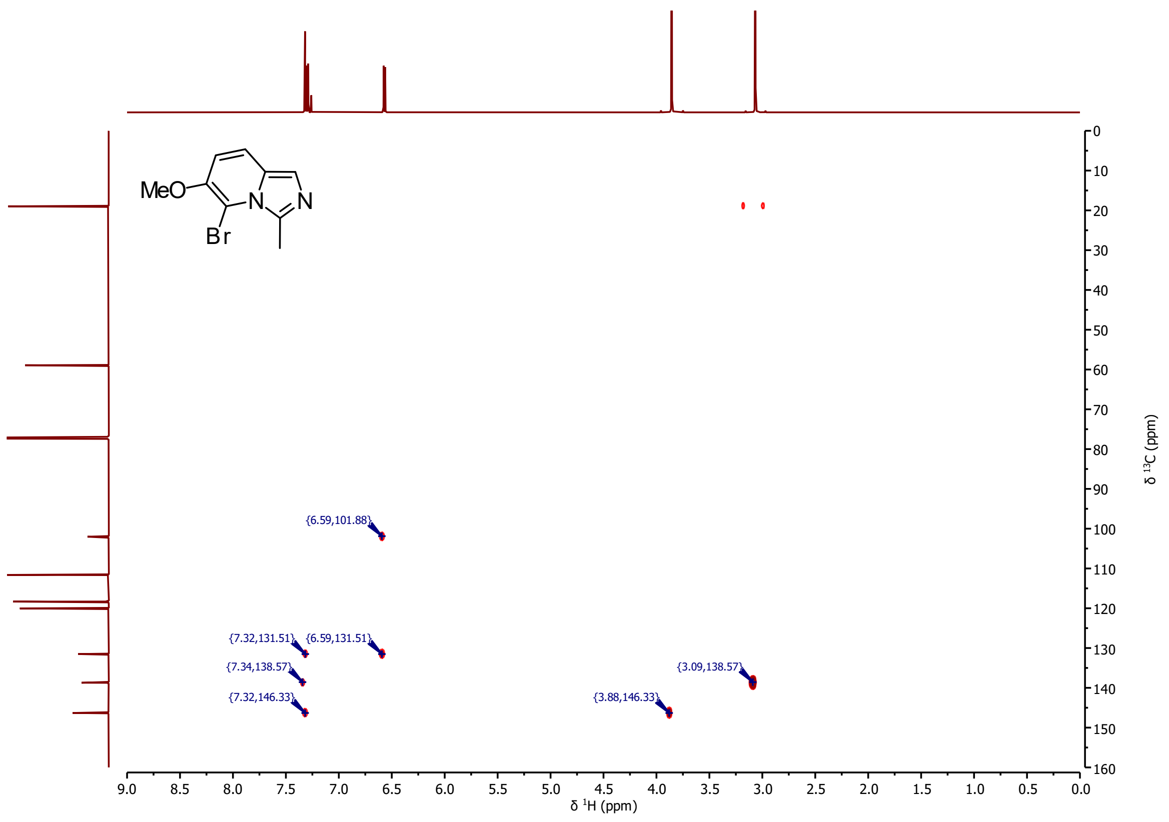


**Figure S170**: **$^{1}$H/$^{13}$C HMBC** (700/176 MHz, $CDCl_3$, 298 K) of **S35**.

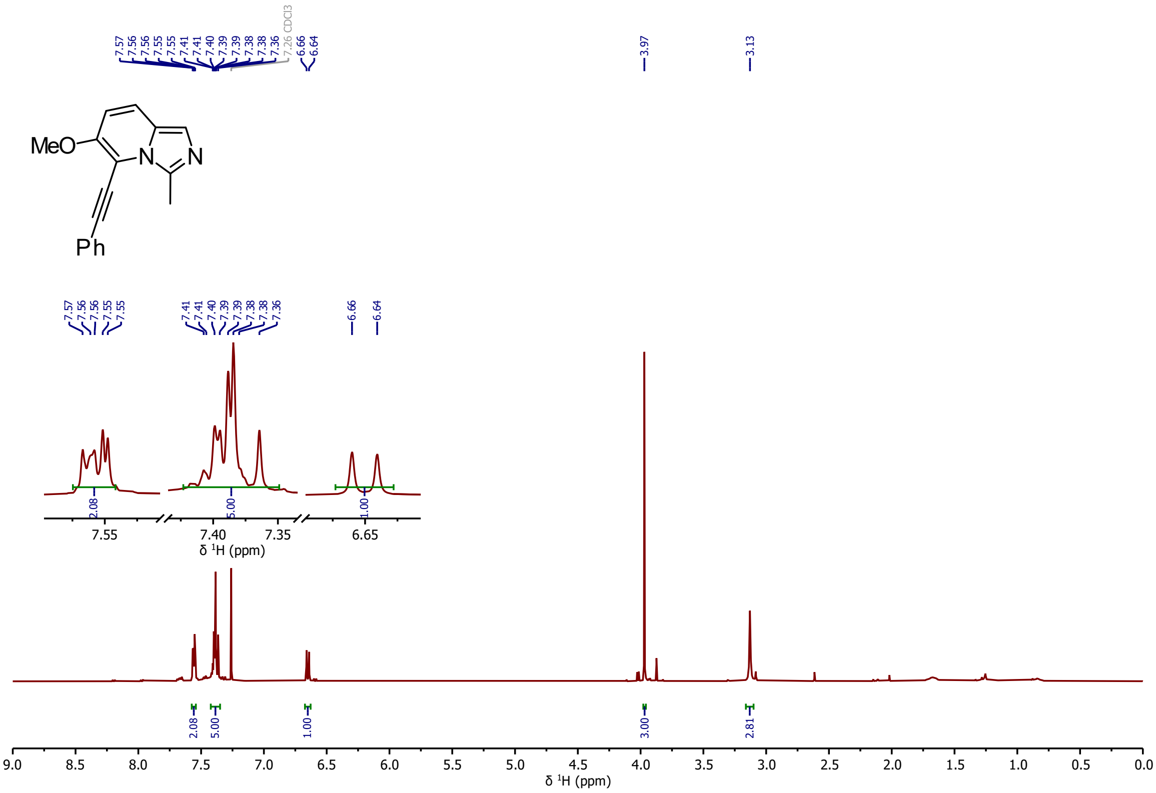

**Figure S171**: **$^{1}$H NMR** (500 MHz, $CDCl_3$, 298 K) of **S36**.

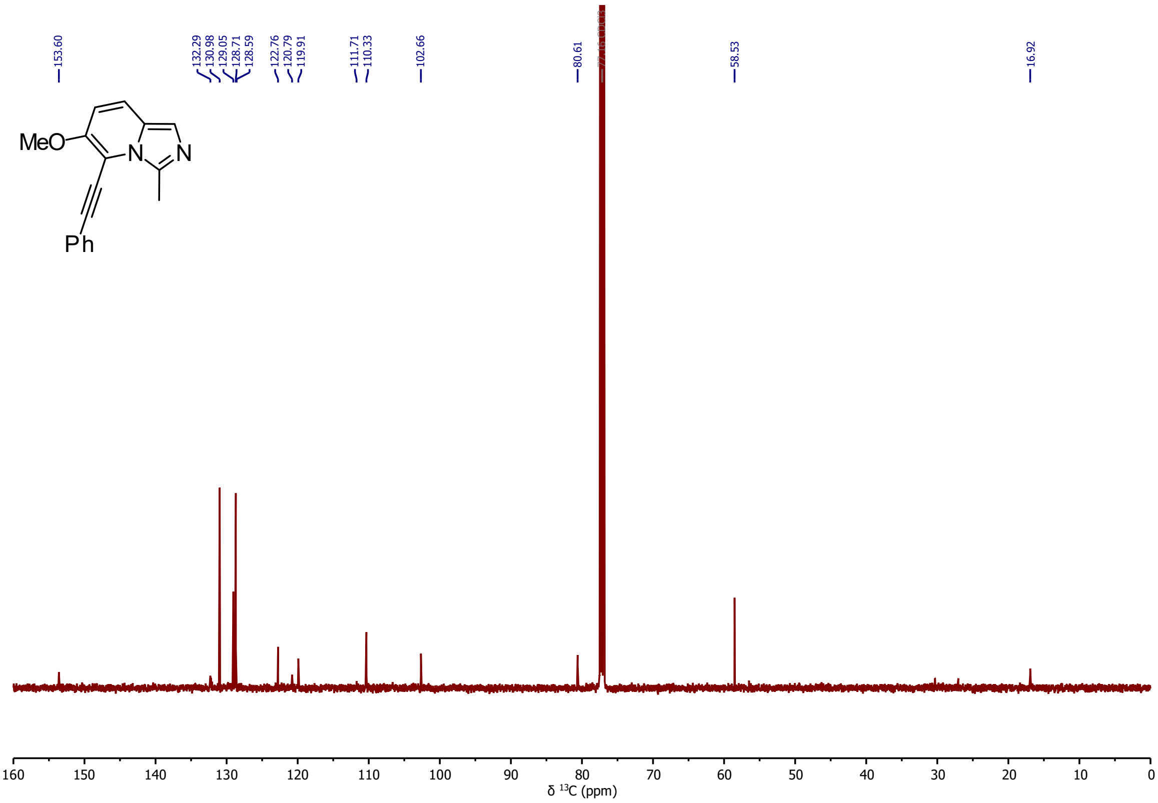

**Figure S172**: **$^{13}$C NMR** (126 MHz, $CDCl_3$, 298 K) of **S36**.

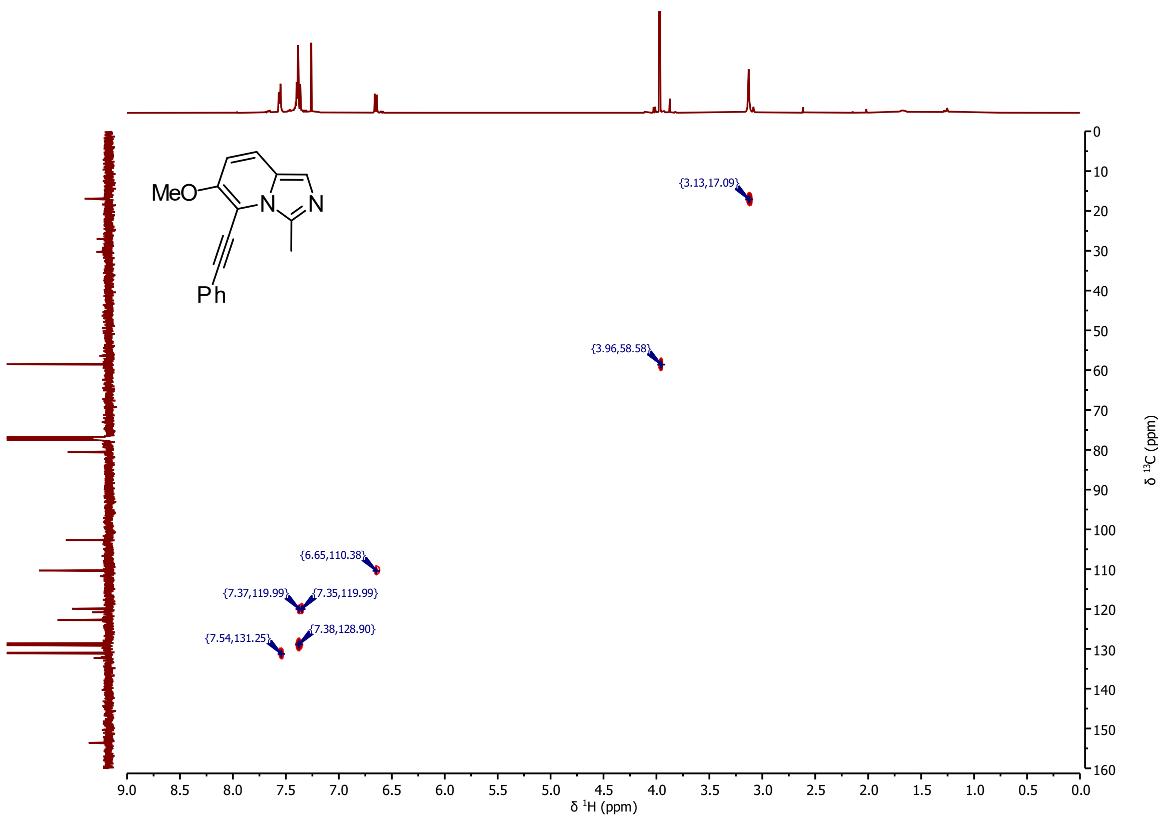

**Figure S173**: **$^{1}H/^{13}C$ HSQC** (500/126 MHz, $CDCl_3$, 298 K) of **S36**.

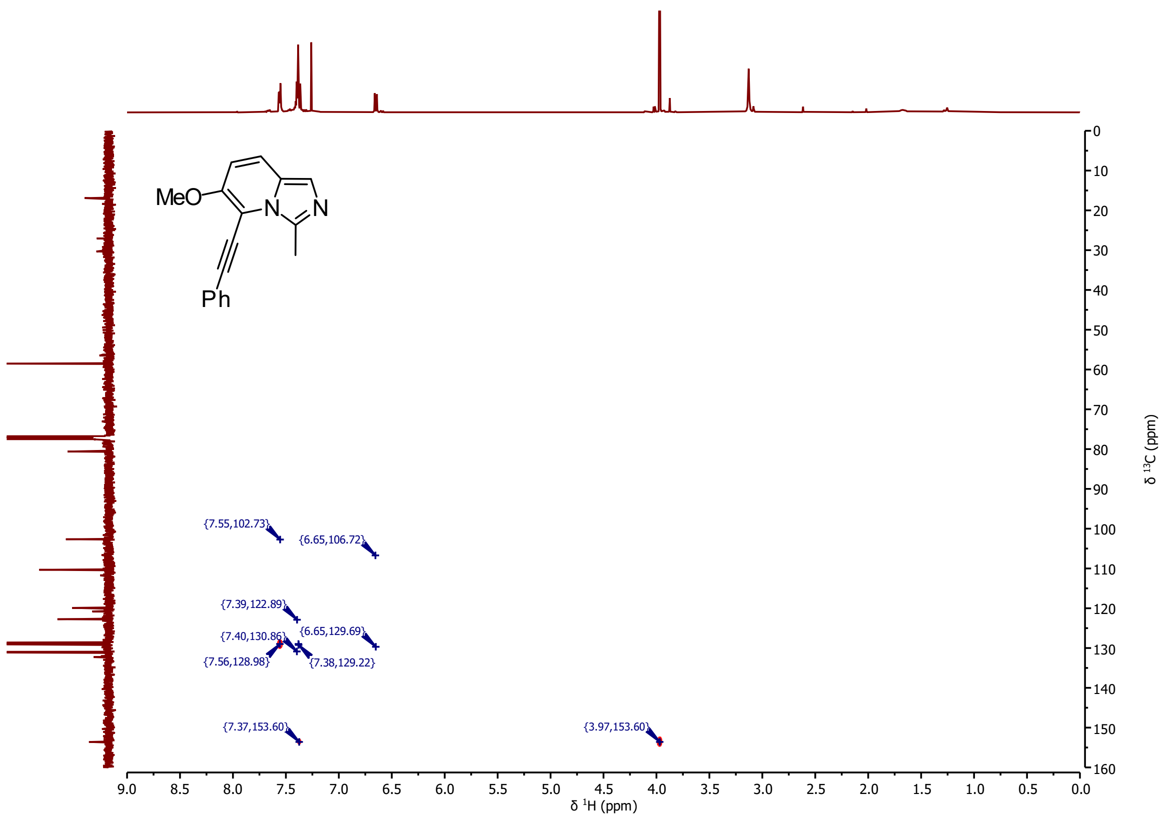

**Figure S174**: **$^{1}H/^{13}C$ HMBC** (500/126 MHz, $CDCl_3$, 298 K) of **S36**.

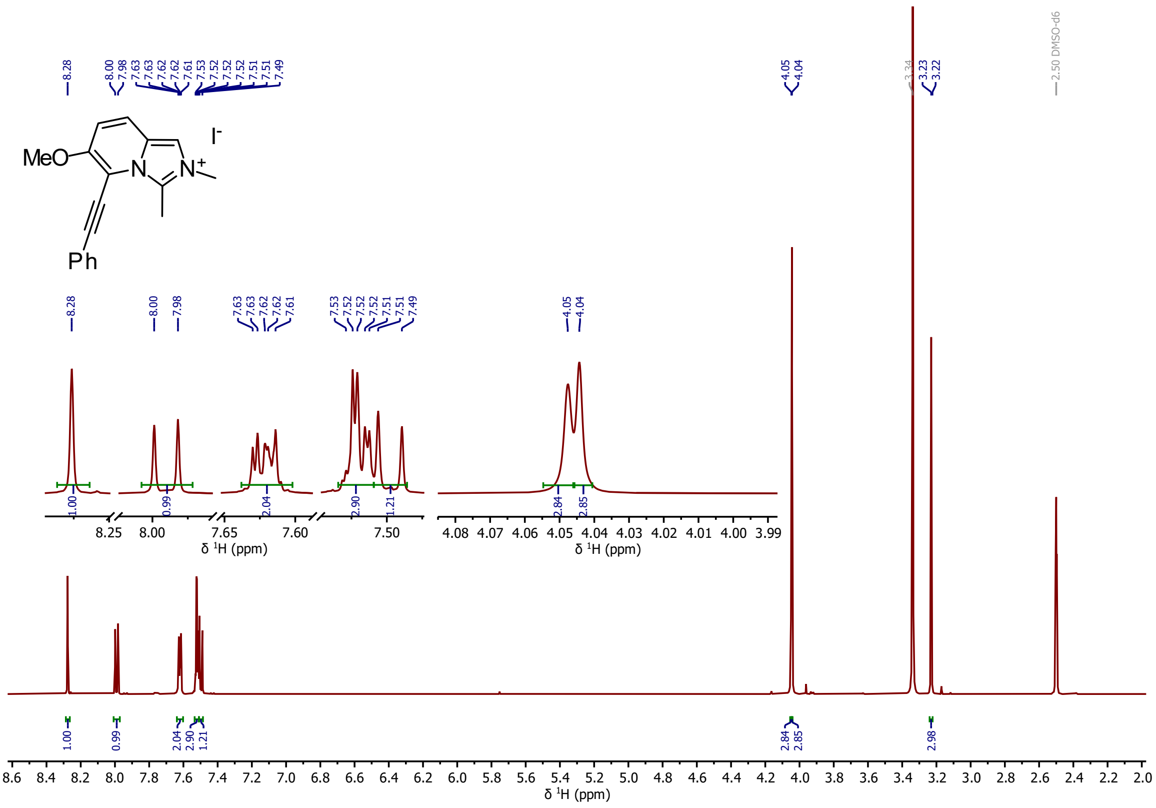

**Figure S175**: **$^{1}$H NMR** (500 MHz, $CDCl_3$, 298 K) of **4h**.

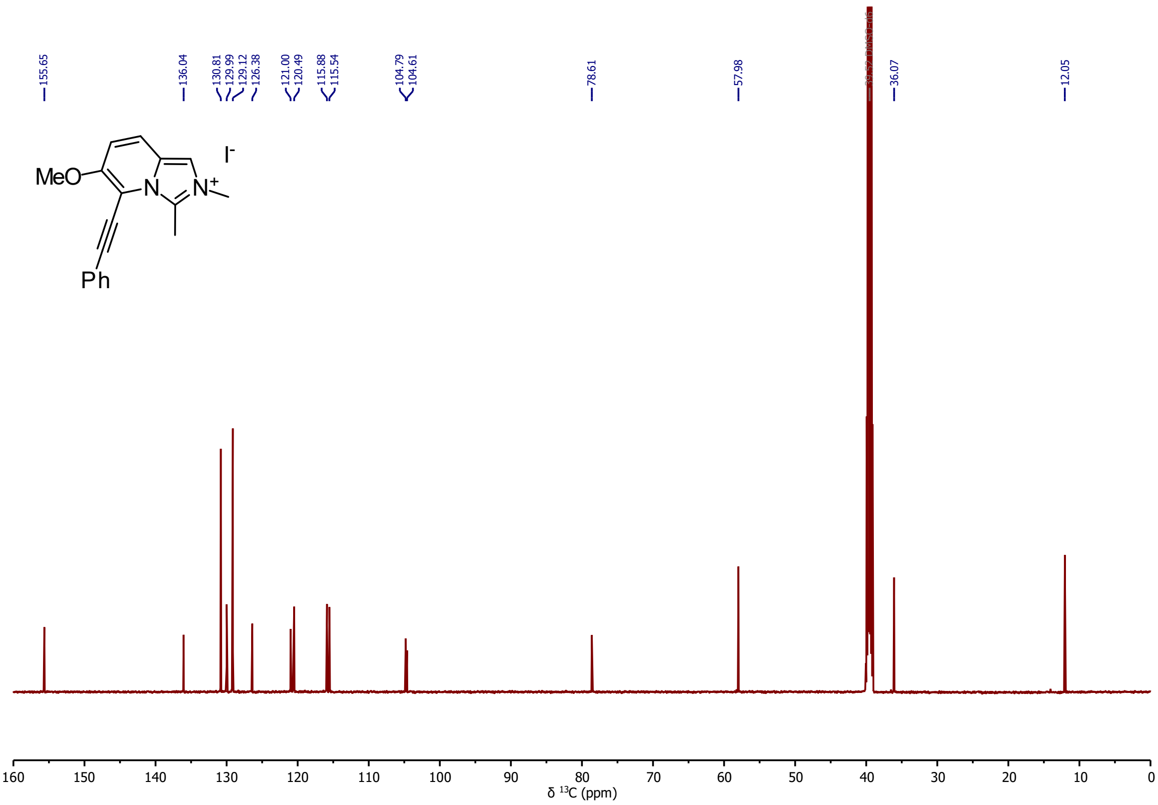

**Figure S176**: **$^{13}$C NMR** (126 MHz, $CDCl_3$, 298 K) of **4h**.

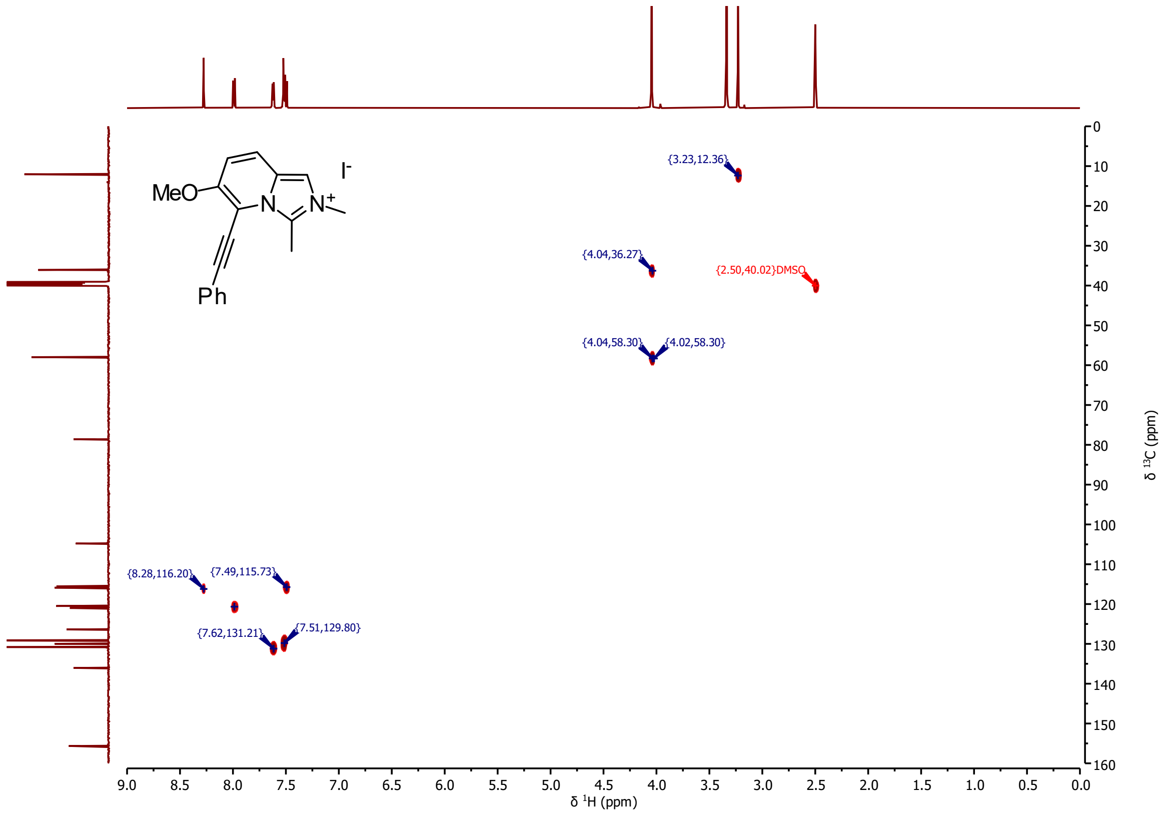

**Figure S177**: **$^{1}H/^{13}C$ HSQC** (500/126 MHz, $CDCl_3$, 298 K) of **4h**.

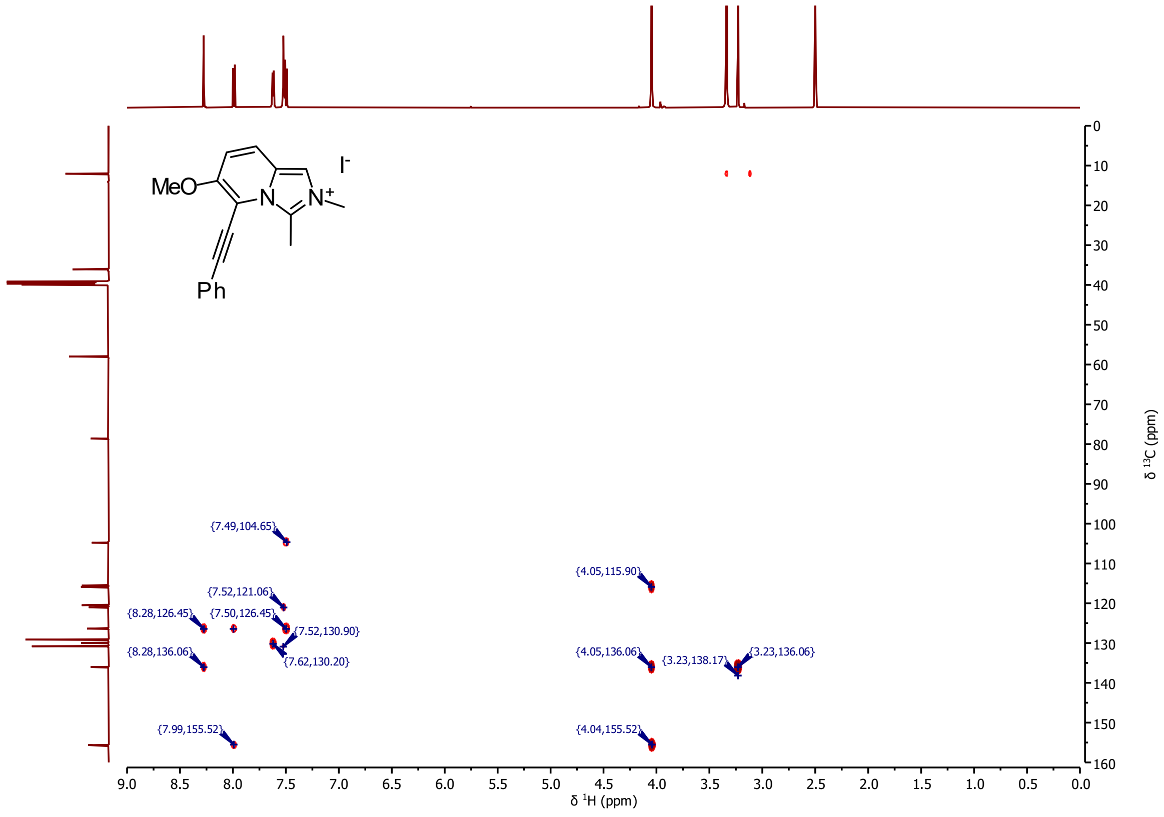

**Figure S178**: **$^{1}H/^{13}C$ HMBC** (500/126 MHz, $CDCl_3$, 298 K) of **4h**.

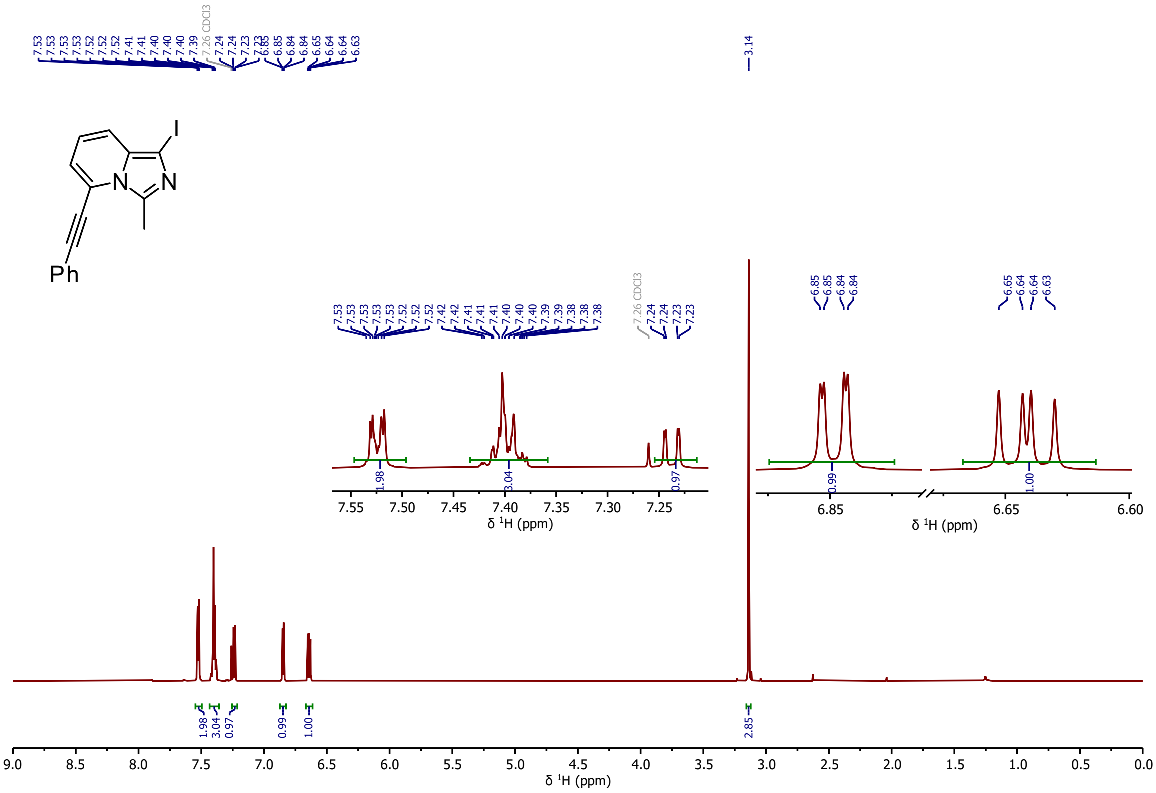


**Figure S179**: **$^{1}$H NMR** (700 MHz, $CDCl_3$, 298 K) of **S37**.

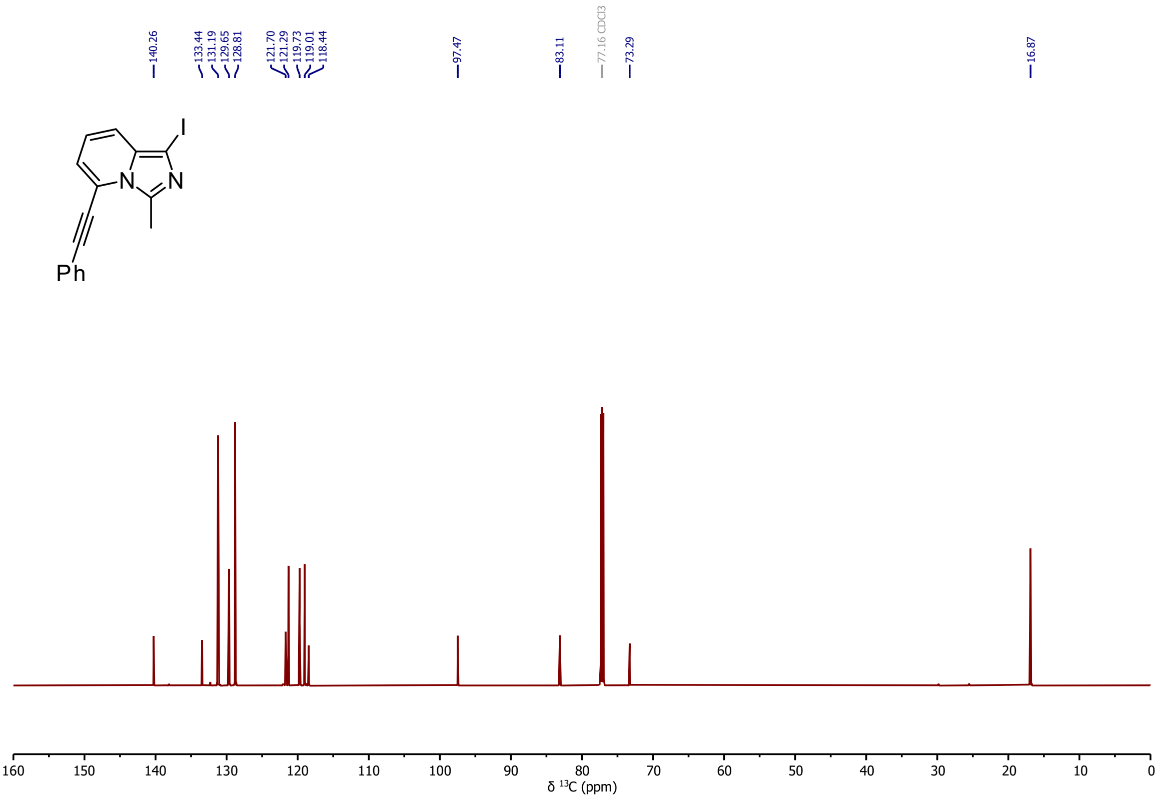


**Figure S180**: **$^{13}$C NMR** (176 MHz, $CDCl_3$, 298 K) of **S37**.

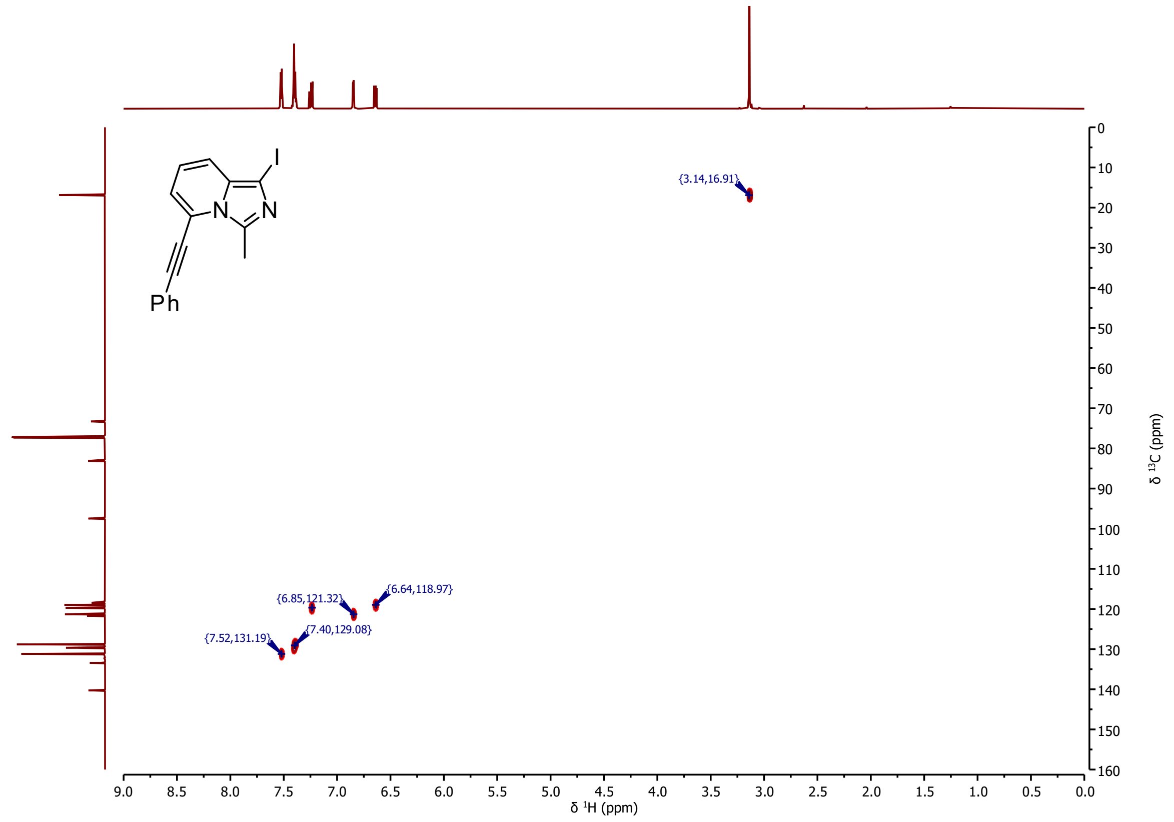


**Figure S181**: **$^1$H/$^{13}$C HSQC** (700/176 MHz, $CDCl_3$, 298 K) of **S37**.

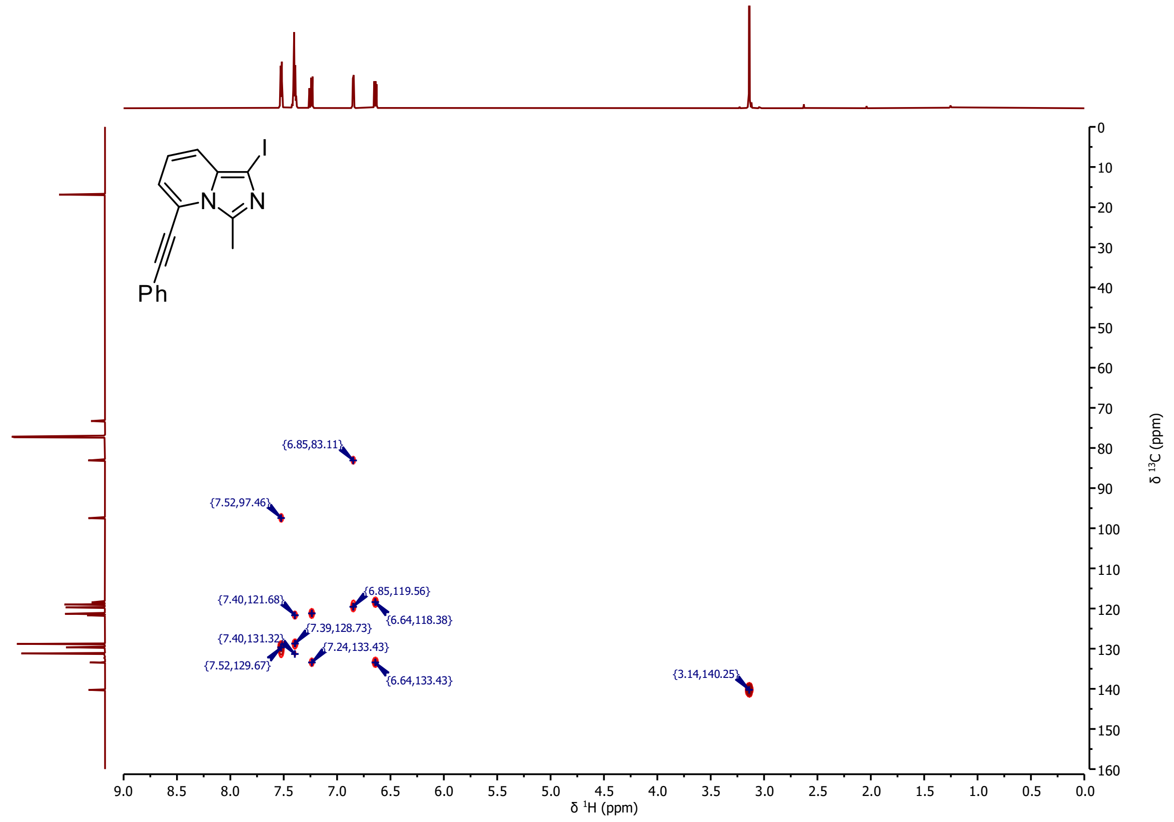


**Figure S182**: **$^1$H/$^{13}$C HMBC** (700/176 MHz, $CDCl_3$, 298 K) of **S37**.

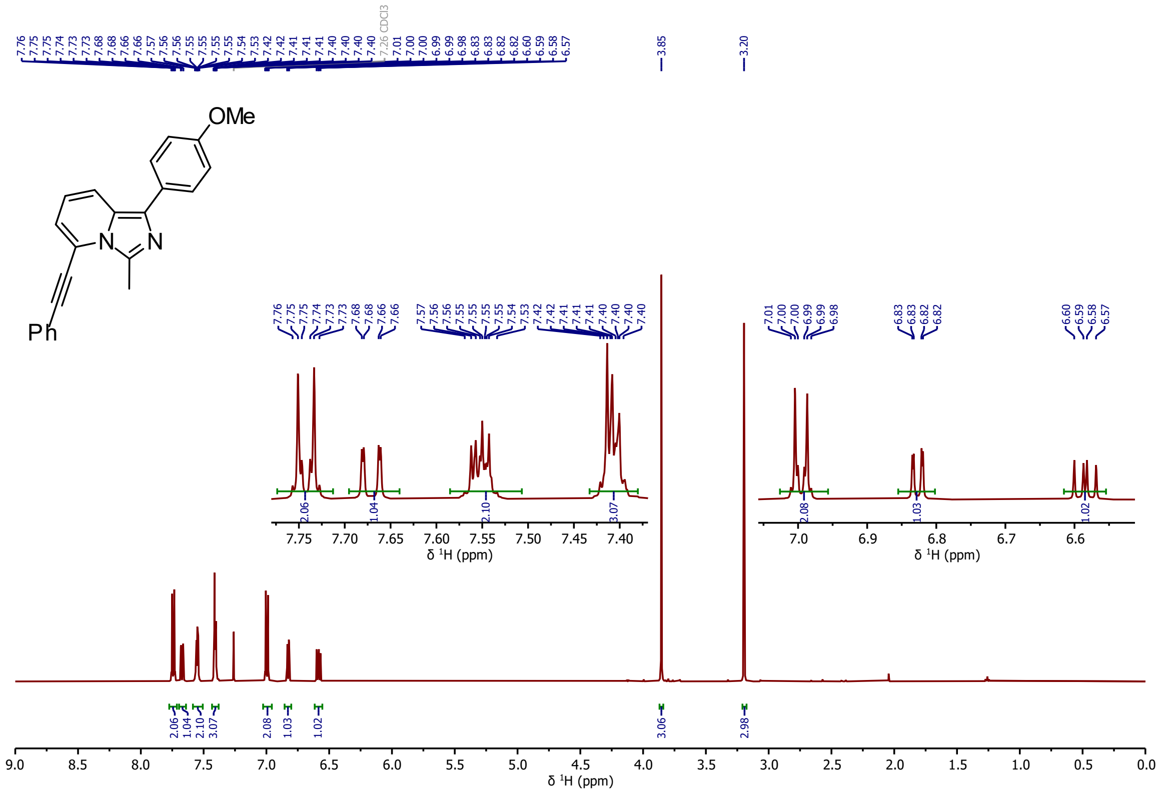


**Figure S183**: **$^1$H NMR** (500 MHz, $CDCl_3$, 298 K) of **S38**.

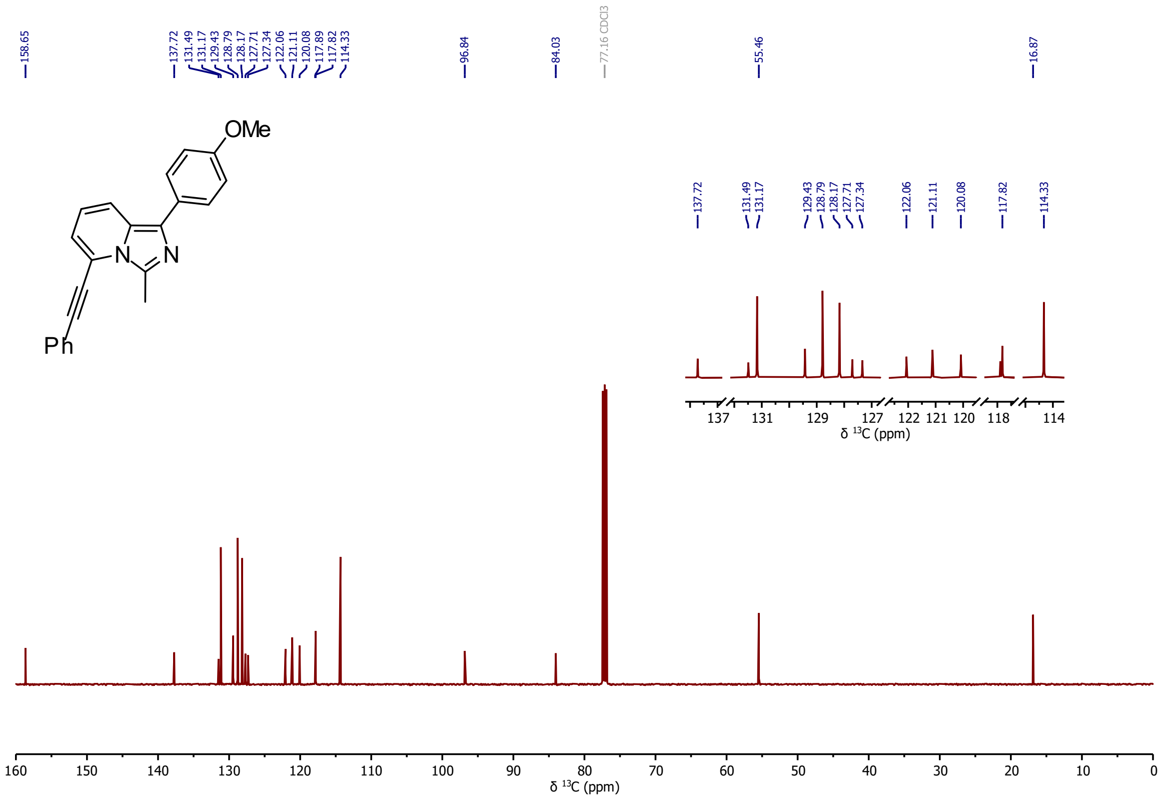


**Figure S184**: **$^{13}$C NMR** (126 MHz, $CDCl_3$, 298 K) of **S38**.

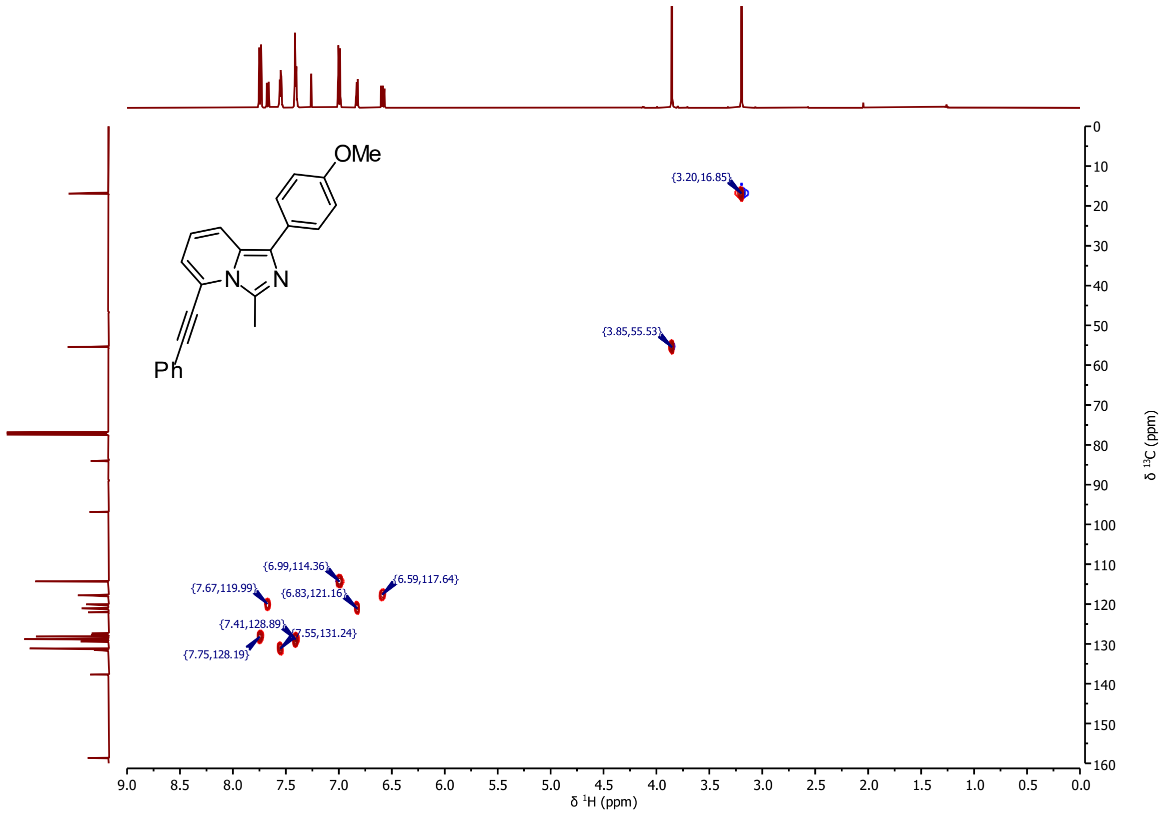

**Figure S185**: **$^1$H/$^{13}$C HSQC** (500/126 MHz, $CDCl_3$, 298 K) of **S38**.

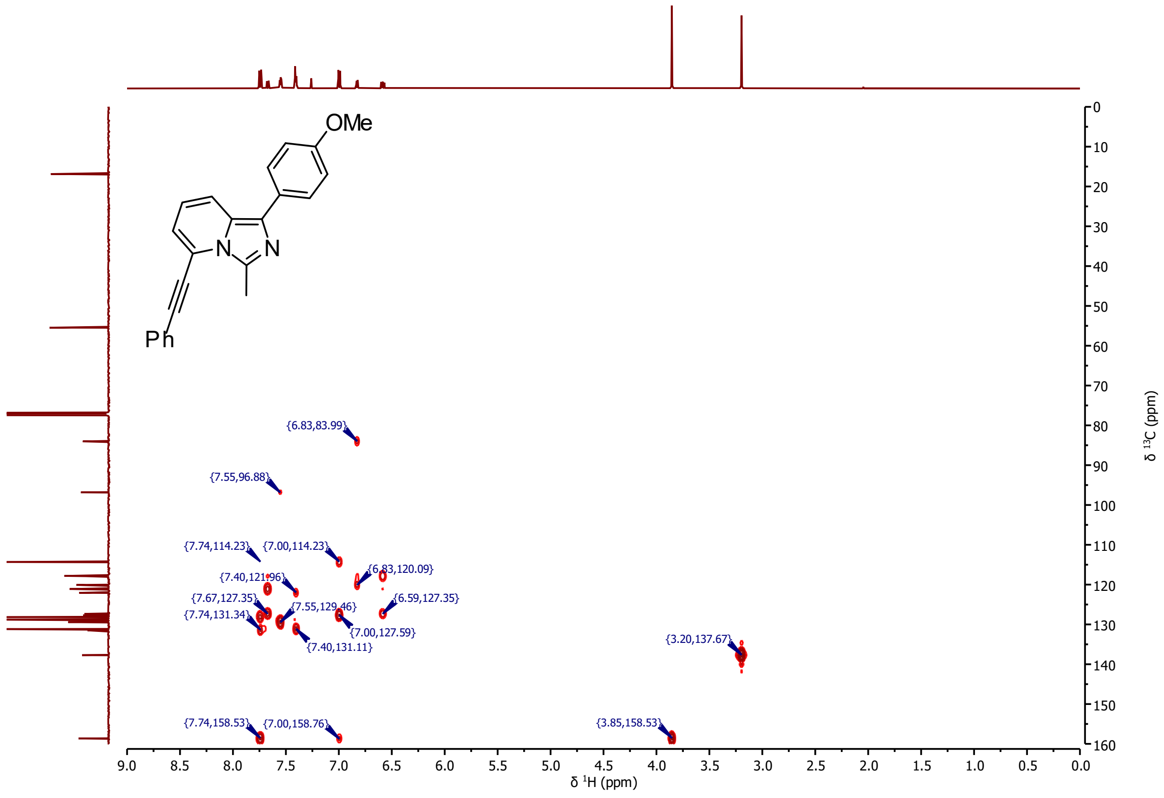

**Figure S186**: **$^1$H/$^{13}$C HMBC** (500/126 MHz, $CDCl_3$, 298 K) of **S38**.

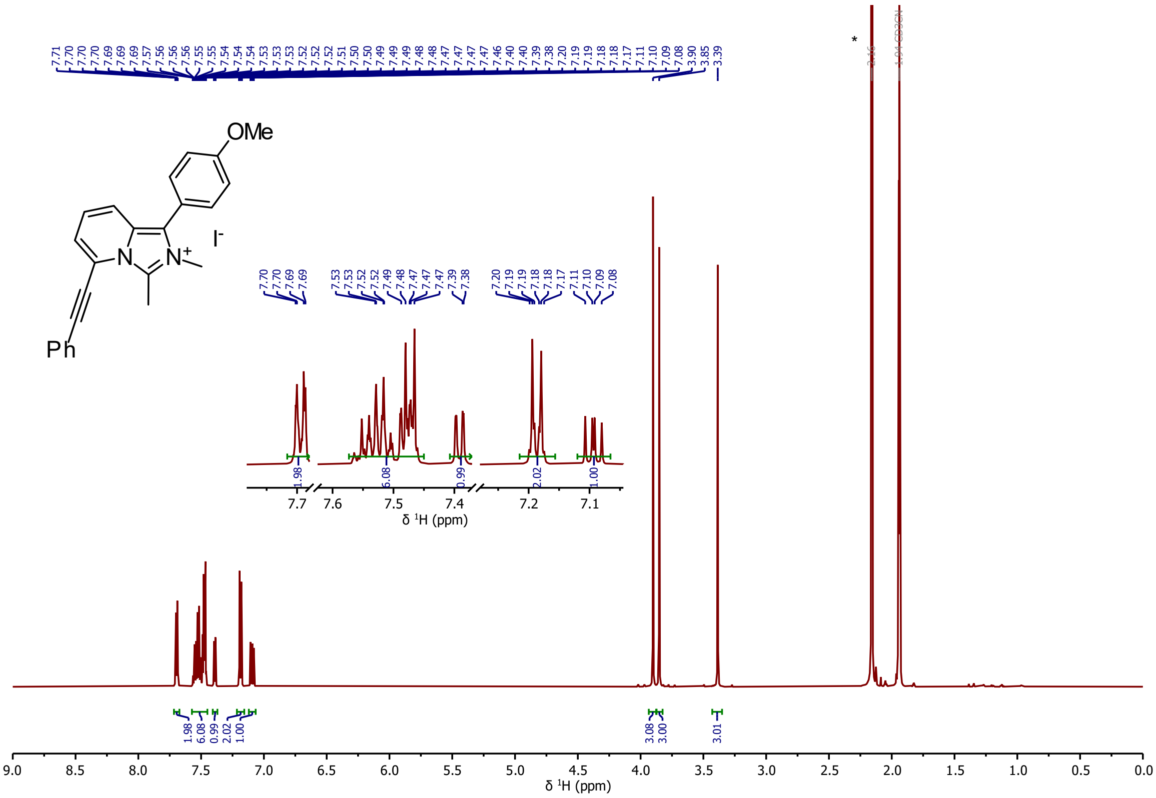

**Figure S187**: **$^{1}$H NMR** (600 MHz, $CD_3CN$, 298 K) of **4i**. * = $H_2O$.

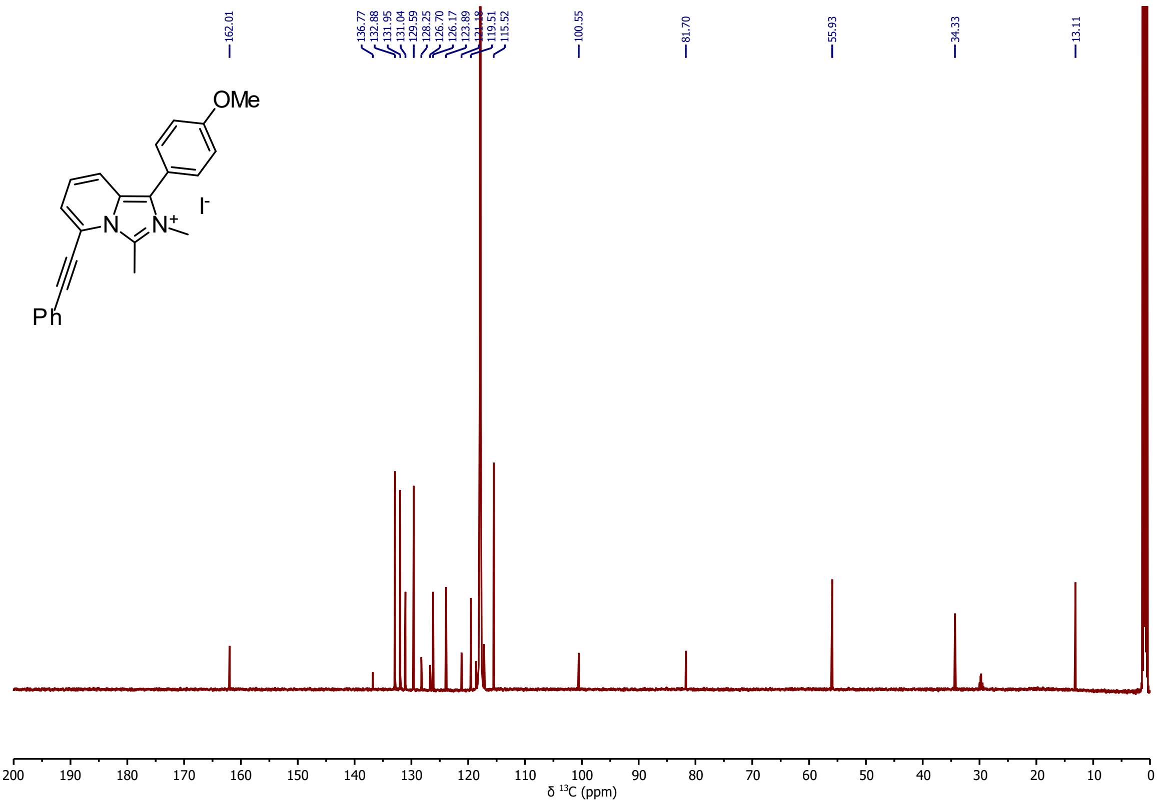

**Figure S188**: **$^{13}$C NMR** (151 MHz, $CD_3CN$, 298 K) of **4i**.

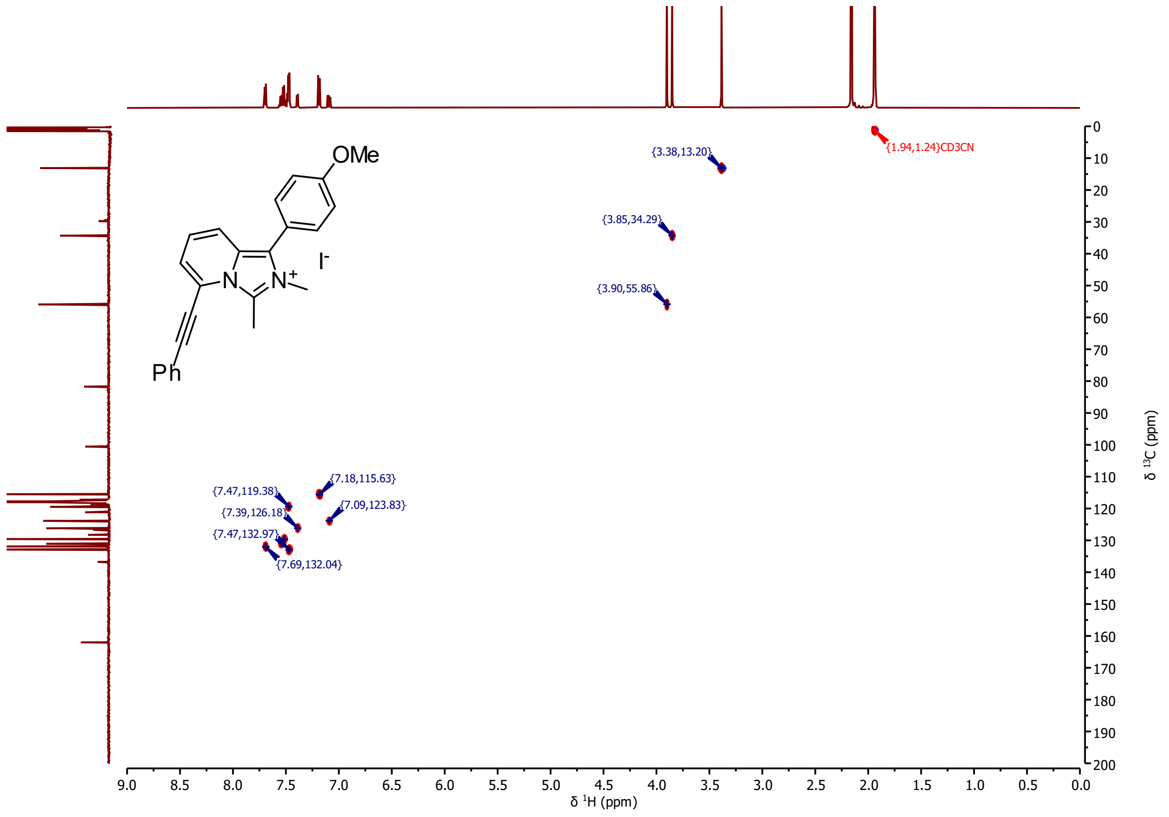


**Figure S189**: **$^{1}H/^{13}C$ HSQC** (600/151 MHz, $CD_3CN$, 298 K) of **4i**.

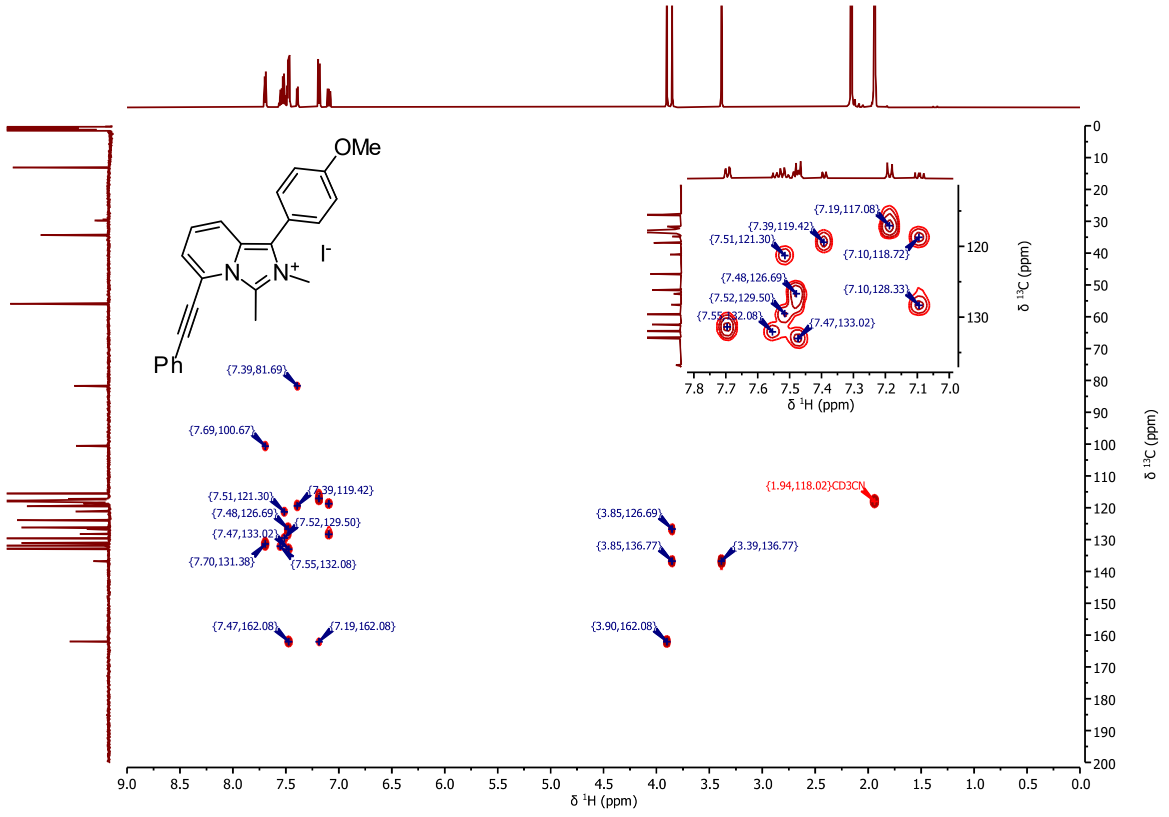


**Figure S190**: **$^{1}H/^{13}C$ HMBC** (600/151 MHz, $CD_3CN$, 298 K) of **4i**.

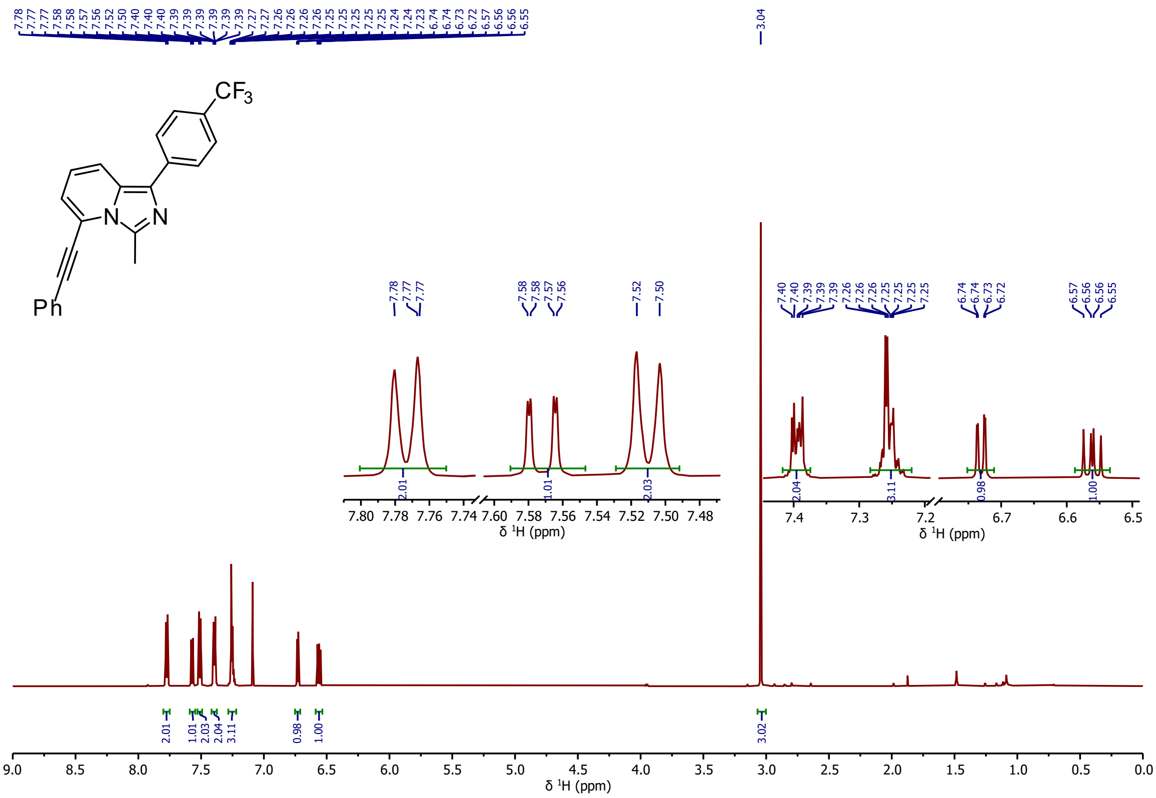

**Figure S191**: **$^{1}$H NMR** (600 MHz, $CDCl_3$, 298 K) of **S39**.

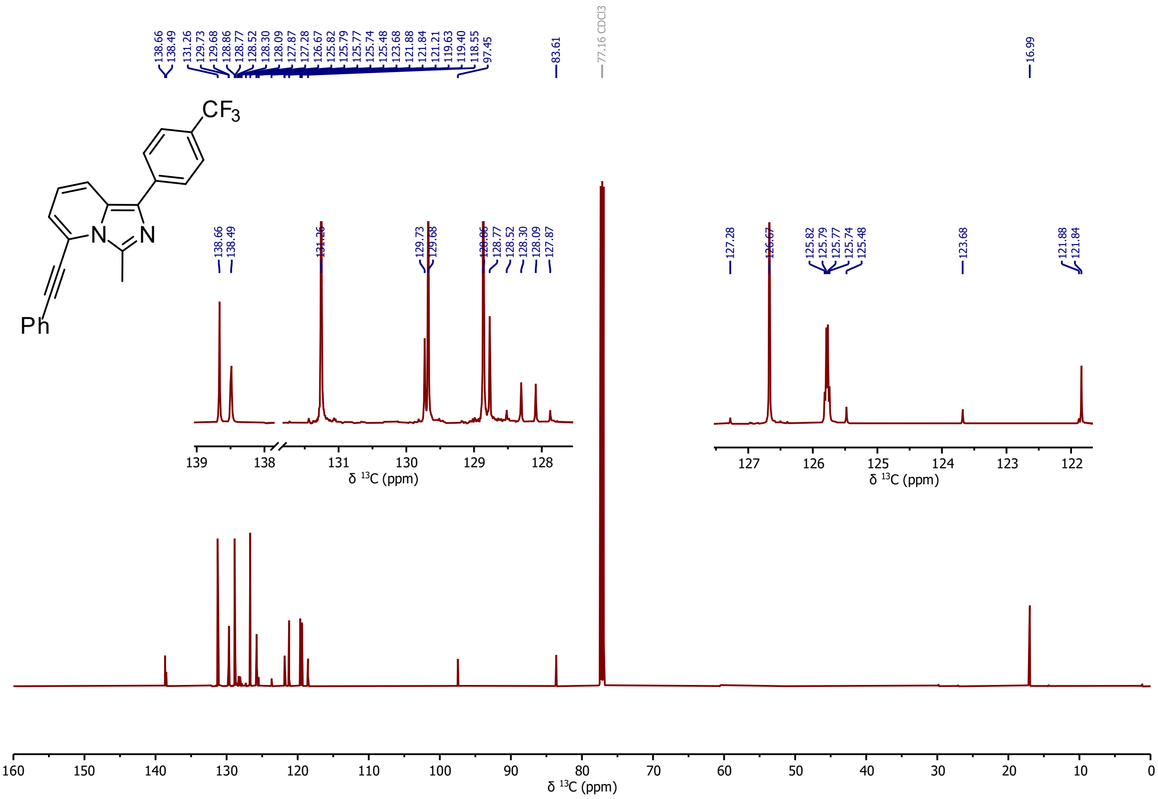

**Figure S192**: **$^{13}$C NMR** (151 MHz, $CDCl_3$, 298 K) of **S39**.

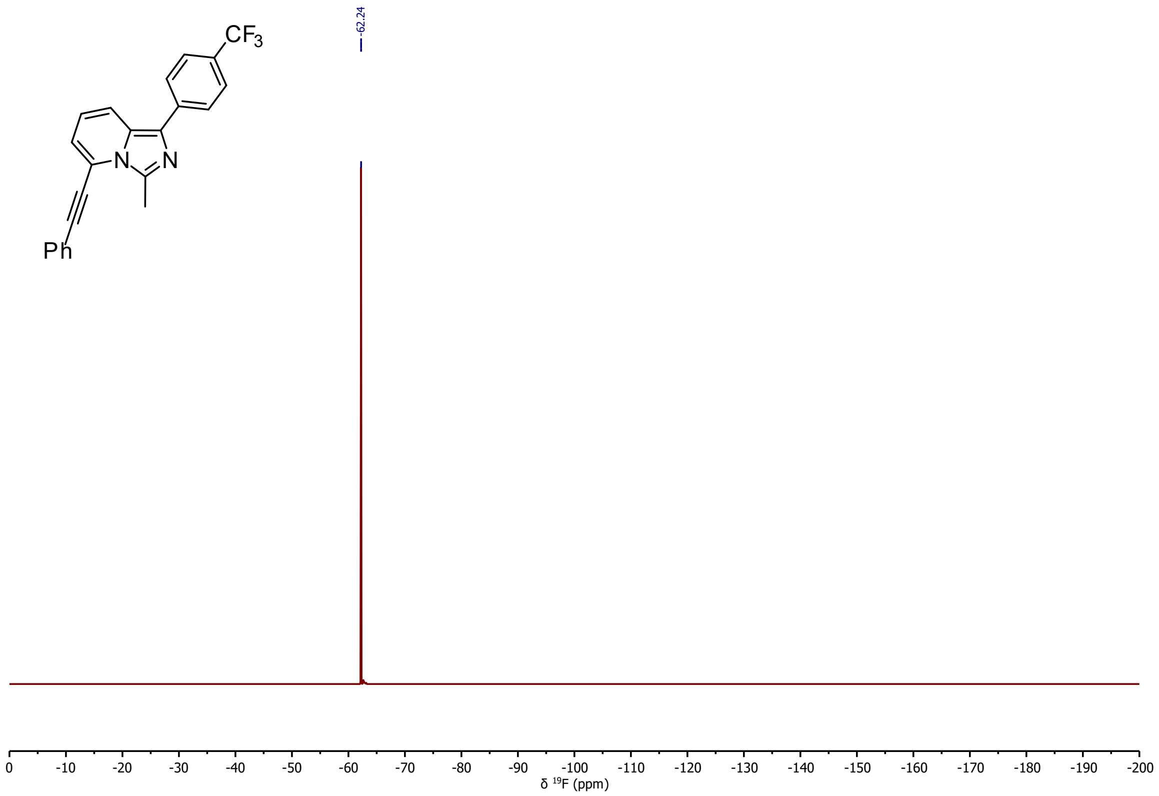


**Figure S193**: $^{19}$**F NMR** (565 MHz, $CDCl_3$, 298 K) of **S39**.

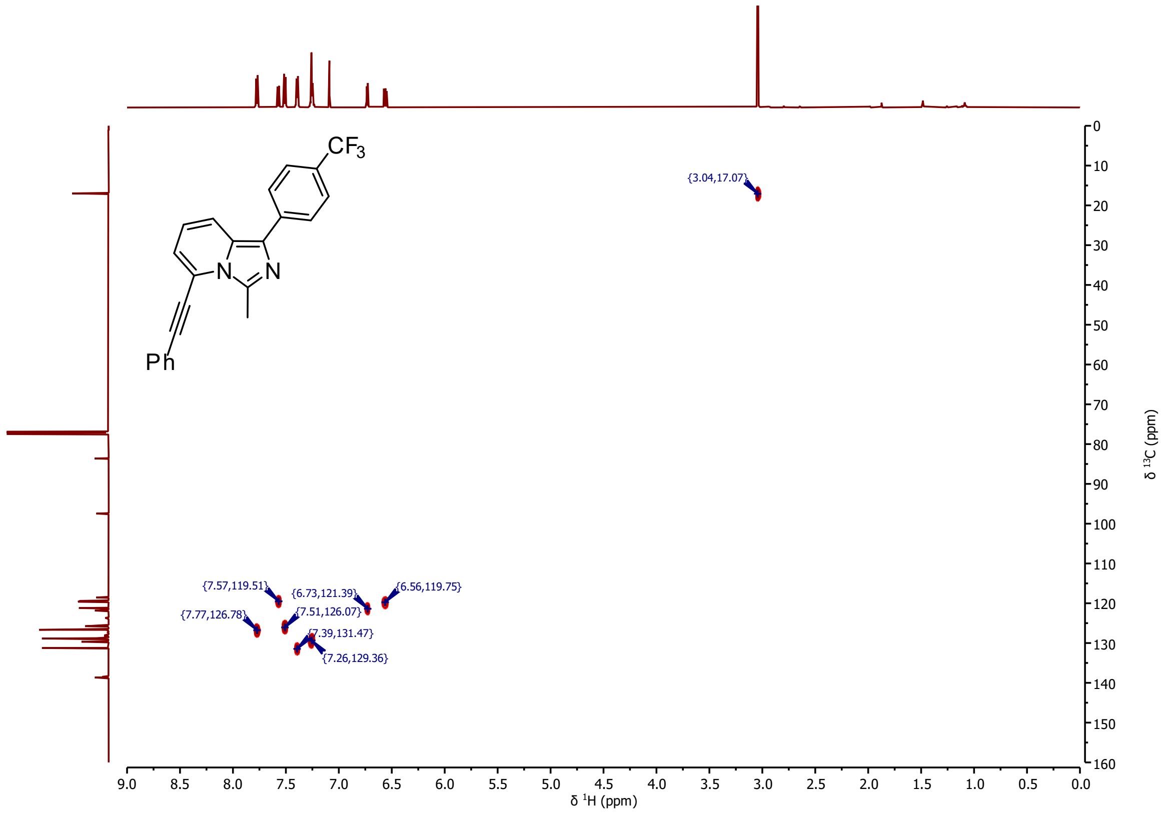


**Figure S194**: $^{1}$**H/**$^{13}$**C HSQC** (600/151 MHz, $CDCl_3$, 298 K) of **S39**.

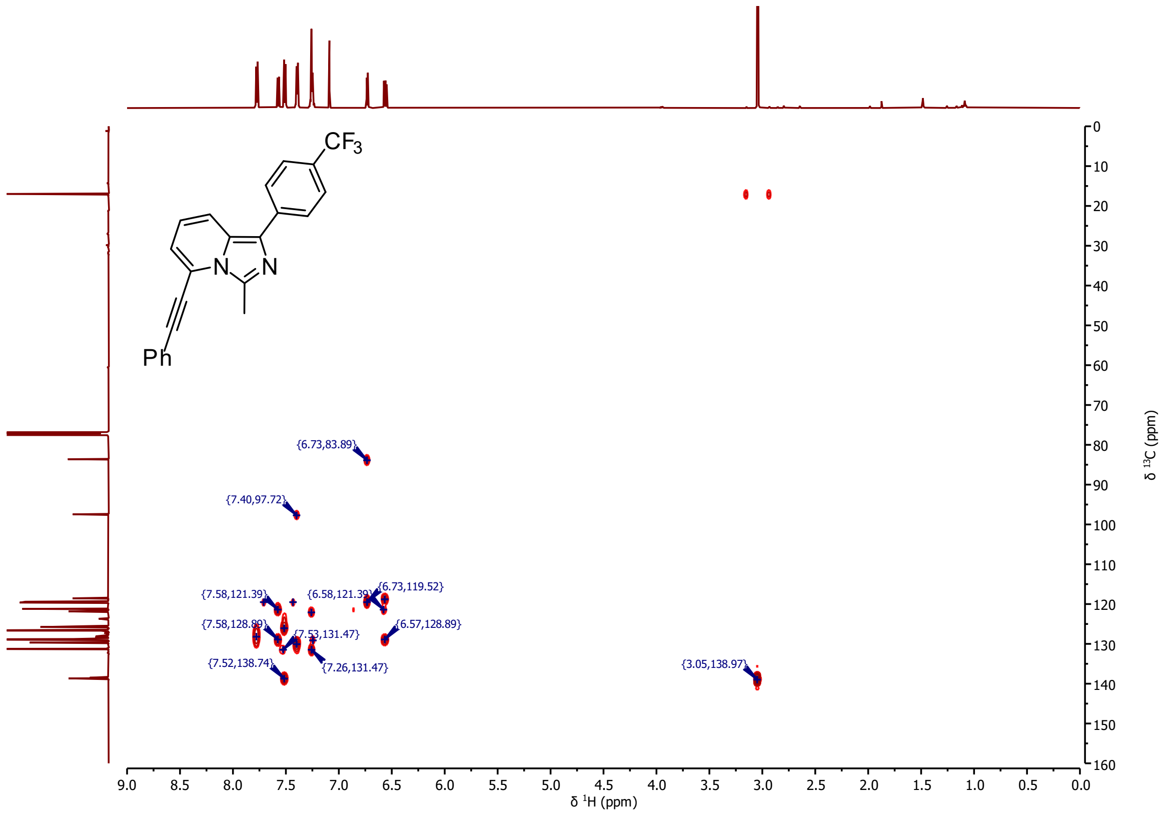

**Figure S195**: **$^{1}$H/$^{13}$C HMBC** (600/151 MHz, $CDCl_3$, 298 K) of **S39**.

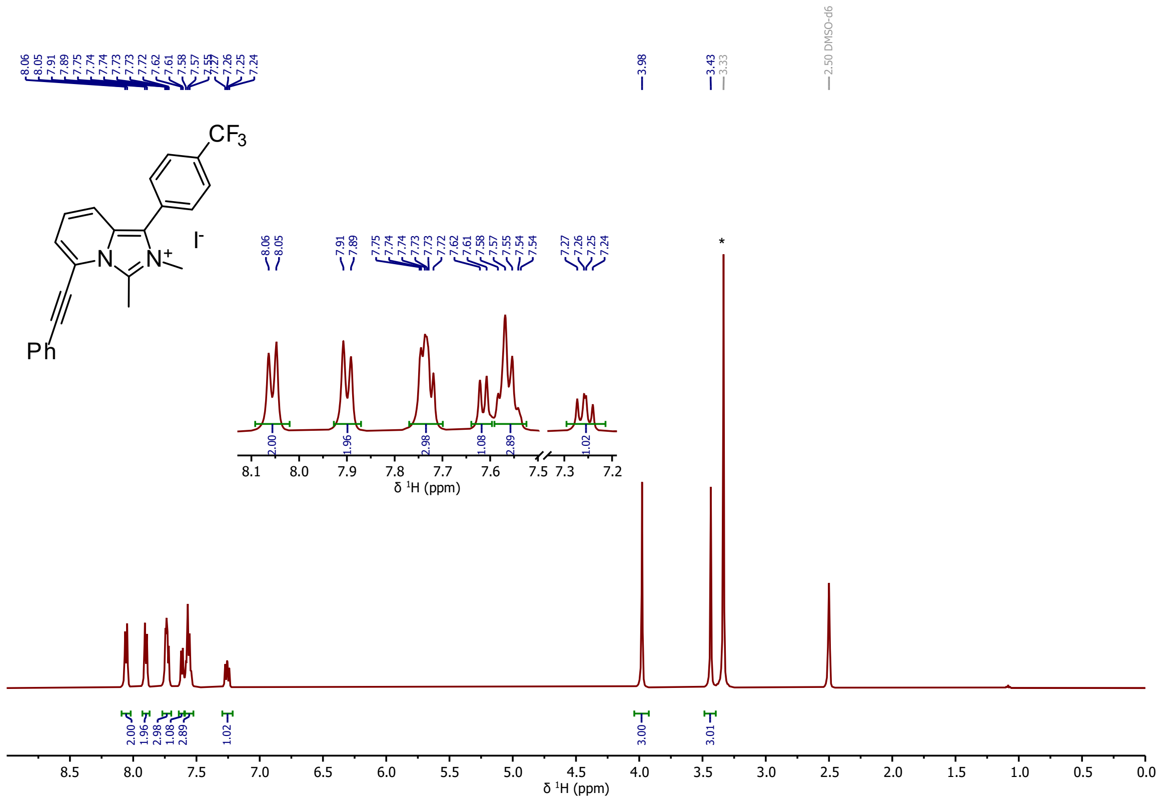

**Figure S196**: **$^{1}$H NMR** (500 MHz, $CDCl_3$, 298 K) of **4j**. * = $H_2O$.

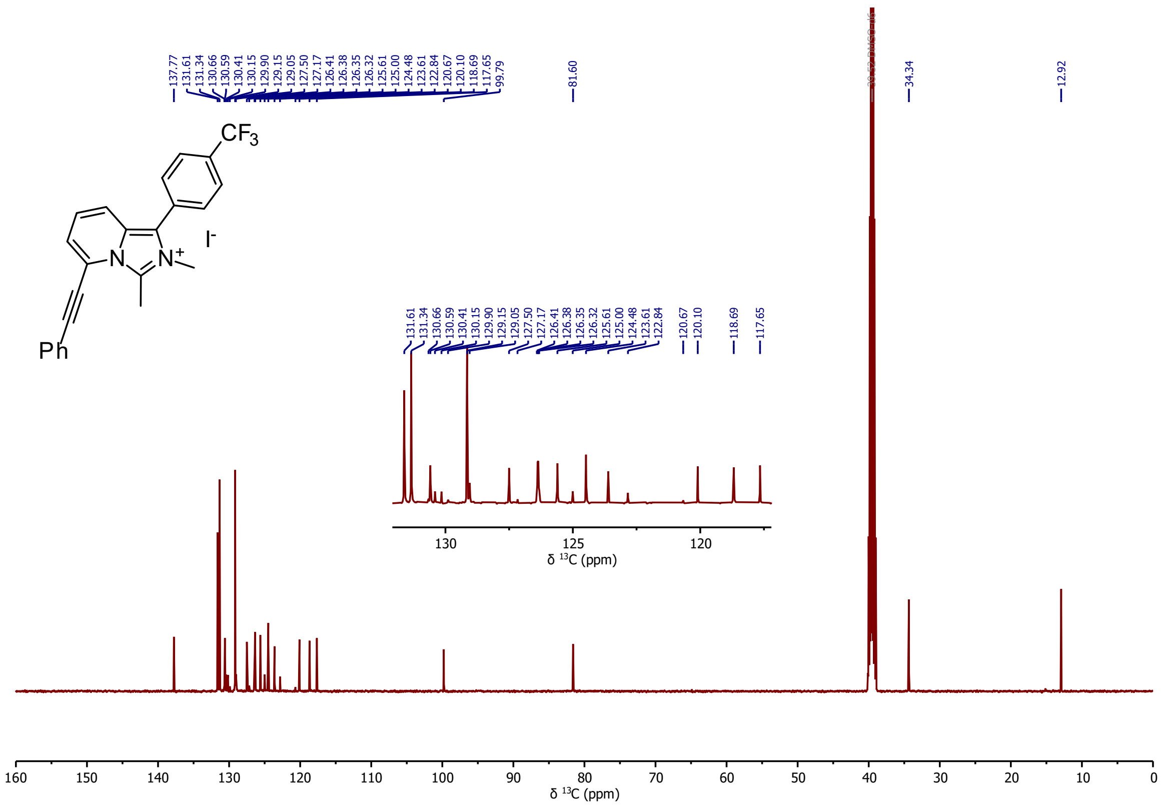

**Figure S197**: $^{13}$**C NMR** (126 MHz, $CDCl_3$, 298 K) of **4j**.

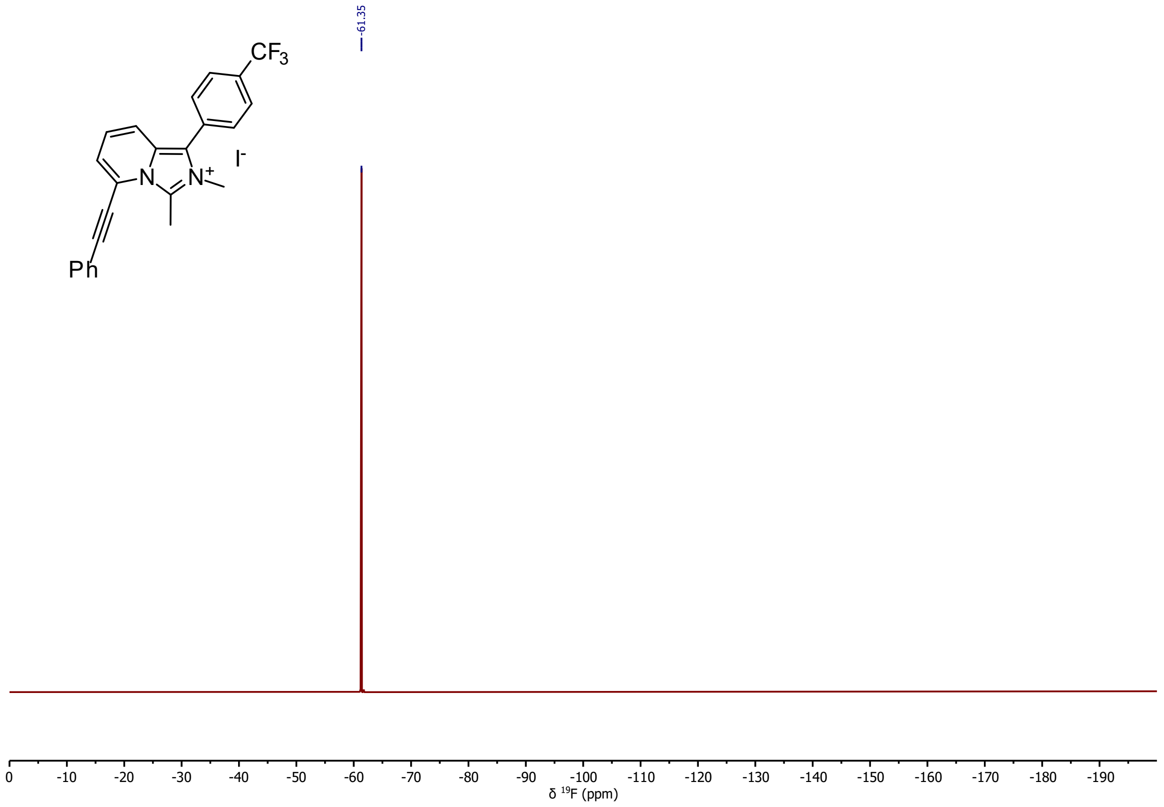

**Figure S198**: $^{19}$**F NMR** (470 MHz, $CDCl_3$, 298 K) of **4j**.

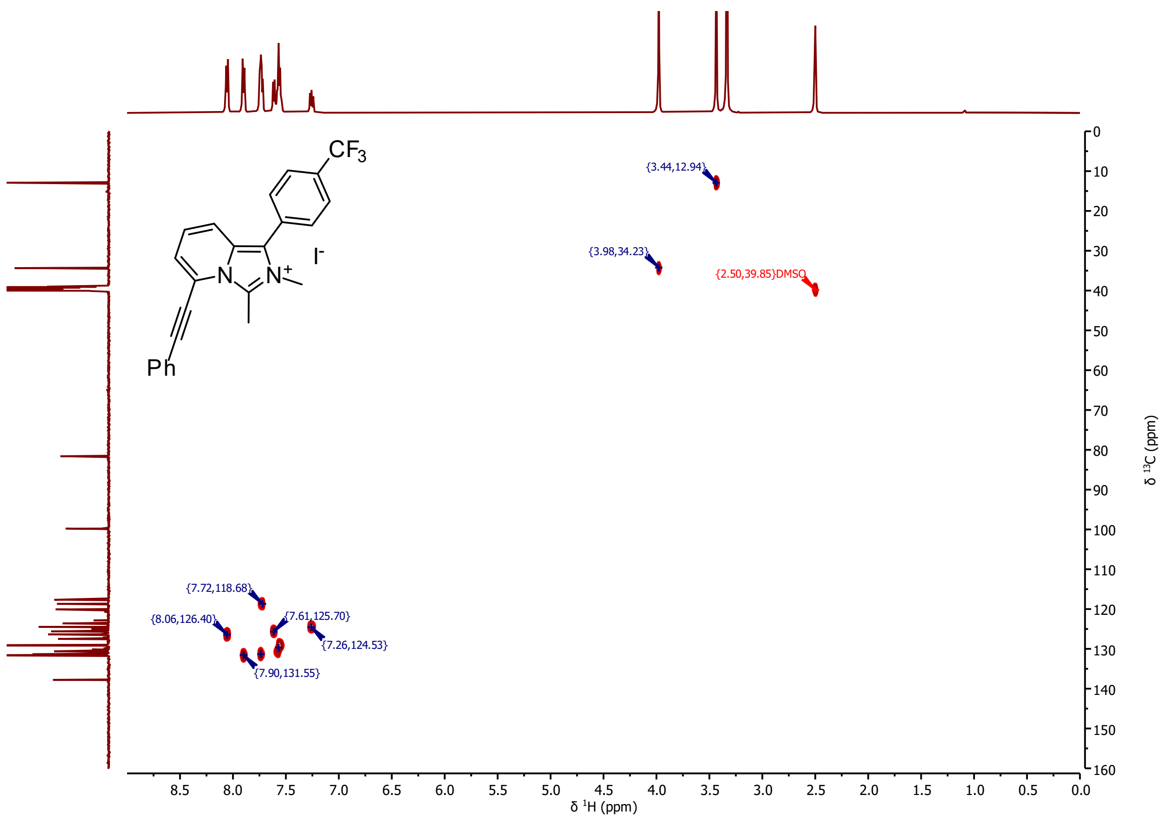

**Figure S199**: **$^{1}$H/$^{13}$C HSQC** (500/126 MHz, $CDCl_3$, 298 K) of **4j**.

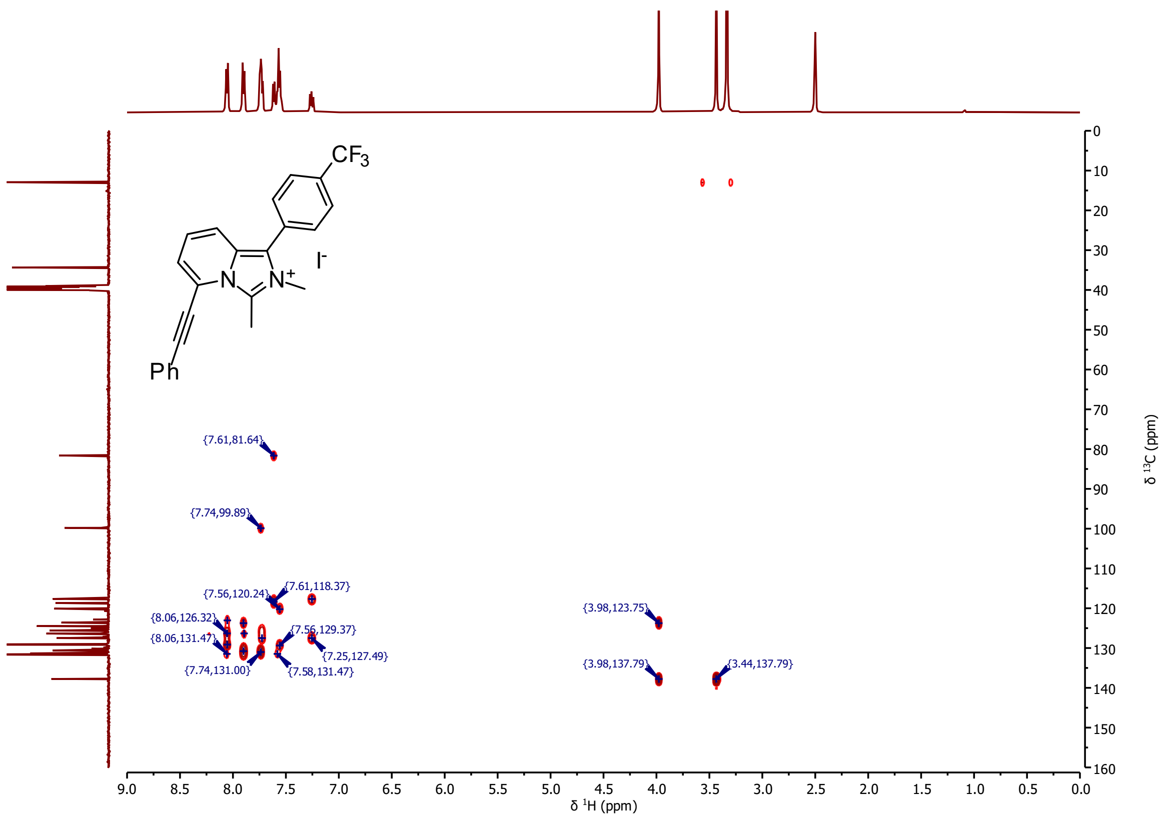

**Figure S200**: **$^{1}$H/$^{13}$C HMBC** (500/126 MHz, $CDCl_3$, 298 K) of **4j**.

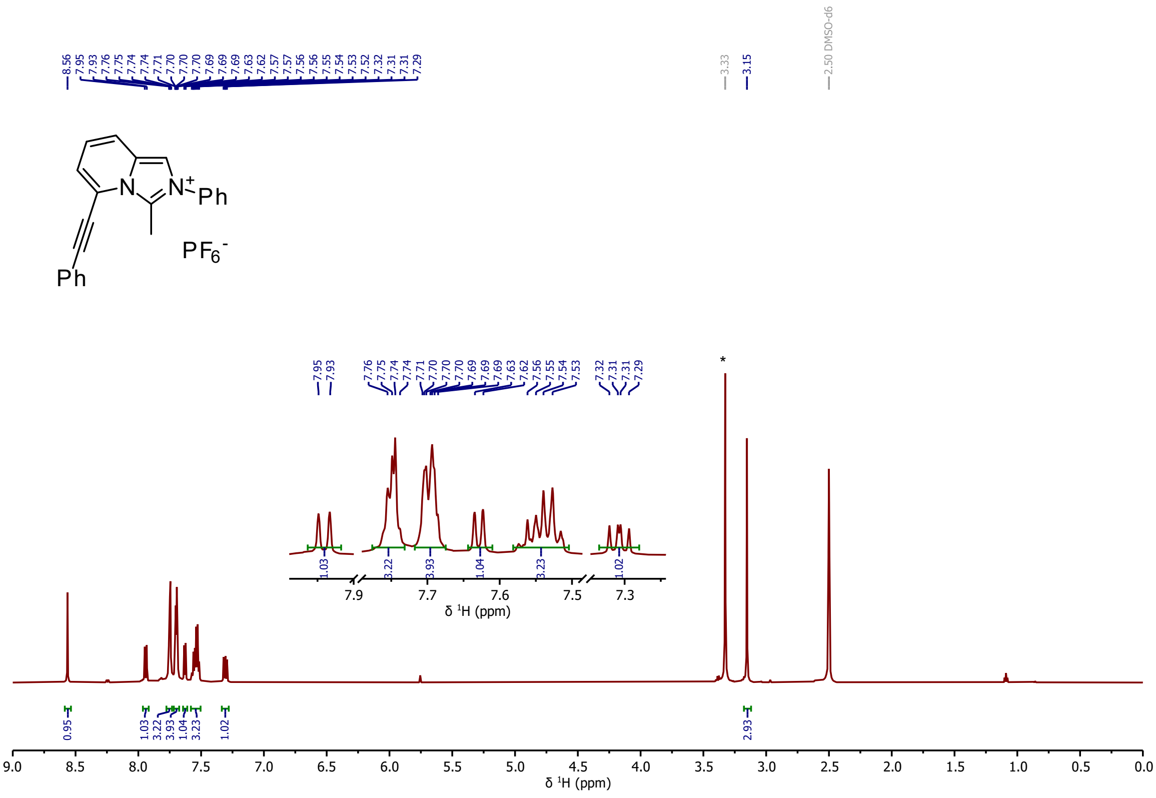

**Figure S201**: **$^{1}$H NMR** (600 MHz, DMSO-d$_6$, 298 K) of **4k**. * = $H_2O$.

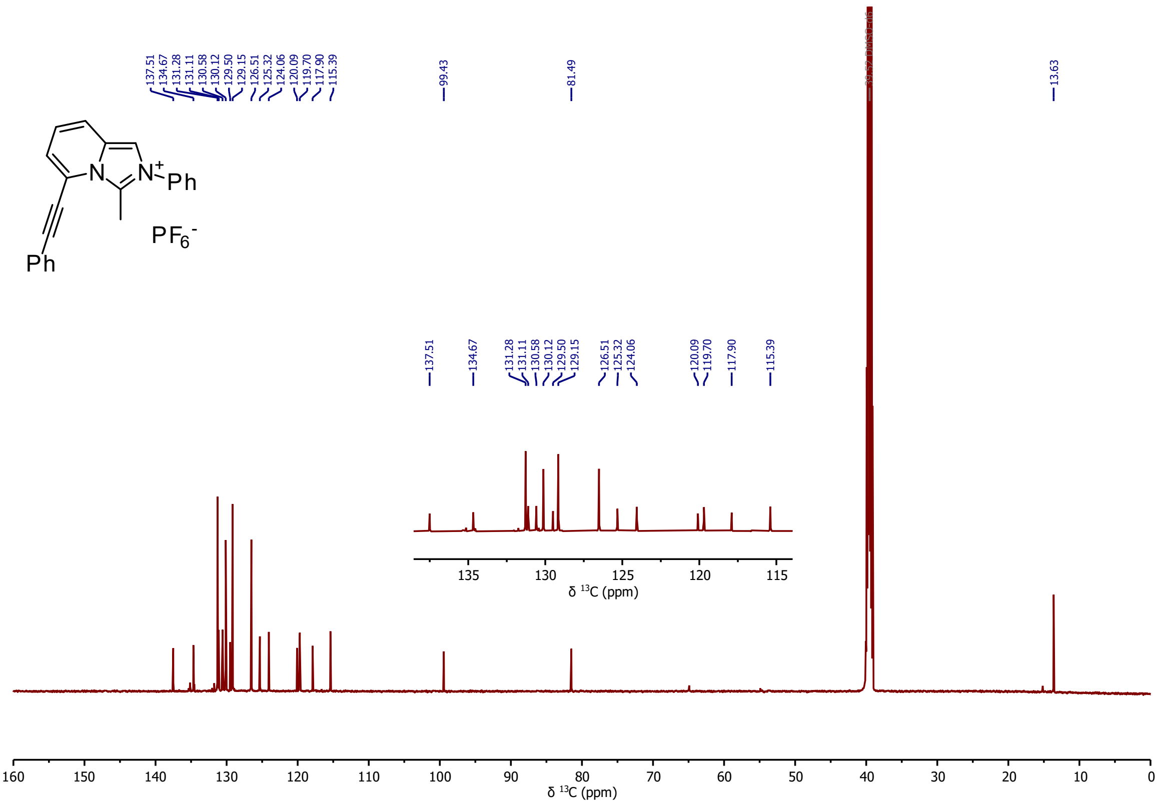

**Figure S202**: **$^{13}$C NMR** (151 MHz, DMSO-d$_6$, 298 K) of **4k**.

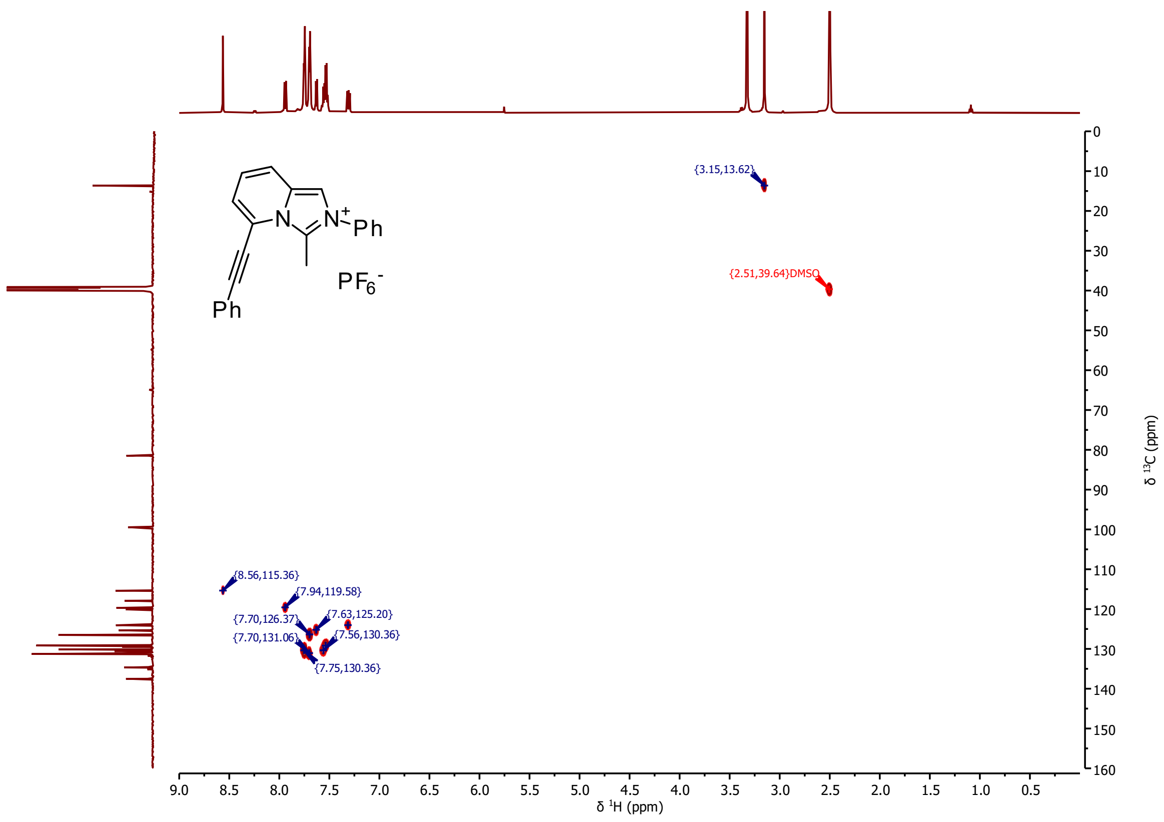

**Figure S203**: **$^1$H/$^{13}$C HSQC** (600/151 MHz, DMSO-$d_6$, 298 K) of **4k**.

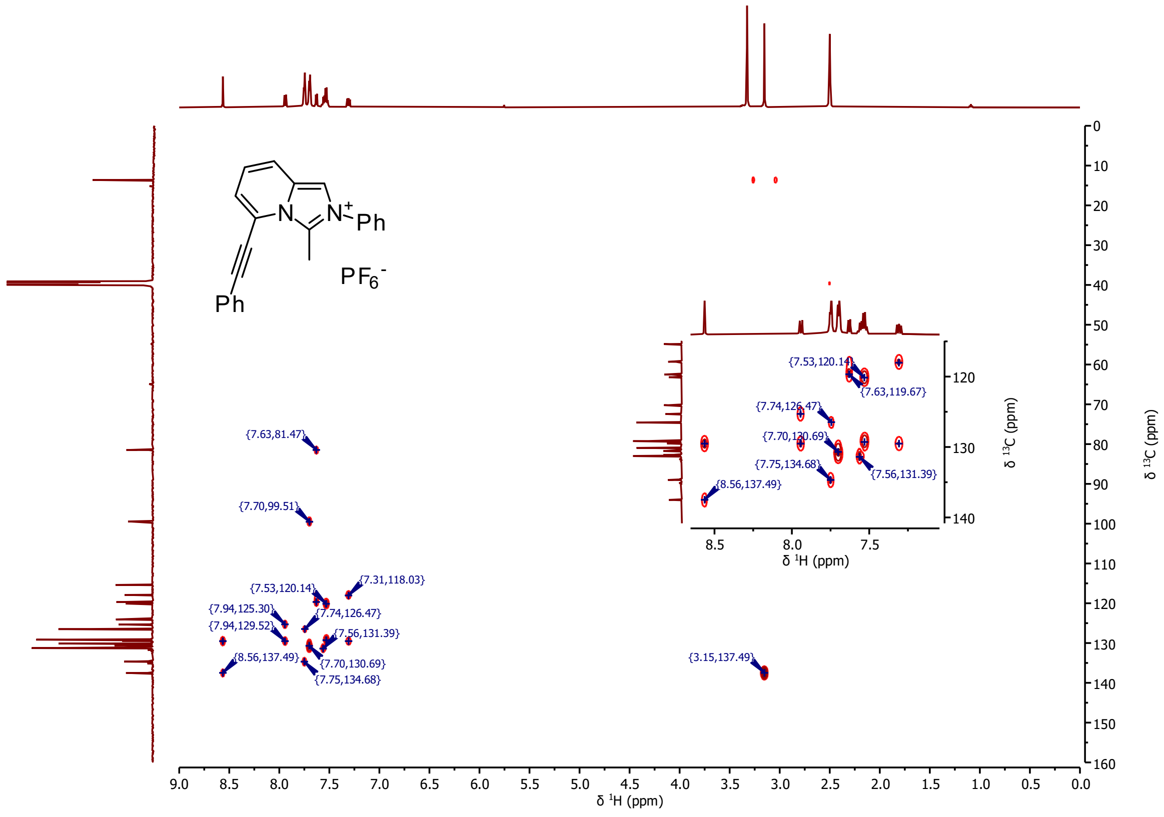

**Figure S204**: **$^1$H/$^{13}$C HMBC** (600/151 MHz, DMSO-$d_6$, 298 K) of **4k**.

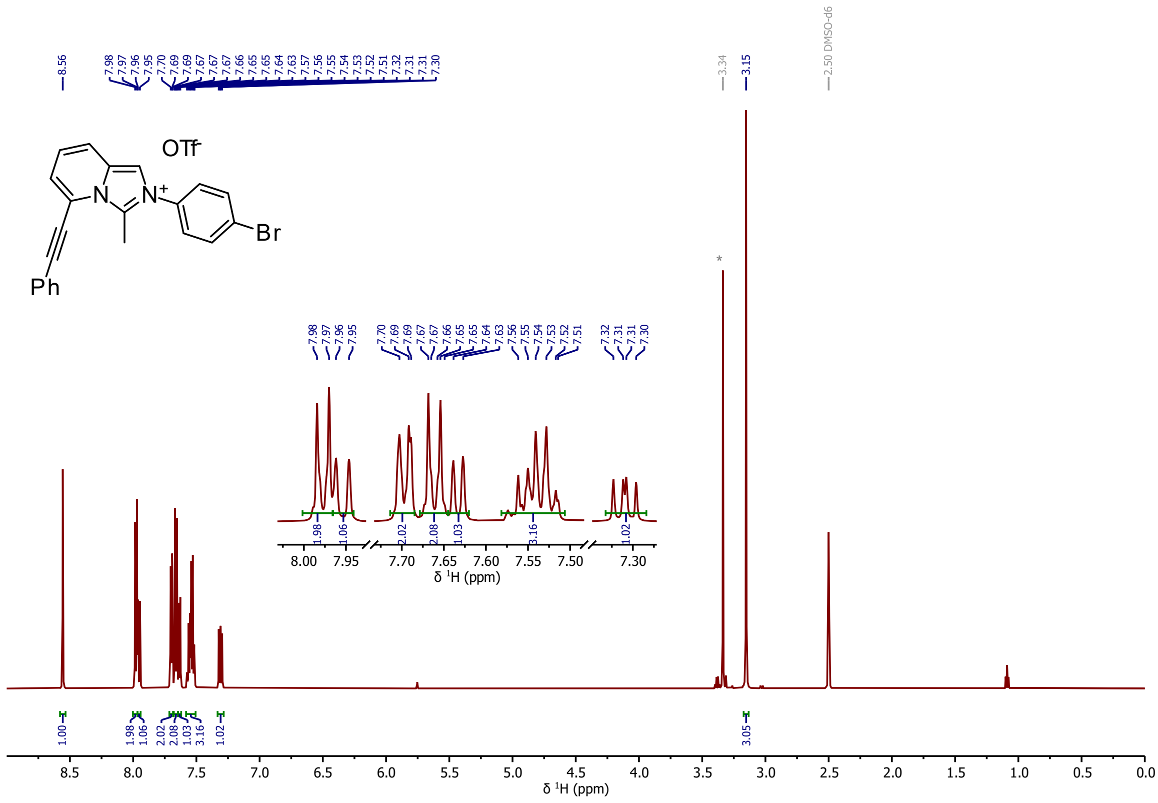


**Figure S205**: **$^1$H NMR** (600 MHz, DMSO-$d_6$, 298 K) of **4l**. * = $H_2O$.

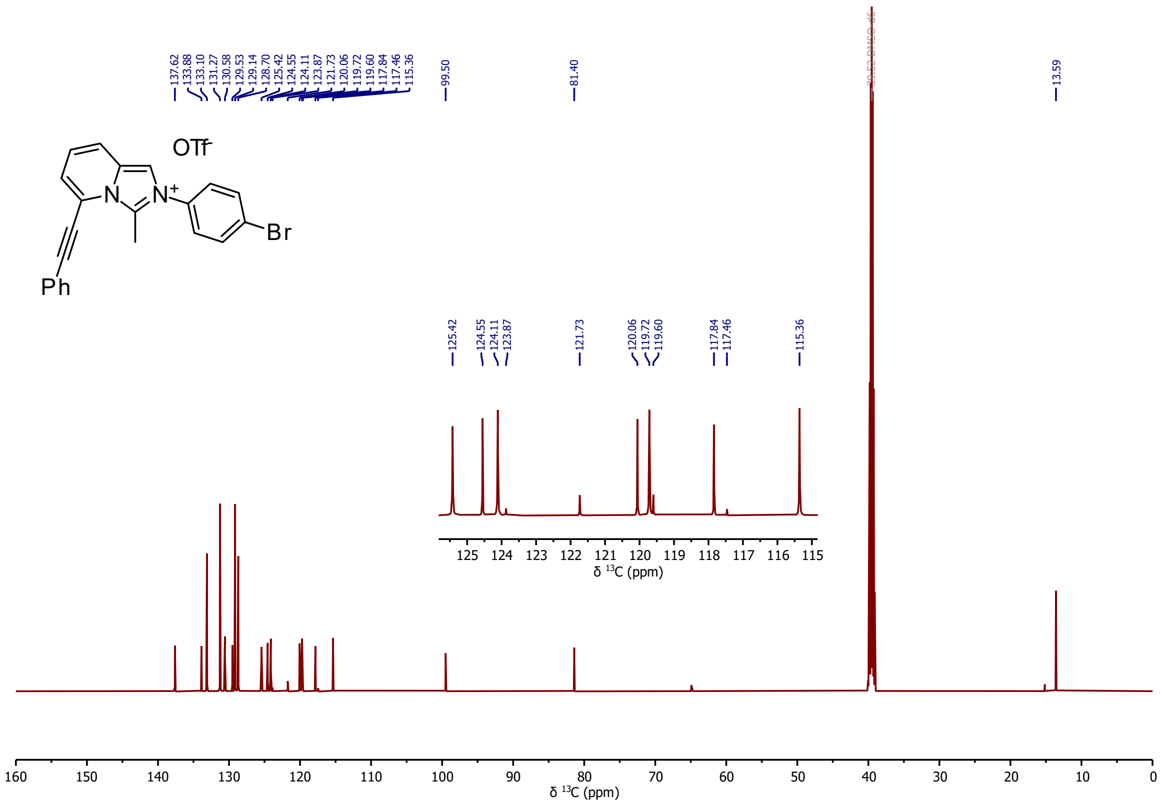


**Figure S206**: **$^{13}$C NMR** (151 MHz, DMSO-$d_6$, 298 K) of **4l**.

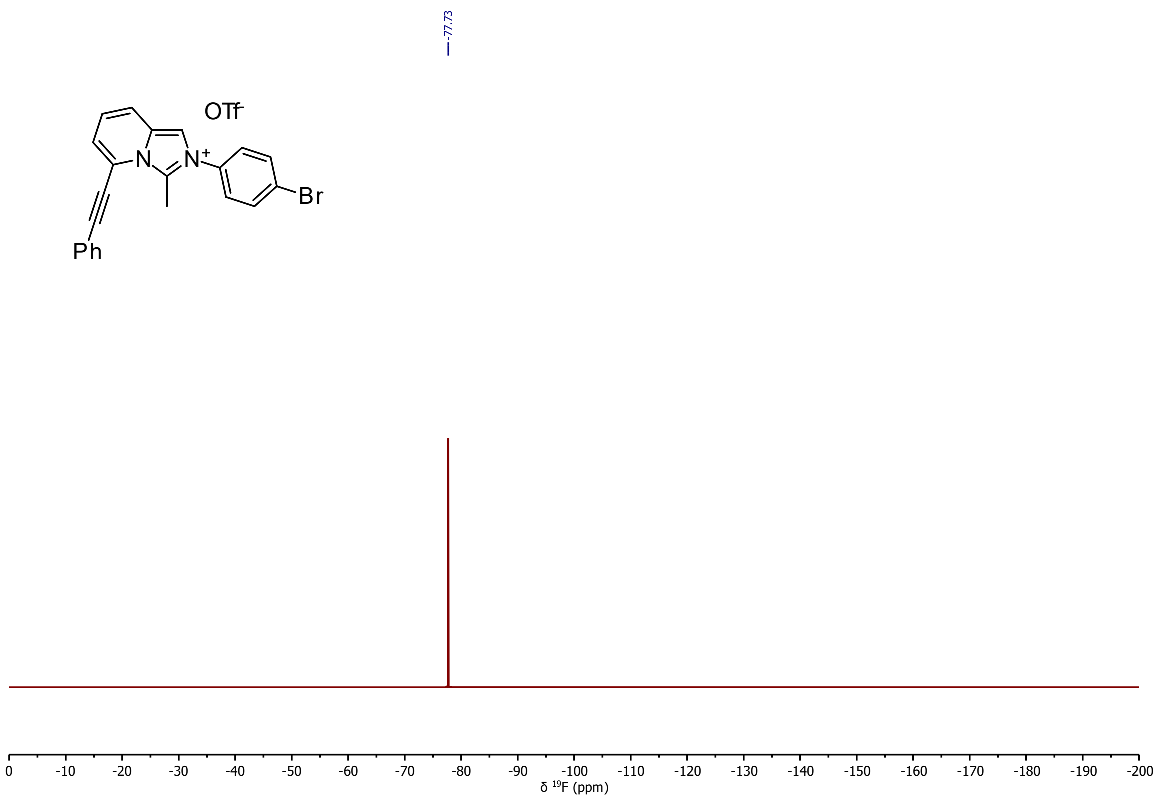

**Figure S207**: **$^{19}$F NMR** (565 MHz, DMSO-d$_6$, 298 K) of **4l**.

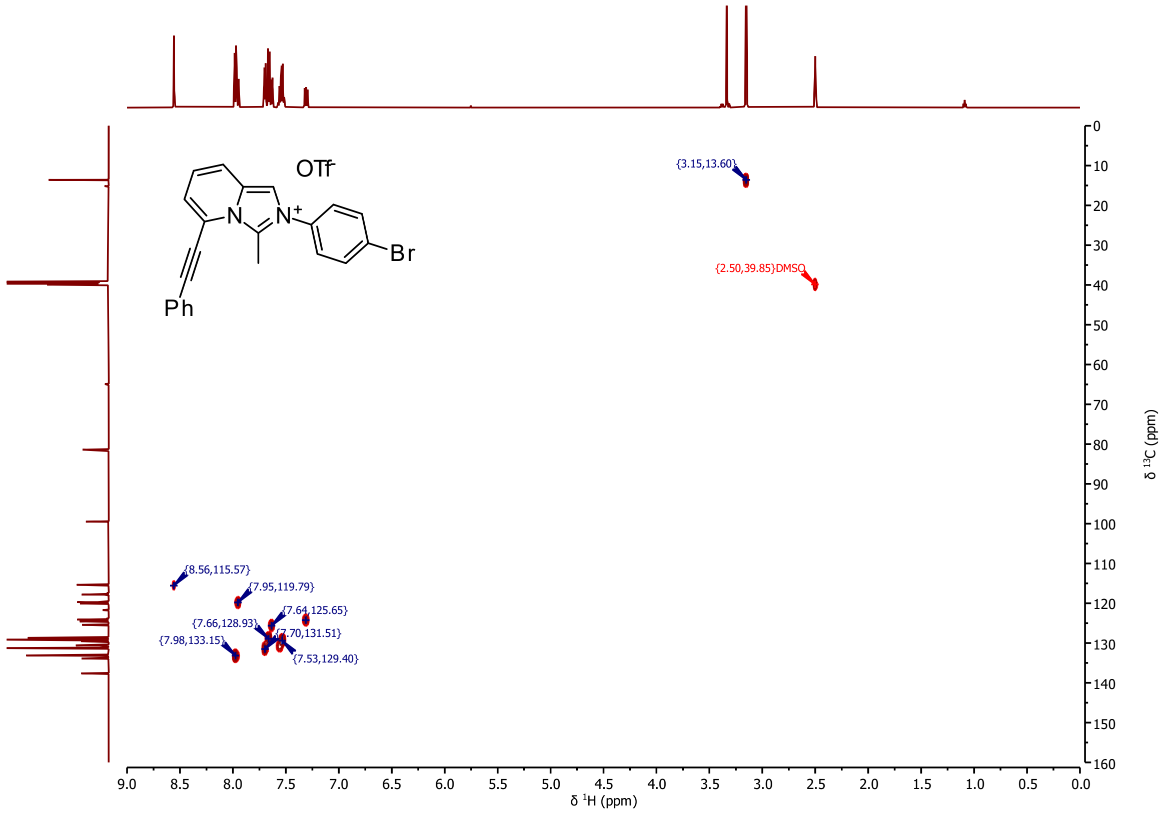

**Figure S208**: **$^1$H/$^{13}$C HSQC** (600/151 MHz, DMSO-d$_6$, 298 K) of **4l**.

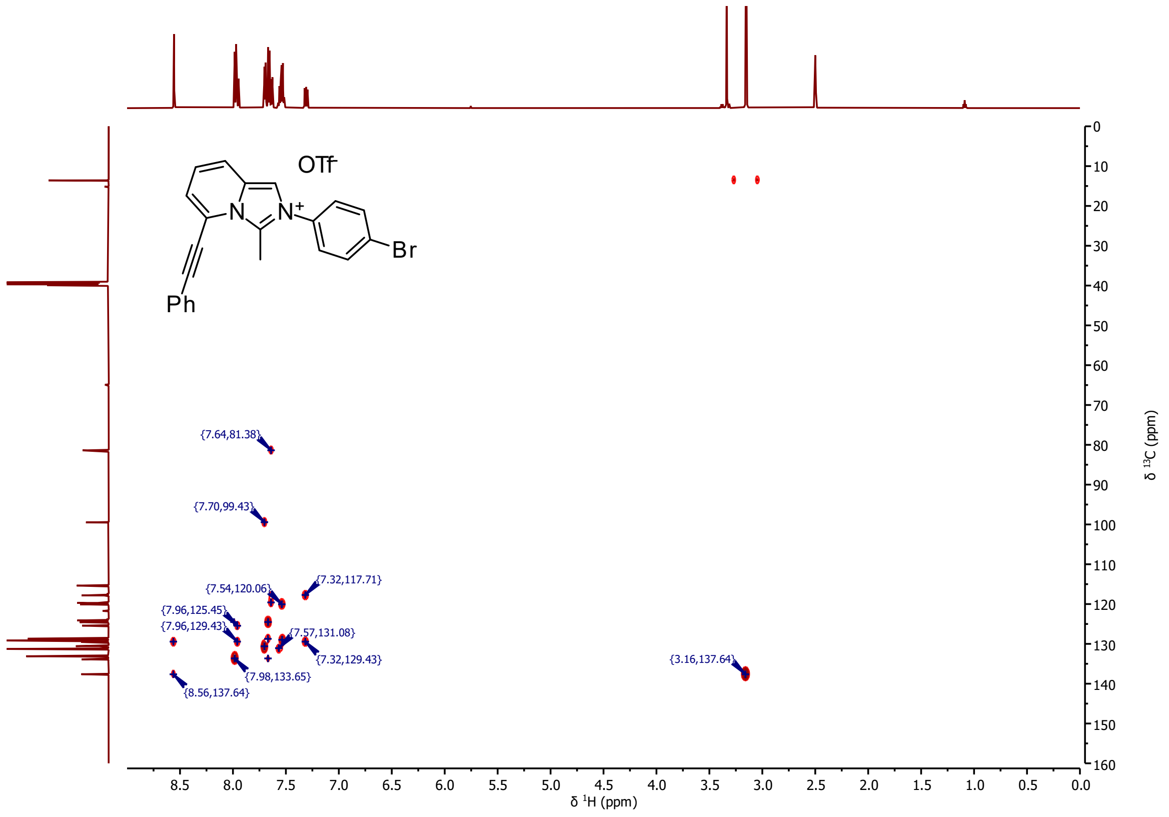

**Figure S209**: **$^1$H/$^{13}$C HMBC** (600/151 MHz, DMSO-d$_6$, 298 K) of **4l**.

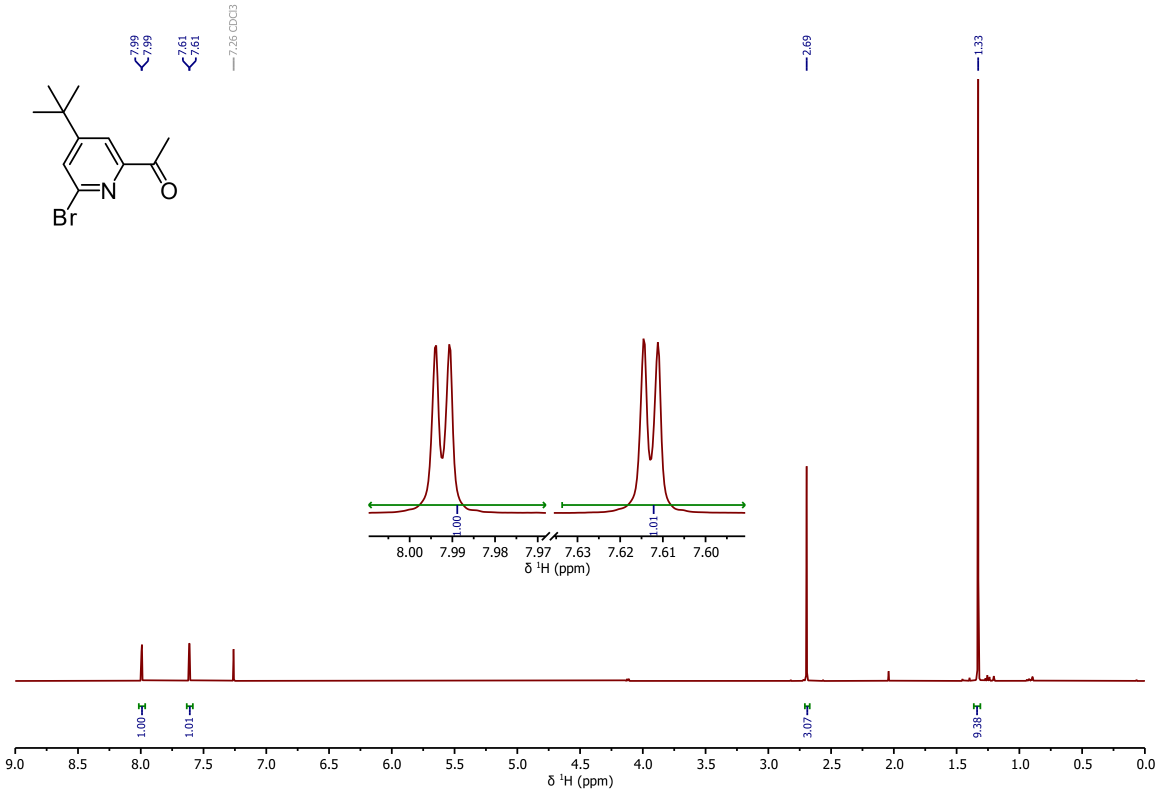

**Figure S210**: **$^1$H NMR** (500 MHz, CDCl$_3$, 298 K) of **S41**.

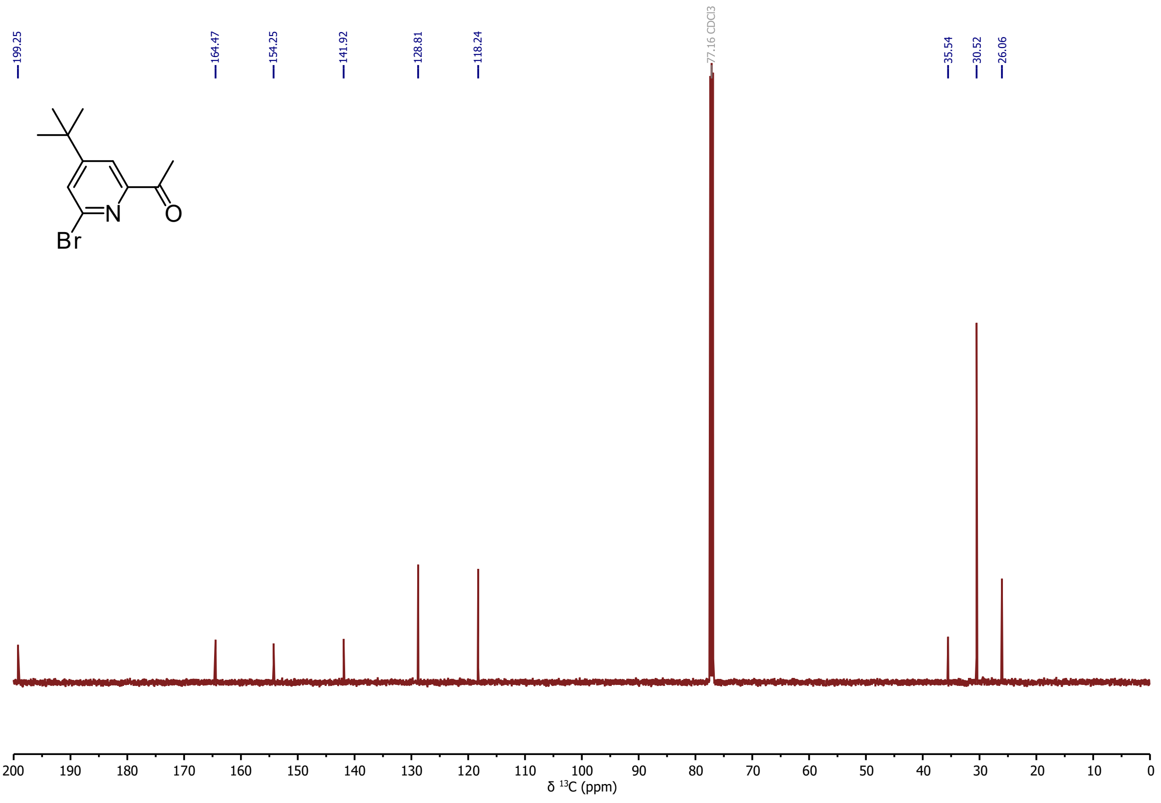


**Figure S211**: **$^{13}$C NMR** (126 MHz, $CDCl_3$, 298 K) of **S41**.

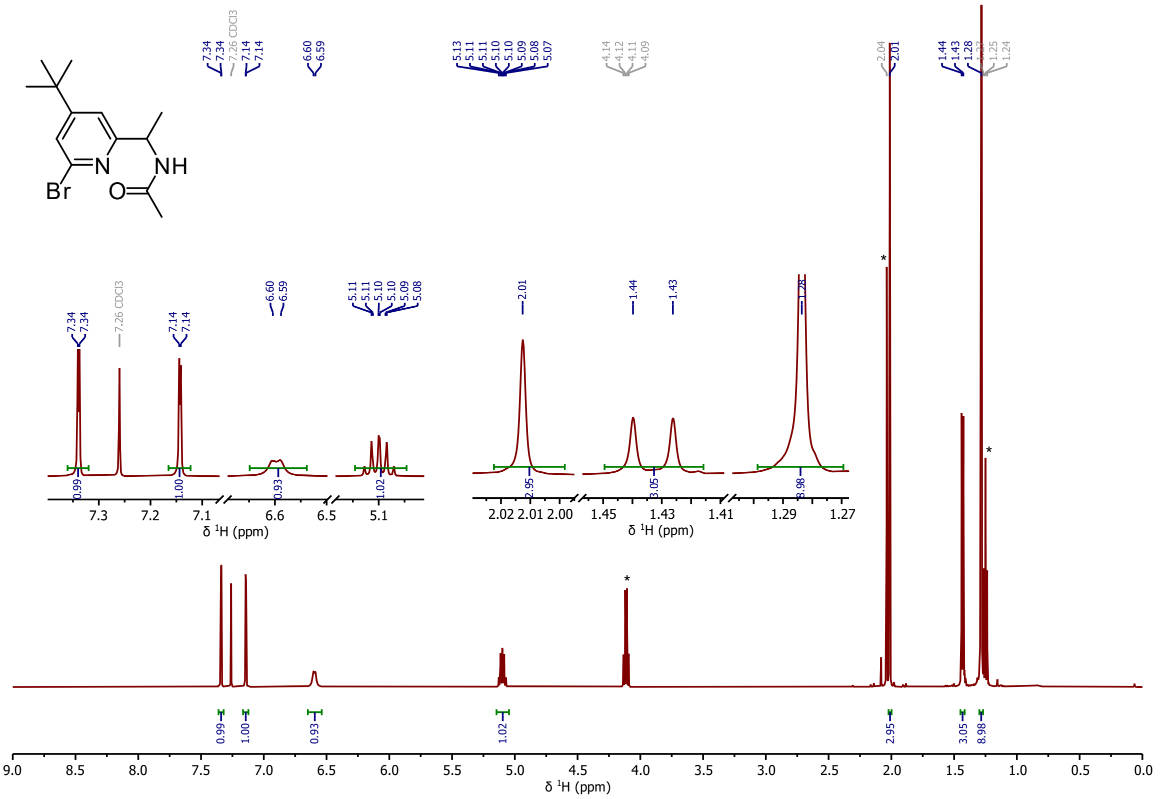


**Figure S212**: **$^{1}$H NMR** (500 MHz, $CDCl_3$, 298 K) of **S42**. * = EtOAc.

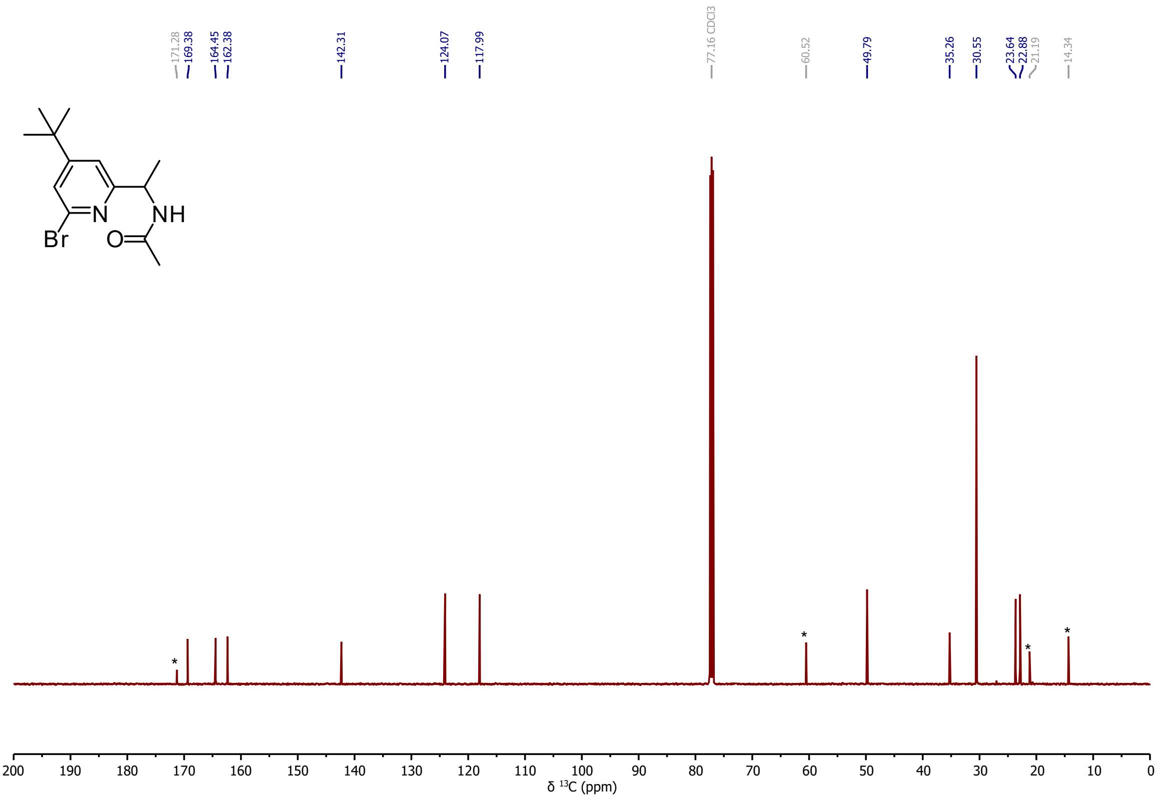

**Figure S213**: **$^{13}$C NMR** (126 MHz, $CDCl_3$, 298 K) of **S42**. * = EtOAc.

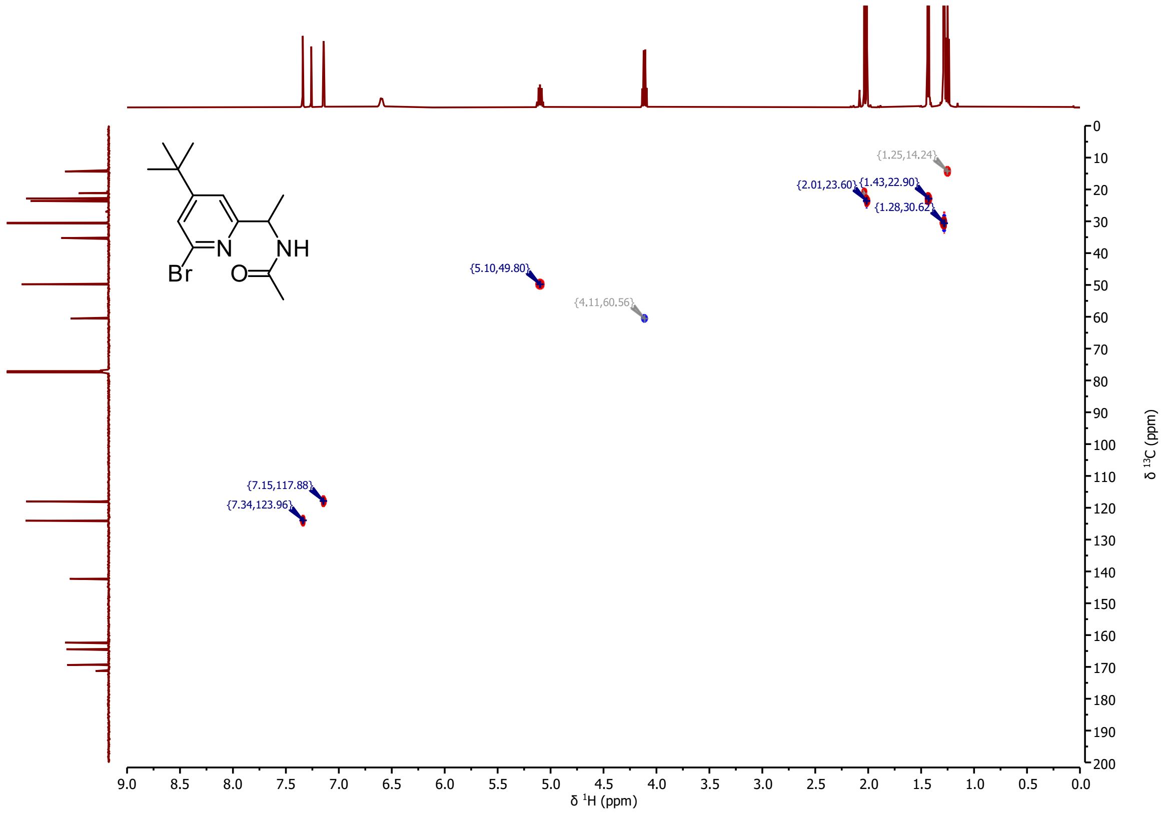

**Figure S214**: **$^{1}$H/$^{13}$C HSQC** (500/126 MHz, $CDCl_3$, 298 K) of **S42**.

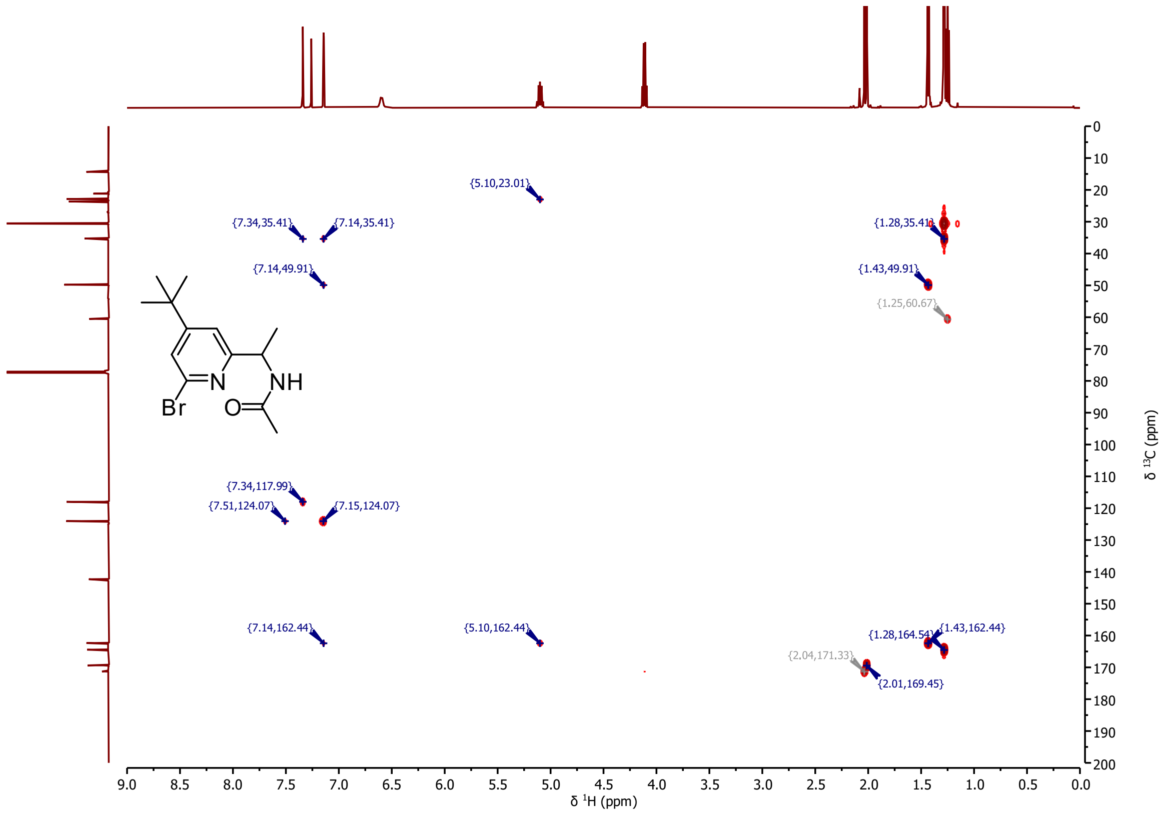

**Figure S215**: **$^1$H/$^{13}$C HMBC** (500/126 MHz, $CDCl_3$, 298 K) of **S42**.

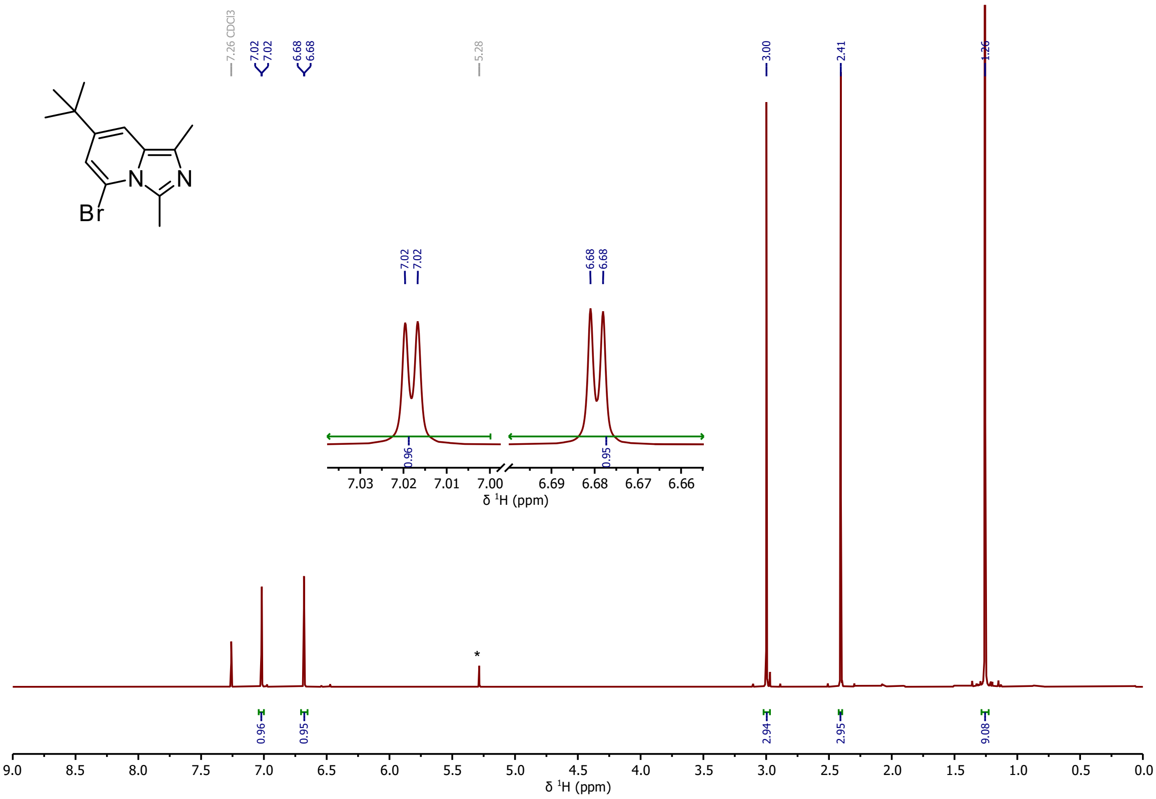

**Figure S216**: **$^1$H NMR** (600 MHz, $CDCl_3$, 298 K) of **S43**. * = $CH_2Cl_2$.

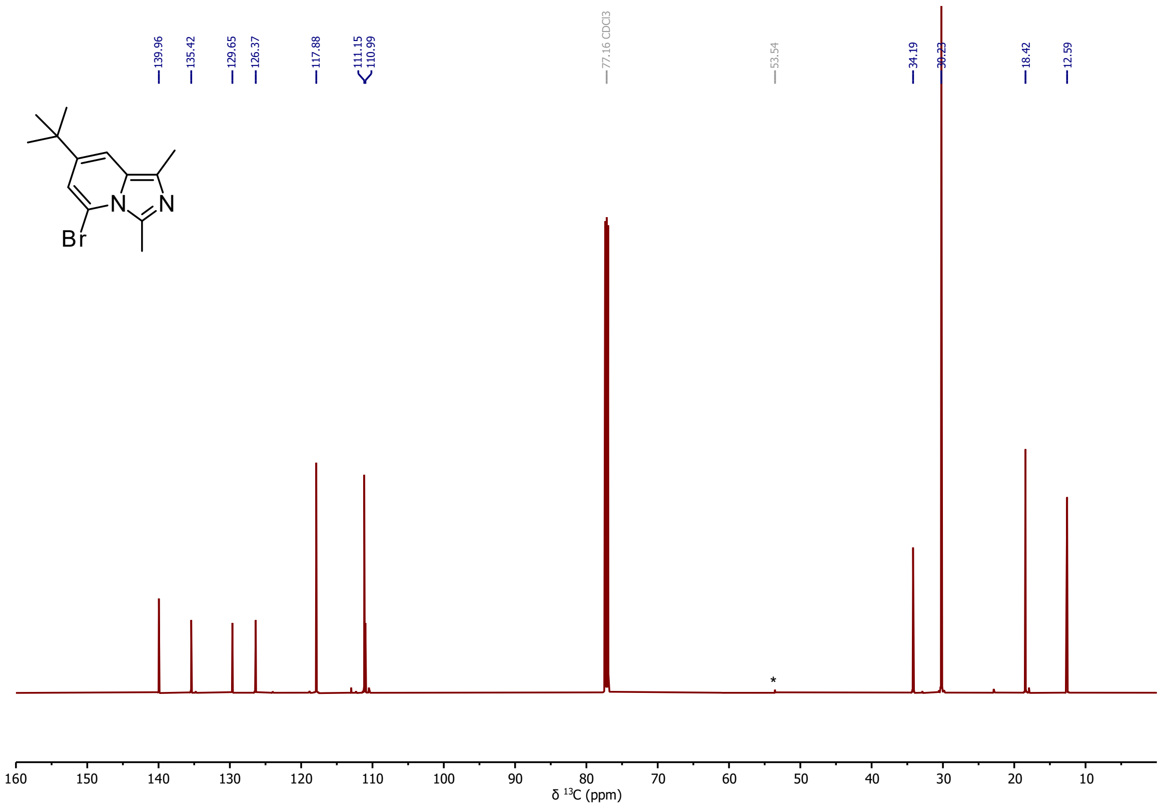


**Figure S217**: **$^{13}$C NMR** (151 MHz, $CDCl_3$, 298 K) of **S43**. * = $CH_2Cl_2$.

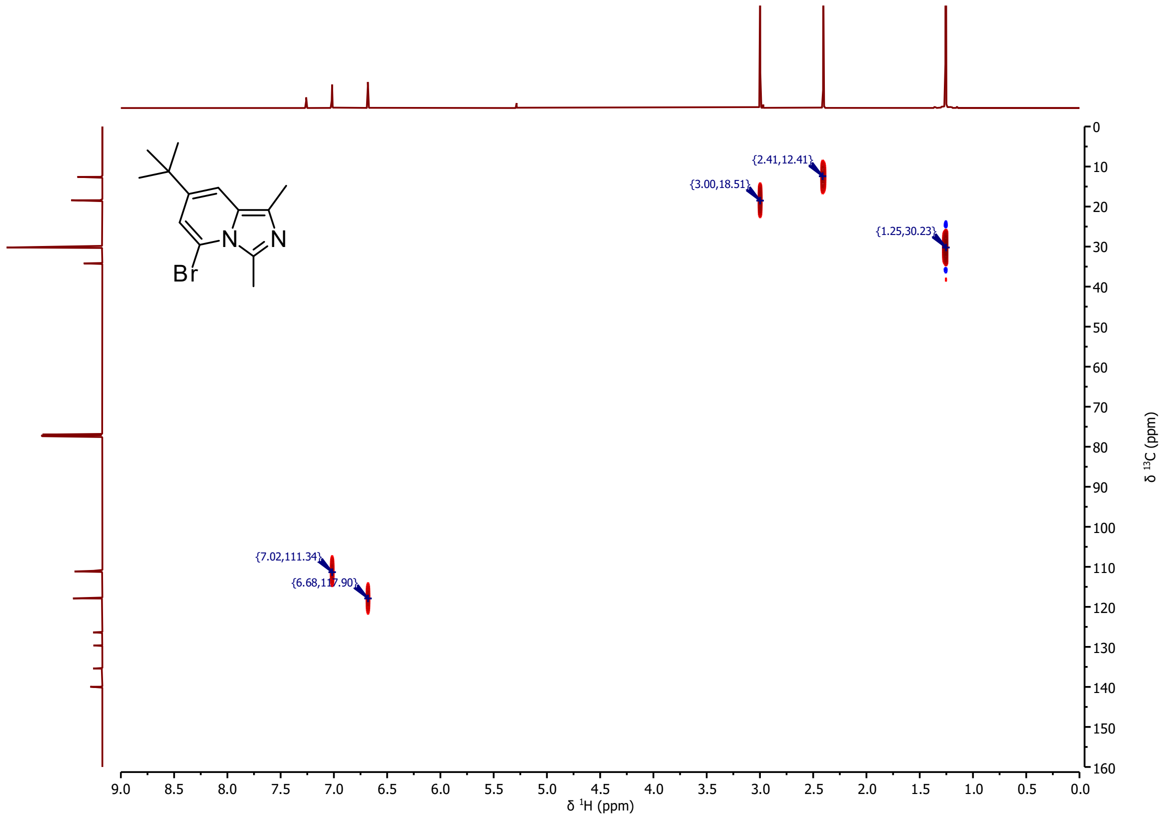


**Figure S218**: **$^{1}$H/$^{13}$C HSQC** (600/151 MHz, $CDCl_3$, 298 K) of **S43**.

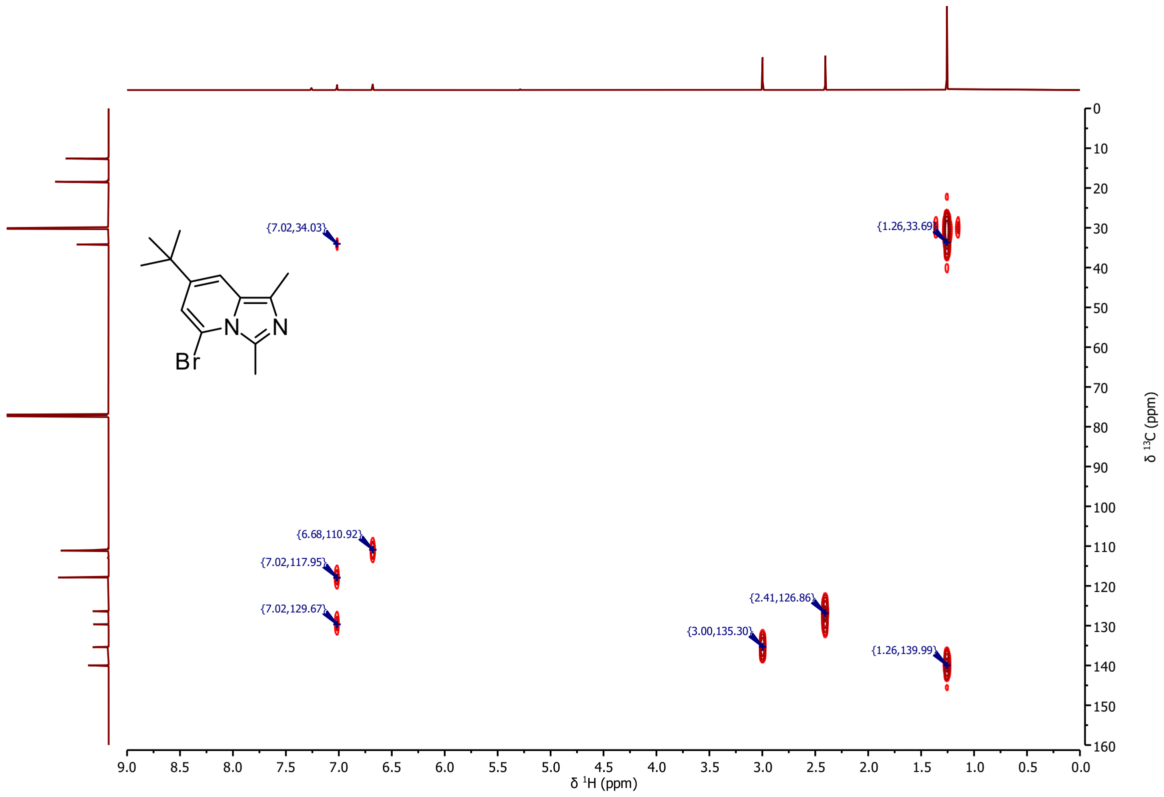

**Figure S219**: **$^1$H/$^{13}$C HMBC** (600/151 MHz, $CDCl_3$, 298 K) of **S43**.

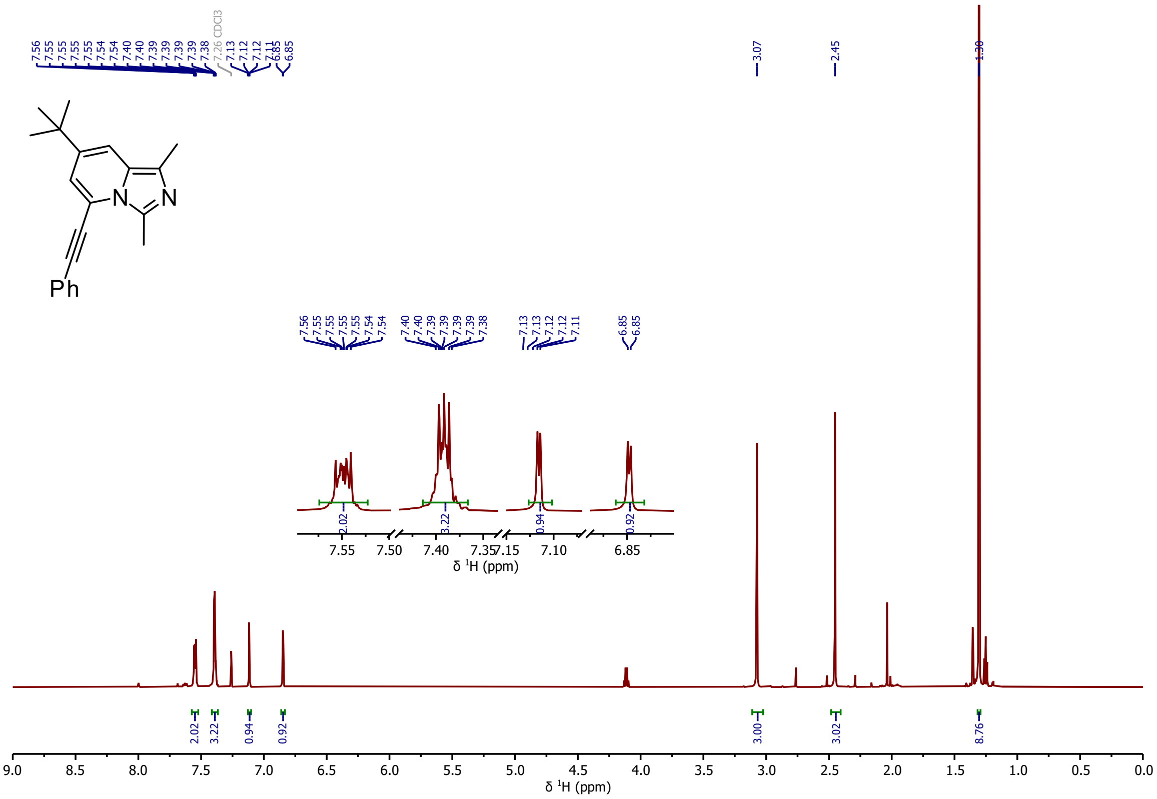

**Figure S220**: **$^1$H NMR** (600 MHz, $CDCl_3$, 298 K) of **S44**.

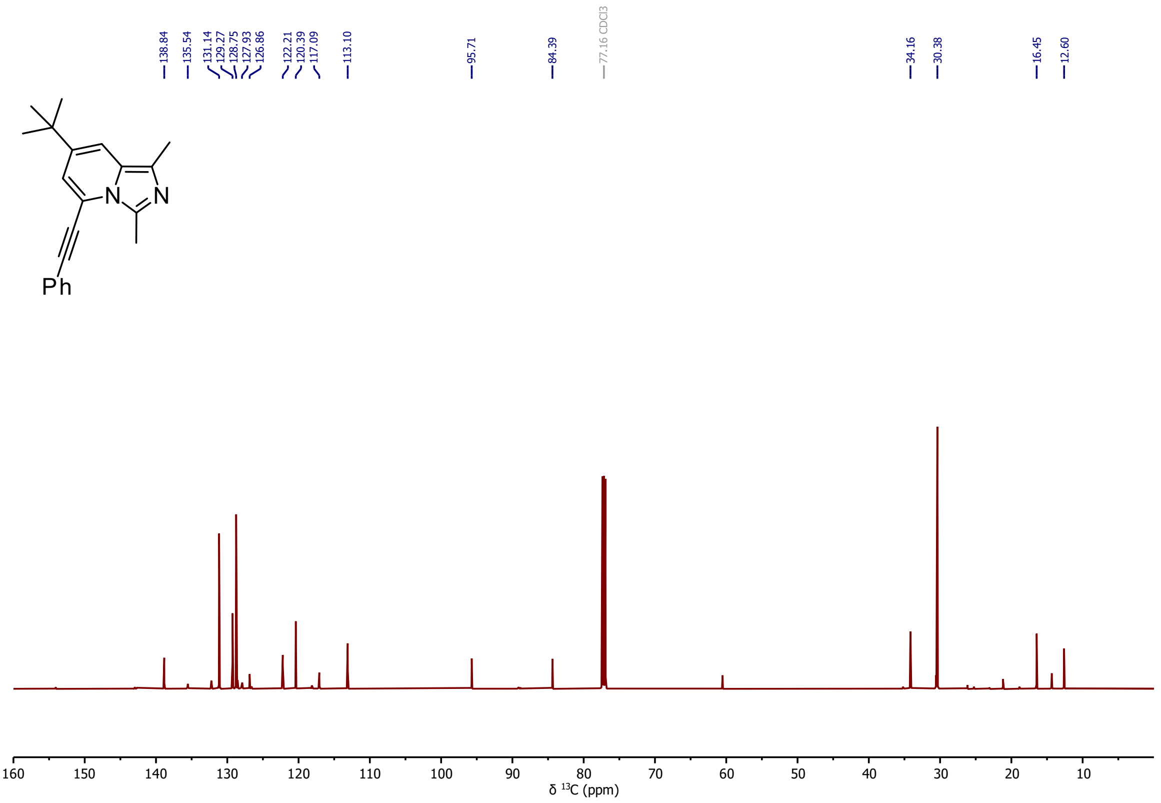


**Figure S221**: **$^{13}$C NMR** (151 MHz, $CDCl_3$, 298 K) of **S44**.

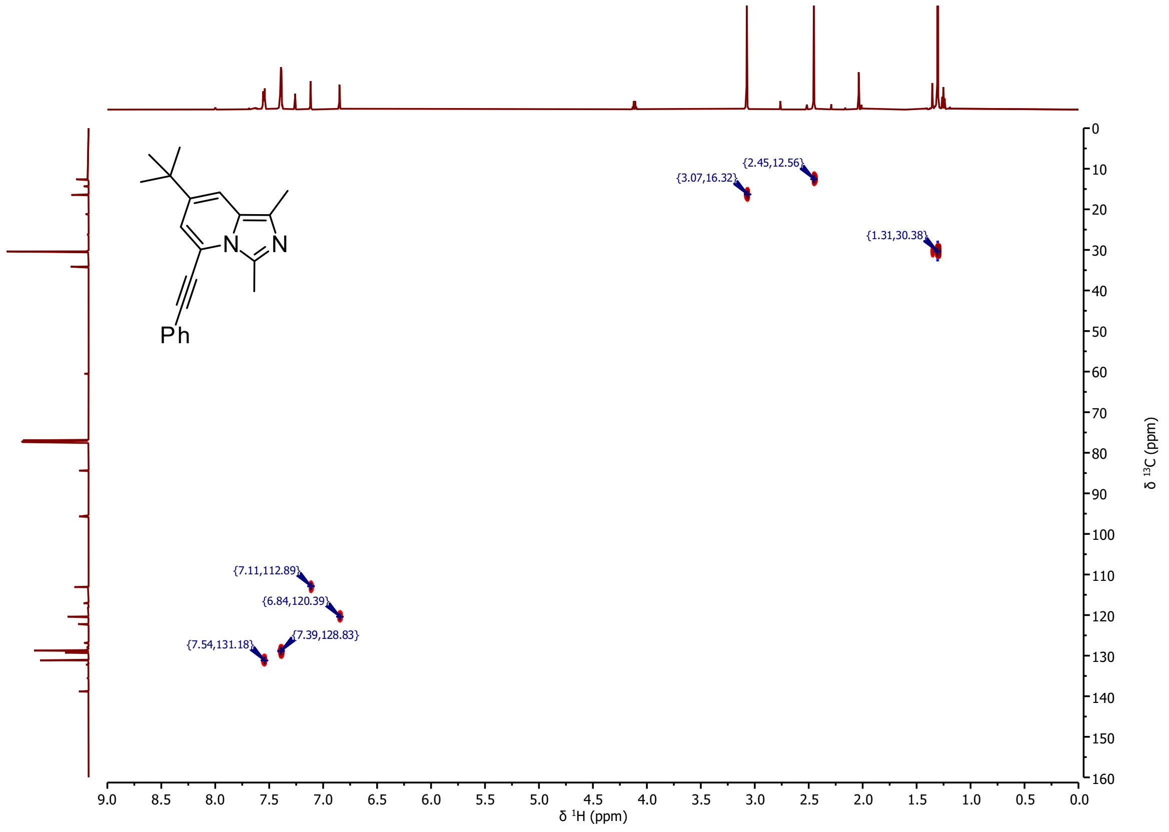


**Figure S222**: **$^{1}$H/$^{13}$C HSQC** (600/151 MHz, $CDCl_3$, 298 K) of **S44**.

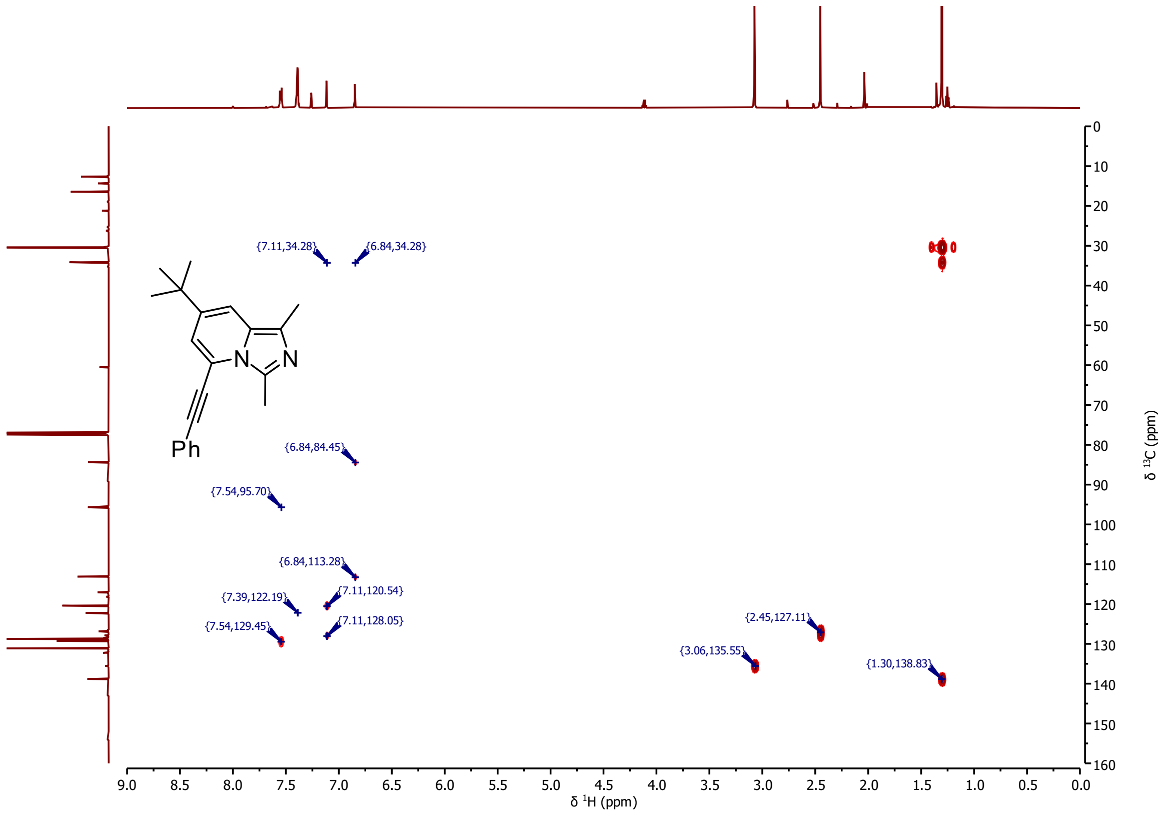

**Figure S223**: **$^1$H/$^{13}$C HMBC** (600/151 MHz, $CDCl_3$, 298 K) of **S44**.

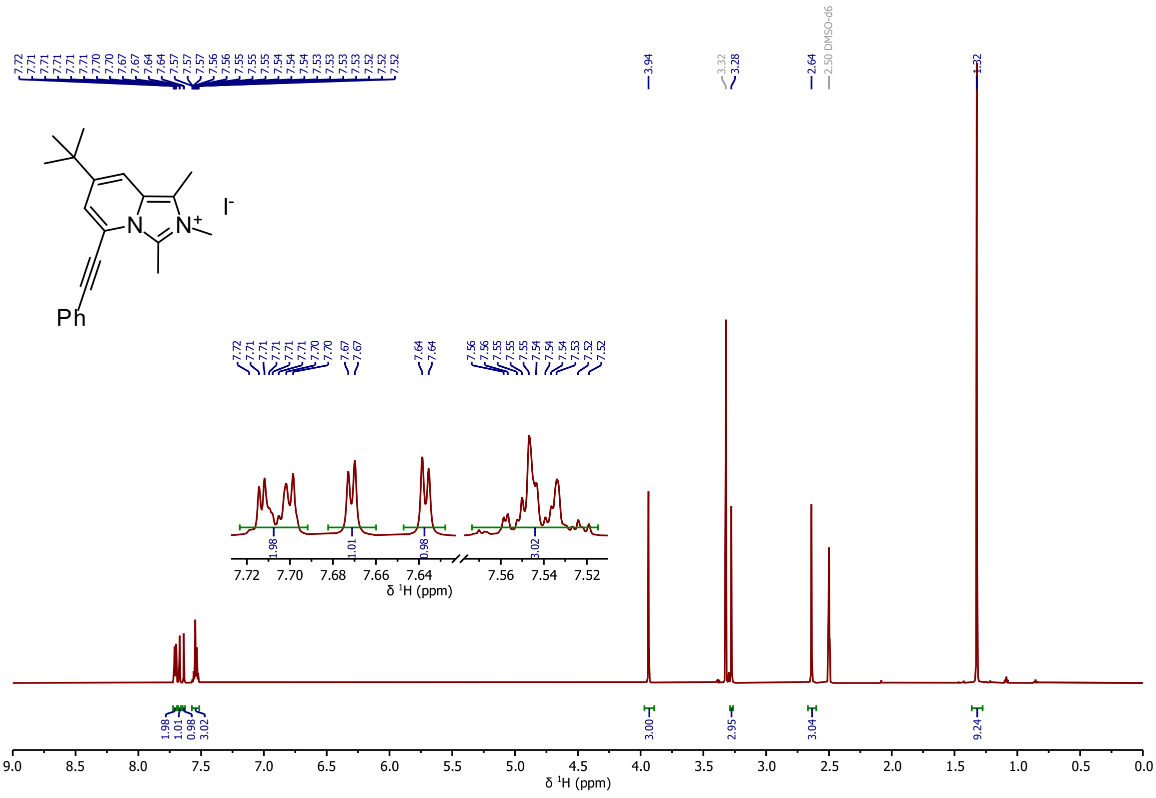

**Figure S224**: **$^1$H NMR** (600 MHz, DMSO-$d_6$, 298 K) of **4m**.

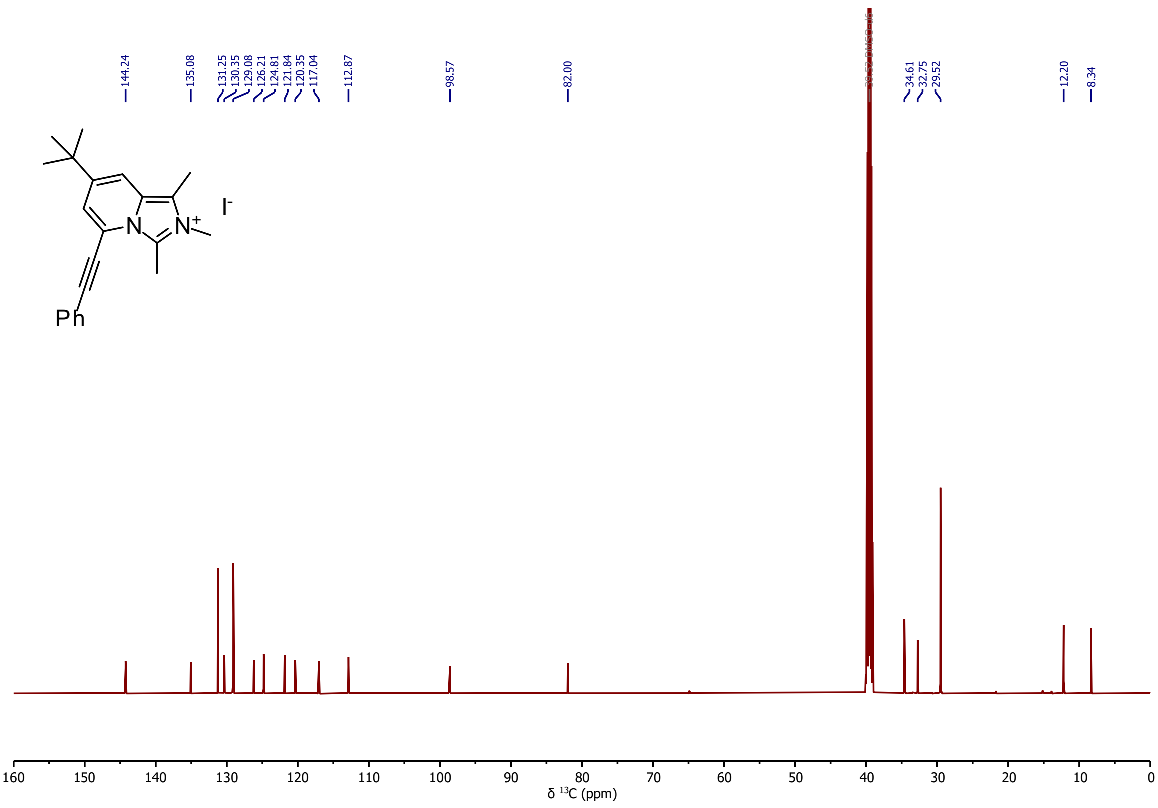

**Figure S225**: **$^{13}$C NMR** (151 MHz, DMSO-d$_6$, 298 K) of **4m**.

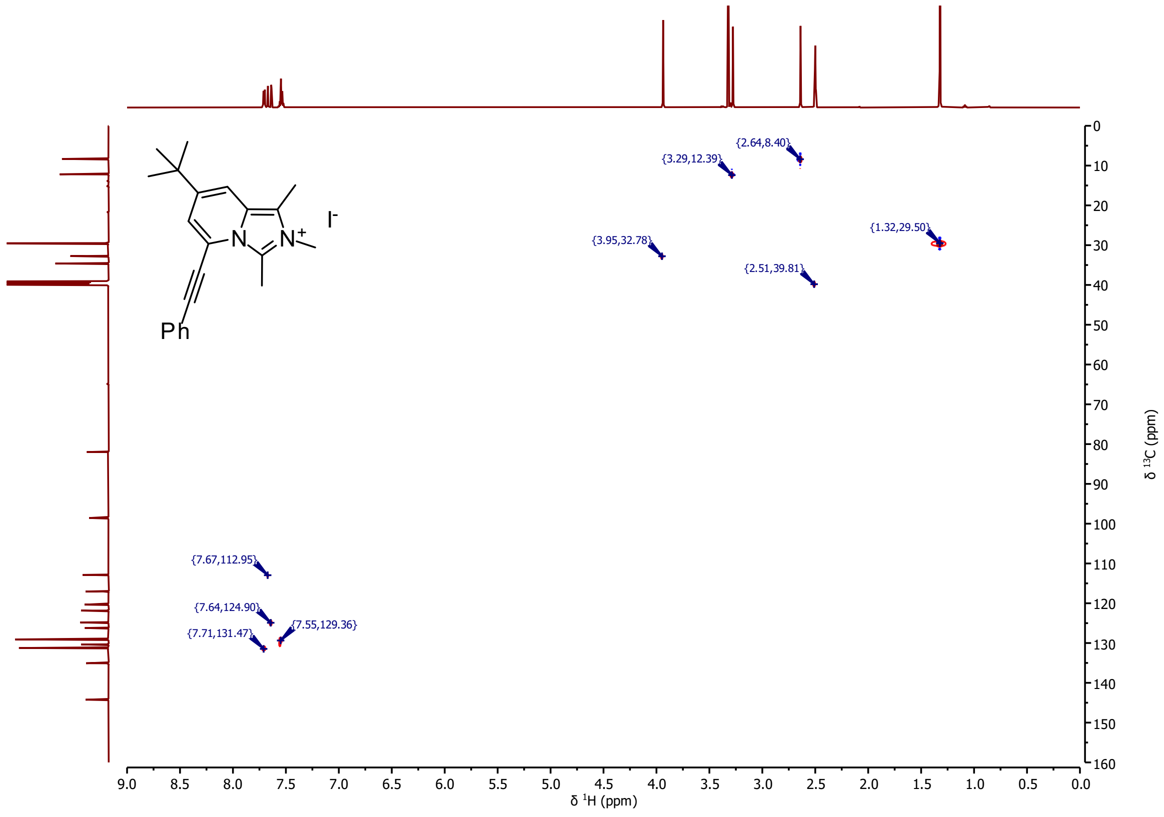

**Figure S226**: **$^{1}$H/$^{13}$C HSQC** (600/151 MHz, DMSO-d$_6$, 298 K) of **4m**.

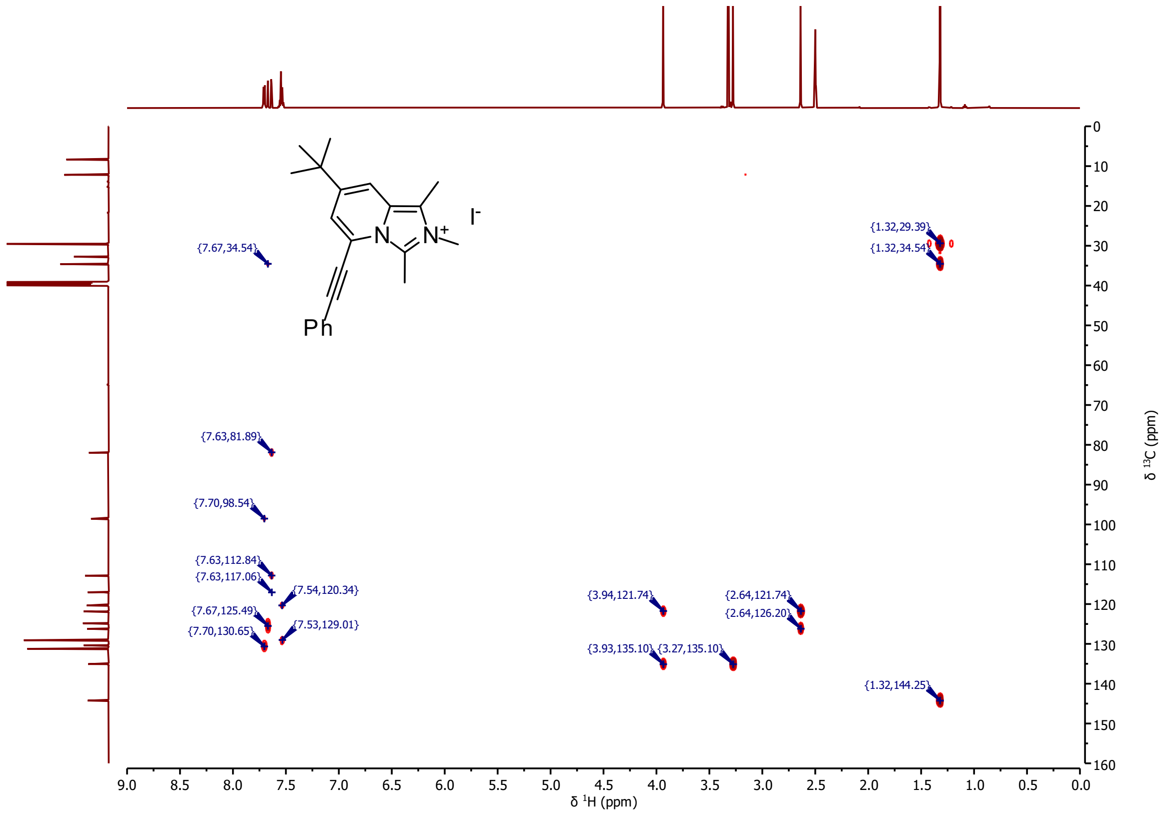

**Figure S227**: **$^{1}$H/$^{13}$C HMBC** (600/151 MHz, DMSO-$d_6$, 298 K) of **4m**.

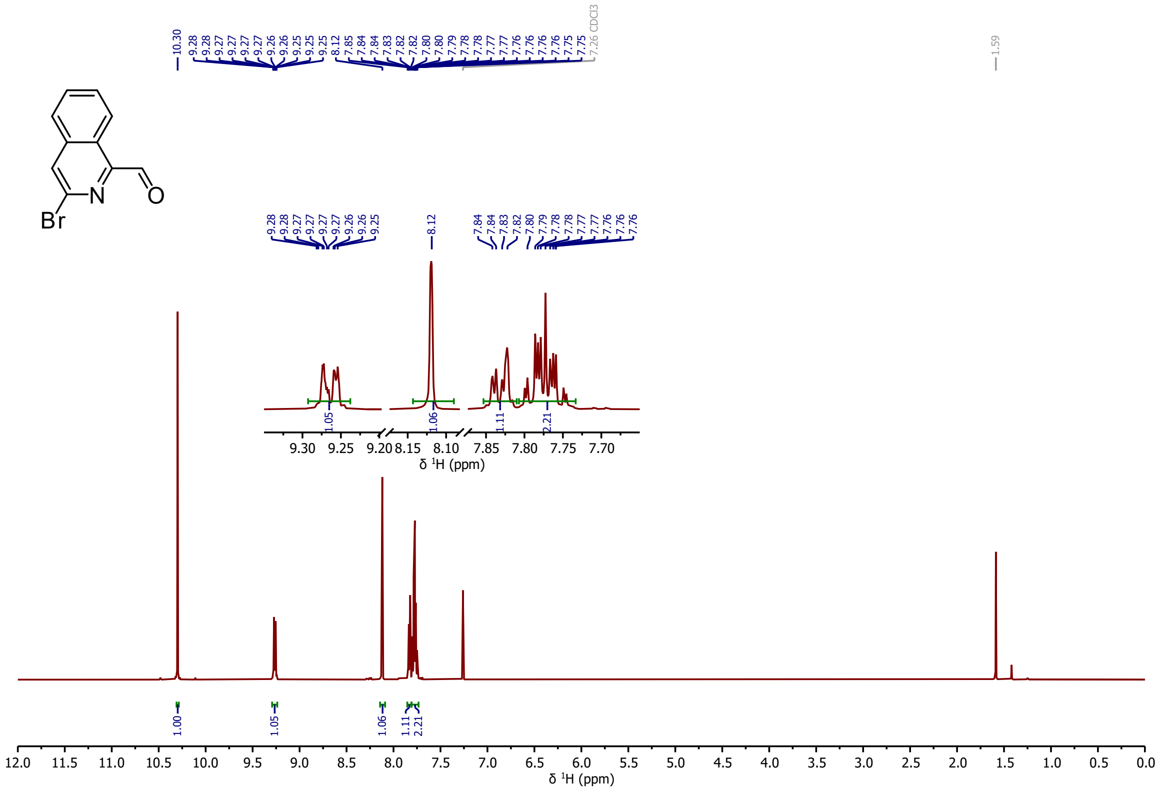

**Figure S228**: **$^{1}$H NMR** (500 MHz, $CDCl_3$, 298 K) of **S46**.

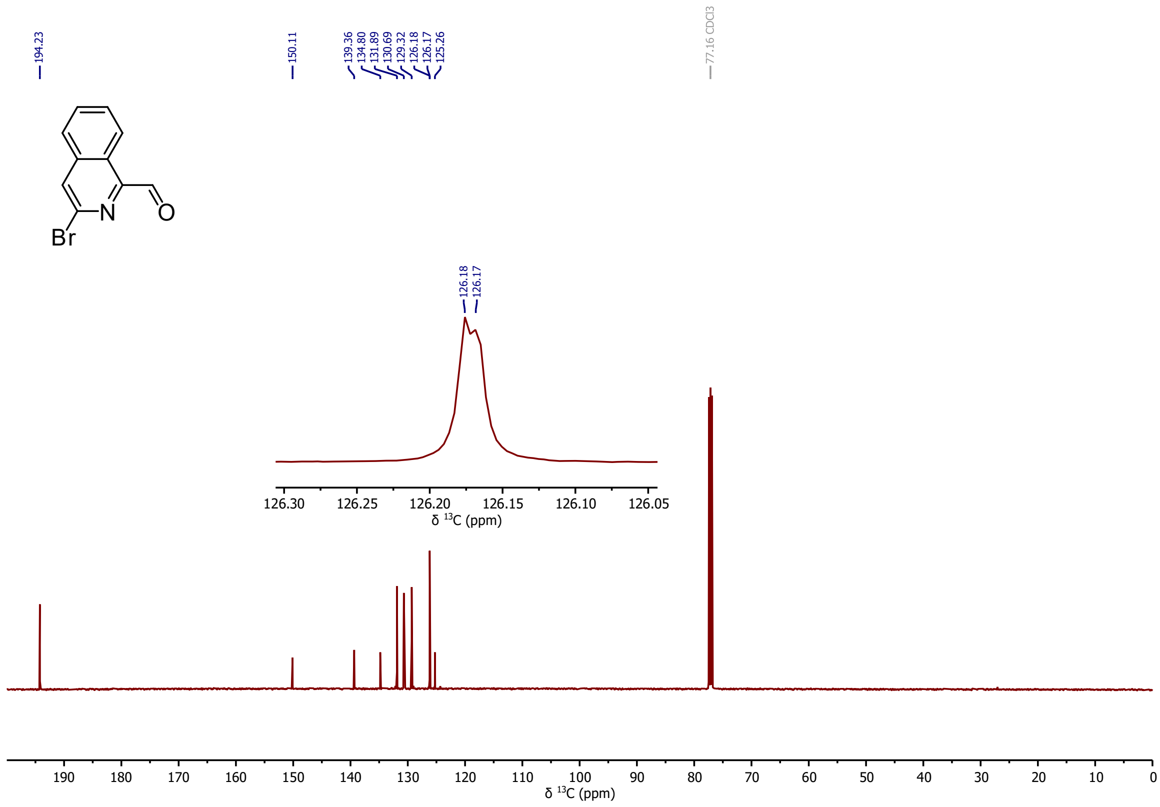


**Figure S229**: $^{13}$**C NMR** (126 MHz, $CDCl_3$, 298 K) of **S46**.

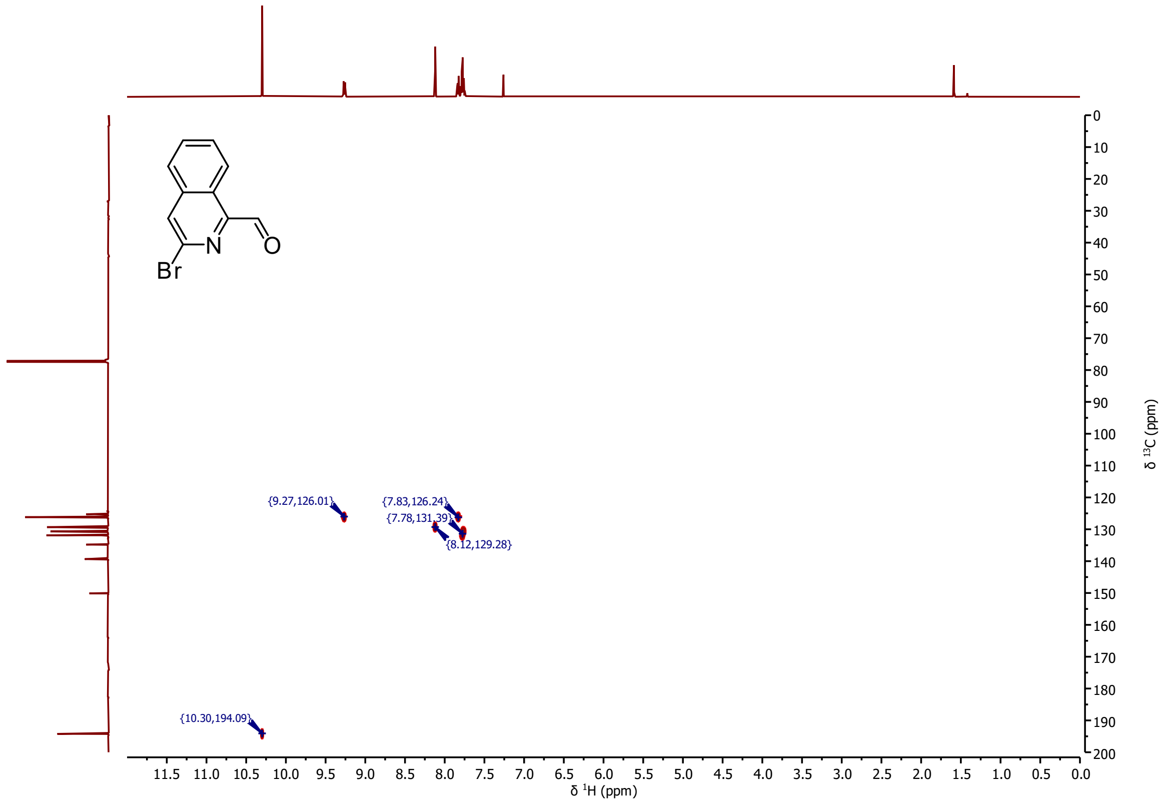


**Figure S230**: $^{1}$**H/**$^{13}$**C HSQC** (500/126 MHz, $CDCl_3$, 298 K) of **S46**.

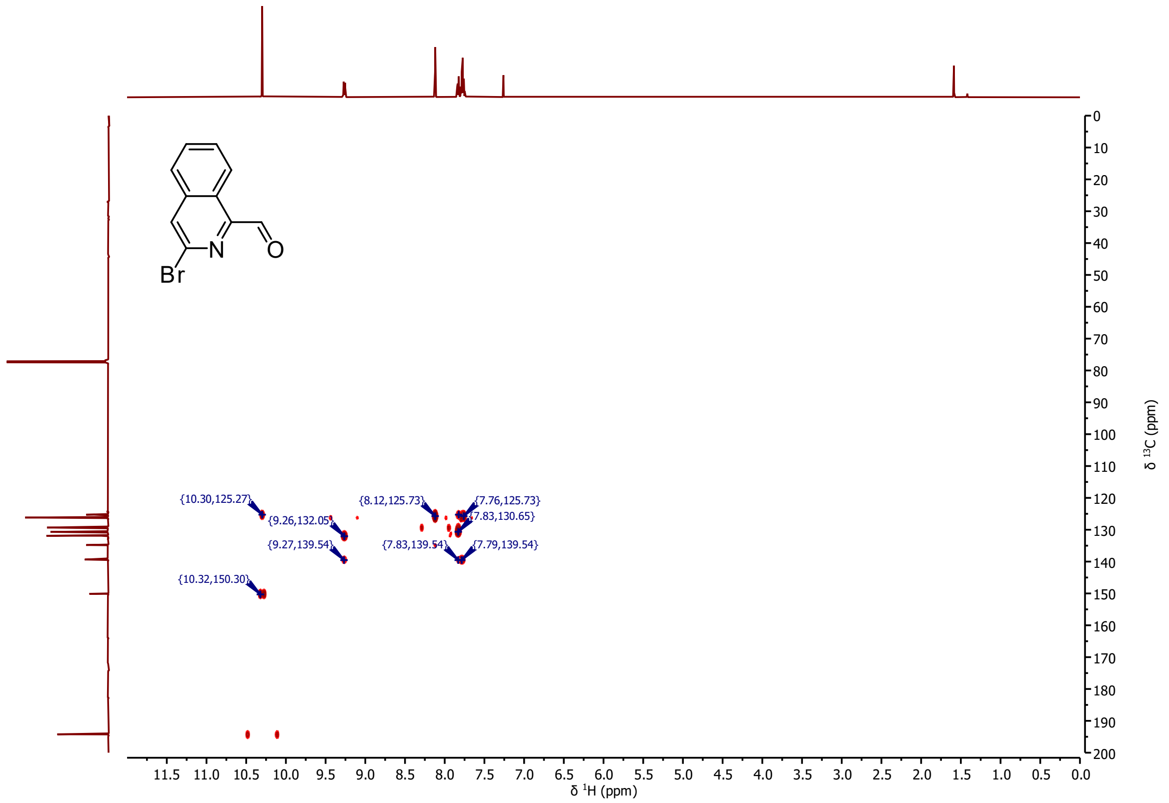

**Figure S231**: **$^1$H/$^{13}$C HMBC** (500/126 MHz, $CDCl_3$, 298 K) of **S46**.

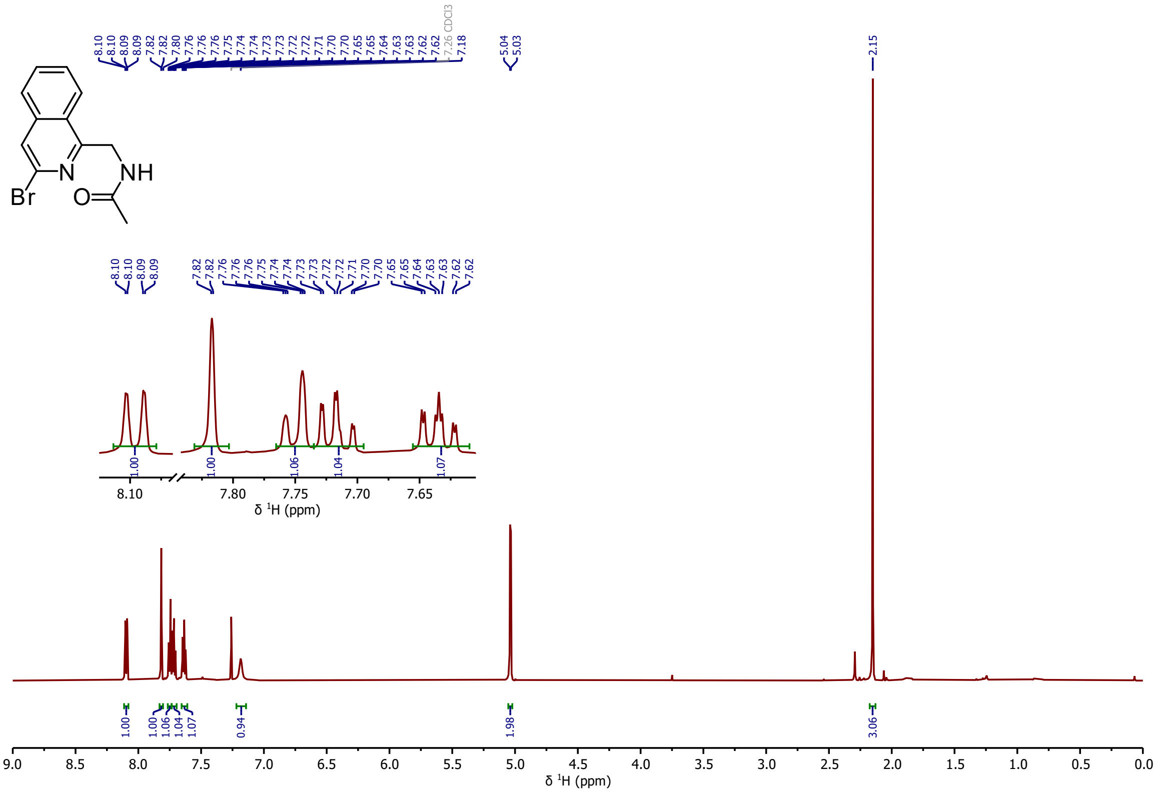

**Figure S232**: **$^1$H NMR** (600 MHz, $CDCl_3$, 298 K) of **S47**.

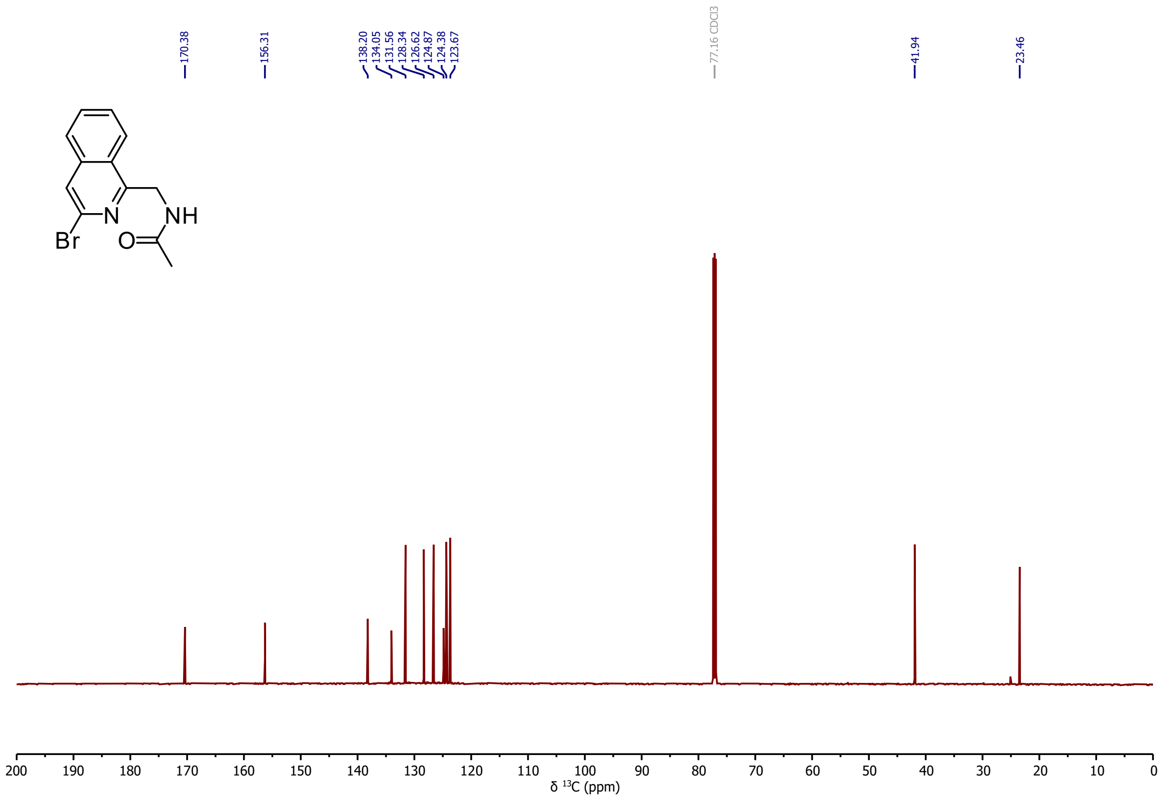


**Figure S233**: **$^{13}$C NMR** (151 MHz, $CDCl_3$, 298 K) of **S47**.

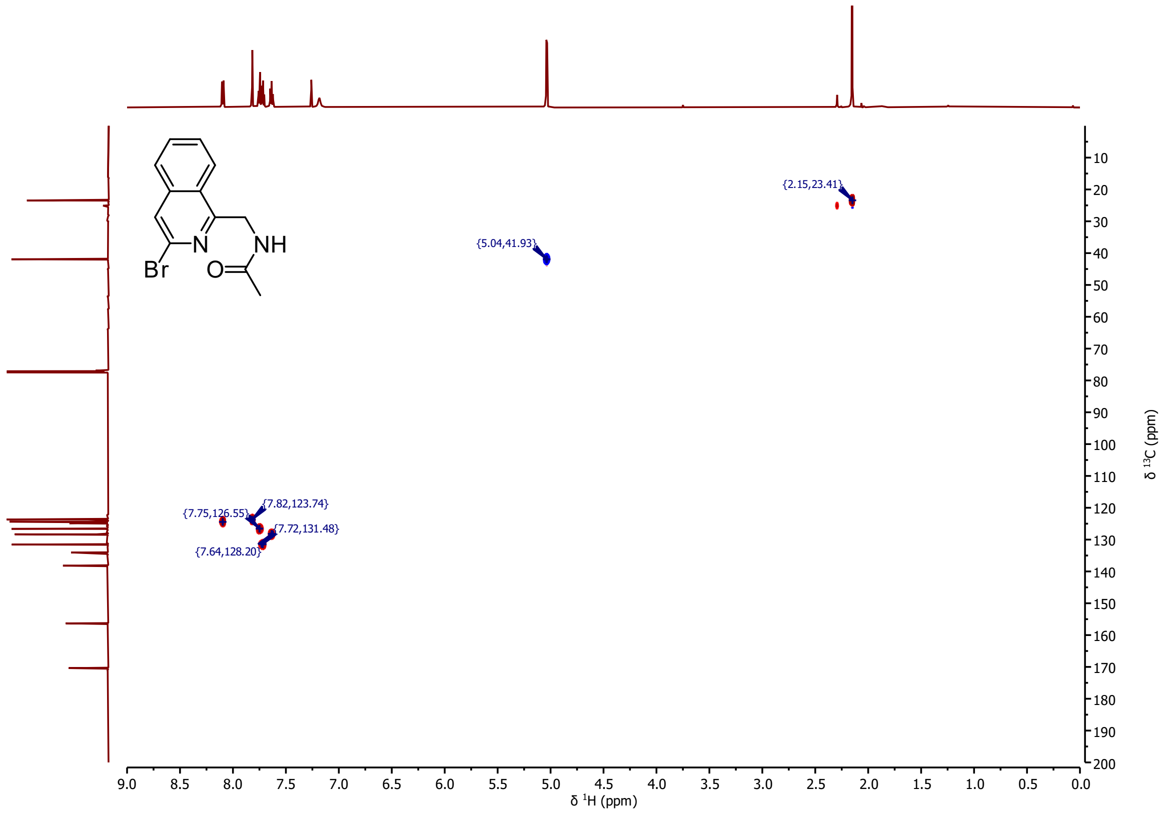


**Figure S234**: **$^{1}$H/$^{13}$C HSQC** (600/151 MHz, $CDCl_3$, 298 K) of **S47**.

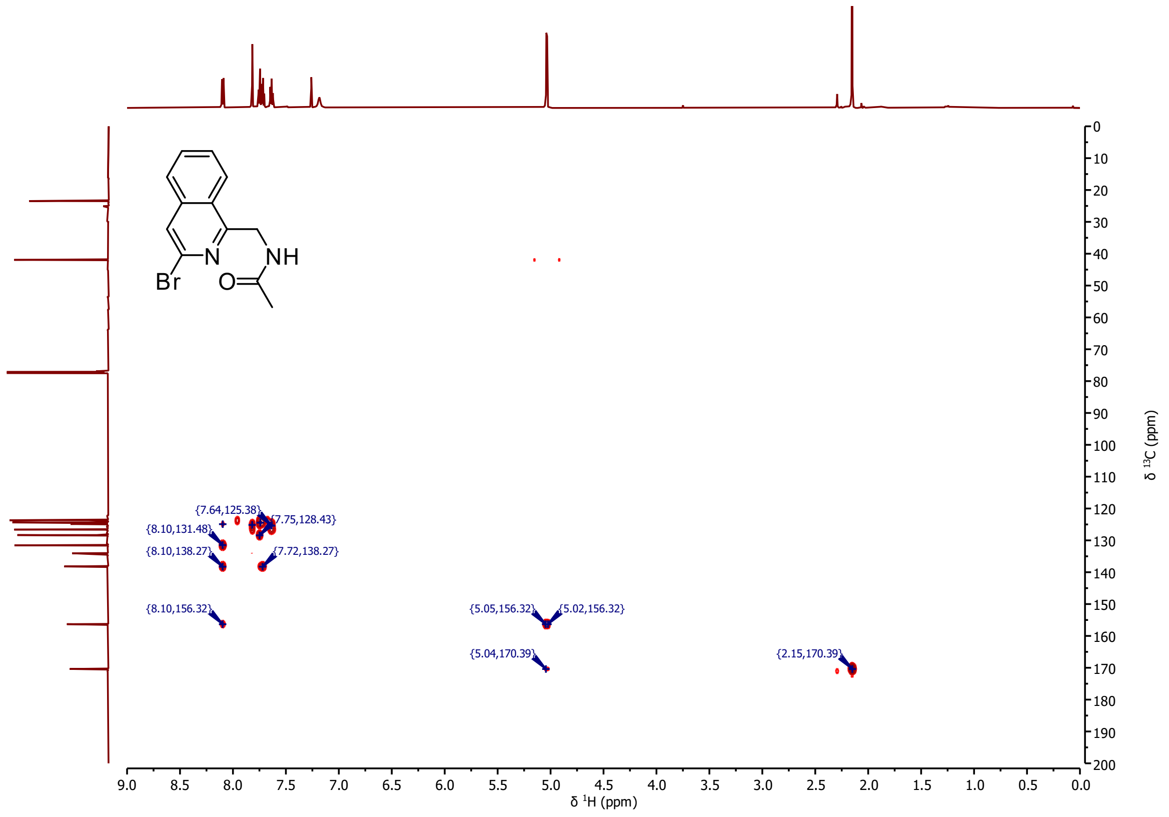


**Figure S235**: **$^{1}$H/$^{13}$C HMBC** (600/151 MHz, $CDCl_3$, 298 K) of **S47**.

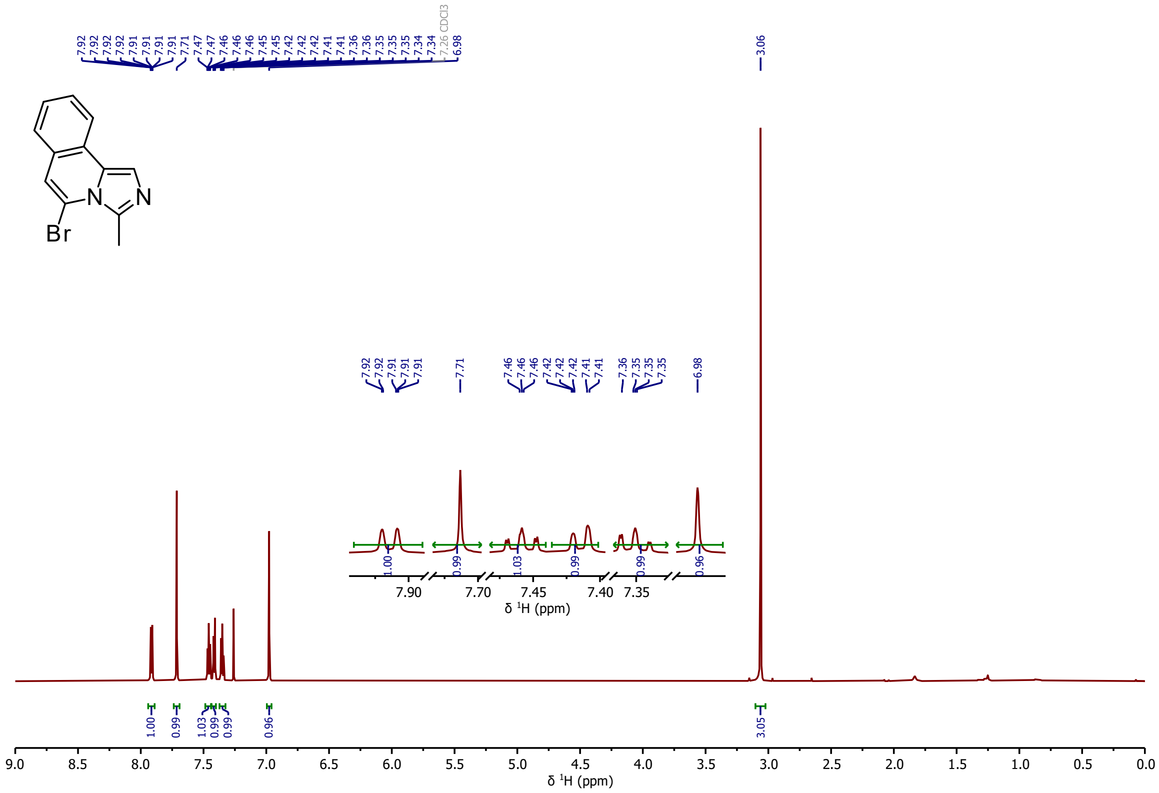


**Figure S236**: **$^{1}$H NMR** (700 MHz, $CDCl_3$, 298 K) of **S48**.

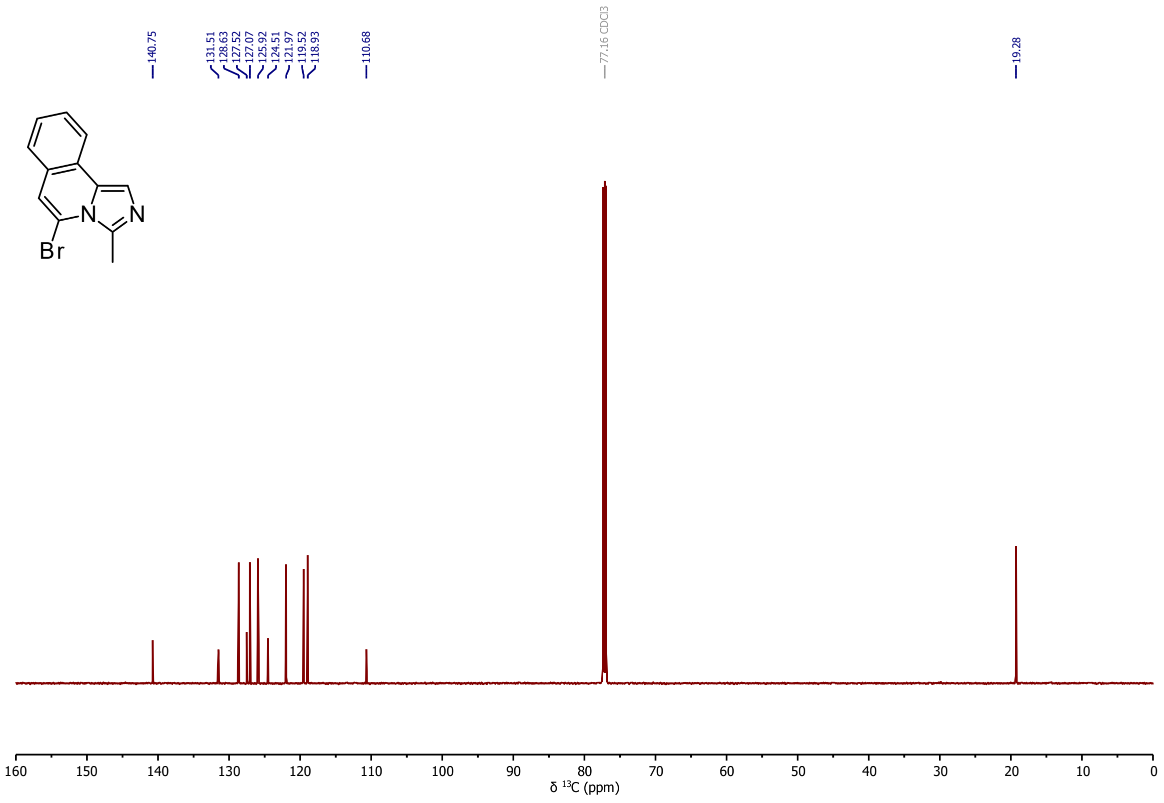

**Figure S237**: **$^{13}$C NMR** (176 MHz, $CDCl_3$, 298 K) of **S48**.

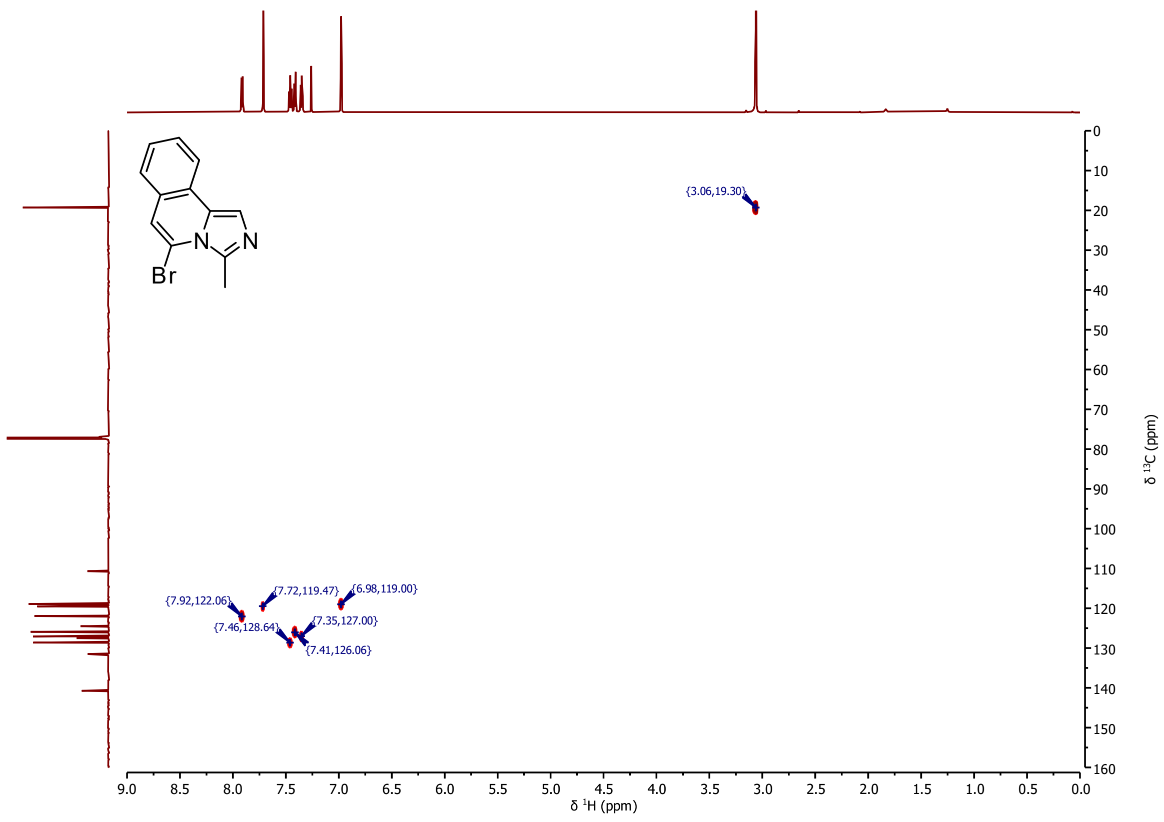

**Figure S238**: **$^{1}$H/$^{13}$C HSQC** (700/176 MHz, $CDCl_3$, 298 K) of **S48**.

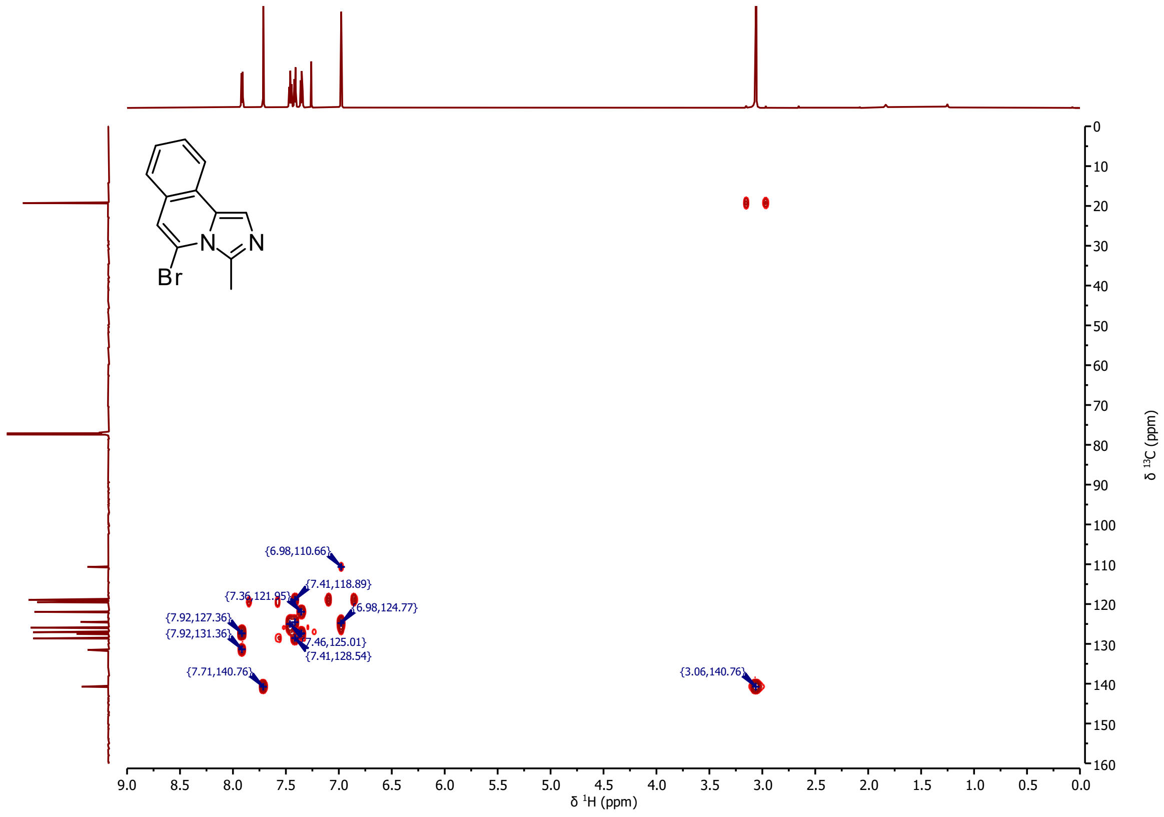

**Figure S239**: **$^1$H/$^{13}$C HMBC** (700/176 MHz, $CDCl_3$, 298 K) of **S48**.

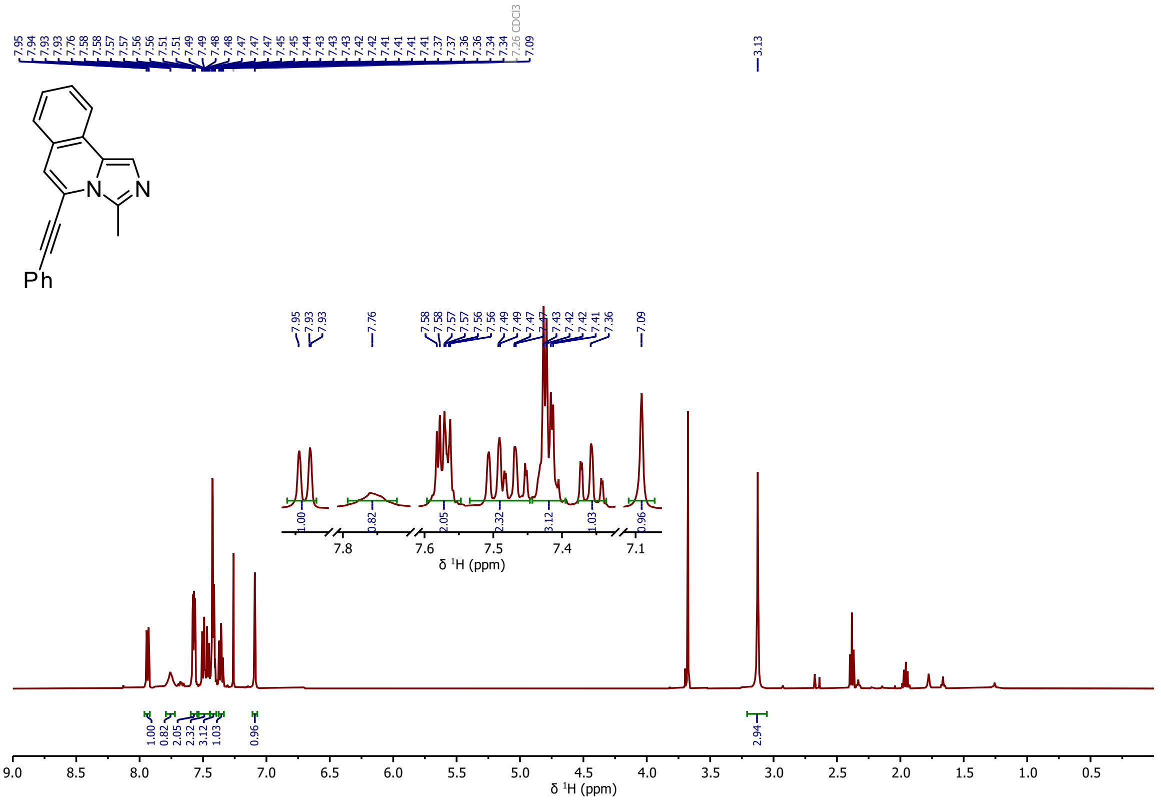

**Figure S240**: **$^1$H NMR** (500 MHz, $CDCl_3$, 298 K) of **S49**.

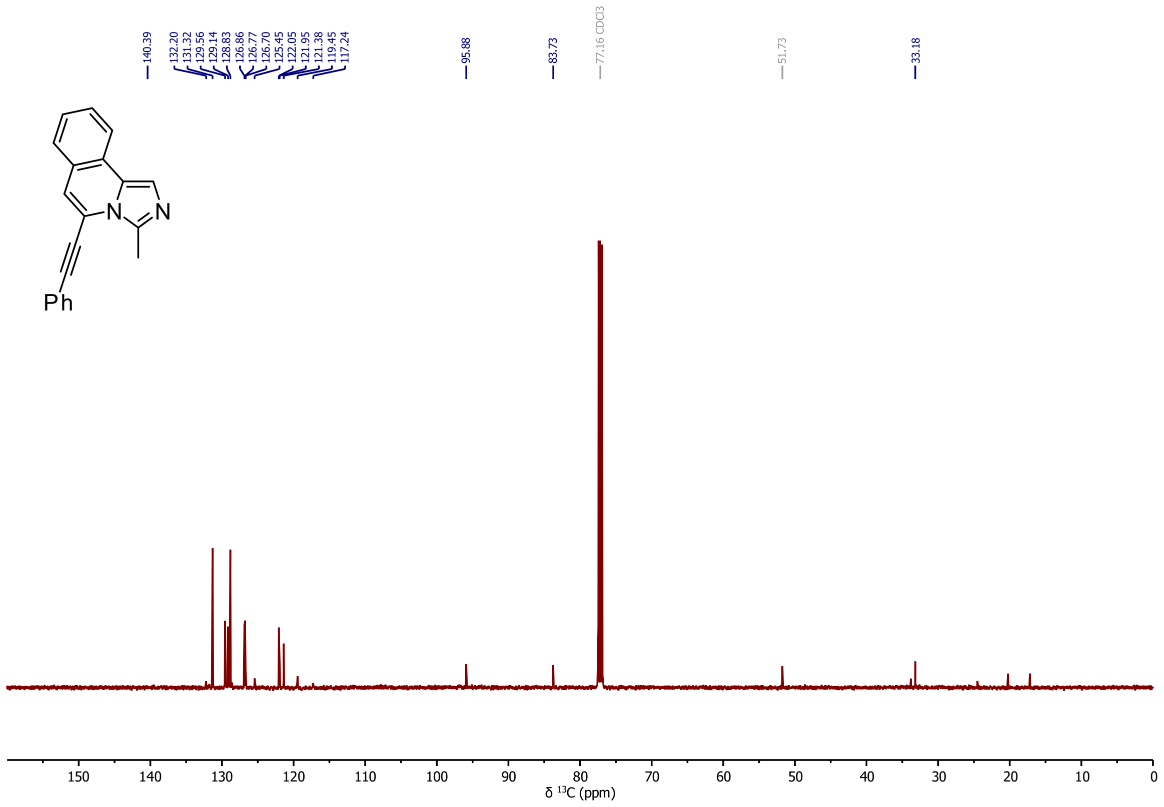

**Figure S241**: **$^{13}$C NMR** (126 MHz, $CDCl_3$, 298 K) of **S49**.

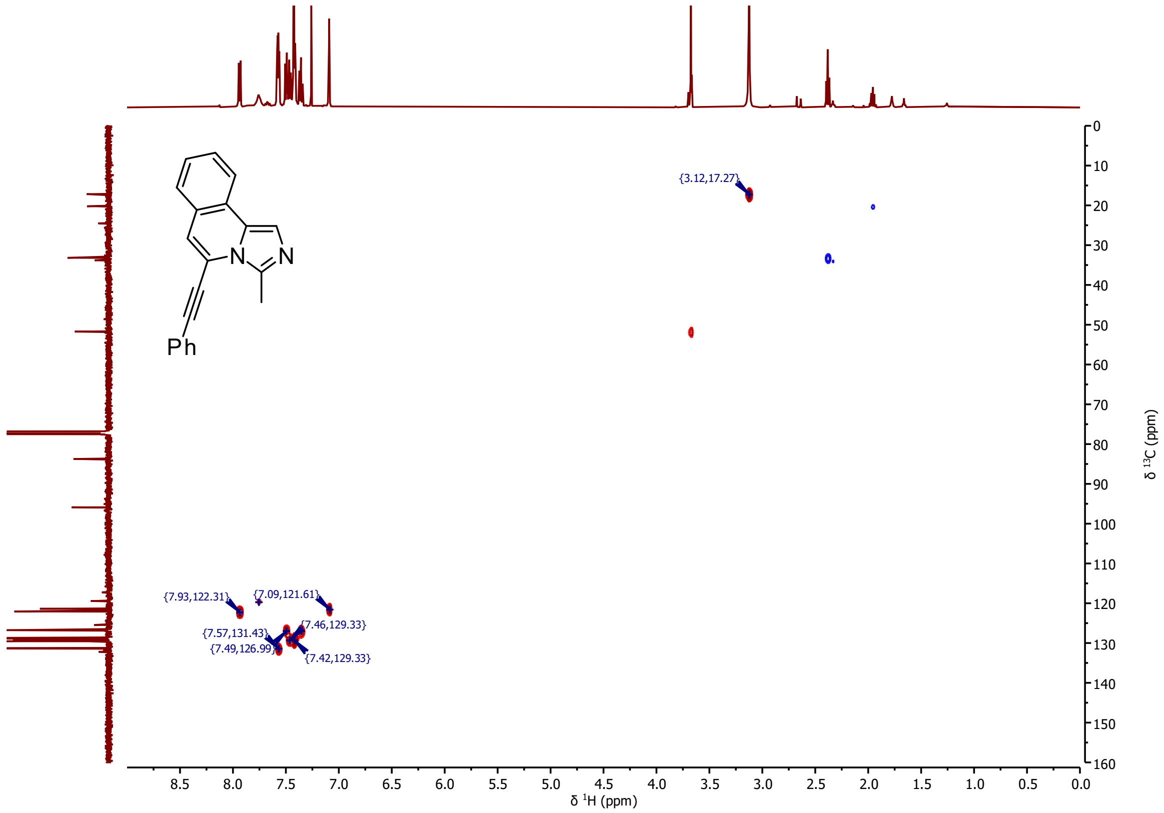

**Figure S242**: **$^{1}$H/$^{13}$C HSQC** (500/126 MHz, $CDCl_3$, 298 K) of **S49**.

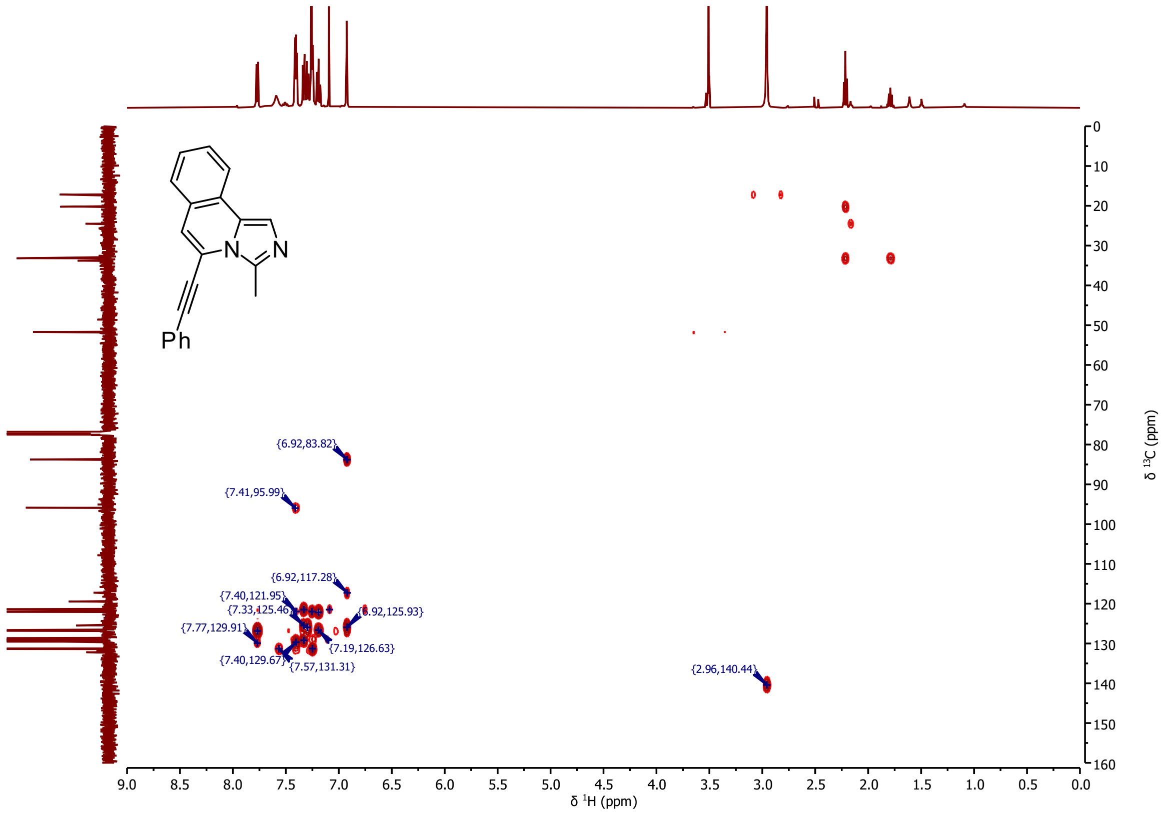


**Figure S243**: **$^{1}H/^{13}C$ HMBC** (500/126 MHz, $CDCl_3$, 298 K) of **S49**.

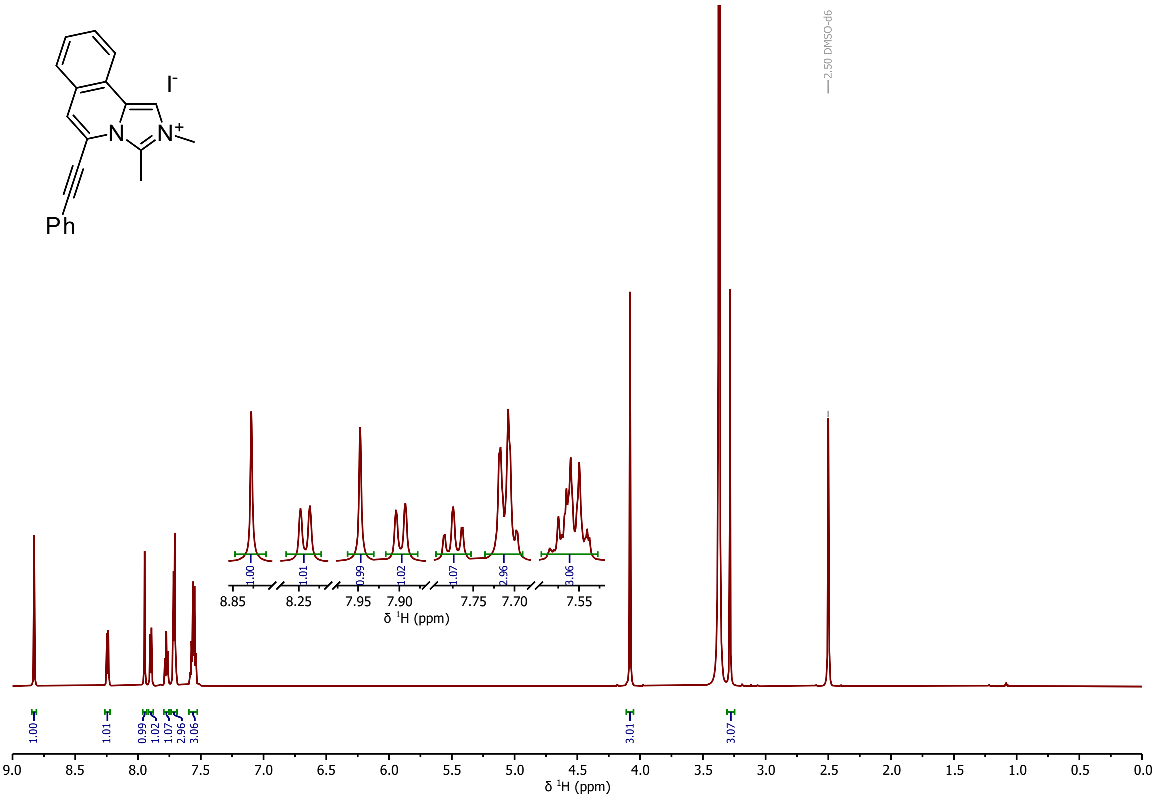


**Figure S244**: **$^{1}H$ NMR** (700 MHz, DMSO-$d_6$, 298 K) of **4n**.

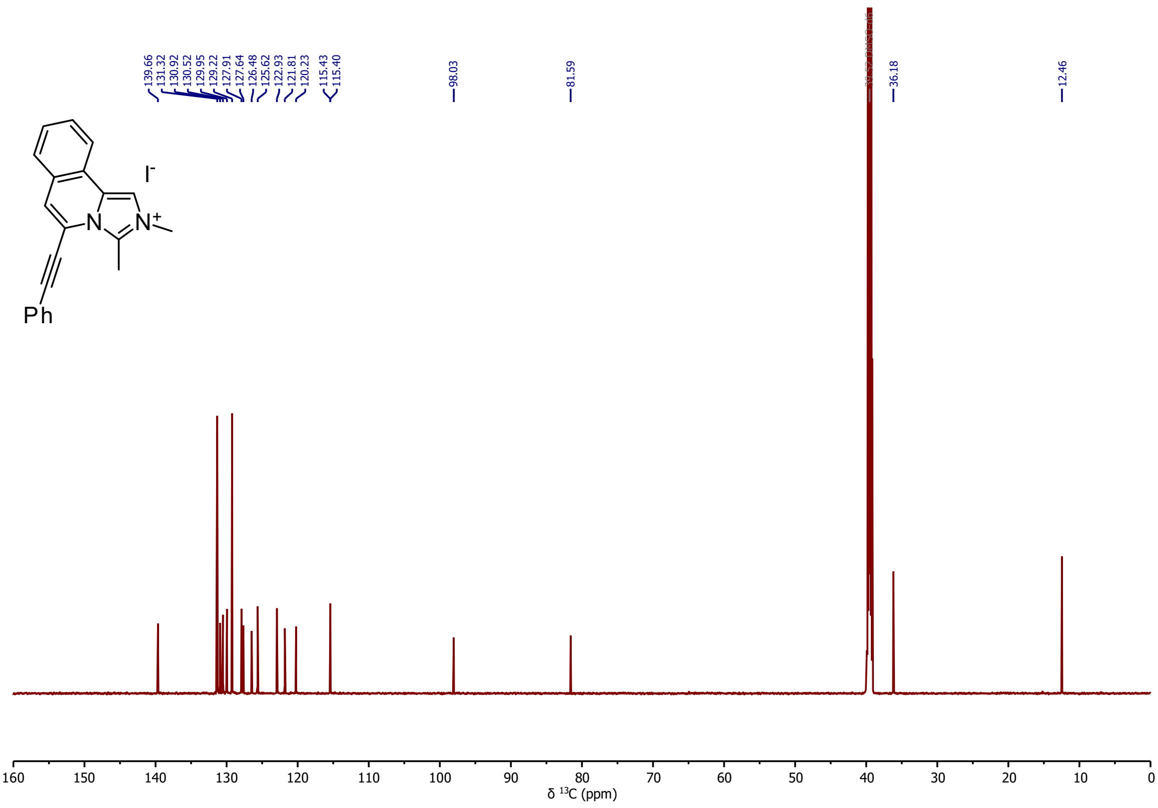

**Figure S245**: **$^{13}$C NMR** (176 MHz, DMSO-d$_6$, 298 K) of **4n**.

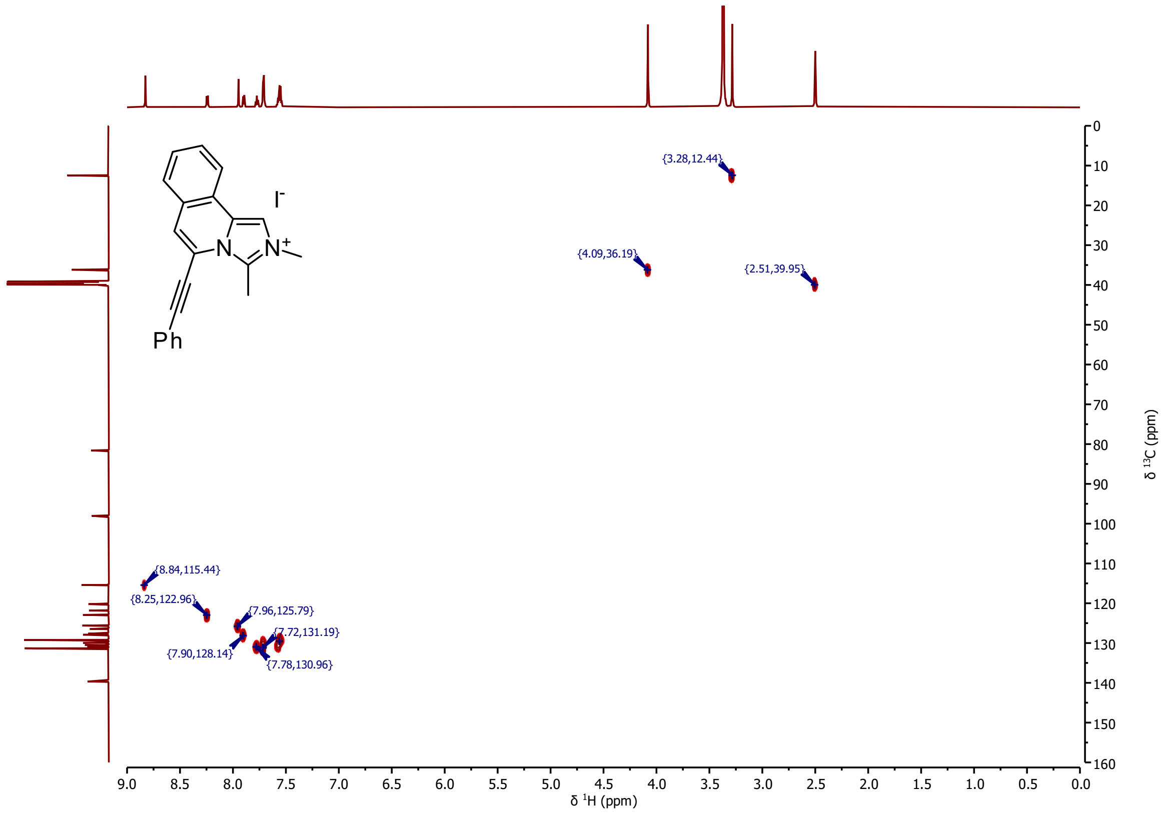

**Figure S246**: **$^{1}$H/$^{13}$C HSQC** (700/176 MHz, DMSO-d$_6$, 298 K) of **4n**.

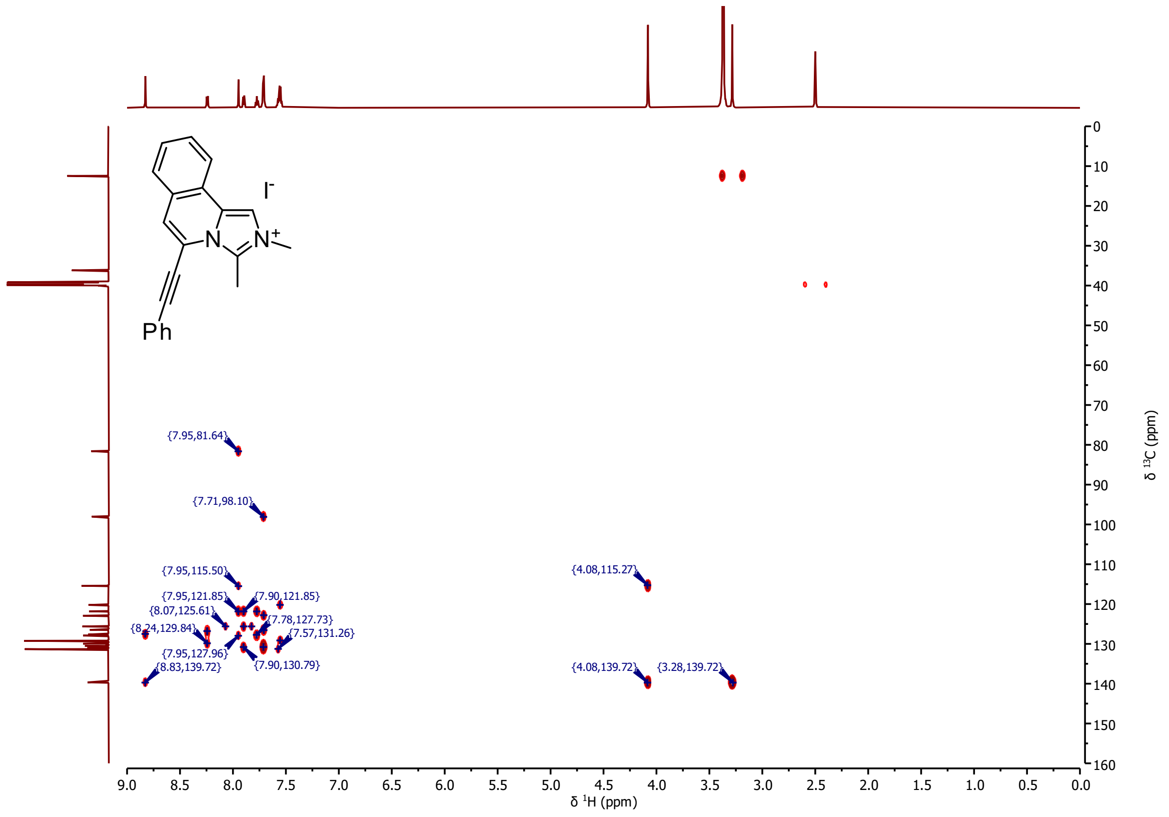


**Figure S247**: **$^{1}$H/$^{13}$C HMBC** (700/176 MHz, DMSO-d$_6$, 298 K) of **4n**.

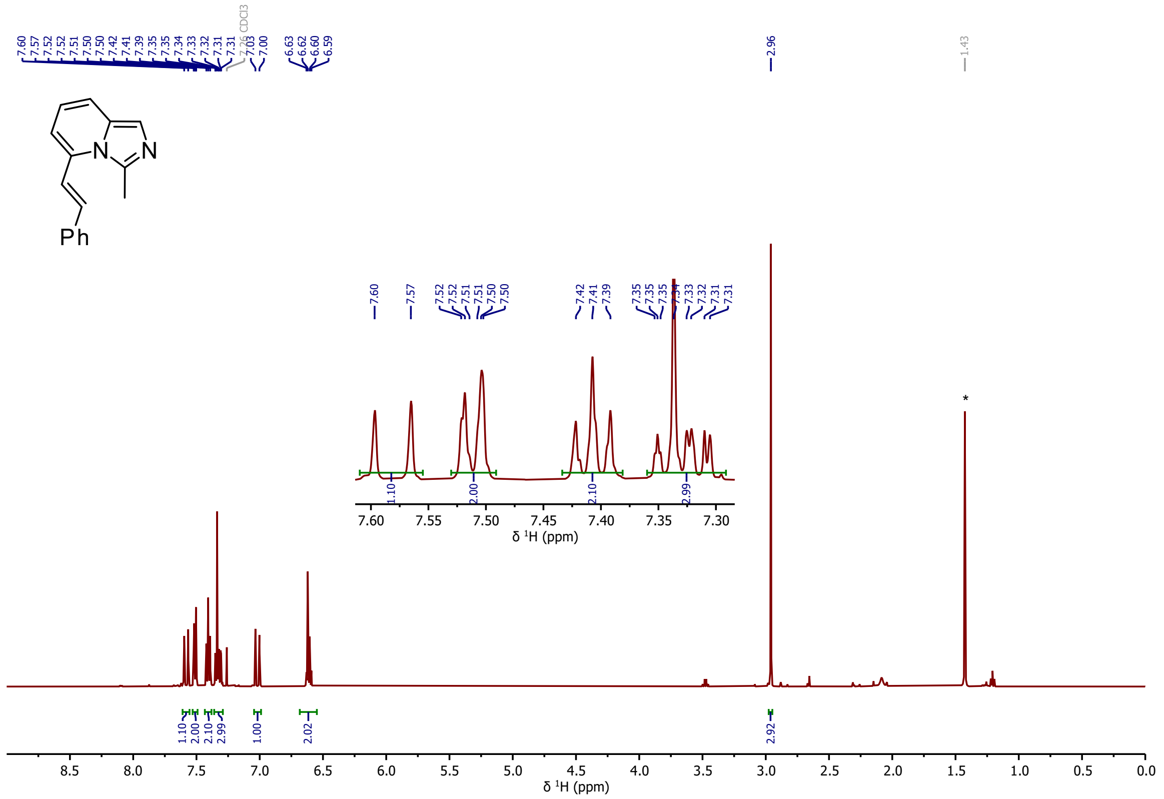


**Figure S248**: **$^{1}$H NMR** (500 MHz, CDCl$_3$, 298 K) of **S50**. * = CyH.

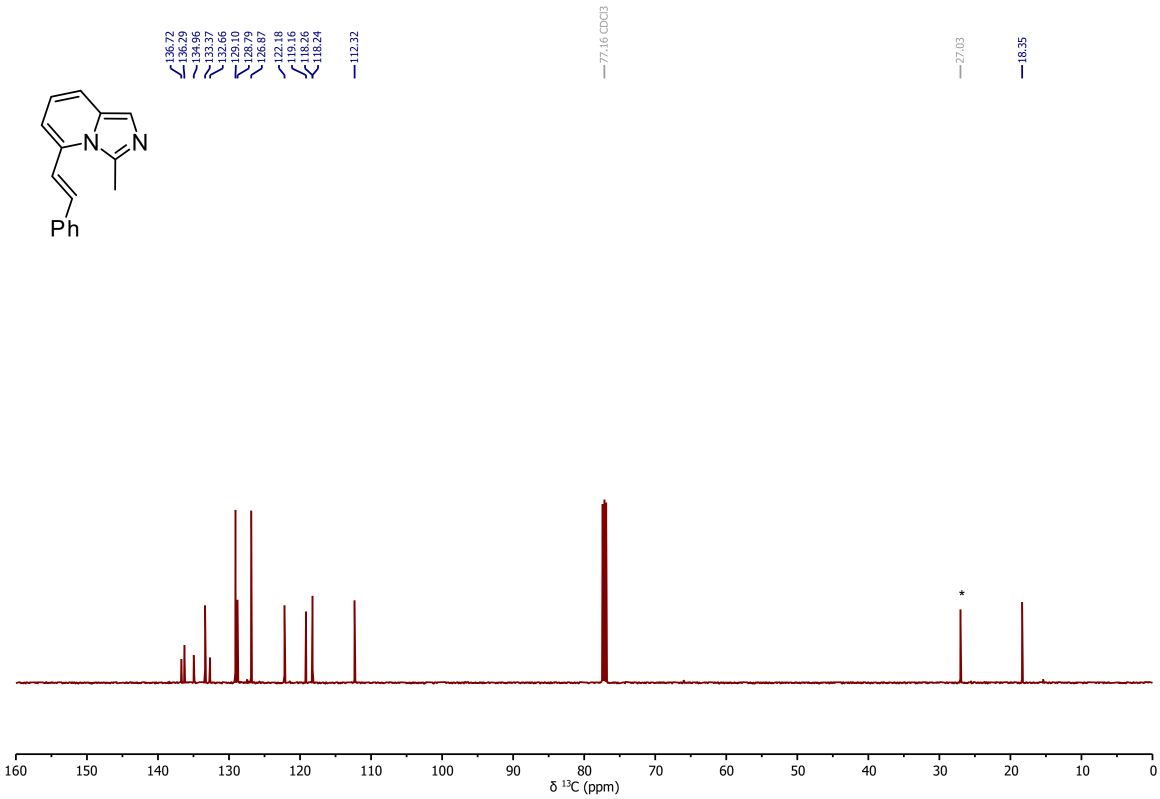


**Figure S249**: **$^{13}$C NMR** (126 MHz, $CDCl_3$, 298 K) of **S50**. * = CyH.

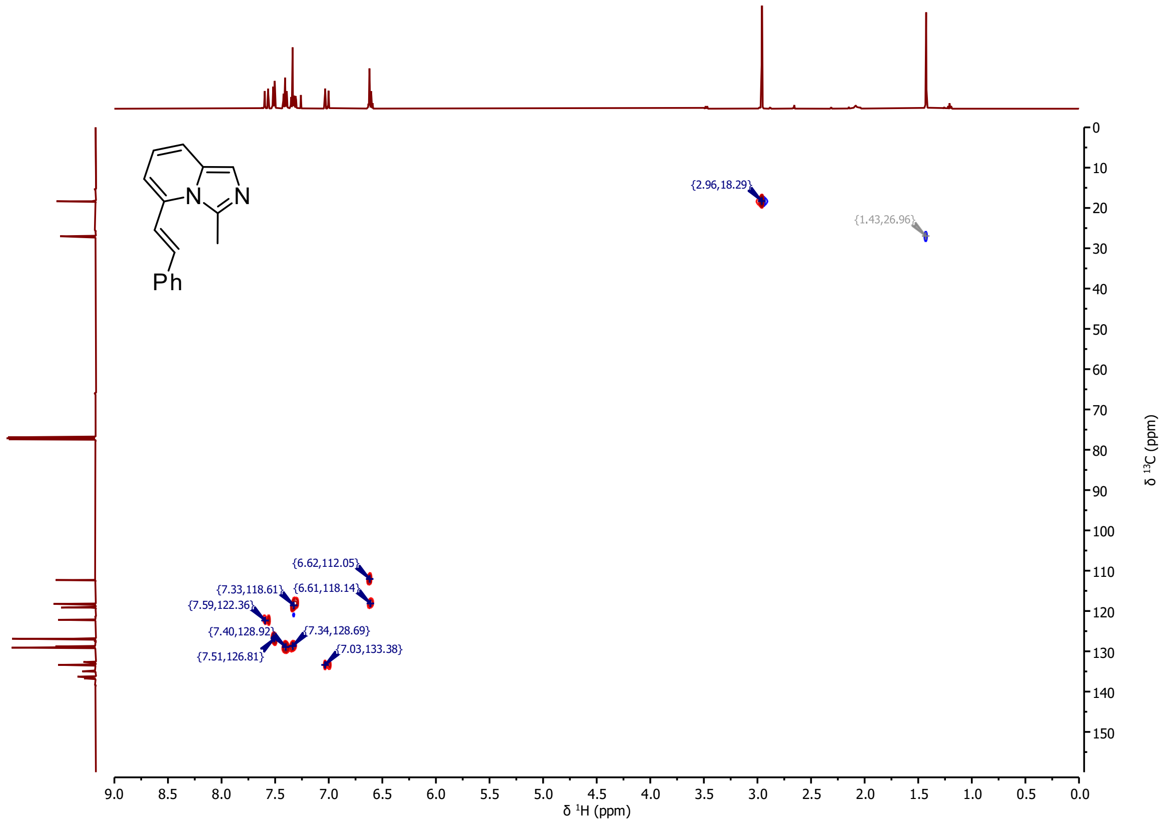


**Figure S250**: **$^{1}$H/$^{13}$C HSQC** (500/126 MHz, $CDCl_3$, 298 K) of **S50**.

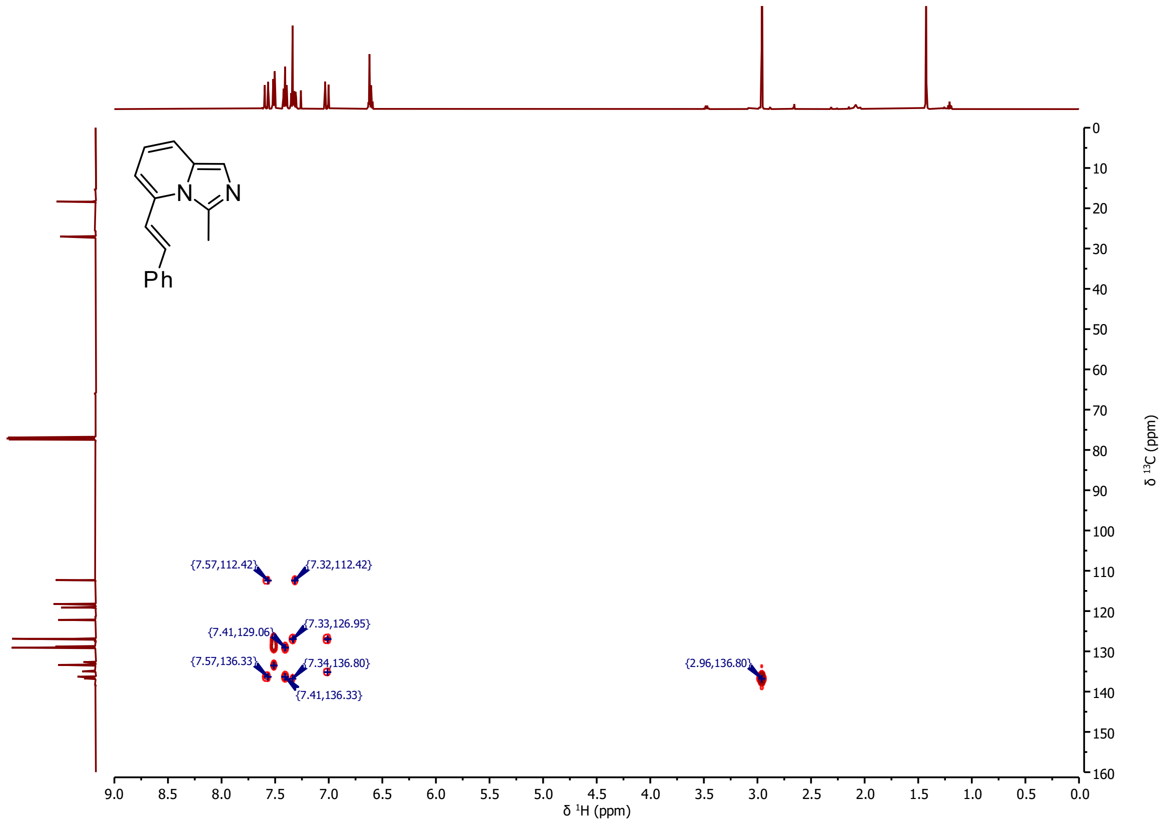

**Figure S251**: **$^1$H/$^{13}$C HMBC** (500/126 MHz, $CDCl_3$, 298 K) of **S50**.

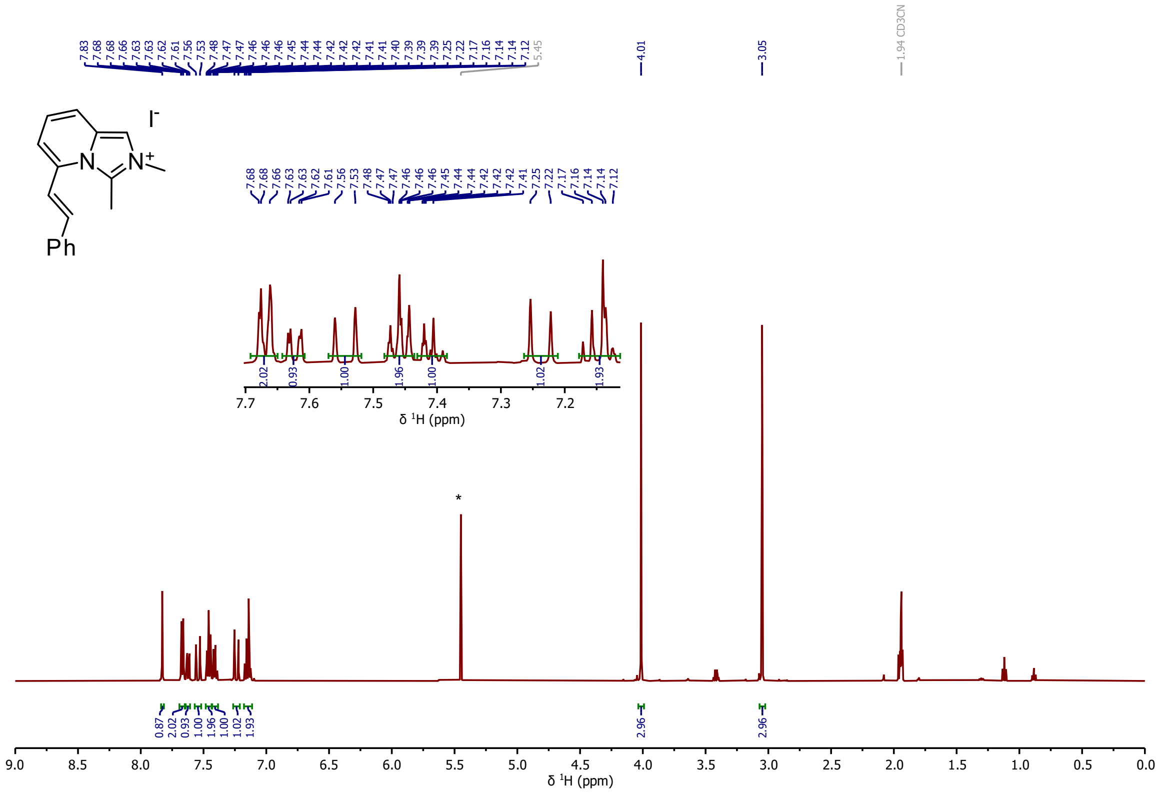

**Figure S252**: **$^1$H NMR** (500 MHz, $CD_3CN$, 298 K) of **4o**. * = $CH_2Cl_2$.

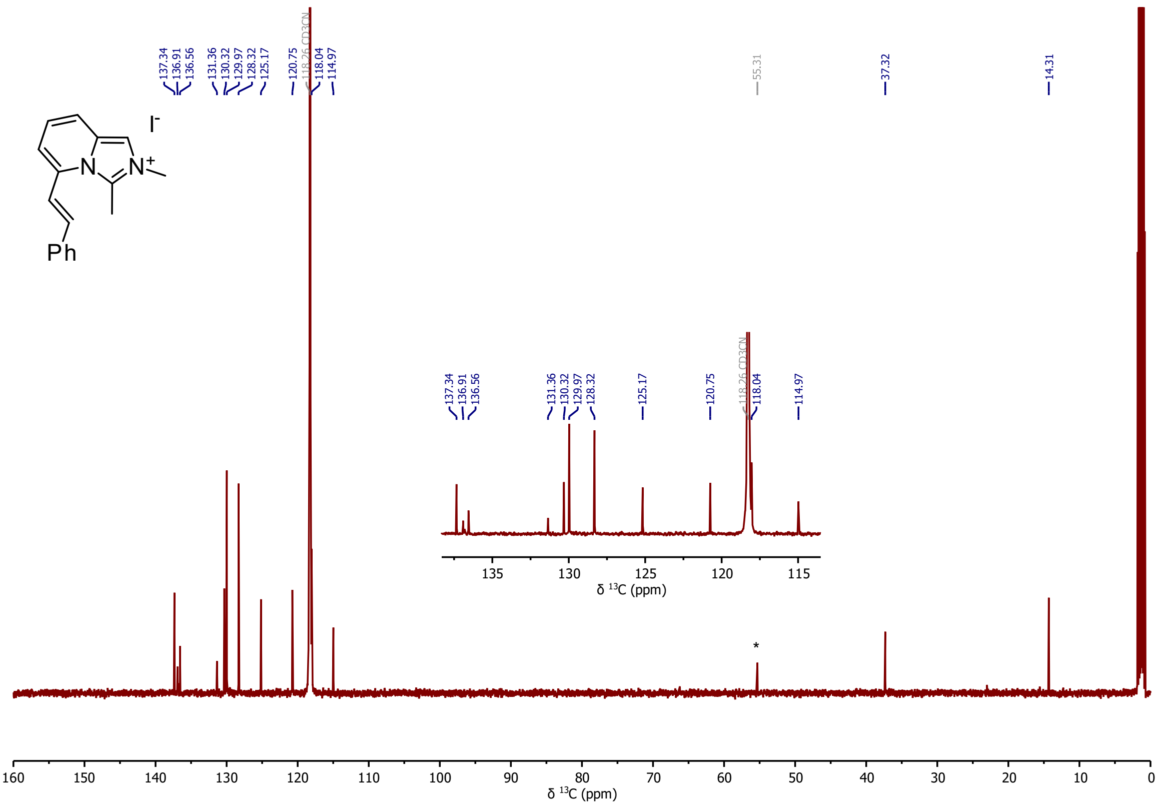


**Figure S253**: **$^{13}$C NMR** (126 MHz, $CD_3CN$, 298 K) of **4o**. * = $CH_2Cl_2$.

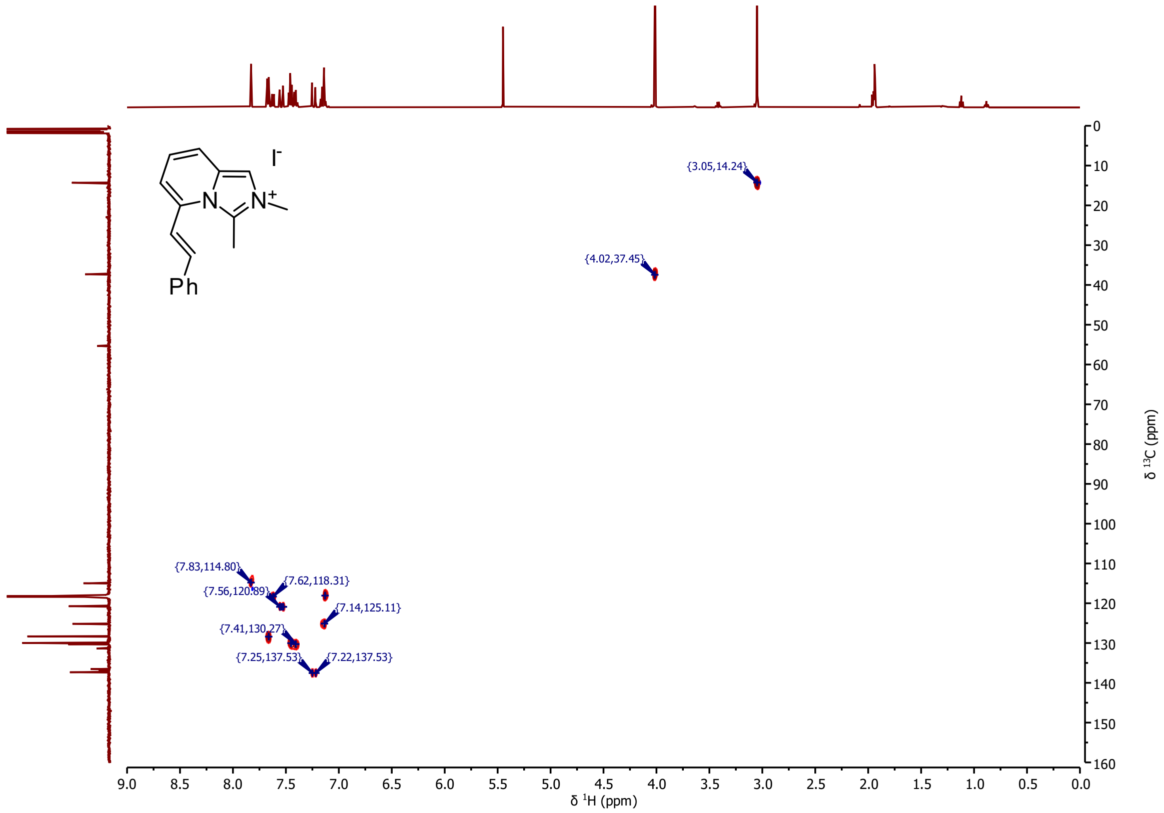


**Figure S254**: **$^{1}$H/$^{13}$C HSQC** (500/126 MHz, $CD_3CN$, 298 K) of **4o**.

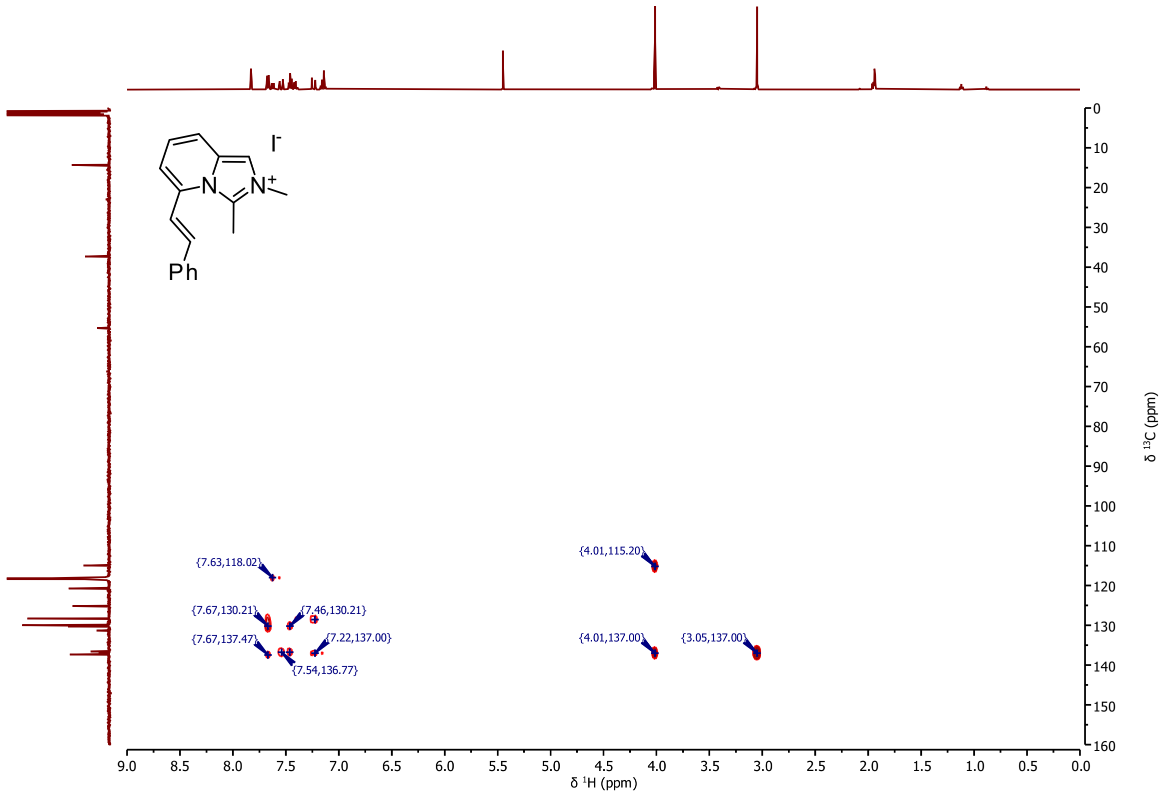

**Figure S255**: **$^{1}$H/$^{13}$C HMBC** (500/126 MHz, $CD_3CN$, 298 K) of **4o**.

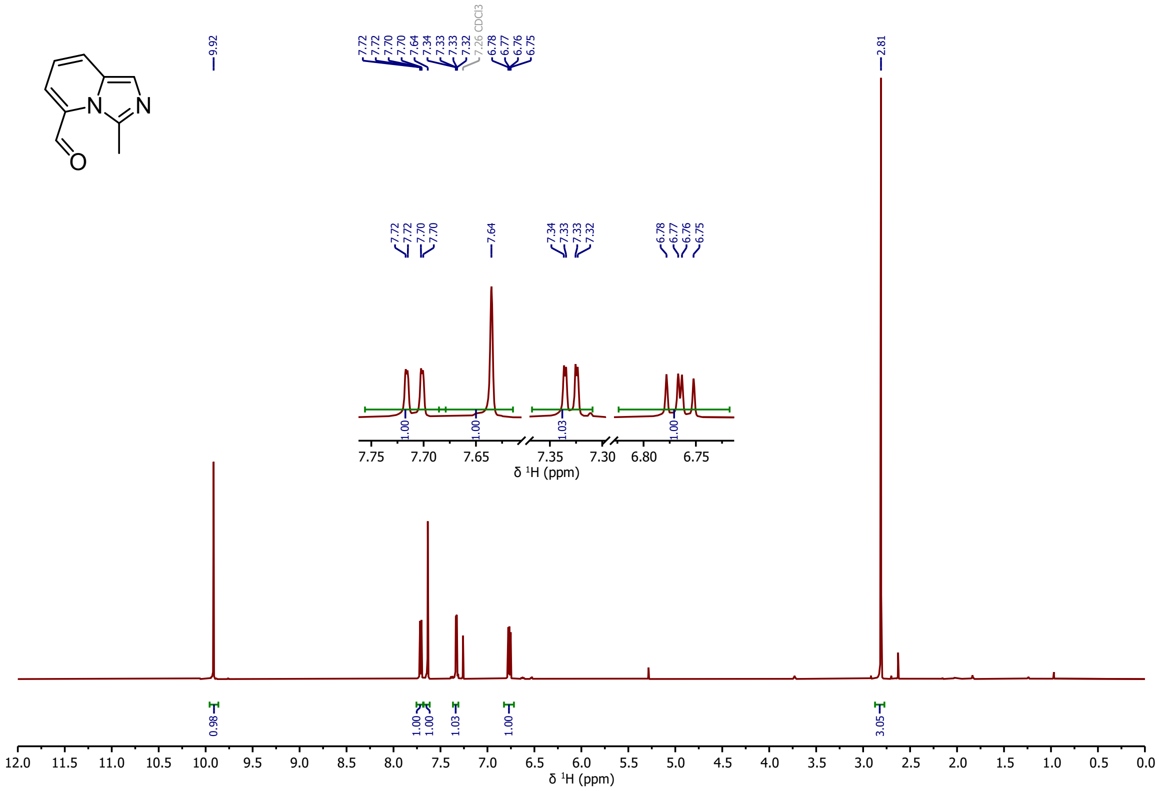

**Figure S256**: **$^{1}$H NMR** (600 MHz, $CDCl_3$, 298 K) of **S51**.

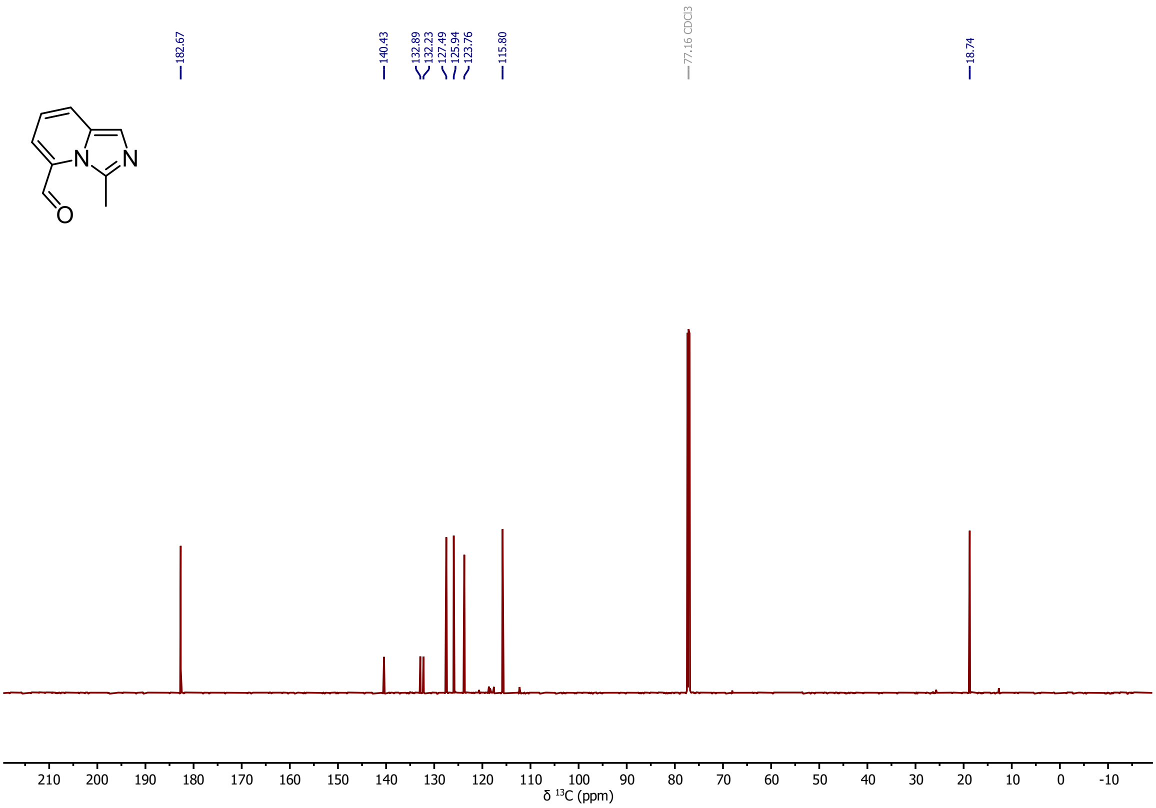


**Figure S257**: **$^{13}$C NMR** (151 MHz, $CDCl_3$, 298 K) of **S51**.

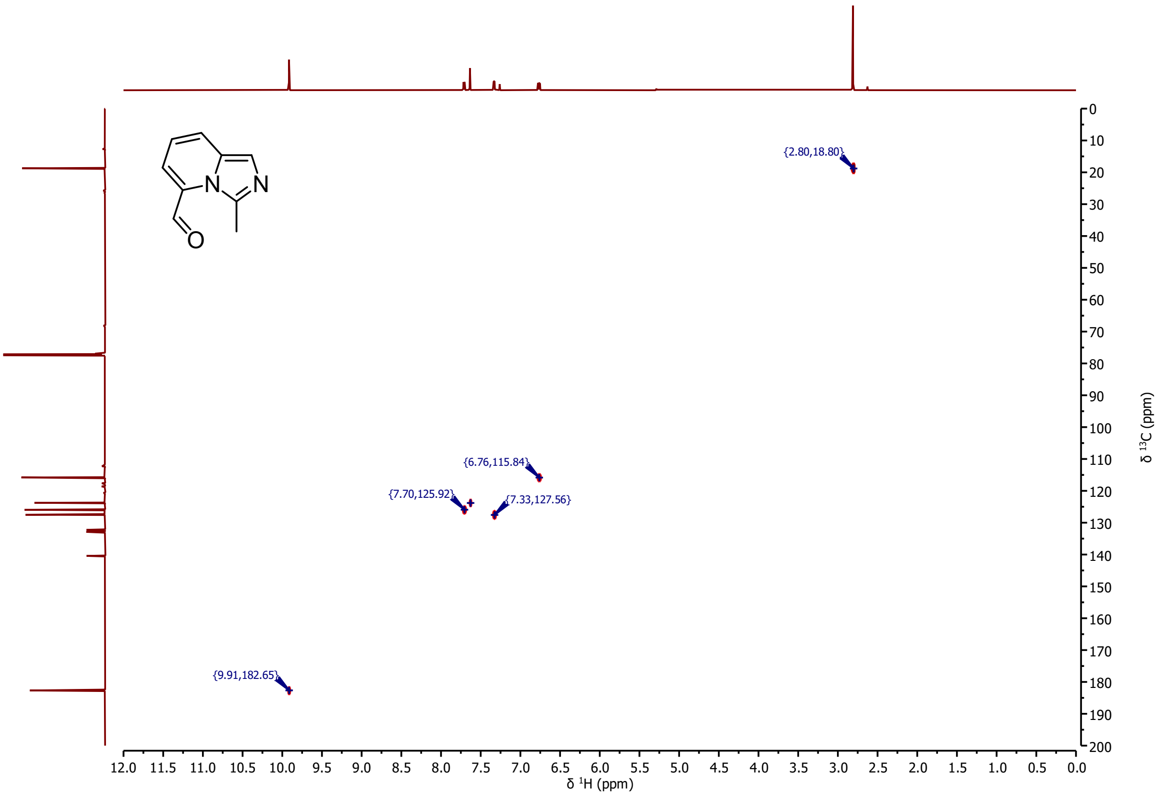


**Figure S258**: **$^{1}$H/$^{13}$C HSQC** (600/151 MHz, $CDCl_3$, 298 K) of **S51**.

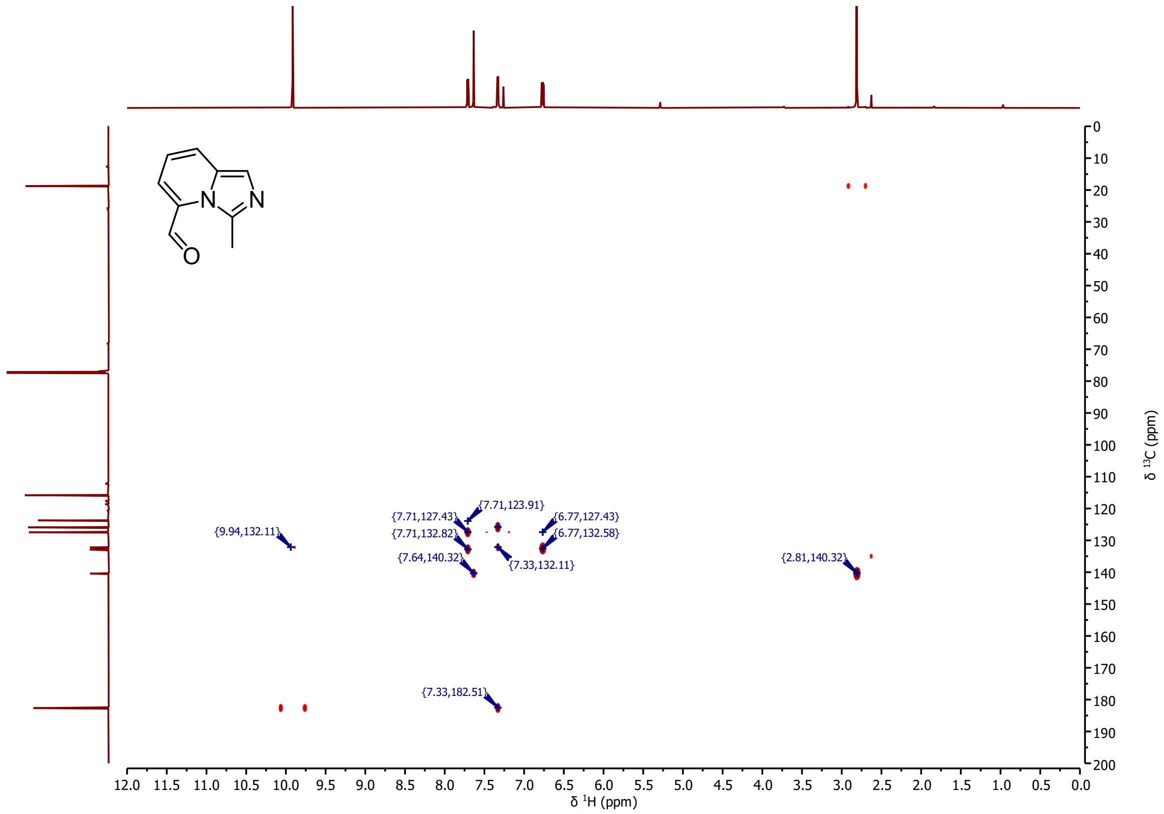

**Figure S259**: **$^1H/^{13}C$ HMBC** (600/151 MHz, $CDCl_3$, 298 K) of **S51**.

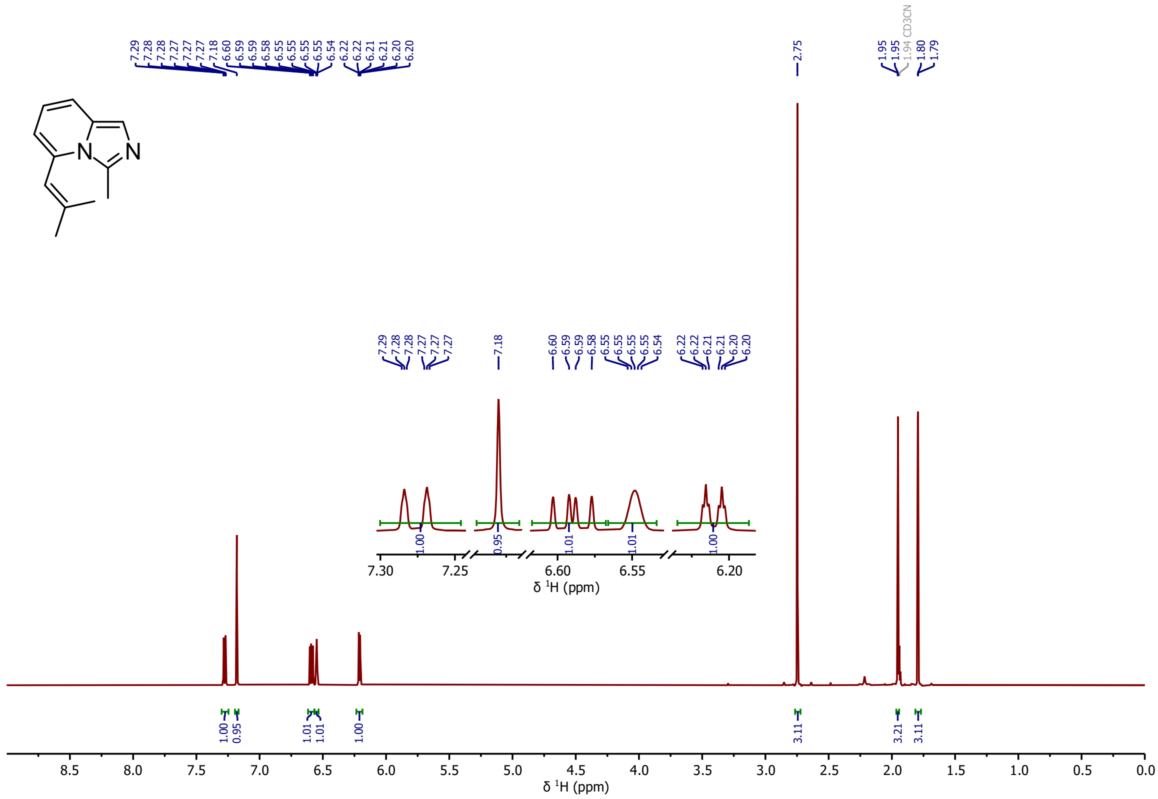

**Figure S260**: **$^1H$ NMR** (600 MHz, $CD_3CN$, 298 K) of **S52**.

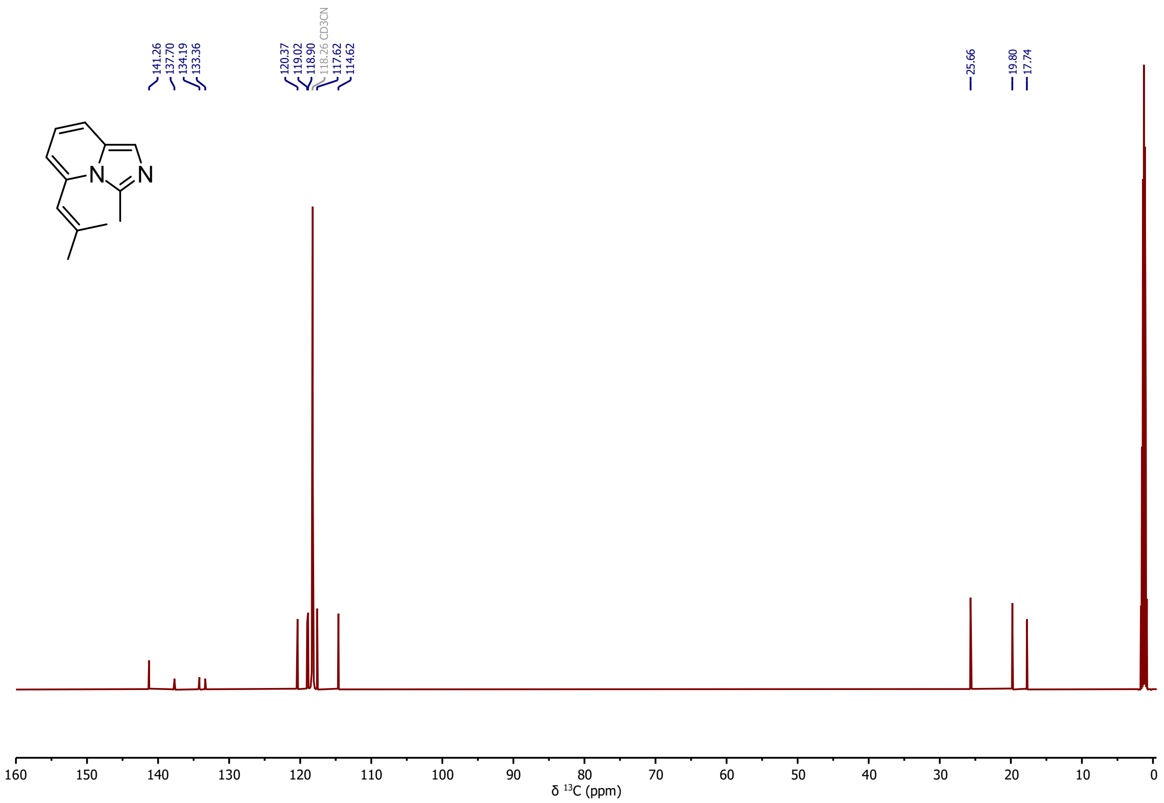


**Figure S261**: **$^{13}$C NMR** (151 MHz, $CD_3CN$, 298 K) of **S52**.

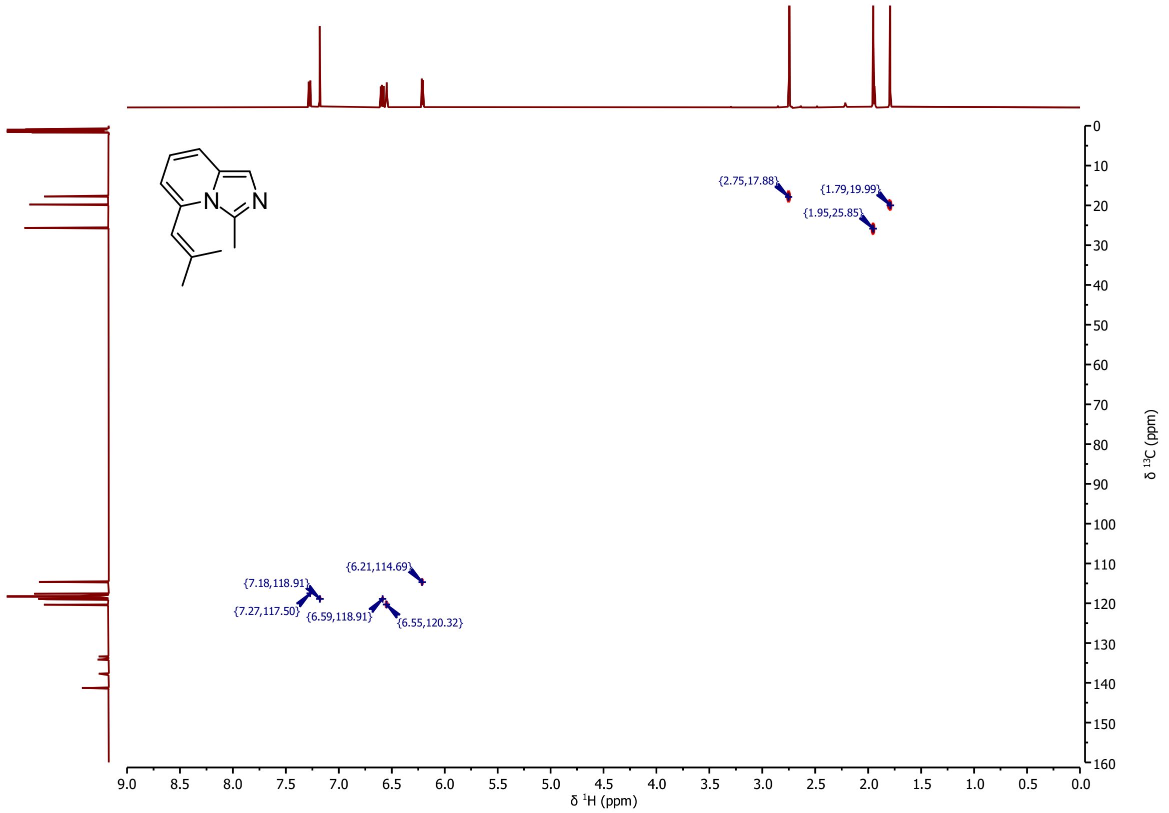


**Figure S262**: **$^{1}$H/$^{13}$C HSQC** (600/151 MHz, $CD_3CN$, 298 K) of **S52**.

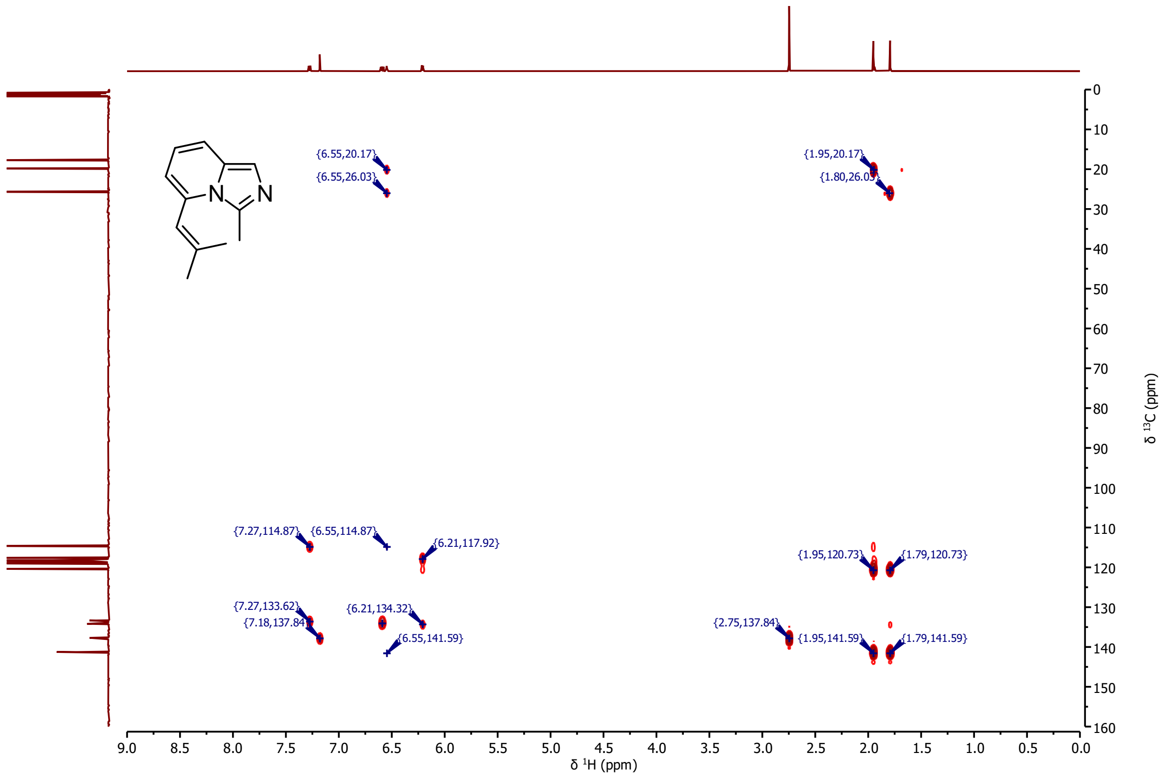

**Figure S263**: **$^{1}$H/$^{13}$C HMBC** (600/151 MHz, $CD_3CN$, 298 K) of **S52**.

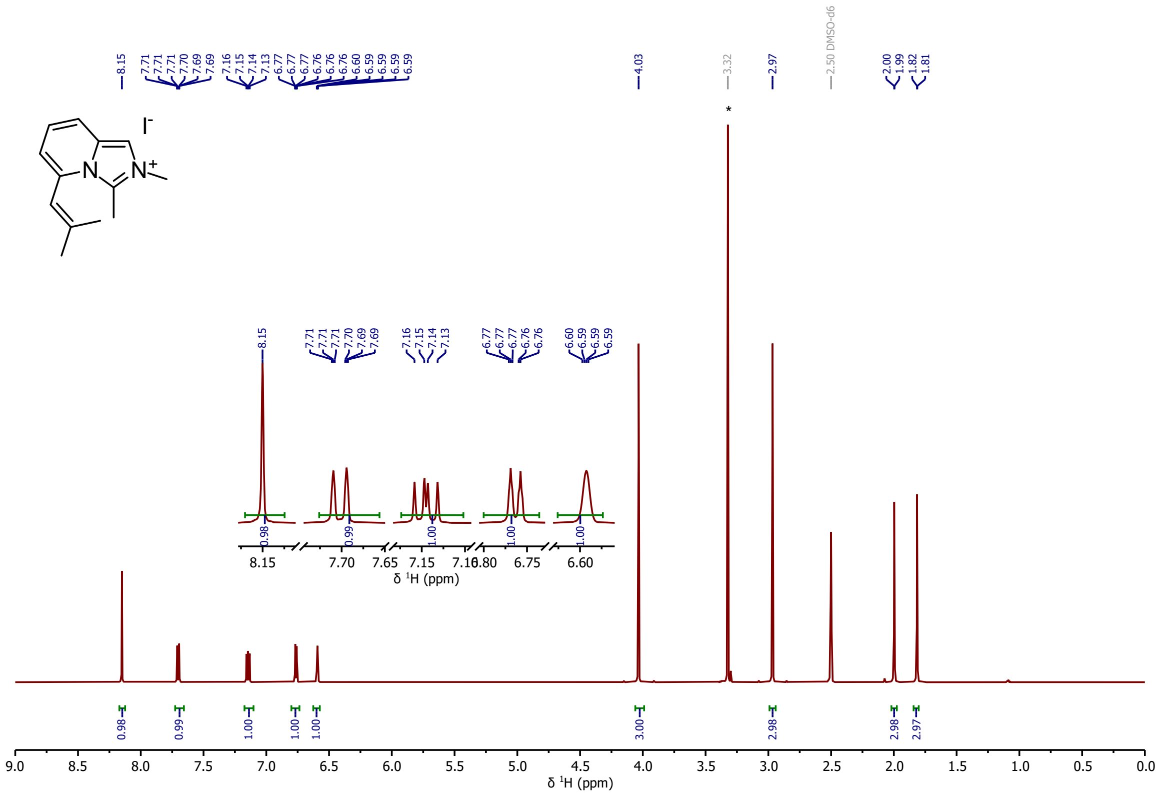

**Figure S264**: **$^{1}$H NMR** (600 MHz, DMSO-$d_6$, 298 K) of **4p**.

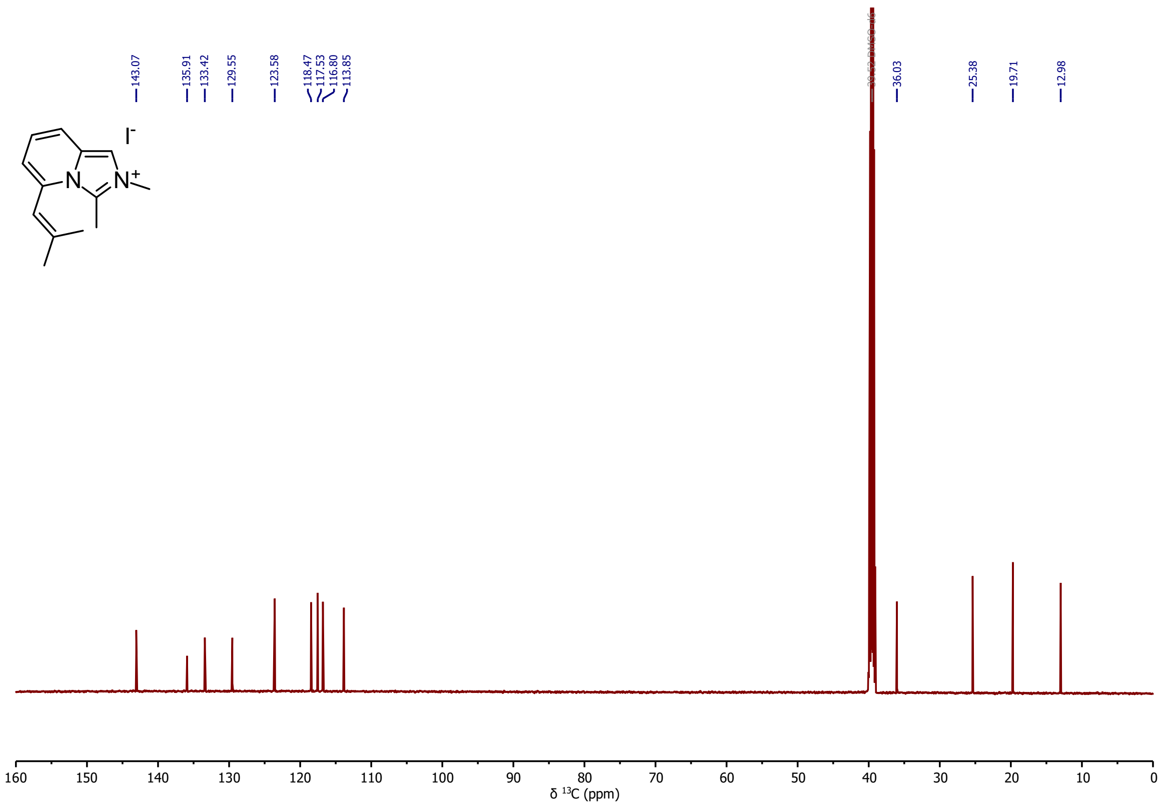


**Figure S265**: **$^{13}$C NMR** (151 MHz, DMSO-$d_6$, 298 K) of **4p**.

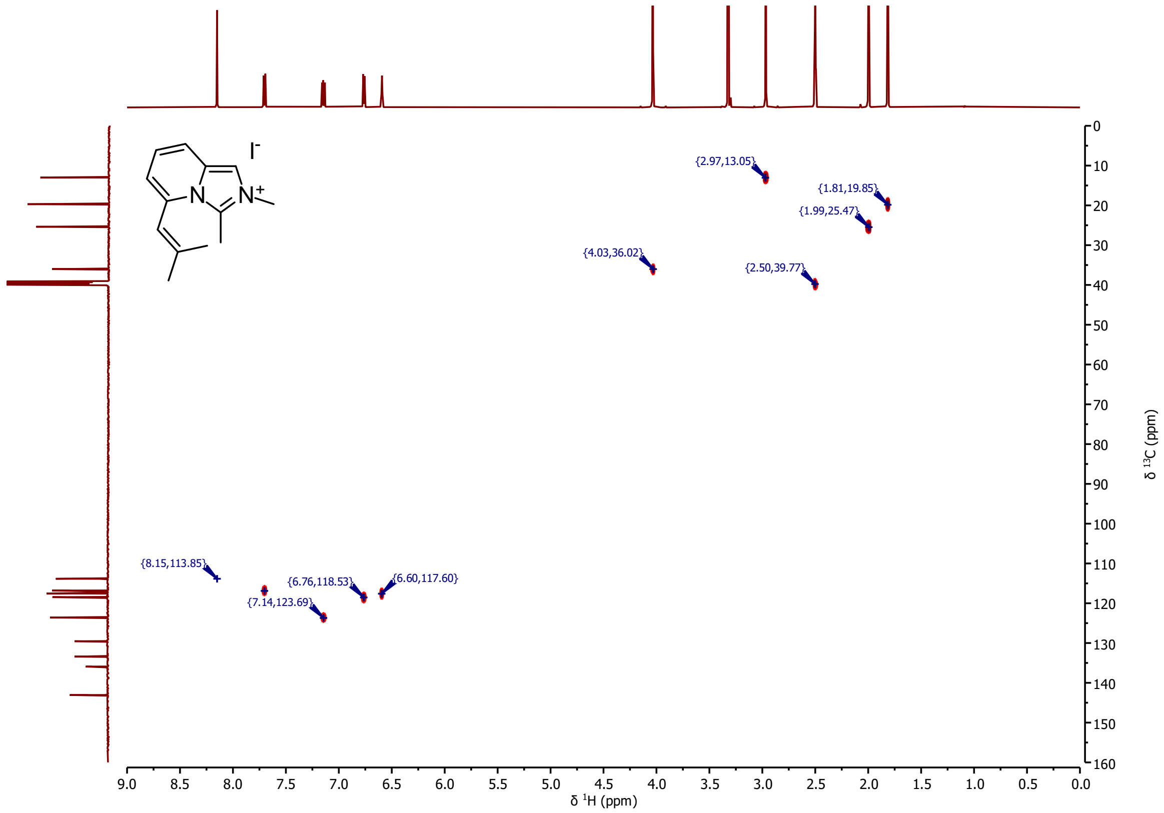


**Figure S266**: **$^{1}$H/$^{13}$C HSQC** (600/151 MHz, DMSO-$d_6$, 298 K) of **4p**.

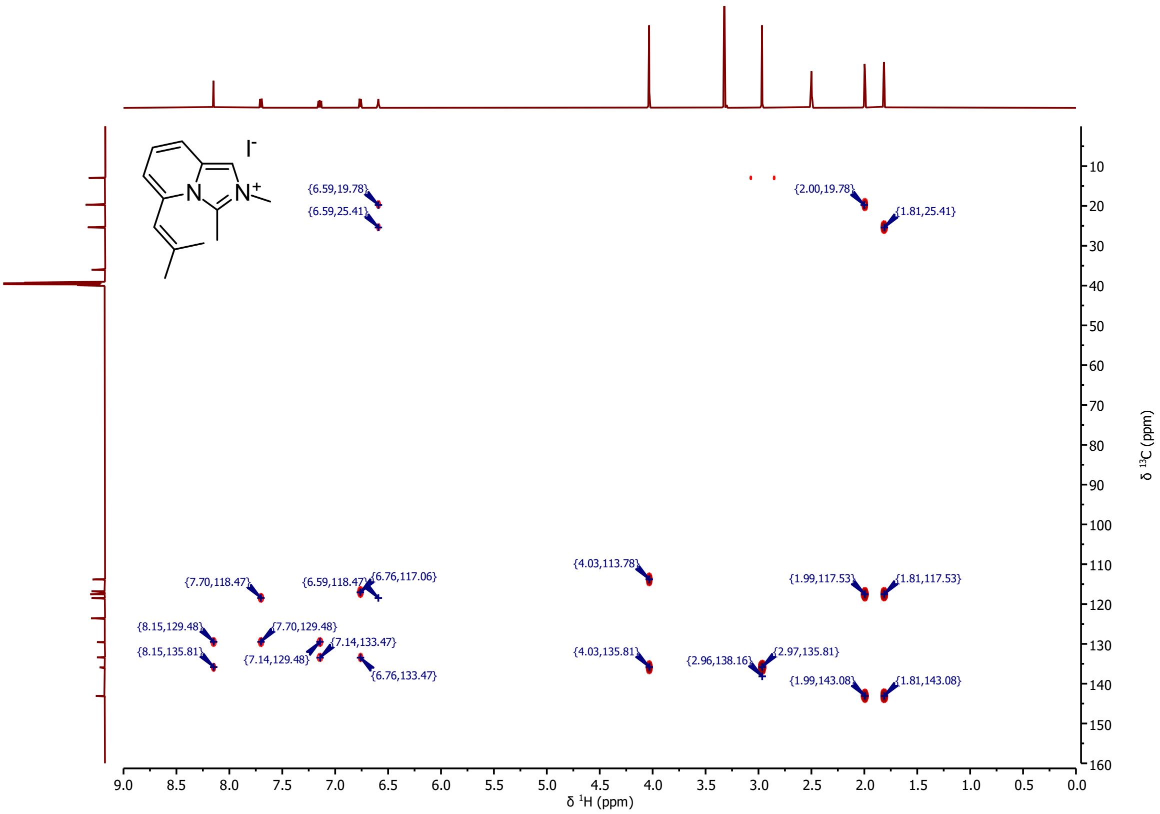

**Figure S267**: $^{1}$**H/**$^{13}$**C HMBC** (600/151 MHz, DMSO-$d_6$, 298 K) of **4p**.

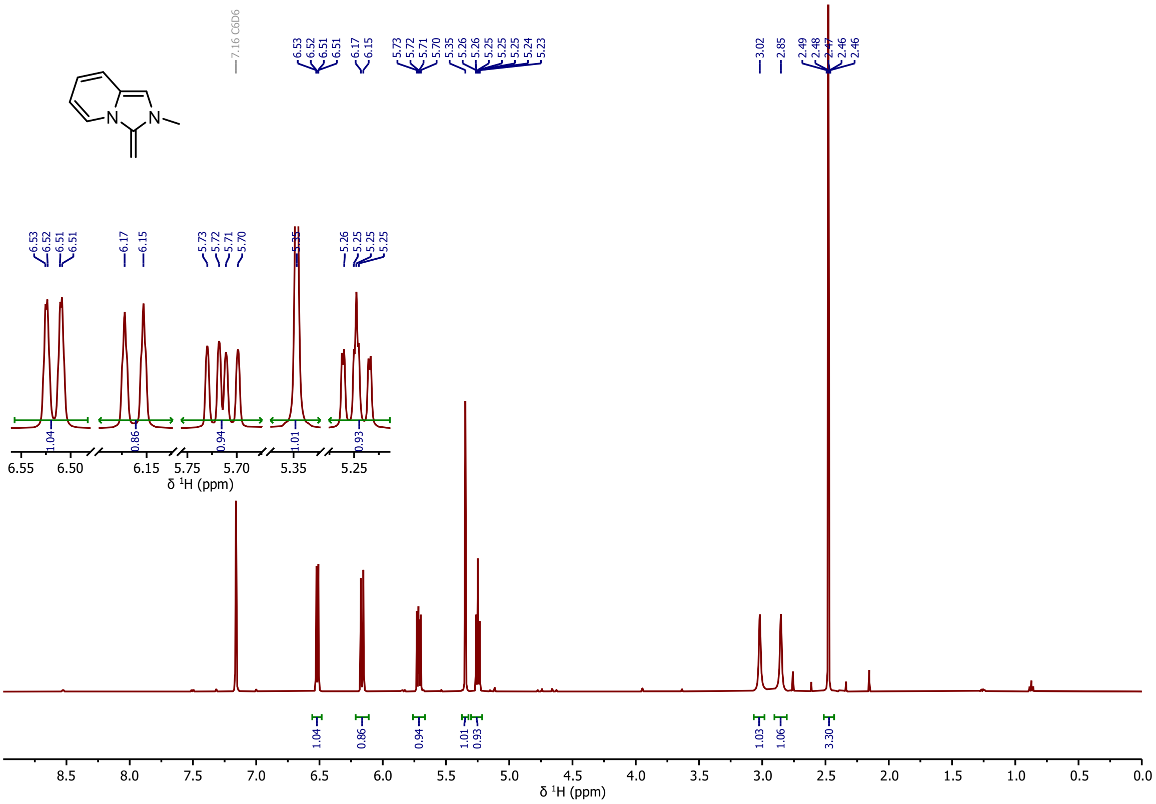

**Figure S268**: $^{1}$**H NMR** (500 MHz, $C_6D_6$, 298 K) of **2a**.

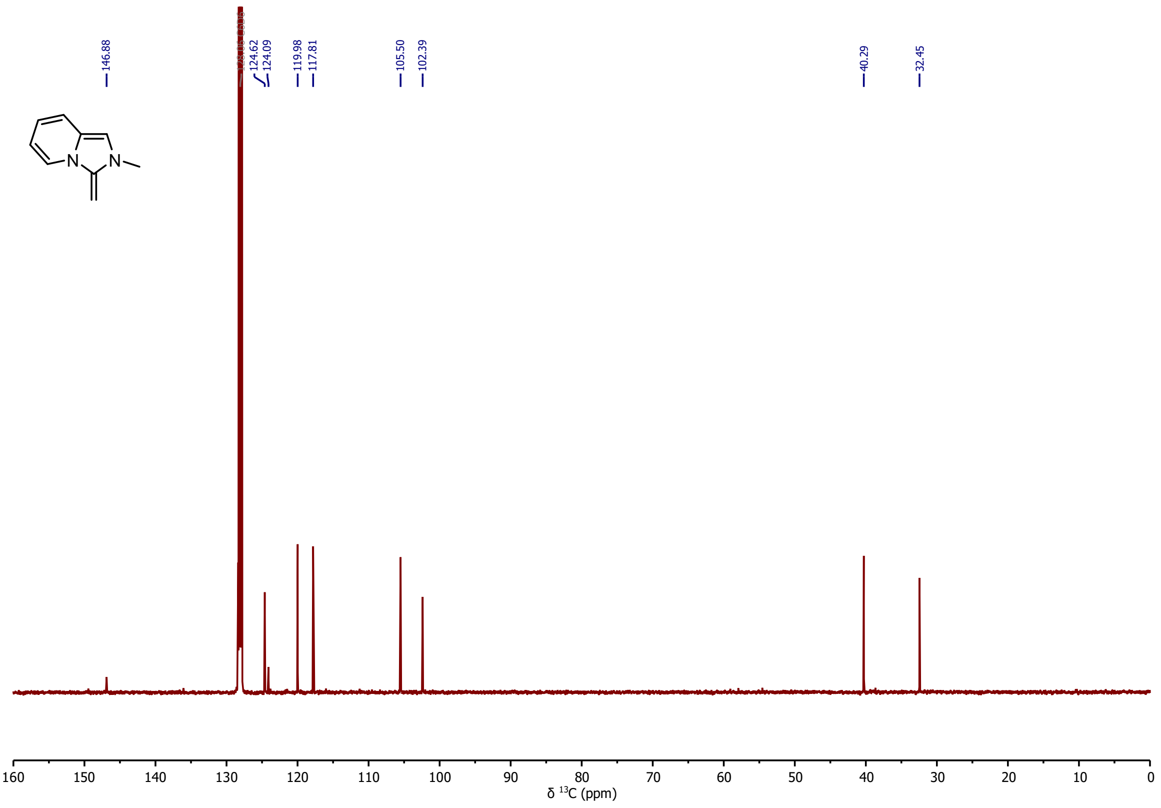


**Figure S269**: **$^{13}$C NMR** (126 MHz, $C_6D_6$, 298 K) of **2a**.

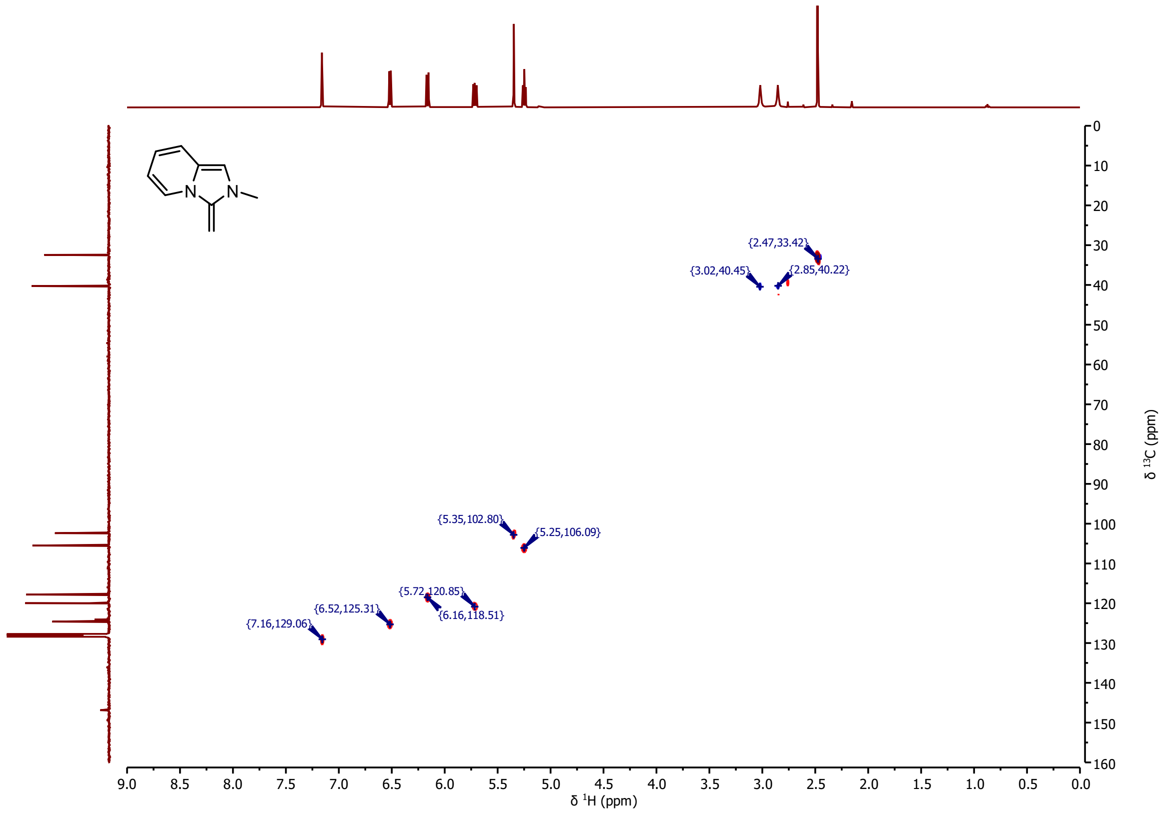


**Figure S270**: **$^1$H/$^{13}$C HSQC** (500/126 MHz, $C_6D_6$, 298 K) of **2a**.

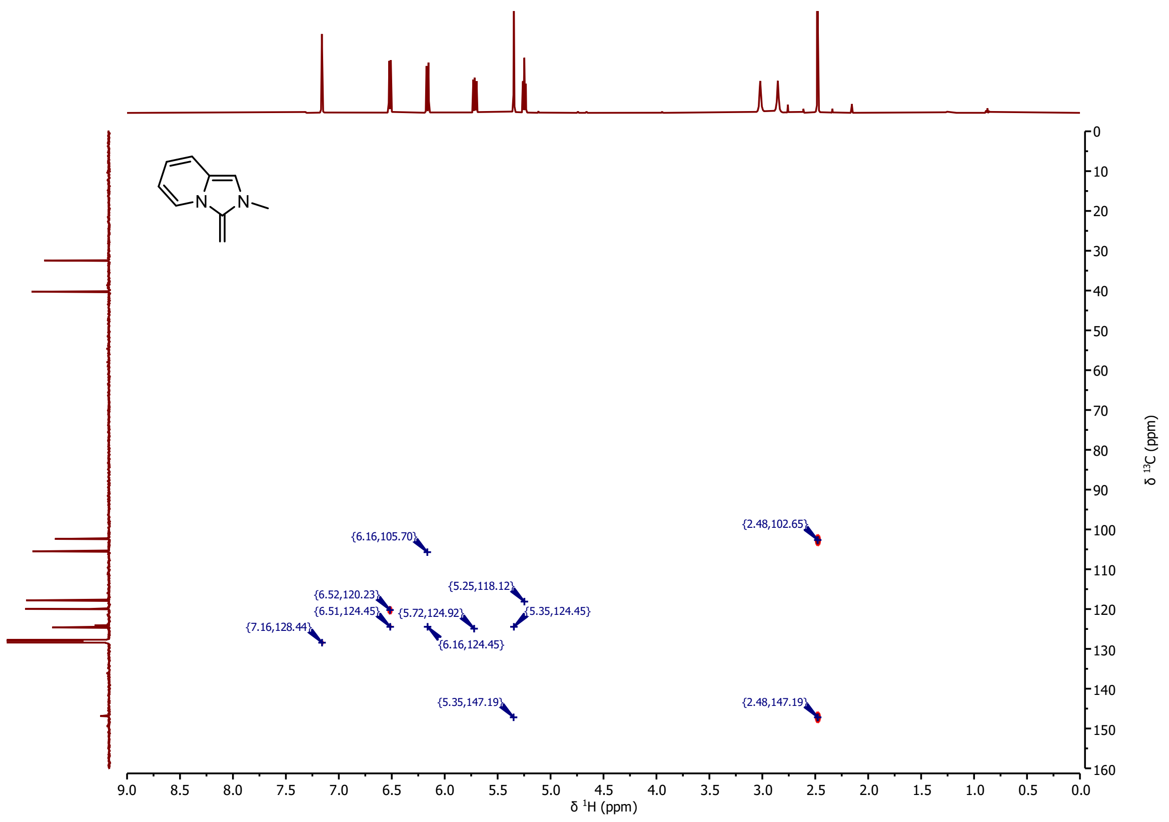

**Figure S271**: **$^1$H/$^{13}$C HMBC** (500/126 MHz, $C_6D_6$, 298 K) of **2a**.

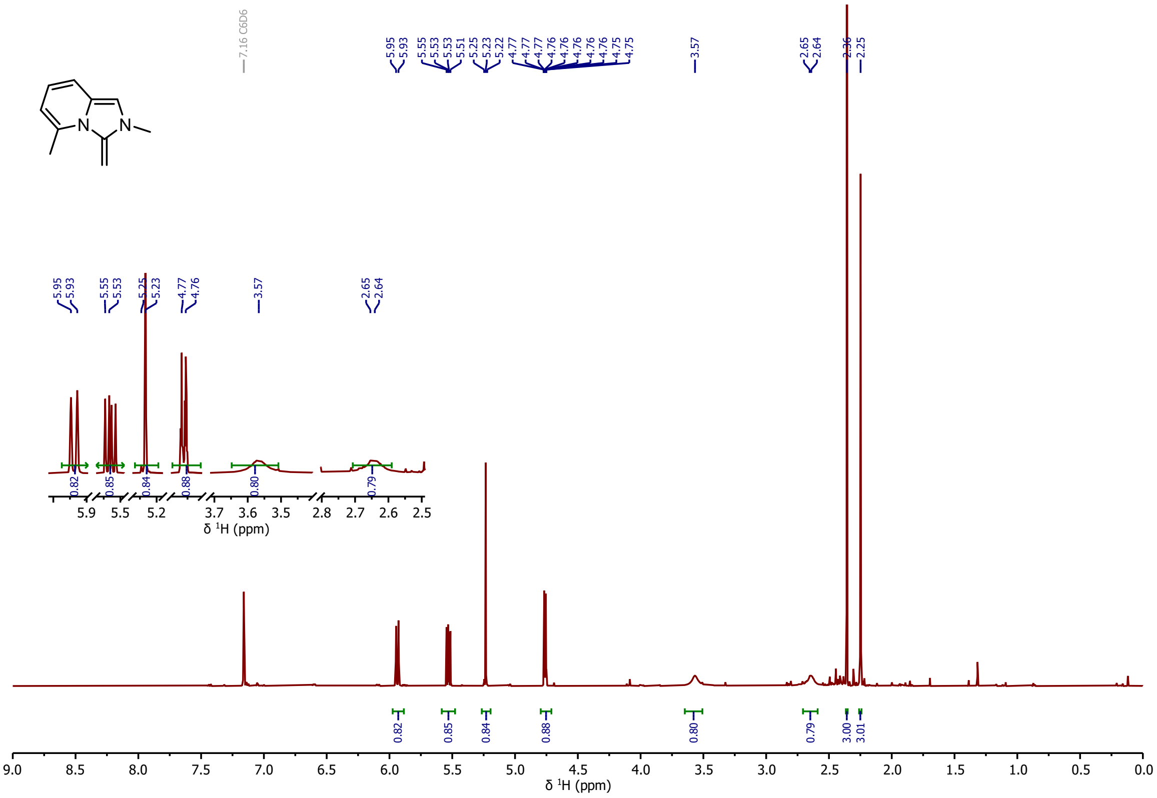

**Figure S272**: **$^1$H NMR** (500 MHz, $C_6D_6$, 298 K) of **2b**.

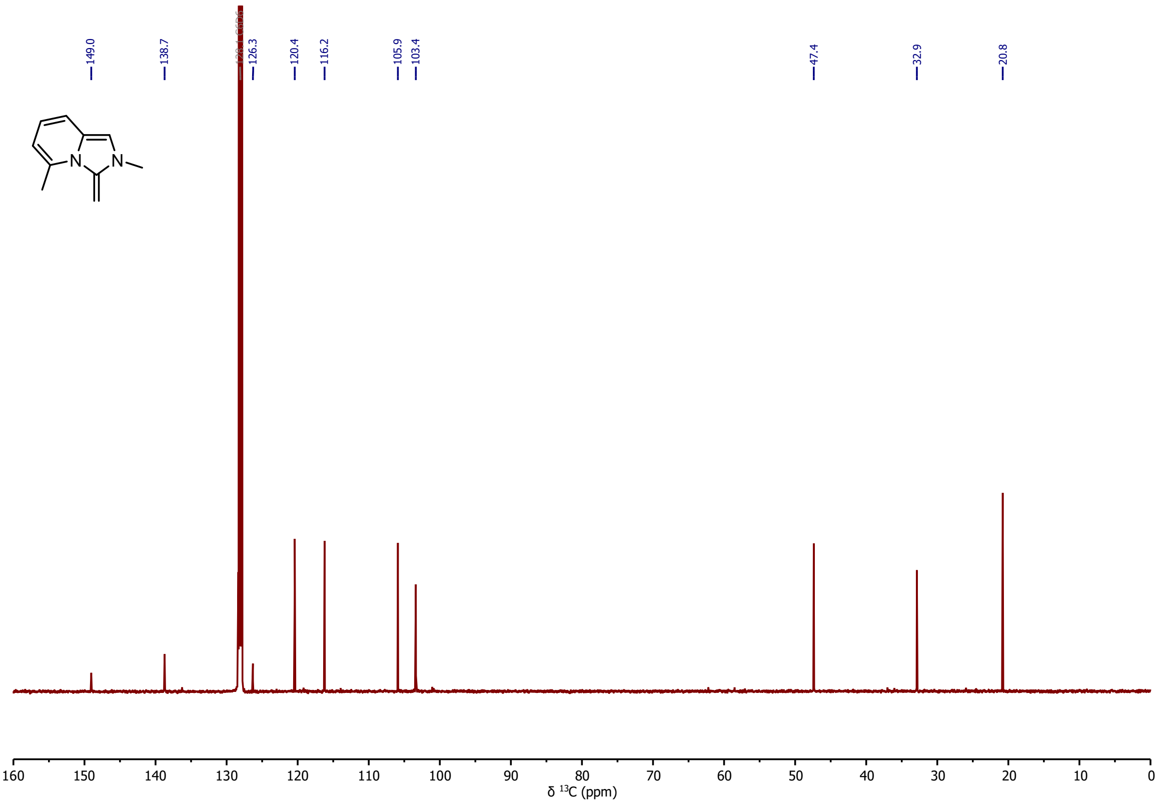

**Figure S273**: **$^{13}$C NMR** (126 MHz, $C_6D_6$, 298 K) of **2b**.

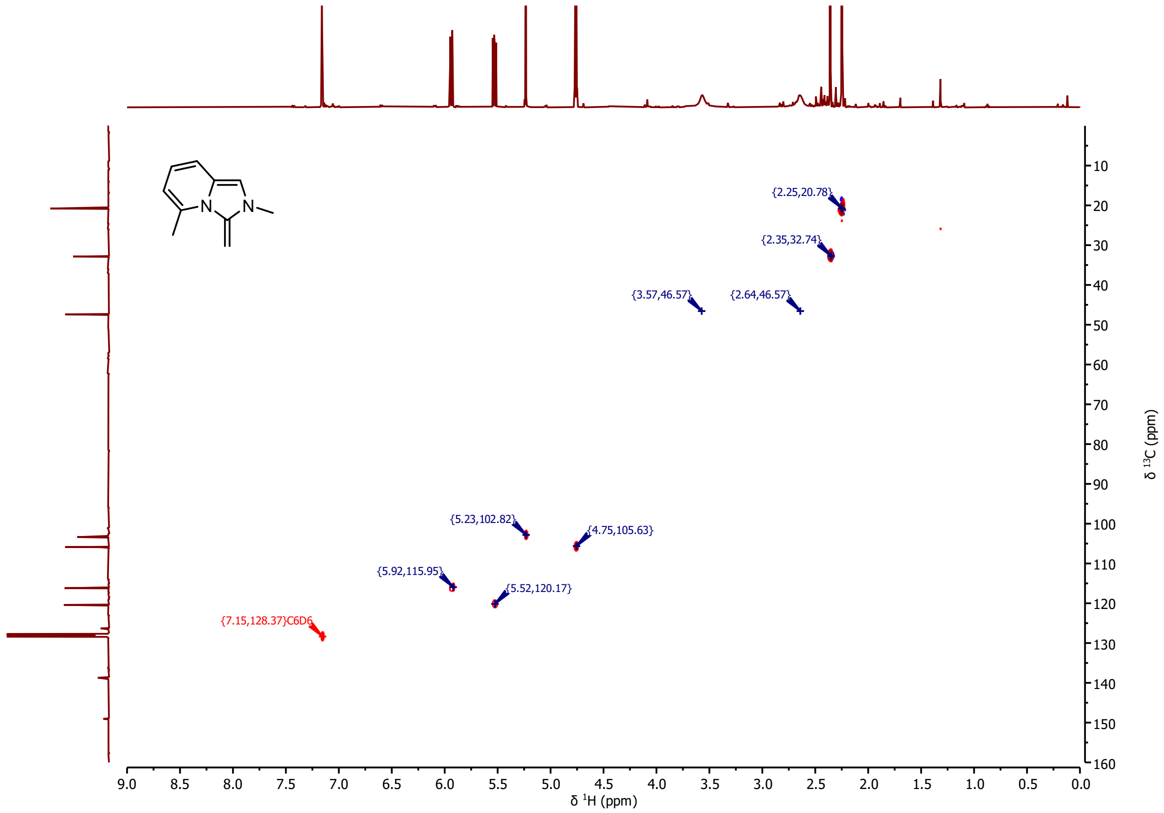

**Figure S274**: **$^{1}$H/$^{13}$C HSQC** (500/126 MHz, $C_6D_6$, 298 K) of **2b**.

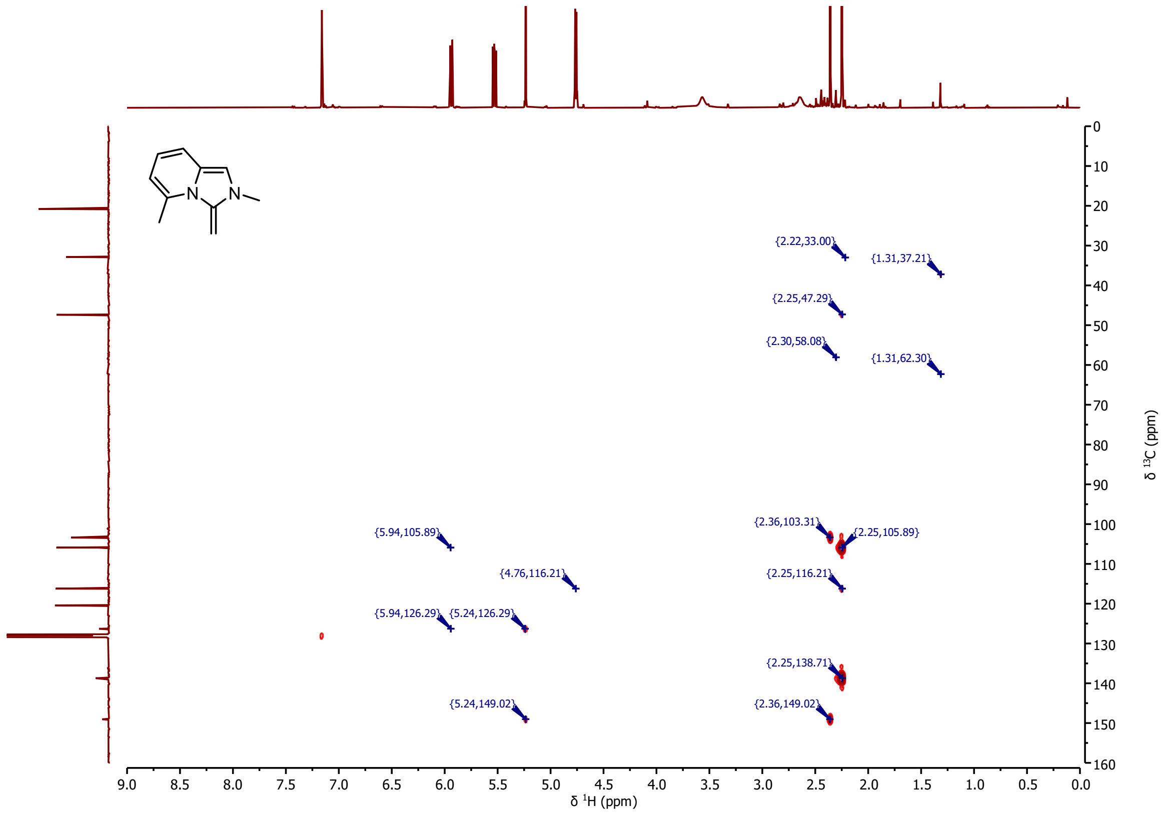


**Figure S275**: **$^1$H/$^{13}$C HMBC** (500/126 MHz, $C_6D_6$, 298 K) of **2b**.

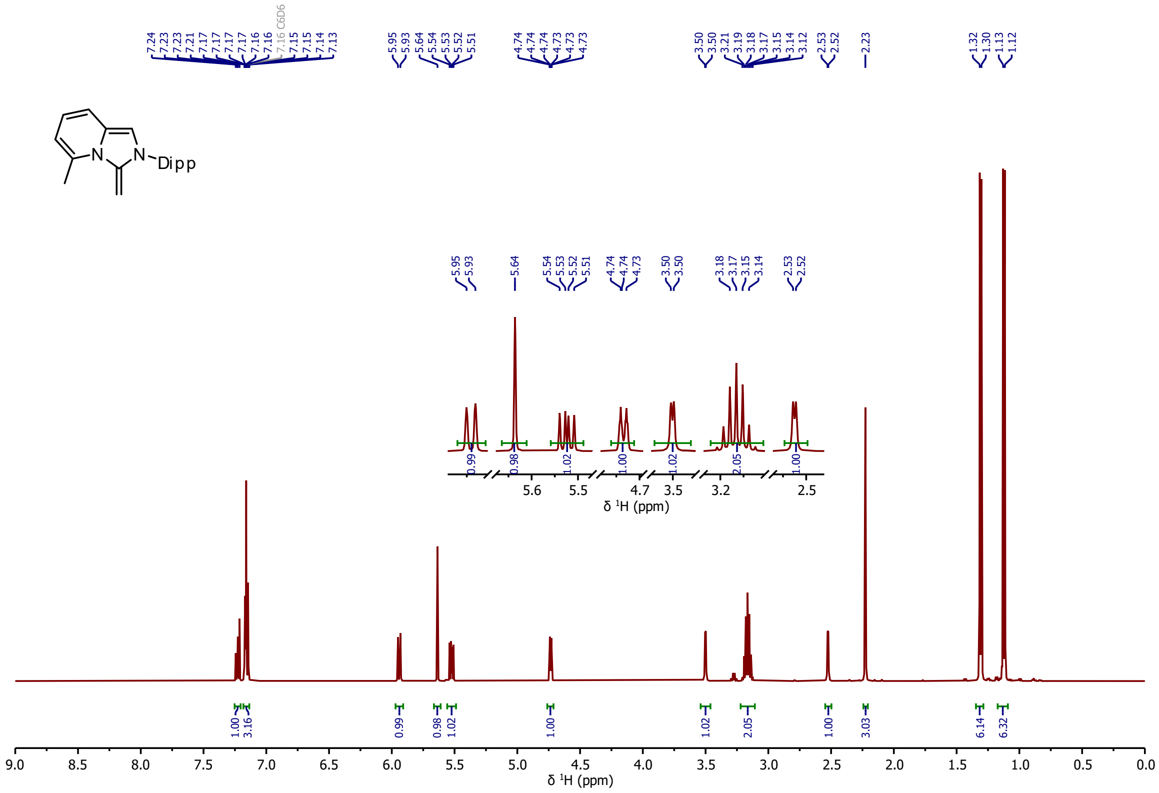


**Figure S276**: **$^1$H NMR** (500 MHz, $C_6D_6$, 298 K) of **2c**.

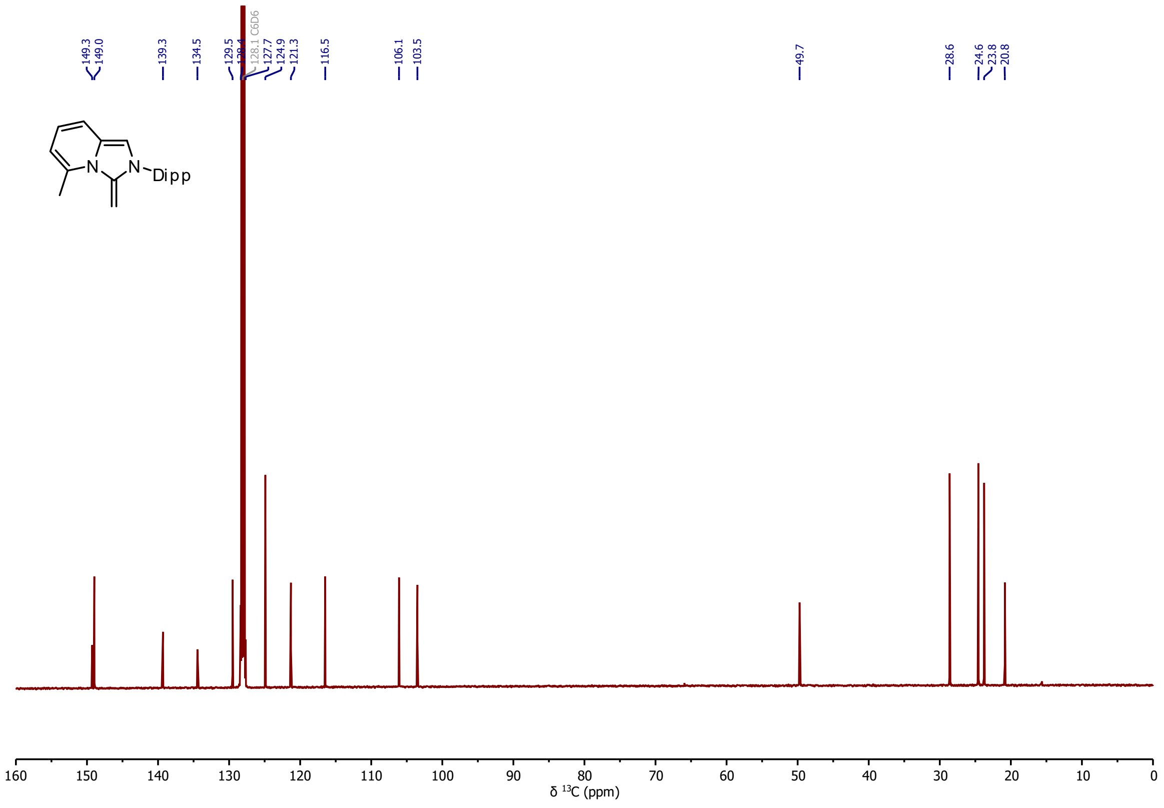


**Figure S277**: **$^{13}$C NMR** (126 MHz, $C_6D_6$, 298 K) of **2c**.

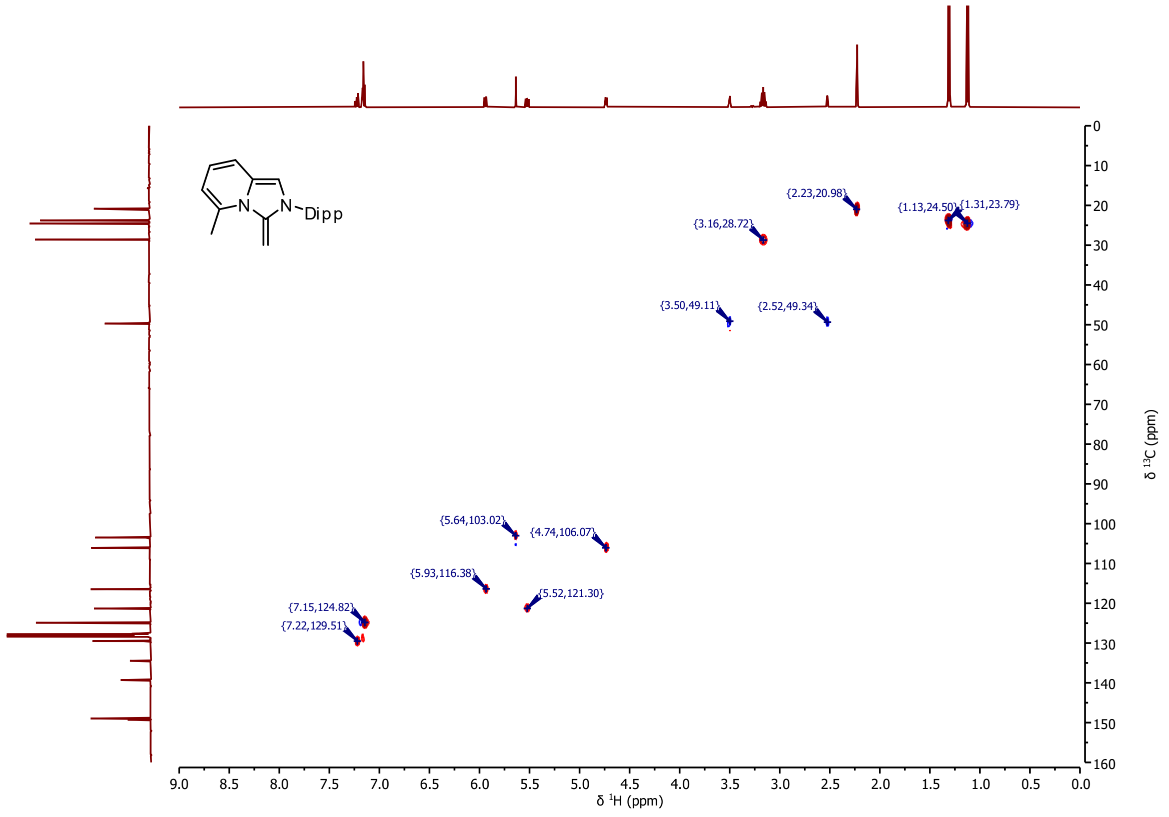


**Figure S278**: **$^{1}$H/$^{13}$C HSQC** (500/126 MHz, $C_6D_6$, 298 K) of **2c**.

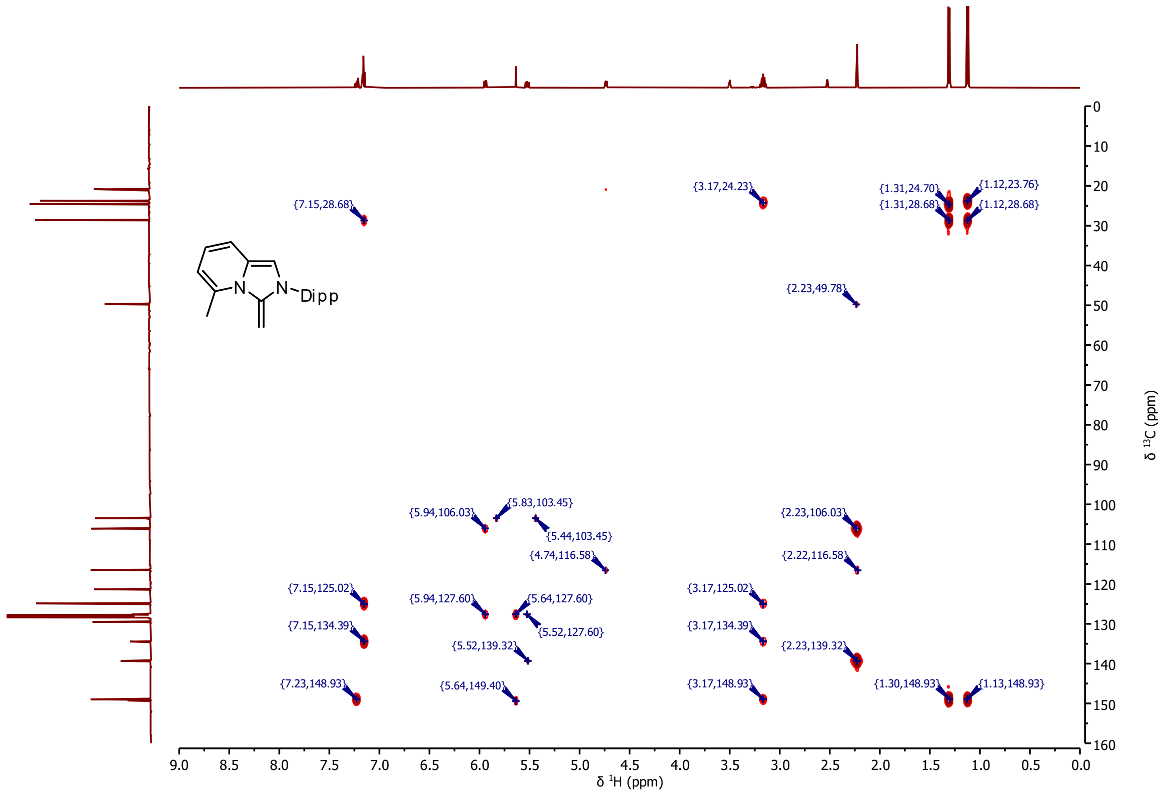

**Figure S279**: **$^{1}$H/$^{13}$C HMBC** (500/126 MHz, $C_6D_6$, 298 K) of **2c**.

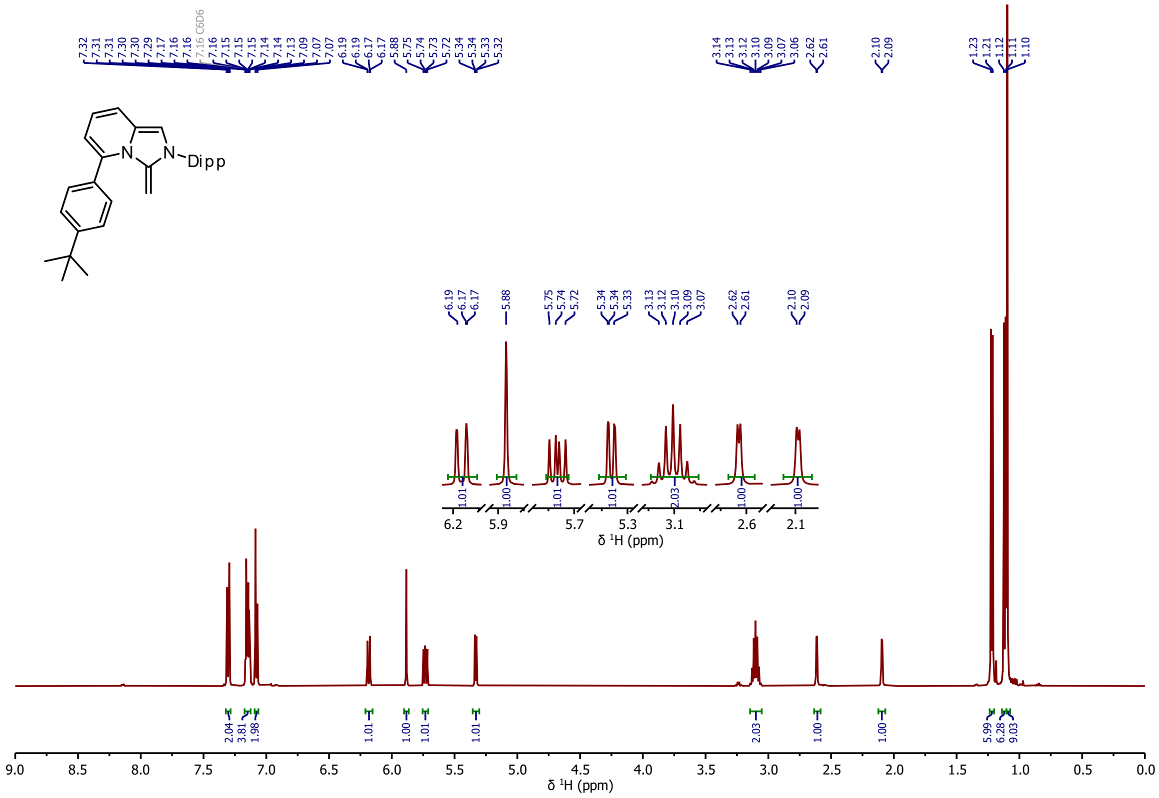

**Figure S280**: **$^{1}$H NMR** (500 MHz, $C_6D_6$, 298 K) of **2d**.

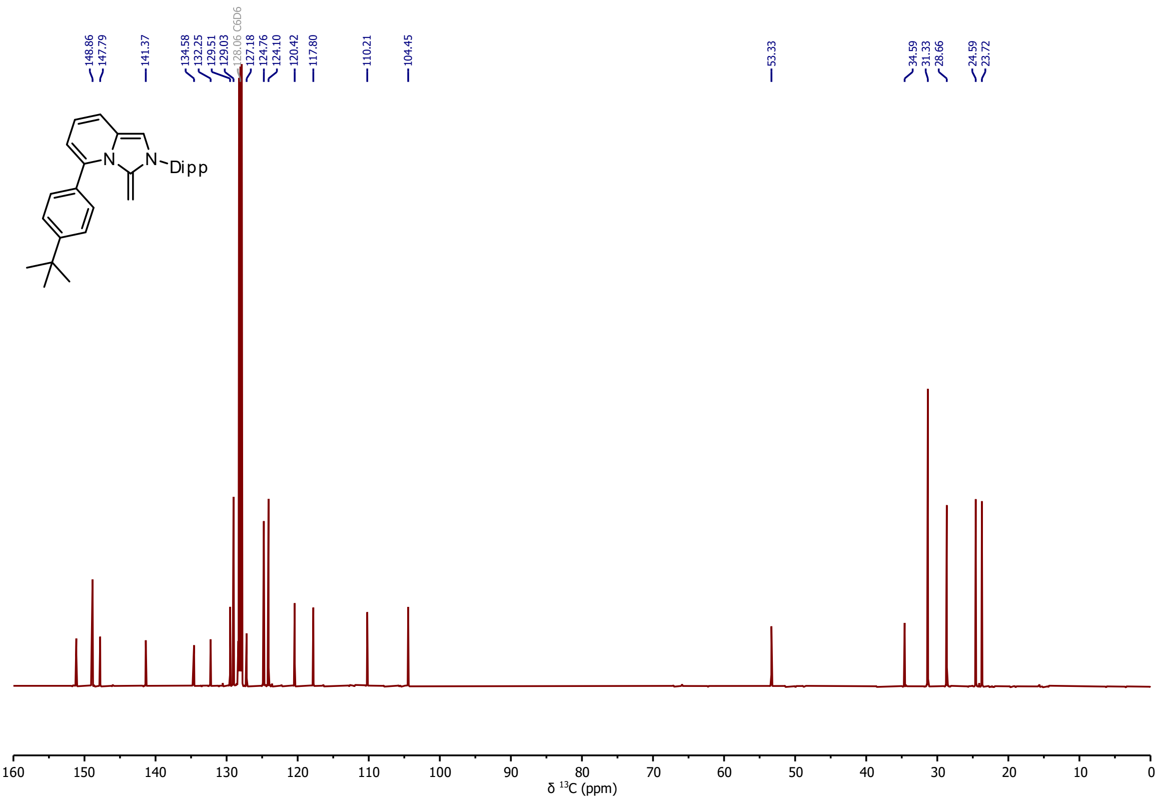

**Figure S281**: $^{13}$**C NMR** (126 MHz, $C_6D_6$, 298 K) of **2d**.

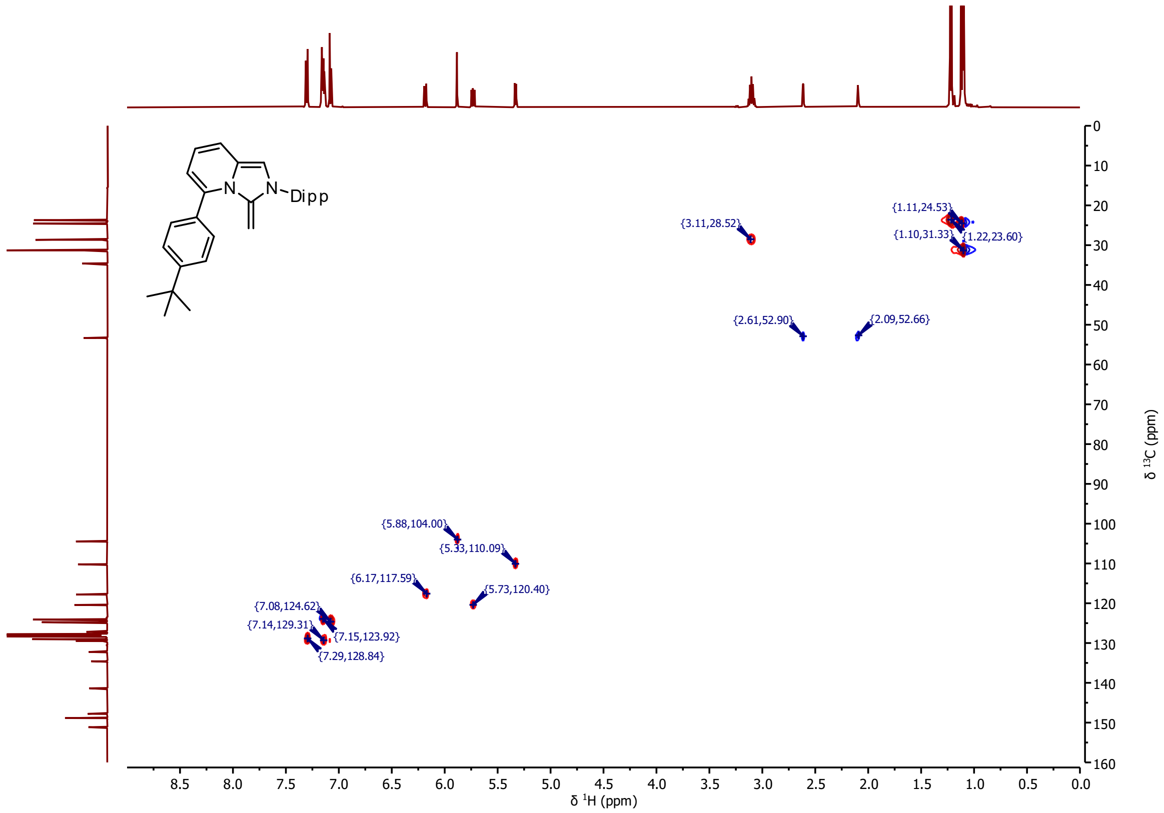

**Figure S282**: $^{1}$**H/**$^{13}$**C HSQC** (500/126 MHz, $C_6D_6$, 298 K) of **2d**.

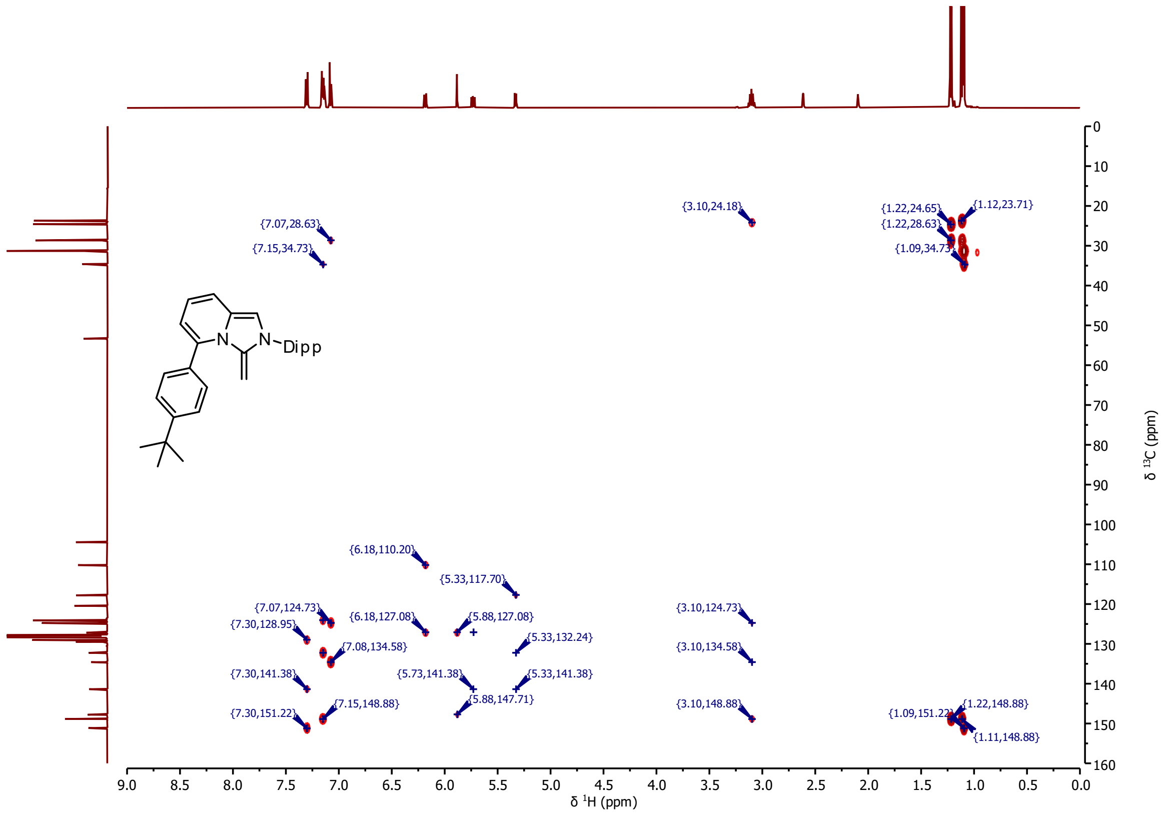

**Figure S283**: $^{1}$**H/$^{13}$C HMBC** (500/126 MHz, $C_6D_6$, 298 K) of **2d**.

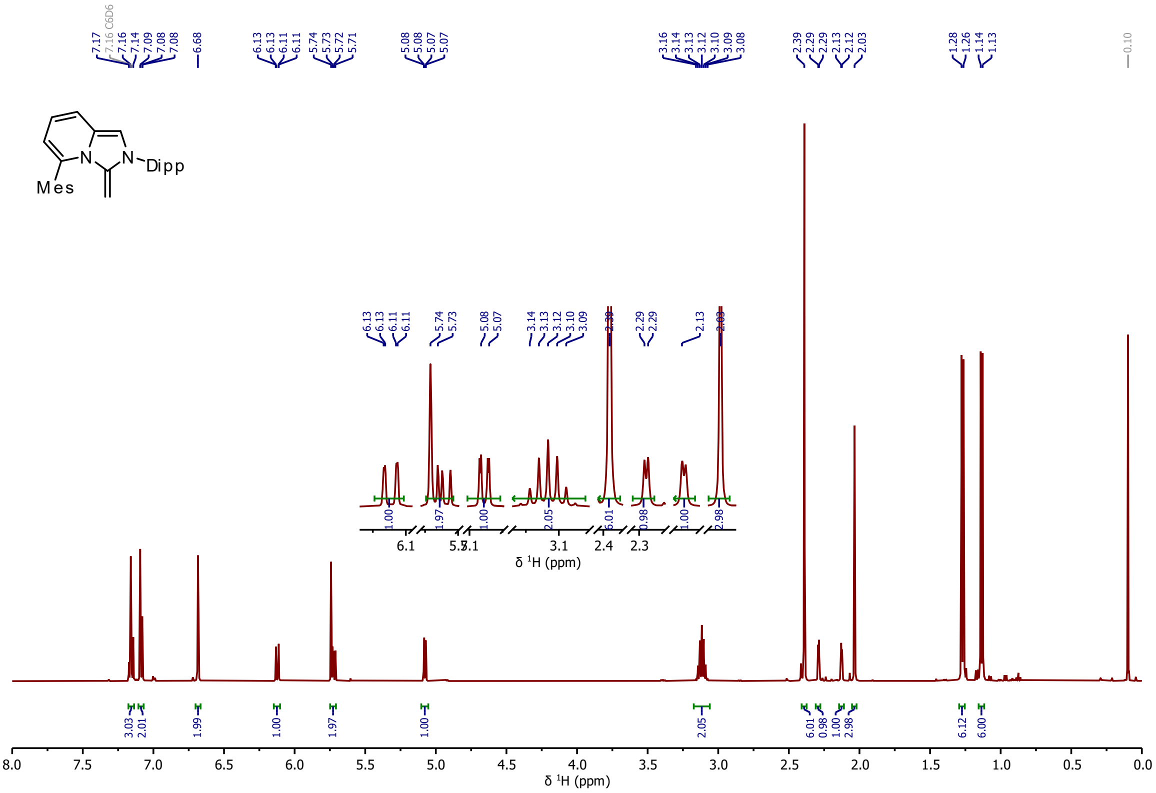

**Figure S284**: $^{1}$**H NMR** (500 MHz, $C_6D_6$, 298 K) of **2e**.

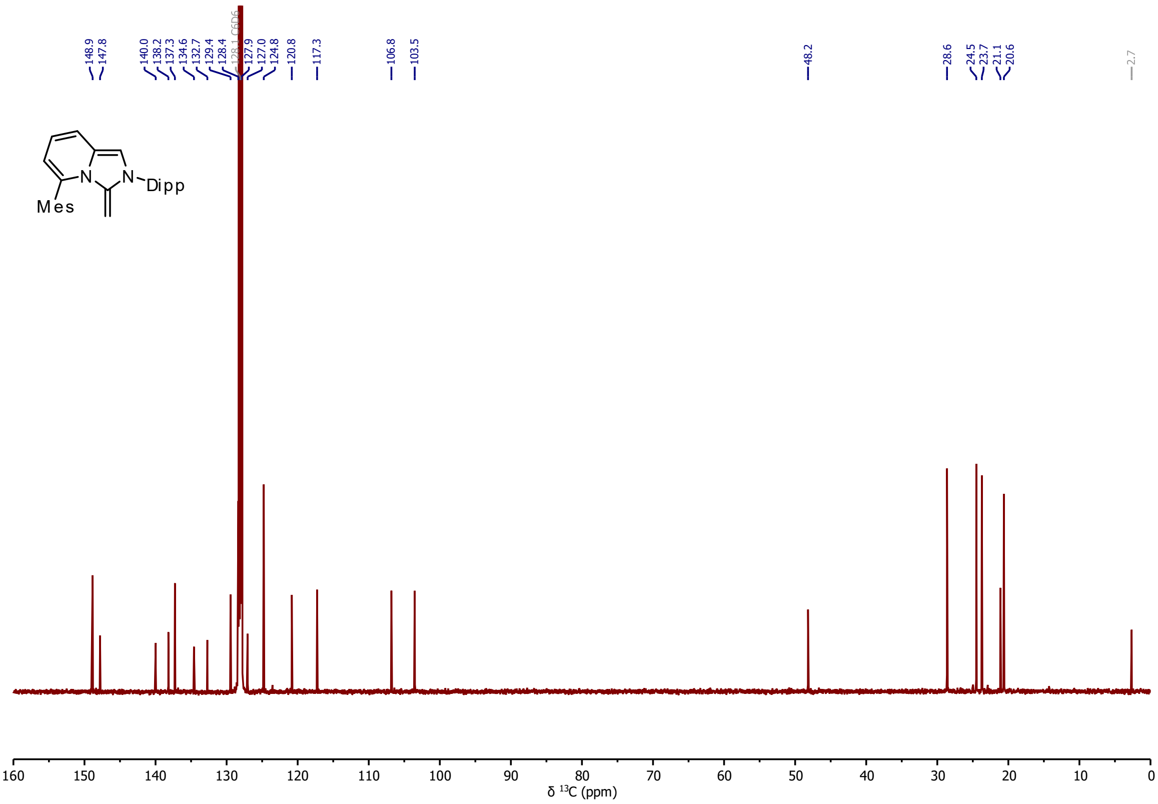


**Figure S285**: **$^{13}$C NMR** (126 MHz, $C_6D_6$, 298 K) of **2e**.

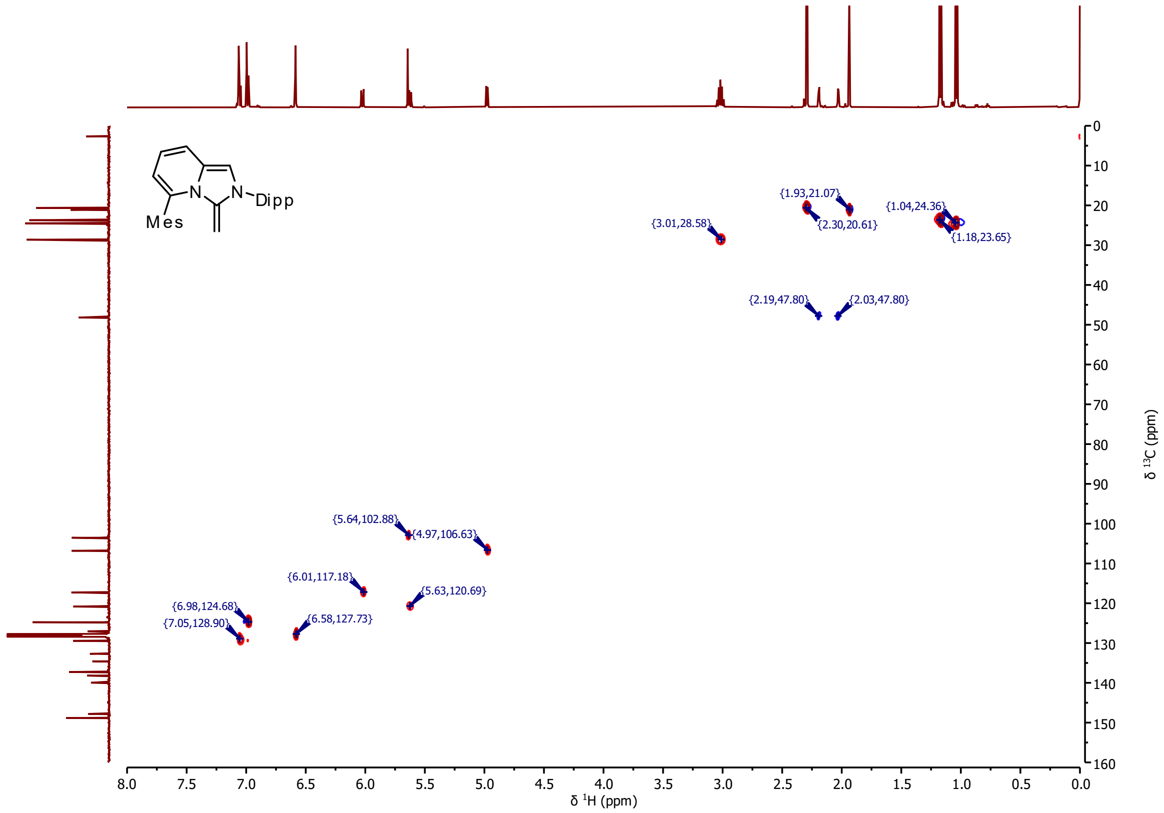


**Figure S286**: **$^{1}$H/$^{13}$C HSQC** (500/126 MHz, $C_6D_6$, 298 K) of **2e**.

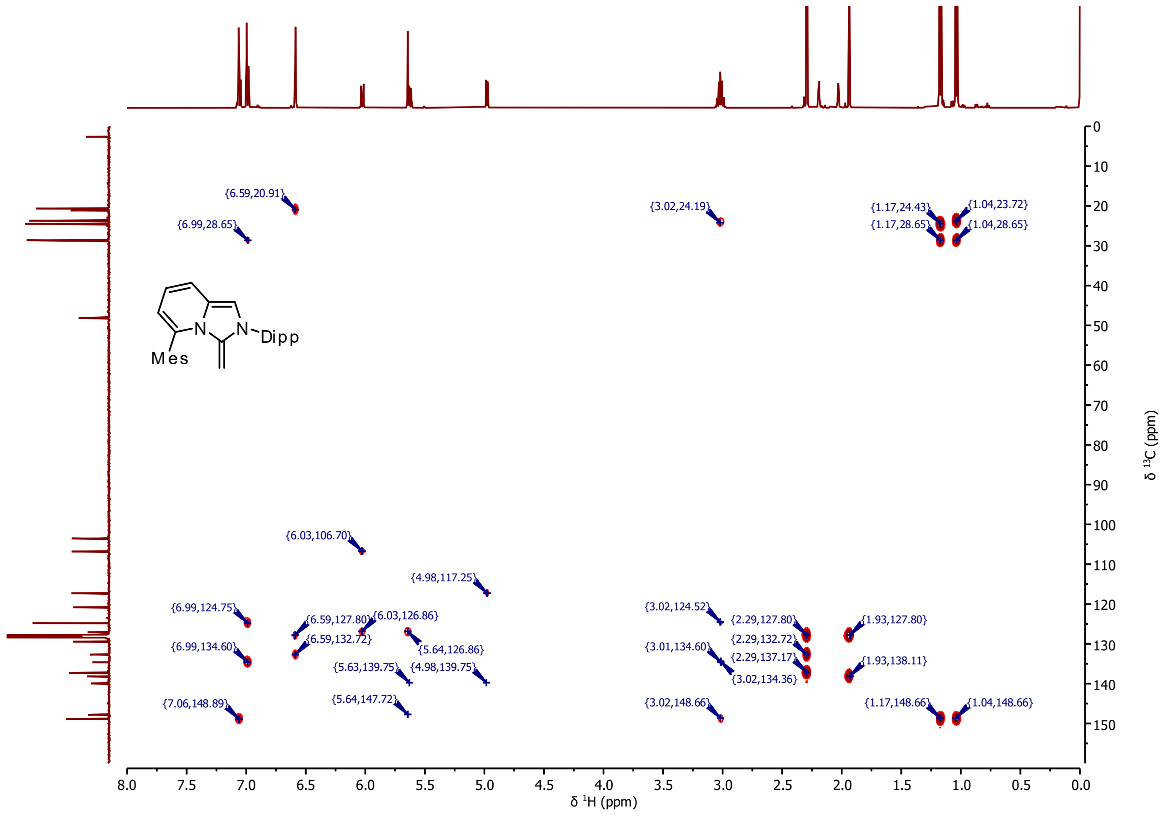

**Figure S287**: **$^1$H/$^{13}$C HMBC** (500/126 MHz, $C_6D_6$, 298 K) of **2e**.

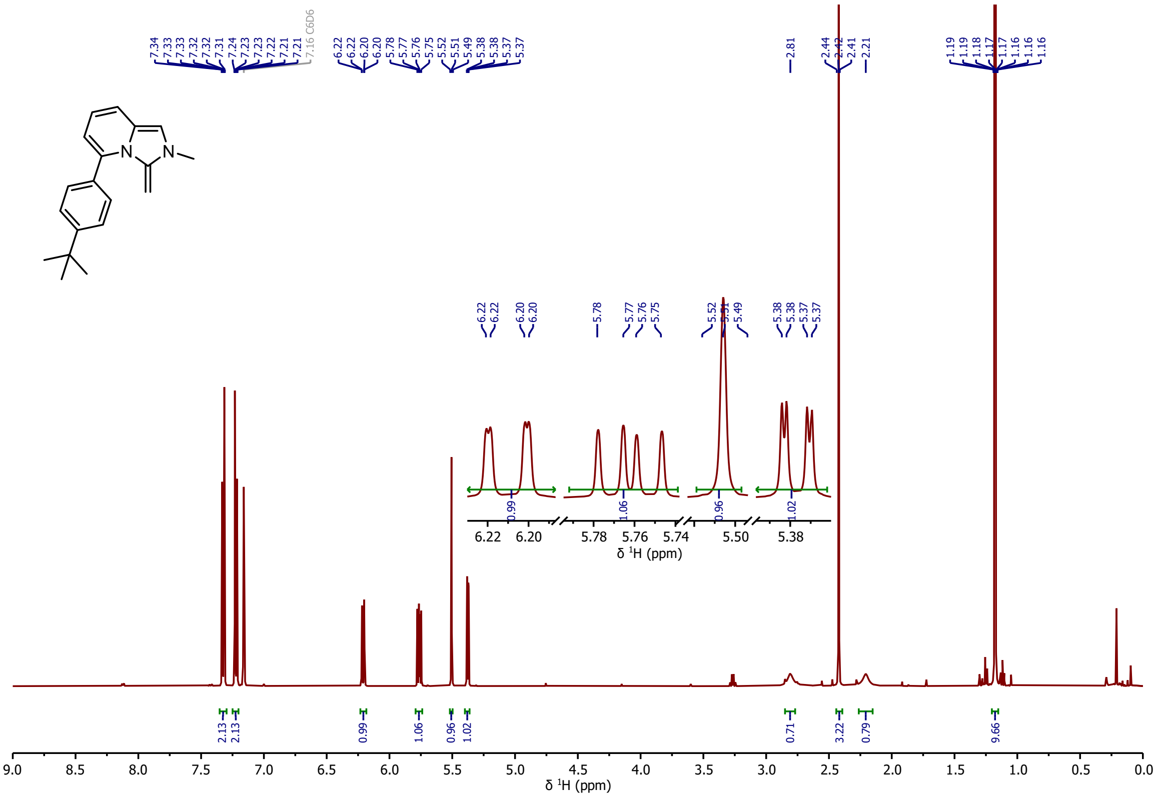

**Figure S288**: **$^1$H NMR** (500 MHz, $C_6D_6$, 298 K) of **2f**.

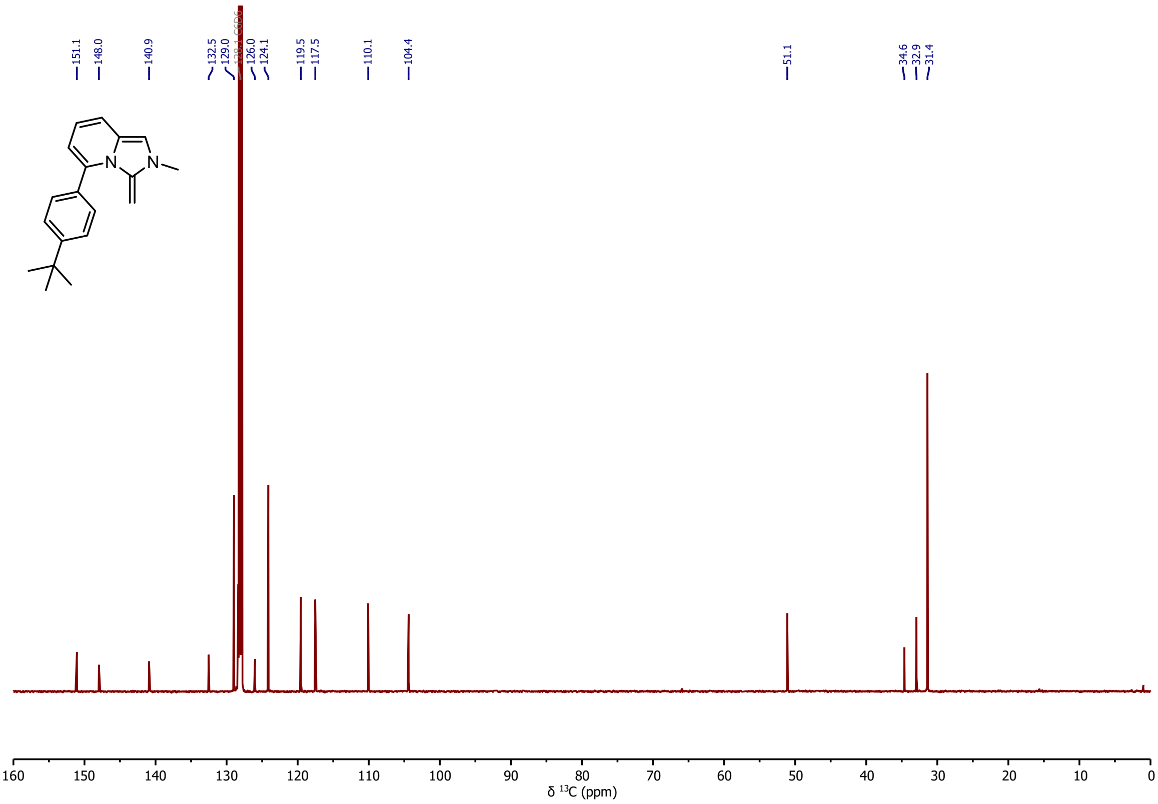

**Figure S289**: **$^{13}$C NMR** (126 MHz, $C_6D_6$, 298 K) of **2f**.

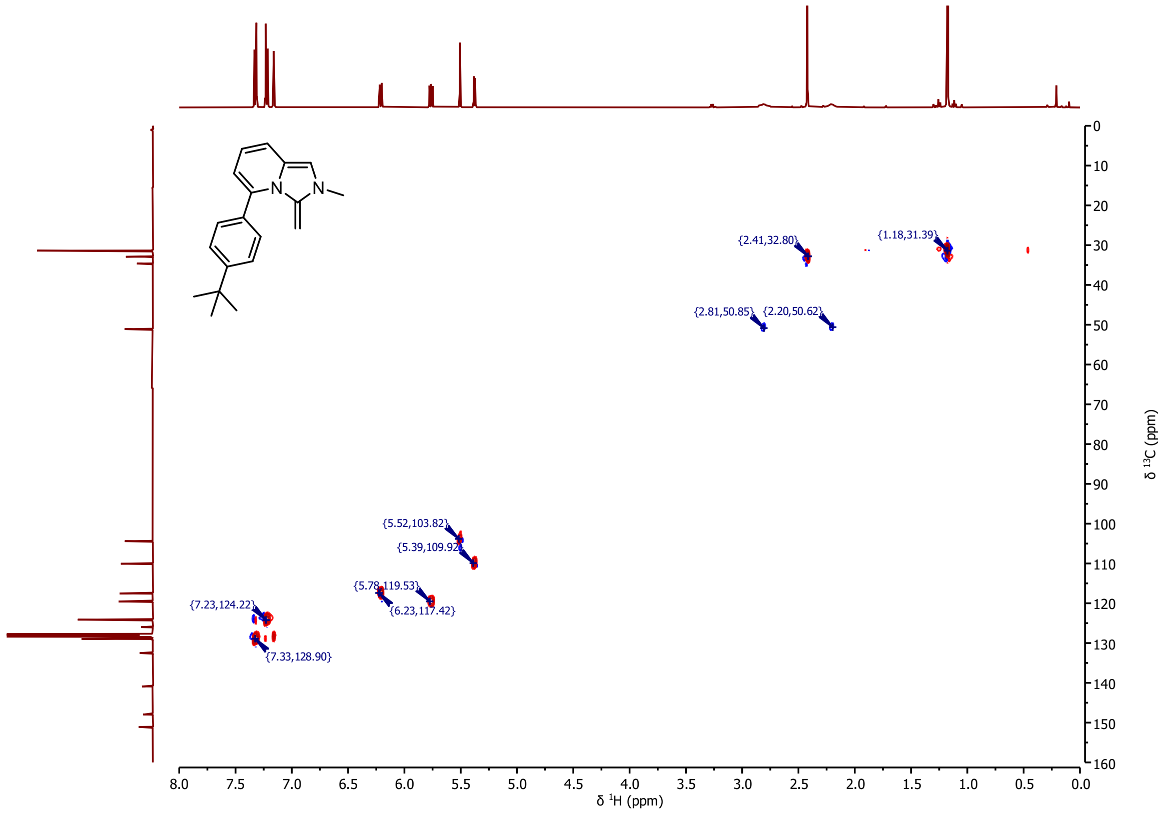

**Figure S290**: **$^{1}$H/$^{13}$C HSQC** (500/126 MHz, $C_6D_6$, 298 K) of **2f**.

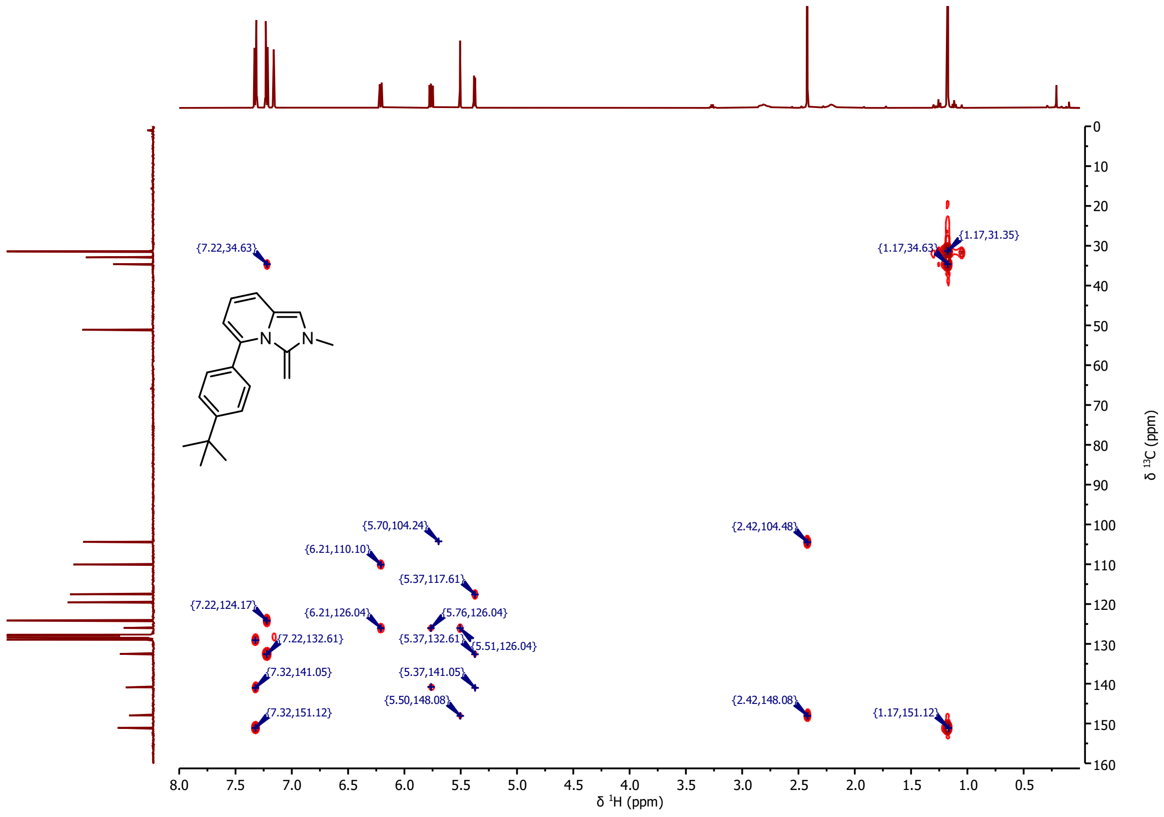

**Figure S291**: **$^{1}$H/$^{13}$C HMBC** (500/126 MHz, $C_6D_6$, 298 K) of **2f**.

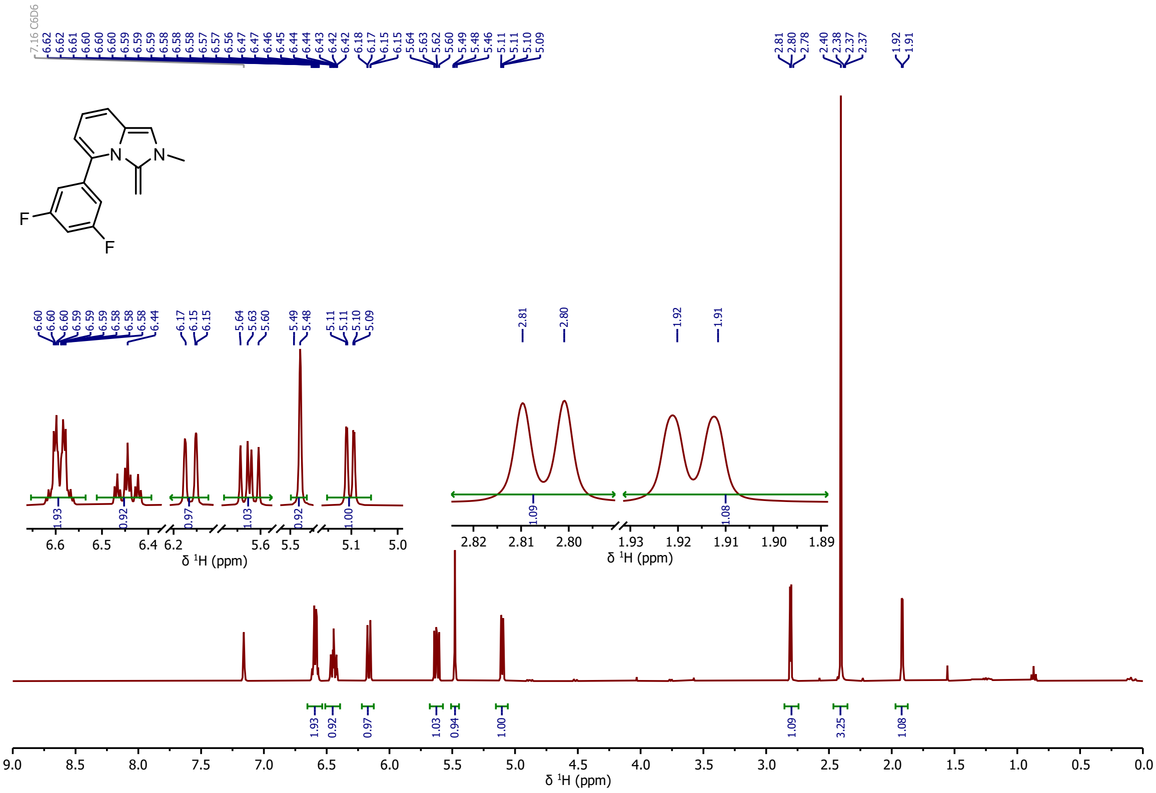

**Figure S292**: **$^{1}$H NMR** (400 MHz, $C_6D_6$, 298 K) of **2g**.

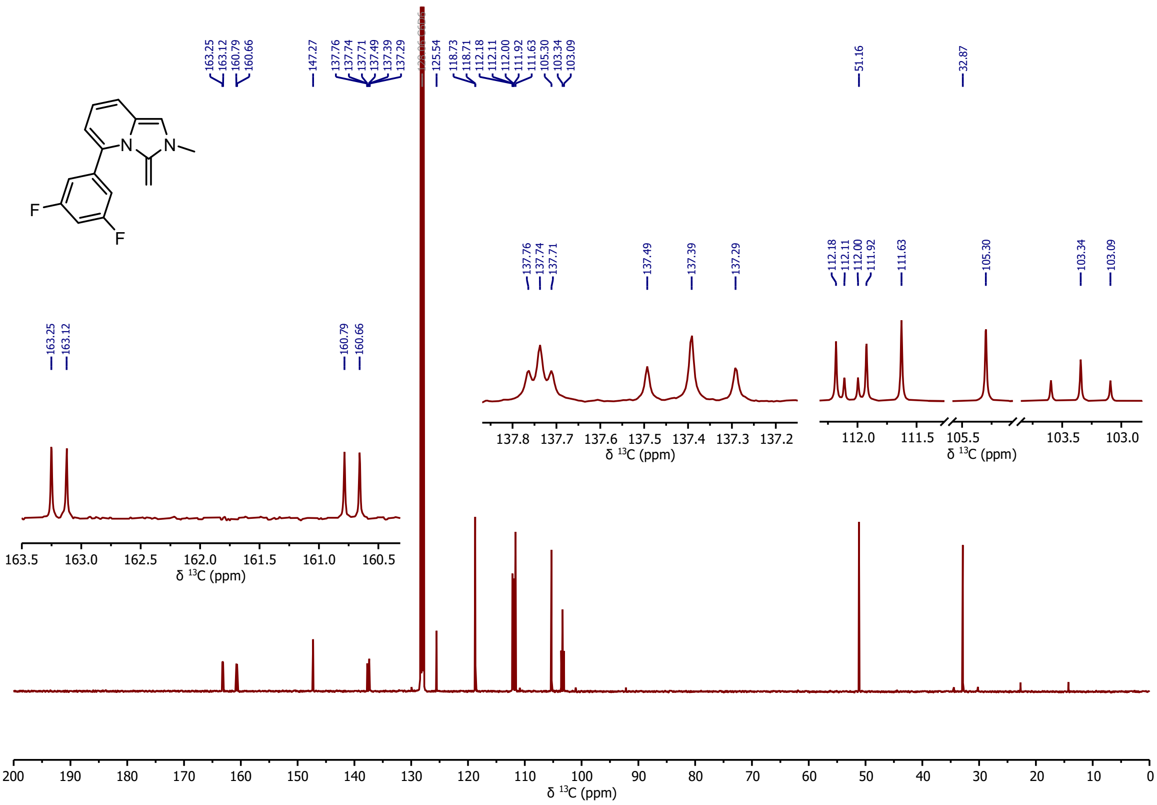

**Figure S293**: **$^{13}$C NMR** (101 MHz, $C_6D_6$, 298 K) of **2g**.

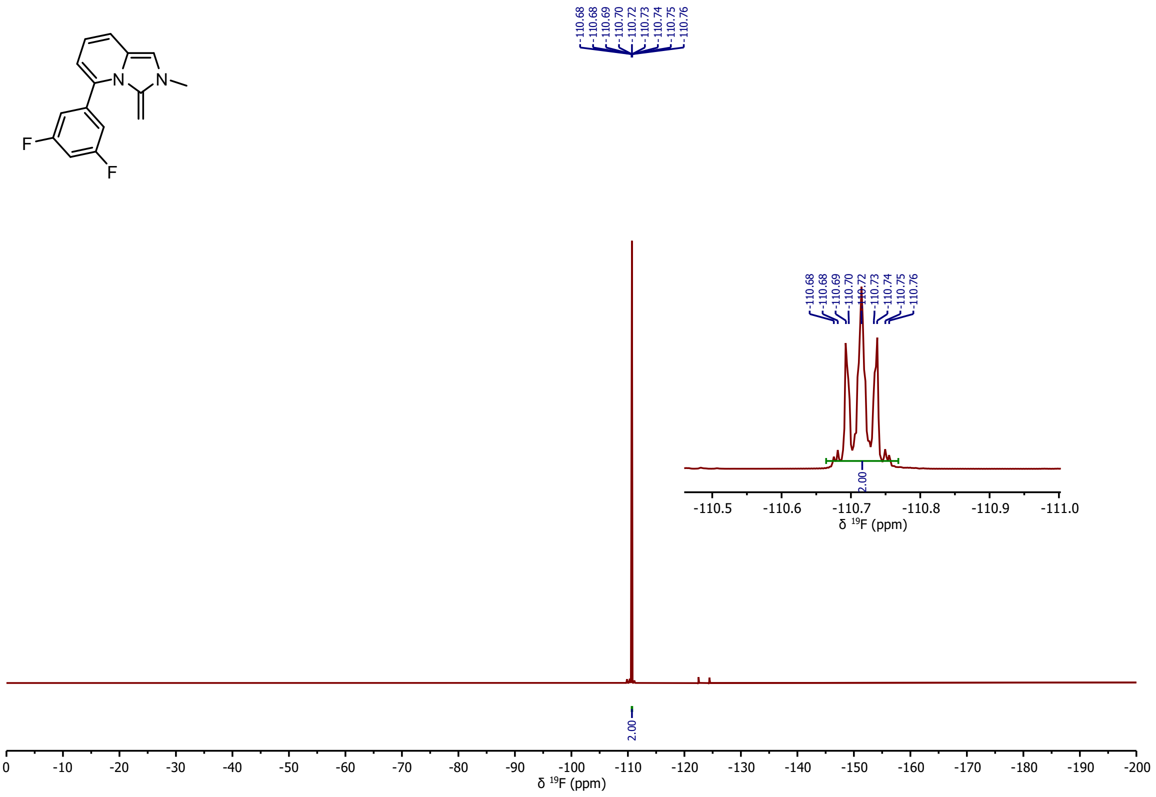

**Figure S294**: **$^{19}$F NMR** (377 MHz, $C_6D_6$, 298 K) of **2g**.

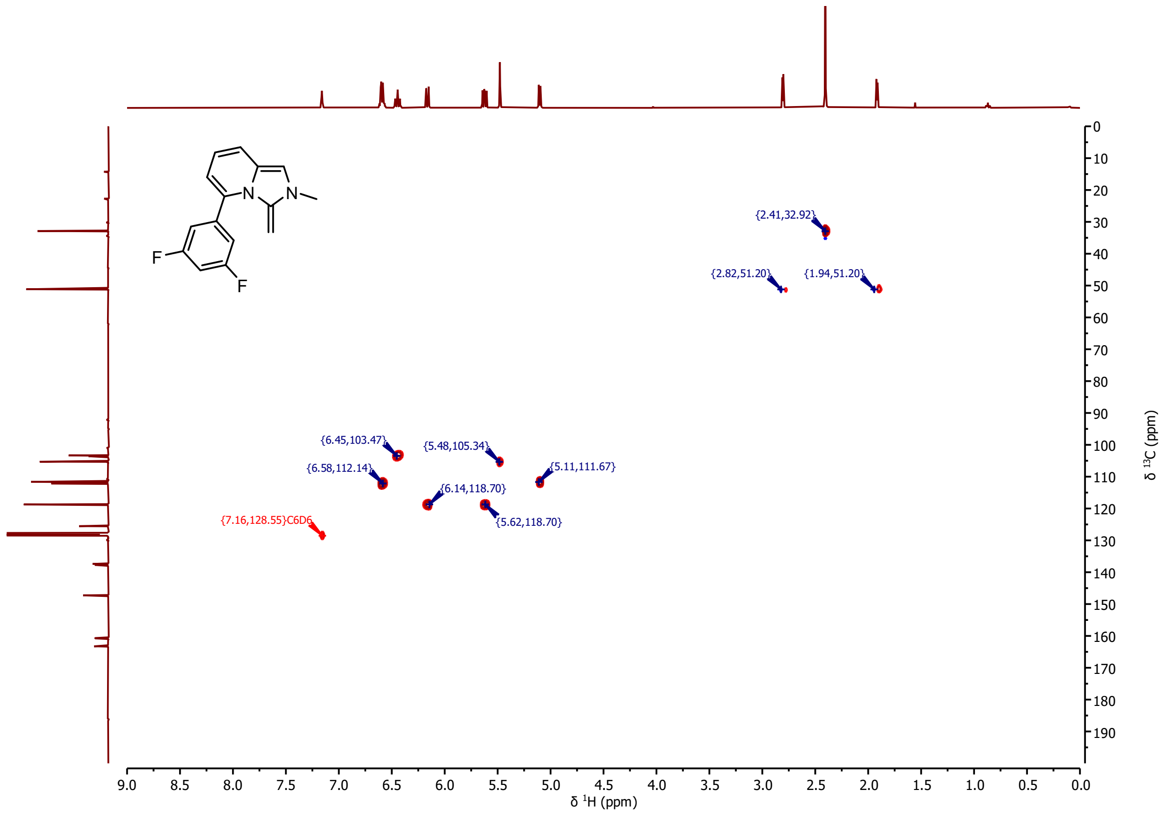


**Figure S295**: **$^{1}$H/$^{13}$C HSQC** (400/101 MHz, $C_6D_6$, 298 K) of **2g**.

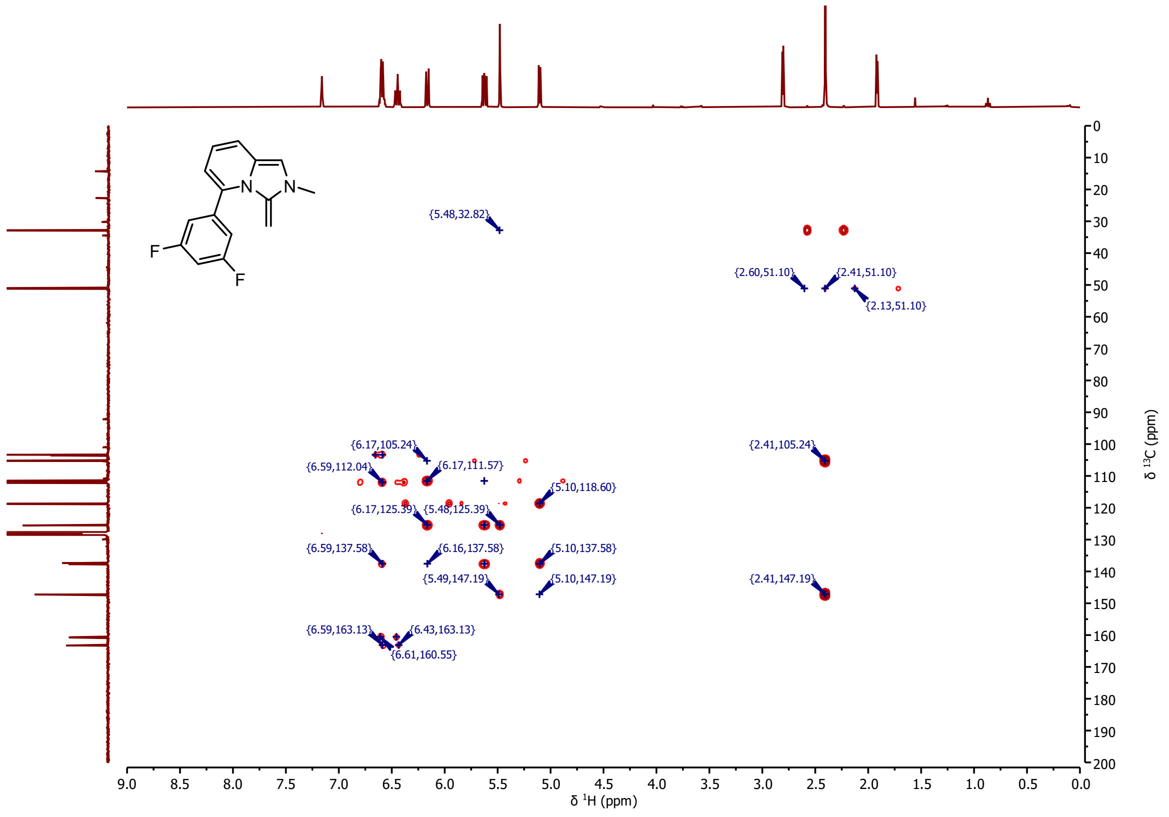


**Figure S296**: **$^{1}$H/$^{13}$C HMBC** (400/101 MHz, $C_6D_6$, 298 K) of **2g**.

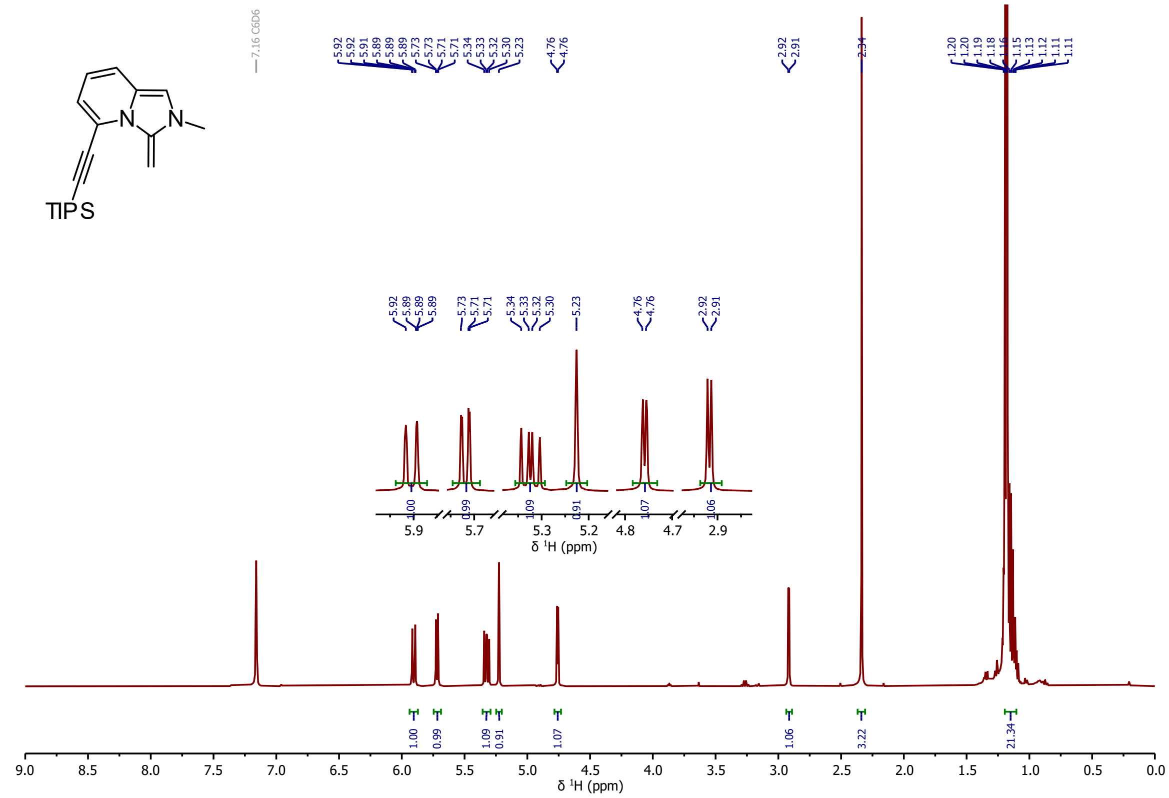


**Figure S297**: **$^{1}$H NMR** (400 MHz, $C_6D_6$, 298 K) of **5b**.

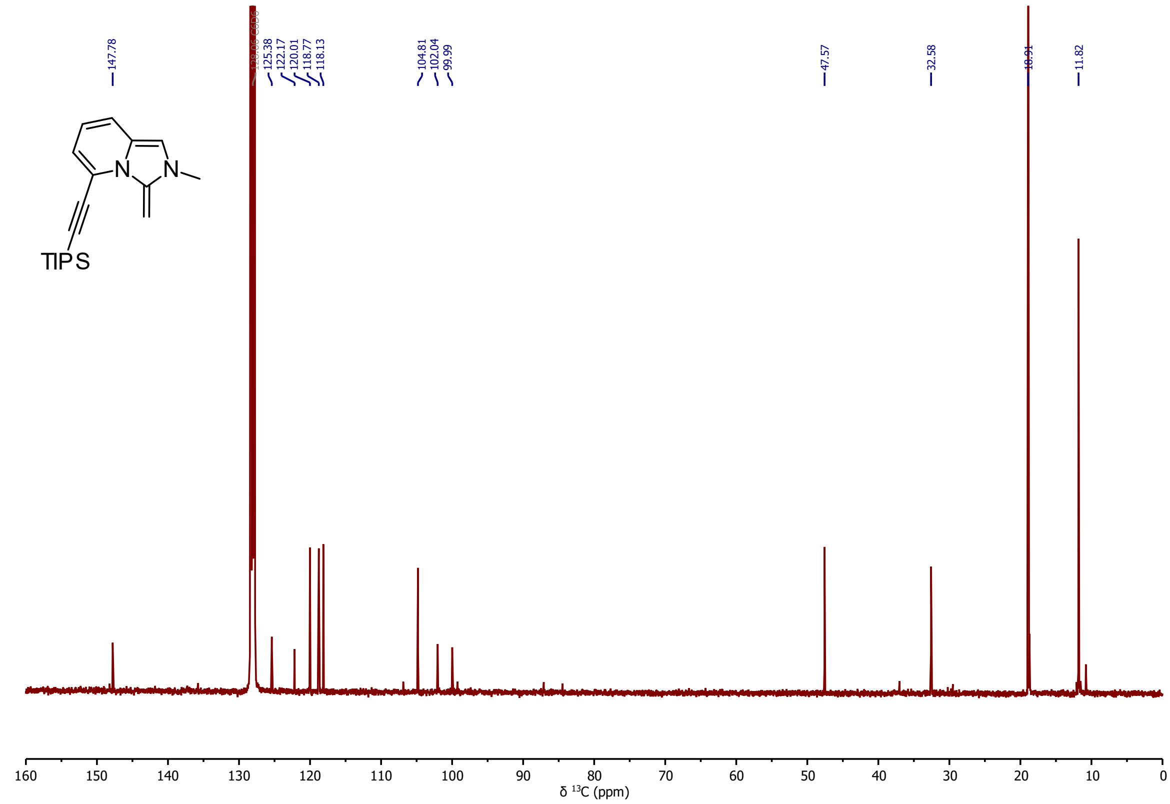


**Figure S298**: **$^{13}$C NMR** (101 MHz, $C_6D_6$, 298 K) of **5b**.

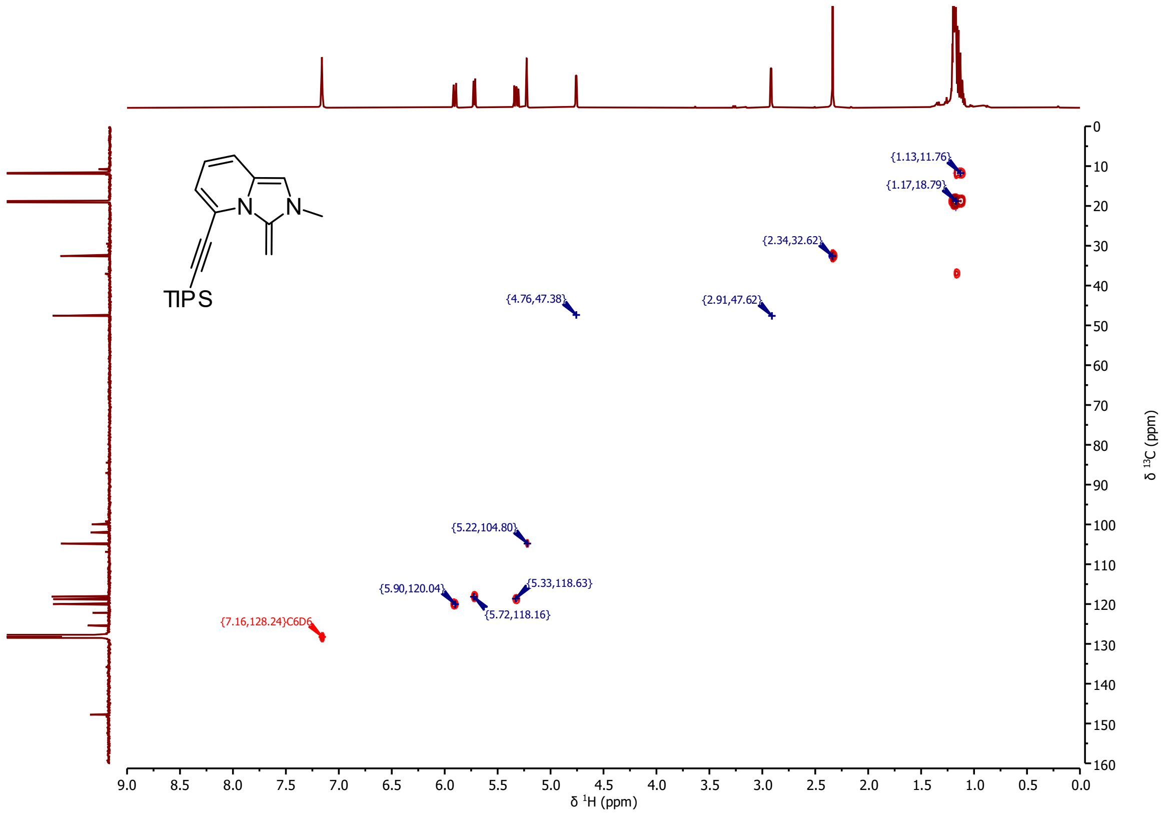


**Figure S299**: **$^{1}H/^{13}C$ HSQC** (400/101 MHz, $C_6D_6$, 298 K) of **5b**.

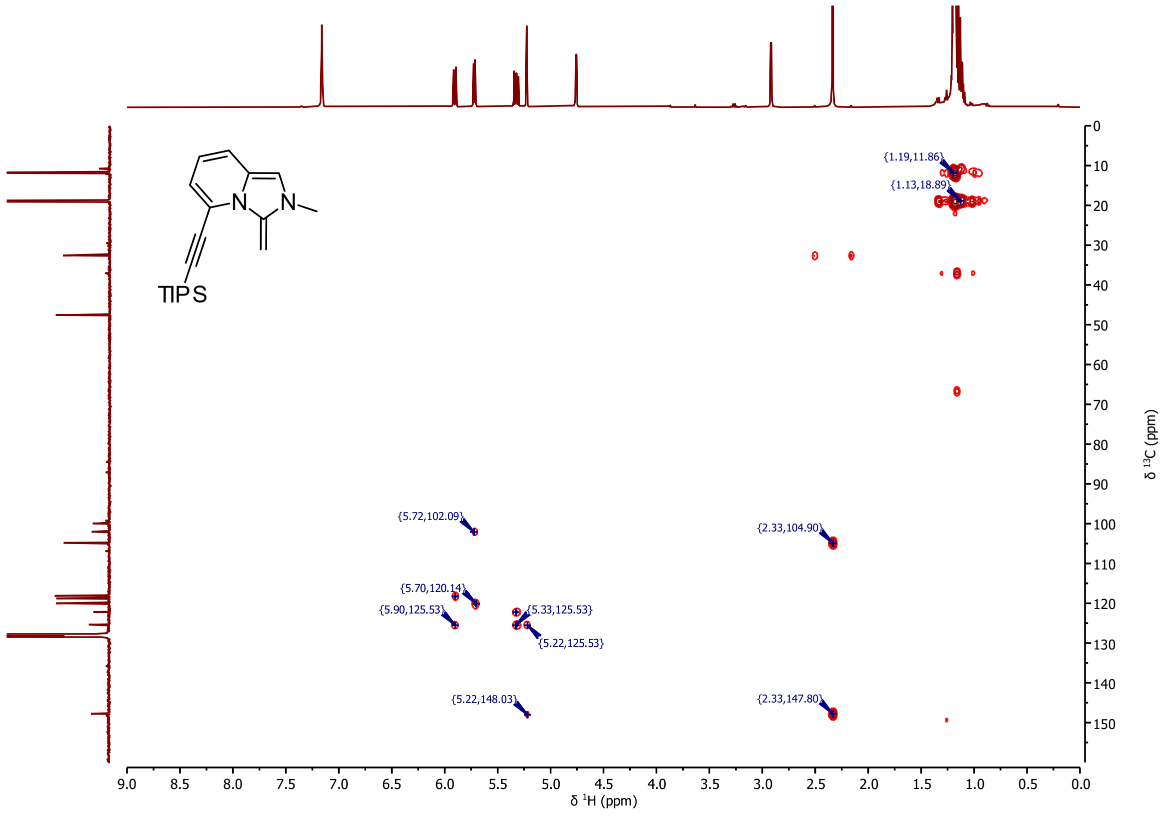


**Figure S300**: **$^{1}H/^{13}C$ HMBC** (400/101 MHz, $C_6D_6$, 298 K) of **5b**.

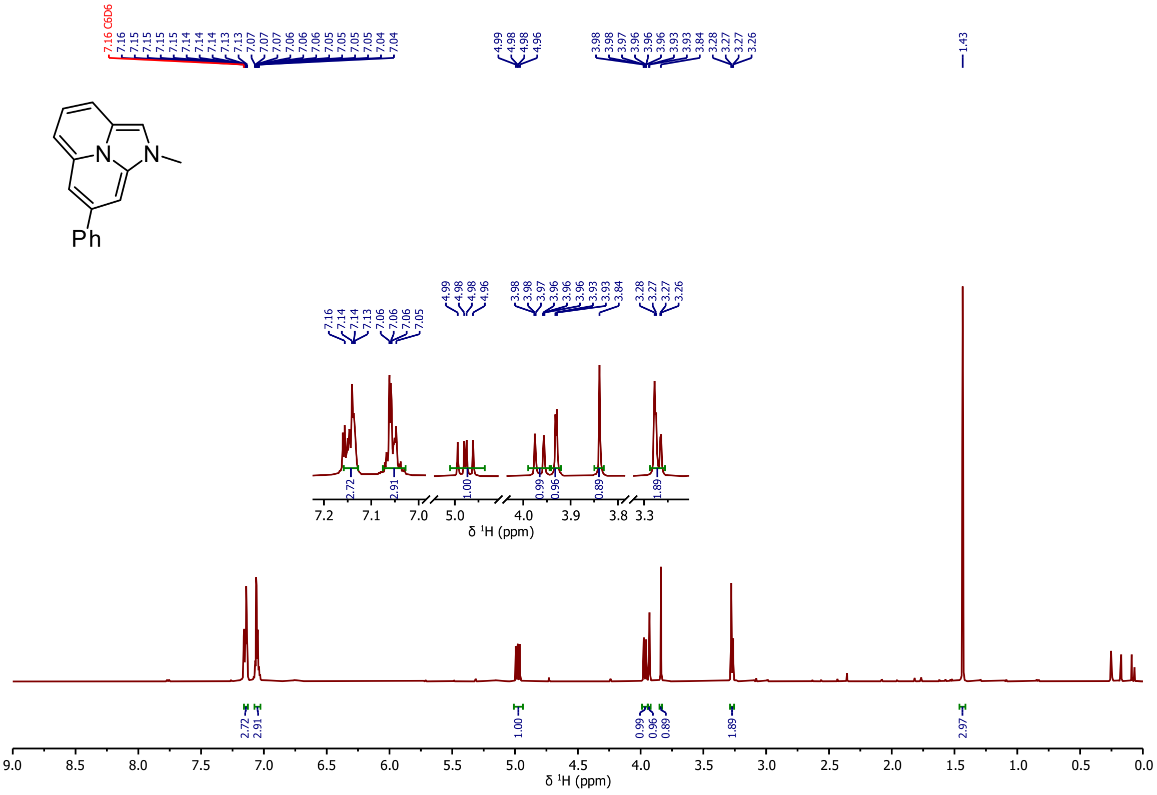


**Figure S301**. **$^{1}$H NMR** (500 MHz, $C_6D_6$, 298 K) of **6a**.

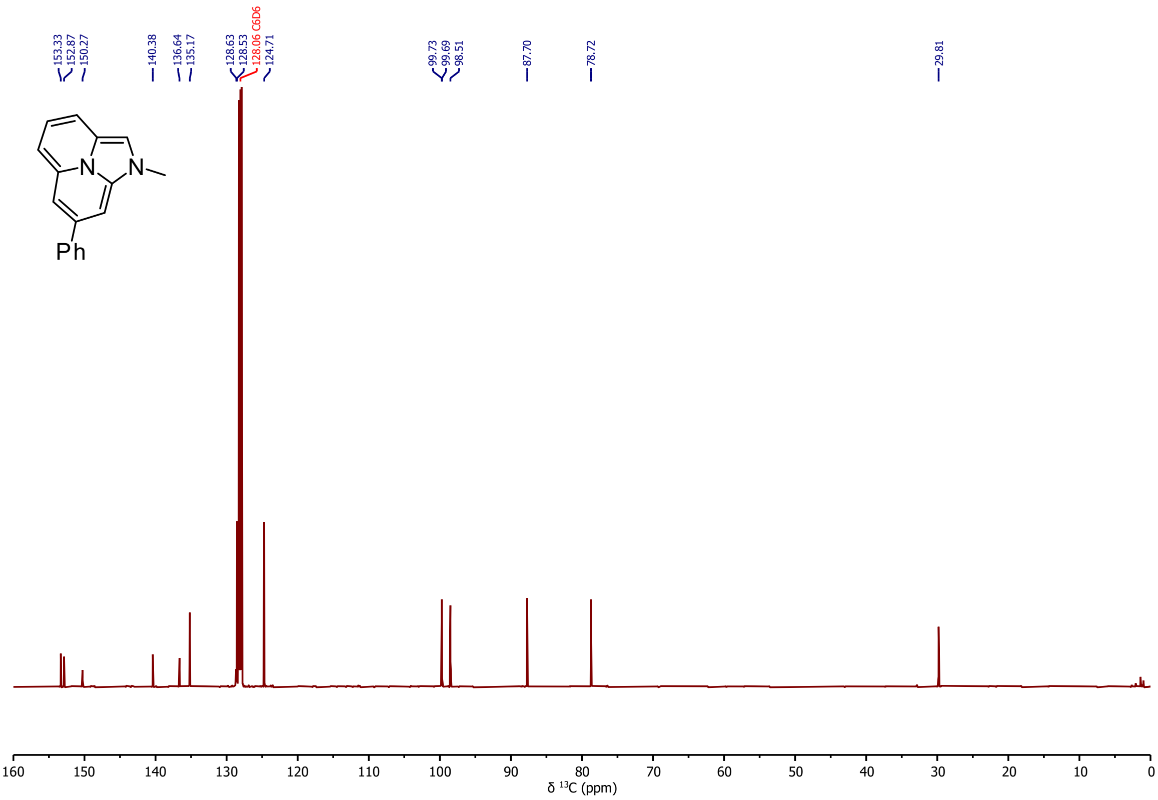


**Figure S302**. **$^{13}$C NMR** (126 MHz, $C_6D_6$, 298 K) of **6a**.

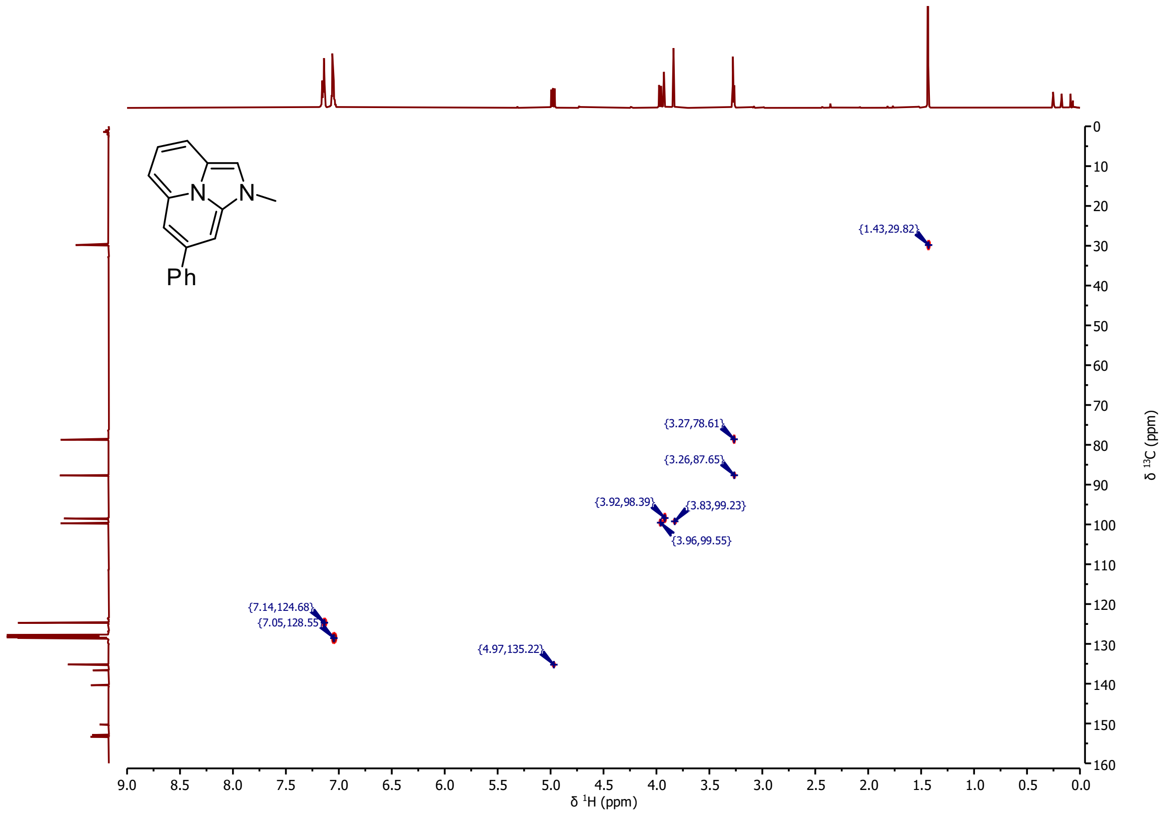

**Figure S303**. $^{1}$**H/**$^{13}$**C HSQC** (500/126 MHz, $C_6D_6$, 298 K) of **6a**.

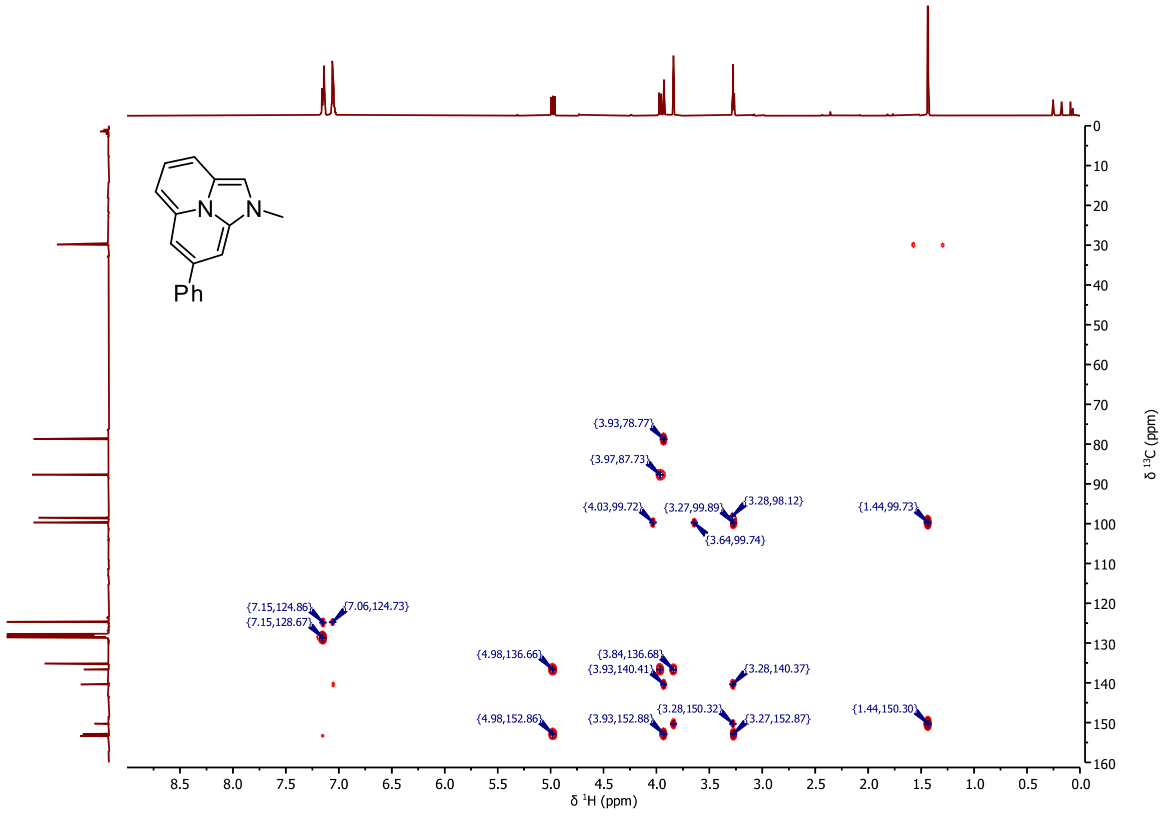

**Figure S304**: $^{1}$**H/**$^{13}$**C HMBC** (500/126 MHz, $C_6D_6$, 298 K) of **6a**.

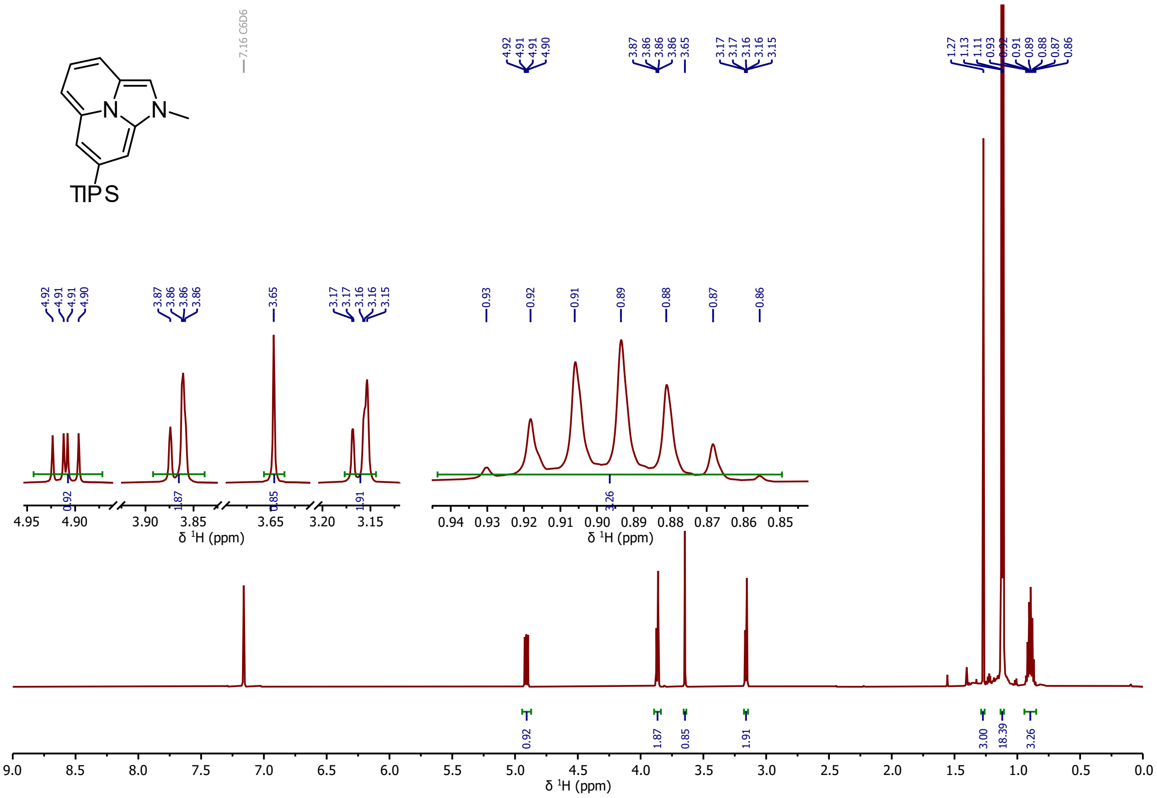

**Figure S305**: **$^1$H NMR** (600 MHz, $C_6D_6$, 298 K) of **6b**.

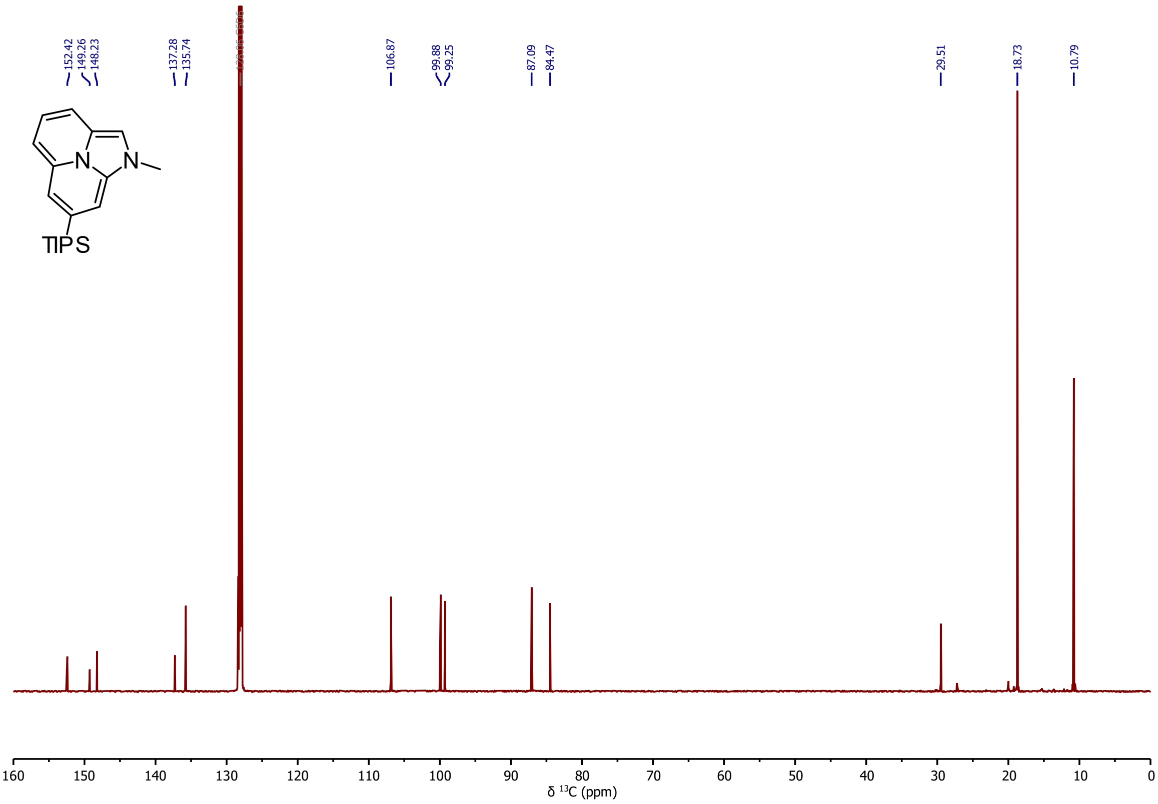

**Figure S306**: **$^{13}$C NMR** (151 MHz, $C_6D_6$, 298 K) of **6b**.

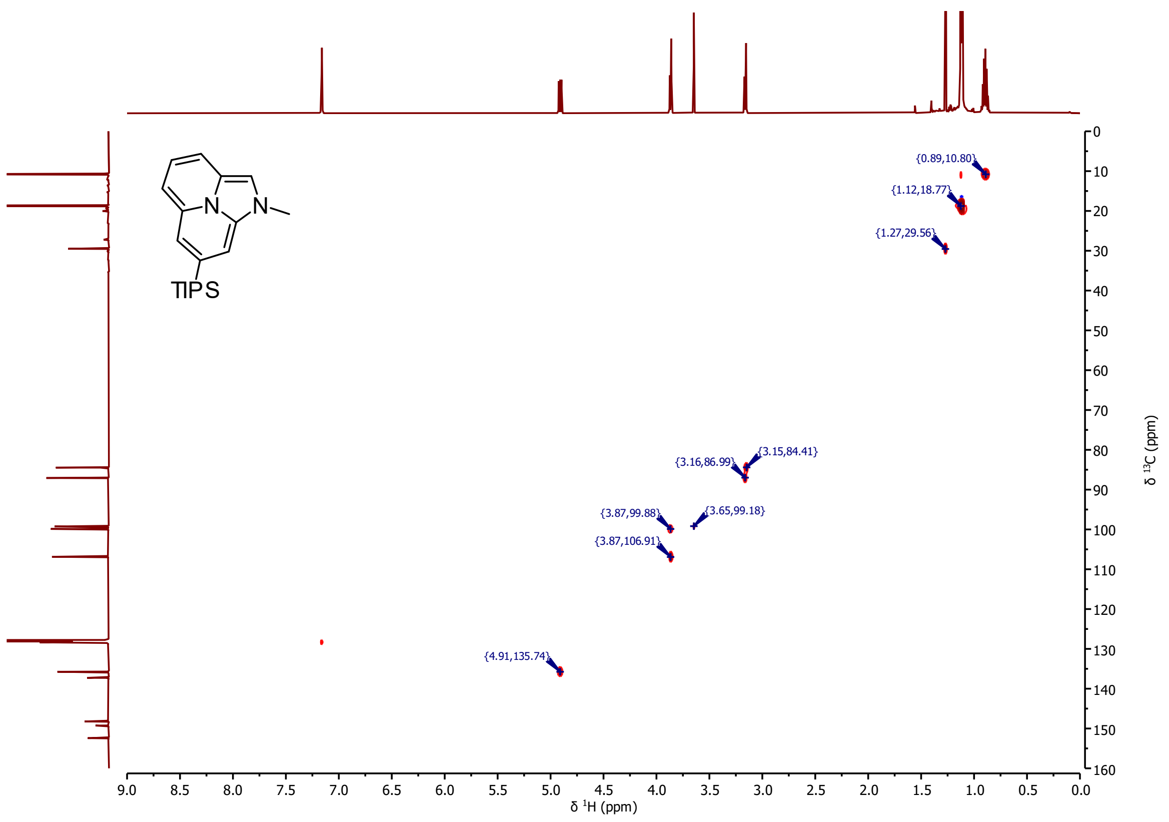

**Figure S307**: $^{1}$**H/**$^{13}$**C HSQC** (600/151 MHz, $C_6D_6$, 298 K) of **6b**.

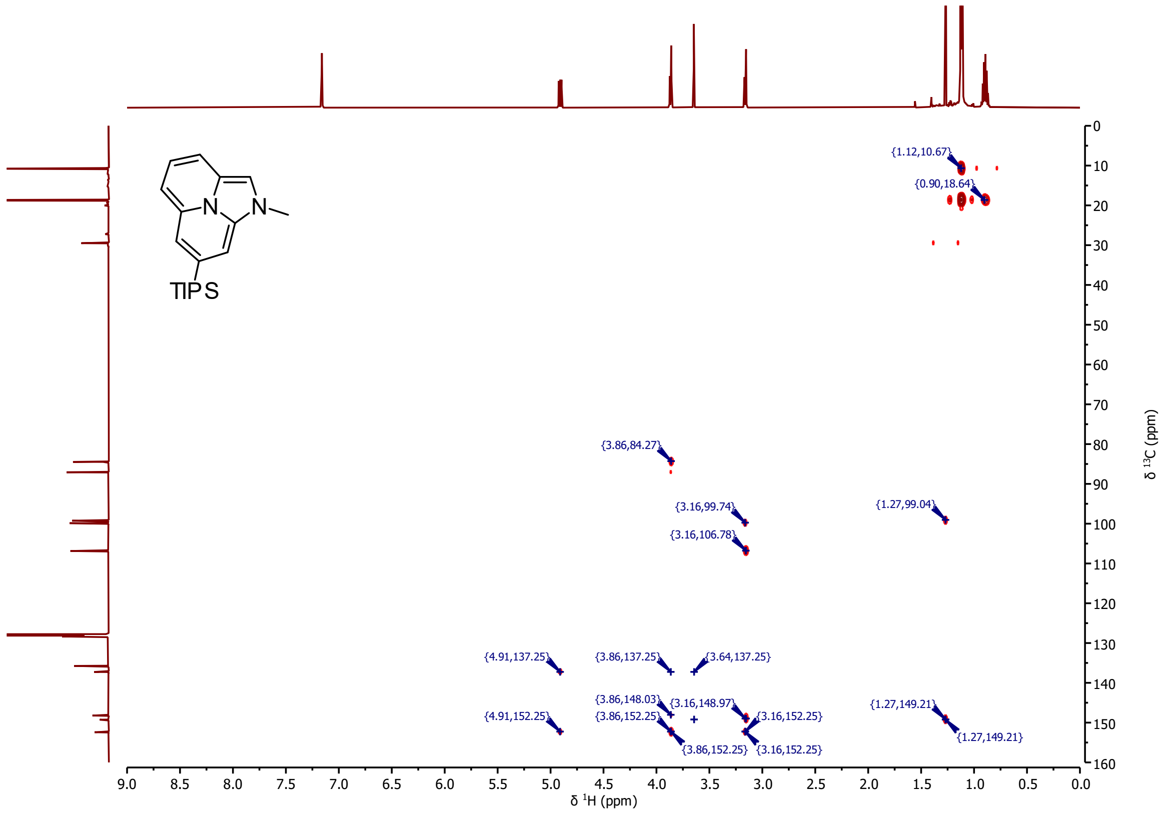

**Figure S308**: $^{1}$**H/**$^{13}$**C HMBC** (600/151 MHz, $C_6D_6$, 298 K) of **6b**.

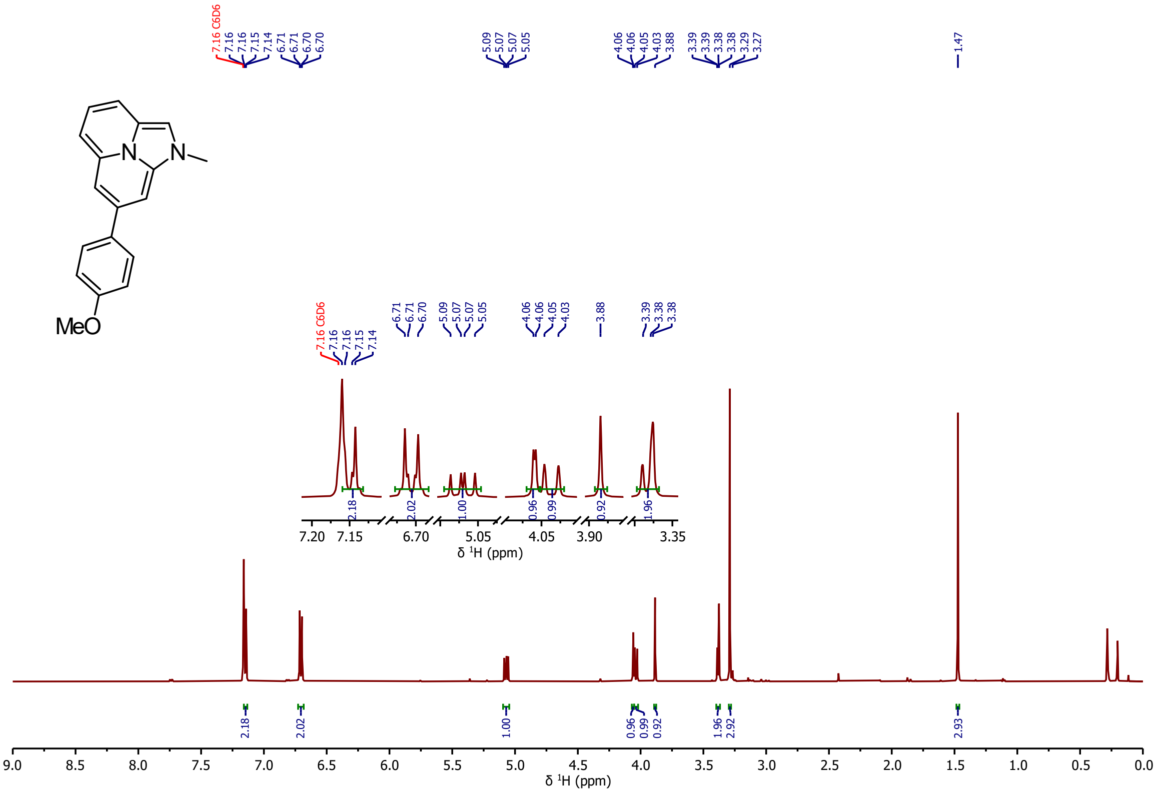

**Figure S309**: **$^1$H NMR** (500 MHz, $C_6D_6$, 298 K) of **6c**.

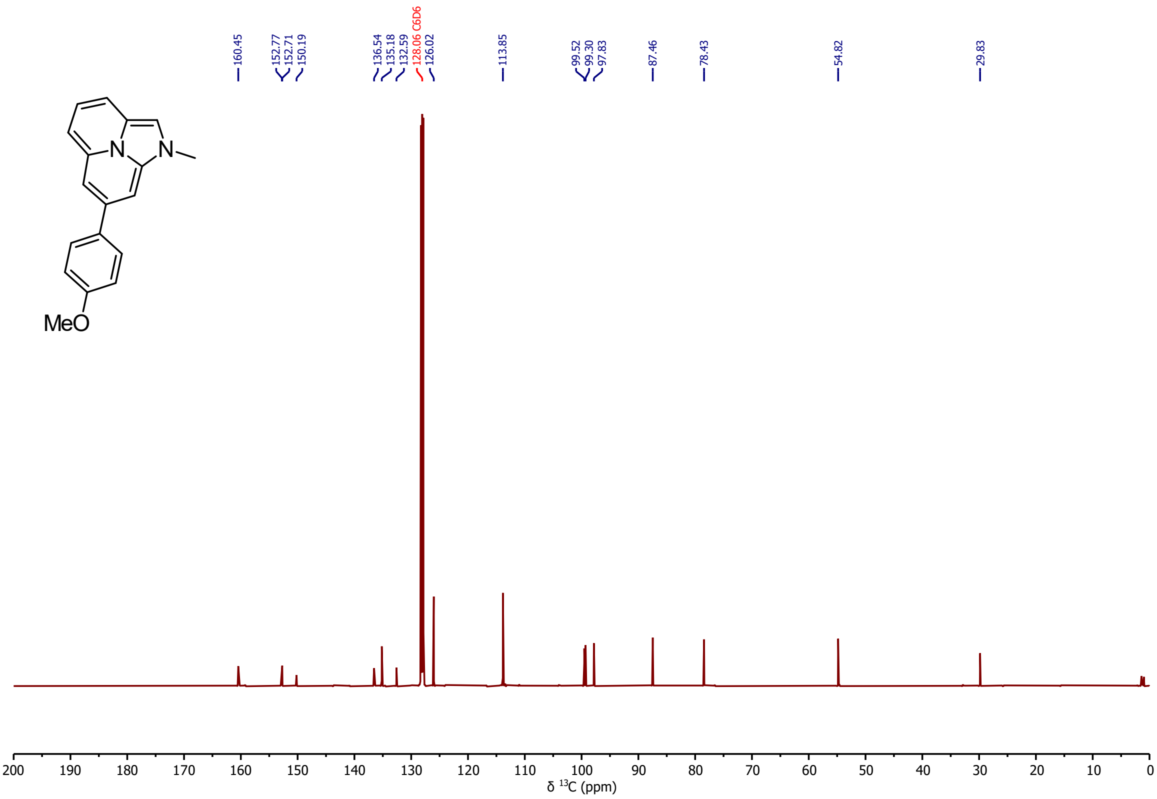

**Figure S310**: **$^{13}$C NMR** (126 MHz, $C_6D_6$, 298 K) of **6c**.

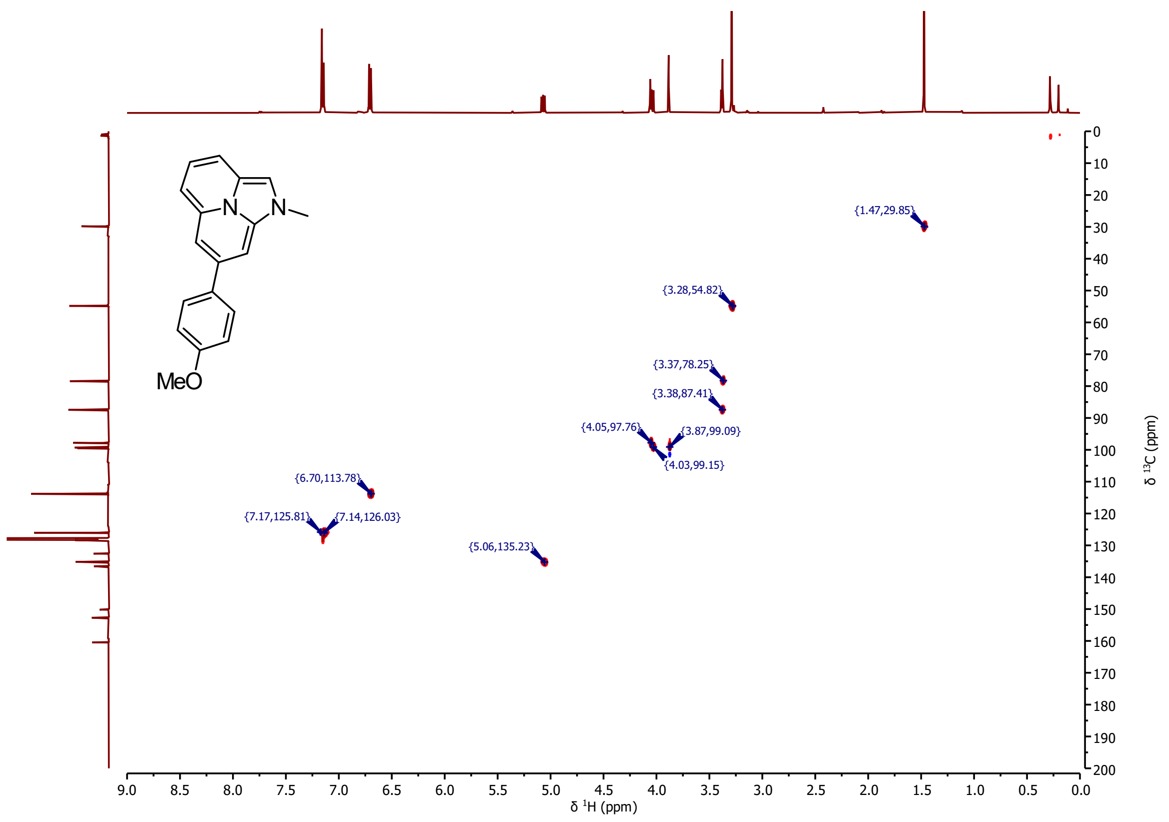

**Figure S311**: **$^{1}$H/$^{13}$C HSQC** (500/126 MHz, $C_6D_6$, 298 K) of **6c**.

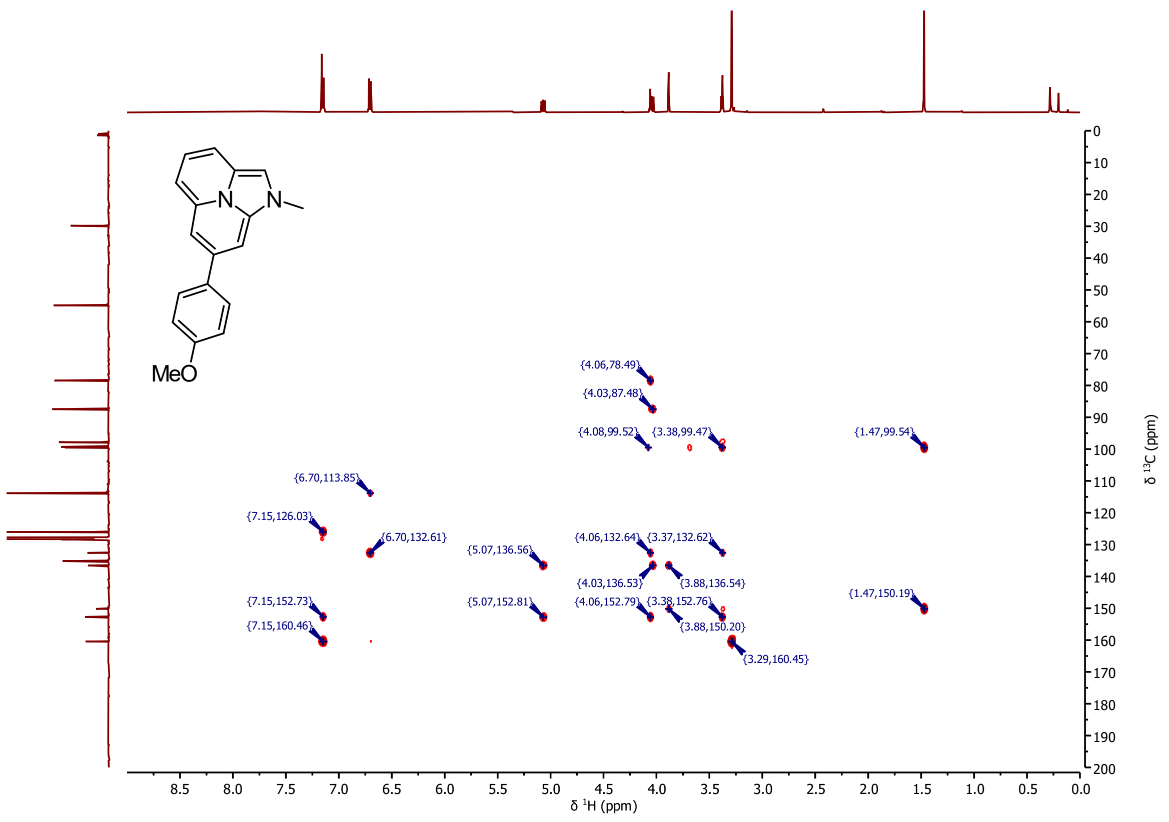

**Figure S312**: **$^{1}$H/$^{13}$C HMBC** (500/126 MHz, $C_6D_6$, 298 K) of **6c**.

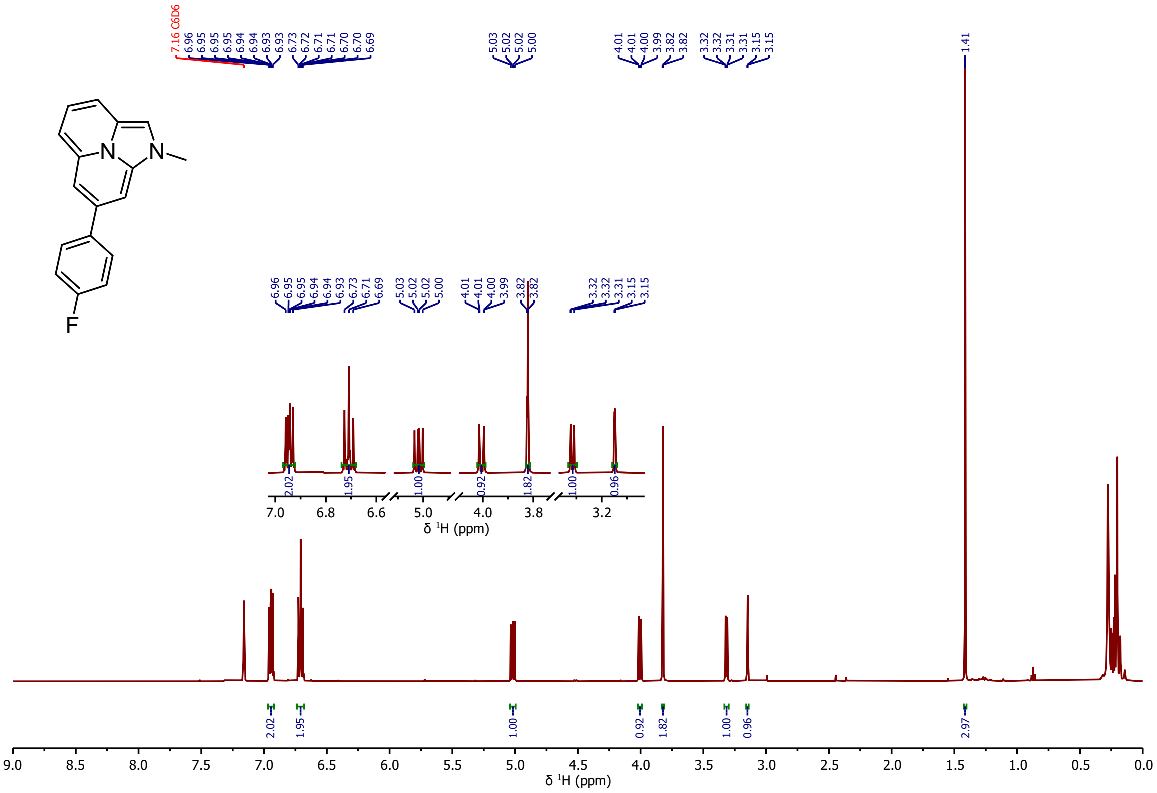

**Figure S313**: **$^{1}$H NMR** (500 MHz, $C_6D_6$, 298 K) of **6d**.

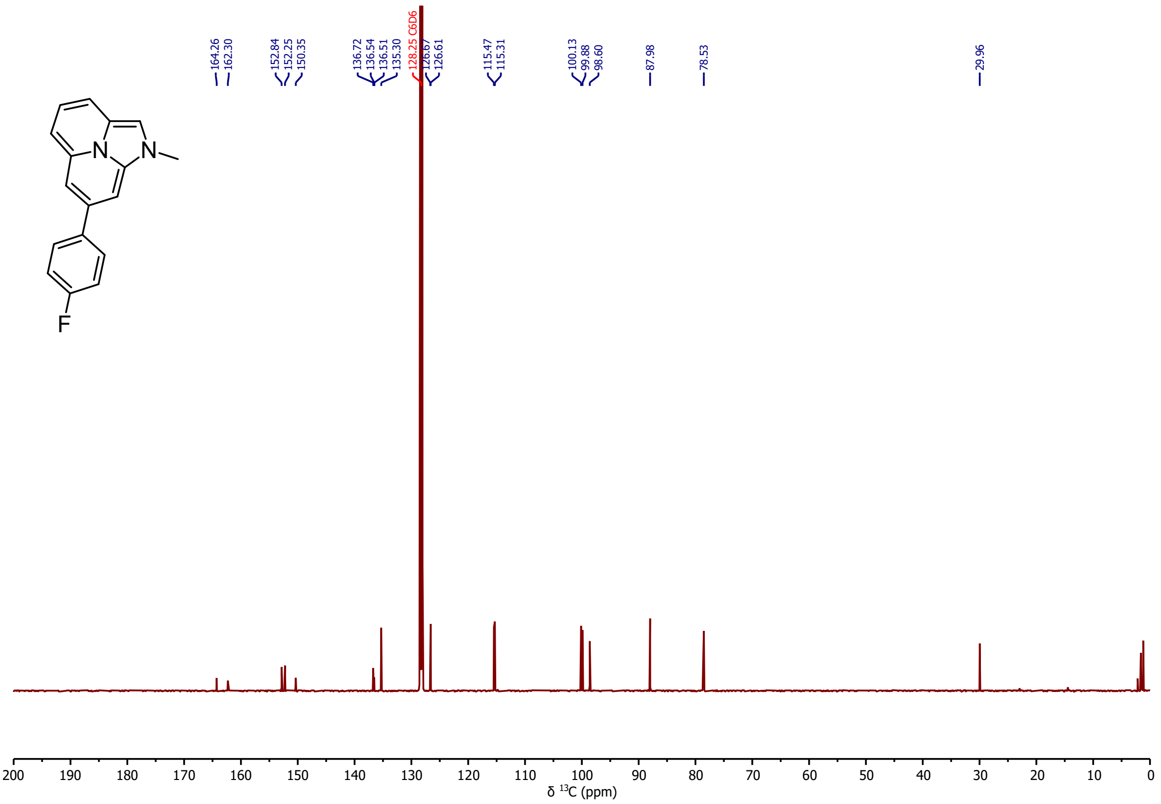

**Figure S314**: **$^{13}$C NMR** (126 MHz, $C_6D_6$, 298 K) of **6d**.

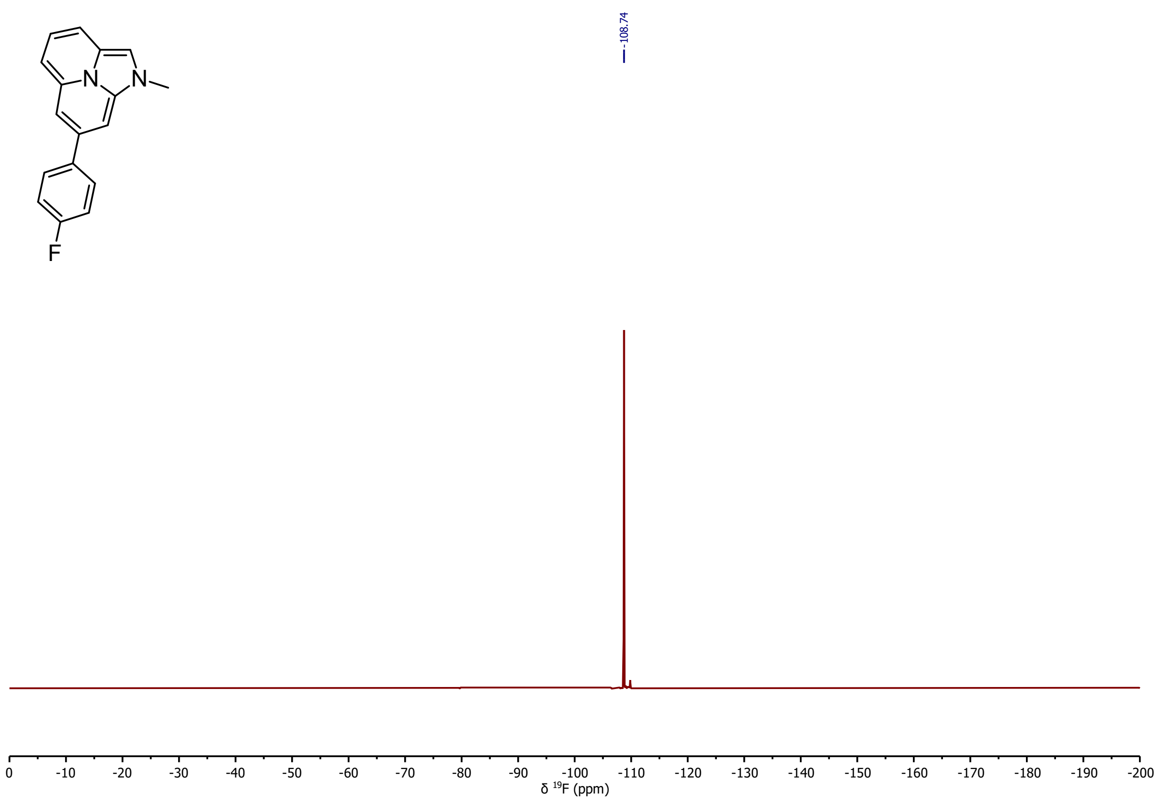


**Figure S315**: **[19]F NMR** (565 MHz, $C_6D_6$, 298 K) of **6d**.

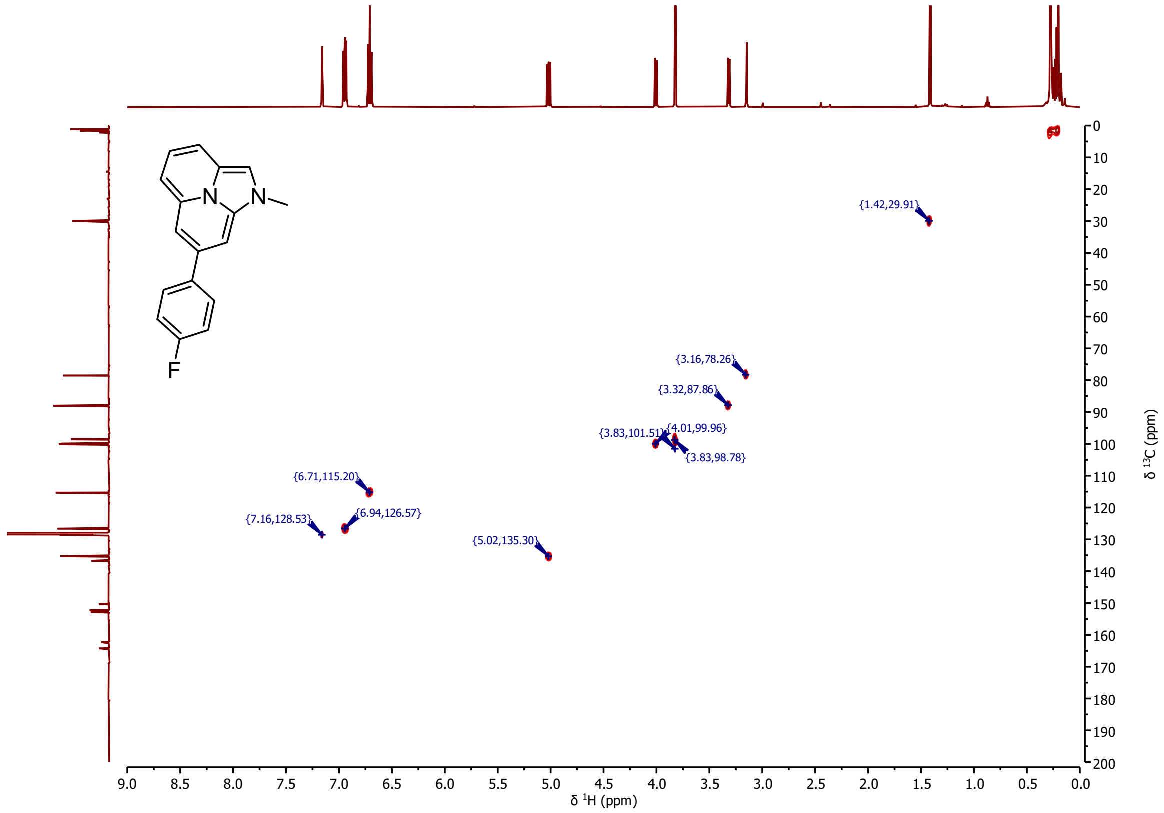


**Figure S316**: **[1]H/[13]C HSQC** (500/126 MHz, $C_6D_6$, 298 K) of **6d**.

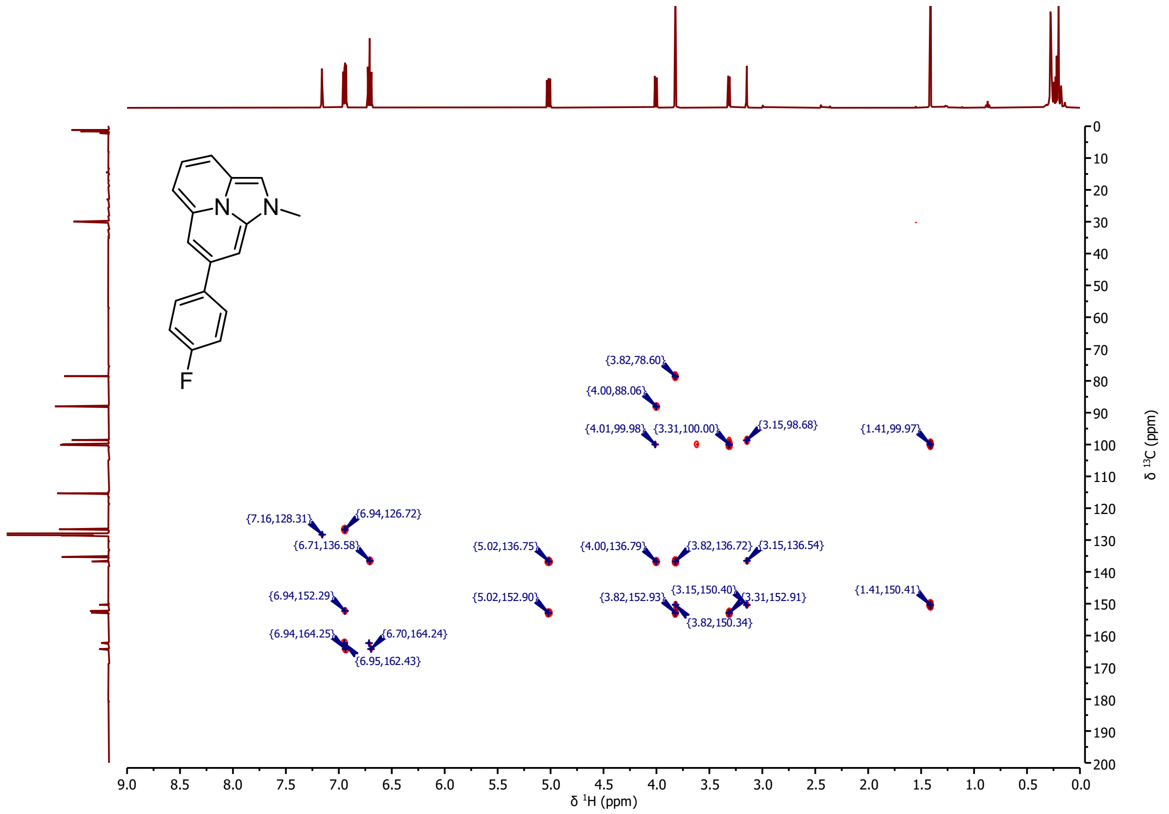

**Figure S317**: **$^{1}H/^{13}C$ HMBC** (500/126 MHz, $C_6D_6$, 298 K) of **6d**.

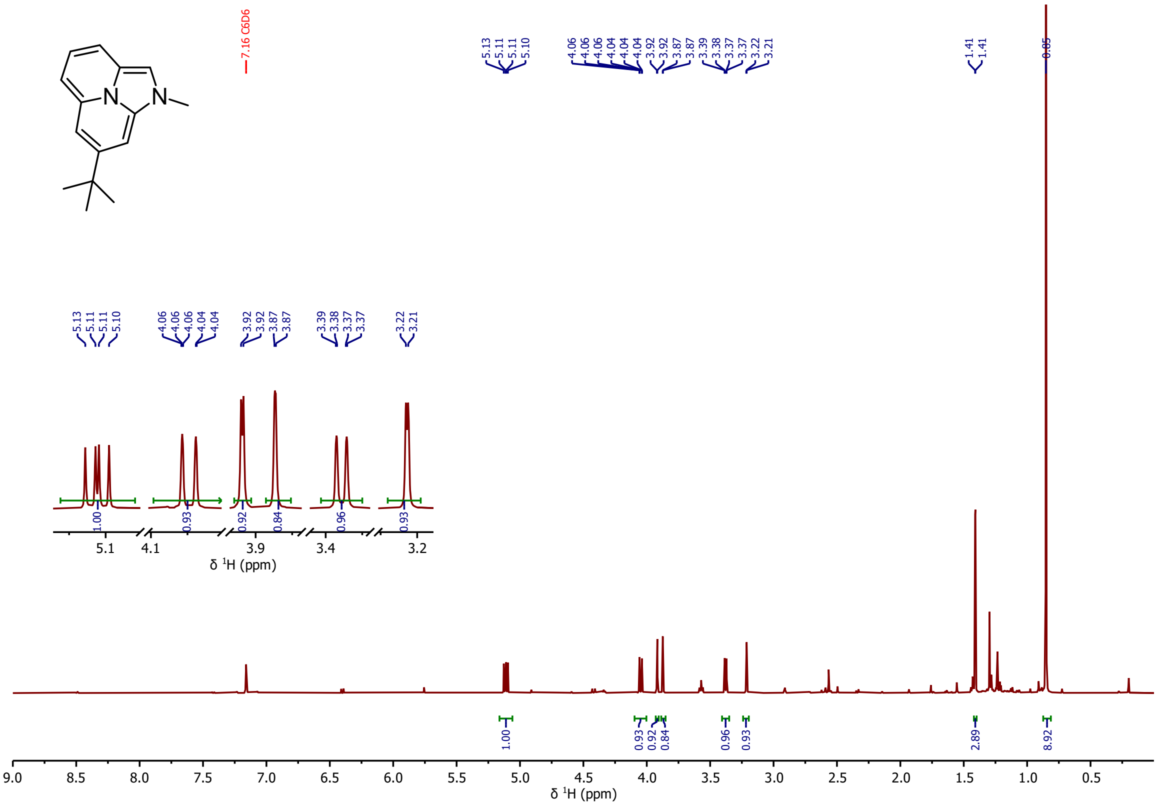

**Figure S318**: **$^{1}H$ NMR** (500 MHz, $C_6D_6$, 298 K) of **6e**.

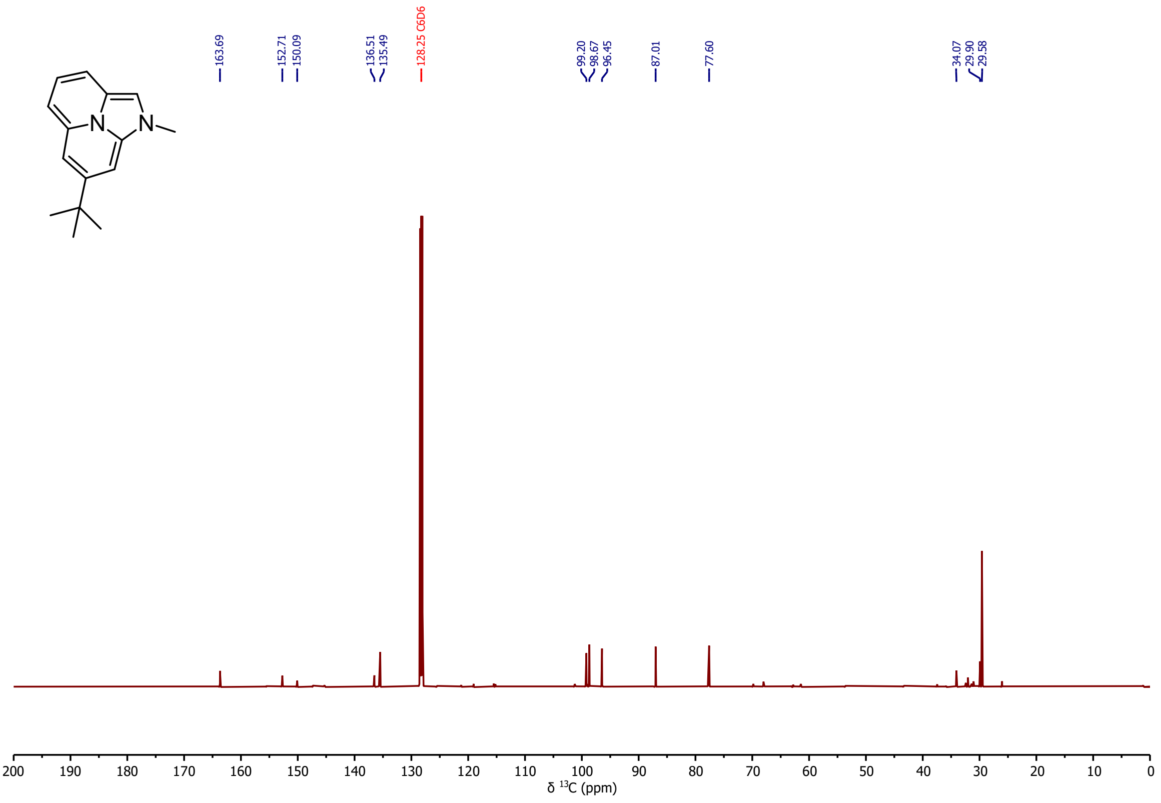


**Figure S319**: **$^{13}$C NMR** (126 MHz, $C_6D_6$, 298 K) of **6e**.

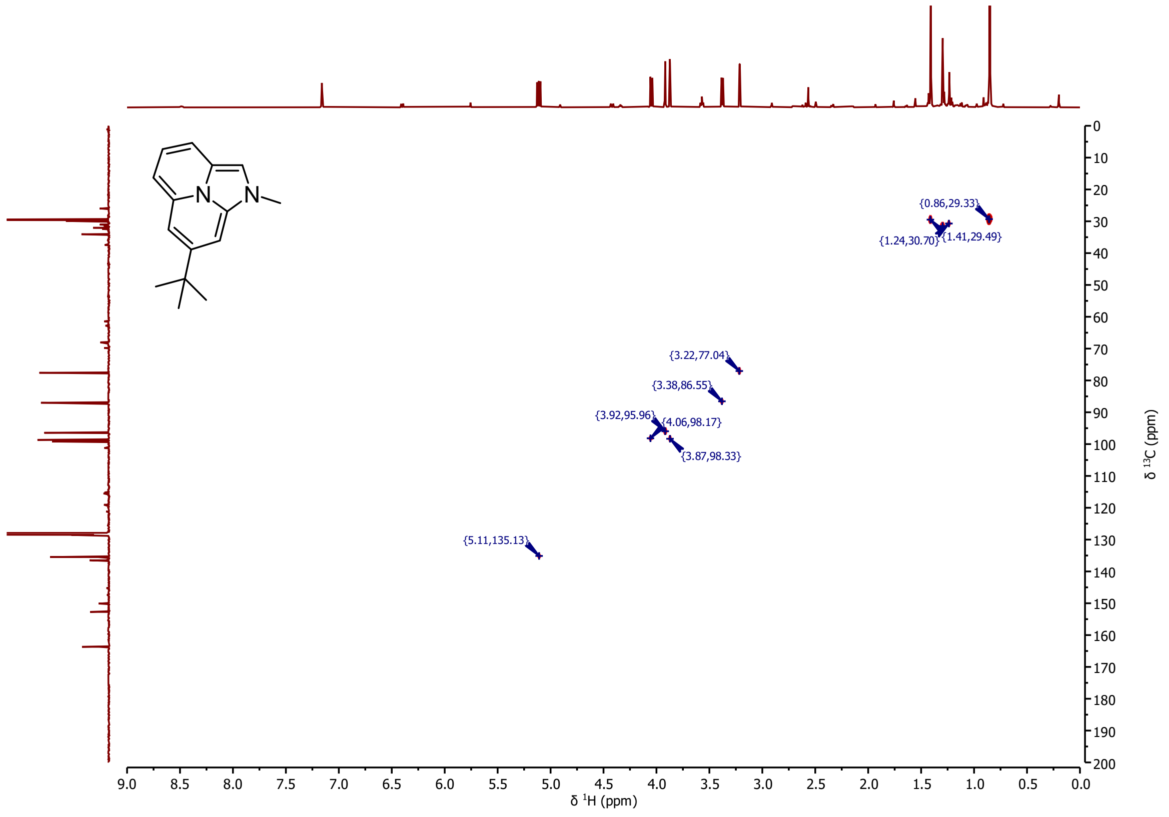


**Figure S320**: **$^{1}$H/$^{13}$C HSQC** (500/126 MHz, $C_6D_6$, 298 K) of **6e**.

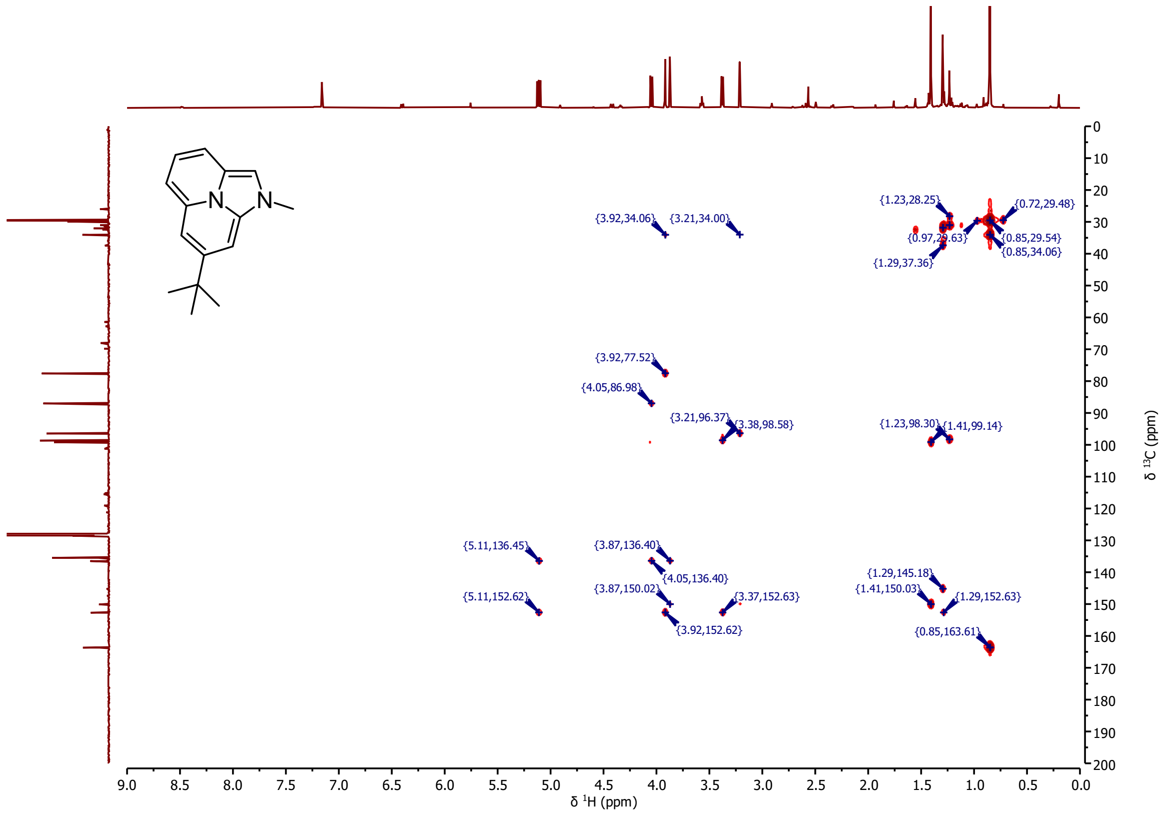

**Figure S321**: **$^{1}H/^{13}C$ HMBC** (500/126 MHz, $C_6D_6$, 298 K) of **6e**.

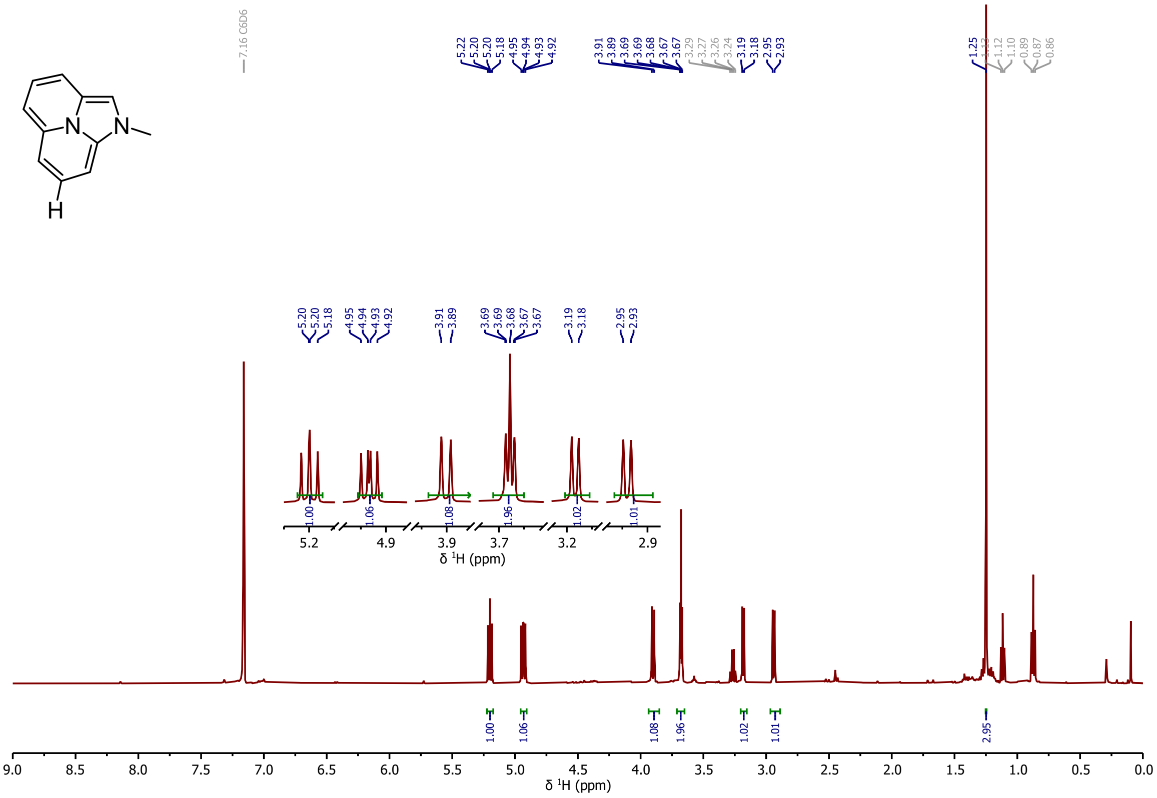

**Figure S322**: **$^{1}H$ NMR** (500 MHz, $C_6D_6$, 298 K) of **6f**.

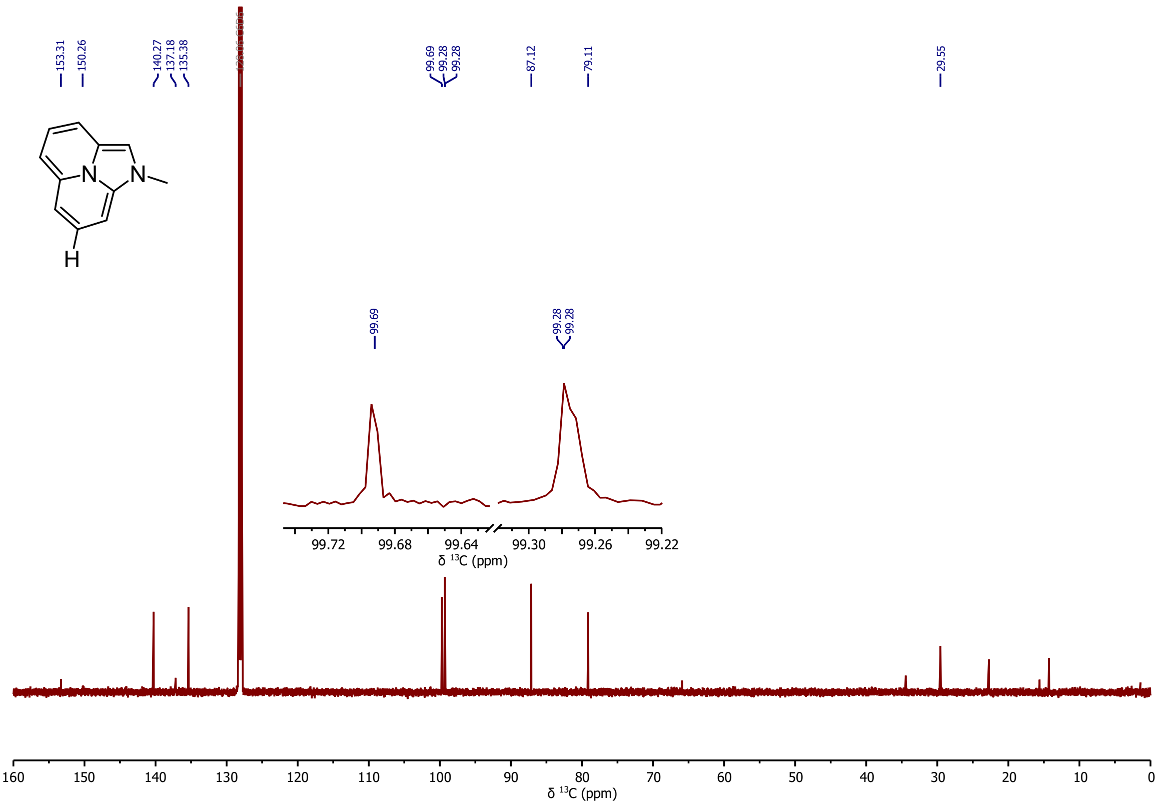

**Figure S323**: **$^{13}$C NMR** (126 MHz, $C_6D_6$, 298 K) of **6f**.

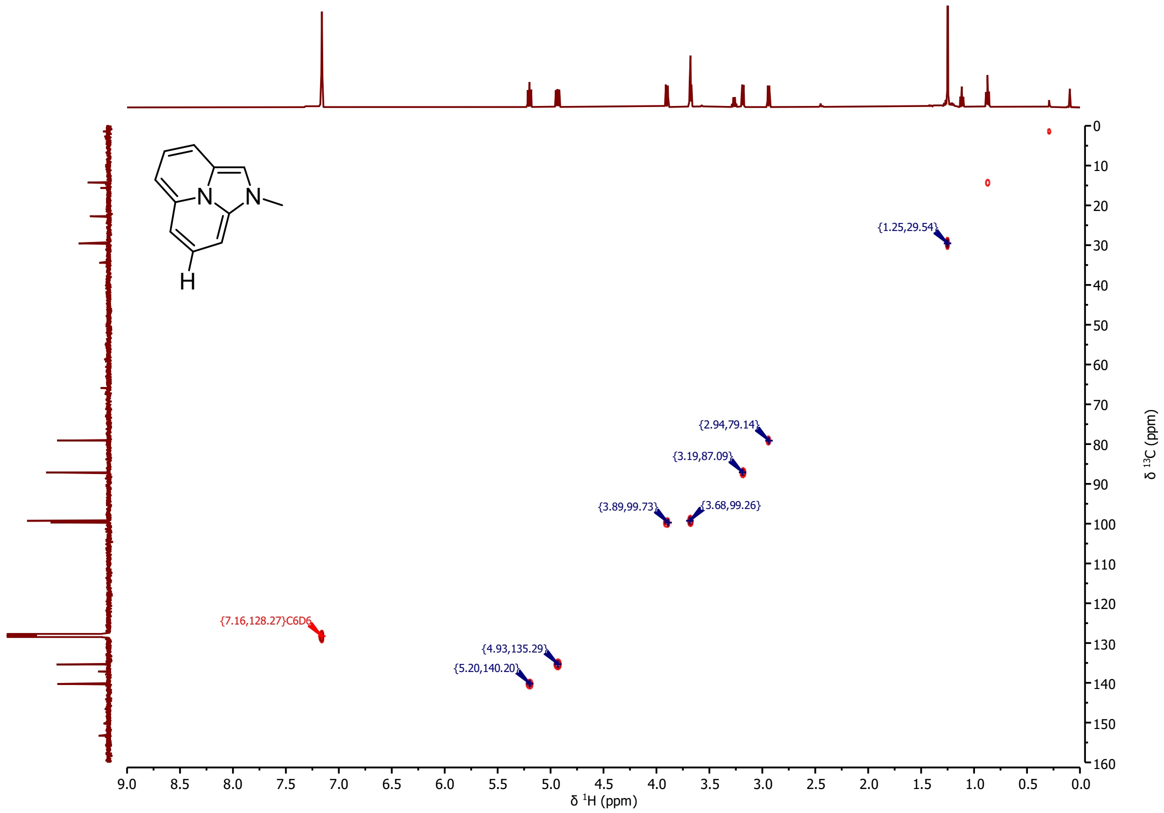

**Figure S324**: **$^{1}$H/$^{13}$C HSQC** (500/126 MHz, $C_6D_6$, 298 K) of **6f**.

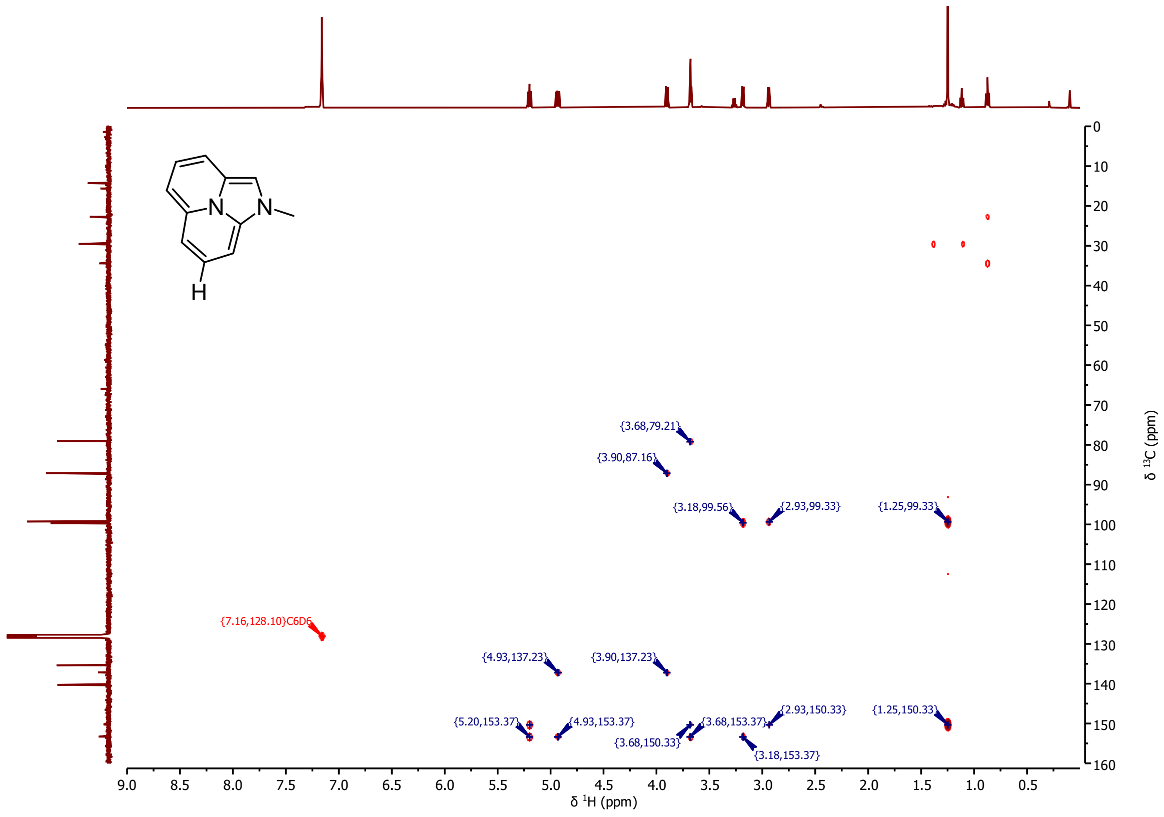

**Figure S325**: **$^{1}H/^{13}C$ HMBC** (500/126 MHz, $C_6D_6$, 298 K) of **6f**.

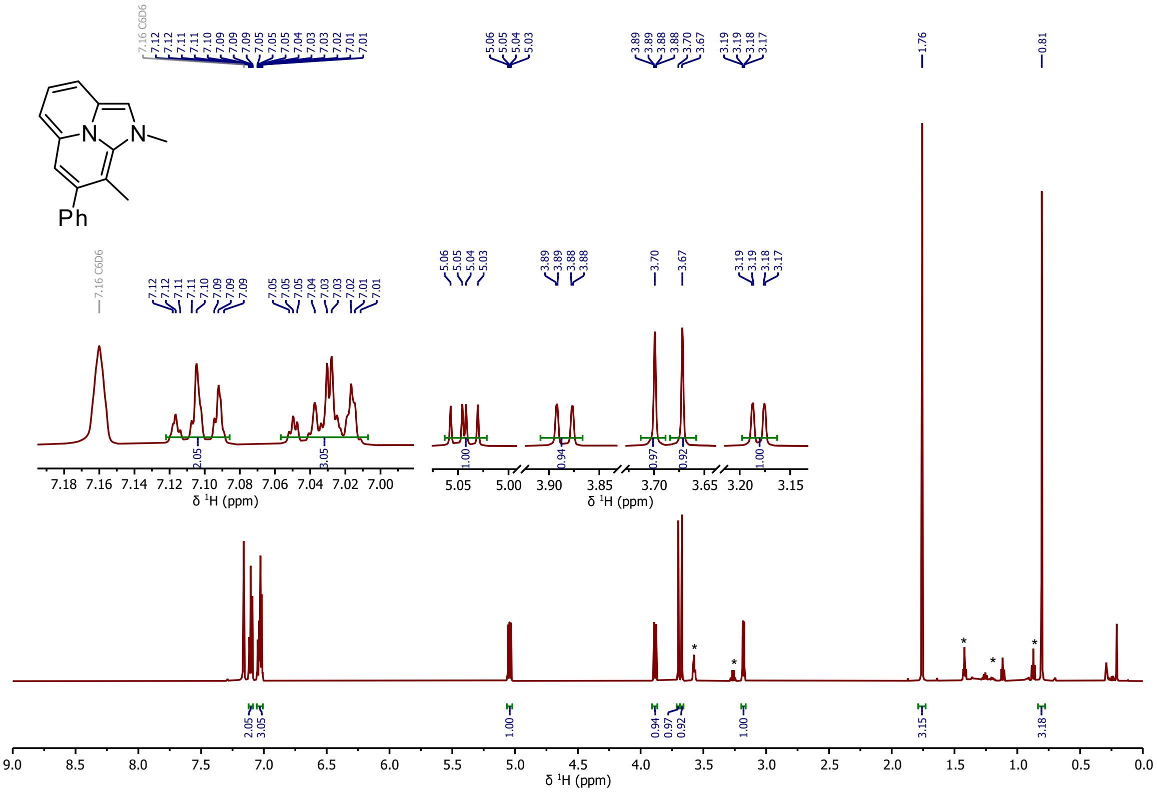

**Figure S326**: **$^{1}H$ NMR** (600 MHz, $C_6D_6$, 298 K) of **6g**. * = thf, $Et_2O$ and pentane.

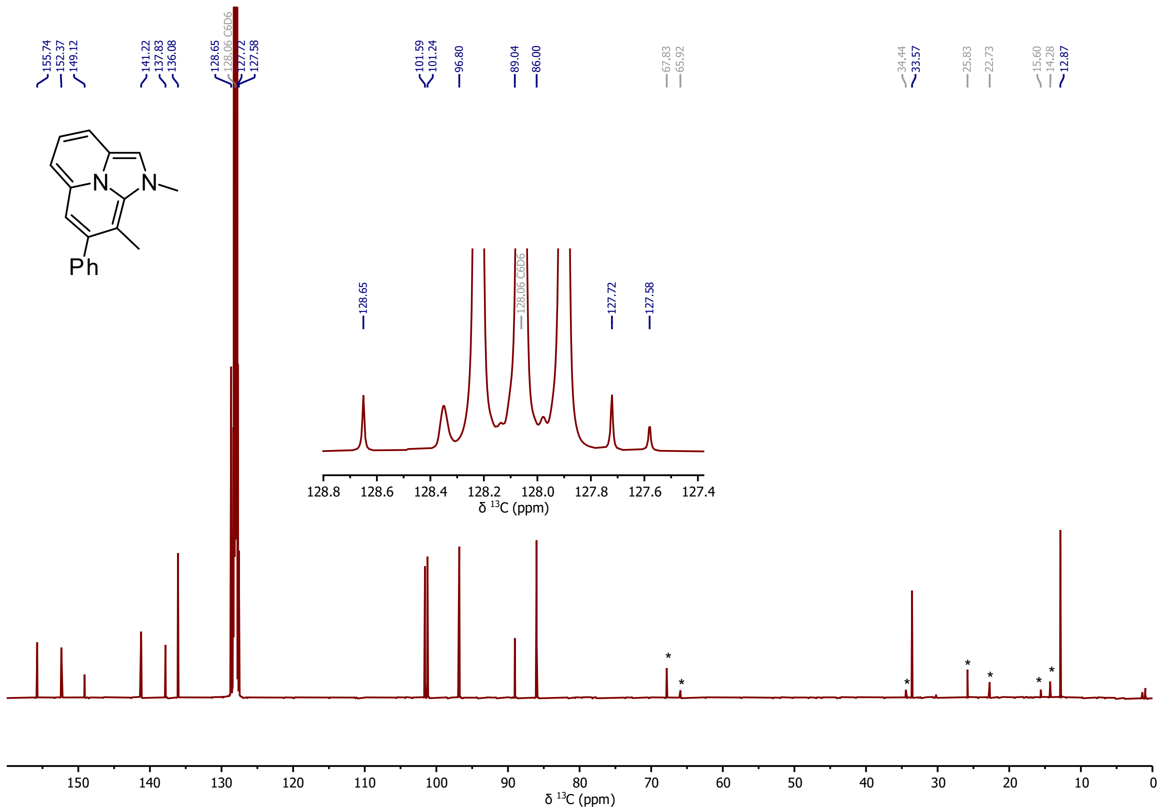

**Figure S327**: **$^{13}$C NMR** (151 MHz, $C_6D_6$, 298 K) of **6g**. * = THF, $Et_2O$ and pentane.

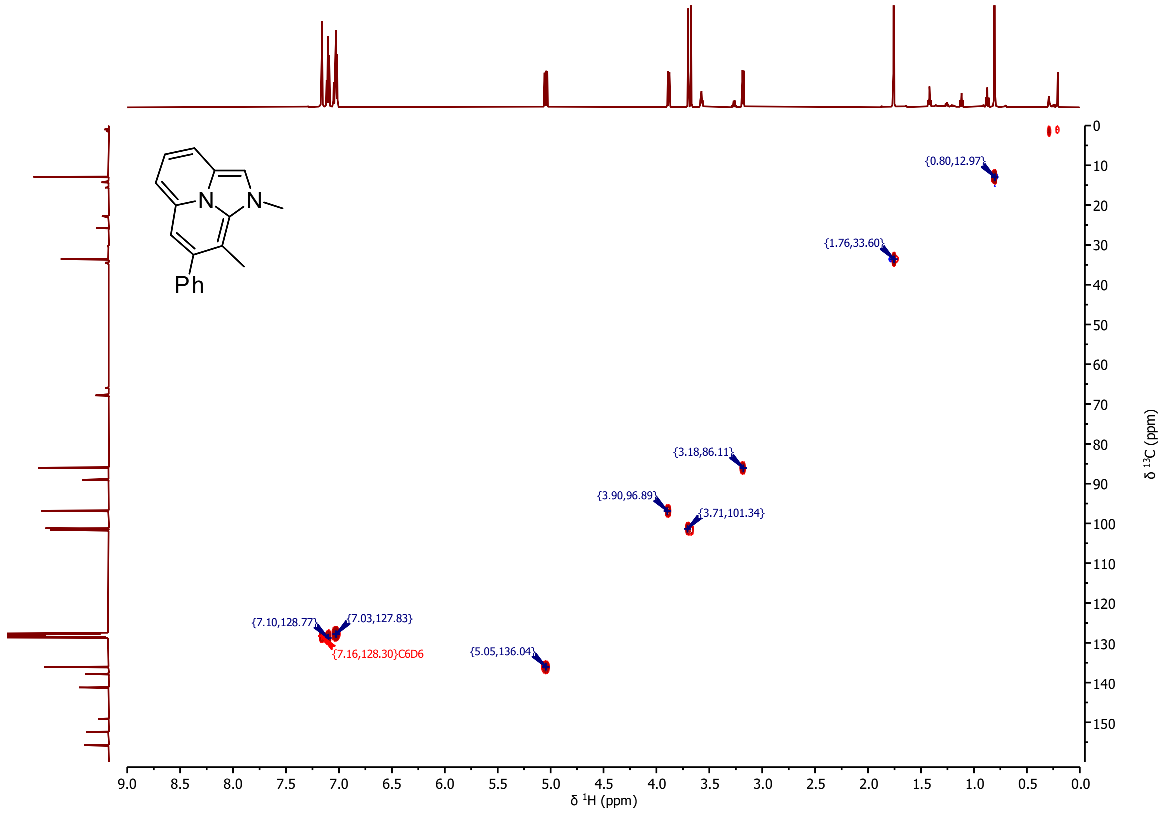

**Figure S328**: **$^{1}$H/$^{13}$C HSQC** (600/151 MHz, $C_6D_6$, 298 K) of **6g**.

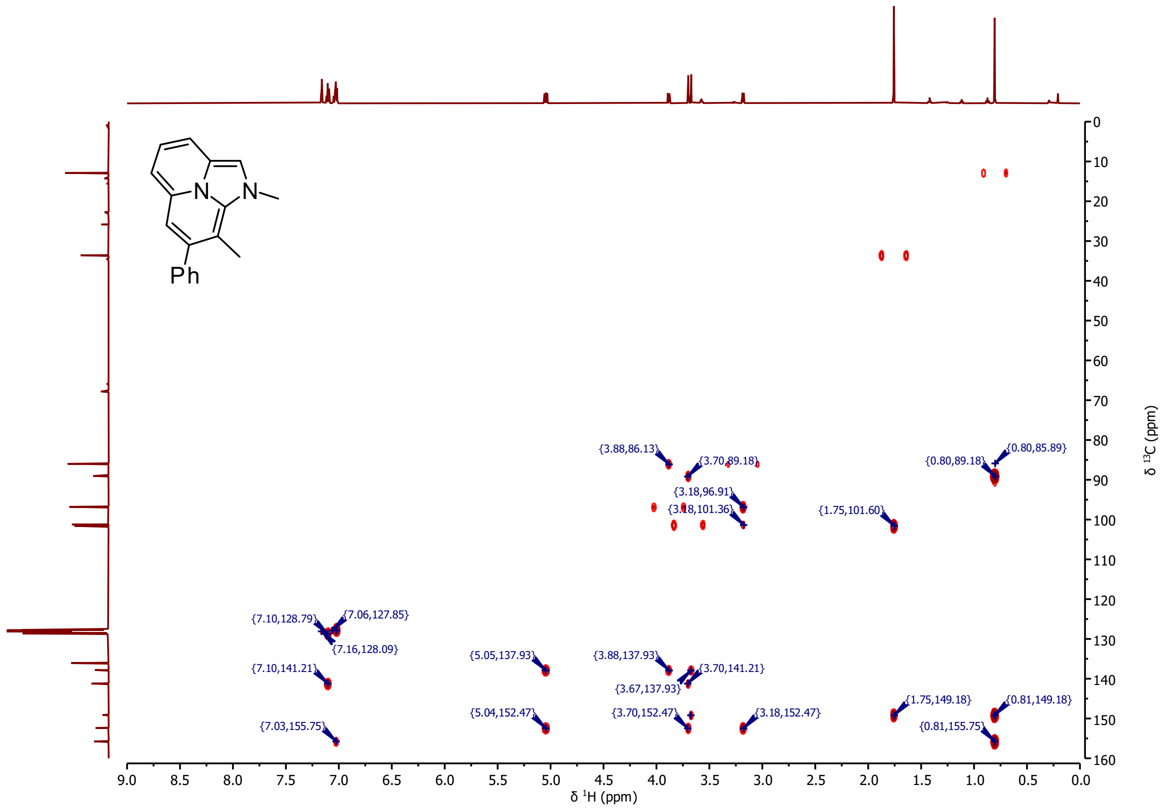

**Figure S329**: **$^{1}H/^{13}C$ HMBC** (600/151 MHz, $C_6D_6$, 298 K) of **6g**.

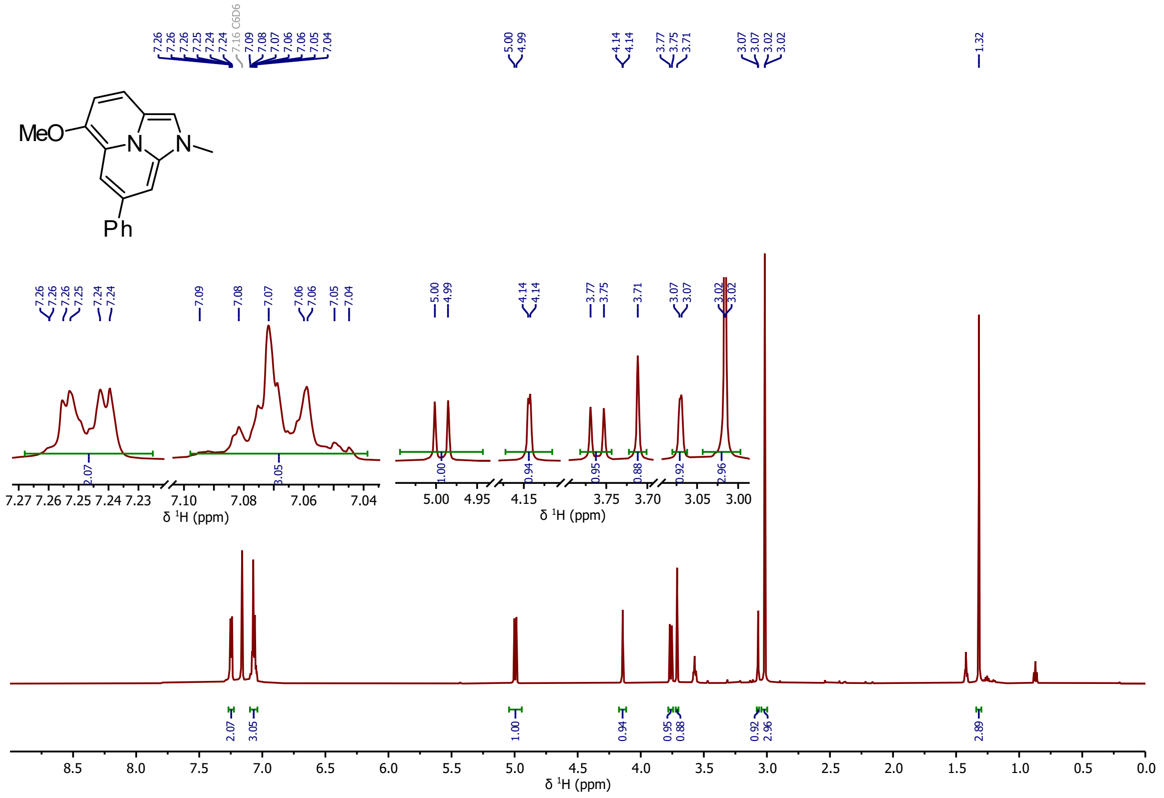

**Figure S330**: **$^{1}H$ NMR** (600 MHz, $C_6D_6$, 298 K) of **6h**. * = THF and pentane.

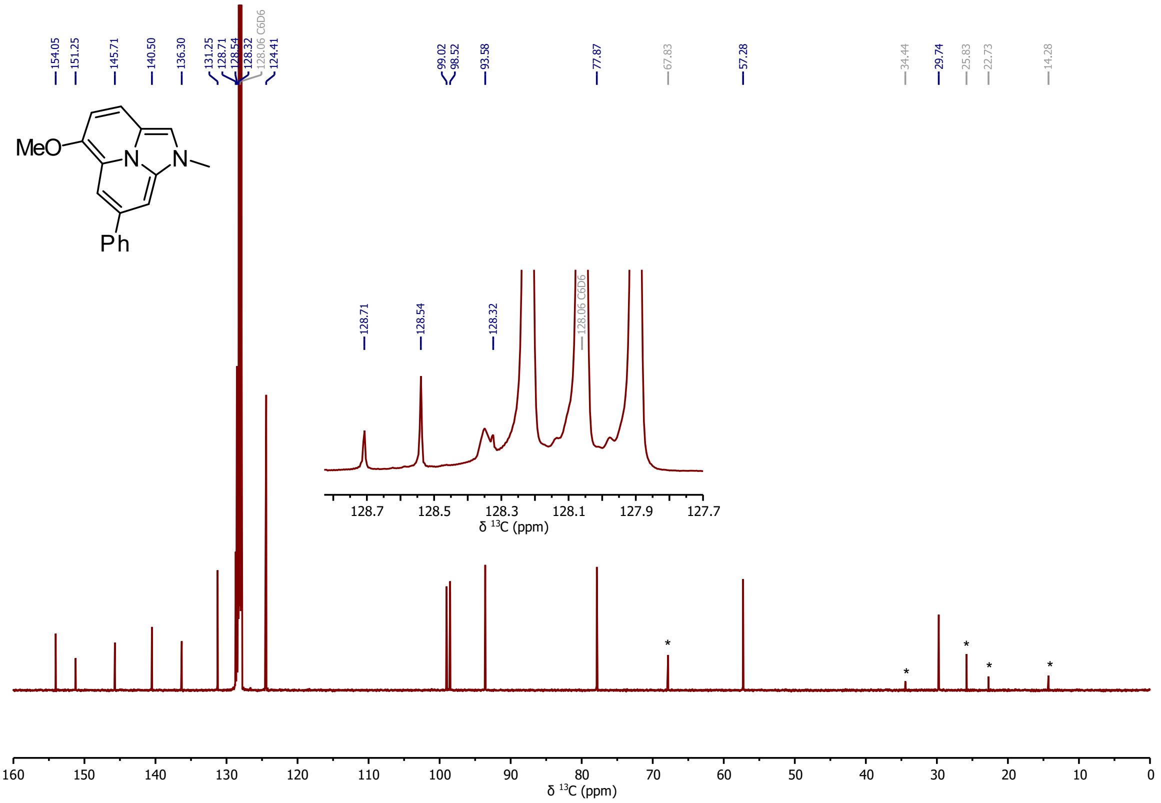

**Figure S331**: **$^{13}$C NMR** (151 MHz, $C_6D_6$, 298 K) of **6h**. * = THF and pentane.

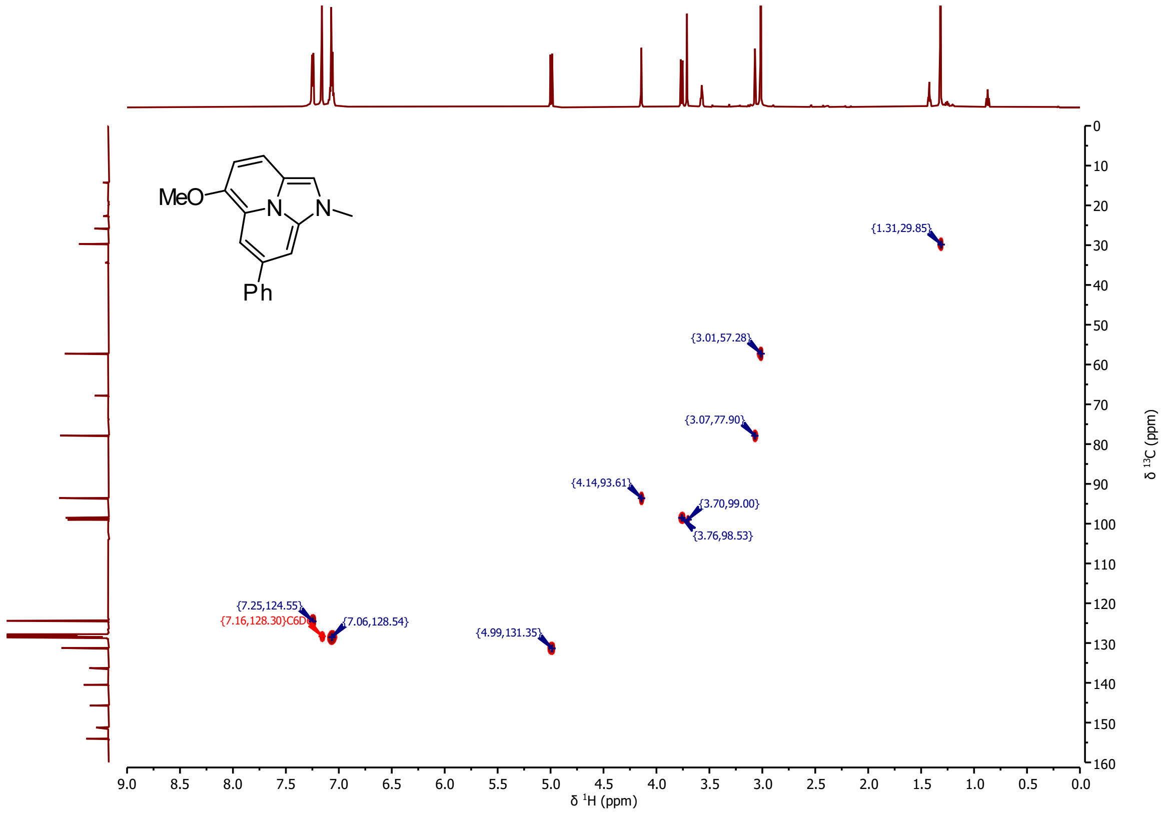

**Figure S332**: **$^{1}$H/$^{13}$C HSQC** (600/151 MHz, $C_6D_6$, 298 K) of **6h**.

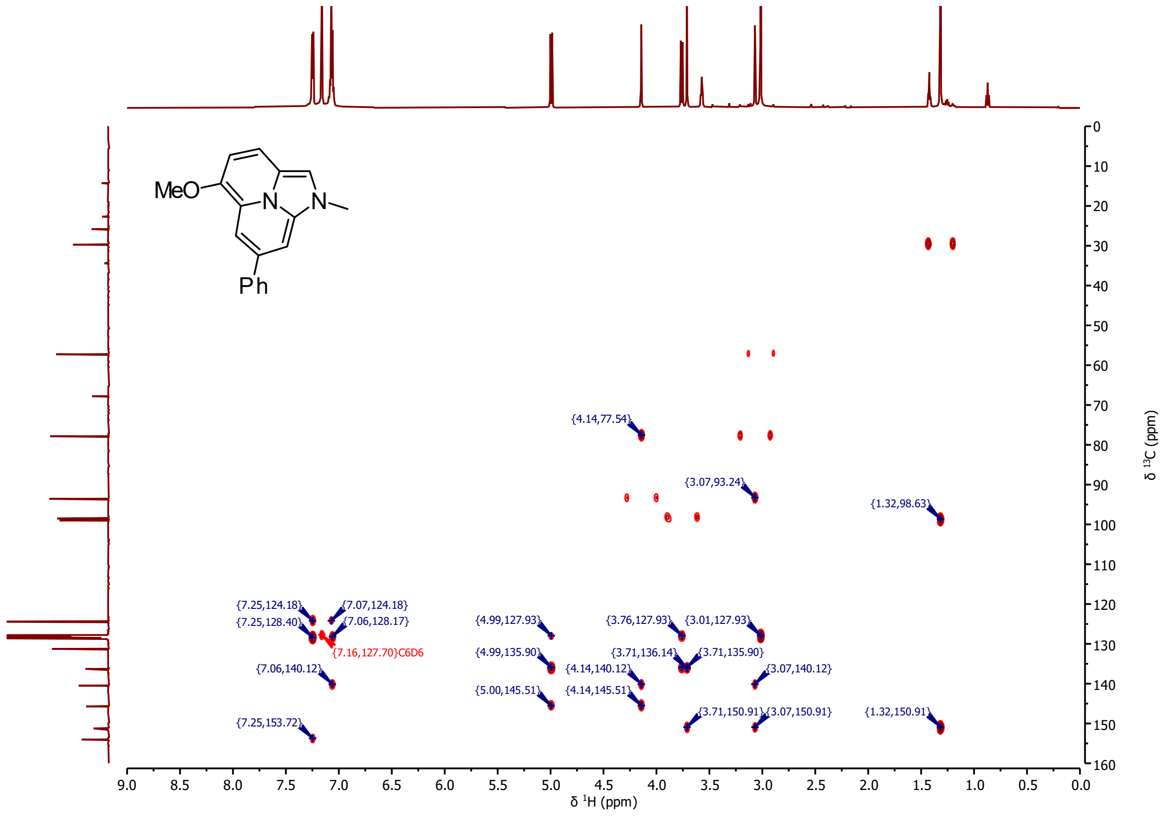

**Figure S333**: $^{1}H/^{13}C$ **HMBC** (600/151 MHz, $C_6D_6$, 298 K) of **6h**.

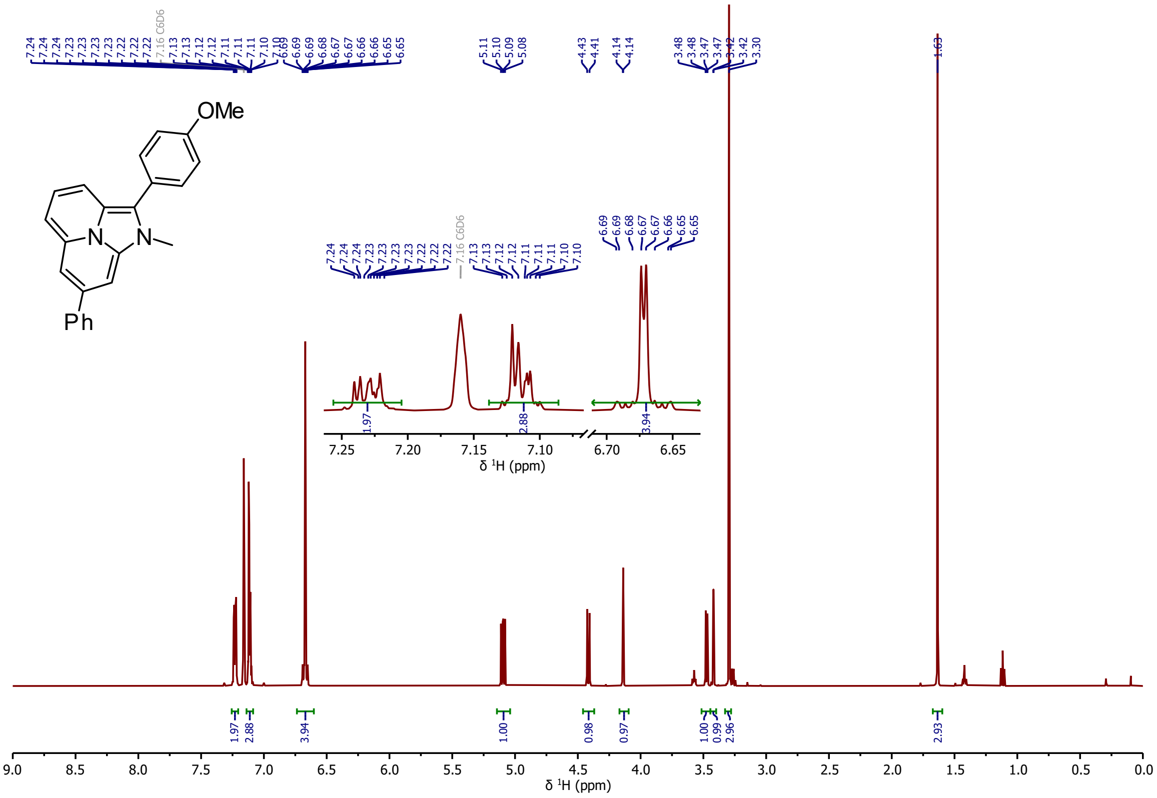

**Figure S334**: $^{1}H$ **NMR** (500 MHz, $C_6D_6$, 298 K) of **6i**.

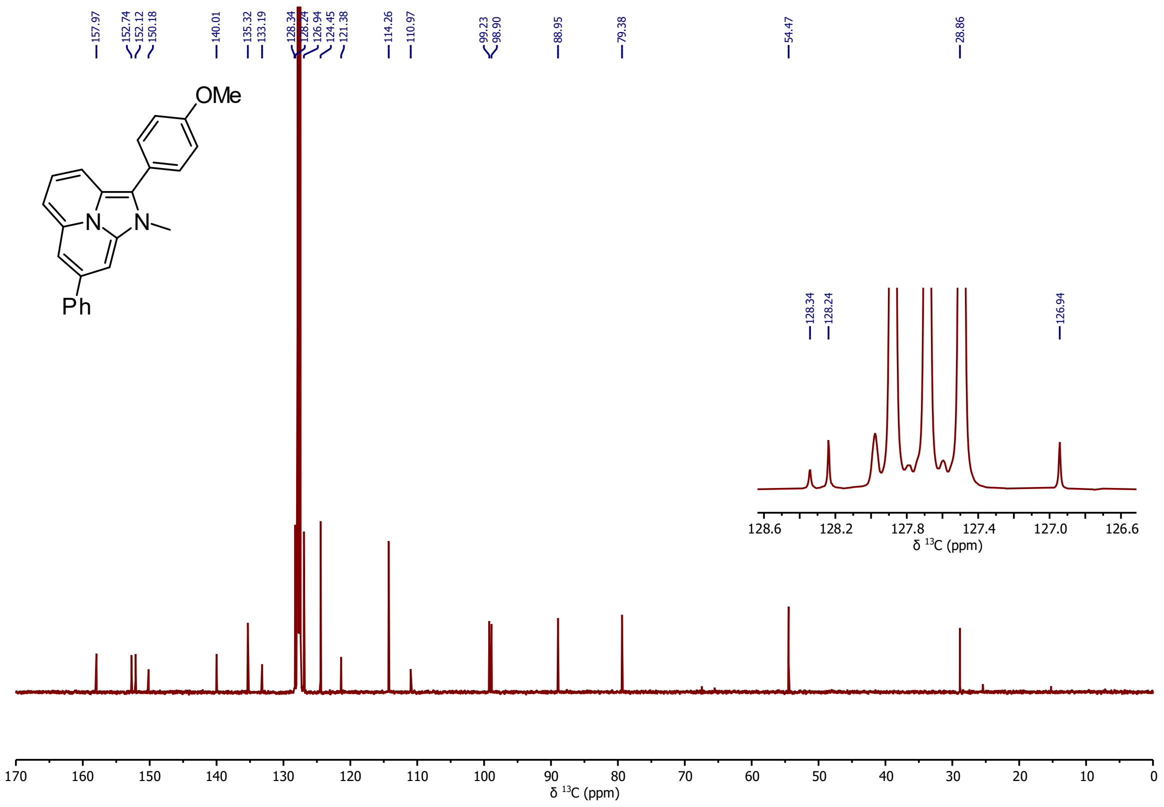

**Figure S335**: $^{13}$**C NMR** (126 MHz, $C_6D_6$, 298 K) of **6i**.

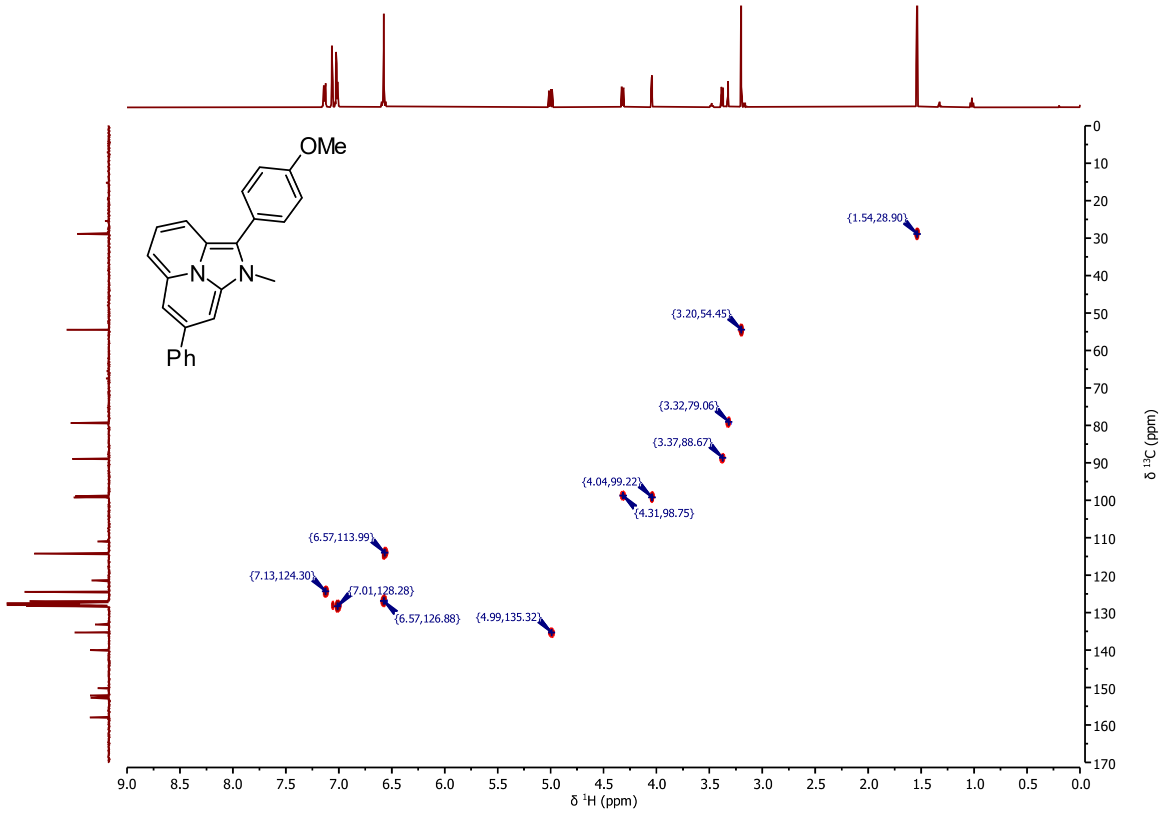

**Figure S336**: $^{1}$**H/**$^{13}$**C HSQC** (500/126 MHz, $C_6D_6$, 298 K) of **6i**.

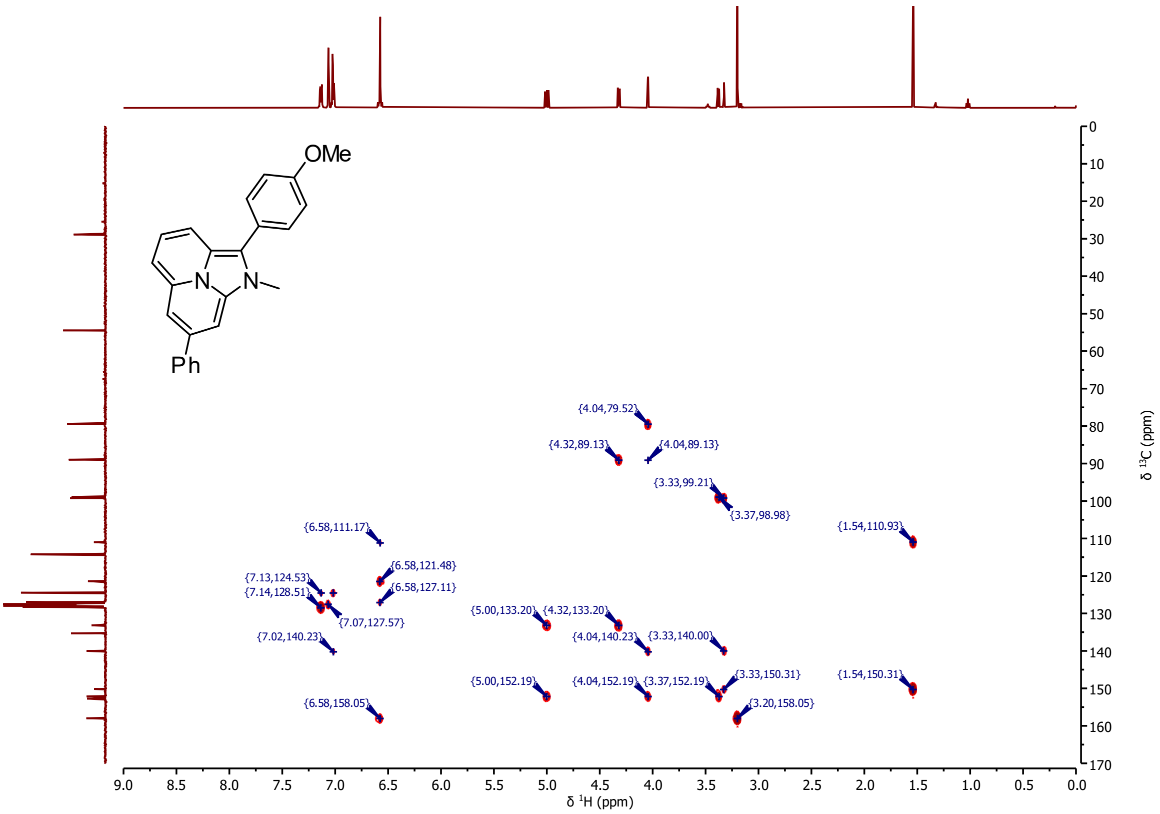


**Figure S337**: **$^1$H/$^{13}$C HMBC** (500/126 MHz, $C_6D_6$, 298 K) of **6i**.

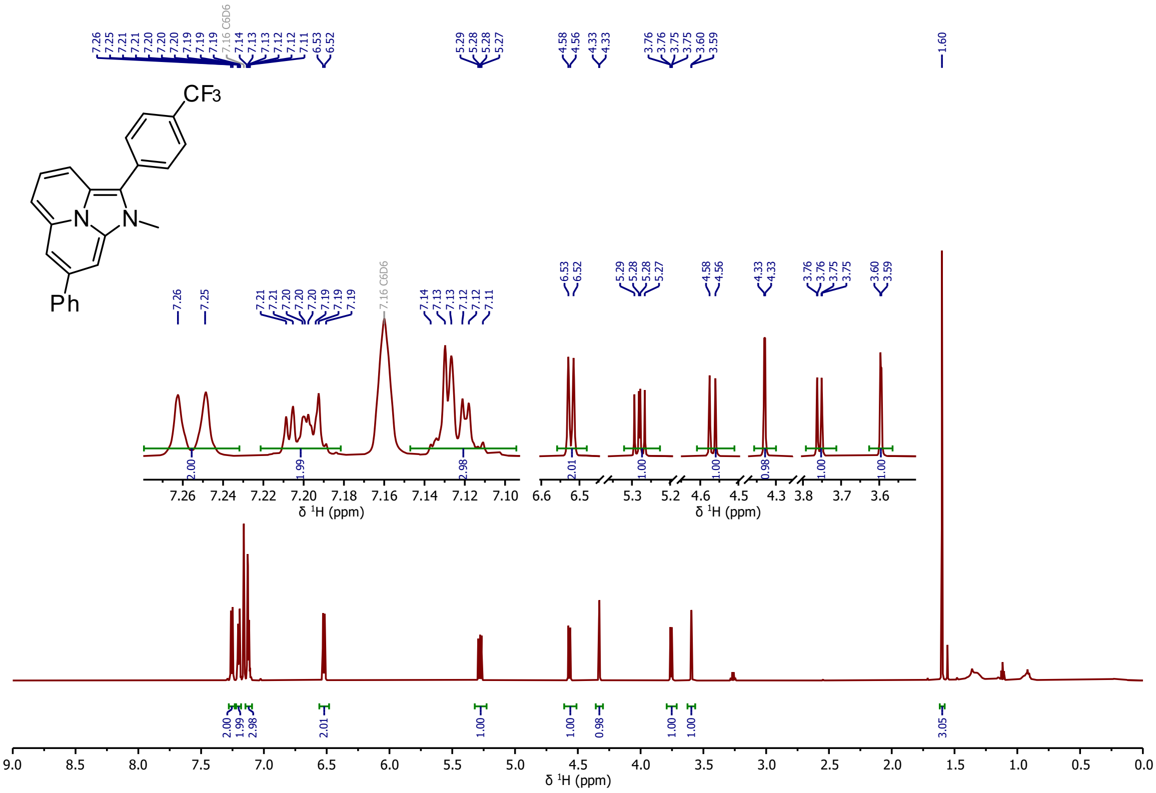


**Figure S338**: **$^1$H NMR** (600 MHz, $C_6D_6$, 298 K) of **6j**.

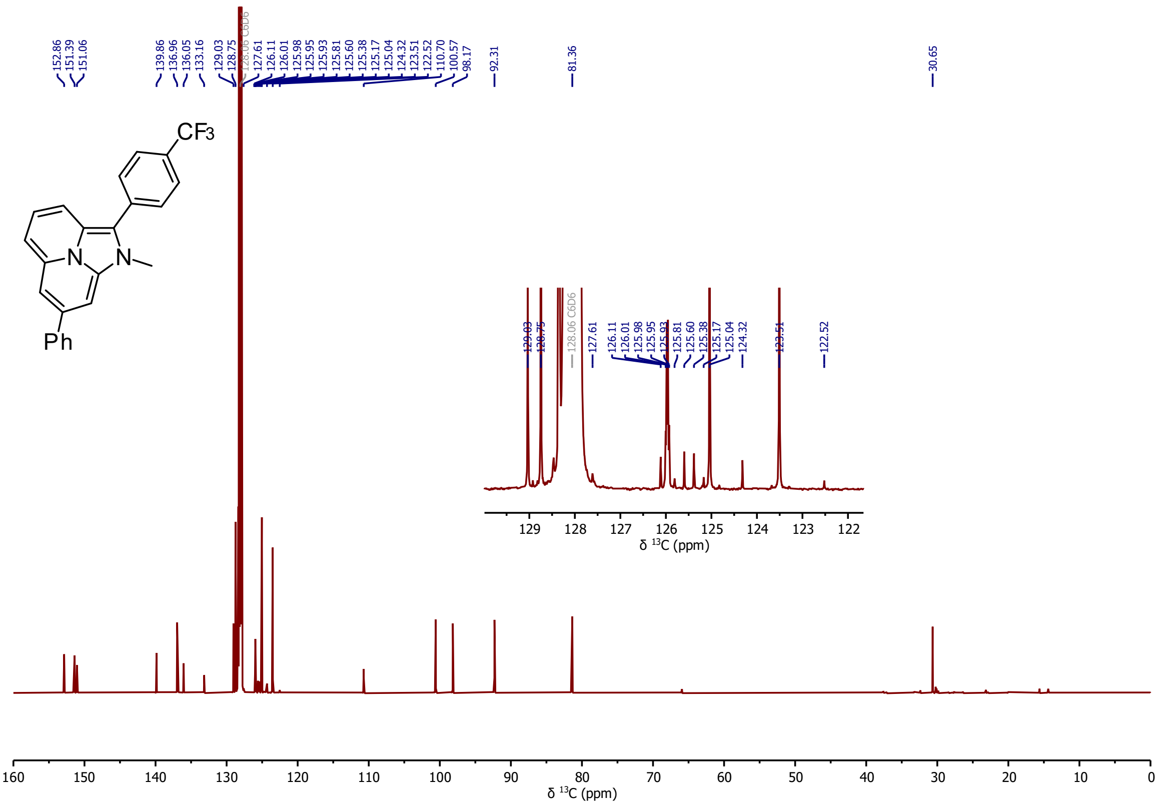

**Figure S339**: **$^{13}$C NMR** (151 MHz, $C_6D_6$, 298 K) of **6j**.

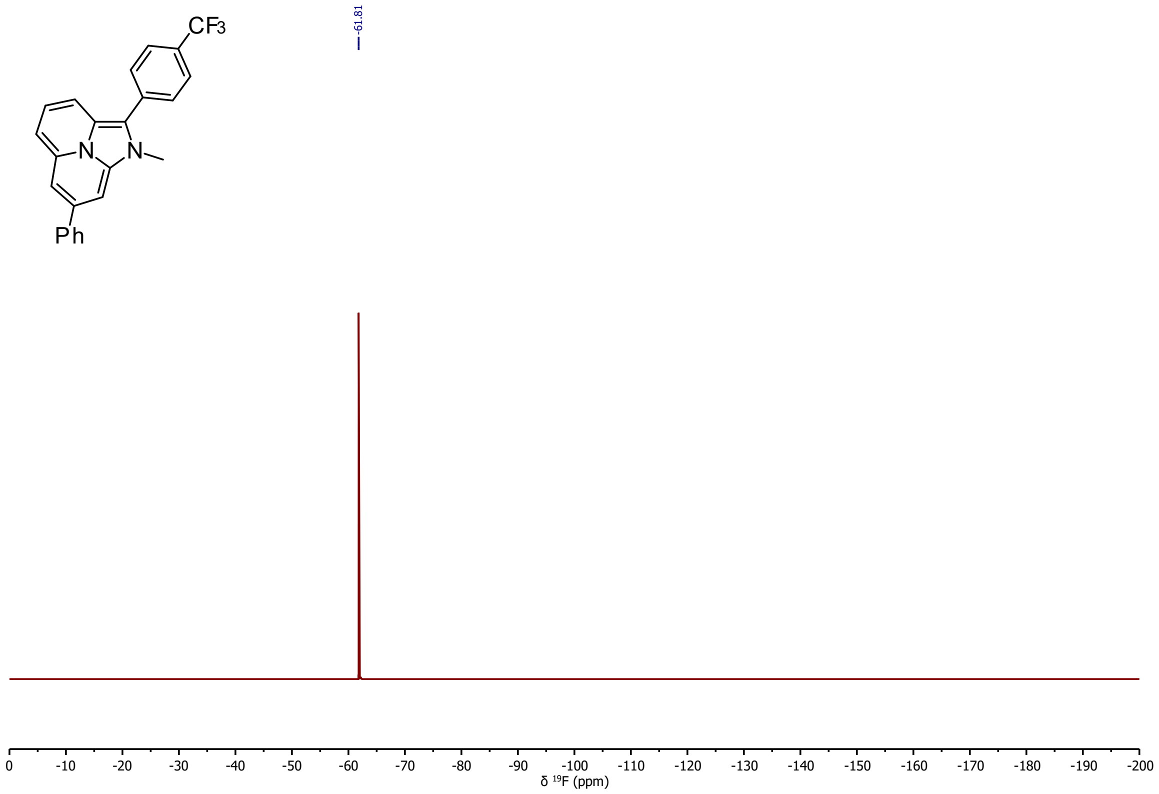

**Figure S340**: **$^{19}$F NMR** (565 MHz, $C_6D_6$, 298 K) of **6j**.

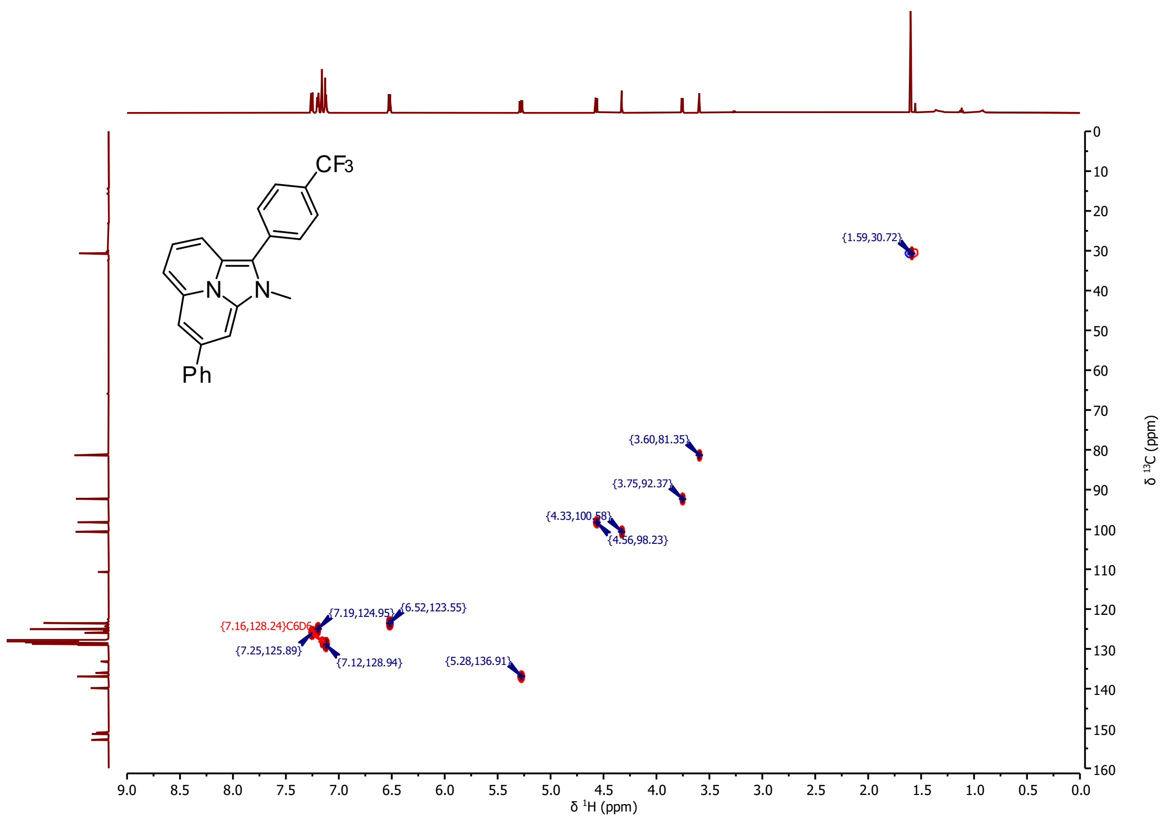


**Figure S341**: **$^{1}$H/$^{13}$C HSQC** (600/151 MHz, $C_6D_6$, 298 K) of **6j**.

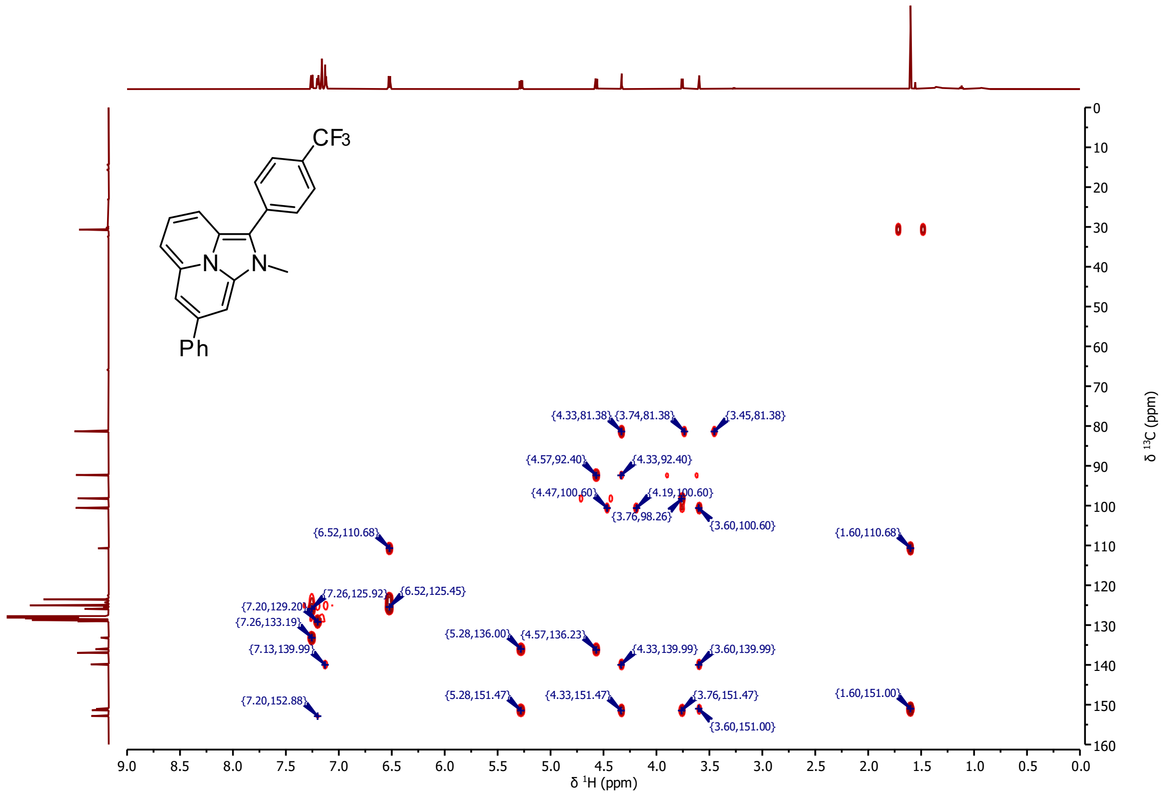


**Figure S342**: **$^{1}$H/$^{13}$C HMBC** (600/151 MHz, $C_6D_6$, 298 K) of **6j**.

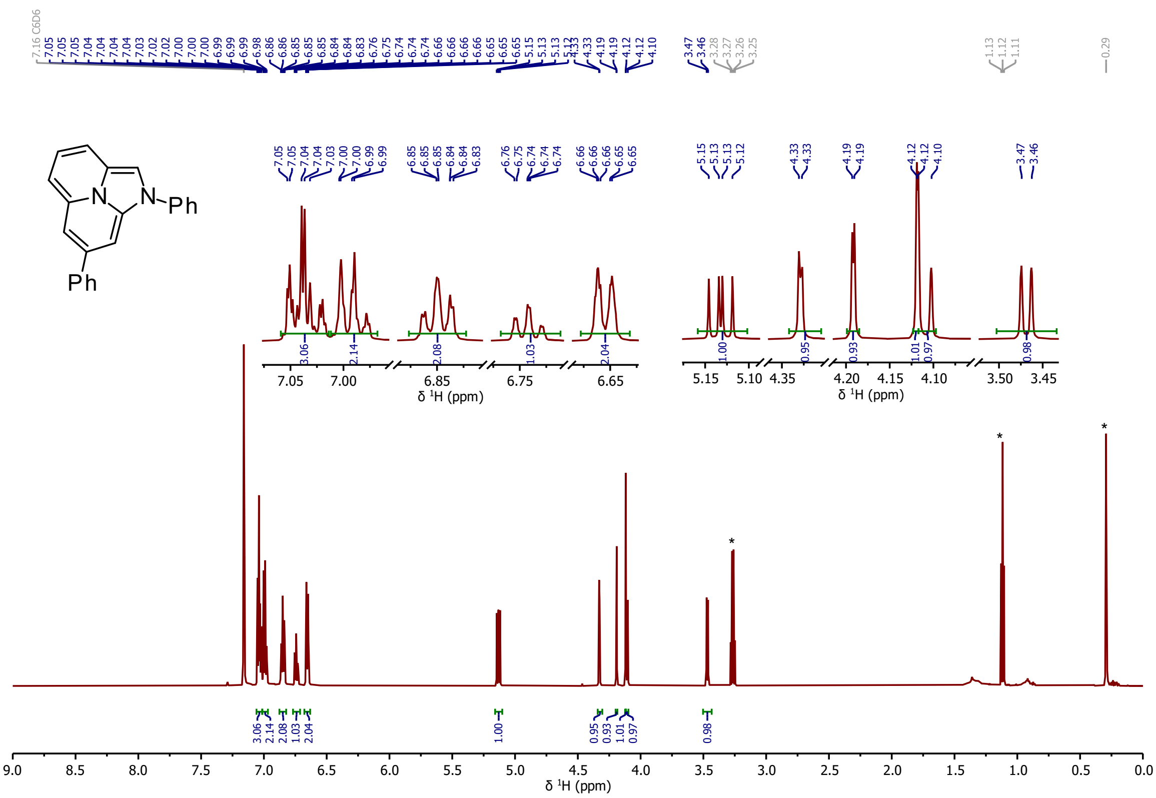

**Figure S343**: **$^{1}$H NMR** (600 MHz, $C_6D_6$, 298 K) of **6k**. * = $Et_2O$ and silicone grease.

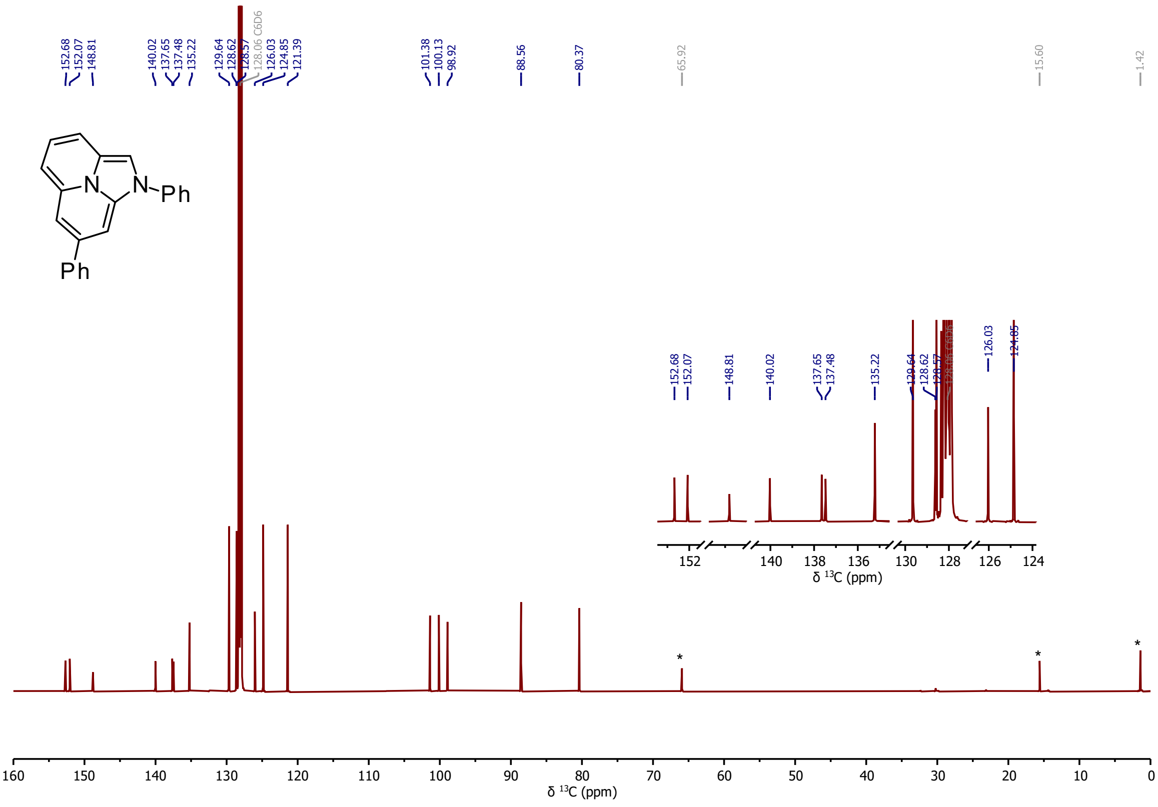

**Figure S344**: **$^{13}$C NMR** (151 MHz, $C_6D_6$, 298 K) of **6k**. * = $Et_2O$ and silicone grease.

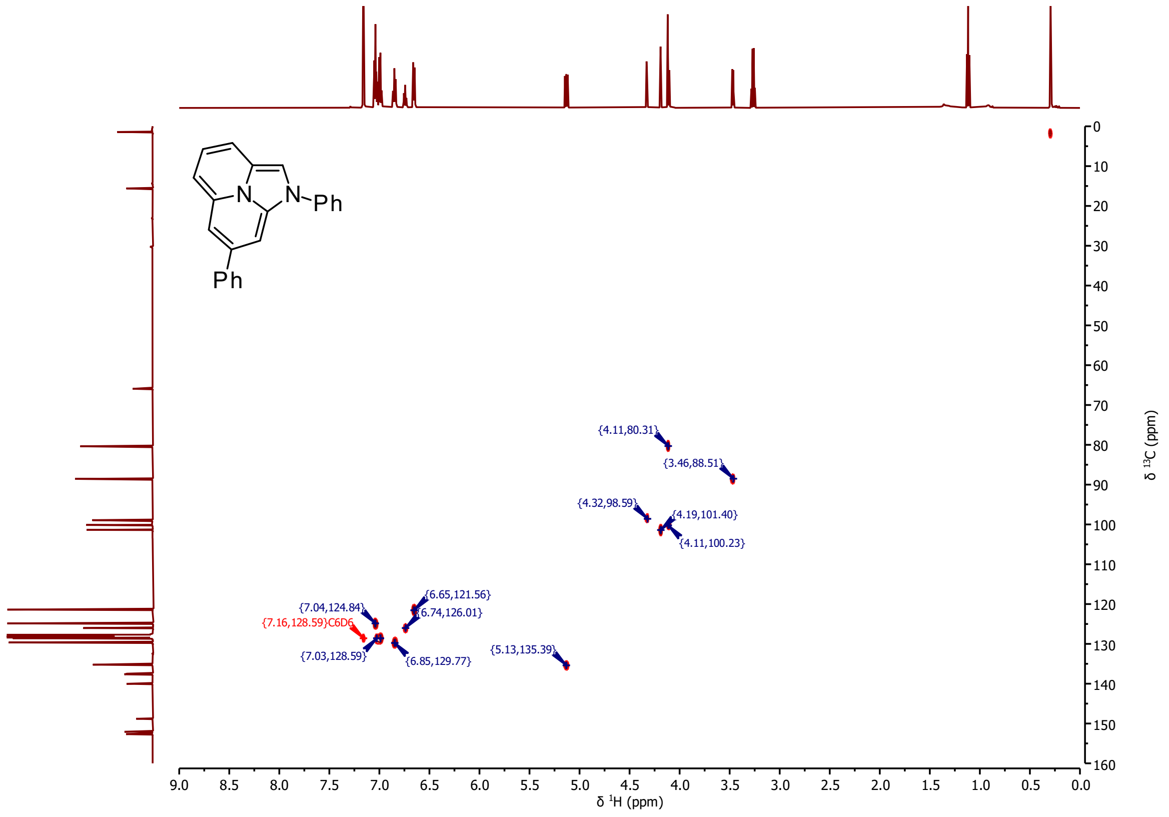

**Figure S345**: **$^1H/^{13}C$ HSQC** (600/151 MHz, $C_6D_6$, 298 K) of **6k**.

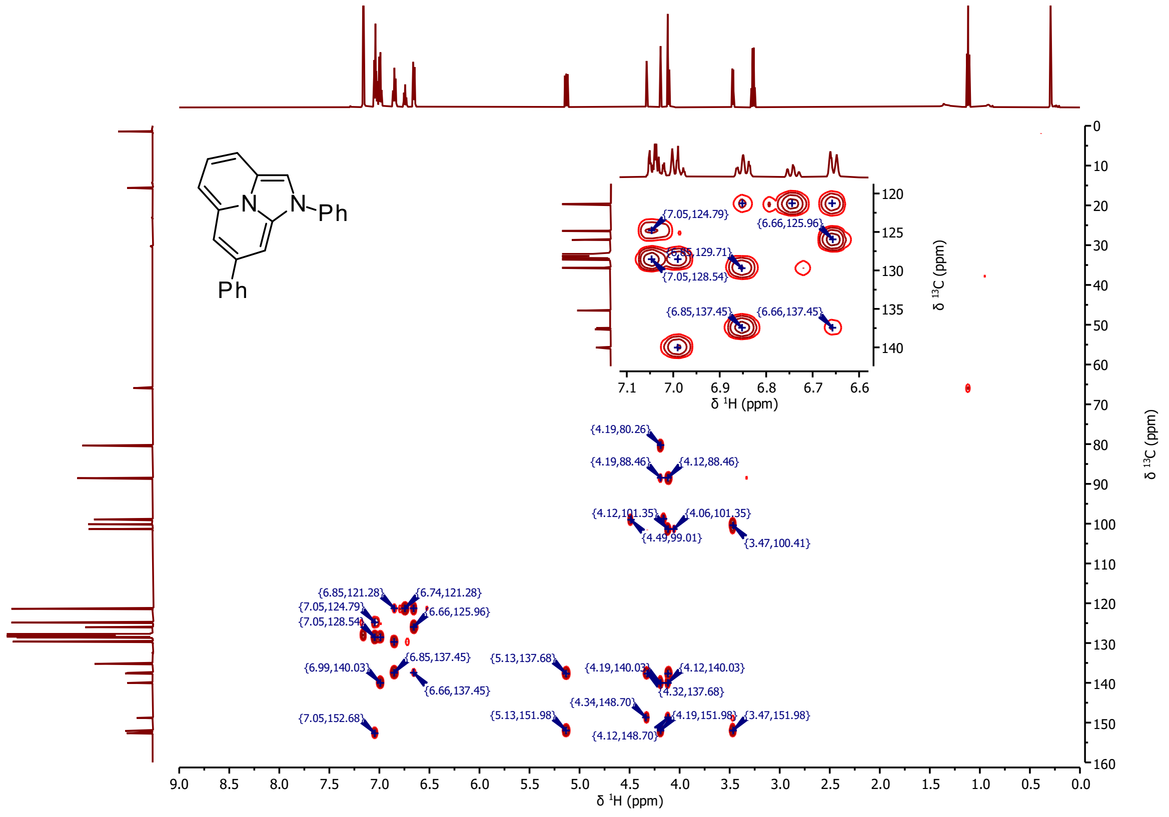

**Figure S346**: **$^1H/^{13}C$ HMBC** (600/151 MHz, $C_6D_6$, 298 K) of **6k**.

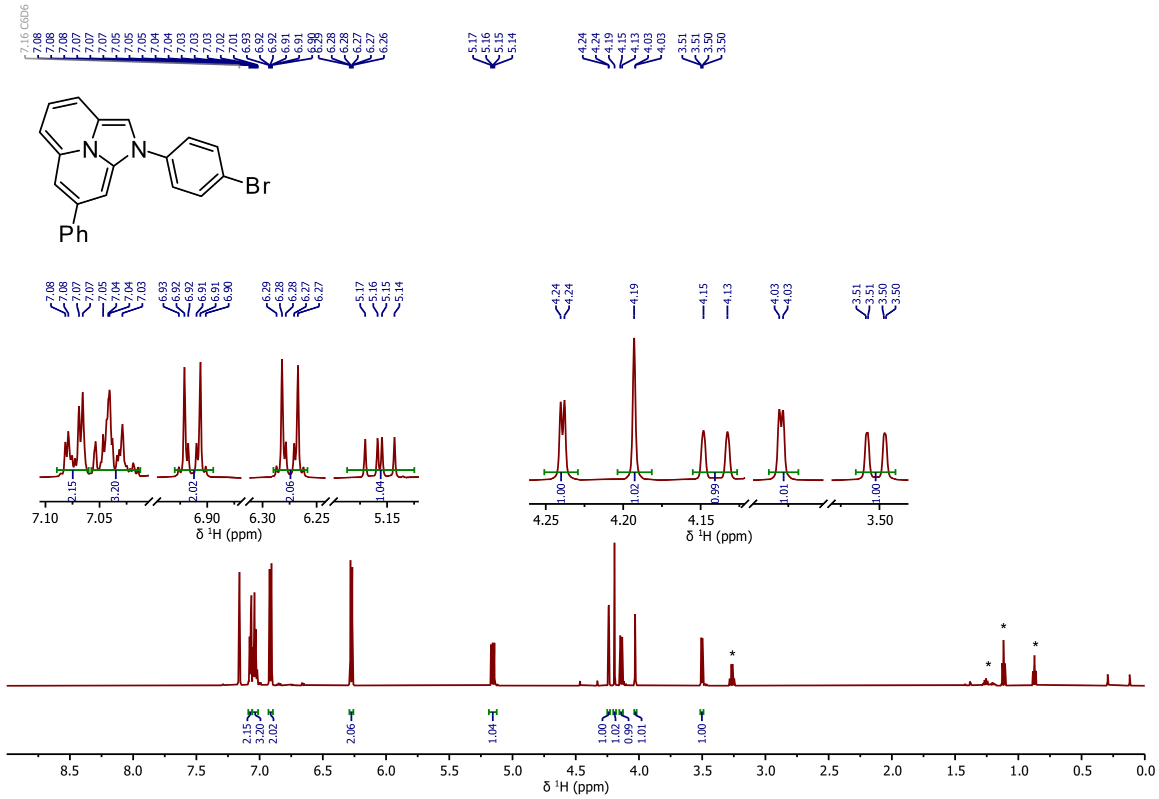

**Figure S347**: **$^{1}$H NMR** (600 MHz, $C_6D_6$, 298 K) of **6l**. * = $Et_2O$ and pentane.

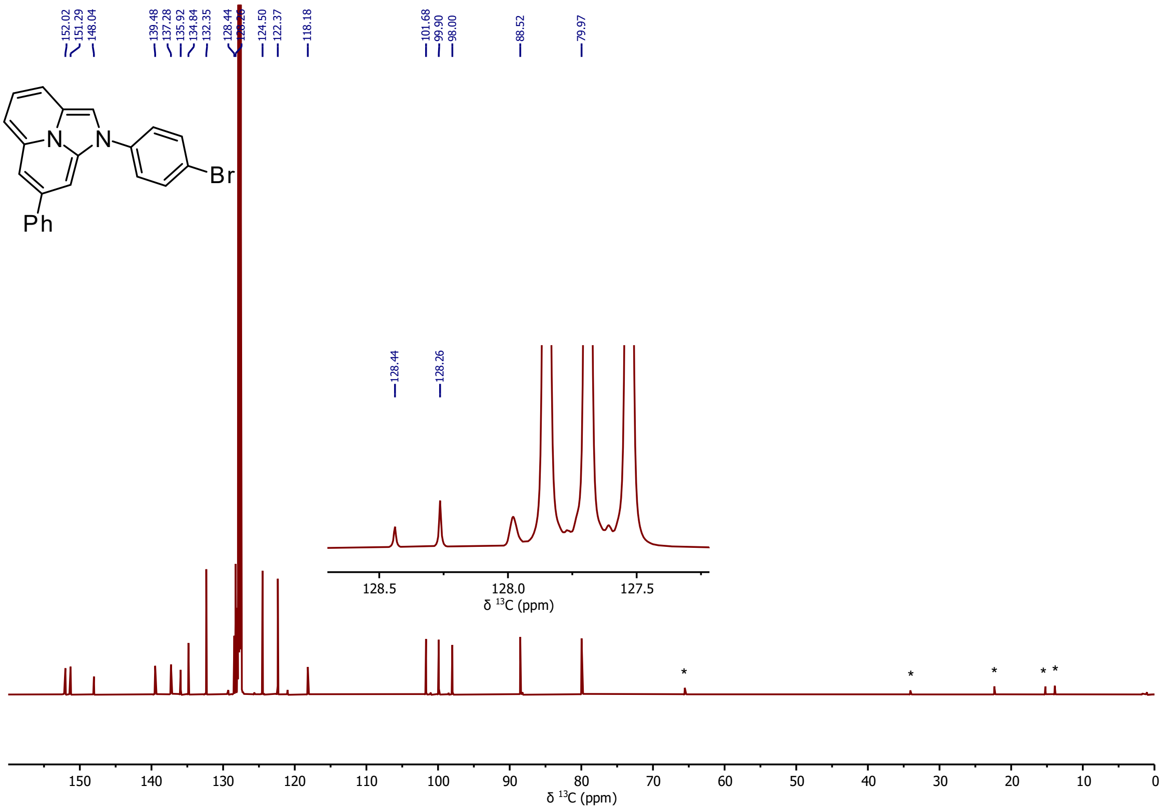

**Figure S348**: **$^{13}$C NMR** (151 MHz, $C_6D_6$, 298 K) of **6l**. * = $Et_2O$ and pentane.

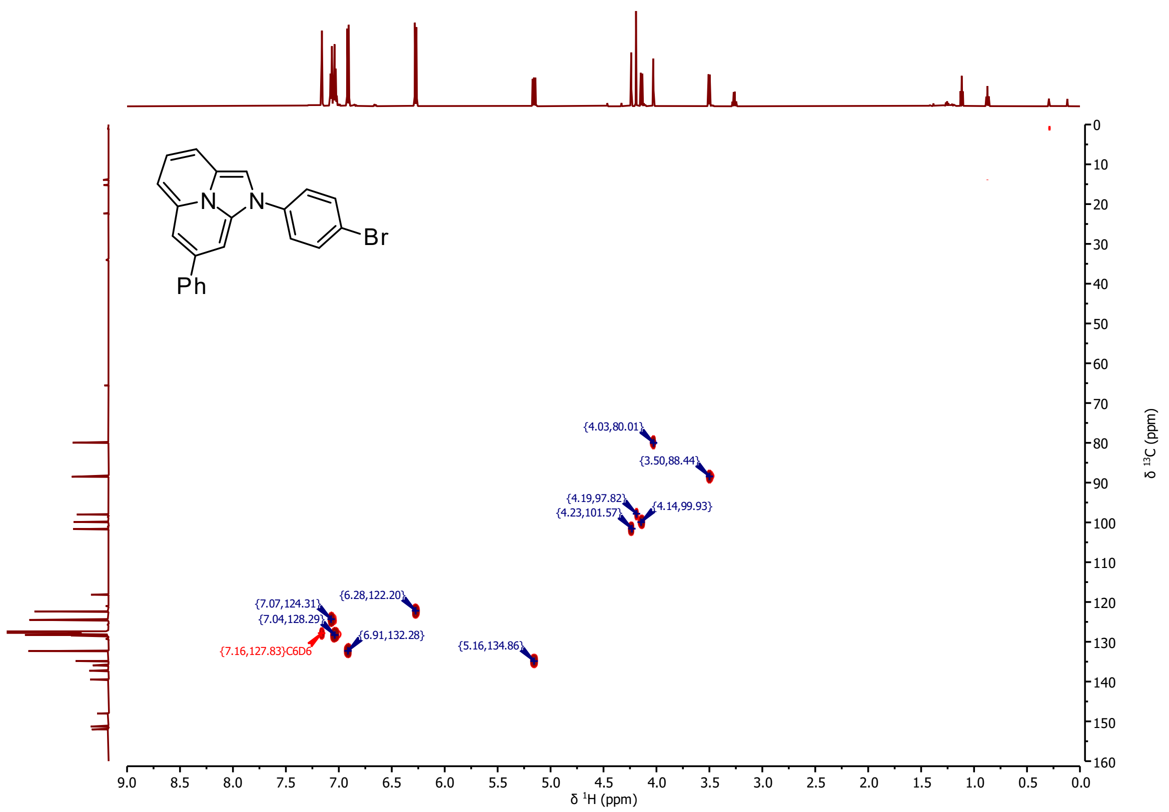

**Figure S349**: **$^1H/^{13}C$ HSQC** (600/151 MHz, $C_6D_6$, 298 K) of **6l**.

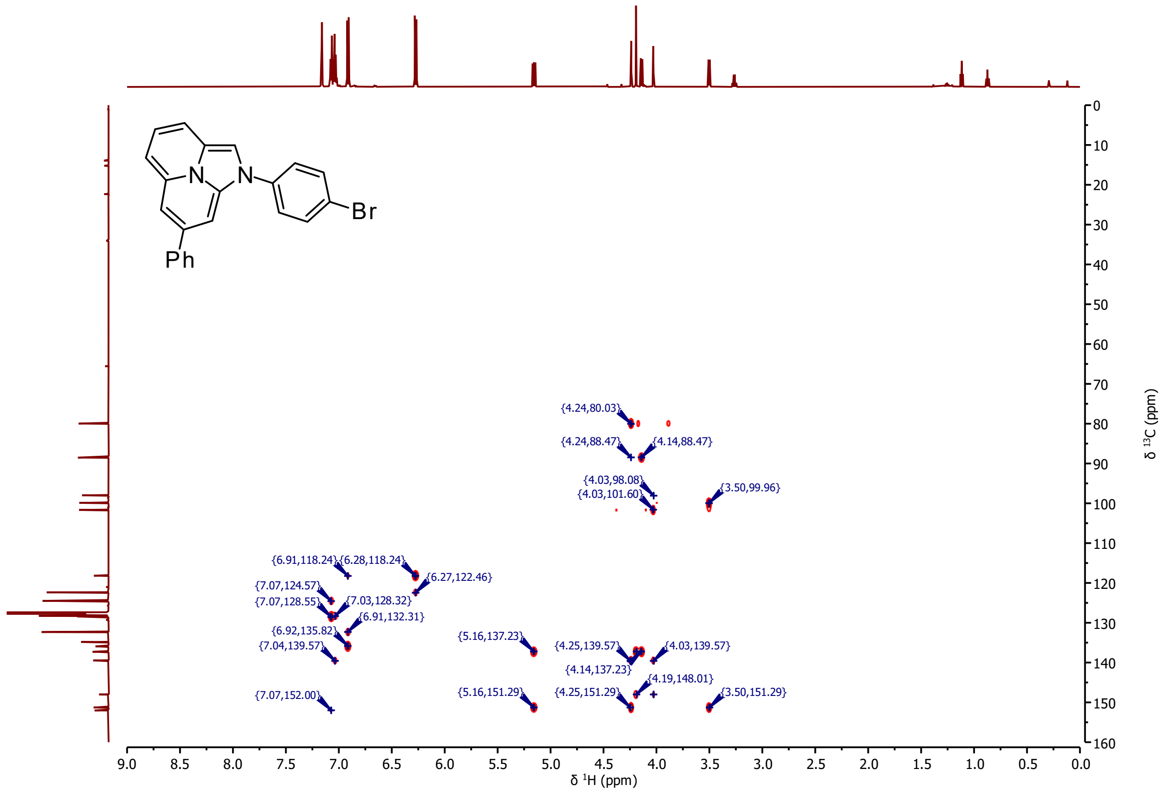

**Figure S350**: **$^1H/^{13}C$ HMBC** (600/151 MHz, $C_6D_6$, 298 K) of **6l**.

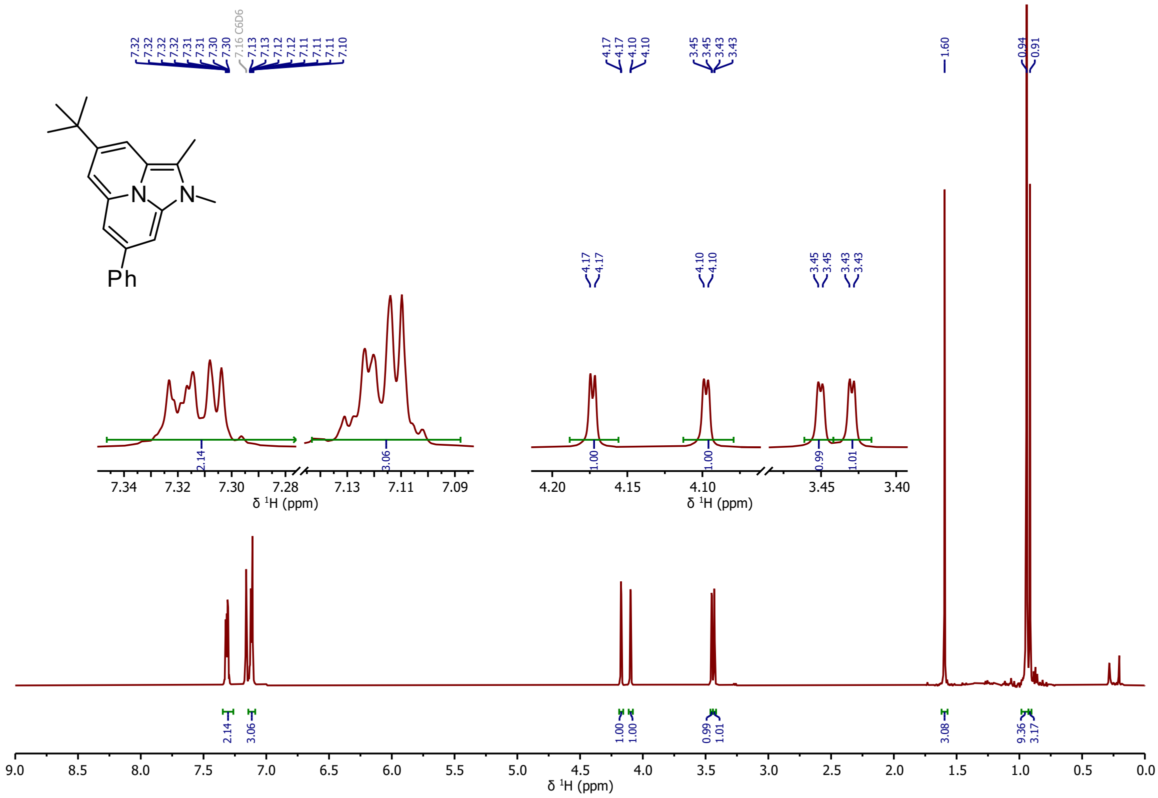


**Figure S351**: **$^{1}$H NMR** (500 MHz, $C_6D_6$, 298 K) of **6m**.

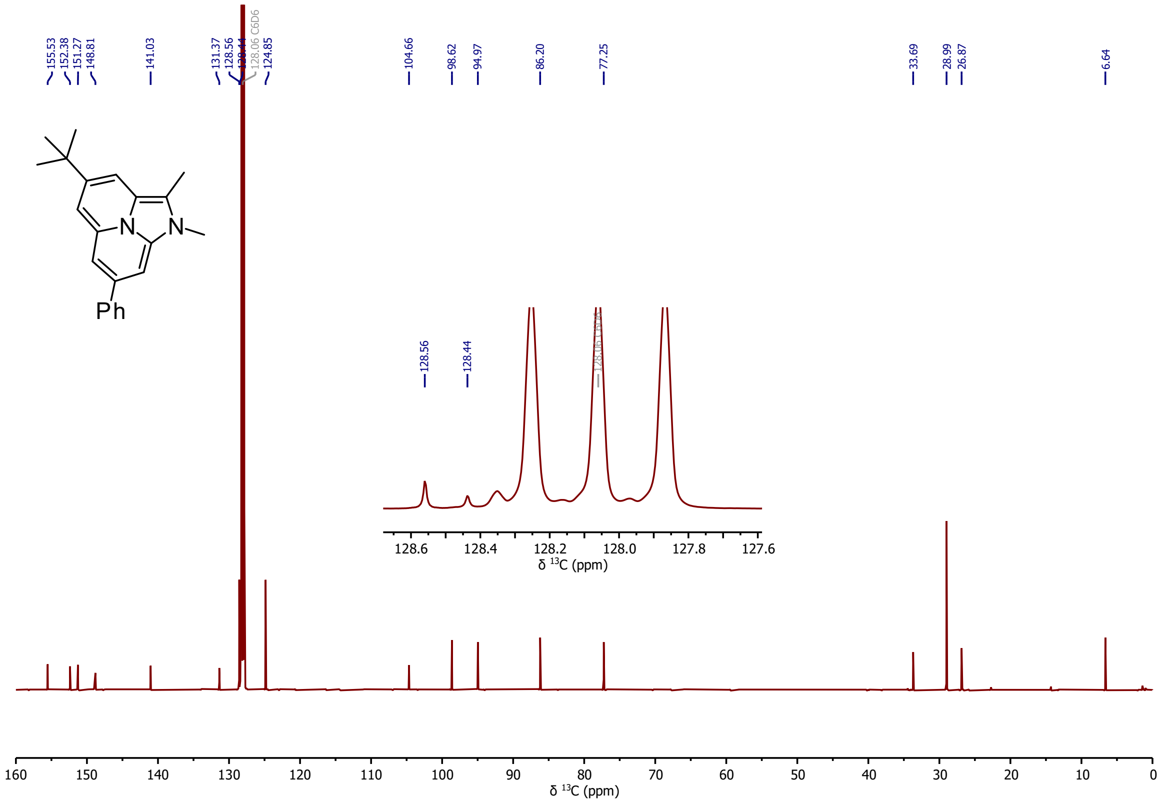


**Figure S352**: **$^{13}$C NMR** (126 MHz, $C_6D_6$, 298 K) of **6m**.

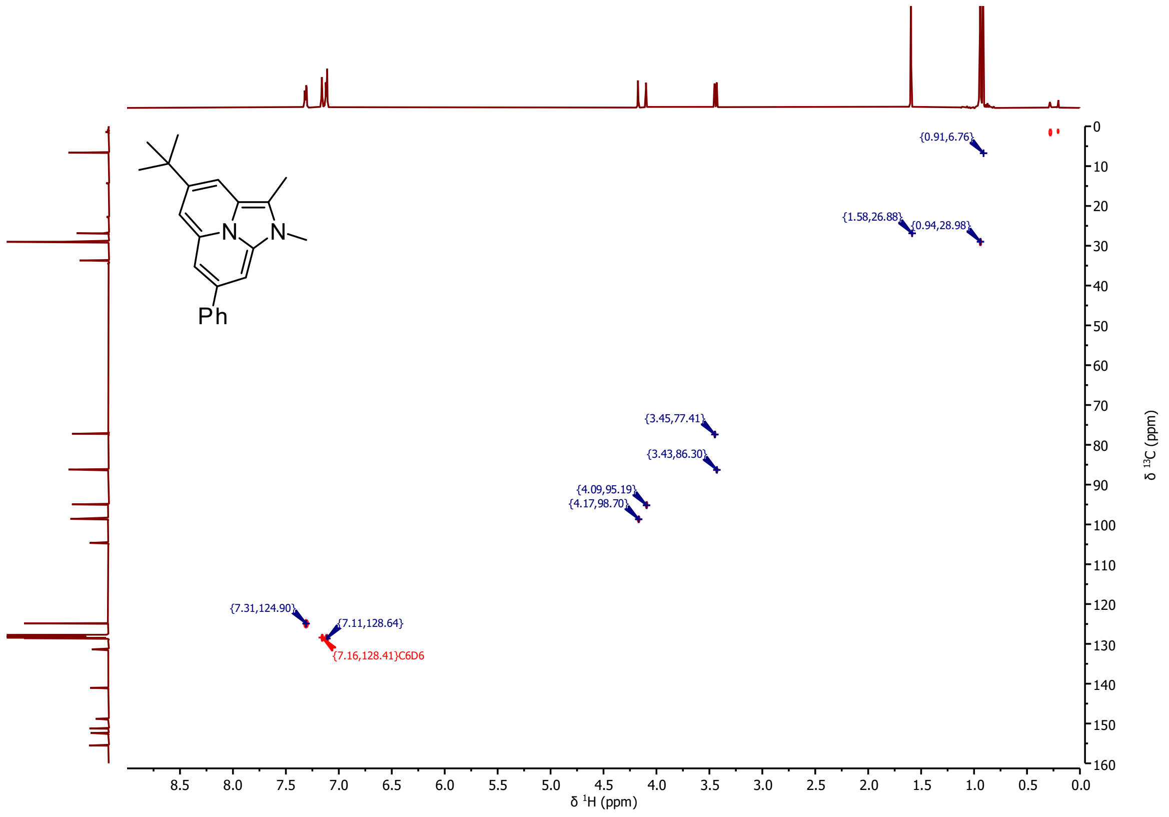

**Figure S353**: **$^{1}H/^{13}C$ HSQC** (500/126 MHz, $C_6D_6$, 298 K) of **6m**.

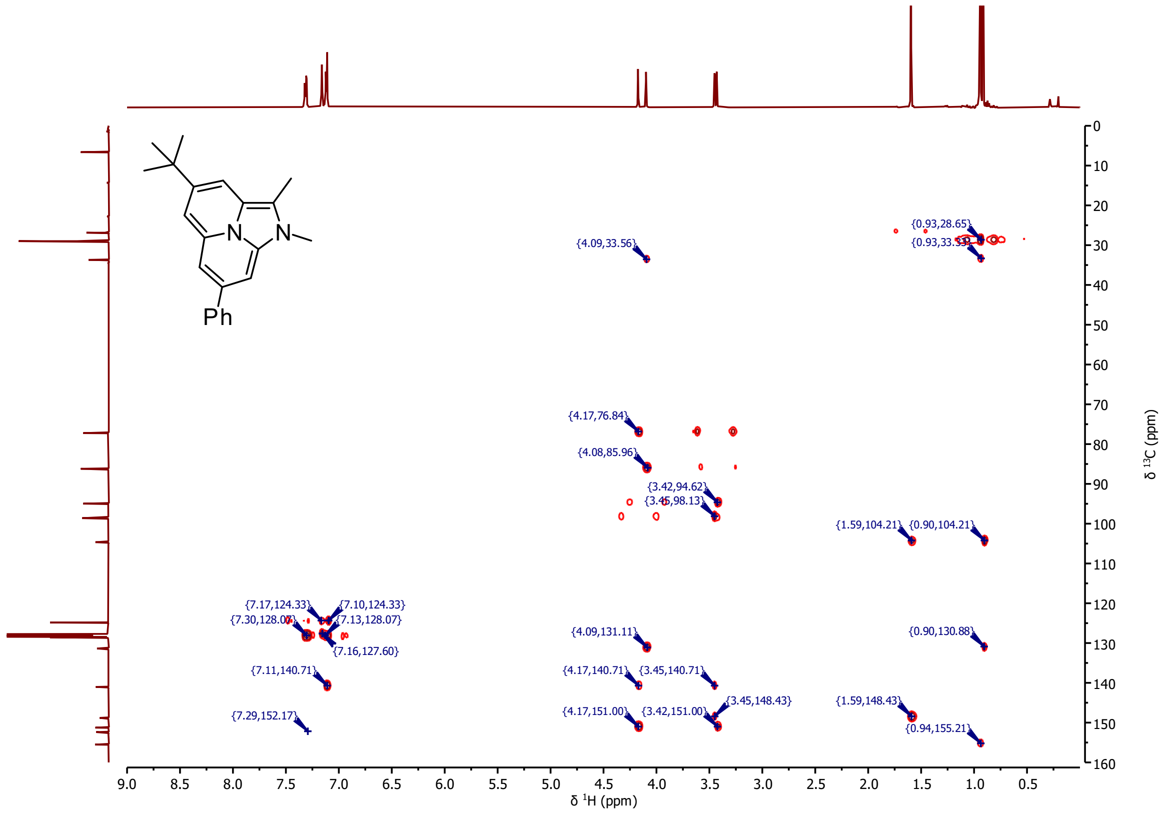

**Figure S354**: **$^{1}H/^{13}C$ HMBC** (500/126 MHz, $C_6D_6$, 298 K) of **6m**.

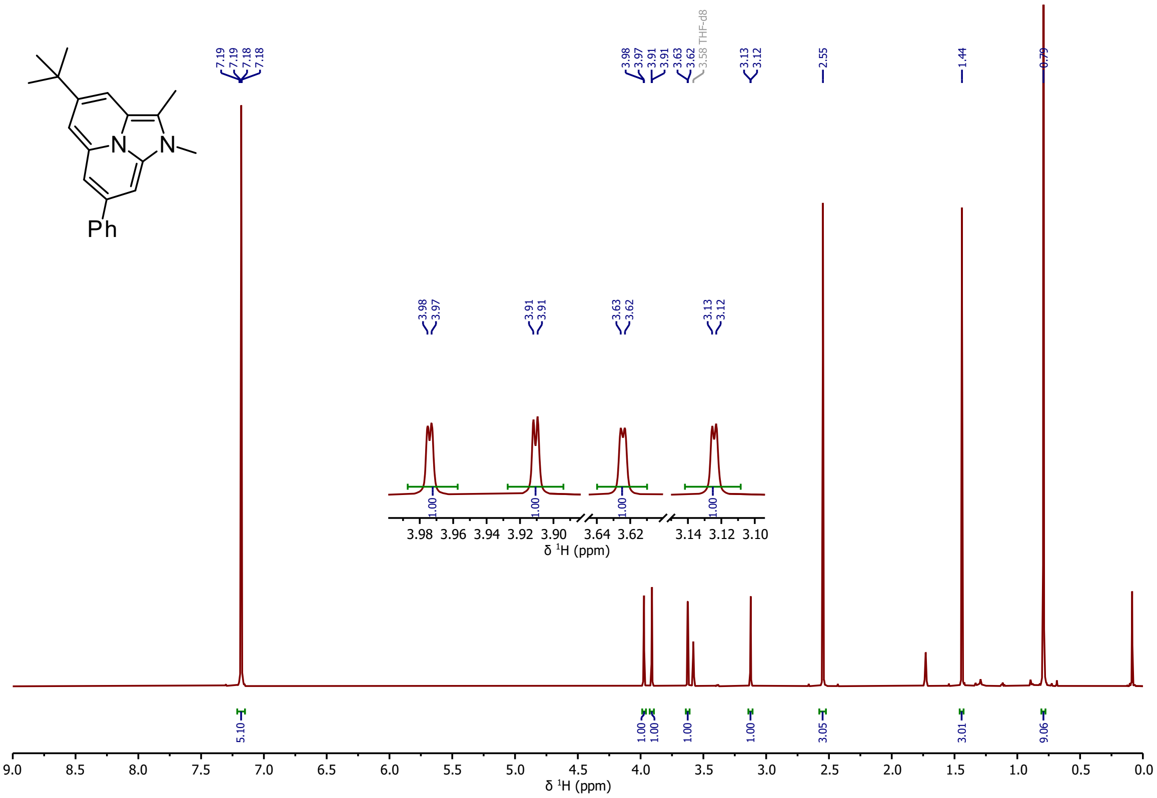


**Figure S355**: **$^1$H NMR** (600 MHz, THF-$d_8$, 298 K) of **6m**.

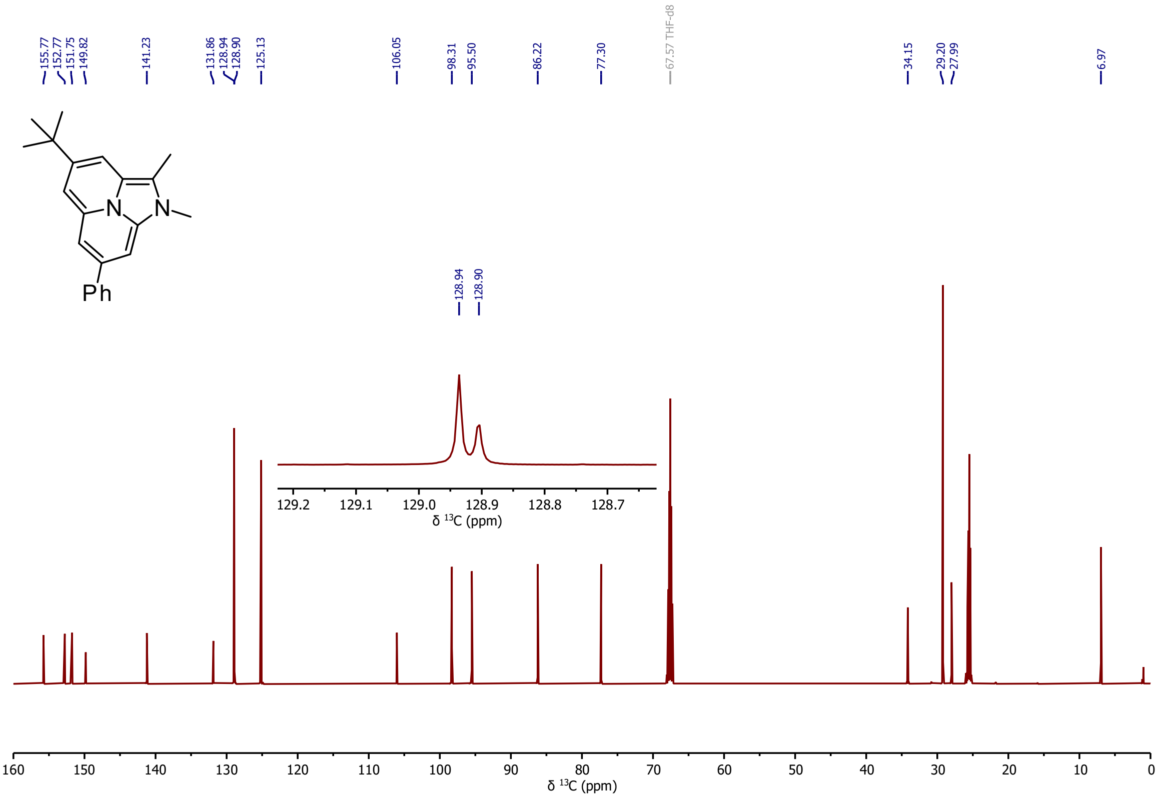


**Figure S356**: **$^{13}$C NMR** (151 MHz, THF-$d_8$, 298 K) of **6m**.

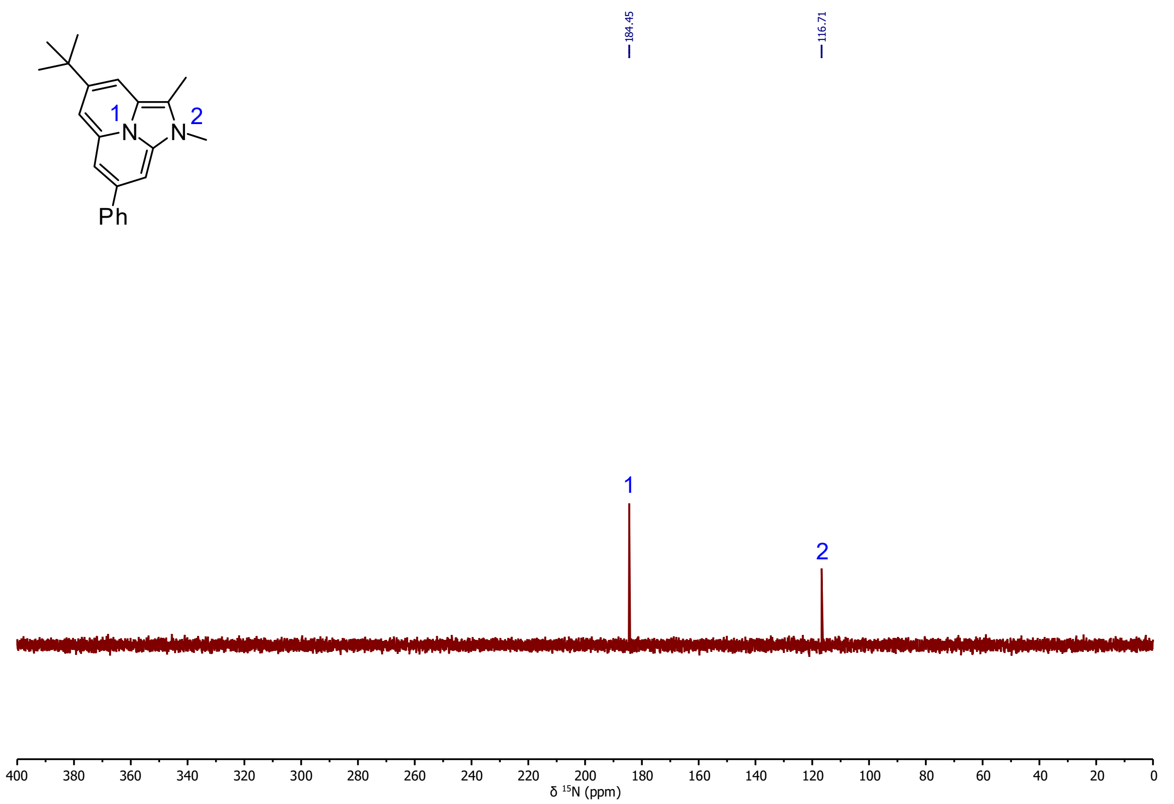

**Figure S357**: $^{15}$**N NMR** (61 MHz, THF-$d_8$, 298 K) of **6m**.

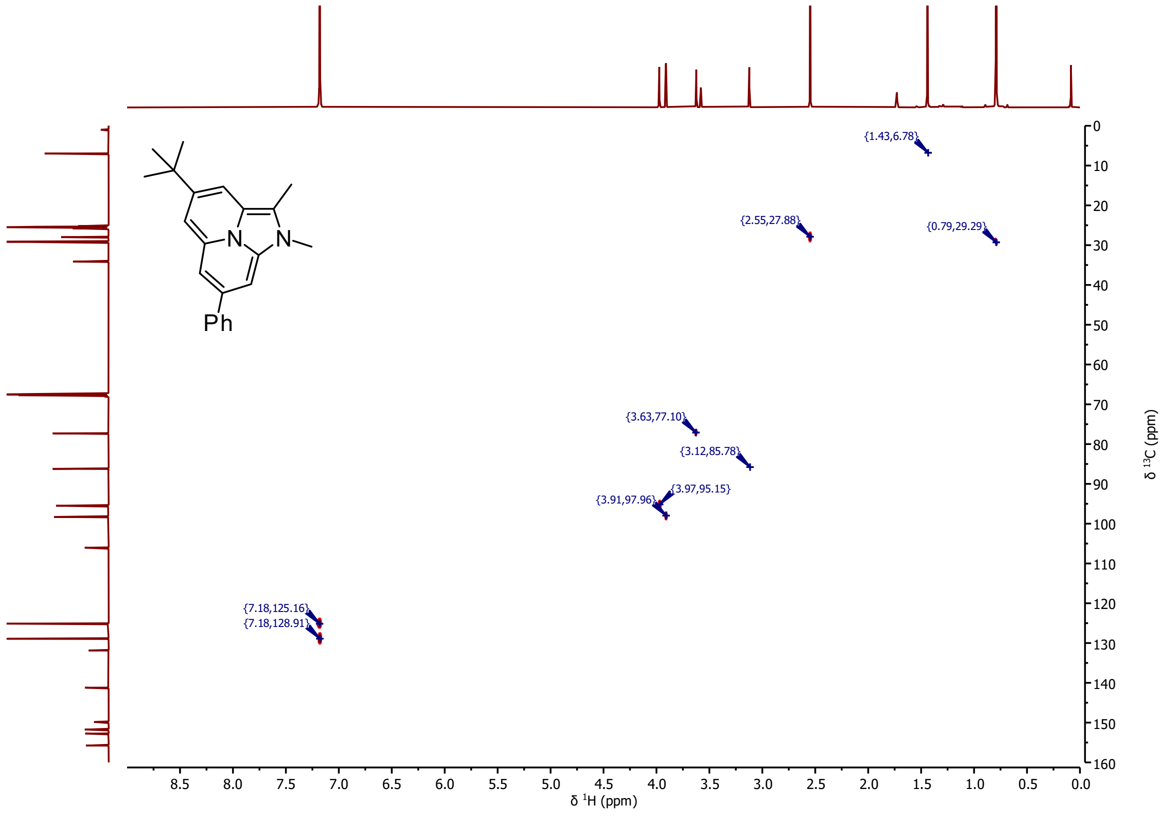

**Figure S358**: $^{1}$**H/**$^{13}$**C HSQC** (600/151 MHz, THF-$d_8$, 298 K) of **6m**.

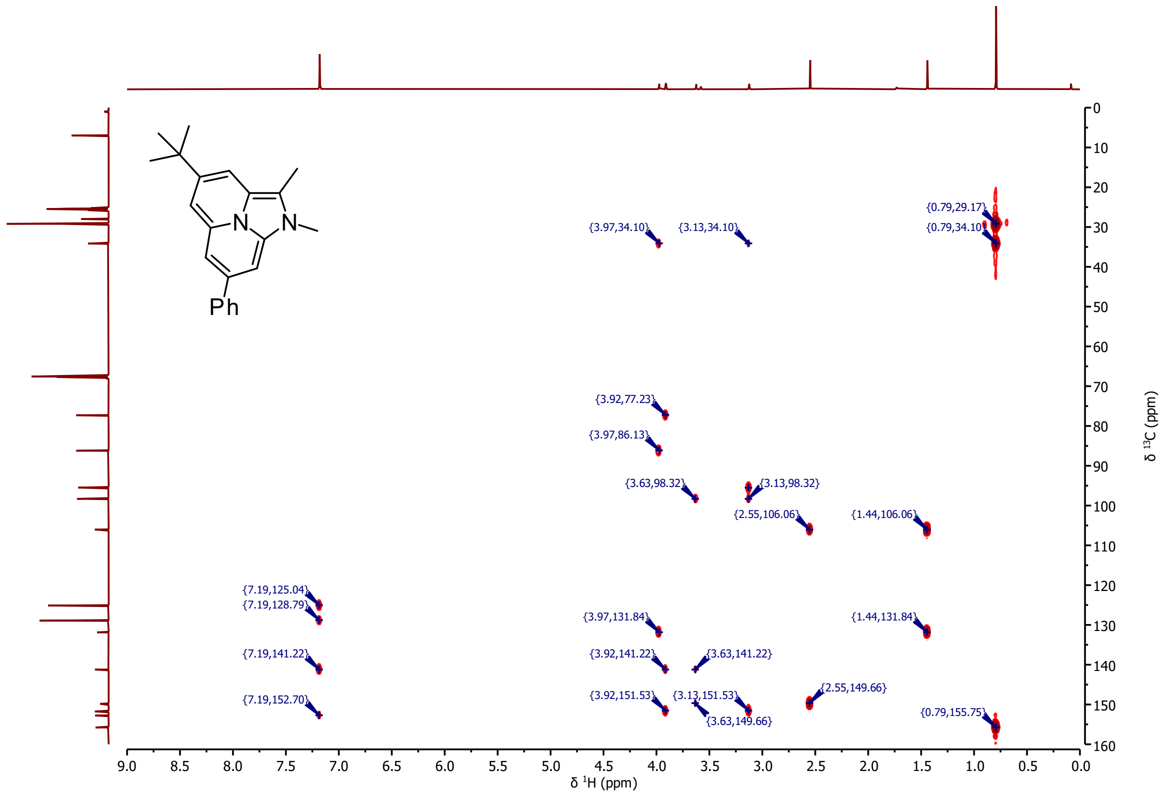

**Figure S359**: **$^{1}$H/$^{13}$C HMBC** (600/151 MHz, THF-$d_8$, 298 K) of **6m**.

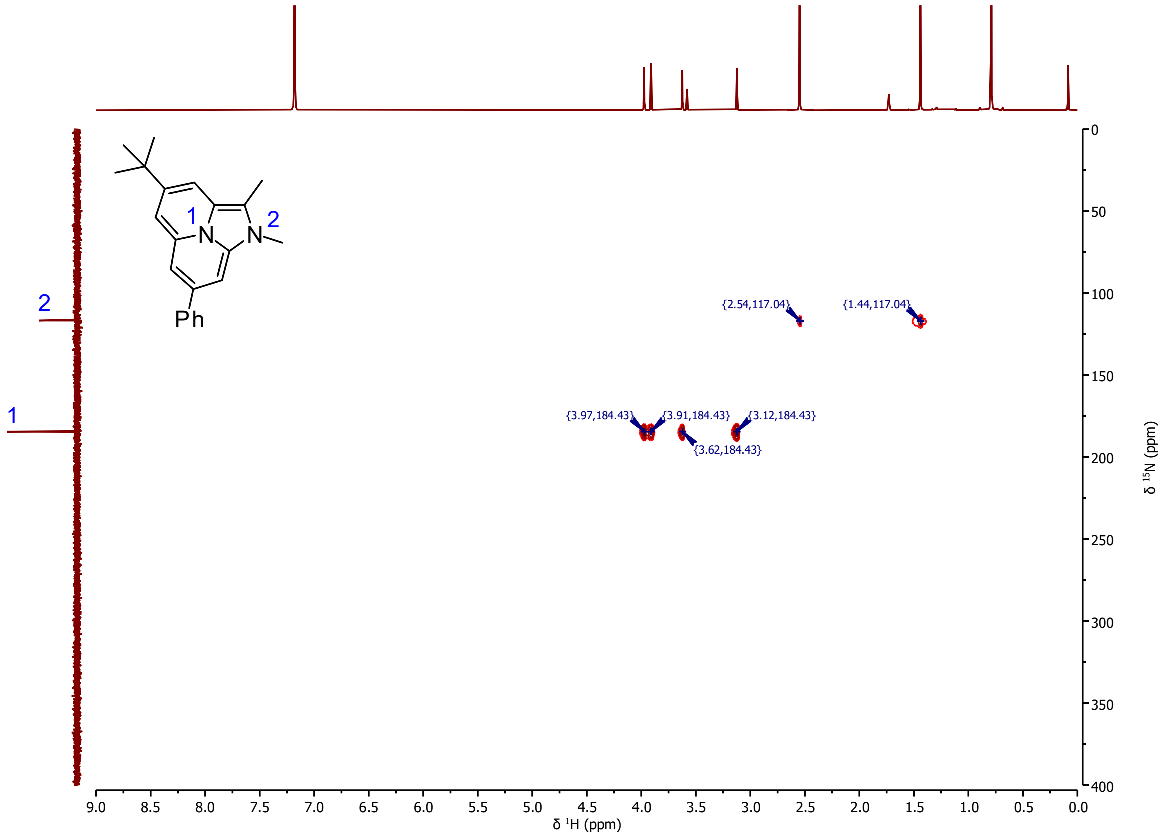

**Figure S360**: **$^{1}$H/$^{15}$N HMBC** (600/61 MHz, THF-$d_8$, 298 K) of **6m**.

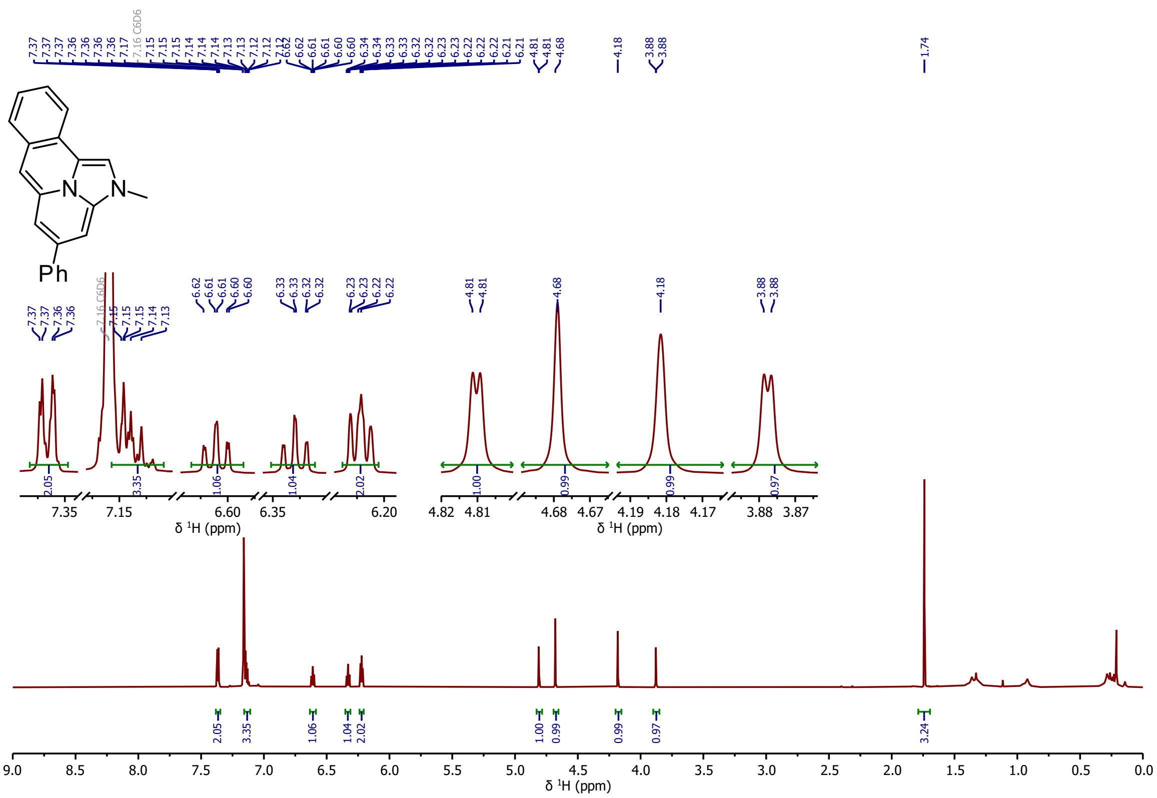

**Figure S361**: **$^1$H NMR** (700 MHz, $C_6D_6$, 298 K) of **6n**.

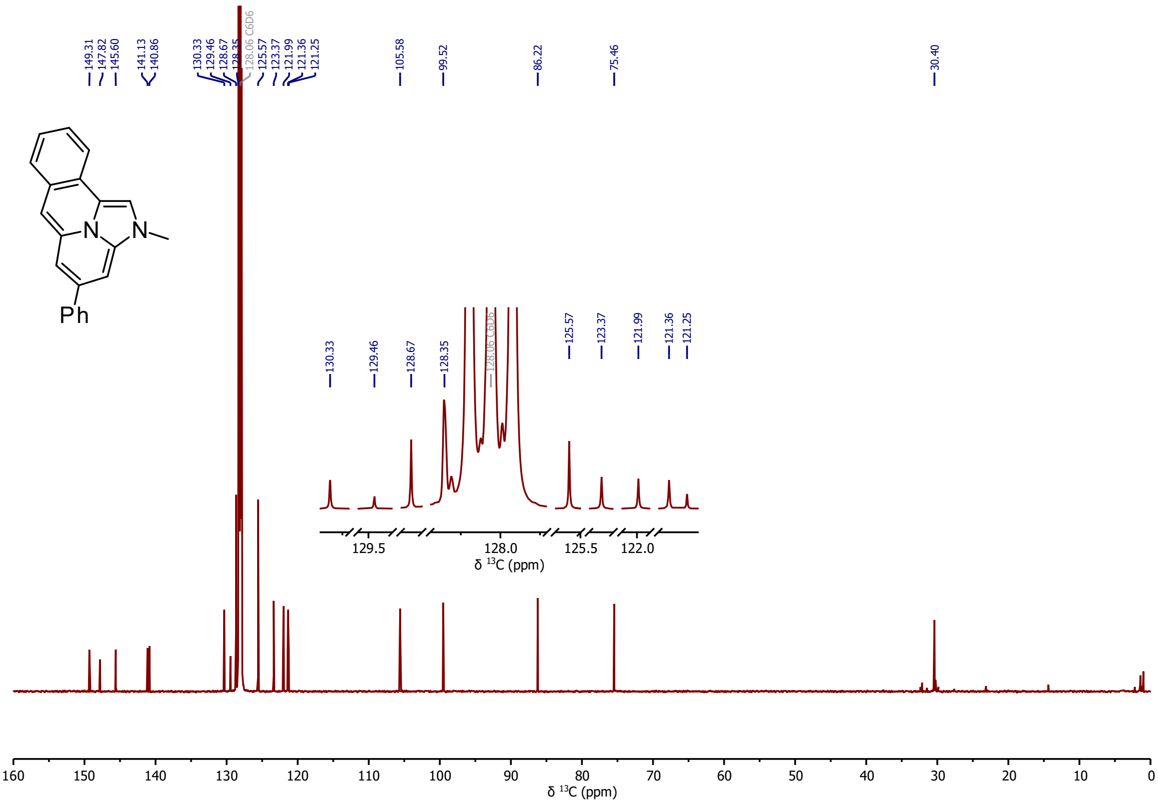

**Figure S362**: **$^{13}$C NMR** (176 MHz, $C_6D_6$, 298 K) of **6n**.

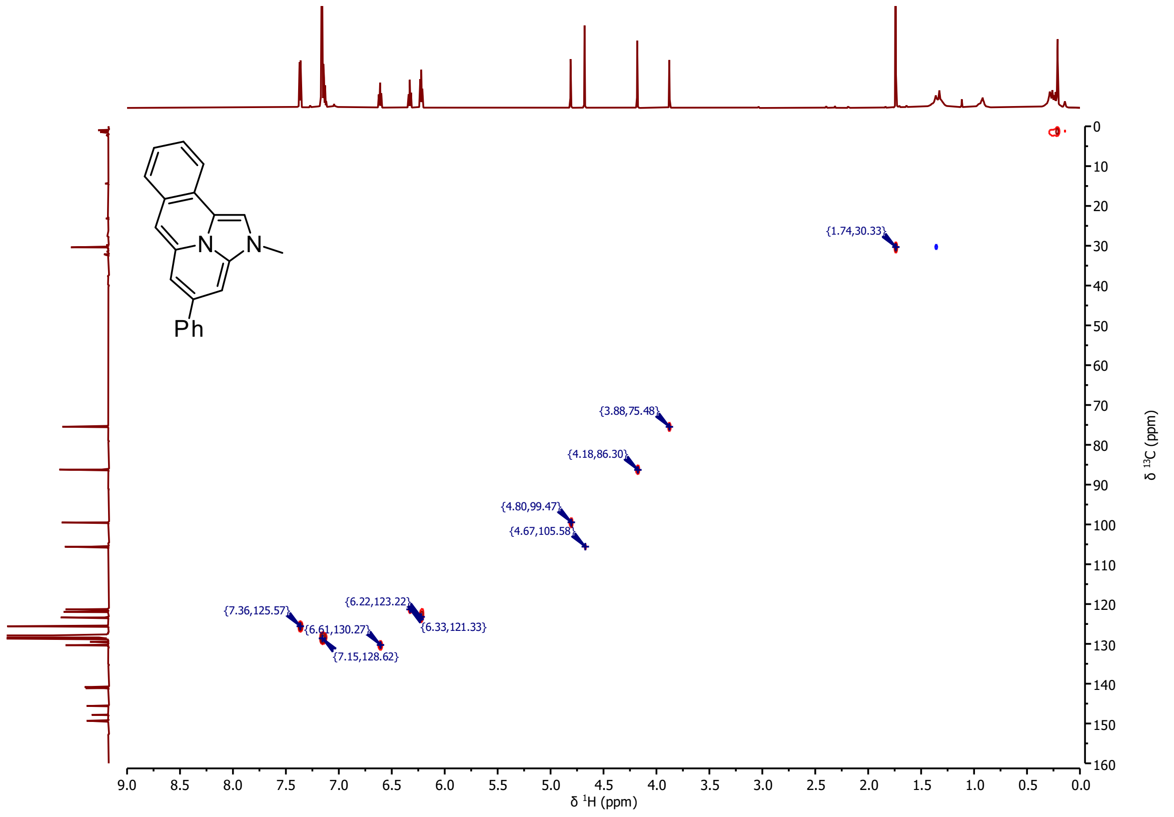

**Figure S363**: **$^1H/^{13}C$ HSQC** (700/176 MHz, $C_6D_6$, 298 K) of **6n**.

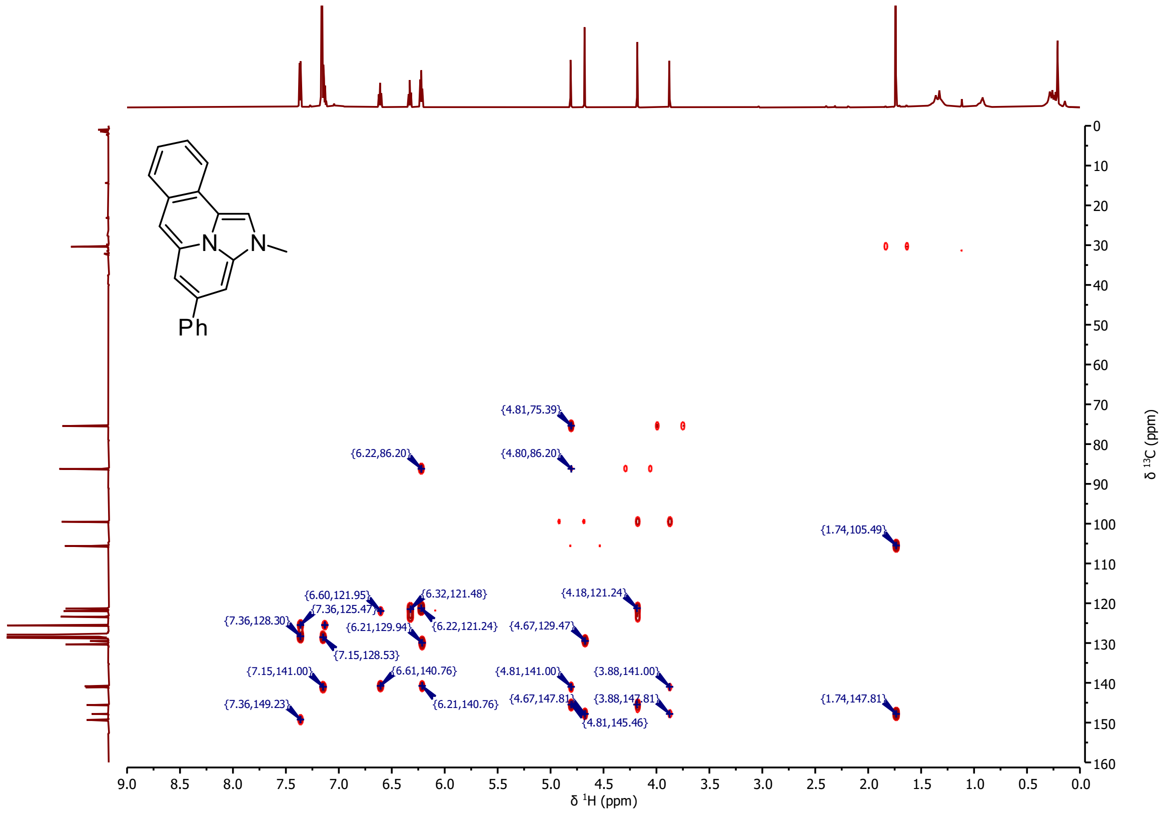

**Figure S364**: **$^1H/^{13}C$ HMBC** (700/176 MHz, $C_6D_6$, 298 K) of **6n**.

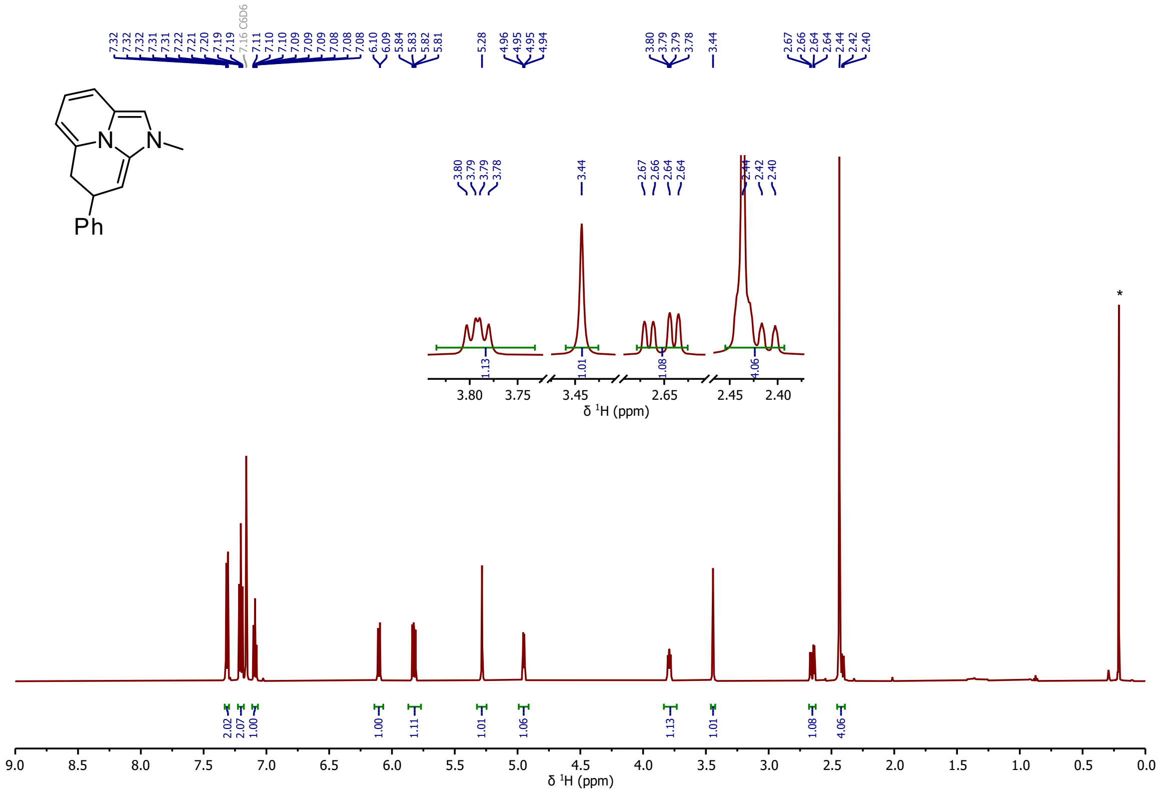


**Figure S365**: **$^1$H NMR** (600 MHz, $C_6D_6$, 298 K) of **6o**. * = KHMDS.

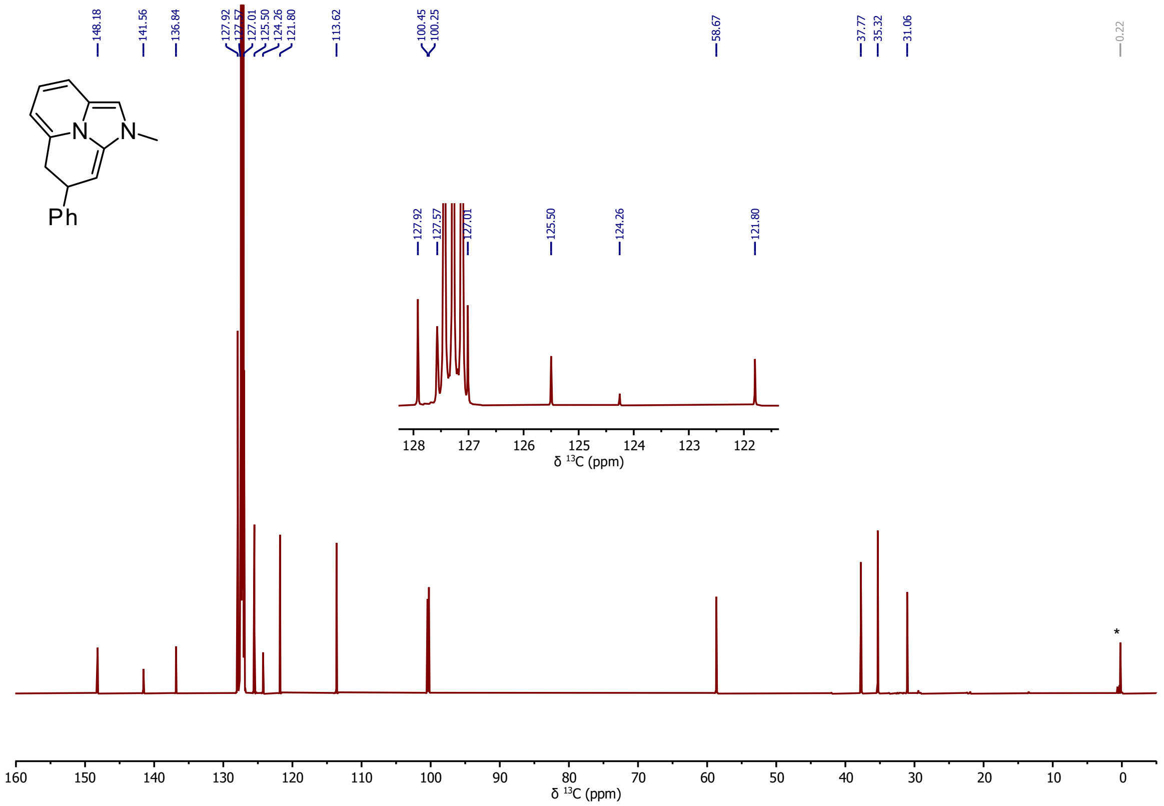


**Figure S366**: **$^{13}$C NMR** (151 MHz, $C_6D_6$, 298 K) of **6o**. * = KHMDS.

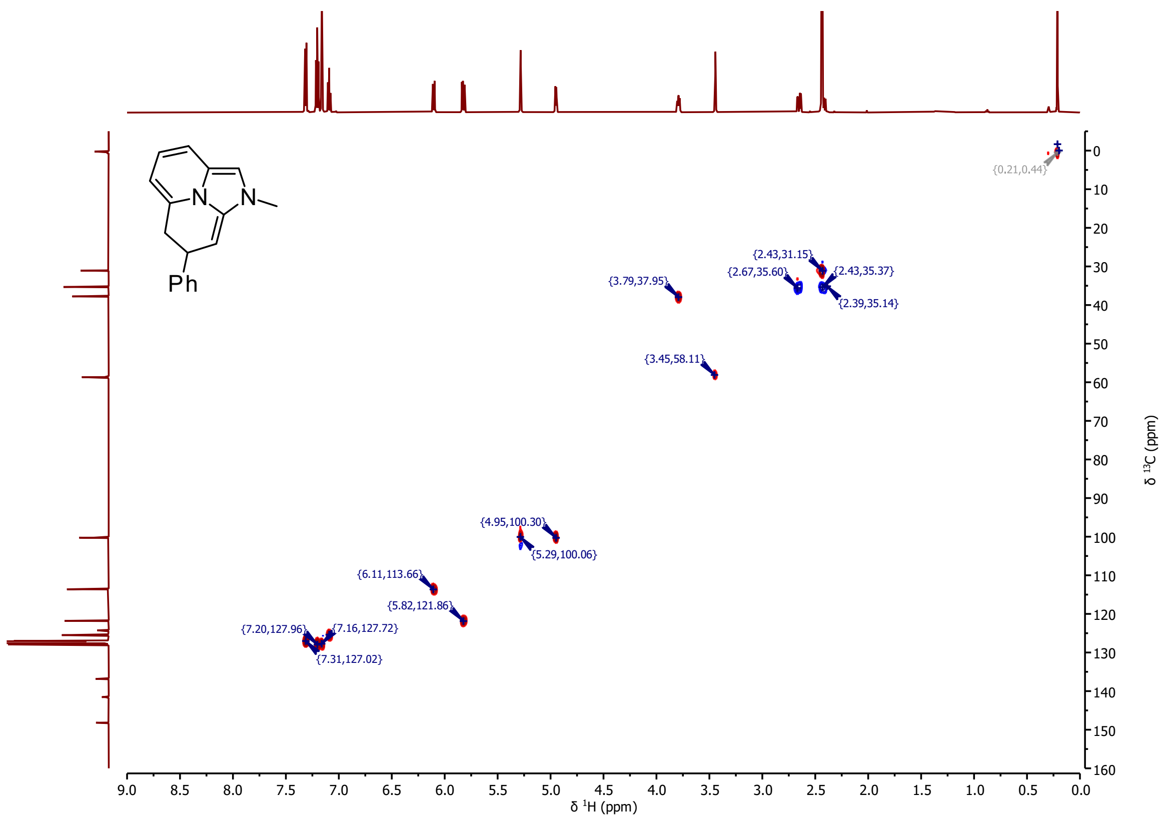

**Figure S367**: $^{1}$**H/**$^{13}$**C HSQC** (600/151 MHz, $C_6D_6$, 298 K) of **6o**.

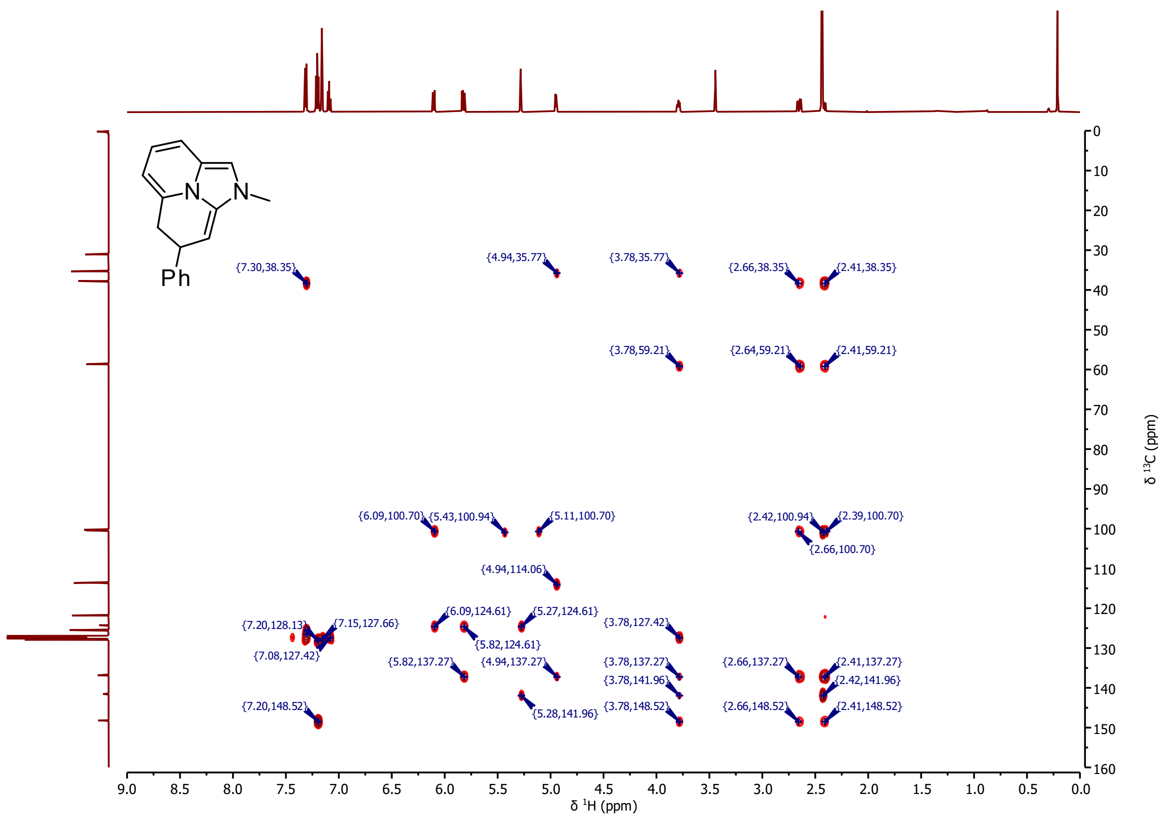

**Figure S368**: $^{1}$**H/**$^{13}$**C HMBC** (600/151 MHz, $C_6D_6$, 298 K) of **6o**.

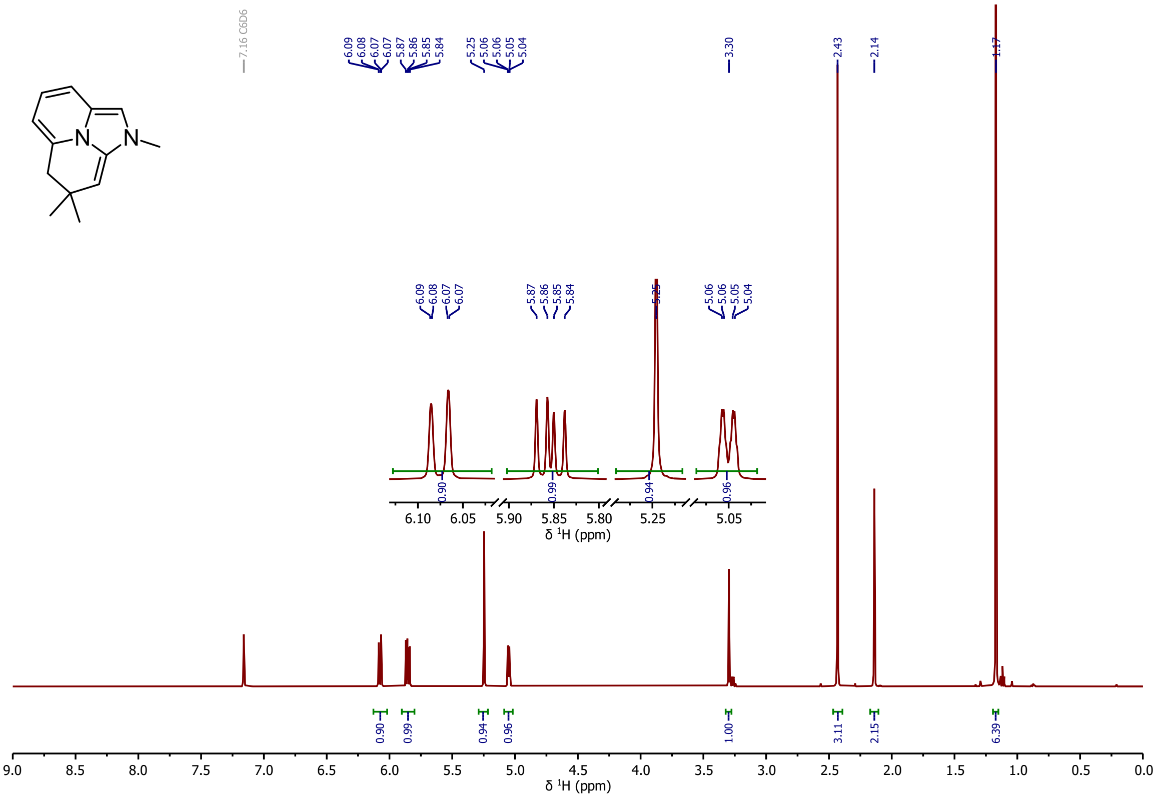


**Figure S369**: **$^{1}$H NMR** (500 MHz, $C_6D_6$, 298 K) of **6p**.

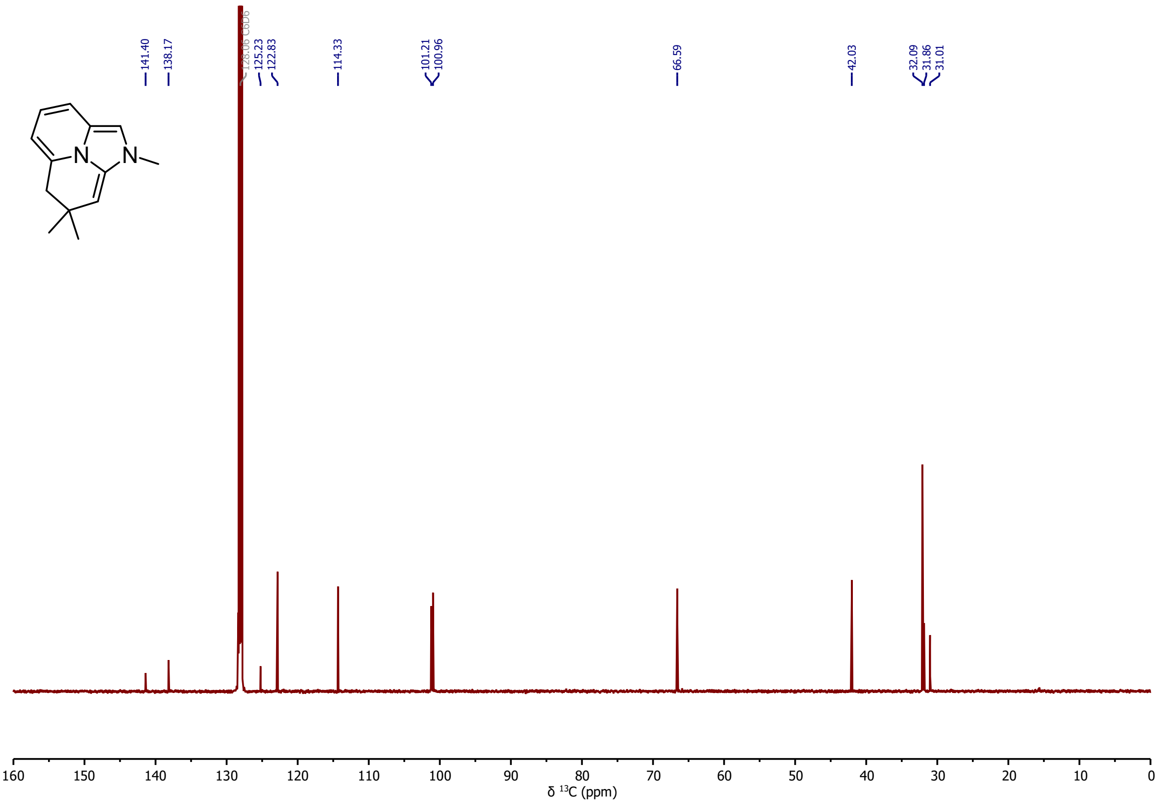


**Figure S370**: **$^{13}$C NMR** (126 MHz, $C_6D_6$, 298 K) of **6p**.

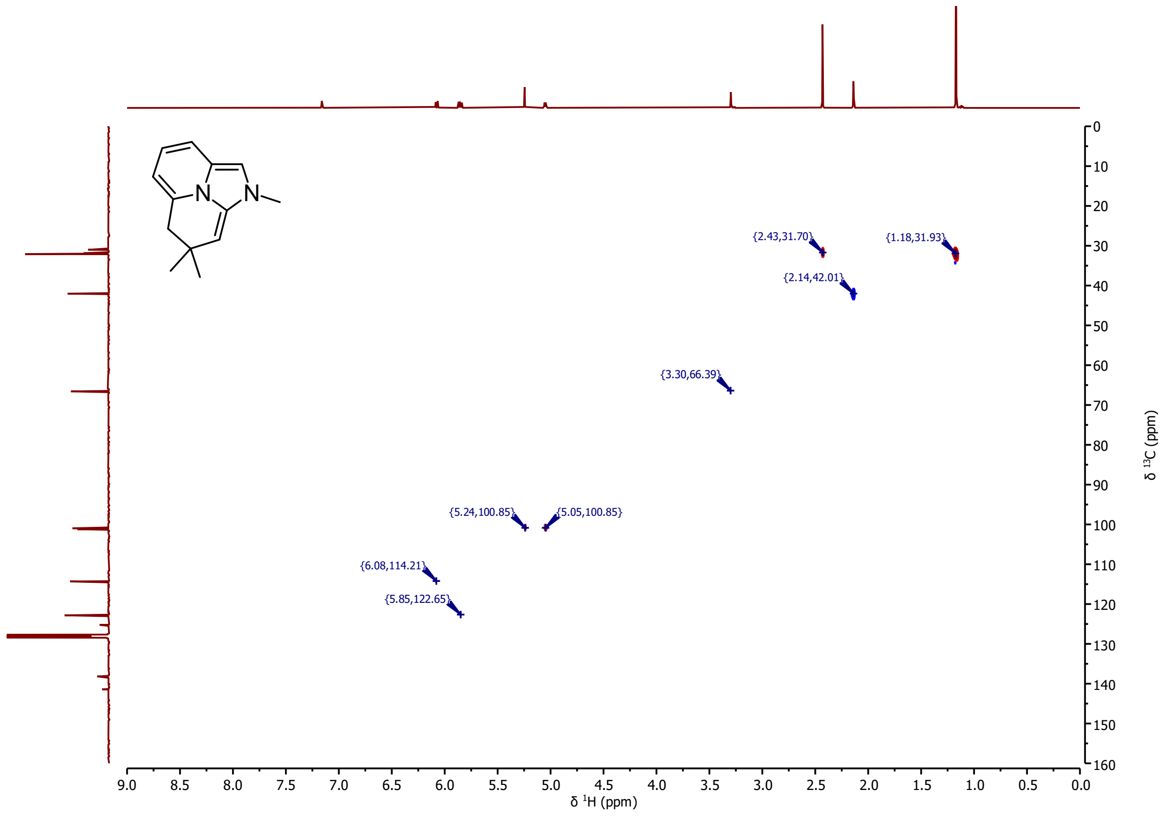

**Figure S371**: **$^1$H/$^{13}$C HSQC** (500/126 MHz, $C_6D_6$, 298 K) of **6p**.

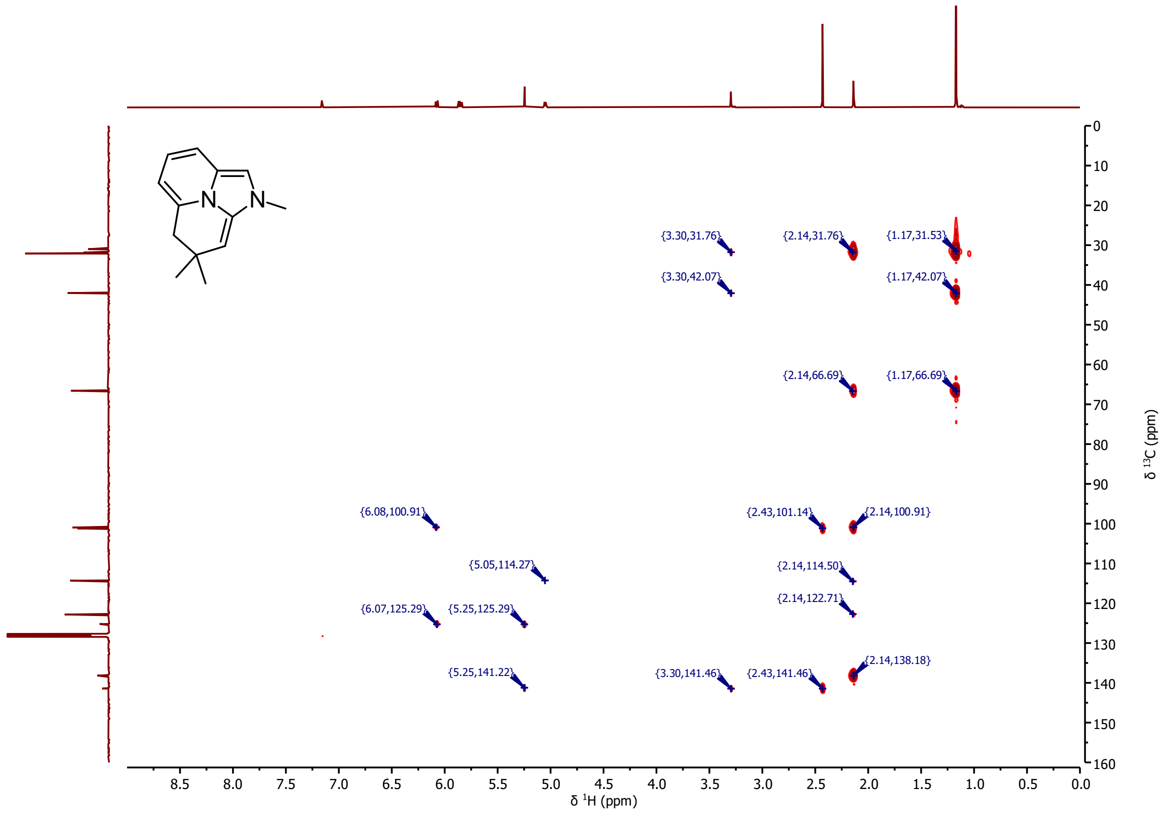

**Figure S372**: **$^1$H/$^{13}$C HMBC** (500/126 MHz, $C_6D_6$, 298 K) of **6p**.

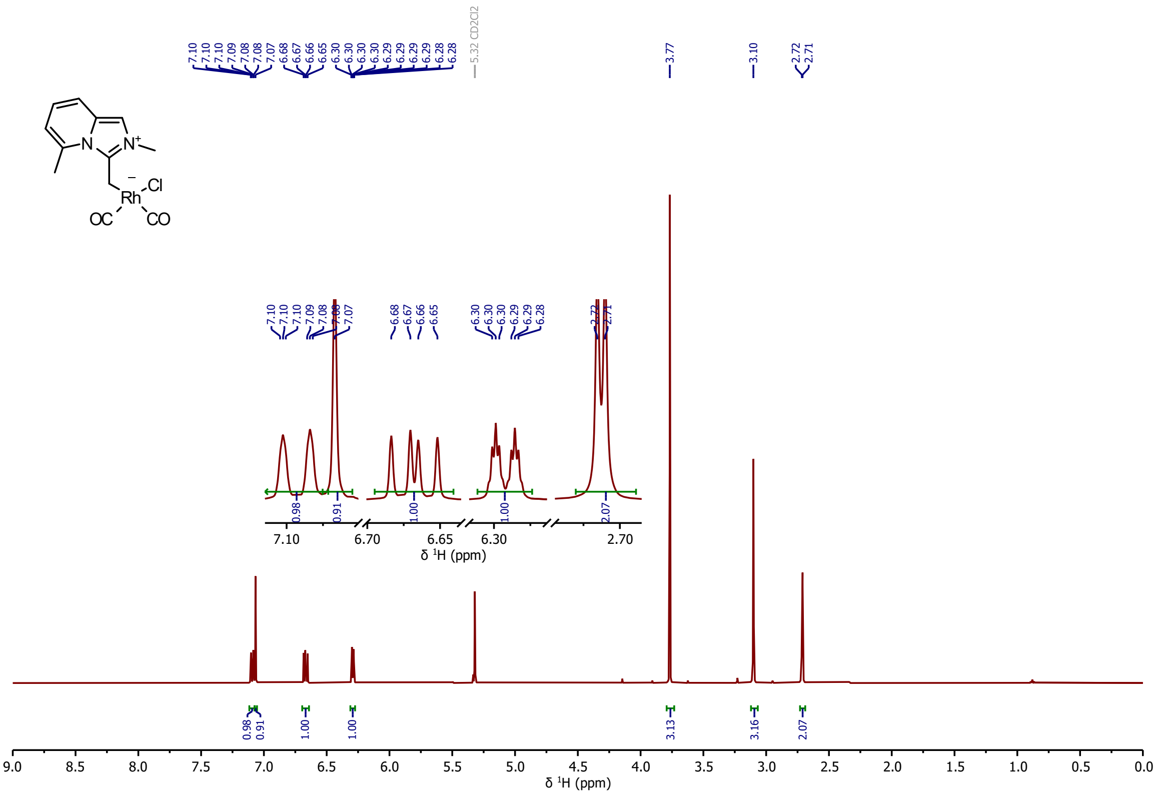

**Figure S373**: **$^{1}$H NMR** (500 MHz, $CD_2Cl_2$, 298 K) of **3a**.

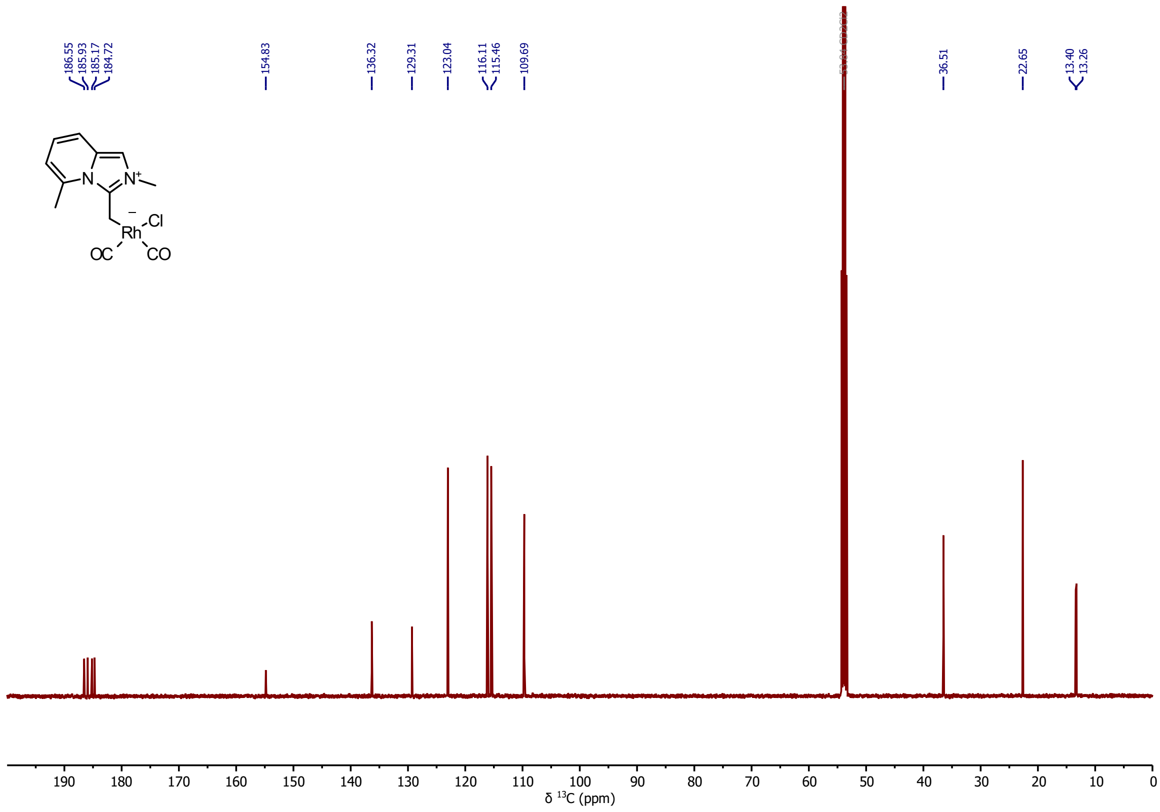

**Figure S374**: **$^{13}$C NMR** (126 MHz, $CD_2Cl_2$, 298 K) of **3a**.

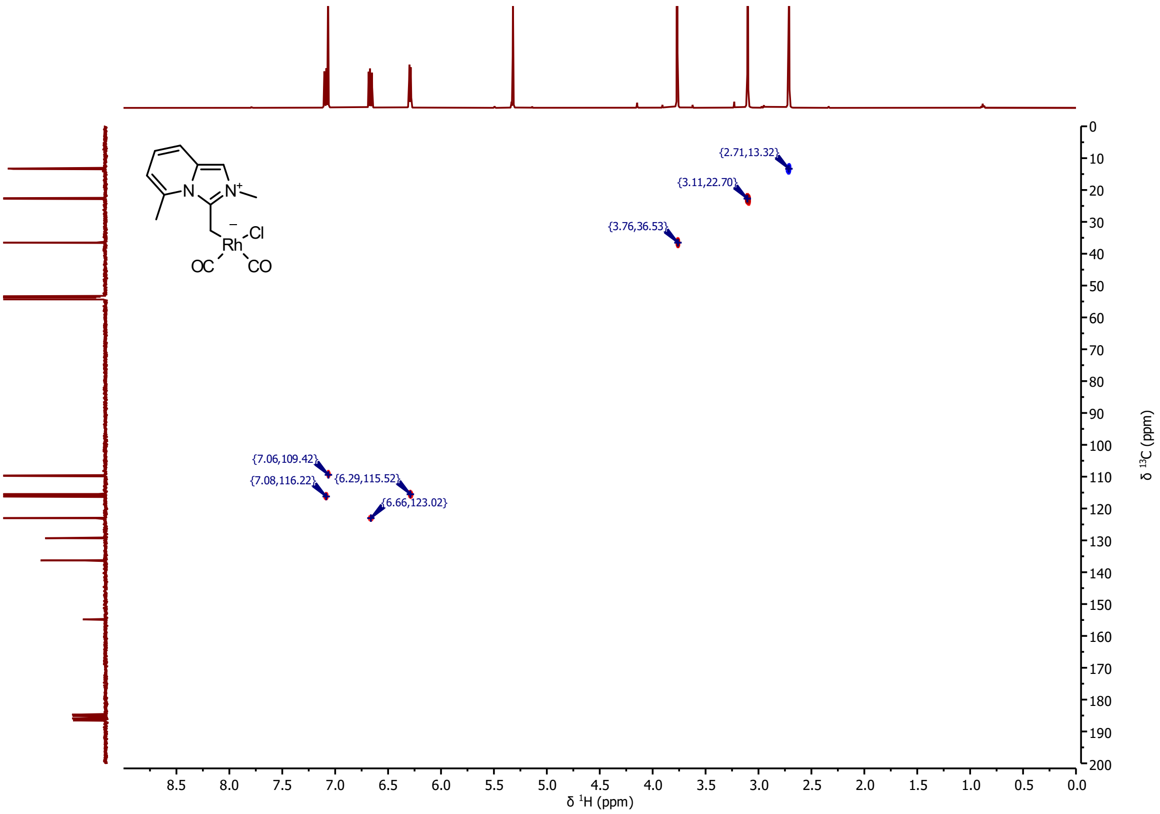


**Figure S375**: $^{1}$**H/**$^{13}$**C HSQC** (500/126 MHz, $CD_2Cl_2$, 298 K) of **3a**.

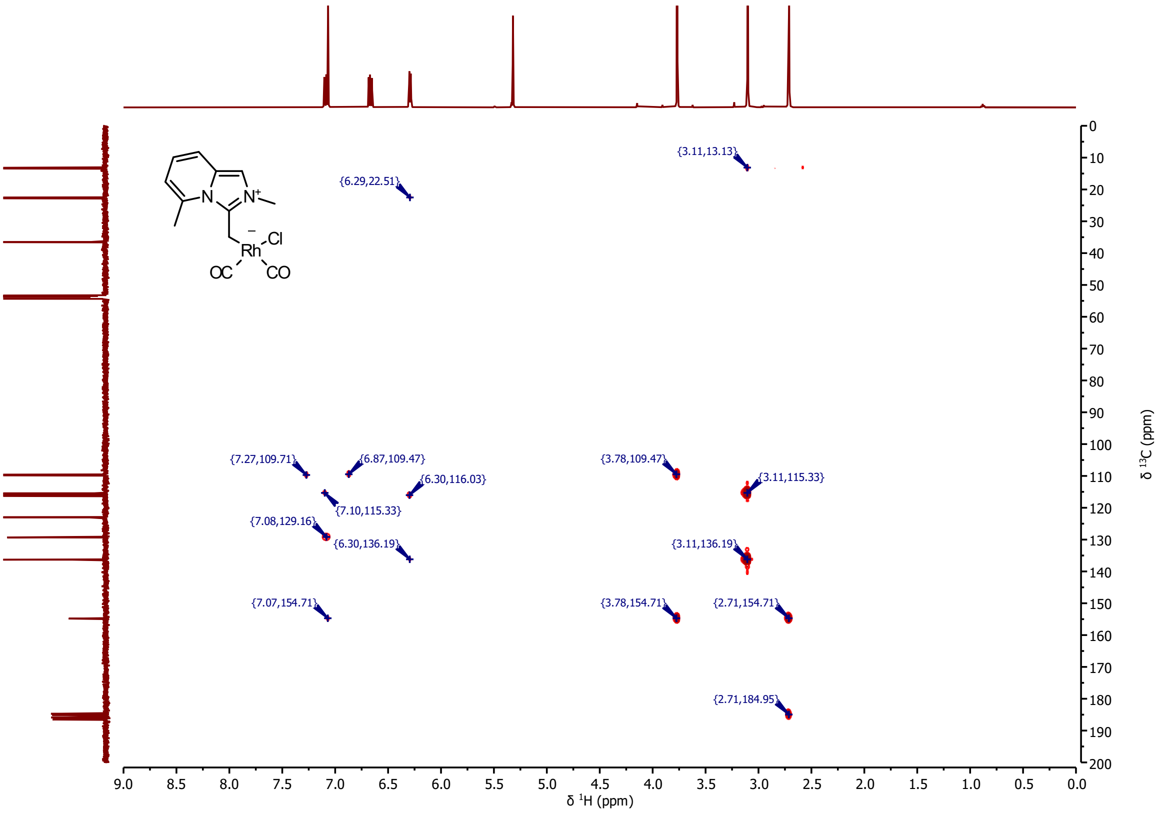


**Figure S376**: $^{1}$**H/**$^{13}$**C HMBC** (500/126 MHz, $CD_2Cl_2$, 298 K) of **3a**.

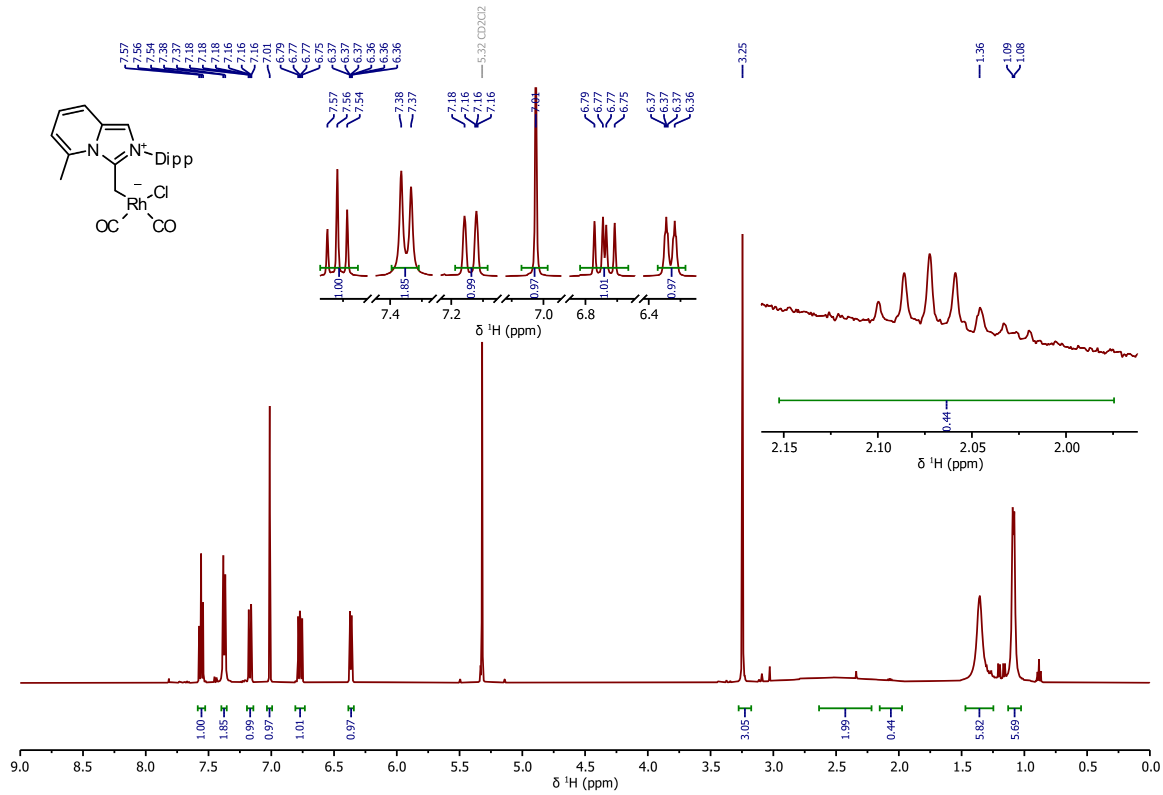


**Figure S377**: **$^1$H NMR** (500 MHz, $CD_2Cl_2$, 298 K) of **3c**.

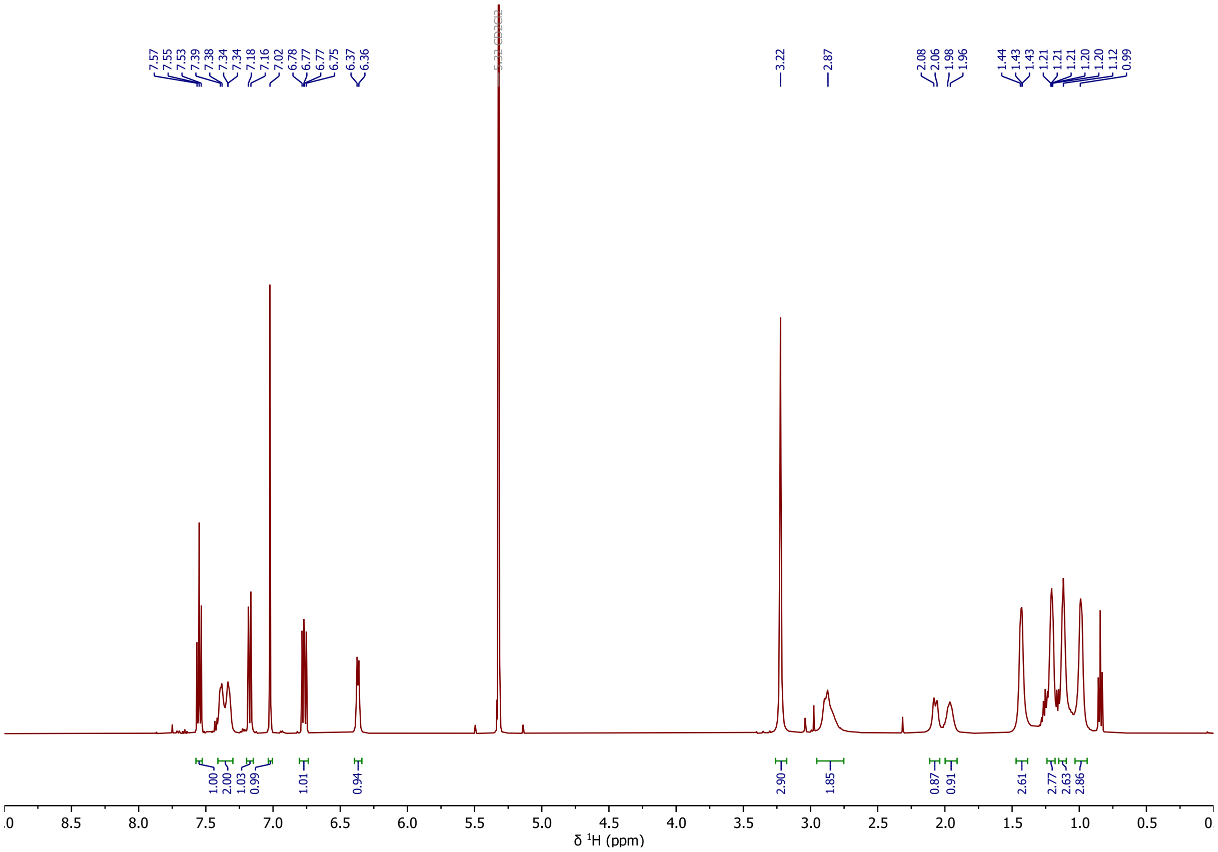


**Figure S378**: **$^1$H NMR** (500 MHz, $CD_2Cl_2$, 253 K) of **3c**.

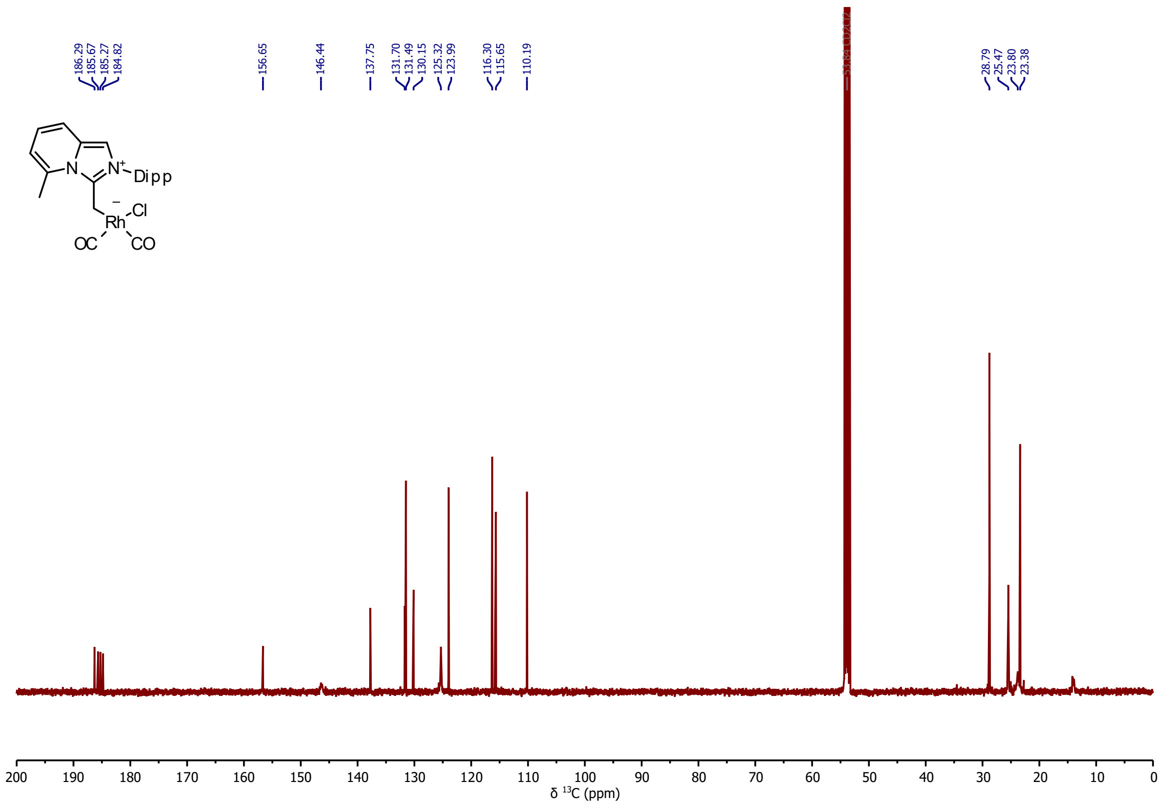


**Figure S379**: **$^{13}$C NMR** (126 MHz, $CD_2Cl_2$, 298 K) of **3c**.

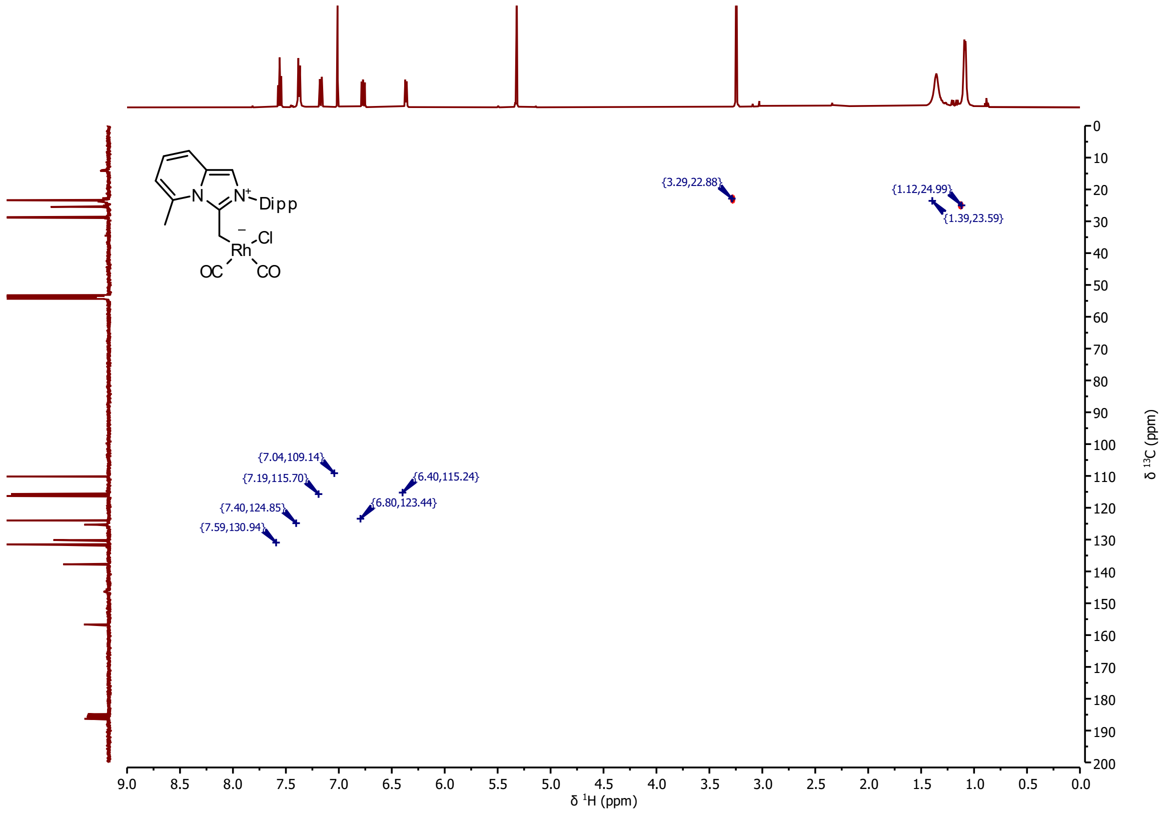


**Figure S380**: **$^1$H/$^{13}$C HSQC** (500/126 MHz, $CD_2Cl_2$, 298 K) of **3c**.

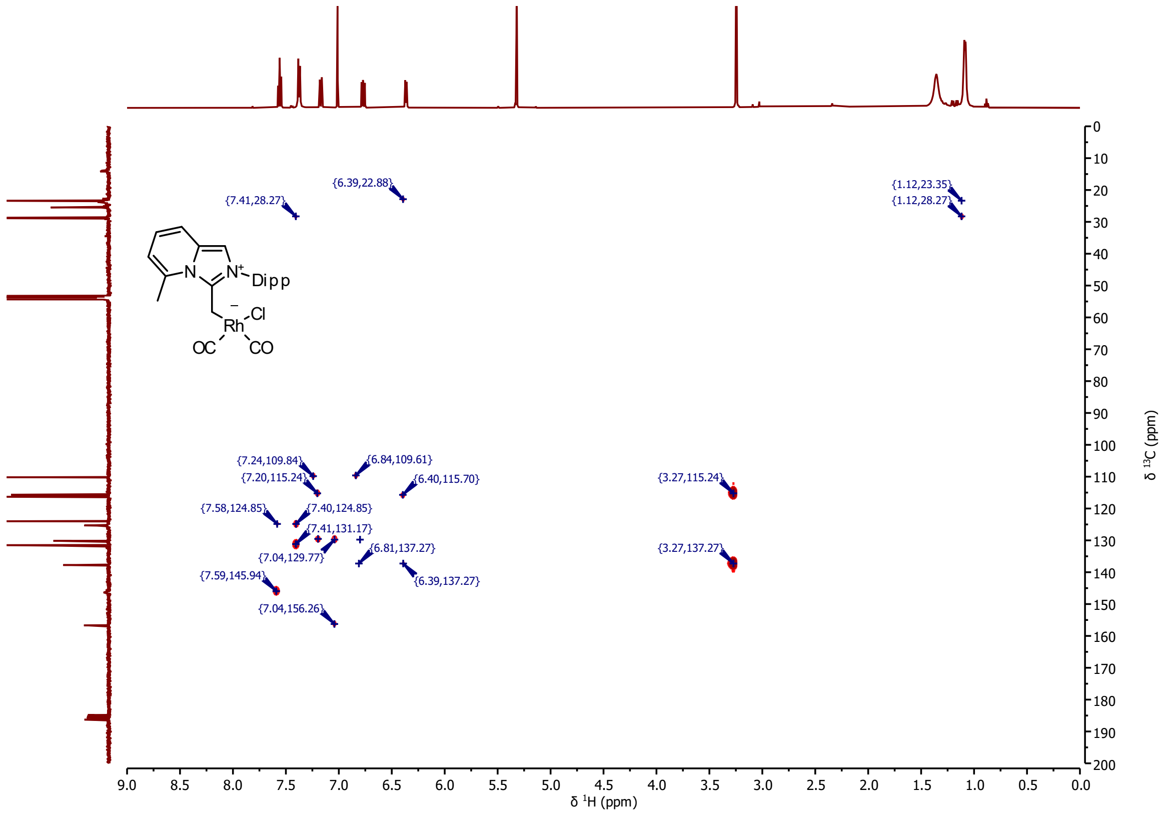


**Figure S381**: $^{1}$**H/**$^{13}$**C HMBC** (500/126 MHz, $CD_2Cl_2$, 298 K) of **3c**.

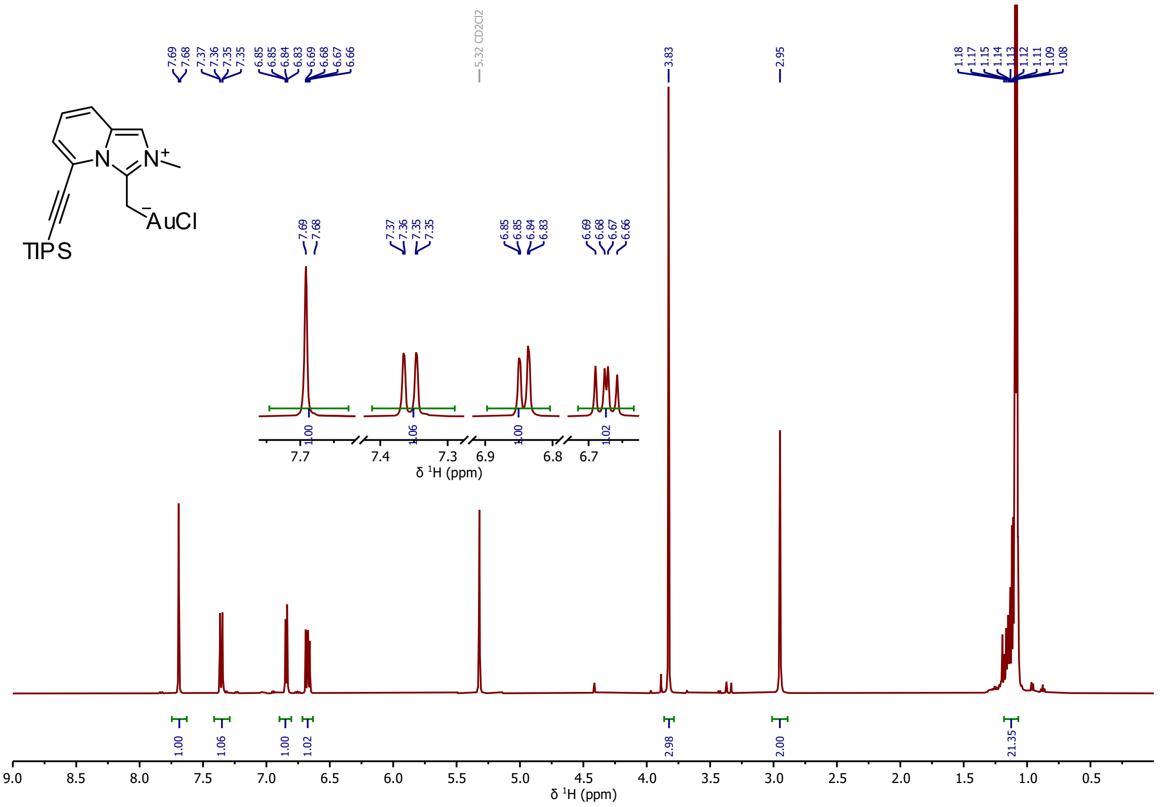


**Figure S382**: $^{1}$**H NMR** (500 MHz, $CD_2Cl_2$, 298 K) of **5b-Au**.

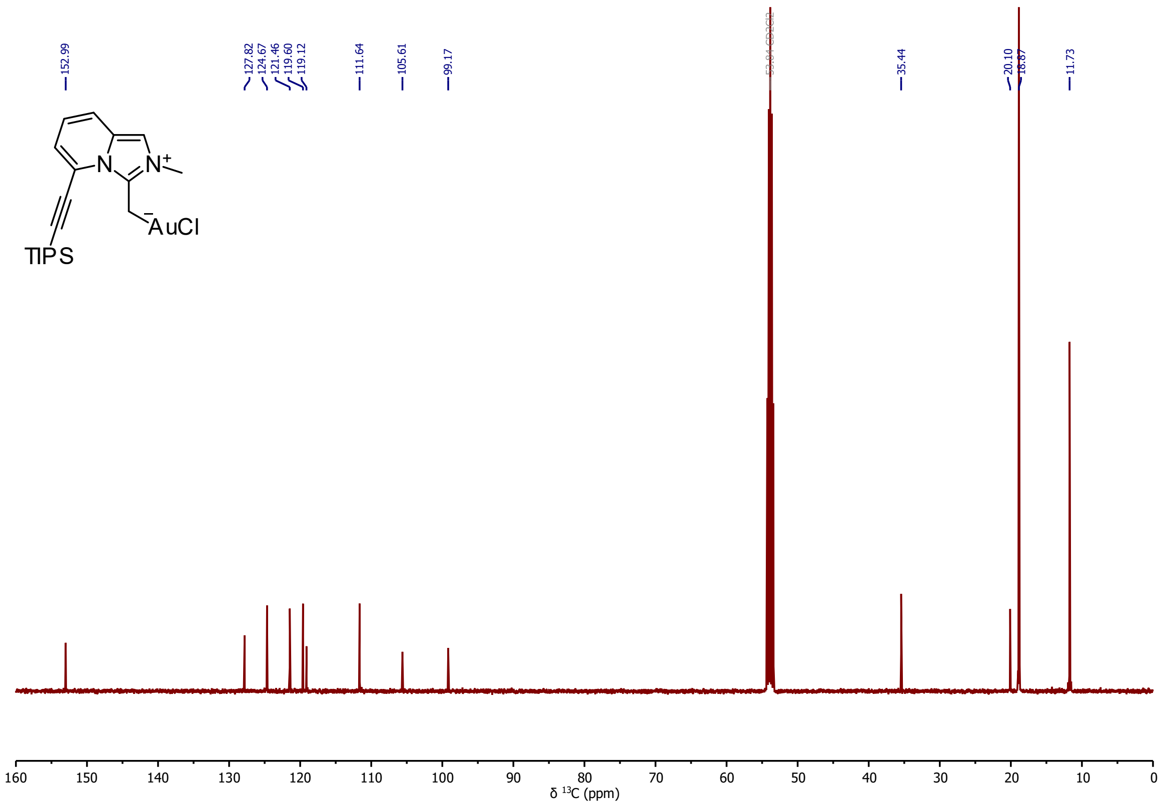


**Figure S383**: **$^{13}$C NMR** (126 MHz, $CD_2Cl_2$, 298 K) of **5b-Au**.

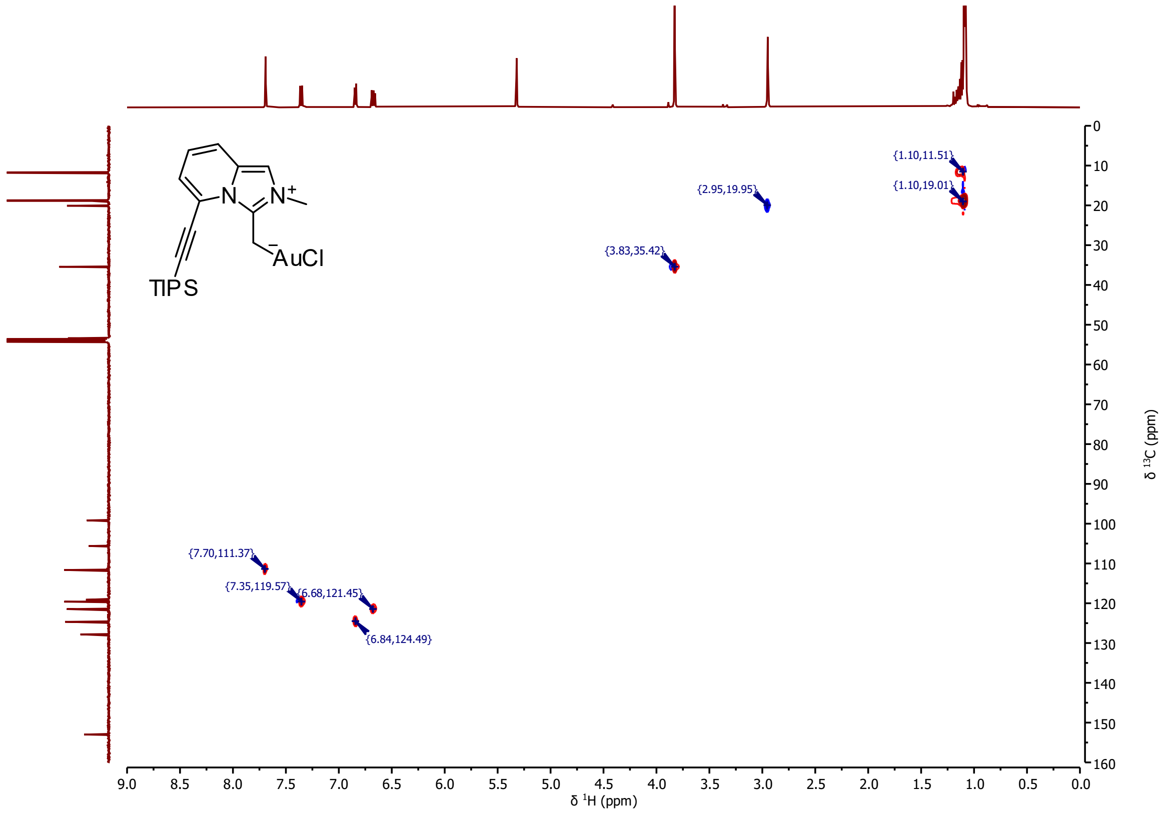


**Figure S384**: **$^{1}$H/$^{13}$C HSQC** (500/126 MHz, $CD_2Cl_2$, 298 K) of **5b-Au**.

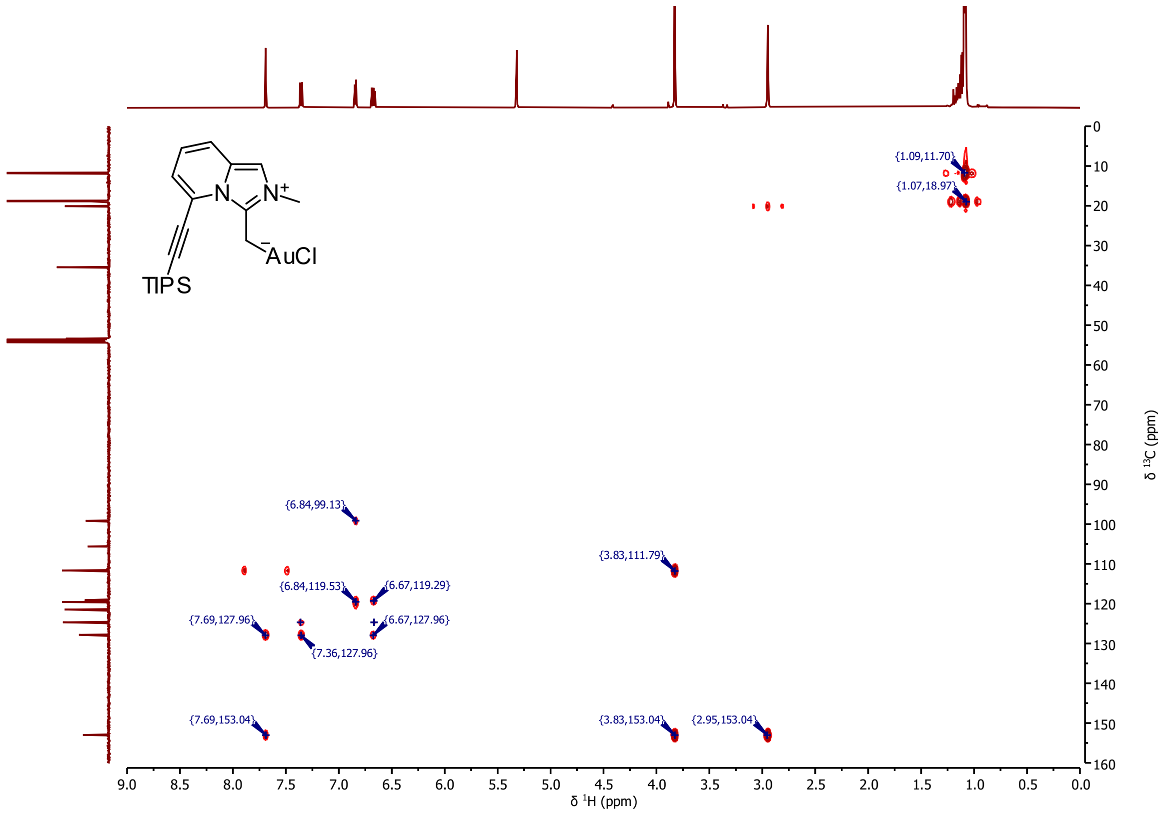


**Figure S385**: **$^{1}$H/$^{13}$C HMBC** (500/126 MHz, $CD_2Cl_2$, 298 K) of **5b-Au**.

## 3.2 Kinetic Experiments

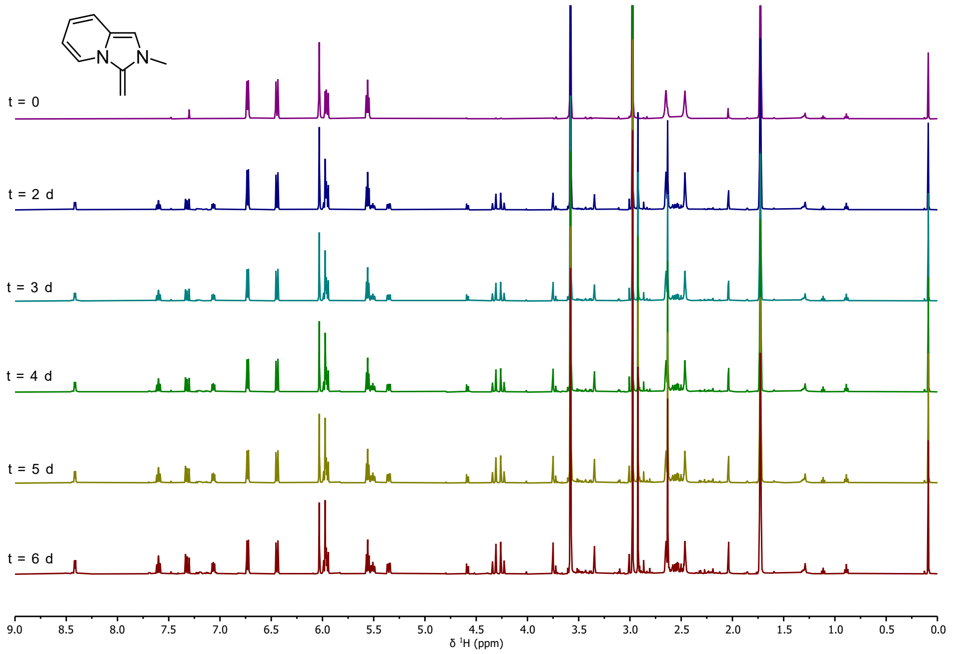


**Figure S386**: Kinetic $^{1}$H NMR (500 MHz, THF-d$_8$, 298 K) experiment over 6 days, showing signs of degradation of **2a** over time with HMDS traces present.

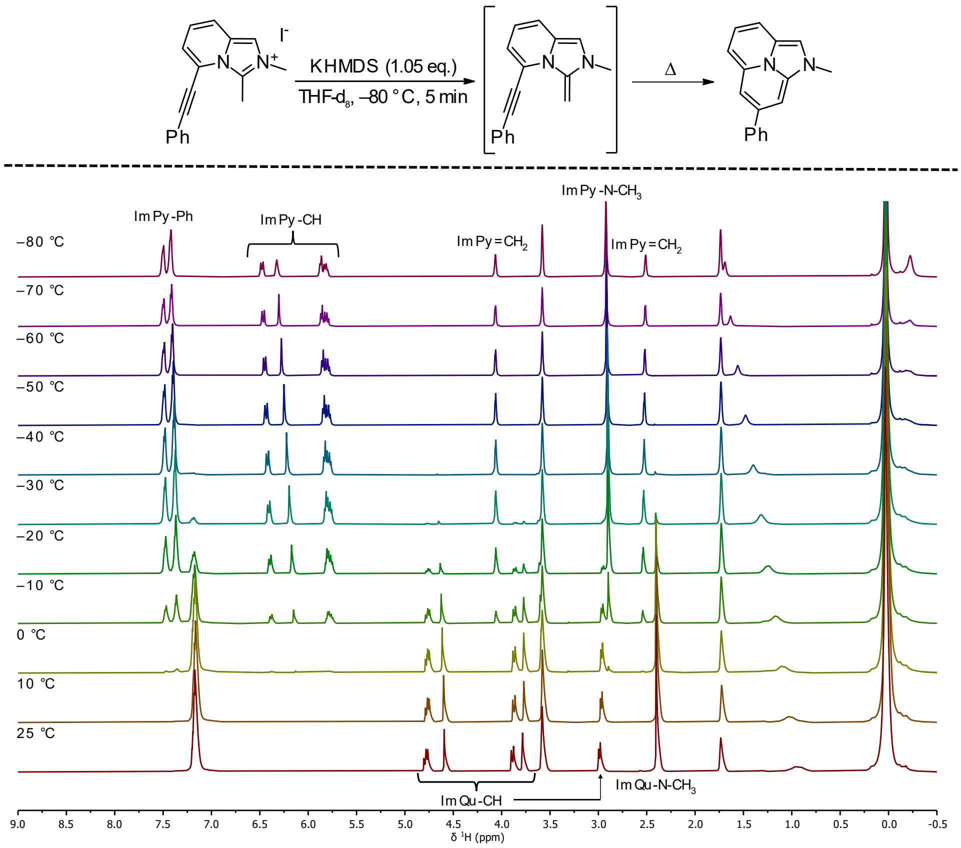


**Figure S387**: Kinetic $^{1}$H NMR (400 MHz, THF-d$_8$, 193-298 K) experiment, forming the thermally labile NHO **5a** *in situ* at low temperatures with subsequent cyclization to **6a**.

## 4. UV-Vis Spectroscopy

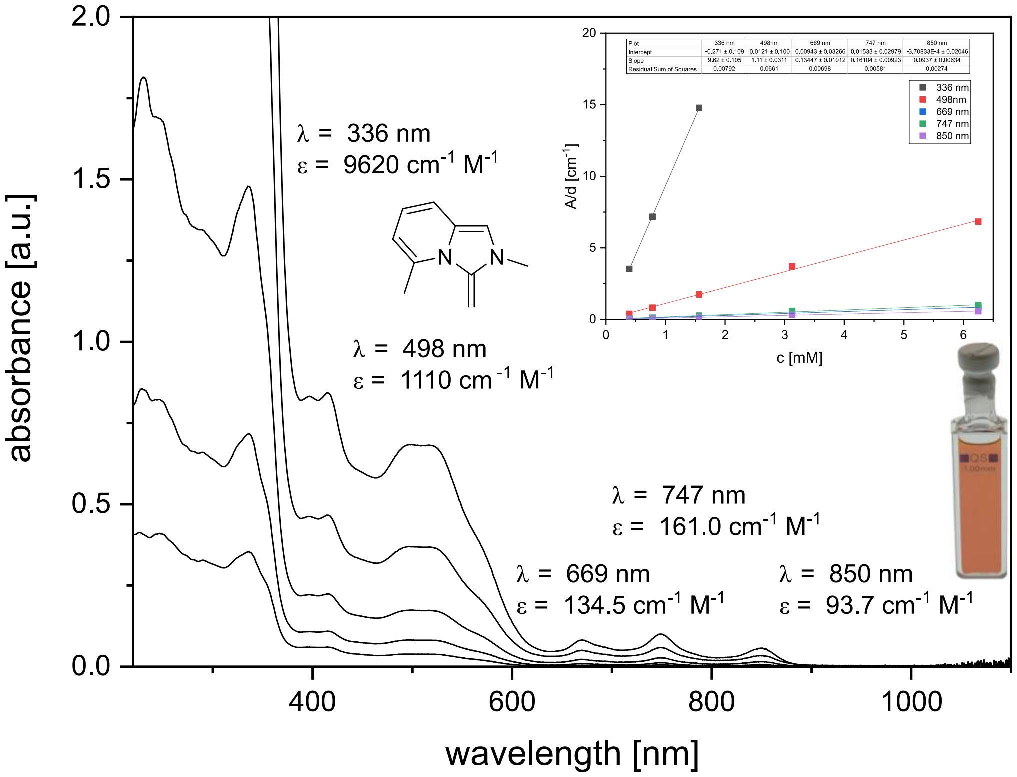


**Figure S388**: UV-Vis spectra of **2b** in THF, measured in 0.1 cm quartz cuvettes.

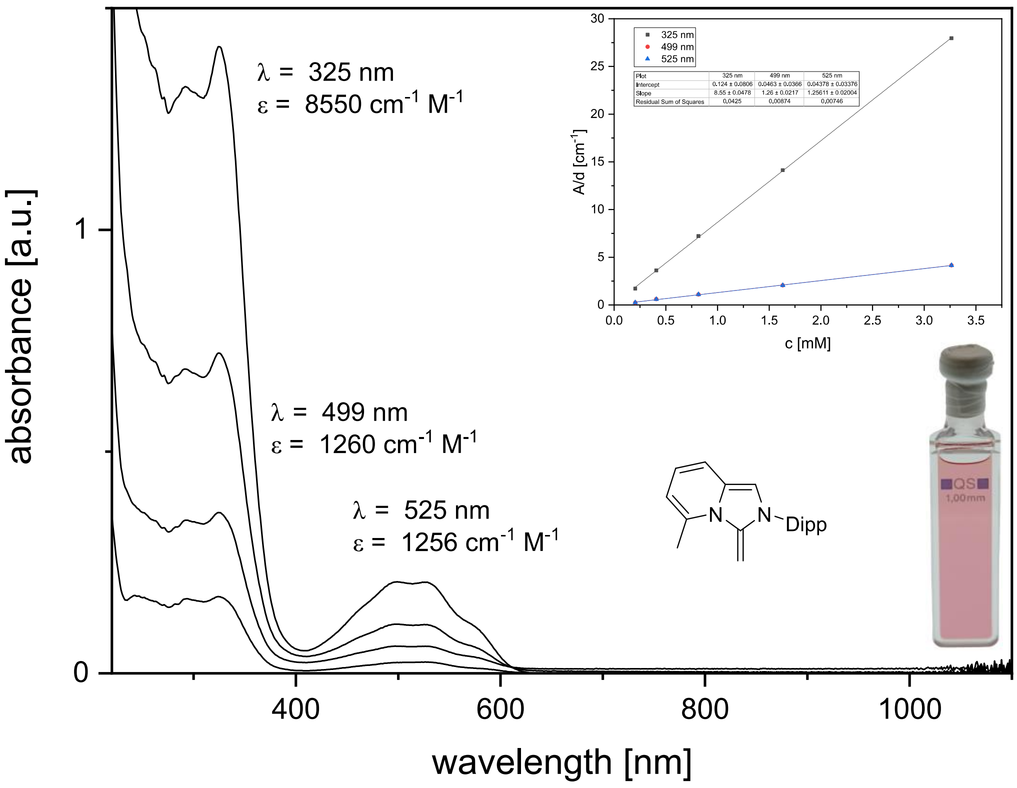


**Figure S389**: UV-Vis spectra of **2c** in THF, measured in 0.1 cm quartz cuvettes.

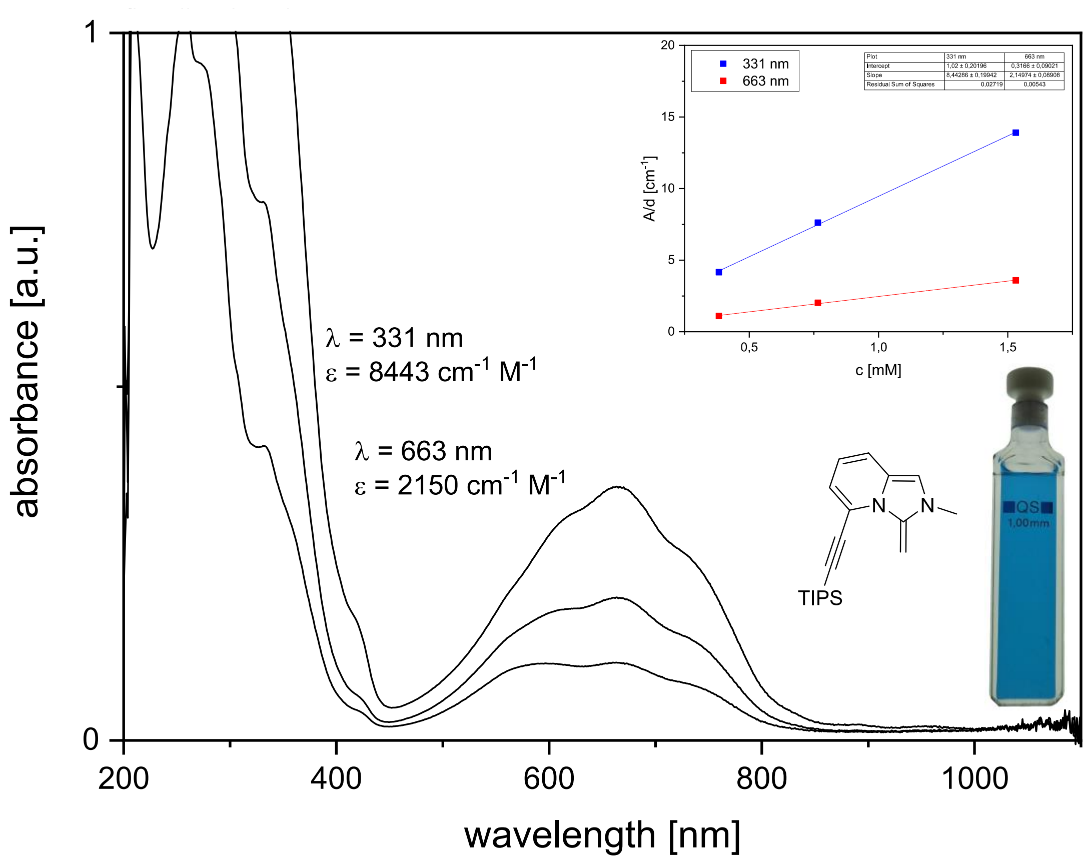


**Figure S390**: UV-Vis spectra of **5b** in THF, measured in 0.1 cm quartz cuvettes.

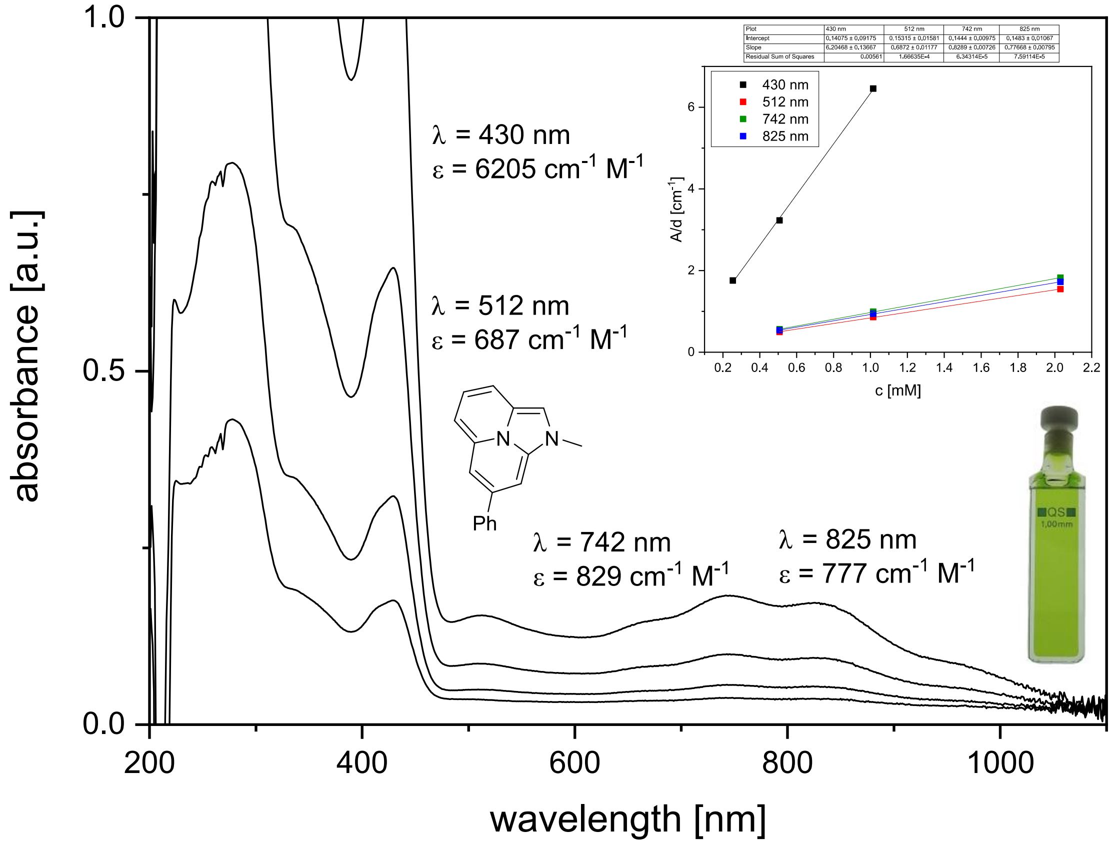


**Figure S391**: UV-Vis spectra of **6a** in THF, measured in 0.1 cm quartz cuvettes.

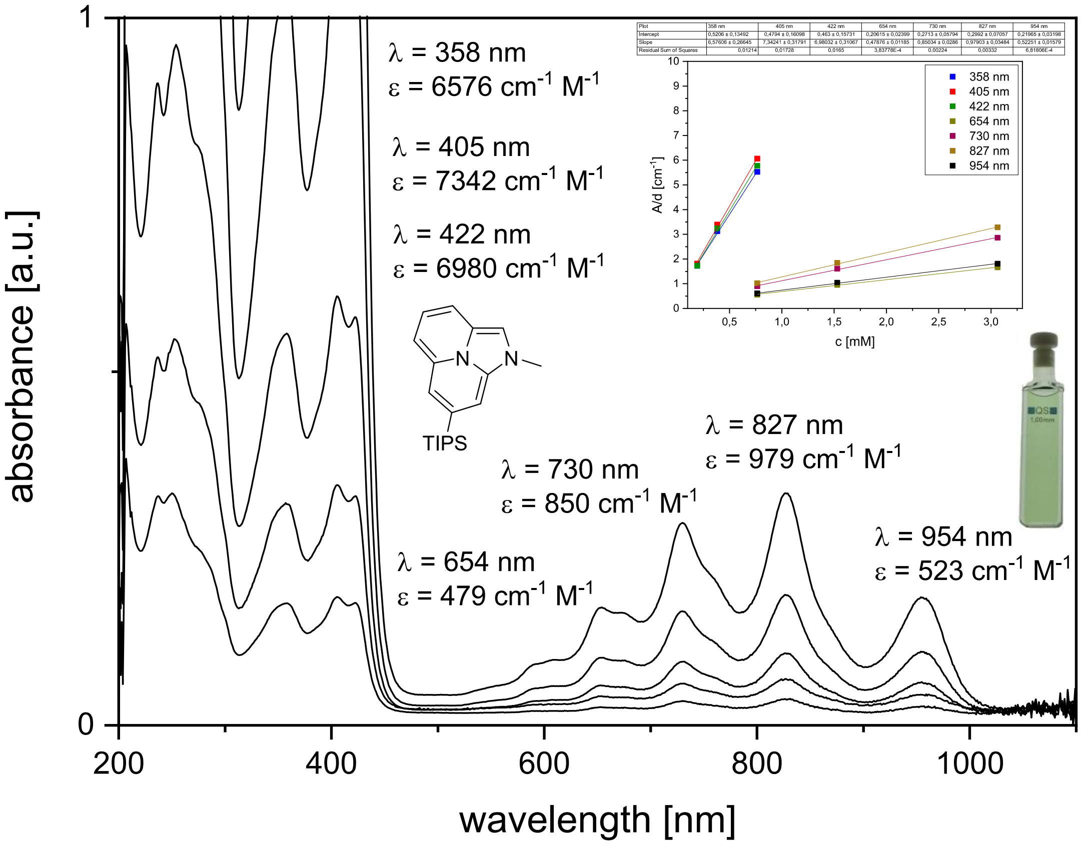


**Figure S392**: UV-Vis spectra of **6b** in THF, measured in 0.1 cm quartz cuvettes.

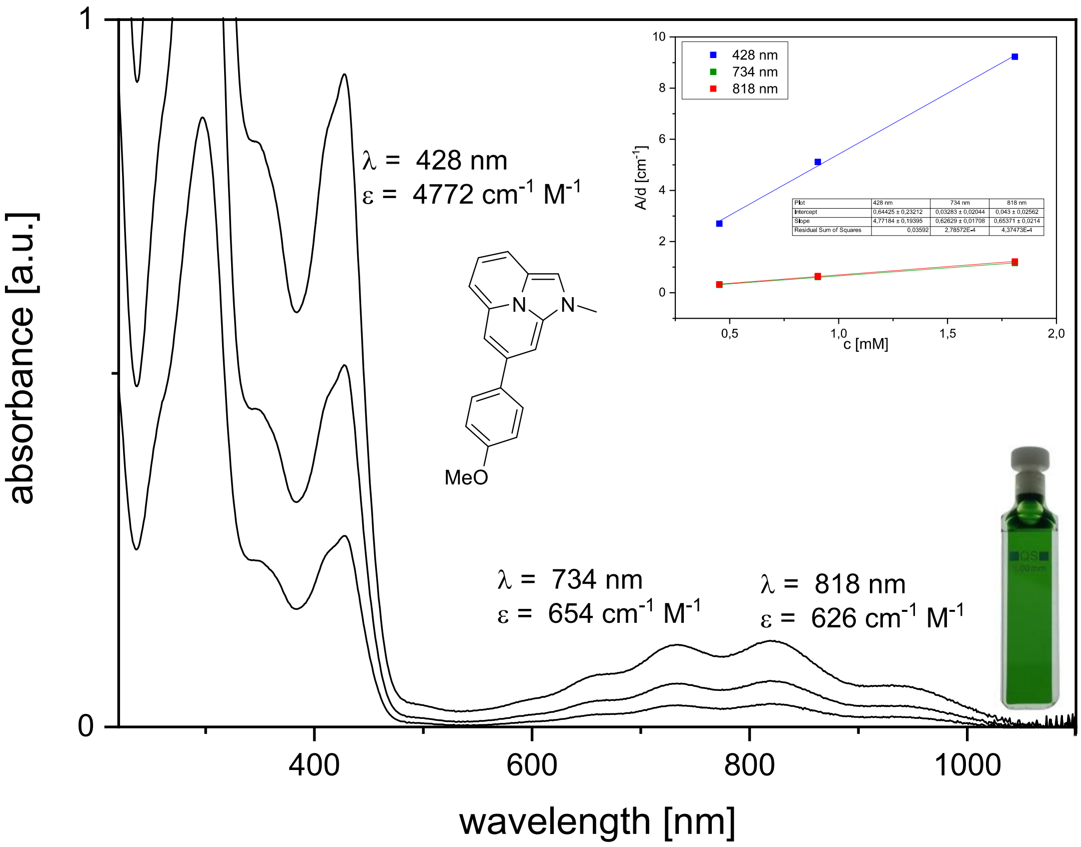


**Figure S393**: UV-Vis spectra of **6c** in THF, measured in 0.1 cm quartz cuvettes.

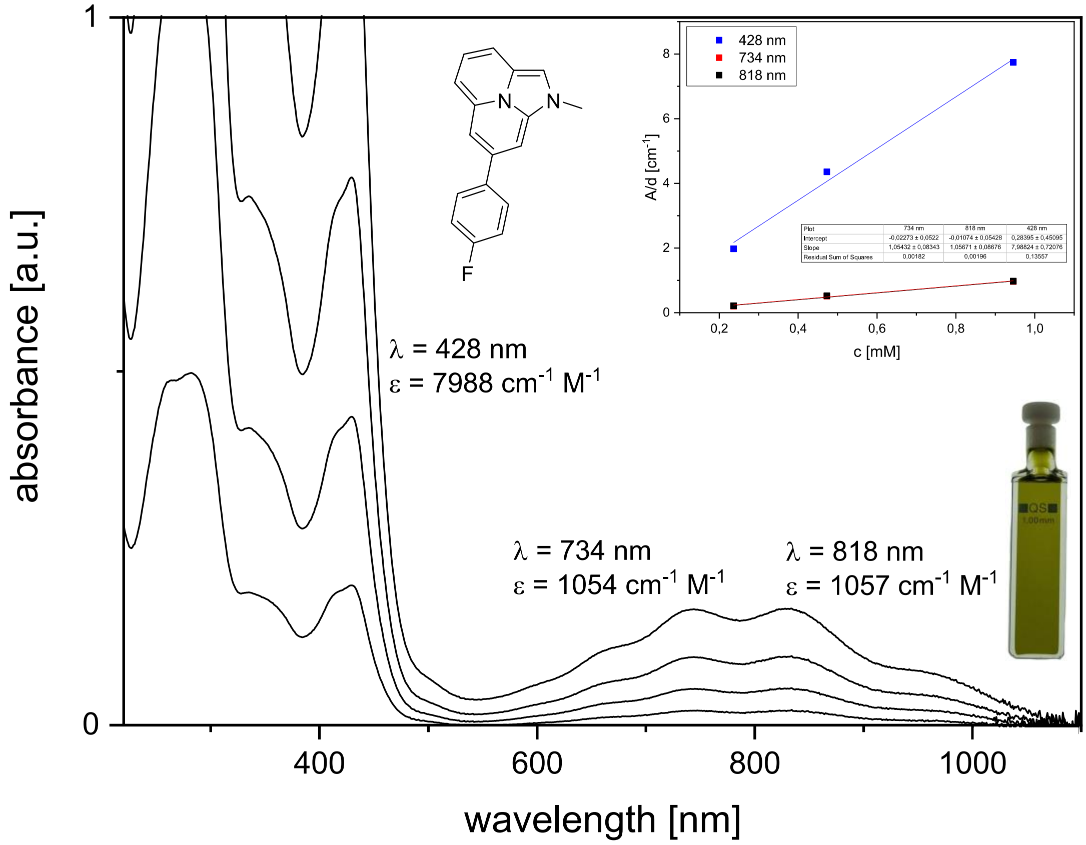


**Figure S394**: UV-Vis spectra of **6d** in THF, measured in 0.1 cm quartz cuvettes.

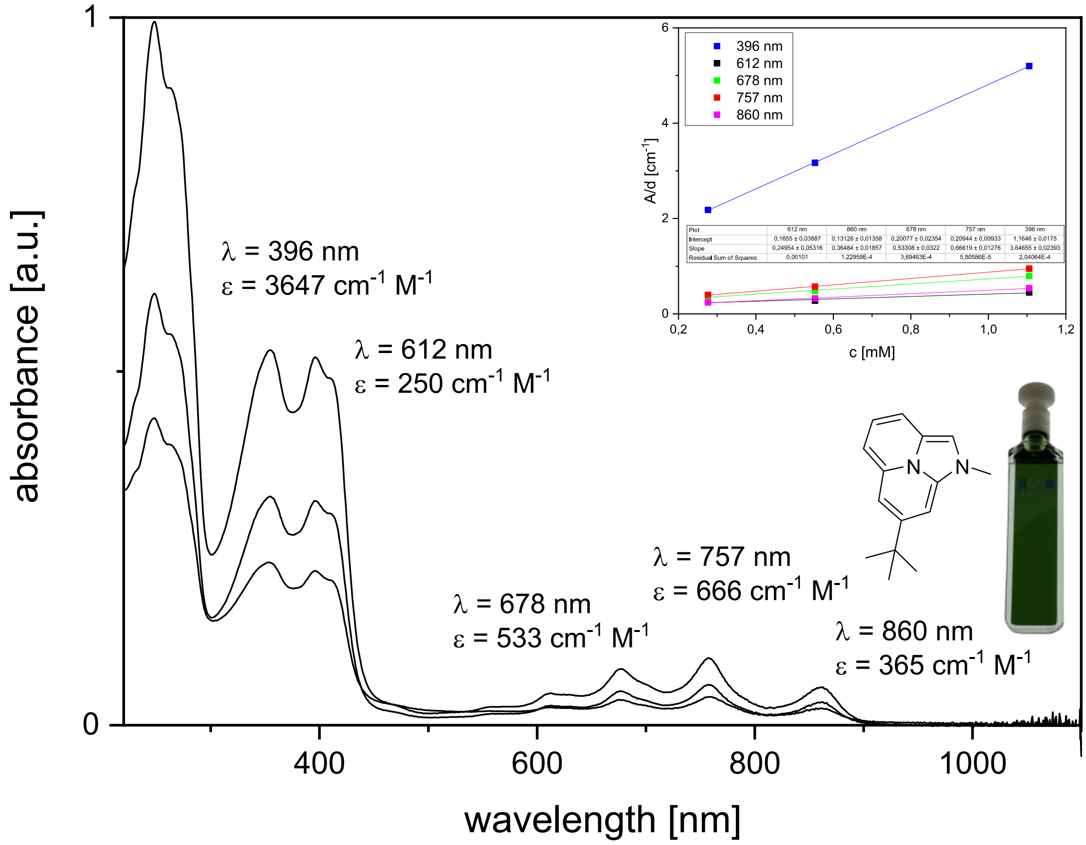


**Figure S395**: UV-Vis spectra of **6e** in THF, measured in 0.1 cm quartz cuvettes.

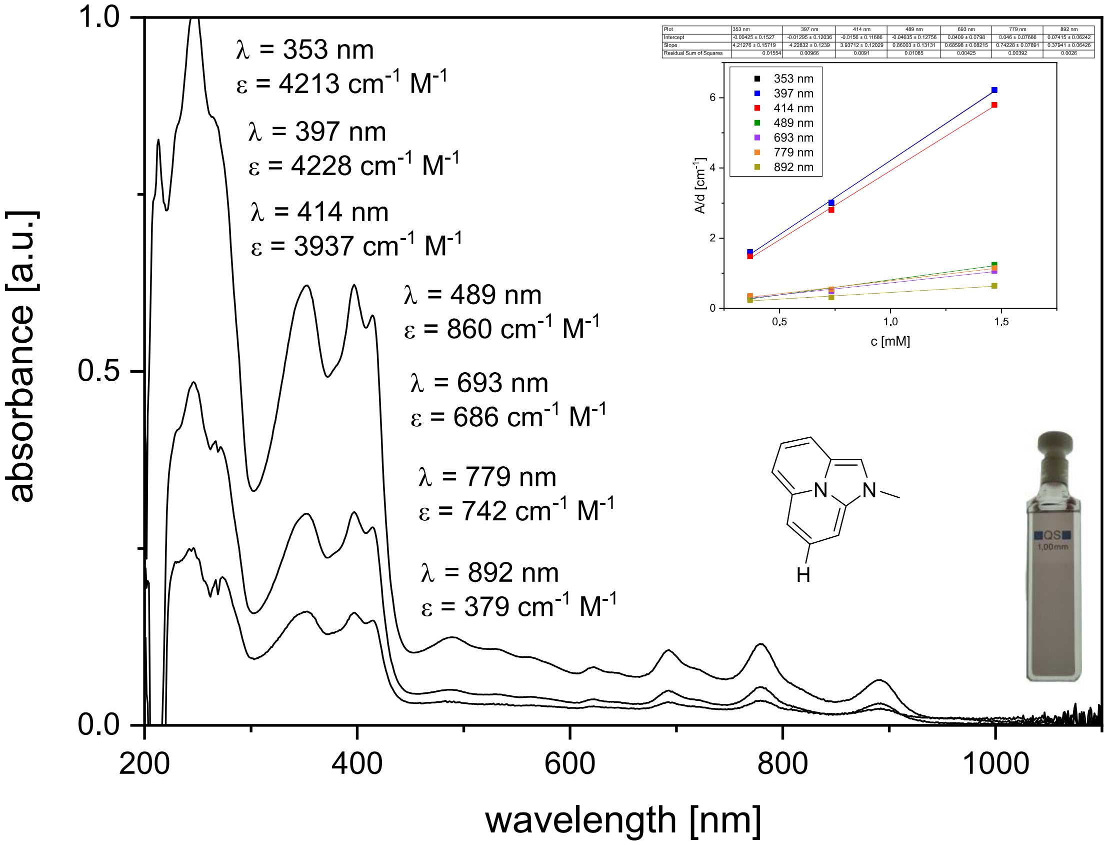


**Figure S396**: UV-Vis spectra of **6f** in THF, measured in 0.1 cm quartz cuvettes.

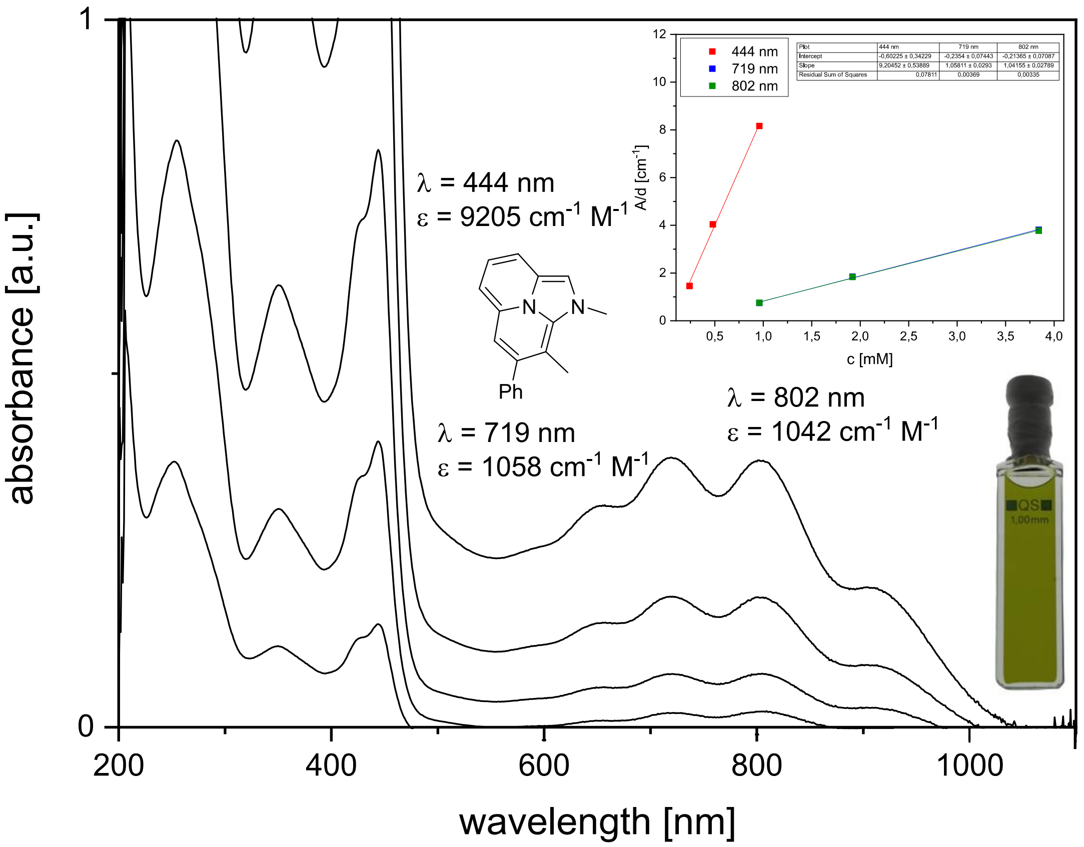


**Figure S397**: UV-Vis spectra of **6g** in THF, measured in 0.1 cm quartz cuvettes.

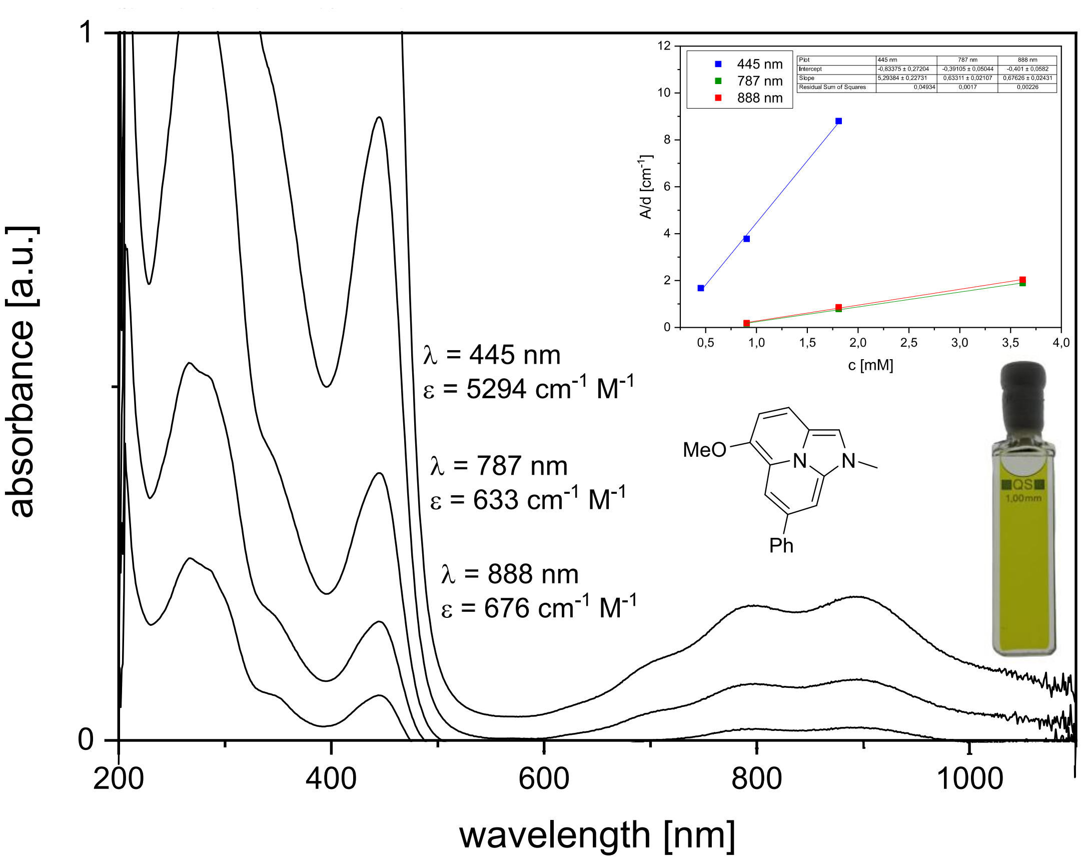


**Figure S398**: UV-Vis spectra of **6h** in THF, measured in 0.1 cm quartz cuvettes.

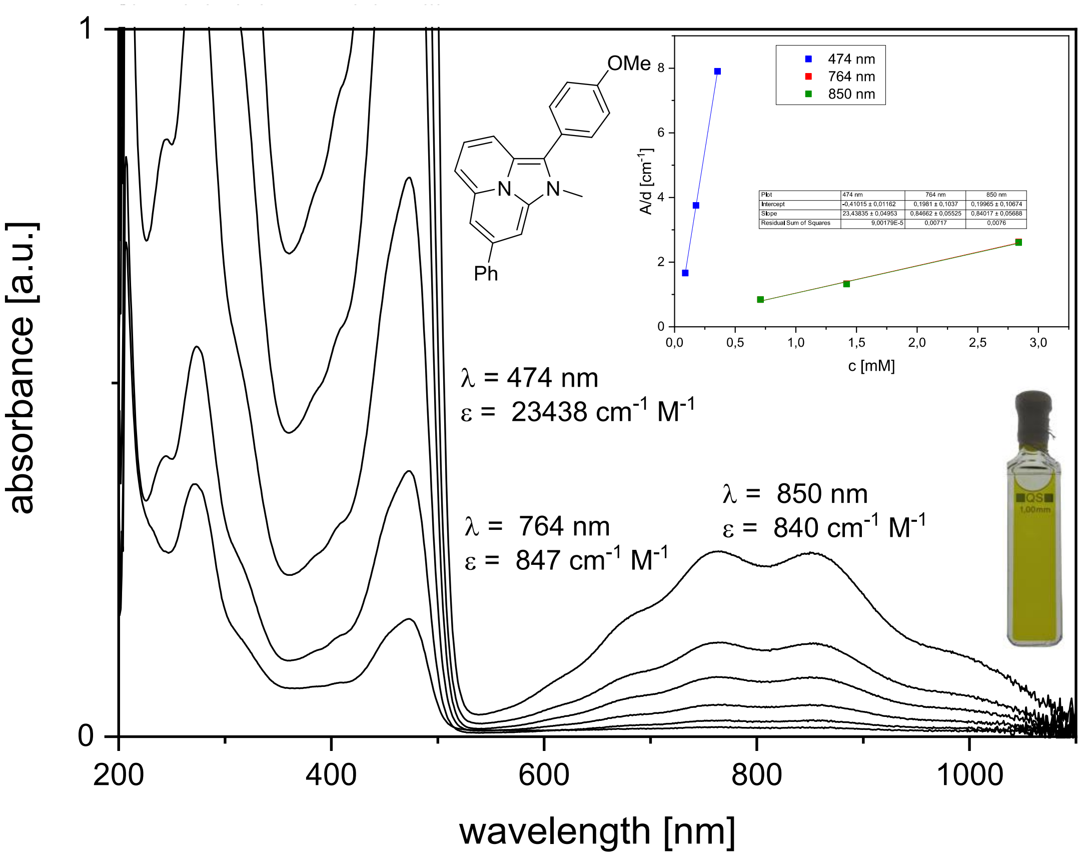


**Figure S399**: UV-Vis spectra of **6i** in THF, measured in 0.1 cm quartz cuvettes.

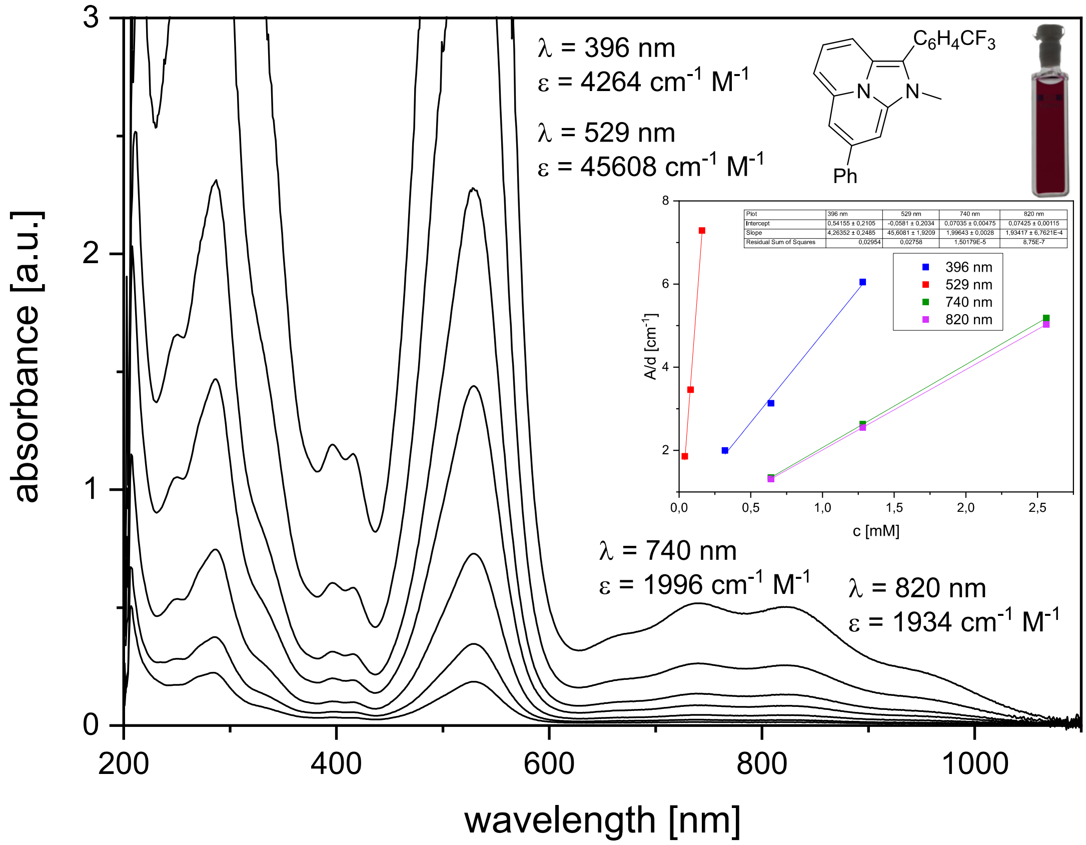


**Figure S400**: UV-Vis spectra of **6j** in THF, measured in 0.1 cm quartz cuvettes.

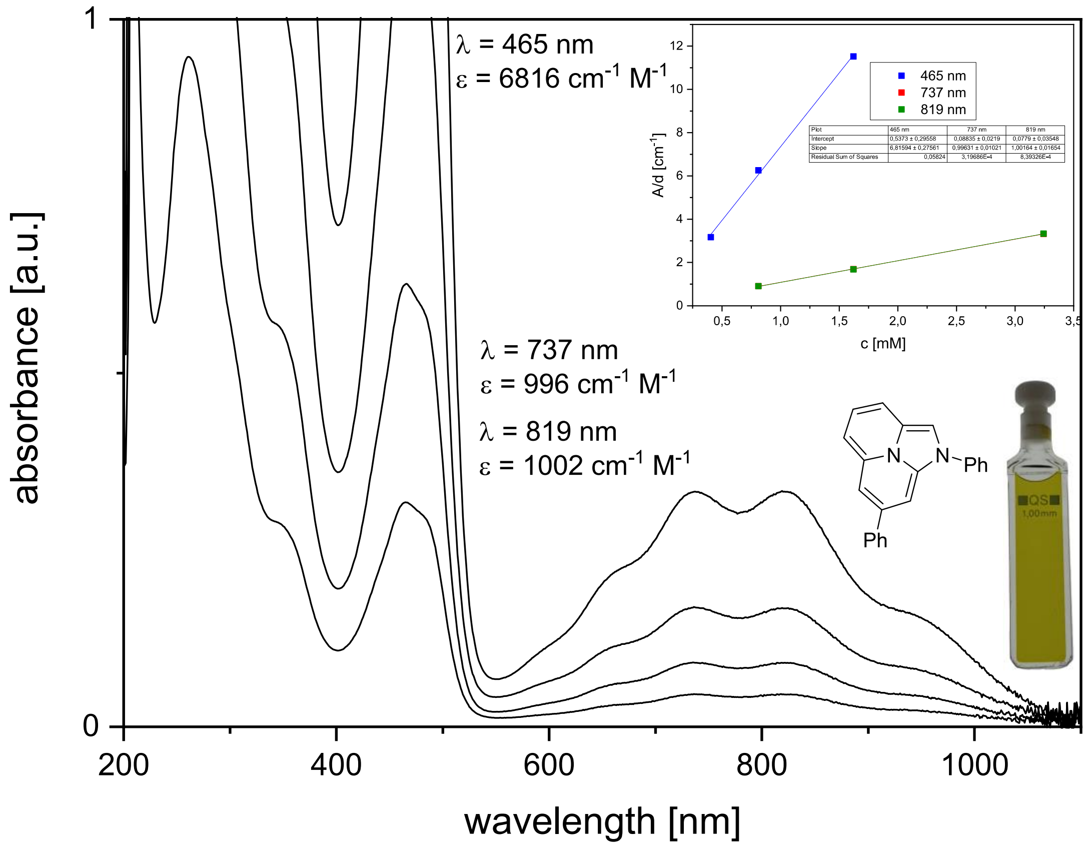


**Figure S401**: UV-Vis spectra of **6k** in THF, measured in 0.1 cm quartz cuvettes.

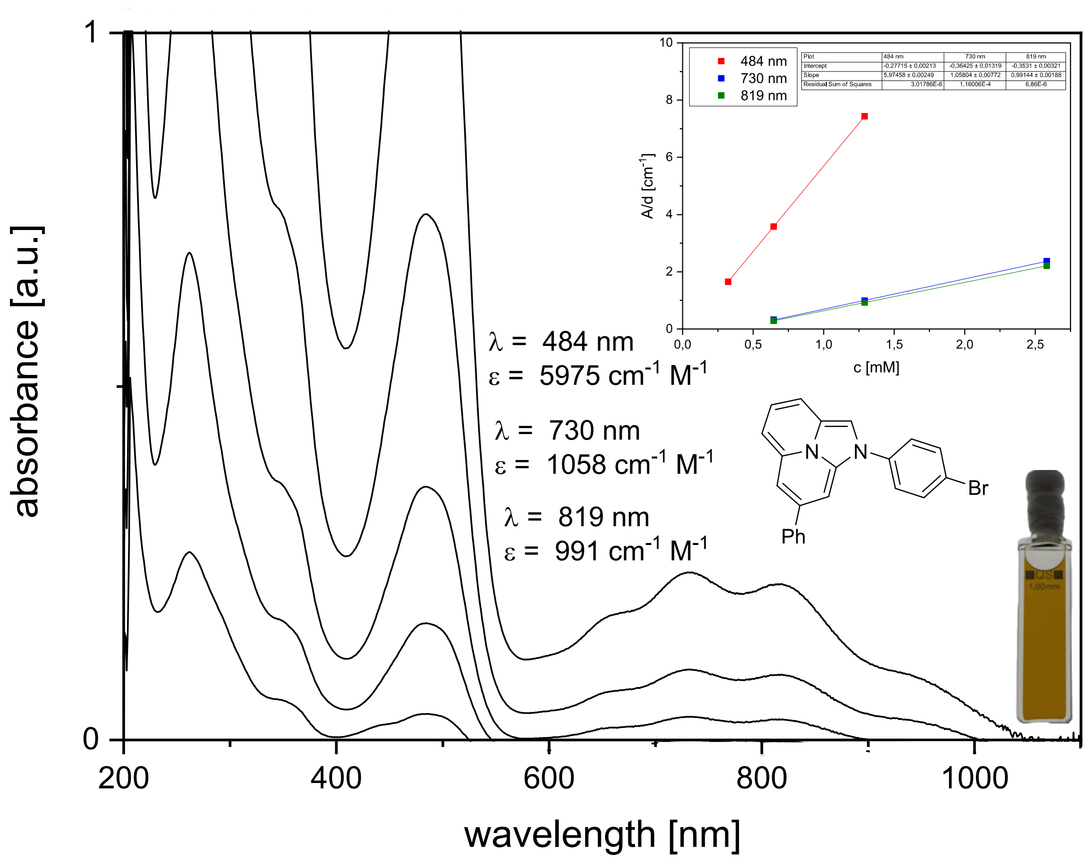


**Figure S402**: UV-Vis spectra of **6l** in THF, measured in 0.1 cm quartz cuvettes.

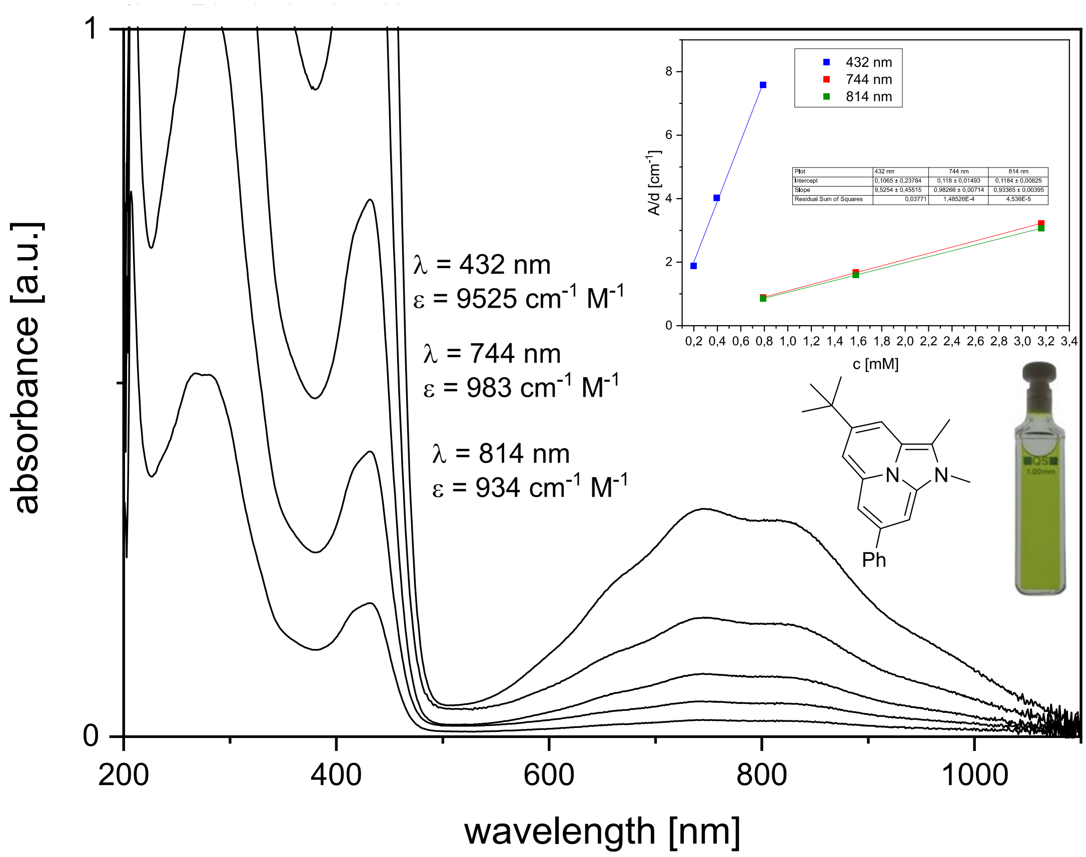


**Figure S403**: UV-Vis spectra of **6m** in THF, measured in 0.1 cm quartz cuvettes.

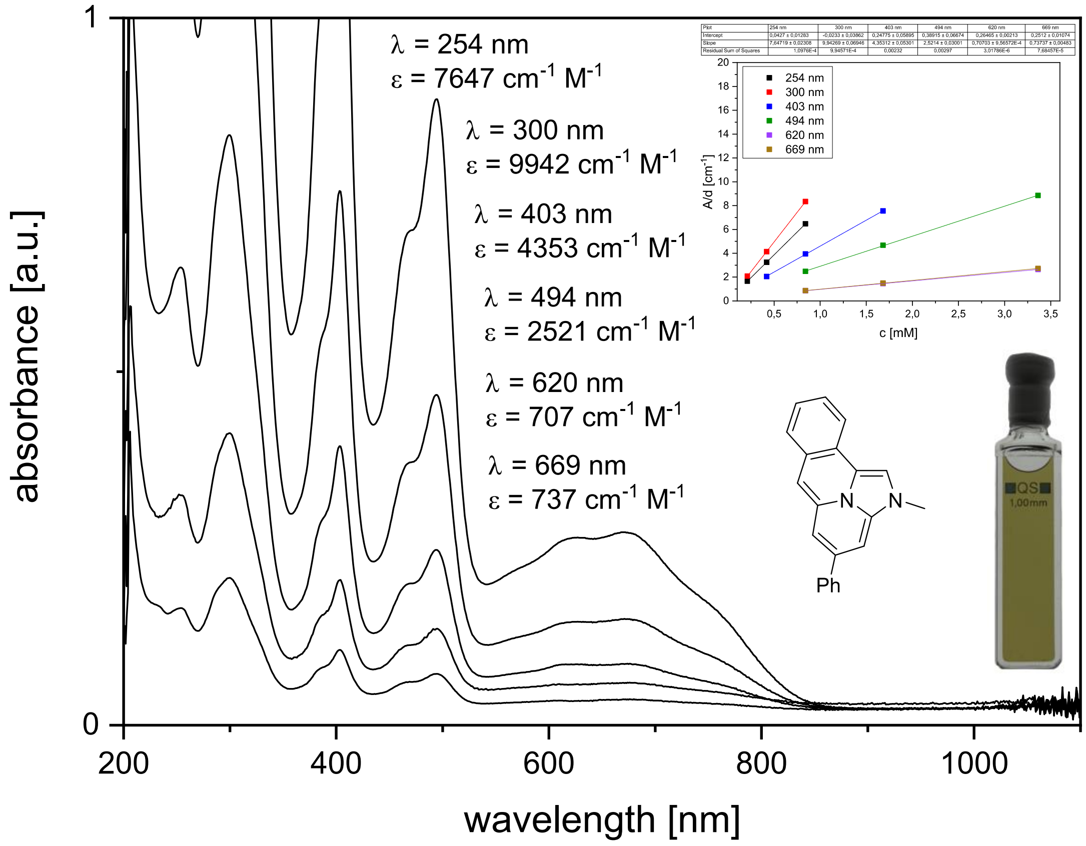


**Figure S404**: UV-Vis spectra of **6n** in THF, measured in 0.1 cm quartz cuvettes.

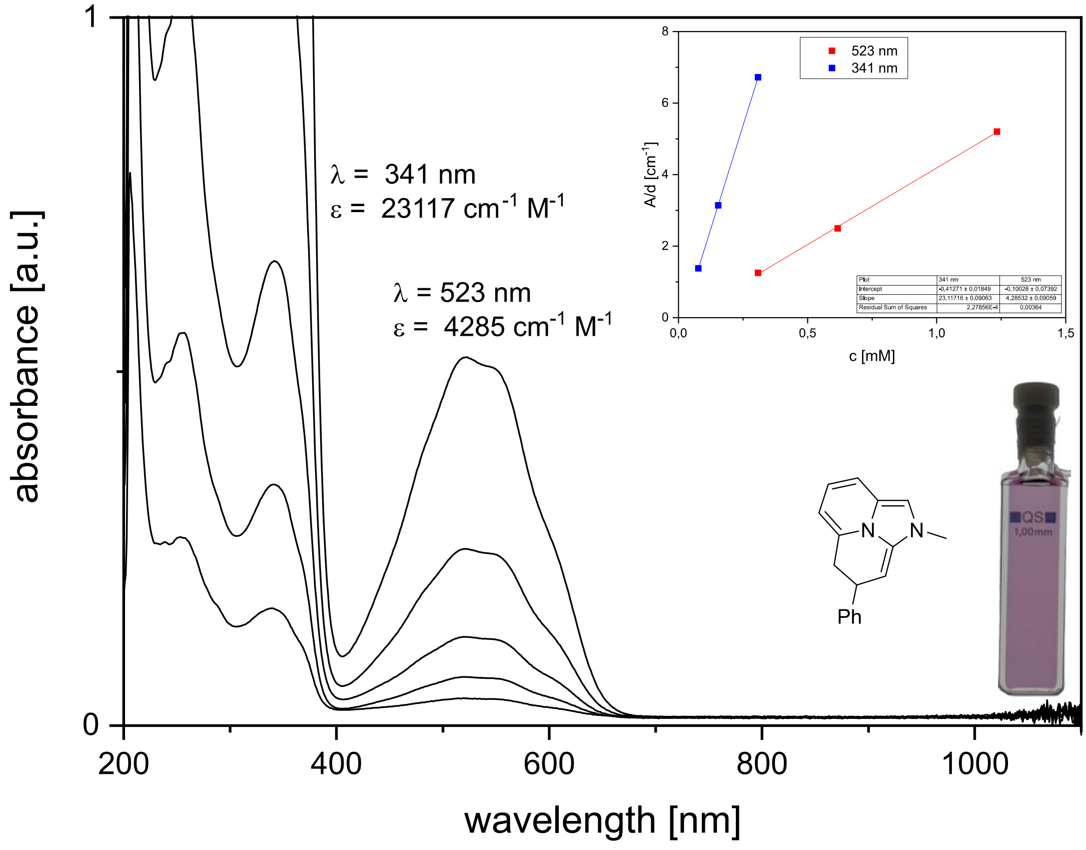


**Figure S405**: UV-Vis spectra of **6o** in THF, measured in 0.1 cm quartz cuvettes.

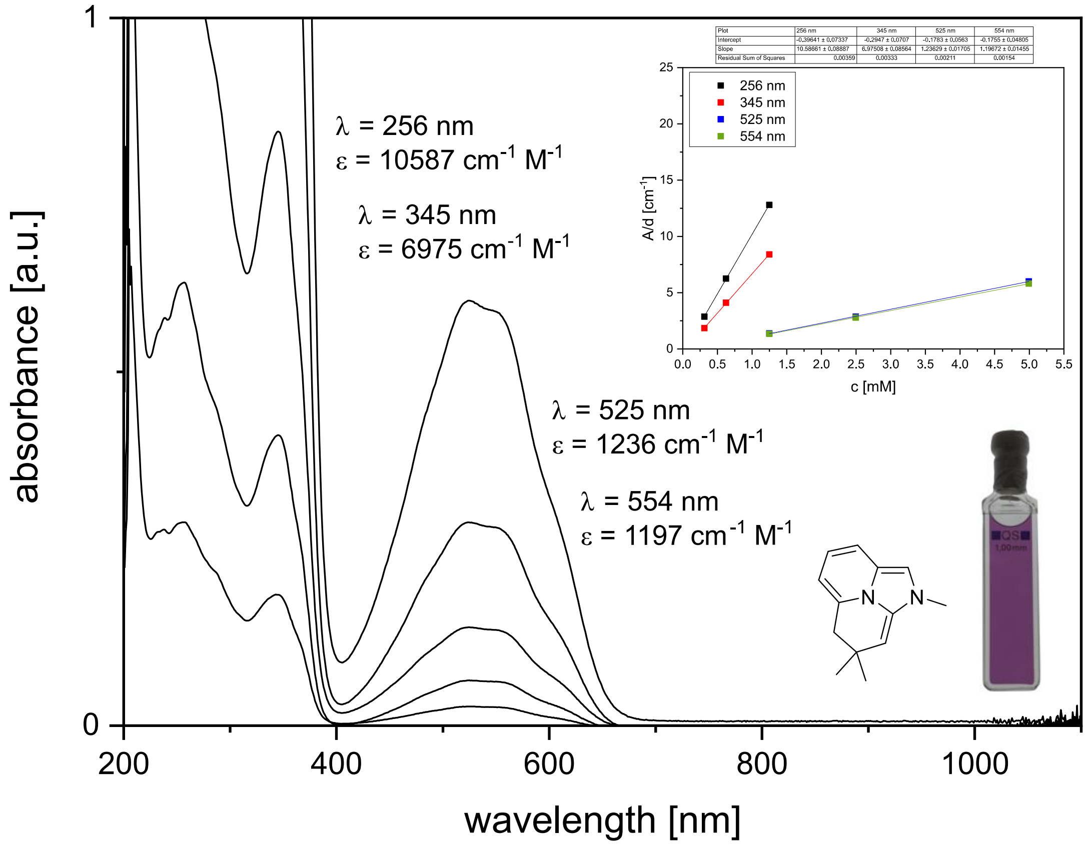


**Figure S406**: UV-Vis spectra of **6p** in THF, measured in 0.1 cm quartz cuvettes.

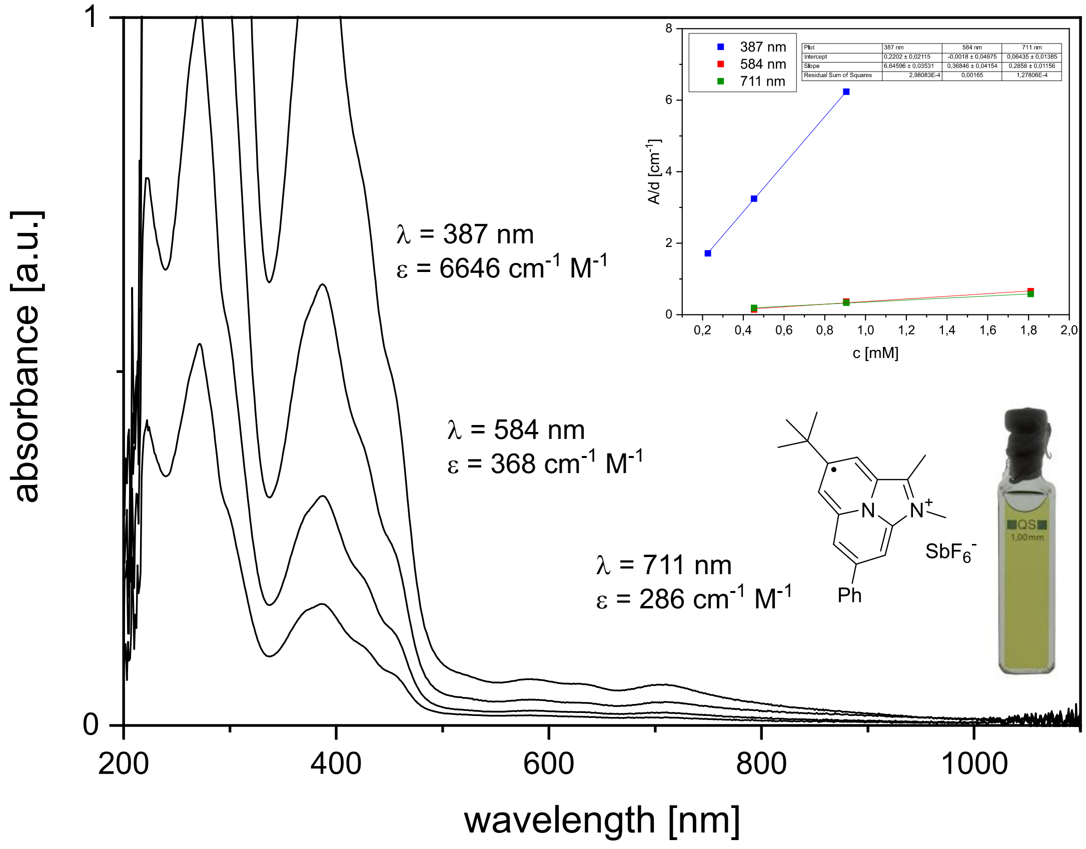


**Figure S407**: UV-Vis spectra of **6m**$^{+\cdot}$ in $CH_2Cl_2$, measured in 0.1 cm quartz cuvettes.

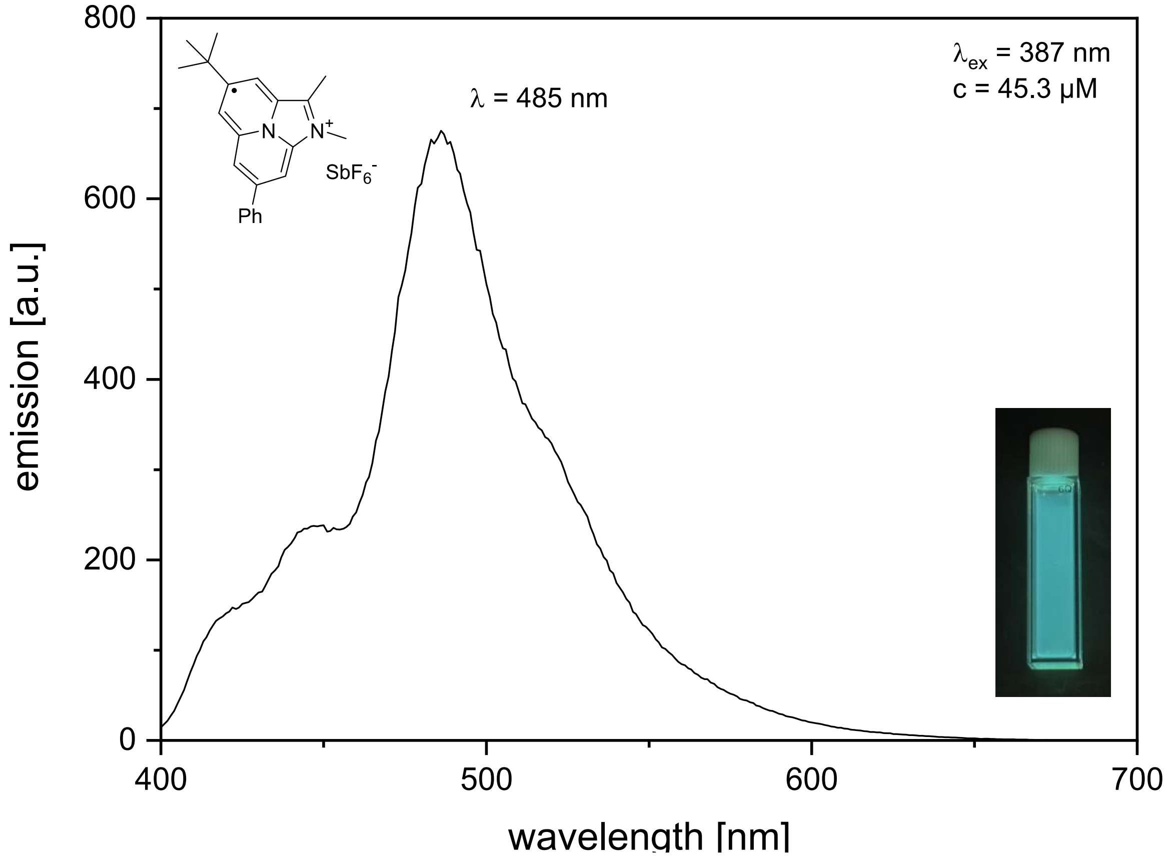


**Figure S408**: Fluorescence emission spectrum of **6m**$^{+\cdot}$ in $CH_2Cl_2$, measured in 1 cm quartz cuvettes.

## 5. EPR Spectroscopy

X-Band EPR experiments were performed using a magnettech MiniScope MS400 Benchtop spectrometer. To account for magnetic field drift the measurement was referenced to TEMPO in $CH_3CN$ (0.2 mM; g = 2.0055). TEMPO was measured after the investigated sample and its spectrum was then simulated. The determined difference to the literature value (for *B* and *g*) was then added/subtracted from the determined value of the investigated sample. Simulations were performed with the EasySpin program.[9] All DFT[10][11] calculations were performed with Gaussian16 program package[12] (version g16, rev.C01). All calculations were performed with the B3LYP functionals employing Ahlrich's def2-SVP or def2-TZVP(P) basis sets.[13][14] Ground states were fully optimized without constraints at the corresponding level of theory and checked by a frequency calculation. Grimme's D3 dispersion correction with Becke-Johnson damping was used in order to take dispersion effects into account.[15][16] Isotopic hyperfine constants and Mulliken spin densities were calculated at the M06-2X/cc-pVDZ level of theory. For the visualization of frontier molecular orbitals IboView[17] and GaussView 6.1 were used.[18]

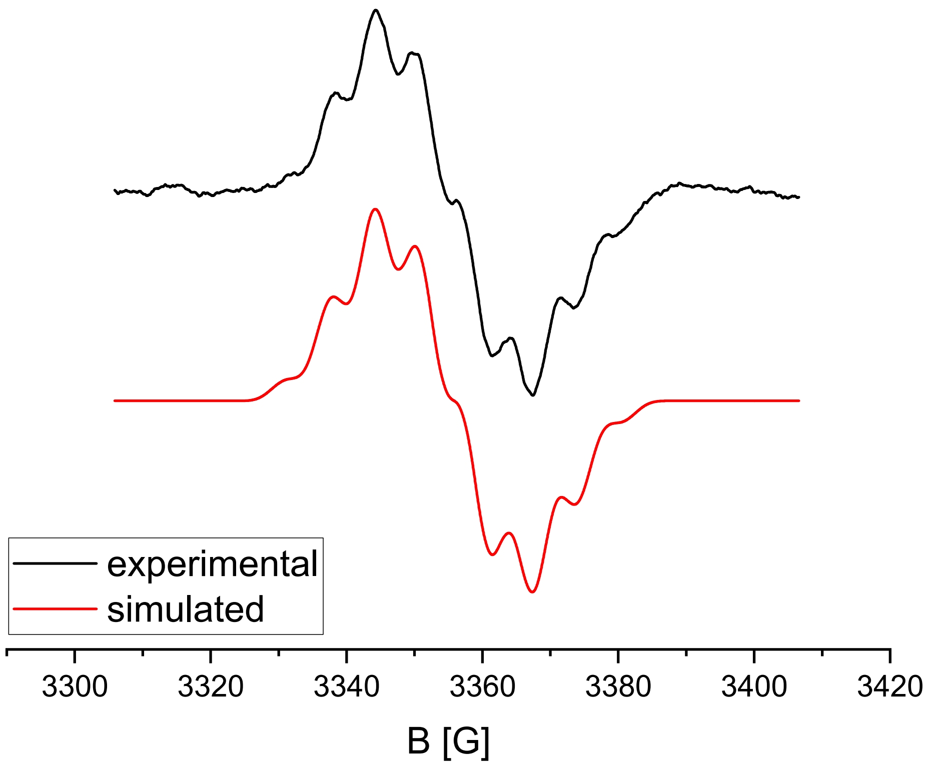


**Figure S409:** X-Band EPR spectrum of **6m**$^{+\cdot}$ in THF (1 mM). Fitting parameter: g = 2.0024; LW 0.2842; Hyperfine coupling: 1xN: 2.3921 MHz; 1xN: 4.2379 MHz; 1xH: 16.7178 MHz; 1xH: 11.0426 MHz; 1xH: 18.6327 MHz; 1xH: 23.9323 MHz; 3xH: 17.9542 MHz; 3xH: 4.7849 MHz.

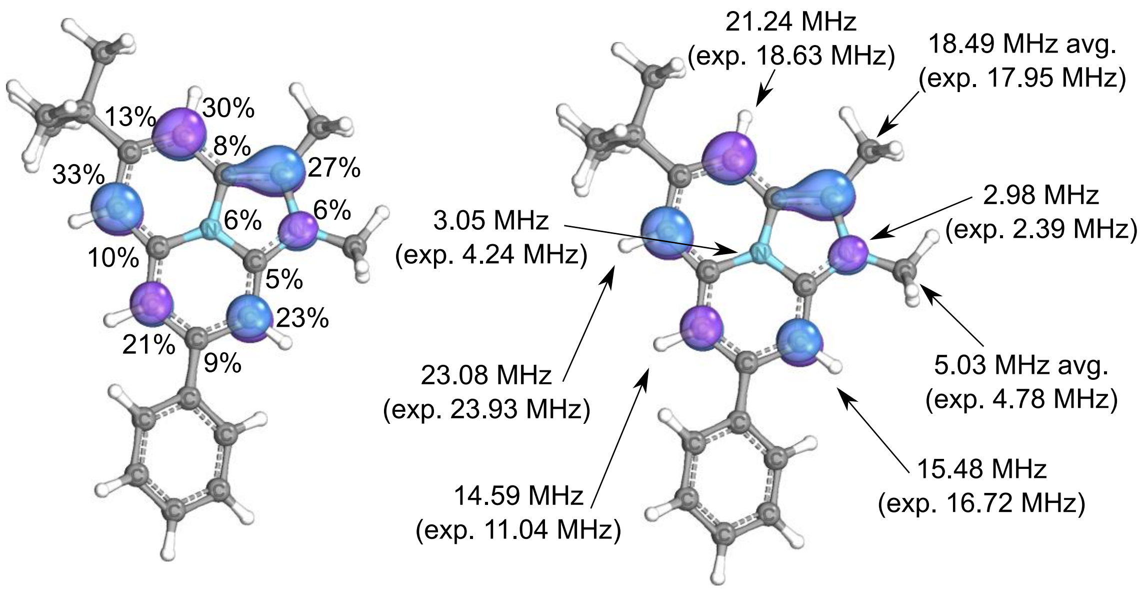


**Figure S410:** Left: SOMO of **6m**$^{+\cdot}$ with the significant Mulliken spin-densities (in %). Isovalue: 0.5; Right: SOMO of **6m**$^{+\cdot}$ with the calculated hfcs. Isovalue: 0.5.

## 6. Cyclic Voltammetry

CVs were measured with a Gamry Instruments Reference 600+ and integrated iR compensation using the positive feedback method. Samples were measured under inert atmosphere in a nitrogen glove box at rt in dry solution of the respective solvent containing tetrabutylammonium hexafluorophosphate (0.1 M). Electrochemical grade tetrabutyl-ammonium hexafluorophosphate was molten under vacuum prior to use. The setup consisted of a three-neck flask with a three-electrode setup containing a glassy carbon working electrode (GC: CH Instruments, ALS Japan; A = 7.1 $mm^2$), a platinum wire as a counter electrode, and an Ag/$AgNO_3$ reference electrode (0.01 M $AgNO_3$ in 0.1 M $nBu_4NPF_6$ in $CH_3CN$). The reference electrode was freshly prepared by using a fritted sample holder (Vycor glass), which was activated by storing it in a $CH_3CN$ solution for one night, followed by diluted $HNO_3$ (1M) for one night, followed by demineralized water for one night, followed by $CH_3CN$ for one night dried and stored in the solvent used for the CV measurement for at least one additional night. To the fritted sample holder was added a freshly prepared 0.01 M $AgNO_3$/0.1 M $nBu_4NPF_6$ solution in $CH_3CN$ and a silver wire. The working electrode was cleaned before measuring a new compound by standard methods: washed with water, polished with an Alox-slurry (0.05 µm), washed with millipore water, sonicated in HPLC grade EtOH for 3 minutes, rinsed with millipore water and HPLC grade EtOH, and dried. The 3-neck cell was filled with a specific amount of compound dissolved in 3 mL the stated solvent and then the CV was measured. The system was furthermore (doubly) referenced internally by addition of ferrocene or diacetylferrocene.

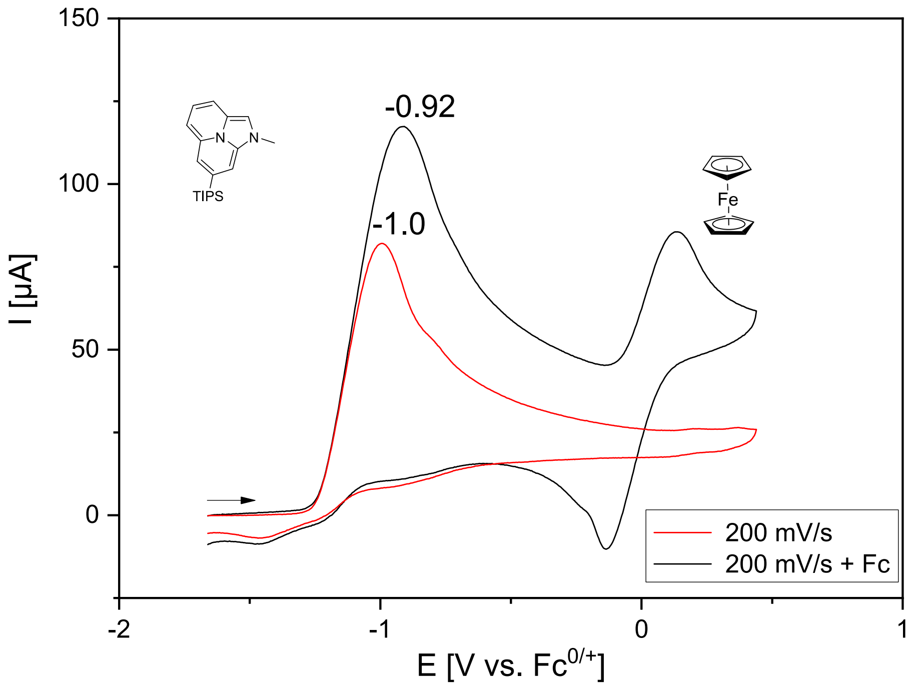


**Figure S411:** Cyclic voltammograms of **6b** (1.7±0.1 mg mL$^{-1}$) in THF (0.1 M n-$Bu_4NPF_6$) at rt; scan rate 200 mV s$^{-1}$ (iR compensation = 2200 Ohm) referenced internally against ferrocene (arrows indicate scanning direction).

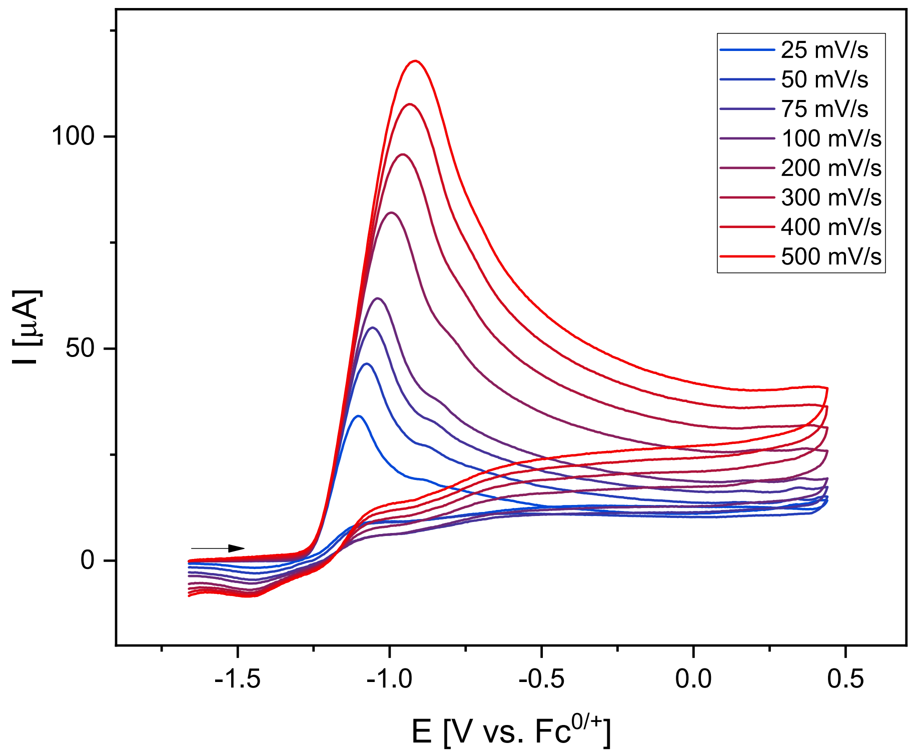


**Figure S412:** Cyclic voltammograms of **6b** (1.7±0.1 mg mL$^{-1}$) in THF (0.1 M n-$Bu_4NPF_6$) at rt; different scan rates (iR compensation = 2200 Ohm referenced internally against ferrocene (arrows indicate scanning direction).

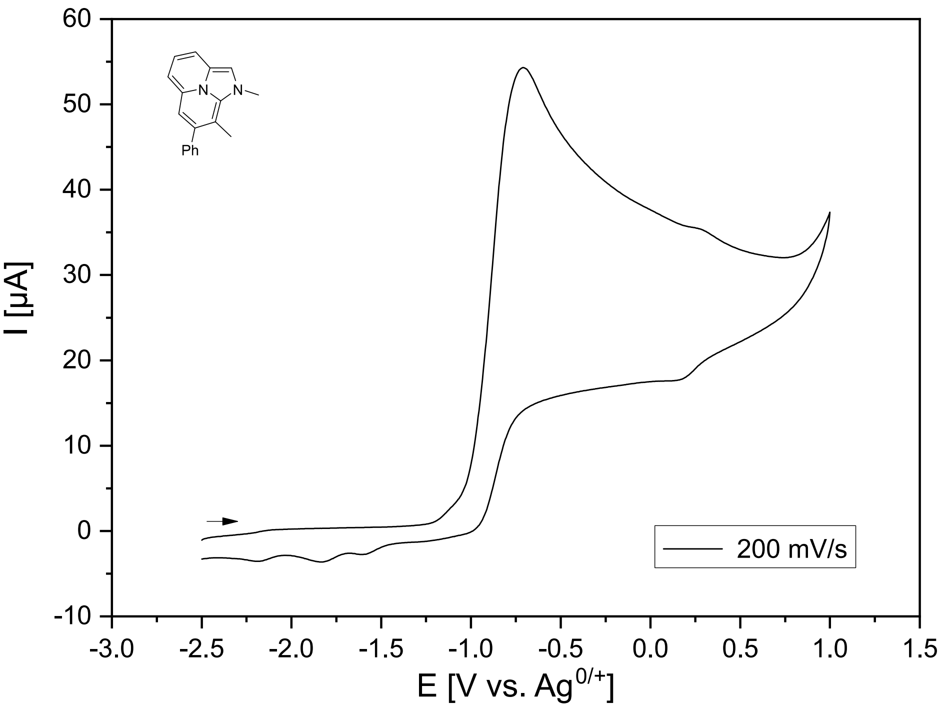


**Figure S413:** Cyclic voltammograms of **6g** (3.0±0.1 mg mL$^{-1}$) in THF (0.1 M n-$Bu_4NPF_6$) at rt; scan rate 200 mV s$^{-1}$ (iR compensation = 2110 Ohm) (arrows indicate scanning direction).

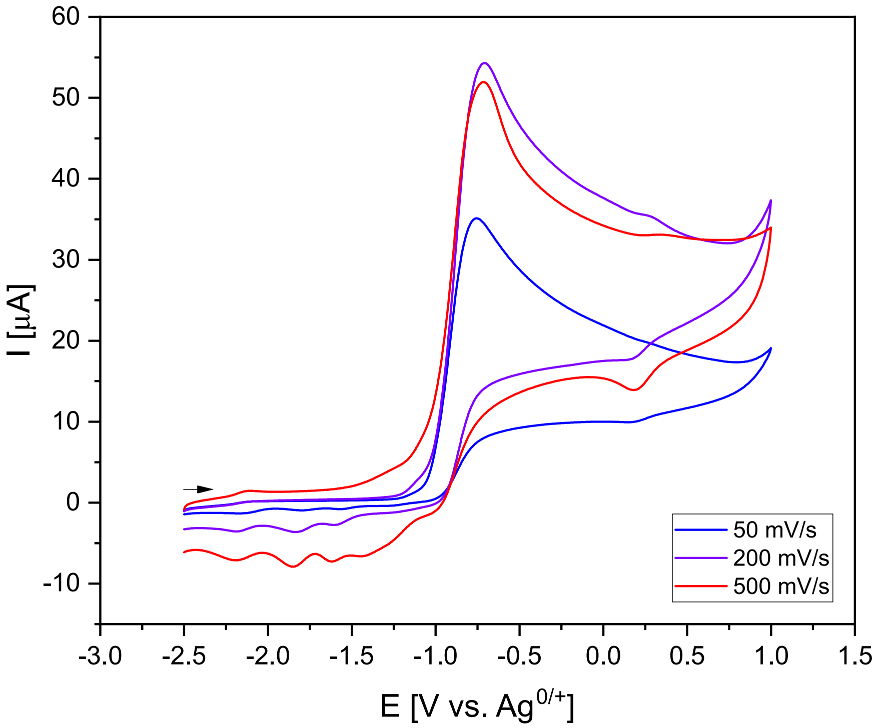


**Figure S414:** Cyclic voltammograms of **6g** (3.0±0.1 mg mL$^{-1}$) in THF (0.1 M n-$Bu_4NPF_6$) at rt; different scan rates (iR compensation = 2110 Ohm (arrows indicate scanning direction).

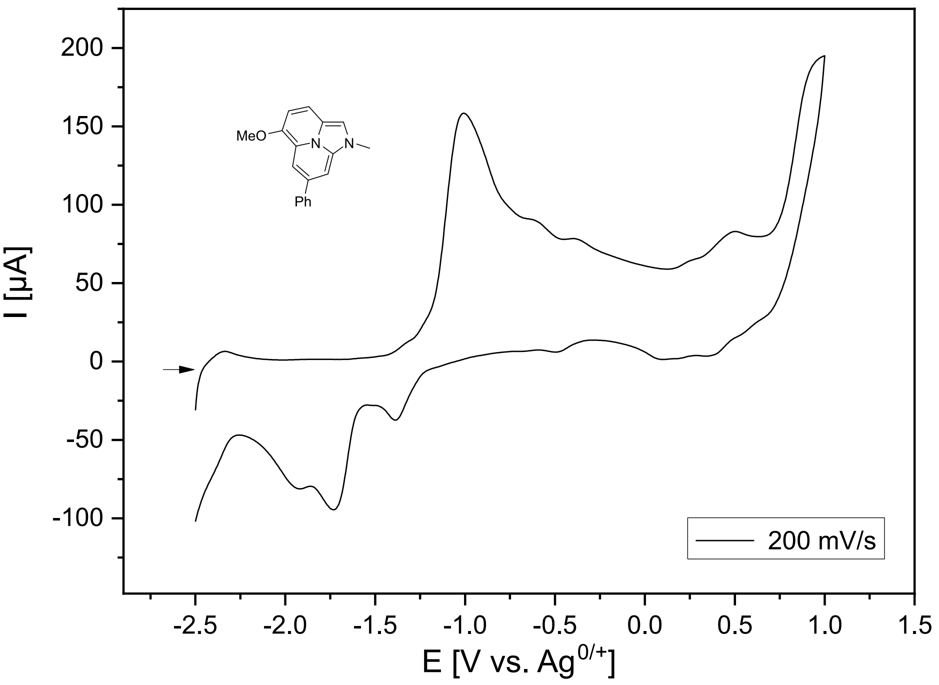


**Figure S415:** Cyclic voltammograms of **6h** (3.0±0.1 mg mL$^{-1}$) in THF (0.1 M n-$Bu_4NPF_6$) at rt; scan rate 200 mV s$^{-1}$ (iR compensation = 2107 Ohm) (arrows indicate scanning direction).

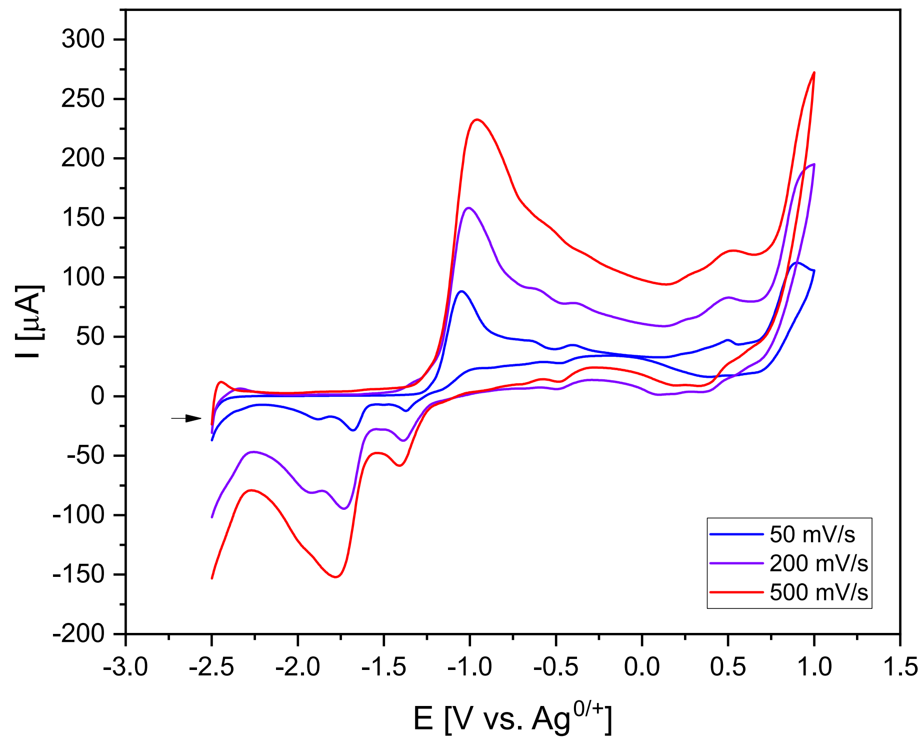


**Figure S416:** Cyclic voltammograms of **6h** (3.0±0.1 mg mL$^{-1}$) in THF (0.1 M n-$Bu_4NPF_6$) at rt; different scan rates (iR compensation = 2107 Ohm (arrows indicate scanning direction).

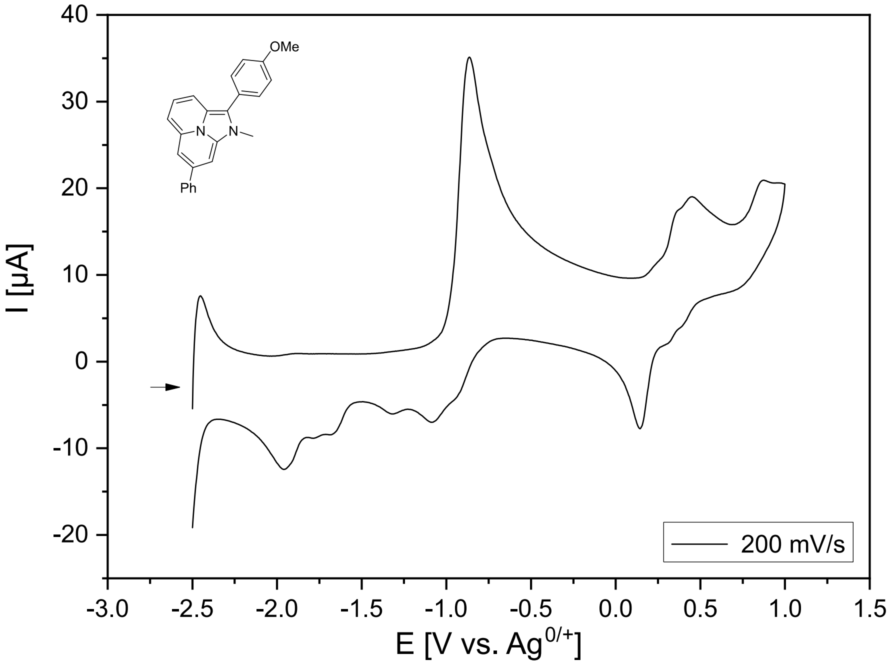


**Figure S417:** Cyclic voltammograms of **6i** (3.0±0.1 mg mL$^{-1}$) in THF (0.1 M n-$Bu_4NPF_6$) at rt; scan rate 200 mV s$^{-1}$ (iR compensation = 2041 Ohm) (arrows indicate scanning direction).

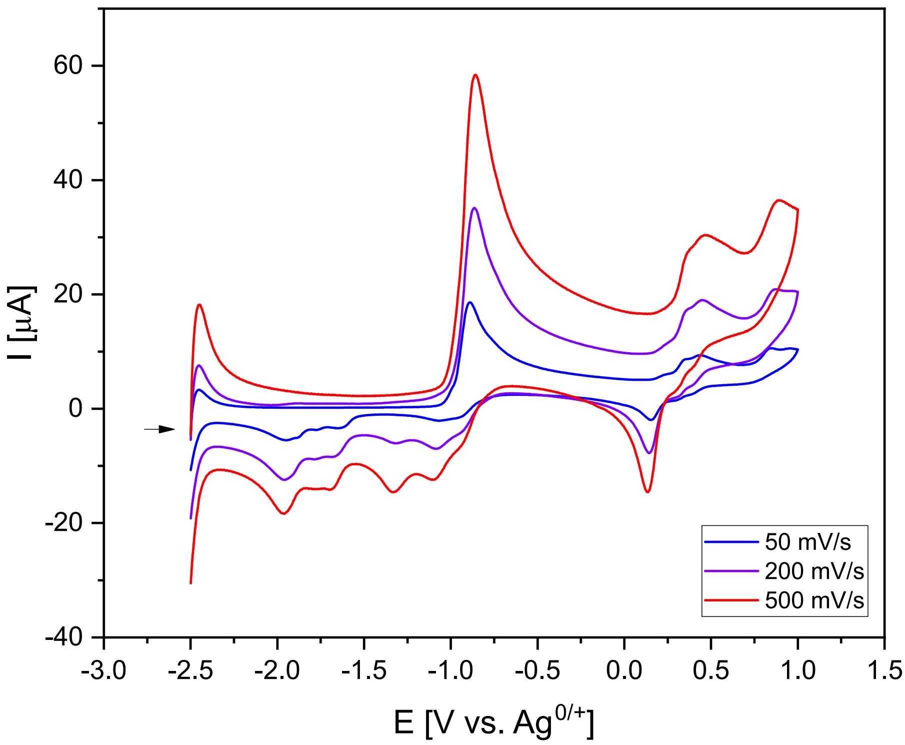


**Figure S418:** Cyclic voltammograms of **6i** (3.0±0.1 mg mL$^{-1}$) in THF (0.1 M n-$Bu_4NPF_6$) at rt; different scan rates (iR compensation = 2041 Ohm (arrows indicate scanning direction).

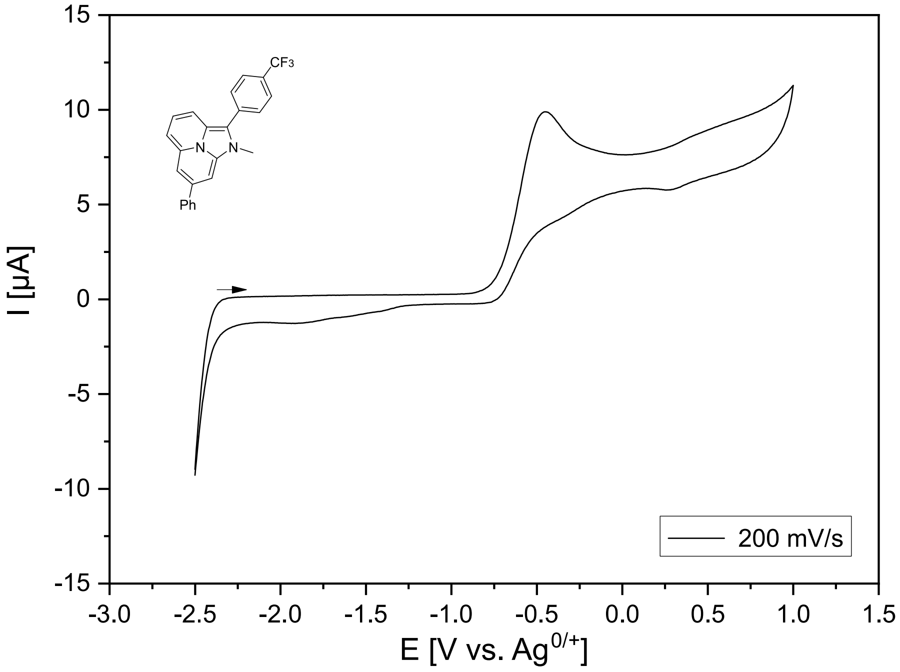


**Figure S419:** Cyclic voltammograms of **6j** (3.0±0.1 mg mL$^{-1}$) in THF (0.1 M n-$Bu_4NPF_6$) at rt; scan rate 200 mV s$^{-1}$ (iR compensation = 2030 Ohm) (arrows indicate scanning direction).

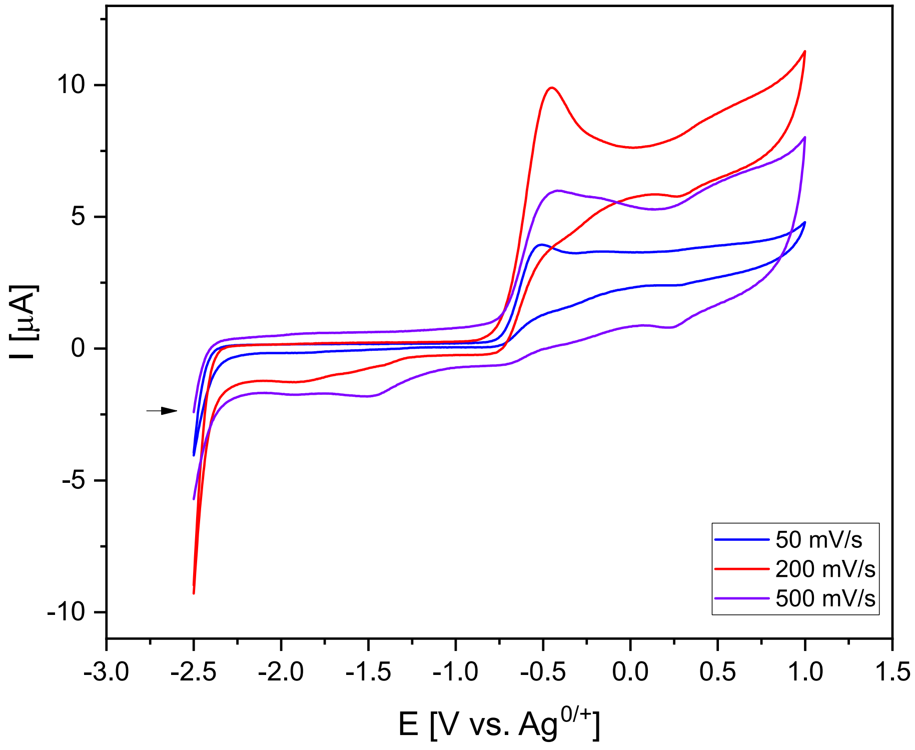


**Figure S420:** Cyclic voltammograms of **6j** (3.0±0.1 mg mL$^{-1}$) in THF (0.1 M n-$Bu_4NPF_6$) at rt; different scan rates (iR compensation = 2030 Ohm (arrows indicate scanning direction).

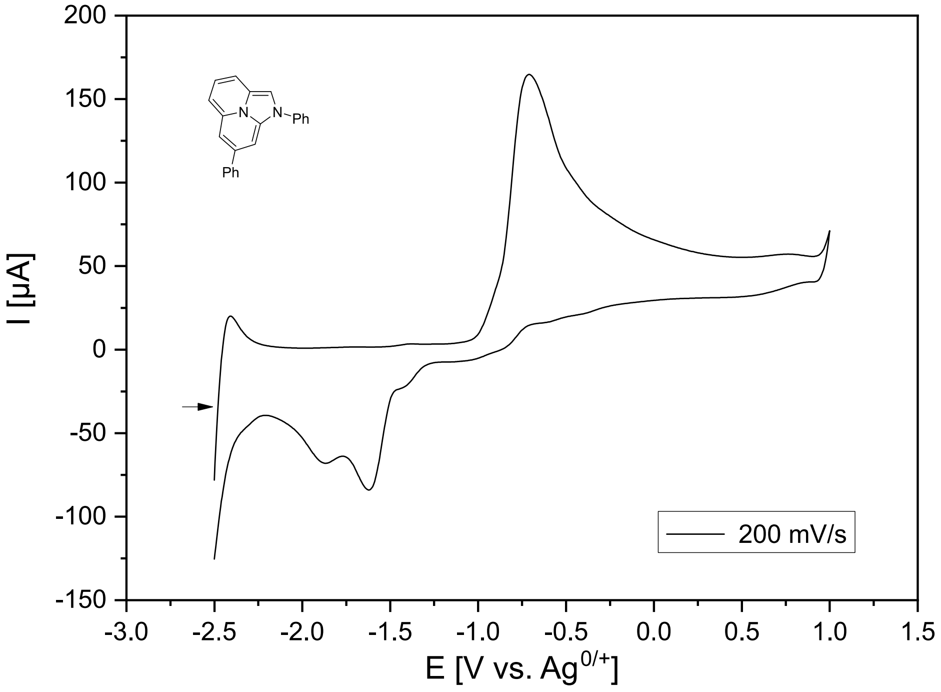


**Figure S421:** Cyclic voltammograms of **6k** (3.0±0.1 mg mL$^{-1}$) in THF (0.1 M n-$Bu_4NPF_6$) at rt; scan rate 200 mV s$^{-1}$ (iR compensation = 2065 Ohm) (arrows indicate scanning direction).

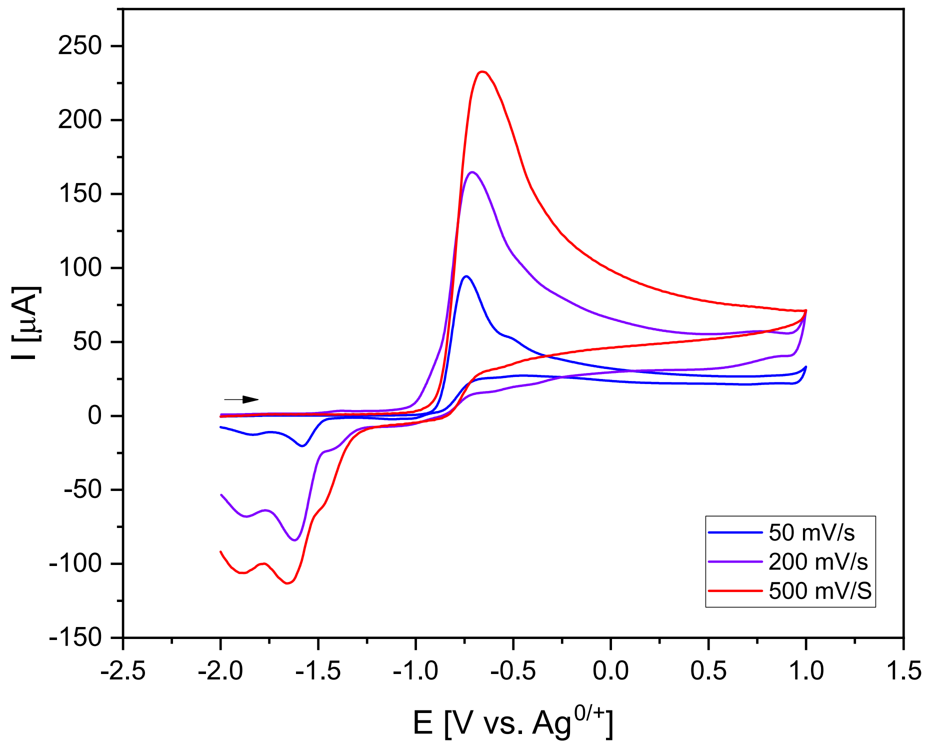


**Figure S422:** Cyclic voltammograms of **6k** (3.0±0.1 mg mL$^{-1}$) in THF (0.1 M n-$Bu_4NPF_6$) at rt; different scan rates (iR compensation = 2065 Ohm (arrows indicate scanning direction).

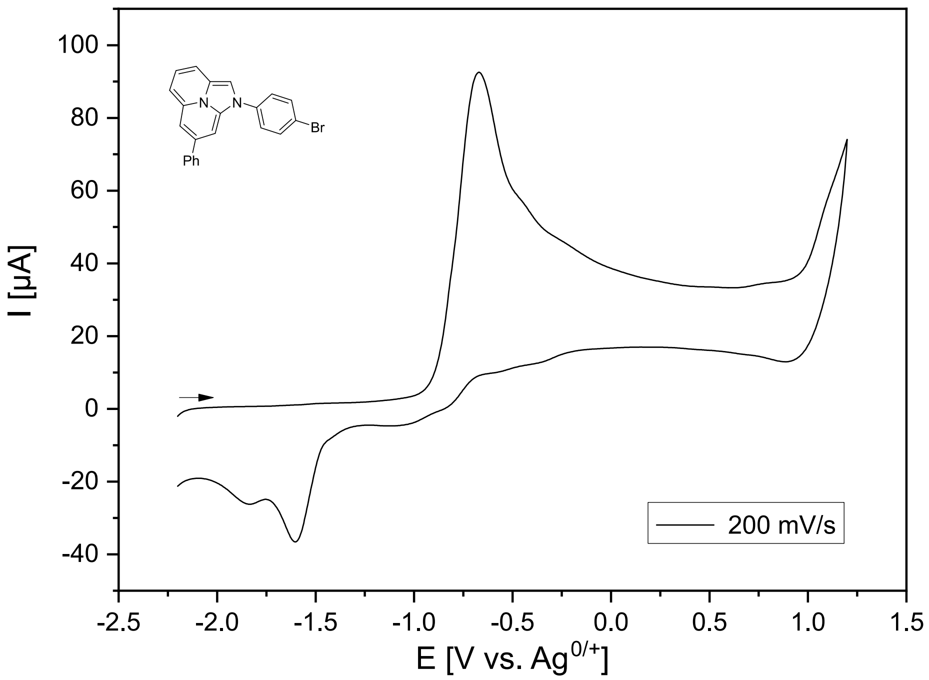


**Figure S423:** Cyclic voltammograms of **6l** (3.0±0.1 mg mL$^{-1}$) in THF (0.1 M n-$Bu_4NPF_6$) at rt; scan rate 200 mV s$^{-1}$ (iR compensation = 1771 Ohm) (arrows indicate scanning direction).

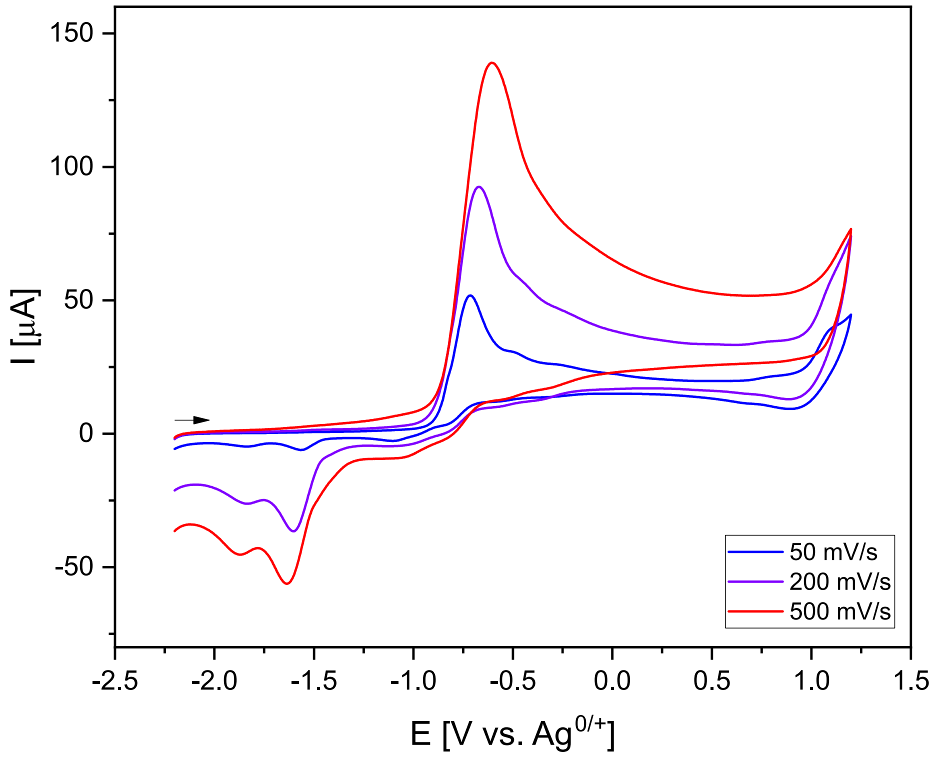


**Figure S424:** Cyclic voltammograms of **6l** (3.0±0.1 mg mL$^{-1}$) in THF (0.1 M n-$Bu_4NPF_6$) at rt; different scan rates (iR compensation = 1771 Ohm (arrows indicate scanning direction).

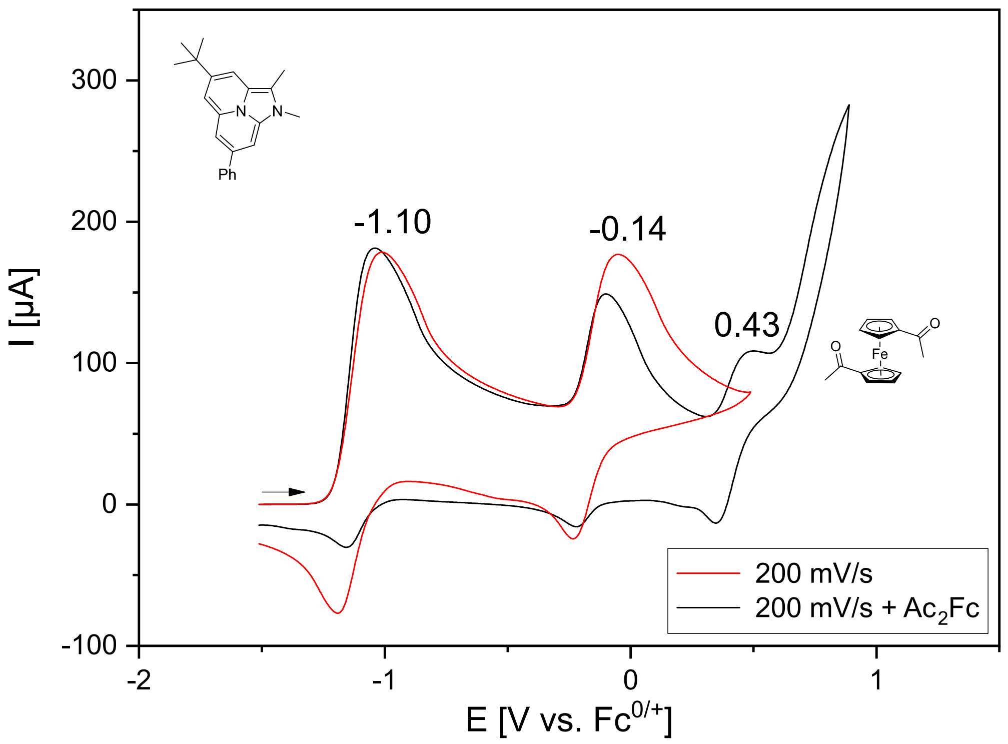


**Figure S425:** Cyclic voltammograms of **6m** (3.3±0.1 mg $mL^{-1}$) in THF (0.1 M n-$Bu_4NPF_6$) at rt; scan rate 200 mV $s^{-1}$ (iR compensation = 2000 Ohm) referenced internally against diacetyl-ferrocene (arrows indicate scanning direction).

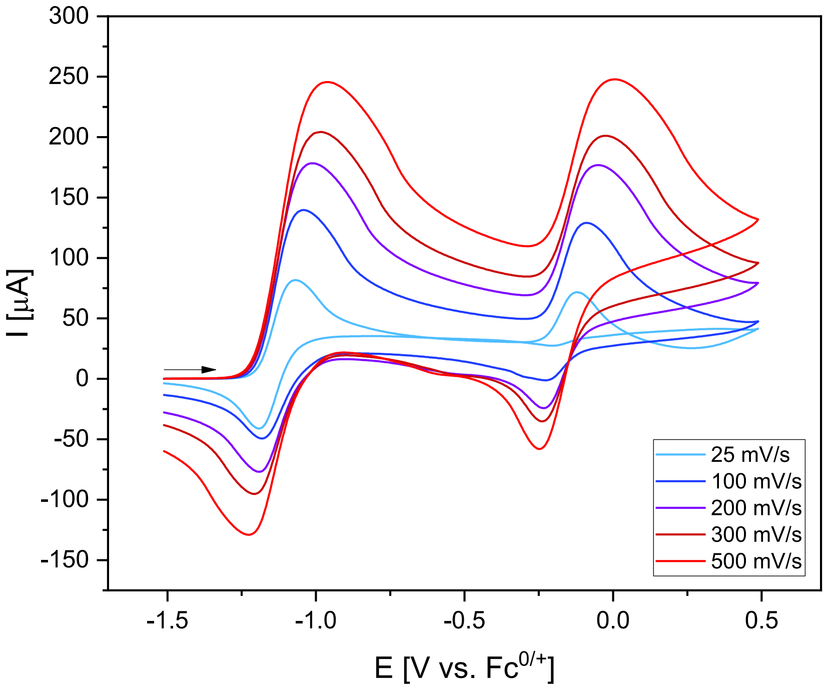


**Figure S426:** Cyclic voltammograms of **6m** (3.3±0.1 mg $mL^{-1}$) in THF (0.1 M n-$Bu_4NPF_6$) at rt; different scan rates (iR compensation = 2000 Ohm referenced internally against diacetyl-ferrocene (arrows indicate scanning direction).

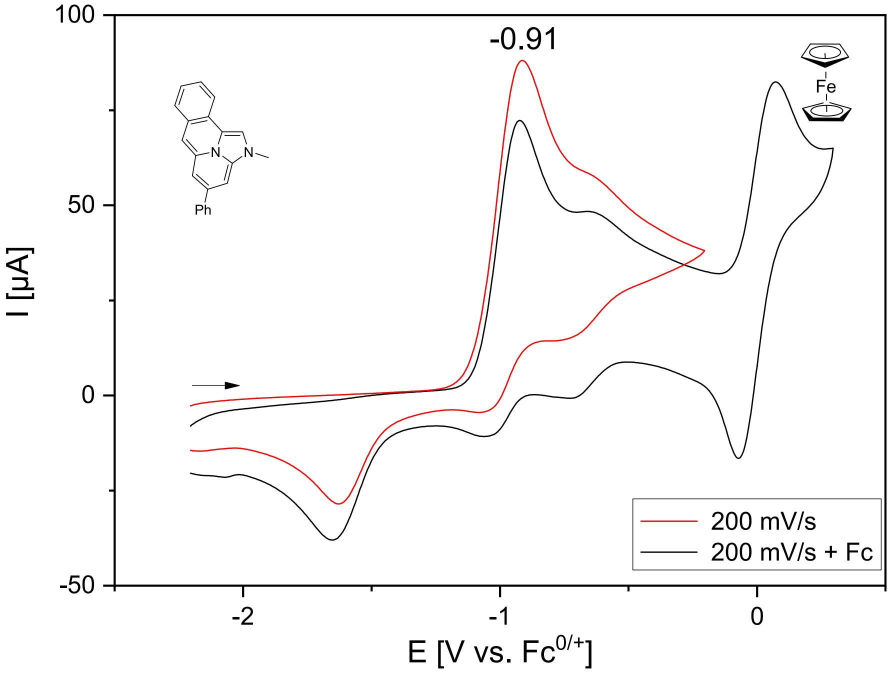


**Figure S427:** Cyclic voltammograms of **6n** (1.8±0.1 mg mL$^{-1}$) in THF (0.1 M n-$Bu_4NPF_6$) at rt; scan rate 200 mV s$^{-1}$ (iR compensation = 2170 Ohm) referenced internally against ferrocene (arrows indicate scanning direction).

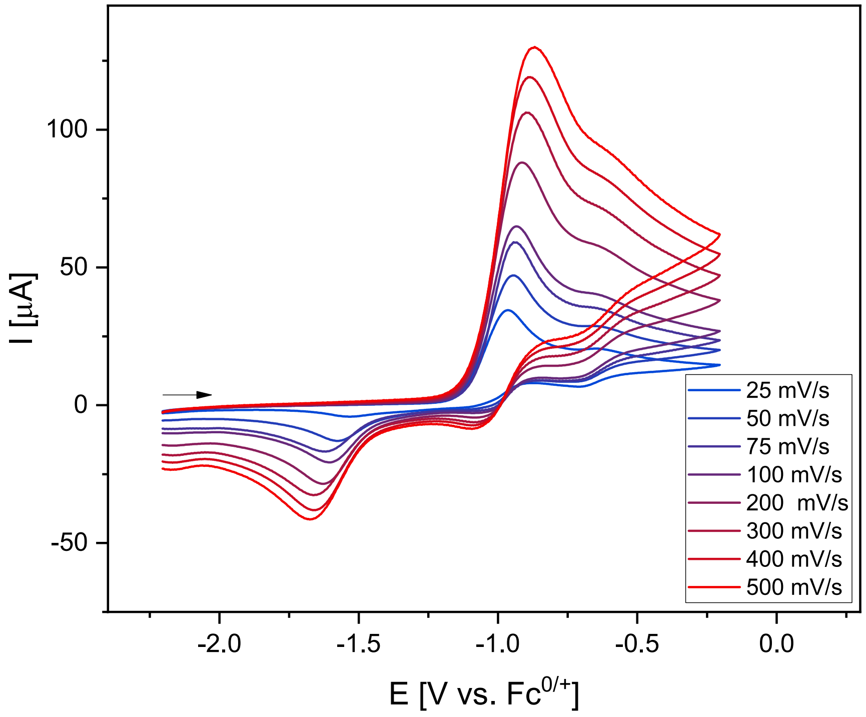


**Figure S428:** Cyclic voltammograms of **6n** (1.8±0.1 mg mL$^{-1}$) in THF (0.1 M n-$Bu_4NPF_6$) at rt; different scan rates (iR compensation = 2170 Ohm referenced internally against ferrocene (arrows indicate scanning direction).

## 6.1 Reversibility Investigations of the First Redox Event of 6m

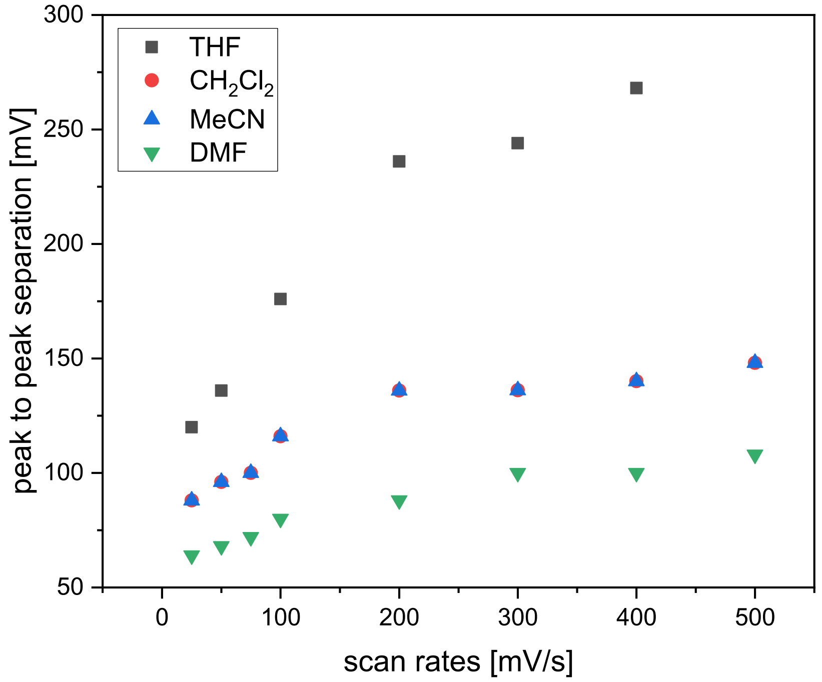


**Figure S429:** Reversibility plot of **6m** (3.3±0.1 mg mL$^{-1}$) in different solvents (0.1 M n-$Bu_4NPF_6$) at rt.

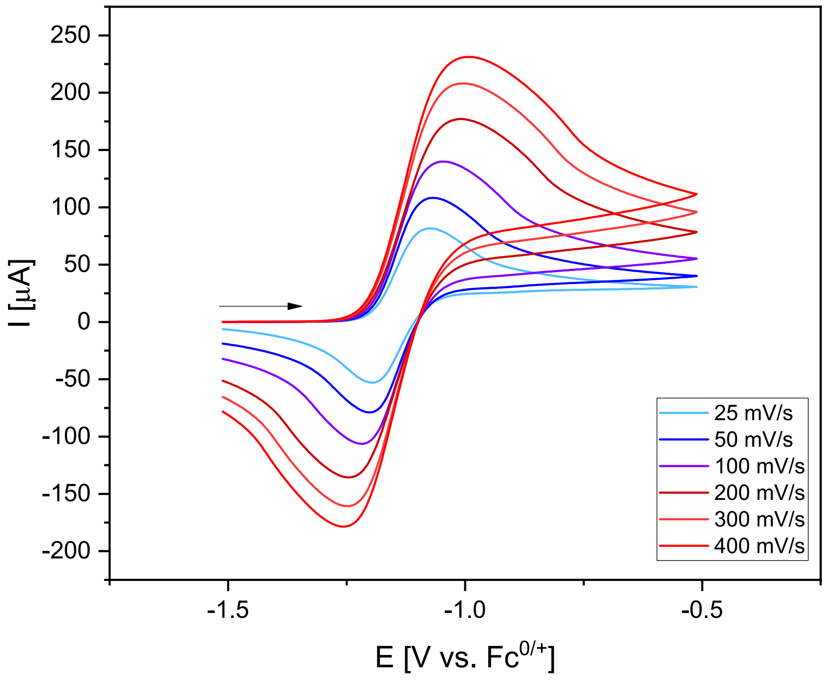


**Figure S430:** Cyclic voltammograms of **6m** (3.3±0.1 mg mL$^{-1}$) in THF (0.1 M n-$Bu_4NPF_6$) at rt; different scan rates (iR compensation = 2000 Ohm referenced internally against diacetylferrocene (arrows indicate scanning direction).

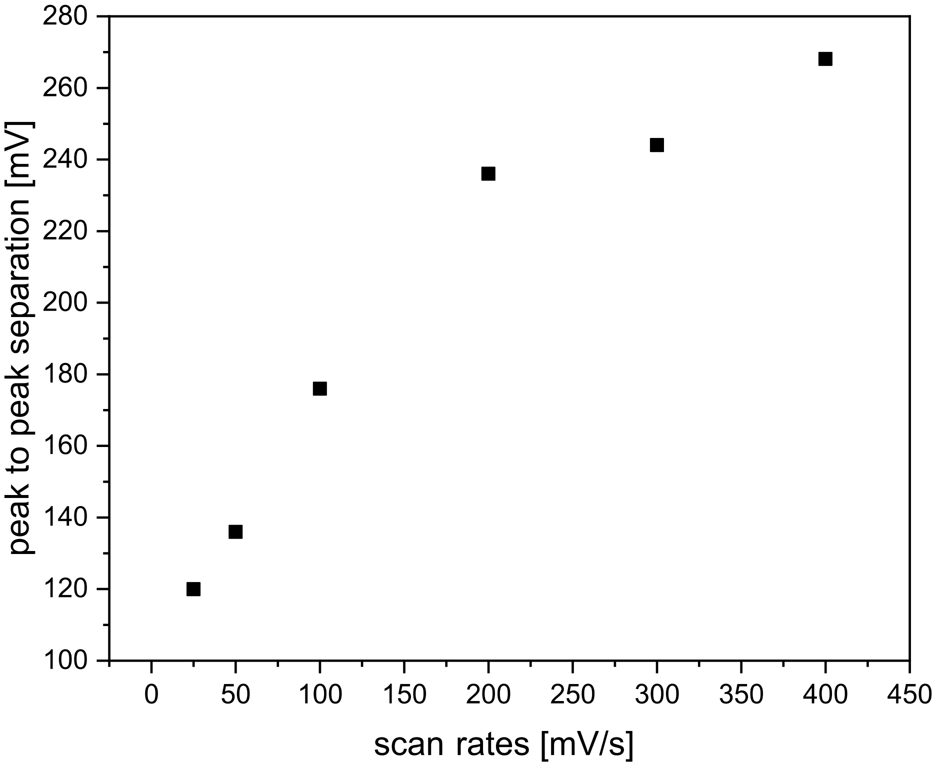


**Figure S431:** Reversibility plot of **6m** (3.3±0.1 mg mL$^{-1}$) in THF (0.1 M n-$Bu_4NPF_6$) at rt.

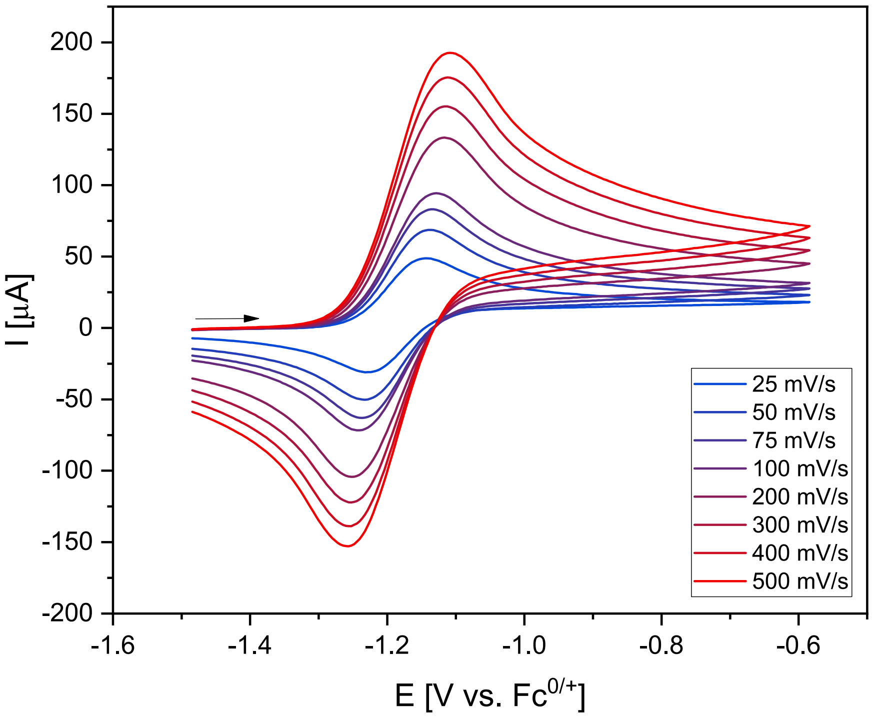


**Figure S432:** Cyclic voltammograms of **6m** (1.8±0.1 mg mL$^{-1}$) in $CH_2Cl_2$ (0.1 M n-$Bu_4NPF_6$) at rt; different scan rates (iR compensation = 800 Ohm referenced internally against diacetylferrocene (arrows indicate scanning direction).

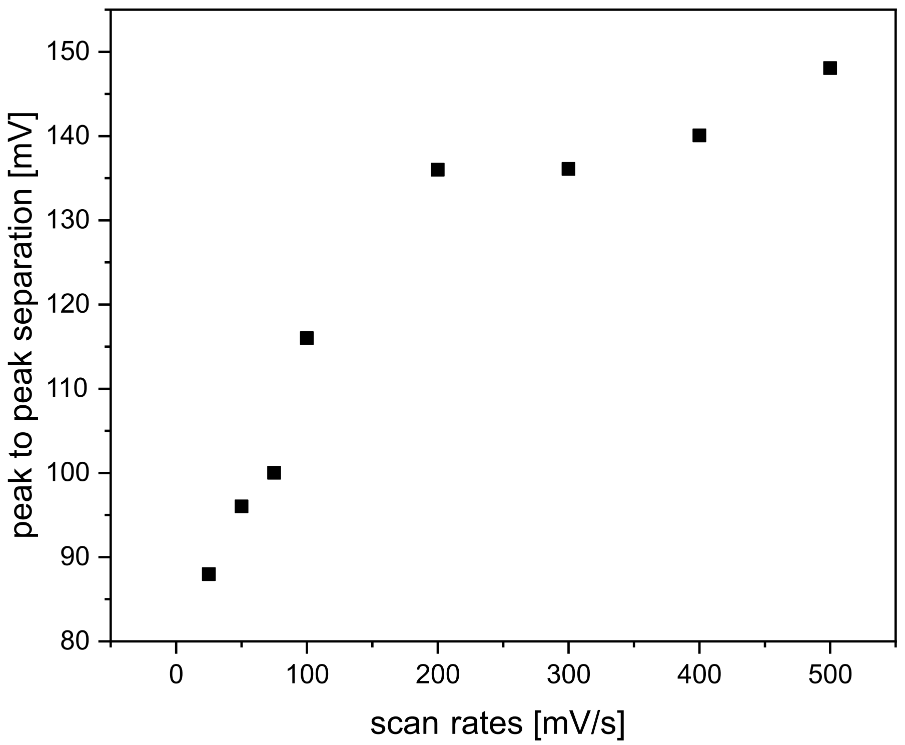


**Figure S433:** Reversibility plot of **6m** (1.8±0.1 mg $mL^{-1}$) in $CH_2Cl_2$ (0.1 M n-$Bu_4NPF_6$) at rt.

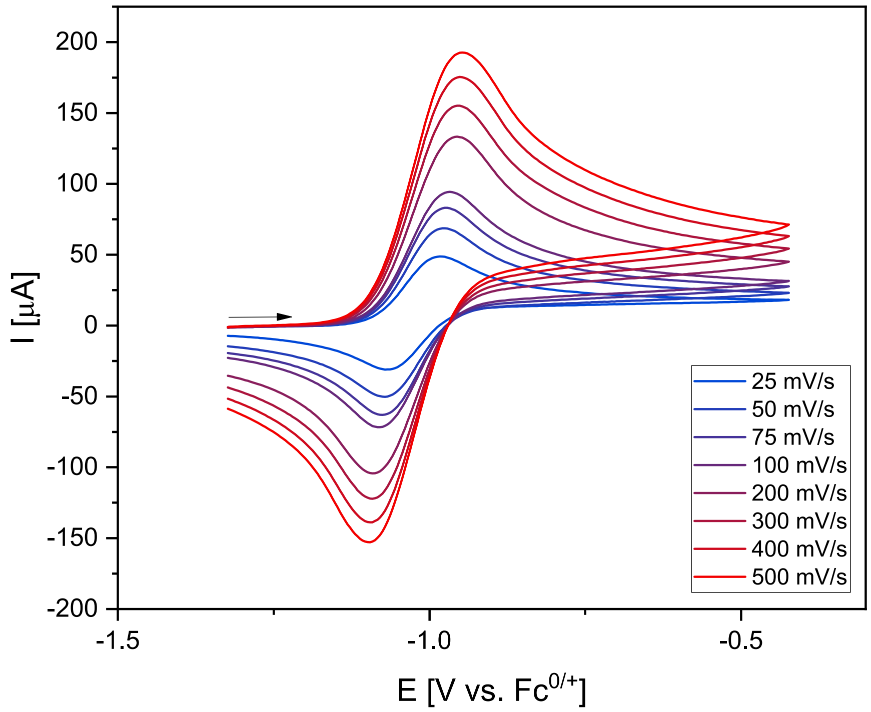


**Figure S434:** Cyclic voltammograms of **6m** (1.8±0.1 mg $mL^{-1}$) in MeCN (0.1 M n-$Bu_4NPF_6$) at rt; different scan rates (iR compensation = 110 Ohm) referenced internally against diacetylferrocene (arrows indicate scanning direction).

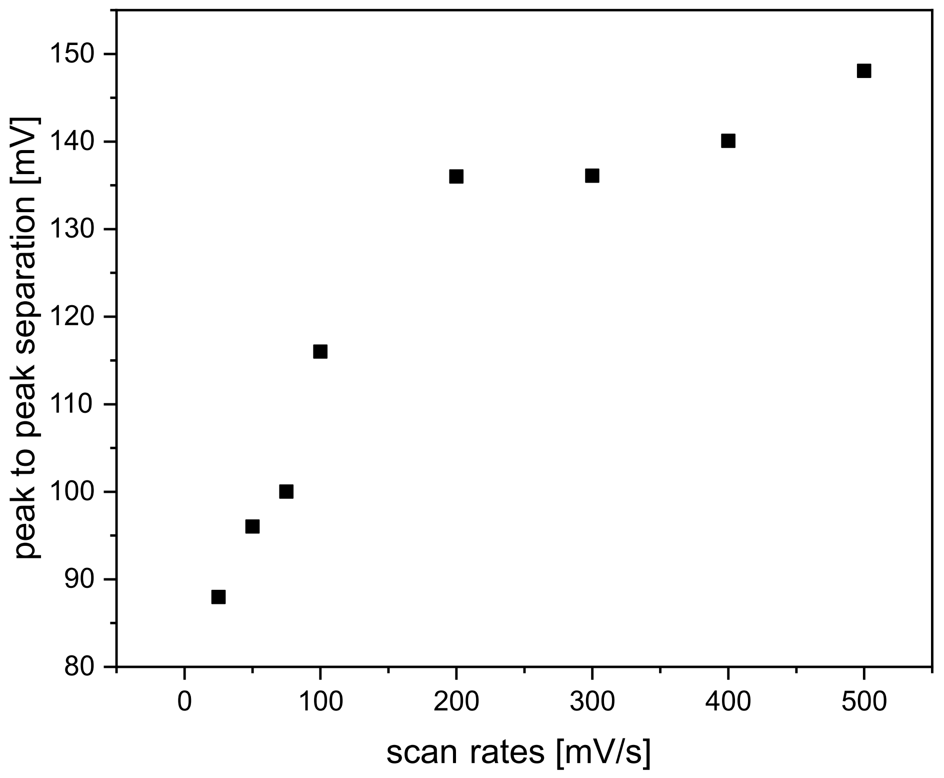


**Figure S435:** Reversibility plot of **6m** (1.8±0.1 mg $mL^{-1}$) in MeCN (0.1 M n-$Bu_4NPF_6$) at rt.

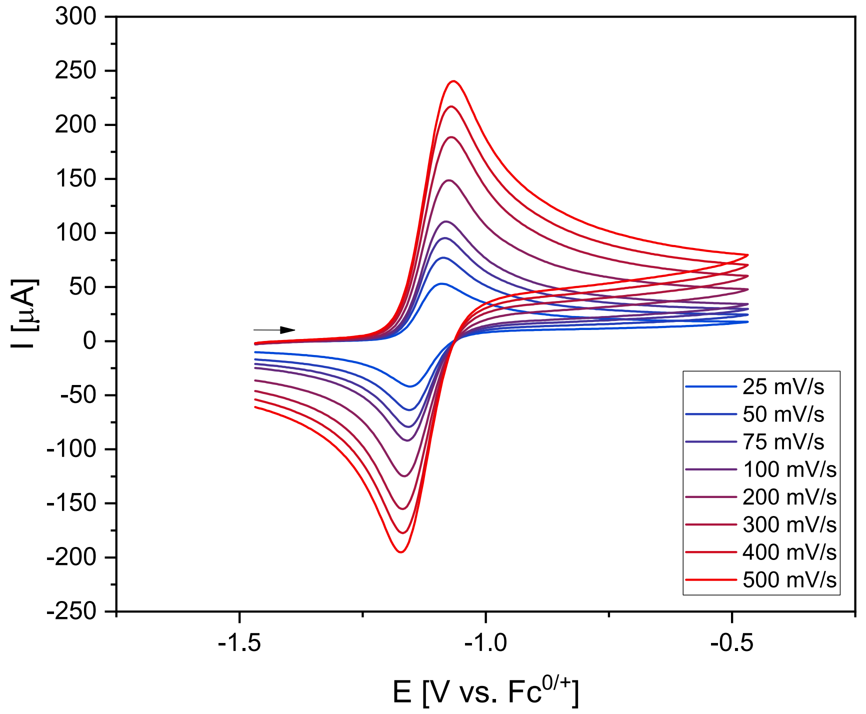


**Figure S436:** Cyclic voltammograms of **6m** (2.2±0.1 mg $mL^{-1}$) in DMF (0.1 M n-$Bu_4NPF_6$) at rt; different scan rates (iR compensation = 230 Ohm (arrows indicate scanning direction) referenced internally against ferrocene (arrows indicate scanning direction).

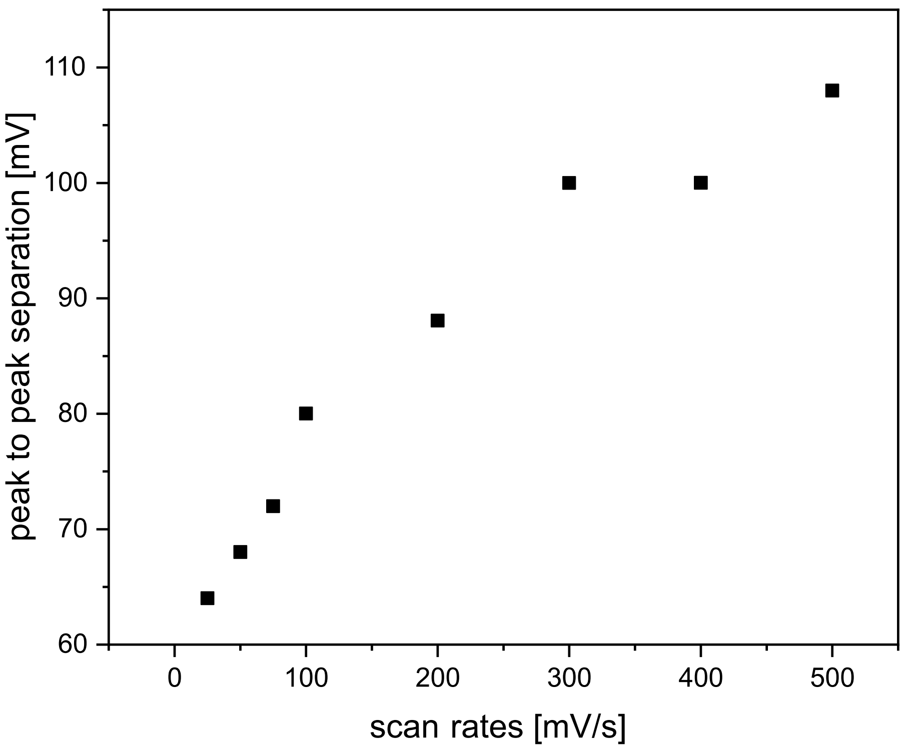


**Figure S437:** Reversibility plot of **6m** (2.2±0.1 mg mL$^{-1}$) in DMF (0.1 M n-$Bu_4NPF_6$) at rt.

### 6.2 Solvent-Influence on 6m

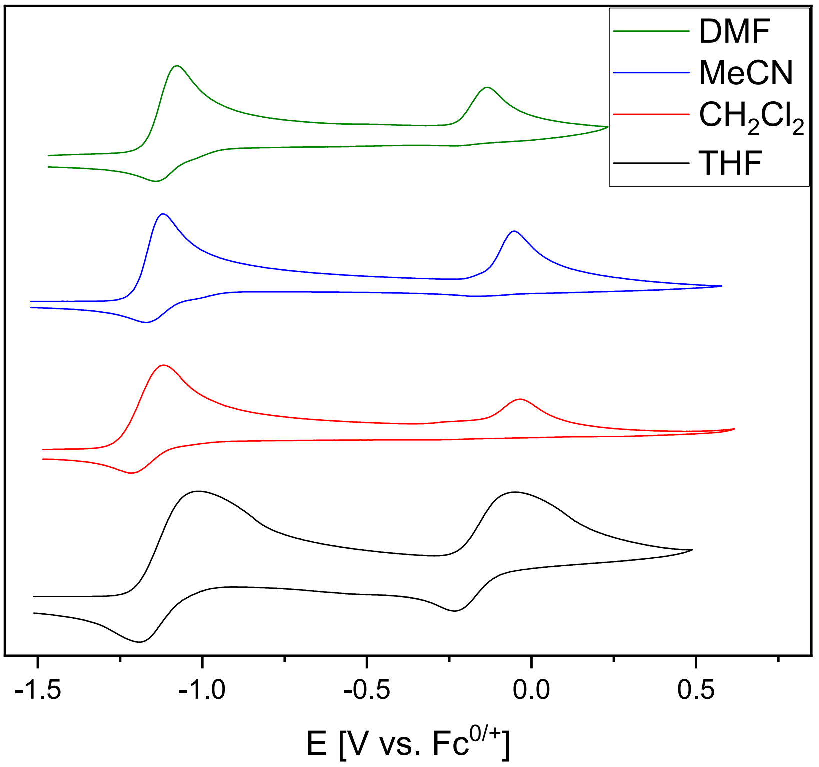


**Figure S438:** Cyclic voltammograms of **6m** in different solvents (0.1 M n-$Bu_4NPF_6$) at rt; 200 mV $s^{-1}$ referenced internally against diacetylferrocene or ferrocene.

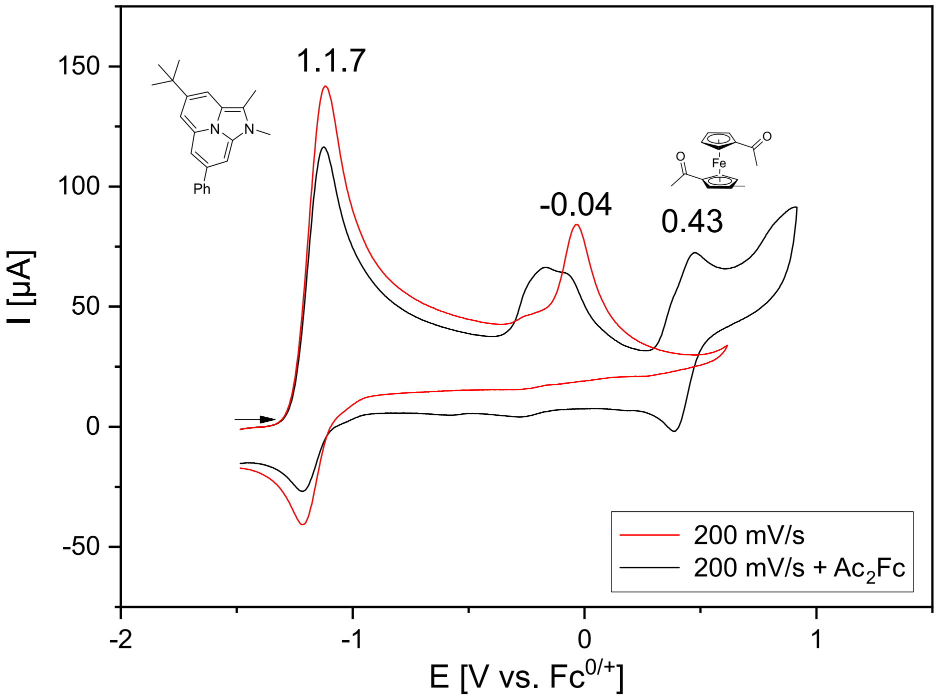


**Figure S439:** Cyclic voltammograms of **6m** (1.8±0.1 mg $mL^{-1}$) in $CH_2Cl_2$ (0.1 M n-$Bu_4NPF_6$) at rt; scan rate 200 mV $s^{-1}$ (iR compensation = 800 Ohm) referenced internally against diacetylferrocene (arrows indicate scanning direction).

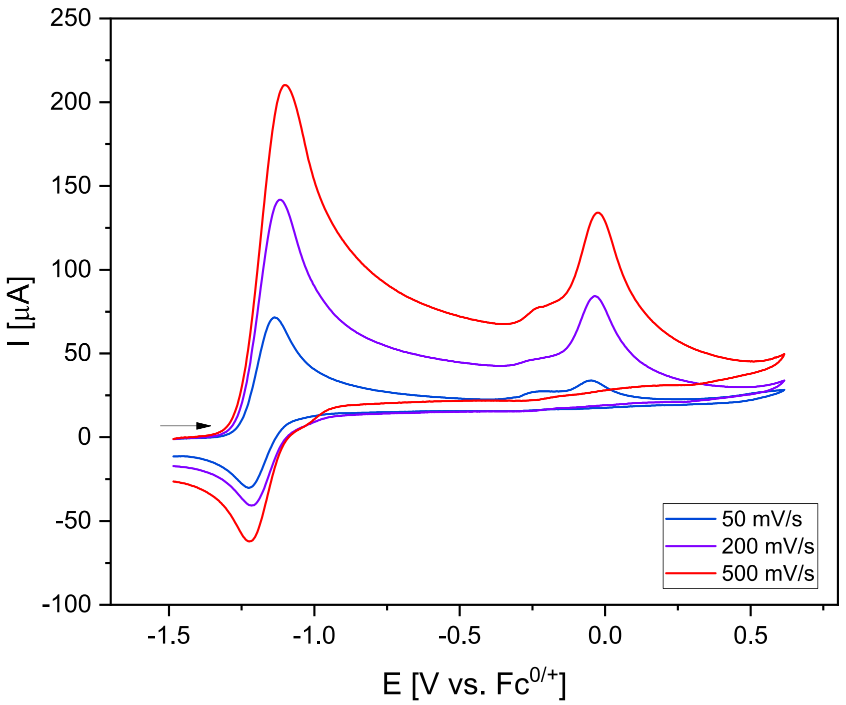


**Figure S440:** Cyclic voltammograms of **6m** (1.8±0.1 mg mL$^{-1}$) in $CH_2Cl_2$ (0.1 M n-$Bu_4NPF_6$) at rt; different scan rates (iR compensation = 800 Ohm (arrows indicate scanning direction) referenced internally against diacetylferrocene (arrows indicate scanning direction).

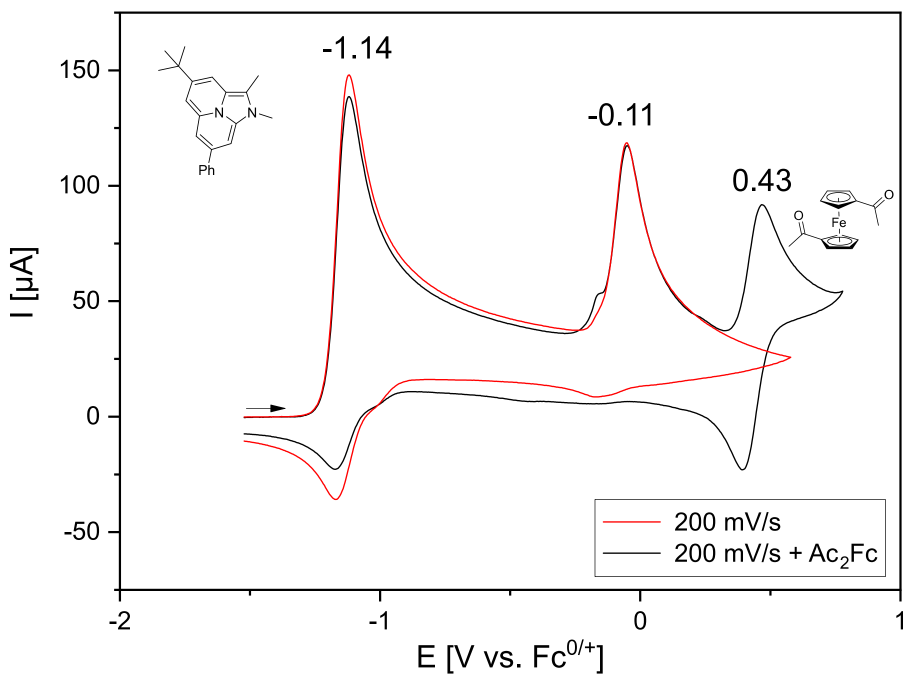


**Figure S441**: Cyclic voltammograms of **6m** (1.8±0.1 mg mL$^{-1}$) in MeCN (0.1 M n-$Bu_4NPF_6$) at rt; scan rate 200 mV s$^{-1}$ (iR compensation = 110 Ohm) referenced internally against diacetylferrocene (arrows indicate scanning direction).

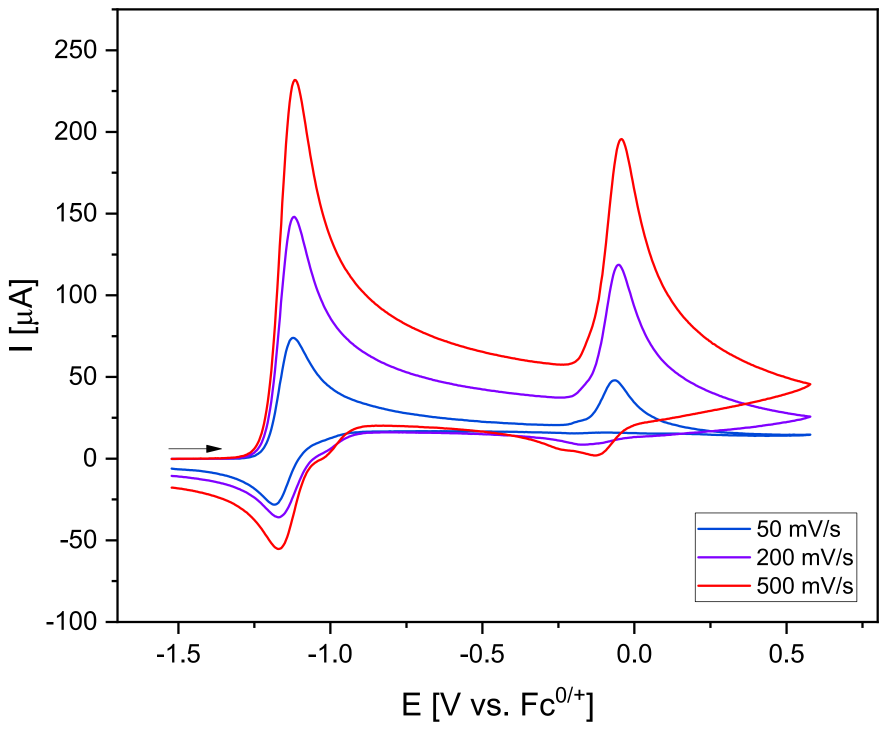


**Figure S442:** Cyclic voltammograms of **6m** (1.8±0.1 mg mL$^{-1}$) in MeCN (0.1 M n-$Bu_4NPF_6$) at rt; different scan rates (iR compensation = 110 Ohm (arrows indicate scanning direction) referenced internally against diacetylferrocene (arrows indicate scanning direction).

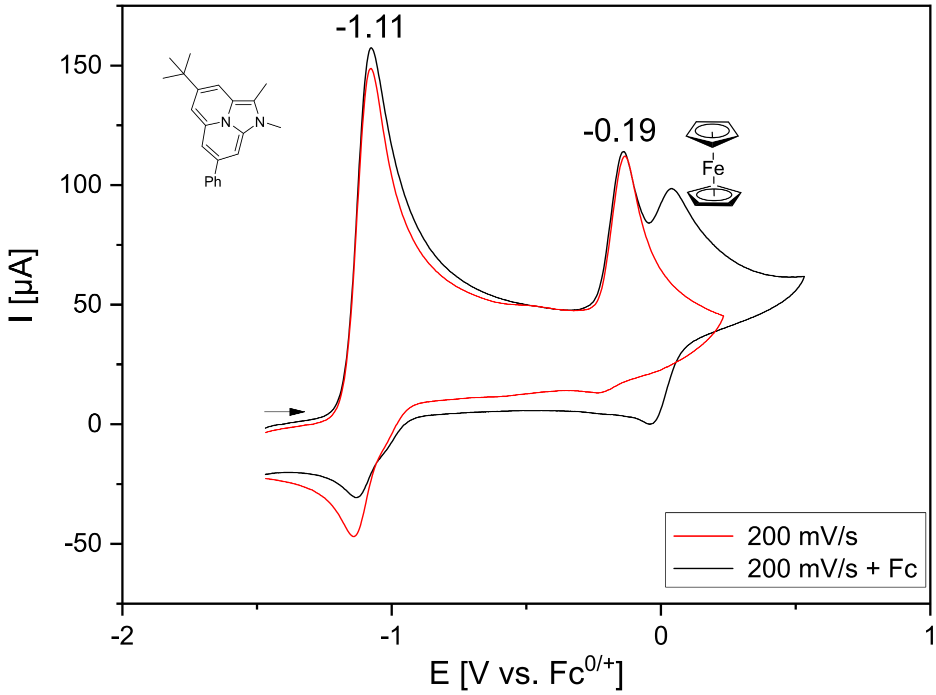


**Figure S443:** Cyclic voltammograms of **6m** (2.2±0.1 mg mL$^{-1}$) in DMF (0.1 M n-$Bu_4NPF_6$) at rt; scan rate 200 mV s$^{-1}$ (iR compensation = 230 Ohm) referenced internally against ferrocene (arrows indicate scanning direction).

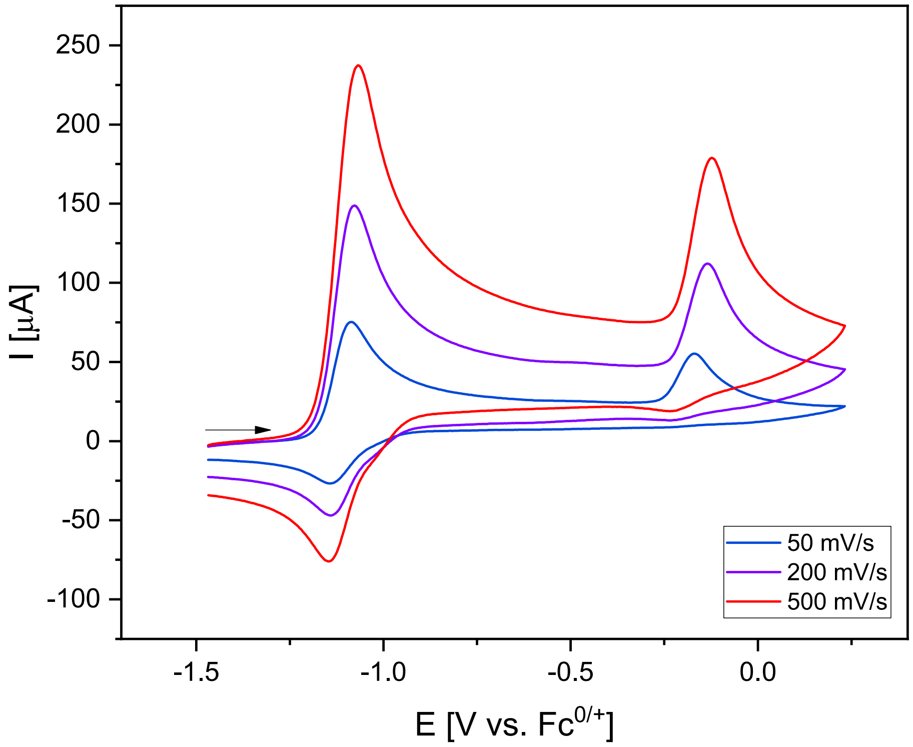


**Figure S444:** Cyclic voltammograms of **6m** (2.2±0.1 mg $mL^{-1}$) in DMF (0.1 M n-$Bu_4NPF_6$) at rt; different scan rates (iR compensation = 230 Ohm (arrows indicate scanning direction) referenced internally against ferrocene (arrows indicate scanning direction).

7. **Spectroelectrochemistry**

Electrochemical measurements were recorded with a Gamry Instruments Reference 600+. The samples were measured starting from the neutral compounds, containing a three-electrode setup (platinum wire, platinum net and Ag/$AgNO_3$ reference electrode) in a UV-vis cell (1 mm diameter from ALS Co., Ltd; SEC-C) under nitrogen. In order to guarantee a clean oxygen free setup, the measurements were performed in a nitrogen filled glove box. The reference electrode was freshly prepared by using a fritted sample holder, which was activated as described for the sample holder preparation for cyclic voltammetry. To the fritted sample holder was added a freshly prepared 0.01 M $AgNO_3$/ 0.1 M *n*-$Bu_4NPF_6$ solution in $CH_3CN$ and a silver wire. The light source was a deuterium/tungsten light source and light detected with a BWTEK ExemplarLS. A blank spectrum and a reference spectrum with just solvent was taken in advance and was subtracted from the measured data.

**Procedure:** A UV-vis spectrum was measured every 10 seconds, while the potential was scanned from negative potentials to positive potentials by a scan rate of 2.0 mV/s.

For the visualization of the data not every spectrum but selected are shown in order to reduce the data amount.

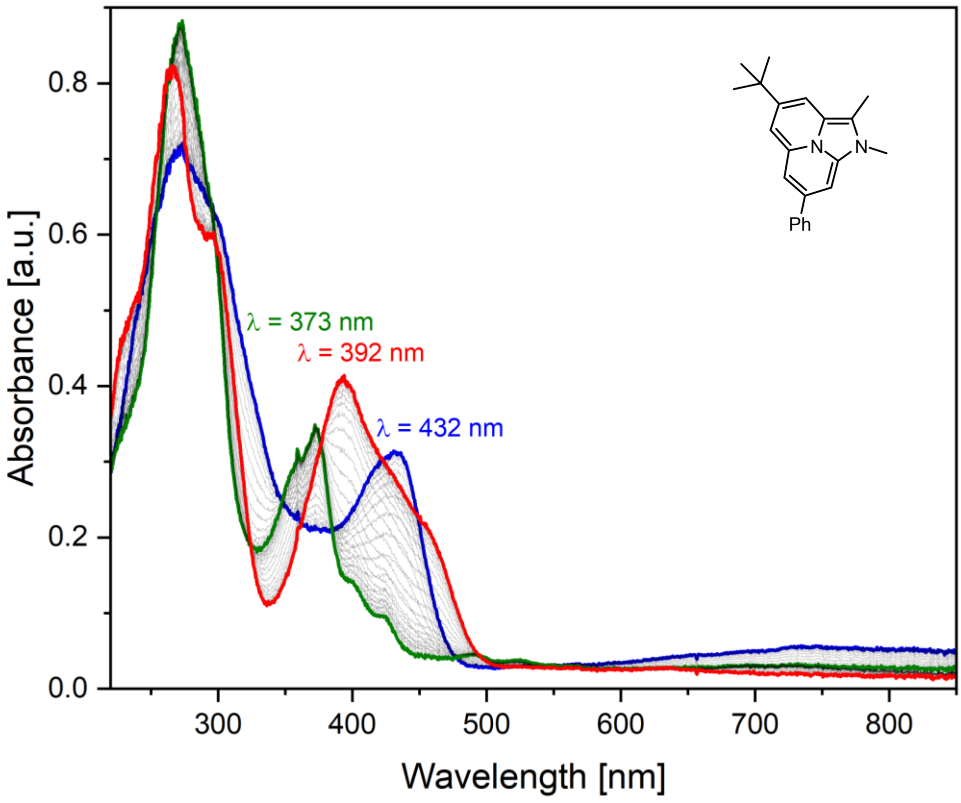


**Figure S445:** UV-vis-SEC of **6m** in THF. Color coding: **neutral** (blue), **radkat** (green), **dicat** (red). Starting potential (vs. $Ag^0/Ag^+$): -1.3 V; End potential (vs. $Ag^0/Ag^+$): 0.7 V.

## 8. X-Ray Characterization Data

All crystal structures (**2a – 6m$^{+\cdot}$**) were determined using the Bruker D8 Venture four-circle diffractometer equipped with a PHOTON II CPAD detector from Bruker AXS GmbH. For all structural measurements, Mo K$\alpha$ radiation was used. The APEX 5 Suite (version 2023.9-2)[19] integrated with SAINT (integration) and SADABS (adsorption correction) programs by Bruker AXS GmbH was used for data collection. The processing and finalization of the crystal structure were performed using Olex2.[20] The crystal structures were determined by the ShelXT[21] structure solution program using the intrinsic phasing option and further refined by the ShelXL[22] refinement package using least-squares minimization.[23] For the hydrogen atoms, the standard values of the SHELXL program were used with $U_{\text{iso}}(\text{H}) = -1.2U_{\text{eq}}(\text{C})$ for $CH_2$ and CH and $U_{\text{iso}}(\text{H}) = -1.5U_{\text{eq}}(\text{C})$ for $CH_3$. Some H atoms were refined freely using independent values for each $U_{\text{iso}}(\text{H})$.

The following points highlight structural peculiarities to be considered during crystallographic refinement and subsequent analysis.

**2a**: Due to disorder of the entire molecule in the asymmetric unit, only one occupied part was used for the structural discussion. The occupancy of this more relevant part is approximately 93% (A), while the second part has an occupancy of only 7% (B). Therefore, the structure of the very weakly occupied part is not further examined and the structural discussion is consistently based on the higher occupied part (A).

**3c**: The unexplained electron density, which resulted in A- and B-alerts, could not be further described or refined. Due to additional disorder of the Dipp-substituent, it is assumed that a minor portion of the crystal is twinned. Even a slight degree of twinning would lead to a significant excess of residual electron density due to the high electron density of the rhodium atom, as observed in the structure. Thus, the constitution of the molecule can be clearly confirmed.

**5b-Au**: In the gold complex, a B-alert is observed because two carbon atoms in a higher occupancy pathway were not refined anisotropically. This can be attributed to the disorder of two carbon atoms in the TIPS group, which cannot be precisely described due to insufficient residual electron density. Consequently, atoms C36A and C37A were refined isotropically, as specified in the annotated CIF file following the IUPAC standard. However, this has only a minor impact on the structural discussion in the manuscript and is not of significant relevance.

**6m$^{+\cdot}$**: The two disorders involving the F-atoms, which are frequently observed in analogous salts, as well as the *tert*-butyl group, were both resolved without issues and refined to the best extent possible, such that no A- or B-alerts are present for the structure.

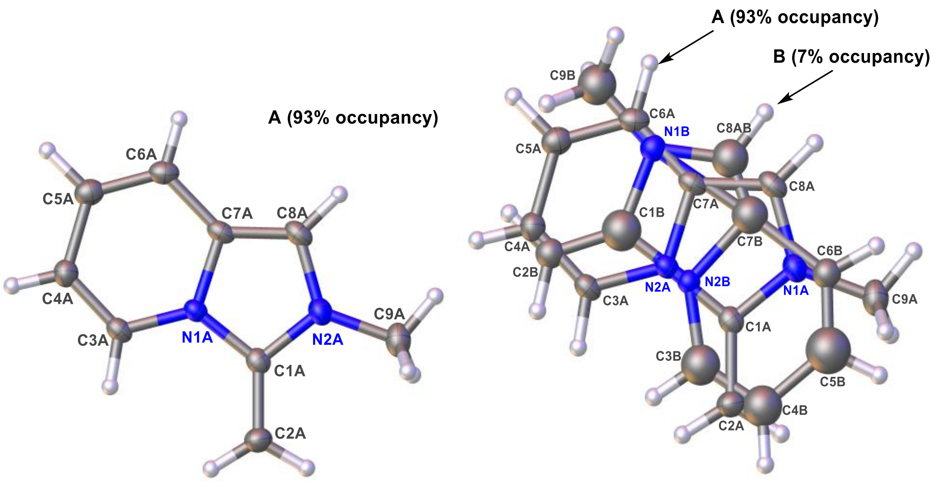


**Figure S446**: X-ray solid state structure of **2a** (93% (A) and 7% (B) occupancy). Thermal ellipsoids are shown with 50% probability. Selected bond lengths and angles in [A] and [°]: C1–C2: 1.354(3); C1–N1: 1.387(2); C1–N2: 1.389(2); C7–C8: 1.348(3).

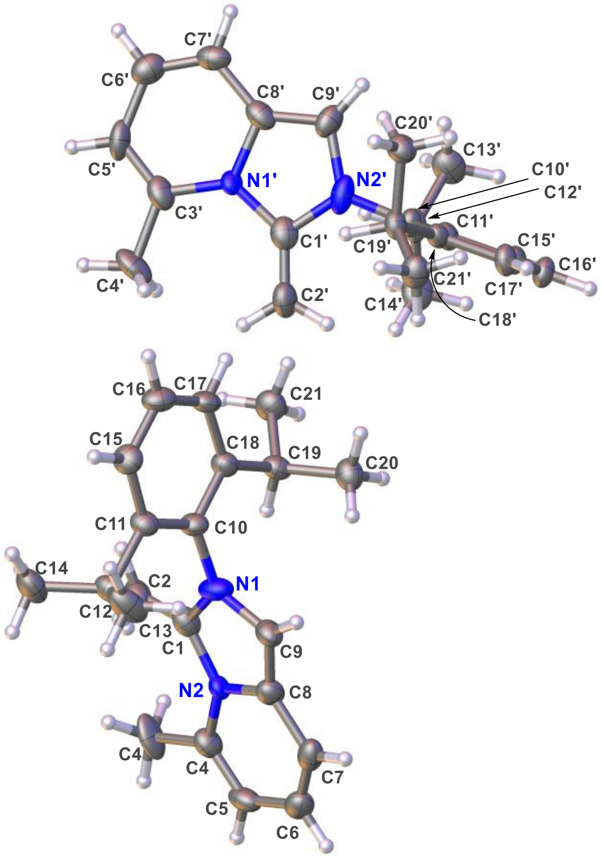


**Figure S447**: X-ray solid state structure of **2c**. Thermal ellipsoids are shown with 50% probability. Selected bond lengths and angles in [A] and [°]: C1–C2: 1.305(10); C1'–C2': 1.291(10); C1–N1: 1.353(8); C1'–N1': 1.430(8); C1–N2: 1.428(8); C1'–N2': 1.363(9); C8–C9: 1.359(9); C8'–C9': 1.349(9).

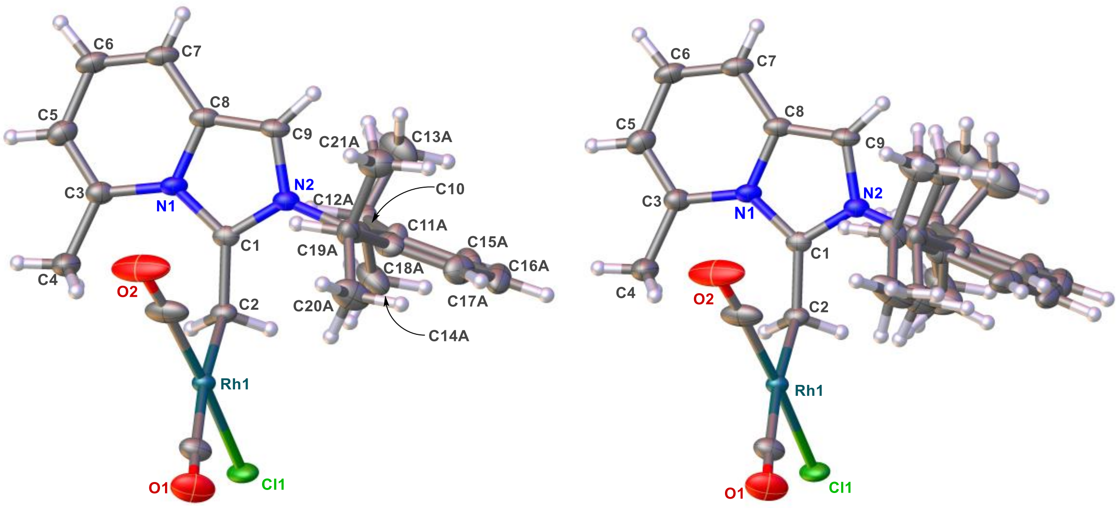


**Figure S448**: X-ray solid state structure of **3c** shown with and without disorders. Thermal ellipsoids are shown with 50% probability. Selected bond lengths and angles in [A] and [°]: C1–C2: 1.458(4); C1–N1: 1.369(3); C1–N2: 1.349(3); C8–C9: 1.364(4).

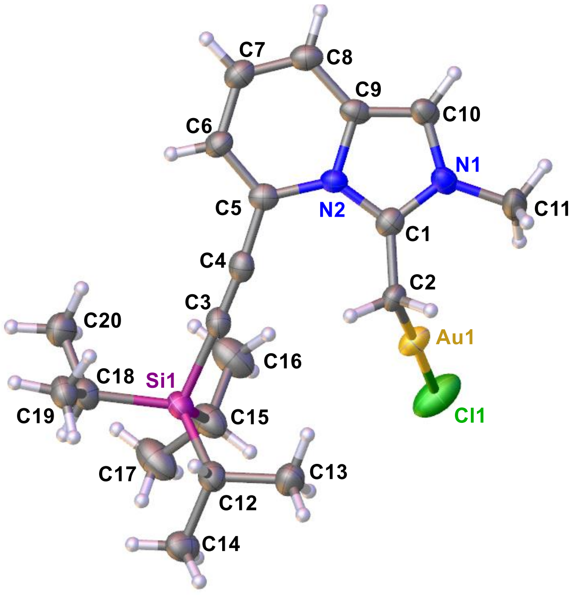


**Figure S449**: X-ray solid state structure of **5b-Au**. Thermal ellipsoids are shown with 50% probability.

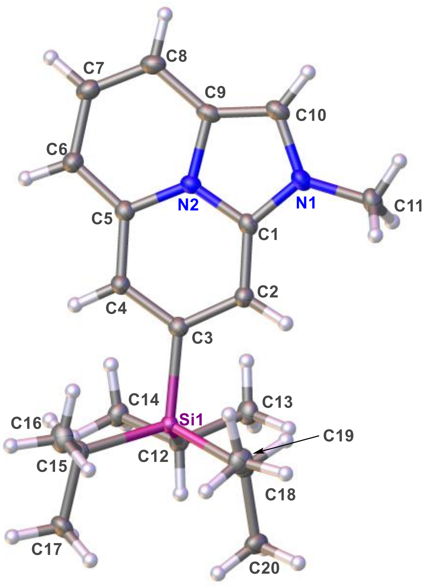


**Figure S450**: X-ray solid state structure of **6b**. Thermal ellipsoids are shown with 50% probability.

**Table S1:** Selected bond lengths for **6b.**

| **Atom** | **Atom** | **Length** / Å |
|---|---|---|
| N1 | C1 | 1.3546(15) |
| N1 | C10 | 1.4093(16) |
| N2 | C1 | 1.3669(15) |
| N2 | C5 | 1.3912(15) |
| N2 | C9 | 1.4110(14) |
| C1 | C2 | 1.3965(16) |
| C2 | C3 | 1.4123(16) |
| C3 | C4 | 1.3965(16) |
| C4 | C5 | 1.4209(16) |
| C5 | C6 | 1.3903(16) |
| C6 | C7 | 1.4195(17) |
| C7 | C8 | 1.3671(18) |
| C8 | C9 | 1.4404(18) |
| C9 | C10 | 1.3597(18) |

**Table S2**: Selected bond angles for **6b**.

| **Atom** | **Atom** | **Atom** | **Angle** / ° |
|---|---|---|---|
| C1 | N2 | C5 | 124.27(10) |
| C1 | N2 | C9 | 110.19(10) |
| C1 | N1 | C11 | 124.06(11) |
| C1 | N1 | C10 | 109.52(10) |
| C1 | C2 | C3 | 117.31(11) |
| C3 | C4 | C5 | 122.56(11) |
| C4 | C3 | C2 | 120.20(10) |
| C5 | N2 | C9 | 125.54(10) |
| C5 | C6 | C7 | 120.16(11) |
| C6 | C5 | N2 | 116.32(10) |
| C6 | C5 | C4 | 129.14(11) |
| C7 | C8 | C9 | 118.47(11) |
| C8 | C7 | C6 | 123.07(12) |
| C9 | C10 | N1 | 107.82(10) |
| C10 | N1 | C11 | 126.26(11) |
| C10 | C9 | N2 | 105.99(11) |
| C10 | C9 | C8 | 137.60(11) |
| N1 | C1 | N2 | 106.45(10) |
| N1 | C1 | C2 | 132.45(11) |
| N2 | C1 | C2 | 121.09(10) |
| N2 | C5 | C4 | 114.53(10) |
| N2 | C9 | C8 | 116.41(11) |

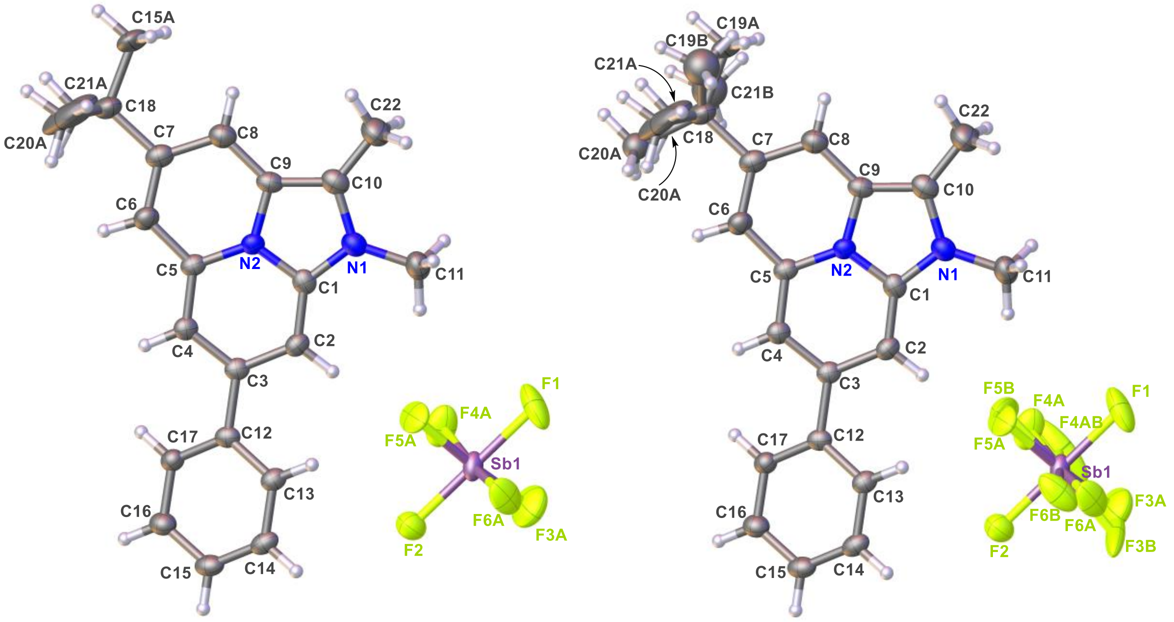


**Figure S451**: X-ray solid state structure of **6m**$^{+\cdot}$ depicted with and without disorders. Thermal ellipsoids are shown with 50% probability.

**Table S3:** Selected bond lengths for **6m**$^{+\cdot}$.

| **Atom** | **Atom** | **Length** / Å |
|---|---|---|
| N1 | C1 | 1.352(6) |
| N1 | C10 | 1.407(7) |
| N2 | C1 | 1.373(5) |
| N2 | C5 | 1.366(6) |
| N2 | C9 | 1.391(5) |
| C1 | C2 | 1.376(6) |
| C2 | C3 | 1.401(7) |
| C3 | C4 | 1.403(6) |
| C4 | C5 | 1.385(6) |
| C5 | C6 | 1.426(6) |
| C6 | C7 | 1.377(6) |
| C7 | C8 | 1.431(7) |
| C8 | C9 | 1.423(7) |
| C9 | C10 | 1.408(7) |

**Table S4**: Selected bond angles for **6m**$^{+\cdot}$.

| **Atom** | **Atom** | **Atom** | **Angle** / ° |
|---|---|---|---|
| C1 | N2 | C9 | 110.6(4) |
| C1 | C2 | C3 | 119.0(4) |
| C1 | N1 | C10 | 110.5(4) |
| C1 | N1 | C11 | 124.4(4) |
| C2 | C3 | C4 | 119.5(4) |
| C4 | C5 | C6 | 127.7(4) |
| C5 | N2 | C1 | 124.1(4) |
| C5 | N2 | C9 | 125.4(3) |
| C5 | C4 | C3 | 121.4(4) |
| C6 | C7 | C8 | 120.4(4) |
| C7 | C6 | C5 | 122.4(4) |
| C9 | C8 | C7 | 117.7(4) |
| C10 | N1 | C11 | 125.1(4) |
| C10 | C9 | C8 | 135.2(4) |
| N1 | C1 | N2 | 106.7(4) |
| N1 | C1 | C2 | 134.0(4) |
| N1 | C10 | C9 | 106.1(4) |
| N2 | C5 | C4 | 116.7(4) |
| N2 | C5 | C6 | 115.5(3) |
| N2 | C1 | C2 | 119.4(4) |
| N2 | C9 | C8 | 118.6(4) |
| N2 | C9 | C10 | 106.2(4) |

**Table S5:** Crystal data and structure refinement **2a-3c***.*

| **Compound** | **2a** | **2c** | **3c** |
|---|---|---|---|
| Internal reference | **NHO(Me)** | **NHO (Dipp)** | **Rh-Komplex** |
| CCDC number | 2388924 | 2388926 | 2388925 |
| Formula | $C_9H_{10}N_2$ | $C_{21}H_{26}N_2$ | $C_{23}H_{26}ClN_2O_2Rh$ |
| Formula weight | 146.19 | 306.44 | 500.82 |
| Temperature / K | 100.00 | 100.00 | 100.00 |
| Crystal system | orthorhombic | orthorhombic | orthorhombic |
| Space group | *Pbca* | $Pca2_1$ | *Pbca* |
| *a* / Å | 15.1557(15) | 15.701(3) | 15.718(10) |
| *b* / Å | 5.7857(6) | 8.296(2) | 14.874(11) |
| *c* / Å | 17.3450(19) | 26.929(6) | 19.523(14) |
| $\alpha$ / ° | 90 | 90 | 90 |
| $\beta$ / ° | 90 | 90 | 90 |
| $\gamma$ / ° | 90 | 90 | 90 |
| Volume / Å$^3$ | 1520.9(3) | 3507.8(14) | 4564(6) |
| *Z* | 8 | 8 | 8 |
| Density (calc.) g / cm$^3$ | 1.277 | 1.160 | 1.458 |
| Absorption coefficient / mm$^{-1}$ | 0.078 | 0.513 | 0.886 |
| *F*(000) | 624.0 | 1328.0 | 2048.0 |
| Crystal size / mm | 0.945 × 0.568 × 0.14 | 0.352 × 0.168 × 0.077 | 0.326 × 0.235 × 0.124 |
| Radiation | MoK$_\alpha$ ($\lambda$ = 0.71073) | CuK$_\alpha$ ($\lambda$ = 1.54178) | MoK$_\alpha$ ($\lambda$ = 0.71073) |
| 2$\theta$ range for data collection / ° | 4.696 to 65.43 | 6.564 to 141.968 | 4.172 to 59.994 |
| Index ranges | $-22 \le h \le 21$<br>$-8 \le k \le 8$<br>$-26 \le l \le 24$ | $-19 \le h \le 16$<br>$-10 \le k \le 9$<br>$-32 \le l \le 32$ | $-22 \le h \le 22$<br>$-20 \le k \le 20$<br>$-27 \le l \le 27$ |
| Reflections collected | 50880 | 36553 | 70332 |
| Independent reflections | 2773 [$R_{int}$ = 0.0524, $R_{sigma}$ = 0.0226] | 6751 [$R_{int}$ = 0.0586, $R_{sigma}$ = 0.0361] | 6655 [$R_{int}$ = 0.0490, $R_{sigma}$ = 0.0207] |
| Refinement method | Full-matrix least-squares on $F^2$ | | |
| Data / restraints / parameters | 2773/0/186 | 6751/1/425 | 6655/0/406 |
| Goodness-of-fit on $F^2$ | 1.218 | 1.051 | 1.086 |
| Final *R* indices [$I$>2$\sigma$(I)] | $R_1$ = 0.0570, w$R_2$ = 0.1397 | $R_1$ = 0.0873, w$R_2$ = 0.2335 | $R_1$ = 0.0452, w$R_2$ = 0.1129 |
| *R* indices (all data) | $R_1$ = 0.0709, w$R_2$ = 0.1464 | $R_1$ = 0.0967, w$R_2$ = 0.2462 | $R_1$ = 0.0518, w$R_2$ = 0.1192 |
| Largest diff. peak and hole/e. Å$^{-3}$ | 0.36/–0.23 | 1.02–0.32 | 4.06–0.80 |

**Table S6:** Crystal data and structure refinement **5b-Au-6m$^{+\cdot}$.**

| Compound | 5b-Au | 6b | 6m$^{+\cdot}$ |
|---|---|---|---|
| Internal reference | **Au-Komplex-TIPS** | **NHO-cyc-TIPS** | **NHO-cyc-Kation x SbF6** |
| CCDC number | 2424969 | 2388927 | 2388968 |
| Formula | $C_{20}H_{30}AuClN_2Si$ | $C_{20}H_{29}N_2Si$ | $C_{23}H_{25.5}F_6N_{2.5}Sb$ |
| Formula weight | 558.96 | 325.54 | 572.71 |
| Temperature / K | 100.0 | 100.00 | 101.00 |
| Crystal system | triclinic | triclinic | orthorhombic |
| Space group | *P*-1 | *P*-1 | $P2_12_12_1$ |
| *a* / Å | 7.9123(10) | 7.5271(4) | 6.7903(14) |
| *b* / Å | 16.636(2) | 9.2847(4) | 16.728(3) |
| *c* / Å | 18.742(3) | 13.8140(7) | 20.345(5) |
| $\alpha$ / ° | 83.265(4) | 90.343(2) | 90 |
| $\beta$ / ° | 81.600(4) | 104.124(2) | 90 |
| $\gamma$ / ° | 87.008(3) | 94.318(2) | 90 |
| Volume / Å$^3$ | 2422.1(6) | 933.28(8) | 2311.0(9) |
| *Z* | 4 | 2 | 4 |
| Density (calc.) g / cm$^3$ | 1.533 | 1.158 | 1.646 |
| Absorption coefficient / mm$^{-1}$ | 6.238 | 0.128 | 1.256 |
| *F*(000) | 1096.0 | 354.0 | 1144.0 |
| Crystal size / mm | 0.324 × 0.152 × 0.063 | 0.412 × 0.213 × 0.036 | 0.345 × 0.069 × 0.045 |
| Radiation | MoK$_\alpha$ ($\lambda$ = 0.71073) | MoK$_\alpha$ ($\lambda$ = 0.71073) | MoK$_\alpha$ ($\lambda$ = 0.71073) |
| 2$\theta$ range for data collection / ° | 4.422 to 53.998 | 5.598 to 56.65 | 4.004 to 59.17 |
| Index ranges | $-10 \leq h \leq 10$<br>$-21 \leq k \leq 21$<br>$-23 \leq l \leq 23$ | $-10 \leq h \leq 10$<br>$-12 \leq k \leq 12$<br>$-18 \leq l \leq 18$ | $-9 \leq h \leq 8$<br>$-22 \leq k \leq 21$<br>$-26 \leq l \leq 26$ |
| Reflections collected | 70685 | 30323 | 46803 |
| Independent reflections | 10527<br>[$R_{int}$ = 0.0819, $R_{sigma}$ = 0.0488] | 4637<br>[$R_{int}$ = 0.0624, $R_{sigma}$ = 0.0386] | 5835<br>[$R_{int}$ = 0.0819, $R_{sigma}$ = 0.0408] |
| Refinement method | Full-matrix least-squares on $F^2$ | | |
| Data / restraints / parameters | 10527/0/46 | 4637/0/324 | 5835/4/338 |
| Goodness-of-fit on $F^2$ | 1.025 | 1.076 | 1.036 |
| Final *R* indices [$I$>2$\sigma$(I)] | $R_1$ = 0.0440, w$R_2$ = 0.0904 | $R_1$ = 0.0426, w$R_2$ = 0.1115 | $R_1$ = 0.0362, w$R_2$ = 0.0748 |
| *R* indices (all data) | $R_1$ = 0.0670, w$R_2$ = 0.1008 | $R_1$ = 0.0475, w$R_2$ = 0.1159 | $R_1$ = 0.0458, w$R_2$ = 0.0802 |
| Largest diff. peak and hole/e. Å$^{-3}$ | 2.24/–2.04 | 0.62/–0.38 | 0.98/–0.49 |
| FLACK parameter[24] | / | / | –0.021(17) |

## 9. Computational Section

All DFT calculations were performed with the Gaussian16 program package14 (version g16, rev.C01)[10][11] or utilizing ORCA 6.0.0.[25][26] Solvent effects were considered implicitly with the SMD solvation model for THF. Gibbs free reaction energies and enthalpies were calculated for standard conditions (p = 1 atm, T = 298 K). Geometries of stationary points were fully optimized without any symmetry constraints. To test for minima or saddle points, harmonic vibrational frequency calculations were performed. The absence of imaginary frequencies confirmed a minimum, while a transition state was confirmed by one imaginary frequency. To check whether a transition state connects related minima, the intrinsic reaction coordinate (IRC) was calculated. For the visualization of computed structures and frontier molecular orbitals (isovalue = 0.6) IboView[17] was used. Natural Population Analyses (NPA) charge calculation and Wiberg Bond Index (WBI) calculation were conducted using NBO7.[27] ACID analysis was performed based on CSGT calculation performed in gaussian 16, utilizing ACID-3.0.4[28][29] for visualization. NICS inputs and 3D-NICS cube files were obtained using py.AROMA.[30] The cube file was visualized with VMD.[31] HOMA values were calculated using MultiWFN[32][33], based on the solid state structure of **6b** or DFT-optimized structures.

### 9.1 Mechanistic Investigation

Geometry optimization, frequency calculations and DLPNO-CCSD(T)[34][35][36][37] single point calculations were performed in ORCA 6.0.0:

## C5-Alkyne Cyclization

**5a-Ph-Alkin:**

!r2SCAN-3c TIGHTOPT FREQ VeryTightSCF defgrid3 SMD(THF)

FINAL SINGLE POINT ENERGY      -765.447699353653

Final Gibbs free energy         ...   -765.25624187 Eh

G-E(el)                         ...      0.21827799 Eh    136.97 kcal/mol

Total Charge           Charge          ....    0

Multiplicity           Mult            ....    1

| | | | |
|---|---|---|---|
| C | 2.56719994535180 | -2.90077421820077 | 0.00477342848524 |
| C | 1.21982097862144 | -2.44706199392698 | 0.00705376958079 |
| C | 0.90259036032171 | -1.10805154285857 | 0.00631139030343 |
| N | 1.94911530082745 | -0.17932837663903 | 0.00353986040183 |
| C | 3.30988005161341 | -0.61893572741669 | 0.00077420300515 |
| C | 3.59429399554826 | -1.99962317157878 | 0.00154573630511 |
| C | 1.91139126072360 | 1.21957312577435 | 0.00261521654099 |
| N | 3.24827877349406 | 1.59372484594862 | -0.00109365509313 |
| C | 4.08859942420493 | 0.49917355965060 | -0.00204832536931 |
| C | -0.44225631577266 | -0.70598741053308 | 0.00793389863667 |
| C | -1.64074243469976 | -0.49013439987186 | 0.00920240139282 |
| C | 0.85301984634767 | 2.08067269060624 | 0.00492507421154 |
| C | 3.66960436824980 | 2.97685380772511 | -0.00294568614625 |

| | | | |
|---|---|---|---|
| C | -3.01663496674486 | -0.18273183818009 | 0.01004119793376 |
| C | -3.44428030240193 | 1.16097887742296 | 0.00873874324581 |
| C | -4.79929706323023 | 1.45889460229109 | 0.00947504321030 |
| C | -5.74825499724709 | 0.43648718811406 | 0.01153115039746 |
| C | -5.33268455689687 | -0.89530706310582 | 0.01284839552513 |
| C | -3.98163827055537 | -1.21035489718703 | 0.01213462786804 |
| H | 2.76750530547581 | -3.96708429228431 | 0.00545672484573 |
| H | 0.40083955451961 | -3.15689853749463 | 0.00921704969779 |
| H | 4.63399953307800 | -2.31158371256839 | -0.00047211823808 |
| H | 5.16302098249934 | 0.59700255405111 | -0.00453895267456 |
| H | 1.04134668179001 | 3.14618759590721 | 0.00376134437535 |
| H | -0.16448404911754 | 1.72777733190569 | 0.00793221046568 |
| H | 3.29077504551167 | 3.49551146083230 | -0.89296457336118 |
| H | 3.29542433614412 | 3.49649566964082 | 0.88847715616382 |
| H | 4.76052292279016 | 3.01235955144561 | -0.00581216890514 |
| H | -2.70430052011295 | 1.95571384220976 | 0.00712666375409 |
| H | -5.11857881195667 | 2.49746677201400 | 0.00843384649168 |
| H | -6.80761795215253 | 0.67654164874479 | 0.01210043707244 |
| H | -6.06863759067167 | -1.69467367828411 | 0.01444997878721 |
| H | -3.65572683555272 | -2.24615926415417 | 0.01315393108976 |

!DLPNO-CCSD(T) DEF2-TZVPP DEF2-TZVPP/C TIGHTSCF TIGHTPNO

FINAL SINGLE POINT ENERGY -764.230284661570

**5a-Ph-6EndoTS:**

! r2SCAN-3c OptTS SlowConv FREQ VeryTightSCF defgrid3 SMD(THF)

FINAL SINGLE POINT ENERGY -765.452591781120

Final Gibbs free energy ... -765.23244177

G-E(el) ... 0.22015001 Eh 138.15 kcal/mol

Total Charge Charge .... 0

Multiplicity Mult .... 1

| | | | |
|---|---|---|---|
| C | 3.48951233097873 | -2.34920501557235 | -0.20162878283968 |
| C | 2.10713839660693 | -2.54337293026624 | 0.08191071331345 |
| C | 1.25105784274505 | -1.48244049036170 | 0.24209680420525 |
| N | 1.86032247280519 | -0.20372445684203 | 0.30402595463364 |
| C | 3.12582648462695 | 0.01734948569064 | -0.29431332559180 |
| C | 3.97653102601904 | -1.09558077998309 | -0.46937325114211 |
| C | 1.22604468828372 | 1.00582520710151 | 0.50995124983907 |
| N | 2.06538589655090 | 1.94812835947785 | -0.04385918584837 |
| C | 3.22277165346389 | 1.36734182312247 | -0.51115103451132 |
| C | -0.15563479734579 | -1.52489376431434 | 0.12705863687001 |
| C | -1.09708882947947 | -0.68593433019657 | 0.18238278169412 |
| C | 0.00531937527139 | 1.21824627589728 | 1.13083144257782 |
| C | 1.78613481170773 | 3.37284911510754 | -0.02955026525499 |
| C | -2.47920097361050 | -0.38133927536548 | -0.00062772240713 |
| C | -2.93361294188836 | 0.91403698020083 | -0.30546224780811 |
| C | -4.28655675730489 | 1.15920219861799 | -0.49888978323913 |
| C | -5.21817048938509 | 0.12899215157094 | -0.37717086242469 |
| C | -4.78051785666556 | -1.15867031169218 | -0.06746827517520 |
| C | -3.43009820507556 | -1.41853230429155 | 0.11716915093704 |

| | | | |
|---|---|---|---|
| H | 4.13092996697432 | -3.21679762917769 | -0.32007863472901 |
| H | 1.67706857132236 | -3.53900095185707 | 0.04342331117881 |
| H | 4.98671774841072 | -0.93675350796841 | -0.83291417336426 |
| H | 4.04757118378047 | 1.95873576384459 | -0.87901805777631 |
| H | -0.37875568412260 | 2.23293939778391 | 1.14186801187008 |
| H | -0.27003275045055 | 0.59147038615622 | 1.96709322400219 |
| H | 0.90173800325523 | 3.59857085451749 | -0.63566004212109 |
| H | 1.60853678137291 | 3.71161520072607 | 0.99760893544793 |
| H | 2.64620254982915 | 3.90251429401169 | -0.44229009610049 |
| H | -2.20932541547705 | 1.71733974714122 | -0.38965694980336 |
| H | -4.61765927812755 | 2.16555050255521 | -0.74124636240078 |
| H | -6.27670341188840 | 0.32793446825910 | -0.51869156133010 |
| H | -5.49836513618802 | -1.96871307022062 | 0.03202768055721 |
| H | -3.08913825699532 | -2.42169539367326 | 0.35505071674133 |

!DLPNO-CCSD(T) DEF2-TZVPP DEF2-TZVPP/C TIGHTSCF TIGHTPNO

FINAL SINGLE POINT ENERGY      -764.202041433056

**5a-Ph-5ExoTS:**

! r2SCAN-3c OptTS SlowConv FREQ VeryTightSCF defgrid3 SMD(THF)

FINAL SINGLE POINT ENERGY      -765.444791114830

Final Gibbs free energy         ...   -765.22611865

G-E(el)                           ...      0.21867246 Eh    137.22 kcal/mol

Total Charge           Charge          ....    0

Multiplicity Mult .... 1

| | | | |
|---|---|---|---|
| C | -2.63095155260008 | -2.80537750107362 | 0.01387052236936 |
| C | -1.26515272552759 | -2.39348317927783 | -0.03176439480247 |
| C | -0.95376486143914 | -1.06087599247457 | -0.12220009306056 |
| N | -2.02117860124581 | -0.19280447366546 | -0.19950474321672 |
| C | -3.35768511495567 | -0.52484530271752 | 0.04660651022409 |
| C | -3.66535474547504 | -1.90860699269574 | 0.08659936733498 |
| C | -1.83248304516742 | 1.15638261312611 | -0.21499230876409 |
| N | -3.05315845182864 | 1.68211782325266 | 0.06492894402329 |
| C | -4.00115049444178 | 0.67891944298719 | 0.22686662871476 |
| C | 0.33034653087160 | -0.40973022634688 | -0.06348157957194 |
| C | 1.55259601125287 | -0.70526035316292 | 0.01751713791175 |
| C | -0.52516599831139 | 1.65508967376333 | -0.40155761359318 |
| C | -3.32037896862842 | 3.10983582530223 | 0.16093370251384 |
| C | 2.90964178752589 | -0.32448511824776 | 0.05478060781319 |
| C | 3.54418830384147 | 0.20169130178661 | -1.09678518327954 |
| C | 4.88475940301316 | 0.55909208797488 | -1.06452665551952 |
| C | 5.63500212366450 | 0.41519694870524 | 0.10370201079632 |
| C | 5.02114603832034 | -0.10106648308730 | 1.24674248564931 |
| C | 3.68613289584886 | -0.47782180620426 | 1.22771468899690 |
| H | -2.84745351448166 | -3.86928839049771 | 0.03872686316081 |
| H | -0.46441327410636 | -3.12069809337223 | 0.03658535012230 |
| H | -4.69571308942617 | -2.22930754302167 | 0.19493997316415 |
| H | -5.03749339130695 | 0.90249289506279 | 0.42513210537170 |
| H | -0.26117673981549 | 2.58221217277459 | 0.10504983040446 |
| H | -0.10458587288149 | 1.55579042790934 | -1.39940558242093 |

| | | | |
|---|---|---|---|
| H | -3.11901063847475 | 3.47124643841206 | 1.17506325142601 |
| H | -2.67249289880246 | 3.63922996983062 | -0.54349970722573 |
| H | -4.36699413789841 | 3.29464396330248 | -0.08903336337820 |
| H | 2.96734904970619 | 0.31946423558711 | -2.01002515270568 |
| H | 5.34944815773870 | 0.96095107468137 | -1.96175867589476 |
| H | 6.68327829737692 | 0.69877860961417 | 0.12273787792262 |
| H | 5.59359130612927 | -0.21810200028585 | 2.16372248168572 |
| H | 3.22070721152498 | -0.89001704794143 | 2.11878571382776 |

!DLPNO-CCSD(T) DEF2-TZVPP DEF2-TZVPP/C TIGHTSCF TIGHTPNO

FINAL SINGLE POINT ENERGY -764.187156847254

**5a-Ph-6EndoCarbene:**

!r2SCAN-3c TIGHTOPT FREQ VeryTightSCF defgrid3 SMD(THF)

FINAL SINGLE POINT ENERGY -765.503389974786

Final Gibbs free energy ... -765.27902320 Eh

G-E(el) ... 0.22436677 Eh 140.79 kcal/mol

Total Charge Charge .... 0

Multiplicity Mult .... 1

| | | | |
|---|---|---|---|
| C | 3.37914652833621 | -2.44338790330174 | 0.06250559612942 |
| C | 1.96263873399838 | -2.55493905147821 | 0.09550039201632 |
| C | 1.12995444766228 | -1.45587418809201 | 0.06951353177102 |
| N | 1.82326990182621 | -0.22765456591820 | 0.01987640340330 |

| | | | |
|---|---|---|---|
| C | 3.21491581860262 | -0.06302047947551 | -0.02655336703973 |
| C | 4.01434985562658 | -1.23077059830309 | -0.00315042905855 |
| C | 1.21800380378044 | 0.96744606281837 | 0.00025657537837 |
| N | 2.18260543110826 | 1.89814843501426 | -0.06569916483865 |
| C | 3.42542972026511 | 1.29884721438549 | -0.08050489474253 |
| C | -0.30711494881187 | -1.46335913924567 | 0.06961402863808 |
| C | -0.94055380507379 | -0.25084611535806 | 0.05472600261989 |
| C | 1.93925246578044 | 3.33895865163734 | -0.07167319782717 |
| C | -2.41906304738391 | -0.16058175212152 | 0.02909759409611 |
| C | -3.10056378600732 | 1.06589172301438 | 0.15487691641078 |
| C | -4.49297584700041 | 1.12770483810179 | 0.13326074949903 |
| C | -5.24981186537804 | -0.02855754428168 | -0.01963416049136 |
| C | -4.59208735905655 | -1.25432868412068 | -0.15111276416672 |
| C | -3.20795350692833 | -1.31809475299716 | -0.12651581879632 |
| H | 3.97217032340348 | -3.35313776850371 | 0.08416705389450 |
| H | 1.49415014348909 | -3.53255778044139 | 0.13404809286884 |
| H | 5.09431997987826 | -1.14066762329396 | -0.03510893516848 |
| H | 4.33552772122052 | 1.87573223877072 | -0.12948359819757 |
| H | 1.14718911882535 | 3.57508187733540 | -0.78665635938303 |
| H | 1.64107203370095 | 3.67214573235037 | 0.92713464812852 |
| H | 2.85924117266993 | 3.84513797674022 | -0.36652644256824 |
| H | -2.55329664707336 | 1.99442154113026 | 0.27926571341509 |
| H | -4.98569961819805 | 2.09127428855699 | 0.23751764336412 |
| H | -6.33522018179896 | 0.02050457975796 | -0.03901845714736 |
| H | -5.16813953581075 | -2.16807500018053 | -0.27651952283694 |
| H | -2.69755098250566 | -2.27015225990855 | -0.22915925007756 |

| | | | |
|---|---|---|---|
| C | -0.24586633989165 | 1.12470124292556 | 0.06680462446033 |
| H | -0.51078414602473 | 1.69171940613100 | 0.97425974054049 |
| H | -0.58851758323072 | 1.74605939835158 | -0.77565944429402 |

!DLPNO-CCSD(T) DEF2-TZVPP DEF2-TZVPP/C TIGHTSCF TIGHTPNO

FINAL SINGLE POINT ENERGY      -764.236299527937

**6a**

!r2SCAN-3c TIGHTOPT FREQ VeryTightSCF defgrid3 SMD(THF)

FINAL SINGLE POINT ENERGY      -765.568659301305

Final Gibbs free energy         ...   -765.34452288 Eh

G-E(el)                           ...      0.22413642 Eh    140.65 kcal/mol

Total Charge           Charge          ....    0

Multiplicity           Mult            ....    1

| | | | |
|---|---|---|---|
| C | 3.38377491263701 | -2.40552490841609 | -0.18350503561329 |
| C | 1.97444822005248 | -2.54995336702006 | -0.19810266706536 |
| C | 1.15617951025336 | -1.42344752144719 | -0.11245341253022 |
| N | 1.80766820030733 | -0.20262560398061 | -0.01713553616612 |
| C | 3.20280308993358 | -0.01613065353496 | 0.00759013931012 |
| C | 4.01498986385414 | -1.19172878424675 | -0.08379466577179 |
| C | 1.16863977938503 | 1.00498253363662 | 0.06611112804663 |
| N | 2.13175856407793 | 1.94792248345690 | 0.13771167025644 |
| C | 3.39923398297916 | 1.33558212986264 | 0.11273670860773 |
| C | -0.25859815722348 | -1.35152713611634 | -0.11304826703965 |

| | | | |
|---|---|---|---|
| C | -0.92950659371288 | -0.12953607152708 | -0.01783113277922 |
| C | -0.22845348078477 | 1.08561909862503 | 0.07445055733504 |
| C | 1.87233304711765 | 3.36894835523425 | 0.27680651849196 |
| C | -2.41058302848488 | -0.10849642535381 | -0.01386076773013 |
| C | -3.11334618215314 | 0.92767535567752 | -0.64509494328929 |
| C | -4.50399045714144 | 0.94607481988403 | -0.64496136460736 |
| C | -5.22005641546140 | -0.06573696244475 | -0.00921204701354 |
| C | -4.53265156796018 | -1.09828999973504 | 0.62503539586013 |
| C | -3.14218441794364 | -1.12199801442267 | 0.62134119644865 |
| H | 3.99125856595929 | -3.30473174963945 | -0.25464281697577 |
| H | 1.51180833677650 | -3.52668895313343 | -0.27515971709592 |
| H | 5.09428328052995 | -1.09616731450833 | -0.07660950801631 |
| H | 4.30644956112267 | 1.91585627060375 | 0.15831053879482 |
| H | -0.80852107171119 | -2.27910182908590 | -0.22181181820095 |
| H | -0.72486322656744 | 2.03878349411651 | 0.19960919927886 |
| H | 1.16941373863569 | 3.70326454421366 | -0.49400007162589 |
| H | 1.45444262227403 | 3.59725898757054 | 1.26490709718786 |
| H | 2.81250697227126 | 3.90971959480958 | 0.15483114784292 |
| H | -2.56566322296101 | 1.71099892678393 | -1.16183485604089 |
| H | -5.03072137569380 | 1.75170875370698 | -1.14949225975410 |
| H | -6.30652373184601 | -0.04946697576872 | -0.00749795690528 |
| H | -5.08182118944833 | -1.88731747165496 | 1.13185364373988 |
| H | -2.61714712907351 | -1.92083860614580 | 1.13756890302004 |

!DLPNO-CCSD(T) DEF2-TZVPP DEF2-TZVPP/C TIGHTSCF TIGHTPNO

FINAL SINGLE POINT ENERGY      -764.315979642310

**Table S7: Summary of results.**

| | ALKIN | 6ENDO-TS | 5EXOTS | 6ENDO-CARBEN | PH-CYCL |
|---|---|---|---|---|---|
| G(R2SCAN-3C) | -765.256242 | -765.232442 | -765.226119 | -765.2790232 | -765.344523 |
| G-E (R2SCAN-3C) | 0.21827799 | 0.22015001 | 0.21867246 | 0.22436677 | 0.22413642 |
| E (DLPNO) | -764.230285 | -764.202041 | -764.187157 | -764.2362995 | -764.31598 |
| G (FINAL) | -764.012007 | -763.981891 | -763.968484 | -764.0119328 | -764.091843 |
| δG (R2-SCAN-3C) | 0 | 14.9347882 | 18.9026059 | -14.29550041 | -55.3971701 |
| δG (FINAL) | 0 | 18.8976038 | 27.3106457 | 0.046381505 | -50.098192 |

## Alkene Cyclization

**5o-trans**

!r2SCAN-3c TIGHTOPT FREQ VeryTightSCF defgrid3 SMD(THF)

FINAL SINGLE POINT ENERGY -766.721603639935

Final Gibbs free energy ... -766.47925489 Eh

G-E(el) ... 0.24234875 Eh 152.08 kcal/mol

Total Charge Charge .... 0

Multiplicity Mult .... 1

| | | | |
|---|---|---|---|
| C | 2.20494787183353 | 2.93254597423490 | -0.18977001916336 |
| C | 0.92078431772393 | 2.31184843568876 | -0.14922016848124 |
| C | 0.77097908119879 | 0.94983839403225 | -0.09478845867893 |
| N | 1.92775347593556 | 0.16883746837486 | -0.02853665082498 |
| C | 3.22478424094053 | 0.76239897198423 | -0.14479799874016 |
| C | 3.33466158131645 | 2.17054748245094 | -0.22062304582755 |
| C | 2.07100895102163 | -1.21227523489762 | 0.17664503001801 |
| N | 3.43523910700271 | -1.43315597774119 | 0.06468509959528 |
| C | 4.13411164112598 | -0.24786581012438 | -0.09228330930249 |
| C | -0.52229531878198 | 0.30365761165948 | -0.22962978838195 |
| C | -1.66981004444205 | 0.81080833274287 | 0.26585118161150 |
| C | 1.14878036377260 | -2.17823677145465 | 0.47179841487195 |
| C | 4.03203981483372 | -2.73461107994751 | 0.26159875763279 |
| C | -3.00495615040217 | 0.25760161805545 | 0.08393456952251 |
| C | -4.08562973280864 | 0.88794933363968 | 0.72647906540203 |

| | | | |
|---|---|---|---|
| C | -5.38100593310185 | 0.40051987935865 | 0.59880064141590 |
| C | -5.62935922395565 | -0.73101052502596 | -0.17429474189002 |
| C | -4.56745861237560 | -1.36769076046383 | -0.81988409861642 |
| C | -3.27349581064332 | -0.88249125695069 | -0.69730459767802 |
| H | 2.26915173180555 | 4.01452386932444 | -0.24771455143529 |
| H | 0.02801301943385 | 2.91681053556618 | -0.25751729013192 |
| H | 4.32534139810457 | 2.60771648156896 | -0.29703391489573 |
| H | 5.21184365083799 | -0.21692609724579 | -0.12975153488111 |
| H | -0.53377064903616 | -0.62039081687250 | -0.79994220679497 |
| H | -1.61926624200945 | 1.70798953067385 | 0.88281798570682 |
| H | 1.49241356152345 | -3.18888898431448 | 0.64948324133881 |
| H | 0.10248150337510 | -1.96192638753267 | 0.61361450504646 |
| H | 3.62444078223463 | -3.45868346654110 | -0.45459992876889 |
| H | 5.11017533073317 | -2.65414994928144 | 0.11042857536797 |
| H | 3.83877090206138 | -3.10468186820599 | 1.27800737888524 |
| H | -3.89701568290899 | 1.77093475699768 | 1.33291063022828 |
| H | -6.19923685830440 | 0.90569574055079 | 1.10503046792182 |
| H | -6.64049606441320 | -1.11509016930223 | -0.27627873271280 |
| H | -4.75343582334942 | -2.24957682903091 | -1.42728253022125 |
| H | -2.46685118028222 | -1.39366143197099 | -1.21518997713826 |

!DLPNO-CCSD(T) DEF2-TZVPP DEF2-TZVPP/C TIGHTSCF TIGHTPNO

FINAL SINGLE POINT ENERGY -765.478111180346

**5o-6Endo-TS**

! r2SCAN-3c OptTS SlowConv FREQ VeryTightSCF defgrid3 SMD(THF)

FINAL SINGLE POINT ENERGY -766.704333713278

Final Gibbs free energy ... -766.46065069

G-E(el) ... 0.24368302 Eh 152.91 kcal/mol

Total Charge Charge .... 0

Multiplicity Mult .... 1

| | | | |
|---|---|---|---|
| C | -3.36593196894450 | -2.41534225451829 | 0.16998094304982 |
| C | -1.98734580636495 | -2.55791036454504 | -0.06230122577320 |
| C | -1.12995607014352 | -1.46061607530263 | -0.08451497258351 |
| N | -1.79340607134687 | -0.20802495643750 | -0.11645338554356 |
| C | -3.13891992716033 | -0.03933133775961 | 0.26275517665725 |
| C | -3.94490034219642 | -1.17665891096443 | 0.40471531966340 |
| C | -1.23550649559361 | 1.01264811500867 | -0.44580163220640 |
| N | -2.19176470822070 | 1.93095359462639 | -0.12085762175462 |
| C | -3.35086855894449 | 1.32637685099678 | 0.29539881422751 |
| C | 0.27279134919026 | -1.45457590148967 | -0.03304291233997 |
| C | 0.98250154542427 | -0.30970341910508 | 0.39167508169188 |
| C | 0.06275752274933 | 1.23665240578983 | -0.92672594369172 |
| C | -2.03039970905828 | 3.36729382530857 | -0.30754720684172 |
| C | 2.43578504036415 | -0.21401414726625 | 0.25848162023539 |
| C | 3.17264239536192 | 0.57820526535680 | 1.15665823489223 |
| C | 4.55196164280091 | 0.69740319133988 | 1.04266384414367 |
| C | 5.23291140488911 | 0.03365621252605 | 0.02178918988327 |
| C | 4.51473397442848 | -0.74559901707822 | -0.88484214561895 |

| | | | |
|---|---|---|---|
| C | 3.13420246272771 | -0.86581577101364 | -0.77316694570173 |
| H | -3.97901605445547 | -3.30980126035265 | 0.23523732156408 |
| H | -1.54354486178449 | -3.54866418507049 | -0.10280347004692 |
| H | -4.98837868235739 | -1.06703518805693 | 0.67789408267296 |
| H | -4.24764561488490 | 1.89116192938456 | 0.49734591943051 |
| H | 0.79853353258061 | -2.32594175806166 | -0.41383988765582 |
| H | 0.57885419117228 | 0.25500830211103 | 1.22767719236812 |
| H | 0.39969717352601 | 2.27129123848075 | -0.93936749076331 |
| H | 0.42860650363341 | 0.62150250397324 | -1.73891189016323 |
| H | -1.79679295933532 | 3.58577427592983 | -1.35406619282567 |
| H | -2.96391223900141 | 3.85957970356240 | -0.03115711783746 |
| H | -1.22295845568398 | 3.74387492569256 | 0.32774683256264 |
| H | 2.64719096493511 | 1.09980904117640 | 1.95404360899664 |
| H | 5.10071132291655 | 1.31037790749839 | 1.75320648480517 |
| H | 6.31144335345905 | 0.12793537218385 | -0.06999125607517 |
| H | 5.03510054212605 | -1.25829826783120 | -1.69000824135168 |
| H | 2.58661460319141 | -1.46009984609269 | -1.50013212806991 |

!DLPNO-CCSD(T) DEF2-TZVPP DEF2-TZVPP/C TIGHTSCF TIGHTPNO

FINAL SINGLE POINT ENERGY      -765.449040594308

**5o-5Exo-TS**

! r2SCAN-3c OptTS SlowConv FREQ VeryTightSCF defgrid3 SMD(THF)

FINAL SINGLE POINT ENERGY      -766.683071269044

Final Gibbs free energy         ...   -766.44114447 Eh

G-E(el) ... 0.24192680 Eh 151.81 kcal/mol

Total Charge Charge .... 0

Multiplicity Mult .... 1

| | | | |
|---|---|---|---|
| C | 2.34427612274676 | 2.97890934935423 | -0.39408702835684 |
| C | 1.02086491157228 | 2.42740408057553 | -0.34129833409889 |
| C | 0.85110058283912 | 1.08488320448262 | -0.18125738483261 |
| N | 2.00293396691773 | 0.35246161837336 | -0.02298438973572 |
| C | 3.31229940462817 | 0.80171144050178 | -0.17204523309794 |
| C | 3.47069992257667 | 2.20316305683617 | -0.34523541468056 |
| C | 1.94186013431853 | -0.97851623542378 | 0.21829560530996 |
| N | 3.22668240640753 | -1.39953297226011 | 0.16549336635966 |
| C | 4.08748200019560 | -0.33603460524743 | -0.07478116252908 |
| C | -0.36567394334551 | 0.22948757871302 | -0.24089618239174 |
| C | -1.52675708603511 | 0.67299862799670 | 0.40291297485853 |
| C | 0.63154377910939 | -1.52099262790004 | 0.44921813399513 |
| C | 3.64774046115802 | -2.78169046070922 | 0.35719140516396 |
| C | -2.82452046970231 | 0.10917281289373 | 0.19354715983888 |
| C | -3.95331283430274 | 0.62157466628933 | 0.88956872412267 |
| C | -5.22631033030207 | 0.10953708749160 | 0.69741297729420 |
| C | -5.44931829505251 | -0.94733300545162 | -0.19294635265181 |
| C | -4.35418639005487 | -1.48182464074353 | -0.87522433630856 |
| C | -3.07274806543525 | -0.97938614282258 | -0.68706647362349 |
| H | 2.44760400720036 | 4.05380182545567 | -0.50952150894072 |
| H | 0.15369085355526 | 3.06383829346908 | -0.47712881625628 |
| H | 4.46500807349495 | 2.62951137901423 | -0.42094417099822 |

| | | | |
|---|---|---|---|
| H | 5.15551598216858 | -0.47301693726157 | -0.14086396964913 |
| H | -0.48755842381216 | -0.21429846809459 | -1.23203131717585 |
| H | -1.41555159282999 | 1.35995905313525 | 1.24151718305161 |
| H | 0.39224081550773 | -2.45709180429353 | -0.05639298169661 |
| H | 0.30387252734626 | -1.50111571939791 | 1.48929882589288 |
| H | 3.84138851483183 | -3.25611543537784 | -0.60999702135989 |
| H | 4.55659646063227 | -2.80132886609224 | 0.96290971120466 |
| H | 2.84680612952201 | -3.31923089539433 | 0.87095524678253 |
| H | -3.80545672192823 | 1.44524366204006 | 1.58610472516685 |
| H | -6.06111411276602 | 0.53746886820732 | 1.24888615055492 |
| H | -6.44757166574381 | -1.34888525686840 | -0.34128164447974 |
| H | -4.50028391300714 | -2.31499943683877 | -1.56007592328068 |
| H | -2.24620221241135 | -1.44082209465221 | -1.22161054345209 |

!DLPNO-CCSD(T) DEF2-TZVPP DEF2-TZVPP/C TIGHTSCF TIGHTPNO

FINAL SINGLE POINT ENERGY      -765.422227177775

**5o Endo6-Carbene**

!r2SCAN-3c TIGHTOPT FREQ VeryTightSCF defgrid3 SMD(THF)

FINAL SINGLE POINT ENERGY      -766.747264965281

Final Gibbs free energy         ...   -766.50020771

G-E(el)                          ...      0.24705725 Eh    155.03 kcal/mol

Total Charge           Charge          ....    0

Multiplicity Mult .... 1

| | | | |
|---|---|---|---|
| C | 3.35779918510720 | -2.30305399490119 | 0.68522977376344 |
| C | 1.97807084631003 | -2.46410148648628 | 0.61657552594390 |
| C | 1.11919112814137 | -1.40195986613763 | 0.21294001298779 |
| N | 1.81734207737163 | -0.18474626489378 | -0.01231932633177 |
| C | 3.20922828361378 | -0.00338861651904 | 0.05851658740470 |
| C | 4.01621780511271 | -1.10478770398068 | 0.39598482375352 |
| C | 1.20237132803978 | 0.98370426614525 | -0.29712304263287 |
| N | 2.15858029152824 | 1.90606291812957 | -0.43983996218002 |
| C | 3.40424501905272 | 1.34100847625971 | -0.22774168852599 |
| C | -0.23382166761293 | -1.38926473721718 | 0.00294793442485 |
| C | -0.94315846888339 | -0.22151557309315 | -0.63902446411120 |
| C | -0.26935901043382 | 1.12787639712302 | -0.26583033533514 |
| C | 1.92272475913026 | 3.32552674628134 | -0.68708485795842 |
| C | -2.41574832904578 | -0.16487830257096 | -0.28211517765369 |
| C | -3.38684325599905 | -0.03479818616708 | -1.27623486901427 |
| C | -4.74033026493338 | 0.03622137424701 | -0.94678386843539 |
| C | -5.13818802127266 | -0.01862378774071 | 0.38579030101250 |
| C | -4.17480143595261 | -0.14638786033978 | 1.38666693433970 |
| C | -2.82597535748113 | -0.22171756323030 | 1.05396161561112 |
| H | 3.95685665377491 | -3.16486503181942 | 0.97227378065653 |
| H | 1.52414185902725 | -3.42645319033270 | 0.82986257996795 |
| H | 5.09282221723297 | -1.00005393140620 | 0.44411704416522 |
| H | 4.30860633892247 | 1.92412982890115 | -0.29633274734473 |
| H | -0.78409894583809 | -2.31501871757995 | 0.13971370635333 |
| H | -0.88734951148151 | -0.29852050991247 | -1.73863132278497 |

| | | | |
|---|---|---|---|
| H | -0.59957892274506 | 1.92485792188222 | -0.94094193306633 |
| H | 1.61054622058894 | 3.82268811239389 | 0.23685475682609 |
| H | 2.85082675349914 | 3.77324603794629 | -1.04556968436618 |
| H | 1.14699446551997 | 3.44684114097748 | -1.44660817090798 |
| H | -3.08050845205838 | 0.00635748080528 | -2.31968457694438 |
| H | -5.48369544432942 | 0.13050030443208 | -1.73425109864165 |
| H | -6.19225803316100 | 0.03411817571208 | 0.64510448143467 |
| H | -4.47750466449691 | -0.19184413934410 | 2.42976887061411 |
| H | -2.07722185563749 | -0.33524207445079 | 1.83541119323296 |
| H | -0.57776669061079 | 1.41572093688703 | 0.75066275374259 |

!DLPNO-CCSD(T) DEF2-TZVPP DEF2-TZVPP/C TIGHTSCF TIGHTPNO

FINAL SINGLE POINT ENERGY -765.495399500837

**5o Endo6-Cycl**

!r2SCAN-3c TIGHTOPT FREQ VeryTightSCF defgrid3 SMD(THF)

FINAL SINGLE POINT ENERGY -766.749473464330

Final Gibbs free energy ... -766.50329182

G-E(el) ... 0.24618164 Eh 154.48 kcal/mol

| | | | |
|---|---|---|---|
| C | 3.40093119513074 | -2.39621839293897 | 0.25457134688377 |
| C | 1.96895999329258 | -2.53276644111894 | 0.22760774998887 |
| C | 1.18224346374257 | -1.42670162205168 | 0.12117228799453 |
| N | 1.80626755187514 | -0.21202721668861 | -0.01591724920851 |

| | | | |
|---|---|---|---|
| C | 3.20604985591935 | -0.00718897857417 | 0.07739577767706 |
| C | 4.00801577825246 | -1.17514788531466 | 0.20298752467891 |
| C | 1.14387353246885 | 1.00276750448908 | -0.16198044746304 |
| N | 2.14283598783375 | 1.94967585856392 | -0.10863043157040 |
| C | 3.40014027708298 | 1.34174077831926 | 0.01395769641616 |
| C | -0.31055631175762 | -1.35764114051118 | 0.24688131374194 |
| C | -0.94724350208535 | -0.20953400264195 | -0.58332904022783 |
| C | -0.20219264949780 | 1.08915118609075 | -0.35880806265772 |
| C | 1.89190811876789 | 3.35770557671814 | -0.31676215757309 |
| C | -2.42136371911372 | -0.10794110345636 | -0.24769232256848 |
| C | -3.38832855637577 | -0.58356988294006 | -1.13580946146113 |
| C | -4.74463324423857 | -0.51836691531569 | -0.81819053977296 |
| C | -5.15185979114906 | 0.02266455268645 | 0.39794944499083 |
| C | -4.19378709648461 | 0.49849106961131 | 1.29314900662534 |
| C | -2.84157865754995 | 0.43573865158204 | 0.97082962777373 |
| H | 4.00802923935490 | -3.29238419703686 | 0.34332695958429 |
| H | 1.50588170911714 | -3.50791828469593 | 0.33136499372583 |
| H | 5.08708385869890 | -1.07182337751577 | 0.25676116509474 |
| H | 4.30853814776359 | 1.92275185883850 | 0.03913058590576 |
| H | -0.54427752410125 | -1.17794208044502 | 1.30731549362714 |
| H | -0.74828336933099 | -2.32380057831018 | -0.02315496797071 |
| H | -0.88203978440474 | -0.51475703783413 | -1.64213737247106 |
| H | -0.68442373261918 | 2.04219738075563 | -0.53945258382556 |
| H | 1.17637268348060 | 3.73560353474857 | 0.42444390199117 |
| H | 2.82929901380271 | 3.90795855792328 | -0.21305997280967 |
| H | 1.48013937681591 | 3.53844373149689 | -1.31966627321041 |

| | | | |
|---|---|---|---|
| H | -3.07543042074120 | -1.00654032427255 | -2.08850236656781 |
| H | -5.48316574352443 | -0.88793774992604 | -1.52503306698189 |
| H | -6.20829831043266 | 0.07677838222265 | 0.64721969543048 |
| H | -4.50302291860853 | 0.92331497961838 | 2.24489526896294 |
| H | -2.09750445138464 | 0.81793060792388 | 1.66650847524679 |

!DLPNO-CCSD(T) DEF2-TZVPP DEF2-TZVPP/C TIGHTSCF TIGHTPNO#

FINAL SINGLE POINT ENERGY -765.510764990949

**Table S8: Summary of results.**

| | ALKEN-TRANS | 6ENDO-TS | 5EXOTS | 6ENDO-CARBEN | PH-CYCL |
|---|---|---|---|---|---|
| G(R2SCAN-3C) | -766.479255 | -766.460651 | -766.441144 | -766.500208 | -766.503292 |
| G-E (R2SCAN-3C) | 0.24234875 | 0.24368302 | 0.2419268 | 0.24705725 | 0.24618164 |
| E (DLPNO) | -765.478111 | -765.449041 | -765.422227 | -765.4954 | -765.510765 |
| G (FINAL) | -765.235762 | -765.205358 | -765.1803 | -765.248342 | -765.264583 |
| δG (R2-SCAN-3C) | 0 | 11.6743118 | 23.9146496 | -13.1480931 | -15.0834013 |
| δG (FINAL) | 0 | 19.0793352 | 34.8029634 | -7.89395654 | -18.0854007 |

## 9.2 Determination of NICS Values and GIAO Calculations

NICS values were calculated on r2SCAN-3c[38] (SMD:THF)[37] optimized structures or the crystal structure of **6b** utilizing the GIAO method as implemented in Gaussian 16. The position of Bq atoms was determined with py.Aroma at the hight indicated, as well as the data evaluation to obtain the NICSzz values above and below the center of cyclic subunits at the height indicated, NICSzz scan and a gaussian type cube file of the NICS-3D-SCAN. A conversion to ICSS (iso-chemical shielding surface) was NOT performed. All NICS values were determined with a range separated functional at cam-b3lyp/6-311+g(2d,p) level of theory to account for long range interactions.

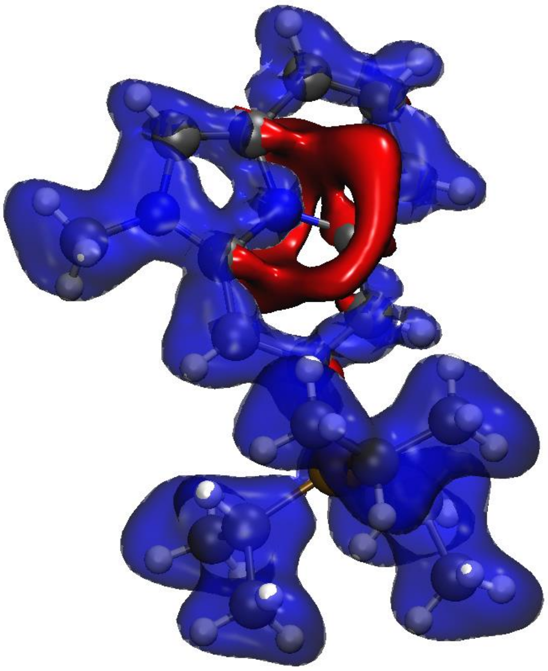

**Figure S452**: 3D-NICS surface of **6b**, based on the solid state structure. Isosurface values red : 5 ; blue : –15.

For comparison with experimental data also GIAO calculations at b3lyp/6-311+g(2d,p) level were evaluated.

**Table S9**: Comparison of experimentally determined and calculated $^{13}C$ NMR shifts of selected entries.

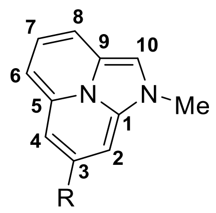


| $C_{xx}$ | R=H exp. | R=H calc. |
|---|---|---|
| 2 | 79.1 | 82.7 |
| 6 | 87.1 | 91.7 |
| 10 | 99.3 | 97.7 |
| 8 | 99.7 | 98.9 |
| 4 | 99.3 | 102.6 |
| 7 | 135.4 | 142.5 |
| 3 | 140.3 | 146.3 |
| 9 | 137.2 | 146.9 |
| 1 | 150.3 | 155.1 |
| 5 | 153.3 | 160.8 |

| $C_{xx}$ | R=Ph exp. | R=Ph calc. |
|---|---|---|
| 2 | 78.7 | 82.7 |
| 6 | 87.7 | 92.4 |
| 10 | 99.7 | 98.3 |
| 8 | 99.7 | 98.9 |
| 4 | 98.5 | 103.1 |
| 7 | 135.2 | 142.3 |
| 9 | 136.6 | 146.4 |
| 1 | 150.3 | 155.2 |
| 5 | 152.9 | 160.4 |
| 3 | 153.3 | 162.2 |

### 9.3 ACID plots

ACID analysis was performed utilizing ACID 3.0.4, based on CSGT calculations performed in gaussian 16 at the cam-b3lyp/6-311+g(2d,p) or def2-TZVP level of theory. In case of **6a**(Ph) also a $\pi$-only plot was generated, selecting the respective orbitals by iop(10/93=2) (list of selected orbitals: 45, 50, 58, 59, 60, 61, 62, 63, 64, 65. The maximum arrow length was set to 1 and the iso-surface (value as indicated) was smoothed.

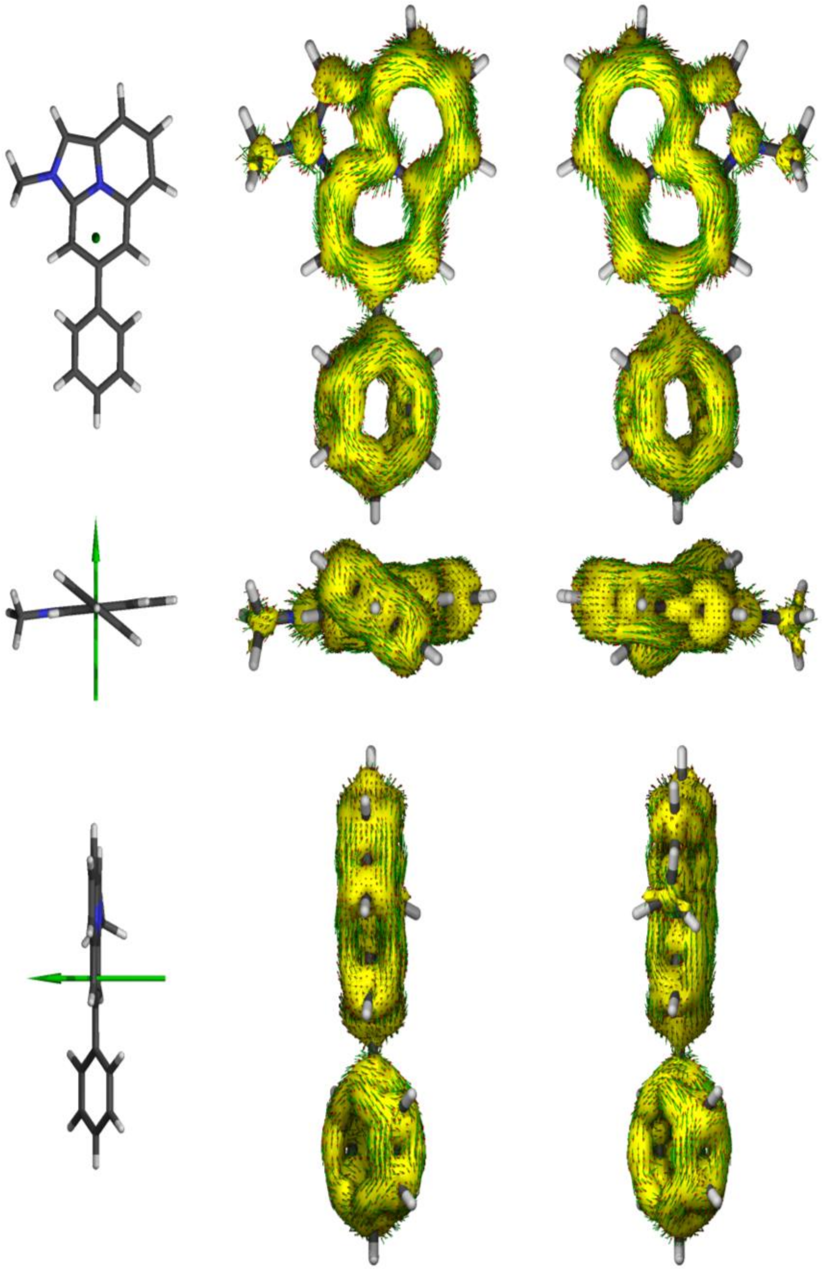

**Figure S453**: ACID plot of **6a**, considering only -orbitals in all dimensions, green arrow indicates direction of magnetic field, isovalue of 0.0035.

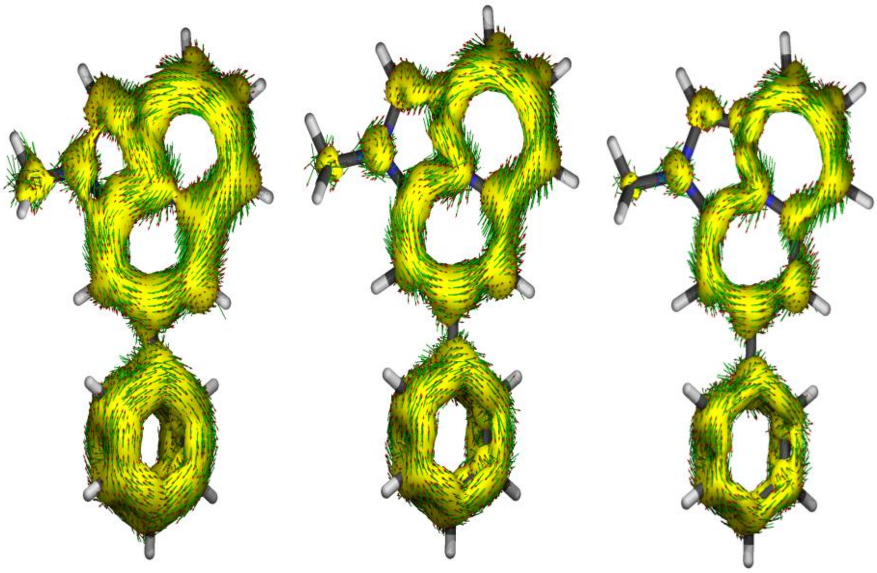

**Figure S454**: ACID plot of **6a**, considering only p-orbitals in all dimensions, surface at isovalues of 0.03, 0.04 and 0.05.

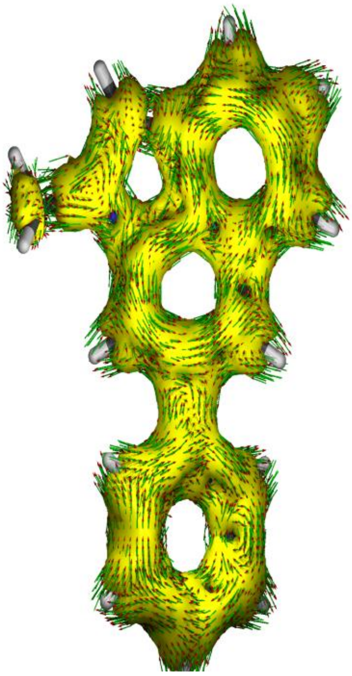

**Figure S455**: ACID plot of **6a**, considering all orbitals (isovalue: 0.045).

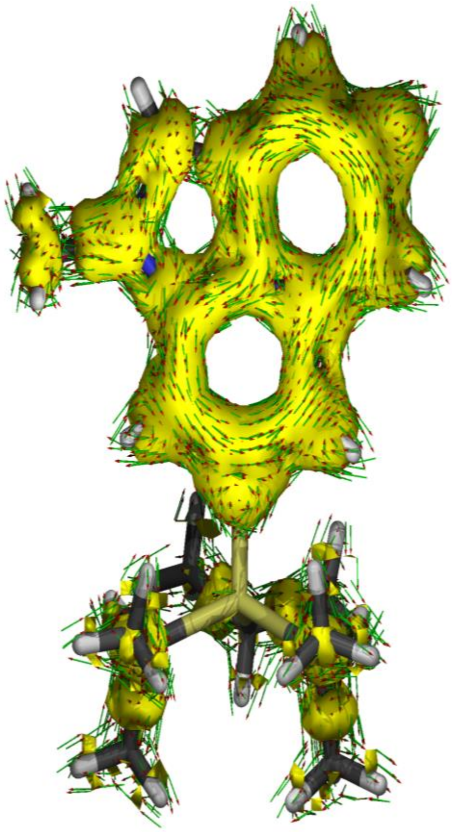

**Figure S456**: ACID plot of **6b**, bases on the solid state structure, considering all orbitals (isovalue: 0.045).

### 9.4 NBO / NRT calculations

NBO calculations were performed utilizing NBO 7.0 as interfaced with Orca 6.0.0 at b3lp/def2-tzvp level using the NBOKEYLIST as specified below.

NRTPAR=50 NRTE2=2

TOPO matrix for the leading resonance structure:

| Atom | 1 | 2 | 3 | 4 | 5 | 6 | 7 | 8 | 9 | 10 | 11 | 12 | 13 | 14 | 15 | 16 | 17 |
|---|---|---|---|---|---|---|---|---|---|---|---|---|---|---|---|---|---|
| 1. C | 0 | 1 | 0 | 0 | 0 | 2 | 0 | 0 | 0 | 0 | 0 | 0 | 0 | 0 | 1 | 0 | 0 |
| 2. C | 1 | 0 | 2 | 0 | 0 | 0 | 0 | 0 | 0 | 0 | 0 | 0 | 0 | 0 | 0 | 1 | 0 |
| 3. C | 0 | 2 | 0 | 1 | 0 | 0 | 0 | 0 | 0 | 1 | 0 | 0 | 0 | 0 | 0 | 0 | 0 |
| 4. N | 0 | 0 | 1 | 1 | 1 | 0 | 1 | 0 | 0 | 0 | 0 | 0 | 0 | 0 | 0 | 0 | 0 |
| 5. C | 0 | 0 | 0 | 1 | 0 | 1 | 0 | 0 | 2 | 0 | 0 | 0 | 0 | 0 | 0 | 0 | 0 |
| 6. C | 2 | 0 | 0 | 0 | 1 | 0 | 0 | 0 | 0 | 0 | 0 | 0 | 0 | 0 | 0 | 0 | 1 |
| 7. C | 0 | 0 | 0 | 1 | 0 | 0 | 0 | 2 | 0 | 0 | 0 | 1 | 0 | 0 | 0 | 0 | 0 |
| 8. N | 0 | 0 | 0 | 0 | 0 | 0 | 2 | 0 | 1 | 0 | 0 | 0 | 1 | 0 | 0 | 0 | 0 |
| 9. C | 0 | 0 | 0 | 0 | 2 | 0 | 0 | 1 | 0 | 0 | 0 | 0 | 0 | 0 | 0 | 0 | 0 |
| 10. C | 0 | 0 | 1 | 0 | 0 | 0 | 0 | 0 | 0 | 0 | 2 | 0 | 0 | 0 | 0 | 0 | 0 |
| 11. C | 0 | 0 | 0 | 0 | 0 | 0 | 0 | 0 | 0 | 2 | 0 | 1 | 0 | 1 | 0 | 0 | 0 |
| 12. C | 0 | 0 | 0 | 0 | 0 | 0 | 1 | 0 | 0 | 0 | 1 | 1 | 0 | 0 | 0 | 0 | 0 |
| 13. C | 0 | 0 | 0 | 0 | 0 | 0 | 0 | 1 | 0 | 0 | 0 | 0 | 0 | 0 | 0 | 0 | 0 |
| 14. H | 0 | 0 | 0 | 0 | 0 | 0 | 0 | 0 | 0 | 0 | 1 | 0 | 0 | 0 | 0 | 0 | 0 |
| 15. H | 1 | 0 | 0 | 0 | 0 | 0 | 0 | 0 | 0 | 0 | 0 | 0 | 0 | 0 | 0 | 0 | 0 |
| 16. H | 0 | 1 | 0 | 0 | 0 | 0 | 0 | 0 | 0 | 0 | 0 | 0 | 0 | 0 | 0 | 0 | 0 |

| | | | | | | | | | | | | | | | | | |
|---|---|---|---|---|---|---|---|---|---|---|---|---|---|---|---|---|---|
| 17. H | 0 | 0 | 0 | 0 | 0 | 1 | 0 | 0 | 0 | 0 | 0 | 0 | 0 | 0 | 0 | 0 | 0 |
| 18. H | 0 | 0 | 0 | 0 | 0 | 0 | 0 | 0 | 1 | 0 | 0 | 0 | 0 | 0 | 0 | 0 | 0 |
| 19. H | 0 | 0 | 0 | 0 | 0 | 0 | 0 | 0 | 0 | 1 | 0 | 0 | 0 | 0 | 0 | 0 | 0 |
| 20. H | 0 | 0 | 0 | 0 | 0 | 0 | 0 | 0 | 0 | 0 | 0 | 1 | 0 | 0 | 0 | 0 | 0 |
| 21. H | 0 | 0 | 0 | 0 | 0 | 0 | 0 | 0 | 0 | 0 | 0 | 0 | 1 | 0 | 0 | 0 | 0 |
| 22. H | 0 | 0 | 0 | 0 | 0 | 0 | 0 | 0 | 0 | 0 | 0 | 0 | 1 | 0 | 0 | 0 | 0 |
| 23. H | 0 | 0 | 0 | 0 | 0 | 0 | 0 | 0 | 0 | 0 | 0 | 0 | 1 | 0 | 0 | 0 | 0 |

| Atom | 18 | 19 | 20 | 21 | 22 | 23 |
|---|---|---|---|---|---|---|
| 1. C | 0 | 0 | 0 | 0 | 0 | 0 |
| 2. C | 0 | 0 | 0 | 0 | 0 | 0 |
| 3. C | 0 | 0 | 0 | 0 | 0 | 0 |
| 4. N | 0 | 0 | 0 | 0 | 0 | 0 |
| 5. C | 0 | 0 | 0 | 0 | 0 | 0 |
| 6. C | 0 | 0 | 0 | 0 | 0 | 0 |
| 7. C | 0 | 0 | 0 | 0 | 0 | 0 |
| 8. N | 0 | 0 | 0 | 0 | 0 | 0 |
| 9. C | 1 | 0 | 0 | 0 | 0 | 0 |
| 10. C | 0 | 1 | 0 | 0 | 0 | 0 |
| 11. C | 0 | 0 | 0 | 0 | 0 | 0 |
| 12. C | 0 | 0 | 1 | 0 | 0 | 0 |
| 13. C | 0 | 0 | 0 | 1 | 1 | 1 |
| 14. H | 0 | 0 | 0 | 0 | 0 | 0 |
| 15. H | 0 | 0 | 0 | 0 | 0 | 0 |

16. H 0 0 0 0 0 0

17. H 0 0 0 0 0 0

18. H 0 0 0 0 0 0

19. H 0 0 0 0 0 0

20. H 0 0 0 0 0 0

21. H 0 0 0 0 0 0

22. H 0 0 0 0 0 0

23. H 0 0 0 0 0 0

| | Resonance | |
|---|---|---|
| RS | Weight(%) | Added(Removed) |
| 1 | 14.53 | |
| 2 | 14.09 | ( C 7- N 8), ( C 10- C 11), C 11- C 12, N 8, C 10, ( C 12) |
| 3 | 12.77 | C 1- C 2, ( C 1- C 6), ( C 2- C 3), ( C 7- N 8), C 7- C 12, C 6, N 8, ( C 12) |
| 4 | 10.38 | ( C 5- C 9), ( C 7- N 8), C 7- C 12, N 8- C 9, C 5, ( C 12) |
| 5 | 8.06 | ( C 7- N 8), ( C 10- C 11), C 11- C 12, C 7, N 8, ( C 12) |
| 6 | 6.67 | ( C 2- C 3), C 3- C 10, ( C 7- N 8), C 7- C 12, ( C 10- C 11), N 8, C 11, ( C 12) |
| 7 | 5.96 | ( C 7- N 8), ( C 10- C 11), C 11- C 12, C 7, N 8, ( C 12) |

8 5.76 C 1- C 2, ( C 1- C 6), ( C 2- C 3), ( C 7- N 8),

C 7- C 12, C 3, N 8, ( C 12)

9 5.03 C 1- C 2, ( C 1- C 6), ( C 2- C 3), ( C 7- N 8),

C 7- C 12, C 3, N 8, ( C 12)

10 4.75 ( C 5- C 9), ( C 7- N 8), C 7- C 12, N 8- C 9,

C 5, ( C 12)

11 4.52 N 4- C 5, ( C 5- C 9), ( C 7- N 8), C 7- C 12,

( N 4), N 8, C 9, ( C 12)

12 3.69 ( C 1- C 6), C 5- C 6, ( C 5- C 9), ( C 7- N 8),

C 7- C 12, N 8, C 9, ( C 12)

13 2.94 ( C 2- C 3), C 3- C 10, ( C 7- N 8), C 7- C 12,

( C 10- C 11), C 2, N 8, ( C 12)

14 0.86 ( C 1- C 6), C 5- C 6, ( C 5- C 9), ( C 7- N 8),

C 7- C 12, C 1, N 8, ( C 12)

others 0.00

--------------------------------------------------------------------------

100.00 * Total *

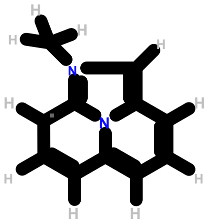
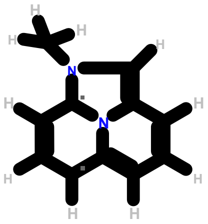
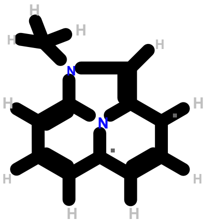
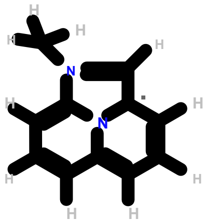

Vgt =14. 53%   r hoNL=2. 64463Wgt =14. 09%   r hoNL=3. 51316Wgt =12. 77%   r hoNL=3. 74009Wgt =10. 38%   r hoNL=3. 8940

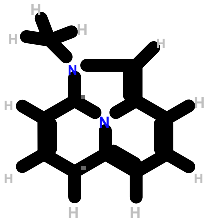
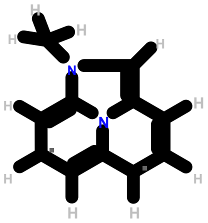
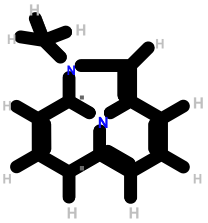
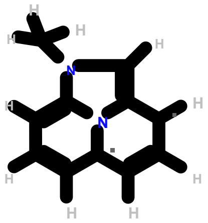

Wgt =8. 06%   r hoNL=3. 24796Wgt =6. 67%   r hoNL=3. 47793Wgt =5. 96%   r hoNL=3. 89422Wgt =5. 76%   r hoNL=4. 1460[illegible]

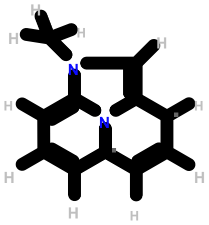
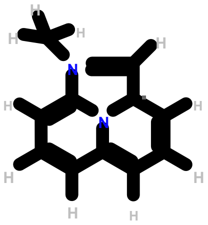
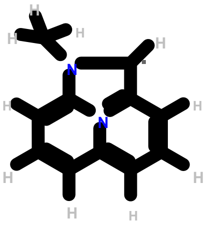
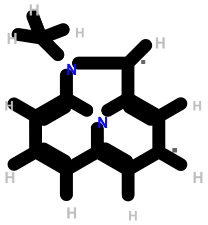

Wgt =5. 03%   r hoNL=3. 46327Wgt =4. 75%   r hoNL=3. 12069Wgt =4. 52%   r hoNL=2. 92081Wgt =3. 69%   r hoNL=3. 4577[illegible]

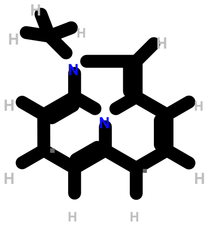

Wgt =2. 94%   r hoNL =3. 21430

---

NATURAL RESONANCE THEORY ANALYSIS:

Parent structure threshold:  50% of leading weight

Delocalization list threshold:  1 kcal/mol

Maximum search cycles:  unlimited

C1 symmetry, 1 symmetry operator(s), 1 unique atom permutation(s)

5 initial TOPO matrices: NLS = 1; NBI = 4; SYM = 0

cycle  structures    D(w)    kmax  CHOOSE   ION  CULL    E2   SYM  dbmax  dbrms

--------------------------------------------------------------------------------

| | | | | | | | | | | |
|---|---|---|---|---|---|---|---|---|---|---|
| 1 | 2/2 | 0.07039347 | 2 | 40 | 0 | 0 | 140 | 0 | 2.000 | 1.257 |
| 2 | 21/130 | 0.05937479 | 24 | 1224 | -8 | 0 | 245 | 0 | 0.400 | 0.163 |
| 3 | 35/444 | 0.05598959 | 74 | 6663 | -53 | 0 | 294 | 0 | 0.165 | 0.076 |
| 4 | 56/648 | 0.05486742 | 94 | 9410 | -115 | 0 | 136 | 0 | 0.087 | 0.037 |
| 5 | 98/659 | 0.05470521 | 104 | 4346 | -94 | 0 | 157 | 0 | 0.053 | 0.020 |
| 6 | 106/794 | 0.05454344 | 121 | 9237 | -16 | 0 | 446 | 0 | 0.039 | 0.012 |
| 7 | 130/1089 | 0.05424629 | 179 | 19944 | -89 | -87 | 73 | 0 | 0.123 | 0.026 |
| 8 | 125/1040 | 0.05424453 | 52 | 6976 | -8 | -187 | 811 | 0 | 0.005 | 0.001 |
| 9 | 114/1557 | 0.05409072 | 119 | 33317 | -124 | -162 | 409 | 0 | 0.031 | 0.012 |
| 10 | 122/1736 | 0.05401411 | 87 | 22233 | -71 | -62 | 1057 | 0 | 0.026 | 0.008 |
| 11 | 116/2743 | 0.05390518 | 146 | 44337 | -216 | -126 | 165 | 0 | 0.051 | 0.011 |
| 12 | 125/2836 | 0.05389000 | 44 | 7992 | -20 | -277 | 651 | 0 | 0.023 | 0.005 |
| 13 | 134/3306 | 0.05383651 | 83 | 30945 | -94 | -46 | 146 | 0 | 0.030 | 0.007 |
| 14 | 144/3471 | 0.05383645 | 25 | 3885 | -48 | -688 | 409 | 0 | 0.000 | 0.000 |
| 15 | 142/3124 | 0.05382461 | 33 | 13292 | -83 | -337 | 187 | 0 | 0.019 | 0.003 |
| 16 | 143/2976 | 0.05365991 | 52 | 11069 | -28 | -1079 | 88 | 0 | 0.069 | 0.012 |
| 17 | 129/2017 | 0.05360716 | 53 | 4836 | -10 | -219 | 593 | 0 | 0.038 | 0.008 |
| 18 | 136/2416 | 0.05359695 | 72 | 39693 | -88 | -729 | 762 | 0 | 0.014 | 0.004 |
| 19 | 125/2315 | 0.05356857 | 58 | 17241 | -124 | -222 | 421 | 0 | 0.020 | 0.005 |
| 20 | 123/2381 | 0.05354286 | 58 | 17520 | -48 | -350 | 178 | 0 | 0.019 | 0.005 |
| 21 | 120/2143 | 0.05353061 | 22 | 6288 | -16 | -194 | 94 | 0 | 0.016 | 0.003 |
| 22 | 113/2011 | 0.05352332 | 22 | 2756 | -9 | -127 | 94 | 0 | 0.019 | 0.003 |
| 23 | 118/1956 | 0.05351870 | 24 | 2788 | -1 | -626 | 0 | 0 | 0.007 | 0.002 |

QPNRT(118/1956): D(0)=0.07554187; D(w)=0.05351870; dbmax=0.007; dbrms=0.002

Timing(sec): search=6961.81; Gram matrix=4028.77; minimize=49.59; other=32.88

Fully in-core construction of Gram matrix requires 2024 MB of scratch memory

TOPO matrix for the leading resonance structure:

| Atom | 1 | 2 | 3 | 4 | 5 | 6 | 7 | 8 | 9 | 10 | 11 | 12 | 13 | 14 | 15 | 16 | 17 |
|---|---|---|---|---|---|---|---|---|---|---|---|---|---|---|---|---|---|
| 1. C | 0 | 2 | 0 | 0 | 0 | 1 | 0 | 0 | 0 | 0 | 0 | 0 | 0 | 0 | 1 | 0 | 0 |
| 2. C | 2 | 0 | 1 | 0 | 0 | 0 | 0 | 0 | 0 | 0 | 0 | 0 | 0 | 0 | 0 | 1 | 0 |
| 3. C | 0 | 1 | 0 | 1 | 0 | 0 | 0 | 0 | 0 | 2 | 0 | 0 | 0 | 0 | 0 | 0 | 0 |
| 4. N | 0 | 0 | 1 | 1 | 1 | 0 | 1 | 0 | 0 | 0 | 0 | 0 | 0 | 0 | 0 | 0 | 0 |
| 5. C | 0 | 0 | 0 | 1 | 0 | 2 | 0 | 0 | 1 | 0 | 0 | 0 | 0 | 0 | 0 | 0 | 0 |
| 6. C | 1 | 0 | 0 | 0 | 2 | 0 | 0 | 0 | 0 | 0 | 0 | 0 | 0 | 0 | 0 | 0 | 1 |
| 7. C | 0 | 0 | 0 | 1 | 0 | 0 | 0 | 2 | 0 | 0 | 0 | 1 | 0 | 0 | 0 | 0 | 0 |
| 8. N | 0 | 0 | 0 | 0 | 0 | 0 | 2 | 0 | 1 | 0 | 0 | 0 | 1 | 0 | 0 | 0 | 0 |
| 9. C | 0 | 0 | 0 | 0 | 1 | 0 | 0 | 1 | 1 | 0 | 0 | 0 | 0 | 0 | 0 | 0 | 0 |
| 10. C | 0 | 0 | 2 | 0 | 0 | 0 | 0 | 0 | 0 | 0 | 1 | 0 | 0 | 0 | 0 | 0 | 0 |
| 11. C | 0 | 0 | 0 | 0 | 0 | 0 | 0 | 0 | 0 | 1 | 0 | 2 | 0 | 1 | 0 | 0 | 0 |
| 12. C | 0 | 0 | 0 | 0 | 0 | 0 | 1 | 0 | 0 | 0 | 2 | 0 | 0 | 0 | 0 | 0 | 0 |
| 13. C | 0 | 0 | 0 | 0 | 0 | 0 | 0 | 1 | 0 | 0 | 0 | 0 | 0 | 0 | 0 | 0 | 0 |
| 14. H | 0 | 0 | 0 | 0 | 0 | 0 | 0 | 0 | 0 | 0 | 1 | 0 | 0 | 0 | 0 | 0 | 0 |
| 15. H | 1 | 0 | 0 | 0 | 0 | 0 | 0 | 0 | 0 | 0 | 0 | 0 | 0 | 0 | 0 | 0 | 0 |
| 16. H | 0 | 1 | 0 | 0 | 0 | 0 | 0 | 0 | 0 | 0 | 0 | 0 | 0 | 0 | 0 | 0 | 0 |

| | | | | | | | | | | | | | | | | | |
|---|---|---|---|---|---|---|---|---|---|---|---|---|---|---|---|---|---|
| 17. H | 0 | 0 | 0 | 0 | 0 | 1 | 0 | 0 | 0 | 0 | 0 | 0 | 0 | 0 | 0 | 0 | 0 |
| 18. H | 0 | 0 | 0 | 0 | 0 | 0 | 0 | 0 | 1 | 0 | 0 | 0 | 0 | 0 | 0 | 0 | 0 |
| 19. H | 0 | 0 | 0 | 0 | 0 | 0 | 0 | 0 | 0 | 1 | 0 | 0 | 0 | 0 | 0 | 0 | 0 |
| 20. H | 0 | 0 | 0 | 0 | 0 | 0 | 0 | 0 | 0 | 0 | 0 | 1 | 0 | 0 | 0 | 0 | 0 |
| 21. H | 0 | 0 | 0 | 0 | 0 | 0 | 0 | 0 | 0 | 0 | 0 | 0 | 1 | 0 | 0 | 0 | 0 |
| 22. H | 0 | 0 | 0 | 0 | 0 | 0 | 0 | 0 | 0 | 0 | 0 | 0 | 1 | 0 | 0 | 0 | 0 |
| 23. H | 0 | 0 | 0 | 0 | 0 | 0 | 0 | 0 | 0 | 0 | 0 | 0 | 1 | 0 | 0 | 0 | 0 |

| Atom | 18 | 19 | 20 | 21 | 22 | 23 |
|---|---|---|---|---|---|---|
| 1. C | 0 | 0 | 0 | 0 | 0 | 0 |
| 2. C | 0 | 0 | 0 | 0 | 0 | 0 |
| 3. C | 0 | 0 | 0 | 0 | 0 | 0 |
| 4. N | 0 | 0 | 0 | 0 | 0 | 0 |
| 5. C | 0 | 0 | 0 | 0 | 0 | 0 |
| 6. C | 0 | 0 | 0 | 0 | 0 | 0 |
| 7. C | 0 | 0 | 0 | 0 | 0 | 0 |
| 8. N | 0 | 0 | 0 | 0 | 0 | 0 |
| 9. C | 1 | 0 | 0 | 0 | 0 | 0 |
| 10. C | 0 | 1 | 0 | 0 | 0 | 0 |
| 11. C | 0 | 0 | 0 | 0 | 0 | 0 |
| 12. C | 0 | 0 | 1 | 0 | 0 | 0 |
| 13. C | 0 | 0 | 0 | 1 | 1 | 1 |
| 14. H | 0 | 0 | 0 | 0 | 0 | 0 |
| 15. H | 0 | 0 | 0 | 0 | 0 | 0 |

16. H 0 0 0 0 0 0

17. H 0 0 0 0 0 0

18. H 0 0 0 0 0 0

19. H 0 0 0 0 0 0

20. H 0 0 0 0 0 0

21. H 0 0 0 0 0 0

22. H 0 0 0 0 0 0

23. H 0 0 0 0 0 0

| | Resonance | |
|---|---|---|
| RS | Weight(%) | Added(Removed) |
| 1 | 3.14 | |
| 2 | 2.53 | N 4- C 7, ( C 7- N 8), ( N 4), N 8 |
| 3 | 2.38 | N 4- C 7, ( C 5- C 6), C 5- C 9, ( C 7- N 8), ( N 4), C 6, N 8, ( C 9) |
| 4 | 2.32 | ( C 5- C 6), C 5- C 9, C 6, ( C 9) |
| 5 | 2.10 | ( C 1- C 2), C 1- C 6, C 2- C 3, ( C 3- C 10), ( C 3- C 10), C 3- H 14, N 4- C 5, ( C 5- C 6), ( C 7- N 8), C 7- C 12, N 8- C 9, C 10- C 11, C 10- C 11, ( C 10- H 19), ( C 11- C 12), ( C 11- H 14), ( N 4), ( C 9), C 10, H 19 |
| 6 | 2.03 | C 3- N 4, ( C 3- C 10), ( C 7- N 8), N 8- C 12, C 10- C 11, ( C 11- C 12), ( N 4), C 7 |
| 7 | 2.02 | ( C 1- C 2), C 1- C 6, C 2- N 4, ( C 3- C 10), |

( C 5- C 6), C 5- C 9, ( C 7- N 8), C 7- C 12,
C 10- C 11, ( C 11- C 12), C 3, ( N 4), N 8,
( C 9)

8 1.96 ( C 1- C 2), C 1- C 6, C 2- C 3, ( C 3- C 10),
( C 5- C 6), C 5- C 9, ( C 7- N 8), N 8- C 12,
C 10- C 11, ( C 11- C 12), C 7, ( C 9)

9 1.81 N 4- C 5, ( C 5- C 6), ( N 4), C 6

10 1.76 ( C 1- C 2), C 2- C 6, C 3- N 4, ( C 3- C 10),
( C 5- C 6), C 5- C 9, ( C 7- N 8), C 7- C 12,
C 10- C 11, ( C 11- C 12), C 1, ( N 4), N 8,
( C 9)

11 1.63 ( C 1- C 2), C 2- C 10, C 3- N 4, ( C 3- C 10),
( C 5- C 6), C 5- C 9, C 1, ( N 4), C 6,
( C 9)

12 1.61 C 3- N 4, ( C 3- C 10), C 10- C 11, ( C 11- C 12),
( N 4), C 12

13 1.61 ( C 1- C 2), C 1- C 6, C 2- C 3, ( C 3- C 10),
N 4- C 7, ( C 5- C 6), C 5- C 9, ( C 7- N 8),
C 10- C 11, ( C 11- C 12), ( N 4), N 8, ( C 9),
C 12

14 1.59 ( C 7- N 8), N 8- C 9, C 7, ( C 9)

15 1.57 ( C 1- C 2), C 1- C 6, C 2- C 3, C 2- C 3,
( C 2- H 16), ( C 3- N 4), ( C 3- C 10), N 4- C 7,
( C 5- C 6), C 5- C 9, ( C 7- N 8), N 8- C 12,
C 10- C 11, ( C 11- C 12), ( C 9), H 16

16 1.55 ( C 1- C 2), C 1- C 6, C 2- C 3, ( C 3- C 10),
( C 5- C 6), C 5- C 9, C 10- C 11, ( C 11- C 12),
( C 9), C 12

17 1.54 ( C 1- C 2), C 1- C 6, C 2- C 3, ( C 3- C 10),
N 4- C 5, ( C 5- C 6), ( C 7- N 8), C 7- C 12,
N 8- C 9, C 10- C 11, C 10- C 11, ( C 10- H 19),
( C 11- C 12), ( C 11- C 12), C 12- H 19, ( C 12- H 20),
( N 4), ( C 9), C 12, H 20

18 1.45 ( C 1- C 2), C 1- C 6, C 2- C 3, ( C 3- C 10),
N 4- C 5, ( C 5- C 6), ( C 7- N 8), N 8- C 12,
C 10- C 11, ( C 11- C 12), ( N 4), C 7

19 1.45 C 1- C 2, ( C 1- H 15), ( C 2- C 3), ( C 2- H 16),
C 3- C 10, N 4- C 7, ( C 7- N 8), N 8- C 9,
( C 10- C 11), C 11- C 12, ( C 12- H 20), H 15- H 16,
C 2, ( N 4), ( C 9), H 20

20 1.41 ( C 1- C 2), C 1- C 6, C 2- C 3, ( C 3- C 10),
N 4- C 5, ( C 5- C 6), ( C 7- N 8), C 7- C 12,
N 8- C 9, C 10- C 11, ( C 10- H 19), ( C 11- C 12),
( C 11- C 12), C 12- H 19, ( N 4), ( C 9), C 10,
C 11

21 1.41 ( C 1- C 2), ( C 1- C 2), C 1- C 6, C 1- C 6,
C 2- C 3, ( C 3- C 10), N 4- N 8, ( C 5- C 6),
( C 5- C 6), C 6- C 9, ( C 6- H 17), ( C 7- N 8),
C 7- C 12, C 10- C 11, ( C 11- C 12), C 2, ( N 4),
C 5, ( C 9), H 17

22 1.36 ( C 1- C 2), C 2- C 3, ( C 3- C 10), N 4- N 8,

( C 5- C 6), C 5- C 9, ( C 7- N 8), C 7- C 12,

C 10- C 11, ( C 11- C 12), C 1, ( N 4), C 6,

( C 9)

23 1.35 ( C 1- C 2), ( C 1- C 2), C 1- C 6, C 2- C 3,

C 2- C 3, ( C 3- C 10), ( C 3- C 10), N 4- C 5,

( C 5- C 6), ( C 7- N 8), C 7- C 12, N 8- C 9,

C 10- C 11, C 10- C 11, ( C 11- C 12), ( C 11- H 14),

C 1, ( N 4), ( C 9), H 14

24 1.30 ( C 1- C 2), ( C 1- C 2), C 1- C 6, ( C 2- C 3),

C 2- C 10, C 3- N 4, C 3- N 4, ( C 3- C 10),

( N 4- C 7), ( C 5- C 6), C 5- C 9, C 7- N 8,

C 7- N 8, ( C 7- C 12), ( N 8- C 9), ( N 8- C 13),

C 1, C 2, ( N 4), C 13

25 1.28 ( C 1- C 2), C 1- C 6, C 2- C 3, C 2- C 3,

( C 2- H 16), ( C 3- N 4), ( C 3- C 10), N 4- C 5,

( C 5- C 6), ( C 7- N 8), C 7- C 12, N 8- C 9,

C 10- C 11, ( C 11- C 12), ( C 9), H 16

26 1.25 ( C 1- C 2), C 1- C 6, C 2- C 3, ( C 3- C 10),

N 4- C 5, N 4- C 5, ( N 4- C 7), ( C 5- C 6),

( C 5- C 6), ( C 7- N 8), C 7- C 12, N 8- C 9,

C 10- C 11, ( C 11- C 12), ( N 4), C 6, C 7,

( C 9)

27 1.24 ( C 1- C 2), C 1- C 6, C 2- C 3, ( C 3- C 10),

N 4- C 7, ( C 5- C 6), C 5- C 9, ( C 7- N 8),

C 10- C 12, ( C 11- C 12), ( N 4), N 8, ( C 9),
C 11

28 1.24 ( C 1- C 2), C 1- C 6, C 1- C 6, ( C 1- H 15),
C 2- C 3, ( C 3- C 10), N 4- N 8, ( C 5- C 6),
( C 5- C 6), C 5- C 9, ( C 6- H 17), ( C 7- N 8),
C 7- C 12, C 10- C 11, ( C 11- C 12), H 15- H 17,
( N 4), C 5, C 6, ( C 9)

29 1.22 C 3- N 4, ( C 3- C 10), ( C 7- N 8), C 7- C 12,
N 8- C 13, C 10- C 11, ( C 11- C 12), ( C 13- H 21),
( N 4), H 21

30 1.21 C 3- N 4, ( C 3- C 10), ( C 5- C 6), C 5- C 9,
( C 7- N 8), N 8- C 12, C 10- C 11, ( C 11- C 12),
( N 4), C 6, C 7, ( C 9)

31 1.20 ( C 1- C 2), C 1- C 6, C 2- C 3, ( C 3- N 4),
( C 3- C 10), N 4- C 7, ( C 5- C 6), C 5- C 9,
( C 7- N 8), N 8- C 12, C 10- C 11, ( C 11- C 12),
C 3, ( C 9)

32 1.18 ( C 1- C 2), C 1- C 6, C 2- C 3, ( C 3- C 10),
( C 5- C 6), C 5- C 9, C 10- C 12, ( C 11- C 12),
( C 9), C 11

33 1.16 ( C 1- C 2), C 1- C 6, C 2- C 3, ( C 3- C 10),
( C 5- C 6), C 5- C 9, ( C 7- N 8), C 7- C 12,
( N 8- C 9), N 8- C 13, N 8- C 13, C 9- H 23,
C 10- C 11, ( C 11- C 12), ( C 13- H 21), ( C 13- H 22),
( C 13- H 23), ( C 9), C 13, H 22

34 1.14 ( C 1- C 2), C 1- C 6, C 2- C 3, ( C 3- C 10),

( C 5- C 6), C 5- C 9, ( C 7- N 8), C 7- C 12,

N 8- C 13, C 10- C 11, ( C 11- C 12), ( C 13- H 21),

( C 9), H 21

35 1.12 ( C 1- C 2), C 1- C 6, C 2- C 10, ( C 3- C 10),

( C 5- C 6), C 5- C 9, C 3, ( C 9)

36 1.12 ( C 1- C 2), C 1- C 6, C 2- C 10, C 3- N 4,

( C 3- C 10), ( C 5- C 6), ( N 4), C 5

37 1.10 ( C 1- C 2), C 1- C 6, C 2- C 10, C 3- N 4,

( C 3- C 10), ( C 5- C 6), ( N 4), C 5

38 1.04 ( C 1- C 2), C 1- C 6, C 2- C 3, ( C 3- C 10),

( C 5- C 6), C 5- C 9, ( C 7- N 8), N 8- C 12,

C 10- C 11, ( C 11- C 12), C 7, ( C 9)

39 1.04 N 4- N 8, ( C 7- N 8), ( N 4), C 7

40 1.03 ( C 1- C 2), C 1- C 6, C 2- C 3, ( C 3- C 10),

N 4- C 5, ( C 5- C 6), C 10- C 11, ( C 11- C 12),

( N 4), C 12

41 1.03 ( C 1- C 2), C 2- C 6, N 4- C 7, ( C 5- C 6),

C 5- C 9, ( C 7- N 8), C 1, ( N 4), N 8,

( C 9)

42 0.97 ( C 1- C 2), C 2- C 6, ( C 5- C 6), C 5- C 9,

C 1, ( C 9)

43 0.96 ( C 1- C 2), C 1- C 6, C 2- C 10, C 3- N 4,

C 3- N 4, ( C 3- C 10), ( C 3- C 10), ( N 4- C 5),

( C 5- C 6), C 5- C 9, ( N 4), C 5, ( C 9),

C 10

44 0.94 ( C 1- C 2), C 1- C 6, C 2- C 10, C 3- N 4,
( C 3- C 10), ( N 4- C 7), ( C 5- C 6), C 5- C 9,
C 7, ( C 9)

45 0.89 ( C 3- N 4), ( N 4- C 5), N 4- C 7, C 5- C 6,
( C 6- H 17), ( C 7- N 8), N 8- C 9, C 3,
( C 9), H 17

46 0.85 ( C 1- C 2), C 1- C 6, C 2- C 3, ( C 3- C 10),
N 4- N 8, ( C 5- C 6), ( C 7- N 8), C 7- C 12,
C 10- C 11, ( C 11- C 12), ( N 4), C 5

47 0.84 N 4- C 9, ( C 7- N 8), ( N 4), C 7, N 8,
( C 9)

48 0.83 ( C 1- C 2), C 1- C 6, C 2- C 3, ( C 3- C 10),
N 4- N 8, ( C 5- C 6), ( C 7- N 8), C 7- C 12,
C 10- C 11, ( C 11- C 12), ( N 4), C 5

49 0.82 C 3- N 4, ( C 3- C 10), ( C 7- N 8), C 7- C 12,
N 8- C 13, C 10- C 11, ( C 11- C 12), ( C 13- H 22),
( N 4), H 22

50 0.82 ( C 1- C 2), C 1- C 6, C 2- C 3, ( C 3- C 10),
( N 4- C 5), N 4- C 7, N 4- C 7, ( C 5- C 6),
C 5- C 9, ( C 7- N 8), ( C 7- C 12), N 8- C 12,
C 10- C 11, ( C 11- C 12), ( N 4), C 5, ( C 9),
C 12

51 0.81 ( C 3- N 4), N 4- C 7, ( C 7- N 8), N 8- C 9,
C 3, ( C 9)

52 0.80 C 3- N 4, ( C 3- C 10), ( C 5- C 6), C 5- C 9,
C 10- C 11, ( C 11- C 12), ( N 4), C 6, ( C 9),
C 12

53 0.79 ( N 4- C 5), N 4- C 7, N 4- H 18, ( C 7- N 8),
( C 7- N 8), ( C 7- C 12), C 7- H 23, N 8- C 9,
N 8- C 9, ( C 9- H 18), ( C 13- H 23), ( N 4),
C 5, C 7, ( C 9), C 12

54 0.78 ( C 1- C 2), C 1- C 6, C 2- C 3, ( C 3- C 10),
( C 5- C 6), C 5- C 9, ( C 7- N 8), ( C 7- N 8),
C 7- C 12, ( N 8- C 9), N 8- C 13, N 8- C 13,
N 8- C 13, C 10- C 11, ( C 11- C 12), ( C 13- H 21),
( C 13- H 22), ( C 13- H 23), C 7, H 22

55 0.78 N 4- C 7, ( C 5- C 9), ( C 7- N 8), ( C 7- N 8),
( C 7- C 12), C 7- H 23, N 8- C 9, N 8- C 9,
N 8- C 9, ( N 8- C 13), ( C 9- H 18), ( C 13- H 23),
( N 4), C 7, ( C 9), C 12, C 13, H 18

56 0.76 ( C 1- C 2), C 1- C 6, C 1- C 6, ( C 1- H 15),
C 2- C 3, ( C 3- C 10), N 4- N 8, ( C 5- C 6),
( C 5- C 6), C 5- H 15, C 6- C 9, ( C 6- H 17),
( C 7- N 8), C 7- C 12, ( C 9- H 18), C 10- C 11,
( C 11- C 12), ( N 4), H 17, H 18

57 0.76 N 4- C 7, ( C 7- N 8), ( C 7- C 12), N 8- C 9,
C 11- C 12, ( C 11- H 14), ( N 4), C 7, ( C 9),
H 14

58 0.76 N 4- C 7, ( C 7- N 8), N 8- C 9, ( C 10- C 11),

C 11- C 12, ( C 11- H 14), ( C 12- H 20),  H 14- H 20,
( N  4), ( C  9),  C 10,  C 11

59 0.75 ( C  1- C  2),  C  1- C  6,  C  2- C  3, ( C  3- C 10),
( C  5- C  6),  C  5- C  9, ( C  7- N  8),  C  7- C 12,
N  8- C 13,  C 10- C 11, ( C 11- C 12), ( C 13- H 22),
( C  9),  H 22

60 0.73 ( C  1- C  2),  C  3- N  4, ( C  3- C 10), ( C  5- C  6),
C  5- C  9,  C  6- C 10,  C  1,  C  2, ( N  4), ( C  9)

61 0.72 ( C  1- C  2),  C  1- C  6,  N  4- C  7, ( C  5- C  6),
C  5- C  9, ( C  7- N  8),  C  2, ( N  4),  N  8,
( C  9)

62 0.69 ( C  1- C  2),  C  1- C  6,  C  2- C  3, ( C  3- C 10),
N  4- C  5, ( C  5- C  6), ( C  7- N  8),  C  7- C 12,
N  8- C  9, ( C 11- C 12), ( N  4), ( C  9),  C 10,
C 11

63 0.68 ( C  1- C  2),  C  1- C  6,  C  2- C  3, ( C  3- C 10),
N  4- C  9, ( C  5- C  6), ( C  7- N  8),  C  7- C 12,
C 10- C 11, ( C 11- C 12), ( N  4),  C  5,  N  8,
( C  9)

64 0.67 ( C  1- C  2),  C  1- C  6,  C  2- C  3, ( C  3- C 10),
N  4- C  5, ( C  5- C  6),  C 10- C 12, ( C 11- C 12),
( N  4),  C 11

65 0.67 ( C  1- C  2),  C  1- C  6,  C  2- C 10,  C  3- N  4,
( C  3- C 10), ( N  4- C  5), ( N  4- C  7),
( C  5- C  6),  C  5- C  9,  C  7- N  8, ( N  8- C 13),

C 5, ( C 9), C 13

66 0.66 ( C 1- C 2), C 1- C 6, ( C 5- C 6), C 5- C 9,

C 2, ( C 9)

67 0.65 ( C 7- N 8), N 8- C 9, C 7, ( C 9)

68 0.65 ( C 1- C 2), C 1- C 6, C 2- C 3, ( C 3- C 10),

N 4- C 5, ( C 5- C 6), ( C 7- N 8), C 7- C 12,

N 8- C 9, C 10- C 11, C 10- C 11, ( C 10- H 19),

( C 11- C 12), ( C 11- C 12), ( N 4), ( C 9), C 12,

H 19

69 0.61 ( C 1- C 2), C 1- C 6, C 2- C 3, ( C 3- C 10),

( C 5- C 6), C 5- C 9, ( C 7- N 8), ( C 7- N 8),

C 7- C 12, N 8- C 13, C 10- C 11, ( C 11- C 12),

( C 13- H 23), C 7, N 8, ( C 9)

70 0.60 ( C 1- C 2), ( C 1- C 2), C 2- C 3, C 2- C 6,

( C 3- C 10), ( C 5- C 6), C 5- C 9, C 1,

( C 9), C 10

71 0.59 N 4- C 7, ( C 7- N 8), ( C 7- N 8), ( C 7- C 12),

N 8- C 9, N 8- C 9, ( C 9- H 18), ( N 4), C 7,

C 7, ( C 9), C 12

72 0.58 ( C 1- C 2), ( C 1- C 2), C 1- C 6, C 1- C 6,

C 2- C 3, C 2- C 3, ( C 3- C 10), ( C 3- C 10),

N 4- C 5, ( C 5- C 6), ( C 6- H 17), ( C 7- N 8),

C 7- C 12, N 8- C 9, C 10- C 11, ( C 11- C 12),

( N 4), ( C 9), C 10, H 17

73 0.56 N 4- C 7, ( C 7- N 8), N 8- C 9, ( C 10- C 11),

( N 4), ( C 9), C 10, C 11

74 0.56 ( C 1- C 2), C 1- C 6, C 2- C 3, ( C 3- C 10),
( C 5- C 6), C 5- C 9, ( C 7- N 8), C 7- C 12,
C 10- C 12, ( C 11- C 12), ( C 11- C 12), N 8,
( C 9), C 11

75 0.55 ( C 1- C 2), C 1- C 6, C 1- C 6, ( C 1- H 15),
C 2- C 10, C 3- N 4, ( C 3- C 10), ( C 5- C 6),
( C 5- C 6), C 5- C 9, ( N 4), C 5, ( C 9),
H 15

76 0.55 N 4- C 7, ( C 7- N 8), N 8- C 13, ( C 13- H 21),
( N 4), H 21

77 0.53 C 1- C 2, ( C 1- H 15), ( C 2- C 3), C 3- C 10,
N 4- C 7, ( C 7- N 8), N 8- C 9, ( C 10- C 11),
C 11- C 12, ( C 11- H 14), ( C 12- H 20), H 14- H 20,
( N 4), ( C 9), C 11, H 15

78 0.52 ( C 1- C 2), C 1- C 6, ( C 2- C 3), C 2- C 10,
C 3- N 4, C 3- N 4, ( C 3- C 10), ( N 4- C 7),
( C 5- C 6), C 5- C 9, C 7- N 8, ( N 8- C 13),
C 2, ( N 4), ( C 9), C 13

79 0.51 ( C 1- C 2), C 1- C 6, ( C 3- C 10), N 4- C 7,
( C 5- C 6), C 5- C 9, ( C 7- N 8), N 8- C 12,
C 10- C 11, ( C 11- C 12), C 2, C 3, ( N 4),
( C 9)

80 0.50 ( C 1- C 2), ( C 1- C 2), C 1- C 5, C 1- C 6,
C 1- C 6, ( C 1- H 15), C 2- C 3, ( C 3- C 10),

N 4- N 8, ( C 5- C 6), ( C 5- C 6), C 6- C 9,
( C 6- H 17), ( C 7- N 8), C 7- C 12, ( C 9- H 18),
C 10- C 11, ( C 11- C 12), H 15- H 17, C 2, ( N 4),
H 18

81 0.49 ( C 1- C 2), C 1- C 6, ( C 3- C 10), ( C 3- C 10),
N 4- C 5, ( C 5- C 6), ( C 7- N 8), C 7- C 12,
N 8- C 9, ( C 11- C 12), C 2, C 3, ( N 4),
( C 9), C 10, C 11

82 0.49 ( C 1- C 2), C 1- C 6, C 2- C 3, ( C 3- C 10),
N 4- C 5, ( N 4- C 7), ( C 5- C 6), ( C 7- N 8),
C 7- C 12, C 7- C 12, N 8- C 9, C 10- C 11,
C 10- C 11, ( C 10- H 19), ( C 11- C 12), ( C 11- C 12),
( C 11- H 14), C 12- H 19, ( C 12- H 20), H 14- H 20,
( C 9), C 11

83 0.48 ( C 1- C 2), C 1- C 6, C 2- C 3, ( C 3- C 10),
( N 4- C 5), N 4- C 7, ( C 5- C 6), C 5- C 9,
C 5- C 9, ( C 7- N 8), N 8- C 12, ( C 9- H 18),
C 10- C 11, ( C 11- C 12), ( C 9), H 18

84 0.47 ( C 1- C 2), C 1- C 6, C 2- C 3, ( C 3- C 10),
( C 3- C 10), N 4- C 5, ( C 5- C 6), ( C 7- N 8),
C 7- C 12, N 8- C 9, C 10- C 11, C 10- C 11,
C 10- C 12, ( C 10- H 19), ( C 11- C 12), ( C 11- C 12),
( C 11- H 14), H 14- H 19, C 3, ( N 4), ( C 9),
C 11

85 0.46 ( C 1- C 2), C 2- C 6, N 4- C 5, ( C 5- C 6),

C 1, ( N 4)

86 0.46 ( C 1- C 2), C 1- C 6, C 1- C 6, ( C 1- H 15),

C 2- C 3, ( C 2- H 16), ( C 3- C 10), N 4- N 8,

( C 5- C 6), ( C 5- C 6), C 5- C 9, ( C 7- N 8),

C 7- C 12, C 10- C 11, ( C 11- C 12), H 15- H 16,

C 2, ( N 4), C 5, ( C 9)

87 0.45 ( C 1- C 2), C 1- C 6, C 2- C 3, ( C 3- C 10),

( N 4- C 5), ( C 5- C 6), C 5- C 9, C 5- C 9,

( C 7- N 8), C 7- C 12, ( N 8- C 9), N 8- C 13,

N 8- C 13, C 10- C 11, ( C 11- C 12), ( C 13- H 21),

( C 13- H 22), N 4, ( C 9), H 22

88 0.44 ( C 1- C 2), C 1- C 6, ( C 2- C 3), C 2- C 10,

C 3- N 4, ( C 3- C 10), ( C 5- C 6), C 5- C 9,

C 2, C 3, ( N 4), ( C 9)

89 0.43 C 3- N 4, ( C 3- C 10), ( C 5- C 6), C 5- C 9,

C 10- C 12, ( C 11- C 12), ( N 4), C 6, ( C 9),

C 11

90 0.42 ( N 4- C 5), N 4- C 7, N 4- C 7, ( C 7- N 8),

( C 7- C 12), N 8- C 9, ( N 4), C 5, ( C 9),

C 12

91 0.40 ( C 1- C 2), C 1- C 6, C 2- C 10, C 3- N 4,

( C 3- C 10), ( N 4- C 5), ( C 5- C 6), C 5- C 9,

C 5- C 9, ( C 9- H 18), ( C 9), H 18

92 0.40 ( C 1- C 2), C 1- C 6, ( C 3- C 10), ( C 3- C 10),

N 4- C 7, ( C 5- C 6), C 5- C 9, ( C 7- N 8),

N 8- C 12, C 10- C 11, C 10- C 11, ( C 11- C 12),
( C 11- H 14), C 2, C 3, ( N 4), ( C 9), H 14

93 0.38 N 4- C 7, ( C 7- N 8), ( C 7- N 8), ( C 7- C 12),
C 7- H 23, N 8- C 9, N 8- C 9, ( C 9- H 18),
C 11- C 12, ( C 11- H 14), ( C 13- H 23), ( N 4),
C 7, ( C 9), H 14, H 18

94 0.33 N 4- C 7, ( C 7- N 8), ( C 7- N 8), ( C 7- C 12),
C 7- H 23, N 8- C 9, N 8- C 9, ( C 9- H 18),
( C 13- H 23), ( N 4), C 7, ( C 9), C 12, H 18

95 0.33 C 1- C 2, ( C 1- H 15), ( C 2- C 3), C 3- C 10,
N 4- C 7, ( C 7- N 8), N 8- C 9, ( C 10- C 11),
( N 4), ( C 9), C 11, H 15

96 0.29 C 1- C 2, ( C 1- C 6), ( C 1- H 15), ( C 2- C 3),
C 3- C 10, N 4- C 7, C 5- C 6, ( C 5- C 9),
( C 7- N 8), N 8- C 9, ( C 10- C 11), C 11- C 12,
( C 12- H 20), ( N 4), H 15, H 20

97 0.29 ( C 1- C 2), C 1- C 6, C 2- C 3, ( C 3- C 10),
( N 4- C 5), N 4- C 7, ( C 5- C 6), C 5- C 9,
( C 7- N 8), N 8- C 12, C 10- C 11, ( C 11- C 12),
C 5, ( C 9)

98 0.28 ( C 1- C 2), C 1- C 6, C 1- C 6, ( C 1- H 15),
C 2- C 3, ( C 3- C 10), N 4- N 8, ( C 5- C 6),
( C 5- C 6), C 5- C 9, ( C 7- N 8), C 7- C 12,
C 10- C 11, ( C 11- C 12), ( N 4), C 5, ( C 9),
H 15

99 0.28 ( C 1- C 2), ( C 1- C 2), C 2- C 3, C 2- C 3,
( C 3- C 10), ( C 3- C 10), N 4- N 8, ( C 5- C 6),
C 5- C 9, ( C 7- N 8), C 7- C 12, C 10- C 11,
( C 11- C 12), C 1, ( N 4), C 6, ( C 9), C 10

100 0.24 ( C 1- C 2), C 1- C 6, C 2- C 3, ( C 3- C 10),
N 4- C 5, ( C 5- C 6), ( C 7- N 8), C 7- C 12,
N 8- C 13, C 10- C 11, ( C 11- C 12), ( C 13- H 22),
( N 4), H 22

101 0.22 N 4- N 8, ( C 5- C 6), C 5- C 9, ( C 7- N 8),
( N 4), C 6, C 7, ( C 9)

102 0.22 ( C 1- C 2), C 1- C 6, C 2- C 10, C 3- N 4,
( C 3- C 10), ( N 4- C 5), ( C 5- C 6), C 5- C 9,
C 5, ( C 9)

103 0.20 ( C 1- C 2), C 1- C 9, C 2- C 3, ( C 3- C 10),
( C 5- C 6), ( C 7- N 8), C 7- C 12, C 10- C 11,
( C 11- C 12), C 5, N 8, ( C 9)

104 0.19 ( C 1- C 2), ( C 1- C 2), C 1- C 6, C 2- C 3,
C 2- C 3, ( C 3- C 10), ( C 3- C 10), N 4- C 5,
( C 5- C 6), ( C 7- N 8), C 7- C 12, N 8- C 9,
C 10- C 11, ( C 11- C 12), C 1, ( N 4), ( C 9),
C 10

105 0.18 ( C 1- C 2), C 1- C 6, C 1- C 6, ( C 1- H 15),
C 2- C 3, ( C 3- C 10), N 4- N 8, ( C 5- C 6),
( C 5- C 6), C 5- H 18, C 6- C 9, ( C 6- H 17),
( C 7- N 8), C 7- C 12, ( C 9- H 18), C 10- C 11,

( C 11- C 12), H 15- H 17, ( N 4), C 5

106 0.15 N 4- C 7, ( C 7- N 8), N 8- C 13, ( C 13- H 22),

( N 4), H 22

107 0.15 ( C 1- C 2), C 1- C 6, N 4- C 5, ( C 5- C 6),

C 2, ( N 4)

108 0.14 ( C 1- C 2), C 1- C 6, C 2- C 3, ( C 3- C 10),

( N 4- C 5), N 4- N 8, ( C 5- C 6), C 5- C 9,

C 5- C 9, ( C 7- N 8), C 7- C 12, ( C 9- H 18),

C 10- C 11, ( C 11- C 12), ( C 9), H 18

109 0.13 ( C 2- C 3), N 4- C 7, ( C 7- N 8), N 8- C 9,

C 2, C 3, ( N 4), ( C 9)

110 0.12 ( C 1- C 2), ( C 1- C 2), C 1- C 6, C 1- C 6,

( C 1- H 15), C 2- C 3, C 2- C 3, ( C 3- C 10),

( C 3- C 10), N 4- N 8, ( C 5- C 6), ( C 5- C 6),

C 6- C 9, ( C 6- H 17), ( C 7- N 8), C 7- C 12,

( C 9- H 18), C 10- C 11, ( C 11- C 12), H 15- H 17,

( N 4), C 5, C 10, H 18

111 0.11 ( N 4- C 5), N 4- C 7, N 4- H 18, ( C 7- N 8),

( C 7- N 8), ( C 7- C 12), N 8- C 9, N 8- C 9,

( C 9- H 18), ( N 4), C 5, C 7, ( C 9), C 12

others 0.21

--------------------------------------------------------------------------

100.00 * Total *

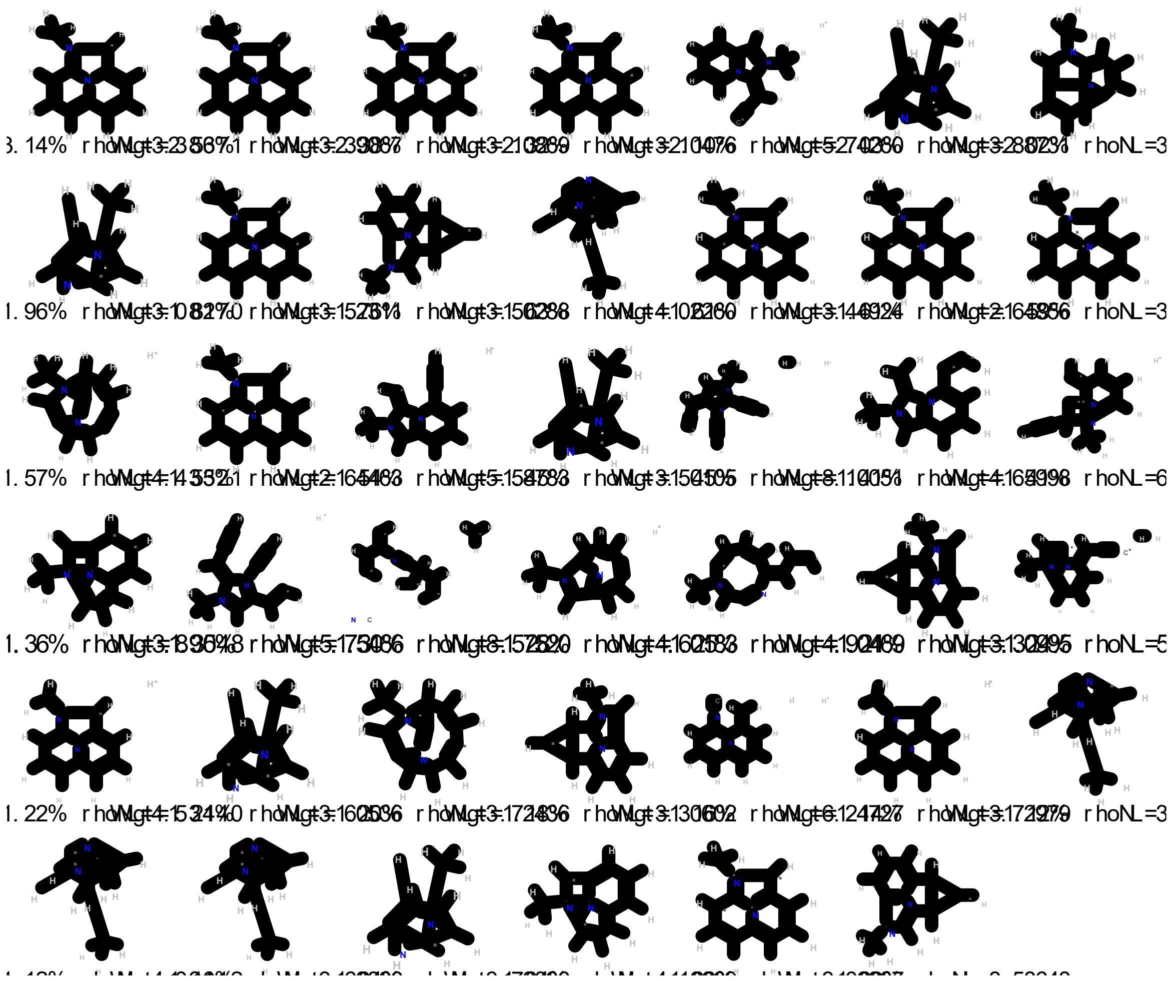

NRTPAR=40 NRTE2=1

NATURAL RESONANCE THEORY ANALYSIS:

Parent structure threshold:  40%

Delocalization list threshold:  1 kcal/mol

Maximum search cycles:  unlimited

C1 symmetry, 1 symmetry operator(s), 1 unique atom permutation(s)

5 initial TOPO matrices: NLS = 1; NBI = 4; SYM = 0

| cycle | structures | D(w) | kmax | CHOOSE | ION | CULL | E2 | SYM | dbmax | dbrms |
|---|---|---|---|---|---|---|---|---|---|---|
| 1 | 2/2 | 0.07040160 | 2 | 40 | 0 | 0 | 140 | 0 | 2.000 | 1.257 |
| 2 | 21/130 | 0.05938247 | 24 | 1224 | -8 | 0 | 0 | 0 | 0.399 | 0.163 |

QPNRT(21/130): D(0)=0.07348827; D(w)=0.05938247; dbmax=0.399; dbrms=0.163

Timing(sec): search=95.62; Gram matrix=2.45; minimize=0.02; other=0.86

TOPO matrix for the leading resonance structure:

| Atom | | 1 | 2 | 3 | 4 | 5 | 6 | 7 | 8 | 9 | 10 | 11 | 12 | 13 | 14 | 15 | 16 | 17 |
|---|---|---|---|---|---|---|---|---|---|---|---|---|---|---|---|---|---|---|
| 1. | C | 0 | 1 | 0 | 0 | 0 | 2 | 0 | 0 | 0 | 0 | 0 | 0 | 0 | 0 | 1 | 0 | 0 |
| 2. | C | 1 | 0 | 2 | 0 | 0 | 0 | 0 | 0 | 0 | 0 | 0 | 0 | 0 | 0 | 0 | 1 | 0 |
| 3. | C | 0 | 2 | 0 | 1 | 0 | 0 | 0 | 0 | 0 | 1 | 0 | 0 | 0 | 0 | 0 | 0 | 0 |
| 4. | N | 0 | 0 | 1 | 0 | 1 | 0 | 2 | 0 | 0 | 0 | 0 | 0 | 0 | 0 | 0 | 0 | 0 |
| 5. | C | 0 | 0 | 0 | 1 | 0 | 1 | 0 | 0 | 2 | 0 | 0 | 0 | 0 | 0 | 0 | 0 | 0 |
| 6. | C | 2 | 0 | 0 | 0 | 1 | 0 | 0 | 0 | 0 | 0 | 0 | 0 | 0 | 0 | 0 | 0 | 1 |
| 7. | C | 0 | 0 | 0 | 2 | 0 | 0 | 0 | 1 | 0 | 0 | 0 | 1 | 0 | 0 | 0 | 0 | 0 |
| 8. | N | 0 | 0 | 0 | 0 | 0 | 0 | 1 | 1 | 1 | 0 | 0 | 0 | 1 | 0 | 0 | 0 | 0 |
| 9. | C | 0 | 0 | 0 | 0 | 2 | 0 | 0 | 1 | 0 | 0 | 0 | 0 | 0 | 0 | 0 | 0 | 0 |
| 10. | C | 0 | 0 | 1 | 0 | 0 | 0 | 0 | 0 | 0 | 1 | 1 | 0 | 0 | 0 | 0 | 0 | 0 |

| | | | | | | | | | | | | | | | | | | |
|---|---|---|---|---|---|---|---|---|---|---|---|---|---|---|---|---|---|---|
| 11. | C | 0 | 0 | 0 | 0 | 0 | 0 | 0 | 0 | 0 | 1 | 0 | 2 | 0 | 1 | 0 | 0 | 0 |
| 12. | C | 0 | 0 | 0 | 0 | 0 | 0 | 1 | 0 | 0 | 0 | 2 | 0 | 0 | 0 | 0 | 0 | 0 |
| 13. | C | 0 | 0 | 0 | 0 | 0 | 0 | 0 | 1 | 0 | 0 | 0 | 0 | 0 | 0 | 0 | 0 | 0 |
| 14. | H | 0 | 0 | 0 | 0 | 0 | 0 | 0 | 0 | 0 | 0 | 1 | 0 | 0 | 0 | 0 | 0 | 0 |
| 15. | H | 1 | 0 | 0 | 0 | 0 | 0 | 0 | 0 | 0 | 0 | 0 | 0 | 0 | 0 | 0 | 0 | 0 |
| 16. | H | 0 | 1 | 0 | 0 | 0 | 0 | 0 | 0 | 0 | 0 | 0 | 0 | 0 | 0 | 0 | 0 | 0 |
| 17. | H | 0 | 0 | 0 | 0 | 0 | 1 | 0 | 0 | 0 | 0 | 0 | 0 | 0 | 0 | 0 | 0 | 0 |
| 18. | H | 0 | 0 | 0 | 0 | 0 | 0 | 0 | 0 | 1 | 0 | 0 | 0 | 0 | 0 | 0 | 0 | 0 |
| 19. | H | 0 | 0 | 0 | 0 | 0 | 0 | 0 | 0 | 0 | 1 | 0 | 0 | 0 | 0 | 0 | 0 | 0 |
| 20. | H | 0 | 0 | 0 | 0 | 0 | 0 | 0 | 0 | 0 | 0 | 0 | 1 | 0 | 0 | 0 | 0 | 0 |
| 21. | H | 0 | 0 | 0 | 0 | 0 | 0 | 0 | 0 | 0 | 0 | 0 | 0 | 1 | 0 | 0 | 0 | 0 |
| 22. | H | 0 | 0 | 0 | 0 | 0 | 0 | 0 | 0 | 0 | 0 | 0 | 0 | 1 | 0 | 0 | 0 | 0 |
| 23. | H | 0 | 0 | 0 | 0 | 0 | 0 | 0 | 0 | 0 | 0 | 0 | 0 | 1 | 0 | 0 | 0 | 0 |

| Atom | | 18 | 19 | 20 | 21 | 22 | 23 |
|---|---|---|---|---|---|---|---|
| 1. | C | 0 | 0 | 0 | 0 | 0 | 0 |
| 2. | C | 0 | 0 | 0 | 0 | 0 | 0 |
| 3. | C | 0 | 0 | 0 | 0 | 0 | 0 |
| 4. | N | 0 | 0 | 0 | 0 | 0 | 0 |
| 5. | C | 0 | 0 | 0 | 0 | 0 | 0 |
| 6. | C | 0 | 0 | 0 | 0 | 0 | 0 |
| 7. | C | 0 | 0 | 0 | 0 | 0 | 0 |
| 8. | N | 0 | 0 | 0 | 0 | 0 | 0 |
| 9. | C | 1 | 0 | 0 | 0 | 0 | 0 |

| | | | | | | | |
|---|---|---|---|---|---|---|---|
| 10. | C | 0 | 1 | 0 | 0 | 0 | 0 |
| 11. | C | 0 | 0 | 0 | 0 | 0 | 0 |
| 12. | C | 0 | 0 | 1 | 0 | 0 | 0 |
| 13. | C | 0 | 0 | 0 | 1 | 1 | 1 |
| 14. | H | 0 | 0 | 0 | 0 | 0 | 0 |
| 15. | H | 0 | 0 | 0 | 0 | 0 | 0 |
| 16. | H | 0 | 0 | 0 | 0 | 0 | 0 |
| 17. | H | 0 | 0 | 0 | 0 | 0 | 0 |
| 18. | H | 0 | 0 | 0 | 0 | 0 | 0 |
| 19. | H | 0 | 0 | 0 | 0 | 0 | 0 |
| 20. | H | 0 | 0 | 0 | 0 | 0 | 0 |
| 21. | H | 0 | 0 | 0 | 0 | 0 | 0 |
| 22. | H | 0 | 0 | 0 | 0 | 0 | 0 |
| 23. | H | 0 | 0 | 0 | 0 | 0 | 0 |

| RS | Resonance Weight(%) | Added(Removed) |
|---|---|---|
| 1 | 17.66 | |
| 2 | 12.95 | C 1- C 2, ( C 1- C 6), ( C 2- C 3), ( N 4- C 7), C 7- C 12, C 10- C 11, ( C 11- C 12), N 4, C 6, ( C 10) |
| 3 | 9.89 | ( N 4- C 7), ( C 5- C 9), C 7- C 12, N 8- C 9, C 10- C 11, ( C 11- C 12), N 4, C 5, ( N 8), ( C 10) |

4 7.52 ( N 4- C 7), C 7- N 8, C 10- C 11, ( C 11- C 12),
N 4, ( N 8), ( C 10), C 12

5 7.30 ( N 4- C 7), N 4, C 7, ( C 10)

6 5.67 N 4- C 5, ( N 4- C 7), ( C 5- C 9), C 7- C 12,
C 10- C 11, ( C 11- C 12), C 9, ( C 10)

7 5.33 C 1- C 2, ( C 1- C 6), ( C 2- C 3), ( N 4- C 7),
C 7- C 12, C 10- C 11, ( C 11- C 12), C 3, N 4,
( C 10)

8 3.78 ( C 2- C 3), C 3- C 10, ( C 11- C 12), ( C 10),
C 11, C 12

9 3.74 ( C 5- C 9), N 8- C 9, C 10- C 11, ( C 11- C 12),
C 5, ( N 8), ( C 10), C 12

10 3.54 ( C 2- C 3), C 3- C 10, ( N 4- C 7), C 7- C 12,
( C 11- C 12), N 4, ( C 10), C 11

11 3.46 ( N 4- C 7), ( C 5- C 9), C 7- C 12, N 8- C 9,
C 10- C 11, ( C 11- C 12), N 4, C 5, ( N 8),
( C 10)

12 3.22 C 1- C 2, ( C 1- C 6), ( C 2- C 3), C 10- C 11,
( C 11- C 12), C 3, ( C 10), C 12

13 2.99 C 1- C 2, ( C 1- C 6), ( C 2- C 3), ( N 4- C 7),
C 7- C 12, C 10- C 11, ( C 11- C 12), C 3, N 4,
( C 10)

14 2.38 ( N 4- C 7), N 4, C 7, ( C 10)

15 2.20 ( C 1- C 6), C 5- C 6, ( C 5- C 9), C 10- C 11,
( C 11- C 12), C 9, ( C 10), C 12

16 2.02 ( C 2- C 3), C 3- C 10, ( C 11- C 12), C 2,
( C 10), C 12

17 1.96 ( C 1- C 6), ( N 4- C 7), C 5- C 6, ( C 5- C 9),
C 7- C 12, C 10- C 11, ( C 11- C 12), N 4, C 9,
( C 10)

18 1.79 ( C 2- C 3), C 3- C 10, ( N 4- C 7), C 7- C 12,
( C 11- C 12), C 2, N 4, ( C 10)

19 1.46 ( C 12- H 20), ( C 10), C 12, H 20

20 0.70 ( C 1- C 6), C 5- C 6, ( C 5- C 9), C 10- C 11,
( C 11- C 12), C 1, ( C 10), C 12

21 0.46 ( C 1- C 6), ( N 4- C 7), C 5- C 6, ( C 5- C 9),
C 7- C 12, C 10- C 11, ( C 11- C 12), C 1, N 4,
( C 10)

others 0.00

-------------------------------------------------------------------------

100.00 * Total *

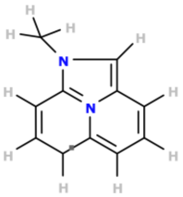
Wgt=17.66% rhoNL=2.76560

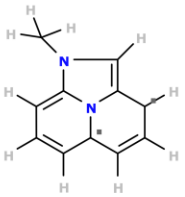
Wgt=12.95% rhoNL=3.74011

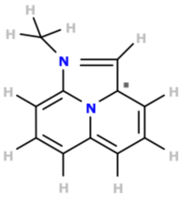
Wgt=9.89% rhoNL=3.89448

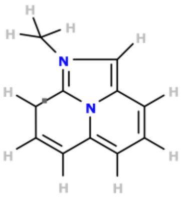
Wgt=7.52% rhoNL=2.64590

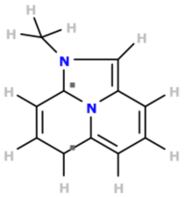
Wgt=7.30% rhoNL=3.24862

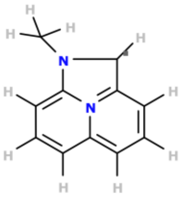
Wgt=5.67% rhoNL=2.92024

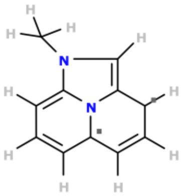
Wgt=5.33% rhoNL=4.14590

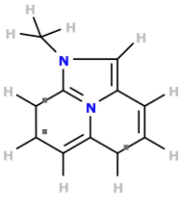
Wgt=3.78% rhoNL=3.62482

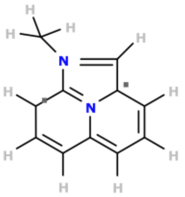
Wgt=3.74% rhoNL=3.26801

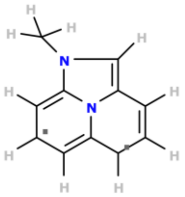
Wgt=3.54% rhoNL=3.47822

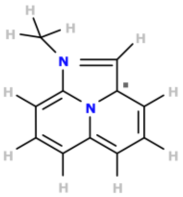
Wgt=3.46% rhoNL=3.12141

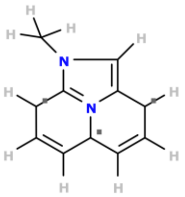
Wgt=3.22% rhoNL=3.60960

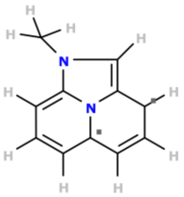
Wgt=2.99% rhoNL=3.46300

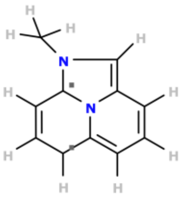
Wgt=2.38% rhoNL=3.89534

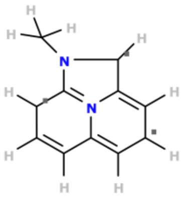
Wgt=2.20% rhoNL=3.60317

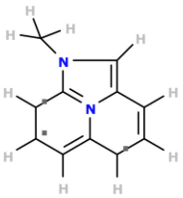
Wgt=2.02% rhoNL=3.36228

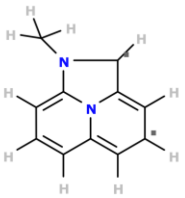
Wgt=1.96% rhoNL=3.45657

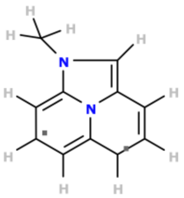
Wgt=1.79% rhoNL=3.21569

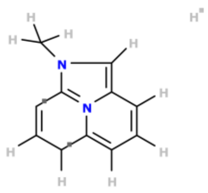
Wgt=1.46% rhoNL=3.91501

## 9.5 TD-DFT calculation

TD-DFT[39][40][41] calculations were performed in ORCA 6.0.0, based on a r2-SCAN-3c(SMD:THF) optimized geometry or on the solid state structure, utilizing the TDA approximation and a spin scaled double hybrid functional as suggested by ref 32.[42]

**6a-neutral**

E -962.085229238965

| | | | |
|---|---|---|---|
| N | -0.53179972929530 | 0.69530084268170 | -0.01164356880143 |
| C | 2.07091655912240 | -0.13027221601468 | 0.00695575378957 |
| C | 1.80835463868786 | 1.25365829724960 | -0.03249050762233 |
| H | 2.59201955656682 | 1.99445956462376 | -0.11738279573983 |
| C | -0.32660220927872 | -0.67377511198469 | 0.02937132118333 |
| C | 3.47824836157291 | -0.59330316368006 | 0.00925169078103 |
| N | -0.14895786813643 | 2.83780205509462 | -0.06387974857460 |
| C | 1.03877718609692 | -1.06851890493331 | 0.04291048376014 |
| H | 1.25543316834305 | -2.12840271487895 | 0.11141274897180 |
| C | 0.46173347333346 | 1.63077678910032 | -0.03286066143003 |
| C | 4.46973317248893 | 0.13092896550627 | 0.68665757338201 |
| H | 4.19844166519139 | 1.02959075302574 | 1.23386787938785 |
| C | 3.85183979123334 | -1.76411605298758 | -0.66594018968114 |
| H | 3.10445947962002 | -2.32681238733258 | -1.21828135314373 |
| C | -2.94430259687230 | 0.46687411302043 | -0.00738921605927 |
| H | -3.91921024512278 | 0.93588390183246 | -0.01523278354557 |
| C | -1.78934603293355 | 1.31767437886404 | -0.04079573653173 |
| C | -2.77822188124057 | -0.89560258916797 | 0.03906634407183 |
| C | -3.97332741086656 | -1.85053658909368 | 0.08061088248394 |
| C | -1.46428548217568 | -1.46926956859696 | 0.05392799766444 |

| | | | |
|---|---|---|---|
| H | -1.32509062586836 | -2.54284597849849 | 0.08654148607905 |
| C | 5.79036049924949 | -0.30543333536310 | 0.69296469676869 |
| H | 6.54174484286372 | 0.26437641261310 | 1.23321251362518 |
| C | -1.55434754317443 | 2.66779470927301 | -0.08637081378585 |
| C | 6.14912061789552 | -1.47014298222692 | 0.01803685640827 |
| H | 7.18144825108331 | -1.80923663973834 | 0.02142022254239 |
| C | 5.17381022563467 | -2.19596488855193 | -0.66246317958792 |
| H | 5.44453822271507 | -3.10068652524849 | -1.20037011548453 |
| C | -2.50011617769977 | 3.80699492050448 | -0.13017162941947 |
| H | -2.42681097862834 | 4.45044698262290 | 0.75705757029502 |
| H | -3.52196920367019 | 3.42054904133760 | -0.17358803294541 |
| H | -2.34722352374563 | 4.44231723514041 | -1.01261071085374 |
| C | 0.57124022971778 | 4.09388379315320 | -0.15922113730884 |
| H | -0.10174987911725 | 4.91672724912622 | 0.08675896357025 |
| H | 0.96741704323487 | 4.24727798769919 | -1.17125834074774 |
| H | 1.40335320149449 | 4.10065198275245 | 0.55269267705300 |
| C | -5.31776930448377 | -1.11585904250190 | 0.05396139660650 |
| H | -5.43728185847596 | -0.45257782084203 | 0.91802731672072 |
| H | -6.13126955468238 | -1.84925013495149 | 0.08354177952401 |
| H | -5.43933142702064 | -0.52158348016946 | -0.85870466205352 |
| C | -3.91650621478299 | -2.79226667562449 | -1.13759467223940 |
| H | -3.94623454888760 | -2.22172528254887 | -2.07335269629442 |
| H | -4.77714663726602 | -3.47193223720097 | -1.12476171365394 |
| H | -3.00769529624988 | -3.40197278410539 | -1.14041008949990 |
| C | -3.90916127346010 | -2.68902831754780 | 1.37181385307554 |

| | | | |
|---|---|---|---|
| H | -2.99800579005029 | -3.29337074151690 | 1.42057206271817 |
| H | -4.76777002130583 | -3.36977850300502 | 1.41892937840715 |
| H | -3.93658387165469 | -2.04323630690947 | 2.25737390613447 |

! VeryTightSCF SCS-PBE-QIDH def2-tzvpp def2/J def2-TZVP/C SMD(THF) RIJCOSX

nroots 30; maxdim 20

------------------------------------

TD-DFT/TDA EXCITED STATES (SINGLETS)

------------------------------------

the weight of the individual excitations are printed if larger than 1.0e-02

STATE 1: E= 0.063030 au 1.715 eV 13833.5 cm**-1 <S**2> = 0.000000 Mult 1

84a -> 85a : 0.863822 (c= 0.92942024)

84a -> 87a : 0.075703 (c= -0.27514175)

84a -> 88a : 0.034781 (c= 0.18649555)

STATE 2: E= 0.120844 au 3.288 eV 26522.3 cm**-1 <S**2> = 0.000000 Mult 1

84a -> 85a : 0.032561 (c= 0.18044572)

84a -> 86a : 0.130242 (c= 0.36089110)

84a -> 87a : 0.651226 (c= 0.80698577)

84a -> 88a : 0.139899 (c= 0.37403040)

STATE 3: E= 0.137843 au 3.751 eV 30253.1 cm**-1 <S**2> = 0.000000 Mult 1

84a -> 85a : 0.043243 (c= -0.20795039)

84a -> 86a : 0.331374 (c= 0.57565084)

84a -> 87a : 0.198503 (c= -0.44553725)

84a -> 88a : 0.225156 (c= 0.47450591)

84a -> 90a : 0.154353 (c= -0.39287840)

STATE  4:  E=   0.143019 au      3.892 eV    31389.0 cm**-1 <S**2> =   0.000000 Mult 1

84a ->  85a  :     0.018858 (c= -0.13732415)

84a ->  86a  :     0.422976 (c= -0.65036623)

84a ->  88a  :     0.218692 (c=  0.46764490)

84a ->  89a  :     0.125782 (c=  0.35465786)

84a ->  90a  :     0.149383 (c= -0.38650161)

84a ->  95a  :     0.010153 (c=  0.10076177)

HOMO: 84a

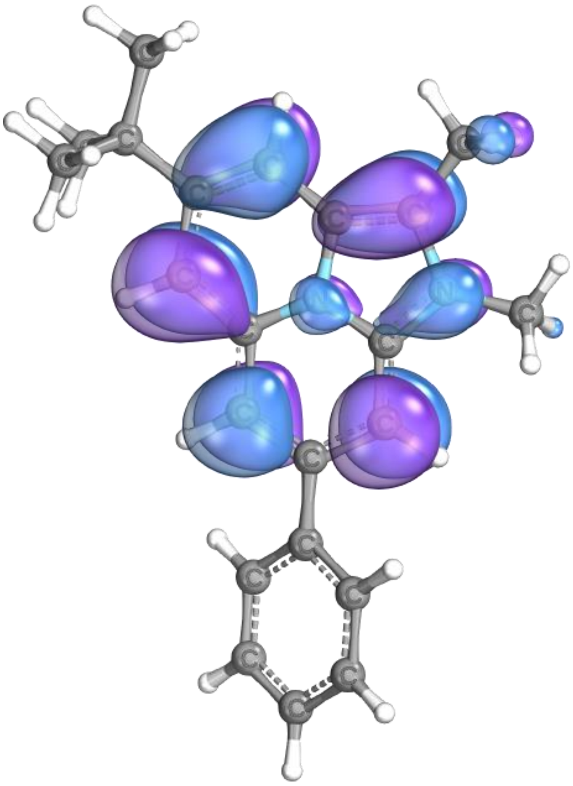

LUMO: 85a

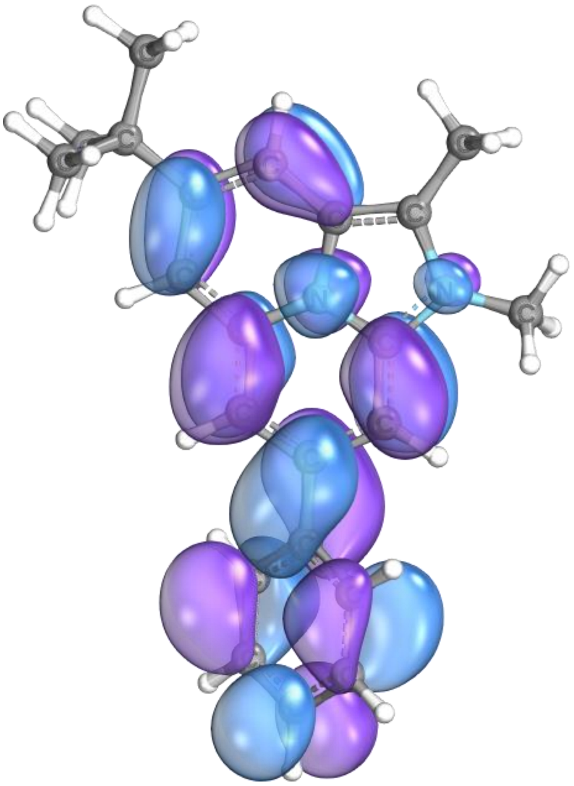

LUMO+1: 86a

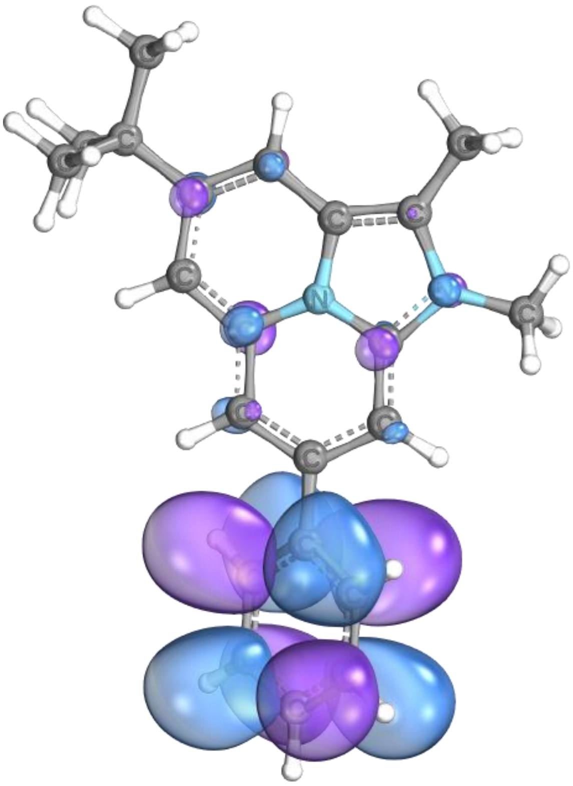

LUMO+2 87a

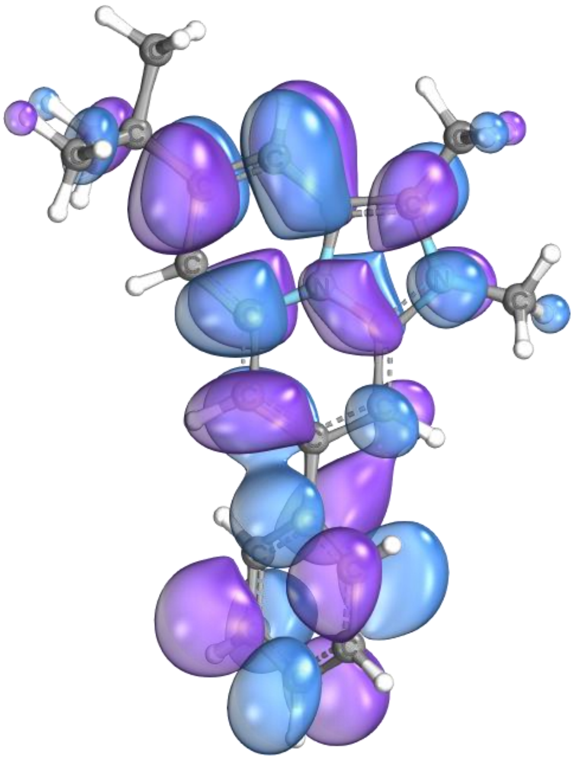

**6m**$^{+'}$

| | | | |
|---|---|---|---|
| N | -0.52915838403187 | 0.69365969416284 | -0.00770765788683 |
| C | 2.07163062600327 | -0.13748304051059 | 0.01593210687243 |
| C | 1.79484861430307 | 1.23977384364483 | -0.02858603855979 |
| H | 2.58366413247890 | 1.97854668657415 | -0.09430648791632 |
| C | -0.31484181705746 | -0.66192125665443 | 0.03893730823484 |
| C | 3.47502494639410 | -0.59907333967565 | 0.01819040117373 |
| N | -0.16038422966977 | 2.84090979770523 | -0.07287612221782 |
| C | 1.02622144263932 | -1.07164085542152 | 0.05354620796724 |
| H | 1.23139302629679 | -2.13388490655511 | 0.11826276065675 |

| | | | |
|---|---|---|---|
| C | 0.46168640632687 | 1.63020888565186 | -0.03284865319000 |
| C | 4.46427202794701 | 0.12674834725236 | 0.69605517946891 |
| H | 4.19658702782277 | 1.02340618345582 | 1.24828185384298 |
| C | 3.83980191311931 | -1.77065327239835 | -0.65947873407045 |
| H | 3.09193089785668 | -2.33242238168735 | -1.21206747917724 |
| C | -2.91684434278474 | 0.47346289105297 | -0.01228988188386 |
| H | -3.89058525234718 | 0.94436422792690 | -0.03447874313412 |
| C | -1.77605214175612 | 1.29934086400402 | -0.03905491450011 |
| C | -2.76197830705891 | -0.90935045894526 | 0.04375585119357 |
| C | -3.96332292024341 | -1.85373893547779 | 0.08016205631846 |
| C | -1.46580741616834 | -1.46469682717289 | 0.06816299456216 |
| H | -1.31773059637790 | -2.53731630425412 | 0.10752335258774 |
| C | 5.78303661786951 | -0.31234018858198 | 0.69883950316934 |
| H | 6.53648074329490 | 0.25353387733100 | 1.23952520718123 |
| C | -1.53535524345375 | 2.66971960713709 | -0.08905678429966 |
| C | 6.13677430239141 | -1.47589755609104 | 0.01941041540238 |
| H | 7.16856221695088 | -1.81594517043479 | 0.02002453770494 |
| C | 5.16132570100149 | -2.20130767970086 | -0.66088746608728 |
| H | 5.43131697365781 | -3.10431340762410 | -1.20124414727354 |
| C | -2.48978147703973 | 3.79499014816335 | -0.14607863464611 |
| H | -2.42436818378387 | 4.42325357753732 | 0.75082735274023 |
| H | -3.50824284867120 | 3.40776087187246 | -0.21744686773248 |
| H | -2.30032805031145 | 4.43442047918160 | -1.01624498495300 |
| C | 0.52967552560418 | 4.12298704144819 | -0.16721975893647 |
| H | -0.04856576288227 | 4.88232480610372 | 0.36207755066984 |

| | | | |
|---|---|---|---|
| H | 0.64625833897586 | 4.41500259695120 | -1.21630180584663 |
| H | 1.51218199249254 | 4.04038051283754 | 0.30068797634945 |
| C | -5.29670176716795 | -1.10076267015741 | 0.04744006759864 |
| H | -5.41264441534389 | -0.43635117454482 | 0.91109545194545 |
| H | -6.11664002790114 | -1.82577032817901 | 0.07568405754050 |
| H | -5.40981495770041 | -0.50890304540230 | -0.86781454209542 |
| C | -3.90095772615448 | -2.79247175650867 | -1.13981135507517 |
| H | -3.92244234900305 | -2.22182764576283 | -2.07519994585391 |
| H | -4.76618567684299 | -3.46486761001847 | -1.12950653570677 |
| H | -2.99734316629385 | -3.41023447285613 | -1.13557526684269 |
| C | -3.90418303222692 | -2.68865596472226 | 1.37369441931814 |
| H | -2.99929208795136 | -3.30258764445216 | 1.42367402456745 |
| H | -4.76826292216936 | -3.36128761006474 | 1.41590492950541 |
| H | -3.92998537103326 | -2.04262043613981 | 2.25855024131387 |

## 10. Literature


[1] M. Li, Y. Xie, Y. Ye, Y. Zou, H. Jiang, W. Zeng, *Org. Lett.* **2014**, *16*, 6232–6235.

[2] C. Belle, C. Bougault, M. T. Averbuch, A. Durif, J. L. Pierre, J. M. Latour, L. Le Pape, *J. Am. Chem. Soc.* **2001**, *123*, 8053–8066.

[3] Y. Álvarez-Casao, B. Estepa, D. Monge, A. Ros, J. Iglesias-Sigüenza, E. Álvarez, R. Fernández, J. M. Lassaletta, *Tetrahedron* **2016**, *72*, 5184–5190.

[4] C.-Y. Wang, Y.-H. Liu, S.-M. Peng, S.-T. Liu, *J. Organomet. Chem.* **2006**, *691*, 4012–4020.

[5] É. Lévesque, W. S. Bechara, L. Constantineau-Forget, G. Pelletier, N. M. Rachel, J. N. Pelletier, A. B. Charette, *J. Org. Chem.* **2017**, *82*, 5046–5067.

[6] G. Pelletier, A. B. Charette, *Org. Lett.* **2013**, *15*, 2290–2293.

[7] T. R. Kelly, F. Lang, *J. Org. Chem.* **1996**, *61*, 4623–4633.

[8] Y.-S. Li, J.-L. Liao, K.-T. Lin, W.-Y. Hung, S.-H. Liu, G.-H. Lee, P.-T. Chou, Y. Chi, *Inorg. Chem.* **2017**, *56*, 10054–10060.

[9] S. Stoll, A. Schweiger, *J. Magn. Reson.* **2006**, *178*, 42–55.

[10] P. Hohenberg, W. Kohn, *Phys. Rev.* **1964**, *136*, B864-B871.

[11] W. Kohn, L. J. Sham, *Phys. Rev.* **1965**, *140*, A1133-A1138.

[12] Gaussian 16, Revision C.01, M. J. Frisch, G. W. Trucks, H. B. Schlegel, G. E. Scuseria, M. A. Robb, J. R. Cheeseman, G. Scalmani, V. Barone, G. A. Petersson, H. Nakatsuji, X. Li, M. Caricato, A. V. Marenich, J. Bloino, B. G. Janesko, R. Gomperts, B. Mennucci, H. P. Hratchian, J. V. Ortiz, A. F. Izmaylov, J. L. Sonnenberg, D. Williams-Young, F. Ding, F. Lipparini, F. Egidi, J. Goings, B. Peng, A. Petrone, T. Henderson, D. Ranasinghe, V. G. Zakrzewski, J. Gao, N. Rega, G. Zheng, W. Liang, M. Hada, M. Ehara, K. Toyota, R. Fukuda, J. Hasegawa, M. Ishida, T. Nakajima, Y. Honda, O. Kitao, H. Nakai, T. Vreven, K. Throssell, J. A. Montgomery, Jr., J. E. Peralta, F. Ogliaro, M. J. Bearpark, J. J. Heyd, E. N. Brothers, K. N. Kudin, V. N. Staroverov, T. A. Keith, R. Kobayashi, J. Normand, K. Raghavachari, A. P. Rendell, J. C. Burant, S. S. Iyengar, J. Tomasi, M. Cossi, J. M. Millam, M. Klene, C. Adamo, R. Cammi, J. W. Ochterski, R. L. Martin, K. Morokuma, O. Farkas, J. B. Foresman, D. J. Fox, Gaussian, Inc., Wallingford CT, **2016**.

[13] F. Weigend, R. Ahlrichs, *Phys. Chem. Chem. Phys.* **2005**, *7*, 3297–3305.

[14] F. Weigend, *Phys. Chem. Chem. Phys.* **2006**, *8*, 1057–1065.

[15] S. Grimme, J. Antony, S. Ehrlich, H. Krieg, *J. Chem. Phys.* **2010**, *132*, 154104.

[16] S. Grimme, S. Ehrlich, L. Goerigk, *J. Comput. Chem.* **2011**, *32*, 1456–1465.

[17] G. Knizia, *J. Chem. Theory Comput.* **2013**, *9*, 4834–4843.

[18] GaussView, Version 6.1, Dennington, R., T. A. Keith, Millam, J. M. Semichem Inc., Shawnee Mission, KS, **2016**.

[19] Apex 5, Bruker AXS Inc.: Madison, WI **2023**.

[20] O. V. Dolomanov, L. J. Bourhis, R. J. Gildea, J. A. K. Howard, H. Puschmann, *J. Appl. Crystallogr.* **2009**, *42*, 339–341.
[21] G. M. Sheldrick, *Acta Cryst. A* **2015**, *71*, 3–8.
[22] G. M. Sheldrick, *Acta Cryst. C* **2015**, *71*, 3–8.
[23] G. M. Sheldrick, *Acta Cryst. A* **2008**, *64*, 112–122.
[24] H. D. Flack, *Acta Cryst. A* **1983**, *39*, 876–881.
[25] F. Neese, *WIREs Comput. Mol. Sci.* **2012**, *2*, 73–78.
[26] F. Neese, *WIREs Comput. Mol. Sci.* **2022**, *12*.
[27] NBO 7.0. E. D. Glendening, J, K. Badenhoop, A. E. Reed, J. E. Carpenter, J. A. Bohmann, C. M. Morales, P. Karafiloglou, C. R. Landis, and F. Weinhold, Theoretical Chemistry Institute, University of Wisconsin, Madison, **2018**.
[28] R. Herges, D. Geuenich, *J. Phys. Chem. A* **2001**, *105*, 3214–3220.
[29] D. Geuenich, K. Hess, F. Köhler, R. Herges, *Chem. Rev.* **2005**, *105*, 3758–3772.
[30] Z. Wang, *Chemistry* **2024**, *6*, 1692–1703.
[31] W. Humphrey, A. Dalke, K. Schulten, *J. Mol. Graph.* **1996**, *14*, 33-8, 27-8.
[32] T. Lu, F. Chen, *J. Comput. Chem.* **2012**, *33*, 580–592.
[33] T. Lu, *J. Chem. Phys.* **2024**, *161*, 82503.
[34] F. Neese, A. Hansen, D. G. Liakos, *J. Chem. Phys.* **2009**, *131*, 64103.
[35] F. Neese, F. Wennmohs, A. Hansen, *J. Chem. Phys.* **2009**, *130*, 114108.
[36] G. Bistoni, C. Riplinger, Y. Minenkov, L. Cavallo, A. A. Auer, F. Neese, *J. Chem. Theory Comput.* **2017**, *13*, 3220–3227.
[37] A. V. Marenich, C. J. Cramer, D. G. Truhlar, *J. Phys. Chem. B* **2009**, *113*, 6378–6396.
[38] S. Grimme, A. Hansen, S. Ehlert, J.-M. Mewes, *J. Chem. Phys.* **2021**, *154*, 64103.
[39] M. Casanova-Páez, M. B. Dardis, L. Goerigk, *J. Chem. Theory Comput.* **2019**, *15*, 4735–4744.
[40] M. Casanova-Páez, L. Goerigk, *J. Chem. Phys.* **2020**, *153*, 64106.
[41] M. Casanova-Páez, L. Goerigk, *J. Chem. Theory Comput.* **2021**, *17*, 5165–5186.
[42] P. P. Fehér, Á. Madarász, A. Stirling, *Chemistry Methods* **2023**, *3*, e202200069.
